# Global basal cell carcinoma in 55+ population: 1990– 2021 burden、risk-factor trends and 2050 forecast

**DOI:** 10.3389/fonc.2025.1702129

**Published:** 2025-12-19

**Authors:** Yan-Xia Cai, Minglei Rong

**Affiliations:** Department of Dermatology and Venereology, Sanya Central Hospital (The Third People’s Hospital of Hainan Province), Sanya, China

**Keywords:** basal cell carcinoma, burden, risk factors, forecast, global burden of disease

## Abstract

**Objective:**

This study map the 1990–2021 basal-cell carcinoma burden in people ≥55 years worldwide, pinpoint key risk drivers, and offer concise intervention guidance for elder-focused prevention.

**Methods:**

We focused on adults aged ≥55 years because this group shows the highest basal-cell carcinoma (BCC) frequency and matches the Global Burden of Disease (GBD) age strata. Incidence and disability-adjusted life-year (DALY) counts for 1990–2021 were downloaded from the Global Health Data Exchange. After direct age-standardisation, we plotted global, regional and national rates, generated world maps, computed annual percent change (APC) and contrasted 1990 versus 2021 distributions—all with R 4.2.1. A Bayesian hierarchical model then projected the 2050 burden.

**Results:**

1990-2021, Among adults ≥55 years, global basal-cell carcinoma incidence rose and then plateaued, while DALYs climbed before edging downward. The steepest gains in both age-standardized incidence and DALY rates occurred in high-income North America. Men consistently outpaced women across all metrics. The USA, Brazil and China recorded the largest absolute caseloads, yet the USA always posted the highest incidence rate and Nepal the lowest. Overall, the BCC burden has expanded over the past three decades.

**Conclusion:**

Over the last three decades, both new cases and the overall toll of basal-cell carcinoma among older adults have risen worldwide, fueled by expanding and aging populations, greater ultraviolet exposure, and better case detection. Looking forward, vigorous sun-protection education and unified UV-shielding measures are essential to curb incidence and ease the growing burden.

## Introduction

Basal cell carcinoma (BCC) is a common skin cancer originating from the basal layer of the epidermis. It typically affects sun-exposed areas, including the head, face, and neck.

BCC grows slowly and rarely metastasizes, but untreated cases can invade surrounding tissues, leading to disfiguring changes. Several treatment options for BCC are available, including surgical excision (1, 2), pharmacotherapy (3), and neoadjuvant therapy (4). Mohs micrographic surgery is the preferred treatment due to its high cure rate and low recurrence rate (5), although some pathological subtypes, such as sclerodermiform variants with infiltrative tendencies, still have higher recurrence rates. The epidemiology of BCC shows a global increase, with approximately 4 million new cases in 2019. Incidence among individuals over 55 years old has increased significantly, surpassing that of other skin cancers (6). Studies also show an increased risk of transformation into malignant melanoma, highlighting the importance of epidemiological research on BCC for public health (7). This study analyzed global BCC incidence, age and gender distribution, incidence in different countries and regions, disease burden, and risk factors from 1990 to 2021 to enhance early recognition and treatment of BCC and provide robust evidence for global BCC prevention and control.

## Methods

### Study population

The study population comprised elderly patients aged over 55 years diagnosed with BCC. Studies indicate that BCC predominantly occurs in patients over 50 years old, with dermatoscopy showing arborizing capillary dilation, large blue-gray ovoid nests, and ulcers more frequently in elderly patients (8). BCC is closely associated with sun exposure, but other potential etiological factors include advanced age, local trauma, chronic inflammation, radiotherapy, and indoor tanning (9–11). Thus, this study included patients aged over 55 years with BCC, aligning with the age grouping in the GBD database to facilitate data analysis and comparison. Moreover, this age group is the peak incidence age for BCC (12). Based on the above rationale and previous studies, patients aged over 55 years with BCC were selected for this study.

### Data collection

In this study, basal cell carcinoma (BCC) was defined by the ICD-10 topography code C44 (all sub-sites C44.0–C44.9) combined with the ICD-O-3 morphology code 8090/3.

Corresponding incidence and prevalence estimates were extracted from the Global Health Data Exchange (GHDx, http://ghdx.healthdata.org/) by querying “non-melanoma skin cancer (basal-cell carcinoma)” for cause and “incidence, prevalence” for measure. DALYs(Disability-Adjusted Life Years)” for measurements; “all locations” for location; “1990–2021” for years; “number and rate” for metrics; “male, female and both” for sex; and “Age-standardized,55+ years and corresponding 5-year bands” for age. We followed the Guidelines for Accurate and Transparent Health Estimates Reporting guidelines for cross-sectional studies.

### Socio-demographic index

The Socio-Demographic Index (SDI) is a comprehensive indicator developed by the GBD study team, used to measure the development level of a country or region. The value of the SDI ranges between 0 and 1. Based on the SDI value, countries and regions around the world are categorized into five levels: Low SDI, Low-Middle SDI, Middle SDI, High-Middle SDI, and High SDI. Regions with low SDI typically have low levels of economic development, lower educational attainment, and higher fertility rates. In contrast, high SDI regions are usually developed countries, characterized by higher per capita income, robust educational systems, low fertility rates, and well-developed healthcare services. The SDI is not only a measure of development level but also an important covariate that can be used to predict disease burdens and health status across different regions. It provides a crucial reference for global health research and policy-making.

### Statistical analysis

Data from the GBD database were used to describe the incidence and DALY rates of BCC among individuals aged 55 and older globally, regionally, and nationally from 1990 to 2021 and calculated the ASIR, ASMR, and ASDR of BCC for individuals aged 55 and older globally, regionally, and nationally, and created world maps of ASIR and ASDR. For analyzing the temporal trends of BCC burden, the estimated annual percentage change (EAPC) was calculated based on a linear regression model, with the model form specified as:


$$\text{y}={\text{β}}_{0}+{\text{β}}_{1}{\text{x}}_{1}$$

In this model, x_1_ represents the calendar year (1990–2021), and y denotes the natural logarithm of the age-standardized rate (ASR) — including ASIR, ASMR, and ASDR, respectively, to assess trends in incidence, mortality, and DALY burden. β_0_ is the intercept term, and β_1_ is the regression coefficient corresponding to the calendar year. The EAPC, which quantifies the annual trend of ASRs over the study period, was derived using the formula:


$$\text{EAPC}=100\times (\exp({\text{β}}_{1})\;-1$$

The 95% confidence intervals (CIs) for EAPC were computed by substituting the upper and lower bounds of β_1_ (obtained from the linear regression analysis) into the above formula.The EAPC was calculated using the formula 100* (exp(β) -1), and the 95% CI was derived from the linear regression model. World maps of the EAPC of ASIR, EAPC of ASMR, and EAPC of ASDR were created. Patients with BCC aged over 55 years were divided into eight age groups: 55-59, 60-64, 65-69, 70-74, 75-79, 80-84, 85-89, and 90–94 years. The age burden composition of patients with BCC aged 55 and older in 1990 and 2021 was compared. The age composition of the incidence rate of BCC aged 55 and older globally in 1990 and 2021 was compared, and a Bayesian model was used to predict the disease burden in 2050. All statistics were performed using the R program (version 4.2.1, R core team).

## Results

### Time and sex trends of 55+ year BCC from 1990 to 2021

From 1990 to 2021, the incidence of BCC among both sexes combined showed a fluctuating trend globally. It increased from 1990 to 2005 and then stabilized from 2005 to 2021. The incidence rate per 100,000 population increased from 135 (150-175) in 1990 to 230 (215-255) in 2005. For males, it increased from 152 per 100,000 in 1990 to 290 per 100,000 in 2005. For females, it increased from 126 per 100,000 in 1990 to 190 per 100,000 in 2005. (Trends in the number of cases) From 1990 to 2005, the incidence and prevalence of BCC among both sexes combined showed an increasing trend. From 2005 to 2019, these values stabilized, with a slight downward trend observed in females. The DALYs for BCC also showed fluctuations, increasing from 1990 to 2005, then decreasing from 2005 to 2019, with a relatively stable trend from 2015 to 2019. Overall, there was a slight decrease. DALYs increased from 0.07 in 2000 to 0.13 in 2005, with stable values from 1990 to 2000 and an increase from 2000 to 2005. From 2000 to 2005, the ASIR and ASDR of BCC showed an increasing trend, with the ASIR rising from 130 (102-152) per 100,000 in 2000 to 230 (215-255) per 100,000 in 2005. The ASDR also increased from 0.07 (0.04-0.07) per 100,000 in 2000 to 0.13 (0.08-0.13) per 100,000 in 2005.

### Gender differences

**Figure 1:**
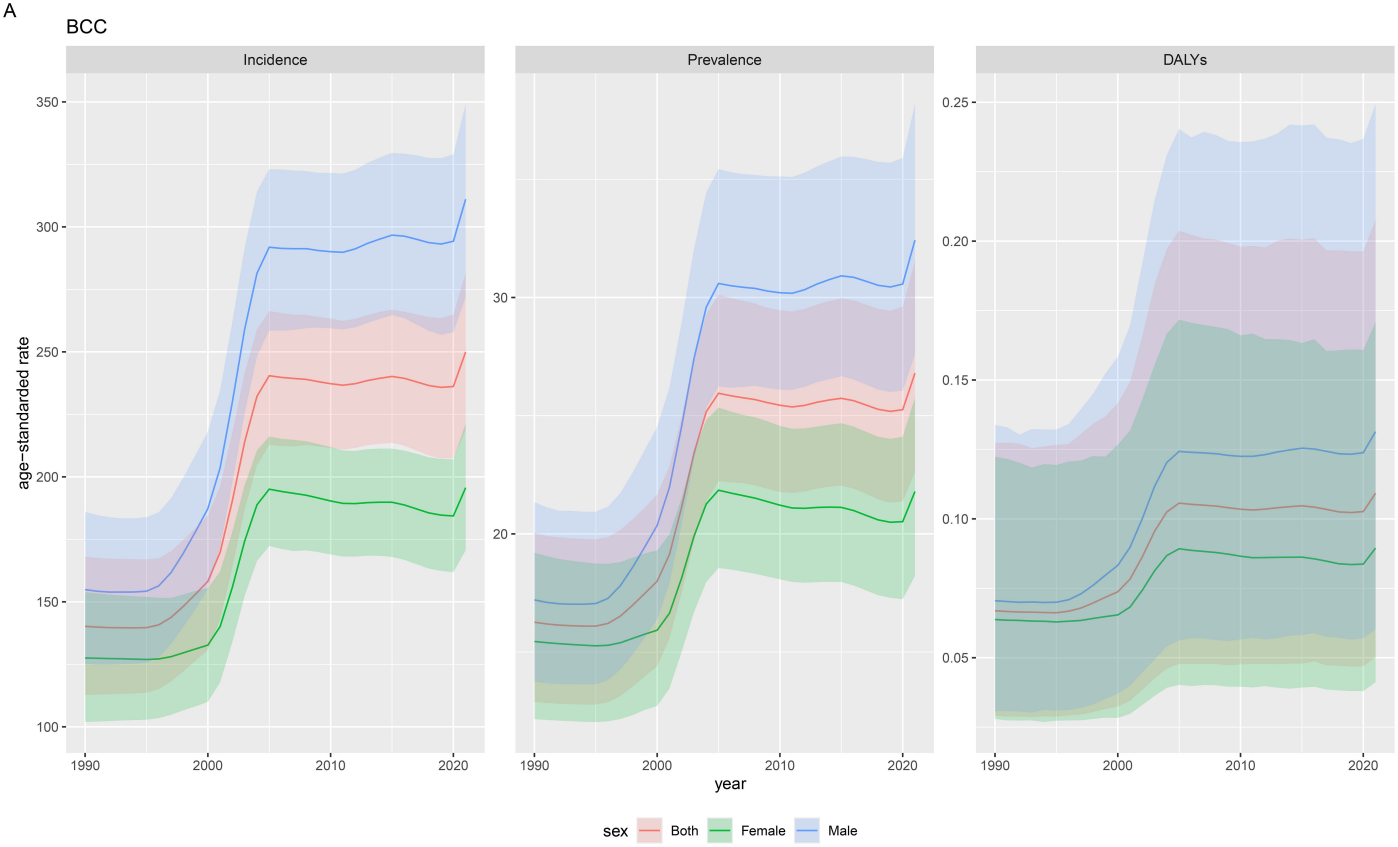
Change in the 55+ year BCC, 1990 to 2021.

From 1990 to 2019, the incidence, prevalence, and DALYs of BCC among elderly males were consistently higher than those among elderly females. In 2005, the number of BCC cases among elderly males was 1.5 times that of elderly females (49.3 (43.7-54.6) vs 33.0 (36.5-29.1)), and DALYs were 1.4 times higher in males than in females (0.021 (0.039-0.009) vs 0.015 (0.028-0.007)). From 2000 to 2005, the ASIR and ASDR of BCC among elderly males and females both showed an increasing trend, similar to the overall population trend, with males consistently having higher values than females (**Figure 1**, **Supplementary Data 1**).

### Region and sex trends of 55+ year BCC from 1990 to 2021

The incidence and DALYs of BCC among the elderly have increased in all global regions, with the most significant rises in age-standardized incidence and DALYs observed in High-income North America. The age-standardized incidence rate increased from 1000.01 (772.06-1232.60) in 1990 to 2293.86 (2059.24-2545.26) in 2021, and the age-standardized DALY rate rose from 0.42 (0.18-0.79) in 1990 to 0.91 (0.43-1.74) in 2021. The highest increase in age-standardized incidence was in High-income North America, and the highest increase in age-standardized DALYs was also in High-income North America. In 2021, regions with High SDI and High-income North America had higher incidence and DALYs for BCC.

At the regional level, from 1990 to 2021, the ASIR of BCC among the elderly population increased globally. The most significant rise was in High-income North America, from 1000.01 (772.06-1232.60) in 1990 to 2293.86 (2059.24-2545.26) in 2021. The second-largest increase was in High SDI regions, from 384.36 (304.23-467.37) in 1990 to 824.67 (734.18-915.28) in 2021. The smallest increase was in Oceania, from 0.16 (0.05-0.36) in 1990 to 0.17 (0.05-0.36) in 2021.

At the regional level, from 1990 to 2021, the incidence rates of BCC increased across the SDI quintiles, from Low SDI to High SDI regions. The highest incidence rate was in High SDI regions, at 824.67 (734.18-915.28) in 2021. Similarly, the DALY rates increased with higher SDI levels, with the highest DALY rate in High SDI regions at 0.34 (0.16-0.66) in 2021. Among the 21 regions, High-income North America had the highest incidence and DALY rates, followed by Western Europe. The smallest increases were in Oceania, followed by South Asia.

Globally, the age-standardized DALYs for BCC among the elderly have shown an increasing trend, with the most significant rise in High-income North America, from 0.42 (0.18-0.79) in 1990 to 0.91 (0.43-1.74) in 2021. The smallest increase was in Oceania, from 0.0001 (0.00002-0.0002) in 1990 to 0.0001 (0.000025-0.00027) in 2021, followed by South Asia, from 0.0024 (0.00100-0.0049) in 1990 to 0.0027 (0.00111-0.00534) in 2021. In all regions, the DALYs for males were consistently higher than those for females (**Table 1**).

**Table 1:** Regional burden in 1990 and 2021.

Location	Sex	Num_1990(95%UI)	ASIR_1990(95%UI)	Num_2021(95%UI)	ASIR_2021(95%UI)	Percentage change (%)		Num_1990(95%UI)	ASiR_1990(95%UI)	Num_2021(95%UI)	ASIR_2021(95%UI)	Percentage change (%)	
						1990-2021						1990-2021	
High SDI	Male	369277.4 (293194.7-448563.1)	77.5 (61.5-94.1)	1709503.8 (1513450.7-1902210.2)	180.6 (159.9-201)	133.2 (107.6-172.9)		160.15 (69.56-301.61)	0.03 (0.01-0.06)	690.01 (319.76-1315.39)	0.07 (0.03-0.14)	117 (92.4-149.9)	
High SDI	Female	164.8 (71.5-317.2)	0 (0-0.1)	486.7 (226.1-943.1)	0 (0-0.1)	87.2 (66.6-117.2)		164.83 (71.47-317.25)	0.03 (0.01-0.05)	486.72 (226.07-943.11)	0.04 (0.02-0.09)	69.1 (51.6-97.4)	
High-middle SDI	Both	125893.1 (107466.4-145732.7)	12.4 (10.6-14.3)	422345.7 (343110.7-504315.1)	20.7 (16.8-24.7)	67 (58.7-74.1)		74.56 (32.35-146.9)	0.01 (0-0.01)	225.44 (98.08-426.94)	0.01 (0-0.02)	50.5 (38.1-64)	
High-middle SDI	Male	60218.6 (51807.2-68765)	13.6 (11.7-15.5)	222533.1 (181440.7-264434.5)	23.8 (19.4-28.3)	75.1 (65.5-83.4)		33.54 (14.69-66.85)	0.01 (0-0.02)	112.67 (48.35-212.24)	0.01 (0.01-0.02)	59.2 (41.4-78.3)	
High-middle SDI	Female	41 (17.7-79.7)	0 (0-0)	112.8 (48.6-216.1)	0 (0-0)	57.5 (49.8-65)		41.02 (17.7-79.69)	0.01 (0-0.01)	112.77 (48.65-216.12)	0.01 (0-0.02)	42.3 (29.9-56.8)	
Middle SDI	Both	73499.2 (60071.3-87831.3)	7.2 (5.9-8.6)	393823 (313601.5-475730.3)	14.2 (11.3-17.2)	97.9 (91.5-104.4)		36.73 (16.01-69.07)	0 (0-0.01)	192.58 (85.91-366.79)	0.01 (0-0.01)	93.6 (80.3-109.8)	
Middle SDI	Male	38510.5 (31449.8-46027.9)	7.7 (6.3-9.2)	215555.4 (173840.4-257629)	16.4 (13.2-19.5)	111.6 (103.5-120.2)		18.82 (8.21-35.28)	0 (0-0.01)	101.92 (47.05-193.45)	0.01 (0-0.01)	104.7 (83.8-128.8)	
Middle SDI	Female	17.9 (7.8-33.7)	0 (0-0)	90.7 (39.6-172.4)	0 (0-0)	84.2 (75.4-92.5)		17.91 (7.85-33.69)	0 (0-0.01)	90.66 (39.56-172.38)	0.01 (0-0.01)	83 (64.8-100.1)	
Low-middle SDI	Both	20710 (16320.5-25330.1)	3.5 (2.7-4.3)	43542.1 (33044-55722.9)	3.1 (2.3-3.9)	-12.1 (-17.7--7.3)		10.36 (4.48-19.74)	0 (0-0)	22.8 (9.64-44.24)	0 (0-0)	-8 (-14.8--2.3)	
Low-middle SDI	Male	11641.8 (9222.7-14162.4)	3.9 (3.1-4.7)	22594.5 (17148.1-28897.7)	3.3 (2.5-4.2)	-13.9 (-19.7--8.5)		5.7 (2.48-11.12)	0 (0-0)	11.61 (4.9-22.25)	0 (0-0)	-9.6 (-17.6--2.1)	
Low-middle SDI	Female	4.7 (2-8.8)	0 (0-0)	11.2 (4.7-21.9)	0 (0-0)	-8.9 (-14.3--4.2)		4.66 (1.98-8.83)	0 (0-0)	11.19 (4.73-21.92)	0 (0-0)	-5.3 (-12.8-1.9)	
Low SDI	Both	4002.6 (2873.3-5317.5)	1.8 (1.3-2.4)	8682.5 (6181.2-11475.2)	1.8 (1.3-2.4)	-1.4 (-3.3-0.2)		2.18 (0.89-4.34)	0 (0-0)	4.7 (1.92-9.29)	0 (0-0)	-1.9 (-3.9-0.2)	
Low SDI	Male	2475.5 (1775.8-3270.8)	2.2 (1.6-2.9)	5135.8 (3680.8-6783.3)	2.2 (1.6-2.9)	-1.2 (-3.6-0.8)		1.35 (0.55-2.66)	0 (0-0)	2.78 (1.13-5.42)	0 (0-0)	-1.6 (-4.2-1)	
Low SDI	Female	0.8 (0.3-1.7)	0 (0-0)	1.9 (0.8-3.9)	0 (0-0)	0.8 (-1.8-3.1)		0.83 (0.33-1.67)	0 (0-0)	1.92 (0.78-3.85)	0 (0-0)	0.2 (-2.8-3.4)	
Andean Latin America	Both	2393.7 (2144.7-2691.3)	12.1 (10.8-13.6)	5308 (4031.9-6660.7)	9.1 (6.9-11.4)	-24.9 (-38.6--12.5)		1.43 (0.66-2.83)	0.01 (0-0.01)	3.28 (1.4-6.5)	0.01 (0-0.01)	-22.4 (-34.8--10)	
Andean Latin America	Male	1196.7 (1069.2-1337.4)	12.4 (11.1-13.9)	2831.6 (2163.1-3624.4)	10.2 (7.8-13)	-18.3 (-33.5--3.1)		0.71 (0.32-1.43)	0.01 (0-0.01)	1.73 (0.72-3.42)	0.01 (0-0.01)	-16.1 (-30.1--0.1)	
Andean Latin America	Female	0.7 (0.3-1.4)	0 (0-0)	1.5 (0.7-3.1)	0 (0-0)	-31.1 (-43.6--19.2)		0.72 (0.32-1.43)	0.01 (0-0.01)	1.55 (0.66-3.07)	0.01 (0-0.01)	-28.3 (-40.9--16.6)	
Australasia	Both	5073.2 (3865.6-6334.9)	21.9 (16.7-27.3)	11271.4 (8600.2-14185.2)	21.7 (16.5-27.3)	-0.9 (-7.8-5.3)		3.2 (1.33-6.14)	0.01 (0.01-0.03)	7.07 (2.97-13.73)	0.01 (0.01-0.03)	-1.4 (-24-31.4)	
Australasia	Male	2661.4 (2010.4-3347.7)	25 (18.9-31.4)	6455.6 (4960.5-8220.3)	26.1 (20-33.2)	4.3 (-5.2-14.7)		1.58 (0.59-3.17)	0.01 (0.01-0.03)	3.81 (1.61-7.48)	0.02 (0.01-0.03)	4.1 (-30.6-58.7)	
Australasia	Female	1.6 (0.7-3.2)	0 (0-0)	3.3 (1.4-6.1)	0 (0-0)	-8.1 (-16--1.7)		1.62 (0.68-3.23)	0.01 (0.01-0.03)	3.26 (1.4-6.08)	0.01 (0.01-0.02)	-7.7 (-33.9-25.1)	
Caribbean	Both	1162 (871.9-1460.3)	4.6 (3.4-5.8)	2166.5 (1629.7-2718.4)	4 (3-5)	-13.2 (-19.4--7.4)		0.74 (0.3-1.46)	0 (0-0.01)	1.39 (0.58-2.71)	0 (0-0)	-12.9 (-19--7)	
Caribbean	Male	784.4 (585.4-981.9)	6.4 (4.8-8)	1420.5 (1056.8-1780.6)	5.5 (4.1-6.9)	-13.2 (-19.5--6.1)		0.49 (0.2-0.94)	0 (0-0.01)	0.88 (0.37-1.69)	0 (0-0.01)	-12.9 (-19.7--4.9)	
Caribbean	Female	0.3 (0.1-0.5)	0 (0-0)	0.5 (0.2-1)	0 (0-0)	-10.5 (-18.1--3.2)		0.26 (0.11-0.51)	0 (0-0)	0.51 (0.21-1)	0 (0-0)	-10.6 (-18.2--1.5)	
Central Asia	Both	8148.3 (6268.4-10225.8)	17.3 (13.3-21.7)	14209.6 (10743.3-18126.3)	16.6 (12.5-21.1)	-4.1 (-8.1--0.6)		4.42 (1.86-8.47)	0.01 (0-0.02)	7.54 (3.19-14.4)	0.01 (0-0.02)	-6.1 (-11.6--0.6)	
Central Asia	Male	3205.5 (2455.3-4051.9)	16.8 (12.9-21.2)	6211.3 (4679.6-7951.5)	16.5 (12.4-21.1)	-1.7 (-5.9-2)		1.68 (0.73-3.25)	0.01 (0-0.02)	3.24 (1.38-6.17)	0.01 (0-0.02)	-2.4 (-11.2-7.1)	
Central Asia	Female	2.7 (1.1-5.3)	0 (0-0)	4.3 (1.8-8.3)	0 (0-0)	-5.7 (-11--0.8)		2.74 (1.13-5.27)	0.01 (0-0.02)	4.31 (1.79-8.28)	0.01 (0-0.02)	-8.2 (-14.3--2)	
Central Europe	Both	27244.3 (23910.3-30874.8)	17.4 (15.3-19.8)	49048.8 (38788.9-61690.6)	22.5 (17.8-28.3)	28.9 (14.5-43.5)		15.33 (6.75-30.05)	0.01 (0-0.02)	26.93 (11.15-51.99)	0.01 (0.01-0.02)	25.8 (8.8-43.9)	
Central Europe	Male	12949.7 (11383.8-14748.9)	19.2 (16.9-21.9)	23287.2 (18256.5-29483.1)	24.4 (19.1-30.9)	27.3 (12.4-42.4)		6.87 (3.06-13.54)	0.01 (0-0.02)	12.17 (5.14-24.05)	0.01 (0.01-0.03)	25.5 (5.5-45.7)	
Central Europe	Female	8.5 (3.7-16.7)	0 (0-0)	14.8 (6.1-28.6)	0 (0-0)	30.2 (16.3-43.8)		8.47 (3.67-16.71)	0.01 (0-0.02)	14.75 (6.1-28.61)	0.01 (0-0.02)	25.9 (6.8-45.9)	
Central Latin America	Both	16427.9 (12657.8-20394.6)	20.5 (15.8-25.5)	53118.9 (41073.8-65304.9)	21.1 (16.3-25.9)	2.6 (0.1-5)		8.59 (3.66-16.35)	0.01 (0-0.02)	27.63 (11.91-52.66)	0.01 (0-0.02)	2.1 (-3.9-8.3)	
Central Latin America	Male	8423.6 (6510.8-10508.7)	21.9 (16.9-27.3)	26075.5 (20247.9-32506.3)	22.5 (17.5-28.1)	2.9 (-0.2-5.5)		4.31 (1.91-8.2)	0.01 (0-0.02)	13.19 (5.72-25.43)	0.01 (0-0.02)	1.8 (-7.6-11.7)	
Central Latin America	Female	4.3 (1.8-8.2)	0 (0-0)	14.4 (6.4-27.2)	0 (0-0)	2.8 (-0.8-6.1)		4.28 (1.78-8.19)	0.01 (0-0.02)	14.44 (6.37-27.16)	0.01 (0-0.02)	2.8 (-4.3-10.2)	
Central Sub-Saharan Africa	Both	649.6 (462.1-861)	2.9 (2.1-3.9)	1541.4 (1115.7-2050.1)	2.9 (2.1-3.9)	-1.1 (-7.2-4)		0.34 (0.14-0.69)	0 (0-0)	0.81 (0.33-1.58)	0 (0-0)	-2.1 (-9-4.7)	
Central Sub-Saharan Africa	Male	392.7 (276.1-516.8)	3.8 (2.7-5)	893 (642.4-1181.4)	3.7 (2.6-4.9)	-3.4 (-9.7-3.5)		0.21 (0.09-0.42)	0 (0-0)	0.47 (0.2-0.91)	0 (0-0)	-4.7 (-12.9-4.2)	
Central Sub-Saharan Africa	Female	0.1 (0.1-0.3)	0 (0-0)	0.3 (0.1-0.7)	0 (0-0)	3.5 (-3.9-14.3)		0.14 (0.06-0.27)	0 (0-0)	0.34 (0.14-0.67)	0 (0-0)	2.8 (-6.1-13.7)	
East Asia	Both	19062 (14715.9-23777.1)	2.2 (1.7-2.7)	511623.1 (416764.4-611553.4)	22.1 (18-26.5)	919.6 (847.4-1004.7)		11.68 (4.98-22.57)	0 (0-0)	250.9 (112.88-479.39)	0.01 (0-0.02)	716.2 (638.2-816.4)	
East Asia	Male	9868.8 (7581.8-12372.4)	2.3 (1.8-2.9)	288446.1 (238123.1-341262.2)	25.8 (21.3-30.5)	1027.2 (940.2-1136.6)		5.75 (2.49-11.17)	0 (0-0)	135.77 (61.51-252.8)	0.01 (0.01-0.02)	811.3 (694.8-939.1)	
East Asia	Female	5.9 (2.5-11.4)	0 (0-0)	115.1 (50.7-220.7)	0 (0-0)	809 (749-879.4)		5.93 (2.49-11.42)	0 (0-0)	115.14 (50.75-220.66)	0.01 (0-0.02)	626.9 (560.1-709.4)	
Eastern Europe	Both	34939.7 (27507.3-43645.7)	12.1 (9.5-15.1)	55081.2 (42339.5-69658.5)	15.1 (11.6-19)	24.2 (20-27.3)		20.36 (8.6-39.25)	0.01 (0-0.01)	31.31 (13.08-60.18)	0.01 (0-0.02)	21.1 (15.9-26.8)	
Eastern Europe	Male	11979.7 (9302-14890)	11.7 (9.1-14.6)	20954.9 (16054.5-26531.6)	14.9 (11.4-18.8)	26.8 (20.8-32.6)		6.61 (2.83-12.67)	0.01 (0-0.01)	11.38 (4.83-21.99)	0.01 (0-0.02)	24.8 (14.6-36)	
Eastern Europe	Female	13.8 (5.7-26.7)	0 (0-0)	19.9 (8.2-38.5)	0 (0-0)	22.9 (17.9-27.3)		13.76 (5.74-26.75)	0.01 (0-0.01)	19.93 (8.2-38.5)	0.01 (0-0.02)	19.8 (14.1-25.6)	
Eastern Sub-Saharan Africa	Both	1870.4 (1358.1-2443.2)	2.6 (1.9-3.4)	4038.8 (2914.1-5293.5)	2.5 (1.8-3.3)	-2.8 (-4.9--1.3)		1.02 (0.42-2.02)	0 (0-0)	2.2 (0.92-4.32)	0 (0-0)	-3.3 (-5.6--1.2)	
Eastern Sub-Saharan Africa	Male	1153.5 (851.1-1496.9)	3.2 (2.4-4.2)	2353.5 (1719.4-3076.9)	3.1 (2.3-4.1)	-3.2 (-6--1)		0.63 (0.26-1.23)	0 (0-0)	1.27 (0.53-2.48)	0 (0-0)	-3.6 (-6.9--0.5)	
Eastern Sub-Saharan Africa	Female	0.4 (0.2-0.8)	0 (0-0)	0.9 (0.4-1.8)	0 (0-0)	0.5 (-1.7-3)		0.4 (0.16-0.79)	0 (0-0)	0.93 (0.38-1.83)	0 (0-0)	-0.1 (-3.3-3.4)	
High-income Asia Pacific	Both	3999.4 (3200.6-4895.5)	1.9 (1.6-2.4)	16124.1 (13106.4-19609.7)	3.9 (3.2-4.7)	100 (83.9-118.4)		3.76 (1.58-7.27)	0 (0-0)	14.07 (5.76-27.38)	0 (0-0.01)	85.5 (68.5-101.7)	
High-income Asia Pacific	Male	1936.7 (1517.8-2395.6)	2.1 (1.7-2.6)	7296.6 (5879.4-8986.4)	3.8 (3.1-4.7)	79.3 (65.7-96.3)		1.7 (0.71-3.28)	0 (0-0)	6.2 (2.56-12.2)	0 (0-0.01)	73.3 (57.9-87.9)	
High-income Asia Pacific	Female	2.1 (0.9-4)	0 (0-0)	7.9 (3.3-15.4)	0 (0-0)	119.5 (101.7-141.8)		2.06 (0.86-3.97)	0 (0-0)	7.87 (3.29-15.36)	0 (0-0.01)	96 (76.6-114.6)	
High-income North America	Both	579286.8 (447239.9-714021)	169.7 (131-209.2)	2581376.3 (2317348.6-2864288.1)	389.3 (349.5-432)	129.4 (98.2-177.9)		243.52 (103.99-459.56)	0.07 (0.03-0.13)	1022.57 (482.49-1962.2)	0.15 (0.07-0.3)	116.2 (87-160.8)	
High-income North America	Male	301495.5 (234217.8-370108.9)	204 (158.5-250.4)	1567088.5 (1401530.4-1736787.8)	508.6 (454.9-563.7)	149.3 (115.5-202.7)		122.37 (53-229.12)	0.08 (0.04-0.16)	611.21 (287.89-1155.6)	0.2 (0.09-0.38)	139.6 (106.4-185.8)	
High-income North America	Female	121.2 (51.2-232)	0.1 (0-0.1)	411.4 (193.2-791.4)	0.1 (0.1-0.2)	99.1 (72.2-141.7)		121.15 (51.15-231.97)	0.06 (0.03-0.12)	411.36 (193.19-791.37)	0.12 (0.05-0.22)	85.1 (59.6-125)	
North Africa and Middle East	Both	8297.2 (6785.9-10043)	5 (4.1-6)	19081 (14379.5-23867.7)	4.2 (3.2-5.3)	-14.7 (-22.3--8.7)		5.12 (2.28-9.91)	0 (0-0.01)	12.06 (5.03-22.69)	0 (0-0.01)	-12.7 (-21.4--6.8)	
North Africa and Middle East	Male	4988.2 (4079.3-6034.5)	5.9 (4.8-7.1)	11568.8 (8802.6-14454.9)	5.1 (3.9-6.4)	-13.5 (-21.1--7.3)		3.01 (1.32-5.83)	0 (0-0.01)	7.13 (3-13.39)	0 (0-0.01)	-11.5 (-18.9--5)	
North Africa and Middle East	Female	2.1 (0.9-4.1)	0 (0-0)	4.9 (2-9.4)	0 (0-0)	-16.4 (-26.3--9.8)		2.12 (0.94-4.1)	0 (0-0.01)	4.92 (2.04-9.35)	0 (0-0)	-14.2 (-23.5--6.5)	
Oceania	Both	0.8 (0.3-1.7)	0 (0-0.1)	2.1 (0.7-4.4)	0 (0-0.1)	-0.1 (-12.9-15.3)		0 (0-0)	0 (0-0)	0 (0-0)	0 (0-0)	-0.5 (-15.1-14.1)	
Oceania	Male	0.4 (0.1-0.9)	0 (0-0.1)	1.1 (0.3-2.3)	0 (0-0.1)	-0.4 (-4.8-3.5)		0 (0-0)	0 (0-0)	0 (0-0)	0 (0-0)	-0.7 (-10.3-10.1)	
Oceania	Female	0 (0-0)	0 (0-0)	0 (0-0)	0 (0-0)	0.3 (-25.5-31.3)		0 (0-0)	0 (0-0)	0 (0-0)	0 (0-0)	-0.2 (-28-27.7)	
South Asia	Both	4311.4 (3037.8-5940.8)	0.8 (0.5-1.1)	12390.3 (8922-16595)	0.8 (0.6-1.1)	9.9 (3.7-18)		2.37 (0.95-4.68)	0 (0-0)	6.8 (2.77-13.26)	0 (0-0)	9.9 (3.2-17.8)	
South Asia	Male	2404.5 (1697.4-3309.4)	0.8 (0.6-1.1)	6428.9 (4657.9-8628.9)	0.9 (0.6-1.2)	9.6 (4-17.4)		1.3 (0.53-2.55)	0 (0-0)	3.49 (1.42-6.82)	0 (0-0)	10.3 (3.5-17.5)	
South Asia	Female	1.1 (0.4-2.1)	0 (0-0)	3.3 (1.3-6.5)	0 (0-0)	11.2 (4-20.2)		1.07 (0.43-2.13)	0 (0-0)	3.31 (1.33-6.52)	0 (0-0)	10.2 (2.6-19.5)	
Southeast Asia	Both	3801.2 (3218.6-4550.8)	1.5 (1.3-1.8)	6825 (4914.8-9009.5)	1 (0.7-1.3)	-33.6 (-43.5--25.2)		2.38 (1.04-4.55)	0 (0-0)	4.35 (1.74-8.38)	0 (0-0)	-32.6 (-43.4--23.5)	
Southeast Asia	Male	1919.1 (1602.6-2300.6)	1.6 (1.4-2)	3527.4 (2534.7-4662.6)	1.1 (0.8-1.5)	-31.6 (-41.5--22.5)		1.13 (0.49-2.15)	0 (0-0)	2.12 (0.85-4.1)	0 (0-0)	-30.3 (-40.6--20.8)	
Southeast Asia	Female	1.2 (0.5-2.4)	0 (0-0)	2.2 (0.9-4.3)	0 (0-0)	-35.6 (-45.6--27)		1.25 (0.54-2.4)	0 (0-0)	2.22 (0.89-4.32)	0 (0-0)	-34.6 (-45.9--25.1)	
Southern Latin America	Both	7911.5 (6732.4-9147.8)	16.9 (14.4-19.6)	15302.4 (11840.9-19189.5)	17.6 (13.7-22.1)	4.1 (-10.3-17.8)		4.78 (2.13-9.3)	0.01 (0-0.02)	9.23 (3.79-17.61)	0.01 (0-0.02)	3.8 (-15.8-26.8)	
Southern Latin America	Male	4054.4 (3471.2-4679.3)	19.5 (16.7-22.5)	7859.8 (6104.3-9895.6)	20.3 (15.7-25.5)	4.2 (-10.2-20.9)		2.31 (1.03-4.53)	0.01 (0-0.02)	4.48 (1.82-8.5)	0.01 (0-0.02)	4.3 (-22.7-36.3)	
Southern Latin America	Female	2.5 (1.1-5)	0 (0-0)	4.7 (2-9.6)	0 (0-0)	4 (-11.6-22.3)		2.48 (1.09-4.99)	0.01 (0-0.02)	4.75 (1.97-9.55)	0.01 (0-0.02)	3.4 (-17.1-29.8)	
Southern Sub-Saharan Africa	Both	2918.6 (2170.4-3725.2)	11.2 (8.3-14.3)	7700.8 (5661.6-9855.5)	13.4 (9.9-17.2)	19.9 (15.8-23.9)		1.5 (0.62-2.89)	0.01 (0-0.01)	3.89 (1.62-7.48)	0.01 (0-0.01)	17.8 (13.4-21.7)	
Southern Sub-Saharan Africa	Male	1593.6 (1188-2042.2)	14.2 (10.6-18.2)	4040.8 (2957.7-5214.8)	17 (12.4-21.9)	19.6 (14.8-24.5)		0.8 (0.33-1.54)	0.01 (0-0.01)	2.01 (0.83-3.86)	0.01 (0-0.02)	17.7 (12.6-23.4)	
Southern Sub-Saharan Africa	Female	0.7 (0.3-1.4)	0 (0-0)	1.9 (0.8-3.6)	0 (0-0)	22.2 (17.2-26.9)		0.7 (0.29-1.36)	0 (0-0.01)	1.88 (0.79-3.62)	0.01 (0-0.01)	19.5 (14-24.5)	
Tropical Latin America	Both	42195.5 (36168.3-48282.3)	47.3 (40.5-54.1)	68794.7 (54413.2-84606.7)	26.4 (20.8-32.4)	-44.3 (-50--38.7)		17.83 (7.92-33.2)	0.02 (0.01-0.04)	31.55 (13.74-61.06)	0.01 (0.01-0.02)	-39.5 (-49.5--27.5)	
Tropical Latin America	Male	22980.2 (19815.4-26381)	55.1 (47.5-63.3)	35117.8 (27934.2-43336.4)	29.8 (23.7-36.8)	-45.9 (-51.6--40)		9.58 (4.16-18.15)	0.02 (0.01-0.04)	15.57 (6.88-30.14)	0.01 (0.01-0.03)	-42.4 (-54.3--26.1)	
Tropical Latin America	Female	8.2 (3.8-15.9)	0 (0-0)	16 (6.9-30.8)	0 (0-0)	-41.9 (-47.8--36)		8.25 (3.79-15.91)	0.02 (0.01-0.03)	15.98 (6.93-30.83)	0.01 (0-0.02)	-35.7 (-49.6--19.3)	
Western Europe	Both	170168.2 (149768.5-194060.2)	29.7 (26.2-33.9)	276330.7 (216026.4-346573)	31.4 (24.6-39.4)	5.7 (-6.6-17.1)		99.89 (43.8-197.66)	0.02 (0.01-0.03)	157.34 (64.75-295.34)	0.02 (0.01-0.03)	2.6 (-11.2-16.6)	
Western Europe	Male	87468.1 (77324.7-99388.5)	35.7 (31.6-40.6)	151997 (116973.4-192761.6)	37.4 (28.8-47.4)	4.7 (-8-17.4)		48.14 (21.39-93.71)	0.02 (0.01-0.04)	82 (34.25-152.29)	0.02 (0.01-0.04)	2.6 (-15-22.4)	
Western Europe	Female	51.7 (22.9-101.8)	0 (0-0)	75.3 (30.9-144.9)	0 (0-0)	4.2 (-7.5-15.4)		51.75 (22.89-101.76)	0.02 (0.01-0.03)	75.34 (30.93-144.91)	0.02 (0.01-0.03)	0.9 (-13.7-18.3)	
Western Sub-Saharan Africa	Both	1574.9 (1114.2-2102.9)	1.9 (1.3-2.5)	3393.5 (2393.5-4567.3)	1.8 (1.3-2.4)	-3.2 (-5.2--1.6)		0.91 (0.38-1.82)	0 (0-0)	1.94 (0.79-3.84)	0 (0-0)	-4.6 (-6.8--2.6)	
Western Sub-Saharan Africa	Male	986.2 (698.4-1316.2)	2.2 (1.6-3)	2030.5 (1435-2703.7)	2.3 (1.6-3)	2.2 (0.5-4.1)		0.58 (0.24-1.14)	0 (0-0)	1.17 (0.48-2.3)	0 (0-0)	0.6 (-1.7-3.4)	
Western Sub-Saharan Africa	Female	0.3 (0.1-0.7)	0 (0-0)	0.8 (0.3-1.5)	0 (0-0)	-5.7 (-10--1.9)		0.34 (0.14-0.66)	0 (0-0)	0.77 (0.31-1.55)	0 (0-0)	-6.6 (-11.3--2.2)	

### Trends in national burden of 55+ year BCC from 1990 to 2021

In 1990, the highest number of BCC cases among the elderly were reported in the United States of America (572,530 cases, range 442,328–705,463), Brazil (42,195 cases, range 36,167–48,281), and France (34,021 cases, range 30,344–37,645). In 2021, the highest number of BCC cases were reported in the United States of America (2,562,722 cases, range 2,301,511–2,842,198), China (511,566 cases, range 416,717–611,458), and Brazil (68,793 cases, range 54,410–84,605). In 1990, the highest age-standardized incidence rates (ASIR) were in the United States of America (1,091 per 100,000, range 843–1,345), Ireland (385 per 100,000, range 342–425), and Switzerland (350 per 100,000, range 335–365). The lowest ASIRs were in Bhutan (0.03 per 100,000, range 0.008–0.06), Nepal (0.03 per 100,000, range 0.008–0.058), and Bangladesh (0.03 per 100,000, range 0.009–0.059). In 2021, the highest age-standardized incidence rates (ASIR) were in the United States of America (2,556 per 100,000, range 2,296–2,835), Sweden (271 per 100,000, range 219–333), and Greenland (264 per 100,000, range 197–333). The lowest ASIRs were in Nepal (0.03 per 100,000, range 0.009–0.058), Bangladesh (0.03 per 100,000, range 0.009–0.058), and Bhutan (0.03 per 100,000, range 0.009–0.057).

**Figure 2:**
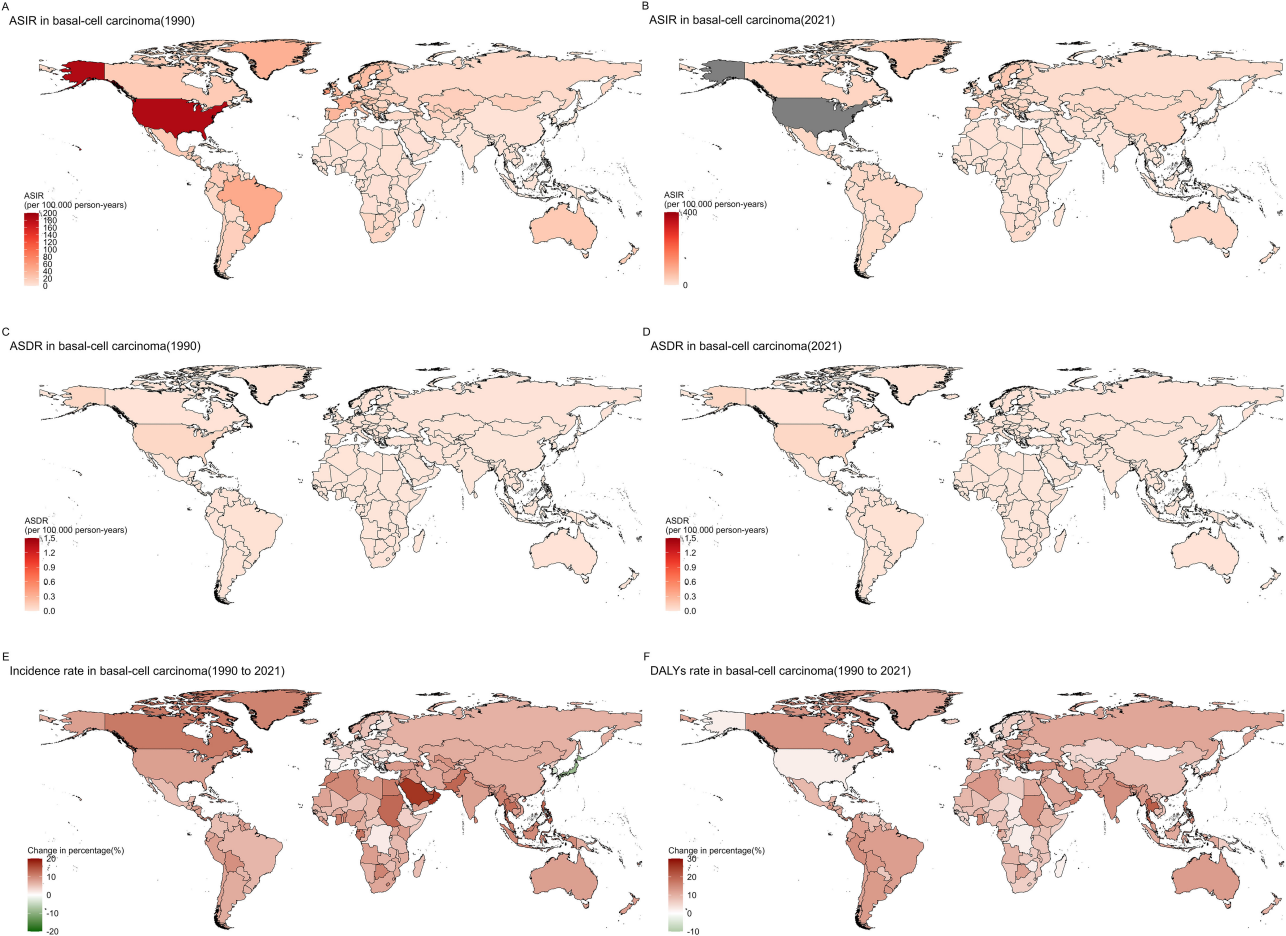
Country trends of 55+ year BCC from 1990 to 2021. **(A)** ASIR in basal-cell carcinoma(1990); **(B)** ASIR in basal-cell carcinoma(2021); **(C)** ASDR in basal-cell carcinoma(1990); **(D)** ASDR in basal-cell carcinoma(2021); **(E)** Incidence rate in basal-cell carcinoma(1990 to 2021); **(F)** DALYs rate in basal-cell carcinoma(1990 to 2021).

Among 198 countries and territories, the age-standardized incidence rate (ASIR) of BCC in the elderly population has shown an increasing trend. The most significant increase was in the United States of America, rising from 1091.3 per 100,000 (843.1–1344.7) in 1990 to 2556.4 per 100,000 (2835.2–2295.8) in 2021. Sweden also saw an increase from 185.1 per 100,000 (141.3–238.9) in 1990 to 271.4 per 100,000 (219.3–333.0) in 2021. The smallest increase was in Nepal, rising from 0.025 per 100,000 (0.009–0.058) in 1990 to 0.03 per 100,000 (0.009–0.057) in 20 (**Figure 2**, **Supplementary Data 2**).

### The burden trend by age group from1990 to 2021

**Figure 3:**
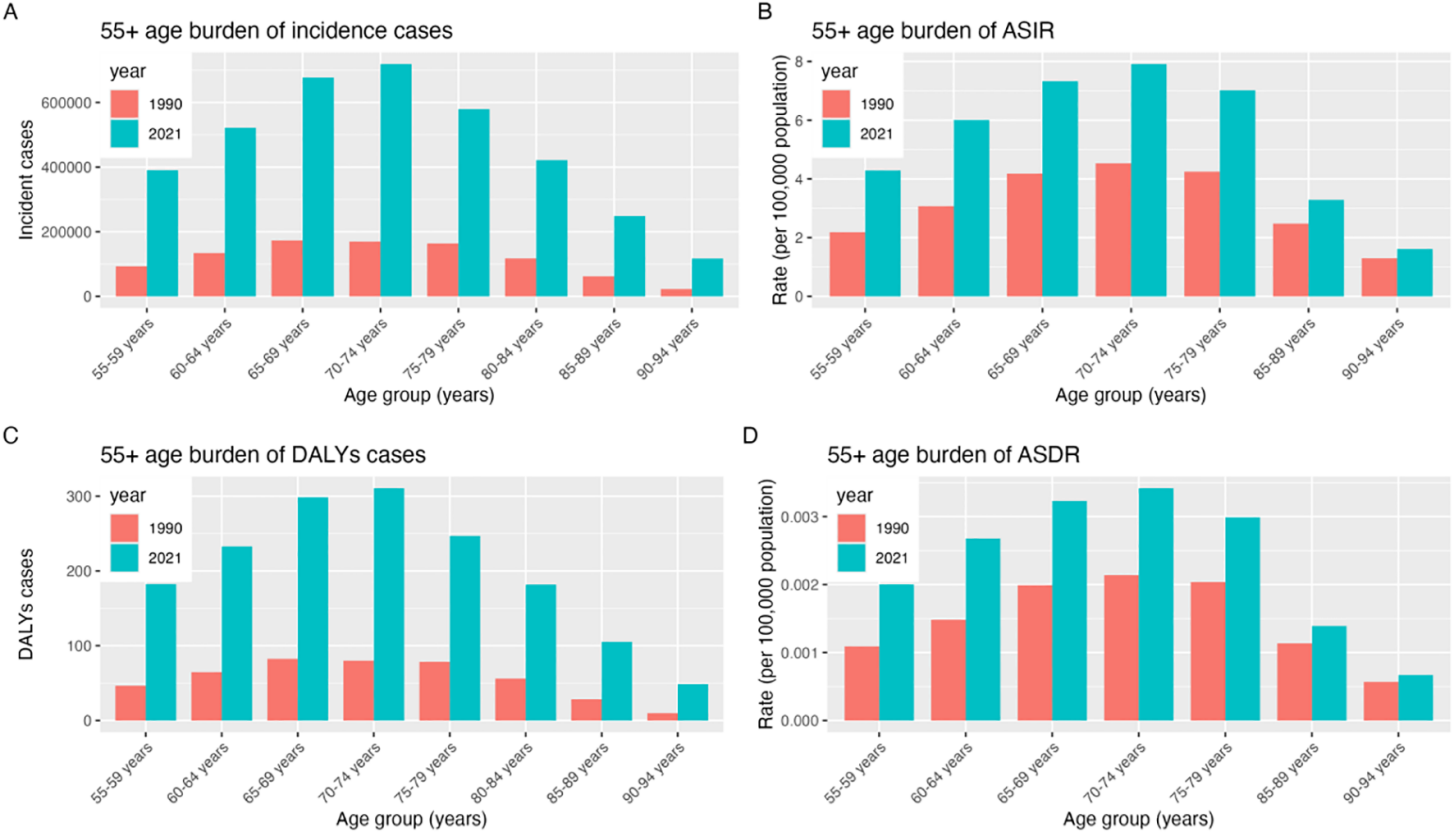
Differences by age group of 55+ year BCC **(A)** 55+ age burden of incidence cases; **(B)** 55+ age burden of ASIR; **(C)** 55+ age burden of DALYs cases; **(D)** .55+ age burden of ASDR.

Comparison of age-standardized incidence rates (ASIR), DALYs, and age-standardized DALY rates (ASDR) for BCC globally in 1990 and 2021. The ASIR and DALYs for BCC increased with age, with the highest proportion of patients in the 70–74 years age group. In 2021, the number of new BCC cases, DALYs, ASIR, and ASDR were all higher than in 1990 (**Figure 3**, **Supplementary Data 3**).

### The burden trend by SDI group at different time periods

**Figure 4:**
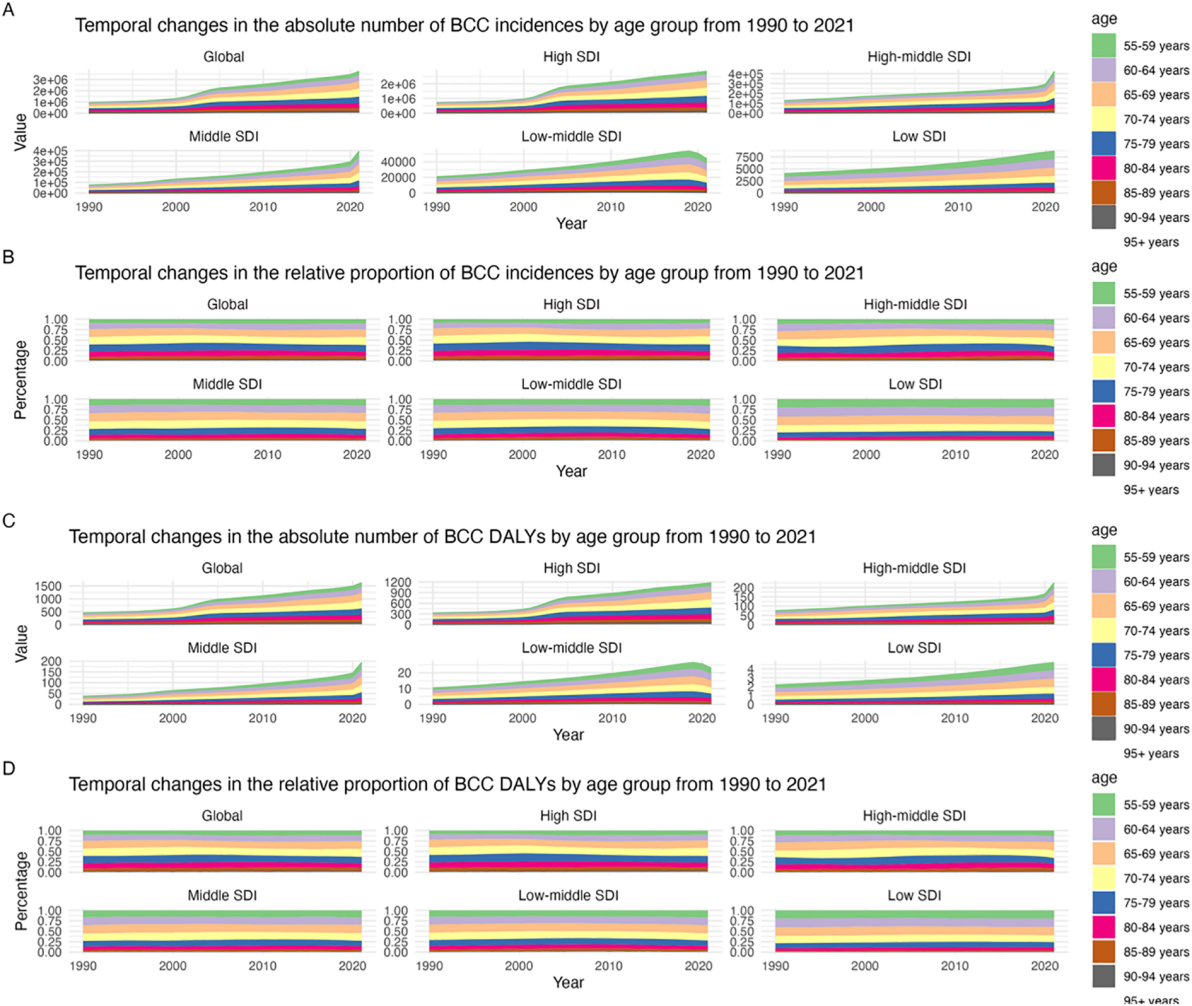
Distribution of disease burden of Basal cell carcinoma from 1990 to 2021 **(A)** Temporal changes in the absolute number of BCC incidences by age group from 1990 to 2021; **(B)** Temporal changes in the relative proportion of BCC incidences by age group from 1990 to 2021; **(C)** Temporal changes in the absolute number of BCC DALYs by age group from 1990 to 2021; **(D)** Temporal changes in the relative proportion of BCC DALYs by age group from 1990 to 2021.

From 1990 to 2021, the number of new cases of BCC among individuals aged 55 and older increased globally, rising from approximately 500,000 cases in 1990 to nearly 1.5 million cases in 2021. In different SDI countries, the increase in the number of new cases among 55+ BCC patients was smaller in high SDI countries and larger in low SDI countries. In terms of relative proportion, the proportion of 55+ BCC patients remained relatively stable globally but varied among different SDI countries. For example, in high SDI countries, the relative proportion of 55+ BCC patients was about 15% in 2021, while in low SDI countries, it was close to 50%. In terms of DALYs for BCC, the number of 55+ BCC patients also increased globally from approximately 20,000 in 1990 to nearly 60,000 in 2021. In different SDI countries, the increase in DALYs among 55+ BCC patients was smaller in high SDI countries and larger in low SDI countries. In terms of relative proportion, the proportion of DALYs for 55+ BCC patients remained relatively stable globally but varied among different SDI countries. For example, in high SDI countries, the relative proportion of DALYs for 55+ BCC patients was about 10% in 2021, while in low SDI countries, it was close to 40% (**Figure 4**, **Supplementary Data 4**).

### 55+ BCC projections of trends in the burden of disease in 2050

**Figure 5:**
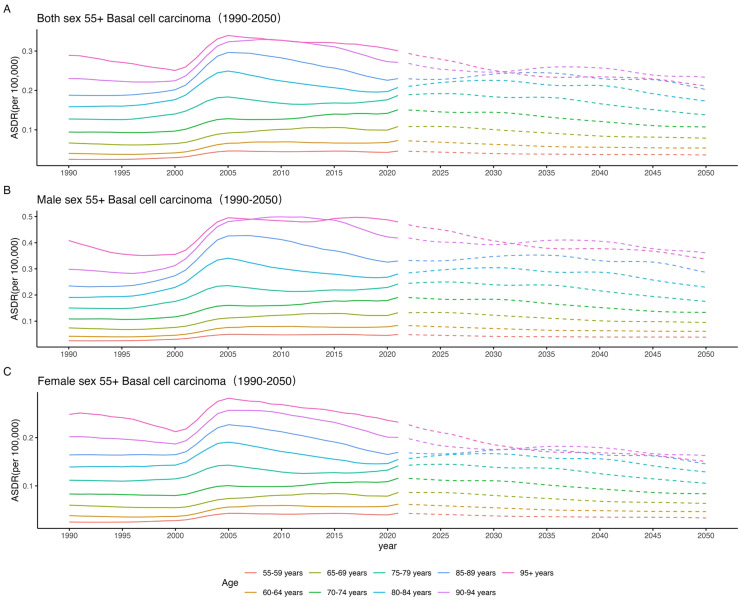
55+BCC projections of trends in the burden of disease in 2050; **(A)** Both sex; **(B)** Male sex; **(C)** Female sex.

Since 2021, the disease burden of 55+ BCC patients has shown varying upward trends across different genders. Specifically, as age increases, the disease burden of 55+ BCC patients significantly rises in the older age groups (e.g., 85-89, 90-94, 95+ years), indicating a higher concentration of BCC burden in these age groups. Meanwhile, the disease burden of BCC also increases in the middle and lower age groups (e.g., 55-59, 60-64, 65–69 years). By 2050, it is projected that 55+ BCC patients will have a higher disease burden across all age groups, with the most significant increases expected in the 90–94 and 95+ age groups. This suggests that the disease burden of BCC may further increase in the coming decades, with the trend being particularly pronounced in the elderly population. In males, the disease burden of 55+ BCC patients has shown a clear upward trend since 2021. Specifically, the disease burden significantly increases in the older age groups (e.g., 85-89, 90-94, 95+ years), with the ASDR expected to reach a higher level in the 95+ age group by 2050. The disease burden also rises in the middle and lower age groups (e.g., 55-59, 60-64, 65–69 years), but the increase is relatively smaller. In females, the disease burden of 55+ BCC patients has also shown an upward trend since 2021, but compared to males, the increase is more pronounced in the older age groups. Specifically, the ASDR for female BCC patients in the 90–94 and 95+ age groups is expected to reach a higher level by 2050, with a larger increase. Although the disease burden also increases in the middle and lower age groups (e.g., 55-59, 60-64, 65–69 years), the increase is relatively more gradual (**Figure 5**, **Supplementary Data 5**).

## Discussion

BCC is one of the most common skin malignancies globally, with varying incidence rates reported across different regions. A systematic review analyzing data from 38 different countries between 1955 and 2007 found that Australia had the highest annual incidence rate (>1000 per 100,000 person-years), while some regions in Africa had the lowest incidence rate (<1/100,000 person-years) (13, 14). Over the past 30 years, there has been no literature specifically reporting on the incidence of BCC in the elderly population globally and its distribution across different countries and regions, although there have been reports on the overall incidence of skin cancer (15). Studies on the burden of BCC in the elderly are rare. Therefore, analyzing factors such as the incidence rate, age of onset, gender distribution, incidence rate distribution across different countries and regions, and disease burden comparison for elderly BCC globally from 1990 to 2021 is of significant importance to clinical practice, epidemiology, and public health.

### Analysis of temporal trends

From the data presented, we observe that from 1990 to 2021, the number of new cases of BCC among the elderly population globally showed a certain fluctuation, with a continuous increase from 1990 to 2005 and a stabilization from 2005 to 2021. The fluctuations are mainly influenced by changes in lifestyle and environment, ultraviolet (UV) exposure, population aging, genetic factors, and medical resources and diagnostic capabilities. Changes in lifestyle and environment, such as increased use of tobacco, alcohol, overweight, environmental pollution, lack of exercise, and emotional stress, which are cancer risk factors, have significantly increased the risk of cancer. After 2005, as awareness of the hazards of UV exposure increased, people may have adopted more preventive measures, such as using sunscreen and avoiding sun exposure during peak hours, which may have helped stabilize or even reduce the incidence of BCC. Therefore, as public awareness of skin cancer and its association with UV exposure increases, more preventive measures will be taken, which will to some extent reduce the incidence of the disease. In the future, screening for BCC should focus on elderly patients over 60 years old and implement relevant preventive measures.

### Analysis of gender trends

According to global BCC data, the incidence rates in males are consistently higher than those in females across different regions. Possible reasons include: First, outdoor work and recreational activities increase exposure to ultraviolet (UV) radiation, which is a major risk factor for BCC. Men are more likely to be exposed to UV radiation due to their higher engagement in outdoor work. Second, men are less likely than women to use sunscreen, hats, and other protective gear, which increases their risk of developing BCC.

### Analysis of global trends

From 1990 to 2021, the incidence and DALYs of BCC among the elderly population globally showed an upward trend, with a significant increase from 1990 to 2005 and a stabilization from 2005 to 2021. The reasons for this trend are multifaceted, including genetic, environmental, and medical factors. The occurrence of BCC is associated with genetic factors, with certain genetic disorders such as Gorlin syndrome, xeroderma pigmentosum, and ocular albinism increasing the risk of developing BCC (16–21).Abnormalities in the PTCH gene, which controls cell proliferation and differentiation, may be associated with the development of BCC (22, 23). In terms of environmental factors, UV exposure is an extremely important cause of BCC, with long-term sun exposure particularly affecting exposed areas of the body, especially the face (24, 25). UV exposure and genetic mutations play a major role, with the abnormal activation of the Hedgehog (Hh) signaling pathway being considered the most important mechanism in the development of BCC (26, 27). Importantly, the carcinogenic effects of UV radiation are further amplified in the elderly population. Aging is associated with impaired skin barrier function and diminished DNA repair capacity, which compromise the skin’s ability to counteract UV-induced DNA damage. These age-related changes may exacerbate the accumulation of mutations in key regulatory genes such as PTCH, thereby promoting abnormal activation of the Hedgehog signaling pathway and increasing the risk of BCC initiation. Consequently, individuals aged ≥55 years represent a high-risk group for BCC development. This underscores the necessity of targeted sun protection interventions in the elderly, not only to reduce UV exposure but also to mitigate the molecular vulnerabilities associated with aging.Long-term intake of inorganic arsenic or arsenic-rich water and food may also be associated with the development of BCC (28). Medical factors include ionizing radiation, with long-term occupational exposure to low-to-moderate levels of ionizing radiation also increasing the risk of BCC (29). With advancements in pharmacotherapy, the increased use of immunosuppressants has led to a higher risk of BCC in individuals with long-term immune suppression, such as kidney transplant recipients, whose overall incidence of BCC is 7 to 16 times that of the general population (30). Secondly, population aging is a significant factor, as BCC is an indolent skin tumor that typically takes decades to develop, and thus most cases occur in individuals over 60 years old. The incidence of BCC is associated with geographical latitude and negatively correlated with skin pigmentation (31–33).The risk of developing BCC increases with age. The use of dermatoscopy and other imaging techniques has improved diagnostic accuracy, potentially leading to an increase in reported cases (34–37).These factors collectively contribute to the increase in the incidence of BCC among the elderly and changes in age-standardized incidence rates. UV exposure and genetic factors play a significant role in the development of BCC, while population aging and advancements in medical technology also influence the incidence statistics.

### Analysis of SDI quintile trends

At the regional level, from 1990 to 2021, the incidence rates across the five SDI quintiles increased progressively from Low SDI to High SDI, with the highest incidence in High SDI regions. The reasons for the high incidence in High SDI regions may include the following: First, people in these regions tend to engage in more outdoor activities and may have less ozone layer protection or stronger sunlight exposure, leading to increased UV exposure and a higher incidence of skin cancers such as BCC (38). Second, High SDI regions typically have higher life expectancy and more severe population aging than other regions, which increases the risk of age-related cancers (39). Additionally, these regions may have more industrial activities, increasing residents’ exposure to various risk factors. Moreover, High SDI regions usually have better medical resources and diagnostic capabilities, which may lead to more cases being detected and reported. Finally, these regions may face more environmental pollution, including cancer risk factors such as tobacco, alcohol, overweight, environmental pollution, lack of exercise, and emotional stress. These factors collectively contribute to the relatively higher cancer incidence in High SDI regions.

### The burden trend by SDI group at different time periods

Globally, from 1990 to 2021, the number of new cases and DALYs of BCC among individuals aged 55 and older showed an upward trend, with significant differences across countries with different SDI levels. This phenomenon reflects the complex situation of global population structure changes, uneven distribution of medical resources, and the shift in disease burden. First, the intensification of global population aging is an important background factor for the increase in the number of 55+ BCC patients. As life expectancy increases and the proportion of the elderly population rises, the incidence of age-related diseases naturally increases. The increase in BCC cases is closely related to changes in population structure. However, the differences between countries with different SDI levels reveal deeper issues. The increase in the number of cases and DALYs is more significant in low SDI countries, which may be related to the relative scarcity of medical resources and insufficient disease prevention and control capabilities in these countries (39–41). In contrast, although high SDI countries have abundant medical resources, the relative proportion of BCC is still high due to more severe population aging, indicating that even in areas with better medical conditions, age-related diseases remain a significant public health challenge. This is consistent with the findings of spinal cord injury studies, which also show that population growth and aging are the main driving forces behind changes in the burden of spinal cord injuries. From a public health policy perspective, this trend suggests that countries need to develop targeted response strategies based on their SDI levels. Low SDI countries should focus on strengthening basic medical infrastructure, improving disease screening, and early intervention capabilities to slow the rapid growth of age-related diseases such as BCC. High SDI countries need to focus on the long-term management and rehabilitation of age-related diseases, optimize the allocation of medical resources, and address the growing burden of age-related diseases. Moreover, global cooperation and experience sharing are crucial. Efforts by international organizations and non-governmental organizations to promote the sharing of medical technology and resources can help narrow the health gap between countries with different SDI levels and jointly address the challenges posed by global population aging (42).

### Projections of trends in the burden of disease for 55+ BCC in 2050

Since 2021, the disease burden of 55+ BCC patients has shown varying upward trends across different genders and age groups, revealing the severe challenges faced by public health in the future. In terms of age distribution, the disease burden of BCC is particularly concentrated in the older age groups (e.g., 85-89, 90-94, 95+ years), with significant increases. This trend may be related to the physiological decline, accumulation of chronic diseases, and increased use of medical resources in the elderly population. As global population aging intensifies and the proportion of the elderly increases, the high burden of BCC is expected to worsen. By 2050, the disease burden of BCC is projected to reach higher levels across all age groups, particularly in the 90–94 and 95+ age groups, posing significant pressure on the healthcare and social security systems. In terms of gender differences, the disease burden of BCC is increasing for both males and females, but the increase is more pronounced in older females. This may be related to the longer average life expectancy of women, longer accumulation of chronic diseases, and the combined effects of physiological and social factors. The increased disease burden in older females highlights the need for future public health policies to pay more attention to the health needs of women, especially in geriatric care and chronic disease management.

Additionally, the disease burden of BCC in middle-aged groups (such as 55-59, 60-64, and 65–69 years) has also shown an upward trend, albeit with smaller increases. This suggests that the age of onset for BCC may be gradually decreasing, which warrants attention. Over the next few decades, changes in population structure and lifestyle are expected to further increase the disease burden of BCC, particularly in the elderly population. In conclusion, future public health policies should focus on the BCC disease burden in the elderly and women, strengthen early screening, prevention, and intervention measures, and optimize the allocation of medical resources to address the ongoing increase in BCC disease burden. Additionally, further research into the pathogenesis and risk factors of BCC is needed to develop more effective treatments and health management strategies.

The 30-year rise in elderly BCC incidence and DALYs is driven not only by ultraviolet exposure, population ageing and immunosuppressant use, but also by heterogenous health-system responses that the GBD lens cannot fully capture: national screening policies range from systematic dermoscopy programmesin Australia to absent formal guidelines in much of sub-Saharan Africa, while high-income countries’ higher recorded rates partly reflect reimbursed skin-clinic visits and pathological confirmation of every excised lesion. Conversely, under- and miscoding of BCC as “other malignant skin” (ICD-10 C44.9, C46.-) remains common where pathology services are scarce; GBD 2021 attempts to separate BCC from SCC and rarer NMSC through a Bayesian meta-regression that redistributes garbage codes and incorporates cancer-registry flag variables, yet this modelling step still depends on the very records that are missing or biased in low-SDI settings. Consequently, the apparent surge in North-West Europe and the relative plateau in parts of South-East Asia may mirror differential detection capacity rather than true epidemiological divergence. Future burden estimates should therefore be triangulated with health-facility audits, claims-data validation studies and geospatial UV-dosimetry, while policy discussions must shift from crude incidence rankings to context-specific prevention packages—shade-planning regulations, opportunistic screening for high-risk outdoor workers, and subsidised topical therapies—that simultaneously reduce under-diagnosis in resource-limited regions and overtreatment in well-served ones.

## Limitations and future prospects

This study has key limitations to note. First, full dependence on GBD data brings constraints: roughly one-tenth of countries lack non-melanoma skin cancer (NMSC) registries (cross-regional data borrowing may skew BCC counts), variable pathology standards misclassify up to a quarter of BCC cases (only partly corrected by GBD redistribution), 2018-median data misses post-COVID-19 tele-dermatology surges (distorting recent detection trends), and national aggregates hide sub-national extremes (e.g., Queensland’s BCC incidence is 2.3-fold higher than Australia’s national estimate). Second, no primary clinical data limits analysis of BCC subtypes, treatments (cryotherapy, PDT, imiquimod, 5-fluorouracil), or patient outcomes, preventing assessment of treatment’s impact on burden metrics. Third, regional BCC differences may reflect diagnostic/reporting disparities rather than true epidemiology—low SDI countries face under-diagnosis, under-reporting, and poor pathology infrastructure, risking underestimated burden, and emerging studies show some low-SDI regions’ actual incidence may approach high-SDI areas when accounting for under-ascertainment. Fourth, BCC is often grouped with other NMSC in datasets, diluting its trends and biasing case capture/burden assessment amid diverse treatments. Thus, GBD estimates are low-resolution proxies. Future work should integrate clinical cohort data with GBD, audit regional registries (especially low-SDI), conduct sensitivity analyses, advocate for disaggregated NMSC data, and build treatment-stratified models; decision-makers should validate GBD insights with local data (sentinel networks, UV-spatial layers) before resource allocation.

## Conclusion

In summary, over the past 30 years, the number of new cases of BCC among the elderly has generally increased globally, which is related to the growth of the global population. The incidence and DALYs of BCC have increased in all global regions, which on one hand reflects the impact of climate and environmental changes on the disease, and on the other hand, highlights the significant role of population aging and the wider use of immunosuppressants. On the other hand, improvements in medical resources have led to earlier diagnosis and treatment of BCC for more patients, especially in developed countries. These high-income regions typically have more advanced medical resources and diagnostic technologies, which may have increased the detection and reporting rates of BCC, thereby raising the incidence data. Meanwhile, the implementation of various treatments and surgeries has also increased the disease burden of BCC, particularly in developed countries. By analyzing the potential causes and disease burden of BCC, we can enhance global preventive measures against BCC in the future, reduce the occurrence and burden of this disease, which is a favorable measure for preventing BCC, and also decrease the use of global health resources.

## Data Availability

The original contributions presented in the study are included in the article/**Supplementary Material**, further inquiries can be directed to the corresponding author/s.

## References

[1] Nahhas AF Scarbrough CA Trotter S . A review of the global guidelines on surgical margins for nonmelanoma skin cancers. J Clin Aesthet Dermatol. (2017) 10:37–46., PMID: 28458773 PMC5404779

[2] Fraga SD Besaw RJ Murad F . Complete margin assessment versus sectional assessment in surgically excised high-risk keratinocyte carcinomas: A systematic review and meta-analysis. Dermatol Surg. (2022) 48:704–10. doi: 10.1097/DSS.0000000000003462, PMID: 35778249

[3] Lacerda PN Lange EP Luna NM . Recurrence rate of basal cell carcinoma among different micrographic surgery techniques: systematic review with meta-analysis. J Eur Acad Dermatol Venereol. (2022) 36:1178–90. doi: 10.1111/jdv.18048, PMID: 35274381

[4] Jeremic G Brandt MG Jordan K . Using photodynamic therapy as a neoadjuvant treatment in the surgical excision of nonmelanotic skin cancers: prospective study. J Otolaryngol Head Neck Surg. (2011) 40 Suppl 1:S82–89., PMID: 21453666

[5] Nasr I Mcgrath EJ Harwood CA . British Association of Dermatologists guidelines for the management of adults with basal cell carcinoma 2021. Br J Dermatol. (2021) 185:899–920. doi: 10.1111/bjd.20524, PMID: 34050920

[6] Zhang W Zeng W Jiang A . Global, regional and national incidence, mortality and disability-adjusted life-years of skin cancers and trend analysis from 1990 to 2019: An analysis of the Global Burden of Disease Study 2019. Cancer Med. (2021) 10:4905–22. doi: 10.1002/cam4.4046, PMID: 34105887 PMC8290243

[7] Kushnir-Grinbaum D Krausz J Rahal N . Risk of melanoma in patients with basal cell carcinoma: A population-based cohort study. Acta Derm Venereol. (2023) 103:adv00841. doi: 10.2340/actadv.v103.4402, PMID: 36600530 PMC9885282

[8] Wojtowicz I Żychowska M . Dermoscopy of basal cell carcinoma part 2: dermoscopic findings by lesion subtype, location, age of onset, size and patient phototype. Cancers (Basel). (2025) 17:1–21. doi: 10.3390/cancers17020176, PMID: 39857958 PMC11764052

[9] Jeremian R Malinowski A Lytvyn Y . Skin photoageing following sun exposure is associated with decreased epigenetic and biologic age, and correlates with basal cell carcinoma phenotype. Br J Dermatol. (2024) 190:590–2. doi: 10.1093/bjd/ljad527, PMID: 38133632 PMC10941323

[10] Bertaina C Salerni G Celoria M . Dermoscopy of pigmented vulvar basal cell carcinoma. Dermatol Pract Concept. (2019) 9:239–40., PMID: 31384508 10.5826/dpc.0903a20 PMC6659589

[11] An S Kim K Moon S . Indoor tanning and the risk of overall and early-onset melanoma and non-melanoma skin cancer: systematic review and meta-analysis. Cancers (Basel). (2021) 13:1–11. doi: 10.3390/cancers13235940, PMID: 34885049 PMC8656707

[12] Vila-Payeras A Domínguez C Solà A . Incidental skin cancer detection in a hospital department: A prospective study. Actas Dermosifiliogr (Engl Ed). (2020) 111:496–502. doi: 10.1016/j.ad.2020.04.006, PMID: 32401722

[13] Bray F Ferlay J Soerjomataram I . Global cancer statistics 2018: GLOBOCAN estimates of incidence and mortality worldwide for 36 cancers in 185 countries. CA Cancer J Clin. (2018) 68:394–424. doi: 10.3322/caac.21492, PMID: 30207593

[14] Verkouteren JAC Ramdas KHR Wakkee M . Epidemiology of basal cell carcinoma: scholarly review. Br J Dermatol. (2017) 177:359–72. doi: 10.1111/bjd.15321, PMID: 28220485

[15] Bray F Laversanne M Sung H . Global cancer statistics 2022: GLOBOCAN estimates of incidence and mortality worldwide for 36 cancers in 185 countries. CA Cancer J Clin. (2024) 74:229–63. doi: 10.3322/caac.21834, PMID: 38572751

[16] Greig A Aloni K Orchard G . Treatment of multiple facial basal cell carcinomas in a child with xeroderma pigmentosum complementation group C with Mohs micrographic surgery. Br J Dermatol. (2021) 184:e4. doi: 10.1111/bjd.19323, PMID: 33325539

[17] Hernandez LE Mohsin N Levin N . Basal cell carcinoma: An updated review of pathogenesis and treatment options. Dermatol Ther. (2022) 35:e15501. doi: 10.1111/dth.15501, PMID: 35393669

[18] Loveridge-Easther C Weatherhead R . Unusual basal cell carcinoma in an adult woman with gorlin-goltz syndrome. JAMA Ophthalmol. (2022) 140:e220637. doi: 10.1001/jamaophthalmol.2022.0637, PMID: 35587820

[19] Matsuo M Niwa H Iwata H . A case of metastasis of giant basal cell carcinoma in oculocutaneous albinism. J Dermatol. (2025) 52:e43–4. doi: 10.1111/1346-8138.17425, PMID: 39133569

[20] Ramos AN Ramos JGR Fernandes JD . Prevalence of premalignant and Malignant skin lesions in oculocutaneous albinism patients. Rev Assoc Med Bras (1992). (2021) 67:77–82. doi: 10.1590/1806-9282.67.01.20200356, PMID: 34161467

[21] Wang X Xie K Guo X . Multiple facial basal cell carcinoma with xeroderma pigmentosum. J Craniofac Surg. (2023) 34:e761–2. doi: 10.1097/SCS.0000000000009642, PMID: 37603892

[22] Mushtaq S . The immunogenetics of non-melanoma skin cancer. Adv Exp Med Biol. (2022) 1367:397–409., PMID: 35286705 10.1007/978-3-030-92616-8_16

[23] Paudel S Raina K Tiku VR . Chemopreventive efficacy of silibinin against basal cell carcinoma growth and progression in UVB-irradiated Ptch+/- mice. Carcinogenesis. (2022) 43:557–70. doi: 10.1093/carcin/bgac023, PMID: 35184170 PMC9234765

[24] Thomson J Hogan S Leonardi-Bee J . Interventions for basal cell carcinoma of the skin. Cochrane Database Syst Rev. (2020) 11:Cd003412. 33202063 10.1002/14651858.CD003412.pub3 PMC8164471

[25] Sharma P Wadhwan V Bansal V . Basal cell carcinoma: Histopathological gamut. Indian J Dent Res. (2021) 32:407–10. doi: 10.4103/ijdr.IJDR_227_19, PMID: 35229784

[26] Jain R Dubey SK Singhvi G . The Hedgehog pathway and its inhibitors: Emerging therapeutic approaches for basal cell carcinoma. Drug Discov Today. (2022) 27:1176–83. doi: 10.1016/j.drudis.2021.12.005, PMID: 34896624

[27] Basset-Seguin N Herms F . Update in the management of basal cell carcinoma. Acta Derm Venereol. (2020) 100:adv00140. doi: 10.2340/00015555-3495, PMID: 32346750 PMC9189749

[28] Jain C Garg N Singh S . Basal cell carcinoma-clinico-pathological study in Eastern India in correlation with different risk factors. Indian J Pathol Microbiol. (2022) 65:869–72. doi: 10.4103/ijpm.ijpm_180_21, PMID: 36308196

[29] Council ML . Commentary on "*In vivo* imaging characterization of basal cell carcinoma cutaneous response to high-dose ionizing radiation therapy: A prospective study of reflectance confocal microscopy, dermoscopy, and ultrasound. J Am Acad Dermatol. (2021) 84:1792–3. doi: 10.1016/j.jaad.2020.08.066, PMID: 32827606

[30] Taniguchi N Takahara T Ito T . Clinicopathologic analysis of Malignant or premalignant cutaneous neoplasms in Japanese kidney transplant recipients. Int J Clin Exp Pathol. (2021) 14:1138–47. PMC8748015 35027994

[31] Muntyanu A Ghazawi FM Pastukhova E . National trends in incidence and geographic distribution of melanoma and keratinocyte carcinoma in the Russian Federation. Front Med (Lausanne). (2023) 10:1188038. doi: 10.3389/fmed.2023.1188038, PMID: 37547610 PMC10397511

[32] Munjal A Ferguson N . Skin cancer in skin of color. Dermatol Clin. (2023) 41:481–9. doi: 10.1016/j.det.2023.02.013, PMID: 37236716

[33] Salmen NL Curtis DP Baumann AN . Skin color reporting in basal cell carcinoma-related randomized controlled trials in top dermatology journals: a systematic review. Arch Dermatol Res. (2024) 316:451. doi: 10.1007/s00403-024-03187-7, PMID: 38967663 PMC11226525

[34] Álvarez-Salafranca M Ara M Zaballos P . Dermoscopy in basal cell carcinoma: an updated review. Actas Dermosifiliogr (Engl Ed). (2021) 112:330–8., PMID: 33259816 10.1016/j.ad.2020.11.011

[35] Ungureanu L Cosgarea I Şenilă S . Role of dermoscopy in the assessment of basal cell carcinoma. Front Med (Lausanne). (2021) 8:718855. doi: 10.3389/fmed.2021.718855, PMID: 34490305 PMC8417571

[36] Vaccari S Barisani A Schiavi C . Basal cell carcinoma of the eyelid margin: Dermoscopic clues in a case series. Dermatol Ther. (2021) 34:e15006. doi: 10.1111/dth.15006, PMID: 34043265

[37] Arias-Rodriguez C Muñoz-Monsalve AM Cuesta D . Dermoscopy of very small basal cell carcinoma (≤3mm). Bras Dermatol. (2023) 98:755–63. doi: 10.1016/j.abd.2022.12.004, PMID: 37422343 PMC10589476

[38] Lu J Zhao X Gan S . Global, regional and national burden of tracheal, bronchus, and lung cancer attributable to ambient particulate matter pollution from 1990 to 2021: an analysis of the global burden of disease study. BMC Public Health. (2025) 25:108. doi: 10.1186/s12889-024-21226-w, PMID: 39789484 PMC11720299

[39] Li T Zhang H Lian M . Global status and attributable risk factors of breast, cervical, ovarian, and uterine cancers from 1990 to 2021. J Hematol Oncol. (2025) 18:5. doi: 10.1186/s13045-025-01660-y, PMID: 39794860 PMC11721161

[40] Hu W Fang L Ni R . Changing trends in the disease burden of non-melanoma skin cancer globally from 1990 to 2019 and its predicted level in 25 years. BMC Cancer. (2022) 22:836. doi: 10.1186/s12885-022-09940-3, PMID: 35907848 PMC9339183

[41] Urban K Chu S Giesey RL . Burden of skin disease and associated socioeconomic status in Asia: A cross-sectional analysis from the Global Burden of Disease Study 1990–2017. JAAD Int. (2021) 2:40–50. doi: 10.1016/j.jdin.2020.10.006, PMID: 34409353 PMC8362322

[42] Lu Y Shang Z Zhang W . Global, regional, and national burden of spinal cord injury from 1990 to 2021 and projections for 2050: A systematic analysis for the Global Burden of Disease 2021 study. Ageing Res Rev. (2025) 103:102598. doi: 10.1016/j.arr.2024.102598, PMID: 39603465

