# Supplementary material for: Global basal cell carcinoma in 55+ population: 1990– 2021 burden、risk-factor trends and 2050 forecast
Source: Front Oncol. 2025 Dec 19;15:1702129. doi: 10.3389/fonc.2025.1702129 (PMC12757240; doi:10.3389/fonc.2025.1702129)
Supplement: Supplementary file 1 [file DataSheet1.pdf]

Supplementary Data1

Time and sex trends of 55+ year BCC from 1990 to 2021

measure	location	sex	age	cause	metric	year	val	upper	lower
DALYs (Dis	Global	Male	55+ years	Non-melanoma skin cancer (basal-cell	Rate	1990	0.0705449	0.133815105	0.030752985
DALYs (Dis	Global	Female	55+ years	Non-melanoma skin cancer (basal-cell	Rate	1990	0.0637453	0.122357611	0.027929331
DALYs (Dis	Global	Both	55+ years	Non-melanoma skin cancer (basal-cell	Rate	1990	0.0668998	0.12731367	0.029076749
DALYs (Dis	Global	Male	55+ years	Non-melanoma skin cancer (basal-cell	Rate	1996	0.0708656	0.134119583	0.031234133
DALYs (Dis	Global	Female	55+ years	Non-melanoma skin cancer (basal-cell	Rate	1996	0.0631459	0.120620621	0.027399223
DALYs (Dis	Global	Both	55+ years	Non-melanoma skin cancer (basal-cell	Rate	1996	0.0667468	0.126951749	0.029271977
DALYs (Dis	Global	Male	55+ years	Non-melanoma skin cancer (basal-cell	Rate	1995	0.0699821	0.132151279	0.030896487
DALYs (Dis	Global	Female	55+ years	Non-melanoma skin cancer (basal-cell	Rate	1995	0.062887	0.119406839	0.02734433
DALYs (Dis	Global	Both	55+ years	Non-melanoma skin cancer (basal-cell	Rate	1995	0.066195	0.126613065	0.028770557
DALYs (Dis	Global	Male	55+ years	Non-melanoma skin cancer (basal-cell	Rate	1993	0.0700726	0.132353048	0.030207212
DALYs (Dis	Global	Female	55+ years	Non-melanoma skin cancer (basal-cell	Rate	1993	0.0631941	0.118513174	0.027416095
DALYs (Dis	Global	Both	55+ years	Non-melanoma skin cancer (basal-cell	Rate	1993	0.0663968	0.125453287	0.028642346
DALYs (Dis	Global	Male	55+ years	Non-melanoma skin cancer (basal-cell	Rate	1994	0.069895	0.132172442	0.03123523
DALYs (Dis	Global	Female	55+ years	Non-melanoma skin cancer (basal-cell	Rate	1994	0.0631175	0.11971999	0.026838375
DALYs (Dis	Global	Both	55+ years	Non-melanoma skin cancer (basal-cell	Rate	1994	0.0662758	0.12605101	0.028914265
DALYs (Dis	Global	Male	55+ years	Non-melanoma skin cancer (basal-cell	Rate	1992	0.0699479	0.130287182	0.030639764
DALYs (Dis	Global	Female	55+ years	Non-melanoma skin cancer (basal-cell	Rate	1992	0.0634222	0.12021069	0.027366436
DALYs (Dis	Global	Both	55+ years	Non-melanoma skin cancer (basal-cell	Rate	1992	0.0664579	0.127059559	0.028821712
DALYs (Dis	Global	Male	55+ years	Non-melanoma skin cancer (basal-cell	Rate	1991	0.0702288	0.132944787	0.030750648
DALYs (Dis	Global	Female	55+ years	Non-melanoma skin cancer (basal-cell	Rate	1991	0.0635067	0.121529729	0.027419733
DALYs (Dis	Global	Both	55+ years	Non-melanoma skin cancer (basal-cell	Rate	1991	0.0666299	0.12743623	0.02884719
DALYs (Dis	Global	Male	55+ years	Non-melanoma skin cancer (basal-cell	Rate	2002	0.1000776	0.19194326	0.044745698
DALYs (Dis	Global	Female	55+ years	Non-melanoma skin cancer (basal-cell	Rate	2002	0.0743122	0.142943514	0.033007534
DALYs (Dis	Global	Both	55+ years	Non-melanoma skin cancer (basal-cell	Rate	2002	0.0863644	0.165691886	0.038504975
DALYs (Dis	Global	Male	55+ years	Non-melanoma skin cancer (basal-cell	Rate	2000	0.083233	0.158231211	0.037073919
DALYs (Dis	Global	Female	55+ years	Non-melanoma skin cancer (basal-cell	Rate	2000	0.0654008	0.12666245	0.028329232
DALYs (Dis	Global	Both	55+ years	Non-melanoma skin cancer (basal-cell	Rate	2000	0.0737318	0.141567381	0.032430765
DALYs (Dis	Global	Male	55+ years	Non-melanoma skin cancer (basal-cell	Rate	2001	0.0897697	0.169620027	0.039952716
DALYs (Dis	Global	Female	55+ years	Non-melanoma skin cancer (basal-cell	Rate	2001	0.0682445	0.13177901	0.029789609
DALYs (Dis	Global	Both	55+ years	Non-melanoma skin cancer (basal-cell	Rate	2001	0.0783054	0.149246527	0.034520786
DALYs (Dis	Global	Male	55+ years	Non-melanoma skin cancer (basal-cell	Rate	1999	0.0797428	0.152379904	0.035216655
DALYs (Dis	Global	Female	55+ years	Non-melanoma skin cancer (basal-cell	Rate	1999	0.0648112	0.122413267	0.028418598
DALYs (Dis	Global	Both	55+ years	Non-melanoma skin cancer (basal-cell	Rate	1999	0.0717842	0.13687973	0.031591439
DALYs (Dis	Global	Male	55+ years	Non-melanoma skin cancer (basal-cell	Rate	1998	0.0761051	0.145074323	0.033256715
DALYs (Dis	Global	Female	55+ years	Non-melanoma skin cancer (basal-cell	Rate	1998	0.0641424	0.12260445	0.027956443
DALYs (Dis	Global	Both	55+ years	Non-melanoma skin cancer (basal-cell	Rate	1998	0.0697268	0.134175944	0.030488164
DALYs (Dis	Global	Male	55+ years	Non-melanoma skin cancer (basal-cell	Rate	1997	0.0729992	0.13949488	0.031960831
DALYs (Dis	Global	Female	55+ years	Non-melanoma skin cancer (basal-cell	Rate	1997	0.0634159	0.120989194	0.027470252
DALYs (Dis	Global	Both	55+ years	Non-melanoma skin cancer (basal-cell	Rate	1997	0.0678874	0.130587951	0.029507771
DALYs (Dis	Global	Male	55+ years	Non-melanoma skin cancer (basal-cell	Rate	2003	0.1117311	0.214684158	0.049347488
DALYs (Dis	Global	Female	55+ years	Non-melanoma skin cancer (basal-cell	Rate	2003	0.0813022	0.155432011	0.036269012
DALYs (Dis	Global	Both	55+ years	Non-melanoma skin cancer (basal-cell	Rate	2003	0.0955488	0.184328956	0.04257284
DALYs (Dis	Global	Male	55+ years	Non-melanoma skin cancer (basal-cell	Rate	2007	0.1237432	0.239357027	0.056887394
DALYs (Dis	Global	Female	55+ years	Non-melanoma skin cancer (basal-cell	Rate	2007	0.0882652	0.169808074	0.040163663
DALYs (Dis	Global	Both	55+ years	Non-melanoma skin cancer (basal-cell	Rate	2007	0.1049247	0.200973537	0.047730173
DALYs (Dis	Global	Male	55+ years	Non-melanoma skin cancer (basal-cell	Rate	2006	0.1239476	0.237306245	0.056991299
DALYs (Dis	Global	Female	55+ years	Non-melanoma skin cancer (basal-cell	Rate	2006	0.0886596	0.170606952	0.039816021
DALYs (Dis	Global	Both	55+ years	Non-melanoma skin cancer (basal-cell	Rate	2006	0.1052188	0.202235309	0.047793488
DALYs (Dis	Global	Male	55+ years	Non-melanoma skin cancer (basal-cell	Rate	2005	0.1242836	0.24039241	0.056306423
DALYs (Dis	Global	Female	55+ years	Non-melanoma skin cancer (basal-cell	Rate	2005	0.0892246	0.171679157	0.04023995
DALYs (Dis	Global	Both	55+ years	Non-melanoma skin cancer (basal-cell	Rate	2005	0.1056651	0.203749264	0.047777613
DALYs (Dis	Global	Male	55+ years	Non-melanoma skin cancer (basal-cell	Rate	2004	0.1203311	0.231130418	0.054195197
DALYs (Dis	Global	Female	55+ years	Non-melanoma skin cancer (basal-cell	Rate	2004	0.0868022	0.166675756	0.039182538

DALYs (Dis	Global	Both	55+ years	Non-melanoma skin cancer (basal-cell	Rate	2004	0.1025174	0.197196783	0.046112206
DALYs (Dis	Global	Male	55+ years	Non-melanoma skin cancer (basal-cell	Rate	2010	0.1225123	0.235660329	0.05659118
DALYs (Dis	Global	Female	55+ years	Non-melanoma skin cancer (basal-cell	Rate	2010	0.0865469	0.166085539	0.03957842
DALYs (Dis	Global	Both	55+ years	Non-melanoma skin cancer (basal-cell	Rate	2010	0.1034644	0.198017579	0.04758578
DALYs (Dis	Global	Male	55+ years	Non-melanoma skin cancer (basal-cell	Rate	2009	0.1228502	0.236039746	0.056011426
DALYs (Dis	Global	Female	55+ years	Non-melanoma skin cancer (basal-cell	Rate	2009	0.0872851	0.168252242	0.039559379
DALYs (Dis	Global	Both	55+ years	Non-melanoma skin cancer (basal-cell	Rate	2009	0.1040059	0.199308633	0.047294253
DALYs (Dis	Global	Male	55+ years	Non-melanoma skin cancer (basal-cell	Rate	2013	0.1240727	0.238711776	0.057028858
DALYs (Dis	Global	Female	55+ years	Non-melanoma skin cancer (basal-cell	Rate	2013	0.0861372	0.164701098	0.039206178
DALYs (Dis	Global	Both	55+ years	Non-melanoma skin cancer (basal-cell	Rate	2013	0.1040013	0.200104061	0.047673056
DALYs (Dis	Global	Male	55+ years	Non-melanoma skin cancer (basal-cell	Rate	2008	0.1234517	0.238178814	0.05635898
DALYs (Dis	Global	Female	55+ years	Non-melanoma skin cancer (basal-cell	Rate	2008	0.0878841	0.169099354	0.040075815
DALYs (Dis	Global	Both	55+ years	Non-melanoma skin cancer (basal-cell	Rate	2008	0.1045961	0.200573942	0.047739807
DALYs (Dis	Global	Male	55+ years	Non-melanoma skin cancer (basal-cell	Rate	2011	0.1225223	0.235909686	0.057116058
DALYs (Dis	Global	Female	55+ years	Non-melanoma skin cancer (basal-cell	Rate	2011	0.0860008	0.166671847	0.039286058
DALYs (Dis	Global	Both	55+ years	Non-melanoma skin cancer (basal-cell	Rate	2011	0.1031869	0.198304627	0.047780727
DALYs (Dis	Global	Male	55+ years	Non-melanoma skin cancer (basal-cell	Rate	2012	0.1231225	0.236930786	0.05658312
DALYs (Dis	Global	Female	55+ years	Non-melanoma skin cancer (basal-cell	Rate	2012	0.0860658	0.164722024	0.039766145
DALYs (Dis	Global	Both	55+ years	Non-melanoma skin cancer (basal-cell	Rate	2012	0.1035107	0.197683549	0.047834424
DALYs (Dis	Global	Male	55+ years	Non-melanoma skin cancer (basal-cell	Rate	2017	0.1243168	0.237239317	0.057498111
DALYs (Dis	Global	Female	55+ years	Non-melanoma skin cancer (basal-cell	Rate	2017	0.084772	0.160456863	0.038561256
DALYs (Dis	Global	Both	55+ years	Non-melanoma skin cancer (basal-cell	Rate	2017	0.1034045	0.196634719	0.047287711
DALYs (Dis	Global	Male	55+ years	Non-melanoma skin cancer (basal-cell	Rate	2016	0.1251519	0.242010463	0.058862796
DALYs (Dis	Global	Female	55+ years	Non-melanoma skin cancer (basal-cell	Rate	2016	0.0856032	0.164669047	0.039539632
DALYs (Dis	Global	Both	55+ years	Non-melanoma skin cancer (basal-cell	Rate	2016	0.1042339	0.201090137	0.048728663
DALYs (Dis	Global	Male	55+ years	Non-melanoma skin cancer (basal-cell	Rate	2014	0.1248226	0.24191729	0.057922559
DALYs (Dis	Global	Female	55+ years	Non-melanoma skin cancer (basal-cell	Rate	2014	0.0861823	0.164371452	0.038858717
DALYs (Dis	Global	Both	55+ years	Non-melanoma skin cancer (basal-cell	Rate	2014	0.104381	0.200954328	0.047861818
DALYs (Dis	Global	Male	55+ years	Non-melanoma skin cancer (basal-cell	Rate	2015	0.125439	0.241685062	0.05821636
DALYs (Dis	Global	Female	55+ years	Non-melanoma skin cancer (basal-cell	Rate	2015	0.0862225	0.163314523	0.039245455
DALYs (Dis	Global	Both	55+ years	Non-melanoma skin cancer (basal-cell	Rate	2015	0.1046946	0.200505998	0.047897958
DALYs (Dis	Global	Male	55+ years	Non-melanoma skin cancer (basal-cell	Rate	2019	0.1232557	0.235348679	0.056535682
DALYs (Dis	Global	Female	55+ years	Non-melanoma skin cancer (basal-cell	Rate	2019	0.0835389	0.160992883	0.038007316
DALYs (Dis	Global	Both	55+ years	Non-melanoma skin cancer (basal-cell	Rate	2019	0.1022608	0.196368636	0.04678969
DALYs (Dis	Global	Male	55+ years	Non-melanoma skin cancer (basal-cell	Rate	2018	0.123442	0.236674187	0.057177394
DALYs (Dis	Global	Female	55+ years	Non-melanoma skin cancer (basal-cell	Rate	2018	0.0838308	0.160680462	0.038234786
DALYs (Dis	Global	Both	55+ years	Non-melanoma skin cancer (basal-cell	Rate	2018	0.1024989	0.196730339	0.047128653
DALYs (Dis	Global	Male	55+ years	Non-melanoma skin cancer (basal-cell	Rate	2021	0.1314179	0.249340141	0.060311662
DALYs (Dis	Global	Female	55+ years	Non-melanoma skin cancer (basal-cell	Rate	2021	0.0894595	0.170783787	0.041208997
DALYs (Dis	Global	Both	55+ years	Non-melanoma skin cancer (basal-cell	Rate	2021	0.1092111	0.207704858	0.050148379
DALYs (Dis	Global	Male	55+ years	Non-melanoma skin cancer (basal-cell	Rate	2020	0.1238319	0.236818225	0.057010225
DALYs (Dis	Global	Female	55+ years	Non-melanoma skin cancer (basal-cell	Rate	2020	0.0837118	0.160826945	0.037954893
DALYs (Dis	Global	Both	55+ years	Non-melanoma skin cancer (basal-cell	Rate	2020	0.1026185	0.19627946	0.046934775
Prevalence	Global	Male	55+ years	Non-melanoma skin cancer (basal-cell	Rate	1990	17.207952	21.34464813	13.74820651
Prevalence	Global	Female	55+ years	Non-melanoma skin cancer (basal-cell	Rate	1990	15.44801	19.20228662	12.16235978
Prevalence	Global	Both	55+ years	Non-melanoma skin cancer (basal-cell	Rate	1990	16.264471	20.04718911	12.89400305
Prevalence	Global	Male	55+ years	Non-melanoma skin cancer (basal-cell	Rate	1991	17.102322	21.12076048	13.68093175
Prevalence	Global	Female	55+ years	Non-melanoma skin cancer (basal-cell	Rate	1991	15.399248	19.0640388	12.13074633
Prevalence	Global	Both	55+ years	Non-melanoma skin cancer (basal-cell	Rate	1991	16.190526	19.91131281	12.84852907
Prevalence	Global	Male	55+ years	Non-melanoma skin cancer (basal-cell	Rate	1992	17.043317	20.98115435	13.64629793
Prevalence	Global	Female	55+ years	Non-melanoma skin cancer (basal-cell	Rate	1992	15.3557	18.93733858	12.10066512
Prevalence	Global	Both	55+ years	Non-melanoma skin cancer (basal-cell	Rate	1992	16.140751	19.85211186	12.81276369
Prevalence	Global	Male	55+ years	Non-melanoma skin cancer (basal-cell	Rate	1993	17.027368	20.97173612	13.64372137
Prevalence	Global	Female	55+ years	Non-melanoma skin cancer (basal-cell	Rate	1993	15.32261	18.86514292	12.07783889
Prevalence	Global	Both	55+ years	Non-melanoma skin cancer (basal-cell	Rate	1993	16.116374	19.8112627	12.79487959
Prevalence	Global	Male	55+ years	Non-melanoma skin cancer (basal-cell	Rate	1994	17.024938	20.93753608	13.63681637

Prevalence	Global	Female	55+ years	Non-melanoma skin cancer (basal-cell	Rate	1994	15.290462	18.79729586	12.05355915
Prevalence	Global	Both	55+ years	Non-melanoma skin cancer (basal-cell	Rate	1994	16.09873	19.78155441	12.781598
Prevalence	Global	Male	55+ years	Non-melanoma skin cancer (basal-cell	Rate	1995	17.055418	20.93564636	13.64356551
Prevalence	Global	Female	55+ years	Non-melanoma skin cancer (basal-cell	Rate	1995	15.264724	18.7444696	12.04154651
Prevalence	Global	Both	55+ years	Non-melanoma skin cancer (basal-cell	Rate	1995	16.099603	19.77518898	12.78009485
Prevalence	Global	Male	55+ years	Non-melanoma skin cancer (basal-cell	Rate	1996	17.273083	21.14973177	13.81972712
Prevalence	Global	Female	55+ years	Non-melanoma skin cancer (basal-cell	Rate	1996	15.287731	18.73217282	12.07356398
Prevalence	Global	Both	55+ years	Non-melanoma skin cancer (basal-cell	Rate	1996	16.213796	19.86035968	12.88228589
Prevalence	Global	Male	55+ years	Non-melanoma skin cancer (basal-cell	Rate	1997	17.809554	21.73096381	14.26067235
Prevalence	Global	Female	55+ years	Non-melanoma skin cancer (basal-cell	Rate	1997	15.396239	18.8077244	12.16930756
Prevalence	Global	Both	55+ years	Non-melanoma skin cancer (basal-cell	Rate	1997	16.522274	20.16307938	13.14897028
Prevalence	Global	Male	55+ years	Non-melanoma skin cancer (basal-cell	Rate	1998	18.586226	22.58728651	14.90433515
Prevalence	Global	Female	55+ years	Non-melanoma skin cancer (basal-cell	Rate	1998	15.577663	18.99087553	12.3341569
Prevalence	Global	Both	55+ years	Non-melanoma skin cancer (basal-cell	Rate	1998	16.982117	20.61072022	13.54358183
Prevalence	Global	Male	55+ years	Non-melanoma skin cancer (basal-cell	Rate	1999	19.457318	23.54938938	15.62473272
Prevalence	Global	Female	55+ years	Non-melanoma skin cancer (basal-cell	Rate	1999	15.758907	19.17334118	12.55802538
Prevalence	Global	Both	55+ years	Non-melanoma skin cancer (basal-cell	Rate	1999	17.486051	21.1093582	13.97464683
Prevalence	Global	Male	55+ years	Non-melanoma skin cancer (basal-cell	Rate	2000	20.36086	24.52520384	16.39579119
Prevalence	Global	Female	55+ years	Non-melanoma skin cancer (basal-cell	Rate	2000	15.926662	19.29604915	12.72320095
Prevalence	Global	Both	55+ years	Non-melanoma skin cancer (basal-cell	Rate	2000	17.998267	21.66156782	14.40882627
Prevalence	Global	Male	55+ years	Non-melanoma skin cancer (basal-cell	Rate	2001	21.969825	26.18721469	17.91604346
Prevalence	Global	Female	55+ years	Non-melanoma skin cancer (basal-cell	Rate	2001	16.640957	19.98072866	13.46521007
Prevalence	Global	Both	55+ years	Non-melanoma skin cancer (basal-cell	Rate	2001	19.131676	22.84117881	15.57503421
Prevalence	Global	Male	55+ years	Non-melanoma skin cancer (basal-cell	Rate	2002	24.564041	28.96159349	20.40296021
Prevalence	Global	Female	55+ years	Non-melanoma skin cancer (basal-cell	Rate	2002	18.144595	21.52474871	15.02344553
Prevalence	Global	Both	55+ years	Non-melanoma skin cancer (basal-cell	Rate	2002	21.1474	24.96814215	17.53238984
Prevalence	Global	Male	55+ years	Non-melanoma skin cancer (basal-cell	Rate	2003	27.420498	32.04835662	23.15591275
Prevalence	Global	Female	55+ years	Non-melanoma skin cancer (basal-cell	Rate	2003	19.902712	23.45836026	16.69396361
Prevalence	Global	Both	55+ years	Non-melanoma skin cancer (basal-cell	Rate	2003	23.422487	27.42427831	19.71007847
Prevalence	Global	Male	55+ years	Non-melanoma skin cancer (basal-cell	Rate	2004	29.595667	34.44195465	25.34015889
Prevalence	Global	Female	55+ years	Non-melanoma skin cancer (basal-cell	Rate	2004	21.26813	24.82116341	17.9687204
Prevalence	Global	Both	55+ years	Non-melanoma skin cancer (basal-cell	Rate	2004	25.17129	29.28006221	21.38001414
Prevalence	Global	Male	55+ years	Non-melanoma skin cancer (basal-cell	Rate	2005	30.593697	35.43215416	26.24233541
Prevalence	Global	Female	55+ years	Non-melanoma skin cancer (basal-cell	Rate	2005	21.848612	25.34337539	18.5571675
Prevalence	Global	Both	55+ years	Non-melanoma skin cancer (basal-cell	Rate	2005	25.949526	30.11199803	22.23512607
Prevalence	Global	Male	55+ years	Non-melanoma skin cancer (basal-cell	Rate	2006	30.497707	35.29985812	26.182001
Prevalence	Global	Female	55+ years	Non-melanoma skin cancer (basal-cell	Rate	2006	21.727368	25.17355228	18.49445472
Prevalence	Global	Both	55+ years	Non-melanoma skin cancer (basal-cell	Rate	2006	25.842922	29.97192881	22.18050698
Prevalence	Global	Male	55+ years	Non-melanoma skin cancer (basal-cell	Rate	2007	30.432055	35.21487684	26.21735199
Prevalence	Global	Female	55+ years	Non-melanoma skin cancer (basal-cell	Rate	2007	21.618689	25.03390569	18.40183563
Prevalence	Global	Both	55+ years	Non-melanoma skin cancer (basal-cell	Rate	2007	25.757205	29.85887209	22.14894458
Prevalence	Global	Male	55+ years	Non-melanoma skin cancer (basal-cell	Rate	2008	30.381712	35.18280104	26.26325543
Prevalence	Global	Female	55+ years	Non-melanoma skin cancer (basal-cell	Rate	2008	21.509171	24.92420217	18.31076976
Prevalence	Global	Both	55+ years	Non-melanoma skin cancer (basal-cell	Rate	2008	25.678071	29.73338156	22.04988083
Prevalence	Global	Male	55+ years	Non-melanoma skin cancer (basal-cell	Rate	2009	30.26995	35.12734295	26.17345782
Prevalence	Global	Female	55+ years	Non-melanoma skin cancer (basal-cell	Rate	2009	21.361807	24.75908318	18.19612065
Prevalence	Global	Both	55+ years	Non-melanoma skin cancer (basal-cell	Rate	2009	25.549941	29.55658147	21.90389347
Prevalence	Global	Male	55+ years	Non-melanoma skin cancer (basal-cell	Rate	2010	30.201217	35.12186434	26.0984817
Prevalence	Global	Female	55+ years	Non-melanoma skin cancer (basal-cell	Rate	2010	21.213623	24.56543712	18.07882462
Prevalence	Global	Both	55+ years	Non-melanoma skin cancer (basal-cell	Rate	2010	25.441227	29.4547619	21.7755064
Prevalence	Global	Male	55+ years	Non-melanoma skin cancer (basal-cell	Rate	2011	30.177445	35.09716207	26.08966219
Prevalence	Global	Female	55+ years	Non-melanoma skin cancer (basal-cell	Rate	2011	21.096402	24.44855471	17.96947558
Prevalence	Global	Both	55+ years	Non-melanoma skin cancer (basal-cell	Rate	2011	25.369712	29.4022787	21.7311538
Prevalence	Global	Male	55+ years	Non-melanoma skin cancer (basal-cell	Rate	2012	30.327147	35.27275278	26.22139675
Prevalence	Global	Female	55+ years	Non-melanoma skin cancer (basal-cell	Rate	2012	21.081132	24.46110805	17.93617092
Prevalence	Global	Both	55+ years	Non-melanoma skin cancer (basal-cell	Rate	2012	25.433812	29.51062527	21.7963267

Prevalence	Global	Male	55+ years	Non-melanoma skin cancer (basal-cell	Rate	2013	30.564061	35.53882832	26.4148668
Prevalence	Global	Female	55+ years	Non-melanoma skin cancer (basal-cell	Rate	2013	21.117851	24.5351245	17.95797967
Prevalence	Global	Both	55+ years	Non-melanoma skin cancer (basal-cell	Rate	2013	25.566145	29.69556693	21.9168792
Prevalence	Global	Male	55+ years	Non-melanoma skin cancer (basal-cell	Rate	2014	30.753034	35.7550189	26.53856204
Prevalence	Global	Female	55+ years	Non-melanoma skin cancer (basal-cell	Rate	2014	21.132645	24.61341466	17.97184771
Prevalence	Global	Both	55+ years	Non-melanoma skin cancer (basal-cell	Rate	2014	25.663641	29.84289084	21.99694633
Prevalence	Global	Male	55+ years	Non-melanoma skin cancer (basal-cell	Rate	2015	30.914965	35.9628162	26.67306049
Prevalence	Global	Female	55+ years	Non-melanoma skin cancer (basal-cell	Rate	2015	21.122099	24.68137407	17.96519992
Prevalence	Global	Both	55+ years	Non-melanoma skin cancer (basal-cell	Rate	2015	25.734822	29.96173421	22.04067885
Prevalence	Global	Male	55+ years	Non-melanoma skin cancer (basal-cell	Rate	2016	30.854733	35.95456811	26.53352406
Prevalence	Global	Female	55+ years	Non-melanoma skin cancer (basal-cell	Rate	2016	20.987755	24.53584782	17.81820413
Prevalence	Global	Both	55+ years	Non-melanoma skin cancer (basal-cell	Rate	2016	25.635926	29.87611339	21.91551925
Prevalence	Global	Male	55+ years	Non-melanoma skin cancer (basal-cell	Rate	2017	30.687679	35.85455498	26.31955377
Prevalence	Global	Female	55+ years	Non-melanoma skin cancer (basal-cell	Rate	2017	20.787529	24.32121164	17.61330495
Prevalence	Global	Both	55+ years	Non-melanoma skin cancer (basal-cell	Rate	2017	25.452217	29.69833962	21.70331132
Prevalence	Global	Male	55+ years	Non-melanoma skin cancer (basal-cell	Rate	2018	30.514037	35.73895713	26.10625592
Prevalence	Global	Female	55+ years	Non-melanoma skin cancer (basal-cell	Rate	2018	20.592836	24.10964953	17.40973495
Prevalence	Global	Both	55+ years	Non-melanoma skin cancer (basal-cell	Rate	2018	25.268528	29.5161023	21.46324319
Prevalence	Global	Male	55+ years	Non-melanoma skin cancer (basal-cell	Rate	2019	30.44402	35.69615276	26.00391782
Prevalence	Global	Female	55+ years	Non-melanoma skin cancer (basal-cell	Rate	2019	20.491611	24.02045588	17.29630649
Prevalence	Global	Both	55+ years	Non-melanoma skin cancer (basal-cell	Rate	2019	25.183016	29.43605162	21.31521799
Prevalence	Global	Male	55+ years	Non-melanoma skin cancer (basal-cell	Rate	2020	30.566809	35.90151162	26.05519839
Prevalence	Global	Female	55+ years	Non-melanoma skin cancer (basal-cell	Rate	2020	20.518006	24.10843838	17.23692916
Prevalence	Global	Both	55+ years	Non-melanoma skin cancer (basal-cell	Rate	2020	25.253534	29.61384953	21.38066262
Prevalence	Global	Male	55+ years	Non-melanoma skin cancer (basal-cell	Rate	2021	32.422281	38.17965909	27.58410996
Prevalence	Global	Female	55+ years	Non-melanoma skin cancer (basal-cell	Rate	2021	21.793562	25.75373265	18.2261484
Prevalence	Global	Both	55+ years	Non-melanoma skin cancer (basal-cell	Rate	2021	26.796959	31.53681717	22.61472233
Incidence	Global	Male	55+ years	Non-melanoma skin cancer (basal-cell	Rate	1990	154.88556	185.969118	125.1925418
Incidence	Global	Female	55+ years	Non-melanoma skin cancer (basal-cell	Rate	1990	127.51882	153.8356707	101.9145067
Incidence	Global	Both	55+ years	Non-melanoma skin cancer (basal-cell	Rate	1990	140.21462	168.0834369	112.8742324
Incidence	Global	Male	55+ years	Non-melanoma skin cancer (basal-cell	Rate	1991	154.23418	184.6778137	124.8771867
Incidence	Global	Female	55+ years	Non-melanoma skin cancer (basal-cell	Rate	1991	127.4227	153.3531631	102.1212054
Incidence	Global	Both	55+ years	Non-melanoma skin cancer (basal-cell	Rate	1991	139.87978	167.5153119	112.8721152
Incidence	Global	Male	55+ years	Non-melanoma skin cancer (basal-cell	Rate	1992	153.90766	183.8294653	124.818998
Incidence	Global	Female	55+ years	Non-melanoma skin cancer (basal-cell	Rate	1992	127.302	152.8625352	102.3122523
Incidence	Global	Both	55+ years	Non-melanoma skin cancer (basal-cell	Rate	1992	139.6785	167.2385717	112.9631067
Incidence	Global	Male	55+ years	Non-melanoma skin cancer (basal-cell	Rate	1993	153.90092	183.4085374	125.0106102
Incidence	Global	Female	55+ years	Non-melanoma skin cancer (basal-cell	Rate	1993	127.21505	152.5466793	102.5280227
Incidence	Global	Both	55+ years	Non-melanoma skin cancer (basal-cell	Rate	1993	139.64044	167.2055132	113.167722
Incidence	Global	Male	55+ years	Non-melanoma skin cancer (basal-cell	Rate	1994	153.9386	183.4354662	125.23161
Incidence	Global	Female	55+ years	Non-melanoma skin cancer (basal-cell	Rate	1994	127.06798	152.2220578	102.6898286
Incidence	Global	Both	55+ years	Non-melanoma skin cancer (basal-cell	Rate	1994	139.58973	167.0245232	113.3492754
Incidence	Global	Male	55+ years	Non-melanoma skin cancer (basal-cell	Rate	1995	154.25001	183.8564267	125.6564329
Incidence	Global	Female	55+ years	Non-melanoma skin cancer (basal-cell	Rate	1995	126.94134	151.9888626	102.8464725
Incidence	Global	Both	55+ years	Non-melanoma skin cancer (basal-cell	Rate	1995	139.67352	166.9938017	113.6176355
Incidence	Global	Male	55+ years	Non-melanoma skin cancer (basal-cell	Rate	1996	156.39043	185.7899568	127.7795049
Incidence	Global	Female	55+ years	Non-melanoma skin cancer (basal-cell	Rate	1996	127.15651	151.5767771	103.4835108
Incidence	Global	Both	55+ years	Non-melanoma skin cancer (basal-cell	Rate	1996	140.79264	167.3925557	115.1348049
Incidence	Global	Male	55+ years	Non-melanoma skin cancer (basal-cell	Rate	1997	161.70026	191.4631991	133.0942326
Incidence	Global	Female	55+ years	Non-melanoma skin cancer (basal-cell	Rate	1997	128.0563	151.5777284	104.8052668
Incidence	Global	Both	55+ years	Non-melanoma skin cancer (basal-cell	Rate	1997	143.75433	170.1948048	118.1444285
Incidence	Global	Male	55+ years	Non-melanoma skin cancer (basal-cell	Rate	1998	169.42908	199.6515261	140.0283208
Incidence	Global	Female	55+ years	Non-melanoma skin cancer (basal-cell	Rate	1998	129.59324	152.8858677	106.6073941
Incidence	Global	Both	55+ years	Non-melanoma skin cancer (basal-cell	Rate	1998	148.18936	174.7149813	122.1951458
Incidence	Global	Male	55+ years	Non-melanoma skin cancer (basal-cell	Rate	1999	178.15216	208.7438737	147.1953249
Incidence	Global	Female	55+ years	Non-melanoma skin cancer (basal-cell	Rate	1999	131.15442	154.3815163	108.2089901

Incidence	Global	Both	55+ years	Non-melanoma skin cancer (basal-cell	Rate	1999	153.10219	179.5074104	126.5139842
Incidence	Global	Male	55+ years	Non-melanoma skin cancer (basal-cell	Rate	2000	187.29465	218.0697361	155.1882597
Incidence	Global	Female	55+ years	Non-melanoma skin cancer (basal-cell	Rate	2000	132.69607	155.4018396	109.9977938
Incidence	Global	Both	55+ years	Non-melanoma skin cancer (basal-cell	Rate	2000	158.20389	184.5522045	130.7837302
Incidence	Global	Male	55+ years	Non-melanoma skin cancer (basal-cell	Rate	2001	203.64812	234.6601077	171.7238616
Incidence	Global	Female	55+ years	Non-melanoma skin cancer (basal-cell	Rate	2001	140.11214	162.1956806	117.8106129
Incidence	Global	Both	55+ years	Non-melanoma skin cancer (basal-cell	Rate	2001	169.80893	196.0958195	142.9558901
Incidence	Global	Male	55+ years	Non-melanoma skin cancer (basal-cell	Rate	2002	230.03716	262.1084673	197.7891446
Incidence	Global	Female	55+ years	Non-melanoma skin cancer (basal-cell	Rate	2002	155.88924	178.1068639	133.9408649
Incidence	Global	Both	55+ years	Non-melanoma skin cancer (basal-cell	Rate	2002	190.57319	217.6483221	163.9794833
Incidence	Global	Male	55+ years	Non-melanoma skin cancer (basal-cell	Rate	2003	259.13563	291.7865033	225.6025817
Incidence	Global	Female	55+ years	Non-melanoma skin cancer (basal-cell	Rate	2003	174.34913	196.6822849	152.4947972
Incidence	Global	Both	55+ years	Non-melanoma skin cancer (basal-cell	Rate	2003	214.04557	241.4891288	187.1469984
Incidence	Global	Male	55+ years	Non-melanoma skin cancer (basal-cell	Rate	2004	281.43891	314.0782707	248.2947224
Incidence	Global	Female	55+ years	Non-melanoma skin cancer (basal-cell	Rate	2004	188.8217	210.5528812	166.3383655
Incidence	Global	Both	55+ years	Non-melanoma skin cancer (basal-cell	Rate	2004	232.23187	258.865786	204.6804858
Incidence	Global	Male	55+ years	Non-melanoma skin cancer (basal-cell	Rate	2005	291.87541	323.0844359	258.5348714
Incidence	Global	Female	55+ years	Non-melanoma skin cancer (basal-cell	Rate	2005	195.07307	216.1191154	172.3031979
Incidence	Global	Both	55+ years	Non-melanoma skin cancer (basal-cell	Rate	2005	240.4675	266.3086559	212.8489478
Incidence	Global	Male	55+ years	Non-melanoma skin cancer (basal-cell	Rate	2006	291.3869	322.9555386	258.4631477
Incidence	Global	Female	55+ years	Non-melanoma skin cancer (basal-cell	Rate	2006	194.15806	215.2027203	171.1794414
Incidence	Global	Both	55+ years	Non-melanoma skin cancer (basal-cell	Rate	2006	239.78349	265.5123254	212.3302995
Incidence	Global	Male	55+ years	Non-melanoma skin cancer (basal-cell	Rate	2007	291.27471	322.5488681	258.8145362
Incidence	Global	Female	55+ years	Non-melanoma skin cancer (basal-cell	Rate	2007	193.40655	214.7936084	170.3495169
Incidence	Global	Both	55+ years	Non-melanoma skin cancer (basal-cell	Rate	2007	239.36277	264.919673	212.2237802
Incidence	Global	Male	55+ years	Non-melanoma skin cancer (basal-cell	Rate	2008	291.28148	322.3088821	259.4774356
Incidence	Global	Female	55+ years	Non-melanoma skin cancer (basal-cell	Rate	2008	192.65665	214.2172406	170.5822067
Incidence	Global	Both	55+ years	Non-melanoma skin cancer (basal-cell	Rate	2008	238.99705	264.7355044	212.7488634
Incidence	Global	Male	55+ years	Non-melanoma skin cancer (basal-cell	Rate	2009	290.54409	321.6513045	259.7180043
Incidence	Global	Female	55+ years	Non-melanoma skin cancer (basal-cell	Rate	2009	191.52147	213.0929561	169.9243156
Incidence	Global	Both	55+ years	Non-melanoma skin cancer (basal-cell	Rate	2009	238.07662	263.9518388	212.1958995
Incidence	Global	Male	55+ years	Non-melanoma skin cancer (basal-cell	Rate	2010	290.07843	321.580164	259.4410805
Incidence	Global	Female	55+ years	Non-melanoma skin cancer (basal-cell	Rate	2010	190.35433	211.8543593	168.9426396
Incidence	Global	Both	55+ years	Non-melanoma skin cancer (basal-cell	Rate	2010	237.26276	263.2859093	211.5327775
Incidence	Global	Male	55+ years	Non-melanoma skin cancer (basal-cell	Rate	2011	289.83609	321.3568922	258.9605131
Incidence	Global	Female	55+ years	Non-melanoma skin cancer (basal-cell	Rate	2011	189.38497	210.5684879	168.1271728
Incidence	Global	Both	55+ years	Non-melanoma skin cancer (basal-cell	Rate	2011	236.65474	262.3961533	210.9208077
Incidence	Global	Male	55+ years	Non-melanoma skin cancer (basal-cell	Rate	2012	291.20075	322.7947921	259.8511465
Incidence	Global	Female	55+ years	Non-melanoma skin cancer (basal-cell	Rate	2012	189.30287	210.3814411	168.0945721
Incidence	Global	Both	55+ years	Non-melanoma skin cancer (basal-cell	Rate	2012	237.27261	263.1815941	211.5961338
Incidence	Global	Male	55+ years	Non-melanoma skin cancer (basal-cell	Rate	2013	293.38869	325.5708267	261.6056025
Incidence	Global	Female	55+ years	Non-melanoma skin cancer (basal-cell	Rate	2013	189.68138	211.0748916	168.4735881
Incidence	Global	Both	55+ years	Non-melanoma skin cancer (basal-cell	Rate	2013	238.51796	264.6870007	212.6899241
Incidence	Global	Male	55+ years	Non-melanoma skin cancer (basal-cell	Rate	2014	295.10899	327.6784411	263.1819383
Incidence	Global	Female	55+ years	Non-melanoma skin cancer (basal-cell	Rate	2014	189.84973	211.31573	168.415539
Incidence	Global	Both	55+ years	Non-melanoma skin cancer (basal-cell	Rate	2014	239.42458	265.9655818	213.2672838
Incidence	Global	Male	55+ years	Non-melanoma skin cancer (basal-cell	Rate	2015	296.6905	329.5886748	264.7420303
Incidence	Global	Female	55+ years	Non-melanoma skin cancer (basal-cell	Rate	2015	189.83445	211.3013218	167.9827887
Incidence	Global	Both	55+ years	Non-melanoma skin cancer (basal-cell	Rate	2015	240.16674	266.8034743	213.6222967
Incidence	Global	Male	55+ years	Non-melanoma skin cancer (basal-cell	Rate	2016	296.29816	329.2640275	263.4888448
Incidence	Global	Female	55+ years	Non-melanoma skin cancer (basal-cell	Rate	2016	188.76871	210.498966	166.8150965
Incidence	Global	Both	55+ years	Non-melanoma skin cancer (basal-cell	Rate	2016	239.42407	266.0400882	212.5036445
Incidence	Global	Male	55+ years	Non-melanoma skin cancer (basal-cell	Rate	2017	295.04146	328.645756	260.992774
Incidence	Global	Female	55+ years	Non-melanoma skin cancer (basal-cell	Rate	2017	187.16563	209.2495346	165.075049
Incidence	Global	Both	55+ years	Non-melanoma skin cancer (basal-cell	Rate	2017	237.99386	264.9981925	210.5772406
Incidence	Global	Male	55+ years	Non-melanoma skin cancer (basal-cell	Rate	2018	293.69959	327.5517743	258.3799775

Incidence	Global	Female	55+ years	Non-melanoma skin cancer (basal-cell	Rate	2018	185.57424	207.8068174	163.2983921
Incidence	Global	Both	55+ years	Non-melanoma skin cancer (basal-cell	Rate	2018	236.53187	263.9116493	208.5991208
Incidence	Global	Male	55+ years	Non-melanoma skin cancer (basal-cell	Rate	2019	293.13465	327.4909245	256.8410523
Incidence	Global	Female	55+ years	Non-melanoma skin cancer (basal-cell	Rate	2019	184.6873	207.1751507	162.3893921
Incidence	Global	Both	55+ years	Non-melanoma skin cancer (basal-cell	Rate	2019	235.80763	263.48792	207.3366221
Incidence	Global	Male	55+ years	Non-melanoma skin cancer (basal-cell	Rate	2020	294.25005	328.9193447	257.9095408
Incidence	Global	Female	55+ years	Non-melanoma skin cancer (basal-cell	Rate	2020	184.353	206.9957094	161.8692438
Incidence	Global	Both	55+ years	Non-melanoma skin cancer (basal-cell	Rate	2020	236.14232	264.8340223	207.4458816
Incidence	Global	Male	55+ years	Non-melanoma skin cancer (basal-cell	Rate	2021	311.05515	349.1176312	272.0165765
Incidence	Global	Female	55+ years	Non-melanoma skin cancer (basal-cell	Rate	2021	195.66491	221.2731501	170.6524801
Incidence	Global	Both	55+ years	Non-melanoma skin cancer (basal-cell	Rate	2021	249.98408	281.3911243	218.6565021
DALYs (Dis	Global	Male	Age-standa	Non-melanoma skin cancer (basal-cell	Rate	1990	0.0119242	0.022618769	0.005198177
DALYs (Dis	Global	Female	Age-standa	Non-melanoma skin cancer (basal-cell	Rate	1990	0.0107749	0.020682108	0.004720895
DALYs (Dis	Global	Both	Age-standa	Non-melanoma skin cancer (basal-cell	Rate	1990	0.0113081	0.021519831	0.004914843
DALYs (Dis	Global	Male	Age-standa	Non-melanoma skin cancer (basal-cell	Rate	1996	0.0119784	0.022670235	0.005279506
DALYs (Dis	Global	Female	Age-standa	Non-melanoma skin cancer (basal-cell	Rate	1996	0.0106736	0.020388505	0.004631291
DALYs (Dis	Global	Both	Age-standa	Non-melanoma skin cancer (basal-cell	Rate	1996	0.0112822	0.021458656	0.004947843
DALYs (Dis	Global	Male	Age-standa	Non-melanoma skin cancer (basal-cell	Rate	1995	0.0118291	0.022337532	0.005222434
DALYs (Dis	Global	Female	Age-standa	Non-melanoma skin cancer (basal-cell	Rate	1995	0.0106298	0.02018334	0.004622012
DALYs (Dis	Global	Both	Age-standa	Non-melanoma skin cancer (basal-cell	Rate	1995	0.0111889	0.021401408	0.004863088
DALYs (Dis	Global	Male	Age-standa	Non-melanoma skin cancer (basal-cell	Rate	1993	0.0118444	0.022371637	0.005105925
DALYs (Dis	Global	Female	Age-standa	Non-melanoma skin cancer (basal-cell	Rate	1993	0.0106817	0.020032283	0.004634143
DALYs (Dis	Global	Both	Age-standa	Non-melanoma skin cancer (basal-cell	Rate	1993	0.0112231	0.021205371	0.004841416
DALYs (Dis	Global	Male	Age-standa	Non-melanoma skin cancer (basal-cell	Rate	1994	0.0118144	0.022341109	0.005279691
DALYs (Dis	Global	Female	Age-standa	Non-melanoma skin cancer (basal-cell	Rate	1994	0.0106688	0.020236271	0.004536491
DALYs (Dis	Global	Both	Age-standa	Non-melanoma skin cancer (basal-cell	Rate	1994	0.0112026	0.021306404	0.004887379
DALYs (Dis	Global	Male	Age-standa	Non-melanoma skin cancer (basal-cell	Rate	1992	0.0118233	0.022022444	0.00517904
DALYs (Dis	Global	Female	Age-standa	Non-melanoma skin cancer (basal-cell	Rate	1992	0.0107203	0.020319214	0.004625749
DALYs (Dis	Global	Both	Age-standa	Non-melanoma skin cancer (basal-cell	Rate	1992	0.0112334	0.021476879	0.004871734
DALYs (Dis	Global	Male	Age-standa	Non-melanoma skin cancer (basal-cell	Rate	1991	0.0118708	0.022471659	0.005197782
DALYs (Dis	Global	Female	Age-standa	Non-melanoma skin cancer (basal-cell	Rate	1991	0.0107345	0.020542172	0.004634758
DALYs (Dis	Global	Both	Age-standa	Non-melanoma skin cancer (basal-cell	Rate	1991	0.0112625	0.021540547	0.004876041
DALYs (Dis	Global	Male	Age-standa	Non-melanoma skin cancer (basal-cell	Rate	2002	0.0169161	0.032444171	0.007563366
DALYs (Dis	Global	Female	Age-standa	Non-melanoma skin cancer (basal-cell	Rate	2002	0.012561	0.024161744	0.005579264
DALYs (Dis	Global	Both	Age-standa	Non-melanoma skin cancer (basal-cell	Rate	2002	0.0145982	0.028006901	0.006508496
DALYs (Dis	Global	Male	Age-standa	Non-melanoma skin cancer (basal-cell	Rate	2000	0.0140689	0.026745823	0.006266605
DALYs (Dis	Global	Female	Age-standa	Non-melanoma skin cancer (basal-cell	Rate	2000	0.0110547	0.021409755	0.00478849
DALYs (Dis	Global	Both	Age-standa	Non-melanoma skin cancer (basal-cell	Rate	2000	0.0124629	0.023929136	0.005481773
DALYs (Dis	Global	Male	Age-standa	Non-melanoma skin cancer (basal-cell	Rate	2001	0.0151738	0.028670875	0.006753208
DALYs (Dis	Global	Female	Age-standa	Non-melanoma skin cancer (basal-cell	Rate	2001	0.0115354	0.022274608	0.005035338
DALYs (Dis	Global	Both	Age-standa	Non-melanoma skin cancer (basal-cell	Rate	2001	0.013236	0.025227142	0.005835049
DALYs (Dis	Global	Male	Age-standa	Non-melanoma skin cancer (basal-cell	Rate	1999	0.0134789	0.025756777	0.005952672
DALYs (Dis	Global	Female	Age-standa	Non-melanoma skin cancer (basal-cell	Rate	1999	0.010955	0.020691516	0.004803596
DALYs (Dis	Global	Both	Age-standa	Non-melanoma skin cancer (basal-cell	Rate	1999	0.0121337	0.023136782	0.005339901
DALYs (Dis	Global	Male	Age-standa	Non-melanoma skin cancer (basal-cell	Rate	1998	0.012864	0.024521915	0.005621383
DALYs (Dis	Global	Female	Age-standa	Non-melanoma skin cancer (basal-cell	Rate	1998	0.010842	0.020723832	0.004725478
DALYs (Dis	Global	Both	Age-standa	Non-melanoma skin cancer (basal-cell	Rate	1998	0.0117859	0.022679761	0.005153415
DALYs (Dis	Global	Male	Age-standa	Non-melanoma skin cancer (basal-cell	Rate	1997	0.0123391	0.023578821	0.00540234
DALYs (Dis	Global	Female	Age-standa	Non-melanoma skin cancer (basal-cell	Rate	1997	0.0107192	0.020450805	0.004643297
DALYs (Dis	Global	Both	Age-standa	Non-melanoma skin cancer (basal-cell	Rate	1997	0.011475	0.022073283	0.004987699
DALYs (Dis	Global	Male	Age-standa	Non-melanoma skin cancer (basal-cell	Rate	2003	0.0188859	0.036288066	0.008341206
DALYs (Dis	Global	Female	Age-standa	Non-melanoma skin cancer (basal-cell	Rate	2003	0.0137425	0.026272675	0.006130552
DALYs (Dis	Global	Both	Age-standa	Non-melanoma skin cancer (basal-cell	Rate	2003	0.0161506	0.031157126	0.007196088
DALYs (Dis	Global	Male	Age-standa	Non-melanoma skin cancer (basal-cell	Rate	2007	0.0209163	0.040458521	0.009615677
DALYs (Dis	Global	Female	Age-standa	Non-melanoma skin cancer (basal-cell	Rate	2007	0.0149195	0.028702661	0.006788864
DALYs (Dis	Global	Both	Age-standa	Non-melanoma skin cancer (basal-cell	Rate	2007	0.0177354	0.033970559	0.008067832

DALYs (Dis	Global	Male	Age-standa	Non-melanoma skin cancer (basal-cell	Rate	2006	0.0209509	0.040111877	0.00963324
DALYs (Dis	Global	Female	Age-standa	Non-melanoma skin cancer (basal-cell	Rate	2006	0.0149861	0.028837695	0.006730103
DALYs (Dis	Global	Both	Age-standa	Non-melanoma skin cancer (basal-cell	Rate	2006	0.0177851	0.034183837	0.008078534
DALYs (Dis	Global	Male	Age-standa	Non-melanoma skin cancer (basal-cell	Rate	2005	0.0210077	0.040633532	0.009517475
DALYs (Dis	Global	Female	Age-standa	Non-melanoma skin cancer (basal-cell	Rate	2005	0.0150816	0.02901893	0.006801759
DALYs (Dis	Global	Both	Age-standa	Non-melanoma skin cancer (basal-cell	Rate	2005	0.0178606	0.03443974	0.00807585
DALYs (Dis	Global	Male	Age-standa	Non-melanoma skin cancer (basal-cell	Rate	2004	0.0203396	0.039067977	0.009160615
DALYs (Dis	Global	Female	Age-standa	Non-melanoma skin cancer (basal-cell	Rate	2004	0.0146722	0.028173205	0.006623025
DALYs (Dis	Global	Both	Age-standa	Non-melanoma skin cancer (basal-cell	Rate	2004	0.0173285	0.033332175	0.007794347
DALYs (Dis	Global	Male	Age-standa	Non-melanoma skin cancer (basal-cell	Rate	2010	0.0207083	0.039833668	0.009565608
DALYs (Dis	Global	Female	Age-standa	Non-melanoma skin cancer (basal-cell	Rate	2010	0.014629	0.028073441	0.006689941
DALYs (Dis	Global	Both	Age-standa	Non-melanoma skin cancer (basal-cell	Rate	2010	0.0174886	0.033470914	0.008043425
DALYs (Dis	Global	Male	Age-standa	Non-melanoma skin cancer (basal-cell	Rate	2009	0.0207654	0.039897801	0.009467612
DALYs (Dis	Global	Female	Age-standa	Non-melanoma skin cancer (basal-cell	Rate	2009	0.0147538	0.028439678	0.006686722
DALYs (Dis	Global	Both	Age-standa	Non-melanoma skin cancer (basal-cell	Rate	2009	0.0175801	0.033689141	0.007994148
DALYs (Dis	Global	Male	Age-standa	Non-melanoma skin cancer (basal-cell	Rate	2013	0.020972	0.040349454	0.009639589
DALYs (Dis	Global	Female	Age-standa	Non-melanoma skin cancer (basal-cell	Rate	2013	0.0145598	0.027839429	0.006627021
DALYs (Dis	Global	Both	Age-standa	Non-melanoma skin cancer (basal-cell	Rate	2013	0.0175793	0.033823592	0.008058177
DALYs (Dis	Global	Male	Age-standa	Non-melanoma skin cancer (basal-cell	Rate	2008	0.020867	0.040259368	0.009526359
DALYs (Dis	Global	Female	Age-standa	Non-melanoma skin cancer (basal-cell	Rate	2008	0.014855	0.028582866	0.006774016
DALYs (Dis	Global	Both	Age-standa	Non-melanoma skin cancer (basal-cell	Rate	2008	0.0176799	0.033903016	0.00806946
DALYs (Dis	Global	Male	Age-standa	Non-melanoma skin cancer (basal-cell	Rate	2011	0.0207099	0.039875817	0.009654328
DALYs (Dis	Global	Female	Age-standa	Non-melanoma skin cancer (basal-cell	Rate	2011	0.0145367	0.028172544	0.006640523
DALYs (Dis	Global	Both	Age-standa	Non-melanoma skin cancer (basal-cell	Rate	2011	0.0174417	0.033519434	0.008076377
DALYs (Dis	Global	Male	Age-standa	Non-melanoma skin cancer (basal-cell	Rate	2012	0.0208114	0.040048414	0.009564245
DALYs (Dis	Global	Female	Age-standa	Non-melanoma skin cancer (basal-cell	Rate	2012	0.0145477	0.027842966	0.006721672
DALYs (Dis	Global	Both	Age-standa	Non-melanoma skin cancer (basal-cell	Rate	2012	0.0174964	0.033414453	0.008085453
DALYs (Dis	Global	Male	Age-standa	Non-melanoma skin cancer (basal-cell	Rate	2017	0.0210133	0.040100565	0.009718906
DALYs (Dis	Global	Female	Age-standa	Non-melanoma skin cancer (basal-cell	Rate	2017	0.014329	0.027122026	0.00651801
DALYs (Dis	Global	Both	Age-standa	Non-melanoma skin cancer (basal-cell	Rate	2017	0.0174785	0.033237169	0.007993042
DALYs (Dis	Global	Male	Age-standa	Non-melanoma skin cancer (basal-cell	Rate	2016	0.0211544	0.040907031	0.009949579
DALYs (Dis	Global	Female	Age-standa	Non-melanoma skin cancer (basal-cell	Rate	2016	0.0144695	0.027834011	0.006683384
DALYs (Dis	Global	Both	Age-standa	Non-melanoma skin cancer (basal-cell	Rate	2016	0.0176187	0.033990268	0.008236606
DALYs (Dis	Global	Male	Age-standa	Non-melanoma skin cancer (basal-cell	Rate	2014	0.0210988	0.040891282	0.009790651
DALYs (Dis	Global	Female	Age-standa	Non-melanoma skin cancer (basal-cell	Rate	2014	0.0145674	0.027783709	0.006568289
DALYs (Dis	Global	Both	Age-standa	Non-melanoma skin cancer (basal-cell	Rate	2014	0.0176435	0.033967312	0.008090084
DALYs (Dis	Global	Male	Age-standa	Non-melanoma skin cancer (basal-cell	Rate	2015	0.021203	0.040852029	0.009840312
DALYs (Dis	Global	Female	Age-standa	Non-melanoma skin cancer (basal-cell	Rate	2015	0.0145742	0.027605056	0.00663366
DALYs (Dis	Global	Both	Age-standa	Non-melanoma skin cancer (basal-cell	Rate	2015	0.0176965	0.033891531	0.008096192
DALYs (Dis	Global	Male	Age-standa	Non-melanoma skin cancer (basal-cell	Rate	2019	0.0208339	0.03978099	0.009556227
DALYs (Dis	Global	Female	Age-standa	Non-melanoma skin cancer (basal-cell	Rate	2019	0.0141206	0.027212629	0.006424377
DALYs (Dis	Global	Both	Age-standa	Non-melanoma skin cancer (basal-cell	Rate	2019	0.0172851	0.033192193	0.007908862
DALYs (Dis	Global	Male	Age-standa	Non-melanoma skin cancer (basal-cell	Rate	2018	0.0208654	0.040005041	0.009664696
DALYs (Dis	Global	Female	Age-standa	Non-melanoma skin cancer (basal-cell	Rate	2018	0.0141699	0.027159821	0.006462826
DALYs (Dis	Global	Both	Age-standa	Non-melanoma skin cancer (basal-cell	Rate	2018	0.0173254	0.033253332	0.007966157
DALYs (Dis	Global	Male	Age-standa	Non-melanoma skin cancer (basal-cell	Rate	2021	0.0222136	0.042145967	0.010194481
DALYs (Dis	Global	Female	Age-standa	Non-melanoma skin cancer (basal-cell	Rate	2021	0.0151213	0.028867586	0.006965557
DALYs (Dis	Global	Both	Age-standa	Non-melanoma skin cancer (basal-cell	Rate	2021	0.01846	0.035108355	0.008476581
DALYs (Dis	Global	Male	Age-standa	Non-melanoma skin cancer (basal-cell	Rate	2020	0.0209313	0.040029387	0.009636439
DALYs (Dis	Global	Female	Age-standa	Non-melanoma skin cancer (basal-cell	Rate	2020	0.0141498	0.02718458	0.006415516
DALYs (Dis	Global	Both	Age-standa	Non-melanoma skin cancer (basal-cell	Rate	2020	0.0173456	0.03317712	0.007933386
Prevalence	Global	Male	Age-standa	Non-melanoma skin cancer (basal-cell	Rate	1990	2.9086604	3.60788613	2.323859512
Prevalence	Global	Female	Age-standa	Non-melanoma skin cancer (basal-cell	Rate	1990	2.6111773	3.245762738	2.055803819
Prevalence	Global	Both	Age-standa	Non-melanoma skin cancer (basal-cell	Rate	1990	2.7491837	3.388576617	2.179473489
Prevalence	Global	Male	Age-standa	Non-melanoma skin cancer (basal-cell	Rate	1991	2.8908058	3.570042398	2.312488057
Prevalence	Global	Female	Age-standa	Non-melanoma skin cancer (basal-cell	Rate	1991	2.602935	3.222394706	2.050460197

Prevalence	Global	Both	Age-standa	Non-melanoma skin cancer (basal-cell	Rate	1991	2.7366848	3.365609443	2.171787022
Prevalence	Global	Male	Age-standa	Non-melanoma skin cancer (basal-cell	Rate	1992	2.880832	3.546444771	2.306633903
Prevalence	Global	Female	Age-standa	Non-melanoma skin cancer (basal-cell	Rate	1992	2.5955742	3.200978568	2.045375571
Prevalence	Global	Both	Age-standa	Non-melanoma skin cancer (basal-cell	Rate	1992	2.7282713	3.355602707	2.1657416
Prevalence	Global	Male	Age-standa	Non-melanoma skin cancer (basal-cell	Rate	1993	2.8781363	3.544852808	2.306198387
Prevalence	Global	Female	Age-standa	Non-melanoma skin cancer (basal-cell	Rate	1993	2.589981	3.188775334	2.041517253
Prevalence	Global	Both	Age-standa	Non-melanoma skin cancer (basal-cell	Rate	1993	2.7241509	3.348697972	2.162718651
Prevalence	Global	Male	Age-standa	Non-melanoma skin cancer (basal-cell	Rate	1994	2.8777255	3.539071974	2.305031234
Prevalence	Global	Female	Age-standa	Non-melanoma skin cancer (basal-cell	Rate	1994	2.5845471	3.177307144	2.037413248
Prevalence	Global	Both	Age-standa	Non-melanoma skin cancer (basal-cell	Rate	1994	2.7211686	3.343676379	2.160473663
Prevalence	Global	Male	Age-standa	Non-melanoma skin cancer (basal-cell	Rate	1995	2.8828775	3.538752555	2.306172042
Prevalence	Global	Female	Age-standa	Non-melanoma skin cancer (basal-cell	Rate	1995	2.5801964	3.168377922	2.035382752
Prevalence	Global	Both	Age-standa	Non-melanoma skin cancer (basal-cell	Rate	1995	2.7213161	3.34260043	2.160219586
Prevalence	Global	Male	Age-standa	Non-melanoma skin cancer (basal-cell	Rate	1996	2.9196694	3.574939414	2.335948642
Prevalence	Global	Female	Age-standa	Non-melanoma skin cancer (basal-cell	Rate	1996	2.5840854	3.166299397	2.040794665
Prevalence	Global	Both	Age-standa	Non-melanoma skin cancer (basal-cell	Rate	1996	2.7406181	3.356996835	2.177492938
Prevalence	Global	Male	Age-standa	Non-melanoma skin cancer (basal-cell	Rate	1997	3.0103491	3.673185074	2.410481619
Prevalence	Global	Female	Age-standa	Non-melanoma skin cancer (basal-cell	Rate	1997	2.6024264	3.179069881	2.056978202
Prevalence	Global	Both	Age-standa	Non-melanoma skin cancer (basal-cell	Rate	1997	2.7927602	3.40816555	2.222570603
Prevalence	Global	Male	Age-standa	Non-melanoma skin cancer (basal-cell	Rate	1998	3.14163	3.81792931	2.519279949
Prevalence	Global	Female	Age-standa	Non-melanoma skin cancer (basal-cell	Rate	1998	2.6330926	3.210027919	2.084842689
Prevalence	Global	Both	Age-standa	Non-melanoma skin cancer (basal-cell	Rate	1998	2.8704874	3.483830286	2.289271799
Prevalence	Global	Male	Age-standa	Non-melanoma skin cancer (basal-cell	Rate	1999	3.2888707	3.980553569	2.641048759
Prevalence	Global	Female	Age-standa	Non-melanoma skin cancer (basal-cell	Rate	1999	2.6637282	3.240870089	2.122683181
Prevalence	Global	Both	Age-standa	Non-melanoma skin cancer (basal-cell	Rate	1999	2.9556675	3.56811507	2.362134721
Prevalence	Global	Male	Age-standa	Non-melanoma skin cancer (basal-cell	Rate	2000	3.4415964	4.145495499	2.771380781
Prevalence	Global	Female	Age-standa	Non-melanoma skin cancer (basal-cell	Rate	2000	2.6920839	3.261611419	2.150602809
Prevalence	Global	Both	Age-standa	Non-melanoma skin cancer (basal-cell	Rate	2000	3.0422473	3.661455069	2.435524078
Prevalence	Global	Male	Age-standa	Non-melanoma skin cancer (basal-cell	Rate	2001	3.7135597	4.426425214	3.028349042
Prevalence	Global	Female	Age-standa	Non-melanoma skin cancer (basal-cell	Rate	2001	2.8128212	3.377342805	2.27602462
Prevalence	Global	Both	Age-standa	Non-melanoma skin cancer (basal-cell	Rate	2001	3.2338274	3.860844729	2.63264822
Prevalence	Global	Male	Age-standa	Non-melanoma skin cancer (basal-cell	Rate	2002	4.1520601	4.895378495	3.448712609
Prevalence	Global	Female	Age-standa	Non-melanoma skin cancer (basal-cell	Rate	2002	3.0669811	3.638328533	2.539413178
Prevalence	Global	Both	Age-standa	Non-melanoma skin cancer (basal-cell	Rate	2002	3.5745453	4.220365368	2.963500065
Prevalence	Global	Male	Age-standa	Non-melanoma skin cancer (basal-cell	Rate	2003	4.6348871	5.417134104	3.914044209
Prevalence	Global	Female	Age-standa	Non-melanoma skin cancer (basal-cell	Rate	2003	3.3641557	3.965166916	2.821780868
Prevalence	Global	Both	Age-standa	Non-melanoma skin cancer (basal-cell	Rate	2003	3.9591032	4.635526092	3.3315948
Prevalence	Global	Male	Age-standa	Non-melanoma skin cancer (basal-cell	Rate	2004	5.0025559	5.821724008	4.283247362
Prevalence	Global	Female	Age-standa	Non-melanoma skin cancer (basal-cell	Rate	2004	3.5949524	4.195521549	3.037253024
Prevalence	Global	Both	Age-standa	Non-melanoma skin cancer (basal-cell	Rate	2004	4.2547034	4.949209267	3.613864046
Prevalence	Global	Male	Age-standa	Non-melanoma skin cancer (basal-cell	Rate	2005	5.1712529	5.989097443	4.43574227
Prevalence	Global	Female	Age-standa	Non-melanoma skin cancer (basal-cell	Rate	2005	3.6930711	4.283791046	3.136718244
Prevalence	Global	Both	Age-standa	Non-melanoma skin cancer (basal-cell	Rate	2005	4.3862487	5.089831388	3.758403626
Prevalence	Global	Male	Age-standa	Non-melanoma skin cancer (basal-cell	Rate	2006	5.1550278	5.966735441	4.425543943
Prevalence	Global	Female	Age-standa	Non-melanoma skin cancer (basal-cell	Rate	2006	3.6725773	4.255085843	3.126117903
Prevalence	Global	Both	Age-standa	Non-melanoma skin cancer (basal-cell	Rate	2006	4.3682294	5.066155486	3.749171361
Prevalence	Global	Male	Age-standa	Non-melanoma skin cancer (basal-cell	Rate	2007	5.1439306	5.952371055	4.431519322
Prevalence	Global	Female	Age-standa	Non-melanoma skin cancer (basal-cell	Rate	2007	3.6542073	4.23148138	3.110462498
Prevalence	Global	Both	Age-standa	Non-melanoma skin cancer (basal-cell	Rate	2007	4.3537407	5.047045508	3.743836369
Prevalence	Global	Male	Age-standa	Non-melanoma skin cancer (basal-cell	Rate	2008	5.1354212	5.946949281	4.43927838
Prevalence	Global	Female	Age-standa	Non-melanoma skin cancer (basal-cell	Rate	2008	3.6356954	4.212938191	3.095069633
Prevalence	Global	Both	Age-standa	Non-melanoma skin cancer (basal-cell	Rate	2008	4.3403647	5.025833842	3.727091622
Prevalence	Global	Male	Age-standa	Non-melanoma skin cancer (basal-cell	Rate	2009	5.1165301	5.9375752	4.424099889
Prevalence	Global	Female	Age-standa	Non-melanoma skin cancer (basal-cell	Rate	2009	3.6107866	4.185028128	3.075690491
Prevalence	Global	Both	Age-standa	Non-melanoma skin cancer (basal-cell	Rate	2009	4.3187068	4.995949321	3.702415377
Prevalence	Global	Male	Age-standa	Non-melanoma skin cancer (basal-cell	Rate	2010	5.104912	5.936649152	4.411426675

Prevalence	Global	Female	Age-standa	Non-melanoma skin cancer (basal-cell	Rate	2010	3.585739	4.152296132	3.055863942
Prevalence	Global	Both	Age-standa	Non-melanoma skin cancer (basal-cell	Rate	2010	4.3003308	4.978738758	3.680714108
Prevalence	Global	Male	Age-standa	Non-melanoma skin cancer (basal-cell	Rate	2011	5.1008939	5.932473726	4.409935912
Prevalence	Global	Female	Age-standa	Non-melanoma skin cancer (basal-cell	Rate	2011	3.5659251	4.132539496	3.037380674
Prevalence	Global	Both	Age-standa	Non-melanoma skin cancer (basal-cell	Rate	2011	4.2882427	4.969867522	3.673217188
Prevalence	Global	Male	Age-standa	Non-melanoma skin cancer (basal-cell	Rate	2012	5.1261981	5.962153826	4.432203008
Prevalence	Global	Female	Age-standa	Non-melanoma skin cancer (basal-cell	Rate	2012	3.563344	4.134661387	3.031751186
Prevalence	Global	Both	Age-standa	Non-melanoma skin cancer (basal-cell	Rate	2012	4.2990776	4.988181343	3.684233364
Prevalence	Global	Male	Age-standa	Non-melanoma skin cancer (basal-cell	Rate	2013	5.1662436	6.007128577	4.464905252
Prevalence	Global	Female	Age-standa	Non-melanoma skin cancer (basal-cell	Rate	2013	3.5695506	4.147172388	3.035437518
Prevalence	Global	Both	Age-standa	Non-melanoma skin cancer (basal-cell	Rate	2013	4.3214457	5.019442035	3.704610355
Prevalence	Global	Male	Age-standa	Non-melanoma skin cancer (basal-cell	Rate	2014	5.1981857	6.043671274	4.485813461
Prevalence	Global	Female	Age-standa	Non-melanoma skin cancer (basal-cell	Rate	2014	3.5720512	4.160405776	3.037781634
Prevalence	Global	Both	Age-standa	Non-melanoma skin cancer (basal-cell	Rate	2014	4.3379255	5.044344198	3.718144102
Prevalence	Global	Male	Age-standa	Non-melanoma skin cancer (basal-cell	Rate	2015	5.2255569	6.078795254	4.508547735
Prevalence	Global	Female	Age-standa	Non-melanoma skin cancer (basal-cell	Rate	2015	3.5702686	4.171892956	3.036657958
Prevalence	Global	Both	Age-standa	Non-melanoma skin cancer (basal-cell	Rate	2015	4.3499573	5.064432292	3.725536211
Prevalence	Global	Male	Age-standa	Non-melanoma skin cancer (basal-cell	Rate	2016	5.2153758	6.077401079	4.484961891
Prevalence	Global	Female	Age-standa	Non-melanoma skin cancer (basal-cell	Rate	2016	3.5475605	4.147294652	3.011811257
Prevalence	Global	Both	Age-standa	Non-melanoma skin cancer (basal-cell	Rate	2016	4.333241	5.049959804	3.704380482
Prevalence	Global	Male	Age-standa	Non-melanoma skin cancer (basal-cell	Rate	2017	5.1871387	6.060495859	4.448794489
Prevalence	Global	Female	Age-standa	Non-melanoma skin cancer (basal-cell	Rate	2017	3.5137162	4.111014695	2.977177147
Prevalence	Global	Both	Age-standa	Non-melanoma skin cancer (basal-cell	Rate	2017	4.3021885	5.019910703	3.668510973
Prevalence	Global	Male	Age-standa	Non-melanoma skin cancer (basal-cell	Rate	2018	5.157788	6.040956352	4.412740752
Prevalence	Global	Female	Age-standa	Non-melanoma skin cancer (basal-cell	Rate	2018	3.4808074	4.07525435	2.942767707
Prevalence	Global	Both	Age-standa	Non-melanoma skin cancer (basal-cell	Rate	2018	4.2711397	4.989107126	3.627932254
Prevalence	Global	Male	Age-standa	Non-melanoma skin cancer (basal-cell	Rate	2019	5.1459531	6.033721129	4.395442541
Prevalence	Global	Female	Age-standa	Non-melanoma skin cancer (basal-cell	Rate	2019	3.4636973	4.060177946	2.923594894
Prevalence	Global	Both	Age-standa	Non-melanoma skin cancer (basal-cell	Rate	2019	4.2566855	4.975576158	3.602911553
Prevalence	Global	Male	Age-standa	Non-melanoma skin cancer (basal-cell	Rate	2020	5.1667081	6.068432939	4.404110497
Prevalence	Global	Female	Age-standa	Non-melanoma skin cancer (basal-cell	Rate	2020	3.4681587	4.075049628	2.913558342
Prevalence	Global	Both	Age-standa	Non-melanoma skin cancer (basal-cell	Rate	2020	4.2686052	5.005629341	3.61397366
Prevalence	Global	Male	Age-standa	Non-melanoma skin cancer (basal-cell	Rate	2021	5.4803386	6.453508235	4.662542437
Prevalence	Global	Female	Age-standa	Non-melanoma skin cancer (basal-cell	Rate	2021	3.683766	4.35315374	3.080766082
Prevalence	Global	Both	Age-standa	Non-melanoma skin cancer (basal-cell	Rate	2021	4.5294903	5.330668584	3.822566787
Incidence	Global	Male	Age-standa	Non-melanoma skin cancer (basal-cell	Rate	1990	26.180309	31.43436225	21.16129685
Incidence	Global	Female	Age-standa	Non-melanoma skin cancer (basal-cell	Rate	1990	21.554507	26.00284527	17.22661029
Incidence	Global	Both	Age-standa	Non-melanoma skin cancer (basal-cell	Rate	1990	23.700479	28.41114536	19.07913286
Incidence	Global	Male	Age-standa	Non-melanoma skin cancer (basal-cell	Rate	1991	26.070206	31.21609307	21.10799236
Incidence	Global	Female	Age-standa	Non-melanoma skin cancer (basal-cell	Rate	1991	21.538261	25.921287	17.26154857
Incidence	Global	Both	Age-standa	Non-melanoma skin cancer (basal-cell	Rate	1991	23.643882	28.31511518	19.07877498
Incidence	Global	Male	Age-standa	Non-melanoma skin cancer (basal-cell	Rate	1992	26.015013	31.07269672	21.09815674
Incidence	Global	Female	Age-standa	Non-melanoma skin cancer (basal-cell	Rate	1992	21.517859	25.83835616	17.29384124
Incidence	Global	Both	Age-standa	Non-melanoma skin cancer (basal-cell	Rate	1992	23.609858	28.26833778	19.09415528
Incidence	Global	Male	Age-standa	Non-melanoma skin cancer (basal-cell	Rate	1993	26.013875	31.00154728	21.13054495
Incidence	Global	Female	Age-standa	Non-melanoma skin cancer (basal-cell	Rate	1993	21.503161	25.78496703	17.33031291
Incidence	Global	Both	Age-standa	Non-melanoma skin cancer (basal-cell	Rate	1993	23.603425	28.2627499	19.12874141
Incidence	Global	Male	Age-standa	Non-melanoma skin cancer (basal-cell	Rate	1994	26.020244	31.00609906	21.16790054
Incidence	Global	Female	Age-standa	Non-melanoma skin cancer (basal-cell	Rate	1994	21.478303	25.73009626	17.35766296
Incidence	Global	Both	Age-standa	Non-melanoma skin cancer (basal-cell	Rate	1994	23.594853	28.23215716	19.15942937
Incidence	Global	Male	Age-standa	Non-melanoma skin cancer (basal-cell	Rate	1995	26.072882	31.07725401	21.23970837
Incidence	Global	Female	Age-standa	Non-melanoma skin cancer (basal-cell	Rate	1995	21.456897	25.69067927	17.38414049
Incidence	Global	Both	Age-standa	Non-melanoma skin cancer (basal-cell	Rate	1995	23.609017	28.2269643	19.20479029
Incidence	Global	Male	Age-standa	Non-melanoma skin cancer (basal-cell	Rate	1996	26.434677	31.40407862	21.59857124
Incidence	Global	Female	Age-standa	Non-melanoma skin cancer (basal-cell	Rate	1996	21.493266	25.62102444	17.49181908
Incidence	Global	Both	Age-standa	Non-melanoma skin cancer (basal-cell	Rate	1996	23.798181	28.2943657	19.46123745

Incidence	Global	Male	Age-standa	Non-melanoma skin cancer (basal-cell	Rate	1997	27.332198	32.36302684	22.49691974
Incidence	Global	Female	Age-standa	Non-melanoma skin cancer (basal-cell	Rate	1997	21.645358	25.62118525	17.71523551
Incidence	Global	Both	Age-standa	Non-melanoma skin cancer (basal-cell	Rate	1997	24.298796	28.7680299	19.96995416
Incidence	Global	Male	Age-standa	Non-melanoma skin cancer (basal-cell	Rate	1998	28.638599	33.74709986	23.66898874
Incidence	Global	Female	Age-standa	Non-melanoma skin cancer (basal-cell	Rate	1998	21.905147	25.84230006	18.01984911
Incidence	Global	Both	Age-standa	Non-melanoma skin cancer (basal-cell	Rate	1998	25.048449	29.53207539	20.65464697
Incidence	Global	Male	Age-standa	Non-melanoma skin cancer (basal-cell	Rate	1999	30.113061	35.28397948	24.88042753
Incidence	Global	Female	Age-standa	Non-melanoma skin cancer (basal-cell	Rate	1999	22.169032	26.09510955	18.2905669
Incidence	Global	Both	Age-standa	Non-melanoma skin cancer (basal-cell	Rate	1999	25.878865	30.34213974	21.38466027
Incidence	Global	Male	Age-standa	Non-melanoma skin cancer (basal-cell	Rate	2000	31.658416	36.86033011	26.2314734
Incidence	Global	Female	Age-standa	Non-melanoma skin cancer (basal-cell	Rate	2000	22.429619	26.26757481	18.59292841
Incidence	Global	Both	Age-standa	Non-melanoma skin cancer (basal-cell	Rate	2000	26.741205	31.19486133	22.10637549
Incidence	Global	Male	Age-standa	Non-melanoma skin cancer (basal-cell	Rate	2001	34.422645	39.66460082	29.02648639
Incidence	Global	Female	Age-standa	Non-melanoma skin cancer (basal-cell	Rate	2001	23.683157	27.41593784	19.91352931
Incidence	Global	Both	Age-standa	Non-melanoma skin cancer (basal-cell	Rate	2001	28.702805	33.14607872	24.16383582
Incidence	Global	Male	Age-standa	Non-melanoma skin cancer (basal-cell	Rate	2002	38.883184	44.30419738	33.43230148
Incidence	Global	Female	Age-standa	Non-melanoma skin cancer (basal-cell	Rate	2002	26.34996	30.10540535	22.640026
Incidence	Global	Both	Age-standa	Non-melanoma skin cancer (basal-cell	Rate	2002	32.212589	36.78909849	27.71745403
Incidence	Global	Male	Age-standa	Non-melanoma skin cancer (basal-cell	Rate	2003	43.801699	49.32067615	38.13360709
Incidence	Global	Female	Age-standa	Non-melanoma skin cancer (basal-cell	Rate	2003	29.470236	33.24520897	25.77619741
Incidence	Global	Both	Age-standa	Non-melanoma skin cancer (basal-cell	Rate	2003	36.180126	40.81891034	31.63345939
Incidence	Global	Male	Age-standa	Non-melanoma skin cancer (basal-cell	Rate	2004	47.571623	53.08865387	41.9692599
Incidence	Global	Female	Age-standa	Non-melanoma skin cancer (basal-cell	Rate	2004	31.916534	35.58975604	28.11617591
Incidence	Global	Both	Age-standa	Non-melanoma skin cancer (basal-cell	Rate	2004	39.254155	43.75608692	34.59714497
Incidence	Global	Male	Age-standa	Non-melanoma skin cancer (basal-cell	Rate	2005	49.335705	54.61096607	43.70015242
Incidence	Global	Female	Age-standa	Non-melanoma skin cancer (basal-cell	Rate	2005	32.973203	36.53061667	29.12441161
Incidence	Global	Both	Age-standa	Non-melanoma skin cancer (basal-cell	Rate	2005	40.646224	45.0141553	35.9778602
Incidence	Global	Male	Age-standa	Non-melanoma skin cancer (basal-cell	Rate	2006	49.25313	54.58917857	43.68802896
Incidence	Global	Female	Age-standa	Non-melanoma skin cancer (basal-cell	Rate	2006	32.81854	36.37571839	28.93446303
Incidence	Global	Both	Age-standa	Non-melanoma skin cancer (basal-cell	Rate	2006	40.530606	44.87955155	35.89019307
Incidence	Global	Male	Age-standa	Non-melanoma skin cancer (basal-cell	Rate	2007	49.234167	54.52043905	43.74742416
Incidence	Global	Female	Age-standa	Non-melanoma skin cancer (basal-cell	Rate	2007	32.691512	36.30656621	28.79418089
Incidence	Global	Both	Age-standa	Non-melanoma skin cancer (basal-cell	Rate	2007	40.459492	44.7793755	35.87218812
Incidence	Global	Male	Age-standa	Non-melanoma skin cancer (basal-cell	Rate	2008	49.235313	54.47987421	43.85947406
Incidence	Global	Female	Age-standa	Non-melanoma skin cancer (basal-cell	Rate	2008	32.564756	36.20914275	28.83351245
Incidence	Global	Both	Age-standa	Non-melanoma skin cancer (basal-cell	Rate	2008	40.397674	44.74824548	35.96094293
Incidence	Global	Male	Age-standa	Non-melanoma skin cancer (basal-cell	Rate	2009	49.110672	54.36872387	43.90013738
Incidence	Global	Female	Age-standa	Non-melanoma skin cancer (basal-cell	Rate	2009	32.372876	36.01910492	28.7223091
Incidence	Global	Both	Age-standa	Non-melanoma skin cancer (basal-cell	Rate	2009	40.242094	44.61578248	35.86747544
Incidence	Global	Male	Age-standa	Non-melanoma skin cancer (basal-cell	Rate	2010	49.03196	54.35669898	43.85332895
Incidence	Global	Female	Age-standa	Non-melanoma skin cancer (basal-cell	Rate	2010	32.175594	35.80974489	28.55637641
Incidence	Global	Both	Age-standa	Non-melanoma skin cancer (basal-cell	Rate	2010	40.104527	44.50322041	35.75538792
Incidence	Global	Male	Age-standa	Non-melanoma skin cancer (basal-cell	Rate	2011	48.990998	54.31895934	43.77209863
Incidence	Global	Female	Age-standa	Non-melanoma skin cancer (basal-cell	Rate	2011	32.011744	35.59239403	28.41853804
Incidence	Global	Both	Age-standa	Non-melanoma skin cancer (basal-cell	Rate	2011	40.001753	44.35282494	35.65194666
Incidence	Global	Male	Age-standa	Non-melanoma skin cancer (basal-cell	Rate	2012	49.221667	54.56200758	43.92264241
Incidence	Global	Female	Age-standa	Non-melanoma skin cancer (basal-cell	Rate	2012	31.997867	35.56077752	28.41302753
Incidence	Global	Both	Age-standa	Non-melanoma skin cancer (basal-cell	Rate	2012	40.106192	44.48558801	35.76609704
Incidence	Global	Male	Age-standa	Non-melanoma skin cancer (basal-cell	Rate	2013	49.591494	55.03124074	44.21919812
Incidence	Global	Female	Age-standa	Non-melanoma skin cancer (basal-cell	Rate	2013	32.061846	35.67799146	28.47709262
Incidence	Global	Both	Age-standa	Non-melanoma skin cancer (basal-cell	Rate	2013	40.316693	44.74004691	35.95098042
Incidence	Global	Male	Age-standa	Non-melanoma skin cancer (basal-cell	Rate	2014	49.882276	55.38749084	44.48564619
Incidence	Global	Female	Age-standa	Non-melanoma skin cancer (basal-cell	Rate	2014	32.090303	35.71870038	28.46728058
Incidence	Global	Both	Age-standa	Non-melanoma skin cancer (basal-cell	Rate	2014	40.469939	44.95616549	36.04857154
Incidence	Global	Male	Age-standa	Non-melanoma skin cancer (basal-cell	Rate	2015	50.149598	55.71037766	44.74934856
Incidence	Global	Female	Age-standa	Non-melanoma skin cancer (basal-cell	Rate	2015	32.087719	35.71626497	28.39413279

Incidence	Global	Both	Age-standa	Non-melanoma skin cancer (basal-cell	Rate	2015	40.595387	45.09779447	36.10857937
Incidence	Global	Male	Age-standa	Non-melanoma skin cancer (basal-cell	Rate	2016	50.083282	55.65550253	44.5375226
Incidence	Global	Female	Age-standa	Non-melanoma skin cancer (basal-cell	Rate	2016	31.907577	35.58064275	28.19675776
Incidence	Global	Both	Age-standa	Non-melanoma skin cancer (basal-cell	Rate	2016	40.469854	44.96875931	35.91949358
Incidence	Global	Male	Age-standa	Non-melanoma skin cancer (basal-cell	Rate	2017	49.870861	55.55099608	44.11561172
Incidence	Global	Female	Age-standa	Non-melanoma skin cancer (basal-cell	Rate	2017	31.636609	35.36945134	27.90263751
Incidence	Global	Both	Age-standa	Non-melanoma skin cancer (basal-cell	Rate	2017	40.228105	44.79264766	35.5938735
Incidence	Global	Male	Age-standa	Non-melanoma skin cancer (basal-cell	Rate	2018	49.644046	55.36608033	43.6739707
Incidence	Global	Female	Age-standa	Non-melanoma skin cancer (basal-cell	Rate	2018	31.367616	35.12558884	27.60232918
Incidence	Global	Both	Age-standa	Non-melanoma skin cancer (basal-cell	Rate	2018	39.980985	44.60898925	35.25951189
Incidence	Global	Male	Age-standa	Non-melanoma skin cancer (basal-cell	Rate	2019	49.548553	55.35579489	43.41384615
Incidence	Global	Female	Age-standa	Non-melanoma skin cancer (basal-cell	Rate	2019	31.217696	35.01881822	27.44868089
Incidence	Global	Both	Age-standa	Non-melanoma skin cancer (basal-cell	Rate	2019	39.858566	44.53736628	35.04611172
Incidence	Global	Male	Age-standa	Non-melanoma skin cancer (basal-cell	Rate	2020	49.73709	55.59724079	43.59445277
Incidence	Global	Female	Age-standa	Non-melanoma skin cancer (basal-cell	Rate	2020	31.16119	34.98848724	27.36076023
Incidence	Global	Both	Age-standa	Non-melanoma skin cancer (basal-cell	Rate	2020	39.915139	44.76489797	35.06457985
Incidence	Global	Male	Age-standa	Non-melanoma skin cancer (basal-cell	Rate	2021	52.577656	59.0113574	45.97896519
Incidence	Global	Female	Age-standa	Non-melanoma skin cancer (basal-cell	Rate	2021	33.073242	37.40180321	28.84539076
Incidence	Global	Both	Age-standa	Non-melanoma skin cancer (basal-cell	Rate	2021	42.254811	47.56354512	36.95951117

Supplementary Data 2

Trends in national burden of 55+ year BCC from 1990 to 2021

DALYs (Dis	Timor-Lest	Female	55+ years	Non-melanoma skin cancer (basal-cell	Rate	1990	0.0018855	0.003725168	0.000724887
DALYs (Dis	Timor-Lest	Both	55+ years	Non-melanoma skin cancer (basal-cell	Rate	1990	0.0022867	0.004492653	0.000859107
DALYs (Dis	Colombia	Male	55+ years	Non-melanoma skin cancer (basal-cell	Number	1990	0.9472857	1.906090914	0.412930893
DALYs (Dis	Colombia	Female	55+ years	Non-melanoma skin cancer (basal-cell	Number	1990	0.9219098	1.783222058	0.382177825
DALYs (Dis	Colombia	Both	55+ years	Non-melanoma skin cancer (basal-cell	Number	1990	1.8691955	3.704220782	0.79007915
DALYs (Dis	Colombia	Male	55+ years	Non-melanoma skin cancer (basal-cell	Percent	1990	8.36E-07	1.64E-06	3.69E-07
DALYs (Dis	Colombia	Female	55+ years	Non-melanoma skin cancer (basal-cell	Percent	1990	8.80E-07	1.65E-06	3.86E-07
DALYs (Dis	Colombia	Both	55+ years	Non-melanoma skin cancer (basal-cell	Percent	1990	8.57E-07	1.62E-06	3.76E-07
DALYs (Dis	Colombia	Male	55+ years	Non-melanoma skin cancer (basal-cell	Rate	1990	0.0677593	0.136342608	0.02953693
DALYs (Dis	Colombia	Female	55+ years	Non-melanoma skin cancer (basal-cell	Rate	1990	0.0622502	0.120408662	0.025805827
DALYs (Dis	Colombia	Both	55+ years	Non-melanoma skin cancer (basal-cell	Rate	1990	0.0649254	0.128663877	0.027442923
DALYs (Dis	Thailand	Male	55+ years	Non-melanoma skin cancer (basal-cell	Number	1990	0.4284361	0.83974947	0.193990797
DALYs (Dis	Thailand	Female	55+ years	Non-melanoma skin cancer (basal-cell	Number	1990	0.4929241	0.995119052	0.221871524
DALYs (Dis	Thailand	Both	55+ years	Non-melanoma skin cancer (basal-cell	Number	1990	0.9213602	1.824359024	0.418109044
DALYs (Dis	Thailand	Male	55+ years	Non-melanoma skin cancer (basal-cell	Percent	1990	1.73E-07	3.30E-07	7.98E-08
DALYs (Dis	Thailand	Female	55+ years	Non-melanoma skin cancer (basal-cell	Percent	1990	2.24E-07	4.30E-07	1.04E-07
DALYs (Dis	Thailand	Both	55+ years	Non-melanoma skin cancer (basal-cell	Percent	1990	1.97E-07	3.76E-07	9.14E-08
DALYs (Dis	Thailand	Male	55+ years	Non-melanoma skin cancer (basal-cell	Rate	1990	0.0154827	0.030346704	0.007010402
DALYs (Dis	Thailand	Female	55+ years	Non-melanoma skin cancer (basal-cell	Rate	1990	0.0155639	0.031420535	0.007005515
DALYs (Dis	Thailand	Both	55+ years	Non-melanoma skin cancer (basal-cell	Rate	1990	0.0155261	0.030742704	0.007045654
DALYs (Dis	Guinea	Male	55+ years	Non-melanoma skin cancer (basal-cell	Number	1990	0.0206586	0.041376151	0.008331169
DALYs (Dis	Guinea	Female	55+ years	Non-melanoma skin cancer (basal-cell	Number	1990	0.0127502	0.025356424	0.005240651
DALYs (Dis	Guinea	Both	55+ years	Non-melanoma skin cancer (basal-cell	Number	1990	0.0334088	0.065300385	0.013739772
DALYs (Dis	Guinea	Male	55+ years	Non-melanoma skin cancer (basal-cell	Percent	1990	6.26E-08	1.24E-07	2.63E-08
DALYs (Dis	Guinea	Female	55+ years	Non-melanoma skin cancer (basal-cell	Percent	1990	3.72E-08	7.24E-08	1.56E-08
DALYs (Dis	Guinea	Both	55+ years	Non-melanoma skin cancer (basal-cell	Percent	1990	4.97E-08	9.70E-08	2.07E-08
DALYs (Dis	Guinea	Male	55+ years	Non-melanoma skin cancer (basal-cell	Rate	1990	0.0073705	0.014761936	0.002972345
DALYs (Dis	Guinea	Female	55+ years	Non-melanoma skin cancer (basal-cell	Rate	1990	0.0046686	0.009284498	0.001918915
DALYs (Dis	Guinea	Both	55+ years	Non-melanoma skin cancer (basal-cell	Rate	1990	0.0060371	0.011799972	0.002482817
DALYs (Dis	Canada	Male	55+ years	Non-melanoma skin cancer (basal-cell	Number	1990	2.0200073	4.160849736	0.801021173
DALYs (Dis	Canada	Female	55+ years	Non-melanoma skin cancer (basal-cell	Number	1990	2.3356066	4.672220737	0.941751807
DALYs (Dis	Canada	Both	55+ years	Non-melanoma skin cancer (basal-cell	Number	1990	4.3556139	8.656565809	1.7894439
DALYs (Dis	Canada	Male	55+ years	Non-melanoma skin cancer (basal-cell	Percent	1990	9.69E-07	1.94E-06	3.87E-07
DALYs (Dis	Canada	Female	55+ years	Non-melanoma skin cancer (basal-cell	Percent	1990	1.23E-06	2.44E-06	5.11E-07
DALYs (Dis	Canada	Both	55+ years	Non-melanoma skin cancer (basal-cell	Percent	1990	1.09E-06	2.13E-06	4.66E-07

DALYs (Dis	Canada	Male	55+ years	Non-melanoma skin cancer (basal-cell	Rate	1990	0.0821449	0.169203625	0.03257404
DALYs (Dis	Canada	Female	55+ years	Non-melanoma skin cancer (basal-cell	Rate	1990	0.0778552	0.155744014	0.031392397
DALYs (Dis	Canada	Both	55+ years	Non-melanoma skin cancer (basal-cell	Rate	1990	0.0797876	0.158573787	0.032779615
DALYs (Dis	Seychelles	Male	55+ years	Non-melanoma skin cancer (basal-cell	Number	1990	0.0001172	0.000234011	3.91E-05
DALYs (Dis	Seychelles	Female	55+ years	Non-melanoma skin cancer (basal-cell	Number	1990	0.0001478	0.000297078	5.04E-05
DALYs (Dis	Seychelles	Both	55+ years	Non-melanoma skin cancer (basal-cell	Number	1990	0.0002649	0.000526338	8.96E-05
DALYs (Dis	Seychelles	Male	55+ years	Non-melanoma skin cancer (basal-cell	Percent	1990	2.19E-08	4.25E-08	7.48E-09
DALYs (Dis	Seychelles	Female	55+ years	Non-melanoma skin cancer (basal-cell	Percent	1990	3.53E-08	6.91E-08	1.21E-08
DALYs (Dis	Seychelles	Both	55+ years	Non-melanoma skin cancer (basal-cell	Percent	1990	2.78E-08	5.45E-08	9.44E-09
DALYs (Dis	Seychelles	Male	55+ years	Non-melanoma skin cancer (basal-cell	Rate	1990	0.0028002	0.005593447	0.000934498
DALYs (Dis	Seychelles	Female	55+ years	Non-melanoma skin cancer (basal-cell	Rate	1990	0.0027323	0.005492952	0.000931876
DALYs (Dis	Seychelles	Both	55+ years	Non-melanoma skin cancer (basal-cell	Rate	1990	0.0027619	0.005487245	0.000934355
DALYs (Dis	Guyana	Male	55+ years	Non-melanoma skin cancer (basal-cell	Number	1990	0.0037569	0.007330408	0.001573983
DALYs (Dis	Guyana	Female	55+ years	Non-melanoma skin cancer (basal-cell	Number	1990	0.0015398	0.0030171	0.000623626
DALYs (Dis	Guyana	Both	55+ years	Non-melanoma skin cancer (basal-cell	Number	1990	0.0052967	0.010207232	0.002221365
DALYs (Dis	Guyana	Male	55+ years	Non-melanoma skin cancer (basal-cell	Percent	1990	8.74E-08	1.71E-07	3.55E-08
DALYs (Dis	Guyana	Female	55+ years	Non-melanoma skin cancer (basal-cell	Percent	1990	4.25E-08	8.26E-08	1.78E-08
DALYs (Dis	Guyana	Both	55+ years	Non-melanoma skin cancer (basal-cell	Percent	1990	6.68E-08	1.29E-07	2.79E-08
DALYs (Dis	Guyana	Male	55+ years	Non-melanoma skin cancer (basal-cell	Rate	1990	0.0127499	0.02487771	0.005341733
DALYs (Dis	Guyana	Female	55+ years	Non-melanoma skin cancer (basal-cell	Rate	1990	0.0048373	0.00947817	0.001959111
DALYs (Dis	Guyana	Both	55+ years	Non-melanoma skin cancer (basal-cell	Rate	1990	0.0086409	0.016651858	0.003623887
DALYs (Dis	Slovakia	Male	55+ years	Non-melanoma skin cancer (basal-cell	Number	1990	0.3875976	0.75951109	0.165724514
DALYs (Dis	Slovakia	Female	55+ years	Non-melanoma skin cancer (basal-cell	Number	1990	0.4787129	0.974522591	0.200184674
DALYs (Dis	Slovakia	Both	55+ years	Non-melanoma skin cancer (basal-cell	Number	1990	0.8663105	1.707041708	0.371181542
DALYs (Dis	Slovakia	Male	55+ years	Non-melanoma skin cancer (basal-cell	Percent	1990	6.58E-07	1.28E-06	2.92E-07
DALYs (Dis	Slovakia	Female	55+ years	Non-melanoma skin cancer (basal-cell	Percent	1990	9.30E-07	1.82E-06	4.06E-07
DALYs (Dis	Slovakia	Both	55+ years	Non-melanoma skin cancer (basal-cell	Percent	1990	7.85E-07	1.50E-06	3.51E-07
DALYs (Dis	Slovakia	Male	55+ years	Non-melanoma skin cancer (basal-cell	Rate	1990	0.0878315	0.172108883	0.037553976
DALYs (Dis	Slovakia	Female	55+ years	Non-melanoma skin cancer (basal-cell	Rate	1990	0.0802996	0.16346708	0.033579113
DALYs (Dis	Slovakia	Both	55+ years	Non-melanoma skin cancer (basal-cell	Rate	1990	0.0835034	0.16454125	0.03577808
DALYs (Dis	Greenland	Male	55+ years	Non-melanoma skin cancer (basal-cell	Number	1990	0.0030123	0.006099001	0.00120414
DALYs (Dis	Greenland	Female	55+ years	Non-melanoma skin cancer (basal-cell	Number	1990	0.0029345	0.005890725	0.001191181
DALYs (Dis	Greenland	Both	55+ years	Non-melanoma skin cancer (basal-cell	Number	1990	0.0059468	0.011555338	0.002462672
DALYs (Dis	Greenland	Male	55+ years	Non-melanoma skin cancer (basal-cell	Percent	1990	9.07E-07	1.79E-06	3.67E-07
DALYs (Dis	Greenland	Female	55+ years	Non-melanoma skin cancer (basal-cell	Percent	1990	9.44E-07	1.90E-06	3.91E-07
DALYs (Dis	Greenland	Both	55+ years	Non-melanoma skin cancer (basal-cell	Percent	1990	9.24E-07	1.80E-06	3.86E-07
DALYs (Dis	Greenland	Male	55+ years	Non-melanoma skin cancer (basal-cell	Rate	1990	0.1118289	0.226422814	0.044703179
DALYs (Dis	Greenland	Female	55+ years	Non-melanoma skin cancer (basal-cell	Rate	1990	0.1041885	0.209144984	0.042291831
DALYs (Dis	Greenland	Both	55+ years	Non-melanoma skin cancer (basal-cell	Rate	1990	0.1079235	0.209707824	0.0446929
DALYs (Dis	Rwanda	Male	55+ years	Non-melanoma skin cancer (basal-cell	Number	1990	0.0202838	0.039702289	0.008335135
DALYs (Dis	Rwanda	Female	55+ years	Non-melanoma skin cancer (basal-cell	Number	1990	0.0159274	0.031592636	0.006468988
DALYs (Dis	Rwanda	Both	55+ years	Non-melanoma skin cancer (basal-cell	Number	1990	0.0362112	0.072054475	0.014810605
DALYs (Dis	Rwanda	Male	55+ years	Non-melanoma skin cancer (basal-cell	Percent	1990	5.14E-08	1.00E-07	2.18E-08
DALYs (Dis	Rwanda	Female	55+ years	Non-melanoma skin cancer (basal-cell	Percent	1990	4.03E-08	7.82E-08	1.65E-08
DALYs (Dis	Rwanda	Both	55+ years	Non-melanoma skin cancer (basal-cell	Percent	1990	4.58E-08	8.79E-08	1.96E-08
DALYs (Dis	Rwanda	Male	55+ years	Non-melanoma skin cancer (basal-cell	Rate	1990	0.0097626	0.019108601	0.004011677
DALYs (Dis	Rwanda	Female	55+ years	Non-melanoma skin cancer (basal-cell	Rate	1990	0.0061218	0.012142754	0.002486381
DALYs (Dis	Rwanda	Both	55+ years	Non-melanoma skin cancer (basal-cell	Rate	1990	0.0077383	0.015397944	0.003165006
DALYs (Dis	China	Male	55+ years	Non-melanoma skin cancer (basal-cell	Number	1990	5.6208006	10.95188615	2.43307582
DALYs (Dis	China	Female	55+ years	Non-melanoma skin cancer (basal-cell	Number	1990	5.8467486	11.25670861	2.451742923
DALYs (Dis	China	Both	55+ years	Non-melanoma skin cancer (basal-cell	Number	1990	11.467549	22.20324041	4.88493904
DALYs (Dis	China	Male	55+ years	Non-melanoma skin cancer (basal-cell	Percent	1990	7.69E-08	1.50E-07	3.27E-08
DALYs (Dis	China	Female	55+ years	Non-melanoma skin cancer (basal-cell	Percent	1990	9.04E-08	1.75E-07	3.86E-08
DALYs (Dis	China	Both	55+ years	Non-melanoma skin cancer (basal-cell	Percent	1990	8.30E-08	1.59E-07	3.54E-08
DALYs (Dis	China	Male	55+ years	Non-melanoma skin cancer (basal-cell	Rate	1990	0.0079569	0.015503728	0.003444315
DALYs (Dis	China	Female	55+ years	Non-melanoma skin cancer (basal-cell	Rate	1990	0.0080227	0.015446031	0.003364189

DALYs (Dis	China	Both	55+ years	Non-melanoma skin cancer (basal-cell	Rate	1990	0.0079903	0.015470699	0.003403711
DALYs (Dis	Bangladesh	Male	55+ years	Non-melanoma skin cancer (basal-cell	Number	1990	0.0050333	0.012805868	0.00106569
DALYs (Dis	Bangladesh	Female	55+ years	Non-melanoma skin cancer (basal-cell	Number	1990	0.0027734	0.00718075	0.000632994
DALYs (Dis	Bangladesh	Both	55+ years	Non-melanoma skin cancer (basal-cell	Number	1990	0.0078067	0.018638978	0.00208029
DALYs (Dis	Bangladesh	Male	55+ years	Non-melanoma skin cancer (basal-cell	Percent	1990	9.45E-10	2.49E-09	2.00E-10
DALYs (Dis	Bangladesh	Female	55+ years	Non-melanoma skin cancer (basal-cell	Percent	1990	8.06E-10	2.02E-09	1.83E-10
DALYs (Dis	Bangladesh	Both	55+ years	Non-melanoma skin cancer (basal-cell	Percent	1990	8.90E-10	2.10E-09	2.37E-10
DALYs (Dis	Bangladesh	Male	55+ years	Non-melanoma skin cancer (basal-cell	Rate	1990	0.0001195	0.000303954	2.53E-05
DALYs (Dis	Bangladesh	Female	55+ years	Non-melanoma skin cancer (basal-cell	Rate	1990	8.25E-05	0.000213613	1.88E-05
DALYs (Dis	Bangladesh	Both	55+ years	Non-melanoma skin cancer (basal-cell	Rate	1990	0.0001031	0.00024607	2.75E-05
DALYs (Dis	Denmark	Male	55+ years	Non-melanoma skin cancer (basal-cell	Number	1990	0.7978465	1.661730612	0.336275331
DALYs (Dis	Denmark	Female	55+ years	Non-melanoma skin cancer (basal-cell	Number	1990	0.8530553	1.75108512	0.358397534
DALYs (Dis	Denmark	Both	55+ years	Non-melanoma skin cancer (basal-cell	Number	1990	1.6509019	3.236298317	0.735121985
DALYs (Dis	Denmark	Male	55+ years	Non-melanoma skin cancer (basal-cell	Percent	1990	1.34E-06	2.73E-06	5.73E-07
DALYs (Dis	Denmark	Female	55+ years	Non-melanoma skin cancer (basal-cell	Percent	1990	1.43E-06	2.84E-06	6.08E-07
DALYs (Dis	Denmark	Both	55+ years	Non-melanoma skin cancer (basal-cell	Percent	1990	1.39E-06	2.70E-06	6.46E-07
DALYs (Dis	Denmark	Male	55+ years	Non-melanoma skin cancer (basal-cell	Rate	1990	0.1389013	0.289299488	0.058543954
DALYs (Dis	Denmark	Female	55+ years	Non-melanoma skin cancer (basal-cell	Rate	1990	0.1168193	0.239797516	0.04907976
DALYs (Dis	Denmark	Both	55+ years	Non-melanoma skin cancer (basal-cell	Rate	1990	0.1265415	0.248061982	0.056347036
DALYs (Dis	Bulgaria	Male	55+ years	Non-melanoma skin cancer (basal-cell	Number	1990	0.8745545	1.677618674	0.35487113
DALYs (Dis	Bulgaria	Female	55+ years	Non-melanoma skin cancer (basal-cell	Number	1990	0.8736967	1.815682994	0.35646079
DALYs (Dis	Bulgaria	Both	55+ years	Non-melanoma skin cancer (basal-cell	Number	1990	1.7482512	3.454321597	0.718224698
DALYs (Dis	Bulgaria	Male	55+ years	Non-melanoma skin cancer (basal-cell	Percent	1990	6.69E-07	1.26E-06	2.80E-07
DALYs (Dis	Bulgaria	Female	55+ years	Non-melanoma skin cancer (basal-cell	Percent	1990	8.07E-07	1.63E-06	3.42E-07
DALYs (Dis	Bulgaria	Both	55+ years	Non-melanoma skin cancer (basal-cell	Percent	1990	7.31E-07	1.43E-06	3.16E-07
DALYs (Dis	Bulgaria	Male	55+ years	Non-melanoma skin cancer (basal-cell	Rate	1990	0.0834766	0.160129426	0.033872602
DALYs (Dis	Bulgaria	Female	55+ years	Non-melanoma skin cancer (basal-cell	Rate	1990	0.0718722	0.149362027	0.029323239
DALYs (Dis	Bulgaria	Both	55+ years	Non-melanoma skin cancer (basal-cell	Rate	1990	0.0772438	0.152623921	0.031733661
DALYs (Dis	United Sta	Male	55+ years	Non-melanoma skin cancer (basal-cell	Number	1990	120.33999	225.2976703	52.22223298
DALYs (Dis	United Sta	Female	55+ years	Non-melanoma skin cancer (basal-cell	Number	1990	118.81005	227.6163312	50.16765616
DALYs (Dis	United Sta	Both	55+ years	Non-melanoma skin cancer (basal-cell	Number	1990	239.15004	451.9544584	102.4319172
DALYs (Dis	United Sta	Male	55+ years	Non-melanoma skin cancer (basal-cell	Percent	1990	5.57E-06	1.03E-05	2.53E-06
DALYs (Dis	United Sta	Female	55+ years	Non-melanoma skin cancer (basal-cell	Percent	1990	5.45E-06	1.00E-05	2.43E-06
DALYs (Dis	United Sta	Both	55+ years	Non-melanoma skin cancer (basal-cell	Percent	1990	5.51E-06	1.00E-05	2.48E-06
DALYs (Dis	United Sta	Male	55+ years	Non-melanoma skin cancer (basal-cell	Rate	1990	0.5320318	0.996057197	0.230878246
DALYs (Dis	United Sta	Female	55+ years	Non-melanoma skin cancer (basal-cell	Rate	1990	0.3981133	0.762705574	0.168103715
DALYs (Dis	United Sta	Both	55+ years	Non-melanoma skin cancer (basal-cell	Rate	1990	0.4558519	0.861485489	0.195248899
DALYs (Dis	Germany	Male	55+ years	Non-melanoma skin cancer (basal-cell	Number	1990	5.7956883	12.10765881	2.446723115
DALYs (Dis	Germany	Female	55+ years	Non-melanoma skin cancer (basal-cell	Number	1990	7.2528044	13.79399518	3.192260719
DALYs (Dis	Germany	Both	55+ years	Non-melanoma skin cancer (basal-cell	Number	1990	13.048493	25.78695851	5.561828266
DALYs (Dis	Germany	Male	55+ years	Non-melanoma skin cancer (basal-cell	Percent	1990	7.05E-07	1.42E-06	3.04E-07
DALYs (Dis	Germany	Female	55+ years	Non-melanoma skin cancer (basal-cell	Percent	1990	7.33E-07	1.37E-06	3.37E-07
DALYs (Dis	Germany	Both	55+ years	Non-melanoma skin cancer (basal-cell	Percent	1990	7.21E-07	1.36E-06	3.10E-07
DALYs (Dis	Germany	Male	55+ years	Non-melanoma skin cancer (basal-cell	Rate	1990	0.0689643	0.144071977	0.029114153
DALYs (Dis	Germany	Female	55+ years	Non-melanoma skin cancer (basal-cell	Rate	1990	0.0576567	0.10965631	0.025377095
DALYs (Dis	Germany	Both	55+ years	Non-melanoma skin cancer (basal-cell	Rate	1990	0.0621854	0.122893385	0.026506108
DALYs (Dis	Costa Rica	Male	55+ years	Non-melanoma skin cancer (basal-cell	Number	1990	0.1395306	0.292485656	0.057432926
DALYs (Dis	Costa Rica	Female	55+ years	Non-melanoma skin cancer (basal-cell	Number	1990	0.1510896	0.298993946	0.061568906
DALYs (Dis	Costa Rica	Both	55+ years	Non-melanoma skin cancer (basal-cell	Number	1990	0.2906201	0.590176797	0.123818304
DALYs (Dis	Costa Rica	Male	55+ years	Non-melanoma skin cancer (basal-cell	Percent	1990	1.36E-06	2.74E-06	5.64E-07
DALYs (Dis	Costa Rica	Female	55+ years	Non-melanoma skin cancer (basal-cell	Percent	1990	1.67E-06	3.26E-06	7.05E-07
DALYs (Dis	Costa Rica	Both	55+ years	Non-melanoma skin cancer (basal-cell	Percent	1990	1.51E-06	2.94E-06	6.67E-07
DALYs (Dis	Costa Rica	Male	55+ years	Non-melanoma skin cancer (basal-cell	Rate	1990	0.1010228	0.211765194	0.041582534
DALYs (Dis	Costa Rica	Female	55+ years	Non-melanoma skin cancer (basal-cell	Rate	1990	0.1032896	0.204401671	0.042090442
DALYs (Dis	Costa Rica	Both	55+ years	Non-melanoma skin cancer (basal-cell	Rate	1990	0.1021887	0.207519701	0.043537356
DALYs (Dis	Gabon	Male	55+ years	Non-melanoma skin cancer (basal-cell	Number	1990	0.0055969	0.010542445	0.002346581

DALYs (Dis	Gabon	Female	55+ years	Non-melanoma skin cancer (basal-cell	Number	1990	0.0043	0.008577209	0.001807941
DALYs (Dis	Gabon	Both	55+ years	Non-melanoma skin cancer (basal-cell	Number	1990	0.0098969	0.019040544	0.0041971
DALYs (Dis	Gabon	Male	55+ years	Non-melanoma skin cancer (basal-cell	Percent	1990	7.82E-08	1.45E-07	3.32E-08
DALYs (Dis	Gabon	Female	55+ years	Non-melanoma skin cancer (basal-cell	Percent	1990	7.16E-08	1.40E-07	3.10E-08
DALYs (Dis	Gabon	Both	55+ years	Non-melanoma skin cancer (basal-cell	Percent	1990	7.52E-08	1.43E-07	3.22E-08
DALYs (Dis	Gabon	Male	55+ years	Non-melanoma skin cancer (basal-cell	Rate	1990	0.012823	0.024153432	0.005376169
DALYs (Dis	Gabon	Female	55+ years	Non-melanoma skin cancer (basal-cell	Rate	1990	0.0079257	0.015809446	0.003332384
DALYs (Dis	Gabon	Both	55+ years	Non-melanoma skin cancer (basal-cell	Rate	1990	0.0101091	0.019448672	0.004287064
DALYs (Dis	Malawi	Male	55+ years	Non-melanoma skin cancer (basal-cell	Number	1990	0.0262703	0.050828914	0.010546961
DALYs (Dis	Malawi	Female	55+ years	Non-melanoma skin cancer (basal-cell	Number	1990	0.0131806	0.025981222	0.005340737
DALYs (Dis	Malawi	Both	55+ years	Non-melanoma skin cancer (basal-cell	Number	1990	0.0394509	0.077576572	0.015910203
DALYs (Dis	Malawi	Male	55+ years	Non-melanoma skin cancer (basal-cell	Percent	1990	6.02E-08	1.13E-07	2.51E-08
DALYs (Dis	Malawi	Female	55+ years	Non-melanoma skin cancer (basal-cell	Percent	1990	3.18E-08	6.42E-08	1.29E-08
DALYs (Dis	Malawi	Both	55+ years	Non-melanoma skin cancer (basal-cell	Percent	1990	4.63E-08	8.91E-08	1.98E-08
DALYs (Dis	Malawi	Male	55+ years	Non-melanoma skin cancer (basal-cell	Rate	1990	0.0089408	0.017298972	0.003589523
DALYs (Dis	Malawi	Female	55+ years	Non-melanoma skin cancer (basal-cell	Rate	1990	0.0039327	0.007752052	0.001593523
DALYs (Dis	Malawi	Both	55+ years	Non-melanoma skin cancer (basal-cell	Rate	1990	0.0062722	0.012333729	0.002529528
DALYs (Dis	San Marino	Male	55+ years	Non-melanoma skin cancer (basal-cell	Number	1990	0.0031637	0.006716615	0.001270595
DALYs (Dis	San Marino	Female	55+ years	Non-melanoma skin cancer (basal-cell	Number	1990	0.0029383	0.00605264	0.001155296
DALYs (Dis	San Marino	Both	55+ years	Non-melanoma skin cancer (basal-cell	Number	1990	0.006102	0.012630207	0.002631276
DALYs (Dis	San Marino	Male	55+ years	Non-melanoma skin cancer (basal-cell	Percent	1990	1.54E-06	3.20E-06	6.32E-07
DALYs (Dis	San Marino	Female	55+ years	Non-melanoma skin cancer (basal-cell	Percent	1990	1.60E-06	3.17E-06	6.63E-07
DALYs (Dis	San Marino	Both	55+ years	Non-melanoma skin cancer (basal-cell	Percent	1990	1.57E-06	3.17E-06	6.83E-07
DALYs (Dis	San Marino	Male	55+ years	Non-melanoma skin cancer (basal-cell	Rate	1990	0.1166648	0.247685426	0.046855141
DALYs (Dis	San Marino	Female	55+ years	Non-melanoma skin cancer (basal-cell	Rate	1990	0.0929438	0.191454805	0.036543874
DALYs (Dis	San Marino	Both	55+ years	Non-melanoma skin cancer (basal-cell	Rate	1990	0.1038963	0.215050108	0.044801814
DALYs (Dis	Namibia	Male	55+ years	Non-melanoma skin cancer (basal-cell	Number	1990	0.0193512	0.037979494	0.008251531
DALYs (Dis	Namibia	Female	55+ years	Non-melanoma skin cancer (basal-cell	Number	1990	0.0143986	0.028226735	0.005987494
DALYs (Dis	Namibia	Both	55+ years	Non-melanoma skin cancer (basal-cell	Number	1990	0.0337498	0.065660917	0.014238139
DALYs (Dis	Namibia	Male	55+ years	Non-melanoma skin cancer (basal-cell	Percent	1990	2.73E-07	5.40E-07	1.18E-07
DALYs (Dis	Namibia	Female	55+ years	Non-melanoma skin cancer (basal-cell	Percent	1990	2.39E-07	4.70E-07	1.03E-07
DALYs (Dis	Namibia	Both	55+ years	Non-melanoma skin cancer (basal-cell	Percent	1990	2.57E-07	5.00E-07	1.12E-07
DALYs (Dis	Namibia	Male	55+ years	Non-melanoma skin cancer (basal-cell	Rate	1990	0.0396868	0.077890979	0.016922812
DALYs (Dis	Namibia	Female	55+ years	Non-melanoma skin cancer (basal-cell	Rate	1990	0.0240982	0.04724155	0.010020942
DALYs (Dis	Namibia	Both	55+ years	Non-melanoma skin cancer (basal-cell	Rate	1990	0.0311031	0.06051161	0.013121545
DALYs (Dis	Ghana	Male	55+ years	Non-melanoma skin cancer (basal-cell	Number	1990	0.0388506	0.077592135	0.016040247
DALYs (Dis	Ghana	Female	55+ years	Non-melanoma skin cancer (basal-cell	Number	1990	0.0255451	0.049733094	0.010403945
DALYs (Dis	Ghana	Both	55+ years	Non-melanoma skin cancer (basal-cell	Number	1990	0.0643957	0.128729794	0.026487214
DALYs (Dis	Ghana	Male	55+ years	Non-melanoma skin cancer (basal-cell	Percent	1990	6.57E-08	1.29E-07	2.78E-08
DALYs (Dis	Ghana	Female	55+ years	Non-melanoma skin cancer (basal-cell	Percent	1990	4.58E-08	9.03E-08	1.96E-08
DALYs (Dis	Ghana	Both	55+ years	Non-melanoma skin cancer (basal-cell	Percent	1990	5.61E-08	1.10E-07	2.36E-08
DALYs (Dis	Ghana	Male	55+ years	Non-melanoma skin cancer (basal-cell	Rate	1990	0.0078366	0.015651116	0.00323548
DALYs (Dis	Ghana	Female	55+ years	Non-melanoma skin cancer (basal-cell	Rate	1990	0.0048616	0.009465021	0.001980041
DALYs (Dis	Ghana	Both	55+ years	Non-melanoma skin cancer (basal-cell	Rate	1990	0.0063059	0.012605714	0.002593729
DALYs (Dis	Armenia	Male	55+ years	Non-melanoma skin cancer (basal-cell	Number	1990	0.1131838	0.222468264	0.049006565
DALYs (Dis	Armenia	Female	55+ years	Non-melanoma skin cancer (basal-cell	Number	1990	0.1615085	0.321175618	0.067271539
DALYs (Dis	Armenia	Both	55+ years	Non-melanoma skin cancer (basal-cell	Number	1990	0.2746923	0.554811299	0.116088608
DALYs (Dis	Armenia	Male	55+ years	Non-melanoma skin cancer (basal-cell	Percent	1990	5.41E-07	1.05E-06	2.39E-07
DALYs (Dis	Armenia	Female	55+ years	Non-melanoma skin cancer (basal-cell	Percent	1990	7.78E-07	1.50E-06	3.35E-07
DALYs (Dis	Armenia	Both	55+ years	Non-melanoma skin cancer (basal-cell	Percent	1990	6.59E-07	1.29E-06	2.87E-07
DALYs (Dis	Armenia	Male	55+ years	Non-melanoma skin cancer (basal-cell	Rate	1990	0.0536099	0.105372868	0.02321213
DALYs (Dis	Armenia	Female	55+ years	Non-melanoma skin cancer (basal-cell	Rate	1990	0.0585935	0.11651891	0.024405359
DALYs (Dis	Armenia	Both	55+ years	Non-melanoma skin cancer (basal-cell	Rate	1990	0.056432	0.113978755	0.023848893
DALYs (Dis	Mali	Male	55+ years	Non-melanoma skin cancer (basal-cell	Number	1990	0.033216	0.065163751	0.013557215
DALYs (Dis	Mali	Female	55+ years	Non-melanoma skin cancer (basal-cell	Number	1990	0.023477	0.046960227	0.009993412
DALYs (Dis	Mali	Both	55+ years	Non-melanoma skin cancer (basal-cell	Number	1990	0.0566931	0.112129788	0.023595354

DALYs (Dis	Mali	Male	55+ years	Non-melanoma skin cancer (basal-cell	Percent	1990	7.90E-08	1.55E-07	3.26E-08
DALYs (Dis	Mali	Female	55+ years	Non-melanoma skin cancer (basal-cell	Percent	1990	5.54E-08	1.11E-07	2.40E-08
DALYs (Dis	Mali	Both	55+ years	Non-melanoma skin cancer (basal-cell	Percent	1990	6.72E-08	1.32E-07	2.78E-08
DALYs (Dis	Mali	Male	55+ years	Non-melanoma skin cancer (basal-cell	Rate	1990	0.0097562	0.019139953	0.003982037
DALYs (Dis	Mali	Female	55+ years	Non-melanoma skin cancer (basal-cell	Rate	1990	0.0071533	0.014308511	0.003044935
DALYs (Dis	Mali	Both	55+ years	Non-melanoma skin cancer (basal-cell	Rate	1990	0.0084786	0.016769399	0.003528767
DALYs (Dis	Palestine	Male	55+ years	Non-melanoma skin cancer (basal-cell	Number	1990	0.0096015	0.018455205	0.003993367
DALYs (Dis	Palestine	Female	55+ years	Non-melanoma skin cancer (basal-cell	Number	1990	0.0070512	0.013354063	0.002879253
DALYs (Dis	Palestine	Both	55+ years	Non-melanoma skin cancer (basal-cell	Number	1990	0.0166527	0.031499922	0.006825885
DALYs (Dis	Palestine	Male	55+ years	Non-melanoma skin cancer (basal-cell	Percent	1990	1.28E-07	2.40E-07	5.57E-08
DALYs (Dis	Palestine	Female	55+ years	Non-melanoma skin cancer (basal-cell	Percent	1990	9.78E-08	1.87E-07	4.30E-08
DALYs (Dis	Palestine	Both	55+ years	Non-melanoma skin cancer (basal-cell	Percent	1990	1.13E-07	2.10E-07	4.92E-08
DALYs (Dis	Palestine	Male	55+ years	Non-melanoma skin cancer (basal-cell	Rate	1990	0.0147133	0.028280594	0.0061194
DALYs (Dis	Palestine	Female	55+ years	Non-melanoma skin cancer (basal-cell	Rate	1990	0.0088266	0.016716477	0.003604219
DALYs (Dis	Palestine	Both	55+ years	Non-melanoma skin cancer (basal-cell	Rate	1990	0.0114733	0.021702664	0.004702865
DALYs (Dis	Vanuatu	Male	55+ years	Non-melanoma skin cancer (basal-cell	Number	1990	5.10E-06	1.40E-05	1.01E-06
DALYs (Dis	Vanuatu	Female	55+ years	Non-melanoma skin cancer (basal-cell	Number	1990	4.57E-06	1.20E-05	1.03E-06
DALYs (Dis	Vanuatu	Both	55+ years	Non-melanoma skin cancer (basal-cell	Number	1990	9.67E-06	2.60E-05	2.15E-06
DALYs (Dis	Vanuatu	Male	55+ years	Non-melanoma skin cancer (basal-cell	Percent	1990	6.61E-10	1.82E-09	1.37E-10
DALYs (Dis	Vanuatu	Female	55+ years	Non-melanoma skin cancer (basal-cell	Percent	1990	9.78E-10	2.55E-09	2.26E-10
DALYs (Dis	Vanuatu	Both	55+ years	Non-melanoma skin cancer (basal-cell	Percent	1990	7.81E-10	2.12E-09	1.73E-10
DALYs (Dis	Vanuatu	Male	55+ years	Non-melanoma skin cancer (basal-cell	Rate	1990	9.32E-05	0.000256225	1.85E-05
DALYs (Dis	Vanuatu	Female	55+ years	Non-melanoma skin cancer (basal-cell	Rate	1990	0.0001017	0.000266858	2.29E-05
DALYs (Dis	Vanuatu	Both	55+ years	Non-melanoma skin cancer (basal-cell	Rate	1990	9.70E-05	0.000261031	2.16E-05
DALYs (Dis	Cook Islan	Male	55+ years	Non-melanoma skin cancer (basal-cell	Number	1990	1.28E-06	3.41E-06	2.86E-07
DALYs (Dis	Cook Islan	Female	55+ years	Non-melanoma skin cancer (basal-cell	Number	1990	1.24E-06	3.19E-06	2.97E-07
DALYs (Dis	Cook Islan	Both	55+ years	Non-melanoma skin cancer (basal-cell	Number	1990	2.52E-06	6.59E-06	6.43E-07
DALYs (Dis	Cook Islan	Male	55+ years	Non-melanoma skin cancer (basal-cell	Percent	1990	9.87E-10	2.60E-09	2.20E-10
DALYs (Dis	Cook Islan	Female	55+ years	Non-melanoma skin cancer (basal-cell	Percent	1990	1.39E-09	3.56E-09	3.35E-10
DALYs (Dis	Cook Islan	Both	55+ years	Non-melanoma skin cancer (basal-cell	Percent	1990	1.15E-09	2.95E-09	3.03E-10
DALYs (Dis	Cook Islan	Male	55+ years	Non-melanoma skin cancer (basal-cell	Rate	1990	0.0001122	0.000298954	2.50E-05
DALYs (Dis	Cook Islan	Female	55+ years	Non-melanoma skin cancer (basal-cell	Rate	1990	0.0001254	0.00032157	2.99E-05
DALYs (Dis	Cook Islan	Both	55+ years	Non-melanoma skin cancer (basal-cell	Rate	1990	0.0001183	0.000308976	3.01E-05
DALYs (Dis	Serbia	Male	55+ years	Non-melanoma skin cancer (basal-cell	Number	1990	0.5151461	1.020370666	0.236534792
DALYs (Dis	Serbia	Female	55+ years	Non-melanoma skin cancer (basal-cell	Number	1990	0.5066949	1.043027014	0.229301637
DALYs (Dis	Serbia	Both	55+ years	Non-melanoma skin cancer (basal-cell	Number	1990	1.021841	2.055276509	0.467348612
DALYs (Dis	Serbia	Male	55+ years	Non-melanoma skin cancer (basal-cell	Percent	1990	4.84E-07	9.18E-07	2.27E-07
DALYs (Dis	Serbia	Female	55+ years	Non-melanoma skin cancer (basal-cell	Percent	1990	5.19E-07	1.04E-06	2.38E-07
DALYs (Dis	Serbia	Both	55+ years	Non-melanoma skin cancer (basal-cell	Percent	1990	5.00E-07	9.84E-07	2.29E-07
DALYs (Dis	Serbia	Male	55+ years	Non-melanoma skin cancer (basal-cell	Rate	1990	0.0538711	0.106704591	0.02473547
DALYs (Dis	Serbia	Female	55+ years	Non-melanoma skin cancer (basal-cell	Rate	1990	0.0450995	0.092837026	0.020409521
DALYs (Dis	Serbia	Both	55+ years	Non-melanoma skin cancer (basal-cell	Rate	1990	0.0491326	0.098822737	0.022471268
DALYs (Dis	Norway	Male	55+ years	Non-melanoma skin cancer (basal-cell	Number	1990	0.54882	1.056710256	0.220172029
DALYs (Dis	Norway	Female	55+ years	Non-melanoma skin cancer (basal-cell	Number	1990	0.5657658	1.1039349	0.244113097
DALYs (Dis	Norway	Both	55+ years	Non-melanoma skin cancer (basal-cell	Number	1990	1.1145857	2.158719353	0.459486368
DALYs (Dis	Norway	Male	55+ years	Non-melanoma skin cancer (basal-cell	Percent	1990	1.15E-06	2.18E-06	4.76E-07
DALYs (Dis	Norway	Female	55+ years	Non-melanoma skin cancer (basal-cell	Percent	1990	1.27E-06	2.38E-06	5.67E-07
DALYs (Dis	Norway	Both	55+ years	Non-melanoma skin cancer (basal-cell	Percent	1990	1.21E-06	2.28E-06	5.08E-07
DALYs (Dis	Norway	Male	55+ years	Non-melanoma skin cancer (basal-cell	Rate	1990	0.1145937	0.220641194	0.045971939
DALYs (Dis	Norway	Female	55+ years	Non-melanoma skin cancer (basal-cell	Rate	1990	0.0936704	0.182771807	0.040416325
DALYs (Dis	Norway	Both	55+ years	Non-melanoma skin cancer (basal-cell	Rate	1990	0.1029238	0.199341856	0.042430187
DALYs (Dis	Sri Lanka	Male	55+ years	Non-melanoma skin cancer (basal-cell	Number	1990	0.0357593	0.070640133	0.015905976
DALYs (Dis	Sri Lanka	Female	55+ years	Non-melanoma skin cancer (basal-cell	Number	1990	0.0186949	0.036496472	0.007813614
DALYs (Dis	Sri Lanka	Both	55+ years	Non-melanoma skin cancer (basal-cell	Number	1990	0.0544542	0.105280478	0.024158941
DALYs (Dis	Sri Lanka	Male	55+ years	Non-melanoma skin cancer (basal-cell	Percent	1990	3.89E-08	7.41E-08	1.72E-08
DALYs (Dis	Sri Lanka	Female	55+ years	Non-melanoma skin cancer (basal-cell	Percent	1990	2.99E-08	5.51E-08	1.28E-08

DALYs (Dis	Sri Lanka	Both	55+ years	Non-melanoma skin cancer (basal-cell	Percent	1990	3.52E-08	6.44E-08	1.59E-08
DALYs (Dis	Sri Lanka	Male	55+ years	Non-melanoma skin cancer (basal-cell	Rate	1990	0.003987	0.007875957	0.001773422
DALYs (Dis	Sri Lanka	Female	55+ years	Non-melanoma skin cancer (basal-cell	Rate	1990	0.0021175	0.004133853	0.000885026
DALYs (Dis	Sri Lanka	Both	55+ years	Non-melanoma skin cancer (basal-cell	Rate	1990	0.0030596	0.005915375	0.001357414
DALYs (Dis	Haiti	Male	55+ years	Non-melanoma skin cancer (basal-cell	Number	1990	0.0322715	0.064841836	0.013289144
DALYs (Dis	Haiti	Female	55+ years	Non-melanoma skin cancer (basal-cell	Number	1990	0.0116342	0.022682418	0.004645789
DALYs (Dis	Haiti	Both	55+ years	Non-melanoma skin cancer (basal-cell	Number	1990	0.0439057	0.087251133	0.017975677
DALYs (Dis	Haiti	Male	55+ years	Non-melanoma skin cancer (basal-cell	Percent	1990	8.91E-08	1.77E-07	3.81E-08
DALYs (Dis	Haiti	Female	55+ years	Non-melanoma skin cancer (basal-cell	Percent	1990	3.12E-08	6.06E-08	1.30E-08
DALYs (Dis	Haiti	Both	55+ years	Non-melanoma skin cancer (basal-cell	Percent	1990	5.98E-08	1.16E-07	2.54E-08
DALYs (Dis	Haiti	Male	55+ years	Non-melanoma skin cancer (basal-cell	Rate	1990	0.0121658	0.02444429	0.005009785
DALYs (Dis	Haiti	Female	55+ years	Non-melanoma skin cancer (basal-cell	Rate	1990	0.0042446	0.008275509	0.001694981
DALYs (Dis	Haiti	Both	55+ years	Non-melanoma skin cancer (basal-cell	Rate	1990	0.0081404	0.016176951	0.003332812
DALYs (Dis	Trinidad a	Male	55+ years	Non-melanoma skin cancer (basal-cell	Number	2021	0.0208793	0.040452771	0.008643731
DALYs (Dis	Trinidad a	Female	55+ years	Non-melanoma skin cancer (basal-cell	Number	2021	0.0125242	0.024608402	0.005086783
DALYs (Dis	Trinidad a	Both	55+ years	Non-melanoma skin cancer (basal-cell	Number	2021	0.0334035	0.064546469	0.014071877
DALYs (Dis	Trinidad a	Male	55+ years	Non-melanoma skin cancer (basal-cell	Percent	2021	1.11E-07	2.22E-07	4.48E-08
DALYs (Dis	Trinidad a	Female	55+ years	Non-melanoma skin cancer (basal-cell	Percent	2021	8.57E-08	1.64E-07	3.68E-08
DALYs (Dis	Trinidad a	Both	55+ years	Non-melanoma skin cancer (basal-cell	Percent	2021	1.00E-07	1.96E-07	4.08E-08
DALYs (Dis	Trinidad a	Male	55+ years	Non-melanoma skin cancer (basal-cell	Rate	2021	0.0125135	0.024244447	0.005180424
DALYs (Dis	Trinidad a	Female	55+ years	Non-melanoma skin cancer (basal-cell	Rate	2021	0.0069009	0.013559466	0.002802866
DALYs (Dis	Trinidad a	Both	55+ years	Non-melanoma skin cancer (basal-cell	Rate	2021	0.0095894	0.018529796	0.00403971
DALYs (Dis	Nicaragua	Male	55+ years	Non-melanoma skin cancer (basal-cell	Number	2021	0.2456292	0.505447437	0.102337936
DALYs (Dis	Nicaragua	Female	55+ years	Non-melanoma skin cancer (basal-cell	Number	2021	0.2877376	0.541210902	0.122421731
DALYs (Dis	Nicaragua	Both	55+ years	Non-melanoma skin cancer (basal-cell	Number	2021	0.5333669	1.051322784	0.226575472
DALYs (Dis	Nicaragua	Male	55+ years	Non-melanoma skin cancer (basal-cell	Percent	2021	6.45E-07	1.26E-06	2.82E-07
DALYs (Dis	Nicaragua	Female	55+ years	Non-melanoma skin cancer (basal-cell	Percent	2021	8.70E-07	1.58E-06	3.93E-07
DALYs (Dis	Nicaragua	Both	55+ years	Non-melanoma skin cancer (basal-cell	Percent	2021	7.49E-07	1.43E-06	3.40E-07
DALYs (Dis	Nicaragua	Male	55+ years	Non-melanoma skin cancer (basal-cell	Rate	2021	0.0671663	0.138212455	0.027983874
DALYs (Dis	Nicaragua	Female	55+ years	Non-melanoma skin cancer (basal-cell	Rate	2021	0.0641798	0.120717007	0.027306148
DALYs (Dis	Nicaragua	Both	55+ years	Non-melanoma skin cancer (basal-cell	Rate	2021	0.0655215	0.129149813	0.027833678
DALYs (Dis	Türkiye	Male	55+ years	Non-melanoma skin cancer (basal-cell	Number	2021	2.2101382	4.232907636	0.939047478
DALYs (Dis	Türkiye	Female	55+ years	Non-melanoma skin cancer (basal-cell	Number	2021	1.966581	3.65942012	0.806981563
DALYs (Dis	Türkiye	Both	55+ years	Non-melanoma skin cancer (basal-cell	Number	2021	4.1767192	7.851563128	1.744404046
DALYs (Dis	Türkiye	Male	55+ years	Non-melanoma skin cancer (basal-cell	Percent	2021	2.98E-07	5.46E-07	1.29E-07
DALYs (Dis	Türkiye	Female	55+ years	Non-melanoma skin cancer (basal-cell	Percent	2021	3.20E-07	5.72E-07	1.43E-07
DALYs (Dis	Türkiye	Both	55+ years	Non-melanoma skin cancer (basal-cell	Percent	2021	3.08E-07	5.50E-07	1.33E-07
DALYs (Dis	Türkiye	Male	55+ years	Non-melanoma skin cancer (basal-cell	Rate	2021	0.0283522	0.054300702	0.012046315
DALYs (Dis	Türkiye	Female	55+ years	Non-melanoma skin cancer (basal-cell	Rate	2021	0.0225372	0.041937232	0.00924807
DALYs (Dis	Türkiye	Both	55+ years	Non-melanoma skin cancer (basal-cell	Rate	2021	0.0252809	0.047524011	0.010558544
DALYs (Dis	Cyprus	Male	55+ years	Non-melanoma skin cancer (basal-cell	Number	1990	0.0488646	0.100544716	0.019673335
DALYs (Dis	Cyprus	Female	55+ years	Non-melanoma skin cancer (basal-cell	Number	1990	0.0470103	0.092375526	0.020961642
DALYs (Dis	Cyprus	Both	55+ years	Non-melanoma skin cancer (basal-cell	Number	1990	0.0958749	0.196364949	0.040537153
DALYs (Dis	Cyprus	Male	55+ years	Non-melanoma skin cancer (basal-cell	Percent	1990	7.93E-07	1.59E-06	3.27E-07
DALYs (Dis	Cyprus	Female	55+ years	Non-melanoma skin cancer (basal-cell	Percent	1990	7.56E-07	1.44E-06	3.49E-07
DALYs (Dis	Cyprus	Both	55+ years	Non-melanoma skin cancer (basal-cell	Percent	1990	7.74E-07	1.55E-06	3.41E-07
DALYs (Dis	Cyprus	Male	55+ years	Non-melanoma skin cancer (basal-cell	Rate	1990	0.0756245	0.155606363	0.030447111
DALYs (Dis	Cyprus	Female	55+ years	Non-melanoma skin cancer (basal-cell	Rate	1990	0.0626177	0.123044325	0.027920936
DALYs (Dis	Cyprus	Both	55+ years	Non-melanoma skin cancer (basal-cell	Rate	1990	0.0686341	0.140572161	0.029019411
DALYs (Dis	Cyprus	Male	55+ years	Non-melanoma skin cancer (basal-cell	Number	2021	0.1344968	0.271281636	0.052193039
DALYs (Dis	Cyprus	Female	55+ years	Non-melanoma skin cancer (basal-cell	Number	2021	0.1134947	0.220472002	0.045946239
DALYs (Dis	Cyprus	Both	55+ years	Non-melanoma skin cancer (basal-cell	Number	2021	0.2479915	0.481334974	0.10213881
DALYs (Dis	Cyprus	Male	55+ years	Non-melanoma skin cancer (basal-cell	Percent	2021	1.23E-06	2.44E-06	5.07E-07
DALYs (Dis	Cyprus	Female	55+ years	Non-melanoma skin cancer (basal-cell	Percent	2021	1.16E-06	2.20E-06	4.91E-07
DALYs (Dis	Cyprus	Both	55+ years	Non-melanoma skin cancer (basal-cell	Percent	2021	1.20E-06	2.28E-06	5.22E-07
DALYs (Dis	Cyprus	Male	55+ years	Non-melanoma skin cancer (basal-cell	Rate	2021	0.0798799	0.161118701	0.030998319

DALYs (Dis	Cyprus	Female	55+ years	Non-melanoma skin cancer (basal-cell	Rate	2021	0.06266	0.121721735	0.02536674
DALYs (Dis	Cyprus	Both	55+ years	Non-melanoma skin cancer (basal-cell	Rate	2021	0.0709558	0.13772037	0.029224128
DALYs (Dis	Egypt	Male	55+ years	Non-melanoma skin cancer (basal-cell	Number	1990	0.3235235	0.628242043	0.14346608
DALYs (Dis	Egypt	Female	55+ years	Non-melanoma skin cancer (basal-cell	Number	1990	0.1459258	0.278850421	0.064117549
DALYs (Dis	Egypt	Both	55+ years	Non-melanoma skin cancer (basal-cell	Number	1990	0.4694492	0.905513137	0.212397263
DALYs (Dis	Egypt	Male	55+ years	Non-melanoma skin cancer (basal-cell	Percent	1990	1.17E-07	2.21E-07	5.41E-08
DALYs (Dis	Egypt	Female	55+ years	Non-melanoma skin cancer (basal-cell	Percent	1990	5.77E-08	1.08E-07	2.62E-08
DALYs (Dis	Egypt	Both	55+ years	Non-melanoma skin cancer (basal-cell	Percent	1990	8.89E-08	1.65E-07	4.11E-08
DALYs (Dis	Egypt	Male	55+ years	Non-melanoma skin cancer (basal-cell	Rate	1990	0.0142	0.027574626	0.006296973
DALYs (Dis	Egypt	Female	55+ years	Non-melanoma skin cancer (basal-cell	Rate	1990	0.0066539	0.012715071	0.002923643
DALYs (Dis	Egypt	Both	55+ years	Non-melanoma skin cancer (basal-cell	Rate	1990	0.0104989	0.020251204	0.004750125
DALYs (Dis	Zimbabwe	Male	55+ years	Non-melanoma skin cancer (basal-cell	Number	1990	0.1002469	0.195428119	0.043206347
DALYs (Dis	Zimbabwe	Female	55+ years	Non-melanoma skin cancer (basal-cell	Number	1990	0.0692397	0.136921818	0.029255394
DALYs (Dis	Zimbabwe	Both	55+ years	Non-melanoma skin cancer (basal-cell	Number	1990	0.1694866	0.33043693	0.072521658
DALYs (Dis	Zimbabwe	Male	55+ years	Non-melanoma skin cancer (basal-cell	Percent	1990	2.46E-07	4.81E-07	1.09E-07
DALYs (Dis	Zimbabwe	Female	55+ years	Non-melanoma skin cancer (basal-cell	Percent	1990	2.22E-07	4.38E-07	9.40E-08
DALYs (Dis	Zimbabwe	Both	55+ years	Non-melanoma skin cancer (basal-cell	Percent	1990	2.36E-07	4.60E-07	1.02E-07
DALYs (Dis	Zimbabwe	Male	55+ years	Non-melanoma skin cancer (basal-cell	Rate	1990	0.0303123	0.059092805	0.013064569
DALYs (Dis	Zimbabwe	Female	55+ years	Non-melanoma skin cancer (basal-cell	Rate	1990	0.0208481	0.041227143	0.008808796
DALYs (Dis	Zimbabwe	Both	55+ years	Non-melanoma skin cancer (basal-cell	Rate	1990	0.0255702	0.04985247	0.010941222
DALYs (Dis	Bhutan	Male	55+ years	Non-melanoma skin cancer (basal-cell	Number	1990	2.25E-05	6.36E-05	4.58E-06
DALYs (Dis	Bhutan	Female	55+ years	Non-melanoma skin cancer (basal-cell	Number	1990	1.51E-05	3.83E-05	3.51E-06
DALYs (Dis	Bhutan	Both	55+ years	Non-melanoma skin cancer (basal-cell	Number	1990	3.76E-05	9.22E-05	9.75E-06
DALYs (Dis	Bhutan	Male	55+ years	Non-melanoma skin cancer (basal-cell	Percent	1990	1.13E-09	3.33E-09	2.31E-10
DALYs (Dis	Bhutan	Female	55+ years	Non-melanoma skin cancer (basal-cell	Percent	1990	7.22E-10	1.92E-09	1.58E-10
DALYs (Dis	Bhutan	Both	55+ years	Non-melanoma skin cancer (basal-cell	Percent	1990	9.20E-10	2.33E-09	2.32E-10
DALYs (Dis	Bhutan	Male	55+ years	Non-melanoma skin cancer (basal-cell	Rate	1990	0.0001123	0.000317183	2.28E-05
DALYs (Dis	Bhutan	Female	55+ years	Non-melanoma skin cancer (basal-cell	Rate	1990	7.68E-05	0.000194921	1.79E-05
DALYs (Dis	Bhutan	Both	55+ years	Non-melanoma skin cancer (basal-cell	Rate	1990	9.47E-05	0.000232362	2.46E-05
DALYs (Dis	Uzbekistan	Male	55+ years	Non-melanoma skin cancer (basal-cell	Number	1990	0.4164844	0.794699029	0.176812863
DALYs (Dis	Uzbekistan	Female	55+ years	Non-melanoma skin cancer (basal-cell	Number	1990	0.6066072	1.197035993	0.248622405
DALYs (Dis	Uzbekistan	Both	55+ years	Non-melanoma skin cancer (basal-cell	Number	1990	1.0230916	1.997238106	0.432804356
DALYs (Dis	Uzbekistan	Male	55+ years	Non-melanoma skin cancer (basal-cell	Percent	1990	5.16E-07	9.82E-07	2.22E-07
DALYs (Dis	Uzbekistan	Female	55+ years	Non-melanoma skin cancer (basal-cell	Percent	1990	6.85E-07	1.34E-06	2.97E-07
DALYs (Dis	Uzbekistan	Both	55+ years	Non-melanoma skin cancer (basal-cell	Percent	1990	6.04E-07	1.16E-06	2.66E-07
DALYs (Dis	Uzbekistan	Male	55+ years	Non-melanoma skin cancer (basal-cell	Rate	1990	0.0510899	0.097485234	0.021689524
DALYs (Dis	Uzbekistan	Female	55+ years	Non-melanoma skin cancer (basal-cell	Rate	1990	0.0531884	0.104958228	0.021799651
DALYs (Dis	Uzbekistan	Both	55+ years	Non-melanoma skin cancer (basal-cell	Rate	1990	0.0523137	0.10212461	0.022130549
DALYs (Dis	Iraq	Male	55+ years	Non-melanoma skin cancer (basal-cell	Number	2021	0.2293803	0.447848654	0.10294476
DALYs (Dis	Iraq	Female	55+ years	Non-melanoma skin cancer (basal-cell	Number	2021	0.1704726	0.325995768	0.076003364
DALYs (Dis	Iraq	Both	55+ years	Non-melanoma skin cancer (basal-cell	Number	2021	0.399853	0.774315787	0.178633838
DALYs (Dis	Iraq	Male	55+ years	Non-melanoma skin cancer (basal-cell	Percent	2021	9.77E-08	1.92E-07	4.39E-08
DALYs (Dis	Iraq	Female	55+ years	Non-melanoma skin cancer (basal-cell	Percent	2021	9.82E-08	1.90E-07	4.31E-08
DALYs (Dis	Iraq	Both	55+ years	Non-melanoma skin cancer (basal-cell	Percent	2021	9.79E-08	1.92E-07	4.31E-08
DALYs (Dis	Iraq	Male	55+ years	Non-melanoma skin cancer (basal-cell	Rate	2021	0.0120667	0.023559461	0.005415497
DALYs (Dis	Iraq	Female	55+ years	Non-melanoma skin cancer (basal-cell	Rate	2021	0.0085912	0.016429062	0.003830307
DALYs (Dis	Iraq	Both	55+ years	Non-melanoma skin cancer (basal-cell	Rate	2021	0.0102917	0.019929924	0.004597812
DALYs (Dis	Tuvalu	Male	55+ years	Non-melanoma skin cancer (basal-cell	Number	1990	5.01E-07	1.35E-06	1.01E-07
DALYs (Dis	Tuvalu	Female	55+ years	Non-melanoma skin cancer (basal-cell	Number	1990	6.47E-07	1.69E-06	1.38E-07
DALYs (Dis	Tuvalu	Both	55+ years	Non-melanoma skin cancer (basal-cell	Number	1990	1.15E-06	3.07E-06	2.58E-07
DALYs (Dis	Tuvalu	Male	55+ years	Non-melanoma skin cancer (basal-cell	Percent	1990	6.46E-10	1.73E-09	1.33E-10
DALYs (Dis	Tuvalu	Female	55+ years	Non-melanoma skin cancer (basal-cell	Percent	1990	7.94E-10	2.09E-09	1.65E-10
DALYs (Dis	Tuvalu	Both	55+ years	Non-melanoma skin cancer (basal-cell	Percent	1990	7.21E-10	1.90E-09	1.65E-10
DALYs (Dis	Tuvalu	Male	55+ years	Non-melanoma skin cancer (basal-cell	Rate	1990	9.68E-05	0.000260452	1.95E-05
DALYs (Dis	Tuvalu	Female	55+ years	Non-melanoma skin cancer (basal-cell	Rate	1990	0.0001004	0.0002621	2.14E-05
DALYs (Dis	Tuvalu	Both	55+ years	Non-melanoma skin cancer (basal-cell	Rate	1990	9.88E-05	0.000263966	2.22E-05

DALYs (Dis	Liberia	Male	55+ years	Non-melanoma skin cancer (basal-cell	Number	1990	0.0090057	0.017678932	0.003679091
DALYs (Dis	Liberia	Female	55+ years	Non-melanoma skin cancer (basal-cell	Number	1990	0.0041435	0.008093339	0.001671866
DALYs (Dis	Liberia	Both	55+ years	Non-melanoma skin cancer (basal-cell	Number	1990	0.0131492	0.026093648	0.005304593
DALYs (Dis	Liberia	Male	55+ years	Non-melanoma skin cancer (basal-cell	Percent	1990	7.05E-08	1.36E-07	2.96E-08
DALYs (Dis	Liberia	Female	55+ years	Non-melanoma skin cancer (basal-cell	Percent	1990	3.92E-08	7.48E-08	1.63E-08
DALYs (Dis	Liberia	Both	55+ years	Non-melanoma skin cancer (basal-cell	Percent	1990	5.63E-08	1.09E-07	2.34E-08
DALYs (Dis	Liberia	Male	55+ years	Non-melanoma skin cancer (basal-cell	Rate	1990	0.0087357	0.017149016	0.003568812
DALYs (Dis	Liberia	Female	55+ years	Non-melanoma skin cancer (basal-cell	Rate	1990	0.0048548	0.009482606	0.001958851
DALYs (Dis	Liberia	Both	55+ years	Non-melanoma skin cancer (basal-cell	Rate	1990	0.0069779	0.013847237	0.002815013
DALYs (Dis	Singapore	Male	55+ years	Non-melanoma skin cancer (basal-cell	Number	1990	0.0427514	0.083039343	0.019408876
DALYs (Dis	Singapore	Female	55+ years	Non-melanoma skin cancer (basal-cell	Number	1990	0.0534774	0.101780064	0.023828264
DALYs (Dis	Singapore	Both	55+ years	Non-melanoma skin cancer (basal-cell	Number	1990	0.0962288	0.186199922	0.043617125
DALYs (Dis	Singapore	Male	55+ years	Non-melanoma skin cancer (basal-cell	Percent	1990	2.82E-07	5.30E-07	1.30E-07
DALYs (Dis	Singapore	Female	55+ years	Non-melanoma skin cancer (basal-cell	Percent	1990	4.12E-07	7.54E-07	1.92E-07
DALYs (Dis	Singapore	Both	55+ years	Non-melanoma skin cancer (basal-cell	Percent	1990	3.42E-07	6.37E-07	1.58E-07
DALYs (Dis	Singapore	Male	55+ years	Non-melanoma skin cancer (basal-cell	Rate	1990	0.0254627	0.049458196	0.011559918
DALYs (Dis	Singapore	Female	55+ years	Non-melanoma skin cancer (basal-cell	Rate	1990	0.0274707	0.052283168	0.012240286
DALYs (Dis	Singapore	Both	55+ years	Non-melanoma skin cancer (basal-cell	Rate	1990	0.0265408	0.051355742	0.012030025
DALYs (Dis	Taiwan (Pr	Male	55+ years	Non-melanoma skin cancer (basal-cell	Number	1990	0.1204465	0.237145033	0.055781528
DALYs (Dis	Taiwan (Pr	Female	55+ years	Non-melanoma skin cancer (basal-cell	Number	1990	0.077793	0.153653371	0.033713438
DALYs (Dis	Taiwan (Pr	Both	55+ years	Non-melanoma skin cancer (basal-cell	Number	1990	0.1982394	0.391368376	0.089146507
DALYs (Dis	Taiwan (Pr	Male	55+ years	Non-melanoma skin cancer (basal-cell	Percent	1990	9.84E-08	1.88E-07	4.56E-08
DALYs (Dis	Taiwan (Pr	Female	55+ years	Non-melanoma skin cancer (basal-cell	Percent	1990	9.16E-08	1.74E-07	4.10E-08
DALYs (Dis	Taiwan (Pr	Both	55+ years	Non-melanoma skin cancer (basal-cell	Percent	1990	9.56E-08	1.83E-07	4.40E-08
DALYs (Dis	Taiwan (Pr	Male	55+ years	Non-melanoma skin cancer (basal-cell	Rate	1990	0.0079673	0.015686739	0.003689853
DALYs (Dis	Taiwan (Pr	Female	55+ years	Non-melanoma skin cancer (basal-cell	Rate	1990	0.0061649	0.012176656	0.002671708
DALYs (Dis	Taiwan (Pr	Both	55+ years	Non-melanoma skin cancer (basal-cell	Rate	1990	0.0071473	0.014110366	0.003214081
DALYs (Dis	Netherland	Male	55+ years	Non-melanoma skin cancer (basal-cell	Number	1990	0.8581548	1.733675486	0.342454779
DALYs (Dis	Netherland	Female	55+ years	Non-melanoma skin cancer (basal-cell	Number	1990	1.1057084	2.145449186	0.482275241
DALYs (Dis	Netherland	Both	55+ years	Non-melanoma skin cancer (basal-cell	Number	1990	1.9638632	3.847624897	0.804149945
DALYs (Dis	Netherland	Male	55+ years	Non-melanoma skin cancer (basal-cell	Percent	1990	6.42E-07	1.27E-06	2.66E-07
DALYs (Dis	Netherland	Female	55+ years	Non-melanoma skin cancer (basal-cell	Percent	1990	8.71E-07	1.66E-06	3.82E-07
DALYs (Dis	Netherland	Both	55+ years	Non-melanoma skin cancer (basal-cell	Percent	1990	7.54E-07	1.43E-06	3.19E-07
DALYs (Dis	Netherland	Male	55+ years	Non-melanoma skin cancer (basal-cell	Rate	1990	0.0595324	0.120269482	0.02375696
DALYs (Dis	Netherland	Female	55+ years	Non-melanoma skin cancer (basal-cell	Rate	1990	0.0591454	0.114762056	0.025797347
DALYs (Dis	Netherland	Both	55+ years	Non-melanoma skin cancer (basal-cell	Rate	1990	0.0593139	0.116208441	0.024287454
DALYs (Dis	Bosnia and	Male	55+ years	Non-melanoma skin cancer (basal-cell	Number	1990	0.2040836	0.404727897	0.084537962
DALYs (Dis	Bosnia and	Female	55+ years	Non-melanoma skin cancer (basal-cell	Number	1990	0.2428586	0.468512744	0.103851874
DALYs (Dis	Bosnia and	Both	55+ years	Non-melanoma skin cancer (basal-cell	Number	1990	0.4469422	0.879401378	0.186436493
DALYs (Dis	Bosnia and	Male	55+ years	Non-melanoma skin cancer (basal-cell	Percent	1990	6.62E-07	1.28E-06	2.79E-07
DALYs (Dis	Bosnia and	Female	55+ years	Non-melanoma skin cancer (basal-cell	Percent	1990	8.08E-07	1.53E-06	3.57E-07
DALYs (Dis	Bosnia and	Both	55+ years	Non-melanoma skin cancer (basal-cell	Percent	1990	7.34E-07	1.42E-06	3.17E-07
DALYs (Dis	Bosnia and	Male	55+ years	Non-melanoma skin cancer (basal-cell	Rate	1990	0.0633024	0.125537938	0.026221868
DALYs (Dis	Bosnia and	Female	55+ years	Non-melanoma skin cancer (basal-cell	Rate	1990	0.0590685	0.113952457	0.025259027
DALYs (Dis	Bosnia and	Both	55+ years	Non-melanoma skin cancer (basal-cell	Rate	1990	0.0609293	0.119884184	0.025415911
DALYs (Dis	Viet Nam	Male	55+ years	Non-melanoma skin cancer (basal-cell	Number	1990	0.1802393	0.357746332	0.078366257
DALYs (Dis	Viet Nam	Female	55+ years	Non-melanoma skin cancer (basal-cell	Number	1990	0.291148	0.588179848	0.13071693
DALYs (Dis	Viet Nam	Both	55+ years	Non-melanoma skin cancer (basal-cell	Number	1990	0.4713874	0.945317024	0.208033645
DALYs (Dis	Viet Nam	Male	55+ years	Non-melanoma skin cancer (basal-cell	Percent	1990	5.54E-08	1.06E-07	2.49E-08
DALYs (Dis	Viet Nam	Female	55+ years	Non-melanoma skin cancer (basal-cell	Percent	1990	9.34E-08	1.80E-07	4.17E-08
DALYs (Dis	Viet Nam	Both	55+ years	Non-melanoma skin cancer (basal-cell	Percent	1990	7.40E-08	1.41E-07	3.29E-08
DALYs (Dis	Viet Nam	Male	55+ years	Non-melanoma skin cancer (basal-cell	Rate	1990	0.006073	0.012053987	0.002640491
DALYs (Dis	Viet Nam	Female	55+ years	Non-melanoma skin cancer (basal-cell	Rate	1990	0.007271	0.014688985	0.003264476
DALYs (Dis	Viet Nam	Both	55+ years	Non-melanoma skin cancer (basal-cell	Rate	1990	0.0067611	0.013558586	0.002983806
DALYs (Dis	Antigua an	Male	55+ years	Non-melanoma skin cancer (basal-cell	Number	1990	0.0006093	0.001210416	0.000246201
DALYs (Dis	Antigua an	Female	55+ years	Non-melanoma skin cancer (basal-cell	Number	1990	0.0003327	0.000649616	0.000129776

DALYs (Dis	Antigua an	Both	55+ years	Non-melanoma skin cancer (basal-cell	Number	1990	0.0009421	0.001849639	0.000370082
DALYs (Dis	Antigua an	Male	55+ years	Non-melanoma skin cancer (basal-cell	Percent	1990	1.47E-07	2.83E-07	6.16E-08
DALYs (Dis	Antigua an	Female	55+ years	Non-melanoma skin cancer (basal-cell	Percent	1990	8.92E-08	1.69E-07	3.68E-08
DALYs (Dis	Antigua an	Both	55+ years	Non-melanoma skin cancer (basal-cell	Percent	1990	1.20E-07	2.30E-07	4.94E-08
DALYs (Dis	Antigua an	Male	55+ years	Non-melanoma skin cancer (basal-cell	Rate	1990	0.016125	0.032031805	0.006515336
DALYs (Dis	Antigua an	Female	55+ years	Non-melanoma skin cancer (basal-cell	Rate	1990	0.0068435	0.013361217	0.002669225
DALYs (Dis	Antigua an	Both	55+ years	Non-melanoma skin cancer (basal-cell	Rate	1990	0.0109026	0.021406003	0.004282987
DALYs (Dis	Thailand	Male	55+ years	Non-melanoma skin cancer (basal-cell	Number	2021	0.6731042	1.250562047	0.27443536
DALYs (Dis	Thailand	Female	55+ years	Non-melanoma skin cancer (basal-cell	Number	2021	0.7614213	1.494736827	0.306541261
DALYs (Dis	Thailand	Both	55+ years	Non-melanoma skin cancer (basal-cell	Number	2021	1.4345255	2.739921655	0.588789251
DALYs (Dis	Thailand	Male	55+ years	Non-melanoma skin cancer (basal-cell	Percent	2021	9.84E-08	1.88E-07	3.99E-08
DALYs (Dis	Thailand	Female	55+ years	Non-melanoma skin cancer (basal-cell	Percent	2021	1.23E-07	2.36E-07	5.09E-08
DALYs (Dis	Thailand	Both	55+ years	Non-melanoma skin cancer (basal-cell	Percent	2021	1.10E-07	2.09E-07	4.73E-08
DALYs (Dis	Thailand	Male	55+ years	Non-melanoma skin cancer (basal-cell	Rate	2021	0.0075644	0.014053922	0.003084128
DALYs (Dis	Thailand	Female	55+ years	Non-melanoma skin cancer (basal-cell	Rate	2021	0.0072243	0.014182004	0.002908451
DALYs (Dis	Thailand	Both	55+ years	Non-melanoma skin cancer (basal-cell	Rate	2021	0.00738	0.014095707	0.003029065
DALYs (Dis	Ethiopia	Male	55+ years	Non-melanoma skin cancer (basal-cell	Number	2021	0.3696902	0.718994575	0.147807141
DALYs (Dis	Ethiopia	Female	55+ years	Non-melanoma skin cancer (basal-cell	Number	2021	0.2172233	0.423978478	0.089500911
DALYs (Dis	Ethiopia	Both	55+ years	Non-melanoma skin cancer (basal-cell	Number	2021	0.5869134	1.146919945	0.237484209
DALYs (Dis	Ethiopia	Male	55+ years	Non-melanoma skin cancer (basal-cell	Percent	2021	7.60E-08	1.49E-07	2.99E-08
DALYs (Dis	Ethiopia	Female	55+ years	Non-melanoma skin cancer (basal-cell	Percent	2021	6.14E-08	1.15E-07	2.58E-08
DALYs (Dis	Ethiopia	Both	55+ years	Non-melanoma skin cancer (basal-cell	Percent	2021	6.98E-08	1.33E-07	2.82E-08
DALYs (Dis	Ethiopia	Male	55+ years	Non-melanoma skin cancer (basal-cell	Rate	2021	0.0106174	0.020649344	0.004244984
DALYs (Dis	Ethiopia	Female	55+ years	Non-melanoma skin cancer (basal-cell	Rate	2021	0.006458	0.012604705	0.002660825
DALYs (Dis	Ethiopia	Both	55+ years	Non-melanoma skin cancer (basal-cell	Rate	2021	0.0085736	0.016754175	0.003469163
DALYs (Dis	Sweden	Male	55+ years	Non-melanoma skin cancer (basal-cell	Number	1990	1.2698159	2.602211903	0.485328295
DALYs (Dis	Sweden	Female	55+ years	Non-melanoma skin cancer (basal-cell	Number	1990	1.2602696	2.629102877	0.533873426
DALYs (Dis	Sweden	Both	55+ years	Non-melanoma skin cancer (basal-cell	Number	1990	2.5300856	5.063946787	1.049184241
DALYs (Dis	Sweden	Male	55+ years	Non-melanoma skin cancer (basal-cell	Percent	1990	1.32E-06	2.64E-06	5.19E-07
DALYs (Dis	Sweden	Female	55+ years	Non-melanoma skin cancer (basal-cell	Percent	1990	1.37E-06	2.70E-06	6.03E-07
DALYs (Dis	Sweden	Both	55+ years	Non-melanoma skin cancer (basal-cell	Percent	1990	1.34E-06	2.69E-06	5.79E-07
DALYs (Dis	Sweden	Male	55+ years	Non-melanoma skin cancer (basal-cell	Rate	1990	0.1196942	0.245287335	0.045747575
DALYs (Dis	Sweden	Female	55+ years	Non-melanoma skin cancer (basal-cell	Rate	1990	0.09597	0.200207212	0.04065467
DALYs (Dis	Sweden	Both	55+ years	Non-melanoma skin cancer (basal-cell	Rate	1990	0.1065715	0.213301977	0.044193409
DALYs (Dis	Albania	Male	55+ years	Non-melanoma skin cancer (basal-cell	Number	2021	0.2981369	0.593938777	0.117373937
DALYs (Dis	Albania	Female	55+ years	Non-melanoma skin cancer (basal-cell	Number	2021	0.2838834	0.569191282	0.116679345
DALYs (Dis	Albania	Both	55+ years	Non-melanoma skin cancer (basal-cell	Number	2021	0.5820203	1.138636262	0.252149112
DALYs (Dis	Albania	Male	55+ years	Non-melanoma skin cancer (basal-cell	Percent	2021	8.73E-07	1.72E-06	3.54E-07
DALYs (Dis	Albania	Female	55+ years	Non-melanoma skin cancer (basal-cell	Percent	2021	1.01E-06	2.02E-06	4.27E-07
DALYs (Dis	Albania	Both	55+ years	Non-melanoma skin cancer (basal-cell	Percent	2021	9.36E-07	1.79E-06	4.00E-07
DALYs (Dis	Albania	Male	55+ years	Non-melanoma skin cancer (basal-cell	Rate	2021	0.0787568	0.156896699	0.031005861
DALYs (Dis	Albania	Female	55+ years	Non-melanoma skin cancer (basal-cell	Rate	2021	0.0696236	0.139596619	0.028616113
DALYs (Dis	Albania	Both	55+ years	Non-melanoma skin cancer (basal-cell	Rate	2021	0.0740207	0.144810489	0.032068043
DALYs (Dis	Republic o	Male	55+ years	Non-melanoma skin cancer (basal-cell	Number	1990	0.1831928	0.370715744	0.083891293
DALYs (Dis	Republic o	Female	55+ years	Non-melanoma skin cancer (basal-cell	Number	1990	0.2601327	0.501468932	0.114425216
DALYs (Dis	Republic o	Both	55+ years	Non-melanoma skin cancer (basal-cell	Number	1990	0.4433255	0.888112535	0.197858938
DALYs (Dis	Republic o	Male	55+ years	Non-melanoma skin cancer (basal-cell	Percent	1990	8.21E-08	1.59E-07	3.82E-08
DALYs (Dis	Republic o	Female	55+ years	Non-melanoma skin cancer (basal-cell	Percent	1990	1.28E-07	2.43E-07	5.71E-08
DALYs (Dis	Republic o	Both	55+ years	Non-melanoma skin cancer (basal-cell	Percent	1990	1.04E-07	2.03E-07	4.76E-08
DALYs (Dis	Republic o	Male	55+ years	Non-melanoma skin cancer (basal-cell	Rate	1990	0.0088023	0.017812753	0.004030945
DALYs (Dis	Republic o	Female	55+ years	Non-melanoma skin cancer (basal-cell	Rate	1990	0.0089838	0.017318492	0.003951735
DALYs (Dis	Republic o	Both	55+ years	Non-melanoma skin cancer (basal-cell	Rate	1990	0.0089079	0.017845228	0.003975665
DALYs (Dis	Slovenia	Male	55+ years	Non-melanoma skin cancer (basal-cell	Number	2021	0.3951345	0.869631815	0.144314131
DALYs (Dis	Slovenia	Female	55+ years	Non-melanoma skin cancer (basal-cell	Number	2021	0.4068953	0.792326924	0.166434938
DALYs (Dis	Slovenia	Both	55+ years	Non-melanoma skin cancer (basal-cell	Number	2021	0.8020298	1.622414503	0.322334799
DALYs (Dis	Slovenia	Male	55+ years	Non-melanoma skin cancer (basal-cell	Percent	2021	1.52E-06	3.21E-06	5.80E-07

DALYs (Dis	Slovenia	Female	55+ years	Non-melanoma skin cancer (basal-cell	Percent	2021	1.66E-06	3.14E-06	7.14E-07
DALYs (Dis	Slovenia	Both	55+ years	Non-melanoma skin cancer (basal-cell	Percent	2021	1.58E-06	3.04E-06	6.57E-07
DALYs (Dis	Slovenia	Male	55+ years	Non-melanoma skin cancer (basal-cell	Rate	2021	0.1179039	0.259488727	0.043061776
DALYs (Dis	Slovenia	Female	55+ years	Non-melanoma skin cancer (basal-cell	Rate	2021	0.1024781	0.19955042	0.041917245
DALYs (Dis	Slovenia	Both	55+ years	Non-melanoma skin cancer (basal-cell	Rate	2021	0.1095387	0.221584175	0.044023454
DALYs (Dis	Bahrain	Male	55+ years	Non-melanoma skin cancer (basal-cell	Number	1990	0.0017419	0.003500473	0.000709411
DALYs (Dis	Bahrain	Female	55+ years	Non-melanoma skin cancer (basal-cell	Number	1990	0.0009245	0.001848378	0.000343704
DALYs (Dis	Bahrain	Both	55+ years	Non-melanoma skin cancer (basal-cell	Number	1990	0.0026664	0.005325029	0.001071146
DALYs (Dis	Bahrain	Male	55+ years	Non-melanoma skin cancer (basal-cell	Percent	1990	9.98E-08	1.97E-07	4.12E-08
DALYs (Dis	Bahrain	Female	55+ years	Non-melanoma skin cancer (basal-cell	Percent	1990	7.01E-08	1.38E-07	2.85E-08
DALYs (Dis	Bahrain	Both	55+ years	Non-melanoma skin cancer (basal-cell	Percent	1990	8.70E-08	1.71E-07	3.43E-08
DALYs (Dis	Bahrain	Male	55+ years	Non-melanoma skin cancer (basal-cell	Rate	1990	0.0113864	0.022882036	0.004637308
DALYs (Dis	Bahrain	Female	55+ years	Non-melanoma skin cancer (basal-cell	Rate	1990	0.0072461	0.014486558	0.002693759
DALYs (Dis	Bahrain	Both	55+ years	Non-melanoma skin cancer (basal-cell	Rate	1990	0.0095035	0.018979211	0.003817726
DALYs (Dis	Oman	Male	55+ years	Non-melanoma skin cancer (basal-cell	Number	1990	0.0076407	0.015098363	0.003421685
DALYs (Dis	Oman	Female	55+ years	Non-melanoma skin cancer (basal-cell	Number	1990	0.0047509	0.009187165	0.001954495
DALYs (Dis	Oman	Both	55+ years	Non-melanoma skin cancer (basal-cell	Number	1990	0.0123916	0.02464855	0.005453969
DALYs (Dis	Oman	Male	55+ years	Non-melanoma skin cancer (basal-cell	Percent	1990	1.23E-07	2.43E-07	5.44E-08
DALYs (Dis	Oman	Female	55+ years	Non-melanoma skin cancer (basal-cell	Percent	1990	1.09E-07	2.12E-07	4.94E-08
DALYs (Dis	Oman	Both	55+ years	Non-melanoma skin cancer (basal-cell	Percent	1990	1.17E-07	2.27E-07	5.30E-08
DALYs (Dis	Oman	Male	55+ years	Non-melanoma skin cancer (basal-cell	Rate	1990	0.0140724	0.027807882	0.006301996
DALYs (Dis	Oman	Female	55+ years	Non-melanoma skin cancer (basal-cell	Rate	1990	0.0102804	0.019879869	0.00422928
DALYs (Dis	Oman	Both	55+ years	Non-melanoma skin cancer (basal-cell	Rate	1990	0.0123289	0.024523804	0.005426367
DALYs (Dis	Vanuatu	Male	55+ years	Non-melanoma skin cancer (basal-cell	Number	2021	1.31E-05	3.55E-05	2.62E-06
DALYs (Dis	Vanuatu	Female	55+ years	Non-melanoma skin cancer (basal-cell	Number	2021	1.44E-05	3.76E-05	3.23E-06
DALYs (Dis	Vanuatu	Both	55+ years	Non-melanoma skin cancer (basal-cell	Number	2021	2.75E-05	7.43E-05	6.67E-06
DALYs (Dis	Vanuatu	Male	55+ years	Non-melanoma skin cancer (basal-cell	Percent	2021	6.63E-10	1.74E-09	1.32E-10
DALYs (Dis	Vanuatu	Female	55+ years	Non-melanoma skin cancer (basal-cell	Percent	2021	9.78E-10	2.67E-09	2.21E-10
DALYs (Dis	Vanuatu	Both	55+ years	Non-melanoma skin cancer (basal-cell	Percent	2021	7.97E-10	2.17E-09	1.98E-10
DALYs (Dis	Vanuatu	Male	55+ years	Non-melanoma skin cancer (basal-cell	Rate	2021	9.30E-05	0.000251739	1.86E-05
DALYs (Dis	Vanuatu	Female	55+ years	Non-melanoma skin cancer (basal-cell	Rate	2021	9.86E-05	0.000257674	2.22E-05
DALYs (Dis	Vanuatu	Both	55+ years	Non-melanoma skin cancer (basal-cell	Rate	2021	9.58E-05	0.000259061	2.33E-05
DALYs (Dis	Netherland	Male	55+ years	Non-melanoma skin cancer (basal-cell	Number	2021	2.3019049	4.347070748	1.054710731
DALYs (Dis	Netherland	Female	55+ years	Non-melanoma skin cancer (basal-cell	Number	2021	2.0265595	4.013731993	0.869879546
DALYs (Dis	Netherland	Both	55+ years	Non-melanoma skin cancer (basal-cell	Number	2021	4.3284645	8.18975084	1.896351952
DALYs (Dis	Netherland	Male	55+ years	Non-melanoma skin cancer (basal-cell	Percent	2021	1.26E-06	2.35E-06	6.10E-07
DALYs (Dis	Netherland	Female	55+ years	Non-melanoma skin cancer (basal-cell	Percent	2021	1.11E-06	2.07E-06	4.85E-07
DALYs (Dis	Netherland	Both	55+ years	Non-melanoma skin cancer (basal-cell	Percent	2021	1.19E-06	2.21E-06	5.27E-07
DALYs (Dis	Netherland	Male	55+ years	Non-melanoma skin cancer (basal-cell	Rate	2021	0.0822674	0.155359296	0.037694145
DALYs (Dis	Netherland	Female	55+ years	Non-melanoma skin cancer (basal-cell	Rate	2021	0.0663837	0.13147719	0.028494508
DALYs (Dis	Netherland	Both	55+ years	Non-melanoma skin cancer (basal-cell	Rate	2021	0.0739798	0.139974854	0.032411436
DALYs (Dis	Saint Vinc	Male	55+ years	Non-melanoma skin cancer (basal-cell	Number	1990	0.0008117	0.00157939	0.000333807
DALYs (Dis	Saint Vinc	Female	55+ years	Non-melanoma skin cancer (basal-cell	Number	1990	0.0004142	0.00081036	0.00017153
DALYs (Dis	Saint Vinc	Both	55+ years	Non-melanoma skin cancer (basal-cell	Number	1990	0.0012259	0.002305708	0.000511207
DALYs (Dis	Saint Vinc	Male	55+ years	Non-melanoma skin cancer (basal-cell	Percent	1990	1.53E-07	2.92E-07	6.59E-08
DALYs (Dis	Saint Vinc	Female	55+ years	Non-melanoma skin cancer (basal-cell	Percent	1990	6.71E-08	1.30E-07	2.90E-08
DALYs (Dis	Saint Vinc	Both	55+ years	Non-melanoma skin cancer (basal-cell	Percent	1990	1.07E-07	2.02E-07	4.67E-08
DALYs (Dis	Saint Vinc	Male	55+ years	Non-melanoma skin cancer (basal-cell	Rate	1990	0.0152582	0.029688711	0.00627476
DALYs (Dis	Saint Vinc	Female	55+ years	Non-melanoma skin cancer (basal-cell	Rate	1990	0.0061869	0.01210453	0.002562187
DALYs (Dis	Saint Vinc	Both	55+ years	Non-melanoma skin cancer (basal-cell	Rate	1990	0.0102035	0.01919102	0.004254916
DALYs (Dis	Tuvalu	Male	55+ years	Non-melanoma skin cancer (basal-cell	Number	2021	8.20E-07	2.16E-06	1.70E-07
DALYs (Dis	Tuvalu	Female	55+ years	Non-melanoma skin cancer (basal-cell	Number	2021	9.91E-07	2.56E-06	2.12E-07
DALYs (Dis	Tuvalu	Both	55+ years	Non-melanoma skin cancer (basal-cell	Number	2021	1.81E-06	4.82E-06	4.20E-07
DALYs (Dis	Tuvalu	Male	55+ years	Non-melanoma skin cancer (basal-cell	Percent	2021	8.46E-10	2.21E-09	1.79E-10
DALYs (Dis	Tuvalu	Female	55+ years	Non-melanoma skin cancer (basal-cell	Percent	2021	9.98E-10	2.59E-09	2.33E-10
DALYs (Dis	Tuvalu	Both	55+ years	Non-melanoma skin cancer (basal-cell	Percent	2021	9.21E-10	2.38E-09	2.14E-10

DALYs (Dis	Tuvalu	Male	55+ years	Non-melanoma skin cancer (basal-cell	Rate	2021	9.75E-05	0.000256342	2.02E-05
DALYs (Dis	Tuvalu	Female	55+ years	Non-melanoma skin cancer (basal-cell	Rate	2021	0.000101	0.000260338	2.16E-05
DALYs (Dis	Tuvalu	Both	55+ years	Non-melanoma skin cancer (basal-cell	Rate	2021	9.94E-05	0.000264492	2.30E-05
DALYs (Dis	Syrian Ara	Male	55+ years	Non-melanoma skin cancer (basal-cell	Number	1990	0.0710614	0.139183107	0.029337898
DALYs (Dis	Syrian Ara	Female	55+ years	Non-melanoma skin cancer (basal-cell	Number	1990	0.0367514	0.072295301	0.015331036
DALYs (Dis	Syrian Ara	Both	55+ years	Non-melanoma skin cancer (basal-cell	Number	1990	0.1078127	0.21201912	0.04444317
DALYs (Dis	Syrian Ara	Male	55+ years	Non-melanoma skin cancer (basal-cell	Percent	1990	1.62E-07	3.02E-07	6.81E-08
DALYs (Dis	Syrian Ara	Female	55+ years	Non-melanoma skin cancer (basal-cell	Percent	1990	1.04E-07	1.96E-07	4.31E-08
DALYs (Dis	Syrian Ara	Both	55+ years	Non-melanoma skin cancer (basal-cell	Percent	1990	1.36E-07	2.53E-07	5.73E-08
DALYs (Dis	Syrian Ara	Male	55+ years	Non-melanoma skin cancer (basal-cell	Rate	1990	0.0152623	0.029893273	0.006301094
DALYs (Dis	Syrian Ara	Female	55+ years	Non-melanoma skin cancer (basal-cell	Rate	1990	0.0088329	0.01737561	0.003684695
DALYs (Dis	Syrian Ara	Both	55+ years	Non-melanoma skin cancer (basal-cell	Rate	1990	0.0122282	0.02404735	0.005040774
DALYs (Dis	Palau	Male	55+ years	Non-melanoma skin cancer (basal-cell	Number	2021	2.08E-06	5.65E-06	4.34E-07
DALYs (Dis	Palau	Female	55+ years	Non-melanoma skin cancer (basal-cell	Number	2021	2.20E-06	5.92E-06	4.86E-07
DALYs (Dis	Palau	Both	55+ years	Non-melanoma skin cancer (basal-cell	Number	2021	4.28E-06	1.17E-05	1.05E-06
DALYs (Dis	Palau	Male	55+ years	Non-melanoma skin cancer (basal-cell	Percent	2021	1.01E-09	2.70E-09	2.15E-10
DALYs (Dis	Palau	Female	55+ years	Non-melanoma skin cancer (basal-cell	Percent	2021	1.21E-09	3.18E-09	2.75E-10
DALYs (Dis	Palau	Both	55+ years	Non-melanoma skin cancer (basal-cell	Percent	2021	1.10E-09	2.88E-09	2.81E-10
DALYs (Dis	Palau	Male	55+ years	Non-melanoma skin cancer (basal-cell	Rate	2021	9.87E-05	0.000267452	2.05E-05
DALYs (Dis	Palau	Female	55+ years	Non-melanoma skin cancer (basal-cell	Rate	2021	0.0001058	0.000285008	2.34E-05
DALYs (Dis	Palau	Both	55+ years	Non-melanoma skin cancer (basal-cell	Rate	2021	0.0001022	0.000279189	2.51E-05
DALYs (Dis	Honduras	Male	55+ years	Non-melanoma skin cancer (basal-cell	Number	1990	0.1001839	0.201375056	0.044657236
DALYs (Dis	Honduras	Female	55+ years	Non-melanoma skin cancer (basal-cell	Number	1990	0.0934972	0.178101274	0.040802592
DALYs (Dis	Honduras	Both	55+ years	Non-melanoma skin cancer (basal-cell	Number	1990	0.1936811	0.375409205	0.087523979
DALYs (Dis	Honduras	Male	55+ years	Non-melanoma skin cancer (basal-cell	Percent	1990	7.42E-07	1.38E-06	3.29E-07
DALYs (Dis	Honduras	Female	55+ years	Non-melanoma skin cancer (basal-cell	Percent	1990	7.86E-07	1.49E-06	3.49E-07
DALYs (Dis	Honduras	Both	55+ years	Non-melanoma skin cancer (basal-cell	Percent	1990	7.61E-07	1.46E-06	3.55E-07
DALYs (Dis	Honduras	Male	55+ years	Non-melanoma skin cancer (basal-cell	Rate	1990	0.0614926	0.123603408	0.027410478
DALYs (Dis	Honduras	Female	55+ years	Non-melanoma skin cancer (basal-cell	Rate	1990	0.0548707	0.10452227	0.023945811
DALYs (Dis	Honduras	Both	55+ years	Non-melanoma skin cancer (basal-cell	Rate	1990	0.0581074	0.112628673	0.026258572
DALYs (Dis	Guatemala	Male	55+ years	Non-melanoma skin cancer (basal-cell	Number	1990	0.1649057	0.341122453	0.070038938
DALYs (Dis	Guatemala	Female	55+ years	Non-melanoma skin cancer (basal-cell	Number	1990	0.1518588	0.292665336	0.065681985
DALYs (Dis	Guatemala	Both	55+ years	Non-melanoma skin cancer (basal-cell	Number	1990	0.3167646	0.626024347	0.137860528
DALYs (Dis	Guatemala	Male	55+ years	Non-melanoma skin cancer (basal-cell	Percent	1990	5.46E-07	1.11E-06	2.35E-07
DALYs (Dis	Guatemala	Female	55+ years	Non-melanoma skin cancer (basal-cell	Percent	1990	5.76E-07	1.08E-06	2.59E-07
DALYs (Dis	Guatemala	Both	55+ years	Non-melanoma skin cancer (basal-cell	Percent	1990	5.60E-07	1.09E-06	2.39E-07
DALYs (Dis	Guatemala	Male	55+ years	Non-melanoma skin cancer (basal-cell	Rate	1990	0.0572766	0.118481899	0.024326591
DALYs (Dis	Guatemala	Female	55+ years	Non-melanoma skin cancer (basal-cell	Rate	1990	0.0528799	0.101911239	0.022871627
DALYs (Dis	Guatemala	Both	55+ years	Non-melanoma skin cancer (basal-cell	Rate	1990	0.0550811	0.10885719	0.023972086
DALYs (Dis	Belarus	Male	55+ years	Non-melanoma skin cancer (basal-cell	Number	1990	0.4184656	0.834580288	0.19252106
DALYs (Dis	Belarus	Female	55+ years	Non-melanoma skin cancer (basal-cell	Number	1990	0.8046645	1.619287252	0.358985173
DALYs (Dis	Belarus	Both	55+ years	Non-melanoma skin cancer (basal-cell	Number	1990	1.2231301	2.424595002	0.561185093
DALYs (Dis	Belarus	Male	55+ years	Non-melanoma skin cancer (basal-cell	Percent	1990	4.29E-07	8.24E-07	1.98E-07
DALYs (Dis	Belarus	Female	55+ years	Non-melanoma skin cancer (basal-cell	Percent	1990	6.86E-07	1.34E-06	3.22E-07
DALYs (Dis	Belarus	Both	55+ years	Non-melanoma skin cancer (basal-cell	Percent	1990	5.70E-07	1.09E-06	2.63E-07
DALYs (Dis	Belarus	Male	55+ years	Non-melanoma skin cancer (basal-cell	Rate	1990	0.0491692	0.098062239	0.022621007
DALYs (Dis	Belarus	Female	55+ years	Non-melanoma skin cancer (basal-cell	Rate	1990	0.0549686	0.110617466	0.024523154
DALYs (Dis	Belarus	Both	55+ years	Non-melanoma skin cancer (basal-cell	Rate	1990	0.0528365	0.104737102	0.024241946
DALYs (Dis	Montenegro	Male	55+ years	Non-melanoma skin cancer (basal-cell	Number	2021	0.0569808	0.111229586	0.024125124
DALYs (Dis	Montenegro	Female	55+ years	Non-melanoma skin cancer (basal-cell	Number	2021	0.0631089	0.124582858	0.026298169
DALYs (Dis	Montenegro	Both	55+ years	Non-melanoma skin cancer (basal-cell	Number	2021	0.1200896	0.231364881	0.050330469
DALYs (Dis	Montenegro	Male	55+ years	Non-melanoma skin cancer (basal-cell	Percent	2021	5.39E-07	1.02E-06	2.31E-07
DALYs (Dis	Montenegro	Female	55+ years	Non-melanoma skin cancer (basal-cell	Percent	2021	6.94E-07	1.40E-06	2.95E-07
DALYs (Dis	Montenegro	Both	55+ years	Non-melanoma skin cancer (basal-cell	Percent	2021	6.11E-07	1.18E-06	2.60E-07
DALYs (Dis	Montenegro	Male	55+ years	Non-melanoma skin cancer (basal-cell	Rate	2021	0.0717952	0.140148136	0.030397408
DALYs (Dis	Montenegro	Female	55+ years	Non-melanoma skin cancer (basal-cell	Rate	2021	0.0664252	0.131129559	0.027680111

DALYs (Dis	Montenegro	Both	55+ years	Non-melanoma skin cancer (basal-cell	Rate	2021	0.0688693	0.132683752	0.028863652
DALYs (Dis	Papua New	Male	55+ years	Non-melanoma skin cancer (basal-cell	Number	2021	0.0004678	0.001262374	9.37E-05
DALYs (Dis	Papua New	Female	55+ years	Non-melanoma skin cancer (basal-cell	Number	2021	0.0003962	0.00108265	9.08E-05
DALYs (Dis	Papua New	Both	55+ years	Non-melanoma skin cancer (basal-cell	Number	2021	0.0008639	0.002297642	0.000220644
DALYs (Dis	Papua New	Male	55+ years	Non-melanoma skin cancer (basal-cell	Percent	2021	7.81E-10	2.08E-09	1.69E-10
DALYs (Dis	Papua New	Female	55+ years	Non-melanoma skin cancer (basal-cell	Percent	2021	9.64E-10	2.56E-09	2.06E-10
DALYs (Dis	Papua New	Both	55+ years	Non-melanoma skin cancer (basal-cell	Percent	2021	8.55E-10	2.31E-09	1.99E-10
DALYs (Dis	Papua New	Male	55+ years	Non-melanoma skin cancer (basal-cell	Rate	2021	0.0001052	0.000283961	2.11E-05
DALYs (Dis	Papua New	Female	55+ years	Non-melanoma skin cancer (basal-cell	Rate	2021	0.0001052	0.00028737	2.41E-05
DALYs (Dis	Papua New	Both	55+ years	Non-melanoma skin cancer (basal-cell	Rate	2021	0.0001052	0.000279756	2.69E-05
DALYs (Dis	United Sta	Male	55+ years	Non-melanoma skin cancer (basal-cell	Number	1990	0.0008775	0.001725944	0.000371272
DALYs (Dis	United Sta	Female	55+ years	Non-melanoma skin cancer (basal-cell	Number	1990	0.0004284	0.000830586	0.000177355
DALYs (Dis	United Sta	Both	55+ years	Non-melanoma skin cancer (basal-cell	Number	1990	0.0013059	0.002566154	0.000557026
DALYs (Dis	United Sta	Male	55+ years	Non-melanoma skin cancer (basal-cell	Percent	1990	1.57E-07	3.09E-07	6.79E-08
DALYs (Dis	United Sta	Female	55+ years	Non-melanoma skin cancer (basal-cell	Percent	1990	8.01E-08	1.53E-07	3.36E-08
DALYs (Dis	United Sta	Both	55+ years	Non-melanoma skin cancer (basal-cell	Percent	1990	1.20E-07	2.33E-07	5.19E-08
DALYs (Dis	United Sta	Male	55+ years	Non-melanoma skin cancer (basal-cell	Rate	1990	0.0134611	0.026475078	0.005695118
DALYs (Dis	United Sta	Female	55+ years	Non-melanoma skin cancer (basal-cell	Rate	1990	0.0057516	0.011151729	0.002381224
DALYs (Dis	United Sta	Both	55+ years	Non-melanoma skin cancer (basal-cell	Rate	1990	0.00935	0.018372751	0.003988108
DALYs (Dis	Maldives	Male	55+ years	Non-melanoma skin cancer (basal-cell	Number	2021	0.0009014	0.001712862	0.000362925
DALYs (Dis	Maldives	Female	55+ years	Non-melanoma skin cancer (basal-cell	Number	2021	0.0005724	0.001127811	0.000229554
DALYs (Dis	Maldives	Both	55+ years	Non-melanoma skin cancer (basal-cell	Number	2021	0.0014738	0.002826965	0.000584882
DALYs (Dis	Maldives	Male	55+ years	Non-melanoma skin cancer (basal-cell	Percent	2021	5.39E-08	1.02E-07	2.18E-08
DALYs (Dis	Maldives	Female	55+ years	Non-melanoma skin cancer (basal-cell	Percent	2021	4.42E-08	8.46E-08	1.86E-08
DALYs (Dis	Maldives	Both	55+ years	Non-melanoma skin cancer (basal-cell	Percent	2021	4.97E-08	9.39E-08	2.03E-08
DALYs (Dis	Maldives	Male	55+ years	Non-melanoma skin cancer (basal-cell	Rate	2021	0.0030709	0.005835548	0.001236448
DALYs (Dis	Maldives	Female	55+ years	Non-melanoma skin cancer (basal-cell	Rate	2021	0.0022855	0.004503284	0.000916596
DALYs (Dis	Maldives	Both	55+ years	Non-melanoma skin cancer (basal-cell	Rate	2021	0.0027093	0.00519697	0.001075222
DALYs (Dis	Finland	Male	55+ years	Non-melanoma skin cancer (basal-cell	Number	2021	1.0533001	2.066457783	0.420703033
DALYs (Dis	Finland	Female	55+ years	Non-melanoma skin cancer (basal-cell	Number	2021	0.9957063	2.030814986	0.42768205
DALYs (Dis	Finland	Both	55+ years	Non-melanoma skin cancer (basal-cell	Number	2021	2.0490064	4.032580211	0.821974044
DALYs (Dis	Finland	Male	55+ years	Non-melanoma skin cancer (basal-cell	Percent	2021	1.64E-06	3.10E-06	6.63E-07
DALYs (Dis	Finland	Female	55+ years	Non-melanoma skin cancer (basal-cell	Percent	2021	1.61E-06	3.17E-06	7.06E-07
DALYs (Dis	Finland	Both	55+ years	Non-melanoma skin cancer (basal-cell	Percent	2021	1.62E-06	3.09E-06	6.78E-07
DALYs (Dis	Finland	Male	55+ years	Non-melanoma skin cancer (basal-cell	Rate	2021	0.1134686	0.222612766	0.045320967
DALYs (Dis	Finland	Female	55+ years	Non-melanoma skin cancer (basal-cell	Rate	2021	0.0918216	0.187276782	0.039439791
DALYs (Dis	Finland	Both	55+ years	Non-melanoma skin cancer (basal-cell	Rate	2021	0.1018055	0.20036004	0.040840044
DALYs (Dis	Tonga	Male	55+ years	Non-melanoma skin cancer (basal-cell	Number	1990	5.15E-06	1.39E-05	1.18E-06
DALYs (Dis	Tonga	Female	55+ years	Non-melanoma skin cancer (basal-cell	Number	1990	6.01E-06	1.61E-05	1.47E-06
DALYs (Dis	Tonga	Both	55+ years	Non-melanoma skin cancer (basal-cell	Number	1990	1.12E-05	2.99E-05	2.76E-06
DALYs (Dis	Tonga	Male	55+ years	Non-melanoma skin cancer (basal-cell	Percent	1990	1.09E-09	2.91E-09	2.50E-10
DALYs (Dis	Tonga	Female	55+ years	Non-melanoma skin cancer (basal-cell	Percent	1990	1.56E-09	4.16E-09	3.86E-10
DALYs (Dis	Tonga	Both	55+ years	Non-melanoma skin cancer (basal-cell	Percent	1990	1.30E-09	3.52E-09	3.35E-10
DALYs (Dis	Tonga	Male	55+ years	Non-melanoma skin cancer (basal-cell	Rate	1990	0.0001103	0.000298109	2.53E-05
DALYs (Dis	Tonga	Female	55+ years	Non-melanoma skin cancer (basal-cell	Rate	1990	0.0001247	0.000335181	3.04E-05
DALYs (Dis	Tonga	Both	55+ years	Non-melanoma skin cancer (basal-cell	Rate	1990	0.0001176	0.000315167	2.90E-05
DALYs (Dis	Slovakia	Male	55+ years	Non-melanoma skin cancer (basal-cell	Number	2021	0.5564861	1.145453372	0.204158407
DALYs (Dis	Slovakia	Female	55+ years	Non-melanoma skin cancer (basal-cell	Number	2021	0.6992496	1.385792414	0.272652799
DALYs (Dis	Slovakia	Both	55+ years	Non-melanoma skin cancer (basal-cell	Number	2021	1.2557358	2.491142288	0.47631227
DALYs (Dis	Slovakia	Male	55+ years	Non-melanoma skin cancer (basal-cell	Percent	2021	6.93E-07	1.39E-06	2.54E-07
DALYs (Dis	Slovakia	Female	55+ years	Non-melanoma skin cancer (basal-cell	Percent	2021	9.41E-07	1.80E-06	3.79E-07
DALYs (Dis	Slovakia	Both	55+ years	Non-melanoma skin cancer (basal-cell	Percent	2021	8.12E-07	1.57E-06	3.23E-07
DALYs (Dis	Slovakia	Male	55+ years	Non-melanoma skin cancer (basal-cell	Rate	2021	0.0779619	0.160474193	0.028601911
DALYs (Dis	Slovakia	Female	55+ years	Non-melanoma skin cancer (basal-cell	Rate	2021	0.0754735	0.149575462	0.029428772
DALYs (Dis	Slovakia	Both	55+ years	Non-melanoma skin cancer (basal-cell	Rate	2021	0.0765563	0.151873298	0.029038532
DALYs (Dis	Chad	Male	55+ years	Non-melanoma skin cancer (basal-cell	Number	2021	0.0401421	0.082625619	0.016184226

DALYs (Dis	Chad	Female	55+ years	Non-melanoma skin cancer (basal-cell	Number	2021	0.0189938	0.038295282	0.007637919
DALYs (Dis	Chad	Both	55+ years	Non-melanoma skin cancer (basal-cell	Number	2021	0.0591359	0.120482767	0.023650012
DALYs (Dis	Chad	Male	55+ years	Non-melanoma skin cancer (basal-cell	Percent	2021	5.35E-08	1.04E-07	2.32E-08
DALYs (Dis	Chad	Female	55+ years	Non-melanoma skin cancer (basal-cell	Percent	2021	3.87E-08	7.74E-08	1.63E-08
DALYs (Dis	Chad	Both	55+ years	Non-melanoma skin cancer (basal-cell	Percent	2021	4.76E-08	9.30E-08	2.09E-08
DALYs (Dis	Chad	Male	55+ years	Non-melanoma skin cancer (basal-cell	Rate	2021	0.007825	0.016106318	0.003154812
DALYs (Dis	Chad	Female	55+ years	Non-melanoma skin cancer (basal-cell	Rate	2021	0.0045711	0.009216207	0.001838155
DALYs (Dis	Chad	Both	55+ years	Non-melanoma skin cancer (basal-cell	Rate	2021	0.0063688	0.012975753	0.002547059
DALYs (Dis	Japan	Male	55+ years	Non-melanoma skin cancer (basal-cell	Number	1990	1.4746297	2.844715525	0.596758593
DALYs (Dis	Japan	Female	55+ years	Non-melanoma skin cancer (basal-cell	Number	1990	1.7455116	3.371205755	0.709987605
DALYs (Dis	Japan	Both	55+ years	Non-melanoma skin cancer (basal-cell	Number	1990	3.2201413	6.176124933	1.305901957
DALYs (Dis	Japan	Male	55+ years	Non-melanoma skin cancer (basal-cell	Percent	1990	1.55E-07	2.96E-07	6.67E-08
DALYs (Dis	Japan	Female	55+ years	Non-melanoma skin cancer (basal-cell	Percent	1990	2.02E-07	3.82E-07	8.71E-08
DALYs (Dis	Japan	Both	55+ years	Non-melanoma skin cancer (basal-cell	Percent	1990	1.77E-07	3.38E-07	7.62E-08
DALYs (Dis	Japan	Male	55+ years	Non-melanoma skin cancer (basal-cell	Rate	1990	0.0112215	0.021647386	0.004541144
DALYs (Dis	Japan	Female	55+ years	Non-melanoma skin cancer (basal-cell	Rate	1990	0.0105968	0.020466153	0.004310243
DALYs (Dis	Japan	Both	55+ years	Non-melanoma skin cancer (basal-cell	Rate	1990	0.010874	0.020855949	0.004409857
DALYs (Dis	Slovenia	Male	55+ years	Non-melanoma skin cancer (basal-cell	Number	1990	0.1231206	0.25124376	0.054165895
DALYs (Dis	Slovenia	Female	55+ years	Non-melanoma skin cancer (basal-cell	Number	1990	0.1851528	0.355260979	0.081550742
DALYs (Dis	Slovenia	Both	55+ years	Non-melanoma skin cancer (basal-cell	Number	1990	0.3082734	0.598077729	0.139704724
DALYs (Dis	Slovenia	Male	55+ years	Non-melanoma skin cancer (basal-cell	Percent	1990	6.61E-07	1.32E-06	2.97E-07
DALYs (Dis	Slovenia	Female	55+ years	Non-melanoma skin cancer (basal-cell	Percent	1990	9.68E-07	1.79E-06	4.28E-07
DALYs (Dis	Slovenia	Both	55+ years	Non-melanoma skin cancer (basal-cell	Percent	1990	8.16E-07	1.54E-06	3.88E-07
DALYs (Dis	Slovenia	Male	55+ years	Non-melanoma skin cancer (basal-cell	Rate	1990	0.0715488	0.146004757	0.031477313
DALYs (Dis	Slovenia	Female	55+ years	Non-melanoma skin cancer (basal-cell	Rate	1990	0.0715906	0.137364028	0.031532139
DALYs (Dis	Slovenia	Both	55+ years	Non-melanoma skin cancer (basal-cell	Rate	1990	0.0715739	0.138859683	0.032436175
DALYs (Dis	Japan	Male	55+ years	Non-melanoma skin cancer (basal-cell	Number	2021	4.9080556	9.50568413	1.965874208
DALYs (Dis	Japan	Female	55+ years	Non-melanoma skin cancer (basal-cell	Number	2021	6.0208749	11.7657365	2.471853556
DALYs (Dis	Japan	Both	55+ years	Non-melanoma skin cancer (basal-cell	Number	2021	10.92893	21.34185969	4.406417505
DALYs (Dis	Japan	Male	55+ years	Non-melanoma skin cancer (basal-cell	Percent	2021	3.20E-07	6.07E-07	1.32E-07
DALYs (Dis	Japan	Female	55+ years	Non-melanoma skin cancer (basal-cell	Percent	2021	4.17E-07	7.92E-07	1.79E-07
DALYs (Dis	Japan	Both	55+ years	Non-melanoma skin cancer (basal-cell	Percent	2021	3.67E-07	6.95E-07	1.54E-07
DALYs (Dis	Japan	Male	55+ years	Non-melanoma skin cancer (basal-cell	Rate	2021	0.0206639	0.040020868	0.008276731
DALYs (Dis	Japan	Female	55+ years	Non-melanoma skin cancer (basal-cell	Rate	2021	0.0211637	0.041357163	0.008688691
DALYs (Dis	Japan	Both	55+ years	Non-melanoma skin cancer (basal-cell	Rate	2021	0.0209363	0.040884077	0.008441266
DALYs (Dis	Burkina Fa	Male	55+ years	Non-melanoma skin cancer (basal-cell	Number	1990	0.0275078	0.054814391	0.011510104
DALYs (Dis	Burkina Fa	Female	55+ years	Non-melanoma skin cancer (basal-cell	Number	1990	0.0176927	0.035111715	0.007053191
DALYs (Dis	Burkina Fa	Both	55+ years	Non-melanoma skin cancer (basal-cell	Number	1990	0.0452005	0.088469073	0.018858957
DALYs (Dis	Burkina Fa	Male	55+ years	Non-melanoma skin cancer (basal-cell	Percent	1990	5.57E-08	1.10E-07	2.32E-08
DALYs (Dis	Burkina Fa	Female	55+ years	Non-melanoma skin cancer (basal-cell	Percent	1990	4.14E-08	8.22E-08	1.66E-08
DALYs (Dis	Burkina Fa	Both	55+ years	Non-melanoma skin cancer (basal-cell	Percent	1990	4.90E-08	9.75E-08	2.00E-08
DALYs (Dis	Burkina Fa	Male	55+ years	Non-melanoma skin cancer (basal-cell	Rate	1990	0.0078423	0.015627216	0.003281454
DALYs (Dis	Burkina Fa	Female	55+ years	Non-melanoma skin cancer (basal-cell	Rate	1990	0.00475	0.009426572	0.001893596
DALYs (Dis	Burkina Fa	Both	55+ years	Non-melanoma skin cancer (basal-cell	Rate	1990	0.0062497	0.012232354	0.002607572
DALYs (Dis	Iraq	Male	55+ years	Non-melanoma skin cancer (basal-cell	Number	1990	0.0846982	0.164786405	0.033485414
DALYs (Dis	Iraq	Female	55+ years	Non-melanoma skin cancer (basal-cell	Number	1990	0.0576957	0.110461868	0.02343308
DALYs (Dis	Iraq	Both	55+ years	Non-melanoma skin cancer (basal-cell	Number	1990	0.142394	0.272976247	0.056943007
DALYs (Dis	Iraq	Male	55+ years	Non-melanoma skin cancer (basal-cell	Percent	1990	1.27E-07	2.49E-07	5.34E-08
DALYs (Dis	Iraq	Female	55+ years	Non-melanoma skin cancer (basal-cell	Percent	1990	1.02E-07	1.94E-07	4.38E-08
DALYs (Dis	Iraq	Both	55+ years	Non-melanoma skin cancer (basal-cell	Percent	1990	1.15E-07	2.23E-07	4.93E-08
DALYs (Dis	Iraq	Male	55+ years	Non-melanoma skin cancer (basal-cell	Rate	1990	0.013836	0.026918825	0.005470039
DALYs (Dis	Iraq	Female	55+ years	Non-melanoma skin cancer (basal-cell	Rate	1990	0.0088746	0.01699089	0.003604401
DALYs (Dis	Iraq	Both	55+ years	Non-melanoma skin cancer (basal-cell	Rate	1990	0.0112807	0.021625572	0.004511107
DALYs (Dis	Andorra	Male	55+ years	Non-melanoma skin cancer (basal-cell	Number	2021	0.0144564	0.028563168	0.005307906
DALYs (Dis	Andorra	Female	55+ years	Non-melanoma skin cancer (basal-cell	Number	2021	0.0110697	0.022583978	0.004820724
DALYs (Dis	Andorra	Both	55+ years	Non-melanoma skin cancer (basal-cell	Number	2021	0.025526	0.049919223	0.010589926

DALYs (Dis	Andorra	Male	55+ years	Non-melanoma skin cancer (basal-cell	Percent	2021	1.81E-06	3.61E-06	6.72E-07
DALYs (Dis	Andorra	Female	55+ years	Non-melanoma skin cancer (basal-cell	Percent	2021	1.71E-06	3.24E-06	7.63E-07
DALYs (Dis	Andorra	Both	55+ years	Non-melanoma skin cancer (basal-cell	Percent	2021	1.77E-06	3.34E-06	7.36E-07
DALYs (Dis	Andorra	Male	55+ years	Non-melanoma skin cancer (basal-cell	Rate	2021	0.1066081	0.210638257	0.039143002
DALYs (Dis	Andorra	Female	55+ years	Non-melanoma skin cancer (basal-cell	Rate	2021	0.0858017	0.175049688	0.037365706
DALYs (Dis	Andorra	Both	55+ years	Non-melanoma skin cancer (basal-cell	Rate	2021	0.0964639	0.188646632	0.040019729
DALYs (Dis	Denmark	Male	55+ years	Non-melanoma skin cancer (basal-cell	Number	2021	1.1179443	2.264671299	0.456385323
DALYs (Dis	Denmark	Female	55+ years	Non-melanoma skin cancer (basal-cell	Number	2021	0.9556945	1.961045139	0.389896269
DALYs (Dis	Denmark	Both	55+ years	Non-melanoma skin cancer (basal-cell	Number	2021	2.0736388	4.048013263	0.901996029
DALYs (Dis	Denmark	Male	55+ years	Non-melanoma skin cancer (basal-cell	Percent	2021	1.78E-06	3.56E-06	7.53E-07
DALYs (Dis	Denmark	Female	55+ years	Non-melanoma skin cancer (basal-cell	Percent	2021	1.59E-06	3.20E-06	6.71E-07
DALYs (Dis	Denmark	Both	55+ years	Non-melanoma skin cancer (basal-cell	Percent	2021	1.69E-06	3.26E-06	7.59E-07
DALYs (Dis	Denmark	Male	55+ years	Non-melanoma skin cancer (basal-cell	Rate	2021	0.1221818	0.247509355	0.049879043
DALYs (Dis	Denmark	Female	55+ years	Non-melanoma skin cancer (basal-cell	Rate	2021	0.0946009	0.194117036	0.038594475
DALYs (Dis	Denmark	Both	55+ years	Non-melanoma skin cancer (basal-cell	Rate	2021	0.107709	0.210262081	0.046851517
DALYs (Dis	Algeria	Male	55+ years	Non-melanoma skin cancer (basal-cell	Number	2021	0.5774611	1.129461462	0.2426614
DALYs (Dis	Algeria	Female	55+ years	Non-melanoma skin cancer (basal-cell	Number	2021	0.3446146	0.673367469	0.140167728
DALYs (Dis	Algeria	Both	55+ years	Non-melanoma skin cancer (basal-cell	Number	2021	0.9220757	1.805626393	0.390678623
DALYs (Dis	Algeria	Male	55+ years	Non-melanoma skin cancer (basal-cell	Percent	2021	2.08E-07	3.91E-07	9.00E-08
DALYs (Dis	Algeria	Female	55+ years	Non-melanoma skin cancer (basal-cell	Percent	2021	1.53E-07	2.94E-07	6.52E-08
DALYs (Dis	Algeria	Both	55+ years	Non-melanoma skin cancer (basal-cell	Percent	2021	1.84E-07	3.52E-07	7.96E-08
DALYs (Dis	Algeria	Male	55+ years	Non-melanoma skin cancer (basal-cell	Rate	2021	0.0186828	0.036541956	0.007850929
DALYs (Dis	Algeria	Female	55+ years	Non-melanoma skin cancer (basal-cell	Rate	2021	0.0115545	0.022577222	0.00469966
DALYs (Dis	Algeria	Both	55+ years	Non-melanoma skin cancer (basal-cell	Rate	2021	0.0151823	0.029730221	0.00643265
DALYs (Dis	Albania	Male	55+ years	Non-melanoma skin cancer (basal-cell	Number	1990	0.1194007	0.23290844	0.047679236
DALYs (Dis	Albania	Female	55+ years	Non-melanoma skin cancer (basal-cell	Number	1990	0.1203575	0.255640693	0.050489909
DALYs (Dis	Albania	Both	55+ years	Non-melanoma skin cancer (basal-cell	Number	1990	0.2397582	0.462010466	0.100544957
DALYs (Dis	Albania	Male	55+ years	Non-melanoma skin cancer (basal-cell	Percent	1990	7.61E-07	1.45E-06	3.12E-07
DALYs (Dis	Albania	Female	55+ years	Non-melanoma skin cancer (basal-cell	Percent	1990	9.99E-07	2.05E-06	4.40E-07
DALYs (Dis	Albania	Both	55+ years	Non-melanoma skin cancer (basal-cell	Percent	1990	8.64E-07	1.64E-06	3.78E-07
DALYs (Dis	Albania	Male	55+ years	Non-melanoma skin cancer (basal-cell	Rate	1990	0.0701915	0.136918767	0.028028964
DALYs (Dis	Albania	Female	55+ years	Non-melanoma skin cancer (basal-cell	Rate	1990	0.0680303	0.144497098	0.02853867
DALYs (Dis	Albania	Both	55+ years	Non-melanoma skin cancer (basal-cell	Rate	1990	0.0690897	0.133134807	0.028973442
DALYs (Dis	Monaco	Male	55+ years	Non-melanoma skin cancer (basal-cell	Number	1990	0.0055684	0.011839671	0.002209824
DALYs (Dis	Monaco	Female	55+ years	Non-melanoma skin cancer (basal-cell	Number	1990	0.0058198	0.012246921	0.002332905
DALYs (Dis	Monaco	Both	55+ years	Non-melanoma skin cancer (basal-cell	Number	1990	0.0113882	0.023434126	0.004684102
DALYs (Dis	Monaco	Male	55+ years	Non-melanoma skin cancer (basal-cell	Percent	1990	1.29E-06	2.70E-06	5.47E-07
DALYs (Dis	Monaco	Female	55+ years	Non-melanoma skin cancer (basal-cell	Percent	1990	1.37E-06	2.84E-06	5.58E-07
DALYs (Dis	Monaco	Both	55+ years	Non-melanoma skin cancer (basal-cell	Percent	1990	1.33E-06	2.68E-06	5.52E-07
DALYs (Dis	Monaco	Male	55+ years	Non-melanoma skin cancer (basal-cell	Rate	1990	0.1150884	0.244702133	0.045672615
DALYs (Dis	Monaco	Female	55+ years	Non-melanoma skin cancer (basal-cell	Rate	1990	0.0932138	0.196154577	0.037365305
DALYs (Dis	Monaco	Both	55+ years	Non-melanoma skin cancer (basal-cell	Rate	1990	0.1027643	0.211462954	0.042268018
DALYs (Dis	Finland	Male	55+ years	Non-melanoma skin cancer (basal-cell	Number	1990	0.4788907	0.959124891	0.199198815
DALYs (Dis	Finland	Female	55+ years	Non-melanoma skin cancer (basal-cell	Number	1990	0.6284932	1.253572938	0.248804156
DALYs (Dis	Finland	Both	55+ years	Non-melanoma skin cancer (basal-cell	Number	1990	1.1073839	2.165532661	0.464666721
DALYs (Dis	Finland	Male	55+ years	Non-melanoma skin cancer (basal-cell	Percent	1990	9.82E-07	1.94E-06	4.13E-07
DALYs (Dis	Finland	Female	55+ years	Non-melanoma skin cancer (basal-cell	Percent	1990	1.22E-06	2.39E-06	5.01E-07
DALYs (Dis	Finland	Both	55+ years	Non-melanoma skin cancer (basal-cell	Percent	1990	1.11E-06	2.14E-06	4.63E-07
DALYs (Dis	Finland	Male	55+ years	Non-melanoma skin cancer (basal-cell	Rate	1990	0.0994996	0.199278252	0.041387719
DALYs (Dis	Finland	Female	55+ years	Non-melanoma skin cancer (basal-cell	Rate	1990	0.089015	0.177546572	0.035238735
DALYs (Dis	Finland	Both	55+ years	Non-melanoma skin cancer (basal-cell	Rate	1990	0.093265	0.182383322	0.039134695
DALYs (Dis	United Sta	Male	55+ years	Non-melanoma skin cancer (basal-cell	Number	2021	605.71912	1143.732644	286.0231237
DALYs (Dis	United Sta	Female	55+ years	Non-melanoma skin cancer (basal-cell	Number	2021	405.25907	780.3290242	190.4020206
DALYs (Dis	United Sta	Both	55+ years	Non-melanoma skin cancer (basal-cell	Number	2021	1010.9782	1935.716845	478.2465149
DALYs (Dis	United Sta	Male	55+ years	Non-melanoma skin cancer (basal-cell	Percent	2021	1.54E-05	2.83E-05	7.50E-06
DALYs (Dis	United Sta	Female	55+ years	Non-melanoma skin cancer (basal-cell	Percent	2021	1.08E-05	1.99E-05	5.37E-06

DALYs (Dis	United Sta	Both	55+ years	Non-melanoma skin cancer (basal-cell	Percent	2021	1.32E-05	2.43E-05	6.50E-06
DALYs (Dis	United Sta	Male	55+ years	Non-melanoma skin cancer (basal-cell	Rate	2021	1.3031405	2.460619556	0.615348434
DALYs (Dis	United Sta	Female	55+ years	Non-melanoma skin cancer (basal-cell	Rate	2021	0.7537422	1.451335646	0.354129131
DALYs (Dis	United Sta	Both	55+ years	Non-melanoma skin cancer (basal-cell	Rate	2021	1.0084796	1.930932781	0.477064543
DALYs (Dis	France	Male	55+ years	Non-melanoma skin cancer (basal-cell	Number	1990	8.9771709	17.89958023	3.805900809
DALYs (Dis	France	Female	55+ years	Non-melanoma skin cancer (basal-cell	Number	1990	10.62791	21.59353041	4.358999175
DALYs (Dis	France	Both	55+ years	Non-melanoma skin cancer (basal-cell	Number	1990	19.605081	40.91167456	8.54796306
DALYs (Dis	France	Male	55+ years	Non-melanoma skin cancer (basal-cell	Percent	1990	1.69E-06	3.33E-06	7.43E-07
DALYs (Dis	France	Female	55+ years	Non-melanoma skin cancer (basal-cell	Percent	1990	2.08E-06	4.14E-06	8.78E-07
DALYs (Dis	France	Both	55+ years	Non-melanoma skin cancer (basal-cell	Percent	1990	1.89E-06	3.83E-06	8.58E-07
DALYs (Dis	France	Male	55+ years	Non-melanoma skin cancer (basal-cell	Rate	1990	0.1501567	0.299397459	0.063659428
DALYs (Dis	France	Female	55+ years	Non-melanoma skin cancer (basal-cell	Rate	1990	0.133886	0.272026307	0.054912857
DALYs (Dis	France	Both	55+ years	Non-melanoma skin cancer (basal-cell	Rate	1990	0.1408759	0.293978259	0.061422939
DALYs (Dis	Portugal	Male	55+ years	Non-melanoma skin cancer (basal-cell	Number	1990	0.4837369	0.990917439	0.209441911
DALYs (Dis	Portugal	Female	55+ years	Non-melanoma skin cancer (basal-cell	Number	1990	0.6532325	1.292542453	0.280415868
DALYs (Dis	Portugal	Both	55+ years	Non-melanoma skin cancer (basal-cell	Number	1990	1.1369694	2.229369275	0.484730425
DALYs (Dis	Portugal	Male	55+ years	Non-melanoma skin cancer (basal-cell	Percent	1990	4.64E-07	9.23E-07	2.02E-07
DALYs (Dis	Portugal	Female	55+ years	Non-melanoma skin cancer (basal-cell	Percent	1990	6.56E-07	1.26E-06	2.92E-07
DALYs (Dis	Portugal	Both	55+ years	Non-melanoma skin cancer (basal-cell	Percent	1990	5.58E-07	1.07E-06	2.48E-07
DALYs (Dis	Portugal	Male	55+ years	Non-melanoma skin cancer (basal-cell	Rate	1990	0.0453435	0.092884413	0.0196322
DALYs (Dis	Portugal	Female	55+ years	Non-melanoma skin cancer (basal-cell	Rate	1990	0.0476416	0.094267762	0.020451302
DALYs (Dis	Portugal	Both	55+ years	Non-melanoma skin cancer (basal-cell	Rate	1990	0.0466359	0.091443747	0.019882559
DALYs (Dis	Pakistan	Male	55+ years	Non-melanoma skin cancer (basal-cell	Number	1990	0.0333026	0.065378809	0.013330001
DALYs (Dis	Pakistan	Female	55+ years	Non-melanoma skin cancer (basal-cell	Number	1990	0.021795	0.044514596	0.008533114
DALYs (Dis	Pakistan	Both	55+ years	Non-melanoma skin cancer (basal-cell	Number	1990	0.0550976	0.11067391	0.022314163
DALYs (Dis	Pakistan	Male	55+ years	Non-melanoma skin cancer (basal-cell	Percent	1990	6.15E-09	1.18E-08	2.57E-09
DALYs (Dis	Pakistan	Female	55+ years	Non-melanoma skin cancer (basal-cell	Percent	1990	5.32E-09	1.06E-08	2.13E-09
DALYs (Dis	Pakistan	Both	55+ years	Non-melanoma skin cancer (basal-cell	Percent	1990	5.79E-09	1.12E-08	2.39E-09
DALYs (Dis	Pakistan	Male	55+ years	Non-melanoma skin cancer (basal-cell	Rate	1990	0.0006486	0.001273241	0.0002596
DALYs (Dis	Pakistan	Female	55+ years	Non-melanoma skin cancer (basal-cell	Rate	1990	0.000547	0.001117155	0.00021415
DALYs (Dis	Pakistan	Both	55+ years	Non-melanoma skin cancer (basal-cell	Rate	1990	0.0006042	0.0012136	0.000244687
DALYs (Dis	El Salvado	Male	55+ years	Non-melanoma skin cancer (basal-cell	Number	1990	0.152035	0.298284766	0.062011103
DALYs (Dis	El Salvado	Female	55+ years	Non-melanoma skin cancer (basal-cell	Number	1990	0.1667057	0.327918614	0.070864608
DALYs (Dis	El Salvado	Both	55+ years	Non-melanoma skin cancer (basal-cell	Number	1990	0.3187408	0.610803099	0.132421189
DALYs (Dis	El Salvado	Male	55+ years	Non-melanoma skin cancer (basal-cell	Percent	1990	8.05E-07	1.53E-06	3.41E-07
DALYs (Dis	El Salvado	Female	55+ years	Non-melanoma skin cancer (basal-cell	Percent	1990	9.69E-07	1.78E-06	4.31E-07
DALYs (Dis	El Salvado	Both	55+ years	Non-melanoma skin cancer (basal-cell	Percent	1990	8.83E-07	1.63E-06	3.85E-07
DALYs (Dis	El Salvado	Male	55+ years	Non-melanoma skin cancer (basal-cell	Rate	1990	0.0669183	0.1312903	0.027294241
DALYs (Dis	El Salvado	Female	55+ years	Non-melanoma skin cancer (basal-cell	Rate	1990	0.0636856	0.125272855	0.027071997
DALYs (Dis	El Salvado	Both	55+ years	Non-melanoma skin cancer (basal-cell	Rate	1990	0.0651877	0.124919255	0.027082306
DALYs (Dis	Norway	Male	55+ years	Non-melanoma skin cancer (basal-cell	Number	2021	0.8992776	1.737319265	0.374901918
DALYs (Dis	Norway	Female	55+ years	Non-melanoma skin cancer (basal-cell	Number	2021	0.7574619	1.460160255	0.305634065
DALYs (Dis	Norway	Both	55+ years	Non-melanoma skin cancer (basal-cell	Number	2021	1.6567395	3.205122041	0.67511697
DALYs (Dis	Norway	Male	55+ years	Non-melanoma skin cancer (basal-cell	Percent	2021	1.97E-06	3.68E-06	8.98E-07
DALYs (Dis	Norway	Female	55+ years	Non-melanoma skin cancer (basal-cell	Percent	2021	1.62E-06	3.04E-06	6.92E-07
DALYs (Dis	Norway	Both	55+ years	Non-melanoma skin cancer (basal-cell	Percent	2021	1.79E-06	3.38E-06	7.79E-07
DALYs (Dis	Norway	Male	55+ years	Non-melanoma skin cancer (basal-cell	Rate	2021	0.1147317	0.221650758	0.047830756
DALYs (Dis	Norway	Female	55+ years	Non-melanoma skin cancer (basal-cell	Rate	2021	0.0903972	0.174258881	0.036475072
DALYs (Dis	Norway	Both	55+ years	Non-melanoma skin cancer (basal-cell	Rate	2021	0.1021584	0.197635352	0.041629298
DALYs (Dis	Chad	Male	55+ years	Non-melanoma skin cancer (basal-cell	Number	1990	0.0189397	0.03675939	0.007823216
DALYs (Dis	Chad	Female	55+ years	Non-melanoma skin cancer (basal-cell	Number	1990	0.0115464	0.022825919	0.004640801
DALYs (Dis	Chad	Both	55+ years	Non-melanoma skin cancer (basal-cell	Number	1990	0.030486	0.05950924	0.012539736
DALYs (Dis	Chad	Male	55+ years	Non-melanoma skin cancer (basal-cell	Percent	1990	6.23E-08	1.19E-07	2.61E-08
DALYs (Dis	Chad	Female	55+ years	Non-melanoma skin cancer (basal-cell	Percent	1990	4.12E-08	7.93E-08	1.75E-08
DALYs (Dis	Chad	Both	55+ years	Non-melanoma skin cancer (basal-cell	Percent	1990	5.22E-08	9.92E-08	2.22E-08
DALYs (Dis	Chad	Male	55+ years	Non-melanoma skin cancer (basal-cell	Rate	1990	0.0083614	0.016228337	0.003453751

DALYs (Dis	Chad	Female	55+ years	Non-melanoma skin cancer (basal-cell	Rate	1990	0.0048741	0.009635588	0.001959038
DALYs (Dis	Chad	Both	55+ years	Non-melanoma skin cancer (basal-cell	Rate	1990	0.0065787	0.012841723	0.002705997
DALYs (Dis	El Salvado	Male	55+ years	Non-melanoma skin cancer (basal-cell	Number	2021	0.3077697	0.595484986	0.12882799
DALYs (Dis	El Salvado	Female	55+ years	Non-melanoma skin cancer (basal-cell	Number	2021	0.3943168	0.735293859	0.172023014
DALYs (Dis	El Salvado	Both	55+ years	Non-melanoma skin cancer (basal-cell	Number	2021	0.7020865	1.304212648	0.304084597
DALYs (Dis	El Salvado	Male	55+ years	Non-melanoma skin cancer (basal-cell	Percent	2021	6.93E-07	1.31E-06	3.00E-07
DALYs (Dis	El Salvado	Female	55+ years	Non-melanoma skin cancer (basal-cell	Percent	2021	8.81E-07	1.63E-06	3.93E-07
DALYs (Dis	El Salvado	Both	55+ years	Non-melanoma skin cancer (basal-cell	Percent	2021	7.87E-07	1.47E-06	3.43E-07
DALYs (Dis	El Salvado	Male	55+ years	Non-melanoma skin cancer (basal-cell	Rate	2021	0.0721908	0.139677607	0.030218034
DALYs (Dis	El Salvado	Female	55+ years	Non-melanoma skin cancer (basal-cell	Rate	2021	0.0661322	0.123318655	0.02885057
DALYs (Dis	El Salvado	Both	55+ years	Non-melanoma skin cancer (basal-cell	Rate	2021	0.0686581	0.127540963	0.029736901
DALYs (Dis	Mongolia	Male	55+ years	Non-melanoma skin cancer (basal-cell	Number	2021	0.0780197	0.151752674	0.032231133
DALYs (Dis	Mongolia	Female	55+ years	Non-melanoma skin cancer (basal-cell	Number	2021	0.1093036	0.214365709	0.046824429
DALYs (Dis	Mongolia	Both	55+ years	Non-melanoma skin cancer (basal-cell	Number	2021	0.1873234	0.368813654	0.079678441
DALYs (Dis	Mongolia	Male	55+ years	Non-melanoma skin cancer (basal-cell	Percent	2021	3.85E-07	7.47E-07	1.64E-07
DALYs (Dis	Mongolia	Female	55+ years	Non-melanoma skin cancer (basal-cell	Percent	2021	6.51E-07	1.26E-06	2.79E-07
DALYs (Dis	Mongolia	Both	55+ years	Non-melanoma skin cancer (basal-cell	Percent	2021	5.05E-07	9.74E-07	2.24E-07
DALYs (Dis	Mongolia	Male	55+ years	Non-melanoma skin cancer (basal-cell	Rate	2021	0.046392	0.090235034	0.019165246
DALYs (Dis	Mongolia	Female	55+ years	Non-melanoma skin cancer (basal-cell	Rate	2021	0.0482297	0.094587921	0.020661072
DALYs (Dis	Mongolia	Both	55+ years	Non-melanoma skin cancer (basal-cell	Rate	2021	0.0474469	0.093416411	0.020181666
DALYs (Dis	Nauru	Male	55+ years	Non-melanoma skin cancer (basal-cell	Number	1990	3.60E-07	9.49E-07	7.32E-08
DALYs (Dis	Nauru	Female	55+ years	Non-melanoma skin cancer (basal-cell	Number	1990	2.92E-07	7.85E-07	6.39E-08
DALYs (Dis	Nauru	Both	55+ years	Non-melanoma skin cancer (basal-cell	Number	1990	6.51E-07	1.74E-06	1.44E-07
DALYs (Dis	Nauru	Male	55+ years	Non-melanoma skin cancer (basal-cell	Percent	1990	5.20E-10	1.40E-09	1.06E-10
DALYs (Dis	Nauru	Female	55+ years	Non-melanoma skin cancer (basal-cell	Percent	1990	7.64E-10	1.87E-09	1.64E-10
DALYs (Dis	Nauru	Both	55+ years	Non-melanoma skin cancer (basal-cell	Percent	1990	6.06E-10	1.58E-09	1.33E-10
DALYs (Dis	Nauru	Male	55+ years	Non-melanoma skin cancer (basal-cell	Rate	1990	8.77E-05	0.00023118	1.78E-05
DALYs (Dis	Nauru	Female	55+ years	Non-melanoma skin cancer (basal-cell	Rate	1990	9.34E-05	0.000251373	2.05E-05
DALYs (Dis	Nauru	Both	55+ years	Non-melanoma skin cancer (basal-cell	Rate	1990	9.01E-05	0.000240252	1.99E-05
DALYs (Dis	Russian Fe	Male	55+ years	Non-melanoma skin cancer (basal-cell	Number	2021	7.3625467	13.8661294	3.161040195
DALYs (Dis	Russian Fe	Female	55+ years	Non-melanoma skin cancer (basal-cell	Number	2021	12.958315	25.01246204	5.405844311
DALYs (Dis	Russian Fe	Both	55+ years	Non-melanoma skin cancer (basal-cell	Number	2021	20.320862	38.66513547	8.507263689
DALYs (Dis	Russian Fe	Male	55+ years	Non-melanoma skin cancer (basal-cell	Percent	2021	3.26E-07	6.04E-07	1.43E-07
DALYs (Dis	Russian Fe	Female	55+ years	Non-melanoma skin cancer (basal-cell	Percent	2021	5.09E-07	9.59E-07	2.17E-07
DALYs (Dis	Russian Fe	Both	55+ years	Non-melanoma skin cancer (basal-cell	Percent	2021	4.23E-07	7.89E-07	1.83E-07
DALYs (Dis	Russian Fe	Male	55+ years	Non-melanoma skin cancer (basal-cell	Rate	2021	0.0449573	0.084669625	0.019302004
DALYs (Dis	Russian Fe	Female	55+ years	Non-melanoma skin cancer (basal-cell	Rate	2021	0.0495946	0.095728746	0.020689475
DALYs (Dis	Russian Fe	Both	55+ years	Non-melanoma skin cancer (basal-cell	Rate	2021	0.0478079	0.090965613	0.020014632
DALYs (Dis	Comoros	Male	55+ years	Non-melanoma skin cancer (basal-cell	Number	1990	0.0016876	0.003221172	0.000711408
DALYs (Dis	Comoros	Female	55+ years	Non-melanoma skin cancer (basal-cell	Number	1990	0.0010565	0.002067566	0.000435667
DALYs (Dis	Comoros	Both	55+ years	Non-melanoma skin cancer (basal-cell	Number	1990	0.0027441	0.005334203	0.001155437
DALYs (Dis	Comoros	Male	55+ years	Non-melanoma skin cancer (basal-cell	Percent	1990	8.15E-08	1.56E-07	3.43E-08
DALYs (Dis	Comoros	Female	55+ years	Non-melanoma skin cancer (basal-cell	Percent	1990	5.66E-08	1.09E-07	2.39E-08
DALYs (Dis	Comoros	Both	55+ years	Non-melanoma skin cancer (basal-cell	Percent	1990	6.97E-08	1.35E-07	2.94E-08
DALYs (Dis	Comoros	Male	55+ years	Non-melanoma skin cancer (basal-cell	Rate	1990	0.0105238	0.020087068	0.004436302
DALYs (Dis	Comoros	Female	55+ years	Non-melanoma skin cancer (basal-cell	Rate	1990	0.006435	0.012592904	0.00265351
DALYs (Dis	Comoros	Both	55+ years	Non-melanoma skin cancer (basal-cell	Rate	1990	0.0084553	0.016435917	0.00356017
DALYs (Dis	Nicaragua	Male	55+ years	Non-melanoma skin cancer (basal-cell	Number	1990	0.0767836	0.15725145	0.03288174
DALYs (Dis	Nicaragua	Female	55+ years	Non-melanoma skin cancer (basal-cell	Number	1990	0.0836149	0.162149778	0.033594003
DALYs (Dis	Nicaragua	Both	55+ years	Non-melanoma skin cancer (basal-cell	Number	1990	0.1603985	0.321119051	0.066180636
DALYs (Dis	Nicaragua	Male	55+ years	Non-melanoma skin cancer (basal-cell	Percent	1990	9.80E-07	1.89E-06	4.24E-07
DALYs (Dis	Nicaragua	Female	55+ years	Non-melanoma skin cancer (basal-cell	Percent	1990	1.14E-06	2.15E-06	4.90E-07
DALYs (Dis	Nicaragua	Both	55+ years	Non-melanoma skin cancer (basal-cell	Percent	1990	1.06E-06	1.96E-06	4.73E-07
DALYs (Dis	Nicaragua	Male	55+ years	Non-melanoma skin cancer (basal-cell	Rate	1990	0.0655766	0.134299749	0.028082472
DALYs (Dis	Nicaragua	Female	55+ years	Non-melanoma skin cancer (basal-cell	Rate	1990	0.0636413	0.123416049	0.025569194
DALYs (Dis	Nicaragua	Both	55+ years	Non-melanoma skin cancer (basal-cell	Rate	1990	0.0645533	0.129236177	0.026634771

DALYs (Dis	Spain	Male	55+ years	Non-melanoma skin cancer (basal-cell	Number	1990	6.395449	13.03695434	2.714599473
DALYs (Dis	Spain	Female	55+ years	Non-melanoma skin cancer (basal-cell	Number	1990	6.3156463	13.92619665	2.653218186
DALYs (Dis	Spain	Both	55+ years	Non-melanoma skin cancer (basal-cell	Number	1990	12.711095	27.56719243	5.556195326
DALYs (Dis	Spain	Male	55+ years	Non-melanoma skin cancer (basal-cell	Percent	1990	1.80E-06	3.63E-06	7.88E-07
DALYs (Dis	Spain	Female	55+ years	Non-melanoma skin cancer (basal-cell	Percent	1990	1.90E-06	4.01E-06	8.20E-07
DALYs (Dis	Spain	Both	55+ years	Non-melanoma skin cancer (basal-cell	Percent	1990	1.85E-06	3.82E-06	8.57E-07
DALYs (Dis	Spain	Male	55+ years	Non-melanoma skin cancer (basal-cell	Rate	1990	0.1513838	0.308591787	0.064256043
DALYs (Dis	Spain	Female	55+ years	Non-melanoma skin cancer (basal-cell	Rate	1990	0.1183606	0.2609887	0.049723552
DALYs (Dis	Spain	Both	55+ years	Non-melanoma skin cancer (basal-cell	Rate	1990	0.1329529	0.288341695	0.058115558
DALYs (Dis	Portugal	Male	55+ years	Non-melanoma skin cancer (basal-cell	Number	2021	1.3667852	2.798046016	0.494809939
DALYs (Dis	Portugal	Female	55+ years	Non-melanoma skin cancer (basal-cell	Number	2021	1.3685004	2.652747636	0.559751907
DALYs (Dis	Portugal	Both	55+ years	Non-melanoma skin cancer (basal-cell	Number	2021	2.7352855	5.405038171	1.080652131
DALYs (Dis	Portugal	Male	55+ years	Non-melanoma skin cancer (basal-cell	Percent	2021	1.07E-06	2.14E-06	4.03E-07
DALYs (Dis	Portugal	Female	55+ years	Non-melanoma skin cancer (basal-cell	Percent	2021	1.08E-06	2.03E-06	4.68E-07
DALYs (Dis	Portugal	Both	55+ years	Non-melanoma skin cancer (basal-cell	Percent	2021	1.07E-06	2.10E-06	4.41E-07
DALYs (Dis	Portugal	Male	55+ years	Non-melanoma skin cancer (basal-cell	Rate	2021	0.079422	0.162590577	0.02875272
DALYs (Dis	Portugal	Female	55+ years	Non-melanoma skin cancer (basal-cell	Rate	2021	0.0630724	0.122261735	0.025798247
DALYs (Dis	Portugal	Both	55+ years	Non-melanoma skin cancer (basal-cell	Rate	2021	0.0703042	0.138924009	0.027775664
DALYs (Dis	Jamaica	Male	55+ years	Non-melanoma skin cancer (basal-cell	Number	2021	0.0430501	0.084085441	0.017469901
DALYs (Dis	Jamaica	Female	55+ years	Non-melanoma skin cancer (basal-cell	Number	2021	0.0273463	0.051808584	0.0115515
DALYs (Dis	Jamaica	Both	55+ years	Non-melanoma skin cancer (basal-cell	Number	2021	0.0703964	0.135007478	0.029145514
DALYs (Dis	Jamaica	Male	55+ years	Non-melanoma skin cancer (basal-cell	Percent	2021	1.81E-07	3.41E-07	7.33E-08
DALYs (Dis	Jamaica	Female	55+ years	Non-melanoma skin cancer (basal-cell	Percent	2021	1.30E-07	2.43E-07	5.68E-08
DALYs (Dis	Jamaica	Both	55+ years	Non-melanoma skin cancer (basal-cell	Percent	2021	1.57E-07	2.99E-07	6.63E-08
DALYs (Dis	Jamaica	Male	55+ years	Non-melanoma skin cancer (basal-cell	Rate	2021	0.016849	0.032909527	0.006837405
DALYs (Dis	Jamaica	Female	55+ years	Non-melanoma skin cancer (basal-cell	Rate	2021	0.0100028	0.01895063	0.004225327
DALYs (Dis	Jamaica	Both	55+ years	Non-melanoma skin cancer (basal-cell	Rate	2021	0.0133102	0.025526474	0.005510674
DALYs (Dis	Brunei Dar	Male	55+ years	Non-melanoma skin cancer (basal-cell	Number	2021	0.0033997	0.006767371	0.001370288
DALYs (Dis	Brunei Dar	Female	55+ years	Non-melanoma skin cancer (basal-cell	Number	2021	0.002976	0.005900643	0.001192218
DALYs (Dis	Brunei Dar	Both	55+ years	Non-melanoma skin cancer (basal-cell	Number	2021	0.0063758	0.012277793	0.002501191
DALYs (Dis	Brunei Dar	Male	55+ years	Non-melanoma skin cancer (basal-cell	Percent	2021	1.68E-07	3.18E-07	7.00E-08
DALYs (Dis	Brunei Dar	Female	55+ years	Non-melanoma skin cancer (basal-cell	Percent	2021	1.63E-07	3.19E-07	7.04E-08
DALYs (Dis	Brunei Dar	Both	55+ years	Non-melanoma skin cancer (basal-cell	Percent	2021	1.66E-07	3.11E-07	7.14E-08
DALYs (Dis	Brunei Dar	Male	55+ years	Non-melanoma skin cancer (basal-cell	Rate	2021	0.0115551	0.023000994	0.004657347
DALYs (Dis	Brunei Dar	Female	55+ years	Non-melanoma skin cancer (basal-cell	Rate	2021	0.0097727	0.019376537	0.003915005
DALYs (Dis	Brunei Dar	Both	55+ years	Non-melanoma skin cancer (basal-cell	Rate	2021	0.0106486	0.020505848	0.004177383
DALYs (Dis	Algeria	Male	55+ years	Non-melanoma skin cancer (basal-cell	Number	1990	0.2128012	0.418595714	0.095899967
DALYs (Dis	Algeria	Female	55+ years	Non-melanoma skin cancer (basal-cell	Number	1990	0.1294539	0.250002372	0.056926497
DALYs (Dis	Algeria	Both	55+ years	Non-melanoma skin cancer (basal-cell	Number	1990	0.342255	0.671137397	0.155543083
DALYs (Dis	Algeria	Male	55+ years	Non-melanoma skin cancer (basal-cell	Percent	1990	2.62E-07	5.15E-07	1.20E-07
DALYs (Dis	Algeria	Female	55+ years	Non-melanoma skin cancer (basal-cell	Percent	1990	1.77E-07	3.36E-07	8.13E-08
DALYs (Dis	Algeria	Both	55+ years	Non-melanoma skin cancer (basal-cell	Percent	1990	2.22E-07	4.25E-07	1.03E-07
DALYs (Dis	Algeria	Male	55+ years	Non-melanoma skin cancer (basal-cell	Rate	1990	0.0207701	0.040856314	0.009360151
DALYs (Dis	Algeria	Female	55+ years	Non-melanoma skin cancer (basal-cell	Rate	1990	0.0120931	0.023354321	0.005317868
DALYs (Dis	Algeria	Both	55+ years	Non-melanoma skin cancer (basal-cell	Rate	1990	0.0163365	0.032034715	0.007424379
DALYs (Dis	Türkiye	Male	55+ years	Non-melanoma skin cancer (basal-cell	Number	1990	0.9635706	1.96381096	0.432703949
DALYs (Dis	Türkiye	Female	55+ years	Non-melanoma skin cancer (basal-cell	Number	1990	0.9573069	1.892884368	0.435044551
DALYs (Dis	Türkiye	Both	55+ years	Non-melanoma skin cancer (basal-cell	Number	1990	1.9208775	3.946401373	0.86935855
DALYs (Dis	Türkiye	Male	55+ years	Non-melanoma skin cancer (basal-cell	Percent	1990	3.20E-07	6.10E-07	1.45E-07
DALYs (Dis	Türkiye	Female	55+ years	Non-melanoma skin cancer (basal-cell	Percent	1990	4.16E-07	7.96E-07	1.94E-07
DALYs (Dis	Türkiye	Both	55+ years	Non-melanoma skin cancer (basal-cell	Percent	1990	3.61E-07	6.88E-07	1.66E-07
DALYs (Dis	Türkiye	Male	55+ years	Non-melanoma skin cancer (basal-cell	Rate	1990	0.0338326	0.068952665	0.015192954
DALYs (Dis	Türkiye	Female	55+ years	Non-melanoma skin cancer (basal-cell	Rate	1990	0.0306195	0.06054389	0.013914896
DALYs (Dis	Türkiye	Both	55+ years	Non-melanoma skin cancer (basal-cell	Rate	1990	0.0321511	0.066053832	0.014551096
DALYs (Dis	Trinidad a	Male	55+ years	Non-melanoma skin cancer (basal-cell	Number	1990	0.0075879	0.014816977	0.00334716
DALYs (Dis	Trinidad a	Female	55+ years	Non-melanoma skin cancer (basal-cell	Number	1990	0.0045141	0.008601493	0.001874868

DALYs (Dis	Trinidad a	Both	55+ years	Non-melanoma skin cancer (basal-cell	Number	1990	0.012102	0.023752819	0.005194798
DALYs (Dis	Trinidad a	Male	55+ years	Non-melanoma skin cancer (basal-cell	Percent	1990	1.02E-07	1.96E-07	4.65E-08
DALYs (Dis	Trinidad a	Female	55+ years	Non-melanoma skin cancer (basal-cell	Percent	1990	6.69E-08	1.29E-07	2.90E-08
DALYs (Dis	Trinidad a	Both	55+ years	Non-melanoma skin cancer (basal-cell	Percent	1990	8.55E-08	1.67E-07	3.81E-08
DALYs (Dis	Trinidad a	Male	55+ years	Non-melanoma skin cancer (basal-cell	Rate	1990	0.011623	0.02269631	0.005127104
DALYs (Dis	Trinidad a	Female	55+ years	Non-melanoma skin cancer (basal-cell	Rate	1990	0.0062682	0.011943823	0.002603395
DALYs (Dis	Trinidad a	Both	55+ years	Non-melanoma skin cancer (basal-cell	Rate	1990	0.0088143	0.017299956	0.003783542
DALYs (Dis	Saint Luci	Male	55+ years	Non-melanoma skin cancer (basal-cell	Number	1990	0.0009988	0.001942078	0.00039937
DALYs (Dis	Saint Luci	Female	55+ years	Non-melanoma skin cancer (basal-cell	Number	1990	0.0005244	0.001005252	0.000217347
DALYs (Dis	Saint Luci	Both	55+ years	Non-melanoma skin cancer (basal-cell	Number	1990	0.0015231	0.002976869	0.000605563
DALYs (Dis	Saint Luci	Male	55+ years	Non-melanoma skin cancer (basal-cell	Percent	1990	1.36E-07	2.58E-07	5.62E-08
DALYs (Dis	Saint Luci	Female	55+ years	Non-melanoma skin cancer (basal-cell	Percent	1990	6.99E-08	1.30E-07	3.02E-08
DALYs (Dis	Saint Luci	Both	55+ years	Non-melanoma skin cancer (basal-cell	Percent	1990	1.03E-07	1.93E-07	4.23E-08
DALYs (Dis	Saint Luci	Male	55+ years	Non-melanoma skin cancer (basal-cell	Rate	1990	0.015513	0.030164792	0.006203098
DALYs (Dis	Saint Luci	Female	55+ years	Non-melanoma skin cancer (basal-cell	Rate	1990	0.0064013	0.012272077	0.002653365
DALYs (Dis	Saint Luci	Both	55+ years	Non-melanoma skin cancer (basal-cell	Rate	1990	0.0104112	0.020348259	0.004139298
DALYs (Dis	Burundi	Male	55+ years	Non-melanoma skin cancer (basal-cell	Number	2021	0.0396017	0.081707977	0.016193033
DALYs (Dis	Burundi	Female	55+ years	Non-melanoma skin cancer (basal-cell	Number	2021	0.0226401	0.045032285	0.009137997
DALYs (Dis	Burundi	Both	55+ years	Non-melanoma skin cancer (basal-cell	Number	2021	0.0622418	0.126028636	0.025378017
DALYs (Dis	Burundi	Male	55+ years	Non-melanoma skin cancer (basal-cell	Percent	2021	6.60E-08	1.29E-07	2.75E-08
DALYs (Dis	Burundi	Female	55+ years	Non-melanoma skin cancer (basal-cell	Percent	2021	5.49E-08	1.05E-07	2.34E-08
DALYs (Dis	Burundi	Both	55+ years	Non-melanoma skin cancer (basal-cell	Percent	2021	6.15E-08	1.19E-07	2.58E-08
DALYs (Dis	Burundi	Male	55+ years	Non-melanoma skin cancer (basal-cell	Rate	2021	0.0093675	0.01932752	0.003830362
DALYs (Dis	Burundi	Female	55+ years	Non-melanoma skin cancer (basal-cell	Rate	2021	0.005993	0.01192044	0.002418908
DALYs (Dis	Burundi	Both	55+ years	Non-melanoma skin cancer (basal-cell	Rate	2021	0.0077751	0.015743184	0.003170159
DALYs (Dis	Haiti	Male	55+ years	Non-melanoma skin cancer (basal-cell	Number	2021	0.0694569	0.135438445	0.028478146
DALYs (Dis	Haiti	Female	55+ years	Non-melanoma skin cancer (basal-cell	Number	2021	0.025413	0.050945152	0.01010288
DALYs (Dis	Haiti	Both	55+ years	Non-melanoma skin cancer (basal-cell	Number	2021	0.0948699	0.186665825	0.038501639
DALYs (Dis	Haiti	Male	55+ years	Non-melanoma skin cancer (basal-cell	Percent	2021	8.07E-08	1.57E-07	3.38E-08
DALYs (Dis	Haiti	Female	55+ years	Non-melanoma skin cancer (basal-cell	Percent	2021	3.13E-08	6.13E-08	1.23E-08
DALYs (Dis	Haiti	Both	55+ years	Non-melanoma skin cancer (basal-cell	Percent	2021	5.67E-08	1.10E-07	2.36E-08
DALYs (Dis	Haiti	Male	55+ years	Non-melanoma skin cancer (basal-cell	Rate	2021	0.0123461	0.02407437	0.00506203
DALYs (Dis	Haiti	Female	55+ years	Non-melanoma skin cancer (basal-cell	Rate	2021	0.0040824	0.008183898	0.00162294
DALYs (Dis	Haiti	Both	55+ years	Non-melanoma skin cancer (basal-cell	Rate	2021	0.0080053	0.015751217	0.003248841
DALYs (Dis	Croatia	Male	55+ years	Non-melanoma skin cancer (basal-cell	Number	1990	0.2505136	0.475467975	0.102308363
DALYs (Dis	Croatia	Female	55+ years	Non-melanoma skin cancer (basal-cell	Number	1990	0.36271	0.718999951	0.149210021
DALYs (Dis	Croatia	Both	55+ years	Non-melanoma skin cancer (basal-cell	Number	1990	0.6132236	1.193263911	0.246172873
DALYs (Dis	Croatia	Male	55+ years	Non-melanoma skin cancer (basal-cell	Percent	1990	4.57E-07	8.64E-07	1.91E-07
DALYs (Dis	Croatia	Female	55+ years	Non-melanoma skin cancer (basal-cell	Percent	1990	6.83E-07	1.32E-06	2.90E-07
DALYs (Dis	Croatia	Both	55+ years	Non-melanoma skin cancer (basal-cell	Percent	1990	5.68E-07	1.09E-06	2.45E-07
DALYs (Dis	Croatia	Male	55+ years	Non-melanoma skin cancer (basal-cell	Rate	1990	0.054571	0.10357422	0.022286483
DALYs (Dis	Croatia	Female	55+ years	Non-melanoma skin cancer (basal-cell	Rate	1990	0.0554499	0.109918383	0.022810745
DALYs (Dis	Croatia	Both	55+ years	Non-melanoma skin cancer (basal-cell	Rate	1990	0.0550875	0.107193981	0.022114345
DALYs (Dis	Sudan	Male	55+ years	Non-melanoma skin cancer (basal-cell	Number	2021	0.2438281	0.483185912	0.10292663
DALYs (Dis	Sudan	Female	55+ years	Non-melanoma skin cancer (basal-cell	Number	2021	0.1146935	0.21640245	0.046836741
DALYs (Dis	Sudan	Both	55+ years	Non-melanoma skin cancer (basal-cell	Number	2021	0.3585216	0.701114797	0.151559261
DALYs (Dis	Sudan	Male	55+ years	Non-melanoma skin cancer (basal-cell	Percent	2021	1.24E-07	2.43E-07	5.12E-08
DALYs (Dis	Sudan	Female	55+ years	Non-melanoma skin cancer (basal-cell	Percent	2021	8.70E-08	1.58E-07	3.59E-08
DALYs (Dis	Sudan	Both	55+ years	Non-melanoma skin cancer (basal-cell	Percent	2021	1.09E-07	2.10E-07	4.53E-08
DALYs (Dis	Sudan	Male	55+ years	Non-melanoma skin cancer (basal-cell	Rate	2021	0.0142484	0.028235551	0.006014642
DALYs (Dis	Sudan	Female	55+ years	Non-melanoma skin cancer (basal-cell	Rate	2021	0.0081774	0.015429023	0.003339358
DALYs (Dis	Sudan	Both	55+ years	Non-melanoma skin cancer (basal-cell	Rate	2021	0.0115138	0.022516117	0.004867286
DALYs (Dis	Qatar	Male	55+ years	Non-melanoma skin cancer (basal-cell	Number	2021	0.0103579	0.021041296	0.004040363
DALYs (Dis	Qatar	Female	55+ years	Non-melanoma skin cancer (basal-cell	Number	2021	0.003079	0.00659775	0.001179616
DALYs (Dis	Qatar	Both	55+ years	Non-melanoma skin cancer (basal-cell	Number	2021	0.0134369	0.02727664	0.005102687
DALYs (Dis	Qatar	Male	55+ years	Non-melanoma skin cancer (basal-cell	Percent	2021	1.80E-07	3.52E-07	7.07E-08

DALYs (Dis	Qatar	Female	55+ years	Non-melanoma skin cancer (basal-cell	Percent	2021	1.09E-07	2.16E-07	4.38E-08
DALYs (Dis	Qatar	Both	55+ years	Non-melanoma skin cancer (basal-cell	Percent	2021	1.57E-07	3.08E-07	6.21E-08
DALYs (Dis	Qatar	Male	55+ years	Non-melanoma skin cancer (basal-cell	Rate	2021	0.0098353	0.019979801	0.003836534
DALYs (Dis	Qatar	Female	55+ years	Non-melanoma skin cancer (basal-cell	Rate	2021	0.0064319	0.013782283	0.002464143
DALYs (Dis	Qatar	Both	55+ years	Non-melanoma skin cancer (basal-cell	Rate	2021	0.0087717	0.017806445	0.003331082
DALYs (Dis	Ethiopia	Male	55+ years	Non-melanoma skin cancer (basal-cell	Number	1990	0.1717813	0.343883283	0.072736795
DALYs (Dis	Ethiopia	Female	55+ years	Non-melanoma skin cancer (basal-cell	Number	1990	0.0925692	0.184364275	0.037456156
DALYs (Dis	Ethiopia	Both	55+ years	Non-melanoma skin cancer (basal-cell	Number	1990	0.2643505	0.528750289	0.111218631
DALYs (Dis	Ethiopia	Male	55+ years	Non-melanoma skin cancer (basal-cell	Percent	1990	5.00E-08	9.89E-08	2.06E-08
DALYs (Dis	Ethiopia	Female	55+ years	Non-melanoma skin cancer (basal-cell	Percent	1990	3.74E-08	7.27E-08	1.53E-08
DALYs (Dis	Ethiopia	Both	55+ years	Non-melanoma skin cancer (basal-cell	Percent	1990	4.47E-08	8.81E-08	1.85E-08
DALYs (Dis	Ethiopia	Male	55+ years	Non-melanoma skin cancer (basal-cell	Rate	1990	0.0100492	0.020117135	0.004255095
DALYs (Dis	Ethiopia	Female	55+ years	Non-melanoma skin cancer (basal-cell	Rate	1990	0.0061566	0.012261713	0.002491137
DALYs (Dis	Ethiopia	Both	55+ years	Non-melanoma skin cancer (basal-cell	Rate	1990	0.0082276	0.016456686	0.00346154
DALYs (Dis	Italy	Male	55+ years	Non-melanoma skin cancer (basal-cell	Number	2021	14.277989	27.83065965	5.791088553
DALYs (Dis	Italy	Female	55+ years	Non-melanoma skin cancer (basal-cell	Number	2021	13.045641	25.04880116	5.583085511
DALYs (Dis	Italy	Both	55+ years	Non-melanoma skin cancer (basal-cell	Number	2021	27.32363	53.17610414	11.39163778
DALYs (Dis	Italy	Male	55+ years	Non-melanoma skin cancer (basal-cell	Percent	2021	2.02E-06	3.83E-06	8.64E-07
DALYs (Dis	Italy	Female	55+ years	Non-melanoma skin cancer (basal-cell	Percent	2021	1.77E-06	3.34E-06	7.97E-07
DALYs (Dis	Italy	Both	55+ years	Non-melanoma skin cancer (basal-cell	Percent	2021	1.90E-06	3.62E-06	8.27E-07
DALYs (Dis	Italy	Male	55+ years	Non-melanoma skin cancer (basal-cell	Rate	2021	0.1381693	0.269319555	0.056040835
DALYs (Dis	Italy	Female	55+ years	Non-melanoma skin cancer (basal-cell	Rate	2021	0.1053504	0.202282156	0.045086332
DALYs (Dis	Italy	Both	55+ years	Non-melanoma skin cancer (basal-cell	Rate	2021	0.1202794	0.234082784	0.050146327
DALYs (Dis	Barbados	Male	55+ years	Non-melanoma skin cancer (basal-cell	Number	1990	0.0034575	0.00663249	0.001356063
DALYs (Dis	Barbados	Female	55+ years	Non-melanoma skin cancer (basal-cell	Number	1990	0.0018869	0.003691983	0.00075922
DALYs (Dis	Barbados	Both	55+ years	Non-melanoma skin cancer (basal-cell	Number	1990	0.0053444	0.01036415	0.002147007
DALYs (Dis	Barbados	Male	55+ years	Non-melanoma skin cancer (basal-cell	Percent	1990	1.82E-07	3.46E-07	7.70E-08
DALYs (Dis	Barbados	Female	55+ years	Non-melanoma skin cancer (basal-cell	Percent	1990	8.54E-08	1.63E-07	3.57E-08
DALYs (Dis	Barbados	Both	55+ years	Non-melanoma skin cancer (basal-cell	Percent	1990	1.30E-07	2.46E-07	5.51E-08
DALYs (Dis	Barbados	Male	55+ years	Non-melanoma skin cancer (basal-cell	Rate	1990	0.0175984	0.033758614	0.006902206
DALYs (Dis	Barbados	Female	55+ years	Non-melanoma skin cancer (basal-cell	Rate	1990	0.0069275	0.013554524	0.002787353
DALYs (Dis	Barbados	Both	55+ years	Non-melanoma skin cancer (basal-cell	Rate	1990	0.011399	0.022105556	0.004579322
DALYs (Dis	Canada	Male	55+ years	Non-melanoma skin cancer (basal-cell	Number	2021	5.470365	11.50784277	2.096900349
DALYs (Dis	Canada	Female	55+ years	Non-melanoma skin cancer (basal-cell	Number	2021	6.0909431	12.79248523	2.393427587
DALYs (Dis	Canada	Both	55+ years	Non-melanoma skin cancer (basal-cell	Number	2021	11.561308	23.23738377	4.72326091
DALYs (Dis	Canada	Male	55+ years	Non-melanoma skin cancer (basal-cell	Percent	2021	1.50E-06	3.16E-06	6.02E-07
DALYs (Dis	Canada	Female	55+ years	Non-melanoma skin cancer (basal-cell	Percent	2021	1.74E-06	3.44E-06	7.03E-07
DALYs (Dis	Canada	Both	55+ years	Non-melanoma skin cancer (basal-cell	Percent	2021	1.62E-06	3.15E-06	6.70E-07
DALYs (Dis	Canada	Male	55+ years	Non-melanoma skin cancer (basal-cell	Rate	2021	0.0943028	0.198381961	0.036148148
DALYs (Dis	Canada	Female	55+ years	Non-melanoma skin cancer (basal-cell	Rate	2021	0.0941365	0.197709816	0.036990789
DALYs (Dis	Canada	Both	55+ years	Non-melanoma skin cancer (basal-cell	Rate	2021	0.0942151	0.189365438	0.038490666
DALYs (Dis	Guyana	Male	55+ years	Non-melanoma skin cancer (basal-cell	Number	2021	0.0063828	0.012394431	0.002663093
DALYs (Dis	Guyana	Female	55+ years	Non-melanoma skin cancer (basal-cell	Number	2021	0.0027531	0.00551545	0.00110714
DALYs (Dis	Guyana	Both	55+ years	Non-melanoma skin cancer (basal-cell	Number	2021	0.0091359	0.017696731	0.003786116
DALYs (Dis	Guyana	Male	55+ years	Non-melanoma skin cancer (basal-cell	Percent	2021	7.72E-08	1.55E-07	3.02E-08
DALYs (Dis	Guyana	Female	55+ years	Non-melanoma skin cancer (basal-cell	Percent	2021	4.15E-08	8.01E-08	1.67E-08
DALYs (Dis	Guyana	Both	55+ years	Non-melanoma skin cancer (basal-cell	Percent	2021	6.13E-08	1.21E-07	2.37E-08
DALYs (Dis	Guyana	Male	55+ years	Non-melanoma skin cancer (basal-cell	Rate	2021	0.012124	0.023543072	0.005058514
DALYs (Dis	Guyana	Female	55+ years	Non-melanoma skin cancer (basal-cell	Rate	2021	0.0045907	0.009196758	0.001846105
DALYs (Dis	Guyana	Both	55+ years	Non-melanoma skin cancer (basal-cell	Rate	2021	0.0081124	0.015714023	0.003361926
DALYs (Dis	Estonia	Male	55+ years	Non-melanoma skin cancer (basal-cell	Number	1990	0.0743283	0.152726611	0.031349475
DALYs (Dis	Estonia	Female	55+ years	Non-melanoma skin cancer (basal-cell	Number	1990	0.1510064	0.329378731	0.062468762
DALYs (Dis	Estonia	Both	55+ years	Non-melanoma skin cancer (basal-cell	Number	1990	0.2253346	0.477936236	0.096434253
DALYs (Dis	Estonia	Male	55+ years	Non-melanoma skin cancer (basal-cell	Percent	1990	4.39E-07	8.88E-07	1.94E-07
DALYs (Dis	Estonia	Female	55+ years	Non-melanoma skin cancer (basal-cell	Percent	1990	7.52E-07	1.57E-06	3.27E-07
DALYs (Dis	Estonia	Both	55+ years	Non-melanoma skin cancer (basal-cell	Percent	1990	6.09E-07	1.25E-06	2.69E-07

DALYs (Dis	Estonia	Male	55+ years	Non-melanoma skin cancer (basal-cell	Rate	1990	0.057049	0.117221913	0.024061592
DALYs (Dis	Estonia	Female	55+ years	Non-melanoma skin cancer (basal-cell	Rate	1990	0.0661776	0.144348093	0.02737653
DALYs (Dis	Estonia	Both	55+ years	Non-melanoma skin cancer (basal-cell	Rate	1990	0.0628597	0.133325928	0.026901468
DALYs (Dis	Oman	Male	55+ years	Non-melanoma skin cancer (basal-cell	Number	2021	0.0214879	0.041127899	0.009470818
DALYs (Dis	Oman	Female	55+ years	Non-melanoma skin cancer (basal-cell	Number	2021	0.0143863	0.0284063	0.00653417
DALYs (Dis	Oman	Both	55+ years	Non-melanoma skin cancer (basal-cell	Number	2021	0.0358742	0.069093626	0.015923451
DALYs (Dis	Oman	Male	55+ years	Non-melanoma skin cancer (basal-cell	Percent	2021	1.27E-07	2.37E-07	5.82E-08
DALYs (Dis	Oman	Female	55+ years	Non-melanoma skin cancer (basal-cell	Percent	2021	1.37E-07	2.54E-07	6.37E-08
DALYs (Dis	Oman	Both	55+ years	Non-melanoma skin cancer (basal-cell	Percent	2021	1.30E-07	2.47E-07	6.13E-08
DALYs (Dis	Oman	Male	55+ years	Non-melanoma skin cancer (basal-cell	Rate	2021	0.0122182	0.023385705	0.005385195
DALYs (Dis	Oman	Female	55+ years	Non-melanoma skin cancer (basal-cell	Rate	2021	0.0105405	0.020812478	0.004787398
DALYs (Dis	Oman	Both	55+ years	Non-melanoma skin cancer (basal-cell	Rate	2021	0.0114851	0.022120255	0.005097877
DALYs (Dis	Latvia	Male	55+ years	Non-melanoma skin cancer (basal-cell	Number	1990	0.10757	0.210951319	0.047575077
DALYs (Dis	Latvia	Female	55+ years	Non-melanoma skin cancer (basal-cell	Number	1990	0.2052759	0.407301551	0.091238017
DALYs (Dis	Latvia	Both	55+ years	Non-melanoma skin cancer (basal-cell	Number	1990	0.3128458	0.619759131	0.140155288
DALYs (Dis	Latvia	Male	55+ years	Non-melanoma skin cancer (basal-cell	Percent	1990	3.65E-07	6.94E-07	1.63E-07
DALYs (Dis	Latvia	Female	55+ years	Non-melanoma skin cancer (basal-cell	Percent	1990	5.84E-07	1.11E-06	2.69E-07
DALYs (Dis	Latvia	Both	55+ years	Non-melanoma skin cancer (basal-cell	Percent	1990	4.84E-07	9.30E-07	2.21E-07
DALYs (Dis	Latvia	Male	55+ years	Non-melanoma skin cancer (basal-cell	Rate	1990	0.0476207	0.093387171	0.021061266
DALYs (Dis	Latvia	Female	55+ years	Non-melanoma skin cancer (basal-cell	Rate	1990	0.0514026	0.101991402	0.022846693
DALYs (Dis	Latvia	Both	55+ years	Non-melanoma skin cancer (basal-cell	Rate	1990	0.0500363	0.099123734	0.022416314
DALYs (Dis	Colombia	Male	55+ years	Non-melanoma skin cancer (basal-cell	Number	2021	3.0577637	6.242875531	1.237399673
DALYs (Dis	Colombia	Female	55+ years	Non-melanoma skin cancer (basal-cell	Number	2021	3.4414106	6.502543621	1.482109179
DALYs (Dis	Colombia	Both	55+ years	Non-melanoma skin cancer (basal-cell	Number	2021	6.4991743	12.61524003	2.713113639
DALYs (Dis	Colombia	Male	55+ years	Non-melanoma skin cancer (basal-cell	Percent	2021	8.45E-07	1.72E-06	3.61E-07
DALYs (Dis	Colombia	Female	55+ years	Non-melanoma skin cancer (basal-cell	Percent	2021	1.05E-06	1.93E-06	4.60E-07
DALYs (Dis	Colombia	Both	55+ years	Non-melanoma skin cancer (basal-cell	Percent	2021	9.43E-07	1.79E-06	4.09E-07
DALYs (Dis	Colombia	Male	55+ years	Non-melanoma skin cancer (basal-cell	Rate	2021	0.0710332	0.145024664	0.028745323
DALYs (Dis	Colombia	Female	55+ years	Non-melanoma skin cancer (basal-cell	Rate	2021	0.0654409	0.123650619	0.028183389
DALYs (Dis	Colombia	Both	55+ years	Non-melanoma skin cancer (basal-cell	Rate	2021	0.0679581	0.131910241	0.028369454
DALYs (Dis	India	Male	55+ years	Non-melanoma skin cancer (basal-cell	Number	2021	3.4206265	6.694652984	1.397496324
DALYs (Dis	India	Female	55+ years	Non-melanoma skin cancer (basal-cell	Number	2021	3.2615965	6.423874042	1.308458312
DALYs (Dis	India	Both	55+ years	Non-melanoma skin cancer (basal-cell	Number	2021	6.682223	13.01334361	2.722770492
DALYs (Dis	India	Male	55+ years	Non-melanoma skin cancer (basal-cell	Percent	2021	2.90E-08	5.51E-08	1.22E-08
DALYs (Dis	India	Female	55+ years	Non-melanoma skin cancer (basal-cell	Percent	2021	3.23E-08	6.29E-08	1.38E-08
DALYs (Dis	India	Both	55+ years	Non-melanoma skin cancer (basal-cell	Percent	2021	3.05E-08	5.75E-08	1.28E-08
DALYs (Dis	India	Male	55+ years	Non-melanoma skin cancer (basal-cell	Rate	2021	0.003521	0.006891002	0.001438484
DALYs (Dis	India	Female	55+ years	Non-melanoma skin cancer (basal-cell	Rate	2021	0.0031393	0.006183061	0.001259408
DALYs (Dis	India	Both	55+ years	Non-melanoma skin cancer (basal-cell	Rate	2021	0.0033237	0.006472839	0.001354306
DALYs (Dis	Somalia	Male	55+ years	Non-melanoma skin cancer (basal-cell	Number	1990	0.0146397	0.02878194	0.006053352
DALYs (Dis	Somalia	Female	55+ years	Non-melanoma skin cancer (basal-cell	Number	1990	0.0106549	0.021044843	0.004377899
DALYs (Dis	Somalia	Both	55+ years	Non-melanoma skin cancer (basal-cell	Number	1990	0.0252946	0.05011763	0.010516101
DALYs (Dis	Somalia	Male	55+ years	Non-melanoma skin cancer (basal-cell	Percent	1990	4.55E-08	8.65E-08	1.84E-08
DALYs (Dis	Somalia	Female	55+ years	Non-melanoma skin cancer (basal-cell	Percent	1990	3.87E-08	7.40E-08	1.66E-08
DALYs (Dis	Somalia	Both	55+ years	Non-melanoma skin cancer (basal-cell	Percent	1990	4.24E-08	8.20E-08	1.77E-08
DALYs (Dis	Somalia	Male	55+ years	Non-melanoma skin cancer (basal-cell	Rate	1990	0.0086256	0.016958115	0.003566592
DALYs (Dis	Somalia	Female	55+ years	Non-melanoma skin cancer (basal-cell	Rate	1990	0.0055976	0.01105608	0.002299965
DALYs (Dis	Somalia	Both	55+ years	Non-melanoma skin cancer (basal-cell	Rate	1990	0.0070249	0.013918856	0.002920571
DALYs (Dis	India	Male	55+ years	Non-melanoma skin cancer (basal-cell	Number	1990	1.2580024	2.477950217	0.510643953
DALYs (Dis	India	Female	55+ years	Non-melanoma skin cancer (basal-cell	Number	1990	1.0436007	2.080960245	0.41583866
DALYs (Dis	India	Both	55+ years	Non-melanoma skin cancer (basal-cell	Number	1990	2.3016031	4.563870431	0.928578966
DALYs (Dis	India	Male	55+ years	Non-melanoma skin cancer (basal-cell	Percent	1990	2.63E-08	5.15E-08	1.08E-08
DALYs (Dis	India	Female	55+ years	Non-melanoma skin cancer (basal-cell	Percent	1990	2.62E-08	5.23E-08	1.07E-08
DALYs (Dis	India	Both	55+ years	Non-melanoma skin cancer (basal-cell	Percent	1990	2.62E-08	5.15E-08	1.09E-08
DALYs (Dis	India	Male	55+ years	Non-melanoma skin cancer (basal-cell	Rate	1990	0.0031661	0.006236354	0.001285158
DALYs (Dis	India	Female	55+ years	Non-melanoma skin cancer (basal-cell	Rate	1990	0.0028255	0.005634088	0.001125861

DALYs (Dis	India	Both	55+ years	Non-melanoma skin cancer (basal-cell	Rate	1990	0.003002	0.005952683	0.001211151
DALYs (Dis	Palestine	Male	55+ years	Non-melanoma skin cancer (basal-cell	Number	2021	0.0278169	0.053562796	0.011688908
DALYs (Dis	Palestine	Female	55+ years	Non-melanoma skin cancer (basal-cell	Number	2021	0.0188231	0.037177223	0.007824057
DALYs (Dis	Palestine	Both	55+ years	Non-melanoma skin cancer (basal-cell	Number	2021	0.0466399	0.090738923	0.019391822
DALYs (Dis	Palestine	Male	55+ years	Non-melanoma skin cancer (basal-cell	Percent	2021	1.39E-07	2.62E-07	6.12E-08
DALYs (Dis	Palestine	Female	55+ years	Non-melanoma skin cancer (basal-cell	Percent	2021	1.12E-07	2.17E-07	4.76E-08
DALYs (Dis	Palestine	Both	55+ years	Non-melanoma skin cancer (basal-cell	Percent	2021	1.27E-07	2.44E-07	5.60E-08
DALYs (Dis	Palestine	Male	55+ years	Non-melanoma skin cancer (basal-cell	Rate	2021	0.0130869	0.025199538	0.005499248
DALYs (Dis	Palestine	Female	55+ years	Non-melanoma skin cancer (basal-cell	Rate	2021	0.0086461	0.017076819	0.003593867
DALYs (Dis	Palestine	Both	55+ years	Non-melanoma skin cancer (basal-cell	Rate	2021	0.0108399	0.021089301	0.004506996
DALYs (Dis	Senegal	Male	55+ years	Non-melanoma skin cancer (basal-cell	Number	2021	0.0802314	0.158292175	0.033841364
DALYs (Dis	Senegal	Female	55+ years	Non-melanoma skin cancer (basal-cell	Number	2021	0.0558425	0.11186201	0.022380351
DALYs (Dis	Senegal	Both	55+ years	Non-melanoma skin cancer (basal-cell	Number	2021	0.1360739	0.270145029	0.055963383
DALYs (Dis	Senegal	Male	55+ years	Non-melanoma skin cancer (basal-cell	Percent	2021	9.51E-08	1.82E-07	4.14E-08
DALYs (Dis	Senegal	Female	55+ years	Non-melanoma skin cancer (basal-cell	Percent	2021	7.80E-08	1.47E-07	3.44E-08
DALYs (Dis	Senegal	Both	55+ years	Non-melanoma skin cancer (basal-cell	Percent	2021	8.73E-08	1.64E-07	3.80E-08
DALYs (Dis	Senegal	Male	55+ years	Non-melanoma skin cancer (basal-cell	Rate	2021	0.0129017	0.025454287	0.005441885
DALYs (Dis	Senegal	Female	55+ years	Non-melanoma skin cancer (basal-cell	Rate	2021	0.0083435	0.016713358	0.003343859
DALYs (Dis	Senegal	Both	55+ years	Non-melanoma skin cancer (basal-cell	Rate	2021	0.0105388	0.020922572	0.004334331
DALYs (Dis	Chile	Male	55+ years	Non-melanoma skin cancer (basal-cell	Number	2021	1.2409951	2.41687034	0.502867356
DALYs (Dis	Chile	Female	55+ years	Non-melanoma skin cancer (basal-cell	Number	2021	1.3912469	2.735191094	0.57652359
DALYs (Dis	Chile	Both	55+ years	Non-melanoma skin cancer (basal-cell	Number	2021	2.632242	4.99825566	1.076997207
DALYs (Dis	Chile	Male	55+ years	Non-melanoma skin cancer (basal-cell	Percent	2021	8.18E-07	1.51E-06	3.39E-07
DALYs (Dis	Chile	Female	55+ years	Non-melanoma skin cancer (basal-cell	Percent	2021	9.58E-07	1.81E-06	4.15E-07
DALYs (Dis	Chile	Both	55+ years	Non-melanoma skin cancer (basal-cell	Percent	2021	8.86E-07	1.65E-06	3.77E-07
DALYs (Dis	Chile	Male	55+ years	Non-melanoma skin cancer (basal-cell	Rate	2021	0.0611106	0.119014578	0.024762829
DALYs (Dis	Chile	Female	55+ years	Non-melanoma skin cancer (basal-cell	Rate	2021	0.0568564	0.111779697	0.023560925
DALYs (Dis	Chile	Both	55+ years	Non-melanoma skin cancer (basal-cell	Rate	2021	0.0587858	0.111625945	0.024052557
DALYs (Dis	United Rep	Male	55+ years	Non-melanoma skin cancer (basal-cell	Number	1990	0.0928756	0.178984061	0.038860019
DALYs (Dis	United Rep	Female	55+ years	Non-melanoma skin cancer (basal-cell	Number	1990	0.0593341	0.117648056	0.024091428
DALYs (Dis	United Rep	Both	55+ years	Non-melanoma skin cancer (basal-cell	Number	1990	0.1522097	0.298145941	0.062703098
DALYs (Dis	United Rep	Male	55+ years	Non-melanoma skin cancer (basal-cell	Percent	1990	7.88E-08	1.53E-07	3.35E-08
DALYs (Dis	United Rep	Female	55+ years	Non-melanoma skin cancer (basal-cell	Percent	1990	6.00E-08	1.16E-07	2.51E-08
DALYs (Dis	United Rep	Both	55+ years	Non-melanoma skin cancer (basal-cell	Percent	1990	7.02E-08	1.37E-07	2.98E-08
DALYs (Dis	United Rep	Male	55+ years	Non-melanoma skin cancer (basal-cell	Rate	1990	0.0104382	0.020115785	0.004367427
DALYs (Dis	United Rep	Female	55+ years	Non-melanoma skin cancer (basal-cell	Rate	1990	0.0064888	0.012866107	0.002634662
DALYs (Dis	United Rep	Both	55+ years	Non-melanoma skin cancer (basal-cell	Rate	1990	0.0084365	0.01652536	0.00347545
DALYs (Dis	Cambodia	Male	55+ years	Non-melanoma skin cancer (basal-cell	Number	2021	0.0220782	0.043730405	0.008612261
DALYs (Dis	Cambodia	Female	55+ years	Non-melanoma skin cancer (basal-cell	Number	2021	0.0253969	0.047368731	0.010023081
DALYs (Dis	Cambodia	Both	55+ years	Non-melanoma skin cancer (basal-cell	Number	2021	0.0474751	0.092297315	0.01886909
DALYs (Dis	Cambodia	Male	55+ years	Non-melanoma skin cancer (basal-cell	Percent	2021	2.00E-08	3.96E-08	8.10E-09
DALYs (Dis	Cambodia	Female	55+ years	Non-melanoma skin cancer (basal-cell	Percent	2021	2.19E-08	4.04E-08	8.67E-09
DALYs (Dis	Cambodia	Both	55+ years	Non-melanoma skin cancer (basal-cell	Percent	2021	2.09E-08	4.01E-08	8.27E-09
DALYs (Dis	Cambodia	Male	55+ years	Non-melanoma skin cancer (basal-cell	Rate	2021	0.0024222	0.004797641	0.000944847
DALYs (Dis	Cambodia	Female	55+ years	Non-melanoma skin cancer (basal-cell	Rate	2021	0.0020119	0.003752535	0.000794025
DALYs (Dis	Cambodia	Both	55+ years	Non-melanoma skin cancer (basal-cell	Rate	2021	0.002184	0.004245876	0.000868019
DALYs (Dis	Azerbaijan	Male	55+ years	Non-melanoma skin cancer (basal-cell	Number	1990	0.1764672	0.348443594	0.075133635
DALYs (Dis	Azerbaijan	Female	55+ years	Non-melanoma skin cancer (basal-cell	Number	1990	0.2826518	0.541405703	0.113697677
DALYs (Dis	Azerbaijan	Both	55+ years	Non-melanoma skin cancer (basal-cell	Number	1990	0.459119	0.88019963	0.192811436
DALYs (Dis	Azerbaijan	Male	55+ years	Non-melanoma skin cancer (basal-cell	Percent	1990	4.55E-07	8.92E-07	1.93E-07
DALYs (Dis	Azerbaijan	Female	55+ years	Non-melanoma skin cancer (basal-cell	Percent	1990	7.16E-07	1.34E-06	3.08E-07
DALYs (Dis	Azerbaijan	Both	55+ years	Non-melanoma skin cancer (basal-cell	Percent	1990	5.87E-07	1.11E-06	2.58E-07
DALYs (Dis	Azerbaijan	Male	55+ years	Non-melanoma skin cancer (basal-cell	Rate	1990	0.0487246	0.096209295	0.020745263
DALYs (Dis	Azerbaijan	Female	55+ years	Non-melanoma skin cancer (basal-cell	Rate	1990	0.0554186	0.106151697	0.022292342
DALYs (Dis	Azerbaijan	Both	55+ years	Non-melanoma skin cancer (basal-cell	Rate	1990	0.052639	0.100916869	0.022106265
DALYs (Dis	Australia	Male	55+ years	Non-melanoma skin cancer (basal-cell	Number	1990	1.3269894	2.669396701	0.497429991

DALYs (Dis	Australia	Female	55+ years	Non-melanoma skin cancer (basal-cell	Number	1990	1.3706153	2.737393279	0.559089641
DALYs (Dis	Australia	Both	55+ years	Non-melanoma skin cancer (basal-cell	Number	1990	2.6976047	5.174994201	1.116432836
DALYs (Dis	Australia	Male	55+ years	Non-melanoma skin cancer (basal-cell	Percent	1990	1.00E-06	1.96E-06	3.84E-07
DALYs (Dis	Australia	Female	55+ years	Non-melanoma skin cancer (basal-cell	Percent	1990	1.15E-06	2.21E-06	5.03E-07
DALYs (Dis	Australia	Both	55+ years	Non-melanoma skin cancer (basal-cell	Percent	1990	1.07E-06	2.00E-06	4.61E-07
DALYs (Dis	Australia	Male	55+ years	Non-melanoma skin cancer (basal-cell	Rate	1990	0.0880888	0.177201165	0.033020635
DALYs (Dis	Australia	Female	55+ years	Non-melanoma skin cancer (basal-cell	Rate	1990	0.0772128	0.154209364	0.031495971
DALYs (Dis	Australia	Both	55+ years	Non-melanoma skin cancer (basal-cell	Rate	1990	0.0822055	0.157700341	0.03402165
DALYs (Dis	Costa Rica	Male	55+ years	Non-melanoma skin cancer (basal-cell	Number	2021	0.3516701	0.718628586	0.137357007
DALYs (Dis	Costa Rica	Female	55+ years	Non-melanoma skin cancer (basal-cell	Number	2021	0.396544	0.741849126	0.165663694
DALYs (Dis	Costa Rica	Both	55+ years	Non-melanoma skin cancer (basal-cell	Number	2021	0.7482141	1.441655079	0.305844878
DALYs (Dis	Costa Rica	Male	55+ years	Non-melanoma skin cancer (basal-cell	Percent	2021	1.03E-06	2.07E-06	4.28E-07
DALYs (Dis	Costa Rica	Female	55+ years	Non-melanoma skin cancer (basal-cell	Percent	2021	1.31E-06	2.38E-06	5.50E-07
DALYs (Dis	Costa Rica	Both	55+ years	Non-melanoma skin cancer (basal-cell	Percent	2021	1.16E-06	2.18E-06	4.86E-07
DALYs (Dis	Costa Rica	Male	55+ years	Non-melanoma skin cancer (basal-cell	Rate	2021	0.0801243	0.163731901	0.031295337
DALYs (Dis	Costa Rica	Female	55+ years	Non-melanoma skin cancer (basal-cell	Rate	2021	0.0764706	0.143060196	0.031947036
DALYs (Dis	Costa Rica	Both	55+ years	Non-melanoma skin cancer (basal-cell	Rate	2021	0.0781455	0.150570319	0.031943259
DALYs (Dis	Iran (Isla	Male	55+ years	Non-melanoma skin cancer (basal-cell	Number	1990	0.6921365	1.353918492	0.28614054
DALYs (Dis	Iran (Isla	Female	55+ years	Non-melanoma skin cancer (basal-cell	Number	1990	0.4116127	0.79130127	0.170818343
DALYs (Dis	Iran (Isla	Both	55+ years	Non-melanoma skin cancer (basal-cell	Number	1990	1.1037492	2.13415044	0.458418354
DALYs (Dis	Iran (Isla	Male	55+ years	Non-melanoma skin cancer (basal-cell	Percent	1990	3.33E-07	6.42E-07	1.43E-07
DALYs (Dis	Iran (Isla	Female	55+ years	Non-melanoma skin cancer (basal-cell	Percent	1990	2.67E-07	4.99E-07	1.17E-07
DALYs (Dis	Iran (Isla	Both	55+ years	Non-melanoma skin cancer (basal-cell	Percent	1990	3.05E-07	5.78E-07	1.32E-07
DALYs (Dis	Iran (Isla	Male	55+ years	Non-melanoma skin cancer (basal-cell	Rate	1990	0.0280561	0.054881835	0.011598865
DALYs (Dis	Iran (Isla	Female	55+ years	Non-melanoma skin cancer (basal-cell	Rate	1990	0.0192076	0.03692555	0.007971125
DALYs (Dis	Iran (Isla	Both	55+ years	Non-melanoma skin cancer (basal-cell	Rate	1990	0.0239428	0.046294596	0.009944141
DALYs (Dis	South Suda	Male	55+ years	Non-melanoma skin cancer (basal-cell	Number	1990	0.0262144	0.052715954	0.010482485
DALYs (Dis	South Suda	Female	55+ years	Non-melanoma skin cancer (basal-cell	Number	1990	0.0105944	0.021018832	0.004268164
DALYs (Dis	South Suda	Both	55+ years	Non-melanoma skin cancer (basal-cell	Number	1990	0.0368087	0.072740941	0.015124691
DALYs (Dis	South Suda	Male	55+ years	Non-melanoma skin cancer (basal-cell	Percent	1990	6.85E-08	1.30E-07	2.96E-08
DALYs (Dis	South Suda	Female	55+ years	Non-melanoma skin cancer (basal-cell	Percent	1990	5.24E-08	9.99E-08	2.24E-08
DALYs (Dis	South Suda	Both	55+ years	Non-melanoma skin cancer (basal-cell	Percent	1990	6.30E-08	1.19E-07	2.70E-08
DALYs (Dis	South Suda	Male	55+ years	Non-melanoma skin cancer (basal-cell	Rate	1990	0.0104092	0.020932391	0.004162373
DALYs (Dis	South Suda	Female	55+ years	Non-melanoma skin cancer (basal-cell	Rate	1990	0.0065051	0.01290589	0.002620719
DALYs (Dis	South Suda	Both	55+ years	Non-melanoma skin cancer (basal-cell	Rate	1990	0.008876	0.017540555	0.003647127
DALYs (Dis	Fiji	Male	55+ years	Non-melanoma skin cancer (basal-cell	Number	2021	6.21E-05	0.000167131	1.25E-05
DALYs (Dis	Fiji	Female	55+ years	Non-melanoma skin cancer (basal-cell	Number	2021	7.26E-05	0.000190717	1.69E-05
DALYs (Dis	Fiji	Both	55+ years	Non-melanoma skin cancer (basal-cell	Number	2021	0.0001346	0.000358539	3.25E-05
DALYs (Dis	Fiji	Male	55+ years	Non-melanoma skin cancer (basal-cell	Percent	2021	6.43E-10	1.68E-09	1.31E-10
DALYs (Dis	Fiji	Female	55+ years	Non-melanoma skin cancer (basal-cell	Percent	2021	8.83E-10	2.26E-09	2.00E-10
DALYs (Dis	Fiji	Both	55+ years	Non-melanoma skin cancer (basal-cell	Percent	2021	7.53E-10	1.99E-09	1.77E-10
DALYs (Dis	Fiji	Male	55+ years	Non-melanoma skin cancer (basal-cell	Rate	2021	9.59E-05	0.000258057	1.92E-05
DALYs (Dis	Fiji	Female	55+ years	Non-melanoma skin cancer (basal-cell	Rate	2021	0.0001008	0.000264975	2.35E-05
DALYs (Dis	Fiji	Both	55+ years	Non-melanoma skin cancer (basal-cell	Rate	2021	9.85E-05	0.000262204	2.38E-05
DALYs (Dis	Mauritania	Male	55+ years	Non-melanoma skin cancer (basal-cell	Number	2021	0.0163237	0.03254862	0.006540108
DALYs (Dis	Mauritania	Female	55+ years	Non-melanoma skin cancer (basal-cell	Number	2021	0.0087219	0.017240223	0.003543578
DALYs (Dis	Mauritania	Both	55+ years	Non-melanoma skin cancer (basal-cell	Number	2021	0.0250456	0.049369632	0.010213849
DALYs (Dis	Mauritania	Male	55+ years	Non-melanoma skin cancer (basal-cell	Percent	2021	8.87E-08	1.66E-07	3.81E-08
DALYs (Dis	Mauritania	Female	55+ years	Non-melanoma skin cancer (basal-cell	Percent	2021	5.01E-08	9.49E-08	2.16E-08
DALYs (Dis	Mauritania	Both	55+ years	Non-melanoma skin cancer (basal-cell	Percent	2021	6.99E-08	1.32E-07	3.09E-08
DALYs (Dis	Mauritania	Male	55+ years	Non-melanoma skin cancer (basal-cell	Rate	2021	0.009084	0.018113127	0.003639534
DALYs (Dis	Mauritania	Female	55+ years	Non-melanoma skin cancer (basal-cell	Rate	2021	0.0048709	0.009628105	0.001978973
DALYs (Dis	Mauritania	Both	55+ years	Non-melanoma skin cancer (basal-cell	Rate	2021	0.0069812	0.013761273	0.002847005
DALYs (Dis	Nepal	Male	55+ years	Non-melanoma skin cancer (basal-cell	Number	1990	0.0008479	0.002296265	0.000171014
DALYs (Dis	Nepal	Female	55+ years	Non-melanoma skin cancer (basal-cell	Number	1990	0.0005629	0.001413618	0.000130445
DALYs (Dis	Nepal	Both	55+ years	Non-melanoma skin cancer (basal-cell	Number	1990	0.0014108	0.003410853	0.00035643

DALYs (Dis	Nepal	Male	55+ years	Non-melanoma skin cancer (basal-cell	Percent	1990	8.64E-10	2.43E-09	1.72E-10
DALYs (Dis	Nepal	Female	55+ years	Non-melanoma skin cancer (basal-cell	Percent	1990	6.53E-10	1.64E-09	1.49E-10
DALYs (Dis	Nepal	Both	55+ years	Non-melanoma skin cancer (basal-cell	Percent	1990	7.65E-10	1.85E-09	1.91E-10
DALYs (Dis	Nepal	Male	55+ years	Non-melanoma skin cancer (basal-cell	Rate	1990	0.0001063	0.00028789	2.14E-05
DALYs (Dis	Nepal	Female	55+ years	Non-melanoma skin cancer (basal-cell	Rate	1990	7.59E-05	0.000190591	1.76E-05
DALYs (Dis	Nepal	Both	55+ years	Non-melanoma skin cancer (basal-cell	Rate	1990	9.16E-05	0.000221582	2.32E-05
DALYs (Dis	Benin	Male	55+ years	Non-melanoma skin cancer (basal-cell	Number	2021	0.0311367	0.06200272	0.012861285
DALYs (Dis	Benin	Female	55+ years	Non-melanoma skin cancer (basal-cell	Number	2021	0.0220412	0.042508419	0.008693604
DALYs (Dis	Benin	Both	55+ years	Non-melanoma skin cancer (basal-cell	Number	2021	0.0531779	0.103904766	0.021638784
DALYs (Dis	Benin	Male	55+ years	Non-melanoma skin cancer (basal-cell	Percent	2021	5.90E-08	1.12E-07	2.52E-08
DALYs (Dis	Benin	Female	55+ years	Non-melanoma skin cancer (basal-cell	Percent	2021	4.90E-08	9.25E-08	2.02E-08
DALYs (Dis	Benin	Both	55+ years	Non-melanoma skin cancer (basal-cell	Percent	2021	5.44E-08	1.03E-07	2.32E-08
DALYs (Dis	Benin	Male	55+ years	Non-melanoma skin cancer (basal-cell	Rate	2021	0.0079906	0.015911775	0.003300595
DALYs (Dis	Benin	Female	55+ years	Non-melanoma skin cancer (basal-cell	Rate	2021	0.0050392	0.009718515	0.001987581
DALYs (Dis	Benin	Both	55+ years	Non-melanoma skin cancer (basal-cell	Rate	2021	0.0064297	0.012563119	0.002616344
DALYs (Dis	Kiribati	Male	55+ years	Non-melanoma skin cancer (basal-cell	Number	1990	2.34E-06	6.15E-06	4.27E-07
DALYs (Dis	Kiribati	Female	55+ years	Non-melanoma skin cancer (basal-cell	Number	1990	3.05E-06	8.19E-06	7.09E-07
DALYs (Dis	Kiribati	Both	55+ years	Non-melanoma skin cancer (basal-cell	Number	1990	5.39E-06	1.43E-05	1.31E-06
DALYs (Dis	Kiribati	Male	55+ years	Non-melanoma skin cancer (basal-cell	Percent	1990	5.41E-10	1.42E-09	1.00E-10
DALYs (Dis	Kiribati	Female	55+ years	Non-melanoma skin cancer (basal-cell	Percent	1990	7.50E-10	2.02E-09	1.79E-10
DALYs (Dis	Kiribati	Both	55+ years	Non-melanoma skin cancer (basal-cell	Percent	1990	6.43E-10	1.72E-09	1.60E-10
DALYs (Dis	Kiribati	Male	55+ years	Non-melanoma skin cancer (basal-cell	Rate	1990	8.82E-05	0.00023185	1.61E-05
DALYs (Dis	Kiribati	Female	55+ years	Non-melanoma skin cancer (basal-cell	Rate	1990	9.23E-05	0.000247723	2.14E-05
DALYs (Dis	Kiribati	Both	55+ years	Non-melanoma skin cancer (basal-cell	Rate	1990	9.05E-05	0.000240014	2.20E-05
DALYs (Dis	Bhutan	Male	55+ years	Non-melanoma skin cancer (basal-cell	Number	2021	6.17E-05	0.000153808	1.26E-05
DALYs (Dis	Bhutan	Female	55+ years	Non-melanoma skin cancer (basal-cell	Number	2021	3.88E-05	0.000102022	8.64E-06
DALYs (Dis	Bhutan	Both	55+ years	Non-melanoma skin cancer (basal-cell	Number	2021	0.0001005	0.000240006	2.51E-05
DALYs (Dis	Bhutan	Male	55+ years	Non-melanoma skin cancer (basal-cell	Percent	2021	1.48E-09	3.71E-09	3.08E-10
DALYs (Dis	Bhutan	Female	55+ years	Non-melanoma skin cancer (basal-cell	Percent	2021	1.02E-09	2.61E-09	2.27E-10
DALYs (Dis	Bhutan	Both	55+ years	Non-melanoma skin cancer (basal-cell	Percent	2021	1.26E-09	3.06E-09	3.28E-10
DALYs (Dis	Bhutan	Male	55+ years	Non-melanoma skin cancer (basal-cell	Rate	2021	0.0001221	0.000304408	2.50E-05
DALYs (Dis	Bhutan	Female	55+ years	Non-melanoma skin cancer (basal-cell	Rate	2021	7.92E-05	0.000207939	1.76E-05
DALYs (Dis	Bhutan	Both	55+ years	Non-melanoma skin cancer (basal-cell	Rate	2021	0.0001009	0.000240994	2.52E-05
DALYs (Dis	Saudi Arab	Male	55+ years	Non-melanoma skin cancer (basal-cell	Number	1990	0.0672953	0.128746636	0.027947861
DALYs (Dis	Saudi Arab	Female	55+ years	Non-melanoma skin cancer (basal-cell	Number	1990	0.031321	0.059988747	0.012537667
DALYs (Dis	Saudi Arab	Both	55+ years	Non-melanoma skin cancer (basal-cell	Number	1990	0.0986163	0.18899885	0.040403761
DALYs (Dis	Saudi Arab	Male	55+ years	Non-melanoma skin cancer (basal-cell	Percent	1990	1.26E-07	2.40E-07	5.58E-08
DALYs (Dis	Saudi Arab	Female	55+ years	Non-melanoma skin cancer (basal-cell	Percent	1990	8.55E-08	1.58E-07	3.64E-08
DALYs (Dis	Saudi Arab	Both	55+ years	Non-melanoma skin cancer (basal-cell	Percent	1990	1.10E-07	2.11E-07	4.81E-08
DALYs (Dis	Saudi Arab	Male	55+ years	Non-melanoma skin cancer (basal-cell	Rate	1990	0.0128971	0.02467416	0.005356179
DALYs (Dis	Saudi Arab	Female	55+ years	Non-melanoma skin cancer (basal-cell	Rate	1990	0.0084854	0.016251979	0.003396669
DALYs (Dis	Saudi Arab	Both	55+ years	Non-melanoma skin cancer (basal-cell	Rate	1990	0.0110692	0.02121428	0.004535142
DALYs (Dis	Senegal	Male	55+ years	Non-melanoma skin cancer (basal-cell	Number	1990	0.0357092	0.069446657	0.014875512
DALYs (Dis	Senegal	Female	55+ years	Non-melanoma skin cancer (basal-cell	Number	1990	0.0215687	0.04179162	0.008493289
DALYs (Dis	Senegal	Both	55+ years	Non-melanoma skin cancer (basal-cell	Number	1990	0.057278	0.111473522	0.023331383
DALYs (Dis	Senegal	Male	55+ years	Non-melanoma skin cancer (basal-cell	Percent	1990	1.04E-07	2.02E-07	4.38E-08
DALYs (Dis	Senegal	Female	55+ years	Non-melanoma skin cancer (basal-cell	Percent	1990	7.88E-08	1.51E-07	3.15E-08
DALYs (Dis	Senegal	Both	55+ years	Non-melanoma skin cancer (basal-cell	Percent	1990	9.26E-08	1.77E-07	3.88E-08
DALYs (Dis	Senegal	Male	55+ years	Non-melanoma skin cancer (basal-cell	Rate	1990	0.0131416	0.025557551	0.005474441
DALYs (Dis	Senegal	Female	55+ years	Non-melanoma skin cancer (basal-cell	Rate	1990	0.0084021	0.016279976	0.003308571
DALYs (Dis	Senegal	Both	55+ years	Non-melanoma skin cancer (basal-cell	Rate	1990	0.0108392	0.02109514	0.004415208
DALYs (Dis	Jamaica	Male	55+ years	Non-melanoma skin cancer (basal-cell	Number	1990	0.0267723	0.051368991	0.011536619
DALYs (Dis	Jamaica	Female	55+ years	Non-melanoma skin cancer (basal-cell	Number	1990	0.0213301	0.041064544	0.00935871
DALYs (Dis	Jamaica	Both	55+ years	Non-melanoma skin cancer (basal-cell	Number	1990	0.0481023	0.09229063	0.020730502
DALYs (Dis	Jamaica	Male	55+ years	Non-melanoma skin cancer (basal-cell	Percent	1990	2.42E-07	4.54E-07	1.09E-07
DALYs (Dis	Jamaica	Female	55+ years	Non-melanoma skin cancer (basal-cell	Percent	1990	1.87E-07	3.49E-07	8.59E-08

DALYs (Dis	Jamaica	Both	55+ years	Non-melanoma skin cancer (basal-cell	Percent	1990	2.14E-07	3.97E-07	9.75E-08
DALYs (Dis	Jamaica	Male	55+ years	Non-melanoma skin cancer (basal-cell	Rate	1990	0.0195763	0.037561744	0.008435741
DALYs (Dis	Jamaica	Female	55+ years	Non-melanoma skin cancer (basal-cell	Rate	1990	0.0135198	0.026028315	0.005931917
DALYs (Dis	Jamaica	Both	55+ years	Non-melanoma skin cancer (basal-cell	Rate	1990	0.016332	0.031335145	0.007038562
DALYs (Dis	Somalia	Male	55+ years	Non-melanoma skin cancer (basal-cell	Number	2021	0.0336889	0.068959245	0.014181242
DALYs (Dis	Somalia	Female	55+ years	Non-melanoma skin cancer (basal-cell	Number	2021	0.031454	0.062503989	0.013160403
DALYs (Dis	Somalia	Both	55+ years	Non-melanoma skin cancer (basal-cell	Number	2021	0.0651429	0.132389593	0.02752899
DALYs (Dis	Somalia	Male	55+ years	Non-melanoma skin cancer (basal-cell	Percent	2021	3.49E-08	6.90E-08	1.47E-08
DALYs (Dis	Somalia	Female	55+ years	Non-melanoma skin cancer (basal-cell	Percent	2021	3.34E-08	6.60E-08	1.47E-08
DALYs (Dis	Somalia	Both	55+ years	Non-melanoma skin cancer (basal-cell	Percent	2021	3.42E-08	6.75E-08	1.47E-08
DALYs (Dis	Somalia	Male	55+ years	Non-melanoma skin cancer (basal-cell	Rate	2021	0.008684	0.017775702	0.003655515
DALYs (Dis	Somalia	Female	55+ years	Non-melanoma skin cancer (basal-cell	Rate	2021	0.0056662	0.011259551	0.002370732
DALYs (Dis	Somalia	Both	55+ years	Non-melanoma skin cancer (basal-cell	Rate	2021	0.0069076	0.014038288	0.002919111
DALYs (Dis	Saint Kitt	Male	55+ years	Non-melanoma skin cancer (basal-cell	Number	2021	0.000726	0.001435971	0.000294805
DALYs (Dis	Saint Kitt	Female	55+ years	Non-melanoma skin cancer (basal-cell	Number	2021	0.0003311	0.000644682	0.00013463
DALYs (Dis	Saint Kitt	Both	55+ years	Non-melanoma skin cancer (basal-cell	Number	2021	0.0010571	0.002057803	0.000431797
DALYs (Dis	Saint Kitt	Male	55+ years	Non-melanoma skin cancer (basal-cell	Percent	2021	1.10E-07	2.18E-07	4.47E-08
DALYs (Dis	Saint Kitt	Female	55+ years	Non-melanoma skin cancer (basal-cell	Percent	2021	6.66E-08	1.26E-07	2.87E-08
DALYs (Dis	Saint Kitt	Both	55+ years	Non-melanoma skin cancer (basal-cell	Percent	2021	9.15E-08	1.77E-07	3.76E-08
DALYs (Dis	Saint Kitt	Male	55+ years	Non-melanoma skin cancer (basal-cell	Rate	2021	0.0119021	0.023540447	0.004832859
DALYs (Dis	Saint Kitt	Female	55+ years	Non-melanoma skin cancer (basal-cell	Rate	2021	0.0048834	0.009509489	0.001985886
DALYs (Dis	Saint Kitt	Both	55+ years	Non-melanoma skin cancer (basal-cell	Rate	2021	0.0082076	0.015977519	0.003352623
DALYs (Dis	Benin	Male	55+ years	Non-melanoma skin cancer (basal-cell	Number	1990	0.0134082	0.026450904	0.005593558
DALYs (Dis	Benin	Female	55+ years	Non-melanoma skin cancer (basal-cell	Number	1990	0.0085114	0.016696523	0.003356997
DALYs (Dis	Benin	Both	55+ years	Non-melanoma skin cancer (basal-cell	Number	1990	0.0219196	0.042936934	0.009144069
DALYs (Dis	Benin	Male	55+ years	Non-melanoma skin cancer (basal-cell	Percent	1990	6.32E-08	1.21E-07	2.67E-08
DALYs (Dis	Benin	Female	55+ years	Non-melanoma skin cancer (basal-cell	Percent	1990	4.65E-08	8.97E-08	1.85E-08
DALYs (Dis	Benin	Both	55+ years	Non-melanoma skin cancer (basal-cell	Percent	1990	5.54E-08	1.06E-07	2.33E-08
DALYs (Dis	Benin	Male	55+ years	Non-melanoma skin cancer (basal-cell	Rate	1990	0.0086575	0.017078971	0.003611681
DALYs (Dis	Benin	Female	55+ years	Non-melanoma skin cancer (basal-cell	Rate	1990	0.0051569	0.010116109	0.002033942
DALYs (Dis	Benin	Both	55+ years	Non-melanoma skin cancer (basal-cell	Rate	1990	0.0068515	0.013421023	0.00285821
DALYs (Dis	Djibouti	Male	55+ years	Non-melanoma skin cancer (basal-cell	Number	2021	0.0054894	0.011070675	0.002250581
DALYs (Dis	Djibouti	Female	55+ years	Non-melanoma skin cancer (basal-cell	Number	2021	0.002957	0.005858285	0.001147148
DALYs (Dis	Djibouti	Both	55+ years	Non-melanoma skin cancer (basal-cell	Number	2021	0.0084464	0.016691907	0.003414884
DALYs (Dis	Djibouti	Male	55+ years	Non-melanoma skin cancer (basal-cell	Percent	2021	7.09E-08	1.41E-07	3.11E-08
DALYs (Dis	Djibouti	Female	55+ years	Non-melanoma skin cancer (basal-cell	Percent	2021	6.42E-08	1.26E-07	2.74E-08
DALYs (Dis	Djibouti	Both	55+ years	Non-melanoma skin cancer (basal-cell	Percent	2021	6.84E-08	1.34E-07	3.04E-08
DALYs (Dis	Djibouti	Male	55+ years	Non-melanoma skin cancer (basal-cell	Rate	2021	0.0095248	0.019208747	0.003904987
DALYs (Dis	Djibouti	Female	55+ years	Non-melanoma skin cancer (basal-cell	Rate	2021	0.0064184	0.012715952	0.002489991
DALYs (Dis	Djibouti	Both	55+ years	Non-melanoma skin cancer (basal-cell	Rate	2021	0.0081447	0.016095741	0.003292919
DALYs (Dis	Palau	Male	55+ years	Non-melanoma skin cancer (basal-cell	Number	1990	7.81E-07	2.08E-06	1.63E-07
DALYs (Dis	Palau	Female	55+ years	Non-melanoma skin cancer (basal-cell	Number	1990	8.59E-07	2.24E-06	1.98E-07
DALYs (Dis	Palau	Both	55+ years	Non-melanoma skin cancer (basal-cell	Number	1990	1.64E-06	4.38E-06	4.07E-07
DALYs (Dis	Palau	Male	55+ years	Non-melanoma skin cancer (basal-cell	Percent	1990	7.76E-10	2.08E-09	1.67E-10
DALYs (Dis	Palau	Female	55+ years	Non-melanoma skin cancer (basal-cell	Percent	1990	1.01E-09	2.70E-09	2.35E-10
DALYs (Dis	Palau	Both	55+ years	Non-melanoma skin cancer (basal-cell	Percent	1990	8.84E-10	2.37E-09	2.24E-10
DALYs (Dis	Palau	Male	55+ years	Non-melanoma skin cancer (basal-cell	Rate	1990	9.88E-05	0.000263111	2.06E-05
DALYs (Dis	Palau	Female	55+ years	Non-melanoma skin cancer (basal-cell	Rate	1990	0.0001061	0.000277218	2.44E-05
DALYs (Dis	Palau	Both	55+ years	Non-melanoma skin cancer (basal-cell	Rate	1990	0.0001025	0.000273645	2.54E-05
DALYs (Dis	Bahamas	Male	55+ years	Non-melanoma skin cancer (basal-cell	Number	1990	0.0015593	0.003010133	0.000656124
DALYs (Dis	Bahamas	Female	55+ years	Non-melanoma skin cancer (basal-cell	Number	1990	0.0008486	0.001650861	0.000335532
DALYs (Dis	Bahamas	Both	55+ years	Non-melanoma skin cancer (basal-cell	Number	1990	0.0024079	0.004632723	0.001001706
DALYs (Dis	Bahamas	Male	55+ years	Non-melanoma skin cancer (basal-cell	Percent	1990	1.43E-07	2.74E-07	6.05E-08
DALYs (Dis	Bahamas	Female	55+ years	Non-melanoma skin cancer (basal-cell	Percent	1990	8.24E-08	1.56E-07	3.32E-08
DALYs (Dis	Bahamas	Both	55+ years	Non-melanoma skin cancer (basal-cell	Percent	1990	1.14E-07	2.16E-07	4.80E-08
DALYs (Dis	Bahamas	Male	55+ years	Non-melanoma skin cancer (basal-cell	Rate	1990	0.0144482	0.027891687	0.006079601

DALYs (Dis	Bahamas	Female	55+ years	Non-melanoma skin cancer (basal-cell	Rate	1990	0.0061655	0.011994824	0.002437908
DALYs (Dis	Bahamas	Both	55+ years	Non-melanoma skin cancer (basal-cell	Rate	1990	0.0098058	0.018866462	0.004079382
DALYs (Dis	Fiji	Male	55+ years	Non-melanoma skin cancer (basal-cell	Number	1990	2.72E-05	7.19E-05	5.37E-06
DALYs (Dis	Fiji	Female	55+ years	Non-melanoma skin cancer (basal-cell	Number	1990	2.87E-05	7.64E-05	6.82E-06
DALYs (Dis	Fiji	Both	55+ years	Non-melanoma skin cancer (basal-cell	Number	1990	5.60E-05	0.000149934	1.36E-05
DALYs (Dis	Fiji	Male	55+ years	Non-melanoma skin cancer (basal-cell	Percent	1990	7.18E-10	1.87E-09	1.48E-10
DALYs (Dis	Fiji	Female	55+ years	Non-melanoma skin cancer (basal-cell	Percent	1990	1.01E-09	2.76E-09	2.45E-10
DALYs (Dis	Fiji	Both	55+ years	Non-melanoma skin cancer (basal-cell	Percent	1990	8.41E-10	2.20E-09	2.05E-10
DALYs (Dis	Fiji	Male	55+ years	Non-melanoma skin cancer (basal-cell	Rate	1990	9.71E-05	0.000256368	1.91E-05
DALYs (Dis	Fiji	Female	55+ years	Non-melanoma skin cancer (basal-cell	Rate	1990	0.0001015	0.000269614	2.41E-05
DALYs (Dis	Fiji	Both	55+ years	Non-melanoma skin cancer (basal-cell	Rate	1990	9.93E-05	0.000266006	2.41E-05
DALYs (Dis	Romania	Male	55+ years	Non-melanoma skin cancer (basal-cell	Number	2021	1.6280455	3.360819358	0.675510414
DALYs (Dis	Romania	Female	55+ years	Non-melanoma skin cancer (basal-cell	Number	2021	2.0353983	3.915635991	0.850899132
DALYs (Dis	Romania	Both	55+ years	Non-melanoma skin cancer (basal-cell	Number	2021	3.6634438	7.420448006	1.515669954
DALYs (Dis	Romania	Male	55+ years	Non-melanoma skin cancer (basal-cell	Percent	2021	4.79E-07	9.59E-07	2.03E-07
DALYs (Dis	Romania	Female	55+ years	Non-melanoma skin cancer (basal-cell	Percent	2021	6.52E-07	1.24E-06	2.87E-07
DALYs (Dis	Romania	Both	55+ years	Non-melanoma skin cancer (basal-cell	Percent	2021	5.62E-07	1.11E-06	2.44E-07
DALYs (Dis	Romania	Male	55+ years	Non-melanoma skin cancer (basal-cell	Rate	2021	0.0630715	0.130200253	0.026169698
DALYs (Dis	Romania	Female	55+ years	Non-melanoma skin cancer (basal-cell	Rate	2021	0.0594436	0.114355833	0.02485044
DALYs (Dis	Romania	Both	55+ years	Non-melanoma skin cancer (basal-cell	Rate	2021	0.061003	0.123563953	0.025238661
DALYs (Dis	Belgium	Male	55+ years	Non-melanoma skin cancer (basal-cell	Number	2021	1.8065763	3.399817017	0.721665496
DALYs (Dis	Belgium	Female	55+ years	Non-melanoma skin cancer (basal-cell	Number	2021	1.6733492	3.326714554	0.640929405
DALYs (Dis	Belgium	Both	55+ years	Non-melanoma skin cancer (basal-cell	Number	2021	3.4799255	6.451056798	1.380366138
DALYs (Dis	Belgium	Male	55+ years	Non-melanoma skin cancer (basal-cell	Percent	2021	1.50E-06	2.77E-06	6.28E-07
DALYs (Dis	Belgium	Female	55+ years	Non-melanoma skin cancer (basal-cell	Percent	2021	1.39E-06	2.71E-06	5.86E-07
DALYs (Dis	Belgium	Both	55+ years	Non-melanoma skin cancer (basal-cell	Percent	2021	1.45E-06	2.63E-06	6.32E-07
DALYs (Dis	Belgium	Male	55+ years	Non-melanoma skin cancer (basal-cell	Rate	2021	0.102746	0.193358949	0.041043527
DALYs (Dis	Belgium	Female	55+ years	Non-melanoma skin cancer (basal-cell	Rate	2021	0.0827375	0.16448692	0.031690276
DALYs (Dis	Belgium	Both	55+ years	Non-melanoma skin cancer (basal-cell	Rate	2021	0.0920427	0.170627996	0.036510159
DALYs (Dis	Cambodia	Male	55+ years	Non-melanoma skin cancer (basal-cell	Number	1990	0.0079185	0.015677571	0.003195844
DALYs (Dis	Cambodia	Female	55+ years	Non-melanoma skin cancer (basal-cell	Number	1990	0.0084344	0.016133373	0.003327497
DALYs (Dis	Cambodia	Both	55+ years	Non-melanoma skin cancer (basal-cell	Number	1990	0.0163529	0.031349021	0.006561699
DALYs (Dis	Cambodia	Male	55+ years	Non-melanoma skin cancer (basal-cell	Percent	1990	1.71E-08	3.44E-08	6.76E-09
DALYs (Dis	Cambodia	Female	55+ years	Non-melanoma skin cancer (basal-cell	Percent	1990	1.67E-08	3.20E-08	6.68E-09
DALYs (Dis	Cambodia	Both	55+ years	Non-melanoma skin cancer (basal-cell	Percent	1990	1.69E-08	3.20E-08	6.66E-09
DALYs (Dis	Cambodia	Male	55+ years	Non-melanoma skin cancer (basal-cell	Rate	1990	0.0025005	0.004950734	0.001009198
DALYs (Dis	Cambodia	Female	55+ years	Non-melanoma skin cancer (basal-cell	Rate	1990	0.0019881	0.003802799	0.000784324
DALYs (Dis	Cambodia	Both	55+ years	Non-melanoma skin cancer (basal-cell	Rate	1990	0.0022071	0.004231085	0.000885613
DALYs (Dis	Bangladesh	Male	55+ years	Non-melanoma skin cancer (basal-cell	Number	2021	0.0144703	0.037092103	0.00292725
DALYs (Dis	Bangladesh	Female	55+ years	Non-melanoma skin cancer (basal-cell	Number	2021	0.0092011	0.024417865	0.00210599
DALYs (Dis	Bangladesh	Both	55+ years	Non-melanoma skin cancer (basal-cell	Number	2021	0.0236713	0.056785977	0.006360139
DALYs (Dis	Bangladesh	Male	55+ years	Non-melanoma skin cancer (basal-cell	Percent	2021	1.26E-09	3.27E-09	2.61E-10
DALYs (Dis	Bangladesh	Female	55+ years	Non-melanoma skin cancer (basal-cell	Percent	2021	1.04E-09	2.76E-09	2.35E-10
DALYs (Dis	Bangladesh	Both	55+ years	Non-melanoma skin cancer (basal-cell	Percent	2021	1.17E-09	2.85E-09	3.09E-10
DALYs (Dis	Bangladesh	Male	55+ years	Non-melanoma skin cancer (basal-cell	Rate	2021	0.0001198	0.000306962	2.42E-05
DALYs (Dis	Bangladesh	Female	55+ years	Non-melanoma skin cancer (basal-cell	Rate	2021	8.16E-05	0.000216422	1.87E-05
DALYs (Dis	Bangladesh	Both	55+ years	Non-melanoma skin cancer (basal-cell	Rate	2021	0.0001013	0.000243027	2.72E-05
DALYs (Dis	Qatar	Male	55+ years	Non-melanoma skin cancer (basal-cell	Number	1990	0.0011549	0.002288898	0.000446039
DALYs (Dis	Qatar	Female	55+ years	Non-melanoma skin cancer (basal-cell	Number	1990	0.0003361	0.000716139	0.00013079
DALYs (Dis	Qatar	Both	55+ years	Non-melanoma skin cancer (basal-cell	Number	1990	0.0014909	0.002975343	0.00057352
DALYs (Dis	Qatar	Male	55+ years	Non-melanoma skin cancer (basal-cell	Percent	1990	1.08E-07	2.17E-07	4.25E-08
DALYs (Dis	Qatar	Female	55+ years	Non-melanoma skin cancer (basal-cell	Percent	1990	7.43E-08	1.54E-07	2.91E-08
DALYs (Dis	Qatar	Both	55+ years	Non-melanoma skin cancer (basal-cell	Percent	1990	9.77E-08	2.00E-07	3.85E-08
DALYs (Dis	Qatar	Male	55+ years	Non-melanoma skin cancer (basal-cell	Rate	1990	0.010194	0.020203994	0.003937164
DALYs (Dis	Qatar	Female	55+ years	Non-melanoma skin cancer (basal-cell	Rate	1990	0.0067129	0.014304626	0.002612485
DALYs (Dis	Qatar	Both	55+ years	Non-melanoma skin cancer (basal-cell	Rate	1990	0.0091272	0.018214212	0.003510927

DALYs (Dis	Gabon	Male	55+ years	Non-melanoma skin cancer (basal-cell	Number	2021	0.0101295	0.019797694	0.004269093
DALYs (Dis	Gabon	Female	55+ years	Non-melanoma skin cancer (basal-cell	Number	2021	0.0070324	0.013663784	0.002882838
DALYs (Dis	Gabon	Both	55+ years	Non-melanoma skin cancer (basal-cell	Number	2021	0.0171619	0.033068937	0.00719852
DALYs (Dis	Gabon	Male	55+ years	Non-melanoma skin cancer (basal-cell	Percent	2021	7.40E-08	1.41E-07	3.22E-08
DALYs (Dis	Gabon	Female	55+ years	Non-melanoma skin cancer (basal-cell	Percent	2021	6.93E-08	1.33E-07	3.01E-08
DALYs (Dis	Gabon	Both	55+ years	Non-melanoma skin cancer (basal-cell	Percent	2021	7.20E-08	1.39E-07	3.16E-08
DALYs (Dis	Gabon	Male	55+ years	Non-melanoma skin cancer (basal-cell	Rate	2021	0.0116542	0.022777675	0.004911684
DALYs (Dis	Gabon	Female	55+ years	Non-melanoma skin cancer (basal-cell	Rate	2021	0.0076577	0.014878643	0.003139154
DALYs (Dis	Gabon	Both	55+ years	Non-melanoma skin cancer (basal-cell	Rate	2021	0.009601	0.0184999	0.004027099
DALYs (Dis	Kazakhstan	Male	55+ years	Non-melanoma skin cancer (basal-cell	Number	2021	0.6556369	1.274892288	0.2745984
DALYs (Dis	Kazakhstan	Female	55+ years	Non-melanoma skin cancer (basal-cell	Number	2021	1.0463422	1.997527503	0.451562014
DALYs (Dis	Kazakhstan	Both	55+ years	Non-melanoma skin cancer (basal-cell	Number	2021	1.7019791	3.252450774	0.736628813
DALYs (Dis	Kazakhstan	Male	55+ years	Non-melanoma skin cancer (basal-cell	Percent	2021	3.62E-07	7.02E-07	1.55E-07
DALYs (Dis	Kazakhstan	Female	55+ years	Non-melanoma skin cancer (basal-cell	Percent	2021	6.17E-07	1.15E-06	2.71E-07
DALYs (Dis	Kazakhstan	Both	55+ years	Non-melanoma skin cancer (basal-cell	Percent	2021	4.85E-07	8.97E-07	2.14E-07
DALYs (Dis	Kazakhstan	Male	55+ years	Non-melanoma skin cancer (basal-cell	Rate	2021	0.050159	0.097534721	0.021007954
DALYs (Dis	Kazakhstan	Female	55+ years	Non-melanoma skin cancer (basal-cell	Rate	2021	0.0560642	0.107029744	0.024195195
DALYs (Dis	Kazakhstan	Both	55+ years	Non-melanoma skin cancer (basal-cell	Rate	2021	0.0536319	0.102489568	0.023212271
DALYs (Dis	Hungary	Male	55+ years	Non-melanoma skin cancer (basal-cell	Number	2021	1.0267126	1.95699347	0.44637863
DALYs (Dis	Hungary	Female	55+ years	Non-melanoma skin cancer (basal-cell	Number	2021	1.3971026	2.917268069	0.572914621
DALYs (Dis	Hungary	Both	55+ years	Non-melanoma skin cancer (basal-cell	Number	2021	2.4238152	4.875526467	1.052496493
DALYs (Dis	Hungary	Male	55+ years	Non-melanoma skin cancer (basal-cell	Percent	2021	6.34E-07	1.22E-06	2.85E-07
DALYs (Dis	Hungary	Female	55+ years	Non-melanoma skin cancer (basal-cell	Percent	2021	8.63E-07	1.78E-06	3.66E-07
DALYs (Dis	Hungary	Both	55+ years	Non-melanoma skin cancer (basal-cell	Percent	2021	7.49E-07	1.47E-06	3.35E-07
DALYs (Dis	Hungary	Male	55+ years	Non-melanoma skin cancer (basal-cell	Rate	2021	0.0778618	0.148410668	0.033851595
DALYs (Dis	Hungary	Female	55+ years	Non-melanoma skin cancer (basal-cell	Rate	2021	0.0750529	0.156716849	0.030777211
DALYs (Dis	Hungary	Both	55+ years	Non-melanoma skin cancer (basal-cell	Rate	2021	0.0762176	0.153312479	0.033096087
DALYs (Dis	Zambia	Male	55+ years	Non-melanoma skin cancer (basal-cell	Number	1990	0.0238126	0.047143152	0.009802788
DALYs (Dis	Zambia	Female	55+ years	Non-melanoma skin cancer (basal-cell	Number	1990	0.0129507	0.026682908	0.005281489
DALYs (Dis	Zambia	Both	55+ years	Non-melanoma skin cancer (basal-cell	Number	1990	0.0367632	0.074529226	0.015168663
DALYs (Dis	Zambia	Male	55+ years	Non-melanoma skin cancer (basal-cell	Percent	1990	6.42E-08	1.26E-07	2.73E-08
DALYs (Dis	Zambia	Female	55+ years	Non-melanoma skin cancer (basal-cell	Percent	1990	4.71E-08	9.45E-08	1.99E-08
DALYs (Dis	Zambia	Both	55+ years	Non-melanoma skin cancer (basal-cell	Percent	1990	5.69E-08	1.13E-07	2.43E-08
DALYs (Dis	Zambia	Male	55+ years	Non-melanoma skin cancer (basal-cell	Rate	1990	0.0097407	0.019284243	0.0040099
DALYs (Dis	Zambia	Female	55+ years	Non-melanoma skin cancer (basal-cell	Rate	1990	0.0059996	0.012361153	0.002446708
DALYs (Dis	Zambia	Both	55+ years	Non-melanoma skin cancer (basal-cell	Rate	1990	0.0079864	0.016190545	0.003295203
DALYs (Dis	Switzerlan	Male	55+ years	Non-melanoma skin cancer (basal-cell	Number	1990	1.5817229	3.103648679	0.688084048
DALYs (Dis	Switzerlan	Female	55+ years	Non-melanoma skin cancer (basal-cell	Number	1990	1.7183456	3.478269406	0.739704595
DALYs (Dis	Switzerlan	Both	55+ years	Non-melanoma skin cancer (basal-cell	Number	1990	3.3000685	6.451639658	1.454216622
DALYs (Dis	Switzerlan	Male	55+ years	Non-melanoma skin cancer (basal-cell	Percent	1990	2.50E-06	4.63E-06	1.14E-06
DALYs (Dis	Switzerlan	Female	55+ years	Non-melanoma skin cancer (basal-cell	Percent	1990	2.73E-06	5.39E-06	1.21E-06
DALYs (Dis	Switzerlan	Both	55+ years	Non-melanoma skin cancer (basal-cell	Percent	1990	2.61E-06	4.82E-06	1.18E-06
DALYs (Dis	Switzerlan	Male	55+ years	Non-melanoma skin cancer (basal-cell	Rate	1990	0.2174959	0.426769348	0.094615471
DALYs (Dis	Switzerlan	Female	55+ years	Non-melanoma skin cancer (basal-cell	Rate	1990	0.1786364	0.361595159	0.076898471
DALYs (Dis	Switzerlan	Both	55+ years	Non-melanoma skin cancer (basal-cell	Rate	1990	0.1953667	0.381942242	0.08609079
DALYs (Dis	Marshall I	Male	55+ years	Non-melanoma skin cancer (basal-cell	Number	2021	2.71E-06	7.11E-06	5.47E-07
DALYs (Dis	Marshall I	Female	55+ years	Non-melanoma skin cancer (basal-cell	Number	2021	2.71E-06	7.35E-06	5.97E-07
DALYs (Dis	Marshall I	Both	55+ years	Non-melanoma skin cancer (basal-cell	Number	2021	5.42E-06	1.46E-05	1.30E-06
DALYs (Dis	Marshall I	Male	55+ years	Non-melanoma skin cancer (basal-cell	Percent	2021	7.13E-10	1.80E-09	1.48E-10
DALYs (Dis	Marshall I	Female	55+ years	Non-melanoma skin cancer (basal-cell	Percent	2021	8.21E-10	2.17E-09	1.81E-10
DALYs (Dis	Marshall I	Both	55+ years	Non-melanoma skin cancer (basal-cell	Percent	2021	7.63E-10	1.97E-09	1.79E-10
DALYs (Dis	Marshall I	Male	55+ years	Non-melanoma skin cancer (basal-cell	Rate	2021	9.22E-05	0.000241896	1.86E-05
DALYs (Dis	Marshall I	Female	55+ years	Non-melanoma skin cancer (basal-cell	Rate	2021	9.30E-05	0.000251748	2.05E-05
DALYs (Dis	Marshall I	Both	55+ years	Non-melanoma skin cancer (basal-cell	Rate	2021	9.26E-05	0.000248851	2.22E-05
DALYs (Dis	Eritrea	Male	55+ years	Non-melanoma skin cancer (basal-cell	Number	1990	0.006048	0.012804377	0.002497396
DALYs (Dis	Eritrea	Female	55+ years	Non-melanoma skin cancer (basal-cell	Number	1990	0.0056863	0.01154491	0.002328207

DALYs (Dis	Eritrea	Both	55+ years	Non-melanoma skin cancer (basal-cell	Number	1990	0.0117343	0.024032464	0.004880907
DALYs (Dis	Eritrea	Male	55+ years	Non-melanoma skin cancer (basal-cell	Percent	1990	3.94E-08	8.27E-08	1.62E-08
DALYs (Dis	Eritrea	Female	55+ years	Non-melanoma skin cancer (basal-cell	Percent	1990	3.96E-08	7.90E-08	1.65E-08
DALYs (Dis	Eritrea	Both	55+ years	Non-melanoma skin cancer (basal-cell	Percent	1990	3.95E-08	8.07E-08	1.70E-08
DALYs (Dis	Eritrea	Male	55+ years	Non-melanoma skin cancer (basal-cell	Rate	1990	0.0079777	0.01688979	0.003294225
DALYs (Dis	Eritrea	Female	55+ years	Non-melanoma skin cancer (basal-cell	Rate	1990	0.005635	0.011440919	0.002307236
DALYs (Dis	Eritrea	Both	55+ years	Non-melanoma skin cancer (basal-cell	Rate	1990	0.00664	0.013599154	0.002761939
DALYs (Dis	Lithuania	Male	55+ years	Non-melanoma skin cancer (basal-cell	Number	2021	0.2372399	0.488484442	0.10592804
DALYs (Dis	Lithuania	Female	55+ years	Non-melanoma skin cancer (basal-cell	Number	2021	0.4364436	0.930431129	0.16677173
DALYs (Dis	Lithuania	Both	55+ years	Non-melanoma skin cancer (basal-cell	Number	2021	0.6736835	1.350097849	0.29269724
DALYs (Dis	Lithuania	Male	55+ years	Non-melanoma skin cancer (basal-cell	Percent	2021	5.26E-07	1.06E-06	2.39E-07
DALYs (Dis	Lithuania	Female	55+ years	Non-melanoma skin cancer (basal-cell	Percent	2021	9.08E-07	1.88E-06	3.59E-07
DALYs (Dis	Lithuania	Both	55+ years	Non-melanoma skin cancer (basal-cell	Percent	2021	7.23E-07	1.42E-06	3.20E-07
DALYs (Dis	Lithuania	Male	55+ years	Non-melanoma skin cancer (basal-cell	Rate	2021	0.0636312	0.131018664	0.028411448
DALYs (Dis	Lithuania	Female	55+ years	Non-melanoma skin cancer (basal-cell	Rate	2021	0.0733587	0.156389584	0.02803148
DALYs (Dis	Lithuania	Both	55+ years	Non-melanoma skin cancer (basal-cell	Rate	2021	0.0696112	0.139504594	0.030244185
DALYs (Dis	Greece	Male	55+ years	Non-melanoma skin cancer (basal-cell	Number	1990	1.3245921	2.738414996	0.565702642
DALYs (Dis	Greece	Female	55+ years	Non-melanoma skin cancer (basal-cell	Number	1990	1.1901101	2.306962263	0.482073173
DALYs (Dis	Greece	Both	55+ years	Non-melanoma skin cancer (basal-cell	Number	1990	2.5147021	5.155615839	1.076646942
DALYs (Dis	Greece	Male	55+ years	Non-melanoma skin cancer (basal-cell	Percent	1990	1.31E-06	2.62E-06	5.87E-07
DALYs (Dis	Greece	Female	55+ years	Non-melanoma skin cancer (basal-cell	Percent	1990	1.24E-06	2.38E-06	5.08E-07
DALYs (Dis	Greece	Both	55+ years	Non-melanoma skin cancer (basal-cell	Percent	1990	1.28E-06	2.52E-06	5.63E-07
DALYs (Dis	Greece	Male	55+ years	Non-melanoma skin cancer (basal-cell	Rate	1990	0.1071034	0.221421714	0.045741368
DALYs (Dis	Greece	Female	55+ years	Non-melanoma skin cancer (basal-cell	Rate	1990	0.0828233	0.160548341	0.033548901
DALYs (Dis	Greece	Both	55+ years	Non-melanoma skin cancer (basal-cell	Rate	1990	0.0940544	0.192829268	0.040268524
DALYs (Dis	Mexico	Male	55+ years	Non-melanoma skin cancer (basal-cell	Number	1990	2.1936333	4.132181281	0.962036018
DALYs (Dis	Mexico	Female	55+ years	Non-melanoma skin cancer (basal-cell	Number	1990	2.1453851	4.137277533	0.898407561
DALYs (Dis	Mexico	Both	55+ years	Non-melanoma skin cancer (basal-cell	Number	1990	4.3390184	8.286230339	1.870674981
DALYs (Dis	Mexico	Male	55+ years	Non-melanoma skin cancer (basal-cell	Percent	1990	7.52E-07	1.41E-06	3.45E-07
DALYs (Dis	Mexico	Female	55+ years	Non-melanoma skin cancer (basal-cell	Percent	1990	7.85E-07	1.50E-06	3.47E-07
DALYs (Dis	Mexico	Both	55+ years	Non-melanoma skin cancer (basal-cell	Percent	1990	7.68E-07	1.46E-06	3.46E-07
DALYs (Dis	Mexico	Male	55+ years	Non-melanoma skin cancer (basal-cell	Rate	1990	0.0656528	0.123671135	0.028792562
DALYs (Dis	Mexico	Female	55+ years	Non-melanoma skin cancer (basal-cell	Rate	1990	0.0594047	0.114559252	0.024876479
DALYs (Dis	Mexico	Both	55+ years	Non-melanoma skin cancer (basal-cell	Rate	1990	0.0624073	0.119179355	0.026905581
DALYs (Dis	Nigeria	Male	55+ years	Non-melanoma skin cancer (basal-cell	Number	2021	0.4930589	0.970112229	0.202137003
DALYs (Dis	Nigeria	Female	55+ years	Non-melanoma skin cancer (basal-cell	Number	2021	0.3332263	0.65757594	0.130695717
DALYs (Dis	Nigeria	Both	55+ years	Non-melanoma skin cancer (basal-cell	Number	2021	0.8262852	1.627075831	0.333815115
DALYs (Dis	Nigeria	Male	55+ years	Non-melanoma skin cancer (basal-cell	Percent	2021	6.35E-08	1.25E-07	2.53E-08
DALYs (Dis	Nigeria	Female	55+ years	Non-melanoma skin cancer (basal-cell	Percent	2021	4.41E-08	8.68E-08	1.80E-08
DALYs (Dis	Nigeria	Both	55+ years	Non-melanoma skin cancer (basal-cell	Percent	2021	5.37E-08	1.04E-07	2.15E-08
DALYs (Dis	Nigeria	Male	55+ years	Non-melanoma skin cancer (basal-cell	Rate	2021	0.0073666	0.014494157	0.003020069
DALYs (Dis	Nigeria	Female	55+ years	Non-melanoma skin cancer (basal-cell	Rate	2021	0.0040107	0.007914605	0.001573058
DALYs (Dis	Nigeria	Both	55+ years	Non-melanoma skin cancer (basal-cell	Rate	2021	0.005508	0.010846079	0.00222521
DALYs (Dis	Burundi	Male	55+ years	Non-melanoma skin cancer (basal-cell	Number	1990	0.0174395	0.033872678	0.007386459
DALYs (Dis	Burundi	Female	55+ years	Non-melanoma skin cancer (basal-cell	Number	1990	0.0128217	0.025954143	0.005207016
DALYs (Dis	Burundi	Both	55+ years	Non-melanoma skin cancer (basal-cell	Number	1990	0.0302612	0.059754469	0.012800874
DALYs (Dis	Burundi	Male	55+ years	Non-melanoma skin cancer (basal-cell	Percent	1990	5.18E-08	1.01E-07	2.18E-08
DALYs (Dis	Burundi	Female	55+ years	Non-melanoma skin cancer (basal-cell	Percent	1990	4.19E-08	8.38E-08	1.74E-08
DALYs (Dis	Burundi	Both	55+ years	Non-melanoma skin cancer (basal-cell	Percent	1990	4.71E-08	9.29E-08	1.99E-08
DALYs (Dis	Burundi	Male	55+ years	Non-melanoma skin cancer (basal-cell	Rate	1990	0.0102446	0.019898001	0.004339066
DALYs (Dis	Burundi	Female	55+ years	Non-melanoma skin cancer (basal-cell	Rate	1990	0.0062206	0.012591997	0.002526253
DALYs (Dis	Burundi	Both	55+ years	Non-melanoma skin cancer (basal-cell	Rate	1990	0.0080408	0.015877462	0.003401342
DALYs (Dis	Rwanda	Male	55+ years	Non-melanoma skin cancer (basal-cell	Number	2021	0.0422078	0.083201621	0.017943235
DALYs (Dis	Rwanda	Female	55+ years	Non-melanoma skin cancer (basal-cell	Number	2021	0.037626	0.074351627	0.015117563
DALYs (Dis	Rwanda	Both	55+ years	Non-melanoma skin cancer (basal-cell	Number	2021	0.0798337	0.155393669	0.033299519
DALYs (Dis	Rwanda	Male	55+ years	Non-melanoma skin cancer (basal-cell	Percent	2021	6.67E-08	1.27E-07	2.92E-08

DALYs (Dis	Rwanda	Female	55+ years	Non-melanoma skin cancer (basal-cell	Percent	2021	5.90E-08	1.12E-07	2.44E-08
DALYs (Dis	Rwanda	Both	55+ years	Non-melanoma skin cancer (basal-cell	Percent	2021	6.28E-08	1.19E-07	2.71E-08
DALYs (Dis	Rwanda	Male	55+ years	Non-melanoma skin cancer (basal-cell	Rate	2021	0.0094483	0.018624851	0.004016629
DALYs (Dis	Rwanda	Female	55+ years	Non-melanoma skin cancer (basal-cell	Rate	2021	0.0061607	0.012173975	0.002475276
DALYs (Dis	Rwanda	Both	55+ years	Non-melanoma skin cancer (basal-cell	Rate	2021	0.0075495	0.014694908	0.003148992
DALYs (Dis	Niger	Male	55+ years	Non-melanoma skin cancer (basal-cell	Number	1990	0.0186481	0.037621472	0.007664227
DALYs (Dis	Niger	Female	55+ years	Non-melanoma skin cancer (basal-cell	Number	1990	0.0094436	0.01873531	0.003849236
DALYs (Dis	Niger	Both	55+ years	Non-melanoma skin cancer (basal-cell	Number	1990	0.0280917	0.056272094	0.011505343
DALYs (Dis	Niger	Male	55+ years	Non-melanoma skin cancer (basal-cell	Percent	1990	6.18E-08	1.24E-07	2.53E-08
DALYs (Dis	Niger	Female	55+ years	Non-melanoma skin cancer (basal-cell	Percent	1990	3.99E-08	7.86E-08	1.65E-08
DALYs (Dis	Niger	Both	55+ years	Non-melanoma skin cancer (basal-cell	Percent	1990	5.21E-08	1.03E-07	2.14E-08
DALYs (Dis	Niger	Male	55+ years	Non-melanoma skin cancer (basal-cell	Rate	1990	0.007898	0.015933735	0.003246012
DALYs (Dis	Niger	Female	55+ years	Non-melanoma skin cancer (basal-cell	Rate	1990	0.0046432	0.009211666	0.001892569
DALYs (Dis	Niger	Both	55+ years	Non-melanoma skin cancer (basal-cell	Rate	1990	0.0063918	0.012803696	0.002617832
DALYs (Dis	Northern M	Male	55+ years	Non-melanoma skin cancer (basal-cell	Number	1990	1.71E-06	4.60E-06	3.29E-07
DALYs (Dis	Northern M	Female	55+ years	Non-melanoma skin cancer (basal-cell	Number	1990	1.15E-06	3.00E-06	2.68E-07
DALYs (Dis	Northern M	Both	55+ years	Non-melanoma skin cancer (basal-cell	Number	1990	2.86E-06	7.68E-06	6.88E-07
DALYs (Dis	Northern M	Male	55+ years	Non-melanoma skin cancer (basal-cell	Percent	1990	1.35E-09	3.69E-09	2.76E-10
DALYs (Dis	Northern M	Female	55+ years	Non-melanoma skin cancer (basal-cell	Percent	1990	1.47E-09	3.79E-09	3.34E-10
DALYs (Dis	Northern M	Both	55+ years	Non-melanoma skin cancer (basal-cell	Percent	1990	1.39E-09	3.67E-09	3.50E-10
DALYs (Dis	Northern M	Male	55+ years	Non-melanoma skin cancer (basal-cell	Rate	1990	0.0001155	0.000309941	2.22E-05
DALYs (Dis	Northern M	Female	55+ years	Non-melanoma skin cancer (basal-cell	Rate	1990	0.0001196	0.000313403	2.80E-05
DALYs (Dis	Northern M	Both	55+ years	Non-melanoma skin cancer (basal-cell	Rate	1990	0.0001171	0.000314482	2.82E-05
DALYs (Dis	Democratic	Male	55+ years	Non-melanoma skin cancer (basal-cell	Number	2021	0.0105836	0.022324063	0.003801983
DALYs (Dis	Democratic	Female	55+ years	Non-melanoma skin cancer (basal-cell	Number	2021	0.0141169	0.028726728	0.004894149
DALYs (Dis	Democratic	Both	55+ years	Non-melanoma skin cancer (basal-cell	Number	2021	0.0247005	0.050925251	0.008603234
DALYs (Dis	Democratic	Male	55+ years	Non-melanoma skin cancer (basal-cell	Percent	2021	4.78E-09	9.57E-09	1.75E-09
DALYs (Dis	Democratic	Female	55+ years	Non-melanoma skin cancer (basal-cell	Percent	2021	5.56E-09	1.11E-08	2.00E-09
DALYs (Dis	Democratic	Both	55+ years	Non-melanoma skin cancer (basal-cell	Percent	2021	5.20E-09	1.03E-08	1.91E-09
DALYs (Dis	Democratic	Male	55+ years	Non-melanoma skin cancer (basal-cell	Rate	2021	0.0004479	0.000944689	0.000160889
DALYs (Dis	Democratic	Female	55+ years	Non-melanoma skin cancer (basal-cell	Rate	2021	0.0004314	0.00087778	0.000149547
DALYs (Dis	Democratic	Both	55+ years	Non-melanoma skin cancer (basal-cell	Rate	2021	0.0004383	0.000903608	0.000152654
DALYs (Dis	Turkmenist	Male	55+ years	Non-melanoma skin cancer (basal-cell	Number	2021	0.1557477	0.300157516	0.0631657
DALYs (Dis	Turkmenist	Female	55+ years	Non-melanoma skin cancer (basal-cell	Number	2021	0.2099106	0.41533233	0.084344414
DALYs (Dis	Turkmenist	Both	55+ years	Non-melanoma skin cancer (basal-cell	Number	2021	0.3656583	0.70044704	0.14926114
DALYs (Dis	Turkmenist	Male	55+ years	Non-melanoma skin cancer (basal-cell	Percent	2021	3.99E-07	7.50E-07	1.70E-07
DALYs (Dis	Turkmenist	Female	55+ years	Non-melanoma skin cancer (basal-cell	Percent	2021	5.80E-07	1.12E-06	2.54E-07
DALYs (Dis	Turkmenist	Both	55+ years	Non-melanoma skin cancer (basal-cell	Percent	2021	4.86E-07	9.14E-07	2.09E-07
DALYs (Dis	Turkmenist	Male	55+ years	Non-melanoma skin cancer (basal-cell	Rate	2021	0.050498	0.097320002	0.020480201
DALYs (Dis	Turkmenist	Female	55+ years	Non-melanoma skin cancer (basal-cell	Rate	2021	0.0525726	0.104020895	0.021124244
DALYs (Dis	Turkmenist	Both	55+ years	Non-melanoma skin cancer (basal-cell	Rate	2021	0.0516685	0.098974991	0.021090988
DALYs (Dis	Taiwan (Pr	Male	55+ years	Non-melanoma skin cancer (basal-cell	Number	2021	0.0071178	0.016313826	0.002260727
DALYs (Dis	Taiwan (Pr	Female	55+ years	Non-melanoma skin cancer (basal-cell	Number	2021	0.0083664	0.019431761	0.002495078
DALYs (Dis	Taiwan (Pr	Both	55+ years	Non-melanoma skin cancer (basal-cell	Number	2021	0.0154842	0.035197019	0.00473479
DALYs (Dis	Taiwan (Pr	Male	55+ years	Non-melanoma skin cancer (basal-cell	Percent	2021	3.06E-09	6.84E-09	1.01E-09
DALYs (Dis	Taiwan (Pr	Female	55+ years	Non-melanoma skin cancer (basal-cell	Percent	2021	4.34E-09	9.72E-09	1.35E-09
DALYs (Dis	Taiwan (Pr	Both	55+ years	Non-melanoma skin cancer (basal-cell	Percent	2021	3.64E-09	8.12E-09	1.17E-09
DALYs (Dis	Taiwan (Pr	Male	55+ years	Non-melanoma skin cancer (basal-cell	Rate	2021	0.0002013	0.000461449	6.39E-05
DALYs (Dis	Taiwan (Pr	Female	55+ years	Non-melanoma skin cancer (basal-cell	Rate	2021	0.0002094	0.000486452	6.25E-05
DALYs (Dis	Taiwan (Pr	Both	55+ years	Non-melanoma skin cancer (basal-cell	Rate	2021	0.0002056	0.000467428	6.29E-05
DALYs (Dis	Iceland	Male	55+ years	Non-melanoma skin cancer (basal-cell	Number	1990	0.0243797	0.049649635	0.00952019
DALYs (Dis	Iceland	Female	55+ years	Non-melanoma skin cancer (basal-cell	Number	1990	0.0222619	0.045074837	0.009342108
DALYs (Dis	Iceland	Both	55+ years	Non-melanoma skin cancer (basal-cell	Number	1990	0.0466416	0.093000041	0.01909807
DALYs (Dis	Iceland	Male	55+ years	Non-melanoma skin cancer (basal-cell	Percent	1990	1.39E-06	2.77E-06	5.61E-07
DALYs (Dis	Iceland	Female	55+ years	Non-melanoma skin cancer (basal-cell	Percent	1990	1.28E-06	2.53E-06	5.53E-07
DALYs (Dis	Iceland	Both	55+ years	Non-melanoma skin cancer (basal-cell	Percent	1990	1.34E-06	2.63E-06	5.61E-07

DALYs (Dis	Iceland	Male	55+ years	Non-melanoma skin cancer (basal-cell	Rate	1990	0.1093158	0.222623836	0.04268755
DALYs (Dis	Iceland	Female	55+ years	Non-melanoma skin cancer (basal-cell	Rate	1990	0.0876014	0.177370774	0.036761463
DALYs (Dis	Iceland	Both	55+ years	Non-melanoma skin cancer (basal-cell	Rate	1990	0.0977508	0.194908141	0.040025458
DALYs (Dis	Lao People	Male	55+ years	Non-melanoma skin cancer (basal-cell	Number	2021	0.0093188	0.01845766	0.00370048
DALYs (Dis	Lao People	Female	55+ years	Non-melanoma skin cancer (basal-cell	Number	2021	0.0077444	0.015308261	0.003022034
DALYs (Dis	Lao People	Both	55+ years	Non-melanoma skin cancer (basal-cell	Number	2021	0.0170632	0.033434052	0.006834421
DALYs (Dis	Lao People	Male	55+ years	Non-melanoma skin cancer (basal-cell	Percent	2021	2.19E-08	4.16E-08	9.05E-09
DALYs (Dis	Lao People	Female	55+ years	Non-melanoma skin cancer (basal-cell	Percent	2021	2.17E-08	4.19E-08	8.85E-09
DALYs (Dis	Lao People	Both	55+ years	Non-melanoma skin cancer (basal-cell	Percent	2021	2.18E-08	4.20E-08	9.04E-09
DALYs (Dis	Lao People	Male	55+ years	Non-melanoma skin cancer (basal-cell	Rate	2021	0.0024928	0.004937366	0.000989867
DALYs (Dis	Lao People	Female	55+ years	Non-melanoma skin cancer (basal-cell	Rate	2021	0.0019394	0.003833651	0.000756809
DALYs (Dis	Lao People	Both	55+ years	Non-melanoma skin cancer (basal-cell	Rate	2021	0.002207	0.004324399	0.000883972
DALYs (Dis	Mauritania	Male	55+ years	Non-melanoma skin cancer (basal-cell	Number	1990	0.0071285	0.014293022	0.00288205
DALYs (Dis	Mauritania	Female	55+ years	Non-melanoma skin cancer (basal-cell	Number	1990	0.0043714	0.008375456	0.001798012
DALYs (Dis	Mauritania	Both	55+ years	Non-melanoma skin cancer (basal-cell	Number	1990	0.0114998	0.02264118	0.004669851
DALYs (Dis	Mauritania	Male	55+ years	Non-melanoma skin cancer (basal-cell	Percent	1990	7.55E-08	1.52E-07	3.06E-08
DALYs (Dis	Mauritania	Female	55+ years	Non-melanoma skin cancer (basal-cell	Percent	1990	3.98E-08	7.54E-08	1.67E-08
DALYs (Dis	Mauritania	Both	55+ years	Non-melanoma skin cancer (basal-cell	Percent	1990	5.63E-08	1.09E-07	2.33E-08
DALYs (Dis	Mauritania	Male	55+ years	Non-melanoma skin cancer (basal-cell	Rate	1990	0.0089449	0.017934943	0.003616409
DALYs (Dis	Mauritania	Female	55+ years	Non-melanoma skin cancer (basal-cell	Rate	1990	0.0050642	0.009703031	0.00208301
DALYs (Dis	Mauritania	Both	55+ years	Non-melanoma skin cancer (basal-cell	Rate	1990	0.0069271	0.013638308	0.002812966
DALYs (Dis	Georgia	Male	55+ years	Non-melanoma skin cancer (basal-cell	Number	1990	0.240471	0.454048412	0.098945332
DALYs (Dis	Georgia	Female	55+ years	Non-melanoma skin cancer (basal-cell	Number	1990	0.4173443	0.829582629	0.171961092
DALYs (Dis	Georgia	Both	55+ years	Non-melanoma skin cancer (basal-cell	Number	1990	0.6578154	1.285153172	0.264016364
DALYs (Dis	Georgia	Male	55+ years	Non-melanoma skin cancer (basal-cell	Percent	1990	4.94E-07	9.47E-07	2.11E-07
DALYs (Dis	Georgia	Female	55+ years	Non-melanoma skin cancer (basal-cell	Percent	1990	7.68E-07	1.48E-06	3.28E-07
DALYs (Dis	Georgia	Both	55+ years	Non-melanoma skin cancer (basal-cell	Percent	1990	6.39E-07	1.23E-06	2.76E-07
DALYs (Dis	Georgia	Male	55+ years	Non-melanoma skin cancer (basal-cell	Rate	1990	0.0544573	0.10282421	0.022407249
DALYs (Dis	Georgia	Female	55+ years	Non-melanoma skin cancer (basal-cell	Rate	1990	0.0623675	0.123971932	0.02569768
DALYs (Dis	Georgia	Both	55+ years	Non-melanoma skin cancer (basal-cell	Rate	1990	0.0592228	0.115701695	0.023769261
DALYs (Dis	Paraguay	Male	55+ years	Non-melanoma skin cancer (basal-cell	Number	2021	0.0008131	0.001815254	0.000252989
DALYs (Dis	Paraguay	Female	55+ years	Non-melanoma skin cancer (basal-cell	Number	2021	0.0007584	0.001817608	0.000187463
DALYs (Dis	Paraguay	Both	55+ years	Non-melanoma skin cancer (basal-cell	Number	2021	0.0015715	0.00353621	0.000465947
DALYs (Dis	Paraguay	Male	55+ years	Non-melanoma skin cancer (basal-cell	Percent	2021	1.55E-09	3.50E-09	4.71E-10
DALYs (Dis	Paraguay	Female	55+ years	Non-melanoma skin cancer (basal-cell	Percent	2021	1.85E-09	4.41E-09	4.70E-10
DALYs (Dis	Paraguay	Both	55+ years	Non-melanoma skin cancer (basal-cell	Percent	2021	1.68E-09	3.83E-09	5.12E-10
DALYs (Dis	Paraguay	Male	55+ years	Non-melanoma skin cancer (basal-cell	Rate	2021	0.0001716	0.000383119	5.34E-05
DALYs (Dis	Paraguay	Female	55+ years	Non-melanoma skin cancer (basal-cell	Rate	2021	0.0001469	0.000352048	3.63E-05
DALYs (Dis	Paraguay	Both	55+ years	Non-melanoma skin cancer (basal-cell	Rate	2021	0.0001587	0.000357155	4.71E-05
DALYs (Dis	Zimbabwe	Male	55+ years	Non-melanoma skin cancer (basal-cell	Number	2021	0.1272804	0.246003685	0.05428911
DALYs (Dis	Zimbabwe	Female	55+ years	Non-melanoma skin cancer (basal-cell	Number	2021	0.1179292	0.231884731	0.050033011
DALYs (Dis	Zimbabwe	Both	55+ years	Non-melanoma skin cancer (basal-cell	Number	2021	0.2452096	0.478420463	0.100976114
DALYs (Dis	Zimbabwe	Male	55+ years	Non-melanoma skin cancer (basal-cell	Percent	2021	1.06E-07	2.02E-07	4.69E-08
DALYs (Dis	Zimbabwe	Female	55+ years	Non-melanoma skin cancer (basal-cell	Percent	2021	1.02E-07	1.94E-07	4.51E-08
DALYs (Dis	Zimbabwe	Both	55+ years	Non-melanoma skin cancer (basal-cell	Percent	2021	1.04E-07	2.01E-07	4.63E-08
DALYs (Dis	Zimbabwe	Male	55+ years	Non-melanoma skin cancer (basal-cell	Rate	2021	0.0283629	0.054818913	0.012097664
DALYs (Dis	Zimbabwe	Female	55+ years	Non-melanoma skin cancer (basal-cell	Rate	2021	0.0179084	0.035213312	0.007597861
DALYs (Dis	Zimbabwe	Both	55+ years	Non-melanoma skin cancer (basal-cell	Rate	2021	0.0221454	0.043207156	0.009119365
DALYs (Dis	Grenada	Male	55+ years	Non-melanoma skin cancer (basal-cell	Number	2021	0.0012274	0.002447324	0.000499161
DALYs (Dis	Grenada	Female	55+ years	Non-melanoma skin cancer (basal-cell	Number	2021	0.0005832	0.001131015	0.000241346
DALYs (Dis	Grenada	Both	55+ years	Non-melanoma skin cancer (basal-cell	Number	2021	0.0018106	0.003530502	0.000732133
DALYs (Dis	Grenada	Male	55+ years	Non-melanoma skin cancer (basal-cell	Percent	2021	1.00E-07	1.96E-07	4.14E-08
DALYs (Dis	Grenada	Female	55+ years	Non-melanoma skin cancer (basal-cell	Percent	2021	5.87E-08	1.10E-07	2.46E-08
DALYs (Dis	Grenada	Both	55+ years	Non-melanoma skin cancer (basal-cell	Percent	2021	8.15E-08	1.57E-07	3.41E-08
DALYs (Dis	Grenada	Male	55+ years	Non-melanoma skin cancer (basal-cell	Rate	2021	0.0125626	0.025048697	0.00510898
DALYs (Dis	Grenada	Female	55+ years	Non-melanoma skin cancer (basal-cell	Rate	2021	0.0056533	0.010962767	0.002339328

DALYs (Dis	Grenada	Both	55+ years	Non-melanoma skin cancer (basal-cell	Rate	2021	0.0090139	0.017575931	0.003644785
DALYs (Dis	Malta	Male	55+ years	Non-melanoma skin cancer (basal-cell	Number	2021	0.0972078	0.211361618	0.04094256
DALYs (Dis	Malta	Female	55+ years	Non-melanoma skin cancer (basal-cell	Number	2021	0.078229	0.152036517	0.032434945
DALYs (Dis	Malta	Both	55+ years	Non-melanoma skin cancer (basal-cell	Number	2021	0.1754368	0.358785897	0.072405258
DALYs (Dis	Malta	Male	55+ years	Non-melanoma skin cancer (basal-cell	Percent	2021	2.22E-06	4.50E-06	9.46E-07
DALYs (Dis	Malta	Female	55+ years	Non-melanoma skin cancer (basal-cell	Percent	2021	1.64E-06	3.10E-06	6.83E-07
DALYs (Dis	Malta	Both	55+ years	Non-melanoma skin cancer (basal-cell	Percent	2021	1.92E-06	3.71E-06	8.34E-07
DALYs (Dis	Malta	Male	55+ years	Non-melanoma skin cancer (basal-cell	Rate	2021	0.130159	0.283008281	0.054821134
DALYs (Dis	Malta	Female	55+ years	Non-melanoma skin cancer (basal-cell	Rate	2021	0.0941165	0.182913617	0.039022159
DALYs (Dis	Malta	Both	55+ years	Non-melanoma skin cancer (basal-cell	Rate	2021	0.1111744	0.227362881	0.045883264
DALYs (Dis	Republic o	Male	55+ years	Non-melanoma skin cancer (basal-cell	Number	1990	0.1514321	0.298586962	0.064430803
DALYs (Dis	Republic o	Female	55+ years	Non-melanoma skin cancer (basal-cell	Number	1990	0.2270039	0.432974867	0.094266382
DALYs (Dis	Republic o	Both	55+ years	Non-melanoma skin cancer (basal-cell	Number	1990	0.378436	0.723445565	0.155398287
DALYs (Dis	Republic o	Male	55+ years	Non-melanoma skin cancer (basal-cell	Percent	1990	4.03E-07	7.81E-07	1.77E-07
DALYs (Dis	Republic o	Female	55+ years	Non-melanoma skin cancer (basal-cell	Percent	1990	5.26E-07	9.81E-07	2.27E-07
DALYs (Dis	Republic o	Both	55+ years	Non-melanoma skin cancer (basal-cell	Percent	1990	4.69E-07	8.77E-07	2.03E-07
DALYs (Dis	Republic o	Male	55+ years	Non-melanoma skin cancer (basal-cell	Rate	1990	0.048578	0.095783825	0.020668782
DALYs (Dis	Republic o	Female	55+ years	Non-melanoma skin cancer (basal-cell	Rate	1990	0.0493182	0.094066939	0.020480057
DALYs (Dis	Republic o	Both	55+ years	Non-melanoma skin cancer (basal-cell	Rate	1990	0.0490193	0.093708886	0.020128951
DALYs (Dis	Ireland	Male	55+ years	Non-melanoma skin cancer (basal-cell	Number	2021	0.8981555	1.919322064	0.359361225
DALYs (Dis	Ireland	Female	55+ years	Non-melanoma skin cancer (basal-cell	Number	2021	0.7412197	1.571863548	0.299821382
DALYs (Dis	Ireland	Both	55+ years	Non-melanoma skin cancer (basal-cell	Number	2021	1.6393752	3.260967404	0.653942741
DALYs (Dis	Ireland	Male	55+ years	Non-melanoma skin cancer (basal-cell	Percent	2021	2.43E-06	4.90E-06	1.03E-06
DALYs (Dis	Ireland	Female	55+ years	Non-melanoma skin cancer (basal-cell	Percent	2021	2.06E-06	4.18E-06	8.40E-07
DALYs (Dis	Ireland	Both	55+ years	Non-melanoma skin cancer (basal-cell	Percent	2021	2.24E-06	4.45E-06	9.40E-07
DALYs (Dis	Ireland	Male	55+ years	Non-melanoma skin cancer (basal-cell	Rate	2021	0.1423881	0.304277743	0.056970961
DALYs (Dis	Ireland	Female	55+ years	Non-melanoma skin cancer (basal-cell	Rate	2021	0.1087488	0.230617616	0.04398861
DALYs (Dis	Ireland	Both	55+ years	Non-melanoma skin cancer (basal-cell	Rate	2021	0.1249173	0.248479589	0.049829208
DALYs (Dis	Saint Luci	Male	55+ years	Non-melanoma skin cancer (basal-cell	Number	2021	0.0029827	0.005846334	0.001213176
DALYs (Dis	Saint Luci	Female	55+ years	Non-melanoma skin cancer (basal-cell	Number	2021	0.0013643	0.002599462	0.000568059
DALYs (Dis	Saint Luci	Both	55+ years	Non-melanoma skin cancer (basal-cell	Number	2021	0.0043469	0.008427127	0.001792977
DALYs (Dis	Saint Luci	Male	55+ years	Non-melanoma skin cancer (basal-cell	Percent	2021	1.46E-07	2.88E-07	5.86E-08
DALYs (Dis	Saint Luci	Female	55+ years	Non-melanoma skin cancer (basal-cell	Percent	2021	8.25E-08	1.54E-07	3.54E-08
DALYs (Dis	Saint Luci	Both	55+ years	Non-melanoma skin cancer (basal-cell	Percent	2021	1.17E-07	2.28E-07	4.90E-08
DALYs (Dis	Saint Luci	Male	55+ years	Non-melanoma skin cancer (basal-cell	Rate	2021	0.0147967	0.029003247	0.00601848
DALYs (Dis	Saint Luci	Female	55+ years	Non-melanoma skin cancer (basal-cell	Rate	2021	0.0062226	0.011856503	0.002590995
DALYs (Dis	Saint Luci	Both	55+ years	Non-melanoma skin cancer (basal-cell	Rate	2021	0.0103297	0.020025551	0.004260687
DALYs (Dis	Bahrain	Male	55+ years	Non-melanoma skin cancer (basal-cell	Number	2021	0.0101639	0.020182731	0.003927239
DALYs (Dis	Bahrain	Female	55+ years	Non-melanoma skin cancer (basal-cell	Number	2021	0.0046186	0.00933131	0.001730442
DALYs (Dis	Bahrain	Both	55+ years	Non-melanoma skin cancer (basal-cell	Number	2021	0.0147825	0.029217223	0.005587841
DALYs (Dis	Bahrain	Male	55+ years	Non-melanoma skin cancer (basal-cell	Percent	2021	1.32E-07	2.58E-07	5.28E-08
DALYs (Dis	Bahrain	Female	55+ years	Non-melanoma skin cancer (basal-cell	Percent	2021	9.07E-08	1.78E-07	3.50E-08
DALYs (Dis	Bahrain	Both	55+ years	Non-melanoma skin cancer (basal-cell	Percent	2021	1.16E-07	2.23E-07	4.53E-08
DALYs (Dis	Bahrain	Male	55+ years	Non-melanoma skin cancer (basal-cell	Rate	2021	0.0104874	0.020825073	0.004052228
DALYs (Dis	Bahrain	Female	55+ years	Non-melanoma skin cancer (basal-cell	Rate	2021	0.0070559	0.014255675	0.002643639
DALYs (Dis	Bahrain	Both	55+ years	Non-melanoma skin cancer (basal-cell	Rate	2021	0.009104	0.017993964	0.003441375
DALYs (Dis	Viet Nam	Male	55+ years	Non-melanoma skin cancer (basal-cell	Number	2021	0.257618	0.514273813	0.101088466
DALYs (Dis	Viet Nam	Female	55+ years	Non-melanoma skin cancer (basal-cell	Number	2021	0.3672163	0.725899987	0.14396851
DALYs (Dis	Viet Nam	Both	55+ years	Non-melanoma skin cancer (basal-cell	Number	2021	0.6248343	1.237051888	0.245791282
DALYs (Dis	Viet Nam	Male	55+ years	Non-melanoma skin cancer (basal-cell	Percent	2021	3.41E-08	6.74E-08	1.38E-08
DALYs (Dis	Viet Nam	Female	55+ years	Non-melanoma skin cancer (basal-cell	Percent	2021	5.83E-08	1.12E-07	2.41E-08
DALYs (Dis	Viet Nam	Both	55+ years	Non-melanoma skin cancer (basal-cell	Percent	2021	4.51E-08	8.75E-08	1.85E-08
DALYs (Dis	Viet Nam	Male	55+ years	Non-melanoma skin cancer (basal-cell	Rate	2021	0.0033726	0.006732615	0.0013234
DALYs (Dis	Viet Nam	Female	55+ years	Non-melanoma skin cancer (basal-cell	Rate	2021	0.0037357	0.007384501	0.001464576
DALYs (Dis	Viet Nam	Both	55+ years	Non-melanoma skin cancer (basal-cell	Rate	2021	0.0035769	0.007081578	0.001407047
DALYs (Dis	Comoros	Male	55+ years	Non-melanoma skin cancer (basal-cell	Number	2021	0.003989	0.007692585	0.001602031

DALYs (Dis	Comoros	Female	55+ years	Non-melanoma skin cancer (basal-cell	Number	2021	0.0028899	0.005759739	0.001178175
DALYs (Dis	Comoros	Both	55+ years	Non-melanoma skin cancer (basal-cell	Number	2021	0.0068789	0.013698162	0.002826269
DALYs (Dis	Comoros	Male	55+ years	Non-melanoma skin cancer (basal-cell	Percent	2021	9.05E-08	1.71E-07	3.77E-08
DALYs (Dis	Comoros	Female	55+ years	Non-melanoma skin cancer (basal-cell	Percent	2021	6.47E-08	1.20E-07	2.76E-08
DALYs (Dis	Comoros	Both	55+ years	Non-melanoma skin cancer (basal-cell	Percent	2021	7.75E-08	1.46E-07	3.30E-08
DALYs (Dis	Comoros	Male	55+ years	Non-melanoma skin cancer (basal-cell	Rate	2021	0.0107782	0.020785426	0.004328699
DALYs (Dis	Comoros	Female	55+ years	Non-melanoma skin cancer (basal-cell	Rate	2021	0.0065949	0.013143778	0.002688606
DALYs (Dis	Comoros	Both	55+ years	Non-melanoma skin cancer (basal-cell	Rate	2021	0.0085103	0.016946764	0.003496536
DALYs (Dis	Afghanista	Male	55+ years	Non-melanoma skin cancer (basal-cell	Number	2021	0.0760354	0.148161736	0.030028722
DALYs (Dis	Afghanista	Female	55+ years	Non-melanoma skin cancer (basal-cell	Number	2021	0.0517749	0.100542647	0.021489575
DALYs (Dis	Afghanista	Both	55+ years	Non-melanoma skin cancer (basal-cell	Number	2021	0.1278103	0.246938189	0.051144386
DALYs (Dis	Afghanista	Male	55+ years	Non-melanoma skin cancer (basal-cell	Percent	2021	7.22E-08	1.41E-07	2.92E-08
DALYs (Dis	Afghanista	Female	55+ years	Non-melanoma skin cancer (basal-cell	Percent	2021	4.50E-08	8.76E-08	1.85E-08
DALYs (Dis	Afghanista	Both	55+ years	Non-melanoma skin cancer (basal-cell	Percent	2021	5.80E-08	1.12E-07	2.35E-08
DALYs (Dis	Afghanista	Male	55+ years	Non-melanoma skin cancer (basal-cell	Rate	2021	0.0145607	0.028372884	0.005750482
DALYs (Dis	Afghanista	Female	55+ years	Non-melanoma skin cancer (basal-cell	Rate	2021	0.0073278	0.014229953	0.003041452
DALYs (Dis	Afghanista	Both	55+ years	Non-melanoma skin cancer (basal-cell	Rate	2021	0.0104016	0.020096678	0.004162306
DALYs (Dis	United Rep	Male	55+ years	Non-melanoma skin cancer (basal-cell	Number	2021	0.2057052	0.397305746	0.088291812
DALYs (Dis	United Rep	Female	55+ years	Non-melanoma skin cancer (basal-cell	Number	2021	0.1382593	0.270935896	0.056710425
DALYs (Dis	United Rep	Both	55+ years	Non-melanoma skin cancer (basal-cell	Number	2021	0.3439645	0.672606418	0.147347107
DALYs (Dis	United Rep	Male	55+ years	Non-melanoma skin cancer (basal-cell	Percent	2021	7.32E-08	1.41E-07	3.22E-08
DALYs (Dis	United Rep	Female	55+ years	Non-melanoma skin cancer (basal-cell	Percent	2021	5.98E-08	1.16E-07	2.53E-08
DALYs (Dis	United Rep	Both	55+ years	Non-melanoma skin cancer (basal-cell	Percent	2021	6.72E-08	1.30E-07	2.88E-08
DALYs (Dis	United Rep	Male	55+ years	Non-melanoma skin cancer (basal-cell	Rate	2021	0.010287	0.019868624	0.004415332
DALYs (Dis	United Rep	Female	55+ years	Non-melanoma skin cancer (basal-cell	Rate	2021	0.0065139	0.012764839	0.002671848
DALYs (Dis	United Rep	Both	55+ years	Non-melanoma skin cancer (basal-cell	Rate	2021	0.0083442	0.01631676	0.003574494
DALYs (Dis	Guatemala	Male	55+ years	Non-melanoma skin cancer (basal-cell	Number	2021	0.5375929	1.087966103	0.236883721
DALYs (Dis	Guatemala	Female	55+ years	Non-melanoma skin cancer (basal-cell	Number	2021	0.554107	1.078739651	0.239247918
DALYs (Dis	Guatemala	Both	55+ years	Non-melanoma skin cancer (basal-cell	Number	2021	1.0916999	2.139485044	0.468240035
DALYs (Dis	Guatemala	Male	55+ years	Non-melanoma skin cancer (basal-cell	Percent	2021	5.52E-07	1.12E-06	2.37E-07
DALYs (Dis	Guatemala	Female	55+ years	Non-melanoma skin cancer (basal-cell	Percent	2021	6.42E-07	1.20E-06	2.91E-07
DALYs (Dis	Guatemala	Both	55+ years	Non-melanoma skin cancer (basal-cell	Percent	2021	5.94E-07	1.13E-06	2.60E-07
DALYs (Dis	Guatemala	Male	55+ years	Non-melanoma skin cancer (basal-cell	Rate	2021	0.0630729	0.127645163	0.027792282
DALYs (Dis	Guatemala	Female	55+ years	Non-melanoma skin cancer (basal-cell	Rate	2021	0.0566319	0.110251359	0.024452061
DALYs (Dis	Guatemala	Both	55+ years	Non-melanoma skin cancer (basal-cell	Rate	2021	0.0596305	0.116862392	0.025576085
DALYs (Dis	Saudi Arab	Male	55+ years	Non-melanoma skin cancer (basal-cell	Number	2021	0.1975098	0.385802374	0.087935309
DALYs (Dis	Saudi Arab	Female	55+ years	Non-melanoma skin cancer (basal-cell	Number	2021	0.0833523	0.161535749	0.036931509
DALYs (Dis	Saudi Arab	Both	55+ years	Non-melanoma skin cancer (basal-cell	Number	2021	0.2808622	0.55119899	0.124900188
DALYs (Dis	Saudi Arab	Male	55+ years	Non-melanoma skin cancer (basal-cell	Percent	2021	1.29E-07	2.46E-07	5.87E-08
DALYs (Dis	Saudi Arab	Female	55+ years	Non-melanoma skin cancer (basal-cell	Percent	2021	9.53E-08	1.81E-07	4.37E-08
DALYs (Dis	Saudi Arab	Both	55+ years	Non-melanoma skin cancer (basal-cell	Percent	2021	1.17E-07	2.19E-07	5.32E-08
DALYs (Dis	Saudi Arab	Male	55+ years	Non-melanoma skin cancer (basal-cell	Rate	2021	0.0109421	0.021373647	0.004871661
DALYs (Dis	Saudi Arab	Female	55+ years	Non-melanoma skin cancer (basal-cell	Rate	2021	0.0069134	0.013398066	0.003063166
DALYs (Dis	Saudi Arab	Both	55+ years	Non-melanoma skin cancer (basal-cell	Rate	2021	0.0093288	0.018307987	0.00414854
DALYs (Dis	Czechia	Male	55+ years	Non-melanoma skin cancer (basal-cell	Number	1990	1.2180913	2.503377034	0.506730358
DALYs (Dis	Czechia	Female	55+ years	Non-melanoma skin cancer (basal-cell	Number	1990	1.504928	2.997669793	0.658344295
DALYs (Dis	Czechia	Both	55+ years	Non-melanoma skin cancer (basal-cell	Number	1990	2.7230194	5.56677069	1.181793469
DALYs (Dis	Czechia	Male	55+ years	Non-melanoma skin cancer (basal-cell	Percent	1990	9.05E-07	1.85E-06	3.85E-07
DALYs (Dis	Czechia	Female	55+ years	Non-melanoma skin cancer (basal-cell	Percent	1990	1.17E-06	2.30E-06	5.22E-07
DALYs (Dis	Czechia	Both	55+ years	Non-melanoma skin cancer (basal-cell	Percent	1990	1.03E-06	2.03E-06	4.66E-07
DALYs (Dis	Czechia	Male	55+ years	Non-melanoma skin cancer (basal-cell	Rate	1990	0.1241015	0.255048822	0.051626654
DALYs (Dis	Czechia	Female	55+ years	Non-melanoma skin cancer (basal-cell	Rate	1990	0.1085561	0.216233195	0.04748885
DALYs (Dis	Czechia	Both	55+ years	Non-melanoma skin cancer (basal-cell	Rate	1990	0.115	0.23509891	0.049910149
DALYs (Dis	Sao Tome a	Male	55+ years	Non-melanoma skin cancer (basal-cell	Number	1990	0.0004575	0.000892771	0.000187661
DALYs (Dis	Sao Tome a	Female	55+ years	Non-melanoma skin cancer (basal-cell	Number	1990	0.0003192	0.000625492	0.000130817
DALYs (Dis	Sao Tome a	Both	55+ years	Non-melanoma skin cancer (basal-cell	Number	1990	0.0007767	0.001521422	0.000314941

DALYs (Dis	Sao Tome a	Male	55+ years	Non-melanoma skin cancer (basal-cell	Percent	1990	8.70E-08	1.68E-07	3.72E-08
DALYs (Dis	Sao Tome a	Female	55+ years	Non-melanoma skin cancer (basal-cell	Percent	1990	5.52E-08	1.06E-07	2.37E-08
DALYs (Dis	Sao Tome a	Both	55+ years	Non-melanoma skin cancer (basal-cell	Percent	1990	7.03E-08	1.34E-07	3.02E-08
DALYs (Dis	Sao Tome a	Male	55+ years	Non-melanoma skin cancer (basal-cell	Rate	1990	0.0086721	0.016924096	0.003557455
DALYs (Dis	Sao Tome a	Female	55+ years	Non-melanoma skin cancer (basal-cell	Rate	1990	0.0053896	0.0105611	0.00220877
DALYs (Dis	Sao Tome a	Both	55+ years	Non-melanoma skin cancer (basal-cell	Rate	1990	0.0069359	0.013586856	0.002812538
DALYs (Dis	United Sta	Male	55+ years	Non-melanoma skin cancer (basal-cell	Number	2021	0.0022784	0.004397186	0.000911781
DALYs (Dis	United Sta	Female	55+ years	Non-melanoma skin cancer (basal-cell	Number	2021	0.0011092	0.002123488	0.0004626
DALYs (Dis	United Sta	Both	55+ years	Non-melanoma skin cancer (basal-cell	Number	2021	0.0033876	0.006509282	0.00140289
DALYs (Dis	United Sta	Male	55+ years	Non-melanoma skin cancer (basal-cell	Percent	2021	1.89E-07	3.63E-07	8.03E-08
DALYs (Dis	United Sta	Female	55+ years	Non-melanoma skin cancer (basal-cell	Percent	2021	1.14E-07	2.25E-07	4.96E-08
DALYs (Dis	United Sta	Both	55+ years	Non-melanoma skin cancer (basal-cell	Percent	2021	1.56E-07	3.03E-07	6.69E-08
DALYs (Dis	United Sta	Male	55+ years	Non-melanoma skin cancer (basal-cell	Rate	2021	0.0152684	0.029467681	0.006110291
DALYs (Dis	United Sta	Female	55+ years	Non-melanoma skin cancer (basal-cell	Rate	2021	0.0064239	0.012297821	0.002679072
DALYs (Dis	United Sta	Both	55+ years	Non-melanoma skin cancer (basal-cell	Rate	2021	0.010524	0.020221914	0.004358257
DALYs (Dis	Republic o	Male	55+ years	Non-melanoma skin cancer (basal-cell	Number	2021	1.1340471	2.263271738	0.502119942
DALYs (Dis	Republic o	Female	55+ years	Non-melanoma skin cancer (basal-cell	Number	2021	1.6947445	3.442712049	0.739094951
DALYs (Dis	Republic o	Both	55+ years	Non-melanoma skin cancer (basal-cell	Number	2021	2.8287916	5.707641516	1.252824796
DALYs (Dis	Republic o	Male	55+ years	Non-melanoma skin cancer (basal-cell	Percent	2021	2.58E-07	4.98E-07	1.24E-07
DALYs (Dis	Republic o	Female	55+ years	Non-melanoma skin cancer (basal-cell	Percent	2021	4.39E-07	8.31E-07	1.97E-07
DALYs (Dis	Republic o	Both	55+ years	Non-melanoma skin cancer (basal-cell	Percent	2021	3.43E-07	6.55E-07	1.57E-07
DALYs (Dis	Republic o	Male	55+ years	Non-melanoma skin cancer (basal-cell	Rate	2021	0.0144993	0.028936924	0.006419824
DALYs (Dis	Republic o	Female	55+ years	Non-melanoma skin cancer (basal-cell	Rate	2021	0.019037	0.038671897	0.008302235
DALYs (Dis	Republic o	Both	55+ years	Non-melanoma skin cancer (basal-cell	Rate	2021	0.0169148	0.03412894	0.007491287
DALYs (Dis	Uganda	Male	55+ years	Non-melanoma skin cancer (basal-cell	Number	2021	0.1243252	0.241700368	0.05446454
DALYs (Dis	Uganda	Female	55+ years	Non-melanoma skin cancer (basal-cell	Number	2021	0.1172364	0.235941246	0.049742933
DALYs (Dis	Uganda	Both	55+ years	Non-melanoma skin cancer (basal-cell	Number	2021	0.2415616	0.47958804	0.104427836
DALYs (Dis	Uganda	Male	55+ years	Non-melanoma skin cancer (basal-cell	Percent	2021	7.22E-08	1.36E-07	3.19E-08
DALYs (Dis	Uganda	Female	55+ years	Non-melanoma skin cancer (basal-cell	Percent	2021	8.11E-08	1.56E-07	3.51E-08
DALYs (Dis	Uganda	Both	55+ years	Non-melanoma skin cancer (basal-cell	Percent	2021	7.62E-08	1.43E-07	3.36E-08
DALYs (Dis	Uganda	Male	55+ years	Non-melanoma skin cancer (basal-cell	Rate	2021	0.0119551	0.023241899	0.005237308
DALYs (Dis	Uganda	Female	55+ years	Non-melanoma skin cancer (basal-cell	Rate	2021	0.0088045	0.01771918	0.003735693
DALYs (Dis	Uganda	Both	55+ years	Non-melanoma skin cancer (basal-cell	Rate	2021	0.0101861	0.020223049	0.004403465
DALYs (Dis	Belize	Male	55+ years	Non-melanoma skin cancer (basal-cell	Number	2021	0.0038352	0.007404459	0.001598707
DALYs (Dis	Belize	Female	55+ years	Non-melanoma skin cancer (basal-cell	Number	2021	0.0013949	0.00276323	0.000544824
DALYs (Dis	Belize	Both	55+ years	Non-melanoma skin cancer (basal-cell	Number	2021	0.0052302	0.010082336	0.002174645
DALYs (Dis	Belize	Male	55+ years	Non-melanoma skin cancer (basal-cell	Percent	2021	1.71E-07	3.34E-07	7.01E-08
DALYs (Dis	Belize	Female	55+ years	Non-melanoma skin cancer (basal-cell	Percent	2021	7.92E-08	1.49E-07	3.35E-08
DALYs (Dis	Belize	Both	55+ years	Non-melanoma skin cancer (basal-cell	Percent	2021	1.31E-07	2.47E-07	5.48E-08
DALYs (Dis	Belize	Male	55+ years	Non-melanoma skin cancer (basal-cell	Rate	2021	0.0150463	0.029048835	0.006271973
DALYs (Dis	Belize	Female	55+ years	Non-melanoma skin cancer (basal-cell	Rate	2021	0.0056712	0.01123415	0.00221503
DALYs (Dis	Belize	Both	55+ years	Non-melanoma skin cancer (basal-cell	Rate	2021	0.0104423	0.020129892	0.004341789
DALYs (Dis	Niue	Male	55+ years	Non-melanoma skin cancer (basal-cell	Number	2021	1.90E-07	4.99E-07	4.05E-08
DALYs (Dis	Niue	Female	55+ years	Non-melanoma skin cancer (basal-cell	Number	2021	2.42E-07	6.33E-07	5.37E-08
DALYs (Dis	Niue	Both	55+ years	Non-melanoma skin cancer (basal-cell	Number	2021	4.33E-07	1.13E-06	1.05E-07
DALYs (Dis	Niue	Male	55+ years	Non-melanoma skin cancer (basal-cell	Percent	2021	9.13E-10	2.30E-09	1.99E-10
DALYs (Dis	Niue	Female	55+ years	Non-melanoma skin cancer (basal-cell	Percent	2021	1.26E-09	3.10E-09	2.82E-10
DALYs (Dis	Niue	Both	55+ years	Non-melanoma skin cancer (basal-cell	Percent	2021	1.08E-09	2.69E-09	2.63E-10
DALYs (Dis	Niue	Male	55+ years	Non-melanoma skin cancer (basal-cell	Rate	2021	0.0001036	0.000271795	2.20E-05
DALYs (Dis	Niue	Female	55+ years	Non-melanoma skin cancer (basal-cell	Rate	2021	0.0001168	0.000304793	2.59E-05
DALYs (Dis	Niue	Both	55+ years	Non-melanoma skin cancer (basal-cell	Rate	2021	0.0001106	0.000288545	2.68E-05
DALYs (Dis	Cameroon	Male	55+ years	Non-melanoma skin cancer (basal-cell	Number	2021	0.0756875	0.150121288	0.031019723
DALYs (Dis	Cameroon	Female	55+ years	Non-melanoma skin cancer (basal-cell	Number	2021	0.0493167	0.09835608	0.019953294
DALYs (Dis	Cameroon	Both	55+ years	Non-melanoma skin cancer (basal-cell	Number	2021	0.1250041	0.245801132	0.05048723
DALYs (Dis	Cameroon	Male	55+ years	Non-melanoma skin cancer (basal-cell	Percent	2021	4.88E-08	9.53E-08	2.12E-08
DALYs (Dis	Cameroon	Female	55+ years	Non-melanoma skin cancer (basal-cell	Percent	2021	4.08E-08	7.90E-08	1.74E-08

DALYs (Dis	Cameroon	Both	55+ years	Non-melanoma skin cancer (basal-cell	Percent	2021	4.53E-08	8.75E-08	1.97E-08
DALYs (Dis	Cameroon	Male	55+ years	Non-melanoma skin cancer (basal-cell	Rate	2021	0.0076617	0.01519646	0.003140061
DALYs (Dis	Cameroon	Female	55+ years	Non-melanoma skin cancer (basal-cell	Rate	2021	0.0047076	0.009388635	0.001904653
DALYs (Dis	Cameroon	Both	55+ years	Non-melanoma skin cancer (basal-cell	Rate	2021	0.0061413	0.012075843	0.002480362
DALYs (Dis	Cook Islan	Male	55+ years	Non-melanoma skin cancer (basal-cell	Number	2021	2.63E-06	6.91E-06	6.01E-07
DALYs (Dis	Cook Islan	Female	55+ years	Non-melanoma skin cancer (basal-cell	Number	2021	2.99E-06	7.57E-06	6.81E-07
DALYs (Dis	Cook Islan	Both	55+ years	Non-melanoma skin cancer (basal-cell	Number	2021	5.62E-06	1.43E-05	1.44E-06
DALYs (Dis	Cook Islan	Male	55+ years	Non-melanoma skin cancer (basal-cell	Percent	2021	1.26E-09	3.17E-09	2.96E-10
DALYs (Dis	Cook Islan	Female	55+ years	Non-melanoma skin cancer (basal-cell	Percent	2021	1.90E-09	4.55E-09	4.57E-10
DALYs (Dis	Cook Islan	Both	55+ years	Non-melanoma skin cancer (basal-cell	Percent	2021	1.54E-09	3.84E-09	3.96E-10
DALYs (Dis	Cook Islan	Male	55+ years	Non-melanoma skin cancer (basal-cell	Rate	2021	0.0001125	0.000296124	2.58E-05
DALYs (Dis	Cook Islan	Female	55+ years	Non-melanoma skin cancer (basal-cell	Rate	2021	0.0001257	0.000318245	2.86E-05
DALYs (Dis	Cook Islan	Both	55+ years	Non-melanoma skin cancer (basal-cell	Rate	2021	0.0001192	0.000303107	3.05E-05
DALYs (Dis	New Zealan	Male	55+ years	Non-melanoma skin cancer (basal-cell	Number	2021	0.5927539	1.172273041	0.211788715
DALYs (Dis	New Zealan	Female	55+ years	Non-melanoma skin cancer (basal-cell	Number	2021	0.5050848	1.026731204	0.206183484
DALYs (Dis	New Zealan	Both	55+ years	Non-melanoma skin cancer (basal-cell	Number	2021	1.0978387	2.180825048	0.425119814
DALYs (Dis	New Zealan	Male	55+ years	Non-melanoma skin cancer (basal-cell	Percent	2021	1.49E-06	2.85E-06	5.68E-07
DALYs (Dis	New Zealan	Female	55+ years	Non-melanoma skin cancer (basal-cell	Percent	2021	1.28E-06	2.49E-06	5.28E-07
DALYs (Dis	New Zealan	Both	55+ years	Non-melanoma skin cancer (basal-cell	Percent	2021	1.38E-06	2.58E-06	5.82E-07
DALYs (Dis	New Zealan	Male	55+ years	Non-melanoma skin cancer (basal-cell	Rate	2021	0.0873578	0.172765029	0.031212595
DALYs (Dis	New Zealan	Female	55+ years	Non-melanoma skin cancer (basal-cell	Rate	2021	0.0675349	0.137284346	0.027568817
DALYs (Dis	New Zealan	Both	55+ years	Non-melanoma skin cancer (basal-cell	Rate	2021	0.0769645	0.152887718	0.029803215
DALYs (Dis	Cabo Verde	Male	55+ years	Non-melanoma skin cancer (basal-cell	Number	1990	0.0016859	0.003200717	0.000686983
DALYs (Dis	Cabo Verde	Female	55+ years	Non-melanoma skin cancer (basal-cell	Number	1990	0.0014864	0.002884064	0.000623045
DALYs (Dis	Cabo Verde	Both	55+ years	Non-melanoma skin cancer (basal-cell	Number	1990	0.0031723	0.0060442	0.001314283
DALYs (Dis	Cabo Verde	Male	55+ years	Non-melanoma skin cancer (basal-cell	Percent	1990	1.15E-07	2.14E-07	4.83E-08
DALYs (Dis	Cabo Verde	Female	55+ years	Non-melanoma skin cancer (basal-cell	Percent	1990	9.09E-08	1.73E-07	3.96E-08
DALYs (Dis	Cabo Verde	Both	55+ years	Non-melanoma skin cancer (basal-cell	Percent	1990	1.02E-07	1.95E-07	4.45E-08
DALYs (Dis	Cabo Verde	Male	55+ years	Non-melanoma skin cancer (basal-cell	Rate	1990	0.0099084	0.01881109	0.0040375
DALYs (Dis	Cabo Verde	Female	55+ years	Non-melanoma skin cancer (basal-cell	Rate	1990	0.0066196	0.012844322	0.002774761
DALYs (Dis	Cabo Verde	Both	55+ years	Non-melanoma skin cancer (basal-cell	Rate	1990	0.0080374	0.015313768	0.003329907
DALYs (Dis	Burkina Fa	Male	55+ years	Non-melanoma skin cancer (basal-cell	Number	2021	0.0558246	0.111240714	0.022716752
DALYs (Dis	Burkina Fa	Female	55+ years	Non-melanoma skin cancer (basal-cell	Number	2021	0.0378599	0.074650218	0.015591772
DALYs (Dis	Burkina Fa	Both	55+ years	Non-melanoma skin cancer (basal-cell	Number	2021	0.0936845	0.186474312	0.037893717
DALYs (Dis	Burkina Fa	Male	55+ years	Non-melanoma skin cancer (basal-cell	Percent	2021	5.13E-08	1.00E-07	2.15E-08
DALYs (Dis	Burkina Fa	Female	55+ years	Non-melanoma skin cancer (basal-cell	Percent	2021	4.30E-08	8.17E-08	1.81E-08
DALYs (Dis	Burkina Fa	Both	55+ years	Non-melanoma skin cancer (basal-cell	Percent	2021	4.76E-08	9.18E-08	2.02E-08
DALYs (Dis	Burkina Fa	Male	55+ years	Non-melanoma skin cancer (basal-cell	Rate	2021	0.0078362	0.015615097	0.003188799
DALYs (Dis	Burkina Fa	Female	55+ years	Non-melanoma skin cancer (basal-cell	Rate	2021	0.0047862	0.009437182	0.001971091
DALYs (Dis	Burkina Fa	Both	55+ years	Non-melanoma skin cancer (basal-cell	Rate	2021	0.0062315	0.012403388	0.00252051
DALYs (Dis	Nepal	Male	55+ years	Non-melanoma skin cancer (basal-cell	Number	2021	0.0020538	0.005326002	0.000396735
DALYs (Dis	Nepal	Female	55+ years	Non-melanoma skin cancer (basal-cell	Number	2021	0.0016232	0.004118631	0.000375155
DALYs (Dis	Nepal	Both	55+ years	Non-melanoma skin cancer (basal-cell	Number	2021	0.003677	0.008943396	0.000956223
DALYs (Dis	Nepal	Male	55+ years	Non-melanoma skin cancer (basal-cell	Percent	2021	8.29E-10	2.15E-09	1.65E-10
DALYs (Dis	Nepal	Female	55+ years	Non-melanoma skin cancer (basal-cell	Percent	2021	7.80E-10	1.96E-09	1.80E-10
DALYs (Dis	Nepal	Both	55+ years	Non-melanoma skin cancer (basal-cell	Percent	2021	8.06E-10	1.92E-09	2.09E-10
DALYs (Dis	Nepal	Male	55+ years	Non-melanoma skin cancer (basal-cell	Rate	2021	0.0001091	0.000282889	2.11E-05
DALYs (Dis	Nepal	Female	55+ years	Non-melanoma skin cancer (basal-cell	Rate	2021	7.82E-05	0.000198475	1.81E-05
DALYs (Dis	Nepal	Both	55+ years	Non-melanoma skin cancer (basal-cell	Rate	2021	9.29E-05	0.000225966	2.42E-05
DALYs (Dis	Cameroon	Male	55+ years	Non-melanoma skin cancer (basal-cell	Number	1990	0.0283445	0.055009951	0.011440111
DALYs (Dis	Cameroon	Female	55+ years	Non-melanoma skin cancer (basal-cell	Number	1990	0.0177286	0.034403564	0.007148383
DALYs (Dis	Cameroon	Both	55+ years	Non-melanoma skin cancer (basal-cell	Number	1990	0.0460731	0.090245378	0.018355667
DALYs (Dis	Cameroon	Male	55+ years	Non-melanoma skin cancer (basal-cell	Percent	1990	6.44E-08	1.23E-07	2.57E-08
DALYs (Dis	Cameroon	Female	55+ years	Non-melanoma skin cancer (basal-cell	Percent	1990	4.41E-08	8.48E-08	1.80E-08
DALYs (Dis	Cameroon	Both	55+ years	Non-melanoma skin cancer (basal-cell	Percent	1990	5.47E-08	1.05E-07	2.19E-08
DALYs (Dis	Cameroon	Male	55+ years	Non-melanoma skin cancer (basal-cell	Rate	1990	0.0077624	0.015064968	0.003132977

DALYs (Dis	Cameroon	Female	55+ years	Non-melanoma skin cancer (basal-cell	Rate	1990	0.0047239	0.00916713	0.001904749
DALYs (Dis	Cameroon	Both	55+ years	Non-melanoma skin cancer (basal-cell	Rate	1990	0.0062224	0.012188007	0.002479008
DALYs (Dis	New Zealan	Male	55+ years	Non-melanoma skin cancer (basal-cell	Number	1990	0.248643	0.490090897	0.102086708
DALYs (Dis	New Zealan	Female	55+ years	Non-melanoma skin cancer (basal-cell	Number	1990	0.2529396	0.516474024	0.106582654
DALYs (Dis	New Zealan	Both	55+ years	Non-melanoma skin cancer (basal-cell	Number	1990	0.5015826	0.962944029	0.214585661
DALYs (Dis	New Zealan	Male	55+ years	Non-melanoma skin cancer (basal-cell	Percent	1990	8.84E-07	1.69E-06	3.84E-07
DALYs (Dis	New Zealan	Female	55+ years	Non-melanoma skin cancer (basal-cell	Percent	1990	9.54E-07	1.89E-06	4.15E-07
DALYs (Dis	New Zealan	Both	55+ years	Non-melanoma skin cancer (basal-cell	Percent	1990	9.18E-07	1.73E-06	4.11E-07
DALYs (Dis	New Zealan	Male	55+ years	Non-melanoma skin cancer (basal-cell	Rate	1990	0.0826867	0.162980584	0.033949113
DALYs (Dis	New Zealan	Female	55+ years	Non-melanoma skin cancer (basal-cell	Rate	1990	0.0707981	0.144561709	0.029832615
DALYs (Dis	New Zealan	Both	55+ years	Non-melanoma skin cancer (basal-cell	Rate	1990	0.0762314	0.146349865	0.032613092
DALYs (Dis	Equatorial	Male	55+ years	Non-melanoma skin cancer (basal-cell	Number	2021	0.0040732	0.007985218	0.001674432
DALYs (Dis	Equatorial	Female	55+ years	Non-melanoma skin cancer (basal-cell	Number	2021	0.0033541	0.006753031	0.001396744
DALYs (Dis	Equatorial	Both	55+ years	Non-melanoma skin cancer (basal-cell	Number	2021	0.0074273	0.014541702	0.003049799
DALYs (Dis	Equatorial	Male	55+ years	Non-melanoma skin cancer (basal-cell	Percent	2021	7.73E-08	1.48E-07	3.31E-08
DALYs (Dis	Equatorial	Female	55+ years	Non-melanoma skin cancer (basal-cell	Percent	2021	6.52E-08	1.26E-07	2.90E-08
DALYs (Dis	Equatorial	Both	55+ years	Non-melanoma skin cancer (basal-cell	Percent	2021	7.13E-08	1.36E-07	3.12E-08
DALYs (Dis	Equatorial	Male	55+ years	Non-melanoma skin cancer (basal-cell	Rate	2021	0.0122052	0.023927477	0.005017387
DALYs (Dis	Equatorial	Female	55+ years	Non-melanoma skin cancer (basal-cell	Rate	2021	0.0073356	0.014769001	0.003054705
DALYs (Dis	Equatorial	Both	55+ years	Non-melanoma skin cancer (basal-cell	Rate	2021	0.0093902	0.018384657	0.003855773
DALYs (Dis	Nauru	Male	55+ years	Non-melanoma skin cancer (basal-cell	Number	2021	3.56E-07	9.35E-07	7.23E-08
DALYs (Dis	Nauru	Female	55+ years	Non-melanoma skin cancer (basal-cell	Number	2021	4.88E-07	1.28E-06	1.09E-07
DALYs (Dis	Nauru	Both	55+ years	Non-melanoma skin cancer (basal-cell	Number	2021	8.43E-07	2.24E-06	1.92E-07
DALYs (Dis	Nauru	Male	55+ years	Non-melanoma skin cancer (basal-cell	Percent	2021	5.29E-10	1.36E-09	1.09E-10
DALYs (Dis	Nauru	Female	55+ years	Non-melanoma skin cancer (basal-cell	Percent	2021	7.54E-10	1.93E-09	1.75E-10
DALYs (Dis	Nauru	Both	55+ years	Non-melanoma skin cancer (basal-cell	Percent	2021	6.39E-10	1.71E-09	1.46E-10
DALYs (Dis	Nauru	Male	55+ years	Non-melanoma skin cancer (basal-cell	Rate	2021	8.78E-05	0.000230649	1.78E-05
DALYs (Dis	Nauru	Female	55+ years	Non-melanoma skin cancer (basal-cell	Rate	2021	9.39E-05	0.000246559	2.11E-05
DALYs (Dis	Nauru	Both	55+ years	Non-melanoma skin cancer (basal-cell	Rate	2021	9.12E-05	0.000242689	2.08E-05
DALYs (Dis	Suriname	Male	55+ years	Non-melanoma skin cancer (basal-cell	Number	1990	0.0030335	0.00590592	0.001230249
DALYs (Dis	Suriname	Female	55+ years	Non-melanoma skin cancer (basal-cell	Number	1990	0.0012584	0.002406076	0.000521019
DALYs (Dis	Suriname	Both	55+ years	Non-melanoma skin cancer (basal-cell	Number	1990	0.0042919	0.008287845	0.001744491
DALYs (Dis	Suriname	Male	55+ years	Non-melanoma skin cancer (basal-cell	Percent	1990	1.55E-07	2.96E-07	6.61E-08
DALYs (Dis	Suriname	Female	55+ years	Non-melanoma skin cancer (basal-cell	Percent	1990	6.98E-08	1.31E-07	2.99E-08
DALYs (Dis	Suriname	Both	55+ years	Non-melanoma skin cancer (basal-cell	Percent	1990	1.14E-07	2.19E-07	4.84E-08
DALYs (Dis	Suriname	Male	55+ years	Non-melanoma skin cancer (basal-cell	Rate	1990	0.0142777	0.027797131	0.00579036
DALYs (Dis	Suriname	Female	55+ years	Non-melanoma skin cancer (basal-cell	Rate	1990	0.0056506	0.010803818	0.002339494
DALYs (Dis	Suriname	Both	55+ years	Non-melanoma skin cancer (basal-cell	Rate	1990	0.0098627	0.019045023	0.004008748
DALYs (Dis	Georgia	Male	55+ years	Non-melanoma skin cancer (basal-cell	Number	2021	0.253354	0.496366648	0.10344454
DALYs (Dis	Georgia	Female	55+ years	Non-melanoma skin cancer (basal-cell	Number	2021	0.4001837	0.815748087	0.164903555
DALYs (Dis	Georgia	Both	55+ years	Non-melanoma skin cancer (basal-cell	Number	2021	0.6535377	1.271436648	0.275589439
DALYs (Dis	Georgia	Male	55+ years	Non-melanoma skin cancer (basal-cell	Percent	2021	4.30E-07	8.32E-07	1.81E-07
DALYs (Dis	Georgia	Female	55+ years	Non-melanoma skin cancer (basal-cell	Percent	2021	6.86E-07	1.34E-06	2.89E-07
DALYs (Dis	Georgia	Both	55+ years	Non-melanoma skin cancer (basal-cell	Percent	2021	5.58E-07	1.05E-06	2.44E-07
DALYs (Dis	Georgia	Male	55+ years	Non-melanoma skin cancer (basal-cell	Rate	2021	0.0594885	0.116548809	0.024289178
DALYs (Dis	Georgia	Female	55+ years	Non-melanoma skin cancer (basal-cell	Rate	2021	0.0641339	0.130732804	0.026427649
DALYs (Dis	Georgia	Both	55+ years	Non-melanoma skin cancer (basal-cell	Rate	2021	0.0622495	0.121104367	0.0262499
DALYs (Dis	Micronesia	Male	55+ years	Non-melanoma skin cancer (basal-cell	Number	1990	3.58E-06	9.46E-06	7.48E-07
DALYs (Dis	Micronesia	Female	55+ years	Non-melanoma skin cancer (basal-cell	Number	1990	3.84E-06	9.90E-06	8.93E-07
DALYs (Dis	Micronesia	Both	55+ years	Non-melanoma skin cancer (basal-cell	Number	1990	7.42E-06	1.92E-05	1.80E-06
DALYs (Dis	Micronesia	Male	55+ years	Non-melanoma skin cancer (basal-cell	Percent	1990	6.27E-10	1.59E-09	1.35E-10
DALYs (Dis	Micronesia	Female	55+ years	Non-melanoma skin cancer (basal-cell	Percent	1990	7.64E-10	2.01E-09	1.85E-10
DALYs (Dis	Micronesia	Both	55+ years	Non-melanoma skin cancer (basal-cell	Percent	1990	6.91E-10	1.78E-09	1.70E-10
DALYs (Dis	Micronesia	Male	55+ years	Non-melanoma skin cancer (basal-cell	Rate	1990	9.17E-05	0.000242232	1.91E-05
DALYs (Dis	Micronesia	Female	55+ years	Non-melanoma skin cancer (basal-cell	Rate	1990	9.47E-05	0.00024417	2.20E-05
DALYs (Dis	Micronesia	Both	55+ years	Non-melanoma skin cancer (basal-cell	Rate	1990	9.32E-05	0.000241472	2.27E-05

DALYs (Dis	Kiribati	Male	55+ years	Non-melanoma skin cancer (basal-cell	Number	2021	4.83E-06	1.30E-05	9.83E-07
DALYs (Dis	Kiribati	Female	55+ years	Non-melanoma skin cancer (basal-cell	Number	2021	6.55E-06	1.76E-05	1.54E-06
DALYs (Dis	Kiribati	Both	55+ years	Non-melanoma skin cancer (basal-cell	Number	2021	1.14E-05	3.09E-05	2.77E-06
DALYs (Dis	Kiribati	Male	55+ years	Non-melanoma skin cancer (basal-cell	Percent	2021	6.52E-10	1.68E-09	1.34E-10
DALYs (Dis	Kiribati	Female	55+ years	Non-melanoma skin cancer (basal-cell	Percent	2021	8.59E-10	2.25E-09	2.10E-10
DALYs (Dis	Kiribati	Both	55+ years	Non-melanoma skin cancer (basal-cell	Percent	2021	7.57E-10	2.00E-09	1.87E-10
DALYs (Dis	Kiribati	Male	55+ years	Non-melanoma skin cancer (basal-cell	Rate	2021	8.81E-05	0.000237299	1.79E-05
DALYs (Dis	Kiribati	Female	55+ years	Non-melanoma skin cancer (basal-cell	Rate	2021	9.23E-05	0.00024802	2.16E-05
DALYs (Dis	Kiribati	Both	55+ years	Non-melanoma skin cancer (basal-cell	Rate	2021	9.05E-05	0.000245818	2.20E-05
DALYs (Dis	Panama	Male	55+ years	Non-melanoma skin cancer (basal-cell	Number	2021	0.193305	0.371571697	0.085729591
DALYs (Dis	Panama	Female	55+ years	Non-melanoma skin cancer (basal-cell	Number	2021	0.2017947	0.394267556	0.091438791
DALYs (Dis	Panama	Both	55+ years	Non-melanoma skin cancer (basal-cell	Number	2021	0.3950996	0.764330161	0.177038645
DALYs (Dis	Panama	Male	55+ years	Non-melanoma skin cancer (basal-cell	Percent	2021	7.50E-07	1.42E-06	3.31E-07
DALYs (Dis	Panama	Female	55+ years	Non-melanoma skin cancer (basal-cell	Percent	2021	9.20E-07	1.72E-06	4.19E-07
DALYs (Dis	Panama	Both	55+ years	Non-melanoma skin cancer (basal-cell	Percent	2021	8.28E-07	1.57E-06	3.81E-07
DALYs (Dis	Panama	Male	55+ years	Non-melanoma skin cancer (basal-cell	Rate	2021	0.0536182	0.103065072	0.023779331
DALYs (Dis	Panama	Female	55+ years	Non-melanoma skin cancer (basal-cell	Rate	2021	0.052682	0.1029303	0.023871663
DALYs (Dis	Panama	Both	55+ years	Non-melanoma skin cancer (basal-cell	Rate	2021	0.0531359	0.102792692	0.023809447
DALYs (Dis	Uganda	Male	55+ years	Non-melanoma skin cancer (basal-cell	Number	1990	0.072668	0.14466457	0.031134379
DALYs (Dis	Uganda	Female	55+ years	Non-melanoma skin cancer (basal-cell	Number	1990	0.0500957	0.094192421	0.020611862
DALYs (Dis	Uganda	Both	55+ years	Non-melanoma skin cancer (basal-cell	Number	1990	0.1227637	0.238952277	0.052081587
DALYs (Dis	Uganda	Male	55+ years	Non-melanoma skin cancer (basal-cell	Percent	1990	8.54E-08	1.65E-07	3.87E-08
DALYs (Dis	Uganda	Female	55+ years	Non-melanoma skin cancer (basal-cell	Percent	1990	8.17E-08	1.55E-07	3.56E-08
DALYs (Dis	Uganda	Both	55+ years	Non-melanoma skin cancer (basal-cell	Percent	1990	8.38E-08	1.59E-07	3.75E-08
DALYs (Dis	Uganda	Male	55+ years	Non-melanoma skin cancer (basal-cell	Rate	1990	0.0141569	0.028183004	0.006065482
DALYs (Dis	Uganda	Female	55+ years	Non-melanoma skin cancer (basal-cell	Rate	1990	0.0094057	0.017685014	0.003869962
DALYs (Dis	Uganda	Both	55+ years	Non-melanoma skin cancer (basal-cell	Rate	1990	0.0117374	0.022846225	0.00497952
DALYs (Dis	France	Male	55+ years	Non-melanoma skin cancer (basal-cell	Number	2021	13.280521	26.01123673	5.38371469
DALYs (Dis	France	Female	55+ years	Non-melanoma skin cancer (basal-cell	Number	2021	13.083665	27.41933137	5.119473583
DALYs (Dis	France	Both	55+ years	Non-melanoma skin cancer (basal-cell	Number	2021	26.364186	52.01577057	11.45525796
DALYs (Dis	France	Male	55+ years	Non-melanoma skin cancer (basal-cell	Percent	2021	1.95E-06	3.65E-06	8.00E-07
DALYs (Dis	France	Female	55+ years	Non-melanoma skin cancer (basal-cell	Percent	2021	1.92E-06	3.79E-06	7.98E-07
DALYs (Dis	France	Both	55+ years	Non-melanoma skin cancer (basal-cell	Percent	2021	1.93E-06	3.67E-06	8.65E-07
DALYs (Dis	France	Male	55+ years	Non-melanoma skin cancer (basal-cell	Rate	2021	0.1328617	0.260223028	0.053860051
DALYs (Dis	France	Female	55+ years	Non-melanoma skin cancer (basal-cell	Rate	2021	0.1080012	0.226337156	0.042259495
DALYs (Dis	France	Both	55+ years	Non-melanoma skin cancer (basal-cell	Rate	2021	0.1192403	0.235257725	0.051810016
DALYs (Dis	Indonesia	Male	55+ years	Non-melanoma skin cancer (basal-cell	Number	1990	0.2124851	0.409419306	0.083662433
DALYs (Dis	Indonesia	Female	55+ years	Non-melanoma skin cancer (basal-cell	Number	1990	0.1758972	0.341272177	0.068140249
DALYs (Dis	Indonesia	Both	55+ years	Non-melanoma skin cancer (basal-cell	Number	1990	0.3883823	0.737909055	0.151608396
DALYs (Dis	Indonesia	Male	55+ years	Non-melanoma skin cancer (basal-cell	Percent	1990	2.64E-08	5.23E-08	1.06E-08
DALYs (Dis	Indonesia	Female	55+ years	Non-melanoma skin cancer (basal-cell	Percent	1990	2.20E-08	4.21E-08	8.77E-09
DALYs (Dis	Indonesia	Both	55+ years	Non-melanoma skin cancer (basal-cell	Percent	1990	2.42E-08	4.74E-08	9.65E-09
DALYs (Dis	Indonesia	Male	55+ years	Non-melanoma skin cancer (basal-cell	Rate	1990	0.0027427	0.005284754	0.001079908
DALYs (Dis	Indonesia	Female	55+ years	Non-melanoma skin cancer (basal-cell	Rate	1990	0.0020919	0.004058646	0.000810371
DALYs (Dis	Indonesia	Both	55+ years	Non-melanoma skin cancer (basal-cell	Rate	1990	0.002404	0.004567484	0.00093842
DALYs (Dis	Marshall I	Male	55+ years	Non-melanoma skin cancer (basal-cell	Number	1990	1.16E-06	3.17E-06	2.37E-07
DALYs (Dis	Marshall I	Female	55+ years	Non-melanoma skin cancer (basal-cell	Number	1990	1.22E-06	3.16E-06	2.84E-07
DALYs (Dis	Marshall I	Both	55+ years	Non-melanoma skin cancer (basal-cell	Number	1990	2.38E-06	6.28E-06	5.96E-07
DALYs (Dis	Marshall I	Male	55+ years	Non-melanoma skin cancer (basal-cell	Percent	1990	5.60E-10	1.45E-09	1.17E-10
DALYs (Dis	Marshall I	Female	55+ years	Non-melanoma skin cancer (basal-cell	Percent	1990	7.66E-10	1.97E-09	1.80E-10
DALYs (Dis	Marshall I	Both	55+ years	Non-melanoma skin cancer (basal-cell	Percent	1990	6.49E-10	1.69E-09	1.64E-10
DALYs (Dis	Marshall I	Male	55+ years	Non-melanoma skin cancer (basal-cell	Rate	1990	9.37E-05	0.000255194	1.91E-05
DALYs (Dis	Marshall I	Female	55+ years	Non-melanoma skin cancer (basal-cell	Rate	1990	9.31E-05	0.000241594	2.17E-05
DALYs (Dis	Marshall I	Both	55+ years	Non-melanoma skin cancer (basal-cell	Rate	1990	9.34E-05	0.00024635	2.34E-05
DALYs (Dis	Malaysia	Male	55+ years	Non-melanoma skin cancer (basal-cell	Number	1990	0.06114	0.118871311	0.026682249
DALYs (Dis	Malaysia	Female	55+ years	Non-melanoma skin cancer (basal-cell	Number	1990	0.0585166	0.114066941	0.025295064

DALYs (Dis	Malaysia	Both	55+ years	Non-melanoma skin cancer (basal-cell	Number	1990	0.1196566	0.233312599	0.052263908
DALYs (Dis	Malaysia	Male	55+ years	Non-melanoma skin cancer (basal-cell	Percent	1990	8.34E-08	1.56E-07	3.75E-08
DALYs (Dis	Malaysia	Female	55+ years	Non-melanoma skin cancer (basal-cell	Percent	1990	9.03E-08	1.69E-07	4.10E-08
DALYs (Dis	Malaysia	Both	55+ years	Non-melanoma skin cancer (basal-cell	Percent	1990	8.67E-08	1.60E-07	3.98E-08
DALYs (Dis	Malaysia	Male	55+ years	Non-melanoma skin cancer (basal-cell	Rate	1990	0.0086142	0.016748125	0.00375934
DALYs (Dis	Malaysia	Female	55+ years	Non-melanoma skin cancer (basal-cell	Rate	1990	0.007638	0.014888751	0.003301674
DALYs (Dis	Malaysia	Both	55+ years	Non-melanoma skin cancer (basal-cell	Rate	1990	0.0081074	0.015808294	0.003541186
DALYs (Dis	United Ara	Male	55+ years	Non-melanoma skin cancer (basal-cell	Number	2021	0.0565786	0.114616037	0.022601089
DALYs (Dis	United Ara	Female	55+ years	Non-melanoma skin cancer (basal-cell	Number	2021	0.009962	0.020248522	0.003896973
DALYs (Dis	United Ara	Both	55+ years	Non-melanoma skin cancer (basal-cell	Number	2021	0.0665406	0.135496539	0.026715994
DALYs (Dis	United Ara	Male	55+ years	Non-melanoma skin cancer (basal-cell	Percent	2021	2.12E-07	4.23E-07	8.95E-08
DALYs (Dis	United Ara	Female	55+ years	Non-melanoma skin cancer (basal-cell	Percent	2021	8.57E-08	1.71E-07	3.54E-08
DALYs (Dis	United Ara	Both	55+ years	Non-melanoma skin cancer (basal-cell	Percent	2021	1.74E-07	3.46E-07	7.26E-08
DALYs (Dis	United Ara	Male	55+ years	Non-melanoma skin cancer (basal-cell	Rate	2021	0.0102792	0.020823462	0.00410617
DALYs (Dis	United Ara	Female	55+ years	Non-melanoma skin cancer (basal-cell	Rate	2021	0.0059417	0.012077058	0.002324316
DALYs (Dis	United Ara	Both	55+ years	Non-melanoma skin cancer (basal-cell	Rate	2021	0.0092665	0.018869313	0.003720482
DALYs (Dis	Guam	Male	55+ years	Non-melanoma skin cancer (basal-cell	Number	2021	2.07E-05	5.26E-05	4.71E-06
DALYs (Dis	Guam	Female	55+ years	Non-melanoma skin cancer (basal-cell	Number	2021	2.51E-05	6.25E-05	5.85E-06
DALYs (Dis	Guam	Both	55+ years	Non-melanoma skin cancer (basal-cell	Number	2021	4.58E-05	0.000115594	1.12E-05
DALYs (Dis	Guam	Male	55+ years	Non-melanoma skin cancer (basal-cell	Percent	2021	1.49E-09	3.78E-09	3.39E-10
DALYs (Dis	Guam	Female	55+ years	Non-melanoma skin cancer (basal-cell	Percent	2021	2.19E-09	5.30E-09	5.33E-10
DALYs (Dis	Guam	Both	55+ years	Non-melanoma skin cancer (basal-cell	Percent	2021	1.80E-09	4.50E-09	4.49E-10
DALYs (Dis	Guam	Male	55+ years	Non-melanoma skin cancer (basal-cell	Rate	2021	0.0001151	0.000291713	2.61E-05
DALYs (Dis	Guam	Female	55+ years	Non-melanoma skin cancer (basal-cell	Rate	2021	0.0001274	0.000317802	2.97E-05
DALYs (Dis	Guam	Both	55+ years	Non-melanoma skin cancer (basal-cell	Rate	2021	0.0001215	0.000306734	2.97E-05
DALYs (Dis	Jordan	Male	55+ years	Non-melanoma skin cancer (basal-cell	Number	2021	0.121957	0.234029635	0.050992661
DALYs (Dis	Jordan	Female	55+ years	Non-melanoma skin cancer (basal-cell	Number	2021	0.0679926	0.133083263	0.027169746
DALYs (Dis	Jordan	Both	55+ years	Non-melanoma skin cancer (basal-cell	Number	2021	0.1899497	0.363785272	0.078735014
DALYs (Dis	Jordan	Male	55+ years	Non-melanoma skin cancer (basal-cell	Percent	2021	2.40E-07	4.44E-07	1.02E-07
DALYs (Dis	Jordan	Female	55+ years	Non-melanoma skin cancer (basal-cell	Percent	2021	1.72E-07	3.26E-07	7.36E-08
DALYs (Dis	Jordan	Both	55+ years	Non-melanoma skin cancer (basal-cell	Percent	2021	2.10E-07	3.91E-07	9.07E-08
DALYs (Dis	Jordan	Male	55+ years	Non-melanoma skin cancer (basal-cell	Rate	2021	0.0184307	0.035367591	0.007706236
DALYs (Dis	Jordan	Female	55+ years	Non-melanoma skin cancer (basal-cell	Rate	2021	0.0114171	0.022346834	0.00456224
DALYs (Dis	Jordan	Both	55+ years	Non-melanoma skin cancer (basal-cell	Rate	2021	0.0151084	0.02893519	0.00626252
DALYs (Dis	Tunisia	Male	55+ years	Non-melanoma skin cancer (basal-cell	Number	1990	0.0916681	0.177595372	0.038788751
DALYs (Dis	Tunisia	Female	55+ years	Non-melanoma skin cancer (basal-cell	Number	1990	0.0583717	0.11315776	0.023396912
DALYs (Dis	Tunisia	Both	55+ years	Non-melanoma skin cancer (basal-cell	Number	1990	0.1500398	0.292690651	0.062616386
DALYs (Dis	Tunisia	Male	55+ years	Non-melanoma skin cancer (basal-cell	Percent	1990	2.57E-07	4.87E-07	1.13E-07
DALYs (Dis	Tunisia	Female	55+ years	Non-melanoma skin cancer (basal-cell	Percent	1990	2.18E-07	4.21E-07	9.42E-08
DALYs (Dis	Tunisia	Both	55+ years	Non-melanoma skin cancer (basal-cell	Percent	1990	2.40E-07	4.60E-07	1.04E-07
DALYs (Dis	Tunisia	Male	55+ years	Non-melanoma skin cancer (basal-cell	Rate	1990	0.0202458	0.039223763	0.008566894
DALYs (Dis	Tunisia	Female	55+ years	Non-melanoma skin cancer (basal-cell	Rate	1990	0.0139663	0.027074724	0.005598069
DALYs (Dis	Tunisia	Both	55+ years	Non-melanoma skin cancer (basal-cell	Rate	1990	0.0172317	0.033614743	0.007191325
DALYs (Dis	Kenya	Male	55+ years	Non-melanoma skin cancer (basal-cell	Number	1990	0.0602249	0.117064829	0.024800541
DALYs (Dis	Kenya	Female	55+ years	Non-melanoma skin cancer (basal-cell	Number	1990	0.053234	0.106647623	0.021557271
DALYs (Dis	Kenya	Both	55+ years	Non-melanoma skin cancer (basal-cell	Number	1990	0.113459	0.22559345	0.046836841
DALYs (Dis	Kenya	Male	55+ years	Non-melanoma skin cancer (basal-cell	Percent	1990	8.48E-08	1.62E-07	3.57E-08
DALYs (Dis	Kenya	Female	55+ years	Non-melanoma skin cancer (basal-cell	Percent	1990	8.39E-08	1.62E-07	3.55E-08
DALYs (Dis	Kenya	Both	55+ years	Non-melanoma skin cancer (basal-cell	Percent	1990	8.43E-08	1.63E-07	3.58E-08
DALYs (Dis	Kenya	Male	55+ years	Non-melanoma skin cancer (basal-cell	Rate	1990	0.0092386	0.017958017	0.003804461
DALYs (Dis	Kenya	Female	55+ years	Non-melanoma skin cancer (basal-cell	Rate	1990	0.0078183	0.01566299	0.003166046
DALYs (Dis	Kenya	Both	55+ years	Non-melanoma skin cancer (basal-cell	Rate	1990	0.008513	0.016926661	0.003514248
DALYs (Dis	Niger	Male	55+ years	Non-melanoma skin cancer (basal-cell	Number	2021	0.0539683	0.105499022	0.021756708
DALYs (Dis	Niger	Female	55+ years	Non-melanoma skin cancer (basal-cell	Number	2021	0.03171	0.063231867	0.012656915
DALYs (Dis	Niger	Both	55+ years	Non-melanoma skin cancer (basal-cell	Number	2021	0.0856783	0.166446053	0.034540535
DALYs (Dis	Niger	Male	55+ years	Non-melanoma skin cancer (basal-cell	Percent	2021	6.28E-08	1.22E-07	2.67E-08

DALYs (Dis	Niger	Female	55+ years	Non-melanoma skin cancer (basal-cell	Percent	2021	4.20E-08	8.17E-08	1.76E-08
DALYs (Dis	Niger	Both	55+ years	Non-melanoma skin cancer (basal-cell	Percent	2021	5.30E-08	1.03E-07	2.29E-08
DALYs (Dis	Niger	Male	55+ years	Non-melanoma skin cancer (basal-cell	Rate	2021	0.0078799	0.015403934	0.003176701
DALYs (Dis	Niger	Female	55+ years	Non-melanoma skin cancer (basal-cell	Rate	2021	0.0044831	0.008939501	0.001789391
DALYs (Dis	Niger	Both	55+ years	Non-melanoma skin cancer (basal-cell	Rate	2021	0.0061541	0.011955488	0.002480978
DALYs (Dis	Indonesia	Male	55+ years	Non-melanoma skin cancer (basal-cell	Number	2021	0.5515502	1.070717547	0.215632331
DALYs (Dis	Indonesia	Female	55+ years	Non-melanoma skin cancer (basal-cell	Number	2021	0.4456366	0.848311623	0.173814097
DALYs (Dis	Indonesia	Both	55+ years	Non-melanoma skin cancer (basal-cell	Number	2021	0.9971868	1.897213866	0.388549219
DALYs (Dis	Indonesia	Male	55+ years	Non-melanoma skin cancer (basal-cell	Percent	2021	2.43E-08	4.77E-08	9.70E-09
DALYs (Dis	Indonesia	Female	55+ years	Non-melanoma skin cancer (basal-cell	Percent	2021	2.39E-08	4.61E-08	9.12E-09
DALYs (Dis	Indonesia	Both	55+ years	Non-melanoma skin cancer (basal-cell	Percent	2021	2.41E-08	4.67E-08	9.53E-09
DALYs (Dis	Indonesia	Male	55+ years	Non-melanoma skin cancer (basal-cell	Rate	2021	0.0027019	0.005245154	0.001056324
DALYs (Dis	Indonesia	Female	55+ years	Non-melanoma skin cancer (basal-cell	Rate	2021	0.002076	0.003951869	0.000809715
DALYs (Dis	Indonesia	Both	55+ years	Non-melanoma skin cancer (basal-cell	Rate	2021	0.0023811	0.004530168	0.000927778
DALYs (Dis	Czechia	Male	55+ years	Non-melanoma skin cancer (basal-cell	Number	2021	1.7123459	3.722883024	0.693556873
DALYs (Dis	Czechia	Female	55+ years	Non-melanoma skin cancer (basal-cell	Number	2021	1.908939	3.753814788	0.74372502
DALYs (Dis	Czechia	Both	55+ years	Non-melanoma skin cancer (basal-cell	Number	2021	3.6212849	7.250290998	1.468558596
DALYs (Dis	Czechia	Male	55+ years	Non-melanoma skin cancer (basal-cell	Percent	2021	1.09E-06	2.28E-06	4.49E-07
DALYs (Dis	Czechia	Female	55+ years	Non-melanoma skin cancer (basal-cell	Percent	2021	1.35E-06	2.59E-06	5.40E-07
DALYs (Dis	Czechia	Both	55+ years	Non-melanoma skin cancer (basal-cell	Percent	2021	1.21E-06	2.33E-06	5.06E-07
DALYs (Dis	Czechia	Male	55+ years	Non-melanoma skin cancer (basal-cell	Rate	2021	0.1089894	0.236958349	0.044144307
DALYs (Dis	Czechia	Female	55+ years	Non-melanoma skin cancer (basal-cell	Rate	2021	0.0993023	0.195272068	0.038688303
DALYs (Dis	Czechia	Both	55+ years	Non-melanoma skin cancer (basal-cell	Rate	2021	0.1036589	0.207538738	0.042037319
DALYs (Dis	Angola	Male	55+ years	Non-melanoma skin cancer (basal-cell	Number	1990	0.0360596	0.074193694	0.014612654
DALYs (Dis	Angola	Female	55+ years	Non-melanoma skin cancer (basal-cell	Number	1990	0.0216236	0.043995359	0.009078848
DALYs (Dis	Angola	Both	55+ years	Non-melanoma skin cancer (basal-cell	Number	1990	0.0576832	0.115375138	0.023945007
DALYs (Dis	Angola	Male	55+ years	Non-melanoma skin cancer (basal-cell	Percent	1990	6.68E-08	1.32E-07	2.87E-08
DALYs (Dis	Angola	Female	55+ years	Non-melanoma skin cancer (basal-cell	Percent	1990	5.68E-08	1.12E-07	2.42E-08
DALYs (Dis	Angola	Both	55+ years	Non-melanoma skin cancer (basal-cell	Percent	1990	6.26E-08	1.23E-07	2.68E-08
DALYs (Dis	Angola	Male	55+ years	Non-melanoma skin cancer (basal-cell	Rate	1990	0.0115087	0.023679395	0.004663722
DALYs (Dis	Angola	Female	55+ years	Non-melanoma skin cancer (basal-cell	Rate	1990	0.0069847	0.01421106	0.002932583
DALYs (Dis	Angola	Both	55+ years	Non-melanoma skin cancer (basal-cell	Rate	1990	0.0092603	0.01852192	0.003844048
DALYs (Dis	Ecuador	Male	55+ years	Non-melanoma skin cancer (basal-cell	Number	2021	0.5862835	1.152088887	0.247113669
DALYs (Dis	Ecuador	Female	55+ years	Non-melanoma skin cancer (basal-cell	Number	2021	0.5386815	1.079125227	0.224937341
DALYs (Dis	Ecuador	Both	55+ years	Non-melanoma skin cancer (basal-cell	Number	2021	1.124965	2.250140697	0.477720437
DALYs (Dis	Ecuador	Male	55+ years	Non-melanoma skin cancer (basal-cell	Percent	2021	4.74E-07	9.15E-07	2.11E-07
DALYs (Dis	Ecuador	Female	55+ years	Non-melanoma skin cancer (basal-cell	Percent	2021	5.14E-07	9.83E-07	2.31E-07
DALYs (Dis	Ecuador	Both	55+ years	Non-melanoma skin cancer (basal-cell	Percent	2021	4.92E-07	9.45E-07	2.20E-07
DALYs (Dis	Ecuador	Male	55+ years	Non-melanoma skin cancer (basal-cell	Rate	2021	0.0445739	0.087590872	0.018787528
DALYs (Dis	Ecuador	Female	55+ years	Non-melanoma skin cancer (basal-cell	Rate	2021	0.0370938	0.074309043	0.015489285
DALYs (Dis	Ecuador	Both	55+ years	Non-melanoma skin cancer (basal-cell	Rate	2021	0.0406489	0.081305323	0.017261683
DALYs (Dis	Dominica	Male	55+ years	Non-melanoma skin cancer (basal-cell	Number	2021	0.0010001	0.001987555	0.000415285
DALYs (Dis	Dominica	Female	55+ years	Non-melanoma skin cancer (basal-cell	Number	2021	0.0004609	0.000887381	0.000185249
DALYs (Dis	Dominica	Both	55+ years	Non-melanoma skin cancer (basal-cell	Number	2021	0.001461	0.002875231	0.000608097
DALYs (Dis	Dominica	Male	55+ years	Non-melanoma skin cancer (basal-cell	Percent	2021	1.21E-07	2.40E-07	5.05E-08
DALYs (Dis	Dominica	Female	55+ years	Non-melanoma skin cancer (basal-cell	Percent	2021	6.71E-08	1.24E-07	2.85E-08
DALYs (Dis	Dominica	Both	55+ years	Non-melanoma skin cancer (basal-cell	Percent	2021	9.64E-08	1.87E-07	4.10E-08
DALYs (Dis	Dominica	Male	55+ years	Non-melanoma skin cancer (basal-cell	Rate	2021	0.0134273	0.026684552	0.005575542
DALYs (Dis	Dominica	Female	55+ years	Non-melanoma skin cancer (basal-cell	Rate	2021	0.0060375	0.011623356	0.002426487
DALYs (Dis	Dominica	Both	55+ years	Non-melanoma skin cancer (basal-cell	Rate	2021	0.0096868	0.019062979	0.004031725
DALYs (Dis	Bosnia and	Male	55+ years	Non-melanoma skin cancer (basal-cell	Number	2021	0.3758532	0.75502686	0.158409159
DALYs (Dis	Bosnia and	Female	55+ years	Non-melanoma skin cancer (basal-cell	Number	2021	0.410898	0.815630975	0.162387881
DALYs (Dis	Bosnia and	Both	55+ years	Non-melanoma skin cancer (basal-cell	Number	2021	0.7867512	1.549224172	0.324017555
DALYs (Dis	Bosnia and	Male	55+ years	Non-melanoma skin cancer (basal-cell	Percent	2021	7.17E-07	1.46E-06	3.08E-07
DALYs (Dis	Bosnia and	Female	55+ years	Non-melanoma skin cancer (basal-cell	Percent	2021	8.48E-07	1.64E-06	3.42E-07
DALYs (Dis	Bosnia and	Both	55+ years	Non-melanoma skin cancer (basal-cell	Percent	2021	7.80E-07	1.53E-06	3.35E-07

DALYs (Dis	Bosnia and	Male	55+ years	Non-melanoma skin cancer (basal-cell	Rate	2021	0.0765201	0.153716147	0.032250569
DALYs (Dis	Bosnia and	Female	55+ years	Non-melanoma skin cancer (basal-cell	Rate	2021	0.0680198	0.135019007	0.026881581
DALYs (Dis	Bosnia and	Both	55+ years	Non-melanoma skin cancer (basal-cell	Rate	2021	0.0718318	0.141446967	0.029583388
DALYs (Dis	Mozambique	Male	55+ years	Non-melanoma skin cancer (basal-cell	Number	2021	0.0719162	0.141057783	0.029669369
DALYs (Dis	Mozambique	Female	55+ years	Non-melanoma skin cancer (basal-cell	Number	2021	0.0610018	0.121069448	0.025081333
DALYs (Dis	Mozambique	Both	55+ years	Non-melanoma skin cancer (basal-cell	Number	2021	0.132918	0.262727567	0.055379728
DALYs (Dis	Mozambique	Male	55+ years	Non-melanoma skin cancer (basal-cell	Percent	2021	4.16E-08	8.29E-08	1.75E-08
DALYs (Dis	Mozambique	Female	55+ years	Non-melanoma skin cancer (basal-cell	Percent	2021	4.63E-08	8.82E-08	1.92E-08
DALYs (Dis	Mozambique	Both	55+ years	Non-melanoma skin cancer (basal-cell	Percent	2021	4.36E-08	8.40E-08	1.84E-08
DALYs (Dis	Mozambique	Male	55+ years	Non-melanoma skin cancer (basal-cell	Rate	2021	0.0089694	0.017592683	0.003700355
DALYs (Dis	Mozambique	Female	55+ years	Non-melanoma skin cancer (basal-cell	Rate	2021	0.0061784	0.01226223	0.002540303
DALYs (Dis	Mozambique	Both	55+ years	Non-melanoma skin cancer (basal-cell	Rate	2021	0.0074292	0.01468462	0.003095337
DALYs (Dis	Israel	Male	55+ years	Non-melanoma skin cancer (basal-cell	Number	1990	0.4296915	0.90247227	0.161326739
DALYs (Dis	Israel	Female	55+ years	Non-melanoma skin cancer (basal-cell	Number	1990	0.3673079	0.713554642	0.154626081
DALYs (Dis	Israel	Both	55+ years	Non-melanoma skin cancer (basal-cell	Number	1990	0.7969994	1.574444367	0.347295245
DALYs (Dis	Israel	Male	55+ years	Non-melanoma skin cancer (basal-cell	Percent	1990	1.43E-06	2.97E-06	5.50E-07
DALYs (Dis	Israel	Female	55+ years	Non-melanoma skin cancer (basal-cell	Percent	1990	1.22E-06	2.31E-06	5.23E-07
DALYs (Dis	Israel	Both	55+ years	Non-melanoma skin cancer (basal-cell	Percent	1990	1.32E-06	2.58E-06	5.80E-07
DALYs (Dis	Israel	Male	55+ years	Non-melanoma skin cancer (basal-cell	Rate	1990	0.1167938	0.245299594	0.043849972
DALYs (Dis	Israel	Female	55+ years	Non-melanoma skin cancer (basal-cell	Rate	1990	0.0829814	0.161204744	0.034932795
DALYs (Dis	Israel	Both	55+ years	Non-melanoma skin cancer (basal-cell	Rate	1990	0.0983288	0.194245182	0.042847133
DALYs (Dis	Latvia	Male	55+ years	Non-melanoma skin cancer (basal-cell	Number	2021	0.1359252	0.266262885	0.05549578
DALYs (Dis	Latvia	Female	55+ years	Non-melanoma skin cancer (basal-cell	Number	2021	0.2481118	0.483311256	0.107617805
DALYs (Dis	Latvia	Both	55+ years	Non-melanoma skin cancer (basal-cell	Number	2021	0.384037	0.752840124	0.16380011
DALYs (Dis	Latvia	Male	55+ years	Non-melanoma skin cancer (basal-cell	Percent	2021	4.27E-07	8.20E-07	1.77E-07
DALYs (Dis	Latvia	Female	55+ years	Non-melanoma skin cancer (basal-cell	Percent	2021	7.06E-07	1.36E-06	3.18E-07
DALYs (Dis	Latvia	Both	55+ years	Non-melanoma skin cancer (basal-cell	Percent	2021	5.73E-07	1.12E-06	2.54E-07
DALYs (Dis	Latvia	Male	55+ years	Non-melanoma skin cancer (basal-cell	Rate	2021	0.0544813	0.106723016	0.02224372
DALYs (Dis	Latvia	Female	55+ years	Non-melanoma skin cancer (basal-cell	Rate	2021	0.0607329	0.11830501	0.026342704
DALYs (Dis	Latvia	Both	55+ years	Non-melanoma skin cancer (basal-cell	Rate	2021	0.0583626	0.114410004	0.024892897
DALYs (Dis	Lao People	Male	55+ years	Non-melanoma skin cancer (basal-cell	Number	1990	0.0041799	0.008619515	0.001638842
DALYs (Dis	Lao People	Female	55+ years	Non-melanoma skin cancer (basal-cell	Number	1990	0.003496	0.006665181	0.001407184
DALYs (Dis	Lao People	Both	55+ years	Non-melanoma skin cancer (basal-cell	Number	1990	0.0076759	0.015225249	0.003042647
DALYs (Dis	Lao People	Male	55+ years	Non-melanoma skin cancer (basal-cell	Percent	1990	1.56E-08	3.26E-08	6.22E-09
DALYs (Dis	Lao People	Female	55+ years	Non-melanoma skin cancer (basal-cell	Percent	1990	1.49E-08	2.90E-08	6.11E-09
DALYs (Dis	Lao People	Both	55+ years	Non-melanoma skin cancer (basal-cell	Percent	1990	1.53E-08	3.06E-08	6.15E-09
DALYs (Dis	Lao People	Male	55+ years	Non-melanoma skin cancer (basal-cell	Rate	1990	0.0025517	0.005261998	0.001000472
DALYs (Dis	Lao People	Female	55+ years	Non-melanoma skin cancer (basal-cell	Rate	1990	0.0019517	0.003720905	0.000785575
DALYs (Dis	Lao People	Both	55+ years	Non-melanoma skin cancer (basal-cell	Rate	1990	0.0022383	0.004439691	0.000887238
DALYs (Dis	Uzbekistan	Male	55+ years	Non-melanoma skin cancer (basal-cell	Number	2021	1.0191519	1.970268274	0.435443806
DALYs (Dis	Uzbekistan	Female	55+ years	Non-melanoma skin cancer (basal-cell	Number	2021	1.2182105	2.427143537	0.497873135
DALYs (Dis	Uzbekistan	Both	55+ years	Non-melanoma skin cancer (basal-cell	Number	2021	2.2373624	4.319088103	0.940462393
DALYs (Dis	Uzbekistan	Male	55+ years	Non-melanoma skin cancer (basal-cell	Percent	2021	5.28E-07	9.85E-07	2.29E-07
DALYs (Dis	Uzbekistan	Female	55+ years	Non-melanoma skin cancer (basal-cell	Percent	2021	6.56E-07	1.25E-06	2.80E-07
DALYs (Dis	Uzbekistan	Both	55+ years	Non-melanoma skin cancer (basal-cell	Percent	2021	5.90E-07	1.11E-06	2.61E-07
DALYs (Dis	Uzbekistan	Male	55+ years	Non-melanoma skin cancer (basal-cell	Rate	2021	0.0481043	0.092997205	0.020553068
DALYs (Dis	Uzbekistan	Female	55+ years	Non-melanoma skin cancer (basal-cell	Rate	2021	0.0481502	0.095933803	0.019678632
DALYs (Dis	Uzbekistan	Both	55+ years	Non-melanoma skin cancer (basal-cell	Rate	2021	0.0481293	0.092910573	0.020230868
DALYs (Dis	Serbia	Male	55+ years	Non-melanoma skin cancer (basal-cell	Number	2021	0.9213838	1.81770498	0.367938921
DALYs (Dis	Serbia	Female	55+ years	Non-melanoma skin cancer (basal-cell	Number	2021	0.954744	1.980119545	0.383096662
DALYs (Dis	Serbia	Both	55+ years	Non-melanoma skin cancer (basal-cell	Number	2021	1.8761278	3.686243607	0.755607834
DALYs (Dis	Serbia	Male	55+ years	Non-melanoma skin cancer (basal-cell	Percent	2021	5.97E-07	1.16E-06	2.46E-07
DALYs (Dis	Serbia	Female	55+ years	Non-melanoma skin cancer (basal-cell	Percent	2021	6.55E-07	1.28E-06	2.73E-07
DALYs (Dis	Serbia	Both	55+ years	Non-melanoma skin cancer (basal-cell	Percent	2021	6.25E-07	1.21E-06	2.64E-07
DALYs (Dis	Serbia	Male	55+ years	Non-melanoma skin cancer (basal-cell	Rate	2021	0.0724633	0.142955557	0.028936991
DALYs (Dis	Serbia	Female	55+ years	Non-melanoma skin cancer (basal-cell	Rate	2021	0.0622509	0.129107168	0.024978555

DALYs (Dis	Serbia	Both	55+ years	Non-melanoma skin cancer (basal-cell	Rate	2021	0.0668799	0.131406588	0.02693578
DALYs (Dis	Kyrgyzstan	Male	55+ years	Non-melanoma skin cancer (basal-cell	Number	1990	0.1155894	0.231017341	0.050348647
DALYs (Dis	Kyrgyzstan	Female	55+ years	Non-melanoma skin cancer (basal-cell	Number	1990	0.2008427	0.413324231	0.08359676
DALYs (Dis	Kyrgyzstan	Both	55+ years	Non-melanoma skin cancer (basal-cell	Number	1990	0.3164321	0.631466982	0.13984613
DALYs (Dis	Kyrgyzstan	Male	55+ years	Non-melanoma skin cancer (basal-cell	Percent	1990	4.92E-07	9.82E-07	2.12E-07
DALYs (Dis	Kyrgyzstan	Female	55+ years	Non-melanoma skin cancer (basal-cell	Percent	1990	7.65E-07	1.48E-06	3.36E-07
DALYs (Dis	Kyrgyzstan	Both	55+ years	Non-melanoma skin cancer (basal-cell	Percent	1990	6.36E-07	1.23E-06	2.84E-07
DALYs (Dis	Kyrgyzstan	Male	55+ years	Non-melanoma skin cancer (basal-cell	Rate	1990	0.0568127	0.113546119	0.024746599
DALYs (Dis	Kyrgyzstan	Female	55+ years	Non-melanoma skin cancer (basal-cell	Rate	1990	0.0644843	0.132705444	0.026840297
DALYs (Dis	Kyrgyzstan	Both	55+ years	Non-melanoma skin cancer (basal-cell	Rate	1990	0.0614531	0.122634777	0.02715898
DALYs (Dis	Eswatini	Male	55+ years	Non-melanoma skin cancer (basal-cell	Number	2021	0.0120462	0.022891797	0.004888126
DALYs (Dis	Eswatini	Female	55+ years	Non-melanoma skin cancer (basal-cell	Number	2021	0.012319	0.023607729	0.00526135
DALYs (Dis	Eswatini	Both	55+ years	Non-melanoma skin cancer (basal-cell	Number	2021	0.0243652	0.047734026	0.010393443
DALYs (Dis	Eswatini	Male	55+ years	Non-melanoma skin cancer (basal-cell	Percent	2021	1.08E-07	2.09E-07	4.46E-08
DALYs (Dis	Eswatini	Female	55+ years	Non-melanoma skin cancer (basal-cell	Percent	2021	1.19E-07	2.32E-07	5.15E-08
DALYs (Dis	Eswatini	Both	55+ years	Non-melanoma skin cancer (basal-cell	Percent	2021	1.13E-07	2.20E-07	4.87E-08
DALYs (Dis	Eswatini	Male	55+ years	Non-melanoma skin cancer (basal-cell	Rate	2021	0.0341642	0.06492323	0.013863173
DALYs (Dis	Eswatini	Female	55+ years	Non-melanoma skin cancer (basal-cell	Rate	2021	0.0222849	0.042706041	0.009517705
DALYs (Dis	Eswatini	Both	55+ years	Non-melanoma skin cancer (basal-cell	Rate	2021	0.0269112	0.05272183	0.01147947
DALYs (Dis	South Suda	Male	55+ years	Non-melanoma skin cancer (basal-cell	Number	2021	0.0316013	0.062314417	0.013118875
DALYs (Dis	South Suda	Female	55+ years	Non-melanoma skin cancer (basal-cell	Number	2021	0.0176279	0.035317399	0.007293276
DALYs (Dis	South Suda	Both	55+ years	Non-melanoma skin cancer (basal-cell	Number	2021	0.0492291	0.097591627	0.020513105
DALYs (Dis	South Suda	Male	55+ years	Non-melanoma skin cancer (basal-cell	Percent	2021	5.50E-08	1.08E-07	2.38E-08
DALYs (Dis	South Suda	Female	55+ years	Non-melanoma skin cancer (basal-cell	Percent	2021	4.83E-08	9.53E-08	2.14E-08
DALYs (Dis	South Suda	Both	55+ years	Non-melanoma skin cancer (basal-cell	Percent	2021	5.24E-08	1.03E-07	2.32E-08
DALYs (Dis	South Suda	Male	55+ years	Non-melanoma skin cancer (basal-cell	Rate	2021	0.0095167	0.018765887	0.003950728
DALYs (Dis	South Suda	Female	55+ years	Non-melanoma skin cancer (basal-cell	Rate	2021	0.0060648	0.012150748	0.00250921
DALYs (Dis	South Suda	Both	55+ years	Non-melanoma skin cancer (basal-cell	Rate	2021	0.0079055	0.015671769	0.003294101
DALYs (Dis	Morocco	Male	55+ years	Non-melanoma skin cancer (basal-cell	Number	2021	0.2922017	0.578197443	0.120985815
DALYs (Dis	Morocco	Female	55+ years	Non-melanoma skin cancer (basal-cell	Number	2021	0.1864408	0.361750243	0.074906642
DALYs (Dis	Morocco	Both	55+ years	Non-melanoma skin cancer (basal-cell	Number	2021	0.4786425	0.93754499	0.195892457
DALYs (Dis	Morocco	Male	55+ years	Non-melanoma skin cancer (basal-cell	Percent	2021	9.42E-08	1.80E-07	4.10E-08
DALYs (Dis	Morocco	Female	55+ years	Non-melanoma skin cancer (basal-cell	Percent	2021	6.85E-08	1.30E-07	2.95E-08
DALYs (Dis	Morocco	Both	55+ years	Non-melanoma skin cancer (basal-cell	Percent	2021	8.22E-08	1.56E-07	3.58E-08
DALYs (Dis	Morocco	Male	55+ years	Non-melanoma skin cancer (basal-cell	Rate	2021	0.00976	0.019312769	0.00404113
DALYs (Dis	Morocco	Female	55+ years	Non-melanoma skin cancer (basal-cell	Rate	2021	0.0061856	0.012001838	0.002485188
DALYs (Dis	Morocco	Both	55+ years	Non-melanoma skin cancer (basal-cell	Rate	2021	0.0079668	0.015604983	0.003260535
DALYs (Dis	Malawi	Male	55+ years	Non-melanoma skin cancer (basal-cell	Number	2021	0.047018	0.093866531	0.019754398
DALYs (Dis	Malawi	Female	55+ years	Non-melanoma skin cancer (basal-cell	Number	2021	0.0266211	0.053290803	0.010544017
DALYs (Dis	Malawi	Both	55+ years	Non-melanoma skin cancer (basal-cell	Number	2021	0.073639	0.148082371	0.030308565
DALYs (Dis	Malawi	Male	55+ years	Non-melanoma skin cancer (basal-cell	Percent	2021	4.36E-08	8.61E-08	1.83E-08
DALYs (Dis	Malawi	Female	55+ years	Non-melanoma skin cancer (basal-cell	Percent	2021	2.96E-08	5.81E-08	1.24E-08
DALYs (Dis	Malawi	Both	55+ years	Non-melanoma skin cancer (basal-cell	Percent	2021	3.72E-08	7.23E-08	1.55E-08
DALYs (Dis	Malawi	Male	55+ years	Non-melanoma skin cancer (basal-cell	Rate	2021	0.0090694	0.018106051	0.003810454
DALYs (Dis	Malawi	Female	55+ years	Non-melanoma skin cancer (basal-cell	Rate	2021	0.0040911	0.008189745	0.001620407
DALYs (Dis	Malawi	Both	55+ years	Non-melanoma skin cancer (basal-cell	Rate	2021	0.0062986	0.012666054	0.002592408
DALYs (Dis	Niue	Male	55+ years	Non-melanoma skin cancer (basal-cell	Number	1990	1.65E-07	4.27E-07	3.62E-08
DALYs (Dis	Niue	Female	55+ years	Non-melanoma skin cancer (basal-cell	Number	1990	2.43E-07	6.06E-07	5.70E-08
DALYs (Dis	Niue	Both	55+ years	Non-melanoma skin cancer (basal-cell	Number	1990	4.07E-07	1.03E-06	9.76E-08
DALYs (Dis	Niue	Male	55+ years	Non-melanoma skin cancer (basal-cell	Percent	1990	8.16E-10	2.08E-09	1.84E-10
DALYs (Dis	Niue	Female	55+ years	Non-melanoma skin cancer (basal-cell	Percent	1990	1.06E-09	2.67E-09	2.53E-10
DALYs (Dis	Niue	Both	55+ years	Non-melanoma skin cancer (basal-cell	Percent	1990	9.46E-10	2.37E-09	2.38E-10
DALYs (Dis	Niue	Male	55+ years	Non-melanoma skin cancer (basal-cell	Rate	1990	0.0001041	0.000270522	2.29E-05
DALYs (Dis	Niue	Female	55+ years	Non-melanoma skin cancer (basal-cell	Rate	1990	0.0001178	0.000293925	2.76E-05
DALYs (Dis	Niue	Both	55+ years	Non-melanoma skin cancer (basal-cell	Rate	1990	0.0001119	0.000283605	2.68E-05
DALYs (Dis	Spain	Male	55+ years	Non-melanoma skin cancer (basal-cell	Number	2021	8.9995264	17.44842576	3.525407714

DALYs (Dis	Spain	Female	55+ years	Non-melanoma skin cancer (basal-cell	Number	2021	8.780889	18.90640045	3.514624036
DALYs (Dis	Spain	Both	55+ years	Non-melanoma skin cancer (basal-cell	Number	2021	17.780415	35.30897423	7.138217787
DALYs (Dis	Spain	Male	55+ years	Non-melanoma skin cancer (basal-cell	Percent	2021	1.89E-06	3.63E-06	7.63E-07
DALYs (Dis	Spain	Female	55+ years	Non-melanoma skin cancer (basal-cell	Percent	2021	1.90E-06	3.93E-06	8.15E-07
DALYs (Dis	Spain	Both	55+ years	Non-melanoma skin cancer (basal-cell	Percent	2021	1.89E-06	3.80E-06	7.91E-07
DALYs (Dis	Spain	Male	55+ years	Non-melanoma skin cancer (basal-cell	Rate	2021	0.127798	0.247776883	0.050062656
DALYs (Dis	Spain	Female	55+ years	Non-melanoma skin cancer (basal-cell	Rate	2021	0.1041243	0.224193107	0.0416766
DALYs (Dis	Spain	Both	55+ years	Non-melanoma skin cancer (basal-cell	Rate	2021	0.1148971	0.228166692	0.046127184
DALYs (Dis	Lebanon	Male	55+ years	Non-melanoma skin cancer (basal-cell	Number	2021	0.1389277	0.273219544	0.057076486
DALYs (Dis	Lebanon	Female	55+ years	Non-melanoma skin cancer (basal-cell	Number	2021	0.1053948	0.210246881	0.042830247
DALYs (Dis	Lebanon	Both	55+ years	Non-melanoma skin cancer (basal-cell	Number	2021	0.2443226	0.484839146	0.100059825
DALYs (Dis	Lebanon	Male	55+ years	Non-melanoma skin cancer (basal-cell	Percent	2021	2.73E-07	5.18E-07	1.15E-07
DALYs (Dis	Lebanon	Female	55+ years	Non-melanoma skin cancer (basal-cell	Percent	2021	2.52E-07	4.69E-07	1.09E-07
DALYs (Dis	Lebanon	Both	55+ years	Non-melanoma skin cancer (basal-cell	Percent	2021	2.63E-07	4.98E-07	1.12E-07
DALYs (Dis	Lebanon	Male	55+ years	Non-melanoma skin cancer (basal-cell	Rate	2021	0.0312567	0.061470304	0.012841354
DALYs (Dis	Lebanon	Female	55+ years	Non-melanoma skin cancer (basal-cell	Rate	2021	0.0196796	0.039257799	0.007997366
DALYs (Dis	Lebanon	Both	55+ years	Non-melanoma skin cancer (basal-cell	Rate	2021	0.0249302	0.049471945	0.01020989
DALYs (Dis	North Mace	Male	55+ years	Non-melanoma skin cancer (basal-cell	Number	1990	0.1039896	0.200339533	0.043504636
DALYs (Dis	North Mace	Female	55+ years	Non-melanoma skin cancer (basal-cell	Number	1990	0.0972364	0.185611434	0.040722291
DALYs (Dis	North Mace	Both	55+ years	Non-melanoma skin cancer (basal-cell	Number	1990	0.201226	0.384015341	0.085562869
DALYs (Dis	North Mace	Male	55+ years	Non-melanoma skin cancer (basal-cell	Percent	1990	6.07E-07	1.16E-06	2.55E-07
DALYs (Dis	North Mace	Female	55+ years	Non-melanoma skin cancer (basal-cell	Percent	1990	6.57E-07	1.24E-06	2.86E-07
DALYs (Dis	North Mace	Both	55+ years	Non-melanoma skin cancer (basal-cell	Percent	1990	6.30E-07	1.18E-06	2.74E-07
DALYs (Dis	North Mace	Male	55+ years	Non-melanoma skin cancer (basal-cell	Rate	1990	0.0667374	0.128571865	0.027919962
DALYs (Dis	North Mace	Female	55+ years	Non-melanoma skin cancer (basal-cell	Rate	1990	0.0561583	0.107198776	0.023518916
DALYs (Dis	North Mace	Both	55+ years	Non-melanoma skin cancer (basal-cell	Rate	1990	0.0611692	0.116734024	0.026009633
DALYs (Dis	Armenia	Male	55+ years	Non-melanoma skin cancer (basal-cell	Number	2021	0.2062178	0.397766633	0.081717998
DALYs (Dis	Armenia	Female	55+ years	Non-melanoma skin cancer (basal-cell	Number	2021	0.2817551	0.559168942	0.117975162
DALYs (Dis	Armenia	Both	55+ years	Non-melanoma skin cancer (basal-cell	Number	2021	0.4879729	0.932490397	0.198673785
DALYs (Dis	Armenia	Male	55+ years	Non-melanoma skin cancer (basal-cell	Percent	2021	6.09E-07	1.16E-06	2.45E-07
DALYs (Dis	Armenia	Female	55+ years	Non-melanoma skin cancer (basal-cell	Percent	2021	8.70E-07	1.66E-06	3.67E-07
DALYs (Dis	Armenia	Both	55+ years	Non-melanoma skin cancer (basal-cell	Percent	2021	7.36E-07	1.40E-06	3.00E-07
DALYs (Dis	Armenia	Male	55+ years	Non-melanoma skin cancer (basal-cell	Rate	2021	0.0613839	0.118401313	0.02432461
DALYs (Dis	Armenia	Female	55+ years	Non-melanoma skin cancer (basal-cell	Rate	2021	0.062519	0.124074727	0.026177663
DALYs (Dis	Armenia	Both	55+ years	Non-melanoma skin cancer (basal-cell	Rate	2021	0.0620342	0.118544109	0.025256675
DALYs (Dis	Northern M	Male	55+ years	Non-melanoma skin cancer (basal-cell	Number	2021	5.55E-06	1.56E-05	1.10E-06
DALYs (Dis	Northern M	Female	55+ years	Non-melanoma skin cancer (basal-cell	Number	2021	5.85E-06	1.55E-05	1.34E-06
DALYs (Dis	Northern M	Both	55+ years	Non-melanoma skin cancer (basal-cell	Number	2021	1.14E-05	3.02E-05	2.76E-06
DALYs (Dis	Northern M	Male	55+ years	Non-melanoma skin cancer (basal-cell	Percent	2021	1.14E-09	3.06E-09	2.29E-10
DALYs (Dis	Northern M	Female	55+ years	Non-melanoma skin cancer (basal-cell	Percent	2021	1.67E-09	4.37E-09	4.05E-10
DALYs (Dis	Northern M	Both	55+ years	Non-melanoma skin cancer (basal-cell	Percent	2021	1.36E-09	3.58E-09	3.41E-10
DALYs (Dis	Northern M	Male	55+ years	Non-melanoma skin cancer (basal-cell	Rate	2021	0.0001146	0.000321119	2.28E-05
DALYs (Dis	Northern M	Female	55+ years	Non-melanoma skin cancer (basal-cell	Rate	2021	0.00012	0.00031759	2.75E-05
DALYs (Dis	Northern M	Both	55+ years	Non-melanoma skin cancer (basal-cell	Rate	2021	0.0001173	0.000310579	2.84E-05
DALYs (Dis	Timor-Lest	Male	55+ years	Non-melanoma skin cancer (basal-cell	Number	2021	0.0021642	0.004251703	0.00087549
DALYs (Dis	Timor-Lest	Female	55+ years	Non-melanoma skin cancer (basal-cell	Number	2021	0.0016003	0.003085304	0.000617604
DALYs (Dis	Timor-Lest	Both	55+ years	Non-melanoma skin cancer (basal-cell	Number	2021	0.0037644	0.007221944	0.001565268
DALYs (Dis	Timor-Lest	Male	55+ years	Non-melanoma skin cancer (basal-cell	Percent	2021	2.61E-08	5.07E-08	1.09E-08
DALYs (Dis	Timor-Lest	Female	55+ years	Non-melanoma skin cancer (basal-cell	Percent	2021	2.30E-08	4.39E-08	9.09E-09
DALYs (Dis	Timor-Lest	Both	55+ years	Non-melanoma skin cancer (basal-cell	Percent	2021	2.47E-08	4.72E-08	1.02E-08
DALYs (Dis	Timor-Lest	Male	55+ years	Non-melanoma skin cancer (basal-cell	Rate	2021	0.0030653	0.006022017	0.001240025
DALYs (Dis	Timor-Lest	Female	55+ years	Non-melanoma skin cancer (basal-cell	Rate	2021	0.0022404	0.004319433	0.000864647
DALYs (Dis	Timor-Lest	Both	55+ years	Non-melanoma skin cancer (basal-cell	Rate	2021	0.0026504	0.005084762	0.00110206
DALYs (Dis	Congo	Male	55+ years	Non-melanoma skin cancer (basal-cell	Number	2021	0.0241448	0.047383399	0.009922177
DALYs (Dis	Congo	Female	55+ years	Non-melanoma skin cancer (basal-cell	Number	2021	0.0148687	0.029853462	0.006094319
DALYs (Dis	Congo	Both	55+ years	Non-melanoma skin cancer (basal-cell	Number	2021	0.0390135	0.076362534	0.01603926

DALYs (Dis	Congo	Male	55+ years	Non-melanoma skin cancer (basal-cell	Percent	2021	7.55E-08	1.45E-07	3.21E-08
DALYs (Dis	Congo	Female	55+ years	Non-melanoma skin cancer (basal-cell	Percent	2021	5.11E-08	1.01E-07	2.19E-08
DALYs (Dis	Congo	Both	55+ years	Non-melanoma skin cancer (basal-cell	Percent	2021	6.39E-08	1.24E-07	2.82E-08
DALYs (Dis	Congo	Male	55+ years	Non-melanoma skin cancer (basal-cell	Rate	2021	0.0113635	0.022300516	0.004669772
DALYs (Dis	Congo	Female	55+ years	Non-melanoma skin cancer (basal-cell	Rate	2021	0.006696	0.013444371	0.002744549
DALYs (Dis	Congo	Both	55+ years	Non-melanoma skin cancer (basal-cell	Rate	2021	0.0089783	0.017573656	0.003691188
DALYs (Dis	Tonga	Male	55+ years	Non-melanoma skin cancer (basal-cell	Number	2021	6.98E-06	1.81E-05	1.46E-06
DALYs (Dis	Tonga	Female	55+ years	Non-melanoma skin cancer (basal-cell	Number	2021	8.89E-06	2.30E-05	2.29E-06
DALYs (Dis	Tonga	Both	55+ years	Non-melanoma skin cancer (basal-cell	Number	2021	1.59E-05	4.06E-05	4.01E-06
DALYs (Dis	Tonga	Male	55+ years	Non-melanoma skin cancer (basal-cell	Percent	2021	1.11E-09	2.87E-09	2.39E-10
DALYs (Dis	Tonga	Female	55+ years	Non-melanoma skin cancer (basal-cell	Percent	2021	1.55E-09	3.79E-09	3.88E-10
DALYs (Dis	Tonga	Both	55+ years	Non-melanoma skin cancer (basal-cell	Percent	2021	1.32E-09	3.19E-09	3.36E-10
DALYs (Dis	Tonga	Male	55+ years	Non-melanoma skin cancer (basal-cell	Rate	2021	0.0001098	0.000285156	2.30E-05
DALYs (Dis	Tonga	Female	55+ years	Non-melanoma skin cancer (basal-cell	Rate	2021	0.0001258	0.000324665	3.24E-05
DALYs (Dis	Tonga	Both	55+ years	Non-melanoma skin cancer (basal-cell	Rate	2021	0.0001182	0.00030254	2.99E-05
DALYs (Dis	Namibia	Male	55+ years	Non-melanoma skin cancer (basal-cell	Number	2021	0.0367986	0.070835942	0.01611918
DALYs (Dis	Namibia	Female	55+ years	Non-melanoma skin cancer (basal-cell	Number	2021	0.0322653	0.061377229	0.014160817
DALYs (Dis	Namibia	Both	55+ years	Non-melanoma skin cancer (basal-cell	Number	2021	0.0690639	0.131179662	0.029629673
DALYs (Dis	Namibia	Male	55+ years	Non-melanoma skin cancer (basal-cell	Percent	2021	1.83E-07	3.50E-07	8.12E-08
DALYs (Dis	Namibia	Female	55+ years	Non-melanoma skin cancer (basal-cell	Percent	2021	1.83E-07	3.52E-07	8.28E-08
DALYs (Dis	Namibia	Both	55+ years	Non-melanoma skin cancer (basal-cell	Percent	2021	1.83E-07	3.52E-07	8.20E-08
DALYs (Dis	Namibia	Male	55+ years	Non-melanoma skin cancer (basal-cell	Rate	2021	0.0393169	0.075683546	0.017222284
DALYs (Dis	Namibia	Female	55+ years	Non-melanoma skin cancer (basal-cell	Rate	2021	0.0243784	0.046374235	0.01069936
DALYs (Dis	Namibia	Both	55+ years	Non-melanoma skin cancer (basal-cell	Rate	2021	0.0305664	0.058057738	0.013113555
DALYs (Dis	Sri Lanka	Male	55+ years	Non-melanoma skin cancer (basal-cell	Number	2021	0.0674105	0.133303805	0.027186885
DALYs (Dis	Sri Lanka	Female	55+ years	Non-melanoma skin cancer (basal-cell	Number	2021	0.0484237	0.095122752	0.01815871
DALYs (Dis	Sri Lanka	Both	55+ years	Non-melanoma skin cancer (basal-cell	Number	2021	0.1158342	0.231478144	0.045122084
DALYs (Dis	Sri Lanka	Male	55+ years	Non-melanoma skin cancer (basal-cell	Percent	2021	3.77E-08	7.55E-08	1.47E-08
DALYs (Dis	Sri Lanka	Female	55+ years	Non-melanoma skin cancer (basal-cell	Percent	2021	2.85E-08	5.54E-08	1.08E-08
DALYs (Dis	Sri Lanka	Both	55+ years	Non-melanoma skin cancer (basal-cell	Percent	2021	3.32E-08	6.55E-08	1.30E-08
DALYs (Dis	Sri Lanka	Male	55+ years	Non-melanoma skin cancer (basal-cell	Rate	2021	0.003138	0.006205376	0.001265567
DALYs (Dis	Sri Lanka	Female	55+ years	Non-melanoma skin cancer (basal-cell	Rate	2021	0.0018149	0.003565179	0.000680584
DALYs (Dis	Sri Lanka	Both	55+ years	Non-melanoma skin cancer (basal-cell	Rate	2021	0.002405	0.004806136	0.000936861
DALYs (Dis	Liberia	Male	55+ years	Non-melanoma skin cancer (basal-cell	Number	2021	0.0137783	0.027588913	0.005503874
DALYs (Dis	Liberia	Female	55+ years	Non-melanoma skin cancer (basal-cell	Number	2021	0.0073625	0.014554702	0.003021679
DALYs (Dis	Liberia	Both	55+ years	Non-melanoma skin cancer (basal-cell	Number	2021	0.0211408	0.042664939	0.008526119
DALYs (Dis	Liberia	Male	55+ years	Non-melanoma skin cancer (basal-cell	Percent	2021	6.31E-08	1.21E-07	2.84E-08
DALYs (Dis	Liberia	Female	55+ years	Non-melanoma skin cancer (basal-cell	Percent	2021	3.97E-08	7.76E-08	1.76E-08
DALYs (Dis	Liberia	Both	55+ years	Non-melanoma skin cancer (basal-cell	Percent	2021	5.23E-08	1.01E-07	2.35E-08
DALYs (Dis	Liberia	Male	55+ years	Non-melanoma skin cancer (basal-cell	Rate	2021	0.0080793	0.016177594	0.003227363
DALYs (Dis	Liberia	Female	55+ years	Non-melanoma skin cancer (basal-cell	Rate	2021	0.0046136	0.009120411	0.001893474
DALYs (Dis	Liberia	Both	55+ years	Non-melanoma skin cancer (basal-cell	Rate	2021	0.0064039	0.012924006	0.00258272
DALYs (Dis	San Marino	Male	55+ years	Non-melanoma skin cancer (basal-cell	Number	2021	0.0074564	0.015127437	0.003091823
DALYs (Dis	San Marino	Female	55+ years	Non-melanoma skin cancer (basal-cell	Number	2021	0.0057304	0.011758791	0.002194109
DALYs (Dis	San Marino	Both	55+ years	Non-melanoma skin cancer (basal-cell	Number	2021	0.0131867	0.025051848	0.005598667
DALYs (Dis	San Marino	Male	55+ years	Non-melanoma skin cancer (basal-cell	Percent	2021	2.44E-06	5.02E-06	1.02E-06
DALYs (Dis	San Marino	Female	55+ years	Non-melanoma skin cancer (basal-cell	Percent	2021	1.98E-06	3.69E-06	8.04E-07
DALYs (Dis	San Marino	Both	55+ years	Non-melanoma skin cancer (basal-cell	Percent	2021	2.22E-06	4.04E-06	9.77E-07
DALYs (Dis	San Marino	Male	55+ years	Non-melanoma skin cancer (basal-cell	Rate	2021	0.1321914	0.268189463	0.054813944
DALYs (Dis	San Marino	Female	55+ years	Non-melanoma skin cancer (basal-cell	Rate	2021	0.092628	0.190074259	0.035466537
DALYs (Dis	San Marino	Both	55+ years	Non-melanoma skin cancer (basal-cell	Rate	2021	0.1114967	0.211819164	0.047338025
DALYs (Dis	United Kin	Male	55+ years	Non-melanoma skin cancer (basal-cell	Number	1990	8.2441071	15.43380498	3.50437171
DALYs (Dis	United Kin	Female	55+ years	Non-melanoma skin cancer (basal-cell	Number	1990	8.5394621	16.57807273	3.507221697
DALYs (Dis	United Kin	Both	55+ years	Non-melanoma skin cancer (basal-cell	Number	1990	16.783569	31.6826929	7.052628124
DALYs (Dis	United Kin	Male	55+ years	Non-melanoma skin cancer (basal-cell	Percent	1990	1.28E-06	2.30E-06	5.60E-07
DALYs (Dis	United Kin	Female	55+ years	Non-melanoma skin cancer (basal-cell	Percent	1990	1.28E-06	2.42E-06	5.59E-07

DALYs (Dis	United Kin	Both	55+ years	Non-melanoma skin cancer (basal-cell	Percent	1990	1.28E-06	2.34E-06	5.64E-07
DALYs (Dis	United Kin	Male	55+ years	Non-melanoma skin cancer (basal-cell	Rate	1990	0.1278031	0.239260378	0.054326027
DALYs (Dis	United Kin	Female	55+ years	Non-melanoma skin cancer (basal-cell	Rate	1990	0.1016311	0.197301379	0.041740659
DALYs (Dis	United Kin	Both	55+ years	Non-melanoma skin cancer (basal-cell	Rate	1990	0.1129975	0.213307767	0.047482717
DALYs (Dis	Seychelles	Male	55+ years	Non-melanoma skin cancer (basal-cell	Number	2021	0.0002799	0.000589002	9.97E-05
DALYs (Dis	Seychelles	Female	55+ years	Non-melanoma skin cancer (basal-cell	Number	2021	0.0002541	0.000528846	7.94E-05
DALYs (Dis	Seychelles	Both	55+ years	Non-melanoma skin cancer (basal-cell	Number	2021	0.000534	0.001109633	0.000178893
DALYs (Dis	Seychelles	Male	55+ years	Non-melanoma skin cancer (basal-cell	Percent	2021	2.93E-08	5.96E-08	1.06E-08
DALYs (Dis	Seychelles	Female	55+ years	Non-melanoma skin cancer (basal-cell	Percent	2021	3.30E-08	6.92E-08	1.08E-08
DALYs (Dis	Seychelles	Both	55+ years	Non-melanoma skin cancer (basal-cell	Percent	2021	3.10E-08	6.33E-08	1.06E-08
DALYs (Dis	Seychelles	Male	55+ years	Non-melanoma skin cancer (basal-cell	Rate	2021	0.0027884	0.005868318	0.000993197
DALYs (Dis	Seychelles	Female	55+ years	Non-melanoma skin cancer (basal-cell	Rate	2021	0.0023737	0.004939728	0.000741627
DALYs (Dis	Seychelles	Both	55+ years	Non-melanoma skin cancer (basal-cell	Rate	2021	0.0025744	0.005349448	0.000862427
DALYs (Dis	Nigeria	Male	55+ years	Non-melanoma skin cancer (basal-cell	Number	1990	0.2794693	0.547267429	0.114490829
DALYs (Dis	Nigeria	Female	55+ years	Non-melanoma skin cancer (basal-cell	Number	1990	0.1540435	0.296658235	0.063571537
DALYs (Dis	Nigeria	Both	55+ years	Non-melanoma skin cancer (basal-cell	Number	1990	0.4335129	0.857102133	0.179715479
DALYs (Dis	Nigeria	Male	55+ years	Non-melanoma skin cancer (basal-cell	Percent	1990	6.39E-08	1.30E-07	2.67E-08
DALYs (Dis	Nigeria	Female	55+ years	Non-melanoma skin cancer (basal-cell	Percent	1990	3.99E-08	7.87E-08	1.66E-08
DALYs (Dis	Nigeria	Both	55+ years	Non-melanoma skin cancer (basal-cell	Percent	1990	5.23E-08	1.01E-07	2.17E-08
DALYs (Dis	Nigeria	Male	55+ years	Non-melanoma skin cancer (basal-cell	Rate	1990	0.0072137	0.014126097	0.002955244
DALYs (Dis	Nigeria	Female	55+ years	Non-melanoma skin cancer (basal-cell	Rate	1990	0.0045163	0.008697437	0.001863793
DALYs (Dis	Nigeria	Both	55+ years	Non-melanoma skin cancer (basal-cell	Rate	1990	0.0059507	0.011765254	0.002466915
DALYs (Dis	Belarus	Male	55+ years	Non-melanoma skin cancer (basal-cell	Number	2021	0.5683744	1.097207916	0.243850094
DALYs (Dis	Belarus	Female	55+ years	Non-melanoma skin cancer (basal-cell	Number	2021	1.0395041	2.041562141	0.424774654
DALYs (Dis	Belarus	Both	55+ years	Non-melanoma skin cancer (basal-cell	Number	2021	1.6078785	3.112586874	0.66227944
DALYs (Dis	Belarus	Male	55+ years	Non-melanoma skin cancer (basal-cell	Percent	2021	3.53E-07	6.76E-07	1.53E-07
DALYs (Dis	Belarus	Female	55+ years	Non-melanoma skin cancer (basal-cell	Percent	2021	6.48E-07	1.22E-06	2.75E-07
DALYs (Dis	Belarus	Both	55+ years	Non-melanoma skin cancer (basal-cell	Percent	2021	5.00E-07	9.44E-07	2.13E-07
DALYs (Dis	Belarus	Male	55+ years	Non-melanoma skin cancer (basal-cell	Rate	2021	0.0512358	0.098907229	0.021981738
DALYs (Dis	Belarus	Female	55+ years	Non-melanoma skin cancer (basal-cell	Rate	2021	0.0588197	0.115520522	0.024035609
DALYs (Dis	Belarus	Both	55+ years	Non-melanoma skin cancer (basal-cell	Rate	2021	0.0558951	0.108203575	0.023022973
DALYs (Dis	Germany	Male	55+ years	Non-melanoma skin cancer (basal-cell	Number	2021	14.063429	28.1459329	5.924462428
DALYs (Dis	Germany	Female	55+ years	Non-melanoma skin cancer (basal-cell	Number	2021	12.693435	25.05547158	4.904495877
DALYs (Dis	Germany	Both	55+ years	Non-melanoma skin cancer (basal-cell	Number	2021	26.756864	51.39391353	10.86864115
DALYs (Dis	Germany	Male	55+ years	Non-melanoma skin cancer (basal-cell	Percent	2021	1.30E-06	2.52E-06	5.52E-07
DALYs (Dis	Germany	Female	55+ years	Non-melanoma skin cancer (basal-cell	Percent	2021	1.19E-06	2.27E-06	4.82E-07
DALYs (Dis	Germany	Both	55+ years	Non-melanoma skin cancer (basal-cell	Percent	2021	1.24E-06	2.35E-06	5.27E-07
DALYs (Dis	Germany	Male	55+ years	Non-melanoma skin cancer (basal-cell	Rate	2021	0.0960617	0.192253771	0.040467667
DALYs (Dis	Germany	Female	55+ years	Non-melanoma skin cancer (basal-cell	Rate	2021	0.0752252	0.148486444	0.029065554
DALYs (Dis	Germany	Both	55+ years	Non-melanoma skin cancer (basal-cell	Rate	2021	0.084905	0.163083308	0.034488402
DALYs (Dis	Iceland	Male	55+ years	Non-melanoma skin cancer (basal-cell	Number	2021	0.0535812	0.106628145	0.021596694
DALYs (Dis	Iceland	Female	55+ years	Non-melanoma skin cancer (basal-cell	Number	2021	0.0445323	0.085213436	0.019870825
DALYs (Dis	Iceland	Both	55+ years	Non-melanoma skin cancer (basal-cell	Number	2021	0.0981135	0.192143202	0.042500277
DALYs (Dis	Iceland	Male	55+ years	Non-melanoma skin cancer (basal-cell	Percent	2021	2.16E-06	4.14E-06	8.95E-07
DALYs (Dis	Iceland	Female	55+ years	Non-melanoma skin cancer (basal-cell	Percent	2021	1.69E-06	3.22E-06	7.48E-07
DALYs (Dis	Iceland	Both	55+ years	Non-melanoma skin cancer (basal-cell	Percent	2021	1.92E-06	3.72E-06	8.25E-07
DALYs (Dis	Iceland	Male	55+ years	Non-melanoma skin cancer (basal-cell	Rate	2021	0.1130117	0.224896437	0.045551008
DALYs (Dis	Iceland	Female	55+ years	Non-melanoma skin cancer (basal-cell	Rate	2021	0.0891108	0.17051529	0.039762269
DALYs (Dis	Iceland	Both	55+ years	Non-melanoma skin cancer (basal-cell	Rate	2021	0.1007469	0.197300271	0.043640972
DALYs (Dis	Guinea-Bis	Male	55+ years	Non-melanoma skin cancer (basal-cell	Number	2021	0.0036749	0.007446778	0.001495655
DALYs (Dis	Guinea-Bis	Female	55+ years	Non-melanoma skin cancer (basal-cell	Number	2021	0.0026487	0.005335252	0.001057591
DALYs (Dis	Guinea-Bis	Both	55+ years	Non-melanoma skin cancer (basal-cell	Number	2021	0.0063236	0.012769143	0.002534641
DALYs (Dis	Guinea-Bis	Male	55+ years	Non-melanoma skin cancer (basal-cell	Percent	2021	3.69E-08	7.38E-08	1.52E-08
DALYs (Dis	Guinea-Bis	Female	55+ years	Non-melanoma skin cancer (basal-cell	Percent	2021	3.11E-08	6.04E-08	1.28E-08
DALYs (Dis	Guinea-Bis	Both	55+ years	Non-melanoma skin cancer (basal-cell	Percent	2021	3.42E-08	6.66E-08	1.43E-08
DALYs (Dis	Guinea-Bis	Male	55+ years	Non-melanoma skin cancer (basal-cell	Rate	2021	0.00692	0.014022577	0.002816378

DALYs (Dis	Guinea-Bis	Female	55+ years	Non-melanoma skin cancer (basal-cell	Rate	2021	0.0042884	0.008638027	0.001712291
DALYs (Dis	Guinea-Bis	Both	55+ years	Non-melanoma skin cancer (basal-cell	Rate	2021	0.005505	0.011116137	0.002206524
DALYs (Dis	Kyrgyzstan	Male	55+ years	Non-melanoma skin cancer (basal-cell	Number	2021	0.1875533	0.366284563	0.07936877
DALYs (Dis	Kyrgyzstan	Female	55+ years	Non-melanoma skin cancer (basal-cell	Number	2021	0.2617633	0.498974913	0.110843723
DALYs (Dis	Kyrgyzstan	Both	55+ years	Non-melanoma skin cancer (basal-cell	Number	2021	0.4493166	0.86480782	0.189041843
DALYs (Dis	Kyrgyzstan	Male	55+ years	Non-melanoma skin cancer (basal-cell	Percent	2021	5.15E-07	9.99E-07	2.16E-07
DALYs (Dis	Kyrgyzstan	Female	55+ years	Non-melanoma skin cancer (basal-cell	Percent	2021	7.70E-07	1.44E-06	3.31E-07
DALYs (Dis	Kyrgyzstan	Both	55+ years	Non-melanoma skin cancer (basal-cell	Percent	2021	6.38E-07	1.22E-06	2.83E-07
DALYs (Dis	Kyrgyzstan	Male	55+ years	Non-melanoma skin cancer (basal-cell	Rate	2021	0.0513487	0.100282091	0.021729734
DALYs (Dis	Kyrgyzstan	Female	55+ years	Non-melanoma skin cancer (basal-cell	Rate	2021	0.0544777	0.103845763	0.023068597
DALYs (Dis	Kyrgyzstan	Both	55+ years	Non-melanoma skin cancer (basal-cell	Rate	2021	0.0531264	0.102253317	0.022351967
DALYs (Dis	Belize	Male	55+ years	Non-melanoma skin cancer (basal-cell	Number	1990	0.0012443	0.002407167	0.000520756
DALYs (Dis	Belize	Female	55+ years	Non-melanoma skin cancer (basal-cell	Number	1990	0.000483	0.000935442	0.000195928
DALYs (Dis	Belize	Both	55+ years	Non-melanoma skin cancer (basal-cell	Number	1990	0.0017273	0.003315757	0.000722728
DALYs (Dis	Belize	Male	55+ years	Non-melanoma skin cancer (basal-cell	Percent	1990	2.17E-07	4.11E-07	9.28E-08
DALYs (Dis	Belize	Female	55+ years	Non-melanoma skin cancer (basal-cell	Percent	1990	9.38E-08	1.76E-07	4.06E-08
DALYs (Dis	Belize	Both	55+ years	Non-melanoma skin cancer (basal-cell	Percent	1990	1.59E-07	3.01E-07	6.86E-08
DALYs (Dis	Belize	Male	55+ years	Non-melanoma skin cancer (basal-cell	Rate	1990	0.0160685	0.031085665	0.006724942
DALYs (Dis	Belize	Female	55+ years	Non-melanoma skin cancer (basal-cell	Rate	1990	0.0063143	0.012229711	0.002561512
DALYs (Dis	Belize	Both	55+ years	Non-melanoma skin cancer (basal-cell	Rate	1990	0.0112215	0.021541258	0.004695298
DALYs (Dis	Monaco	Male	55+ years	Non-melanoma skin cancer (basal-cell	Number	2021	0.0086052	0.016592013	0.003423305
DALYs (Dis	Monaco	Female	55+ years	Non-melanoma skin cancer (basal-cell	Number	2021	0.0072794	0.015288501	0.002900131
DALYs (Dis	Monaco	Both	55+ years	Non-melanoma skin cancer (basal-cell	Number	2021	0.0158846	0.029624868	0.006772452
DALYs (Dis	Monaco	Male	55+ years	Non-melanoma skin cancer (basal-cell	Percent	2021	1.43E-06	2.74E-06	5.76E-07
DALYs (Dis	Monaco	Female	55+ years	Non-melanoma skin cancer (basal-cell	Percent	2021	1.30E-06	2.60E-06	5.57E-07
DALYs (Dis	Monaco	Both	55+ years	Non-melanoma skin cancer (basal-cell	Percent	2021	1.36E-06	2.60E-06	5.82E-07
DALYs (Dis	Monaco	Male	55+ years	Non-melanoma skin cancer (basal-cell	Rate	2021	0.1163861	0.224408326	0.046300477
DALYs (Dis	Monaco	Female	55+ years	Non-melanoma skin cancer (basal-cell	Rate	2021	0.0882166	0.185277348	0.035145935
DALYs (Dis	Monaco	Both	55+ years	Non-melanoma skin cancer (basal-cell	Rate	2021	0.1015289	0.189352485	0.043287301
DALYs (Dis	Azerbaijan	Male	55+ years	Non-melanoma skin cancer (basal-cell	Number	2021	0.4234063	0.833875385	0.177296777
DALYs (Dis	Azerbaijan	Female	55+ years	Non-melanoma skin cancer (basal-cell	Number	2021	0.5283189	1.030962311	0.222828461
DALYs (Dis	Azerbaijan	Both	55+ years	Non-melanoma skin cancer (basal-cell	Number	2021	0.9517252	1.83623303	0.401790029
DALYs (Dis	Azerbaijan	Male	55+ years	Non-melanoma skin cancer (basal-cell	Percent	2021	4.44E-07	8.22E-07	1.96E-07
DALYs (Dis	Azerbaijan	Female	55+ years	Non-melanoma skin cancer (basal-cell	Percent	2021	6.18E-07	1.17E-06	2.63E-07
DALYs (Dis	Azerbaijan	Both	55+ years	Non-melanoma skin cancer (basal-cell	Percent	2021	5.26E-07	9.75E-07	2.27E-07
DALYs (Dis	Azerbaijan	Male	55+ years	Non-melanoma skin cancer (basal-cell	Rate	2021	0.0492635	0.097021842	0.020628574
DALYs (Dis	Azerbaijan	Female	55+ years	Non-melanoma skin cancer (basal-cell	Rate	2021	0.0500147	0.097598754	0.021094641
DALYs (Dis	Azerbaijan	Both	55+ years	Non-melanoma skin cancer (basal-cell	Rate	2021	0.0496777	0.095846845	0.020972451
DALYs (Dis	Djibouti	Male	55+ years	Non-melanoma skin cancer (basal-cell	Number	1990	0.0010597	0.002189898	0.000429026
DALYs (Dis	Djibouti	Female	55+ years	Non-melanoma skin cancer (basal-cell	Number	1990	0.0006643	0.001326244	0.000263994
DALYs (Dis	Djibouti	Both	55+ years	Non-melanoma skin cancer (basal-cell	Number	1990	0.001724	0.003480326	0.000710179
DALYs (Dis	Djibouti	Male	55+ years	Non-melanoma skin cancer (basal-cell	Percent	1990	8.41E-08	1.67E-07	3.47E-08
DALYs (Dis	Djibouti	Female	55+ years	Non-melanoma skin cancer (basal-cell	Percent	1990	6.79E-08	1.28E-07	2.74E-08
DALYs (Dis	Djibouti	Both	55+ years	Non-melanoma skin cancer (basal-cell	Percent	1990	7.70E-08	1.49E-07	3.16E-08
DALYs (Dis	Djibouti	Male	55+ years	Non-melanoma skin cancer (basal-cell	Rate	1990	0.0095475	0.019730559	0.003865441
DALYs (Dis	Djibouti	Female	55+ years	Non-melanoma skin cancer (basal-cell	Rate	1990	0.006415	0.012807674	0.002549421
DALYs (Dis	Djibouti	Both	55+ years	Non-melanoma skin cancer (basal-cell	Rate	1990	0.0080355	0.016222203	0.003310225
DALYs (Dis	Panama	Male	55+ years	Non-melanoma skin cancer (basal-cell	Number	1990	0.0672765	0.132451236	0.029994894
DALYs (Dis	Panama	Female	55+ years	Non-melanoma skin cancer (basal-cell	Number	1990	0.0581251	0.116253709	0.026765463
DALYs (Dis	Panama	Both	55+ years	Non-melanoma skin cancer (basal-cell	Number	1990	0.1254016	0.248214845	0.056836486
DALYs (Dis	Panama	Male	55+ years	Non-melanoma skin cancer (basal-cell	Percent	1990	7.84E-07	1.46E-06	3.66E-07
DALYs (Dis	Panama	Female	55+ years	Non-melanoma skin cancer (basal-cell	Percent	1990	8.30E-07	1.58E-06	3.92E-07
DALYs (Dis	Panama	Both	55+ years	Non-melanoma skin cancer (basal-cell	Percent	1990	8.04E-07	1.52E-06	3.80E-07
DALYs (Dis	Panama	Male	55+ years	Non-melanoma skin cancer (basal-cell	Rate	1990	0.0548648	0.108015497	0.024461179
DALYs (Dis	Panama	Female	55+ years	Non-melanoma skin cancer (basal-cell	Rate	1990	0.048572	0.097146981	0.02236646
DALYs (Dis	Panama	Both	55+ years	Non-melanoma skin cancer (basal-cell	Rate	1990	0.0517568	0.102445229	0.023458012

DALYs (Dis	Australia	Male	55+ years	Non-melanoma skin cancer (basal-cell	Number	2021	3.2213154	6.360053977	1.325976608
DALYs (Dis	Australia	Female	55+ years	Non-melanoma skin cancer (basal-cell	Number	2021	2.7523903	5.202275889	1.163393137
DALYs (Dis	Australia	Both	55+ years	Non-melanoma skin cancer (basal-cell	Number	2021	5.9737057	11.57858745	2.545346021
DALYs (Dis	Australia	Male	55+ years	Non-melanoma skin cancer (basal-cell	Percent	2021	1.55E-06	2.96E-06	6.69E-07
DALYs (Dis	Australia	Female	55+ years	Non-melanoma skin cancer (basal-cell	Percent	2021	1.40E-06	2.63E-06	6.18E-07
DALYs (Dis	Australia	Both	55+ years	Non-melanoma skin cancer (basal-cell	Percent	2021	1.47E-06	2.83E-06	6.58E-07
DALYs (Dis	Australia	Male	55+ years	Non-melanoma skin cancer (basal-cell	Rate	2021	0.0914423	0.180540539	0.037640016
DALYs (Dis	Australia	Female	55+ years	Non-melanoma skin cancer (basal-cell	Rate	2021	0.0708458	0.133905261	0.029945444
DALYs (Dis	Australia	Both	55+ years	Non-melanoma skin cancer (basal-cell	Rate	2021	0.0806405	0.156302085	0.034360227
DALYs (Dis	Republic o	Male	55+ years	Non-melanoma skin cancer (basal-cell	Number	2021	0.2225603	0.437677078	0.094248293
DALYs (Dis	Republic o	Female	55+ years	Non-melanoma skin cancer (basal-cell	Number	2021	0.3318372	0.630840688	0.142243096
DALYs (Dis	Republic o	Both	55+ years	Non-melanoma skin cancer (basal-cell	Number	2021	0.5543975	1.06853822	0.23792199
DALYs (Dis	Republic o	Male	55+ years	Non-melanoma skin cancer (basal-cell	Percent	2021	4.14E-07	8.09E-07	1.88E-07
DALYs (Dis	Republic o	Female	55+ years	Non-melanoma skin cancer (basal-cell	Percent	2021	6.34E-07	1.20E-06	2.89E-07
DALYs (Dis	Republic o	Both	55+ years	Non-melanoma skin cancer (basal-cell	Percent	2021	5.22E-07	1.00E-06	2.38E-07
DALYs (Dis	Republic o	Male	55+ years	Non-melanoma skin cancer (basal-cell	Rate	2021	0.0520546	0.102368203	0.022043714
DALYs (Dis	Republic o	Female	55+ years	Non-melanoma skin cancer (basal-cell	Rate	2021	0.0528167	0.100407368	0.022640034
DALYs (Dis	Republic o	Both	55+ years	Non-melanoma skin cancer (basal-cell	Rate	2021	0.0525081	0.101203331	0.022534054
DALYs (Dis	Ghana	Male	55+ years	Non-melanoma skin cancer (basal-cell	Number	2021	0.093279	0.182991166	0.037076585
DALYs (Dis	Ghana	Female	55+ years	Non-melanoma skin cancer (basal-cell	Number	2021	0.0743495	0.146122083	0.030715838
DALYs (Dis	Ghana	Both	55+ years	Non-melanoma skin cancer (basal-cell	Number	2021	0.1676285	0.330320435	0.067603194
DALYs (Dis	Ghana	Male	55+ years	Non-melanoma skin cancer (basal-cell	Percent	2021	5.46E-08	1.05E-07	2.24E-08
DALYs (Dis	Ghana	Female	55+ years	Non-melanoma skin cancer (basal-cell	Percent	2021	4.77E-08	9.13E-08	1.93E-08
DALYs (Dis	Ghana	Both	55+ years	Non-melanoma skin cancer (basal-cell	Percent	2021	5.13E-08	9.73E-08	2.11E-08
DALYs (Dis	Ghana	Male	55+ years	Non-melanoma skin cancer (basal-cell	Rate	2021	0.007848	0.015395827	0.003119411
DALYs (Dis	Ghana	Female	55+ years	Non-melanoma skin cancer (basal-cell	Rate	2021	0.0048323	0.009497113	0.001996357
DALYs (Dis	Ghana	Both	55+ years	Non-melanoma skin cancer (basal-cell	Rate	2021	0.0061466	0.012112201	0.002478876
DALYs (Dis	North Mace	Male	55+ years	Non-melanoma skin cancer (basal-cell	Number	2021	0.1970752	0.397047798	0.080456489
DALYs (Dis	North Mace	Female	55+ years	Non-melanoma skin cancer (basal-cell	Number	2021	0.1848507	0.358857478	0.077239406
DALYs (Dis	North Mace	Both	55+ years	Non-melanoma skin cancer (basal-cell	Number	2021	0.3819259	0.745872168	0.154945952
DALYs (Dis	North Mace	Male	55+ years	Non-melanoma skin cancer (basal-cell	Percent	2021	5.26E-07	1.06E-06	2.14E-07
DALYs (Dis	North Mace	Female	55+ years	Non-melanoma skin cancer (basal-cell	Percent	2021	5.94E-07	1.17E-06	2.53E-07
DALYs (Dis	North Mace	Both	55+ years	Non-melanoma skin cancer (basal-cell	Percent	2021	5.57E-07	1.07E-06	2.35E-07
DALYs (Dis	North Mace	Male	55+ years	Non-melanoma skin cancer (basal-cell	Rate	2021	0.068978	0.138970112	0.028160457
DALYs (Dis	North Mace	Female	55+ years	Non-melanoma skin cancer (basal-cell	Rate	2021	0.0601731	0.116816175	0.025143163
DALYs (Dis	North Mace	Both	55+ years	Non-melanoma skin cancer (basal-cell	Rate	2021	0.064416	0.125799443	0.026133318
DALYs (Dis	Singapore	Male	55+ years	Non-melanoma skin cancer (basal-cell	Number	2021	0.1528506	0.300880651	0.064021367
DALYs (Dis	Singapore	Female	55+ years	Non-melanoma skin cancer (basal-cell	Number	2021	0.1530598	0.302172821	0.061586741
DALYs (Dis	Singapore	Both	55+ years	Non-melanoma skin cancer (basal-cell	Number	2021	0.3059104	0.60298743	0.12738189
DALYs (Dis	Singapore	Male	55+ years	Non-melanoma skin cancer (basal-cell	Percent	2021	4.52E-07	8.50E-07	2.03E-07
DALYs (Dis	Singapore	Female	55+ years	Non-melanoma skin cancer (basal-cell	Percent	2021	5.19E-07	9.79E-07	2.34E-07
DALYs (Dis	Singapore	Both	55+ years	Non-melanoma skin cancer (basal-cell	Percent	2021	4.83E-07	9.21E-07	2.13E-07
DALYs (Dis	Singapore	Male	55+ years	Non-melanoma skin cancer (basal-cell	Rate	2021	0.0202437	0.039848882	0.008479043
DALYs (Dis	Singapore	Female	55+ years	Non-melanoma skin cancer (basal-cell	Rate	2021	0.02004	0.039563164	0.008063486
DALYs (Dis	Singapore	Both	55+ years	Non-melanoma skin cancer (basal-cell	Rate	2021	0.0201412	0.039700855	0.008386858
DALYs (Dis	Bahamas	Male	55+ years	Non-melanoma skin cancer (basal-cell	Number	2021	0.0044767	0.008665903	0.001900904
DALYs (Dis	Bahamas	Female	55+ years	Non-melanoma skin cancer (basal-cell	Number	2021	0.0022637	0.00440827	0.000921314
DALYs (Dis	Bahamas	Both	55+ years	Non-melanoma skin cancer (basal-cell	Number	2021	0.0067404	0.012981203	0.002876838
DALYs (Dis	Bahamas	Male	55+ years	Non-melanoma skin cancer (basal-cell	Percent	2021	1.18E-07	2.21E-07	5.24E-08
DALYs (Dis	Bahamas	Female	55+ years	Non-melanoma skin cancer (basal-cell	Percent	2021	7.10E-08	1.33E-07	3.06E-08
DALYs (Dis	Bahamas	Both	55+ years	Non-melanoma skin cancer (basal-cell	Percent	2021	9.65E-08	1.80E-07	4.25E-08
DALYs (Dis	Bahamas	Male	55+ years	Non-melanoma skin cancer (basal-cell	Rate	2021	0.01374	0.026597546	0.005834287
DALYs (Dis	Bahamas	Female	55+ years	Non-melanoma skin cancer (basal-cell	Rate	2021	0.0057487	0.011195036	0.002339726
DALYs (Dis	Bahamas	Both	55+ years	Non-melanoma skin cancer (basal-cell	Rate	2021	0.009367	0.018039823	0.003997907
DALYs (Dis	Bulgaria	Male	55+ years	Non-melanoma skin cancer (basal-cell	Number	2021	1.2089982	2.549369952	0.473505365
DALYs (Dis	Bulgaria	Female	55+ years	Non-melanoma skin cancer (basal-cell	Number	2021	1.5590898	3.130306381	0.679203487

DALYs (Dis	Bulgaria	Both	55+ years	Non-melanoma skin cancer (basal-cell	Number	2021	2.768088	5.559578676	1.243229638
DALYs (Dis	Bulgaria	Male	55+ years	Non-melanoma skin cancer (basal-cell	Percent	2021	7.14E-07	1.52E-06	2.85E-07
DALYs (Dis	Bulgaria	Female	55+ years	Non-melanoma skin cancer (basal-cell	Percent	2021	1.01E-06	2.02E-06	4.48E-07
DALYs (Dis	Bulgaria	Both	55+ years	Non-melanoma skin cancer (basal-cell	Percent	2021	8.56E-07	1.69E-06	3.89E-07
DALYs (Dis	Bulgaria	Male	55+ years	Non-melanoma skin cancer (basal-cell	Rate	2021	0.1183465	0.24955295	0.046350535
DALYs (Dis	Bulgaria	Female	55+ years	Non-melanoma skin cancer (basal-cell	Rate	2021	0.1150337	0.230962164	0.050113404
DALYs (Dis	Bulgaria	Both	55+ years	Non-melanoma skin cancer (basal-cell	Rate	2021	0.1164575	0.233899641	0.052304497
DALYs (Dis	Lithuania	Male	55+ years	Non-melanoma skin cancer (basal-cell	Number	1990	0.1845727	0.36034239	0.075946377
DALYs (Dis	Lithuania	Female	55+ years	Non-melanoma skin cancer (basal-cell	Number	1990	0.3443363	0.697823433	0.146494271
DALYs (Dis	Lithuania	Both	55+ years	Non-melanoma skin cancer (basal-cell	Number	1990	0.528909	1.039540325	0.228042832
DALYs (Dis	Lithuania	Male	55+ years	Non-melanoma skin cancer (basal-cell	Percent	1990	5.13E-07	9.96E-07	2.16E-07
DALYs (Dis	Lithuania	Female	55+ years	Non-melanoma skin cancer (basal-cell	Percent	1990	8.97E-07	1.78E-06	4.02E-07
DALYs (Dis	Lithuania	Both	55+ years	Non-melanoma skin cancer (basal-cell	Percent	1990	7.11E-07	1.37E-06	3.15E-07
DALYs (Dis	Lithuania	Male	55+ years	Non-melanoma skin cancer (basal-cell	Rate	1990	0.060566	0.118243323	0.024921164
DALYs (Dis	Lithuania	Female	55+ years	Non-melanoma skin cancer (basal-cell	Rate	1990	0.0706037	0.143083707	0.030037603
DALYs (Dis	Lithuania	Both	55+ years	Non-melanoma skin cancer (basal-cell	Rate	1990	0.0667436	0.131180651	0.028776957
DALYs (Dis	Estonia	Male	55+ years	Non-melanoma skin cancer (basal-cell	Number	2021	0.1028128	0.208393131	0.043589937
DALYs (Dis	Estonia	Female	55+ years	Non-melanoma skin cancer (basal-cell	Number	2021	0.1812694	0.346952858	0.072914407
DALYs (Dis	Estonia	Both	55+ years	Non-melanoma skin cancer (basal-cell	Number	2021	0.2840822	0.543855724	0.115607293
DALYs (Dis	Estonia	Male	55+ years	Non-melanoma skin cancer (basal-cell	Percent	2021	5.70E-07	1.10E-06	2.51E-07
DALYs (Dis	Estonia	Female	55+ years	Non-melanoma skin cancer (basal-cell	Percent	2021	9.39E-07	1.74E-06	3.91E-07
DALYs (Dis	Estonia	Both	55+ years	Non-melanoma skin cancer (basal-cell	Percent	2021	7.61E-07	1.41E-06	3.17E-07
DALYs (Dis	Estonia	Male	55+ years	Non-melanoma skin cancer (basal-cell	Rate	2021	0.0599977	0.121610403	0.02543745
DALYs (Dis	Estonia	Female	55+ years	Non-melanoma skin cancer (basal-cell	Rate	2021	0.0681172	0.130377497	0.027399682
DALYs (Dis	Estonia	Both	55+ years	Non-melanoma skin cancer (basal-cell	Rate	2021	0.0649367	0.124316888	0.026426014
DALYs (Dis	Hungary	Male	55+ years	Non-melanoma skin cancer (basal-cell	Number	1990	0.7909398	1.588984201	0.350028527
DALYs (Dis	Hungary	Female	55+ years	Non-melanoma skin cancer (basal-cell	Number	1990	1.0624318	2.133041408	0.465798881
DALYs (Dis	Hungary	Both	55+ years	Non-melanoma skin cancer (basal-cell	Number	1990	1.8533716	3.532019241	0.814493219
DALYs (Dis	Hungary	Male	55+ years	Non-melanoma skin cancer (basal-cell	Percent	1990	5.35E-07	1.05E-06	2.42E-07
DALYs (Dis	Hungary	Female	55+ years	Non-melanoma skin cancer (basal-cell	Percent	1990	7.53E-07	1.46E-06	3.36E-07
DALYs (Dis	Hungary	Both	55+ years	Non-melanoma skin cancer (basal-cell	Percent	1990	6.41E-07	1.21E-06	2.87E-07
DALYs (Dis	Hungary	Male	55+ years	Non-melanoma skin cancer (basal-cell	Rate	1990	0.0738255	0.148314162	0.032671305
DALYs (Dis	Hungary	Female	55+ years	Non-melanoma skin cancer (basal-cell	Rate	1990	0.070624	0.141791562	0.030963464
DALYs (Dis	Hungary	Both	55+ years	Non-melanoma skin cancer (basal-cell	Rate	1990	0.0719556	0.137127783	0.031622039
DALYs (Dis	Ireland	Male	55+ years	Non-melanoma skin cancer (basal-cell	Number	1990	0.6552121	1.294245082	0.265732742
DALYs (Dis	Ireland	Female	55+ years	Non-melanoma skin cancer (basal-cell	Number	1990	0.6546935	1.28153761	0.28946873
DALYs (Dis	Ireland	Both	55+ years	Non-melanoma skin cancer (basal-cell	Number	1990	1.3099055	2.532857309	0.596082961
DALYs (Dis	Ireland	Male	55+ years	Non-melanoma skin cancer (basal-cell	Percent	1990	1.96E-06	3.83E-06	8.04E-07
DALYs (Dis	Ireland	Female	55+ years	Non-melanoma skin cancer (basal-cell	Percent	1990	2.19E-06	4.20E-06	9.89E-07
DALYs (Dis	Ireland	Both	55+ years	Non-melanoma skin cancer (basal-cell	Percent	1990	2.07E-06	3.92E-06	9.61E-07
DALYs (Dis	Ireland	Male	55+ years	Non-melanoma skin cancer (basal-cell	Rate	1990	0.209999	0.414812471	0.085168765
DALYs (Dis	Ireland	Female	55+ years	Non-melanoma skin cancer (basal-cell	Rate	1990	0.17756	0.347567025	0.078507088
DALYs (Dis	Ireland	Both	55+ years	Non-melanoma skin cancer (basal-cell	Rate	1990	0.1924283	0.37208288	0.087566032
DALYs (Dis	Argentina	Male	55+ years	Non-melanoma skin cancer (basal-cell	Number	1990	1.6484271	3.206269484	0.748682045
DALYs (Dis	Argentina	Female	55+ years	Non-melanoma skin cancer (basal-cell	Number	1990	1.7472412	3.561135306	0.739441251
DALYs (Dis	Argentina	Both	55+ years	Non-melanoma skin cancer (basal-cell	Number	1990	3.3956683	6.757142807	1.494578185
DALYs (Dis	Argentina	Male	55+ years	Non-melanoma skin cancer (basal-cell	Percent	1990	6.41E-07	1.21E-06	2.98E-07
DALYs (Dis	Argentina	Female	55+ years	Non-melanoma skin cancer (basal-cell	Percent	1990	7.94E-07	1.52E-06	3.53E-07
DALYs (Dis	Argentina	Both	55+ years	Non-melanoma skin cancer (basal-cell	Percent	1990	7.12E-07	1.38E-06	3.31E-07
DALYs (Dis	Argentina	Male	55+ years	Non-melanoma skin cancer (basal-cell	Rate	1990	0.0664807	0.129308149	0.030194184
DALYs (Dis	Argentina	Female	55+ years	Non-melanoma skin cancer (basal-cell	Rate	1990	0.0566357	0.115431992	0.023968529
DALYs (Dis	Argentina	Both	55+ years	Non-melanoma skin cancer (basal-cell	Rate	1990	0.0610226	0.121430712	0.026858644
DALYs (Dis	Pakistan	Male	55+ years	Non-melanoma skin cancer (basal-cell	Number	2021	0.0503755	0.107318494	0.020034957
DALYs (Dis	Pakistan	Female	55+ years	Non-melanoma skin cancer (basal-cell	Number	2021	0.0387736	0.080018372	0.015675009
DALYs (Dis	Pakistan	Both	55+ years	Non-melanoma skin cancer (basal-cell	Number	2021	0.0891491	0.188132014	0.0352939
DALYs (Dis	Pakistan	Male	55+ years	Non-melanoma skin cancer (basal-cell	Percent	2021	3.90E-09	8.10E-09	1.64E-09

DALYs (Dis	Pakistan	Female	55+ years	Non-melanoma skin cancer (basal-cell	Percent	2021	3.87E-09	7.60E-09	1.55E-09
DALYs (Dis	Pakistan	Both	55+ years	Non-melanoma skin cancer (basal-cell	Percent	2021	3.88E-09	7.61E-09	1.60E-09
DALYs (Dis	Pakistan	Male	55+ years	Non-melanoma skin cancer (basal-cell	Rate	2021	0.000479	0.001020507	0.000190515
DALYs (Dis	Pakistan	Female	55+ years	Non-melanoma skin cancer (basal-cell	Rate	2021	0.0004165	0.000859506	0.000168371
DALYs (Dis	Pakistan	Both	55+ years	Non-melanoma skin cancer (basal-cell	Rate	2021	0.0004497	0.000948916	0.000178018
DALYs (Dis	Suriname	Male	55+ years	Non-melanoma skin cancer (basal-cell	Number	2021	0.0073082	0.014082332	0.003015884
DALYs (Dis	Suriname	Female	55+ years	Non-melanoma skin cancer (basal-cell	Number	2021	0.0035242	0.006749261	0.001458231
DALYs (Dis	Suriname	Both	55+ years	Non-melanoma skin cancer (basal-cell	Number	2021	0.0108324	0.020930202	0.00449379
DALYs (Dis	Suriname	Male	55+ years	Non-melanoma skin cancer (basal-cell	Percent	2021	1.32E-07	2.50E-07	5.56E-08
DALYs (Dis	Suriname	Female	55+ years	Non-melanoma skin cancer (basal-cell	Percent	2021	7.59E-08	1.42E-07	3.30E-08
DALYs (Dis	Suriname	Both	55+ years	Non-melanoma skin cancer (basal-cell	Percent	2021	1.06E-07	2.00E-07	4.49E-08
DALYs (Dis	Suriname	Male	55+ years	Non-melanoma skin cancer (basal-cell	Rate	2021	0.0141114	0.027191522	0.00582336
DALYs (Dis	Suriname	Female	55+ years	Non-melanoma skin cancer (basal-cell	Rate	2021	0.0058821	0.011265052	0.002433903
DALYs (Dis	Suriname	Both	55+ years	Non-melanoma skin cancer (basal-cell	Rate	2021	0.0096975	0.018737419	0.004022992
DALYs (Dis	Saint Vinc	Male	55+ years	Non-melanoma skin cancer (basal-cell	Number	2021	0.0019284	0.003824694	0.000802728
DALYs (Dis	Saint Vinc	Female	55+ years	Non-melanoma skin cancer (basal-cell	Number	2021	0.000732	0.001439309	0.000290943
DALYs (Dis	Saint Vinc	Both	55+ years	Non-melanoma skin cancer (basal-cell	Number	2021	0.0026604	0.005280688	0.001101773
DALYs (Dis	Saint Vinc	Male	55+ years	Non-melanoma skin cancer (basal-cell	Percent	2021	1.49E-07	2.88E-07	6.35E-08
DALYs (Dis	Saint Vinc	Female	55+ years	Non-melanoma skin cancer (basal-cell	Percent	2021	7.81E-08	1.48E-07	3.24E-08
DALYs (Dis	Saint Vinc	Both	55+ years	Non-melanoma skin cancer (basal-cell	Percent	2021	1.19E-07	2.24E-07	5.17E-08
DALYs (Dis	Saint Vinc	Male	55+ years	Non-melanoma skin cancer (basal-cell	Rate	2021	0.0147277	0.029210806	0.006130771
DALYs (Dis	Saint Vinc	Female	55+ years	Non-melanoma skin cancer (basal-cell	Rate	2021	0.0059356	0.011671093	0.002359207
DALYs (Dis	Saint Vinc	Both	55+ years	Non-melanoma skin cancer (basal-cell	Rate	2021	0.0104633	0.020769119	0.004333309
DALYs (Dis	Antigua an	Male	55+ years	Non-melanoma skin cancer (basal-cell	Number	2021	0.0012887	0.002527027	0.000526275
DALYs (Dis	Antigua an	Female	55+ years	Non-melanoma skin cancer (basal-cell	Number	2021	0.0005842	0.001146769	0.000239165
DALYs (Dis	Antigua an	Both	55+ years	Non-melanoma skin cancer (basal-cell	Number	2021	0.001873	0.003650944	0.00076265
DALYs (Dis	Antigua an	Male	55+ years	Non-melanoma skin cancer (basal-cell	Percent	2021	1.55E-07	2.99E-07	6.63E-08
DALYs (Dis	Antigua an	Female	55+ years	Non-melanoma skin cancer (basal-cell	Percent	2021	8.07E-08	1.54E-07	3.53E-08
DALYs (Dis	Antigua an	Both	55+ years	Non-melanoma skin cancer (basal-cell	Percent	2021	1.20E-07	2.30E-07	5.21E-08
DALYs (Dis	Antigua an	Male	55+ years	Non-melanoma skin cancer (basal-cell	Rate	2021	0.0143803	0.028197547	0.005872376
DALYs (Dis	Antigua an	Female	55+ years	Non-melanoma skin cancer (basal-cell	Rate	2021	0.0058977	0.011576794	0.002414408
DALYs (Dis	Antigua an	Both	55+ years	Non-melanoma skin cancer (basal-cell	Rate	2021	0.0099269	0.019350307	0.004042109
DALYs (Dis	Egypt	Male	55+ years	Non-melanoma skin cancer (basal-cell	Number	2021	0.7230317	1.388294637	0.311574443
DALYs (Dis	Egypt	Female	55+ years	Non-melanoma skin cancer (basal-cell	Number	2021	0.3539628	0.701774786	0.14542318
DALYs (Dis	Egypt	Both	55+ years	Non-melanoma skin cancer (basal-cell	Number	2021	1.0769946	2.105924464	0.445767179
DALYs (Dis	Egypt	Male	55+ years	Non-melanoma skin cancer (basal-cell	Percent	2021	9.25E-08	1.79E-07	4.07E-08
DALYs (Dis	Egypt	Female	55+ years	Non-melanoma skin cancer (basal-cell	Percent	2021	6.16E-08	1.22E-07	2.58E-08
DALYs (Dis	Egypt	Both	55+ years	Non-melanoma skin cancer (basal-cell	Percent	2021	7.94E-08	1.55E-07	3.40E-08
DALYs (Dis	Egypt	Male	55+ years	Non-melanoma skin cancer (basal-cell	Rate	2021	0.0122522	0.023525461	0.00527981
DALYs (Dis	Egypt	Female	55+ years	Non-melanoma skin cancer (basal-cell	Rate	2021	0.0068536	0.013588161	0.002815766
DALYs (Dis	Egypt	Both	55+ years	Non-melanoma skin cancer (basal-cell	Rate	2021	0.0097326	0.019030846	0.004028315
DALYs (Dis	Greece	Male	55+ years	Non-melanoma skin cancer (basal-cell	Number	2021	2.0789634	4.222841398	0.79911165
DALYs (Dis	Greece	Female	55+ years	Non-melanoma skin cancer (basal-cell	Number	2021	1.7898415	3.565814406	0.709130245
DALYs (Dis	Greece	Both	55+ years	Non-melanoma skin cancer (basal-cell	Number	2021	3.8688049	7.689920101	1.646056312
DALYs (Dis	Greece	Male	55+ years	Non-melanoma skin cancer (basal-cell	Percent	2021	1.46E-06	2.93E-06	5.74E-07
DALYs (Dis	Greece	Female	55+ years	Non-melanoma skin cancer (basal-cell	Percent	2021	1.35E-06	2.67E-06	5.46E-07
DALYs (Dis	Greece	Both	55+ years	Non-melanoma skin cancer (basal-cell	Percent	2021	1.41E-06	2.73E-06	6.15E-07
DALYs (Dis	Greece	Male	55+ years	Non-melanoma skin cancer (basal-cell	Rate	2021	0.1230919	0.250027197	0.04731403
DALYs (Dis	Greece	Female	55+ years	Non-melanoma skin cancer (basal-cell	Rate	2021	0.0901004	0.179502651	0.035697528
DALYs (Dis	Greece	Both	55+ years	Non-melanoma skin cancer (basal-cell	Rate	2021	0.1052607	0.20922393	0.044785169
DALYs (Dis	Kazakhstan	Male	55+ years	Non-melanoma skin cancer (basal-cell	Number	1990	0.3983213	0.805552551	0.166670409
DALYs (Dis	Kazakhstan	Female	55+ years	Non-melanoma skin cancer (basal-cell	Number	1990	0.7657108	1.48883053	0.321828291
DALYs (Dis	Kazakhstan	Both	55+ years	Non-melanoma skin cancer (basal-cell	Number	1990	1.1640321	2.234245986	0.488258451
DALYs (Dis	Kazakhstan	Male	55+ years	Non-melanoma skin cancer (basal-cell	Percent	1990	3.99E-07	8.02E-07	1.72E-07
DALYs (Dis	Kazakhstan	Female	55+ years	Non-melanoma skin cancer (basal-cell	Percent	1990	6.74E-07	1.29E-06	2.98E-07
DALYs (Dis	Kazakhstan	Both	55+ years	Non-melanoma skin cancer (basal-cell	Percent	1990	5.46E-07	1.03E-06	2.37E-07

DALYs (Dis	Kazakhstan	Male	55+ years	Non-melanoma skin cancer (basal-cell	Rate	1990	0.0505743	0.102279978	0.021161929
DALYs (Dis	Kazakhstan	Female	55+ years	Non-melanoma skin cancer (basal-cell	Rate	1990	0.0587309	0.114195006	0.024684598
DALYs (Dis	Kazakhstan	Both	55+ years	Non-melanoma skin cancer (basal-cell	Rate	1990	0.0556592	0.106832354	0.023346489
DALYs (Dis	Mali	Male	55+ years	Non-melanoma skin cancer (basal-cell	Number	2021	0.0755965	0.151219451	0.031599625
DALYs (Dis	Mali	Female	55+ years	Non-melanoma skin cancer (basal-cell	Number	2021	0.0468673	0.093871489	0.018896418
DALYs (Dis	Mali	Both	55+ years	Non-melanoma skin cancer (basal-cell	Number	2021	0.1224638	0.247022722	0.049932363
DALYs (Dis	Mali	Male	55+ years	Non-melanoma skin cancer (basal-cell	Percent	2021	6.71E-08	1.31E-07	2.95E-08
DALYs (Dis	Mali	Female	55+ years	Non-melanoma skin cancer (basal-cell	Percent	2021	5.40E-08	1.06E-07	2.30E-08
DALYs (Dis	Mali	Both	55+ years	Non-melanoma skin cancer (basal-cell	Percent	2021	6.14E-08	1.19E-07	2.70E-08
DALYs (Dis	Mali	Male	55+ years	Non-melanoma skin cancer (basal-cell	Rate	2021	0.0100837	0.020170997	0.004215039
DALYs (Dis	Mali	Female	55+ years	Non-melanoma skin cancer (basal-cell	Rate	2021	0.0067175	0.013454703	0.002708444
DALYs (Dis	Mali	Both	55+ years	Non-melanoma skin cancer (basal-cell	Rate	2021	0.0084611	0.017066972	0.003449862
DALYs (Dis	Tunisia	Male	55+ years	Non-melanoma skin cancer (basal-cell	Number	2021	0.2180266	0.41555001	0.091469564
DALYs (Dis	Tunisia	Female	55+ years	Non-melanoma skin cancer (basal-cell	Number	2021	0.161707	0.31847813	0.066151214
DALYs (Dis	Tunisia	Both	55+ years	Non-melanoma skin cancer (basal-cell	Number	2021	0.3797336	0.739435809	0.155776516
DALYs (Dis	Tunisia	Male	55+ years	Non-melanoma skin cancer (basal-cell	Percent	2021	1.82E-07	3.43E-07	7.68E-08
DALYs (Dis	Tunisia	Female	55+ years	Non-melanoma skin cancer (basal-cell	Percent	2021	1.78E-07	3.43E-07	7.48E-08
DALYs (Dis	Tunisia	Both	55+ years	Non-melanoma skin cancer (basal-cell	Percent	2021	1.80E-07	3.42E-07	7.54E-08
DALYs (Dis	Tunisia	Male	55+ years	Non-melanoma skin cancer (basal-cell	Rate	2021	0.0191349	0.03647031	0.00802773
DALYs (Dis	Tunisia	Female	55+ years	Non-melanoma skin cancer (basal-cell	Rate	2021	0.0135013	0.026590492	0.005523121
DALYs (Dis	Tunisia	Both	55+ years	Non-melanoma skin cancer (basal-cell	Rate	2021	0.0162478	0.031638567	0.006665279
DALYs (Dis	Croatia	Male	55+ years	Non-melanoma skin cancer (basal-cell	Number	2021	0.4531533	0.902129688	0.196224234
DALYs (Dis	Croatia	Female	55+ years	Non-melanoma skin cancer (basal-cell	Number	2021	0.5401746	1.080574619	0.224490941
DALYs (Dis	Croatia	Both	55+ years	Non-melanoma skin cancer (basal-cell	Number	2021	0.9933279	1.948135284	0.436548101
DALYs (Dis	Croatia	Male	55+ years	Non-melanoma skin cancer (basal-cell	Percent	2021	6.91E-07	1.37E-06	3.02E-07
DALYs (Dis	Croatia	Female	55+ years	Non-melanoma skin cancer (basal-cell	Percent	2021	8.50E-07	1.63E-06	3.71E-07
DALYs (Dis	Croatia	Both	55+ years	Non-melanoma skin cancer (basal-cell	Percent	2021	7.69E-07	1.51E-06	3.34E-07
DALYs (Dis	Croatia	Male	55+ years	Non-melanoma skin cancer (basal-cell	Rate	2021	0.0689414	0.137247402	0.029852988
DALYs (Dis	Croatia	Female	55+ years	Non-melanoma skin cancer (basal-cell	Rate	2021	0.0647672	0.1295615	0.026916589
DALYs (Dis	Croatia	Both	55+ years	Non-melanoma skin cancer (basal-cell	Rate	2021	0.066607	0.130631055	0.029272474
DALYs (Dis	Jordan	Male	55+ years	Non-melanoma skin cancer (basal-cell	Number	1990	0.0221204	0.042709285	0.009344282
DALYs (Dis	Jordan	Female	55+ years	Non-melanoma skin cancer (basal-cell	Number	1990	0.0145453	0.028747522	0.006367526
DALYs (Dis	Jordan	Both	55+ years	Non-melanoma skin cancer (basal-cell	Number	1990	0.0366658	0.071626763	0.015876087
DALYs (Dis	Jordan	Male	55+ years	Non-melanoma skin cancer (basal-cell	Percent	1990	2.66E-07	5.01E-07	1.16E-07
DALYs (Dis	Jordan	Female	55+ years	Non-melanoma skin cancer (basal-cell	Percent	1990	1.64E-07	3.20E-07	7.17E-08
DALYs (Dis	Jordan	Both	55+ years	Non-melanoma skin cancer (basal-cell	Percent	1990	2.13E-07	4.10E-07	9.44E-08
DALYs (Dis	Jordan	Male	55+ years	Non-melanoma skin cancer (basal-cell	Rate	1990	0.0199154	0.038451915	0.00841282
DALYs (Dis	Jordan	Female	55+ years	Non-melanoma skin cancer (basal-cell	Rate	1990	0.013994	0.027657845	0.006126164
DALYs (Dis	Jordan	Both	55+ years	Non-melanoma skin cancer (basal-cell	Rate	1990	0.0170529	0.033312948	0.007383822
DALYs (Dis	Sweden	Male	55+ years	Non-melanoma skin cancer (basal-cell	Number	2021	2.5135138	5.117295562	1.069871023
DALYs (Dis	Sweden	Female	55+ years	Non-melanoma skin cancer (basal-cell	Number	2021	2.2923667	4.598026447	0.983982638
DALYs (Dis	Sweden	Both	55+ years	Non-melanoma skin cancer (basal-cell	Number	2021	4.8058806	9.567185144	2.097422331
DALYs (Dis	Sweden	Male	55+ years	Non-melanoma skin cancer (basal-cell	Percent	2021	2.60E-06	5.16E-06	1.07E-06
DALYs (Dis	Sweden	Female	55+ years	Non-melanoma skin cancer (basal-cell	Percent	2021	2.29E-06	4.58E-06	1.05E-06
DALYs (Dis	Sweden	Both	55+ years	Non-melanoma skin cancer (basal-cell	Percent	2021	2.44E-06	4.71E-06	1.05E-06
DALYs (Dis	Sweden	Male	55+ years	Non-melanoma skin cancer (basal-cell	Rate	2021	0.1551478	0.315867461	0.066038289
DALYs (Dis	Sweden	Female	55+ years	Non-melanoma skin cancer (basal-cell	Rate	2021	0.1312628	0.263286832	0.056343667
DALYs (Dis	Sweden	Both	55+ years	Non-melanoma skin cancer (basal-cell	Rate	2021	0.1427572	0.284190315	0.06230329
DALYs (Dis	Lesotho	Male	55+ years	Non-melanoma skin cancer (basal-cell	Number	2021	0.0233268	0.04701749	0.009882351
DALYs (Dis	Lesotho	Female	55+ years	Non-melanoma skin cancer (basal-cell	Number	2021	0.0252591	0.047851819	0.010669283
DALYs (Dis	Lesotho	Both	55+ years	Non-melanoma skin cancer (basal-cell	Number	2021	0.0485858	0.094379657	0.020715066
DALYs (Dis	Lesotho	Male	55+ years	Non-melanoma skin cancer (basal-cell	Percent	2021	9.77E-08	1.96E-07	4.15E-08
DALYs (Dis	Lesotho	Female	55+ years	Non-melanoma skin cancer (basal-cell	Percent	2021	1.04E-07	1.93E-07	4.56E-08
DALYs (Dis	Lesotho	Both	55+ years	Non-melanoma skin cancer (basal-cell	Percent	2021	1.01E-07	1.96E-07	4.31E-08
DALYs (Dis	Lesotho	Male	55+ years	Non-melanoma skin cancer (basal-cell	Rate	2021	0.0344751	0.069488124	0.014605332
DALYs (Dis	Lesotho	Female	55+ years	Non-melanoma skin cancer (basal-cell	Rate	2021	0.0223746	0.042387319	0.00945089

DALYs (Dis	Lesotho	Both	55+ years	Non-melanoma skin cancer (basal-cell	Rate	2021	0.0269092	0.052272129	0.011473029
DALYs (Dis	Honduras	Male	55+ years	Non-melanoma skin cancer (basal-cell	Number	2021	0.2999392	0.596323701	0.126770917
DALYs (Dis	Honduras	Female	55+ years	Non-melanoma skin cancer (basal-cell	Number	2021	0.3055895	0.567232623	0.134135055
DALYs (Dis	Honduras	Both	55+ years	Non-melanoma skin cancer (basal-cell	Number	2021	0.6055287	1.143767886	0.258544331
DALYs (Dis	Honduras	Male	55+ years	Non-melanoma skin cancer (basal-cell	Percent	2021	4.45E-07	8.86E-07	1.89E-07
DALYs (Dis	Honduras	Female	55+ years	Non-melanoma skin cancer (basal-cell	Percent	2021	5.13E-07	9.69E-07	2.25E-07
DALYs (Dis	Honduras	Both	55+ years	Non-melanoma skin cancer (basal-cell	Percent	2021	4.77E-07	8.99E-07	2.07E-07
DALYs (Dis	Honduras	Male	55+ years	Non-melanoma skin cancer (basal-cell	Rate	2021	0.0611851	0.121645161	0.025860231
DALYs (Dis	Honduras	Female	55+ years	Non-melanoma skin cancer (basal-cell	Rate	2021	0.0544283	0.101029286	0.023890673
DALYs (Dis	Honduras	Both	55+ years	Non-melanoma skin cancer (basal-cell	Rate	2021	0.0575779	0.108757364	0.024584184
DALYs (Dis	China	Male	55+ years	Non-melanoma skin cancer (basal-cell	Number	2021	135.74804	252.7658288	61.5026419
DALYs (Dis	China	Female	55+ years	Non-melanoma skin cancer (basal-cell	Number	2021	115.11333	220.6261256	50.73544808
DALYs (Dis	China	Both	55+ years	Non-melanoma skin cancer (basal-cell	Number	2021	250.86138	479.3169103	112.8593417
DALYs (Dis	China	Male	55+ years	Non-melanoma skin cancer (basal-cell	Percent	2021	9.75E-07	1.82E-06	4.47E-07
DALYs (Dis	China	Female	55+ years	Non-melanoma skin cancer (basal-cell	Percent	2021	1.01E-06	1.90E-06	4.74E-07
DALYs (Dis	China	Both	55+ years	Non-melanoma skin cancer (basal-cell	Percent	2021	9.88E-07	1.89E-06	4.76E-07
DALYs (Dis	China	Male	55+ years	Non-melanoma skin cancer (basal-cell	Rate	2021	0.073859	0.137527013	0.033462888
DALYs (Dis	China	Female	55+ years	Non-melanoma skin cancer (basal-cell	Rate	2021	0.0589836	0.113047953	0.025996643
DALYs (Dis	China	Both	55+ years	Non-melanoma skin cancer (basal-cell	Rate	2021	0.0661982	0.126483818	0.029781717
DALYs (Dis	Cuba	Male	55+ years	Non-melanoma skin cancer (basal-cell	Number	2021	0.4526883	0.869271647	0.186999266
DALYs (Dis	Cuba	Female	55+ years	Non-melanoma skin cancer (basal-cell	Number	2021	0.301419	0.599508117	0.123607857
DALYs (Dis	Cuba	Both	55+ years	Non-melanoma skin cancer (basal-cell	Number	2021	0.7541072	1.478582909	0.310441872
DALYs (Dis	Cuba	Male	55+ years	Non-melanoma skin cancer (basal-cell	Percent	2021	2.52E-07	4.77E-07	1.06E-07
DALYs (Dis	Cuba	Female	55+ years	Non-melanoma skin cancer (basal-cell	Percent	2021	2.07E-07	3.97E-07	8.92E-08
DALYs (Dis	Cuba	Both	55+ years	Non-melanoma skin cancer (basal-cell	Percent	2021	2.32E-07	4.43E-07	9.83E-08
DALYs (Dis	Cuba	Male	55+ years	Non-melanoma skin cancer (basal-cell	Rate	2021	0.0283049	0.054352293	0.011692362
DALYs (Dis	Cuba	Female	55+ years	Non-melanoma skin cancer (basal-cell	Rate	2021	0.0165823	0.032981345	0.006800164
DALYs (Dis	Cuba	Both	55+ years	Non-melanoma skin cancer (basal-cell	Rate	2021	0.022069	0.043270778	0.009085092
DALYs (Dis	United Kin	Male	55+ years	Non-melanoma skin cancer (basal-cell	Number	2021	12.448451	23.88020358	5.26228362
DALYs (Dis	United Kin	Female	55+ years	Non-melanoma skin cancer (basal-cell	Number	2021	10.740512	20.75113597	4.393386397
DALYs (Dis	United Kin	Both	55+ years	Non-melanoma skin cancer (basal-cell	Number	2021	23.188963	44.76154125	9.655616696
DALYs (Dis	United Kin	Male	55+ years	Non-melanoma skin cancer (basal-cell	Percent	2021	1.72E-06	3.23E-06	7.47E-07
DALYs (Dis	United Kin	Female	55+ years	Non-melanoma skin cancer (basal-cell	Percent	2021	1.53E-06	2.85E-06	6.58E-07
DALYs (Dis	United Kin	Both	55+ years	Non-melanoma skin cancer (basal-cell	Percent	2021	1.62E-06	3.04E-06	6.94E-07
DALYs (Dis	United Kin	Male	55+ years	Non-melanoma skin cancer (basal-cell	Rate	2021	0.1249394	0.239674745	0.052815148
DALYs (Dis	United Kin	Female	55+ years	Non-melanoma skin cancer (basal-cell	Rate	2021	0.0970534	0.18751142	0.039699519
DALYs (Dis	United Kin	Both	55+ years	Non-melanoma skin cancer (basal-cell	Rate	2021	0.1102651	0.212844245	0.04591313
DALYs (Dis	Switzerlan	Male	55+ years	Non-melanoma skin cancer (basal-cell	Number	2021	2.0693991	4.24938268	0.844930205
DALYs (Dis	Switzerlan	Female	55+ years	Non-melanoma skin cancer (basal-cell	Number	2021	1.8339109	3.702744681	0.734253673
DALYs (Dis	Switzerlan	Both	55+ years	Non-melanoma skin cancer (basal-cell	Number	2021	3.90331	7.577409141	1.64498323
DALYs (Dis	Switzerlan	Male	55+ years	Non-melanoma skin cancer (basal-cell	Percent	2021	2.65E-06	5.12E-06	1.15E-06
DALYs (Dis	Switzerlan	Female	55+ years	Non-melanoma skin cancer (basal-cell	Percent	2021	2.27E-06	4.42E-06	9.59E-07
DALYs (Dis	Switzerlan	Both	55+ years	Non-melanoma skin cancer (basal-cell	Percent	2021	2.46E-06	4.70E-06	1.08E-06
DALYs (Dis	Switzerlan	Male	55+ years	Non-melanoma skin cancer (basal-cell	Rate	2021	0.1492247	0.306423709	0.060928061
DALYs (Dis	Switzerlan	Female	55+ years	Non-melanoma skin cancer (basal-cell	Rate	2021	0.1187714	0.239804523	0.047553198
DALYs (Dis	Switzerlan	Both	55+ years	Non-melanoma skin cancer (basal-cell	Rate	2021	0.1331808	0.25854098	0.05612678
DALYs (Dis	Yemen	Male	55+ years	Non-melanoma skin cancer (basal-cell	Number	2021	0.1504846	0.288011943	0.061702414
DALYs (Dis	Yemen	Female	55+ years	Non-melanoma skin cancer (basal-cell	Number	2021	0.0934228	0.177926694	0.038619785
DALYs (Dis	Yemen	Both	55+ years	Non-melanoma skin cancer (basal-cell	Number	2021	0.2439074	0.4593723	0.101980484
DALYs (Dis	Yemen	Male	55+ years	Non-melanoma skin cancer (basal-cell	Percent	2021	1.03E-07	1.99E-07	4.39E-08
DALYs (Dis	Yemen	Female	55+ years	Non-melanoma skin cancer (basal-cell	Percent	2021	7.94E-08	1.47E-07	3.44E-08
DALYs (Dis	Yemen	Both	55+ years	Non-melanoma skin cancer (basal-cell	Percent	2021	9.24E-08	1.74E-07	3.98E-08
DALYs (Dis	Yemen	Male	55+ years	Non-melanoma skin cancer (basal-cell	Rate	2021	0.0137188	0.026256436	0.005625064
DALYs (Dis	Yemen	Female	55+ years	Non-melanoma skin cancer (basal-cell	Rate	2021	0.0081096	0.015444941	0.003352394
DALYs (Dis	Yemen	Both	55+ years	Non-melanoma skin cancer (basal-cell	Rate	2021	0.0108455	0.020426299	0.004534631
DALYs (Dis	Madagascar	Male	55+ years	Non-melanoma skin cancer (basal-cell	Number	2021	0.086095	0.169454843	0.036011429

DALYs (Dis	Madagascar	Female	55+ years	Non-melanoma skin cancer (basal-cell	Number	2021	0.057245	0.11727699	0.0231656
DALYs (Dis	Madagascar	Both	55+ years	Non-melanoma skin cancer (basal-cell	Number	2021	0.14334	0.285512143	0.060055642
DALYs (Dis	Madagascar	Male	55+ years	Non-melanoma skin cancer (basal-cell	Percent	2021	6.79E-08	1.33E-07	2.78E-08
DALYs (Dis	Madagascar	Female	55+ years	Non-melanoma skin cancer (basal-cell	Percent	2021	5.14E-08	1.02E-07	2.07E-08
DALYs (Dis	Madagascar	Both	55+ years	Non-melanoma skin cancer (basal-cell	Percent	2021	6.02E-08	1.18E-07	2.48E-08
DALYs (Dis	Madagascar	Male	55+ years	Non-melanoma skin cancer (basal-cell	Rate	2021	0.0095751	0.01884601	0.00400503
DALYs (Dis	Madagascar	Female	55+ years	Non-melanoma skin cancer (basal-cell	Rate	2021	0.0060415	0.012377187	0.002444853
DALYs (Dis	Madagascar	Both	55+ years	Non-melanoma skin cancer (basal-cell	Rate	2021	0.007762	0.015460831	0.003252086
DALYs (Dis	Syrian Ara	Male	55+ years	Non-melanoma skin cancer (basal-cell	Number	2021	0.1811597	0.350173007	0.075020294
DALYs (Dis	Syrian Ara	Female	55+ years	Non-melanoma skin cancer (basal-cell	Number	2021	0.1011879	0.197422327	0.041417211
DALYs (Dis	Syrian Ara	Both	55+ years	Non-melanoma skin cancer (basal-cell	Number	2021	0.2823476	0.540568235	0.115308586
DALYs (Dis	Syrian Ara	Male	55+ years	Non-melanoma skin cancer (basal-cell	Percent	2021	1.48E-07	2.85E-07	6.53E-08
DALYs (Dis	Syrian Ara	Female	55+ years	Non-melanoma skin cancer (basal-cell	Percent	2021	1.12E-07	2.12E-07	4.75E-08
DALYs (Dis	Syrian Ara	Both	55+ years	Non-melanoma skin cancer (basal-cell	Percent	2021	1.33E-07	2.52E-07	5.80E-08
DALYs (Dis	Syrian Ara	Male	55+ years	Non-melanoma skin cancer (basal-cell	Rate	2021	0.0149543	0.028905915	0.00619274
DALYs (Dis	Syrian Ara	Female	55+ years	Non-melanoma skin cancer (basal-cell	Rate	2021	0.0087892	0.017148073	0.003597493
DALYs (Dis	Syrian Ara	Both	55+ years	Non-melanoma skin cancer (basal-cell	Rate	2021	0.0119502	0.022879226	0.004880367
DALYs (Dis	Israel	Male	55+ years	Non-melanoma skin cancer (basal-cell	Number	2021	1.0591848	2.137845676	0.422548091
DALYs (Dis	Israel	Female	55+ years	Non-melanoma skin cancer (basal-cell	Number	2021	0.9253091	1.830358298	0.383971704
DALYs (Dis	Israel	Both	55+ years	Non-melanoma skin cancer (basal-cell	Number	2021	1.9844939	3.819778709	0.85745037
DALYs (Dis	Israel	Male	55+ years	Non-melanoma skin cancer (basal-cell	Percent	2021	1.92E-06	3.81E-06	7.92E-07
DALYs (Dis	Israel	Female	55+ years	Non-melanoma skin cancer (basal-cell	Percent	2021	1.66E-06	3.26E-06	7.11E-07
DALYs (Dis	Israel	Both	55+ years	Non-melanoma skin cancer (basal-cell	Percent	2021	1.79E-06	3.47E-06	8.03E-07
DALYs (Dis	Israel	Male	55+ years	Non-melanoma skin cancer (basal-cell	Rate	2021	0.1143073	0.230716431	0.045601415
DALYs (Dis	Israel	Female	55+ years	Non-melanoma skin cancer (basal-cell	Rate	2021	0.0863216	0.170753102	0.035820506
DALYs (Dis	Israel	Both	55+ years	Non-melanoma skin cancer (basal-cell	Rate	2021	0.099297	0.191128031	0.042903742
DALYs (Dis	Bolivia (P	Male	55+ years	Non-melanoma skin cancer (basal-cell	Number	2021	0.2393037	0.478866146	0.100031743
DALYs (Dis	Bolivia (P	Female	55+ years	Non-melanoma skin cancer (basal-cell	Number	2021	0.21532	0.423428368	0.090677251
DALYs (Dis	Bolivia (P	Both	55+ years	Non-melanoma skin cancer (basal-cell	Number	2021	0.4546237	0.897501007	0.189955326
DALYs (Dis	Bolivia (P	Male	55+ years	Non-melanoma skin cancer (basal-cell	Percent	2021	2.11E-07	4.06E-07	8.93E-08
DALYs (Dis	Bolivia (P	Female	55+ years	Non-melanoma skin cancer (basal-cell	Percent	2021	2.27E-07	4.33E-07	9.42E-08
DALYs (Dis	Bolivia (P	Both	55+ years	Non-melanoma skin cancer (basal-cell	Percent	2021	2.18E-07	4.18E-07	9.34E-08
DALYs (Dis	Bolivia (P	Male	55+ years	Non-melanoma skin cancer (basal-cell	Rate	2021	0.0327561	0.065547672	0.013692444
DALYs (Dis	Bolivia (P	Female	55+ years	Non-melanoma skin cancer (basal-cell	Rate	2021	0.0264225	0.051960135	0.011127271
DALYs (Dis	Bolivia (P	Both	55+ years	Non-melanoma skin cancer (basal-cell	Rate	2021	0.0294165	0.05807295	0.012291091
DALYs (Dis	Iran (Isla	Male	55+ years	Non-melanoma skin cancer (basal-cell	Number	2021	1.5447433	2.964203312	0.644064047
DALYs (Dis	Iran (Isla	Female	55+ years	Non-melanoma skin cancer (basal-cell	Number	2021	1.0204588	1.94634408	0.429890766
DALYs (Dis	Iran (Isla	Both	55+ years	Non-melanoma skin cancer (basal-cell	Number	2021	2.5652021	4.890574495	1.073954813
DALYs (Dis	Iran (Isla	Male	55+ years	Non-melanoma skin cancer (basal-cell	Percent	2021	2.48E-07	4.67E-07	1.08E-07
DALYs (Dis	Iran (Isla	Female	55+ years	Non-melanoma skin cancer (basal-cell	Percent	2021	2.11E-07	4.01E-07	9.31E-08
DALYs (Dis	Iran (Isla	Both	55+ years	Non-melanoma skin cancer (basal-cell	Percent	2021	2.32E-07	4.36E-07	1.02E-07
DALYs (Dis	Iran (Isla	Male	55+ years	Non-melanoma skin cancer (basal-cell	Rate	2021	0.0239841	0.046022997	0.009999907
DALYs (Dis	Iran (Isla	Female	55+ years	Non-melanoma skin cancer (basal-cell	Rate	2021	0.015579	0.029714086	0.006562977
DALYs (Dis	Iran (Isla	Both	55+ years	Non-melanoma skin cancer (basal-cell	Rate	2021	0.0197461	0.037646038	0.008266952
DALYs (Dis	Kuwait	Male	55+ years	Non-melanoma skin cancer (basal-cell	Number	2021	0.0394262	0.075636257	0.01662162
DALYs (Dis	Kuwait	Female	55+ years	Non-melanoma skin cancer (basal-cell	Number	2021	0.0188918	0.037299108	0.007896704
DALYs (Dis	Kuwait	Both	55+ years	Non-melanoma skin cancer (basal-cell	Number	2021	0.058318	0.112284412	0.024415401
DALYs (Dis	Kuwait	Male	55+ years	Non-melanoma skin cancer (basal-cell	Percent	2021	2.41E-07	4.46E-07	1.05E-07
DALYs (Dis	Kuwait	Female	55+ years	Non-melanoma skin cancer (basal-cell	Percent	2021	2.24E-07	4.28E-07	9.57E-08
DALYs (Dis	Kuwait	Both	55+ years	Non-melanoma skin cancer (basal-cell	Percent	2021	2.35E-07	4.42E-07	1.04E-07
DALYs (Dis	Kuwait	Male	55+ years	Non-melanoma skin cancer (basal-cell	Rate	2021	0.0144956	0.027808774	0.006111181
DALYs (Dis	Kuwait	Female	55+ years	Non-melanoma skin cancer (basal-cell	Rate	2021	0.0097261	0.019202911	0.004065505
DALYs (Dis	Kuwait	Both	55+ years	Non-melanoma skin cancer (basal-cell	Rate	2021	0.0125086	0.024083803	0.005236842
DALYs (Dis	Venezuela	Male	55+ years	Non-melanoma skin cancer (basal-cell	Number	2021	1.5133166	3.008928212	0.624363553
DALYs (Dis	Venezuela	Female	55+ years	Non-melanoma skin cancer (basal-cell	Number	2021	1.7598834	3.520294844	0.766318653
DALYs (Dis	Venezuela	Both	55+ years	Non-melanoma skin cancer (basal-cell	Number	2021	3.2732	6.377916018	1.394055185

DALYs (Dis	Venezuela	Male	55+ years	Non-melanoma skin cancer (basal-cell	Percent	2021	5.48E-07	1.09E-06	2.30E-07
DALYs (Dis	Venezuela	Female	55+ years	Non-melanoma skin cancer (basal-cell	Percent	2021	7.83E-07	1.54E-06	3.51E-07
DALYs (Dis	Venezuela	Both	55+ years	Non-melanoma skin cancer (basal-cell	Percent	2021	6.53E-07	1.27E-06	2.91E-07
DALYs (Dis	Venezuela	Male	55+ years	Non-melanoma skin cancer (basal-cell	Rate	2021	0.0628337	0.124932243	0.025923895
DALYs (Dis	Venezuela	Female	55+ years	Non-melanoma skin cancer (basal-cell	Rate	2021	0.0623245	0.12466778	0.027138422
DALYs (Dis	Venezuela	Both	55+ years	Non-melanoma skin cancer (basal-cell	Rate	2021	0.0625589	0.121897666	0.026643824
DALYs (Dis	Central Af	Male	55+ years	Non-melanoma skin cancer (basal-cell	Number	2021	0.0161108	0.032249325	0.006564144
DALYs (Dis	Central Af	Female	55+ years	Non-melanoma skin cancer (basal-cell	Number	2021	0.0122268	0.024445567	0.005051521
DALYs (Dis	Central Af	Both	55+ years	Non-melanoma skin cancer (basal-cell	Number	2021	0.0283376	0.056451376	0.011560021
DALYs (Dis	Central Af	Male	55+ years	Non-melanoma skin cancer (basal-cell	Percent	2021	4.17E-08	8.43E-08	1.77E-08
DALYs (Dis	Central Af	Female	55+ years	Non-melanoma skin cancer (basal-cell	Percent	2021	3.97E-08	7.85E-08	1.71E-08
DALYs (Dis	Central Af	Both	55+ years	Non-melanoma skin cancer (basal-cell	Percent	2021	4.08E-08	8.23E-08	1.74E-08
DALYs (Dis	Central Af	Male	55+ years	Non-melanoma skin cancer (basal-cell	Rate	2021	0.0099425	0.019902014	0.004050928
DALYs (Dis	Central Af	Female	55+ years	Non-melanoma skin cancer (basal-cell	Rate	2021	0.0062491	0.012494026	0.002581811
DALYs (Dis	Central Af	Both	55+ years	Non-melanoma skin cancer (basal-cell	Rate	2021	0.0079222	0.015781829	0.003231777
DALYs (Dis	Barbados	Male	55+ years	Non-melanoma skin cancer (basal-cell	Number	2021	0.0066756	0.01277219	0.002680619
DALYs (Dis	Barbados	Female	55+ years	Non-melanoma skin cancer (basal-cell	Number	2021	0.0031332	0.006209593	0.001292178
DALYs (Dis	Barbados	Both	55+ years	Non-melanoma skin cancer (basal-cell	Number	2021	0.0098088	0.018962748	0.003991517
DALYs (Dis	Barbados	Male	55+ years	Non-melanoma skin cancer (basal-cell	Percent	2021	1.96E-07	3.86E-07	7.78E-08
DALYs (Dis	Barbados	Female	55+ years	Non-melanoma skin cancer (basal-cell	Percent	2021	8.78E-08	1.70E-07	3.64E-08
DALYs (Dis	Barbados	Both	55+ years	Non-melanoma skin cancer (basal-cell	Percent	2021	1.41E-07	2.76E-07	5.74E-08
DALYs (Dis	Barbados	Male	55+ years	Non-melanoma skin cancer (basal-cell	Rate	2021	0.0161057	0.030814449	0.006467316
DALYs (Dis	Barbados	Female	55+ years	Non-melanoma skin cancer (basal-cell	Rate	2021	0.0063081	0.01250166	0.002601518
DALYs (Dis	Barbados	Both	55+ years	Non-melanoma skin cancer (basal-cell	Rate	2021	0.0107649	0.020811004	0.004380561
DALYs (Dis	Gambia	Male	55+ years	Non-melanoma skin cancer (basal-cell	Number	2021	0.0057011	0.011132444	0.002310908
DALYs (Dis	Gambia	Female	55+ years	Non-melanoma skin cancer (basal-cell	Number	2021	0.0039425	0.007677721	0.001574391
DALYs (Dis	Gambia	Both	55+ years	Non-melanoma skin cancer (basal-cell	Number	2021	0.0096436	0.018550402	0.003937546
DALYs (Dis	Gambia	Male	55+ years	Non-melanoma skin cancer (basal-cell	Percent	2021	4.99E-08	9.39E-08	2.12E-08
DALYs (Dis	Gambia	Female	55+ years	Non-melanoma skin cancer (basal-cell	Percent	2021	4.15E-08	7.93E-08	1.73E-08
DALYs (Dis	Gambia	Both	55+ years	Non-melanoma skin cancer (basal-cell	Percent	2021	4.60E-08	8.69E-08	1.95E-08
DALYs (Dis	Gambia	Male	55+ years	Non-melanoma skin cancer (basal-cell	Rate	2021	0.0075076	0.014660005	0.00304317
DALYs (Dis	Gambia	Female	55+ years	Non-melanoma skin cancer (basal-cell	Rate	2021	0.0050094	0.00975534	0.002000426
DALYs (Dis	Gambia	Both	55+ years	Non-melanoma skin cancer (basal-cell	Rate	2021	0.0062361	0.011995841	0.002546262
DALYs (Dis	Tokelau	Male	55+ years	Non-melanoma skin cancer (basal-cell	Number	2021	1.36E-07	3.51E-07	3.02E-08
DALYs (Dis	Tokelau	Female	55+ years	Non-melanoma skin cancer (basal-cell	Number	2021	1.32E-07	3.31E-07	3.03E-08
DALYs (Dis	Tokelau	Both	55+ years	Non-melanoma skin cancer (basal-cell	Number	2021	2.68E-07	6.76E-07	6.80E-08
DALYs (Dis	Tokelau	Male	55+ years	Non-melanoma skin cancer (basal-cell	Percent	2021	1.15E-09	2.88E-09	2.60E-10
DALYs (Dis	Tokelau	Female	55+ years	Non-melanoma skin cancer (basal-cell	Percent	2021	1.08E-09	2.55E-09	2.44E-10
DALYs (Dis	Tokelau	Both	55+ years	Non-melanoma skin cancer (basal-cell	Percent	2021	1.11E-09	2.73E-09	2.75E-10
DALYs (Dis	Tokelau	Male	55+ years	Non-melanoma skin cancer (basal-cell	Rate	2021	0.0001117	0.000288091	2.48E-05
DALYs (Dis	Tokelau	Female	55+ years	Non-melanoma skin cancer (basal-cell	Rate	2021	0.0001011	0.00025275	2.31E-05
DALYs (Dis	Tokelau	Both	55+ years	Non-melanoma skin cancer (basal-cell	Rate	2021	0.0001062	0.000267528	2.69E-05
DALYs (Dis	South Afri	Male	55+ years	Non-melanoma skin cancer (basal-cell	Number	2021	1.7721665	3.397028389	0.731483725
DALYs (Dis	South Afri	Female	55+ years	Non-melanoma skin cancer (basal-cell	Number	2021	1.6580705	3.189502868	0.694892433
DALYs (Dis	South Afri	Both	55+ years	Non-melanoma skin cancer (basal-cell	Number	2021	3.4302369	6.594090434	1.425698963
DALYs (Dis	South Afri	Male	55+ years	Non-melanoma skin cancer (basal-cell	Percent	2021	3.25E-07	6.13E-07	1.38E-07
DALYs (Dis	South Afri	Female	55+ years	Non-melanoma skin cancer (basal-cell	Percent	2021	3.11E-07	5.89E-07	1.34E-07
DALYs (Dis	South Afri	Both	55+ years	Non-melanoma skin cancer (basal-cell	Percent	2021	3.18E-07	5.99E-07	1.36E-07
DALYs (Dis	South Afri	Male	55+ years	Non-melanoma skin cancer (basal-cell	Rate	2021	0.0538445	0.103213405	0.022224991
DALYs (Dis	South Afri	Female	55+ years	Non-melanoma skin cancer (basal-cell	Rate	2021	0.0360663	0.069377954	0.015115276
DALYs (Dis	South Afri	Both	55+ years	Non-melanoma skin cancer (basal-cell	Rate	2021	0.0434837	0.083590626	0.018073011
DALYs (Dis	Solomon Is	Male	55+ years	Non-melanoma skin cancer (basal-cell	Number	2021	2.33E-05	5.89E-05	4.46E-06
DALYs (Dis	Solomon Is	Female	55+ years	Non-melanoma skin cancer (basal-cell	Number	2021	2.23E-05	5.80E-05	4.72E-06
DALYs (Dis	Solomon Is	Both	55+ years	Non-melanoma skin cancer (basal-cell	Number	2021	4.57E-05	0.000115058	1.04E-05
DALYs (Dis	Solomon Is	Male	55+ years	Non-melanoma skin cancer (basal-cell	Percent	2021	6.64E-10	1.70E-09	1.34E-10
DALYs (Dis	Solomon Is	Female	55+ years	Non-melanoma skin cancer (basal-cell	Percent	2021	7.77E-10	2.02E-09	1.66E-10

DALYs (Dis	Solomon Is	Both	55+ years	Non-melanoma skin cancer (basal-cell	Percent	2021	7.15E-10	1.82E-09	1.64E-10
DALYs (Dis	Solomon Is	Male	55+ years	Non-melanoma skin cancer (basal-cell	Rate	2021	8.49E-05	0.000214655	1.63E-05
DALYs (Dis	Solomon Is	Female	55+ years	Non-melanoma skin cancer (basal-cell	Rate	2021	8.34E-05	0.000216548	1.76E-05
DALYs (Dis	Solomon Is	Both	55+ years	Non-melanoma skin cancer (basal-cell	Rate	2021	8.42E-05	0.000212105	1.91E-05
DALYs (Dis	Togo	Male	55+ years	Non-melanoma skin cancer (basal-cell	Number	2021	0.0199789	0.040004356	0.00835866
DALYs (Dis	Togo	Female	55+ years	Non-melanoma skin cancer (basal-cell	Number	2021	0.0168176	0.033346755	0.006777472
DALYs (Dis	Togo	Both	55+ years	Non-melanoma skin cancer (basal-cell	Number	2021	0.0367965	0.073398175	0.015144659
DALYs (Dis	Togo	Male	55+ years	Non-melanoma skin cancer (basal-cell	Percent	2021	5.18E-08	1.03E-07	2.31E-08
DALYs (Dis	Togo	Female	55+ years	Non-melanoma skin cancer (basal-cell	Percent	2021	4.52E-08	8.75E-08	1.93E-08
DALYs (Dis	Togo	Both	55+ years	Non-melanoma skin cancer (basal-cell	Percent	2021	4.86E-08	9.47E-08	2.13E-08
DALYs (Dis	Togo	Male	55+ years	Non-melanoma skin cancer (basal-cell	Rate	2021	0.007319	0.01465498	0.003062066
DALYs (Dis	Togo	Female	55+ years	Non-melanoma skin cancer (basal-cell	Rate	2021	0.0047816	0.009481238	0.001926989
DALYs (Dis	Togo	Both	55+ years	Non-melanoma skin cancer (basal-cell	Rate	2021	0.0058904	0.011749582	0.002424357
DALYs (Dis	Bermuda	Male	55+ years	Non-melanoma skin cancer (basal-cell	Number	2021	0.0010705	0.002080279	0.000435119
DALYs (Dis	Bermuda	Female	55+ years	Non-melanoma skin cancer (basal-cell	Number	2021	0.0008195	0.00163258	0.000300204
DALYs (Dis	Bermuda	Both	55+ years	Non-melanoma skin cancer (basal-cell	Number	2021	0.00189	0.003640221	0.000714505
DALYs (Dis	Bermuda	Male	55+ years	Non-melanoma skin cancer (basal-cell	Percent	2021	1.23E-07	2.35E-07	5.08E-08
DALYs (Dis	Bermuda	Female	55+ years	Non-melanoma skin cancer (basal-cell	Percent	2021	1.16E-07	2.24E-07	4.45E-08
DALYs (Dis	Bermuda	Both	55+ years	Non-melanoma skin cancer (basal-cell	Percent	2021	1.20E-07	2.31E-07	4.66E-08
DALYs (Dis	Bermuda	Male	55+ years	Non-melanoma skin cancer (basal-cell	Rate	2021	0.0101545	0.019733271	0.004127483
DALYs (Dis	Bermuda	Female	55+ years	Non-melanoma skin cancer (basal-cell	Rate	2021	0.0064278	0.01280568	0.002354751
DALYs (Dis	Bermuda	Both	55+ years	Non-melanoma skin cancer (basal-cell	Rate	2021	0.0081146	0.015629397	0.003067747
DALYs (Dis	Malaysia	Male	55+ years	Non-melanoma skin cancer (basal-cell	Number	2021	0.1494063	0.2923399	0.063199847
DALYs (Dis	Malaysia	Female	55+ years	Non-melanoma skin cancer (basal-cell	Number	2021	0.1342965	0.259123064	0.055072031
DALYs (Dis	Malaysia	Both	55+ years	Non-melanoma skin cancer (basal-cell	Number	2021	0.2837028	0.548612846	0.120023657
DALYs (Dis	Malaysia	Male	55+ years	Non-melanoma skin cancer (basal-cell	Percent	2021	5.87E-08	1.14E-07	2.53E-08
DALYs (Dis	Malaysia	Female	55+ years	Non-melanoma skin cancer (basal-cell	Percent	2021	6.87E-08	1.27E-07	2.85E-08
DALYs (Dis	Malaysia	Both	55+ years	Non-melanoma skin cancer (basal-cell	Percent	2021	6.30E-08	1.20E-07	2.69E-08
DALYs (Dis	Malaysia	Male	55+ years	Non-melanoma skin cancer (basal-cell	Rate	2021	0.0061577	0.012048615	0.002604744
DALYs (Dis	Malaysia	Female	55+ years	Non-melanoma skin cancer (basal-cell	Rate	2021	0.0054229	0.010463473	0.002223826
DALYs (Dis	Malaysia	Both	55+ years	Non-melanoma skin cancer (basal-cell	Rate	2021	0.0057866	0.011189809	0.002448069
DALYs (Dis	American S	Male	55+ years	Non-melanoma skin cancer (basal-cell	Number	2021	4.47E-06	1.20E-05	9.68E-07
DALYs (Dis	American S	Female	55+ years	Non-melanoma skin cancer (basal-cell	Number	2021	4.69E-06	1.21E-05	1.06E-06
DALYs (Dis	American S	Both	55+ years	Non-melanoma skin cancer (basal-cell	Number	2021	9.16E-06	2.39E-05	2.21E-06
DALYs (Dis	American S	Male	55+ years	Non-melanoma skin cancer (basal-cell	Percent	2021	1.05E-09	2.73E-09	2.34E-10
DALYs (Dis	American S	Female	55+ years	Non-melanoma skin cancer (basal-cell	Percent	2021	1.26E-09	3.07E-09	2.90E-10
DALYs (Dis	American S	Both	55+ years	Non-melanoma skin cancer (basal-cell	Percent	2021	1.15E-09	2.87E-09	2.70E-10
DALYs (Dis	American S	Male	55+ years	Non-melanoma skin cancer (basal-cell	Rate	2021	0.0001048	0.000281012	2.27E-05
DALYs (Dis	American S	Female	55+ years	Non-melanoma skin cancer (basal-cell	Rate	2021	0.0001122	0.000289161	2.53E-05
DALYs (Dis	American S	Both	55+ years	Non-melanoma skin cancer (basal-cell	Rate	2021	0.0001085	0.000282672	2.61E-05
DALYs (Dis	Cabo Verde	Male	55+ years	Non-melanoma skin cancer (basal-cell	Number	2021	0.0029154	0.005822363	0.001219966
DALYs (Dis	Cabo Verde	Female	55+ years	Non-melanoma skin cancer (basal-cell	Number	2021	0.0027699	0.005345623	0.001151526
DALYs (Dis	Cabo Verde	Both	55+ years	Non-melanoma skin cancer (basal-cell	Number	2021	0.0056852	0.010997446	0.00234934
DALYs (Dis	Cabo Verde	Male	55+ years	Non-melanoma skin cancer (basal-cell	Percent	2021	8.30E-08	1.62E-07	3.36E-08
DALYs (Dis	Cabo Verde	Female	55+ years	Non-melanoma skin cancer (basal-cell	Percent	2021	9.06E-08	1.72E-07	3.81E-08
DALYs (Dis	Cabo Verde	Both	55+ years	Non-melanoma skin cancer (basal-cell	Percent	2021	8.65E-08	1.68E-07	3.58E-08
DALYs (Dis	Cabo Verde	Male	55+ years	Non-melanoma skin cancer (basal-cell	Rate	2021	0.0088213	0.0176172	0.00369135
DALYs (Dis	Cabo Verde	Female	55+ years	Non-melanoma skin cancer (basal-cell	Rate	2021	0.0063533	0.012261483	0.002641305
DALYs (Dis	Cabo Verde	Both	55+ years	Non-melanoma skin cancer (basal-cell	Rate	2021	0.0074175	0.014348328	0.003065175
DALYs (Dis	Mauritius	Male	55+ years	Non-melanoma skin cancer (basal-cell	Number	2021	0.0045326	0.008787868	0.001776692
DALYs (Dis	Mauritius	Female	55+ years	Non-melanoma skin cancer (basal-cell	Number	2021	0.0047287	0.009130266	0.001849166
DALYs (Dis	Mauritius	Both	55+ years	Non-melanoma skin cancer (basal-cell	Number	2021	0.0092613	0.017654591	0.003625711
DALYs (Dis	Mauritius	Male	55+ years	Non-melanoma skin cancer (basal-cell	Percent	2021	2.96E-08	5.74E-08	1.22E-08
DALYs (Dis	Mauritius	Female	55+ years	Non-melanoma skin cancer (basal-cell	Percent	2021	3.50E-08	6.74E-08	1.43E-08
DALYs (Dis	Mauritius	Both	55+ years	Non-melanoma skin cancer (basal-cell	Percent	2021	3.22E-08	6.05E-08	1.32E-08
DALYs (Dis	Mauritius	Male	55+ years	Non-melanoma skin cancer (basal-cell	Rate	2021	0.0029018	0.005626106	0.001137461

DALYs (Dis	Mauritius	Female	55+ years	Non-melanoma skin cancer (basal-cell	Rate	2021	0.0025963	0.005012941	0.001015278
DALYs (Dis	Mauritius	Both	55+ years	Non-melanoma skin cancer (basal-cell	Rate	2021	0.0027373	0.005218127	0.001071643
DALYs (Dis	Argentina	Male	55+ years	Non-melanoma skin cancer (basal-cell	Number	2021	2.9249028	5.942949495	1.162899128
DALYs (Dis	Argentina	Female	55+ years	Non-melanoma skin cancer (basal-cell	Number	2021	3.0130829	6.148006943	1.269955905
DALYs (Dis	Argentina	Both	55+ years	Non-melanoma skin cancer (basal-cell	Number	2021	5.9379857	11.42427546	2.441542529
DALYs (Dis	Argentina	Male	55+ years	Non-melanoma skin cancer (basal-cell	Percent	2021	7.54E-07	1.51E-06	3.14E-07
DALYs (Dis	Argentina	Female	55+ years	Non-melanoma skin cancer (basal-cell	Percent	2021	8.16E-07	1.61E-06	3.64E-07
DALYs (Dis	Argentina	Both	55+ years	Non-melanoma skin cancer (basal-cell	Percent	2021	7.84E-07	1.50E-06	3.32E-07
DALYs (Dis	Argentina	Male	55+ years	Non-melanoma skin cancer (basal-cell	Rate	2021	0.0703414	0.14292286	0.027966731
DALYs (Dis	Argentina	Female	55+ years	Non-melanoma skin cancer (basal-cell	Rate	2021	0.058222	0.118798442	0.024539462
DALYs (Dis	Argentina	Both	55+ years	Non-melanoma skin cancer (basal-cell	Rate	2021	0.0636214	0.122403261	0.02615945
DALYs (Dis	Angola	Male	55+ years	Non-melanoma skin cancer (basal-cell	Number	2021	0.0978771	0.188444341	0.040200882
DALYs (Dis	Angola	Female	55+ years	Non-melanoma skin cancer (basal-cell	Number	2021	0.073689	0.142820035	0.030307117
DALYs (Dis	Angola	Both	55+ years	Non-melanoma skin cancer (basal-cell	Number	2021	0.1715661	0.329141762	0.070342253
DALYs (Dis	Angola	Male	55+ years	Non-melanoma skin cancer (basal-cell	Percent	2021	6.80E-08	1.31E-07	2.81E-08
DALYs (Dis	Angola	Female	55+ years	Non-melanoma skin cancer (basal-cell	Percent	2021	5.98E-08	1.11E-07	2.50E-08
DALYs (Dis	Angola	Both	55+ years	Non-melanoma skin cancer (basal-cell	Percent	2021	6.42E-08	1.22E-07	2.72E-08
DALYs (Dis	Angola	Male	55+ years	Non-melanoma skin cancer (basal-cell	Rate	2021	0.011314	0.021783052	0.004646984
DALYs (Dis	Angola	Female	55+ years	Non-melanoma skin cancer (basal-cell	Rate	2021	0.0068963	0.013365978	0.002836327
DALYs (Dis	Angola	Both	55+ years	Non-melanoma skin cancer (basal-cell	Rate	2021	0.0088727	0.01702196	0.003637834
DALYs (Dis	Democratic	Male	55+ years	Non-melanoma skin cancer (basal-cell	Number	2021	0.3128452	0.613264256	0.131994697
DALYs (Dis	Democratic	Female	55+ years	Non-melanoma skin cancer (basal-cell	Number	2021	0.2336558	0.454339155	0.097454029
DALYs (Dis	Democratic	Both	55+ years	Non-melanoma skin cancer (basal-cell	Number	2021	0.546501	1.070163544	0.224995729
DALYs (Dis	Democratic	Male	55+ years	Non-melanoma skin cancer (basal-cell	Percent	2021	7.17E-08	1.37E-07	3.00E-08
DALYs (Dis	Democratic	Female	55+ years	Non-melanoma skin cancer (basal-cell	Percent	2021	5.98E-08	1.12E-07	2.57E-08
DALYs (Dis	Democratic	Both	55+ years	Non-melanoma skin cancer (basal-cell	Percent	2021	6.61E-08	1.25E-07	2.79E-08
DALYs (Dis	Democratic	Male	55+ years	Non-melanoma skin cancer (basal-cell	Rate	2021	0.0113249	0.022200023	0.004778177
DALYs (Dis	Democratic	Female	55+ years	Non-melanoma skin cancer (basal-cell	Rate	2021	0.0071294	0.013862937	0.002973547
DALYs (Dis	Democratic	Both	55+ years	Non-melanoma skin cancer (basal-cell	Rate	2021	0.0090483	0.017718483	0.003725209
DALYs (Dis	Zambia	Male	55+ years	Non-melanoma skin cancer (basal-cell	Number	2021	0.0480325	0.093886601	0.019937657
DALYs (Dis	Zambia	Female	55+ years	Non-melanoma skin cancer (basal-cell	Number	2021	0.0343655	0.067227237	0.014016091
DALYs (Dis	Zambia	Both	55+ years	Non-melanoma skin cancer (basal-cell	Number	2021	0.082398	0.160746736	0.034153463
DALYs (Dis	Zambia	Male	55+ years	Non-melanoma skin cancer (basal-cell	Percent	2021	4.35E-08	8.73E-08	1.79E-08
DALYs (Dis	Zambia	Female	55+ years	Non-melanoma skin cancer (basal-cell	Percent	2021	4.24E-08	8.25E-08	1.78E-08
DALYs (Dis	Zambia	Both	55+ years	Non-melanoma skin cancer (basal-cell	Percent	2021	4.30E-08	8.40E-08	1.80E-08
DALYs (Dis	Zambia	Male	55+ years	Non-melanoma skin cancer (basal-cell	Rate	2021	0.0093367	0.018249896	0.003875528
DALYs (Dis	Zambia	Female	55+ years	Non-melanoma skin cancer (basal-cell	Rate	2021	0.0060748	0.011883727	0.002477618
DALYs (Dis	Zambia	Both	55+ years	Non-melanoma skin cancer (basal-cell	Rate	2021	0.0076283	0.014881774	0.003161894
DALYs (Dis	Eritrea	Male	55+ years	Non-melanoma skin cancer (basal-cell	Number	2021	0.0152095	0.03047252	0.006491428
DALYs (Dis	Eritrea	Female	55+ years	Non-melanoma skin cancer (basal-cell	Number	2021	0.0153315	0.030336994	0.006320089
DALYs (Dis	Eritrea	Both	55+ years	Non-melanoma skin cancer (basal-cell	Number	2021	0.0305411	0.06099125	0.012809851
DALYs (Dis	Eritrea	Male	55+ years	Non-melanoma skin cancer (basal-cell	Percent	2021	5.17E-08	1.03E-07	2.32E-08
DALYs (Dis	Eritrea	Female	55+ years	Non-melanoma skin cancer (basal-cell	Percent	2021	4.81E-08	9.29E-08	2.07E-08
DALYs (Dis	Eritrea	Both	55+ years	Non-melanoma skin cancer (basal-cell	Percent	2021	4.99E-08	9.70E-08	2.19E-08
DALYs (Dis	Eritrea	Male	55+ years	Non-melanoma skin cancer (basal-cell	Rate	2021	0.0084828	0.016995518	0.003620481
DALYs (Dis	Eritrea	Female	55+ years	Non-melanoma skin cancer (basal-cell	Rate	2021	0.0058671	0.011609506	0.002418602
DALYs (Dis	Eritrea	Both	55+ years	Non-melanoma skin cancer (basal-cell	Rate	2021	0.0069316	0.013842487	0.002907305
DALYs (Dis	Mexico	Male	55+ years	Non-melanoma skin cancer (basal-cell	Number	2021	6.6849385	12.82619674	2.866581767
DALYs (Dis	Mexico	Female	55+ years	Non-melanoma skin cancer (basal-cell	Number	2021	7.098429	13.70487033	2.995969821
DALYs (Dis	Mexico	Both	55+ years	Non-melanoma skin cancer (basal-cell	Number	2021	13.783367	26.42947138	5.839175222
DALYs (Dis	Mexico	Male	55+ years	Non-melanoma skin cancer (basal-cell	Percent	2021	6.07E-07	1.14E-06	2.73E-07
DALYs (Dis	Mexico	Female	55+ years	Non-melanoma skin cancer (basal-cell	Percent	2021	7.38E-07	1.42E-06	3.30E-07
DALYs (Dis	Mexico	Both	55+ years	Non-melanoma skin cancer (basal-cell	Percent	2021	6.68E-07	1.27E-06	2.95E-07
DALYs (Dis	Mexico	Male	55+ years	Non-melanoma skin cancer (basal-cell	Rate	2021	0.066781	0.128130847	0.028636513
DALYs (Dis	Mexico	Female	55+ years	Non-melanoma skin cancer (basal-cell	Rate	2021	0.0615104	0.118757538	0.025961136
DALYs (Dis	Mexico	Both	55+ years	Non-melanoma skin cancer (basal-cell	Rate	2021	0.0639586	0.12264003	0.027095382

DALYs (Dis	Philippine	Male	55+ years	Non-melanoma skin cancer (basal-cell	Number	2021	0.2838243	0.556355894	0.113656962
DALYs (Dis	Philippine	Female	55+ years	Non-melanoma skin cancer (basal-cell	Number	2021	0.3186702	0.616703372	0.129695468
DALYs (Dis	Philippine	Both	55+ years	Non-melanoma skin cancer (basal-cell	Number	2021	0.6024945	1.15712793	0.24507655
DALYs (Dis	Philippine	Male	55+ years	Non-melanoma skin cancer (basal-cell	Percent	2021	3.35E-08	6.53E-08	1.40E-08
DALYs (Dis	Philippine	Female	55+ years	Non-melanoma skin cancer (basal-cell	Percent	2021	4.77E-08	9.13E-08	1.97E-08
DALYs (Dis	Philippine	Both	55+ years	Non-melanoma skin cancer (basal-cell	Percent	2021	3.97E-08	7.68E-08	1.62E-08
DALYs (Dis	Philippine	Male	55+ years	Non-melanoma skin cancer (basal-cell	Rate	2021	0.0044064	0.008637548	0.001764549
DALYs (Dis	Philippine	Female	55+ years	Non-melanoma skin cancer (basal-cell	Rate	2021	0.0042467	0.008218395	0.001728365
DALYs (Dis	Philippine	Both	55+ years	Non-melanoma skin cancer (basal-cell	Rate	2021	0.0043205	0.008297755	0.001757442
DALYs (Dis	Guinea	Male	55+ years	Non-melanoma skin cancer (basal-cell	Number	2021	0.0339113	0.066955553	0.013261868
DALYs (Dis	Guinea	Female	55+ years	Non-melanoma skin cancer (basal-cell	Number	2021	0.0200346	0.039920253	0.008091018
DALYs (Dis	Guinea	Both	55+ years	Non-melanoma skin cancer (basal-cell	Number	2021	0.0539459	0.106752119	0.021529022
DALYs (Dis	Guinea	Male	55+ years	Non-melanoma skin cancer (basal-cell	Percent	2021	4.69E-08	9.00E-08	1.96E-08
DALYs (Dis	Guinea	Female	55+ years	Non-melanoma skin cancer (basal-cell	Percent	2021	3.66E-08	7.23E-08	1.58E-08
DALYs (Dis	Guinea	Both	55+ years	Non-melanoma skin cancer (basal-cell	Percent	2021	4.25E-08	8.25E-08	1.81E-08
DALYs (Dis	Guinea	Male	55+ years	Non-melanoma skin cancer (basal-cell	Rate	2021	0.0071879	0.014192012	0.002811008
DALYs (Dis	Guinea	Female	55+ years	Non-melanoma skin cancer (basal-cell	Rate	2021	0.004535	0.009036334	0.00183148
DALYs (Dis	Guinea	Both	55+ years	Non-melanoma skin cancer (basal-cell	Rate	2021	0.005905	0.01168531	0.002356612
DALYs (Dis	Micronesia	Male	55+ years	Non-melanoma skin cancer (basal-cell	Number	2021	5.74E-06	1.54E-05	1.14E-06
DALYs (Dis	Micronesia	Female	55+ years	Non-melanoma skin cancer (basal-cell	Number	2021	6.41E-06	1.74E-05	1.39E-06
DALYs (Dis	Micronesia	Both	55+ years	Non-melanoma skin cancer (basal-cell	Number	2021	1.22E-05	3.32E-05	2.93E-06
DALYs (Dis	Micronesia	Male	55+ years	Non-melanoma skin cancer (basal-cell	Percent	2021	7.56E-10	1.99E-09	1.51E-10
DALYs (Dis	Micronesia	Female	55+ years	Non-melanoma skin cancer (basal-cell	Percent	2021	9.38E-10	2.47E-09	2.15E-10
DALYs (Dis	Micronesia	Both	55+ years	Non-melanoma skin cancer (basal-cell	Percent	2021	8.42E-10	2.22E-09	2.01E-10
DALYs (Dis	Micronesia	Male	55+ years	Non-melanoma skin cancer (basal-cell	Rate	2021	9.08E-05	0.000243182	1.80E-05
DALYs (Dis	Micronesia	Female	55+ years	Non-melanoma skin cancer (basal-cell	Rate	2021	9.37E-05	0.000254261	2.03E-05
DALYs (Dis	Micronesia	Both	55+ years	Non-melanoma skin cancer (basal-cell	Rate	2021	9.23E-05	0.000252003	2.23E-05
DALYs (Dis	Greenland	Male	55+ years	Non-melanoma skin cancer (basal-cell	Number	2021	0.0088829	0.018083593	0.003475579
DALYs (Dis	Greenland	Female	55+ years	Non-melanoma skin cancer (basal-cell	Number	2021	0.0063866	0.013430669	0.002641487
DALYs (Dis	Greenland	Both	55+ years	Non-melanoma skin cancer (basal-cell	Number	2021	0.0152696	0.030266488	0.006212231
DALYs (Dis	Greenland	Male	55+ years	Non-melanoma skin cancer (basal-cell	Percent	2021	1.45E-06	2.76E-06	5.64E-07
DALYs (Dis	Greenland	Female	55+ years	Non-melanoma skin cancer (basal-cell	Percent	2021	1.58E-06	3.16E-06	6.60E-07
DALYs (Dis	Greenland	Both	55+ years	Non-melanoma skin cancer (basal-cell	Percent	2021	1.50E-06	2.91E-06	6.14E-07
DALYs (Dis	Greenland	Male	55+ years	Non-melanoma skin cancer (basal-cell	Rate	2021	0.1208762	0.246076383	0.047294688
DALYs (Dis	Greenland	Female	55+ years	Non-melanoma skin cancer (basal-cell	Rate	2021	0.1025601	0.215676675	0.042418379
DALYs (Dis	Greenland	Both	55+ years	Non-melanoma skin cancer (basal-cell	Rate	2021	0.1124747	0.2229412	0.045758936
DALYs (Dis	Myanmar	Male	55+ years	Non-melanoma skin cancer (basal-cell	Number	2021	0.0988973	0.188767528	0.039385731
DALYs (Dis	Myanmar	Female	55+ years	Non-melanoma skin cancer (basal-cell	Number	2021	0.1035094	0.207069562	0.040128251
DALYs (Dis	Myanmar	Both	55+ years	Non-melanoma skin cancer (basal-cell	Number	2021	0.2024066	0.39241844	0.078458068
DALYs (Dis	Myanmar	Male	55+ years	Non-melanoma skin cancer (basal-cell	Percent	2021	2.16E-08	4.23E-08	8.66E-09
DALYs (Dis	Myanmar	Female	55+ years	Non-melanoma skin cancer (basal-cell	Percent	2021	2.45E-08	4.75E-08	9.66E-09
DALYs (Dis	Myanmar	Both	55+ years	Non-melanoma skin cancer (basal-cell	Percent	2021	2.30E-08	4.49E-08	9.25E-09
DALYs (Dis	Myanmar	Male	55+ years	Non-melanoma skin cancer (basal-cell	Rate	2021	0.0027157	0.005183527	0.001081526
DALYs (Dis	Myanmar	Female	55+ years	Non-melanoma skin cancer (basal-cell	Rate	2021	0.0021562	0.004313552	0.000835928
DALYs (Dis	Myanmar	Both	55+ years	Non-melanoma skin cancer (basal-cell	Rate	2021	0.0023976	0.004648338	0.000929364
DALYs (Dis	United Ara	Male	55+ years	Non-melanoma skin cancer (basal-cell	Number	1990	0.0041952	0.008268748	0.001701478
DALYs (Dis	United Ara	Female	55+ years	Non-melanoma skin cancer (basal-cell	Number	1990	0.0016412	0.003203479	0.000686237
DALYs (Dis	United Ara	Both	55+ years	Non-melanoma skin cancer (basal-cell	Number	1990	0.0058365	0.011505157	0.002359636
DALYs (Dis	United Ara	Male	55+ years	Non-melanoma skin cancer (basal-cell	Percent	1990	1.25E-07	2.48E-07	5.29E-08
DALYs (Dis	United Ara	Female	55+ years	Non-melanoma skin cancer (basal-cell	Percent	1990	6.77E-08	1.32E-07	2.97E-08
DALYs (Dis	United Ara	Both	55+ years	Non-melanoma skin cancer (basal-cell	Percent	1990	1.01E-07	1.94E-07	4.40E-08
DALYs (Dis	United Ara	Male	55+ years	Non-melanoma skin cancer (basal-cell	Rate	1990	0.0120672	0.023784351	0.004894158
DALYs (Dis	United Ara	Female	55+ years	Non-melanoma skin cancer (basal-cell	Rate	1990	0.0076837	0.01499744	0.003212694
DALYs (Dis	United Ara	Both	55+ years	Non-melanoma skin cancer (basal-cell	Rate	1990	0.0103989	0.02049892	0.004204201
DALYs (Dis	Côte d'Ivo	Male	55+ years	Non-melanoma skin cancer (basal-cell	Number	2021	0.0505846	0.098593416	0.020451563
DALYs (Dis	Côte d'Ivo	Female	55+ years	Non-melanoma skin cancer (basal-cell	Number	2021	0.025124	0.050729562	0.009906236

DALYs (Dis	Côte d'Ivo	Both	55+ years	Non-melanoma skin cancer (basal-cell	Number	2021	0.0757087	0.147854658	0.030038986
DALYs (Dis	Côte d'Ivo	Male	55+ years	Non-melanoma skin cancer (basal-cell	Percent	2021	4.03E-08	7.79E-08	1.70E-08
DALYs (Dis	Côte d'Ivo	Female	55+ years	Non-melanoma skin cancer (basal-cell	Percent	2021	2.98E-08	5.95E-08	1.20E-08
DALYs (Dis	Côte d'Ivo	Both	55+ years	Non-melanoma skin cancer (basal-cell	Percent	2021	3.61E-08	7.13E-08	1.55E-08
DALYs (Dis	Côte d'Ivo	Male	55+ years	Non-melanoma skin cancer (basal-cell	Rate	2021	0.0053687	0.01046398	0.002170578
DALYs (Dis	Côte d'Ivo	Female	55+ years	Non-melanoma skin cancer (basal-cell	Rate	2021	0.0029524	0.005961413	0.001164117
DALYs (Dis	Côte d'Ivo	Both	55+ years	Non-melanoma skin cancer (basal-cell	Rate	2021	0.004222	0.008245377	0.001675177
DALYs (Dis	Ukraine	Male	55+ years	Non-melanoma skin cancer (basal-cell	Number	2021	2.752782	5.407169172	1.160546082
DALYs (Dis	Ukraine	Female	55+ years	Non-melanoma skin cancer (basal-cell	Number	2021	4.7365095	9.330920769	1.87192571
DALYs (Dis	Ukraine	Both	55+ years	Non-melanoma skin cancer (basal-cell	Number	2021	7.4892915	14.33794692	3.131354378
DALYs (Dis	Ukraine	Male	55+ years	Non-melanoma skin cancer (basal-cell	Percent	2021	4.05E-07	8.09E-07	1.68E-07
DALYs (Dis	Ukraine	Female	55+ years	Non-melanoma skin cancer (basal-cell	Percent	2021	6.26E-07	1.24E-06	2.60E-07
DALYs (Dis	Ukraine	Both	55+ years	Non-melanoma skin cancer (basal-cell	Percent	2021	5.19E-07	1.01E-06	2.24E-07
DALYs (Dis	Ukraine	Male	55+ years	Non-melanoma skin cancer (basal-cell	Rate	2021	0.0528183	0.103748679	0.022267682
DALYs (Dis	Ukraine	Female	55+ years	Non-melanoma skin cancer (basal-cell	Rate	2021	0.0566143	0.111530181	0.022374664
DALYs (Dis	Ukraine	Both	55+ years	Non-melanoma skin cancer (basal-cell	Rate	2021	0.0551573	0.105596355	0.023061852
DALYs (Dis	Kenya	Male	55+ years	Non-melanoma skin cancer (basal-cell	Number	2021	0.1436818	0.28193924	0.058704114
DALYs (Dis	Kenya	Female	55+ years	Non-melanoma skin cancer (basal-cell	Number	2021	0.142867	0.28753501	0.058167097
DALYs (Dis	Kenya	Both	55+ years	Non-melanoma skin cancer (basal-cell	Number	2021	0.2865488	0.569028077	0.117719336
DALYs (Dis	Kenya	Male	55+ years	Non-melanoma skin cancer (basal-cell	Percent	2021	5.29E-08	1.02E-07	2.19E-08
DALYs (Dis	Kenya	Female	55+ years	Non-melanoma skin cancer (basal-cell	Percent	2021	6.66E-08	1.28E-07	2.82E-08
DALYs (Dis	Kenya	Both	55+ years	Non-melanoma skin cancer (basal-cell	Percent	2021	5.89E-08	1.13E-07	2.45E-08
DALYs (Dis	Kenya	Male	55+ years	Non-melanoma skin cancer (basal-cell	Rate	2021	0.0082145	0.016118897	0.003356204
DALYs (Dis	Kenya	Female	55+ years	Non-melanoma skin cancer (basal-cell	Rate	2021	0.0071733	0.014437025	0.002920548
DALYs (Dis	Kenya	Both	55+ years	Non-melanoma skin cancer (basal-cell	Rate	2021	0.0076602	0.015211513	0.003146926
DALYs (Dis	Tajikistan	Male	55+ years	Non-melanoma skin cancer (basal-cell	Number	2021	0.2561805	0.508972661	0.106183871
DALYs (Dis	Tajikistan	Female	55+ years	Non-melanoma skin cancer (basal-cell	Number	2021	0.2526694	0.491912228	0.103882995
DALYs (Dis	Tajikistan	Both	55+ years	Non-melanoma skin cancer (basal-cell	Number	2021	0.5088499	0.992246289	0.208954952
DALYs (Dis	Tajikistan	Male	55+ years	Non-melanoma skin cancer (basal-cell	Percent	2021	5.00E-07	9.42E-07	2.17E-07
DALYs (Dis	Tajikistan	Female	55+ years	Non-melanoma skin cancer (basal-cell	Percent	2021	5.90E-07	1.11E-06	2.52E-07
DALYs (Dis	Tajikistan	Both	55+ years	Non-melanoma skin cancer (basal-cell	Percent	2021	5.40E-07	1.02E-06	2.35E-07
DALYs (Dis	Tajikistan	Male	55+ years	Non-melanoma skin cancer (basal-cell	Rate	2021	0.0514315	0.102182786	0.021317773
DALYs (Dis	Tajikistan	Female	55+ years	Non-melanoma skin cancer (basal-cell	Rate	2021	0.0477596	0.092981212	0.019635956
DALYs (Dis	Tajikistan	Both	55+ years	Non-melanoma skin cancer (basal-cell	Rate	2021	0.0495402	0.096602362	0.020343278
DALYs (Dis	Puerto Ric	Male	55+ years	Non-melanoma skin cancer (basal-cell	Number	2021	0.0965279	0.189062513	0.038925517
DALYs (Dis	Puerto Ric	Female	55+ years	Non-melanoma skin cancer (basal-cell	Number	2021	0.0517871	0.102639538	0.021156046
DALYs (Dis	Puerto Ric	Both	55+ years	Non-melanoma skin cancer (basal-cell	Number	2021	0.148315	0.294823428	0.060795619
DALYs (Dis	Puerto Ric	Male	55+ years	Non-melanoma skin cancer (basal-cell	Percent	2021	2.53E-07	4.84E-07	1.06E-07
DALYs (Dis	Puerto Ric	Female	55+ years	Non-melanoma skin cancer (basal-cell	Percent	2021	1.42E-07	2.85E-07	6.24E-08
DALYs (Dis	Puerto Ric	Both	55+ years	Non-melanoma skin cancer (basal-cell	Percent	2021	1.99E-07	3.85E-07	8.30E-08
DALYs (Dis	Puerto Ric	Male	55+ years	Non-melanoma skin cancer (basal-cell	Rate	2021	0.0186087	0.036447571	0.007504082
DALYs (Dis	Puerto Ric	Female	55+ years	Non-melanoma skin cancer (basal-cell	Rate	2021	0.007984	0.015823815	0.003261602
DALYs (Dis	Puerto Ric	Both	55+ years	Non-melanoma skin cancer (basal-cell	Rate	2021	0.0127051	0.025255479	0.005207939
DALYs (Dis	Botswana	Male	55+ years	Non-melanoma skin cancer (basal-cell	Number	2021	0.0367482	0.070682206	0.01515523
DALYs (Dis	Botswana	Female	55+ years	Non-melanoma skin cancer (basal-cell	Number	2021	0.0314348	0.060706939	0.01342631
DALYs (Dis	Botswana	Both	55+ years	Non-melanoma skin cancer (basal-cell	Number	2021	0.0681829	0.132045094	0.028879119
DALYs (Dis	Botswana	Male	55+ years	Non-melanoma skin cancer (basal-cell	Percent	2021	1.76E-07	3.41E-07	7.42E-08
DALYs (Dis	Botswana	Female	55+ years	Non-melanoma skin cancer (basal-cell	Percent	2021	1.78E-07	3.39E-07	7.73E-08
DALYs (Dis	Botswana	Both	55+ years	Non-melanoma skin cancer (basal-cell	Percent	2021	1.77E-07	3.34E-07	7.62E-08
DALYs (Dis	Botswana	Male	55+ years	Non-melanoma skin cancer (basal-cell	Rate	2021	0.0352258	0.067754126	0.01452741
DALYs (Dis	Botswana	Female	55+ years	Non-melanoma skin cancer (basal-cell	Rate	2021	0.022762	0.043958105	0.009722037
DALYs (Dis	Botswana	Both	55+ years	Non-melanoma skin cancer (basal-cell	Rate	2021	0.0281255	0.054468782	0.011912676
DALYs (Dis	Sao Tome a	Male	55+ years	Non-melanoma skin cancer (basal-cell	Number	2021	0.0007307	0.001414509	0.000311714
DALYs (Dis	Sao Tome a	Female	55+ years	Non-melanoma skin cancer (basal-cell	Number	2021	0.0004826	0.000952373	0.000197188
DALYs (Dis	Sao Tome a	Both	55+ years	Non-melanoma skin cancer (basal-cell	Number	2021	0.0012134	0.002379019	0.000501148
DALYs (Dis	Sao Tome a	Male	55+ years	Non-melanoma skin cancer (basal-cell	Percent	2021	7.70E-08	1.47E-07	3.35E-08

DALYs (Dis	Sao Tome a	Female	55+ years	Non-melanoma skin cancer (basal-cell	Percent	2021	5.48E-08	1.05E-07	2.26E-08
DALYs (Dis	Sao Tome a	Both	55+ years	Non-melanoma skin cancer (basal-cell	Percent	2021	6.63E-08	1.28E-07	2.79E-08
DALYs (Dis	Sao Tome a	Male	55+ years	Non-melanoma skin cancer (basal-cell	Rate	2021	0.0083483	0.016160409	0.003561251
DALYs (Dis	Sao Tome a	Female	55+ years	Non-melanoma skin cancer (basal-cell	Rate	2021	0.005064	0.009992437	0.002068923
DALYs (Dis	Sao Tome a	Both	55+ years	Non-melanoma skin cancer (basal-cell	Rate	2021	0.0066363	0.013011572	0.002740932
DALYs (Dis	Poland	Male	55+ years	Non-melanoma skin cancer (basal-cell	Number	2021	3.166712	6.08115548	1.351397414
DALYs (Dis	Poland	Female	55+ years	Non-melanoma skin cancer (basal-cell	Number	2021	4.0921393	7.969299392	1.657833926
DALYs (Dis	Poland	Both	55+ years	Non-melanoma skin cancer (basal-cell	Number	2021	7.2588513	14.12256816	3.016791093
DALYs (Dis	Poland	Male	55+ years	Non-melanoma skin cancer (basal-cell	Percent	2021	5.60E-07	1.05E-06	2.47E-07
DALYs (Dis	Poland	Female	55+ years	Non-melanoma skin cancer (basal-cell	Percent	2021	7.80E-07	1.50E-06	3.28E-07
DALYs (Dis	Poland	Both	55+ years	Non-melanoma skin cancer (basal-cell	Percent	2021	6.66E-07	1.26E-06	2.86E-07
DALYs (Dis	Poland	Male	55+ years	Non-melanoma skin cancer (basal-cell	Rate	2021	0.0604142	0.116015563	0.0257818
DALYs (Dis	Poland	Female	55+ years	Non-melanoma skin cancer (basal-cell	Rate	2021	0.0595335	0.115939341	0.024118579
DALYs (Dis	Poland	Both	55+ years	Non-melanoma skin cancer (basal-cell	Rate	2021	0.0599145	0.116567541	0.024900564
DALYs (Dis	Luxembourg	Male	55+ years	Non-melanoma skin cancer (basal-cell	Number	2021	0.0877739	0.163090443	0.035680574
DALYs (Dis	Luxembourg	Female	55+ years	Non-melanoma skin cancer (basal-cell	Number	2021	0.0782074	0.152901076	0.032148095
DALYs (Dis	Luxembourg	Both	55+ years	Non-melanoma skin cancer (basal-cell	Number	2021	0.1659813	0.303152391	0.07168436
DALYs (Dis	Luxembourg	Male	55+ years	Non-melanoma skin cancer (basal-cell	Percent	2021	1.70E-06	3.12E-06	6.97E-07
DALYs (Dis	Luxembourg	Female	55+ years	Non-melanoma skin cancer (basal-cell	Percent	2021	1.59E-06	3.07E-06	7.20E-07
DALYs (Dis	Luxembourg	Both	55+ years	Non-melanoma skin cancer (basal-cell	Percent	2021	1.65E-06	2.98E-06	7.24E-07
DALYs (Dis	Luxembourg	Male	55+ years	Non-melanoma skin cancer (basal-cell	Rate	2021	0.1023513	0.190176253	0.04160635
DALYs (Dis	Luxembourg	Female	55+ years	Non-melanoma skin cancer (basal-cell	Rate	2021	0.0850594	0.166297187	0.034964684
DALYs (Dis	Luxembourg	Both	55+ years	Non-melanoma skin cancer (basal-cell	Rate	2021	0.0934043	0.170595939	0.040339648
DALYs (Dis	Dominican	Male	55+ years	Non-melanoma skin cancer (basal-cell	Number	2021	0.1286705	0.250118736	0.055649229
DALYs (Dis	Dominican	Female	55+ years	Non-melanoma skin cancer (basal-cell	Number	2021	0.0559166	0.104401588	0.022658259
DALYs (Dis	Dominican	Both	55+ years	Non-melanoma skin cancer (basal-cell	Number	2021	0.1845871	0.357677811	0.078282233
DALYs (Dis	Dominican	Male	55+ years	Non-melanoma skin cancer (basal-cell	Percent	2021	1.78E-07	3.36E-07	8.08E-08
DALYs (Dis	Dominican	Female	55+ years	Non-melanoma skin cancer (basal-cell	Percent	2021	9.72E-08	1.82E-07	4.12E-08
DALYs (Dis	Dominican	Both	55+ years	Non-melanoma skin cancer (basal-cell	Percent	2021	1.42E-07	2.67E-07	6.43E-08
DALYs (Dis	Dominican	Male	55+ years	Non-melanoma skin cancer (basal-cell	Rate	2021	0.0160088	0.03111901	0.006923707
DALYs (Dis	Dominican	Female	55+ years	Non-melanoma skin cancer (basal-cell	Rate	2021	0.0064474	0.012037891	0.002612581
DALYs (Dis	Dominican	Both	55+ years	Non-melanoma skin cancer (basal-cell	Rate	2021	0.0110463	0.021404711	0.004684687
DALYs (Dis	Austria	Male	55+ years	Non-melanoma skin cancer (basal-cell	Number	2021	1.2863323	2.517561748	0.538614568
DALYs (Dis	Austria	Female	55+ years	Non-melanoma skin cancer (basal-cell	Number	2021	1.2332433	2.403344935	0.492412854
DALYs (Dis	Austria	Both	55+ years	Non-melanoma skin cancer (basal-cell	Number	2021	2.5195756	4.972891171	1.06129306
DALYs (Dis	Austria	Male	55+ years	Non-melanoma skin cancer (basal-cell	Percent	2021	1.37E-06	2.61E-06	5.93E-07
DALYs (Dis	Austria	Female	55+ years	Non-melanoma skin cancer (basal-cell	Percent	2021	1.30E-06	2.49E-06	5.39E-07
DALYs (Dis	Austria	Both	55+ years	Non-melanoma skin cancer (basal-cell	Percent	2021	1.34E-06	2.57E-06	5.80E-07
DALYs (Dis	Austria	Male	55+ years	Non-melanoma skin cancer (basal-cell	Rate	2021	0.0948776	0.185690918	0.039727261
DALYs (Dis	Austria	Female	55+ years	Non-melanoma skin cancer (basal-cell	Rate	2021	0.077107	0.150266119	0.030787494
DALYs (Dis	Austria	Both	55+ years	Non-melanoma skin cancer (basal-cell	Rate	2021	0.0852598	0.168277486	0.035913058
DALYs (Dis	Samoa	Male	55+ years	Non-melanoma skin cancer (basal-cell	Number	2021	1.29E-05	3.38E-05	2.77E-06
DALYs (Dis	Samoa	Female	55+ years	Non-melanoma skin cancer (basal-cell	Number	2021	1.42E-05	3.63E-05	3.33E-06
DALYs (Dis	Samoa	Both	55+ years	Non-melanoma skin cancer (basal-cell	Number	2021	2.71E-05	6.87E-05	6.62E-06
DALYs (Dis	Samoa	Male	55+ years	Non-melanoma skin cancer (basal-cell	Percent	2021	1.07E-09	2.77E-09	2.38E-10
DALYs (Dis	Samoa	Female	55+ years	Non-melanoma skin cancer (basal-cell	Percent	2021	1.20E-09	3.02E-09	2.98E-10
DALYs (Dis	Samoa	Both	55+ years	Non-melanoma skin cancer (basal-cell	Percent	2021	1.13E-09	2.85E-09	2.83E-10
DALYs (Dis	Samoa	Male	55+ years	Non-melanoma skin cancer (basal-cell	Rate	2021	0.0001055	0.000276369	2.27E-05
DALYs (Dis	Samoa	Female	55+ years	Non-melanoma skin cancer (basal-cell	Rate	2021	0.0001146	0.000292885	2.69E-05
DALYs (Dis	Samoa	Both	55+ years	Non-melanoma skin cancer (basal-cell	Rate	2021	0.0001101	0.000278888	2.69E-05
DALYs (Dis	Uruguay	Male	55+ years	Non-melanoma skin cancer (basal-cell	Number	2021	0.3160663	0.61508777	0.131788084
DALYs (Dis	Uruguay	Female	55+ years	Non-melanoma skin cancer (basal-cell	Number	2021	0.3441742	0.70650894	0.13751183
DALYs (Dis	Uruguay	Both	55+ years	Non-melanoma skin cancer (basal-cell	Number	2021	0.6602405	1.308415709	0.27475719
DALYs (Dis	Uruguay	Male	55+ years	Non-melanoma skin cancer (basal-cell	Percent	2021	8.02E-07	1.55E-06	3.48E-07
DALYs (Dis	Uruguay	Female	55+ years	Non-melanoma skin cancer (basal-cell	Percent	2021	8.88E-07	1.79E-06	3.80E-07
DALYs (Dis	Uruguay	Both	55+ years	Non-melanoma skin cancer (basal-cell	Percent	2021	8.45E-07	1.67E-06	3.57E-07

DALYs (Dis	Uruguay	Male	55+ years	Non-melanoma skin cancer (basal-cell	Rate	2021	0.0811591	0.157941439	0.033840373
DALYs (Dis	Uruguay	Female	55+ years	Non-melanoma skin cancer (basal-cell	Rate	2021	0.0668492	0.137225668	0.026709008
DALYs (Dis	Uruguay	Both	55+ years	Non-melanoma skin cancer (basal-cell	Rate	2021	0.0730119	0.144689469	0.030383671
DALYs (Dis	Peru	Male	55+ years	Non-melanoma skin cancer (basal-cell	Number	2021	0.9091864	1.802604845	0.376279706
DALYs (Dis	Peru	Female	55+ years	Non-melanoma skin cancer (basal-cell	Number	2021	0.7933488	1.541451731	0.335422003
DALYs (Dis	Peru	Both	55+ years	Non-melanoma skin cancer (basal-cell	Number	2021	1.7025352	3.325430913	0.729351384
DALYs (Dis	Peru	Male	55+ years	Non-melanoma skin cancer (basal-cell	Percent	2021	2.91E-07	5.59E-07	1.22E-07
DALYs (Dis	Peru	Female	55+ years	Non-melanoma skin cancer (basal-cell	Percent	2021	3.26E-07	6.31E-07	1.41E-07
DALYs (Dis	Peru	Both	55+ years	Non-melanoma skin cancer (basal-cell	Percent	2021	3.06E-07	5.88E-07	1.30E-07
DALYs (Dis	Peru	Male	55+ years	Non-melanoma skin cancer (basal-cell	Rate	2021	0.033826	0.067065333	0.013999365
DALYs (Dis	Peru	Female	55+ years	Non-melanoma skin cancer (basal-cell	Rate	2021	0.027304	0.053050903	0.011543949
DALYs (Dis	Peru	Both	55+ years	Non-melanoma skin cancer (basal-cell	Rate	2021	0.0304381	0.059452308	0.0130394
DALYs (Dis	Libya	Male	55+ years	Non-melanoma skin cancer (basal-cell	Number	2021	0.0541393	0.105269267	0.022190374
DALYs (Dis	Libya	Female	55+ years	Non-melanoma skin cancer (basal-cell	Number	2021	0.0284872	0.05490865	0.011396439
DALYs (Dis	Libya	Both	55+ years	Non-melanoma skin cancer (basal-cell	Number	2021	0.0826266	0.161819348	0.033847907
DALYs (Dis	Libya	Male	55+ years	Non-melanoma skin cancer (basal-cell	Percent	2021	1.24E-07	2.35E-07	5.37E-08
DALYs (Dis	Libya	Female	55+ years	Non-melanoma skin cancer (basal-cell	Percent	2021	8.11E-08	1.51E-07	3.38E-08
DALYs (Dis	Libya	Both	55+ years	Non-melanoma skin cancer (basal-cell	Percent	2021	1.05E-07	1.96E-07	4.56E-08
DALYs (Dis	Libya	Male	55+ years	Non-melanoma skin cancer (basal-cell	Rate	2021	0.0128804	0.025044779	0.005279347
DALYs (Dis	Libya	Female	55+ years	Non-melanoma skin cancer (basal-cell	Rate	2021	0.006848	0.013199439	0.002739579
DALYs (Dis	Libya	Both	55+ years	Non-melanoma skin cancer (basal-cell	Rate	2021	0.0098798	0.019349056	0.00404726
DALYs (Dis	Sierra Leo	Male	55+ years	Non-melanoma skin cancer (basal-cell	Number	2021	0.0246096	0.048595999	0.010192791
DALYs (Dis	Sierra Leo	Female	55+ years	Non-melanoma skin cancer (basal-cell	Number	2021	0.0142312	0.028281341	0.005601373
DALYs (Dis	Sierra Leo	Both	55+ years	Non-melanoma skin cancer (basal-cell	Number	2021	0.0388407	0.077410384	0.015846933
DALYs (Dis	Sierra Leo	Male	55+ years	Non-melanoma skin cancer (basal-cell	Percent	2021	6.23E-08	1.19E-07	2.59E-08
DALYs (Dis	Sierra Leo	Female	55+ years	Non-melanoma skin cancer (basal-cell	Percent	2021	4.13E-08	7.69E-08	1.71E-08
DALYs (Dis	Sierra Leo	Both	55+ years	Non-melanoma skin cancer (basal-cell	Percent	2021	5.25E-08	9.98E-08	2.18E-08
DALYs (Dis	Sierra Leo	Male	55+ years	Non-melanoma skin cancer (basal-cell	Rate	2021	0.0079756	0.015749243	0.003303333
DALYs (Dis	Sierra Leo	Female	55+ years	Non-melanoma skin cancer (basal-cell	Rate	2021	0.0048156	0.009569936	0.001895412
DALYs (Dis	Sierra Leo	Both	55+ years	Non-melanoma skin cancer (basal-cell	Rate	2021	0.0064297	0.012814515	0.002623301
DALYs (Dis	Brazil	Male	55+ years	Non-melanoma skin cancer (basal-cell	Number	2021	15.566592	30.13504353	6.879095073
DALYs (Dis	Brazil	Female	55+ years	Non-melanoma skin cancer (basal-cell	Number	2021	15.981812	30.82841074	6.926608416
DALYs (Dis	Brazil	Both	55+ years	Non-melanoma skin cancer (basal-cell	Number	2021	31.548404	61.05747604	13.73966898
DALYs (Dis	Brazil	Male	55+ years	Non-melanoma skin cancer (basal-cell	Percent	2021	8.34E-07	1.57E-06	3.78E-07
DALYs (Dis	Brazil	Female	55+ years	Non-melanoma skin cancer (basal-cell	Percent	2021	9.17E-07	1.74E-06	4.23E-07
DALYs (Dis	Brazil	Both	55+ years	Non-melanoma skin cancer (basal-cell	Percent	2021	8.74E-07	1.64E-06	3.94E-07
DALYs (Dis	Brazil	Male	55+ years	Non-melanoma skin cancer (basal-cell	Rate	2021	0.0798488	0.154577701	0.035286317
DALYs (Dis	Brazil	Female	55+ years	Non-melanoma skin cancer (basal-cell	Rate	2021	0.0671147	0.129462215	0.029087911
DALYs (Dis	Brazil	Both	55+ years	Non-melanoma skin cancer (basal-cell	Rate	2021	0.072847	0.140985108	0.03172566
YLDs (Year	Democratic	Male	55+ years	Non-melanoma skin cancer (basal-cell	Number	1990	0.0043598	0.009392698	0.001549353
YLDs (Year	Democratic	Female	55+ years	Non-melanoma skin cancer (basal-cell	Number	1990	0.0064834	0.013519716	0.002185241
YLDs (Year	Democratic	Both	55+ years	Non-melanoma skin cancer (basal-cell	Number	1990	0.0108432	0.023162127	0.003715873
YLDs (Year	Democratic	Male	55+ years	Non-melanoma skin cancer (basal-cell	Percent	1990	2.63E-08	5.30E-08	1.06E-08
YLDs (Year	Democratic	Female	55+ years	Non-melanoma skin cancer (basal-cell	Percent	1990	1.95E-08	4.18E-08	7.22E-09
YLDs (Year	Democratic	Both	55+ years	Non-melanoma skin cancer (basal-cell	Percent	1990	2.17E-08	4.49E-08	8.46E-09
YLDs (Year	Democratic	Male	55+ years	Non-melanoma skin cancer (basal-cell	Rate	1990	0.0004344	0.000935946	0.000154387
YLDs (Year	Democratic	Female	55+ years	Non-melanoma skin cancer (basal-cell	Rate	1990	0.0003911	0.00081551	0.000131814
YLDs (Year	Democratic	Both	55+ years	Non-melanoma skin cancer (basal-cell	Rate	1990	0.0004074	0.000870307	0.000139622
YLDs (Year	Democratic	Male	55+ years	Non-melanoma skin cancer (basal-cell	Number	2021	0.0105836	0.022324063	0.003801983
YLDs (Year	Democratic	Female	55+ years	Non-melanoma skin cancer (basal-cell	Number	2021	0.0141169	0.028726728	0.004894149
YLDs (Year	Democratic	Both	55+ years	Non-melanoma skin cancer (basal-cell	Number	2021	0.0247005	0.050925251	0.008603234
YLDs (Year	Democratic	Male	55+ years	Non-melanoma skin cancer (basal-cell	Percent	2021	2.64E-08	5.33E-08	1.08E-08
YLDs (Year	Democratic	Female	55+ years	Non-melanoma skin cancer (basal-cell	Percent	2021	2.04E-08	4.08E-08	7.55E-09
YLDs (Year	Democratic	Both	55+ years	Non-melanoma skin cancer (basal-cell	Percent	2021	2.26E-08	4.50E-08	8.95E-09
YLDs (Year	Democratic	Male	55+ years	Non-melanoma skin cancer (basal-cell	Rate	2021	0.0004479	0.000944689	0.000160889
YLDs (Year	Democratic	Female	55+ years	Non-melanoma skin cancer (basal-cell	Rate	2021	0.0004314	0.00087778	0.000149547

YLDs (Year	Democratic	Both	55+ years	Non-melanoma skin cancer (basal-cell	Rate	2021	0.0004383	0.000903608	0.000152654
YLDs (Year	Sri Lanka	Male	55+ years	Non-melanoma skin cancer (basal-cell	Number	1990	0.0357593	0.070640133	0.015905976
YLDs (Year	Sri Lanka	Female	55+ years	Non-melanoma skin cancer (basal-cell	Number	1990	0.0186949	0.036496472	0.007813614
YLDs (Year	Sri Lanka	Both	55+ years	Non-melanoma skin cancer (basal-cell	Number	1990	0.0544542	0.105280478	0.024158941
YLDs (Year	Sri Lanka	Male	55+ years	Non-melanoma skin cancer (basal-cell	Percent	1990	2.01E-07	3.76E-07	9.62E-08
YLDs (Year	Sri Lanka	Female	55+ years	Non-melanoma skin cancer (basal-cell	Percent	1990	1.00E-07	1.91E-07	4.53E-08
YLDs (Year	Sri Lanka	Both	55+ years	Non-melanoma skin cancer (basal-cell	Percent	1990	1.50E-07	2.83E-07	6.98E-08
YLDs (Year	Sri Lanka	Male	55+ years	Non-melanoma skin cancer (basal-cell	Rate	1990	0.003987	0.007875957	0.001773422
YLDs (Year	Sri Lanka	Female	55+ years	Non-melanoma skin cancer (basal-cell	Rate	1990	0.0021175	0.004133853	0.000885026
YLDs (Year	Sri Lanka	Both	55+ years	Non-melanoma skin cancer (basal-cell	Rate	1990	0.0030596	0.005915375	0.001357414
YLDs (Year	Sri Lanka	Male	55+ years	Non-melanoma skin cancer (basal-cell	Number	2021	0.0674105	0.133303805	0.027186885
YLDs (Year	Sri Lanka	Female	55+ years	Non-melanoma skin cancer (basal-cell	Number	2021	0.0484237	0.095122752	0.01815871
YLDs (Year	Sri Lanka	Both	55+ years	Non-melanoma skin cancer (basal-cell	Number	2021	0.1158342	0.231478144	0.045122084
YLDs (Year	Sri Lanka	Male	55+ years	Non-melanoma skin cancer (basal-cell	Percent	2021	1.47E-07	2.83E-07	6.55E-08
YLDs (Year	Sri Lanka	Female	55+ years	Non-melanoma skin cancer (basal-cell	Percent	2021	7.77E-08	1.47E-07	3.16E-08
YLDs (Year	Sri Lanka	Both	55+ years	Non-melanoma skin cancer (basal-cell	Percent	2021	1.07E-07	2.08E-07	4.52E-08
YLDs (Year	Sri Lanka	Male	55+ years	Non-melanoma skin cancer (basal-cell	Rate	2021	0.003138	0.006205376	0.001265567
YLDs (Year	Sri Lanka	Female	55+ years	Non-melanoma skin cancer (basal-cell	Rate	2021	0.0018149	0.003565179	0.000680584
YLDs (Year	Sri Lanka	Both	55+ years	Non-melanoma skin cancer (basal-cell	Rate	2021	0.002405	0.004806136	0.000936861
YLDs (Year	Maldives	Male	55+ years	Non-melanoma skin cancer (basal-cell	Number	1990	0.0002724	0.000549518	0.000104102
YLDs (Year	Maldives	Female	55+ years	Non-melanoma skin cancer (basal-cell	Number	1990	0.0001253	0.00024851	4.85E-05
YLDs (Year	Maldives	Both	55+ years	Non-melanoma skin cancer (basal-cell	Number	1990	0.0003977	0.000790553	0.000152111
YLDs (Year	Maldives	Male	55+ years	Non-melanoma skin cancer (basal-cell	Percent	1990	1.65E-07	3.14E-07	7.05E-08
YLDs (Year	Maldives	Female	55+ years	Non-melanoma skin cancer (basal-cell	Percent	1990	1.00E-07	1.95E-07	4.01E-08
YLDs (Year	Maldives	Both	55+ years	Non-melanoma skin cancer (basal-cell	Percent	1990	1.37E-07	2.66E-07	5.83E-08
YLDs (Year	Maldives	Male	55+ years	Non-melanoma skin cancer (basal-cell	Rate	1990	0.0029803	0.006011529	0.001138839
YLDs (Year	Maldives	Female	55+ years	Non-melanoma skin cancer (basal-cell	Rate	1990	0.0020249	0.004015509	0.000784175
YLDs (Year	Maldives	Both	55+ years	Non-melanoma skin cancer (basal-cell	Rate	1990	0.0025946	0.00515696	0.000992254
YLDs (Year	Maldives	Male	55+ years	Non-melanoma skin cancer (basal-cell	Number	2021	0.0009014	0.001712862	0.000362925
YLDs (Year	Maldives	Female	55+ years	Non-melanoma skin cancer (basal-cell	Number	2021	0.0005724	0.001127811	0.000229554
YLDs (Year	Maldives	Both	55+ years	Non-melanoma skin cancer (basal-cell	Number	2021	0.0014738	0.002826965	0.000584882
YLDs (Year	Maldives	Male	55+ years	Non-melanoma skin cancer (basal-cell	Percent	2021	1.71E-07	3.12E-07	7.29E-08
YLDs (Year	Maldives	Female	55+ years	Non-melanoma skin cancer (basal-cell	Percent	2021	1.10E-07	2.13E-07	4.64E-08
YLDs (Year	Maldives	Both	55+ years	Non-melanoma skin cancer (basal-cell	Percent	2021	1.40E-07	2.64E-07	5.97E-08
YLDs (Year	Maldives	Male	55+ years	Non-melanoma skin cancer (basal-cell	Rate	2021	0.0030709	0.005835548	0.001236448
YLDs (Year	Maldives	Female	55+ years	Non-melanoma skin cancer (basal-cell	Rate	2021	0.0022855	0.004503284	0.000916596
YLDs (Year	Maldives	Both	55+ years	Non-melanoma skin cancer (basal-cell	Rate	2021	0.0027093	0.00519697	0.001075222
YLDs (Year	Lao People	Male	55+ years	Non-melanoma skin cancer (basal-cell	Number	1990	0.0041799	0.008619515	0.001638842
YLDs (Year	Lao People	Female	55+ years	Non-melanoma skin cancer (basal-cell	Number	1990	0.003496	0.006665181	0.001407184
YLDs (Year	Lao People	Both	55+ years	Non-melanoma skin cancer (basal-cell	Number	1990	0.0076759	0.015225249	0.003042647
YLDs (Year	Lao People	Male	55+ years	Non-melanoma skin cancer (basal-cell	Percent	1990	1.33E-07	2.56E-07	5.65E-08
YLDs (Year	Lao People	Female	55+ years	Non-melanoma skin cancer (basal-cell	Percent	1990	9.37E-08	1.83E-07	3.94E-08
YLDs (Year	Lao People	Both	55+ years	Non-melanoma skin cancer (basal-cell	Percent	1990	1.12E-07	2.14E-07	4.68E-08
YLDs (Year	Lao People	Male	55+ years	Non-melanoma skin cancer (basal-cell	Rate	1990	0.0025517	0.005261998	0.001000472
YLDs (Year	Lao People	Female	55+ years	Non-melanoma skin cancer (basal-cell	Rate	1990	0.0019517	0.003720905	0.000785575
YLDs (Year	Lao People	Both	55+ years	Non-melanoma skin cancer (basal-cell	Rate	1990	0.0022383	0.004439691	0.000887238
YLDs (Year	Lao People	Male	55+ years	Non-melanoma skin cancer (basal-cell	Number	2021	0.0093188	0.01845766	0.00370048
YLDs (Year	Lao People	Female	55+ years	Non-melanoma skin cancer (basal-cell	Number	2021	0.0077444	0.015308261	0.003022034
YLDs (Year	Lao People	Both	55+ years	Non-melanoma skin cancer (basal-cell	Number	2021	0.0170632	0.033434052	0.006834421
YLDs (Year	Lao People	Male	55+ years	Non-melanoma skin cancer (basal-cell	Percent	2021	1.36E-07	2.55E-07	5.77E-08
YLDs (Year	Lao People	Female	55+ years	Non-melanoma skin cancer (basal-cell	Percent	2021	9.34E-08	1.78E-07	4.01E-08
YLDs (Year	Lao People	Both	55+ years	Non-melanoma skin cancer (basal-cell	Percent	2021	1.13E-07	2.10E-07	4.76E-08
YLDs (Year	Lao People	Male	55+ years	Non-melanoma skin cancer (basal-cell	Rate	2021	0.0024928	0.004937366	0.000989867
YLDs (Year	Lao People	Female	55+ years	Non-melanoma skin cancer (basal-cell	Rate	2021	0.0019394	0.003833651	0.000756809
YLDs (Year	Lao People	Both	55+ years	Non-melanoma skin cancer (basal-cell	Rate	2021	0.002207	0.004324399	0.000883972
YLDs (Year	Myanmar	Male	55+ years	Non-melanoma skin cancer (basal-cell	Number	1990	0.0494312	0.099587833	0.018760294

YLDs (Year	Myanmar	Female	55+ years	Non-melanoma skin cancer (basal-cell	Number	1990	0.0433674	0.086931286	0.0161373
YLDs (Year	Myanmar	Both	55+ years	Non-melanoma skin cancer (basal-cell	Number	1990	0.0927986	0.181734956	0.035541117
YLDs (Year	Myanmar	Male	55+ years	Non-melanoma skin cancer (basal-cell	Percent	1990	1.35E-07	2.59E-07	5.66E-08
YLDs (Year	Myanmar	Female	55+ years	Non-melanoma skin cancer (basal-cell	Percent	1990	9.90E-08	1.93E-07	4.11E-08
YLDs (Year	Myanmar	Both	55+ years	Non-melanoma skin cancer (basal-cell	Percent	1990	1.15E-07	2.23E-07	4.80E-08
YLDs (Year	Myanmar	Male	55+ years	Non-melanoma skin cancer (basal-cell	Rate	1990	0.0026942	0.005427911	0.001022506
YLDs (Year	Myanmar	Female	55+ years	Non-melanoma skin cancer (basal-cell	Rate	1990	0.0020852	0.004179859	0.000775919
YLDs (Year	Myanmar	Both	55+ years	Non-melanoma skin cancer (basal-cell	Rate	1990	0.0023706	0.004642607	0.000907935
YLDs (Year	Myanmar	Male	55+ years	Non-melanoma skin cancer (basal-cell	Number	2021	0.0988973	0.188767528	0.039385731
YLDs (Year	Myanmar	Female	55+ years	Non-melanoma skin cancer (basal-cell	Number	2021	0.1035094	0.207069562	0.040128251
YLDs (Year	Myanmar	Both	55+ years	Non-melanoma skin cancer (basal-cell	Number	2021	0.2024066	0.39241844	0.078458068
YLDs (Year	Myanmar	Male	55+ years	Non-melanoma skin cancer (basal-cell	Percent	2021	1.38E-07	2.64E-07	6.01E-08
YLDs (Year	Myanmar	Female	55+ years	Non-melanoma skin cancer (basal-cell	Percent	2021	9.88E-08	1.92E-07	4.21E-08
YLDs (Year	Myanmar	Both	55+ years	Non-melanoma skin cancer (basal-cell	Percent	2021	1.15E-07	2.19E-07	4.90E-08
YLDs (Year	Myanmar	Male	55+ years	Non-melanoma skin cancer (basal-cell	Rate	2021	0.0027157	0.005183527	0.001081526
YLDs (Year	Myanmar	Female	55+ years	Non-melanoma skin cancer (basal-cell	Rate	2021	0.0021562	0.004313552	0.000835928
YLDs (Year	Myanmar	Both	55+ years	Non-melanoma skin cancer (basal-cell	Rate	2021	0.0023976	0.004648338	0.000929364
YLDs (Year	China	Male	55+ years	Non-melanoma skin cancer (basal-cell	Number	1990	5.6208006	10.95188615	2.43307582
YLDs (Year	China	Female	55+ years	Non-melanoma skin cancer (basal-cell	Number	1990	5.8467486	11.25670861	2.451742923
YLDs (Year	China	Both	55+ years	Non-melanoma skin cancer (basal-cell	Number	1990	11.467549	22.20324041	4.88493904
YLDs (Year	China	Male	55+ years	Non-melanoma skin cancer (basal-cell	Percent	1990	4.56E-07	8.42E-07	2.14E-07
YLDs (Year	China	Female	55+ years	Non-melanoma skin cancer (basal-cell	Percent	1990	3.86E-07	7.18E-07	1.76E-07
YLDs (Year	China	Both	55+ years	Non-melanoma skin cancer (basal-cell	Percent	1990	4.17E-07	7.72E-07	1.93E-07
YLDs (Year	China	Male	55+ years	Non-melanoma skin cancer (basal-cell	Rate	1990	0.0079569	0.015503728	0.003444315
YLDs (Year	China	Female	55+ years	Non-melanoma skin cancer (basal-cell	Rate	1990	0.0080227	0.015446031	0.003364189
YLDs (Year	China	Both	55+ years	Non-melanoma skin cancer (basal-cell	Rate	1990	0.0079903	0.015470699	0.003403711
YLDs (Year	China	Male	55+ years	Non-melanoma skin cancer (basal-cell	Number	2021	135.74804	252.7658288	61.5026419
YLDs (Year	China	Female	55+ years	Non-melanoma skin cancer (basal-cell	Number	2021	115.11333	220.6261256	50.73544808
YLDs (Year	China	Both	55+ years	Non-melanoma skin cancer (basal-cell	Number	2021	250.86138	479.3169103	112.8593417
YLDs (Year	China	Male	55+ years	Non-melanoma skin cancer (basal-cell	Percent	2021	3.95E-06	6.93E-06	1.94E-06
YLDs (Year	China	Female	55+ years	Non-melanoma skin cancer (basal-cell	Percent	2021	2.74E-06	5.05E-06	1.33E-06
YLDs (Year	China	Both	55+ years	Non-melanoma skin cancer (basal-cell	Percent	2021	3.28E-06	5.86E-06	1.61E-06
YLDs (Year	China	Male	55+ years	Non-melanoma skin cancer (basal-cell	Rate	2021	0.073859	0.137527013	0.033462888
YLDs (Year	China	Female	55+ years	Non-melanoma skin cancer (basal-cell	Rate	2021	0.0589836	0.113047953	0.025996643
YLDs (Year	China	Both	55+ years	Non-melanoma skin cancer (basal-cell	Rate	2021	0.0661982	0.126483818	0.029781717
YLDs (Year	Kazakhstan	Male	55+ years	Non-melanoma skin cancer (basal-cell	Number	1990	0.3983213	0.805552551	0.166670409
YLDs (Year	Kazakhstan	Female	55+ years	Non-melanoma skin cancer (basal-cell	Number	1990	0.7657108	1.48883053	0.321828291
YLDs (Year	Kazakhstan	Both	55+ years	Non-melanoma skin cancer (basal-cell	Number	1990	1.1640321	2.234245986	0.488258451
YLDs (Year	Kazakhstan	Male	55+ years	Non-melanoma skin cancer (basal-cell	Percent	1990	2.55E-06	4.72E-06	1.13E-06
YLDs (Year	Kazakhstan	Female	55+ years	Non-melanoma skin cancer (basal-cell	Percent	1990	2.58E-06	4.80E-06	1.18E-06
YLDs (Year	Kazakhstan	Both	55+ years	Non-melanoma skin cancer (basal-cell	Percent	1990	2.57E-06	4.72E-06	1.18E-06
YLDs (Year	Kazakhstan	Male	55+ years	Non-melanoma skin cancer (basal-cell	Rate	1990	0.0505743	0.102279978	0.021161929
YLDs (Year	Kazakhstan	Female	55+ years	Non-melanoma skin cancer (basal-cell	Rate	1990	0.0587309	0.114195006	0.024684598
YLDs (Year	Kazakhstan	Both	55+ years	Non-melanoma skin cancer (basal-cell	Rate	1990	0.0556592	0.106832354	0.023346489
YLDs (Year	Kazakhstan	Male	55+ years	Non-melanoma skin cancer (basal-cell	Number	2021	0.6556369	1.274892288	0.2745984
YLDs (Year	Kazakhstan	Female	55+ years	Non-melanoma skin cancer (basal-cell	Number	2021	1.0463422	1.997527503	0.451562014
YLDs (Year	Kazakhstan	Both	55+ years	Non-melanoma skin cancer (basal-cell	Number	2021	1.7019791	3.252450774	0.736628813
YLDs (Year	Kazakhstan	Male	55+ years	Non-melanoma skin cancer (basal-cell	Percent	2021	2.52E-06	4.61E-06	1.17E-06
YLDs (Year	Kazakhstan	Female	55+ years	Non-melanoma skin cancer (basal-cell	Percent	2021	2.43E-06	4.50E-06	1.11E-06
YLDs (Year	Kazakhstan	Both	55+ years	Non-melanoma skin cancer (basal-cell	Percent	2021	2.47E-06	4.56E-06	1.13E-06
YLDs (Year	Kazakhstan	Male	55+ years	Non-melanoma skin cancer (basal-cell	Rate	2021	0.050159	0.097534721	0.021007954
YLDs (Year	Kazakhstan	Female	55+ years	Non-melanoma skin cancer (basal-cell	Rate	2021	0.0560642	0.107029744	0.024195195
YLDs (Year	Kazakhstan	Both	55+ years	Non-melanoma skin cancer (basal-cell	Rate	2021	0.0536319	0.102489568	0.023212271
YLDs (Year	Malaysia	Male	55+ years	Non-melanoma skin cancer (basal-cell	Number	1990	0.06114	0.118871311	0.026682249
YLDs (Year	Malaysia	Female	55+ years	Non-melanoma skin cancer (basal-cell	Number	1990	0.0585166	0.114066941	0.025295064
YLDs (Year	Malaysia	Both	55+ years	Non-melanoma skin cancer (basal-cell	Number	1990	0.1196566	0.233312599	0.052263908

YLDs (Year	Malaysia	Male	55+ years	Non-melanoma skin cancer (basal-cell	Percent	1990	4.33E-07	7.92E-07	2.04E-07
YLDs (Year	Malaysia	Female	55+ years	Non-melanoma skin cancer (basal-cell	Percent	1990	3.47E-07	6.50E-07	1.58E-07
YLDs (Year	Malaysia	Both	55+ years	Non-melanoma skin cancer (basal-cell	Percent	1990	3.86E-07	7.22E-07	1.77E-07
YLDs (Year	Malaysia	Male	55+ years	Non-melanoma skin cancer (basal-cell	Rate	1990	0.0086142	0.016748125	0.00375934
YLDs (Year	Malaysia	Female	55+ years	Non-melanoma skin cancer (basal-cell	Rate	1990	0.007638	0.014888751	0.003301674
YLDs (Year	Malaysia	Both	55+ years	Non-melanoma skin cancer (basal-cell	Rate	1990	0.0081074	0.015808294	0.003541186
YLDs (Year	Malaysia	Male	55+ years	Non-melanoma skin cancer (basal-cell	Number	2021	0.1494063	0.2923399	0.063199847
YLDs (Year	Malaysia	Female	55+ years	Non-melanoma skin cancer (basal-cell	Number	2021	0.1342965	0.259123064	0.055072031
YLDs (Year	Malaysia	Both	55+ years	Non-melanoma skin cancer (basal-cell	Number	2021	0.2837028	0.548612846	0.120023657
YLDs (Year	Malaysia	Male	55+ years	Non-melanoma skin cancer (basal-cell	Percent	2021	3.07E-07	5.73E-07	1.37E-07
YLDs (Year	Malaysia	Female	55+ years	Non-melanoma skin cancer (basal-cell	Percent	2021	2.43E-07	4.58E-07	1.06E-07
YLDs (Year	Malaysia	Both	55+ years	Non-melanoma skin cancer (basal-cell	Percent	2021	2.73E-07	5.08E-07	1.22E-07
YLDs (Year	Malaysia	Male	55+ years	Non-melanoma skin cancer (basal-cell	Rate	2021	0.0061577	0.012048615	0.002604744
YLDs (Year	Malaysia	Female	55+ years	Non-melanoma skin cancer (basal-cell	Rate	2021	0.0054229	0.010463473	0.002223826
YLDs (Year	Malaysia	Both	55+ years	Non-melanoma skin cancer (basal-cell	Rate	2021	0.0057866	0.011189809	0.002448069
YLDs (Year	Kiribati	Male	55+ years	Non-melanoma skin cancer (basal-cell	Number	1990	2.34E-06	6.15E-06	4.27E-07
YLDs (Year	Kiribati	Female	55+ years	Non-melanoma skin cancer (basal-cell	Number	1990	3.05E-06	8.19E-06	7.09E-07
YLDs (Year	Kiribati	Both	55+ years	Non-melanoma skin cancer (basal-cell	Number	1990	5.39E-06	1.43E-05	1.31E-06
YLDs (Year	Kiribati	Male	55+ years	Non-melanoma skin cancer (basal-cell	Percent	1990	4.22E-09	1.01E-08	8.23E-10
YLDs (Year	Kiribati	Female	55+ years	Non-melanoma skin cancer (basal-cell	Percent	1990	3.96E-09	9.62E-09	9.35E-10
YLDs (Year	Kiribati	Both	55+ years	Non-melanoma skin cancer (basal-cell	Percent	1990	4.07E-09	9.65E-09	1.05E-09
YLDs (Year	Kiribati	Male	55+ years	Non-melanoma skin cancer (basal-cell	Rate	1990	8.82E-05	0.00023185	1.61E-05
YLDs (Year	Kiribati	Female	55+ years	Non-melanoma skin cancer (basal-cell	Rate	1990	9.23E-05	0.000247723	2.14E-05
YLDs (Year	Kiribati	Both	55+ years	Non-melanoma skin cancer (basal-cell	Rate	1990	9.05E-05	0.000240014	2.20E-05
YLDs (Year	Kiribati	Male	55+ years	Non-melanoma skin cancer (basal-cell	Number	2021	4.83E-06	1.30E-05	9.83E-07
YLDs (Year	Kiribati	Female	55+ years	Non-melanoma skin cancer (basal-cell	Number	2021	6.55E-06	1.76E-05	1.54E-06
YLDs (Year	Kiribati	Both	55+ years	Non-melanoma skin cancer (basal-cell	Number	2021	1.14E-05	3.09E-05	2.77E-06
YLDs (Year	Kiribati	Male	55+ years	Non-melanoma skin cancer (basal-cell	Percent	2021	4.42E-09	1.11E-08	9.16E-10
YLDs (Year	Kiribati	Female	55+ years	Non-melanoma skin cancer (basal-cell	Percent	2021	3.98E-09	9.78E-09	9.27E-10
YLDs (Year	Kiribati	Both	55+ years	Non-melanoma skin cancer (basal-cell	Percent	2021	4.16E-09	1.03E-08	1.08E-09
YLDs (Year	Kiribati	Male	55+ years	Non-melanoma skin cancer (basal-cell	Rate	2021	8.81E-05	0.000237299	1.79E-05
YLDs (Year	Kiribati	Female	55+ years	Non-melanoma skin cancer (basal-cell	Rate	2021	9.23E-05	0.00024802	2.16E-05
YLDs (Year	Kiribati	Both	55+ years	Non-melanoma skin cancer (basal-cell	Rate	2021	9.05E-05	0.000245818	2.20E-05
YLDs (Year	Tajikistan	Male	55+ years	Non-melanoma skin cancer (basal-cell	Number	1990	0.1110848	0.21810135	0.048667467
YLDs (Year	Tajikistan	Female	55+ years	Non-melanoma skin cancer (basal-cell	Number	1990	0.1436111	0.286455312	0.061942045
YLDs (Year	Tajikistan	Both	55+ years	Non-melanoma skin cancer (basal-cell	Number	1990	0.2546959	0.495918067	0.112140239
YLDs (Year	Tajikistan	Male	55+ years	Non-melanoma skin cancer (basal-cell	Percent	1990	3.01E-06	5.39E-06	1.41E-06
YLDs (Year	Tajikistan	Female	55+ years	Non-melanoma skin cancer (basal-cell	Percent	1990	2.63E-06	4.89E-06	1.23E-06
YLDs (Year	Tajikistan	Both	55+ years	Non-melanoma skin cancer (basal-cell	Percent	1990	2.78E-06	5.16E-06	1.34E-06
YLDs (Year	Tajikistan	Male	55+ years	Non-melanoma skin cancer (basal-cell	Rate	1990	0.0540935	0.106205847	0.023698934
YLDs (Year	Tajikistan	Female	55+ years	Non-melanoma skin cancer (basal-cell	Rate	1990	0.0546135	0.10893531	0.023555771
YLDs (Year	Tajikistan	Both	55+ years	Non-melanoma skin cancer (basal-cell	Rate	1990	0.0543854	0.105893831	0.023945406
YLDs (Year	Tajikistan	Male	55+ years	Non-melanoma skin cancer (basal-cell	Number	2021	0.2561805	0.508972661	0.106183871
YLDs (Year	Tajikistan	Female	55+ years	Non-melanoma skin cancer (basal-cell	Number	2021	0.2526694	0.491912228	0.103882995
YLDs (Year	Tajikistan	Both	55+ years	Non-melanoma skin cancer (basal-cell	Number	2021	0.5088499	0.992246289	0.208954952
YLDs (Year	Tajikistan	Male	55+ years	Non-melanoma skin cancer (basal-cell	Percent	2021	2.94E-06	5.39E-06	1.36E-06
YLDs (Year	Tajikistan	Female	55+ years	Non-melanoma skin cancer (basal-cell	Percent	2021	2.32E-06	4.28E-06	1.03E-06
YLDs (Year	Tajikistan	Both	55+ years	Non-melanoma skin cancer (basal-cell	Percent	2021	2.60E-06	4.78E-06	1.16E-06
YLDs (Year	Tajikistan	Male	55+ years	Non-melanoma skin cancer (basal-cell	Rate	2021	0.0514315	0.102182786	0.021317773
YLDs (Year	Tajikistan	Female	55+ years	Non-melanoma skin cancer (basal-cell	Rate	2021	0.0477596	0.092981212	0.019635956
YLDs (Year	Tajikistan	Both	55+ years	Non-melanoma skin cancer (basal-cell	Rate	2021	0.0495402	0.096602362	0.020343278
YLDs (Year	Tonga	Male	55+ years	Non-melanoma skin cancer (basal-cell	Number	1990	5.15E-06	1.39E-05	1.18E-06
YLDs (Year	Tonga	Female	55+ years	Non-melanoma skin cancer (basal-cell	Number	1990	6.01E-06	1.61E-05	1.47E-06
YLDs (Year	Tonga	Both	55+ years	Non-melanoma skin cancer (basal-cell	Number	1990	1.12E-05	2.99E-05	2.76E-06
YLDs (Year	Tonga	Male	55+ years	Non-melanoma skin cancer (basal-cell	Percent	1990	6.09E-09	1.49E-08	1.46E-09
YLDs (Year	Tonga	Female	55+ years	Non-melanoma skin cancer (basal-cell	Percent	1990	5.86E-09	1.39E-08	1.42E-09

YLDs (Year	Tonga	Both	55+ years	Non-melanoma skin cancer (basal-cell	Percent	1990	5.97E-09	1.42E-08	1.59E-09
YLDs (Year	Tonga	Male	55+ years	Non-melanoma skin cancer (basal-cell	Rate	1990	0.0001103	0.000298109	2.53E-05
YLDs (Year	Tonga	Female	55+ years	Non-melanoma skin cancer (basal-cell	Rate	1990	0.0001247	0.000335181	3.04E-05
YLDs (Year	Tonga	Both	55+ years	Non-melanoma skin cancer (basal-cell	Rate	1990	0.0001176	0.000315167	2.90E-05
YLDs (Year	Tonga	Male	55+ years	Non-melanoma skin cancer (basal-cell	Number	2021	6.98E-06	1.81E-05	1.46E-06
YLDs (Year	Tonga	Female	55+ years	Non-melanoma skin cancer (basal-cell	Number	2021	8.89E-06	2.30E-05	2.29E-06
YLDs (Year	Tonga	Both	55+ years	Non-melanoma skin cancer (basal-cell	Number	2021	1.59E-05	4.06E-05	4.01E-06
YLDs (Year	Tonga	Male	55+ years	Non-melanoma skin cancer (basal-cell	Percent	2021	5.52E-09	1.39E-08	1.18E-09
YLDs (Year	Tonga	Female	55+ years	Non-melanoma skin cancer (basal-cell	Percent	2021	5.34E-09	1.28E-08	1.33E-09
YLDs (Year	Tonga	Both	55+ years	Non-melanoma skin cancer (basal-cell	Percent	2021	5.42E-09	1.31E-08	1.43E-09
YLDs (Year	Tonga	Male	55+ years	Non-melanoma skin cancer (basal-cell	Rate	2021	0.0001098	0.000285156	2.30E-05
YLDs (Year	Tonga	Female	55+ years	Non-melanoma skin cancer (basal-cell	Rate	2021	0.0001258	0.000324666	3.24E-05
YLDs (Year	Tonga	Both	55+ years	Non-melanoma skin cancer (basal-cell	Rate	2021	0.0001182	0.00030254	2.99E-05
YLDs (Year	Micronesia	Male	55+ years	Non-melanoma skin cancer (basal-cell	Number	1990	3.58E-06	9.46E-06	7.48E-07
YLDs (Year	Micronesia	Female	55+ years	Non-melanoma skin cancer (basal-cell	Number	1990	3.84E-06	9.90E-06	8.93E-07
YLDs (Year	Micronesia	Both	55+ years	Non-melanoma skin cancer (basal-cell	Number	1990	7.42E-06	1.92E-05	1.80E-06
YLDs (Year	Micronesia	Male	55+ years	Non-melanoma skin cancer (basal-cell	Percent	1990	4.59E-09	1.10E-08	1.01E-09
YLDs (Year	Micronesia	Female	55+ years	Non-melanoma skin cancer (basal-cell	Percent	1990	4.25E-09	1.01E-08	9.66E-10
YLDs (Year	Micronesia	Both	55+ years	Non-melanoma skin cancer (basal-cell	Percent	1990	4.41E-09	1.05E-08	1.15E-09
YLDs (Year	Micronesia	Male	55+ years	Non-melanoma skin cancer (basal-cell	Rate	1990	9.17E-05	0.000242232	1.91E-05
YLDs (Year	Micronesia	Female	55+ years	Non-melanoma skin cancer (basal-cell	Rate	1990	9.47E-05	0.00024417	2.20E-05
YLDs (Year	Micronesia	Both	55+ years	Non-melanoma skin cancer (basal-cell	Rate	1990	9.32E-05	0.000241472	2.27E-05
YLDs (Year	Micronesia	Male	55+ years	Non-melanoma skin cancer (basal-cell	Number	2021	5.74E-06	1.54E-05	1.14E-06
YLDs (Year	Micronesia	Female	55+ years	Non-melanoma skin cancer (basal-cell	Number	2021	6.41E-06	1.74E-05	1.39E-06
YLDs (Year	Micronesia	Both	55+ years	Non-melanoma skin cancer (basal-cell	Number	2021	1.22E-05	3.32E-05	2.93E-06
YLDs (Year	Micronesia	Male	55+ years	Non-melanoma skin cancer (basal-cell	Percent	2021	4.67E-09	1.17E-08	9.53E-10
YLDs (Year	Micronesia	Female	55+ years	Non-melanoma skin cancer (basal-cell	Percent	2021	4.13E-09	1.00E-08	9.47E-10
YLDs (Year	Micronesia	Both	55+ years	Non-melanoma skin cancer (basal-cell	Percent	2021	4.37E-09	1.07E-08	1.08E-09
YLDs (Year	Micronesia	Male	55+ years	Non-melanoma skin cancer (basal-cell	Rate	2021	9.08E-05	0.000243182	1.80E-05
YLDs (Year	Micronesia	Female	55+ years	Non-melanoma skin cancer (basal-cell	Rate	2021	9.37E-05	0.000254261	2.03E-05
YLDs (Year	Micronesia	Both	55+ years	Non-melanoma skin cancer (basal-cell	Rate	2021	9.23E-05	0.000252003	2.23E-05
YLDs (Year	Armenia	Male	55+ years	Non-melanoma skin cancer (basal-cell	Number	1990	0.1131838	0.222468264	0.049006565
YLDs (Year	Armenia	Female	55+ years	Non-melanoma skin cancer (basal-cell	Number	1990	0.1615085	0.321175618	0.067271539
YLDs (Year	Armenia	Both	55+ years	Non-melanoma skin cancer (basal-cell	Number	1990	0.2746923	0.554811299	0.116088608
YLDs (Year	Armenia	Male	55+ years	Non-melanoma skin cancer (basal-cell	Percent	1990	2.85E-06	5.23E-06	1.27E-06
YLDs (Year	Armenia	Female	55+ years	Non-melanoma skin cancer (basal-cell	Percent	1990	2.77E-06	5.06E-06	1.23E-06
YLDs (Year	Armenia	Both	55+ years	Non-melanoma skin cancer (basal-cell	Percent	1990	2.80E-06	5.24E-06	1.23E-06
YLDs (Year	Armenia	Male	55+ years	Non-melanoma skin cancer (basal-cell	Rate	1990	0.0536099	0.105372868	0.02321213
YLDs (Year	Armenia	Female	55+ years	Non-melanoma skin cancer (basal-cell	Rate	1990	0.0585935	0.11651891	0.024405359
YLDs (Year	Armenia	Both	55+ years	Non-melanoma skin cancer (basal-cell	Rate	1990	0.056432	0.113978755	0.023848893
YLDs (Year	Armenia	Male	55+ years	Non-melanoma skin cancer (basal-cell	Number	2021	0.2062178	0.397766633	0.081717998
YLDs (Year	Armenia	Female	55+ years	Non-melanoma skin cancer (basal-cell	Number	2021	0.2817551	0.559168942	0.117975162
YLDs (Year	Armenia	Both	55+ years	Non-melanoma skin cancer (basal-cell	Number	2021	0.4879729	0.932490397	0.198673785
YLDs (Year	Armenia	Male	55+ years	Non-melanoma skin cancer (basal-cell	Percent	2021	3.15E-06	5.62E-06	1.34E-06
YLDs (Year	Armenia	Female	55+ years	Non-melanoma skin cancer (basal-cell	Percent	2021	2.76E-06	5.28E-06	1.17E-06
YLDs (Year	Armenia	Both	55+ years	Non-melanoma skin cancer (basal-cell	Percent	2021	2.91E-06	5.23E-06	1.24E-06
YLDs (Year	Armenia	Male	55+ years	Non-melanoma skin cancer (basal-cell	Rate	2021	0.0613839	0.118401313	0.02432461
YLDs (Year	Armenia	Female	55+ years	Non-melanoma skin cancer (basal-cell	Rate	2021	0.062519	0.124074727	0.026177663
YLDs (Year	Armenia	Both	55+ years	Non-melanoma skin cancer (basal-cell	Rate	2021	0.0620342	0.118544109	0.025256675
YLDs (Year	Indonesia	Male	55+ years	Non-melanoma skin cancer (basal-cell	Number	1990	0.2124851	0.409419306	0.083662433
YLDs (Year	Indonesia	Female	55+ years	Non-melanoma skin cancer (basal-cell	Number	1990	0.1758972	0.341272177	0.068140249
YLDs (Year	Indonesia	Both	55+ years	Non-melanoma skin cancer (basal-cell	Number	1990	0.3883823	0.737909055	0.151608396
YLDs (Year	Indonesia	Male	55+ years	Non-melanoma skin cancer (basal-cell	Percent	1990	1.43E-07	2.71E-07	6.30E-08
YLDs (Year	Indonesia	Female	55+ years	Non-melanoma skin cancer (basal-cell	Percent	1990	9.97E-08	1.88E-07	4.17E-08
YLDs (Year	Indonesia	Both	55+ years	Non-melanoma skin cancer (basal-cell	Percent	1990	1.20E-07	2.30E-07	5.19E-08
YLDs (Year	Indonesia	Male	55+ years	Non-melanoma skin cancer (basal-cell	Rate	1990	0.0027427	0.005284754	0.001079908

YLDs (Year	Indonesia	Female	55+ years	Non-melanoma skin cancer (basal-cell	Rate	1990	0.0020919	0.004058646	0.000810371
YLDs (Year	Indonesia	Both	55+ years	Non-melanoma skin cancer (basal-cell	Rate	1990	0.002404	0.004567484	0.00093842
YLDs (Year	Indonesia	Male	55+ years	Non-melanoma skin cancer (basal-cell	Number	2021	0.5515502	1.070717547	0.215632331
YLDs (Year	Indonesia	Female	55+ years	Non-melanoma skin cancer (basal-cell	Number	2021	0.4456366	0.848311623	0.173814097
YLDs (Year	Indonesia	Both	55+ years	Non-melanoma skin cancer (basal-cell	Number	2021	0.9971868	1.897213866	0.388549219
YLDs (Year	Indonesia	Male	55+ years	Non-melanoma skin cancer (basal-cell	Percent	2021	1.45E-07	2.74E-07	6.25E-08
YLDs (Year	Indonesia	Female	55+ years	Non-melanoma skin cancer (basal-cell	Percent	2021	9.83E-08	1.86E-07	4.11E-08
YLDs (Year	Indonesia	Both	55+ years	Non-melanoma skin cancer (basal-cell	Percent	2021	1.19E-07	2.29E-07	5.14E-08
YLDs (Year	Indonesia	Male	55+ years	Non-melanoma skin cancer (basal-cell	Rate	2021	0.0027019	0.005245154	0.001056324
YLDs (Year	Indonesia	Female	55+ years	Non-melanoma skin cancer (basal-cell	Rate	2021	0.002076	0.003951869	0.000809715
YLDs (Year	Indonesia	Both	55+ years	Non-melanoma skin cancer (basal-cell	Rate	2021	0.0023811	0.004530168	0.000927778
YLDs (Year	Timor-Lest	Male	55+ years	Non-melanoma skin cancer (basal-cell	Number	1990	0.0005923	0.001184449	0.000224898
YLDs (Year	Timor-Lest	Female	55+ years	Non-melanoma skin cancer (basal-cell	Number	1990	0.0004015	0.000793243	0.000154359
YLDs (Year	Timor-Lest	Both	55+ years	Non-melanoma skin cancer (basal-cell	Number	1990	0.0009938	0.001952584	0.000373383
YLDs (Year	Timor-Lest	Male	55+ years	Non-melanoma skin cancer (basal-cell	Percent	1990	1.38E-07	2.64E-07	5.84E-08
YLDs (Year	Timor-Lest	Female	55+ years	Non-melanoma skin cancer (basal-cell	Percent	1990	8.97E-08	1.70E-07	3.80E-08
YLDs (Year	Timor-Lest	Both	55+ years	Non-melanoma skin cancer (basal-cell	Percent	1990	1.13E-07	2.16E-07	4.85E-08
YLDs (Year	Timor-Lest	Male	55+ years	Non-melanoma skin cancer (basal-cell	Rate	1990	0.0026721	0.005343165	0.001014535
YLDs (Year	Timor-Lest	Female	55+ years	Non-melanoma skin cancer (basal-cell	Rate	1990	0.0018855	0.003725168	0.000724887
YLDs (Year	Timor-Lest	Both	55+ years	Non-melanoma skin cancer (basal-cell	Rate	1990	0.0022867	0.004492653	0.000859107
YLDs (Year	Timor-Lest	Male	55+ years	Non-melanoma skin cancer (basal-cell	Number	2021	0.0021642	0.004251703	0.00087549
YLDs (Year	Timor-Lest	Female	55+ years	Non-melanoma skin cancer (basal-cell	Number	2021	0.0016003	0.003085304	0.000617604
YLDs (Year	Timor-Lest	Both	55+ years	Non-melanoma skin cancer (basal-cell	Number	2021	0.0037644	0.007221944	0.001565268
YLDs (Year	Timor-Lest	Male	55+ years	Non-melanoma skin cancer (basal-cell	Percent	2021	1.40E-07	2.69E-07	6.02E-08
YLDs (Year	Timor-Lest	Female	55+ years	Non-melanoma skin cancer (basal-cell	Percent	2021	9.99E-08	1.89E-07	4.16E-08
YLDs (Year	Timor-Lest	Both	55+ years	Non-melanoma skin cancer (basal-cell	Percent	2021	1.19E-07	2.35E-07	5.10E-08
YLDs (Year	Timor-Lest	Male	55+ years	Non-melanoma skin cancer (basal-cell	Rate	2021	0.0030653	0.006022017	0.001240025
YLDs (Year	Timor-Lest	Female	55+ years	Non-melanoma skin cancer (basal-cell	Rate	2021	0.0022404	0.004319433	0.000864647
YLDs (Year	Timor-Lest	Both	55+ years	Non-melanoma skin cancer (basal-cell	Rate	2021	0.0026504	0.005084762	0.00110206
YLDs (Year	Philippine	Male	55+ years	Non-melanoma skin cancer (basal-cell	Number	1990	0.1506658	0.288830148	0.06204331
YLDs (Year	Philippine	Female	55+ years	Non-melanoma skin cancer (basal-cell	Number	1990	0.1522548	0.297916306	0.063368799
YLDs (Year	Philippine	Both	55+ years	Non-melanoma skin cancer (basal-cell	Number	1990	0.3029206	0.582610304	0.125430006
YLDs (Year	Philippine	Male	55+ years	Non-melanoma skin cancer (basal-cell	Percent	1990	3.24E-07	5.92E-07	1.52E-07
YLDs (Year	Philippine	Female	55+ years	Non-melanoma skin cancer (basal-cell	Percent	1990	2.83E-07	5.17E-07	1.30E-07
YLDs (Year	Philippine	Both	55+ years	Non-melanoma skin cancer (basal-cell	Percent	1990	3.02E-07	5.48E-07	1.40E-07
YLDs (Year	Philippine	Male	55+ years	Non-melanoma skin cancer (basal-cell	Rate	1990	0.0065817	0.012617318	0.002710313
YLDs (Year	Philippine	Female	55+ years	Non-melanoma skin cancer (basal-cell	Rate	1990	0.0061273	0.011989238	0.002550191
YLDs (Year	Philippine	Both	55+ years	Non-melanoma skin cancer (basal-cell	Rate	1990	0.0063452	0.012203765	0.002627345
YLDs (Year	Philippine	Male	55+ years	Non-melanoma skin cancer (basal-cell	Number	2021	0.2838243	0.556355894	0.113656962
YLDs (Year	Philippine	Female	55+ years	Non-melanoma skin cancer (basal-cell	Number	2021	0.3186702	0.616703372	0.129695468
YLDs (Year	Philippine	Both	55+ years	Non-melanoma skin cancer (basal-cell	Number	2021	0.6024945	1.15712793	0.24507655
YLDs (Year	Philippine	Male	55+ years	Non-melanoma skin cancer (basal-cell	Percent	2021	2.26E-07	4.20E-07	1.01E-07
YLDs (Year	Philippine	Female	55+ years	Non-melanoma skin cancer (basal-cell	Percent	2021	1.92E-07	3.55E-07	8.54E-08
YLDs (Year	Philippine	Both	55+ years	Non-melanoma skin cancer (basal-cell	Percent	2021	2.07E-07	3.82E-07	9.21E-08
YLDs (Year	Philippine	Male	55+ years	Non-melanoma skin cancer (basal-cell	Rate	2021	0.0044064	0.008637548	0.001764549
YLDs (Year	Philippine	Female	55+ years	Non-melanoma skin cancer (basal-cell	Rate	2021	0.0042467	0.008218395	0.001728365
YLDs (Year	Philippine	Both	55+ years	Non-melanoma skin cancer (basal-cell	Rate	2021	0.0043205	0.008297755	0.001757442
YLDs (Year	Fiji	Male	55+ years	Non-melanoma skin cancer (basal-cell	Number	1990	2.72E-05	7.19E-05	5.37E-06
YLDs (Year	Fiji	Female	55+ years	Non-melanoma skin cancer (basal-cell	Number	1990	2.87E-05	7.64E-05	6.82E-06
YLDs (Year	Fiji	Both	55+ years	Non-melanoma skin cancer (basal-cell	Number	1990	5.60E-05	0.000149934	1.36E-05
YLDs (Year	Fiji	Male	55+ years	Non-melanoma skin cancer (basal-cell	Percent	1990	5.00E-09	1.24E-08	1.02E-09
YLDs (Year	Fiji	Female	55+ years	Non-melanoma skin cancer (basal-cell	Percent	1990	4.67E-09	1.15E-08	1.09E-09
YLDs (Year	Fiji	Both	55+ years	Non-melanoma skin cancer (basal-cell	Percent	1990	4.82E-09	1.18E-08	1.27E-09
YLDs (Year	Fiji	Male	55+ years	Non-melanoma skin cancer (basal-cell	Rate	1990	9.71E-05	0.000256368	1.91E-05
YLDs (Year	Fiji	Female	55+ years	Non-melanoma skin cancer (basal-cell	Rate	1990	0.0001015	0.000269614	2.41E-05
YLDs (Year	Fiji	Both	55+ years	Non-melanoma skin cancer (basal-cell	Rate	1990	9.93E-05	0.000266006	2.41E-05

YLDs (Year	Fiji	Male	55+ years	Non-melanoma skin cancer (basal-cell	Number	2021	6.21E-05	0.000167131	1.25E-05
YLDs (Year	Fiji	Female	55+ years	Non-melanoma skin cancer (basal-cell	Number	2021	7.26E-05	0.000190717	1.69E-05
YLDs (Year	Fiji	Both	55+ years	Non-melanoma skin cancer (basal-cell	Number	2021	0.0001346	0.000358539	3.25E-05
YLDs (Year	Fiji	Male	55+ years	Non-melanoma skin cancer (basal-cell	Percent	2021	4.53E-09	1.12E-08	9.28E-10
YLDs (Year	Fiji	Female	55+ years	Non-melanoma skin cancer (basal-cell	Percent	2021	4.29E-09	1.03E-08	9.61E-10
YLDs (Year	Fiji	Both	55+ years	Non-melanoma skin cancer (basal-cell	Percent	2021	4.39E-09	1.07E-08	1.14E-09
YLDs (Year	Fiji	Male	55+ years	Non-melanoma skin cancer (basal-cell	Rate	2021	9.59E-05	0.000258057	1.92E-05
YLDs (Year	Fiji	Female	55+ years	Non-melanoma skin cancer (basal-cell	Rate	2021	0.0001008	0.000264975	2.35E-05
YLDs (Year	Fiji	Both	55+ years	Non-melanoma skin cancer (basal-cell	Rate	2021	9.85E-05	0.000262204	2.38E-05
YLDs (Year	Georgia	Male	55+ years	Non-melanoma skin cancer (basal-cell	Number	1990	0.240471	0.454048412	0.098945332
YLDs (Year	Georgia	Female	55+ years	Non-melanoma skin cancer (basal-cell	Number	1990	0.4173443	0.829582629	0.171961092
YLDs (Year	Georgia	Both	55+ years	Non-melanoma skin cancer (basal-cell	Number	1990	0.6578154	1.285153172	0.264016364
YLDs (Year	Georgia	Male	55+ years	Non-melanoma skin cancer (basal-cell	Percent	1990	2.94E-06	5.15E-06	1.33E-06
YLDs (Year	Georgia	Female	55+ years	Non-melanoma skin cancer (basal-cell	Percent	1990	2.96E-06	5.60E-06	1.31E-06
YLDs (Year	Georgia	Both	55+ years	Non-melanoma skin cancer (basal-cell	Percent	1990	2.95E-06	5.37E-06	1.34E-06
YLDs (Year	Georgia	Male	55+ years	Non-melanoma skin cancer (basal-cell	Rate	1990	0.0544573	0.10282421	0.022407249
YLDs (Year	Georgia	Female	55+ years	Non-melanoma skin cancer (basal-cell	Rate	1990	0.0623675	0.123971932	0.02569768
YLDs (Year	Georgia	Both	55+ years	Non-melanoma skin cancer (basal-cell	Rate	1990	0.0592228	0.115701695	0.023769261
YLDs (Year	Georgia	Male	55+ years	Non-melanoma skin cancer (basal-cell	Number	2021	0.253354	0.496366648	0.10344454
YLDs (Year	Georgia	Female	55+ years	Non-melanoma skin cancer (basal-cell	Number	2021	0.4001837	0.815748087	0.164903555
YLDs (Year	Georgia	Both	55+ years	Non-melanoma skin cancer (basal-cell	Number	2021	0.6535377	1.271436648	0.275589439
YLDs (Year	Georgia	Male	55+ years	Non-melanoma skin cancer (basal-cell	Percent	2021	2.83E-06	5.18E-06	1.27E-06
YLDs (Year	Georgia	Female	55+ years	Non-melanoma skin cancer (basal-cell	Percent	2021	2.74E-06	5.16E-06	1.21E-06
YLDs (Year	Georgia	Both	55+ years	Non-melanoma skin cancer (basal-cell	Percent	2021	2.77E-06	5.11E-06	1.23E-06
YLDs (Year	Georgia	Male	55+ years	Non-melanoma skin cancer (basal-cell	Rate	2021	0.0594885	0.116548809	0.024289178
YLDs (Year	Georgia	Female	55+ years	Non-melanoma skin cancer (basal-cell	Rate	2021	0.0641339	0.130732804	0.026427649
YLDs (Year	Georgia	Both	55+ years	Non-melanoma skin cancer (basal-cell	Rate	2021	0.0622495	0.121104367	0.0262499
YLDs (Year	Vanuatu	Male	55+ years	Non-melanoma skin cancer (basal-cell	Number	1990	5.10E-06	1.40E-05	1.01E-06
YLDs (Year	Vanuatu	Female	55+ years	Non-melanoma skin cancer (basal-cell	Number	1990	4.57E-06	1.20E-05	1.03E-06
YLDs (Year	Vanuatu	Both	55+ years	Non-melanoma skin cancer (basal-cell	Number	1990	9.67E-06	2.60E-05	2.15E-06
YLDs (Year	Vanuatu	Male	55+ years	Non-melanoma skin cancer (basal-cell	Percent	1990	4.93E-09	1.21E-08	1.03E-09
YLDs (Year	Vanuatu	Female	55+ years	Non-melanoma skin cancer (basal-cell	Percent	1990	4.94E-09	1.22E-08	1.16E-09
YLDs (Year	Vanuatu	Both	55+ years	Non-melanoma skin cancer (basal-cell	Percent	1990	4.94E-09	1.20E-08	1.15E-09
YLDs (Year	Vanuatu	Male	55+ years	Non-melanoma skin cancer (basal-cell	Rate	1990	9.32E-05	0.000256225	1.85E-05
YLDs (Year	Vanuatu	Female	55+ years	Non-melanoma skin cancer (basal-cell	Rate	1990	0.0001017	0.000266858	2.29E-05
YLDs (Year	Vanuatu	Both	55+ years	Non-melanoma skin cancer (basal-cell	Rate	1990	9.70E-05	0.000261031	2.16E-05
YLDs (Year	Vanuatu	Male	55+ years	Non-melanoma skin cancer (basal-cell	Number	2021	1.31E-05	3.55E-05	2.62E-06
YLDs (Year	Vanuatu	Female	55+ years	Non-melanoma skin cancer (basal-cell	Number	2021	1.44E-05	3.76E-05	3.23E-06
YLDs (Year	Vanuatu	Both	55+ years	Non-melanoma skin cancer (basal-cell	Number	2021	2.75E-05	7.43E-05	6.67E-06
YLDs (Year	Vanuatu	Male	55+ years	Non-melanoma skin cancer (basal-cell	Percent	2021	4.85E-09	1.19E-08	1.01E-09
YLDs (Year	Vanuatu	Female	55+ years	Non-melanoma skin cancer (basal-cell	Percent	2021	4.51E-09	1.10E-08	1.04E-09
YLDs (Year	Vanuatu	Both	55+ years	Non-melanoma skin cancer (basal-cell	Percent	2021	4.67E-09	1.14E-08	1.18E-09
YLDs (Year	Vanuatu	Male	55+ years	Non-melanoma skin cancer (basal-cell	Rate	2021	9.30E-05	0.000251739	1.86E-05
YLDs (Year	Vanuatu	Female	55+ years	Non-melanoma skin cancer (basal-cell	Rate	2021	9.86E-05	0.000257674	2.22E-05
YLDs (Year	Vanuatu	Both	55+ years	Non-melanoma skin cancer (basal-cell	Rate	2021	9.58E-05	0.000259061	2.33E-05
YLDs (Year	Cambodia	Male	55+ years	Non-melanoma skin cancer (basal-cell	Number	1990	0.0079185	0.015677571	0.003195844
YLDs (Year	Cambodia	Female	55+ years	Non-melanoma skin cancer (basal-cell	Number	1990	0.0084344	0.016133373	0.003327497
YLDs (Year	Cambodia	Both	55+ years	Non-melanoma skin cancer (basal-cell	Number	1990	0.0163529	0.031349021	0.006561699
YLDs (Year	Cambodia	Male	55+ years	Non-melanoma skin cancer (basal-cell	Percent	1990	1.26E-07	2.28E-07	5.41E-08
YLDs (Year	Cambodia	Female	55+ years	Non-melanoma skin cancer (basal-cell	Percent	1990	8.98E-08	1.71E-07	3.71E-08
YLDs (Year	Cambodia	Both	55+ years	Non-melanoma skin cancer (basal-cell	Percent	1990	1.04E-07	1.95E-07	4.44E-08
YLDs (Year	Cambodia	Male	55+ years	Non-melanoma skin cancer (basal-cell	Rate	1990	0.0025005	0.004950734	0.001009198
YLDs (Year	Cambodia	Female	55+ years	Non-melanoma skin cancer (basal-cell	Rate	1990	0.0019881	0.003802799	0.000784324
YLDs (Year	Cambodia	Both	55+ years	Non-melanoma skin cancer (basal-cell	Rate	1990	0.0022071	0.004231085	0.000885613
YLDs (Year	Cambodia	Male	55+ years	Non-melanoma skin cancer (basal-cell	Number	2021	0.0220782	0.043730405	0.008612261
YLDs (Year	Cambodia	Female	55+ years	Non-melanoma skin cancer (basal-cell	Number	2021	0.0253969	0.047368731	0.010023081

YLDs (Year	Cambodia	Both	55+ years	Non-melanoma skin cancer (basal-cell	Number	2021	0.0474751	0.092297315	0.01886909
YLDs (Year	Cambodia	Male	55+ years	Non-melanoma skin cancer (basal-cell	Percent	2021	1.20E-07	2.32E-07	4.95E-08
YLDs (Year	Cambodia	Female	55+ years	Non-melanoma skin cancer (basal-cell	Percent	2021	9.16E-08	1.72E-07	3.84E-08
YLDs (Year	Cambodia	Both	55+ years	Non-melanoma skin cancer (basal-cell	Percent	2021	1.03E-07	1.96E-07	4.30E-08
YLDs (Year	Cambodia	Male	55+ years	Non-melanoma skin cancer (basal-cell	Rate	2021	0.0024222	0.004797641	0.000944847
YLDs (Year	Cambodia	Female	55+ years	Non-melanoma skin cancer (basal-cell	Rate	2021	0.0020119	0.003752535	0.000794025
YLDs (Year	Cambodia	Both	55+ years	Non-melanoma skin cancer (basal-cell	Rate	2021	0.002184	0.004245876	0.000868019
YLDs (Year	Azerbaijan	Male	55+ years	Non-melanoma skin cancer (basal-cell	Number	1990	0.1764672	0.348443594	0.075133635
YLDs (Year	Azerbaijan	Female	55+ years	Non-melanoma skin cancer (basal-cell	Number	1990	0.2826518	0.541405703	0.113697677
YLDs (Year	Azerbaijan	Both	55+ years	Non-melanoma skin cancer (basal-cell	Number	1990	0.459119	0.88019963	0.192811436
YLDs (Year	Azerbaijan	Male	55+ years	Non-melanoma skin cancer (basal-cell	Percent	1990	2.84E-06	5.12E-06	1.31E-06
YLDs (Year	Azerbaijan	Female	55+ years	Non-melanoma skin cancer (basal-cell	Percent	1990	2.71E-06	4.93E-06	1.25E-06
YLDs (Year	Azerbaijan	Both	55+ years	Non-melanoma skin cancer (basal-cell	Percent	1990	2.76E-06	5.12E-06	1.26E-06
YLDs (Year	Azerbaijan	Male	55+ years	Non-melanoma skin cancer (basal-cell	Rate	1990	0.0487246	0.096209295	0.020745263
YLDs (Year	Azerbaijan	Female	55+ years	Non-melanoma skin cancer (basal-cell	Rate	1990	0.0554186	0.106151697	0.022292342
YLDs (Year	Azerbaijan	Both	55+ years	Non-melanoma skin cancer (basal-cell	Rate	1990	0.052639	0.100916869	0.022106265
YLDs (Year	Azerbaijan	Male	55+ years	Non-melanoma skin cancer (basal-cell	Number	2021	0.4234063	0.833875385	0.177296777
YLDs (Year	Azerbaijan	Female	55+ years	Non-melanoma skin cancer (basal-cell	Number	2021	0.5283189	1.030962311	0.222828461
YLDs (Year	Azerbaijan	Both	55+ years	Non-melanoma skin cancer (basal-cell	Number	2021	0.9517252	1.83623303	0.401790029
YLDs (Year	Azerbaijan	Male	55+ years	Non-melanoma skin cancer (basal-cell	Percent	2021	2.83E-06	5.10E-06	1.30E-06
YLDs (Year	Azerbaijan	Female	55+ years	Non-melanoma skin cancer (basal-cell	Percent	2021	2.37E-06	4.41E-06	1.07E-06
YLDs (Year	Azerbaijan	Both	55+ years	Non-melanoma skin cancer (basal-cell	Percent	2021	2.56E-06	4.71E-06	1.19E-06
YLDs (Year	Azerbaijan	Male	55+ years	Non-melanoma skin cancer (basal-cell	Rate	2021	0.0492635	0.097021842	0.020628574
YLDs (Year	Azerbaijan	Female	55+ years	Non-melanoma skin cancer (basal-cell	Rate	2021	0.0500147	0.097598754	0.021094641
YLDs (Year	Azerbaijan	Both	55+ years	Non-melanoma skin cancer (basal-cell	Rate	2021	0.0496777	0.095846845	0.020972451
YLDs (Year	Samoa	Male	55+ years	Non-melanoma skin cancer (basal-cell	Number	1990	9.53E-06	2.29E-05	2.40E-06
YLDs (Year	Samoa	Female	55+ years	Non-melanoma skin cancer (basal-cell	Number	1990	9.80E-06	2.41E-05	2.94E-06
YLDs (Year	Samoa	Both	55+ years	Non-melanoma skin cancer (basal-cell	Number	1990	1.93E-05	4.62E-05	5.60E-06
YLDs (Year	Samoa	Male	55+ years	Non-melanoma skin cancer (basal-cell	Percent	1990	6.75E-09	1.60E-08	1.82E-09
YLDs (Year	Samoa	Female	55+ years	Non-melanoma skin cancer (basal-cell	Percent	1990	6.19E-09	1.41E-08	1.89E-09
YLDs (Year	Samoa	Both	55+ years	Non-melanoma skin cancer (basal-cell	Percent	1990	6.45E-09	1.45E-08	1.94E-09
YLDs (Year	Samoa	Male	55+ years	Non-melanoma skin cancer (basal-cell	Rate	1990	0.0001328	0.000319213	3.34E-05
YLDs (Year	Samoa	Female	55+ years	Non-melanoma skin cancer (basal-cell	Rate	1990	0.0001354	0.000333752	4.07E-05
YLDs (Year	Samoa	Both	55+ years	Non-melanoma skin cancer (basal-cell	Rate	1990	0.0001341	0.000320596	3.89E-05
YLDs (Year	Samoa	Male	55+ years	Non-melanoma skin cancer (basal-cell	Number	2021	1.29E-05	3.38E-05	2.77E-06
YLDs (Year	Samoa	Female	55+ years	Non-melanoma skin cancer (basal-cell	Number	2021	1.42E-05	3.63E-05	3.33E-06
YLDs (Year	Samoa	Both	55+ years	Non-melanoma skin cancer (basal-cell	Number	2021	2.71E-05	6.87E-05	6.62E-06
YLDs (Year	Samoa	Male	55+ years	Non-melanoma skin cancer (basal-cell	Percent	2021	5.15E-09	1.27E-08	1.13E-09
YLDs (Year	Samoa	Female	55+ years	Non-melanoma skin cancer (basal-cell	Percent	2021	4.89E-09	1.20E-08	1.15E-09
YLDs (Year	Samoa	Both	55+ years	Non-melanoma skin cancer (basal-cell	Percent	2021	5.01E-09	1.24E-08	1.31E-09
YLDs (Year	Samoa	Male	55+ years	Non-melanoma skin cancer (basal-cell	Rate	2021	0.0001055	0.000276369	2.27E-05
YLDs (Year	Samoa	Female	55+ years	Non-melanoma skin cancer (basal-cell	Rate	2021	0.0001146	0.000292885	2.69E-05
YLDs (Year	Samoa	Both	55+ years	Non-melanoma skin cancer (basal-cell	Rate	2021	0.0001101	0.000278888	2.69E-05
YLDs (Year	Kyrgyzstan	Male	55+ years	Non-melanoma skin cancer (basal-cell	Number	1990	0.1155894	0.231017341	0.050348647
YLDs (Year	Kyrgyzstan	Female	55+ years	Non-melanoma skin cancer (basal-cell	Number	1990	0.2008427	0.413324231	0.08359676
YLDs (Year	Kyrgyzstan	Both	55+ years	Non-melanoma skin cancer (basal-cell	Number	1990	0.3164321	0.631466982	0.13984613
YLDs (Year	Kyrgyzstan	Male	55+ years	Non-melanoma skin cancer (basal-cell	Percent	1990	2.96E-06	5.42E-06	1.42E-06
YLDs (Year	Kyrgyzstan	Female	55+ years	Non-melanoma skin cancer (basal-cell	Percent	1990	2.95E-06	5.57E-06	1.34E-06
YLDs (Year	Kyrgyzstan	Both	55+ years	Non-melanoma skin cancer (basal-cell	Percent	1990	2.95E-06	5.58E-06	1.45E-06
YLDs (Year	Kyrgyzstan	Male	55+ years	Non-melanoma skin cancer (basal-cell	Rate	1990	0.0568127	0.113546119	0.024746599
YLDs (Year	Kyrgyzstan	Female	55+ years	Non-melanoma skin cancer (basal-cell	Rate	1990	0.0644843	0.132705444	0.026840297
YLDs (Year	Kyrgyzstan	Both	55+ years	Non-melanoma skin cancer (basal-cell	Rate	1990	0.0614531	0.122634777	0.02715898
YLDs (Year	Kyrgyzstan	Male	55+ years	Non-melanoma skin cancer (basal-cell	Number	2021	0.1875533	0.366284563	0.07936877
YLDs (Year	Kyrgyzstan	Female	55+ years	Non-melanoma skin cancer (basal-cell	Number	2021	0.2617633	0.498974913	0.110843723
YLDs (Year	Kyrgyzstan	Both	55+ years	Non-melanoma skin cancer (basal-cell	Number	2021	0.4493166	0.86480782	0.189041843
YLDs (Year	Kyrgyzstan	Male	55+ years	Non-melanoma skin cancer (basal-cell	Percent	2021	2.84E-06	5.22E-06	1.28E-06

YLDs (Year	Kyrgyzstan	Female	55+ years	Non-melanoma skin cancer (basal-cell	Percent	2021	2.58E-06	4.70E-06	1.17E-06
YLDs (Year	Kyrgyzstan	Both	55+ years	Non-melanoma skin cancer (basal-cell	Percent	2021	2.68E-06	4.91E-06	1.20E-06
YLDs (Year	Kyrgyzstan	Male	55+ years	Non-melanoma skin cancer (basal-cell	Rate	2021	0.0513487	0.100282091	0.021729734
YLDs (Year	Kyrgyzstan	Female	55+ years	Non-melanoma skin cancer (basal-cell	Rate	2021	0.0544777	0.103845763	0.023068597
YLDs (Year	Kyrgyzstan	Both	55+ years	Non-melanoma skin cancer (basal-cell	Rate	2021	0.0531264	0.102253317	0.022351967
YLDs (Year	Thailand	Male	55+ years	Non-melanoma skin cancer (basal-cell	Number	1990	0.4284361	0.83974947	0.193990797
YLDs (Year	Thailand	Female	55+ years	Non-melanoma skin cancer (basal-cell	Number	1990	0.4929241	0.995119052	0.221871524
YLDs (Year	Thailand	Both	55+ years	Non-melanoma skin cancer (basal-cell	Number	1990	0.9213602	1.824359024	0.418109044
YLDs (Year	Thailand	Male	55+ years	Non-melanoma skin cancer (basal-cell	Percent	1990	7.85E-07	1.41E-06	3.74E-07
YLDs (Year	Thailand	Female	55+ years	Non-melanoma skin cancer (basal-cell	Percent	1990	7.39E-07	1.34E-06	3.53E-07
YLDs (Year	Thailand	Both	55+ years	Non-melanoma skin cancer (basal-cell	Percent	1990	7.60E-07	1.34E-06	3.68E-07
YLDs (Year	Thailand	Male	55+ years	Non-melanoma skin cancer (basal-cell	Rate	1990	0.0154827	0.030346704	0.007010402
YLDs (Year	Thailand	Female	55+ years	Non-melanoma skin cancer (basal-cell	Rate	1990	0.0155639	0.031420535	0.007005515
YLDs (Year	Thailand	Both	55+ years	Non-melanoma skin cancer (basal-cell	Rate	1990	0.0155261	0.030742704	0.007045654
YLDs (Year	Thailand	Male	55+ years	Non-melanoma skin cancer (basal-cell	Number	2021	0.6731042	1.250562047	0.27443536
YLDs (Year	Thailand	Female	55+ years	Non-melanoma skin cancer (basal-cell	Number	2021	0.7614213	1.494736827	0.306541261
YLDs (Year	Thailand	Both	55+ years	Non-melanoma skin cancer (basal-cell	Number	2021	1.4345255	2.739921655	0.588789251
YLDs (Year	Thailand	Male	55+ years	Non-melanoma skin cancer (basal-cell	Percent	2021	3.81E-07	6.81E-07	1.72E-07
YLDs (Year	Thailand	Female	55+ years	Non-melanoma skin cancer (basal-cell	Percent	2021	3.35E-07	6.17E-07	1.47E-07
YLDs (Year	Thailand	Both	55+ years	Non-melanoma skin cancer (basal-cell	Percent	2021	3.55E-07	6.48E-07	1.62E-07
YLDs (Year	Thailand	Male	55+ years	Non-melanoma skin cancer (basal-cell	Rate	2021	0.0075644	0.014053922	0.003084128
YLDs (Year	Thailand	Female	55+ years	Non-melanoma skin cancer (basal-cell	Rate	2021	0.0072243	0.014182004	0.002908451
YLDs (Year	Thailand	Both	55+ years	Non-melanoma skin cancer (basal-cell	Rate	2021	0.00738	0.014095707	0.003029065
YLDs (Year	Bulgaria	Male	55+ years	Non-melanoma skin cancer (basal-cell	Number	1990	0.8745545	1.677618674	0.35487113
YLDs (Year	Bulgaria	Female	55+ years	Non-melanoma skin cancer (basal-cell	Number	1990	0.8736967	1.815682994	0.35646079
YLDs (Year	Bulgaria	Both	55+ years	Non-melanoma skin cancer (basal-cell	Number	1990	1.7482512	3.454321597	0.718224698
YLDs (Year	Bulgaria	Male	55+ years	Non-melanoma skin cancer (basal-cell	Percent	1990	3.91E-06	6.96E-06	1.71E-06
YLDs (Year	Bulgaria	Female	55+ years	Non-melanoma skin cancer (basal-cell	Percent	1990	3.29E-06	6.43E-06	1.42E-06
YLDs (Year	Bulgaria	Both	55+ years	Non-melanoma skin cancer (basal-cell	Percent	1990	3.57E-06	6.51E-06	1.62E-06
YLDs (Year	Bulgaria	Male	55+ years	Non-melanoma skin cancer (basal-cell	Rate	1990	0.0834766	0.160129426	0.033872602
YLDs (Year	Bulgaria	Female	55+ years	Non-melanoma skin cancer (basal-cell	Rate	1990	0.0718722	0.149362027	0.029323239
YLDs (Year	Bulgaria	Both	55+ years	Non-melanoma skin cancer (basal-cell	Rate	1990	0.0772438	0.152623921	0.031733661
YLDs (Year	Bulgaria	Male	55+ years	Non-melanoma skin cancer (basal-cell	Number	2021	1.2089982	2.549369952	0.473505365
YLDs (Year	Bulgaria	Female	55+ years	Non-melanoma skin cancer (basal-cell	Number	2021	1.5590898	3.130306381	0.679203487
YLDs (Year	Bulgaria	Both	55+ years	Non-melanoma skin cancer (basal-cell	Number	2021	2.768088	5.559578676	1.243229638
YLDs (Year	Bulgaria	Male	55+ years	Non-melanoma skin cancer (basal-cell	Percent	2021	5.35E-06	1.05E-05	2.26E-06
YLDs (Year	Bulgaria	Female	55+ years	Non-melanoma skin cancer (basal-cell	Percent	2021	4.77E-06	9.50E-06	2.21E-06
YLDs (Year	Bulgaria	Both	55+ years	Non-melanoma skin cancer (basal-cell	Percent	2021	5.01E-06	9.57E-06	2.35E-06
YLDs (Year	Bulgaria	Male	55+ years	Non-melanoma skin cancer (basal-cell	Rate	2021	0.1183465	0.24955295	0.046350535
YLDs (Year	Bulgaria	Female	55+ years	Non-melanoma skin cancer (basal-cell	Rate	2021	0.1150337	0.230962164	0.050113404
YLDs (Year	Bulgaria	Both	55+ years	Non-melanoma skin cancer (basal-cell	Rate	2021	0.1164575	0.233899641	0.052304497
YLDs (Year	Papua New	Male	55+ years	Non-melanoma skin cancer (basal-cell	Number	1990	0.0001637	0.000433654	3.46E-05
YLDs (Year	Papua New	Female	55+ years	Non-melanoma skin cancer (basal-cell	Number	1990	0.0001466	0.000392222	3.13E-05
YLDs (Year	Papua New	Both	55+ years	Non-melanoma skin cancer (basal-cell	Number	1990	0.0003104	0.000821144	7.39E-05
YLDs (Year	Papua New	Male	55+ years	Non-melanoma skin cancer (basal-cell	Percent	1990	5.30E-09	1.34E-08	1.11E-09
YLDs (Year	Papua New	Female	55+ years	Non-melanoma skin cancer (basal-cell	Percent	1990	4.88E-09	1.19E-08	1.06E-09
YLDs (Year	Papua New	Both	55+ years	Non-melanoma skin cancer (basal-cell	Percent	1990	5.09E-09	1.26E-08	1.29E-09
YLDs (Year	Papua New	Male	55+ years	Non-melanoma skin cancer (basal-cell	Rate	1990	0.0001056	0.000279773	2.23E-05
YLDs (Year	Papua New	Female	55+ years	Non-melanoma skin cancer (basal-cell	Rate	1990	0.0001041	0.000278423	2.22E-05
YLDs (Year	Papua New	Both	55+ years	Non-melanoma skin cancer (basal-cell	Rate	1990	0.0001049	0.000277531	2.50E-05
YLDs (Year	Papua New	Male	55+ years	Non-melanoma skin cancer (basal-cell	Number	2021	0.0004678	0.001262374	9.37E-05
YLDs (Year	Papua New	Female	55+ years	Non-melanoma skin cancer (basal-cell	Number	2021	0.0003962	0.00108265	9.08E-05
YLDs (Year	Papua New	Both	55+ years	Non-melanoma skin cancer (basal-cell	Number	2021	0.0008639	0.002297642	0.000220644
YLDs (Year	Papua New	Male	55+ years	Non-melanoma skin cancer (basal-cell	Percent	2021	5.19E-09	1.28E-08	1.11E-09
YLDs (Year	Papua New	Female	55+ years	Non-melanoma skin cancer (basal-cell	Percent	2021	4.80E-09	1.21E-08	1.17E-09
YLDs (Year	Papua New	Both	55+ years	Non-melanoma skin cancer (basal-cell	Percent	2021	5.01E-09	1.24E-08	1.32E-09

YLDs (Year	Papua New	Male	55+ years	Non-melanoma skin cancer (basal-cell	Rate	2021	0.0001052	0.000283961	2.11E-05
YLDs (Year	Papua New	Female	55+ years	Non-melanoma skin cancer (basal-cell	Rate	2021	0.0001052	0.00028737	2.41E-05
YLDs (Year	Papua New	Both	55+ years	Non-melanoma skin cancer (basal-cell	Rate	2021	0.0001052	0.000279756	2.69E-05
YLDs (Year	Marshall I	Male	55+ years	Non-melanoma skin cancer (basal-cell	Number	1990	1.16E-06	3.17E-06	2.37E-07
YLDs (Year	Marshall I	Female	55+ years	Non-melanoma skin cancer (basal-cell	Number	1990	1.22E-06	3.16E-06	2.84E-07
YLDs (Year	Marshall I	Both	55+ years	Non-melanoma skin cancer (basal-cell	Number	1990	2.38E-06	6.28E-06	5.96E-07
YLDs (Year	Marshall I	Male	55+ years	Non-melanoma skin cancer (basal-cell	Percent	1990	4.69E-09	1.14E-08	1.03E-09
YLDs (Year	Marshall I	Female	55+ years	Non-melanoma skin cancer (basal-cell	Percent	1990	4.15E-09	9.83E-09	9.78E-10
YLDs (Year	Marshall I	Both	55+ years	Non-melanoma skin cancer (basal-cell	Percent	1990	4.39E-09	1.05E-08	1.15E-09
YLDs (Year	Marshall I	Male	55+ years	Non-melanoma skin cancer (basal-cell	Rate	1990	9.37E-05	0.000255194	1.91E-05
YLDs (Year	Marshall I	Female	55+ years	Non-melanoma skin cancer (basal-cell	Rate	1990	9.31E-05	0.000241594	2.17E-05
YLDs (Year	Marshall I	Both	55+ years	Non-melanoma skin cancer (basal-cell	Rate	1990	9.34E-05	0.00024635	2.34E-05
YLDs (Year	Marshall I	Male	55+ years	Non-melanoma skin cancer (basal-cell	Number	2021	2.71E-06	7.11E-06	5.47E-07
YLDs (Year	Marshall I	Female	55+ years	Non-melanoma skin cancer (basal-cell	Number	2021	2.71E-06	7.35E-06	5.97E-07
YLDs (Year	Marshall I	Both	55+ years	Non-melanoma skin cancer (basal-cell	Number	2021	5.42E-06	1.46E-05	1.30E-06
YLDs (Year	Marshall I	Male	55+ years	Non-melanoma skin cancer (basal-cell	Percent	2021	4.39E-09	1.09E-08	8.95E-10
YLDs (Year	Marshall I	Female	55+ years	Non-melanoma skin cancer (basal-cell	Percent	2021	3.97E-09	9.88E-09	8.86E-10
YLDs (Year	Marshall I	Both	55+ years	Non-melanoma skin cancer (basal-cell	Percent	2021	4.17E-09	1.01E-08	1.08E-09
YLDs (Year	Marshall I	Male	55+ years	Non-melanoma skin cancer (basal-cell	Rate	2021	9.22E-05	0.000241896	1.86E-05
YLDs (Year	Marshall I	Female	55+ years	Non-melanoma skin cancer (basal-cell	Rate	2021	9.30E-05	0.000251748	2.05E-05
YLDs (Year	Marshall I	Both	55+ years	Non-melanoma skin cancer (basal-cell	Rate	2021	9.26E-05	0.000248851	2.22E-05
YLDs (Year	Viet Nam	Male	55+ years	Non-melanoma skin cancer (basal-cell	Number	1990	0.1802393	0.357746332	0.078366257
YLDs (Year	Viet Nam	Female	55+ years	Non-melanoma skin cancer (basal-cell	Number	1990	0.291148	0.588179848	0.13071693
YLDs (Year	Viet Nam	Both	55+ years	Non-melanoma skin cancer (basal-cell	Number	1990	0.4713874	0.945317024	0.208033645
YLDs (Year	Viet Nam	Male	55+ years	Non-melanoma skin cancer (basal-cell	Percent	1990	3.32E-07	6.03E-07	1.56E-07
YLDs (Year	Viet Nam	Female	55+ years	Non-melanoma skin cancer (basal-cell	Percent	1990	3.54E-07	6.38E-07	1.66E-07
YLDs (Year	Viet Nam	Both	55+ years	Non-melanoma skin cancer (basal-cell	Percent	1990	3.45E-07	6.25E-07	1.64E-07
YLDs (Year	Viet Nam	Male	55+ years	Non-melanoma skin cancer (basal-cell	Rate	1990	0.006073	0.012053987	0.002640491
YLDs (Year	Viet Nam	Female	55+ years	Non-melanoma skin cancer (basal-cell	Rate	1990	0.007271	0.014688985	0.003264476
YLDs (Year	Viet Nam	Both	55+ years	Non-melanoma skin cancer (basal-cell	Rate	1990	0.0067611	0.013558586	0.002983806
YLDs (Year	Viet Nam	Male	55+ years	Non-melanoma skin cancer (basal-cell	Number	2021	0.257618	0.514273813	0.101088466
YLDs (Year	Viet Nam	Female	55+ years	Non-melanoma skin cancer (basal-cell	Number	2021	0.3672163	0.725899987	0.14396851
YLDs (Year	Viet Nam	Both	55+ years	Non-melanoma skin cancer (basal-cell	Number	2021	0.6248343	1.237051888	0.245791282
YLDs (Year	Viet Nam	Male	55+ years	Non-melanoma skin cancer (basal-cell	Percent	2021	1.89E-07	3.68E-07	8.14E-08
YLDs (Year	Viet Nam	Female	55+ years	Non-melanoma skin cancer (basal-cell	Percent	2021	1.86E-07	3.48E-07	8.20E-08
YLDs (Year	Viet Nam	Both	55+ years	Non-melanoma skin cancer (basal-cell	Percent	2021	1.88E-07	3.67E-07	8.17E-08
YLDs (Year	Viet Nam	Male	55+ years	Non-melanoma skin cancer (basal-cell	Rate	2021	0.0033726	0.006732615	0.0013234
YLDs (Year	Viet Nam	Female	55+ years	Non-melanoma skin cancer (basal-cell	Rate	2021	0.0037357	0.007384501	0.001464576
YLDs (Year	Viet Nam	Both	55+ years	Non-melanoma skin cancer (basal-cell	Rate	2021	0.0035769	0.007081578	0.001407047
YLDs (Year	Poland	Male	55+ years	Non-melanoma skin cancer (basal-cell	Number	1990	1.3207819	2.552022677	0.537909825
YLDs (Year	Poland	Female	55+ years	Non-melanoma skin cancer (basal-cell	Number	1990	1.8739041	3.62613194	0.767879288
YLDs (Year	Poland	Both	55+ years	Non-melanoma skin cancer (basal-cell	Number	1990	3.194686	6.154378666	1.312952583
YLDs (Year	Poland	Male	55+ years	Non-melanoma skin cancer (basal-cell	Percent	1990	1.80E-06	3.25E-06	8.41E-07
YLDs (Year	Poland	Female	55+ years	Non-melanoma skin cancer (basal-cell	Percent	1990	1.74E-06	3.19E-06	7.98E-07
YLDs (Year	Poland	Both	55+ years	Non-melanoma skin cancer (basal-cell	Percent	1990	1.77E-06	3.22E-06	8.17E-07
YLDs (Year	Poland	Male	55+ years	Non-melanoma skin cancer (basal-cell	Rate	1990	0.0411946	0.079596358	0.016777148
YLDs (Year	Poland	Female	55+ years	Non-melanoma skin cancer (basal-cell	Rate	1990	0.0416444	0.080584755	0.017064841
YLDs (Year	Poland	Both	55+ years	Non-melanoma skin cancer (basal-cell	Rate	1990	0.0414572	0.079864976	0.017038101
YLDs (Year	Poland	Male	55+ years	Non-melanoma skin cancer (basal-cell	Number	2021	3.166712	6.08115548	1.351397414
YLDs (Year	Poland	Female	55+ years	Non-melanoma skin cancer (basal-cell	Number	2021	4.0921393	7.969299392	1.657833926
YLDs (Year	Poland	Both	55+ years	Non-melanoma skin cancer (basal-cell	Number	2021	7.2588513	14.12256816	3.016791093
YLDs (Year	Poland	Male	55+ years	Non-melanoma skin cancer (basal-cell	Percent	2021	2.68E-06	4.78E-06	1.25E-06
YLDs (Year	Poland	Female	55+ years	Non-melanoma skin cancer (basal-cell	Percent	2021	2.46E-06	4.58E-06	1.13E-06
YLDs (Year	Poland	Both	55+ years	Non-melanoma skin cancer (basal-cell	Percent	2021	2.55E-06	4.67E-06	1.19E-06
YLDs (Year	Poland	Male	55+ years	Non-melanoma skin cancer (basal-cell	Rate	2021	0.0604142	0.116015563	0.0257818
YLDs (Year	Poland	Female	55+ years	Non-melanoma skin cancer (basal-cell	Rate	2021	0.0595335	0.115939341	0.024118579

YLDs (Year	Poland	Both	55+ years	Non-melanoma skin cancer (basal-cell	Rate	2021	0.0599145	0.116567541	0.024900564
YLDs (Year	Republic o	Male	55+ years	Non-melanoma skin cancer (basal-cell	Number	1990	0.1514321	0.298586962	0.064430803
YLDs (Year	Republic o	Female	55+ years	Non-melanoma skin cancer (basal-cell	Number	1990	0.2270039	0.432974867	0.094266382
YLDs (Year	Republic o	Both	55+ years	Non-melanoma skin cancer (basal-cell	Number	1990	0.378436	0.723445565	0.155398287
YLDs (Year	Republic o	Male	55+ years	Non-melanoma skin cancer (basal-cell	Percent	1990	2.35E-06	4.25E-06	1.10E-06
YLDs (Year	Republic o	Female	55+ years	Non-melanoma skin cancer (basal-cell	Percent	1990	2.18E-06	4.05E-06	9.89E-07
YLDs (Year	Republic o	Both	55+ years	Non-melanoma skin cancer (basal-cell	Percent	1990	2.24E-06	4.02E-06	1.05E-06
YLDs (Year	Republic o	Male	55+ years	Non-melanoma skin cancer (basal-cell	Rate	1990	0.048578	0.095783825	0.020668782
YLDs (Year	Republic o	Female	55+ years	Non-melanoma skin cancer (basal-cell	Rate	1990	0.0493182	0.094066939	0.020480057
YLDs (Year	Republic o	Both	55+ years	Non-melanoma skin cancer (basal-cell	Rate	1990	0.0490193	0.093708886	0.020128951
YLDs (Year	Republic o	Male	55+ years	Non-melanoma skin cancer (basal-cell	Number	2021	0.2225603	0.437677078	0.094248293
YLDs (Year	Republic o	Female	55+ years	Non-melanoma skin cancer (basal-cell	Number	2021	0.3318372	0.630840688	0.142243096
YLDs (Year	Republic o	Both	55+ years	Non-melanoma skin cancer (basal-cell	Number	2021	0.5543975	1.06853822	0.23792199
YLDs (Year	Republic o	Male	55+ years	Non-melanoma skin cancer (basal-cell	Percent	2021	2.49E-06	4.58E-06	1.18E-06
YLDs (Year	Republic o	Female	55+ years	Non-melanoma skin cancer (basal-cell	Percent	2021	2.25E-06	4.17E-06	1.03E-06
YLDs (Year	Republic o	Both	55+ years	Non-melanoma skin cancer (basal-cell	Percent	2021	2.34E-06	4.29E-06	1.11E-06
YLDs (Year	Republic o	Male	55+ years	Non-melanoma skin cancer (basal-cell	Rate	2021	0.0520546	0.102368203	0.022043714
YLDs (Year	Republic o	Female	55+ years	Non-melanoma skin cancer (basal-cell	Rate	2021	0.0528167	0.100407368	0.022640034
YLDs (Year	Republic o	Both	55+ years	Non-melanoma skin cancer (basal-cell	Rate	2021	0.0525081	0.101203331	0.022534054
YLDs (Year	Japan	Male	55+ years	Non-melanoma skin cancer (basal-cell	Number	1990	1.4746297	2.844715525	0.596758593
YLDs (Year	Japan	Female	55+ years	Non-melanoma skin cancer (basal-cell	Number	1990	1.7455116	3.371205755	0.709987605
YLDs (Year	Japan	Both	55+ years	Non-melanoma skin cancer (basal-cell	Number	1990	3.2201413	6.176124933	1.305901957
YLDs (Year	Japan	Male	55+ years	Non-melanoma skin cancer (basal-cell	Percent	1990	5.83E-07	1.08E-06	2.74E-07
YLDs (Year	Japan	Female	55+ years	Non-melanoma skin cancer (basal-cell	Percent	1990	5.25E-07	9.75E-07	2.40E-07
YLDs (Year	Japan	Both	55+ years	Non-melanoma skin cancer (basal-cell	Percent	1990	5.50E-07	1.01E-06	2.53E-07
YLDs (Year	Japan	Male	55+ years	Non-melanoma skin cancer (basal-cell	Rate	1990	0.0112215	0.021647386	0.004541144
YLDs (Year	Japan	Female	55+ years	Non-melanoma skin cancer (basal-cell	Rate	1990	0.0105968	0.020466153	0.004310243
YLDs (Year	Japan	Both	55+ years	Non-melanoma skin cancer (basal-cell	Rate	1990	0.010874	0.020855949	0.004409857
YLDs (Year	Japan	Male	55+ years	Non-melanoma skin cancer (basal-cell	Number	2021	4.9080556	9.50568413	1.965874208
YLDs (Year	Japan	Female	55+ years	Non-melanoma skin cancer (basal-cell	Number	2021	6.0208749	11.7657365	2.471853556
YLDs (Year	Japan	Both	55+ years	Non-melanoma skin cancer (basal-cell	Number	2021	10.92893	21.34185969	4.406417505
YLDs (Year	Japan	Male	55+ years	Non-melanoma skin cancer (basal-cell	Percent	2021	9.78E-07	1.81E-06	4.41E-07
YLDs (Year	Japan	Female	55+ years	Non-melanoma skin cancer (basal-cell	Percent	2021	9.35E-07	1.76E-06	4.23E-07
YLDs (Year	Japan	Both	55+ years	Non-melanoma skin cancer (basal-cell	Percent	2021	9.54E-07	1.79E-06	4.32E-07
YLDs (Year	Japan	Male	55+ years	Non-melanoma skin cancer (basal-cell	Rate	2021	0.0206639	0.040020868	0.008276731
YLDs (Year	Japan	Female	55+ years	Non-melanoma skin cancer (basal-cell	Rate	2021	0.0211637	0.041357163	0.008688691
YLDs (Year	Japan	Both	55+ years	Non-melanoma skin cancer (basal-cell	Rate	2021	0.0209363	0.040884077	0.008441266
YLDs (Year	Taiwan (Pr	Male	55+ years	Non-melanoma skin cancer (basal-cell	Number	1990	0.1204465	0.237145033	0.055781528
YLDs (Year	Taiwan (Pr	Female	55+ years	Non-melanoma skin cancer (basal-cell	Number	1990	0.077793	0.153653371	0.033713438
YLDs (Year	Taiwan (Pr	Both	55+ years	Non-melanoma skin cancer (basal-cell	Number	1990	0.1982394	0.391368376	0.089146507
YLDs (Year	Taiwan (Pr	Male	55+ years	Non-melanoma skin cancer (basal-cell	Percent	1990	4.57E-07	8.07E-07	2.19E-07
YLDs (Year	Taiwan (Pr	Female	55+ years	Non-melanoma skin cancer (basal-cell	Percent	1990	3.02E-07	5.55E-07	1.33E-07
YLDs (Year	Taiwan (Pr	Both	55+ years	Non-melanoma skin cancer (basal-cell	Percent	1990	3.80E-07	6.83E-07	1.80E-07
YLDs (Year	Taiwan (Pr	Male	55+ years	Non-melanoma skin cancer (basal-cell	Rate	1990	0.0079673	0.015686739	0.003689853
YLDs (Year	Taiwan (Pr	Female	55+ years	Non-melanoma skin cancer (basal-cell	Rate	1990	0.0061649	0.012176656	0.002671708
YLDs (Year	Taiwan (Pr	Both	55+ years	Non-melanoma skin cancer (basal-cell	Rate	1990	0.0071473	0.014110366	0.003214081
YLDs (Year	Taiwan (Pr	Male	55+ years	Non-melanoma skin cancer (basal-cell	Number	2021	0.0071178	0.016313826	0.002260727
YLDs (Year	Taiwan (Pr	Female	55+ years	Non-melanoma skin cancer (basal-cell	Number	2021	0.0083664	0.019431761	0.002495078
YLDs (Year	Taiwan (Pr	Both	55+ years	Non-melanoma skin cancer (basal-cell	Number	2021	0.0154842	0.035197019	0.00473479
YLDs (Year	Taiwan (Pr	Male	55+ years	Non-melanoma skin cancer (basal-cell	Percent	2021	1.04E-08	2.24E-08	3.57E-09
YLDs (Year	Taiwan (Pr	Female	55+ years	Non-melanoma skin cancer (basal-cell	Percent	2021	9.55E-09	2.14E-08	2.96E-09
YLDs (Year	Taiwan (Pr	Both	55+ years	Non-melanoma skin cancer (basal-cell	Percent	2021	9.92E-09	2.17E-08	3.27E-09
YLDs (Year	Taiwan (Pr	Male	55+ years	Non-melanoma skin cancer (basal-cell	Rate	2021	0.0002013	0.000461449	6.39E-05
YLDs (Year	Taiwan (Pr	Female	55+ years	Non-melanoma skin cancer (basal-cell	Rate	2021	0.0002094	0.000486452	6.25E-05
YLDs (Year	Taiwan (Pr	Both	55+ years	Non-melanoma skin cancer (basal-cell	Rate	2021	0.0002056	0.000467428	6.29E-05
YLDs (Year	North Mace	Male	55+ years	Non-melanoma skin cancer (basal-cell	Number	1990	0.1039896	0.200339533	0.043504636

YLDs (Year	North Mace	Female	55+ years	Non-melanoma skin cancer (basal-cell	Number	1990	0.0972364	0.185611434	0.040722291
YLDs (Year	North Mace	Both	55+ years	Non-melanoma skin cancer (basal-cell	Number	1990	0.201226	0.384015341	0.085562869
YLDs (Year	North Mace	Male	55+ years	Non-melanoma skin cancer (basal-cell	Percent	1990	3.25E-06	6.02E-06	1.41E-06
YLDs (Year	North Mace	Female	55+ years	Non-melanoma skin cancer (basal-cell	Percent	1990	2.53E-06	4.69E-06	1.16E-06
YLDs (Year	North Mace	Both	55+ years	Non-melanoma skin cancer (basal-cell	Percent	1990	2.86E-06	5.25E-06	1.28E-06
YLDs (Year	North Mace	Male	55+ years	Non-melanoma skin cancer (basal-cell	Rate	1990	0.0667374	0.128571865	0.027919962
YLDs (Year	North Mace	Female	55+ years	Non-melanoma skin cancer (basal-cell	Rate	1990	0.0561583	0.107198776	0.023518916
YLDs (Year	North Mace	Both	55+ years	Non-melanoma skin cancer (basal-cell	Rate	1990	0.0611692	0.116734024	0.026009633
YLDs (Year	North Mace	Male	55+ years	Non-melanoma skin cancer (basal-cell	Number	2021	0.1970752	0.397047798	0.080456489
YLDs (Year	North Mace	Female	55+ years	Non-melanoma skin cancer (basal-cell	Number	2021	0.1848507	0.358857478	0.077239406
YLDs (Year	North Mace	Both	55+ years	Non-melanoma skin cancer (basal-cell	Number	2021	0.3819259	0.745872168	0.154945952
YLDs (Year	North Mace	Male	55+ years	Non-melanoma skin cancer (basal-cell	Percent	2021	3.27E-06	6.09E-06	1.43E-06
YLDs (Year	North Mace	Female	55+ years	Non-melanoma skin cancer (basal-cell	Percent	2021	2.50E-06	4.79E-06	1.13E-06
YLDs (Year	North Mace	Both	55+ years	Non-melanoma skin cancer (basal-cell	Percent	2021	2.84E-06	5.34E-06	1.31E-06
YLDs (Year	North Mace	Male	55+ years	Non-melanoma skin cancer (basal-cell	Rate	2021	0.068978	0.138970112	0.028160457
YLDs (Year	North Mace	Female	55+ years	Non-melanoma skin cancer (basal-cell	Rate	2021	0.0601731	0.116816175	0.025143163
YLDs (Year	North Mace	Both	55+ years	Non-melanoma skin cancer (basal-cell	Rate	2021	0.064416	0.125799443	0.026133318
YLDs (Year	Belarus	Male	55+ years	Non-melanoma skin cancer (basal-cell	Number	1990	0.4184656	0.834580288	0.19252106
YLDs (Year	Belarus	Female	55+ years	Non-melanoma skin cancer (basal-cell	Number	1990	0.8046645	1.619287252	0.358985173
YLDs (Year	Belarus	Both	55+ years	Non-melanoma skin cancer (basal-cell	Number	1990	1.2231301	2.424595002	0.561185093
YLDs (Year	Belarus	Male	55+ years	Non-melanoma skin cancer (basal-cell	Percent	1990	2.37E-06	4.19E-06	1.18E-06
YLDs (Year	Belarus	Female	55+ years	Non-melanoma skin cancer (basal-cell	Percent	1990	2.42E-06	4.35E-06	1.16E-06
YLDs (Year	Belarus	Both	55+ years	Non-melanoma skin cancer (basal-cell	Percent	1990	2.40E-06	4.23E-06	1.17E-06
YLDs (Year	Belarus	Male	55+ years	Non-melanoma skin cancer (basal-cell	Rate	1990	0.0491692	0.098062239	0.022621007
YLDs (Year	Belarus	Female	55+ years	Non-melanoma skin cancer (basal-cell	Rate	1990	0.0549686	0.110617466	0.024523154
YLDs (Year	Belarus	Both	55+ years	Non-melanoma skin cancer (basal-cell	Rate	1990	0.0528365	0.104737102	0.024241946
YLDs (Year	Belarus	Male	55+ years	Non-melanoma skin cancer (basal-cell	Number	2021	0.5683744	1.097207916	0.243850094
YLDs (Year	Belarus	Female	55+ years	Non-melanoma skin cancer (basal-cell	Number	2021	1.0395041	2.041562141	0.424774654
YLDs (Year	Belarus	Both	55+ years	Non-melanoma skin cancer (basal-cell	Number	2021	1.6078785	3.112586874	0.66227944
YLDs (Year	Belarus	Male	55+ years	Non-melanoma skin cancer (basal-cell	Percent	2021	2.39E-06	4.37E-06	1.12E-06
YLDs (Year	Belarus	Female	55+ years	Non-melanoma skin cancer (basal-cell	Percent	2021	2.50E-06	4.59E-06	1.14E-06
YLDs (Year	Belarus	Both	55+ years	Non-melanoma skin cancer (basal-cell	Percent	2021	2.46E-06	4.53E-06	1.13E-06
YLDs (Year	Belarus	Male	55+ years	Non-melanoma skin cancer (basal-cell	Rate	2021	0.0512358	0.098907229	0.021981738
YLDs (Year	Belarus	Female	55+ years	Non-melanoma skin cancer (basal-cell	Rate	2021	0.0588197	0.115520522	0.024035609
YLDs (Year	Belarus	Both	55+ years	Non-melanoma skin cancer (basal-cell	Rate	2021	0.0558951	0.108203575	0.023022973
YLDs (Year	Australia	Male	55+ years	Non-melanoma skin cancer (basal-cell	Number	1990	1.3269894	2.669396701	0.497429991
YLDs (Year	Australia	Female	55+ years	Non-melanoma skin cancer (basal-cell	Number	1990	1.3706153	2.737393279	0.559089641
YLDs (Year	Australia	Both	55+ years	Non-melanoma skin cancer (basal-cell	Number	1990	2.6976047	5.174994201	1.116432836
YLDs (Year	Australia	Male	55+ years	Non-melanoma skin cancer (basal-cell	Percent	1990	4.19E-06	8.10E-06	1.76E-06
YLDs (Year	Australia	Female	55+ years	Non-melanoma skin cancer (basal-cell	Percent	1990	3.39E-06	6.34E-06	1.59E-06
YLDs (Year	Australia	Both	55+ years	Non-melanoma skin cancer (basal-cell	Percent	1990	3.74E-06	6.76E-06	1.72E-06
YLDs (Year	Australia	Male	55+ years	Non-melanoma skin cancer (basal-cell	Rate	1990	0.0880888	0.177201165	0.033020635
YLDs (Year	Australia	Female	55+ years	Non-melanoma skin cancer (basal-cell	Rate	1990	0.0772128	0.154209364	0.031495971
YLDs (Year	Australia	Both	55+ years	Non-melanoma skin cancer (basal-cell	Rate	1990	0.0822055	0.157700341	0.03402165
YLDs (Year	Australia	Male	55+ years	Non-melanoma skin cancer (basal-cell	Number	2021	3.2213154	6.360053977	1.325976608
YLDs (Year	Australia	Female	55+ years	Non-melanoma skin cancer (basal-cell	Number	2021	2.7523903	5.202275889	1.163393137
YLDs (Year	Australia	Both	55+ years	Non-melanoma skin cancer (basal-cell	Number	2021	5.9737057	11.57858745	2.545346021
YLDs (Year	Australia	Male	55+ years	Non-melanoma skin cancer (basal-cell	Percent	2021	4.22E-06	7.84E-06	1.90E-06
YLDs (Year	Australia	Female	55+ years	Non-melanoma skin cancer (basal-cell	Percent	2021	3.02E-06	5.64E-06	1.36E-06
YLDs (Year	Australia	Both	55+ years	Non-melanoma skin cancer (basal-cell	Percent	2021	3.57E-06	6.70E-06	1.65E-06
YLDs (Year	Australia	Male	55+ years	Non-melanoma skin cancer (basal-cell	Rate	2021	0.0914423	0.180540539	0.037640016
YLDs (Year	Australia	Female	55+ years	Non-melanoma skin cancer (basal-cell	Rate	2021	0.0708458	0.133905261	0.029945444
YLDs (Year	Australia	Both	55+ years	Non-melanoma skin cancer (basal-cell	Rate	2021	0.0806405	0.156302085	0.034360227
YLDs (Year	Bosnia and	Male	55+ years	Non-melanoma skin cancer (basal-cell	Number	1990	0.2040836	0.404727897	0.084537962
YLDs (Year	Bosnia and	Female	55+ years	Non-melanoma skin cancer (basal-cell	Number	1990	0.2428586	0.468512744	0.103851874
YLDs (Year	Bosnia and	Both	55+ years	Non-melanoma skin cancer (basal-cell	Number	1990	0.4469422	0.879401378	0.186436493

YLDs (Year	Bosnia and	Male	55+ years	Non-melanoma skin cancer (basal-cell	Percent	1990	3.03E-06	5.73E-06	1.35E-06
YLDs (Year	Bosnia and	Female	55+ years	Non-melanoma skin cancer (basal-cell	Percent	1990	2.72E-06	5.03E-06	1.22E-06
YLDs (Year	Bosnia and	Both	55+ years	Non-melanoma skin cancer (basal-cell	Percent	1990	2.85E-06	5.44E-06	1.31E-06
YLDs (Year	Bosnia and	Male	55+ years	Non-melanoma skin cancer (basal-cell	Rate	1990	0.0633024	0.125537938	0.026221868
YLDs (Year	Bosnia and	Female	55+ years	Non-melanoma skin cancer (basal-cell	Rate	1990	0.0590685	0.113952457	0.025259027
YLDs (Year	Bosnia and	Both	55+ years	Non-melanoma skin cancer (basal-cell	Rate	1990	0.0609293	0.119884184	0.025415911
YLDs (Year	Bosnia and	Male	55+ years	Non-melanoma skin cancer (basal-cell	Number	2021	0.3758532	0.75502686	0.158409159
YLDs (Year	Bosnia and	Female	55+ years	Non-melanoma skin cancer (basal-cell	Number	2021	0.410898	0.815630975	0.162387881
YLDs (Year	Bosnia and	Both	55+ years	Non-melanoma skin cancer (basal-cell	Number	2021	0.7867512	1.549224172	0.324017555
YLDs (Year	Bosnia and	Male	55+ years	Non-melanoma skin cancer (basal-cell	Percent	2021	3.45E-06	6.49E-06	1.57E-06
YLDs (Year	Bosnia and	Female	55+ years	Non-melanoma skin cancer (basal-cell	Percent	2021	2.78E-06	5.22E-06	1.23E-06
YLDs (Year	Bosnia and	Both	55+ years	Non-melanoma skin cancer (basal-cell	Percent	2021	3.06E-06	5.80E-06	1.47E-06
YLDs (Year	Bosnia and	Male	55+ years	Non-melanoma skin cancer (basal-cell	Rate	2021	0.0765201	0.153716147	0.032250569
YLDs (Year	Bosnia and	Female	55+ years	Non-melanoma skin cancer (basal-cell	Rate	2021	0.0680198	0.135019007	0.026881581
YLDs (Year	Bosnia and	Both	55+ years	Non-melanoma skin cancer (basal-cell	Rate	2021	0.0718318	0.141446967	0.029583388
YLDs (Year	Slovenia	Male	55+ years	Non-melanoma skin cancer (basal-cell	Number	1990	0.1231206	0.25124376	0.054165895
YLDs (Year	Slovenia	Female	55+ years	Non-melanoma skin cancer (basal-cell	Number	1990	0.1851528	0.355260979	0.081550742
YLDs (Year	Slovenia	Both	55+ years	Non-melanoma skin cancer (basal-cell	Number	1990	0.3082734	0.598077729	0.139704724
YLDs (Year	Slovenia	Male	55+ years	Non-melanoma skin cancer (basal-cell	Percent	1990	3.16E-06	5.97E-06	1.54E-06
YLDs (Year	Slovenia	Female	55+ years	Non-melanoma skin cancer (basal-cell	Percent	1990	3.02E-06	5.43E-06	1.35E-06
YLDs (Year	Slovenia	Both	55+ years	Non-melanoma skin cancer (basal-cell	Percent	1990	3.07E-06	5.34E-06	1.48E-06
YLDs (Year	Slovenia	Male	55+ years	Non-melanoma skin cancer (basal-cell	Rate	1990	0.0715488	0.146004757	0.031477313
YLDs (Year	Slovenia	Female	55+ years	Non-melanoma skin cancer (basal-cell	Rate	1990	0.0715906	0.137364028	0.031532139
YLDs (Year	Slovenia	Both	55+ years	Non-melanoma skin cancer (basal-cell	Rate	1990	0.0715739	0.138859683	0.032436175
YLDs (Year	Slovenia	Male	55+ years	Non-melanoma skin cancer (basal-cell	Number	2021	0.3951345	0.869631815	0.144314131
YLDs (Year	Slovenia	Female	55+ years	Non-melanoma skin cancer (basal-cell	Number	2021	0.4068953	0.792326924	0.166434938
YLDs (Year	Slovenia	Both	55+ years	Non-melanoma skin cancer (basal-cell	Number	2021	0.8020298	1.622414503	0.322334799
YLDs (Year	Slovenia	Male	55+ years	Non-melanoma skin cancer (basal-cell	Percent	2021	5.33E-06	1.06E-05	2.14E-06
YLDs (Year	Slovenia	Female	55+ years	Non-melanoma skin cancer (basal-cell	Percent	2021	4.15E-06	7.80E-06	1.88E-06
YLDs (Year	Slovenia	Both	55+ years	Non-melanoma skin cancer (basal-cell	Percent	2021	4.66E-06	8.56E-06	2.01E-06
YLDs (Year	Slovenia	Male	55+ years	Non-melanoma skin cancer (basal-cell	Rate	2021	0.1179039	0.259488727	0.043061776
YLDs (Year	Slovenia	Female	55+ years	Non-melanoma skin cancer (basal-cell	Rate	2021	0.1024781	0.19955042	0.041917245
YLDs (Year	Slovenia	Both	55+ years	Non-melanoma skin cancer (basal-cell	Rate	2021	0.1095387	0.221584175	0.044023454
YLDs (Year	Czechia	Male	55+ years	Non-melanoma skin cancer (basal-cell	Number	1990	1.2180913	2.503377034	0.506730358
YLDs (Year	Czechia	Female	55+ years	Non-melanoma skin cancer (basal-cell	Number	1990	1.504928	2.997669793	0.658344295
YLDs (Year	Czechia	Both	55+ years	Non-melanoma skin cancer (basal-cell	Number	1990	2.7230194	5.56677069	1.181793469
YLDs (Year	Czechia	Male	55+ years	Non-melanoma skin cancer (basal-cell	Percent	1990	5.57E-06	1.06E-05	2.47E-06
YLDs (Year	Czechia	Female	55+ years	Non-melanoma skin cancer (basal-cell	Percent	1990	4.43E-06	8.38E-06	2.08E-06
YLDs (Year	Czechia	Both	55+ years	Non-melanoma skin cancer (basal-cell	Percent	1990	4.88E-06	8.74E-06	2.43E-06
YLDs (Year	Czechia	Male	55+ years	Non-melanoma skin cancer (basal-cell	Rate	1990	0.1241015	0.255048822	0.051626654
YLDs (Year	Czechia	Female	55+ years	Non-melanoma skin cancer (basal-cell	Rate	1990	0.1085561	0.216233195	0.04748885
YLDs (Year	Czechia	Both	55+ years	Non-melanoma skin cancer (basal-cell	Rate	1990	0.115	0.23509891	0.049910149
YLDs (Year	Czechia	Male	55+ years	Non-melanoma skin cancer (basal-cell	Number	2021	1.7123459	3.722883024	0.693556873
YLDs (Year	Czechia	Female	55+ years	Non-melanoma skin cancer (basal-cell	Number	2021	1.908939	3.753814788	0.74372502
YLDs (Year	Czechia	Both	55+ years	Non-melanoma skin cancer (basal-cell	Number	2021	3.6212849	7.250290998	1.468558596
YLDs (Year	Czechia	Male	55+ years	Non-melanoma skin cancer (basal-cell	Percent	2021	4.70E-06	9.01E-06	2.04E-06
YLDs (Year	Czechia	Female	55+ years	Non-melanoma skin cancer (basal-cell	Percent	2021	3.93E-06	7.59E-06	1.53E-06
YLDs (Year	Czechia	Both	55+ years	Non-melanoma skin cancer (basal-cell	Percent	2021	4.26E-06	7.69E-06	1.78E-06
YLDs (Year	Czechia	Male	55+ years	Non-melanoma skin cancer (basal-cell	Rate	2021	0.1089894	0.236958349	0.044144307
YLDs (Year	Czechia	Female	55+ years	Non-melanoma skin cancer (basal-cell	Rate	2021	0.0993023	0.195272068	0.038688303
YLDs (Year	Czechia	Both	55+ years	Non-melanoma skin cancer (basal-cell	Rate	2021	0.1036589	0.207538738	0.042037319
YLDs (Year	Albania	Male	55+ years	Non-melanoma skin cancer (basal-cell	Number	1990	0.1194007	0.23290844	0.047679236
YLDs (Year	Albania	Female	55+ years	Non-melanoma skin cancer (basal-cell	Number	1990	0.1203575	0.255640693	0.050489909
YLDs (Year	Albania	Both	55+ years	Non-melanoma skin cancer (basal-cell	Number	1990	0.2397582	0.462010466	0.100544957
YLDs (Year	Albania	Male	55+ years	Non-melanoma skin cancer (basal-cell	Percent	1990	3.52E-06	6.71E-06	1.44E-06
YLDs (Year	Albania	Female	55+ years	Non-melanoma skin cancer (basal-cell	Percent	1990	3.24E-06	6.30E-06	1.43E-06

YLDs (Year	Albania	Both	55+ years	Non-melanoma skin cancer (basal-cell	Percent	1990	3.38E-06	6.23E-06	1.54E-06
YLDs (Year	Albania	Male	55+ years	Non-melanoma skin cancer (basal-cell	Rate	1990	0.0701915	0.136918767	0.028028964
YLDs (Year	Albania	Female	55+ years	Non-melanoma skin cancer (basal-cell	Rate	1990	0.0680303	0.144497098	0.02853867
YLDs (Year	Albania	Both	55+ years	Non-melanoma skin cancer (basal-cell	Rate	1990	0.0690897	0.133134807	0.028973442
YLDs (Year	Albania	Male	55+ years	Non-melanoma skin cancer (basal-cell	Number	2021	0.2981369	0.593938777	0.117373937
YLDs (Year	Albania	Female	55+ years	Non-melanoma skin cancer (basal-cell	Number	2021	0.2838834	0.569191282	0.116679345
YLDs (Year	Albania	Both	55+ years	Non-melanoma skin cancer (basal-cell	Number	2021	0.5820203	1.138636262	0.252149112
YLDs (Year	Albania	Male	55+ years	Non-melanoma skin cancer (basal-cell	Percent	2021	3.89E-06	7.45E-06	1.64E-06
YLDs (Year	Albania	Female	55+ years	Non-melanoma skin cancer (basal-cell	Percent	2021	3.18E-06	6.12E-06	1.45E-06
YLDs (Year	Albania	Both	55+ years	Non-melanoma skin cancer (basal-cell	Percent	2021	3.51E-06	6.49E-06	1.63E-06
YLDs (Year	Albania	Male	55+ years	Non-melanoma skin cancer (basal-cell	Rate	2021	0.0787568	0.156896699	0.031005861
YLDs (Year	Albania	Female	55+ years	Non-melanoma skin cancer (basal-cell	Rate	2021	0.0696236	0.139596619	0.028616113
YLDs (Year	Albania	Both	55+ years	Non-melanoma skin cancer (basal-cell	Rate	2021	0.0740207	0.144810489	0.032068043
YLDs (Year	Uzbekistan	Male	55+ years	Non-melanoma skin cancer (basal-cell	Number	1990	0.4164844	0.794699029	0.176812863
YLDs (Year	Uzbekistan	Female	55+ years	Non-melanoma skin cancer (basal-cell	Number	1990	0.6066072	1.197035993	0.248622405
YLDs (Year	Uzbekistan	Both	55+ years	Non-melanoma skin cancer (basal-cell	Number	1990	1.0230916	1.997238106	0.432804356
YLDs (Year	Uzbekistan	Male	55+ years	Non-melanoma skin cancer (basal-cell	Percent	1990	2.76E-06	4.92E-06	1.28E-06
YLDs (Year	Uzbekistan	Female	55+ years	Non-melanoma skin cancer (basal-cell	Percent	1990	2.48E-06	4.67E-06	1.15E-06
YLDs (Year	Uzbekistan	Both	55+ years	Non-melanoma skin cancer (basal-cell	Percent	1990	2.58E-06	4.77E-06	1.20E-06
YLDs (Year	Uzbekistan	Male	55+ years	Non-melanoma skin cancer (basal-cell	Rate	1990	0.0510899	0.097485234	0.021689524
YLDs (Year	Uzbekistan	Female	55+ years	Non-melanoma skin cancer (basal-cell	Rate	1990	0.0531884	0.104958228	0.021799651
YLDs (Year	Uzbekistan	Both	55+ years	Non-melanoma skin cancer (basal-cell	Rate	1990	0.0523137	0.10212461	0.022130549
YLDs (Year	Uzbekistan	Male	55+ years	Non-melanoma skin cancer (basal-cell	Number	2021	1.0191519	1.970268274	0.435443806
YLDs (Year	Uzbekistan	Female	55+ years	Non-melanoma skin cancer (basal-cell	Number	2021	1.2182105	2.427143537	0.497873135
YLDs (Year	Uzbekistan	Both	55+ years	Non-melanoma skin cancer (basal-cell	Number	2021	2.2373624	4.319088103	0.940462393
YLDs (Year	Uzbekistan	Male	55+ years	Non-melanoma skin cancer (basal-cell	Percent	2021	2.62E-06	4.68E-06	1.23E-06
YLDs (Year	Uzbekistan	Female	55+ years	Non-melanoma skin cancer (basal-cell	Percent	2021	2.23E-06	4.19E-06	1.02E-06
YLDs (Year	Uzbekistan	Both	55+ years	Non-melanoma skin cancer (basal-cell	Percent	2021	2.39E-06	4.40E-06	1.11E-06
YLDs (Year	Uzbekistan	Male	55+ years	Non-melanoma skin cancer (basal-cell	Rate	2021	0.0481043	0.092997205	0.020553068
YLDs (Year	Uzbekistan	Female	55+ years	Non-melanoma skin cancer (basal-cell	Rate	2021	0.0481502	0.095933803	0.019678632
YLDs (Year	Uzbekistan	Both	55+ years	Non-melanoma skin cancer (basal-cell	Rate	2021	0.0481293	0.092910573	0.020230868
YLDs (Year	Romania	Male	55+ years	Non-melanoma skin cancer (basal-cell	Number	1990	0.8148629	1.612377121	0.356540976
YLDs (Year	Romania	Female	55+ years	Non-melanoma skin cancer (basal-cell	Number	1990	0.9824778	1.928497115	0.419962721
YLDs (Year	Romania	Both	55+ years	Non-melanoma skin cancer (basal-cell	Number	1990	1.7973407	3.483731332	0.783894177
YLDs (Year	Romania	Male	55+ years	Non-melanoma skin cancer (basal-cell	Percent	1990	1.70E-06	3.05E-06	8.27E-07
YLDs (Year	Romania	Female	55+ years	Non-melanoma skin cancer (basal-cell	Percent	1990	1.62E-06	2.81E-06	7.54E-07
YLDs (Year	Romania	Both	55+ years	Non-melanoma skin cancer (basal-cell	Percent	1990	1.65E-06	2.91E-06	7.87E-07
YLDs (Year	Romania	Male	55+ years	Non-melanoma skin cancer (basal-cell	Rate	1990	0.0363746	0.071974695	0.015915587
YLDs (Year	Romania	Female	55+ years	Non-melanoma skin cancer (basal-cell	Rate	1990	0.03555	0.06978085	0.015195955
YLDs (Year	Romania	Both	55+ years	Non-melanoma skin cancer (basal-cell	Rate	1990	0.0359192	0.069621045	0.015665827
YLDs (Year	Romania	Male	55+ years	Non-melanoma skin cancer (basal-cell	Number	2021	1.6280455	3.360819358	0.675510414
YLDs (Year	Romania	Female	55+ years	Non-melanoma skin cancer (basal-cell	Number	2021	2.0353983	3.915635991	0.850899132
YLDs (Year	Romania	Both	55+ years	Non-melanoma skin cancer (basal-cell	Number	2021	3.6634438	7.420448006	1.515669954
YLDs (Year	Romania	Male	55+ years	Non-melanoma skin cancer (basal-cell	Percent	2021	2.91E-06	5.26E-06	1.37E-06
YLDs (Year	Romania	Female	55+ years	Non-melanoma skin cancer (basal-cell	Percent	2021	2.57E-06	4.72E-06	1.20E-06
YLDs (Year	Romania	Both	55+ years	Non-melanoma skin cancer (basal-cell	Percent	2021	2.71E-06	5.01E-06	1.28E-06
YLDs (Year	Romania	Male	55+ years	Non-melanoma skin cancer (basal-cell	Rate	2021	0.0630715	0.130200253	0.026169698
YLDs (Year	Romania	Female	55+ years	Non-melanoma skin cancer (basal-cell	Rate	2021	0.0594436	0.114355833	0.02485044
YLDs (Year	Romania	Both	55+ years	Non-melanoma skin cancer (basal-cell	Rate	2021	0.061003	0.123563953	0.025238661
YLDs (Year	Lithuania	Male	55+ years	Non-melanoma skin cancer (basal-cell	Number	1990	0.1845727	0.36034239	0.075946377
YLDs (Year	Lithuania	Female	55+ years	Non-melanoma skin cancer (basal-cell	Number	1990	0.3443363	0.697823433	0.146494271
YLDs (Year	Lithuania	Both	55+ years	Non-melanoma skin cancer (basal-cell	Number	1990	0.528909	1.039540325	0.228042832
YLDs (Year	Lithuania	Male	55+ years	Non-melanoma skin cancer (basal-cell	Percent	1990	2.74E-06	4.94E-06	1.27E-06
YLDs (Year	Lithuania	Female	55+ years	Non-melanoma skin cancer (basal-cell	Percent	1990	3.07E-06	6.06E-06	1.40E-06
YLDs (Year	Lithuania	Both	55+ years	Non-melanoma skin cancer (basal-cell	Percent	1990	2.95E-06	5.50E-06	1.37E-06
YLDs (Year	Lithuania	Male	55+ years	Non-melanoma skin cancer (basal-cell	Rate	1990	0.060566	0.118243323	0.024921164

YLDs (Year	Lithuania	Female	55+ years	Non-melanoma skin cancer (basal-cell	Rate	1990	0.0706037	0.143083707	0.030037603
YLDs (Year	Lithuania	Both	55+ years	Non-melanoma skin cancer (basal-cell	Rate	1990	0.0667436	0.131180651	0.028776957
YLDs (Year	Lithuania	Male	55+ years	Non-melanoma skin cancer (basal-cell	Number	2021	0.2372399	0.488484442	0.10592804
YLDs (Year	Lithuania	Female	55+ years	Non-melanoma skin cancer (basal-cell	Number	2021	0.4364436	0.930431129	0.16677173
YLDs (Year	Lithuania	Both	55+ years	Non-melanoma skin cancer (basal-cell	Number	2021	0.6736835	1.350097849	0.29269724
YLDs (Year	Lithuania	Male	55+ years	Non-melanoma skin cancer (basal-cell	Percent	2021	2.83E-06	5.23E-06	1.36E-06
YLDs (Year	Lithuania	Female	55+ years	Non-melanoma skin cancer (basal-cell	Percent	2021	2.98E-06	5.79E-06	1.19E-06
YLDs (Year	Lithuania	Both	55+ years	Non-melanoma skin cancer (basal-cell	Percent	2021	2.92E-06	5.42E-06	1.31E-06
YLDs (Year	Lithuania	Male	55+ years	Non-melanoma skin cancer (basal-cell	Rate	2021	0.0636312	0.131018664	0.028411448
YLDs (Year	Lithuania	Female	55+ years	Non-melanoma skin cancer (basal-cell	Rate	2021	0.0733587	0.156389584	0.02803148
YLDs (Year	Lithuania	Both	55+ years	Non-melanoma skin cancer (basal-cell	Rate	2021	0.0696112	0.139504594	0.030244185
YLDs (Year	Mongolia	Male	55+ years	Non-melanoma skin cancer (basal-cell	Number	1990	0.0406505	0.080384357	0.017590252
YLDs (Year	Mongolia	Female	55+ years	Non-melanoma skin cancer (basal-cell	Number	1990	0.0520093	0.099845205	0.021262985
YLDs (Year	Mongolia	Both	55+ years	Non-melanoma skin cancer (basal-cell	Number	1990	0.0926598	0.178706041	0.038307353
YLDs (Year	Mongolia	Male	55+ years	Non-melanoma skin cancer (basal-cell	Percent	1990	2.69E-06	4.95E-06	1.25E-06
YLDs (Year	Mongolia	Female	55+ years	Non-melanoma skin cancer (basal-cell	Percent	1990	2.55E-06	4.74E-06	1.16E-06
YLDs (Year	Mongolia	Both	55+ years	Non-melanoma skin cancer (basal-cell	Percent	1990	2.61E-06	4.84E-06	1.18E-06
YLDs (Year	Mongolia	Male	55+ years	Non-melanoma skin cancer (basal-cell	Rate	1990	0.0518148	0.102461102	0.022421235
YLDs (Year	Mongolia	Female	55+ years	Non-melanoma skin cancer (basal-cell	Rate	1990	0.0548453	0.105289751	0.022422452
YLDs (Year	Mongolia	Both	55+ years	Non-melanoma skin cancer (basal-cell	Rate	1990	0.0534733	0.103129869	0.022106876
YLDs (Year	Mongolia	Male	55+ years	Non-melanoma skin cancer (basal-cell	Number	2021	0.0780197	0.151752674	0.032231133
YLDs (Year	Mongolia	Female	55+ years	Non-melanoma skin cancer (basal-cell	Number	2021	0.1093036	0.214365709	0.046824429
YLDs (Year	Mongolia	Both	55+ years	Non-melanoma skin cancer (basal-cell	Number	2021	0.1873234	0.368813654	0.079678441
YLDs (Year	Mongolia	Male	55+ years	Non-melanoma skin cancer (basal-cell	Percent	2021	2.38E-06	4.18E-06	1.06E-06
YLDs (Year	Mongolia	Female	55+ years	Non-melanoma skin cancer (basal-cell	Percent	2021	2.29E-06	4.28E-06	1.05E-06
YLDs (Year	Mongolia	Both	55+ years	Non-melanoma skin cancer (basal-cell	Percent	2021	2.32E-06	4.24E-06	1.04E-06
YLDs (Year	Mongolia	Male	55+ years	Non-melanoma skin cancer (basal-cell	Rate	2021	0.046392	0.090235034	0.019165246
YLDs (Year	Mongolia	Female	55+ years	Non-melanoma skin cancer (basal-cell	Rate	2021	0.0482297	0.094587921	0.020661072
YLDs (Year	Mongolia	Both	55+ years	Non-melanoma skin cancer (basal-cell	Rate	2021	0.0474469	0.093416411	0.020181666
YLDs (Year	Turkmenist	Male	55+ years	Non-melanoma skin cancer (basal-cell	Number	1990	0.0693307	0.133402193	0.028267445
YLDs (Year	Turkmenist	Female	55+ years	Non-melanoma skin cancer (basal-cell	Number	1990	0.1050812	0.209721707	0.046232261
YLDs (Year	Turkmenist	Both	55+ years	Non-melanoma skin cancer (basal-cell	Number	1990	0.1744119	0.340552268	0.073525227
YLDs (Year	Turkmenist	Male	55+ years	Non-melanoma skin cancer (basal-cell	Percent	1990	2.88E-06	5.30E-06	1.18E-06
YLDs (Year	Turkmenist	Female	55+ years	Non-melanoma skin cancer (basal-cell	Percent	1990	2.65E-06	4.86E-06	1.24E-06
YLDs (Year	Turkmenist	Both	55+ years	Non-melanoma skin cancer (basal-cell	Percent	1990	2.74E-06	4.92E-06	1.22E-06
YLDs (Year	Turkmenist	Male	55+ years	Non-melanoma skin cancer (basal-cell	Rate	1990	0.0512572	0.098626135	0.020898523
YLDs (Year	Turkmenist	Female	55+ years	Non-melanoma skin cancer (basal-cell	Rate	1990	0.0554487	0.110664961	0.024395621
YLDs (Year	Turkmenist	Both	55+ years	Non-melanoma skin cancer (basal-cell	Rate	1990	0.053703	0.104859206	0.0226391
YLDs (Year	Turkmenist	Male	55+ years	Non-melanoma skin cancer (basal-cell	Number	2021	0.1557477	0.300157516	0.0631657
YLDs (Year	Turkmenist	Female	55+ years	Non-melanoma skin cancer (basal-cell	Number	2021	0.2099106	0.41533233	0.084344414
YLDs (Year	Turkmenist	Both	55+ years	Non-melanoma skin cancer (basal-cell	Number	2021	0.3656583	0.70044704	0.14926114
YLDs (Year	Turkmenist	Male	55+ years	Non-melanoma skin cancer (basal-cell	Percent	2021	2.81E-06	4.89E-06	1.29E-06
YLDs (Year	Turkmenist	Female	55+ years	Non-melanoma skin cancer (basal-cell	Percent	2021	2.52E-06	4.64E-06	1.11E-06
YLDs (Year	Turkmenist	Both	55+ years	Non-melanoma skin cancer (basal-cell	Percent	2021	2.63E-06	4.81E-06	1.20E-06
YLDs (Year	Turkmenist	Male	55+ years	Non-melanoma skin cancer (basal-cell	Rate	2021	0.050498	0.097320002	0.020480201
YLDs (Year	Turkmenist	Female	55+ years	Non-melanoma skin cancer (basal-cell	Rate	2021	0.0525726	0.104020895	0.021124244
YLDs (Year	Turkmenist	Both	55+ years	Non-melanoma skin cancer (basal-cell	Rate	2021	0.0516685	0.098974991	0.021090988
YLDs (Year	Ukraine	Male	55+ years	Non-melanoma skin cancer (basal-cell	Number	1990	2.1657802	4.185834928	0.937524377
YLDs (Year	Ukraine	Female	55+ years	Non-melanoma skin cancer (basal-cell	Number	1990	4.391954	8.468800548	1.829666403
YLDs (Year	Ukraine	Both	55+ years	Non-melanoma skin cancer (basal-cell	Number	1990	6.5577342	12.56302799	2.746455004
YLDs (Year	Ukraine	Male	55+ years	Non-melanoma skin cancer (basal-cell	Percent	1990	2.21E-06	3.88E-06	1.04E-06
YLDs (Year	Ukraine	Female	55+ years	Non-melanoma skin cancer (basal-cell	Percent	1990	2.28E-06	4.21E-06	1.04E-06
YLDs (Year	Ukraine	Both	55+ years	Non-melanoma skin cancer (basal-cell	Percent	1990	2.25E-06	4.00E-06	1.03E-06
YLDs (Year	Ukraine	Male	55+ years	Non-melanoma skin cancer (basal-cell	Rate	1990	0.0481446	0.093049781	0.020840869
YLDs (Year	Ukraine	Female	55+ years	Non-melanoma skin cancer (basal-cell	Rate	1990	0.0551165	0.106278503	0.022961245
YLDs (Year	Ukraine	Both	55+ years	Non-melanoma skin cancer (basal-cell	Rate	1990	0.0526008	0.100770354	0.02202982

YLDs (Year	Ukraine	Male	55+ years	Non-melanoma skin cancer (basal-cell	Number	2021	2.752782	5.407169172	1.160546082
YLDs (Year	Ukraine	Female	55+ years	Non-melanoma skin cancer (basal-cell	Number	2021	4.7365095	9.330920769	1.87192571
YLDs (Year	Ukraine	Both	55+ years	Non-melanoma skin cancer (basal-cell	Number	2021	7.4892915	14.33794692	3.131354378
YLDs (Year	Ukraine	Male	55+ years	Non-melanoma skin cancer (basal-cell	Percent	2021	2.38E-06	4.50E-06	1.07E-06
YLDs (Year	Ukraine	Female	55+ years	Non-melanoma skin cancer (basal-cell	Percent	2021	2.33E-06	4.20E-06	1.07E-06
YLDs (Year	Ukraine	Both	55+ years	Non-melanoma skin cancer (basal-cell	Percent	2021	2.35E-06	4.29E-06	1.04E-06
YLDs (Year	Ukraine	Male	55+ years	Non-melanoma skin cancer (basal-cell	Rate	2021	0.0528183	0.103748679	0.022267682
YLDs (Year	Ukraine	Female	55+ years	Non-melanoma skin cancer (basal-cell	Rate	2021	0.0566143	0.111530181	0.022374664
YLDs (Year	Ukraine	Both	55+ years	Non-melanoma skin cancer (basal-cell	Rate	2021	0.0551573	0.105596355	0.023061852
YLDs (Year	Croatia	Male	55+ years	Non-melanoma skin cancer (basal-cell	Number	1990	0.2505136	0.475467975	0.102308363
YLDs (Year	Croatia	Female	55+ years	Non-melanoma skin cancer (basal-cell	Number	1990	0.36271	0.718999951	0.149210021
YLDs (Year	Croatia	Both	55+ years	Non-melanoma skin cancer (basal-cell	Number	1990	0.6132236	1.193263911	0.246172873
YLDs (Year	Croatia	Male	55+ years	Non-melanoma skin cancer (basal-cell	Percent	1990	2.63E-06	4.82E-06	1.19E-06
YLDs (Year	Croatia	Female	55+ years	Non-melanoma skin cancer (basal-cell	Percent	1990	2.48E-06	4.64E-06	1.10E-06
YLDs (Year	Croatia	Both	55+ years	Non-melanoma skin cancer (basal-cell	Percent	1990	2.54E-06	4.70E-06	1.13E-06
YLDs (Year	Croatia	Male	55+ years	Non-melanoma skin cancer (basal-cell	Rate	1990	0.054571	0.10357422	0.022286483
YLDs (Year	Croatia	Female	55+ years	Non-melanoma skin cancer (basal-cell	Rate	1990	0.0554499	0.109918383	0.022810745
YLDs (Year	Croatia	Both	55+ years	Non-melanoma skin cancer (basal-cell	Rate	1990	0.0550875	0.107193981	0.022114345
YLDs (Year	Croatia	Male	55+ years	Non-melanoma skin cancer (basal-cell	Number	2021	0.4531533	0.902129688	0.196224234
YLDs (Year	Croatia	Female	55+ years	Non-melanoma skin cancer (basal-cell	Number	2021	0.5401746	1.080574619	0.224490941
YLDs (Year	Croatia	Both	55+ years	Non-melanoma skin cancer (basal-cell	Number	2021	0.9933279	1.948135284	0.436548101
YLDs (Year	Croatia	Male	55+ years	Non-melanoma skin cancer (basal-cell	Percent	2021	3.11E-06	6.04E-06	1.40E-06
YLDs (Year	Croatia	Female	55+ years	Non-melanoma skin cancer (basal-cell	Percent	2021	2.62E-06	4.84E-06	1.17E-06
YLDs (Year	Croatia	Both	55+ years	Non-melanoma skin cancer (basal-cell	Percent	2021	2.83E-06	5.35E-06	1.35E-06
YLDs (Year	Croatia	Male	55+ years	Non-melanoma skin cancer (basal-cell	Rate	2021	0.0689414	0.137247402	0.029852988
YLDs (Year	Croatia	Female	55+ years	Non-melanoma skin cancer (basal-cell	Rate	2021	0.0647672	0.1295615	0.026916589
YLDs (Year	Croatia	Both	55+ years	Non-melanoma skin cancer (basal-cell	Rate	2021	0.066607	0.130631055	0.029272474
YLDs (Year	Hungary	Male	55+ years	Non-melanoma skin cancer (basal-cell	Number	1990	0.7909398	1.588984201	0.350028527
YLDs (Year	Hungary	Female	55+ years	Non-melanoma skin cancer (basal-cell	Number	1990	1.0624318	2.133041408	0.465798881
YLDs (Year	Hungary	Both	55+ years	Non-melanoma skin cancer (basal-cell	Number	1990	1.8533716	3.532019241	0.814493219
YLDs (Year	Hungary	Male	55+ years	Non-melanoma skin cancer (basal-cell	Percent	1990	3.19E-06	5.89E-06	1.51E-06
YLDs (Year	Hungary	Female	55+ years	Non-melanoma skin cancer (basal-cell	Percent	1990	2.86E-06	5.56E-06	1.32E-06
YLDs (Year	Hungary	Both	55+ years	Non-melanoma skin cancer (basal-cell	Percent	1990	2.99E-06	5.52E-06	1.40E-06
YLDs (Year	Hungary	Male	55+ years	Non-melanoma skin cancer (basal-cell	Rate	1990	0.0738255	0.148314162	0.032671305
YLDs (Year	Hungary	Female	55+ years	Non-melanoma skin cancer (basal-cell	Rate	1990	0.070624	0.141791562	0.030963464
YLDs (Year	Hungary	Both	55+ years	Non-melanoma skin cancer (basal-cell	Rate	1990	0.0719556	0.137127783	0.031622039
YLDs (Year	Hungary	Male	55+ years	Non-melanoma skin cancer (basal-cell	Number	2021	1.0267126	1.95699347	0.44637863
YLDs (Year	Hungary	Female	55+ years	Non-melanoma skin cancer (basal-cell	Number	2021	1.3971026	2.917268069	0.572914621
YLDs (Year	Hungary	Both	55+ years	Non-melanoma skin cancer (basal-cell	Number	2021	2.4238152	4.875526467	1.052496493
YLDs (Year	Hungary	Male	55+ years	Non-melanoma skin cancer (basal-cell	Percent	2021	3.49E-06	6.23E-06	1.67E-06
YLDs (Year	Hungary	Female	55+ years	Non-melanoma skin cancer (basal-cell	Percent	2021	3.02E-06	5.94E-06	1.26E-06
YLDs (Year	Hungary	Both	55+ years	Non-melanoma skin cancer (basal-cell	Percent	2021	3.20E-06	5.98E-06	1.50E-06
YLDs (Year	Hungary	Male	55+ years	Non-melanoma skin cancer (basal-cell	Rate	2021	0.0778618	0.148410668	0.033851595
YLDs (Year	Hungary	Female	55+ years	Non-melanoma skin cancer (basal-cell	Rate	2021	0.0750529	0.156716849	0.030777211
YLDs (Year	Hungary	Both	55+ years	Non-melanoma skin cancer (basal-cell	Rate	2021	0.0762176	0.153312479	0.033096087
YLDs (Year	Serbia	Male	55+ years	Non-melanoma skin cancer (basal-cell	Number	1990	0.5151461	1.020370666	0.236534792
YLDs (Year	Serbia	Female	55+ years	Non-melanoma skin cancer (basal-cell	Number	1990	0.5066949	1.043027014	0.229301637
YLDs (Year	Serbia	Both	55+ years	Non-melanoma skin cancer (basal-cell	Number	1990	1.021841	2.055276509	0.467348612
YLDs (Year	Serbia	Male	55+ years	Non-melanoma skin cancer (basal-cell	Percent	1990	2.67E-06	4.90E-06	1.30E-06
YLDs (Year	Serbia	Female	55+ years	Non-melanoma skin cancer (basal-cell	Percent	1990	2.10E-06	3.87E-06	9.80E-07
YLDs (Year	Serbia	Both	55+ years	Non-melanoma skin cancer (basal-cell	Percent	1990	2.35E-06	4.32E-06	1.13E-06
YLDs (Year	Serbia	Male	55+ years	Non-melanoma skin cancer (basal-cell	Rate	1990	0.0538711	0.106704591	0.02473547
YLDs (Year	Serbia	Female	55+ years	Non-melanoma skin cancer (basal-cell	Rate	1990	0.0450995	0.092837026	0.020409521
YLDs (Year	Serbia	Both	55+ years	Non-melanoma skin cancer (basal-cell	Rate	1990	0.0491326	0.098822737	0.022471268
YLDs (Year	Serbia	Male	55+ years	Non-melanoma skin cancer (basal-cell	Number	2021	0.9213838	1.81770498	0.367938921
YLDs (Year	Serbia	Female	55+ years	Non-melanoma skin cancer (basal-cell	Number	2021	0.954744	1.980119545	0.383096662

YLDs (Year	Serbia	Both	55+ years	Non-melanoma skin cancer (basal-cell	Number	2021	1.8761278	3.686243607	0.755607834
YLDs (Year	Serbia	Male	55+ years	Non-melanoma skin cancer (basal-cell	Percent	2021	3.32E-06	6.15E-06	1.48E-06
YLDs (Year	Serbia	Female	55+ years	Non-melanoma skin cancer (basal-cell	Percent	2021	2.59E-06	4.89E-06	1.17E-06
YLDs (Year	Serbia	Both	55+ years	Non-melanoma skin cancer (basal-cell	Percent	2021	2.91E-06	5.54E-06	1.30E-06
YLDs (Year	Serbia	Male	55+ years	Non-melanoma skin cancer (basal-cell	Rate	2021	0.0724633	0.142955557	0.028936992
YLDs (Year	Serbia	Female	55+ years	Non-melanoma skin cancer (basal-cell	Rate	2021	0.0622509	0.129107168	0.024978555
YLDs (Year	Serbia	Both	55+ years	Non-melanoma skin cancer (basal-cell	Rate	2021	0.0668799	0.131406588	0.02693578
YLDs (Year	Latvia	Male	55+ years	Non-melanoma skin cancer (basal-cell	Number	1990	0.10757	0.210951319	0.047575077
YLDs (Year	Latvia	Female	55+ years	Non-melanoma skin cancer (basal-cell	Number	1990	0.2052759	0.407301551	0.091238017
YLDs (Year	Latvia	Both	55+ years	Non-melanoma skin cancer (basal-cell	Number	1990	0.3128458	0.619759131	0.140155288
YLDs (Year	Latvia	Male	55+ years	Non-melanoma skin cancer (basal-cell	Percent	1990	2.11E-06	3.68E-06	1.03E-06
YLDs (Year	Latvia	Female	55+ years	Non-melanoma skin cancer (basal-cell	Percent	1990	2.19E-06	3.93E-06	1.06E-06
YLDs (Year	Latvia	Both	55+ years	Non-melanoma skin cancer (basal-cell	Percent	1990	2.16E-06	3.82E-06	1.07E-06
YLDs (Year	Latvia	Male	55+ years	Non-melanoma skin cancer (basal-cell	Rate	1990	0.0476207	0.093387171	0.021061266
YLDs (Year	Latvia	Female	55+ years	Non-melanoma skin cancer (basal-cell	Rate	1990	0.0514026	0.101991402	0.022846693
YLDs (Year	Latvia	Both	55+ years	Non-melanoma skin cancer (basal-cell	Rate	1990	0.0500363	0.099123734	0.022416314
YLDs (Year	Latvia	Male	55+ years	Non-melanoma skin cancer (basal-cell	Number	2021	0.1359252	0.266262885	0.05549578
YLDs (Year	Latvia	Female	55+ years	Non-melanoma skin cancer (basal-cell	Number	2021	0.2481118	0.483311256	0.107617805
YLDs (Year	Latvia	Both	55+ years	Non-melanoma skin cancer (basal-cell	Number	2021	0.384037	0.752840124	0.16380011
YLDs (Year	Latvia	Male	55+ years	Non-melanoma skin cancer (basal-cell	Percent	2021	2.43E-06	4.57E-06	1.07E-06
YLDs (Year	Latvia	Female	55+ years	Non-melanoma skin cancer (basal-cell	Percent	2021	2.47E-06	4.68E-06	1.13E-06
YLDs (Year	Latvia	Both	55+ years	Non-melanoma skin cancer (basal-cell	Percent	2021	2.46E-06	4.49E-06	1.12E-06
YLDs (Year	Latvia	Male	55+ years	Non-melanoma skin cancer (basal-cell	Rate	2021	0.0544813	0.106723016	0.02224372
YLDs (Year	Latvia	Female	55+ years	Non-melanoma skin cancer (basal-cell	Rate	2021	0.0607329	0.11830501	0.026342704
YLDs (Year	Latvia	Both	55+ years	Non-melanoma skin cancer (basal-cell	Rate	2021	0.0583626	0.114410004	0.024892897
YLDs (Year	Solomon Is	Male	55+ years	Non-melanoma skin cancer (basal-cell	Number	1990	1.08E-05	2.78E-05	2.00E-06
YLDs (Year	Solomon Is	Female	55+ years	Non-melanoma skin cancer (basal-cell	Number	1990	8.34E-06	2.26E-05	1.58E-06
YLDs (Year	Solomon Is	Both	55+ years	Non-melanoma skin cancer (basal-cell	Number	1990	1.91E-05	4.94E-05	3.86E-06
YLDs (Year	Solomon Is	Male	55+ years	Non-melanoma skin cancer (basal-cell	Percent	1990	4.66E-09	1.16E-08	9.26E-10
YLDs (Year	Solomon Is	Female	55+ years	Non-melanoma skin cancer (basal-cell	Percent	1990	4.02E-09	1.02E-08	8.30E-10
YLDs (Year	Solomon Is	Both	55+ years	Non-melanoma skin cancer (basal-cell	Percent	1990	4.36E-09	1.10E-08	9.53E-10
YLDs (Year	Solomon Is	Male	55+ years	Non-melanoma skin cancer (basal-cell	Rate	1990	8.48E-05	0.000218342	1.57E-05
YLDs (Year	Solomon Is	Female	55+ years	Non-melanoma skin cancer (basal-cell	Rate	1990	8.45E-05	0.000229079	1.60E-05
YLDs (Year	Solomon Is	Both	55+ years	Non-melanoma skin cancer (basal-cell	Rate	1990	8.47E-05	0.00021831	1.71E-05
YLDs (Year	Solomon Is	Male	55+ years	Non-melanoma skin cancer (basal-cell	Number	2021	2.33E-05	5.89E-05	4.46E-06
YLDs (Year	Solomon Is	Female	55+ years	Non-melanoma skin cancer (basal-cell	Number	2021	2.23E-05	5.80E-05	4.72E-06
YLDs (Year	Solomon Is	Both	55+ years	Non-melanoma skin cancer (basal-cell	Number	2021	4.57E-05	0.000115058	1.04E-05
YLDs (Year	Solomon Is	Male	55+ years	Non-melanoma skin cancer (basal-cell	Percent	2021	4.46E-09	1.10E-08	8.53E-10
YLDs (Year	Solomon Is	Female	55+ years	Non-melanoma skin cancer (basal-cell	Percent	2021	3.68E-09	9.21E-09	7.96E-10
YLDs (Year	Solomon Is	Both	55+ years	Non-melanoma skin cancer (basal-cell	Percent	2021	4.04E-09	1.01E-08	1.01E-09
YLDs (Year	Solomon Is	Male	55+ years	Non-melanoma skin cancer (basal-cell	Rate	2021	8.49E-05	0.000214655	1.63E-05
YLDs (Year	Solomon Is	Female	55+ years	Non-melanoma skin cancer (basal-cell	Rate	2021	8.34E-05	0.000216548	1.76E-05
YLDs (Year	Solomon Is	Both	55+ years	Non-melanoma skin cancer (basal-cell	Rate	2021	8.42E-05	0.000212105	1.91E-05
YLDs (Year	Germany	Male	55+ years	Non-melanoma skin cancer (basal-cell	Number	1990	5.7956883	12.10765881	2.446723115
YLDs (Year	Germany	Female	55+ years	Non-melanoma skin cancer (basal-cell	Number	1990	7.2528044	13.79399518	3.192260719
YLDs (Year	Germany	Both	55+ years	Non-melanoma skin cancer (basal-cell	Number	1990	13.048493	25.78695851	5.561828266
YLDs (Year	Germany	Male	55+ years	Non-melanoma skin cancer (basal-cell	Percent	1990	3.52E-06	6.44E-06	1.54E-06
YLDs (Year	Germany	Female	55+ years	Non-melanoma skin cancer (basal-cell	Percent	1990	2.46E-06	4.44E-06	1.17E-06
YLDs (Year	Germany	Both	55+ years	Non-melanoma skin cancer (basal-cell	Percent	1990	2.84E-06	5.11E-06	1.30E-06
YLDs (Year	Germany	Male	55+ years	Non-melanoma skin cancer (basal-cell	Rate	1990	0.0689643	0.144071977	0.029114153
YLDs (Year	Germany	Female	55+ years	Non-melanoma skin cancer (basal-cell	Rate	1990	0.0576567	0.10965631	0.025377095
YLDs (Year	Germany	Both	55+ years	Non-melanoma skin cancer (basal-cell	Rate	1990	0.0621854	0.122893385	0.026506108
YLDs (Year	Germany	Male	55+ years	Non-melanoma skin cancer (basal-cell	Number	2021	14.063429	28.1459329	5.924462428
YLDs (Year	Germany	Female	55+ years	Non-melanoma skin cancer (basal-cell	Number	2021	12.693435	25.05547158	4.904495877
YLDs (Year	Germany	Both	55+ years	Non-melanoma skin cancer (basal-cell	Number	2021	26.756864	51.39391353	10.86864115
YLDs (Year	Germany	Male	55+ years	Non-melanoma skin cancer (basal-cell	Percent	2021	4.58E-06	8.61E-06	2.04E-06

YLDs (Year	Germany	Female	55+ years	Non-melanoma skin cancer (basal-cell	Percent	2021	3.05E-06	5.77E-06	1.32E-06
YLDs (Year	Germany	Both	55+ years	Non-melanoma skin cancer (basal-cell	Percent	2021	3.70E-06	6.82E-06	1.59E-06
YLDs (Year	Germany	Male	55+ years	Non-melanoma skin cancer (basal-cell	Rate	2021	0.0960617	0.192253771	0.040467667
YLDs (Year	Germany	Female	55+ years	Non-melanoma skin cancer (basal-cell	Rate	2021	0.0752252	0.148486444	0.029065554
YLDs (Year	Germany	Both	55+ years	Non-melanoma skin cancer (basal-cell	Rate	2021	0.084905	0.163083308	0.034488402
YLDs (Year	Argentina	Male	55+ years	Non-melanoma skin cancer (basal-cell	Number	1990	1.6484271	3.206269484	0.748682045
YLDs (Year	Argentina	Female	55+ years	Non-melanoma skin cancer (basal-cell	Number	1990	1.7472412	3.561135306	0.739441251
YLDs (Year	Argentina	Both	55+ years	Non-melanoma skin cancer (basal-cell	Number	1990	3.3956683	6.757142807	1.494578185
YLDs (Year	Argentina	Male	55+ years	Non-melanoma skin cancer (basal-cell	Percent	1990	3.46E-06	6.13E-06	1.71E-06
YLDs (Year	Argentina	Female	55+ years	Non-melanoma skin cancer (basal-cell	Percent	1990	2.63E-06	4.91E-06	1.20E-06
YLDs (Year	Argentina	Both	55+ years	Non-melanoma skin cancer (basal-cell	Percent	1990	2.97E-06	5.48E-06	1.49E-06
YLDs (Year	Argentina	Male	55+ years	Non-melanoma skin cancer (basal-cell	Rate	1990	0.0664807	0.129308149	0.030194184
YLDs (Year	Argentina	Female	55+ years	Non-melanoma skin cancer (basal-cell	Rate	1990	0.0566357	0.115431992	0.023968529
YLDs (Year	Argentina	Both	55+ years	Non-melanoma skin cancer (basal-cell	Rate	1990	0.0610226	0.121430712	0.026858644
YLDs (Year	Argentina	Male	55+ years	Non-melanoma skin cancer (basal-cell	Number	2021	2.9249028	5.942949495	1.162899128
YLDs (Year	Argentina	Female	55+ years	Non-melanoma skin cancer (basal-cell	Number	2021	3.0130829	6.148006943	1.269955905
YLDs (Year	Argentina	Both	55+ years	Non-melanoma skin cancer (basal-cell	Number	2021	5.9379857	11.42427546	2.441542529
YLDs (Year	Argentina	Male	55+ years	Non-melanoma skin cancer (basal-cell	Percent	2021	3.51E-06	6.35E-06	1.52E-06
YLDs (Year	Argentina	Female	55+ years	Non-melanoma skin cancer (basal-cell	Percent	2021	2.54E-06	4.68E-06	1.13E-06
YLDs (Year	Argentina	Both	55+ years	Non-melanoma skin cancer (basal-cell	Percent	2021	2.94E-06	5.31E-06	1.31E-06
YLDs (Year	Argentina	Male	55+ years	Non-melanoma skin cancer (basal-cell	Rate	2021	0.0703414	0.14292286	0.027966731
YLDs (Year	Argentina	Female	55+ years	Non-melanoma skin cancer (basal-cell	Rate	2021	0.058222	0.118798442	0.024539462
YLDs (Year	Argentina	Both	55+ years	Non-melanoma skin cancer (basal-cell	Rate	2021	0.0636214	0.122403261	0.02615945
YLDs (Year	United Sta	Male	55+ years	Non-melanoma skin cancer (basal-cell	Number	1990	120.33999	225.2976703	52.22223298
YLDs (Year	United Sta	Female	55+ years	Non-melanoma skin cancer (basal-cell	Number	1990	118.81005	227.6163312	50.16765616
YLDs (Year	United Sta	Both	55+ years	Non-melanoma skin cancer (basal-cell	Number	1990	239.15004	451.9544584	102.4319172
YLDs (Year	United Sta	Male	55+ years	Non-melanoma skin cancer (basal-cell	Percent	1990	2.37E-05	4.15E-05	1.17E-05
YLDs (Year	United Sta	Female	55+ years	Non-melanoma skin cancer (basal-cell	Percent	1990	1.65E-05	2.95E-05	7.81E-06
YLDs (Year	United Sta	Both	55+ years	Non-melanoma skin cancer (basal-cell	Percent	1990	1.95E-05	3.51E-05	9.59E-06
YLDs (Year	United Sta	Male	55+ years	Non-melanoma skin cancer (basal-cell	Rate	1990	0.5320318	0.996057197	0.230878246
YLDs (Year	United Sta	Female	55+ years	Non-melanoma skin cancer (basal-cell	Rate	1990	0.3981133	0.762705574	0.168103715
YLDs (Year	United Sta	Both	55+ years	Non-melanoma skin cancer (basal-cell	Rate	1990	0.4558519	0.861485489	0.195248899
YLDs (Year	United Sta	Male	55+ years	Non-melanoma skin cancer (basal-cell	Number	2021	605.71912	1143.732644	286.0231237
YLDs (Year	United Sta	Female	55+ years	Non-melanoma skin cancer (basal-cell	Number	2021	405.25907	780.3290242	190.4020206
YLDs (Year	United Sta	Both	55+ years	Non-melanoma skin cancer (basal-cell	Number	2021	1010.9782	1935.716845	478.2465149
YLDs (Year	United Sta	Male	55+ years	Non-melanoma skin cancer (basal-cell	Percent	2021	5.25E-05	9.23E-05	2.67E-05
YLDs (Year	United Sta	Female	55+ years	Non-melanoma skin cancer (basal-cell	Percent	2021	2.84E-05	5.10E-05	1.43E-05
YLDs (Year	United Sta	Both	55+ years	Non-melanoma skin cancer (basal-cell	Percent	2021	3.92E-05	7.00E-05	1.98E-05
YLDs (Year	United Sta	Male	55+ years	Non-melanoma skin cancer (basal-cell	Rate	2021	1.3031405	2.460619556	0.615348434
YLDs (Year	United Sta	Female	55+ years	Non-melanoma skin cancer (basal-cell	Rate	2021	0.7537422	1.451335646	0.354129131
YLDs (Year	United Sta	Both	55+ years	Non-melanoma skin cancer (basal-cell	Rate	2021	1.0084796	1.930932781	0.477064543
YLDs (Year	Slovakia	Male	55+ years	Non-melanoma skin cancer (basal-cell	Number	1990	0.3875976	0.75951109	0.165724514
YLDs (Year	Slovakia	Female	55+ years	Non-melanoma skin cancer (basal-cell	Number	1990	0.4787129	0.974522591	0.200184674
YLDs (Year	Slovakia	Both	55+ years	Non-melanoma skin cancer (basal-cell	Number	1990	0.8663105	1.707041708	0.371181542
YLDs (Year	Slovakia	Male	55+ years	Non-melanoma skin cancer (basal-cell	Percent	1990	3.88E-06	7.12E-06	1.82E-06
YLDs (Year	Slovakia	Female	55+ years	Non-melanoma skin cancer (basal-cell	Percent	1990	3.40E-06	6.57E-06	1.48E-06
YLDs (Year	Slovakia	Both	55+ years	Non-melanoma skin cancer (basal-cell	Percent	1990	3.60E-06	6.51E-06	1.78E-06
YLDs (Year	Slovakia	Male	55+ years	Non-melanoma skin cancer (basal-cell	Rate	1990	0.0878315	0.172108883	0.037553976
YLDs (Year	Slovakia	Female	55+ years	Non-melanoma skin cancer (basal-cell	Rate	1990	0.0802996	0.16346708	0.033579113
YLDs (Year	Slovakia	Both	55+ years	Non-melanoma skin cancer (basal-cell	Rate	1990	0.0835034	0.16454125	0.03577808
YLDs (Year	Slovakia	Male	55+ years	Non-melanoma skin cancer (basal-cell	Number	2021	0.5564861	1.145453372	0.204158407
YLDs (Year	Slovakia	Female	55+ years	Non-melanoma skin cancer (basal-cell	Number	2021	0.6992496	1.385792414	0.272652799
YLDs (Year	Slovakia	Both	55+ years	Non-melanoma skin cancer (basal-cell	Number	2021	1.2557358	2.491142288	0.47631227
YLDs (Year	Slovakia	Male	55+ years	Non-melanoma skin cancer (basal-cell	Percent	2021	3.57E-06	6.86E-06	1.43E-06
YLDs (Year	Slovakia	Female	55+ years	Non-melanoma skin cancer (basal-cell	Percent	2021	3.16E-06	6.03E-06	1.28E-06
YLDs (Year	Slovakia	Both	55+ years	Non-melanoma skin cancer (basal-cell	Percent	2021	3.33E-06	6.24E-06	1.35E-06

YLDs (Year	Slovakia	Male	55+ years	Non-melanoma skin cancer (basal-cell	Rate	2021	0.0779619	0.160474193	0.028601911
YLDs (Year	Slovakia	Female	55+ years	Non-melanoma skin cancer (basal-cell	Rate	2021	0.0754735	0.149575462	0.029428772
YLDs (Year	Slovakia	Both	55+ years	Non-melanoma skin cancer (basal-cell	Rate	2021	0.0765563	0.151873298	0.029038532
YLDs (Year	Austria	Male	55+ years	Non-melanoma skin cancer (basal-cell	Number	1990	0.6463044	1.264918577	0.275288612
YLDs (Year	Austria	Female	55+ years	Non-melanoma skin cancer (basal-cell	Number	1990	0.9052201	1.876788621	0.378378871
YLDs (Year	Austria	Both	55+ years	Non-melanoma skin cancer (basal-cell	Number	1990	1.5515245	3.067762245	0.676699214
YLDs (Year	Austria	Male	55+ years	Non-melanoma skin cancer (basal-cell	Percent	1990	4.15E-06	7.42E-06	1.82E-06
YLDs (Year	Austria	Female	55+ years	Non-melanoma skin cancer (basal-cell	Percent	1990	3.34E-06	6.31E-06	1.57E-06
YLDs (Year	Austria	Both	55+ years	Non-melanoma skin cancer (basal-cell	Percent	1990	3.64E-06	6.69E-06	1.71E-06
YLDs (Year	Austria	Male	55+ years	Non-melanoma skin cancer (basal-cell	Rate	1990	0.0839023	0.164209967	0.035737584
YLDs (Year	Austria	Female	55+ years	Non-melanoma skin cancer (basal-cell	Rate	1990	0.0770002	0.159644065	0.032185799
YLDs (Year	Austria	Both	55+ years	Non-melanoma skin cancer (basal-cell	Rate	1990	0.0797324	0.157651502	0.034775396
YLDs (Year	Austria	Male	55+ years	Non-melanoma skin cancer (basal-cell	Number	2021	1.2863323	2.517561748	0.538614568
YLDs (Year	Austria	Female	55+ years	Non-melanoma skin cancer (basal-cell	Number	2021	1.2332433	2.403344935	0.492412854
YLDs (Year	Austria	Both	55+ years	Non-melanoma skin cancer (basal-cell	Number	2021	2.5195756	4.972891171	1.06129306
YLDs (Year	Austria	Male	55+ years	Non-melanoma skin cancer (basal-cell	Percent	2021	4.64E-06	8.56E-06	2.01E-06
YLDs (Year	Austria	Female	55+ years	Non-melanoma skin cancer (basal-cell	Percent	2021	3.33E-06	6.22E-06	1.45E-06
YLDs (Year	Austria	Both	55+ years	Non-melanoma skin cancer (basal-cell	Percent	2021	3.89E-06	7.32E-06	1.76E-06
YLDs (Year	Austria	Male	55+ years	Non-melanoma skin cancer (basal-cell	Rate	2021	0.0948776	0.185690918	0.039727261
YLDs (Year	Austria	Female	55+ years	Non-melanoma skin cancer (basal-cell	Rate	2021	0.077107	0.150266119	0.030787494
YLDs (Year	Austria	Both	55+ years	Non-melanoma skin cancer (basal-cell	Rate	2021	0.0852598	0.168277486	0.035913058
YLDs (Year	Cyprus	Male	55+ years	Non-melanoma skin cancer (basal-cell	Number	1990	0.0488646	0.100544716	0.019673335
YLDs (Year	Cyprus	Female	55+ years	Non-melanoma skin cancer (basal-cell	Number	1990	0.0470103	0.092375526	0.020961642
YLDs (Year	Cyprus	Both	55+ years	Non-melanoma skin cancer (basal-cell	Number	1990	0.0958749	0.196364949	0.040537153
YLDs (Year	Cyprus	Male	55+ years	Non-melanoma skin cancer (basal-cell	Percent	1990	3.89E-06	7.14E-06	1.69E-06
YLDs (Year	Cyprus	Female	55+ years	Non-melanoma skin cancer (basal-cell	Percent	1990	2.87E-06	5.19E-06	1.37E-06
YLDs (Year	Cyprus	Both	55+ years	Non-melanoma skin cancer (basal-cell	Percent	1990	3.32E-06	6.11E-06	1.47E-06
YLDs (Year	Cyprus	Male	55+ years	Non-melanoma skin cancer (basal-cell	Rate	1990	0.0756245	0.155606363	0.030447111
YLDs (Year	Cyprus	Female	55+ years	Non-melanoma skin cancer (basal-cell	Rate	1990	0.0626177	0.123044325	0.027920936
YLDs (Year	Cyprus	Both	55+ years	Non-melanoma skin cancer (basal-cell	Rate	1990	0.0686341	0.140572161	0.029019411
YLDs (Year	Cyprus	Male	55+ years	Non-melanoma skin cancer (basal-cell	Number	2021	0.1344968	0.271281636	0.052193039
YLDs (Year	Cyprus	Female	55+ years	Non-melanoma skin cancer (basal-cell	Number	2021	0.1134947	0.220472002	0.045946239
YLDs (Year	Cyprus	Both	55+ years	Non-melanoma skin cancer (basal-cell	Number	2021	0.2479915	0.481334974	0.10213881
YLDs (Year	Cyprus	Male	55+ years	Non-melanoma skin cancer (basal-cell	Percent	2021	4.10E-06	7.85E-06	1.71E-06
YLDs (Year	Cyprus	Female	55+ years	Non-melanoma skin cancer (basal-cell	Percent	2021	2.84E-06	5.24E-06	1.26E-06
YLDs (Year	Cyprus	Both	55+ years	Non-melanoma skin cancer (basal-cell	Percent	2021	3.41E-06	6.13E-06	1.54E-06
YLDs (Year	Cyprus	Male	55+ years	Non-melanoma skin cancer (basal-cell	Rate	2021	0.0798799	0.161118701	0.030998319
YLDs (Year	Cyprus	Female	55+ years	Non-melanoma skin cancer (basal-cell	Rate	2021	0.06266	0.121721735	0.02536674
YLDs (Year	Cyprus	Both	55+ years	Non-melanoma skin cancer (basal-cell	Rate	2021	0.0709558	0.13772037	0.029224128
YLDs (Year	Brunei Dar	Male	55+ years	Non-melanoma skin cancer (basal-cell	Number	1990	0.0010562	0.002057551	0.000413638
YLDs (Year	Brunei Dar	Female	55+ years	Non-melanoma skin cancer (basal-cell	Number	1990	0.0008362	0.001585843	0.000350187
YLDs (Year	Brunei Dar	Both	55+ years	Non-melanoma skin cancer (basal-cell	Number	1990	0.0018923	0.003668446	0.000753682
YLDs (Year	Brunei Dar	Male	55+ years	Non-melanoma skin cancer (basal-cell	Percent	1990	6.34E-07	1.15E-06	2.85E-07
YLDs (Year	Brunei Dar	Female	55+ years	Non-melanoma skin cancer (basal-cell	Percent	1990	5.32E-07	9.77E-07	2.32E-07
YLDs (Year	Brunei Dar	Both	55+ years	Non-melanoma skin cancer (basal-cell	Percent	1990	5.84E-07	1.07E-06	2.60E-07
YLDs (Year	Brunei Dar	Male	55+ years	Non-melanoma skin cancer (basal-cell	Rate	1990	0.0129848	0.025296492	0.005085457
YLDs (Year	Brunei Dar	Female	55+ years	Non-melanoma skin cancer (basal-cell	Rate	1990	0.0111151	0.021080257	0.004654963
YLDs (Year	Brunei Dar	Both	55+ years	Non-melanoma skin cancer (basal-cell	Rate	1990	0.0120865	0.023430635	0.004813823
YLDs (Year	Brunei Dar	Male	55+ years	Non-melanoma skin cancer (basal-cell	Number	2021	0.0033997	0.006767371	0.001370288
YLDs (Year	Brunei Dar	Female	55+ years	Non-melanoma skin cancer (basal-cell	Number	2021	0.002976	0.005900643	0.001192218
YLDs (Year	Brunei Dar	Both	55+ years	Non-melanoma skin cancer (basal-cell	Number	2021	0.0063758	0.012277793	0.002501191
YLDs (Year	Brunei Dar	Male	55+ years	Non-melanoma skin cancer (basal-cell	Percent	2021	5.74E-07	1.06E-06	2.58E-07
YLDs (Year	Brunei Dar	Female	55+ years	Non-melanoma skin cancer (basal-cell	Percent	2021	4.71E-07	8.68E-07	2.11E-07
YLDs (Year	Brunei Dar	Both	55+ years	Non-melanoma skin cancer (basal-cell	Percent	2021	5.21E-07	9.58E-07	2.35E-07
YLDs (Year	Brunei Dar	Male	55+ years	Non-melanoma skin cancer (basal-cell	Rate	2021	0.0115551	0.023000994	0.004657347
YLDs (Year	Brunei Dar	Female	55+ years	Non-melanoma skin cancer (basal-cell	Rate	2021	0.0097727	0.019376537	0.003915005

YLDs (Year	Brunei Dar	Both	55+ years	Non-melanoma skin cancer (basal-cell	Rate	2021	0.0106486	0.020505848	0.004177383
YLDs (Year	Montenegro	Male	55+ years	Non-melanoma skin cancer (basal-cell	Number	1990	0.033448	0.065956123	0.013623764
YLDs (Year	Montenegro	Female	55+ years	Non-melanoma skin cancer (basal-cell	Number	1990	0.0385283	0.075113774	0.015589849
YLDs (Year	Montenegro	Both	55+ years	Non-melanoma skin cancer (basal-cell	Number	1990	0.0719763	0.139128931	0.028737953
YLDs (Year	Montenegro	Male	55+ years	Non-melanoma skin cancer (basal-cell	Percent	1990	3.45E-06	6.19E-06	1.52E-06
YLDs (Year	Montenegro	Female	55+ years	Non-melanoma skin cancer (basal-cell	Percent	1990	2.97E-06	5.69E-06	1.40E-06
YLDs (Year	Montenegro	Both	55+ years	Non-melanoma skin cancer (basal-cell	Percent	1990	3.18E-06	5.75E-06	1.47E-06
YLDs (Year	Montenegro	Male	55+ years	Non-melanoma skin cancer (basal-cell	Rate	1990	0.0691439	0.136344924	0.028163133
YLDs (Year	Montenegro	Female	55+ years	Non-melanoma skin cancer (basal-cell	Rate	1990	0.0636123	0.124016973	0.025739699
YLDs (Year	Montenegro	Both	55+ years	Non-melanoma skin cancer (basal-cell	Rate	1990	0.0660685	0.127709416	0.02637918
YLDs (Year	Montenegro	Male	55+ years	Non-melanoma skin cancer (basal-cell	Number	2021	0.0569808	0.111229586	0.024125124
YLDs (Year	Montenegro	Female	55+ years	Non-melanoma skin cancer (basal-cell	Number	2021	0.0631089	0.124582858	0.026298169
YLDs (Year	Montenegro	Both	55+ years	Non-melanoma skin cancer (basal-cell	Number	2021	0.1200896	0.231364881	0.050330469
YLDs (Year	Montenegro	Male	55+ years	Non-melanoma skin cancer (basal-cell	Percent	2021	3.42E-06	6.42E-06	1.46E-06
YLDs (Year	Montenegro	Female	55+ years	Non-melanoma skin cancer (basal-cell	Percent	2021	2.84E-06	5.64E-06	1.19E-06
YLDs (Year	Montenegro	Both	55+ years	Non-melanoma skin cancer (basal-cell	Percent	2021	3.09E-06	5.86E-06	1.38E-06
YLDs (Year	Montenegro	Male	55+ years	Non-melanoma skin cancer (basal-cell	Rate	2021	0.0717952	0.140148136	0.030397408
YLDs (Year	Montenegro	Female	55+ years	Non-melanoma skin cancer (basal-cell	Rate	2021	0.0664252	0.131129559	0.027680111
YLDs (Year	Montenegro	Both	55+ years	Non-melanoma skin cancer (basal-cell	Rate	2021	0.0688693	0.132683752	0.028863652
YLDs (Year	Canada	Male	55+ years	Non-melanoma skin cancer (basal-cell	Number	1990	2.0200073	4.160849736	0.801021173
YLDs (Year	Canada	Female	55+ years	Non-melanoma skin cancer (basal-cell	Number	1990	2.3356066	4.672220737	0.941751807
YLDs (Year	Canada	Both	55+ years	Non-melanoma skin cancer (basal-cell	Number	1990	4.3556139	8.656565809	1.7894439
YLDs (Year	Canada	Male	55+ years	Non-melanoma skin cancer (basal-cell	Percent	1990	4.25E-06	8.34E-06	1.84E-06
YLDs (Year	Canada	Female	55+ years	Non-melanoma skin cancer (basal-cell	Percent	1990	3.57E-06	6.72E-06	1.48E-06
YLDs (Year	Canada	Both	55+ years	Non-melanoma skin cancer (basal-cell	Percent	1990	3.86E-06	7.06E-06	1.69E-06
YLDs (Year	Canada	Male	55+ years	Non-melanoma skin cancer (basal-cell	Rate	1990	0.0821449	0.169203625	0.03257404
YLDs (Year	Canada	Female	55+ years	Non-melanoma skin cancer (basal-cell	Rate	1990	0.0778552	0.155744014	0.031392397
YLDs (Year	Canada	Both	55+ years	Non-melanoma skin cancer (basal-cell	Rate	1990	0.0797876	0.158573787	0.032779615
YLDs (Year	Canada	Male	55+ years	Non-melanoma skin cancer (basal-cell	Number	2021	5.470365	11.50784277	2.096900349
YLDs (Year	Canada	Female	55+ years	Non-melanoma skin cancer (basal-cell	Number	2021	6.0909431	12.79248523	2.393427587
YLDs (Year	Canada	Both	55+ years	Non-melanoma skin cancer (basal-cell	Number	2021	11.561308	23.23738377	4.72326091
YLDs (Year	Canada	Male	55+ years	Non-melanoma skin cancer (basal-cell	Percent	2021	4.47E-06	8.48E-06	1.93E-06
YLDs (Year	Canada	Female	55+ years	Non-melanoma skin cancer (basal-cell	Percent	2021	4.10E-06	8.17E-06	1.73E-06
YLDs (Year	Canada	Both	55+ years	Non-melanoma skin cancer (basal-cell	Percent	2021	4.26E-06	8.27E-06	1.86E-06
YLDs (Year	Canada	Male	55+ years	Non-melanoma skin cancer (basal-cell	Rate	2021	0.0943028	0.198381961	0.036148148
YLDs (Year	Canada	Female	55+ years	Non-melanoma skin cancer (basal-cell	Rate	2021	0.0941365	0.197709816	0.036990789
YLDs (Year	Canada	Both	55+ years	Non-melanoma skin cancer (basal-cell	Rate	2021	0.0942151	0.189365438	0.038490666
YLDs (Year	Portugal	Male	55+ years	Non-melanoma skin cancer (basal-cell	Number	1990	0.4837369	0.990917439	0.209441911
YLDs (Year	Portugal	Female	55+ years	Non-melanoma skin cancer (basal-cell	Number	1990	0.6532325	1.292542453	0.280415868
YLDs (Year	Portugal	Both	55+ years	Non-melanoma skin cancer (basal-cell	Number	1990	1.1369694	2.229369275	0.484730425
YLDs (Year	Portugal	Male	55+ years	Non-melanoma skin cancer (basal-cell	Percent	1990	2.19E-06	4.07E-06	1.01E-06
YLDs (Year	Portugal	Female	55+ years	Non-melanoma skin cancer (basal-cell	Percent	1990	2.14E-06	3.95E-06	9.70E-07
YLDs (Year	Portugal	Both	55+ years	Non-melanoma skin cancer (basal-cell	Percent	1990	2.16E-06	3.91E-06	1.01E-06
YLDs (Year	Portugal	Male	55+ years	Non-melanoma skin cancer (basal-cell	Rate	1990	0.0453435	0.092884413	0.0196322
YLDs (Year	Portugal	Female	55+ years	Non-melanoma skin cancer (basal-cell	Rate	1990	0.0476416	0.094267762	0.020451302
YLDs (Year	Portugal	Both	55+ years	Non-melanoma skin cancer (basal-cell	Rate	1990	0.0466359	0.091443747	0.019882559
YLDs (Year	Portugal	Male	55+ years	Non-melanoma skin cancer (basal-cell	Number	2021	1.3667852	2.798046016	0.494809939
YLDs (Year	Portugal	Female	55+ years	Non-melanoma skin cancer (basal-cell	Number	2021	1.3685004	2.652747636	0.559751907
YLDs (Year	Portugal	Both	55+ years	Non-melanoma skin cancer (basal-cell	Number	2021	2.7352855	5.405038171	1.080652131
YLDs (Year	Portugal	Male	55+ years	Non-melanoma skin cancer (basal-cell	Percent	2021	3.81E-06	7.37E-06	1.47E-06
YLDs (Year	Portugal	Female	55+ years	Non-melanoma skin cancer (basal-cell	Percent	2021	2.66E-06	4.79E-06	1.21E-06
YLDs (Year	Portugal	Both	55+ years	Non-melanoma skin cancer (basal-cell	Percent	2021	3.14E-06	6.00E-06	1.36E-06
YLDs (Year	Portugal	Male	55+ years	Non-melanoma skin cancer (basal-cell	Rate	2021	0.079422	0.162590577	0.02875272
YLDs (Year	Portugal	Female	55+ years	Non-melanoma skin cancer (basal-cell	Rate	2021	0.0630724	0.122261735	0.025798247
YLDs (Year	Portugal	Both	55+ years	Non-melanoma skin cancer (basal-cell	Rate	2021	0.0703042	0.138924009	0.027775664
YLDs (Year	Israel	Male	55+ years	Non-melanoma skin cancer (basal-cell	Number	1990	0.4296915	0.90247227	0.161326739

YLDs (Year	Israel	Female	55+ years	Non-melanoma skin cancer (basal-cell	Number	1990	0.3673079	0.713554642	0.154626081
YLDs (Year	Israel	Both	55+ years	Non-melanoma skin cancer (basal-cell	Number	1990	0.7969994	1.574444367	0.347295245
YLDs (Year	Israel	Male	55+ years	Non-melanoma skin cancer (basal-cell	Percent	1990	6.05E-06	1.20E-05	2.48E-06
YLDs (Year	Israel	Female	55+ years	Non-melanoma skin cancer (basal-cell	Percent	1990	3.86E-06	7.59E-06	1.67E-06
YLDs (Year	Israel	Both	55+ years	Non-melanoma skin cancer (basal-cell	Percent	1990	4.79E-06	9.11E-06	2.08E-06
YLDs (Year	Israel	Male	55+ years	Non-melanoma skin cancer (basal-cell	Rate	1990	0.1167938	0.245299594	0.043849972
YLDs (Year	Israel	Female	55+ years	Non-melanoma skin cancer (basal-cell	Rate	1990	0.0829814	0.161204744	0.034932795
YLDs (Year	Israel	Both	55+ years	Non-melanoma skin cancer (basal-cell	Rate	1990	0.0983288	0.194245182	0.042847133
YLDs (Year	Israel	Male	55+ years	Non-melanoma skin cancer (basal-cell	Number	2021	1.0591848	2.137845676	0.422548091
YLDs (Year	Israel	Female	55+ years	Non-melanoma skin cancer (basal-cell	Number	2021	0.9253091	1.830358298	0.383971704
YLDs (Year	Israel	Both	55+ years	Non-melanoma skin cancer (basal-cell	Number	2021	1.9844939	3.819778709	0.85745037
YLDs (Year	Israel	Male	55+ years	Non-melanoma skin cancer (basal-cell	Percent	2021	5.78E-06	1.16E-05	2.46E-06
YLDs (Year	Israel	Female	55+ years	Non-melanoma skin cancer (basal-cell	Percent	2021	3.84E-06	7.45E-06	1.67E-06
YLDs (Year	Israel	Both	55+ years	Non-melanoma skin cancer (basal-cell	Percent	2021	4.68E-06	9.04E-06	2.09E-06
YLDs (Year	Israel	Male	55+ years	Non-melanoma skin cancer (basal-cell	Rate	2021	0.1143073	0.230716431	0.045601415
YLDs (Year	Israel	Female	55+ years	Non-melanoma skin cancer (basal-cell	Rate	2021	0.0863216	0.170753102	0.035820506
YLDs (Year	Israel	Both	55+ years	Non-melanoma skin cancer (basal-cell	Rate	2021	0.099297	0.191128031	0.042903742
YLDs (Year	Russian Fe	Male	55+ years	Non-melanoma skin cancer (basal-cell	Number	1990	3.5044515	6.713939988	1.45114848
YLDs (Year	Russian Fe	Female	55+ years	Non-melanoma skin cancer (basal-cell	Number	1990	7.6340034	14.67413674	3.115935765
YLDs (Year	Russian Fe	Both	55+ years	Non-melanoma skin cancer (basal-cell	Number	1990	11.138455	21.38807673	4.567743814
YLDs (Year	Russian Fe	Male	55+ years	Non-melanoma skin cancer (basal-cell	Percent	1990	1.45E-06	2.63E-06	6.73E-07
YLDs (Year	Russian Fe	Female	55+ years	Non-melanoma skin cancer (basal-cell	Percent	1990	1.58E-06	2.90E-06	7.15E-07
YLDs (Year	Russian Fe	Both	55+ years	Non-melanoma skin cancer (basal-cell	Percent	1990	1.54E-06	2.78E-06	7.03E-07
YLDs (Year	Russian Fe	Male	55+ years	Non-melanoma skin cancer (basal-cell	Rate	1990	0.031829	0.060979089	0.013179997
YLDs (Year	Russian Fe	Female	55+ years	Non-melanoma skin cancer (basal-cell	Rate	1990	0.0371439	0.071398305	0.01516086
YLDs (Year	Russian Fe	Both	55+ years	Non-melanoma skin cancer (basal-cell	Rate	1990	0.0352899	0.067763702	0.014471953
YLDs (Year	Russian Fe	Male	55+ years	Non-melanoma skin cancer (basal-cell	Number	2021	7.3625467	13.8661294	3.161040195
YLDs (Year	Russian Fe	Female	55+ years	Non-melanoma skin cancer (basal-cell	Number	2021	12.958315	25.01246204	5.405844311
YLDs (Year	Russian Fe	Both	55+ years	Non-melanoma skin cancer (basal-cell	Number	2021	20.320862	38.66513547	8.507263689
YLDs (Year	Russian Fe	Male	55+ years	Non-melanoma skin cancer (basal-cell	Percent	2021	2.03E-06	3.61E-06	9.68E-07
YLDs (Year	Russian Fe	Female	55+ years	Non-melanoma skin cancer (basal-cell	Percent	2021	2.02E-06	3.65E-06	9.14E-07
YLDs (Year	Russian Fe	Both	55+ years	Non-melanoma skin cancer (basal-cell	Percent	2021	2.02E-06	3.66E-06	9.36E-07
YLDs (Year	Russian Fe	Male	55+ years	Non-melanoma skin cancer (basal-cell	Rate	2021	0.0449573	0.084669625	0.019302004
YLDs (Year	Russian Fe	Female	55+ years	Non-melanoma skin cancer (basal-cell	Rate	2021	0.0495946	0.095728747	0.020689475
YLDs (Year	Russian Fe	Both	55+ years	Non-melanoma skin cancer (basal-cell	Rate	2021	0.0478079	0.090965613	0.020014632
YLDs (Year	Finland	Male	55+ years	Non-melanoma skin cancer (basal-cell	Number	1990	0.4788907	0.959124891	0.199198815
YLDs (Year	Finland	Female	55+ years	Non-melanoma skin cancer (basal-cell	Number	1990	0.6284932	1.253572938	0.248804156
YLDs (Year	Finland	Both	55+ years	Non-melanoma skin cancer (basal-cell	Number	1990	1.1073839	2.165532661	0.464666721
YLDs (Year	Finland	Male	55+ years	Non-melanoma skin cancer (basal-cell	Percent	1990	4.77E-06	8.83E-06	2.14E-06
YLDs (Year	Finland	Female	55+ years	Non-melanoma skin cancer (basal-cell	Percent	1990	3.90E-06	7.53E-06	1.60E-06
YLDs (Year	Finland	Both	55+ years	Non-melanoma skin cancer (basal-cell	Percent	1990	4.24E-06	7.93E-06	1.96E-06
YLDs (Year	Finland	Male	55+ years	Non-melanoma skin cancer (basal-cell	Rate	1990	0.0994996	0.199278252	0.041387719
YLDs (Year	Finland	Female	55+ years	Non-melanoma skin cancer (basal-cell	Rate	1990	0.089015	0.177546572	0.035238735
YLDs (Year	Finland	Both	55+ years	Non-melanoma skin cancer (basal-cell	Rate	1990	0.093265	0.182383322	0.039134695
YLDs (Year	Finland	Male	55+ years	Non-melanoma skin cancer (basal-cell	Number	2021	1.0533001	2.066457783	0.420703033
YLDs (Year	Finland	Female	55+ years	Non-melanoma skin cancer (basal-cell	Number	2021	0.9957063	2.030814986	0.42768205
YLDs (Year	Finland	Both	55+ years	Non-melanoma skin cancer (basal-cell	Number	2021	2.0490064	4.032580211	0.821974044
YLDs (Year	Finland	Male	55+ years	Non-melanoma skin cancer (basal-cell	Percent	2021	5.25E-06	9.34E-06	2.19E-06
YLDs (Year	Finland	Female	55+ years	Non-melanoma skin cancer (basal-cell	Percent	2021	3.87E-06	7.36E-06	1.72E-06
YLDs (Year	Finland	Both	55+ years	Non-melanoma skin cancer (basal-cell	Percent	2021	4.47E-06	8.25E-06	1.97E-06
YLDs (Year	Finland	Male	55+ years	Non-melanoma skin cancer (basal-cell	Rate	2021	0.1134686	0.222612766	0.045320967
YLDs (Year	Finland	Female	55+ years	Non-melanoma skin cancer (basal-cell	Rate	2021	0.0918216	0.187276782	0.039439791
YLDs (Year	Finland	Both	55+ years	Non-melanoma skin cancer (basal-cell	Rate	2021	0.1018055	0.20036004	0.040840044
YLDs (Year	Estonia	Male	55+ years	Non-melanoma skin cancer (basal-cell	Number	1990	0.0743283	0.152726611	0.031349475
YLDs (Year	Estonia	Female	55+ years	Non-melanoma skin cancer (basal-cell	Number	1990	0.1510064	0.329378731	0.062468762
YLDs (Year	Estonia	Both	55+ years	Non-melanoma skin cancer (basal-cell	Number	1990	0.2253346	0.477936236	0.096434253

YLDs (Year	Estonia	Male	55+ years	Non-melanoma skin cancer (basal-cell	Percent	1990	2.65E-06	5.01E-06	1.30E-06
YLDs (Year	Estonia	Female	55+ years	Non-melanoma skin cancer (basal-cell	Percent	1990	2.86E-06	5.76E-06	1.23E-06
YLDs (Year	Estonia	Both	55+ years	Non-melanoma skin cancer (basal-cell	Percent	1990	2.79E-06	5.35E-06	1.29E-06
YLDs (Year	Estonia	Male	55+ years	Non-melanoma skin cancer (basal-cell	Rate	1990	0.057049	0.117221913	0.024061592
YLDs (Year	Estonia	Female	55+ years	Non-melanoma skin cancer (basal-cell	Rate	1990	0.0661776	0.144348093	0.02737653
YLDs (Year	Estonia	Both	55+ years	Non-melanoma skin cancer (basal-cell	Rate	1990	0.0628597	0.133325928	0.026901468
YLDs (Year	Estonia	Male	55+ years	Non-melanoma skin cancer (basal-cell	Number	2021	0.1028128	0.208393131	0.043589937
YLDs (Year	Estonia	Female	55+ years	Non-melanoma skin cancer (basal-cell	Number	2021	0.1812694	0.346952858	0.072914407
YLDs (Year	Estonia	Both	55+ years	Non-melanoma skin cancer (basal-cell	Number	2021	0.2840822	0.543855724	0.115607293
YLDs (Year	Estonia	Male	55+ years	Non-melanoma skin cancer (basal-cell	Percent	2021	2.76E-06	5.03E-06	1.31E-06
YLDs (Year	Estonia	Female	55+ years	Non-melanoma skin cancer (basal-cell	Percent	2021	2.80E-06	5.24E-06	1.16E-06
YLDs (Year	Estonia	Both	55+ years	Non-melanoma skin cancer (basal-cell	Percent	2021	2.78E-06	5.06E-06	1.23E-06
YLDs (Year	Estonia	Male	55+ years	Non-melanoma skin cancer (basal-cell	Rate	2021	0.0599977	0.121610403	0.02543745
YLDs (Year	Estonia	Female	55+ years	Non-melanoma skin cancer (basal-cell	Rate	2021	0.0681172	0.130377497	0.027399682
YLDs (Year	Estonia	Both	55+ years	Non-melanoma skin cancer (basal-cell	Rate	2021	0.0649367	0.124316888	0.026426014
YLDs (Year	Republic o	Male	55+ years	Non-melanoma skin cancer (basal-cell	Number	1990	0.1831928	0.370715744	0.083891293
YLDs (Year	Republic o	Female	55+ years	Non-melanoma skin cancer (basal-cell	Number	1990	0.2601327	0.501468932	0.114425216
YLDs (Year	Republic o	Both	55+ years	Non-melanoma skin cancer (basal-cell	Number	1990	0.4433255	0.888112535	0.197858938
YLDs (Year	Republic o	Male	55+ years	Non-melanoma skin cancer (basal-cell	Percent	1990	4.33E-07	7.93E-07	2.10E-07
YLDs (Year	Republic o	Female	55+ years	Non-melanoma skin cancer (basal-cell	Percent	1990	4.42E-07	7.97E-07	2.08E-07
YLDs (Year	Republic o	Both	55+ years	Non-melanoma skin cancer (basal-cell	Percent	1990	4.38E-07	8.05E-07	2.09E-07
YLDs (Year	Republic o	Male	55+ years	Non-melanoma skin cancer (basal-cell	Rate	1990	0.0088023	0.017812753	0.004030945
YLDs (Year	Republic o	Female	55+ years	Non-melanoma skin cancer (basal-cell	Rate	1990	0.0089838	0.017318492	0.003951735
YLDs (Year	Republic o	Both	55+ years	Non-melanoma skin cancer (basal-cell	Rate	1990	0.0089079	0.017845228	0.003975665
YLDs (Year	Republic o	Male	55+ years	Non-melanoma skin cancer (basal-cell	Number	2021	1.1340471	2.263271738	0.502119942
YLDs (Year	Republic o	Female	55+ years	Non-melanoma skin cancer (basal-cell	Number	2021	1.6947445	3.442712049	0.739094951
YLDs (Year	Republic o	Both	55+ years	Non-melanoma skin cancer (basal-cell	Number	2021	2.8287916	5.707641516	1.252824796
YLDs (Year	Republic o	Male	55+ years	Non-melanoma skin cancer (basal-cell	Percent	2021	7.23E-07	1.32E-06	3.45E-07
YLDs (Year	Republic o	Female	55+ years	Non-melanoma skin cancer (basal-cell	Percent	2021	8.91E-07	1.59E-06	4.24E-07
YLDs (Year	Republic o	Both	55+ years	Non-melanoma skin cancer (basal-cell	Percent	2021	8.15E-07	1.45E-06	3.84E-07
YLDs (Year	Republic o	Male	55+ years	Non-melanoma skin cancer (basal-cell	Rate	2021	0.0144993	0.028936925	0.006419824
YLDs (Year	Republic o	Female	55+ years	Non-melanoma skin cancer (basal-cell	Rate	2021	0.019037	0.038671897	0.008302235
YLDs (Year	Republic o	Both	55+ years	Non-melanoma skin cancer (basal-cell	Rate	2021	0.0169148	0.03412894	0.007491287
YLDs (Year	Antigua an	Male	55+ years	Non-melanoma skin cancer (basal-cell	Number	1990	0.0006093	0.001210416	0.000246201
YLDs (Year	Antigua an	Female	55+ years	Non-melanoma skin cancer (basal-cell	Number	1990	0.0003327	0.000649616	0.000129776
YLDs (Year	Antigua an	Both	55+ years	Non-melanoma skin cancer (basal-cell	Number	1990	0.0009421	0.001849639	0.000370082
YLDs (Year	Antigua an	Male	55+ years	Non-melanoma skin cancer (basal-cell	Percent	1990	7.88E-07	1.44E-06	3.59E-07
YLDs (Year	Antigua an	Female	55+ years	Non-melanoma skin cancer (basal-cell	Percent	1990	3.20E-07	5.85E-07	1.43E-07
YLDs (Year	Antigua an	Both	55+ years	Non-melanoma skin cancer (basal-cell	Percent	1990	5.20E-07	9.63E-07	2.33E-07
YLDs (Year	Antigua an	Male	55+ years	Non-melanoma skin cancer (basal-cell	Rate	1990	0.016125	0.032031805	0.006515336
YLDs (Year	Antigua an	Female	55+ years	Non-melanoma skin cancer (basal-cell	Rate	1990	0.0068435	0.013361217	0.002669225
YLDs (Year	Antigua an	Both	55+ years	Non-melanoma skin cancer (basal-cell	Rate	1990	0.0109026	0.021406003	0.004282987
YLDs (Year	Antigua an	Male	55+ years	Non-melanoma skin cancer (basal-cell	Number	2021	0.0012887	0.002527027	0.000526275
YLDs (Year	Antigua an	Female	55+ years	Non-melanoma skin cancer (basal-cell	Number	2021	0.0005842	0.001146769	0.000239165
YLDs (Year	Antigua an	Both	55+ years	Non-melanoma skin cancer (basal-cell	Number	2021	0.001873	0.003650944	0.00076265
YLDs (Year	Antigua an	Male	55+ years	Non-melanoma skin cancer (basal-cell	Percent	2021	6.89E-07	1.26E-06	3.13E-07
YLDs (Year	Antigua an	Female	55+ years	Non-melanoma skin cancer (basal-cell	Percent	2021	2.70E-07	5.13E-07	1.19E-07
YLDs (Year	Antigua an	Both	55+ years	Non-melanoma skin cancer (basal-cell	Percent	2021	4.64E-07	8.76E-07	2.13E-07
YLDs (Year	Antigua an	Male	55+ years	Non-melanoma skin cancer (basal-cell	Rate	2021	0.0143803	0.028197547	0.005872376
YLDs (Year	Antigua an	Female	55+ years	Non-melanoma skin cancer (basal-cell	Rate	2021	0.0058977	0.011576794	0.002414408
YLDs (Year	Antigua an	Both	55+ years	Non-melanoma skin cancer (basal-cell	Rate	2021	0.0099269	0.019350307	0.004042109
YLDs (Year	Barbados	Male	55+ years	Non-melanoma skin cancer (basal-cell	Number	1990	0.0034575	0.00663249	0.001356063
YLDs (Year	Barbados	Female	55+ years	Non-melanoma skin cancer (basal-cell	Number	1990	0.0018869	0.003691983	0.00075922
YLDs (Year	Barbados	Both	55+ years	Non-melanoma skin cancer (basal-cell	Number	1990	0.0053444	0.01036415	0.002147007
YLDs (Year	Barbados	Male	55+ years	Non-melanoma skin cancer (basal-cell	Percent	1990	8.97E-07	1.65E-06	4.13E-07
YLDs (Year	Barbados	Female	55+ years	Non-melanoma skin cancer (basal-cell	Percent	1990	3.22E-07	5.91E-07	1.49E-07

YLDs (Year	Barbados	Both	55+ years	Non-melanoma skin cancer (basal-cell	Percent	1990	5.51E-07	1.02E-06	2.53E-07
YLDs (Year	Barbados	Male	55+ years	Non-melanoma skin cancer (basal-cell	Rate	1990	0.0175984	0.033758614	0.006902206
YLDs (Year	Barbados	Female	55+ years	Non-melanoma skin cancer (basal-cell	Rate	1990	0.0069275	0.013554524	0.002787353
YLDs (Year	Barbados	Both	55+ years	Non-melanoma skin cancer (basal-cell	Rate	1990	0.011399	0.022105556	0.004579322
YLDs (Year	Barbados	Male	55+ years	Non-melanoma skin cancer (basal-cell	Number	2021	0.0066756	0.01277219	0.002680619
YLDs (Year	Barbados	Female	55+ years	Non-melanoma skin cancer (basal-cell	Number	2021	0.0031332	0.006209593	0.001292178
YLDs (Year	Barbados	Both	55+ years	Non-melanoma skin cancer (basal-cell	Number	2021	0.0098088	0.018962748	0.003991517
YLDs (Year	Barbados	Male	55+ years	Non-melanoma skin cancer (basal-cell	Percent	2021	7.92E-07	1.43E-06	3.73E-07
YLDs (Year	Barbados	Female	55+ years	Non-melanoma skin cancer (basal-cell	Percent	2021	2.85E-07	5.37E-07	1.27E-07
YLDs (Year	Barbados	Both	55+ years	Non-melanoma skin cancer (basal-cell	Percent	2021	5.05E-07	9.28E-07	2.32E-07
YLDs (Year	Barbados	Male	55+ years	Non-melanoma skin cancer (basal-cell	Rate	2021	0.0161057	0.030814449	0.006467316
YLDs (Year	Barbados	Female	55+ years	Non-melanoma skin cancer (basal-cell	Rate	2021	0.0063081	0.01250166	0.002601518
YLDs (Year	Barbados	Both	55+ years	Non-melanoma skin cancer (basal-cell	Rate	2021	0.0107649	0.020811004	0.004380561
YLDs (Year	Sweden	Male	55+ years	Non-melanoma skin cancer (basal-cell	Number	1990	1.2698159	2.602211903	0.485328295
YLDs (Year	Sweden	Female	55+ years	Non-melanoma skin cancer (basal-cell	Number	1990	1.2602696	2.629102877	0.533873426
YLDs (Year	Sweden	Both	55+ years	Non-melanoma skin cancer (basal-cell	Number	1990	2.5300856	5.063946787	1.049184241
YLDs (Year	Sweden	Male	55+ years	Non-melanoma skin cancer (basal-cell	Percent	1990	5.96E-06	1.12E-05	2.41E-06
YLDs (Year	Sweden	Female	55+ years	Non-melanoma skin cancer (basal-cell	Percent	1990	4.19E-06	7.97E-06	1.86E-06
YLDs (Year	Sweden	Both	55+ years	Non-melanoma skin cancer (basal-cell	Percent	1990	4.92E-06	9.18E-06	2.14E-06
YLDs (Year	Sweden	Male	55+ years	Non-melanoma skin cancer (basal-cell	Rate	1990	0.1196942	0.245287335	0.045747575
YLDs (Year	Sweden	Female	55+ years	Non-melanoma skin cancer (basal-cell	Rate	1990	0.09597	0.200207212	0.04065467
YLDs (Year	Sweden	Both	55+ years	Non-melanoma skin cancer (basal-cell	Rate	1990	0.1065715	0.213301977	0.044193409
YLDs (Year	Sweden	Male	55+ years	Non-melanoma skin cancer (basal-cell	Number	2021	2.5135138	5.117295562	1.069871023
YLDs (Year	Sweden	Female	55+ years	Non-melanoma skin cancer (basal-cell	Number	2021	2.2923667	4.598026447	0.983982638
YLDs (Year	Sweden	Both	55+ years	Non-melanoma skin cancer (basal-cell	Number	2021	4.8058806	9.567185144	2.097422331
YLDs (Year	Sweden	Male	55+ years	Non-melanoma skin cancer (basal-cell	Percent	2021	7.60E-06	1.50E-05	3.23E-06
YLDs (Year	Sweden	Female	55+ years	Non-melanoma skin cancer (basal-cell	Percent	2021	5.57E-06	1.07E-05	2.50E-06
YLDs (Year	Sweden	Both	55+ years	Non-melanoma skin cancer (basal-cell	Percent	2021	6.47E-06	1.22E-05	2.91E-06
YLDs (Year	Sweden	Male	55+ years	Non-melanoma skin cancer (basal-cell	Rate	2021	0.1551478	0.315867461	0.066038289
YLDs (Year	Sweden	Female	55+ years	Non-melanoma skin cancer (basal-cell	Rate	2021	0.1312628	0.263286832	0.056343667
YLDs (Year	Sweden	Both	55+ years	Non-melanoma skin cancer (basal-cell	Rate	2021	0.1427572	0.284190315	0.06230329
YLDs (Year	Singapore	Male	55+ years	Non-melanoma skin cancer (basal-cell	Number	1990	0.0427514	0.083039343	0.019408876
YLDs (Year	Singapore	Female	55+ years	Non-melanoma skin cancer (basal-cell	Number	1990	0.0534774	0.101780064	0.023828264
YLDs (Year	Singapore	Both	55+ years	Non-melanoma skin cancer (basal-cell	Number	1990	0.0962288	0.186199922	0.043617125
YLDs (Year	Singapore	Male	55+ years	Non-melanoma skin cancer (basal-cell	Percent	1990	1.38E-06	2.45E-06	6.61E-07
YLDs (Year	Singapore	Female	55+ years	Non-melanoma skin cancer (basal-cell	Percent	1990	1.44E-06	2.54E-06	6.95E-07
YLDs (Year	Singapore	Both	55+ years	Non-melanoma skin cancer (basal-cell	Percent	1990	1.41E-06	2.47E-06	6.76E-07
YLDs (Year	Singapore	Male	55+ years	Non-melanoma skin cancer (basal-cell	Rate	1990	0.0254627	0.049458196	0.011559918
YLDs (Year	Singapore	Female	55+ years	Non-melanoma skin cancer (basal-cell	Rate	1990	0.0274707	0.052283168	0.012240286
YLDs (Year	Singapore	Both	55+ years	Non-melanoma skin cancer (basal-cell	Rate	1990	0.0265408	0.051355742	0.012030025
YLDs (Year	Singapore	Male	55+ years	Non-melanoma skin cancer (basal-cell	Number	2021	0.1528506	0.300880651	0.064021367
YLDs (Year	Singapore	Female	55+ years	Non-melanoma skin cancer (basal-cell	Number	2021	0.1530598	0.302172821	0.061586741
YLDs (Year	Singapore	Both	55+ years	Non-melanoma skin cancer (basal-cell	Number	2021	0.3059104	0.60298743	0.12738189
YLDs (Year	Singapore	Male	55+ years	Non-melanoma skin cancer (basal-cell	Percent	2021	1.16E-06	2.06E-06	5.53E-07
YLDs (Year	Singapore	Female	55+ years	Non-melanoma skin cancer (basal-cell	Percent	2021	1.07E-06	1.97E-06	4.82E-07
YLDs (Year	Singapore	Both	55+ years	Non-melanoma skin cancer (basal-cell	Percent	2021	1.11E-06	2.04E-06	5.28E-07
YLDs (Year	Singapore	Male	55+ years	Non-melanoma skin cancer (basal-cell	Rate	2021	0.0202437	0.039848882	0.008479043
YLDs (Year	Singapore	Female	55+ years	Non-melanoma skin cancer (basal-cell	Rate	2021	0.02004	0.039563164	0.008063486
YLDs (Year	Singapore	Both	55+ years	Non-melanoma skin cancer (basal-cell	Rate	2021	0.0201412	0.039700855	0.008386858
YLDs (Year	New Zealan	Male	55+ years	Non-melanoma skin cancer (basal-cell	Number	1990	0.248643	0.490090897	0.102086708
YLDs (Year	New Zealan	Female	55+ years	Non-melanoma skin cancer (basal-cell	Number	1990	0.2529396	0.516474024	0.106582654
YLDs (Year	New Zealan	Both	55+ years	Non-melanoma skin cancer (basal-cell	Number	1990	0.5015826	0.962944029	0.214585661
YLDs (Year	New Zealan	Male	55+ years	Non-melanoma skin cancer (basal-cell	Percent	1990	3.86E-06	6.92E-06	1.76E-06
YLDs (Year	New Zealan	Female	55+ years	Non-melanoma skin cancer (basal-cell	Percent	1990	2.94E-06	5.53E-06	1.30E-06
YLDs (Year	New Zealan	Both	55+ years	Non-melanoma skin cancer (basal-cell	Percent	1990	3.34E-06	6.10E-06	1.64E-06
YLDs (Year	New Zealan	Male	55+ years	Non-melanoma skin cancer (basal-cell	Rate	1990	0.0826867	0.162980584	0.033949113

YLDs (Year	New Zealan	Female	55+ years	Non-melanoma skin cancer (basal-cell	Rate	1990	0.0707981	0.144561709	0.029832615
YLDs (Year	New Zealan	Both	55+ years	Non-melanoma skin cancer (basal-cell	Rate	1990	0.0762314	0.146349865	0.032613092
YLDs (Year	New Zealan	Male	55+ years	Non-melanoma skin cancer (basal-cell	Number	2021	0.5927539	1.172273041	0.211788715
YLDs (Year	New Zealan	Female	55+ years	Non-melanoma skin cancer (basal-cell	Number	2021	0.5050848	1.026731204	0.206183484
YLDs (Year	New Zealan	Both	55+ years	Non-melanoma skin cancer (basal-cell	Number	2021	1.0978387	2.180825048	0.425119814
YLDs (Year	New Zealan	Male	55+ years	Non-melanoma skin cancer (basal-cell	Percent	2021	4.16E-06	7.53E-06	1.75E-06
YLDs (Year	New Zealan	Female	55+ years	Non-melanoma skin cancer (basal-cell	Percent	2021	2.94E-06	5.52E-06	1.29E-06
YLDs (Year	New Zealan	Both	55+ years	Non-melanoma skin cancer (basal-cell	Percent	2021	3.49E-06	6.40E-06	1.52E-06
YLDs (Year	New Zealan	Male	55+ years	Non-melanoma skin cancer (basal-cell	Rate	2021	0.0873578	0.172765029	0.031212595
YLDs (Year	New Zealan	Female	55+ years	Non-melanoma skin cancer (basal-cell	Rate	2021	0.0675349	0.137284346	0.027568817
YLDs (Year	New Zealan	Both	55+ years	Non-melanoma skin cancer (basal-cell	Rate	2021	0.0769645	0.152887718	0.029803215
YLDs (Year	Andorra	Male	55+ years	Non-melanoma skin cancer (basal-cell	Number	1990	0.005426	0.010970404	0.001938545
YLDs (Year	Andorra	Female	55+ years	Non-melanoma skin cancer (basal-cell	Number	1990	0.0040095	0.008320465	0.001574922
YLDs (Year	Andorra	Both	55+ years	Non-melanoma skin cancer (basal-cell	Number	1990	0.0094355	0.01919693	0.003766955
YLDs (Year	Andorra	Male	55+ years	Non-melanoma skin cancer (basal-cell	Percent	1990	5.73E-06	1.12E-05	2.42E-06
YLDs (Year	Andorra	Female	55+ years	Non-melanoma skin cancer (basal-cell	Percent	1990	4.03E-06	7.79E-06	1.68E-06
YLDs (Year	Andorra	Both	55+ years	Non-melanoma skin cancer (basal-cell	Percent	1990	4.86E-06	9.19E-06	2.17E-06
YLDs (Year	Andorra	Male	55+ years	Non-melanoma skin cancer (basal-cell	Rate	1990	0.1065068	0.21533728	0.038051554
YLDs (Year	Andorra	Female	55+ years	Non-melanoma skin cancer (basal-cell	Rate	1990	0.0840043	0.174326388	0.03299701
YLDs (Year	Andorra	Both	55+ years	Non-melanoma skin cancer (basal-cell	Rate	1990	0.0956223	0.194548145	0.038175585
YLDs (Year	Andorra	Male	55+ years	Non-melanoma skin cancer (basal-cell	Number	2021	0.0144564	0.028563168	0.005307906
YLDs (Year	Andorra	Female	55+ years	Non-melanoma skin cancer (basal-cell	Number	2021	0.0110697	0.022583978	0.004820724
YLDs (Year	Andorra	Both	55+ years	Non-melanoma skin cancer (basal-cell	Number	2021	0.025526	0.049919223	0.010589926
YLDs (Year	Andorra	Male	55+ years	Non-melanoma skin cancer (basal-cell	Percent	2021	5.57E-06	1.07E-05	2.29E-06
YLDs (Year	Andorra	Female	55+ years	Non-melanoma skin cancer (basal-cell	Percent	2021	3.75E-06	7.29E-06	1.75E-06
YLDs (Year	Andorra	Both	55+ years	Non-melanoma skin cancer (basal-cell	Percent	2021	4.60E-06	8.50E-06	2.08E-06
YLDs (Year	Andorra	Male	55+ years	Non-melanoma skin cancer (basal-cell	Rate	2021	0.1066081	0.210638257	0.039143002
YLDs (Year	Andorra	Female	55+ years	Non-melanoma skin cancer (basal-cell	Rate	2021	0.0858017	0.175049688	0.037365706
YLDs (Year	Andorra	Both	55+ years	Non-melanoma skin cancer (basal-cell	Rate	2021	0.0964639	0.188646632	0.040019729
YLDs (Year	Belgium	Male	55+ years	Non-melanoma skin cancer (basal-cell	Number	1990	1.079839	2.206866496	0.415105472
YLDs (Year	Belgium	Female	55+ years	Non-melanoma skin cancer (basal-cell	Number	1990	1.1690701	2.285140857	0.44223361
YLDs (Year	Belgium	Both	55+ years	Non-melanoma skin cancer (basal-cell	Number	1990	2.2489091	4.512928834	0.870091027
YLDs (Year	Belgium	Male	55+ years	Non-melanoma skin cancer (basal-cell	Percent	1990	4.86E-06	8.86E-06	2.14E-06
YLDs (Year	Belgium	Female	55+ years	Non-melanoma skin cancer (basal-cell	Percent	1990	3.46E-06	6.60E-06	1.40E-06
YLDs (Year	Belgium	Both	55+ years	Non-melanoma skin cancer (basal-cell	Percent	1990	4.02E-06	7.40E-06	1.73E-06
YLDs (Year	Belgium	Male	55+ years	Non-melanoma skin cancer (basal-cell	Rate	1990	0.095348	0.194862605	0.036653116
YLDs (Year	Belgium	Female	55+ years	Non-melanoma skin cancer (basal-cell	Rate	1990	0.0784928	0.153427252	0.029692125
YLDs (Year	Belgium	Both	55+ years	Non-melanoma skin cancer (basal-cell	Rate	1990	0.0857733	0.172122967	0.033185245
YLDs (Year	Belgium	Male	55+ years	Non-melanoma skin cancer (basal-cell	Number	2021	1.8065763	3.399817017	0.721665496
YLDs (Year	Belgium	Female	55+ years	Non-melanoma skin cancer (basal-cell	Number	2021	1.6733492	3.326714554	0.640929405
YLDs (Year	Belgium	Both	55+ years	Non-melanoma skin cancer (basal-cell	Number	2021	3.4799255	6.451056798	1.380366138
YLDs (Year	Belgium	Male	55+ years	Non-melanoma skin cancer (basal-cell	Percent	2021	4.94E-06	9.33E-06	2.08E-06
YLDs (Year	Belgium	Female	55+ years	Non-melanoma skin cancer (basal-cell	Percent	2021	3.40E-06	6.42E-06	1.51E-06
YLDs (Year	Belgium	Both	55+ years	Non-melanoma skin cancer (basal-cell	Percent	2021	4.06E-06	7.62E-06	1.82E-06
YLDs (Year	Belgium	Male	55+ years	Non-melanoma skin cancer (basal-cell	Rate	2021	0.102746	0.193358949	0.041043527
YLDs (Year	Belgium	Female	55+ years	Non-melanoma skin cancer (basal-cell	Rate	2021	0.0827375	0.16448692	0.031690276
YLDs (Year	Belgium	Both	55+ years	Non-melanoma skin cancer (basal-cell	Rate	2021	0.0920427	0.170627996	0.036510159
YLDs (Year	Greece	Male	55+ years	Non-melanoma skin cancer (basal-cell	Number	1990	1.3245921	2.738414996	0.565702642
YLDs (Year	Greece	Female	55+ years	Non-melanoma skin cancer (basal-cell	Number	1990	1.1901101	2.306962263	0.482073173
YLDs (Year	Greece	Both	55+ years	Non-melanoma skin cancer (basal-cell	Number	1990	2.5147021	5.155615839	1.076646942
YLDs (Year	Greece	Male	55+ years	Non-melanoma skin cancer (basal-cell	Percent	1990	5.48E-06	1.04E-05	2.55E-06
YLDs (Year	Greece	Female	55+ years	Non-melanoma skin cancer (basal-cell	Percent	1990	3.79E-06	6.89E-06	1.61E-06
YLDs (Year	Greece	Both	55+ years	Non-melanoma skin cancer (basal-cell	Percent	1990	4.52E-06	8.24E-06	2.06E-06
YLDs (Year	Greece	Male	55+ years	Non-melanoma skin cancer (basal-cell	Rate	1990	0.1071034	0.221421714	0.045741368
YLDs (Year	Greece	Female	55+ years	Non-melanoma skin cancer (basal-cell	Rate	1990	0.0828233	0.160548341	0.033548901
YLDs (Year	Greece	Both	55+ years	Non-melanoma skin cancer (basal-cell	Rate	1990	0.0940544	0.192829268	0.040268524

YLDs (Year	Greece	Male	55+ years	Non-melanoma skin cancer (basal-cell	Number	2021	2.0789634	4.222841398	0.79911165
YLDs (Year	Greece	Female	55+ years	Non-melanoma skin cancer (basal-cell	Number	2021	1.7898415	3.565814406	0.709130245
YLDs (Year	Greece	Both	55+ years	Non-melanoma skin cancer (basal-cell	Number	2021	3.8688049	7.689920101	1.646056312
YLDs (Year	Greece	Male	55+ years	Non-melanoma skin cancer (basal-cell	Percent	2021	5.79E-06	1.11E-05	2.35E-06
YLDs (Year	Greece	Female	55+ years	Non-melanoma skin cancer (basal-cell	Percent	2021	3.84E-06	7.55E-06	1.63E-06
YLDs (Year	Greece	Both	55+ years	Non-melanoma skin cancer (basal-cell	Percent	2021	4.69E-06	8.97E-06	2.09E-06
YLDs (Year	Greece	Male	55+ years	Non-melanoma skin cancer (basal-cell	Rate	2021	0.1230919	0.250027197	0.04731403
YLDs (Year	Greece	Female	55+ years	Non-melanoma skin cancer (basal-cell	Rate	2021	0.0901004	0.179502651	0.035697528
YLDs (Year	Greece	Both	55+ years	Non-melanoma skin cancer (basal-cell	Rate	2021	0.1052607	0.20922393	0.044785169
YLDs (Year	Luxembourg	Male	55+ years	Non-melanoma skin cancer (basal-cell	Number	1990	0.0396503	0.080423766	0.016139286
YLDs (Year	Luxembourg	Female	55+ years	Non-melanoma skin cancer (basal-cell	Number	1990	0.0462533	0.09339799	0.018579381
YLDs (Year	Luxembourg	Both	55+ years	Non-melanoma skin cancer (basal-cell	Number	1990	0.0859036	0.165687117	0.035017013
YLDs (Year	Luxembourg	Male	55+ years	Non-melanoma skin cancer (basal-cell	Percent	1990	5.12E-06	9.73E-06	2.18E-06
YLDs (Year	Luxembourg	Female	55+ years	Non-melanoma skin cancer (basal-cell	Percent	1990	3.88E-06	7.14E-06	1.61E-06
YLDs (Year	Luxembourg	Both	55+ years	Non-melanoma skin cancer (basal-cell	Percent	1990	4.37E-06	7.71E-06	1.86E-06
YLDs (Year	Luxembourg	Male	55+ years	Non-melanoma skin cancer (basal-cell	Rate	1990	0.0999204	0.202671085	0.040671641
YLDs (Year	Luxembourg	Female	55+ years	Non-melanoma skin cancer (basal-cell	Rate	1990	0.0861264	0.173912485	0.034595887
YLDs (Year	Luxembourg	Both	55+ years	Non-melanoma skin cancer (basal-cell	Rate	1990	0.0919878	0.177421925	0.037497097
YLDs (Year	Luxembourg	Male	55+ years	Non-melanoma skin cancer (basal-cell	Number	2021	0.0877739	0.163090443	0.035680574
YLDs (Year	Luxembourg	Female	55+ years	Non-melanoma skin cancer (basal-cell	Number	2021	0.0782074	0.152901076	0.032148095
YLDs (Year	Luxembourg	Both	55+ years	Non-melanoma skin cancer (basal-cell	Number	2021	0.1659813	0.303152391	0.07168436
YLDs (Year	Luxembourg	Male	55+ years	Non-melanoma skin cancer (basal-cell	Percent	2021	5.34E-06	9.38E-06	2.26E-06
YLDs (Year	Luxembourg	Female	55+ years	Non-melanoma skin cancer (basal-cell	Percent	2021	3.80E-06	7.33E-06	1.76E-06
YLDs (Year	Luxembourg	Both	55+ years	Non-melanoma skin cancer (basal-cell	Percent	2021	4.48E-06	8.00E-06	2.08E-06
YLDs (Year	Luxembourg	Male	55+ years	Non-melanoma skin cancer (basal-cell	Rate	2021	0.1023513	0.190176253	0.04160635
YLDs (Year	Luxembourg	Female	55+ years	Non-melanoma skin cancer (basal-cell	Rate	2021	0.0850594	0.166297187	0.034964684
YLDs (Year	Luxembourg	Both	55+ years	Non-melanoma skin cancer (basal-cell	Rate	2021	0.0934043	0.170595939	0.040339648
YLDs (Year	Spain	Male	55+ years	Non-melanoma skin cancer (basal-cell	Number	1990	6.395449	13.03695434	2.714599473
YLDs (Year	Spain	Female	55+ years	Non-melanoma skin cancer (basal-cell	Number	1990	6.3156463	13.92619665	2.653218186
YLDs (Year	Spain	Both	55+ years	Non-melanoma skin cancer (basal-cell	Number	1990	12.711095	27.56719243	5.556195326
YLDs (Year	Spain	Male	55+ years	Non-melanoma skin cancer (basal-cell	Percent	1990	7.71E-06	1.47E-05	3.61E-06
YLDs (Year	Spain	Female	55+ years	Non-melanoma skin cancer (basal-cell	Percent	1990	5.41E-06	1.08E-05	2.37E-06
YLDs (Year	Spain	Both	55+ years	Non-melanoma skin cancer (basal-cell	Percent	1990	6.36E-06	1.16E-05	3.07E-06
YLDs (Year	Spain	Male	55+ years	Non-melanoma skin cancer (basal-cell	Rate	1990	0.1513838	0.308591787	0.064256043
YLDs (Year	Spain	Female	55+ years	Non-melanoma skin cancer (basal-cell	Rate	1990	0.1183606	0.2609887	0.049723552
YLDs (Year	Spain	Both	55+ years	Non-melanoma skin cancer (basal-cell	Rate	1990	0.1329529	0.288341695	0.058115558
YLDs (Year	Spain	Male	55+ years	Non-melanoma skin cancer (basal-cell	Number	2021	8.9995264	17.44842576	3.525407714
YLDs (Year	Spain	Female	55+ years	Non-melanoma skin cancer (basal-cell	Number	2021	8.780889	18.90640045	3.514624036
YLDs (Year	Spain	Both	55+ years	Non-melanoma skin cancer (basal-cell	Number	2021	17.780415	35.30897423	7.138217787
YLDs (Year	Spain	Male	55+ years	Non-melanoma skin cancer (basal-cell	Percent	2021	6.33E-06	1.19E-05	2.56E-06
YLDs (Year	Spain	Female	55+ years	Non-melanoma skin cancer (basal-cell	Percent	2021	4.45E-06	8.44E-06	1.99E-06
YLDs (Year	Spain	Both	55+ years	Non-melanoma skin cancer (basal-cell	Percent	2021	5.24E-06	1.01E-05	2.27E-06
YLDs (Year	Spain	Male	55+ years	Non-melanoma skin cancer (basal-cell	Rate	2021	0.127798	0.247776883	0.050062656
YLDs (Year	Spain	Female	55+ years	Non-melanoma skin cancer (basal-cell	Rate	2021	0.1041243	0.224193107	0.0416766
YLDs (Year	Spain	Both	55+ years	Non-melanoma skin cancer (basal-cell	Rate	2021	0.1148971	0.228166692	0.046127184
YLDs (Year	Jamaica	Male	55+ years	Non-melanoma skin cancer (basal-cell	Number	1990	0.0267723	0.051368991	0.011536619
YLDs (Year	Jamaica	Female	55+ years	Non-melanoma skin cancer (basal-cell	Number	1990	0.0213301	0.041064544	0.00935871
YLDs (Year	Jamaica	Both	55+ years	Non-melanoma skin cancer (basal-cell	Number	1990	0.0481023	0.09229063	0.020730502
YLDs (Year	Jamaica	Male	55+ years	Non-melanoma skin cancer (basal-cell	Percent	1990	1.05E-06	1.88E-06	4.85E-07
YLDs (Year	Jamaica	Female	55+ years	Non-melanoma skin cancer (basal-cell	Percent	1990	6.53E-07	1.24E-06	3.04E-07
YLDs (Year	Jamaica	Both	55+ years	Non-melanoma skin cancer (basal-cell	Percent	1990	8.26E-07	1.52E-06	3.80E-07
YLDs (Year	Jamaica	Male	55+ years	Non-melanoma skin cancer (basal-cell	Rate	1990	0.0195763	0.037561744	0.008435741
YLDs (Year	Jamaica	Female	55+ years	Non-melanoma skin cancer (basal-cell	Rate	1990	0.0135198	0.026028315	0.005931917
YLDs (Year	Jamaica	Both	55+ years	Non-melanoma skin cancer (basal-cell	Rate	1990	0.016332	0.031335145	0.007038562
YLDs (Year	Jamaica	Male	55+ years	Non-melanoma skin cancer (basal-cell	Number	2021	0.0430501	0.084085441	0.017469901
YLDs (Year	Jamaica	Female	55+ years	Non-melanoma skin cancer (basal-cell	Number	2021	0.0273463	0.051808584	0.0115515

YLDs (Year	Jamaica	Both	55+ years	Non-melanoma skin cancer (basal-cell	Number	2021	0.0703964	0.135007478	0.029145514
YLDs (Year	Jamaica	Male	55+ years	Non-melanoma skin cancer (basal-cell	Percent	2021	8.52E-07	1.59E-06	3.85E-07
YLDs (Year	Jamaica	Female	55+ years	Non-melanoma skin cancer (basal-cell	Percent	2021	4.59E-07	8.43E-07	2.04E-07
YLDs (Year	Jamaica	Both	55+ years	Non-melanoma skin cancer (basal-cell	Percent	2021	6.40E-07	1.16E-06	2.87E-07
YLDs (Year	Jamaica	Male	55+ years	Non-melanoma skin cancer (basal-cell	Rate	2021	0.016849	0.032909527	0.006837405
YLDs (Year	Jamaica	Female	55+ years	Non-melanoma skin cancer (basal-cell	Rate	2021	0.0100028	0.01895063	0.004225327
YLDs (Year	Jamaica	Both	55+ years	Non-melanoma skin cancer (basal-cell	Rate	2021	0.0133102	0.025526474	0.005510674
YLDs (Year	France	Male	55+ years	Non-melanoma skin cancer (basal-cell	Number	1990	8.9771709	17.89958023	3.805900809
YLDs (Year	France	Female	55+ years	Non-melanoma skin cancer (basal-cell	Number	1990	10.62791	21.59353041	4.358999175
YLDs (Year	France	Both	55+ years	Non-melanoma skin cancer (basal-cell	Number	1990	19.605081	40.91167456	8.54796306
YLDs (Year	France	Male	55+ years	Non-melanoma skin cancer (basal-cell	Percent	1990	7.35E-06	1.38E-05	3.46E-06
YLDs (Year	France	Female	55+ years	Non-melanoma skin cancer (basal-cell	Percent	1990	5.77E-06	1.14E-05	2.58E-06
YLDs (Year	France	Both	55+ years	Non-melanoma skin cancer (basal-cell	Percent	1990	6.40E-06	1.22E-05	3.00E-06
YLDs (Year	France	Male	55+ years	Non-melanoma skin cancer (basal-cell	Rate	1990	0.1501567	0.299397459	0.063659428
YLDs (Year	France	Female	55+ years	Non-melanoma skin cancer (basal-cell	Rate	1990	0.133886	0.272026307	0.054912857
YLDs (Year	France	Both	55+ years	Non-melanoma skin cancer (basal-cell	Rate	1990	0.1408759	0.293978259	0.061422939
YLDs (Year	France	Male	55+ years	Non-melanoma skin cancer (basal-cell	Number	2021	13.280521	26.01123673	5.38371469
YLDs (Year	France	Female	55+ years	Non-melanoma skin cancer (basal-cell	Number	2021	13.083665	27.41933137	5.119473583
YLDs (Year	France	Both	55+ years	Non-melanoma skin cancer (basal-cell	Number	2021	26.364186	52.01577057	11.45525796
YLDs (Year	France	Male	55+ years	Non-melanoma skin cancer (basal-cell	Percent	2021	6.31E-06	1.14E-05	2.76E-06
YLDs (Year	France	Female	55+ years	Non-melanoma skin cancer (basal-cell	Percent	2021	4.51E-06	8.77E-06	1.84E-06
YLDs (Year	France	Both	55+ years	Non-melanoma skin cancer (basal-cell	Percent	2021	5.27E-06	9.53E-06	2.34E-06
YLDs (Year	France	Male	55+ years	Non-melanoma skin cancer (basal-cell	Rate	2021	0.1328617	0.260223028	0.053860051
YLDs (Year	France	Female	55+ years	Non-melanoma skin cancer (basal-cell	Rate	2021	0.1080012	0.226337156	0.042259495
YLDs (Year	France	Both	55+ years	Non-melanoma skin cancer (basal-cell	Rate	2021	0.1192403	0.235257725	0.051810016
YLDs (Year	United Kin	Male	55+ years	Non-melanoma skin cancer (basal-cell	Number	1990	8.2441071	15.43380498	3.50437171
YLDs (Year	United Kin	Female	55+ years	Non-melanoma skin cancer (basal-cell	Number	1990	8.5394621	16.57807273	3.507221697
YLDs (Year	United Kin	Both	55+ years	Non-melanoma skin cancer (basal-cell	Number	1990	16.783569	31.6826929	7.052628124
YLDs (Year	United Kin	Male	55+ years	Non-melanoma skin cancer (basal-cell	Percent	1990	6.51E-06	1.18E-05	3.06E-06
YLDs (Year	United Kin	Female	55+ years	Non-melanoma skin cancer (basal-cell	Percent	1990	4.50E-06	8.03E-06	2.14E-06
YLDs (Year	United Kin	Both	55+ years	Non-melanoma skin cancer (basal-cell	Percent	1990	5.30E-06	9.81E-06	2.53E-06
YLDs (Year	United Kin	Male	55+ years	Non-melanoma skin cancer (basal-cell	Rate	1990	0.1278031	0.239260378	0.054326027
YLDs (Year	United Kin	Female	55+ years	Non-melanoma skin cancer (basal-cell	Rate	1990	0.1016311	0.197301379	0.041740659
YLDs (Year	United Kin	Both	55+ years	Non-melanoma skin cancer (basal-cell	Rate	1990	0.1129975	0.213307767	0.047482717
YLDs (Year	United Kin	Male	55+ years	Non-melanoma skin cancer (basal-cell	Number	2021	12.448451	23.88020358	5.26228362
YLDs (Year	United Kin	Female	55+ years	Non-melanoma skin cancer (basal-cell	Number	2021	10.740512	20.75113597	4.393386397
YLDs (Year	United Kin	Both	55+ years	Non-melanoma skin cancer (basal-cell	Number	2021	23.188963	44.76154125	9.655616696
YLDs (Year	United Kin	Male	55+ years	Non-melanoma skin cancer (basal-cell	Percent	2021	5.99E-06	1.09E-05	2.80E-06
YLDs (Year	United Kin	Female	55+ years	Non-melanoma skin cancer (basal-cell	Percent	2021	4.12E-06	7.54E-06	1.93E-06
YLDs (Year	United Kin	Both	55+ years	Non-melanoma skin cancer (basal-cell	Percent	2021	4.95E-06	9.01E-06	2.31E-06
YLDs (Year	United Kin	Male	55+ years	Non-melanoma skin cancer (basal-cell	Rate	2021	0.1249394	0.239674745	0.052815148
YLDs (Year	United Kin	Female	55+ years	Non-melanoma skin cancer (basal-cell	Rate	2021	0.0970534	0.18751142	0.039699519
YLDs (Year	United Kin	Both	55+ years	Non-melanoma skin cancer (basal-cell	Rate	2021	0.1102651	0.212844245	0.04591313
YLDs (Year	Uruguay	Male	55+ years	Non-melanoma skin cancer (basal-cell	Number	1990	0.2299996	0.454063112	0.102281164
YLDs (Year	Uruguay	Female	55+ years	Non-melanoma skin cancer (basal-cell	Number	1990	0.2232117	0.445538229	0.09525012
YLDs (Year	Uruguay	Both	55+ years	Non-melanoma skin cancer (basal-cell	Number	1990	0.4532113	0.872036001	0.207473481
YLDs (Year	Uruguay	Male	55+ years	Non-melanoma skin cancer (basal-cell	Percent	1990	3.96E-06	7.23E-06	1.92E-06
YLDs (Year	Uruguay	Female	55+ years	Non-melanoma skin cancer (basal-cell	Percent	1990	2.75E-06	4.97E-06	1.28E-06
YLDs (Year	Uruguay	Both	55+ years	Non-melanoma skin cancer (basal-cell	Percent	1990	3.25E-06	5.66E-06	1.58E-06
YLDs (Year	Uruguay	Male	55+ years	Non-melanoma skin cancer (basal-cell	Rate	1990	0.0770894	0.152189168	0.034281766
YLDs (Year	Uruguay	Female	55+ years	Non-melanoma skin cancer (basal-cell	Rate	1990	0.0592993	0.118363474	0.025304529
YLDs (Year	Uruguay	Both	55+ years	Non-melanoma skin cancer (basal-cell	Rate	1990	0.0671653	0.129234607	0.030747301
YLDs (Year	Uruguay	Male	55+ years	Non-melanoma skin cancer (basal-cell	Number	2021	0.3160663	0.61508777	0.131788084
YLDs (Year	Uruguay	Female	55+ years	Non-melanoma skin cancer (basal-cell	Number	2021	0.3441742	0.70650894	0.13751183
YLDs (Year	Uruguay	Both	55+ years	Non-melanoma skin cancer (basal-cell	Number	2021	0.6602405	1.308415709	0.27475719
YLDs (Year	Uruguay	Male	55+ years	Non-melanoma skin cancer (basal-cell	Percent	2021	3.92E-06	7.35E-06	1.84E-06

YLDs (Year	Uruguay	Female	55+ years	Non-melanoma skin cancer (basal-cell	Percent	2021	2.84E-06	5.45E-06	1.21E-06
YLDs (Year	Uruguay	Both	55+ years	Non-melanoma skin cancer (basal-cell	Percent	2021	3.27E-06	6.29E-06	1.50E-06
YLDs (Year	Uruguay	Male	55+ years	Non-melanoma skin cancer (basal-cell	Rate	2021	0.0811591	0.157941439	0.033840373
YLDs (Year	Uruguay	Female	55+ years	Non-melanoma skin cancer (basal-cell	Rate	2021	0.0668492	0.137225668	0.026709008
YLDs (Year	Uruguay	Both	55+ years	Non-melanoma skin cancer (basal-cell	Rate	2021	0.0730119	0.144689469	0.030383671
YLDs (Year	Dominican	Male	55+ years	Non-melanoma skin cancer (basal-cell	Number	1990	0.0475461	0.09124258	0.020170154
YLDs (Year	Dominican	Female	55+ years	Non-melanoma skin cancer (basal-cell	Number	1990	0.0187102	0.036157873	0.008062415
YLDs (Year	Dominican	Both	55+ years	Non-melanoma skin cancer (basal-cell	Number	1990	0.0662564	0.127545788	0.02832589
YLDs (Year	Dominican	Male	55+ years	Non-melanoma skin cancer (basal-cell	Percent	1990	8.53E-07	1.56E-06	3.98E-07
YLDs (Year	Dominican	Female	55+ years	Non-melanoma skin cancer (basal-cell	Percent	1990	3.10E-07	5.85E-07	1.43E-07
YLDs (Year	Dominican	Both	55+ years	Non-melanoma skin cancer (basal-cell	Percent	1990	5.71E-07	1.05E-06	2.65E-07
YLDs (Year	Dominican	Male	55+ years	Non-melanoma skin cancer (basal-cell	Rate	1990	0.0156601	0.030052294	0.006643383
YLDs (Year	Dominican	Female	55+ years	Non-melanoma skin cancer (basal-cell	Rate	1990	0.006079	0.01174777	0.002619496
YLDs (Year	Dominican	Both	55+ years	Non-melanoma skin cancer (basal-cell	Rate	1990	0.0108369	0.020861346	0.004632973
YLDs (Year	Dominican	Male	55+ years	Non-melanoma skin cancer (basal-cell	Number	2021	0.1286705	0.250118736	0.055649229
YLDs (Year	Dominican	Female	55+ years	Non-melanoma skin cancer (basal-cell	Number	2021	0.0559166	0.104401588	0.022658259
YLDs (Year	Dominican	Both	55+ years	Non-melanoma skin cancer (basal-cell	Number	2021	0.1845871	0.357677811	0.078282233
YLDs (Year	Dominican	Male	55+ years	Non-melanoma skin cancer (basal-cell	Percent	2021	7.78E-07	1.39E-06	3.53E-07
YLDs (Year	Dominican	Female	55+ years	Non-melanoma skin cancer (basal-cell	Percent	2021	2.95E-07	5.22E-07	1.33E-07
YLDs (Year	Dominican	Both	55+ years	Non-melanoma skin cancer (basal-cell	Percent	2021	5.20E-07	9.17E-07	2.33E-07
YLDs (Year	Dominican	Male	55+ years	Non-melanoma skin cancer (basal-cell	Rate	2021	0.0160088	0.03111901	0.006923707
YLDs (Year	Dominican	Female	55+ years	Non-melanoma skin cancer (basal-cell	Rate	2021	0.0064474	0.012037891	0.002612581
YLDs (Year	Dominican	Both	55+ years	Non-melanoma skin cancer (basal-cell	Rate	2021	0.0110463	0.021404711	0.004684687
YLDs (Year	Malta	Male	55+ years	Non-melanoma skin cancer (basal-cell	Number	1990	0.0489605	0.098726341	0.020432644
YLDs (Year	Malta	Female	55+ years	Non-melanoma skin cancer (basal-cell	Number	1990	0.0348146	0.07409956	0.014778549
YLDs (Year	Malta	Both	55+ years	Non-melanoma skin cancer (basal-cell	Number	1990	0.0837751	0.172207419	0.037915292
YLDs (Year	Malta	Male	55+ years	Non-melanoma skin cancer (basal-cell	Percent	1990	8.31E-06	1.57E-05	3.88E-06
YLDs (Year	Malta	Female	55+ years	Non-melanoma skin cancer (basal-cell	Percent	1990	4.09E-06	7.85E-06	1.79E-06
YLDs (Year	Malta	Both	55+ years	Non-melanoma skin cancer (basal-cell	Percent	1990	5.82E-06	1.09E-05	2.81E-06
YLDs (Year	Malta	Male	55+ years	Non-melanoma skin cancer (basal-cell	Rate	1990	0.1523243	0.307153991	0.063569338
YLDs (Year	Malta	Female	55+ years	Non-melanoma skin cancer (basal-cell	Rate	1990	0.0863808	0.183853246	0.036668022
YLDs (Year	Malta	Both	55+ years	Non-melanoma skin cancer (basal-cell	Rate	1990	0.1156381	0.237704721	0.052335979
YLDs (Year	Malta	Male	55+ years	Non-melanoma skin cancer (basal-cell	Number	2021	0.0972078	0.211361618	0.04094256
YLDs (Year	Malta	Female	55+ years	Non-melanoma skin cancer (basal-cell	Number	2021	0.078229	0.152036517	0.032434945
YLDs (Year	Malta	Both	55+ years	Non-melanoma skin cancer (basal-cell	Number	2021	0.1754368	0.358785897	0.072405258
YLDs (Year	Malta	Male	55+ years	Non-melanoma skin cancer (basal-cell	Percent	2021	6.61E-06	1.33E-05	2.90E-06
YLDs (Year	Malta	Female	55+ years	Non-melanoma skin cancer (basal-cell	Percent	2021	4.16E-06	7.70E-06	1.77E-06
YLDs (Year	Malta	Both	55+ years	Non-melanoma skin cancer (basal-cell	Percent	2021	5.24E-06	9.88E-06	2.29E-06
YLDs (Year	Malta	Male	55+ years	Non-melanoma skin cancer (basal-cell	Rate	2021	0.130159	0.283008281	0.054821134
YLDs (Year	Malta	Female	55+ years	Non-melanoma skin cancer (basal-cell	Rate	2021	0.0941165	0.182913617	0.039022159
YLDs (Year	Malta	Both	55+ years	Non-melanoma skin cancer (basal-cell	Rate	2021	0.1111744	0.227362881	0.045883264
YLDs (Year	Norway	Male	55+ years	Non-melanoma skin cancer (basal-cell	Number	1990	0.54882	1.056710256	0.220172029
YLDs (Year	Norway	Female	55+ years	Non-melanoma skin cancer (basal-cell	Number	1990	0.5657658	1.1039349	0.244113097
YLDs (Year	Norway	Both	55+ years	Non-melanoma skin cancer (basal-cell	Number	1990	1.1145857	2.158719353	0.459486368
YLDs (Year	Norway	Male	55+ years	Non-melanoma skin cancer (basal-cell	Percent	1990	5.20E-06	9.33E-06	2.38E-06
YLDs (Year	Norway	Female	55+ years	Non-melanoma skin cancer (basal-cell	Percent	1990	3.90E-06	7.10E-06	1.75E-06
YLDs (Year	Norway	Both	55+ years	Non-melanoma skin cancer (basal-cell	Percent	1990	4.45E-06	8.03E-06	2.03E-06
YLDs (Year	Norway	Male	55+ years	Non-melanoma skin cancer (basal-cell	Rate	1990	0.1145937	0.220641194	0.045971939
YLDs (Year	Norway	Female	55+ years	Non-melanoma skin cancer (basal-cell	Rate	1990	0.0936704	0.182771807	0.040416325
YLDs (Year	Norway	Both	55+ years	Non-melanoma skin cancer (basal-cell	Rate	1990	0.1029238	0.199341856	0.042430187
YLDs (Year	Norway	Male	55+ years	Non-melanoma skin cancer (basal-cell	Number	2021	0.8992776	1.737319265	0.374901918
YLDs (Year	Norway	Female	55+ years	Non-melanoma skin cancer (basal-cell	Number	2021	0.7574619	1.460160255	0.305634065
YLDs (Year	Norway	Both	55+ years	Non-melanoma skin cancer (basal-cell	Number	2021	1.6567395	3.205122041	0.67511697
YLDs (Year	Norway	Male	55+ years	Non-melanoma skin cancer (basal-cell	Percent	2021	5.65E-06	1.03E-05	2.59E-06
YLDs (Year	Norway	Female	55+ years	Non-melanoma skin cancer (basal-cell	Percent	2021	3.89E-06	7.34E-06	1.81E-06
YLDs (Year	Norway	Both	55+ years	Non-melanoma skin cancer (basal-cell	Percent	2021	4.68E-06	8.55E-06	2.17E-06

YLDs (Year	Norway	Male	55+ years	Non-melanoma skin cancer (basal-cell	Rate	2021	0.1147317	0.221650758	0.047830756
YLDs (Year	Norway	Female	55+ years	Non-melanoma skin cancer (basal-cell	Rate	2021	0.0903972	0.174258881	0.036475072
YLDs (Year	Norway	Both	55+ years	Non-melanoma skin cancer (basal-cell	Rate	2021	0.1021584	0.197635352	0.041629298
YLDs (Year	Netherland	Male	55+ years	Non-melanoma skin cancer (basal-cell	Number	1990	0.8581548	1.733675486	0.342454779
YLDs (Year	Netherland	Female	55+ years	Non-melanoma skin cancer (basal-cell	Number	1990	1.1057084	2.145449186	0.482275241
YLDs (Year	Netherland	Both	55+ years	Non-melanoma skin cancer (basal-cell	Number	1990	1.9638632	3.847624897	0.804149945
YLDs (Year	Netherland	Male	55+ years	Non-melanoma skin cancer (basal-cell	Percent	1990	3.12E-06	5.87E-06	1.34E-06
YLDs (Year	Netherland	Female	55+ years	Non-melanoma skin cancer (basal-cell	Percent	1990	2.67E-06	4.97E-06	1.16E-06
YLDs (Year	Netherland	Both	55+ years	Non-melanoma skin cancer (basal-cell	Percent	1990	2.85E-06	5.18E-06	1.27E-06
YLDs (Year	Netherland	Male	55+ years	Non-melanoma skin cancer (basal-cell	Rate	1990	0.0595324	0.120269482	0.02375696
YLDs (Year	Netherland	Female	55+ years	Non-melanoma skin cancer (basal-cell	Rate	1990	0.0591454	0.114762056	0.025797347
YLDs (Year	Netherland	Both	55+ years	Non-melanoma skin cancer (basal-cell	Rate	1990	0.0593139	0.116208441	0.024287454
YLDs (Year	Netherland	Male	55+ years	Non-melanoma skin cancer (basal-cell	Number	2021	2.3019049	4.347070748	1.054710731
YLDs (Year	Netherland	Female	55+ years	Non-melanoma skin cancer (basal-cell	Number	2021	2.0265595	4.013731993	0.869879546
YLDs (Year	Netherland	Both	55+ years	Non-melanoma skin cancer (basal-cell	Number	2021	4.3284645	8.18975084	1.896351952
YLDs (Year	Netherland	Male	55+ years	Non-melanoma skin cancer (basal-cell	Percent	2021	4.16E-06	7.61E-06	1.91E-06
YLDs (Year	Netherland	Female	55+ years	Non-melanoma skin cancer (basal-cell	Percent	2021	2.89E-06	5.32E-06	1.28E-06
YLDs (Year	Netherland	Both	55+ years	Non-melanoma skin cancer (basal-cell	Percent	2021	3.45E-06	6.33E-06	1.59E-06
YLDs (Year	Netherland	Male	55+ years	Non-melanoma skin cancer (basal-cell	Rate	2021	0.0822674	0.155359296	0.037694145
YLDs (Year	Netherland	Female	55+ years	Non-melanoma skin cancer (basal-cell	Rate	2021	0.0663837	0.13147719	0.028494508
YLDs (Year	Netherland	Both	55+ years	Non-melanoma skin cancer (basal-cell	Rate	2021	0.0739798	0.139974854	0.032411436
YLDs (Year	Cuba	Male	55+ years	Non-melanoma skin cancer (basal-cell	Number	1990	0.2877025	0.562244611	0.119282487
YLDs (Year	Cuba	Female	55+ years	Non-melanoma skin cancer (basal-cell	Number	1990	0.1613131	0.314403682	0.065829825
YLDs (Year	Cuba	Both	55+ years	Non-melanoma skin cancer (basal-cell	Number	1990	0.4490156	0.875038821	0.183694573
YLDs (Year	Cuba	Male	55+ years	Non-melanoma skin cancer (basal-cell	Percent	1990	1.86E-06	3.47E-06	8.66E-07
YLDs (Year	Cuba	Female	55+ years	Non-melanoma skin cancer (basal-cell	Percent	1990	9.00E-07	1.65E-06	4.22E-07
YLDs (Year	Cuba	Both	55+ years	Non-melanoma skin cancer (basal-cell	Percent	1990	1.35E-06	2.52E-06	6.30E-07
YLDs (Year	Cuba	Male	55+ years	Non-melanoma skin cancer (basal-cell	Rate	1990	0.0344145	0.067254784	0.014268377
YLDs (Year	Cuba	Female	55+ years	Non-melanoma skin cancer (basal-cell	Rate	1990	0.0187856	0.036613607	0.007666155
YLDs (Year	Cuba	Both	55+ years	Non-melanoma skin cancer (basal-cell	Rate	1990	0.0264953	0.051633872	0.010839362
YLDs (Year	Cuba	Male	55+ years	Non-melanoma skin cancer (basal-cell	Number	2021	0.4526883	0.869271647	0.186999266
YLDs (Year	Cuba	Female	55+ years	Non-melanoma skin cancer (basal-cell	Number	2021	0.301419	0.599508117	0.123607857
YLDs (Year	Cuba	Both	55+ years	Non-melanoma skin cancer (basal-cell	Number	2021	0.7541072	1.478582909	0.310441872
YLDs (Year	Cuba	Male	55+ years	Non-melanoma skin cancer (basal-cell	Percent	2021	1.42E-06	2.53E-06	6.55E-07
YLDs (Year	Cuba	Female	55+ years	Non-melanoma skin cancer (basal-cell	Percent	2021	7.58E-07	1.37E-06	3.37E-07
YLDs (Year	Cuba	Both	55+ years	Non-melanoma skin cancer (basal-cell	Percent	2021	1.05E-06	1.89E-06	4.76E-07
YLDs (Year	Cuba	Male	55+ years	Non-melanoma skin cancer (basal-cell	Rate	2021	0.0283049	0.054352293	0.011692362
YLDs (Year	Cuba	Female	55+ years	Non-melanoma skin cancer (basal-cell	Rate	2021	0.0165823	0.032981345	0.006800164
YLDs (Year	Cuba	Both	55+ years	Non-melanoma skin cancer (basal-cell	Rate	2021	0.022069	0.043270778	0.009085092
YLDs (Year	Bolivia (P	Male	55+ years	Non-melanoma skin cancer (basal-cell	Number	1990	0.0786674	0.15520074	0.034100136
YLDs (Year	Bolivia (P	Female	55+ years	Non-melanoma skin cancer (basal-cell	Number	1990	0.0721418	0.14357341	0.031576254
YLDs (Year	Bolivia (P	Both	55+ years	Non-melanoma skin cancer (basal-cell	Number	1990	0.1508092	0.297357445	0.065252255
YLDs (Year	Bolivia (P	Male	55+ years	Non-melanoma skin cancer (basal-cell	Percent	1990	1.62E-06	3.06E-06	7.46E-07
YLDs (Year	Bolivia (P	Female	55+ years	Non-melanoma skin cancer (basal-cell	Percent	1990	1.20E-06	2.24E-06	5.74E-07
YLDs (Year	Bolivia (P	Both	55+ years	Non-melanoma skin cancer (basal-cell	Percent	1990	1.39E-06	2.63E-06	6.48E-07
YLDs (Year	Bolivia (P	Male	55+ years	Non-melanoma skin cancer (basal-cell	Rate	1990	0.0320076	0.063146971	0.01387442
YLDs (Year	Bolivia (P	Female	55+ years	Non-melanoma skin cancer (basal-cell	Rate	1990	0.0258986	0.051542319	0.011335758
YLDs (Year	Bolivia (P	Both	55+ years	Non-melanoma skin cancer (basal-cell	Rate	1990	0.0287622	0.056711732	0.012444849
YLDs (Year	Bolivia (P	Male	55+ years	Non-melanoma skin cancer (basal-cell	Number	2021	0.2393037	0.478866146	0.100031743
YLDs (Year	Bolivia (P	Female	55+ years	Non-melanoma skin cancer (basal-cell	Number	2021	0.21532	0.423428368	0.090677251
YLDs (Year	Bolivia (P	Both	55+ years	Non-melanoma skin cancer (basal-cell	Number	2021	0.4546237	0.897501007	0.189955326
YLDs (Year	Bolivia (P	Male	55+ years	Non-melanoma skin cancer (basal-cell	Percent	2021	1.59E-06	2.99E-06	7.48E-07
YLDs (Year	Bolivia (P	Female	55+ years	Non-melanoma skin cancer (basal-cell	Percent	2021	1.15E-06	2.17E-06	5.31E-07
YLDs (Year	Bolivia (P	Both	55+ years	Non-melanoma skin cancer (basal-cell	Percent	2021	1.35E-06	2.50E-06	6.32E-07
YLDs (Year	Bolivia (P	Male	55+ years	Non-melanoma skin cancer (basal-cell	Rate	2021	0.0327561	0.065547672	0.013692444
YLDs (Year	Bolivia (P	Female	55+ years	Non-melanoma skin cancer (basal-cell	Rate	2021	0.0264225	0.051960135	0.011127271

YLDs (Year	Bolivia (P	Both	55+ years	Non-melanoma skin cancer (basal-cell	Rate	2021	0.0294165	0.05807295	0.012291091
YLDs (Year	Ireland	Male	55+ years	Non-melanoma skin cancer (basal-cell	Number	1990	0.6552121	1.294245082	0.265732742
YLDs (Year	Ireland	Female	55+ years	Non-melanoma skin cancer (basal-cell	Number	1990	0.6546935	1.28153761	0.28946873
YLDs (Year	Ireland	Both	55+ years	Non-melanoma skin cancer (basal-cell	Number	1990	1.3099055	2.532857309	0.596082961
YLDs (Year	Ireland	Male	55+ years	Non-melanoma skin cancer (basal-cell	Percent	1990	1.07E-05	1.95E-05	4.80E-06
YLDs (Year	Ireland	Female	55+ years	Non-melanoma skin cancer (basal-cell	Percent	1990	7.97E-06	1.53E-05	3.50E-06
YLDs (Year	Ireland	Both	55+ years	Non-melanoma skin cancer (basal-cell	Percent	1990	9.13E-06	1.69E-05	4.25E-06
YLDs (Year	Ireland	Male	55+ years	Non-melanoma skin cancer (basal-cell	Rate	1990	0.209999	0.414812471	0.085168765
YLDs (Year	Ireland	Female	55+ years	Non-melanoma skin cancer (basal-cell	Rate	1990	0.17756	0.347567025	0.078507088
YLDs (Year	Ireland	Both	55+ years	Non-melanoma skin cancer (basal-cell	Rate	1990	0.1924283	0.37208288	0.087566032
YLDs (Year	Ireland	Male	55+ years	Non-melanoma skin cancer (basal-cell	Number	2021	0.8981555	1.919322064	0.359361225
YLDs (Year	Ireland	Female	55+ years	Non-melanoma skin cancer (basal-cell	Number	2021	0.7412197	1.571863548	0.299821382
YLDs (Year	Ireland	Both	55+ years	Non-melanoma skin cancer (basal-cell	Number	2021	1.6393752	3.260967404	0.653942741
YLDs (Year	Ireland	Male	55+ years	Non-melanoma skin cancer (basal-cell	Percent	2021	7.30E-06	1.43E-05	3.06E-06
YLDs (Year	Ireland	Female	55+ years	Non-melanoma skin cancer (basal-cell	Percent	2021	4.84E-06	9.16E-06	1.96E-06
YLDs (Year	Ireland	Both	55+ years	Non-melanoma skin cancer (basal-cell	Percent	2021	5.93E-06	1.17E-05	2.45E-06
YLDs (Year	Ireland	Male	55+ years	Non-melanoma skin cancer (basal-cell	Rate	2021	0.1423881	0.304277743	0.056970961
YLDs (Year	Ireland	Female	55+ years	Non-melanoma skin cancer (basal-cell	Rate	2021	0.1087488	0.230617616	0.04398861
YLDs (Year	Ireland	Both	55+ years	Non-melanoma skin cancer (basal-cell	Rate	2021	0.1249173	0.248479589	0.049829208
YLDs (Year	Italy	Male	55+ years	Non-melanoma skin cancer (basal-cell	Number	1990	8.3573146	15.9419911	3.686501976
YLDs (Year	Italy	Female	55+ years	Non-melanoma skin cancer (basal-cell	Number	1990	7.7346033	14.78190171	3.315569481
YLDs (Year	Italy	Both	55+ years	Non-melanoma skin cancer (basal-cell	Number	1990	16.091918	31.231098	6.943737967
YLDs (Year	Italy	Male	55+ years	Non-melanoma skin cancer (basal-cell	Percent	1990	6.01E-06	1.06E-05	2.83E-06
YLDs (Year	Italy	Female	55+ years	Non-melanoma skin cancer (basal-cell	Percent	1990	3.89E-06	7.06E-06	1.86E-06
YLDs (Year	Italy	Both	55+ years	Non-melanoma skin cancer (basal-cell	Percent	1990	4.76E-06	8.36E-06	2.25E-06
YLDs (Year	Italy	Male	55+ years	Non-melanoma skin cancer (basal-cell	Rate	1990	0.126013	0.240376048	0.055585703
YLDs (Year	Italy	Female	55+ years	Non-melanoma skin cancer (basal-cell	Rate	1990	0.0900736	0.172143139	0.038611577
YLDs (Year	Italy	Both	55+ years	Non-melanoma skin cancer (basal-cell	Rate	1990	0.1057351	0.205210057	0.045625193
YLDs (Year	Italy	Male	55+ years	Non-melanoma skin cancer (basal-cell	Number	2021	14.277989	27.83065965	5.791088553
YLDs (Year	Italy	Female	55+ years	Non-melanoma skin cancer (basal-cell	Number	2021	13.045641	25.04880116	5.583085511
YLDs (Year	Italy	Both	55+ years	Non-melanoma skin cancer (basal-cell	Number	2021	27.32363	53.17610414	11.39163778
YLDs (Year	Italy	Male	55+ years	Non-melanoma skin cancer (basal-cell	Percent	2021	6.53E-06	1.19E-05	3.09E-06
YLDs (Year	Italy	Female	55+ years	Non-melanoma skin cancer (basal-cell	Percent	2021	4.41E-06	8.28E-06	2.03E-06
YLDs (Year	Italy	Both	55+ years	Non-melanoma skin cancer (basal-cell	Percent	2021	5.31E-06	9.90E-06	2.45E-06
YLDs (Year	Italy	Male	55+ years	Non-melanoma skin cancer (basal-cell	Rate	2021	0.1381693	0.269319555	0.056040835
YLDs (Year	Italy	Female	55+ years	Non-melanoma skin cancer (basal-cell	Rate	2021	0.1053504	0.202282156	0.045086332
YLDs (Year	Italy	Both	55+ years	Non-melanoma skin cancer (basal-cell	Rate	2021	0.1202794	0.234082784	0.050146327
YLDs (Year	Mozambique	Male	55+ years	Non-melanoma skin cancer (basal-cell	Number	1990	0.044321	0.087121939	0.018314989
YLDs (Year	Mozambique	Female	55+ years	Non-melanoma skin cancer (basal-cell	Number	1990	0.0313419	0.061881645	0.012961776
YLDs (Year	Mozambique	Both	55+ years	Non-melanoma skin cancer (basal-cell	Number	1990	0.0756629	0.149483092	0.030831167
YLDs (Year	Mozambique	Male	55+ years	Non-melanoma skin cancer (basal-cell	Percent	1990	4.40E-07	8.07E-07	2.00E-07
YLDs (Year	Mozambique	Female	55+ years	Non-melanoma skin cancer (basal-cell	Percent	1990	2.57E-07	4.77E-07	1.10E-07
YLDs (Year	Mozambique	Both	55+ years	Non-melanoma skin cancer (basal-cell	Percent	1990	3.40E-07	6.32E-07	1.49E-07
YLDs (Year	Mozambique	Male	55+ years	Non-melanoma skin cancer (basal-cell	Rate	1990	0.0093796	0.018437475	0.003875971
YLDs (Year	Mozambique	Female	55+ years	Non-melanoma skin cancer (basal-cell	Rate	1990	0.0062105	0.012262024	0.002568413
YLDs (Year	Mozambique	Both	55+ years	Non-melanoma skin cancer (basal-cell	Rate	1990	0.0077429	0.015297281	0.003155093
YLDs (Year	Mozambique	Male	55+ years	Non-melanoma skin cancer (basal-cell	Number	2021	0.0719162	0.141057783	0.029669369
YLDs (Year	Mozambique	Female	55+ years	Non-melanoma skin cancer (basal-cell	Number	2021	0.0610018	0.121069448	0.025081333
YLDs (Year	Mozambique	Both	55+ years	Non-melanoma skin cancer (basal-cell	Number	2021	0.132918	0.262727567	0.055379728
YLDs (Year	Mozambique	Male	55+ years	Non-melanoma skin cancer (basal-cell	Percent	2021	4.20E-07	7.77E-07	1.88E-07
YLDs (Year	Mozambique	Female	55+ years	Non-melanoma skin cancer (basal-cell	Percent	2021	2.50E-07	4.78E-07	1.11E-07
YLDs (Year	Mozambique	Both	55+ years	Non-melanoma skin cancer (basal-cell	Percent	2021	3.20E-07	5.96E-07	1.40E-07
YLDs (Year	Mozambique	Male	55+ years	Non-melanoma skin cancer (basal-cell	Rate	2021	0.0089694	0.017592683	0.003700355
YLDs (Year	Mozambique	Female	55+ years	Non-melanoma skin cancer (basal-cell	Rate	2021	0.0061784	0.01226223	0.002540303
YLDs (Year	Mozambique	Both	55+ years	Non-melanoma skin cancer (basal-cell	Rate	2021	0.0074292	0.01468462	0.003095337
YLDs (Year	Saint Vinc	Male	55+ years	Non-melanoma skin cancer (basal-cell	Number	1990	0.0008117	0.00157939	0.000333807

YLDs (Year	Saint Vinc	Female	55+ years	Non-melanoma skin cancer (basal-cell	Number	1990	0.0004142	0.00081036	0.00017153
YLDs (Year	Saint Vinc	Both	55+ years	Non-melanoma skin cancer (basal-cell	Number	1990	0.0012259	0.002305708	0.000511207
YLDs (Year	Saint Vinc	Male	55+ years	Non-melanoma skin cancer (basal-cell	Percent	1990	7.73E-07	1.39E-06	3.45E-07
YLDs (Year	Saint Vinc	Female	55+ years	Non-melanoma skin cancer (basal-cell	Percent	1990	2.87E-07	5.35E-07	1.30E-07
YLDs (Year	Saint Vinc	Both	55+ years	Non-melanoma skin cancer (basal-cell	Percent	1990	4.92E-07	9.06E-07	2.20E-07
YLDs (Year	Saint Vinc	Male	55+ years	Non-melanoma skin cancer (basal-cell	Rate	1990	0.0152582	0.029688711	0.00627476
YLDs (Year	Saint Vinc	Female	55+ years	Non-melanoma skin cancer (basal-cell	Rate	1990	0.0061869	0.01210453	0.002562187
YLDs (Year	Saint Vinc	Both	55+ years	Non-melanoma skin cancer (basal-cell	Rate	1990	0.0102035	0.01919102	0.004254916
YLDs (Year	Saint Vinc	Male	55+ years	Non-melanoma skin cancer (basal-cell	Number	2021	0.0019284	0.003824694	0.000802728
YLDs (Year	Saint Vinc	Female	55+ years	Non-melanoma skin cancer (basal-cell	Number	2021	0.000732	0.001439309	0.000290943
YLDs (Year	Saint Vinc	Both	55+ years	Non-melanoma skin cancer (basal-cell	Number	2021	0.0026604	0.005280688	0.001101773
YLDs (Year	Saint Vinc	Male	55+ years	Non-melanoma skin cancer (basal-cell	Percent	2021	6.85E-07	1.23E-06	3.08E-07
YLDs (Year	Saint Vinc	Female	55+ years	Non-melanoma skin cancer (basal-cell	Percent	2021	2.66E-07	5.03E-07	1.18E-07
YLDs (Year	Saint Vinc	Both	55+ years	Non-melanoma skin cancer (basal-cell	Percent	2021	4.77E-07	8.73E-07	2.18E-07
YLDs (Year	Saint Vinc	Male	55+ years	Non-melanoma skin cancer (basal-cell	Rate	2021	0.0147277	0.029210806	0.006130771
YLDs (Year	Saint Vinc	Female	55+ years	Non-melanoma skin cancer (basal-cell	Rate	2021	0.0059356	0.011671093	0.002359207
YLDs (Year	Saint Vinc	Both	55+ years	Non-melanoma skin cancer (basal-cell	Rate	2021	0.0104633	0.020769119	0.004333309
YLDs (Year	Chile	Male	55+ years	Non-melanoma skin cancer (basal-cell	Number	1990	0.4307847	0.844639646	0.174453585
YLDs (Year	Chile	Female	55+ years	Non-melanoma skin cancer (basal-cell	Number	1990	0.5049098	1.020108842	0.204926766
YLDs (Year	Chile	Both	55+ years	Non-melanoma skin cancer (basal-cell	Number	1990	0.9356945	1.796617625	0.390652909
YLDs (Year	Chile	Male	55+ years	Non-melanoma skin cancer (basal-cell	Percent	1990	2.80E-06	5.00E-06	1.23E-06
YLDs (Year	Chile	Female	55+ years	Non-melanoma skin cancer (basal-cell	Percent	1990	2.43E-06	4.67E-06	1.05E-06
YLDs (Year	Chile	Both	55+ years	Non-melanoma skin cancer (basal-cell	Percent	1990	2.59E-06	4.70E-06	1.19E-06
YLDs (Year	Chile	Male	55+ years	Non-melanoma skin cancer (basal-cell	Rate	1990	0.0568211	0.111409125	0.023010667
YLDs (Year	Chile	Female	55+ years	Non-melanoma skin cancer (basal-cell	Rate	1990	0.054669	0.110452092	0.022188407
YLDs (Year	Chile	Both	55+ years	Non-melanoma skin cancer (basal-cell	Rate	1990	0.0556392	0.106832271	0.023229393
YLDs (Year	Chile	Male	55+ years	Non-melanoma skin cancer (basal-cell	Number	2021	1.2409951	2.41687034	0.502867356
YLDs (Year	Chile	Female	55+ years	Non-melanoma skin cancer (basal-cell	Number	2021	1.3912469	2.735191094	0.57652359
YLDs (Year	Chile	Both	55+ years	Non-melanoma skin cancer (basal-cell	Number	2021	2.632242	4.99825566	1.076997207
YLDs (Year	Chile	Male	55+ years	Non-melanoma skin cancer (basal-cell	Percent	2021	2.97E-06	5.40E-06	1.29E-06
YLDs (Year	Chile	Female	55+ years	Non-melanoma skin cancer (basal-cell	Percent	2021	2.40E-06	4.56E-06	1.06E-06
YLDs (Year	Chile	Both	55+ years	Non-melanoma skin cancer (basal-cell	Percent	2021	2.64E-06	4.90E-06	1.20E-06
YLDs (Year	Chile	Male	55+ years	Non-melanoma skin cancer (basal-cell	Rate	2021	0.0611106	0.119014578	0.024762829
YLDs (Year	Chile	Female	55+ years	Non-melanoma skin cancer (basal-cell	Rate	2021	0.0568564	0.111779697	0.023560925
YLDs (Year	Chile	Both	55+ years	Non-melanoma skin cancer (basal-cell	Rate	2021	0.0587858	0.111625945	0.024052557
YLDs (Year	Denmark	Male	55+ years	Non-melanoma skin cancer (basal-cell	Number	1990	0.7978465	1.661730612	0.336275331
YLDs (Year	Denmark	Female	55+ years	Non-melanoma skin cancer (basal-cell	Number	1990	0.8530553	1.75108512	0.358397534
YLDs (Year	Denmark	Both	55+ years	Non-melanoma skin cancer (basal-cell	Number	1990	1.6509019	3.236298317	0.735121985
YLDs (Year	Denmark	Male	55+ years	Non-melanoma skin cancer (basal-cell	Percent	1990	6.87E-06	1.30E-05	3.19E-06
YLDs (Year	Denmark	Female	55+ years	Non-melanoma skin cancer (basal-cell	Percent	1990	4.93E-06	9.31E-06	2.16E-06
YLDs (Year	Denmark	Both	55+ years	Non-melanoma skin cancer (basal-cell	Percent	1990	5.71E-06	1.04E-05	2.73E-06
YLDs (Year	Denmark	Male	55+ years	Non-melanoma skin cancer (basal-cell	Rate	1990	0.1389013	0.289299488	0.058543954
YLDs (Year	Denmark	Female	55+ years	Non-melanoma skin cancer (basal-cell	Rate	1990	0.1168193	0.239797516	0.04907976
YLDs (Year	Denmark	Both	55+ years	Non-melanoma skin cancer (basal-cell	Rate	1990	0.1265415	0.248061982	0.056347036
YLDs (Year	Denmark	Male	55+ years	Non-melanoma skin cancer (basal-cell	Number	2021	1.1179443	2.264671299	0.456385323
YLDs (Year	Denmark	Female	55+ years	Non-melanoma skin cancer (basal-cell	Number	2021	0.9556945	1.961045139	0.389896269
YLDs (Year	Denmark	Both	55+ years	Non-melanoma skin cancer (basal-cell	Number	2021	2.0736388	4.048013263	0.901996029
YLDs (Year	Denmark	Male	55+ years	Non-melanoma skin cancer (basal-cell	Percent	2021	6.14E-06	1.21E-05	2.63E-06
YLDs (Year	Denmark	Female	55+ years	Non-melanoma skin cancer (basal-cell	Percent	2021	4.22E-06	8.33E-06	1.73E-06
YLDs (Year	Denmark	Both	55+ years	Non-melanoma skin cancer (basal-cell	Percent	2021	5.07E-06	9.56E-06	2.27E-06
YLDs (Year	Denmark	Male	55+ years	Non-melanoma skin cancer (basal-cell	Rate	2021	0.1221818	0.247509355	0.049879043
YLDs (Year	Denmark	Female	55+ years	Non-melanoma skin cancer (basal-cell	Rate	2021	0.0946009	0.194117036	0.038594475
YLDs (Year	Denmark	Both	55+ years	Non-melanoma skin cancer (basal-cell	Rate	2021	0.107709	0.210262081	0.046851517
YLDs (Year	Iceland	Male	55+ years	Non-melanoma skin cancer (basal-cell	Number	1990	0.0243797	0.049649635	0.00952019
YLDs (Year	Iceland	Female	55+ years	Non-melanoma skin cancer (basal-cell	Number	1990	0.0222619	0.045074837	0.009342108
YLDs (Year	Iceland	Both	55+ years	Non-melanoma skin cancer (basal-cell	Number	1990	0.0466416	0.093000041	0.01909807

YLDs (Year	Iceland	Male	55+ years	Non-melanoma skin cancer (basal-cell	Percent	1990	5.71E-06	1.11E-05	2.40E-06
YLDs (Year	Iceland	Female	55+ years	Non-melanoma skin cancer (basal-cell	Percent	1990	3.96E-06	7.69E-06	1.77E-06
YLDs (Year	Iceland	Both	55+ years	Non-melanoma skin cancer (basal-cell	Percent	1990	4.71E-06	8.68E-06	2.07E-06
YLDs (Year	Iceland	Male	55+ years	Non-melanoma skin cancer (basal-cell	Rate	1990	0.1093158	0.222623836	0.04268755
YLDs (Year	Iceland	Female	55+ years	Non-melanoma skin cancer (basal-cell	Rate	1990	0.0876014	0.177370774	0.036761463
YLDs (Year	Iceland	Both	55+ years	Non-melanoma skin cancer (basal-cell	Rate	1990	0.0977508	0.194908141	0.040025458
YLDs (Year	Iceland	Male	55+ years	Non-melanoma skin cancer (basal-cell	Number	2021	0.0535812	0.106628145	0.021596694
YLDs (Year	Iceland	Female	55+ years	Non-melanoma skin cancer (basal-cell	Number	2021	0.0445323	0.085213436	0.019870825
YLDs (Year	Iceland	Both	55+ years	Non-melanoma skin cancer (basal-cell	Number	2021	0.0981135	0.192143202	0.042500277
YLDs (Year	Iceland	Male	55+ years	Non-melanoma skin cancer (basal-cell	Percent	2021	5.93E-06	1.13E-05	2.54E-06
YLDs (Year	Iceland	Female	55+ years	Non-melanoma skin cancer (basal-cell	Percent	2021	4.07E-06	7.57E-06	1.74E-06
YLDs (Year	Iceland	Both	55+ years	Non-melanoma skin cancer (basal-cell	Percent	2021	4.91E-06	9.10E-06	2.18E-06
YLDs (Year	Iceland	Male	55+ years	Non-melanoma skin cancer (basal-cell	Rate	2021	0.1130117	0.224896437	0.045551008
YLDs (Year	Iceland	Female	55+ years	Non-melanoma skin cancer (basal-cell	Rate	2021	0.0891108	0.17051529	0.039762269
YLDs (Year	Iceland	Both	55+ years	Non-melanoma skin cancer (basal-cell	Rate	2021	0.1007469	0.197300271	0.043640972
YLDs (Year	Bahamas	Male	55+ years	Non-melanoma skin cancer (basal-cell	Number	1990	0.0015593	0.003010133	0.000656124
YLDs (Year	Bahamas	Female	55+ years	Non-melanoma skin cancer (basal-cell	Number	1990	0.0008486	0.001650861	0.000335532
YLDs (Year	Bahamas	Both	55+ years	Non-melanoma skin cancer (basal-cell	Number	1990	0.0024079	0.004632723	0.001001706
YLDs (Year	Bahamas	Male	55+ years	Non-melanoma skin cancer (basal-cell	Percent	1990	7.78E-07	1.44E-06	3.56E-07
YLDs (Year	Bahamas	Female	55+ years	Non-melanoma skin cancer (basal-cell	Percent	1990	3.03E-07	5.67E-07	1.33E-07
YLDs (Year	Bahamas	Both	55+ years	Non-melanoma skin cancer (basal-cell	Percent	1990	5.01E-07	9.29E-07	2.30E-07
YLDs (Year	Bahamas	Male	55+ years	Non-melanoma skin cancer (basal-cell	Rate	1990	0.0144482	0.027891687	0.006079601
YLDs (Year	Bahamas	Female	55+ years	Non-melanoma skin cancer (basal-cell	Rate	1990	0.0061655	0.011994824	0.002437908
YLDs (Year	Bahamas	Both	55+ years	Non-melanoma skin cancer (basal-cell	Rate	1990	0.0098058	0.018866462	0.004079382
YLDs (Year	Bahamas	Male	55+ years	Non-melanoma skin cancer (basal-cell	Number	2021	0.0044767	0.008665903	0.001900904
YLDs (Year	Bahamas	Female	55+ years	Non-melanoma skin cancer (basal-cell	Number	2021	0.0022637	0.00440827	0.000921314
YLDs (Year	Bahamas	Both	55+ years	Non-melanoma skin cancer (basal-cell	Number	2021	0.0067404	0.012981203	0.002876838
YLDs (Year	Bahamas	Male	55+ years	Non-melanoma skin cancer (basal-cell	Percent	2021	6.97E-07	1.27E-06	3.12E-07
YLDs (Year	Bahamas	Female	55+ years	Non-melanoma skin cancer (basal-cell	Percent	2021	2.71E-07	5.02E-07	1.18E-07
YLDs (Year	Bahamas	Both	55+ years	Non-melanoma skin cancer (basal-cell	Percent	2021	4.57E-07	8.39E-07	2.01E-07
YLDs (Year	Bahamas	Male	55+ years	Non-melanoma skin cancer (basal-cell	Rate	2021	0.01374	0.026597546	0.005834287
YLDs (Year	Bahamas	Female	55+ years	Non-melanoma skin cancer (basal-cell	Rate	2021	0.0057487	0.011195036	0.002339726
YLDs (Year	Bahamas	Both	55+ years	Non-melanoma skin cancer (basal-cell	Rate	2021	0.009367	0.018039823	0.003997907
YLDs (Year	El Salvado	Male	55+ years	Non-melanoma skin cancer (basal-cell	Number	1990	0.152035	0.298284766	0.062011103
YLDs (Year	El Salvado	Female	55+ years	Non-melanoma skin cancer (basal-cell	Number	1990	0.1667057	0.327918614	0.070864608
YLDs (Year	El Salvado	Both	55+ years	Non-melanoma skin cancer (basal-cell	Number	1990	0.3187408	0.610803099	0.132421189
YLDs (Year	El Salvado	Male	55+ years	Non-melanoma skin cancer (basal-cell	Percent	1990	3.18E-06	6.05E-06	1.40E-06
YLDs (Year	El Salvado	Female	55+ years	Non-melanoma skin cancer (basal-cell	Percent	1990	3.01E-06	5.48E-06	1.40E-06
YLDs (Year	El Salvado	Both	55+ years	Non-melanoma skin cancer (basal-cell	Percent	1990	3.09E-06	5.60E-06	1.38E-06
YLDs (Year	El Salvado	Male	55+ years	Non-melanoma skin cancer (basal-cell	Rate	1990	0.0669183	0.1312903	0.027294241
YLDs (Year	El Salvado	Female	55+ years	Non-melanoma skin cancer (basal-cell	Rate	1990	0.0636856	0.125272855	0.027071997
YLDs (Year	El Salvado	Both	55+ years	Non-melanoma skin cancer (basal-cell	Rate	1990	0.0651877	0.124919255	0.027082306
YLDs (Year	El Salvado	Male	55+ years	Non-melanoma skin cancer (basal-cell	Number	2021	0.3077697	0.595484986	0.12882799
YLDs (Year	El Salvado	Female	55+ years	Non-melanoma skin cancer (basal-cell	Number	2021	0.3943168	0.735293859	0.172023014
YLDs (Year	El Salvado	Both	55+ years	Non-melanoma skin cancer (basal-cell	Number	2021	0.7020865	1.304212648	0.304084597
YLDs (Year	El Salvado	Male	55+ years	Non-melanoma skin cancer (basal-cell	Percent	2021	3.15E-06	5.57E-06	1.42E-06
YLDs (Year	El Salvado	Female	55+ years	Non-melanoma skin cancer (basal-cell	Percent	2021	2.95E-06	5.32E-06	1.31E-06
YLDs (Year	El Salvado	Both	55+ years	Non-melanoma skin cancer (basal-cell	Percent	2021	3.03E-06	5.54E-06	1.35E-06
YLDs (Year	El Salvado	Male	55+ years	Non-melanoma skin cancer (basal-cell	Rate	2021	0.0721908	0.139677607	0.030218034
YLDs (Year	El Salvado	Female	55+ years	Non-melanoma skin cancer (basal-cell	Rate	2021	0.0661322	0.123318655	0.02885057
YLDs (Year	El Salvado	Both	55+ years	Non-melanoma skin cancer (basal-cell	Rate	2021	0.0686581	0.127540963	0.029736901
YLDs (Year	Peru	Male	55+ years	Non-melanoma skin cancer (basal-cell	Number	1990	0.3824285	0.761145233	0.17535336
YLDs (Year	Peru	Female	55+ years	Non-melanoma skin cancer (basal-cell	Number	1990	0.3803279	0.771110409	0.173085004
YLDs (Year	Peru	Both	55+ years	Non-melanoma skin cancer (basal-cell	Number	1990	0.7627564	1.516852329	0.349448515
YLDs (Year	Peru	Male	55+ years	Non-melanoma skin cancer (basal-cell	Percent	1990	2.13E-06	3.79E-06	1.04E-06
YLDs (Year	Peru	Female	55+ years	Non-melanoma skin cancer (basal-cell	Percent	1990	1.82E-06	3.26E-06	8.82E-07

YLDs (Year	Peru	Both	55+ years	Non-melanoma skin cancer (basal-cell	Percent	1990	1.96E-06	3.49E-06	9.53E-07
YLDs (Year	Peru	Male	55+ years	Non-melanoma skin cancer (basal-cell	Rate	1990	0.0395878	0.078791265	0.018152006
YLDs (Year	Peru	Female	55+ years	Non-melanoma skin cancer (basal-cell	Rate	1990	0.0378176	0.07667483	0.017210588
YLDs (Year	Peru	Both	55+ years	Non-melanoma skin cancer (basal-cell	Rate	1990	0.0386849	0.076930548	0.01772306
YLDs (Year	Peru	Male	55+ years	Non-melanoma skin cancer (basal-cell	Number	2021	0.9091864	1.802604845	0.376279706
YLDs (Year	Peru	Female	55+ years	Non-melanoma skin cancer (basal-cell	Number	2021	0.7933488	1.541451731	0.335422003
YLDs (Year	Peru	Both	55+ years	Non-melanoma skin cancer (basal-cell	Number	2021	1.7025352	3.325430913	0.729351384
YLDs (Year	Peru	Male	55+ years	Non-melanoma skin cancer (basal-cell	Percent	2021	1.77E-06	3.29E-06	8.28E-07
YLDs (Year	Peru	Female	55+ years	Non-melanoma skin cancer (basal-cell	Percent	2021	1.27E-06	2.34E-06	5.72E-07
YLDs (Year	Peru	Both	55+ years	Non-melanoma skin cancer (basal-cell	Percent	2021	1.50E-06	2.74E-06	6.85E-07
YLDs (Year	Peru	Male	55+ years	Non-melanoma skin cancer (basal-cell	Rate	2021	0.033826	0.067065333	0.013999365
YLDs (Year	Peru	Female	55+ years	Non-melanoma skin cancer (basal-cell	Rate	2021	0.027304	0.053050903	0.011543949
YLDs (Year	Peru	Both	55+ years	Non-melanoma skin cancer (basal-cell	Rate	2021	0.0304381	0.059452308	0.0130394
YLDs (Year	Colombia	Male	55+ years	Non-melanoma skin cancer (basal-cell	Number	1990	0.9472857	1.906090914	0.412930893
YLDs (Year	Colombia	Female	55+ years	Non-melanoma skin cancer (basal-cell	Number	1990	0.9219098	1.783222058	0.382177825
YLDs (Year	Colombia	Both	55+ years	Non-melanoma skin cancer (basal-cell	Number	1990	1.8691955	3.704220782	0.79007915
YLDs (Year	Colombia	Male	55+ years	Non-melanoma skin cancer (basal-cell	Percent	1990	3.34E-06	6.30E-06	1.58E-06
YLDs (Year	Colombia	Female	55+ years	Non-melanoma skin cancer (basal-cell	Percent	1990	2.97E-06	5.34E-06	1.31E-06
YLDs (Year	Colombia	Both	55+ years	Non-melanoma skin cancer (basal-cell	Percent	1990	3.14E-06	5.70E-06	1.44E-06
YLDs (Year	Colombia	Male	55+ years	Non-melanoma skin cancer (basal-cell	Rate	1990	0.0677593	0.136342608	0.02953693
YLDs (Year	Colombia	Female	55+ years	Non-melanoma skin cancer (basal-cell	Rate	1990	0.0622502	0.120408662	0.025805827
YLDs (Year	Colombia	Both	55+ years	Non-melanoma skin cancer (basal-cell	Rate	1990	0.0649254	0.128663877	0.027442923
YLDs (Year	Colombia	Male	55+ years	Non-melanoma skin cancer (basal-cell	Number	2021	3.0577637	6.242875531	1.237399673
YLDs (Year	Colombia	Female	55+ years	Non-melanoma skin cancer (basal-cell	Number	2021	3.4414106	6.502543621	1.482109179
YLDs (Year	Colombia	Both	55+ years	Non-melanoma skin cancer (basal-cell	Number	2021	6.4991743	12.61524003	2.713113639
YLDs (Year	Colombia	Male	55+ years	Non-melanoma skin cancer (basal-cell	Percent	2021	3.50E-06	6.66E-06	1.49E-06
YLDs (Year	Colombia	Female	55+ years	Non-melanoma skin cancer (basal-cell	Percent	2021	3.03E-06	5.43E-06	1.39E-06
YLDs (Year	Colombia	Both	55+ years	Non-melanoma skin cancer (basal-cell	Percent	2021	3.23E-06	6.01E-06	1.46E-06
YLDs (Year	Colombia	Male	55+ years	Non-melanoma skin cancer (basal-cell	Rate	2021	0.0710332	0.145024664	0.028745323
YLDs (Year	Colombia	Female	55+ years	Non-melanoma skin cancer (basal-cell	Rate	2021	0.0654409	0.123650619	0.028183389
YLDs (Year	Colombia	Both	55+ years	Non-melanoma skin cancer (basal-cell	Rate	2021	0.0679581	0.131910241	0.028369454
YLDs (Year	Guyana	Male	55+ years	Non-melanoma skin cancer (basal-cell	Number	1990	0.0037569	0.007330408	0.001573983
YLDs (Year	Guyana	Female	55+ years	Non-melanoma skin cancer (basal-cell	Number	1990	0.0015398	0.0030171	0.000623626
YLDs (Year	Guyana	Both	55+ years	Non-melanoma skin cancer (basal-cell	Number	1990	0.0052967	0.010207232	0.002221365
YLDs (Year	Guyana	Male	55+ years	Non-melanoma skin cancer (basal-cell	Percent	1990	5.88E-07	1.08E-06	2.74E-07
YLDs (Year	Guyana	Female	55+ years	Non-melanoma skin cancer (basal-cell	Percent	1990	2.08E-07	3.85E-07	9.24E-08
YLDs (Year	Guyana	Both	55+ years	Non-melanoma skin cancer (basal-cell	Percent	1990	3.84E-07	7.08E-07	1.76E-07
YLDs (Year	Guyana	Male	55+ years	Non-melanoma skin cancer (basal-cell	Rate	1990	0.0127499	0.02487771	0.005341733
YLDs (Year	Guyana	Female	55+ years	Non-melanoma skin cancer (basal-cell	Rate	1990	0.0048373	0.00947817	0.001959111
YLDs (Year	Guyana	Both	55+ years	Non-melanoma skin cancer (basal-cell	Rate	1990	0.0086409	0.016651858	0.003623887
YLDs (Year	Guyana	Male	55+ years	Non-melanoma skin cancer (basal-cell	Number	2021	0.0063828	0.012394431	0.002663093
YLDs (Year	Guyana	Female	55+ years	Non-melanoma skin cancer (basal-cell	Number	2021	0.0027531	0.00551545	0.00110714
YLDs (Year	Guyana	Both	55+ years	Non-melanoma skin cancer (basal-cell	Number	2021	0.0091359	0.017696731	0.003786116
YLDs (Year	Guyana	Male	55+ years	Non-melanoma skin cancer (basal-cell	Percent	2021	5.31E-07	9.57E-07	2.36E-07
YLDs (Year	Guyana	Female	55+ years	Non-melanoma skin cancer (basal-cell	Percent	2021	1.90E-07	3.55E-07	8.44E-08
YLDs (Year	Guyana	Both	55+ years	Non-melanoma skin cancer (basal-cell	Percent	2021	3.44E-07	6.19E-07	1.52E-07
YLDs (Year	Guyana	Male	55+ years	Non-melanoma skin cancer (basal-cell	Rate	2021	0.012124	0.023543072	0.005058514
YLDs (Year	Guyana	Female	55+ years	Non-melanoma skin cancer (basal-cell	Rate	2021	0.0045907	0.009196758	0.001846105
YLDs (Year	Guyana	Both	55+ years	Non-melanoma skin cancer (basal-cell	Rate	2021	0.0081124	0.015714023	0.003361926
YLDs (Year	Saint Luci	Male	55+ years	Non-melanoma skin cancer (basal-cell	Number	1990	0.0009988	0.001942078	0.00039937
YLDs (Year	Saint Luci	Female	55+ years	Non-melanoma skin cancer (basal-cell	Number	1990	0.0005244	0.001005252	0.000217347
YLDs (Year	Saint Luci	Both	55+ years	Non-melanoma skin cancer (basal-cell	Number	1990	0.0015231	0.002976869	0.000605563
YLDs (Year	Saint Luci	Male	55+ years	Non-melanoma skin cancer (basal-cell	Percent	1990	7.64E-07	1.38E-06	3.37E-07
YLDs (Year	Saint Luci	Female	55+ years	Non-melanoma skin cancer (basal-cell	Percent	1990	2.86E-07	5.31E-07	1.32E-07
YLDs (Year	Saint Luci	Both	55+ years	Non-melanoma skin cancer (basal-cell	Percent	1990	4.85E-07	8.78E-07	2.17E-07
YLDs (Year	Saint Luci	Male	55+ years	Non-melanoma skin cancer (basal-cell	Rate	1990	0.015513	0.030164792	0.006203098

YLDs (Year	Saint Luci	Female	55+ years	Non-melanoma skin cancer (basal-cell	Rate	1990	0.0064013	0.012272077	0.002653365
YLDs (Year	Saint Luci	Both	55+ years	Non-melanoma skin cancer (basal-cell	Rate	1990	0.0104112	0.020348259	0.004139298
YLDs (Year	Saint Luci	Male	55+ years	Non-melanoma skin cancer (basal-cell	Number	2021	0.0029827	0.005846334	0.001213176
YLDs (Year	Saint Luci	Female	55+ years	Non-melanoma skin cancer (basal-cell	Number	2021	0.0013643	0.002599462	0.000568059
YLDs (Year	Saint Luci	Both	55+ years	Non-melanoma skin cancer (basal-cell	Number	2021	0.0043469	0.008427127	0.001792977
YLDs (Year	Saint Luci	Male	55+ years	Non-melanoma skin cancer (basal-cell	Percent	2021	6.96E-07	1.29E-06	3.14E-07
YLDs (Year	Saint Luci	Female	55+ years	Non-melanoma skin cancer (basal-cell	Percent	2021	2.74E-07	5.03E-07	1.23E-07
YLDs (Year	Saint Luci	Both	55+ years	Non-melanoma skin cancer (basal-cell	Percent	2021	4.69E-07	8.67E-07	2.12E-07
YLDs (Year	Saint Luci	Male	55+ years	Non-melanoma skin cancer (basal-cell	Rate	2021	0.0147967	0.029003247	0.00601848
YLDs (Year	Saint Luci	Female	55+ years	Non-melanoma skin cancer (basal-cell	Rate	2021	0.0062226	0.011856503	0.002590995
YLDs (Year	Saint Luci	Both	55+ years	Non-melanoma skin cancer (basal-cell	Rate	2021	0.0103297	0.020025551	0.004260687
YLDs (Year	Belize	Male	55+ years	Non-melanoma skin cancer (basal-cell	Number	1990	0.0012443	0.002407167	0.000520756
YLDs (Year	Belize	Female	55+ years	Non-melanoma skin cancer (basal-cell	Number	1990	0.000483	0.000935442	0.000195928
YLDs (Year	Belize	Both	55+ years	Non-melanoma skin cancer (basal-cell	Number	1990	0.0017273	0.003315757	0.000722728
YLDs (Year	Belize	Male	55+ years	Non-melanoma skin cancer (basal-cell	Percent	1990	8.89E-07	1.62E-06	4.08E-07
YLDs (Year	Belize	Female	55+ years	Non-melanoma skin cancer (basal-cell	Percent	1990	3.11E-07	5.61E-07	1.36E-07
YLDs (Year	Belize	Both	55+ years	Non-melanoma skin cancer (basal-cell	Percent	1990	5.85E-07	1.07E-06	2.63E-07
YLDs (Year	Belize	Male	55+ years	Non-melanoma skin cancer (basal-cell	Rate	1990	0.0160685	0.031085665	0.006724942
YLDs (Year	Belize	Female	55+ years	Non-melanoma skin cancer (basal-cell	Rate	1990	0.0063143	0.012229711	0.002561512
YLDs (Year	Belize	Both	55+ years	Non-melanoma skin cancer (basal-cell	Rate	1990	0.0112215	0.021541258	0.004695299
YLDs (Year	Belize	Male	55+ years	Non-melanoma skin cancer (basal-cell	Number	2021	0.0038352	0.007404459	0.001598707
YLDs (Year	Belize	Female	55+ years	Non-melanoma skin cancer (basal-cell	Number	2021	0.0013949	0.00276323	0.000544824
YLDs (Year	Belize	Both	55+ years	Non-melanoma skin cancer (basal-cell	Number	2021	0.0052302	0.010082336	0.002174645
YLDs (Year	Belize	Male	55+ years	Non-melanoma skin cancer (basal-cell	Percent	2021	7.67E-07	1.40E-06	3.45E-07
YLDs (Year	Belize	Female	55+ years	Non-melanoma skin cancer (basal-cell	Percent	2021	2.68E-07	5.09E-07	1.18E-07
YLDs (Year	Belize	Both	55+ years	Non-melanoma skin cancer (basal-cell	Percent	2021	5.13E-07	9.43E-07	2.30E-07
YLDs (Year	Belize	Male	55+ years	Non-melanoma skin cancer (basal-cell	Rate	2021	0.0150463	0.029048835	0.006271973
YLDs (Year	Belize	Female	55+ years	Non-melanoma skin cancer (basal-cell	Rate	2021	0.0056712	0.01123415	0.00221503
YLDs (Year	Belize	Both	55+ years	Non-melanoma skin cancer (basal-cell	Rate	2021	0.0104423	0.020129892	0.004341789
YLDs (Year	Grenada	Male	55+ years	Non-melanoma skin cancer (basal-cell	Number	1990	0.0007393	0.001404146	0.00031276
YLDs (Year	Grenada	Female	55+ years	Non-melanoma skin cancer (basal-cell	Number	1990	0.0004478	0.000846868	0.000183333
YLDs (Year	Grenada	Both	55+ years	Non-melanoma skin cancer (basal-cell	Number	1990	0.0011871	0.002247509	0.000486523
YLDs (Year	Grenada	Male	55+ years	Non-melanoma skin cancer (basal-cell	Percent	1990	7.24E-07	1.31E-06	3.38E-07
YLDs (Year	Grenada	Female	55+ years	Non-melanoma skin cancer (basal-cell	Percent	1990	2.94E-07	5.52E-07	1.31E-07
YLDs (Year	Grenada	Both	55+ years	Non-melanoma skin cancer (basal-cell	Percent	1990	4.67E-07	8.70E-07	2.13E-07
YLDs (Year	Grenada	Male	55+ years	Non-melanoma skin cancer (basal-cell	Rate	1990	0.0145902	0.02771066	0.006172275
YLDs (Year	Grenada	Female	55+ years	Non-melanoma skin cancer (basal-cell	Rate	1990	0.0064045	0.012111896	0.002622029
YLDs (Year	Grenada	Both	55+ years	Non-melanoma skin cancer (basal-cell	Rate	1990	0.0098441	0.018637286	0.004034454
YLDs (Year	Grenada	Male	55+ years	Non-melanoma skin cancer (basal-cell	Number	2021	0.0012274	0.002447324	0.000499161
YLDs (Year	Grenada	Female	55+ years	Non-melanoma skin cancer (basal-cell	Number	2021	0.0005832	0.001131015	0.000241346
YLDs (Year	Grenada	Both	55+ years	Non-melanoma skin cancer (basal-cell	Number	2021	0.0018106	0.003530502	0.000732133
YLDs (Year	Grenada	Male	55+ years	Non-melanoma skin cancer (basal-cell	Percent	2021	6.04E-07	1.12E-06	2.74E-07
YLDs (Year	Grenada	Female	55+ years	Non-melanoma skin cancer (basal-cell	Percent	2021	2.53E-07	4.58E-07	1.11E-07
YLDs (Year	Grenada	Both	55+ years	Non-melanoma skin cancer (basal-cell	Percent	2021	4.18E-07	7.78E-07	1.87E-07
YLDs (Year	Grenada	Male	55+ years	Non-melanoma skin cancer (basal-cell	Rate	2021	0.0125626	0.025048697	0.00510898
YLDs (Year	Grenada	Female	55+ years	Non-melanoma skin cancer (basal-cell	Rate	2021	0.0056533	0.010962767	0.002339328
YLDs (Year	Grenada	Both	55+ years	Non-melanoma skin cancer (basal-cell	Rate	2021	0.0090139	0.017575931	0.003644785
YLDs (Year	Haiti	Male	55+ years	Non-melanoma skin cancer (basal-cell	Number	1990	0.0322715	0.064841836	0.013289144
YLDs (Year	Haiti	Female	55+ years	Non-melanoma skin cancer (basal-cell	Number	1990	0.0116342	0.022682418	0.004645789
YLDs (Year	Haiti	Both	55+ years	Non-melanoma skin cancer (basal-cell	Number	1990	0.0439057	0.087251133	0.017975677
YLDs (Year	Haiti	Male	55+ years	Non-melanoma skin cancer (basal-cell	Percent	1990	6.02E-07	1.09E-06	2.73E-07
YLDs (Year	Haiti	Female	55+ years	Non-melanoma skin cancer (basal-cell	Percent	1990	1.91E-07	3.58E-07	8.37E-08
YLDs (Year	Haiti	Both	55+ years	Non-melanoma skin cancer (basal-cell	Percent	1990	3.83E-07	7.01E-07	1.70E-07
YLDs (Year	Haiti	Male	55+ years	Non-melanoma skin cancer (basal-cell	Rate	1990	0.0121658	0.02444429	0.005009785
YLDs (Year	Haiti	Female	55+ years	Non-melanoma skin cancer (basal-cell	Rate	1990	0.0042446	0.008275509	0.001694981
YLDs (Year	Haiti	Both	55+ years	Non-melanoma skin cancer (basal-cell	Rate	1990	0.0081404	0.016176951	0.003332812

YLDs (Year	Haiti	Male	55+ years	Non-melanoma skin cancer (basal-cell	Number	2021	0.0694569	0.135438445	0.028478146
YLDs (Year	Haiti	Female	55+ years	Non-melanoma skin cancer (basal-cell	Number	2021	0.025413	0.050945152	0.01010288
YLDs (Year	Haiti	Both	55+ years	Non-melanoma skin cancer (basal-cell	Number	2021	0.0948699	0.186665825	0.038501639
YLDs (Year	Haiti	Male	55+ years	Non-melanoma skin cancer (basal-cell	Percent	2021	5.99E-07	1.09E-06	2.67E-07
YLDs (Year	Haiti	Female	55+ years	Non-melanoma skin cancer (basal-cell	Percent	2021	1.76E-07	3.25E-07	7.56E-08
YLDs (Year	Haiti	Both	55+ years	Non-melanoma skin cancer (basal-cell	Percent	2021	3.64E-07	6.70E-07	1.61E-07
YLDs (Year	Haiti	Male	55+ years	Non-melanoma skin cancer (basal-cell	Rate	2021	0.0123461	0.02407437	0.00506203
YLDs (Year	Haiti	Female	55+ years	Non-melanoma skin cancer (basal-cell	Rate	2021	0.0040824	0.008183898	0.00162294
YLDs (Year	Haiti	Both	55+ years	Non-melanoma skin cancer (basal-cell	Rate	2021	0.0080053	0.015751217	0.003248841
YLDs (Year	Trinidad a	Male	55+ years	Non-melanoma skin cancer (basal-cell	Number	1990	0.0075879	0.014816977	0.00334716
YLDs (Year	Trinidad a	Female	55+ years	Non-melanoma skin cancer (basal-cell	Number	1990	0.0045141	0.008601493	0.001874868
YLDs (Year	Trinidad a	Both	55+ years	Non-melanoma skin cancer (basal-cell	Number	1990	0.012102	0.023752819	0.005194798
YLDs (Year	Trinidad a	Male	55+ years	Non-melanoma skin cancer (basal-cell	Percent	1990	5.73E-07	1.01E-06	2.68E-07
YLDs (Year	Trinidad a	Female	55+ years	Non-melanoma skin cancer (basal-cell	Percent	1990	2.86E-07	5.17E-07	1.31E-07
YLDs (Year	Trinidad a	Both	55+ years	Non-melanoma skin cancer (basal-cell	Percent	1990	4.17E-07	7.48E-07	1.95E-07
YLDs (Year	Trinidad a	Male	55+ years	Non-melanoma skin cancer (basal-cell	Rate	1990	0.011623	0.02269631	0.005127104
YLDs (Year	Trinidad a	Female	55+ years	Non-melanoma skin cancer (basal-cell	Rate	1990	0.0062682	0.011943823	0.002603395
YLDs (Year	Trinidad a	Both	55+ years	Non-melanoma skin cancer (basal-cell	Rate	1990	0.0088143	0.017299956	0.003783542
YLDs (Year	Trinidad a	Male	55+ years	Non-melanoma skin cancer (basal-cell	Number	2021	0.0208793	0.040452771	0.008643731
YLDs (Year	Trinidad a	Female	55+ years	Non-melanoma skin cancer (basal-cell	Number	2021	0.0125242	0.024608402	0.005086783
YLDs (Year	Trinidad a	Both	55+ years	Non-melanoma skin cancer (basal-cell	Number	2021	0.0334035	0.064546469	0.014071877
YLDs (Year	Trinidad a	Male	55+ years	Non-melanoma skin cancer (basal-cell	Percent	2021	5.67E-07	1.04E-06	2.52E-07
YLDs (Year	Trinidad a	Female	55+ years	Non-melanoma skin cancer (basal-cell	Percent	2021	3.01E-07	5.57E-07	1.38E-07
YLDs (Year	Trinidad a	Both	55+ years	Non-melanoma skin cancer (basal-cell	Percent	2021	4.26E-07	7.76E-07	1.89E-07
YLDs (Year	Trinidad a	Male	55+ years	Non-melanoma skin cancer (basal-cell	Rate	2021	0.0125135	0.024244447	0.005180424
YLDs (Year	Trinidad a	Female	55+ years	Non-melanoma skin cancer (basal-cell	Rate	2021	0.0069009	0.013559466	0.002802866
YLDs (Year	Trinidad a	Both	55+ years	Non-melanoma skin cancer (basal-cell	Rate	2021	0.0095894	0.018529796	0.00403971
YLDs (Year	Nicaragua	Male	55+ years	Non-melanoma skin cancer (basal-cell	Number	1990	0.0767836	0.15725145	0.03288174
YLDs (Year	Nicaragua	Female	55+ years	Non-melanoma skin cancer (basal-cell	Number	1990	0.0836149	0.162149778	0.033594003
YLDs (Year	Nicaragua	Both	55+ years	Non-melanoma skin cancer (basal-cell	Number	1990	0.1603985	0.321119051	0.066180636
YLDs (Year	Nicaragua	Male	55+ years	Non-melanoma skin cancer (basal-cell	Percent	1990	3.25E-06	6.08E-06	1.48E-06
YLDs (Year	Nicaragua	Female	55+ years	Non-melanoma skin cancer (basal-cell	Percent	1990	2.98E-06	5.60E-06	1.31E-06
YLDs (Year	Nicaragua	Both	55+ years	Non-melanoma skin cancer (basal-cell	Percent	1990	3.10E-06	5.68E-06	1.43E-06
YLDs (Year	Nicaragua	Male	55+ years	Non-melanoma skin cancer (basal-cell	Rate	1990	0.0655766	0.134299749	0.028082472
YLDs (Year	Nicaragua	Female	55+ years	Non-melanoma skin cancer (basal-cell	Rate	1990	0.0636413	0.123416049	0.025569194
YLDs (Year	Nicaragua	Both	55+ years	Non-melanoma skin cancer (basal-cell	Rate	1990	0.0645533	0.129236177	0.026634771
YLDs (Year	Nicaragua	Male	55+ years	Non-melanoma skin cancer (basal-cell	Number	2021	0.2456292	0.505447437	0.102337936
YLDs (Year	Nicaragua	Female	55+ years	Non-melanoma skin cancer (basal-cell	Number	2021	0.2877376	0.541210902	0.122421731
YLDs (Year	Nicaragua	Both	55+ years	Non-melanoma skin cancer (basal-cell	Number	2021	0.5333669	1.051322784	0.226575472
YLDs (Year	Nicaragua	Male	55+ years	Non-melanoma skin cancer (basal-cell	Percent	2021	3.01E-06	5.75E-06	1.35E-06
YLDs (Year	Nicaragua	Female	55+ years	Non-melanoma skin cancer (basal-cell	Percent	2021	2.82E-06	5.10E-06	1.26E-06
YLDs (Year	Nicaragua	Both	55+ years	Non-melanoma skin cancer (basal-cell	Percent	2021	2.90E-06	5.32E-06	1.35E-06
YLDs (Year	Nicaragua	Male	55+ years	Non-melanoma skin cancer (basal-cell	Rate	2021	0.0671663	0.138212455	0.027983874
YLDs (Year	Nicaragua	Female	55+ years	Non-melanoma skin cancer (basal-cell	Rate	2021	0.0641798	0.120717007	0.027306148
YLDs (Year	Nicaragua	Both	55+ years	Non-melanoma skin cancer (basal-cell	Rate	2021	0.0655215	0.129149813	0.027833678
YLDs (Year	Suriname	Male	55+ years	Non-melanoma skin cancer (basal-cell	Number	1990	0.0030335	0.00590592	0.001230249
YLDs (Year	Suriname	Female	55+ years	Non-melanoma skin cancer (basal-cell	Number	1990	0.0012584	0.002406076	0.000521019
YLDs (Year	Suriname	Both	55+ years	Non-melanoma skin cancer (basal-cell	Number	1990	0.0042919	0.008287845	0.001744491
YLDs (Year	Suriname	Male	55+ years	Non-melanoma skin cancer (basal-cell	Percent	1990	7.50E-07	1.39E-06	3.45E-07
YLDs (Year	Suriname	Female	55+ years	Non-melanoma skin cancer (basal-cell	Percent	1990	2.72E-07	5.03E-07	1.24E-07
YLDs (Year	Suriname	Both	55+ years	Non-melanoma skin cancer (basal-cell	Percent	1990	4.95E-07	9.24E-07	2.23E-07
YLDs (Year	Suriname	Male	55+ years	Non-melanoma skin cancer (basal-cell	Rate	1990	0.0142777	0.027797131	0.00579036
YLDs (Year	Suriname	Female	55+ years	Non-melanoma skin cancer (basal-cell	Rate	1990	0.0056506	0.010803818	0.002339494
YLDs (Year	Suriname	Both	55+ years	Non-melanoma skin cancer (basal-cell	Rate	1990	0.0098627	0.019045023	0.004008748
YLDs (Year	Suriname	Male	55+ years	Non-melanoma skin cancer (basal-cell	Number	2021	0.0073082	0.014082332	0.003015884
YLDs (Year	Suriname	Female	55+ years	Non-melanoma skin cancer (basal-cell	Number	2021	0.0035242	0.006749261	0.001458231

YLDs (Year	Suriname	Both	55+ years	Non-melanoma skin cancer (basal-cell	Number	2021	0.0108324	0.020930202	0.00449379
YLDs (Year	Suriname	Male	55+ years	Non-melanoma skin cancer (basal-cell	Percent	2021	6.59E-07	1.20E-06	3.03E-07
YLDs (Year	Suriname	Female	55+ years	Non-melanoma skin cancer (basal-cell	Percent	2021	2.57E-07	4.68E-07	1.14E-07
YLDs (Year	Suriname	Both	55+ years	Non-melanoma skin cancer (basal-cell	Percent	2021	4.36E-07	7.94E-07	1.94E-07
YLDs (Year	Suriname	Male	55+ years	Non-melanoma skin cancer (basal-cell	Rate	2021	0.0141114	0.027191522	0.00582336
YLDs (Year	Suriname	Female	55+ years	Non-melanoma skin cancer (basal-cell	Rate	2021	0.0058821	0.011265052	0.002433903
YLDs (Year	Suriname	Both	55+ years	Non-melanoma skin cancer (basal-cell	Rate	2021	0.0096975	0.018737419	0.004022992
YLDs (Year	Switzerlan	Male	55+ years	Non-melanoma skin cancer (basal-cell	Number	1990	1.5817229	3.103648679	0.688084048
YLDs (Year	Switzerlan	Female	55+ years	Non-melanoma skin cancer (basal-cell	Number	1990	1.7183456	3.478269406	0.739704595
YLDs (Year	Switzerlan	Both	55+ years	Non-melanoma skin cancer (basal-cell	Number	1990	3.3000685	6.451639658	1.454216622
YLDs (Year	Switzerlan	Male	55+ years	Non-melanoma skin cancer (basal-cell	Percent	1990	1.03E-05	1.77E-05	4.87E-06
YLDs (Year	Switzerlan	Female	55+ years	Non-melanoma skin cancer (basal-cell	Percent	1990	7.64E-06	1.43E-05	3.44E-06
YLDs (Year	Switzerlan	Both	55+ years	Non-melanoma skin cancer (basal-cell	Percent	1990	8.72E-06	1.58E-05	4.10E-06
YLDs (Year	Switzerlan	Male	55+ years	Non-melanoma skin cancer (basal-cell	Rate	1990	0.2174959	0.426769348	0.094615471
YLDs (Year	Switzerlan	Female	55+ years	Non-melanoma skin cancer (basal-cell	Rate	1990	0.1786364	0.361595159	0.076898471
YLDs (Year	Switzerlan	Both	55+ years	Non-melanoma skin cancer (basal-cell	Rate	1990	0.1953667	0.381942242	0.08609079
YLDs (Year	Switzerlan	Male	55+ years	Non-melanoma skin cancer (basal-cell	Number	2021	2.0693991	4.24938268	0.844930205
YLDs (Year	Switzerlan	Female	55+ years	Non-melanoma skin cancer (basal-cell	Number	2021	1.8339109	3.702744681	0.734253673
YLDs (Year	Switzerlan	Both	55+ years	Non-melanoma skin cancer (basal-cell	Number	2021	3.90331	7.577409141	1.64498323
YLDs (Year	Switzerlan	Male	55+ years	Non-melanoma skin cancer (basal-cell	Percent	2021	7.16E-06	1.37E-05	3.17E-06
YLDs (Year	Switzerlan	Female	55+ years	Non-melanoma skin cancer (basal-cell	Percent	2021	4.96E-06	9.54E-06	2.13E-06
YLDs (Year	Switzerlan	Both	55+ years	Non-melanoma skin cancer (basal-cell	Percent	2021	5.92E-06	1.10E-05	2.67E-06
YLDs (Year	Switzerlan	Male	55+ years	Non-melanoma skin cancer (basal-cell	Rate	2021	0.1492247	0.306423709	0.060928061
YLDs (Year	Switzerlan	Female	55+ years	Non-melanoma skin cancer (basal-cell	Rate	2021	0.1187714	0.239804523	0.047553198
YLDs (Year	Switzerlan	Both	55+ years	Non-melanoma skin cancer (basal-cell	Rate	2021	0.1331808	0.25854098	0.05612678
YLDs (Year	Honduras	Male	55+ years	Non-melanoma skin cancer (basal-cell	Number	1990	0.1001839	0.201375056	0.044657236
YLDs (Year	Honduras	Female	55+ years	Non-melanoma skin cancer (basal-cell	Number	1990	0.0934972	0.178101274	0.040802592
YLDs (Year	Honduras	Both	55+ years	Non-melanoma skin cancer (basal-cell	Number	1990	0.1936811	0.375409205	0.087523979
YLDs (Year	Honduras	Male	55+ years	Non-melanoma skin cancer (basal-cell	Percent	1990	3.11E-06	6.01E-06	1.43E-06
YLDs (Year	Honduras	Female	55+ years	Non-melanoma skin cancer (basal-cell	Percent	1990	2.61E-06	4.73E-06	1.22E-06
YLDs (Year	Honduras	Both	55+ years	Non-melanoma skin cancer (basal-cell	Percent	1990	2.85E-06	5.37E-06	1.33E-06
YLDs (Year	Honduras	Male	55+ years	Non-melanoma skin cancer (basal-cell	Rate	1990	0.0614926	0.123603408	0.027410478
YLDs (Year	Honduras	Female	55+ years	Non-melanoma skin cancer (basal-cell	Rate	1990	0.0548707	0.10452227	0.023945811
YLDs (Year	Honduras	Both	55+ years	Non-melanoma skin cancer (basal-cell	Rate	1990	0.0581074	0.112628673	0.026258572
YLDs (Year	Honduras	Male	55+ years	Non-melanoma skin cancer (basal-cell	Number	2021	0.2999392	0.596323701	0.126770917
YLDs (Year	Honduras	Female	55+ years	Non-melanoma skin cancer (basal-cell	Number	2021	0.3055895	0.567232623	0.134135055
YLDs (Year	Honduras	Both	55+ years	Non-melanoma skin cancer (basal-cell	Number	2021	0.6055287	1.143767886	0.258544331
YLDs (Year	Honduras	Male	55+ years	Non-melanoma skin cancer (basal-cell	Percent	2021	2.83E-06	5.24E-06	1.30E-06
YLDs (Year	Honduras	Female	55+ years	Non-melanoma skin cancer (basal-cell	Percent	2021	2.37E-06	4.31E-06	1.05E-06
YLDs (Year	Honduras	Both	55+ years	Non-melanoma skin cancer (basal-cell	Percent	2021	2.58E-06	4.72E-06	1.15E-06
YLDs (Year	Honduras	Male	55+ years	Non-melanoma skin cancer (basal-cell	Rate	2021	0.0611851	0.121645161	0.025860231
YLDs (Year	Honduras	Female	55+ years	Non-melanoma skin cancer (basal-cell	Rate	2021	0.0544283	0.101029286	0.023890673
YLDs (Year	Honduras	Both	55+ years	Non-melanoma skin cancer (basal-cell	Rate	2021	0.0575779	0.108757364	0.024584184
YLDs (Year	Mexico	Male	55+ years	Non-melanoma skin cancer (basal-cell	Number	1990	2.1936333	4.132181281	0.962036018
YLDs (Year	Mexico	Female	55+ years	Non-melanoma skin cancer (basal-cell	Number	1990	2.1453851	4.137277533	0.898407561
YLDs (Year	Mexico	Both	55+ years	Non-melanoma skin cancer (basal-cell	Number	1990	4.3390184	8.286230339	1.870674981
YLDs (Year	Mexico	Male	55+ years	Non-melanoma skin cancer (basal-cell	Percent	1990	2.97E-06	5.34E-06	1.39E-06
YLDs (Year	Mexico	Female	55+ years	Non-melanoma skin cancer (basal-cell	Percent	1990	2.54E-06	4.78E-06	1.16E-06
YLDs (Year	Mexico	Both	55+ years	Non-melanoma skin cancer (basal-cell	Percent	1990	2.74E-06	5.01E-06	1.27E-06
YLDs (Year	Mexico	Male	55+ years	Non-melanoma skin cancer (basal-cell	Rate	1990	0.0656528	0.123671135	0.028792562
YLDs (Year	Mexico	Female	55+ years	Non-melanoma skin cancer (basal-cell	Rate	1990	0.0594047	0.114559252	0.024876479
YLDs (Year	Mexico	Both	55+ years	Non-melanoma skin cancer (basal-cell	Rate	1990	0.0624073	0.119179355	0.026905581
YLDs (Year	Mexico	Male	55+ years	Non-melanoma skin cancer (basal-cell	Number	2021	6.6849385	12.82619674	2.866581767
YLDs (Year	Mexico	Female	55+ years	Non-melanoma skin cancer (basal-cell	Number	2021	7.098429	13.70487033	2.995969821
YLDs (Year	Mexico	Both	55+ years	Non-melanoma skin cancer (basal-cell	Number	2021	13.783367	26.42947138	5.839175222
YLDs (Year	Mexico	Male	55+ years	Non-melanoma skin cancer (basal-cell	Percent	2021	3.10E-06	5.58E-06	1.48E-06

YLDs (Year	Mexico	Female	55+ years	Non-melanoma skin cancer (basal-cell	Percent	2021	2.61E-06	4.84E-06	1.20E-06
YLDs (Year	Mexico	Both	55+ years	Non-melanoma skin cancer (basal-cell	Percent	2021	2.83E-06	5.13E-06	1.33E-06
YLDs (Year	Mexico	Male	55+ years	Non-melanoma skin cancer (basal-cell	Rate	2021	0.066781	0.128130847	0.028636513
YLDs (Year	Mexico	Female	55+ years	Non-melanoma skin cancer (basal-cell	Rate	2021	0.0615104	0.118757538	0.025961136
YLDs (Year	Mexico	Both	55+ years	Non-melanoma skin cancer (basal-cell	Rate	2021	0.0639586	0.12264003	0.027095382
YLDs (Year	Ecuador	Male	55+ years	Non-melanoma skin cancer (basal-cell	Number	1990	0.2528886	0.501364087	0.11507507
YLDs (Year	Ecuador	Female	55+ years	Non-melanoma skin cancer (basal-cell	Number	1990	0.2661123	0.531944648	0.120143498
YLDs (Year	Ecuador	Both	55+ years	Non-melanoma skin cancer (basal-cell	Number	1990	0.519001	1.024975059	0.235784956
YLDs (Year	Ecuador	Male	55+ years	Non-melanoma skin cancer (basal-cell	Percent	1990	3.07E-06	5.50E-06	1.53E-06
YLDs (Year	Ecuador	Female	55+ years	Non-melanoma skin cancer (basal-cell	Percent	1990	2.93E-06	5.34E-06	1.40E-06
YLDs (Year	Ecuador	Both	55+ years	Non-melanoma skin cancer (basal-cell	Percent	1990	3.00E-06	5.31E-06	1.48E-06
YLDs (Year	Ecuador	Male	55+ years	Non-melanoma skin cancer (basal-cell	Rate	1990	0.0599359	0.118825841	0.027273377
YLDs (Year	Ecuador	Female	55+ years	Non-melanoma skin cancer (basal-cell	Rate	1990	0.060769	0.12147411	0.027435795
YLDs (Year	Ecuador	Both	55+ years	Non-melanoma skin cancer (basal-cell	Rate	1990	0.0603602	0.119205366	0.027421967
YLDs (Year	Ecuador	Male	55+ years	Non-melanoma skin cancer (basal-cell	Number	2021	0.5862835	1.152088887	0.247113669
YLDs (Year	Ecuador	Female	55+ years	Non-melanoma skin cancer (basal-cell	Number	2021	0.5386815	1.079125227	0.224937341
YLDs (Year	Ecuador	Both	55+ years	Non-melanoma skin cancer (basal-cell	Number	2021	1.124965	2.250140697	0.477720437
YLDs (Year	Ecuador	Male	55+ years	Non-melanoma skin cancer (basal-cell	Percent	2021	2.15E-06	3.92E-06	9.92E-07
YLDs (Year	Ecuador	Female	55+ years	Non-melanoma skin cancer (basal-cell	Percent	2021	1.66E-06	3.05E-06	7.36E-07
YLDs (Year	Ecuador	Both	55+ years	Non-melanoma skin cancer (basal-cell	Percent	2021	1.88E-06	3.42E-06	8.59E-07
YLDs (Year	Ecuador	Male	55+ years	Non-melanoma skin cancer (basal-cell	Rate	2021	0.0445739	0.087590872	0.018787528
YLDs (Year	Ecuador	Female	55+ years	Non-melanoma skin cancer (basal-cell	Rate	2021	0.0370938	0.074309043	0.015489285
YLDs (Year	Ecuador	Both	55+ years	Non-melanoma skin cancer (basal-cell	Rate	2021	0.0406489	0.081305323	0.017261683
YLDs (Year	Brazil	Male	55+ years	Non-melanoma skin cancer (basal-cell	Number	1990	9.5807043	18.15150401	4.156292902
YLDs (Year	Brazil	Female	55+ years	Non-melanoma skin cancer (basal-cell	Number	1990	8.2461147	15.90644212	3.791684099
YLDs (Year	Brazil	Both	55+ years	Non-melanoma skin cancer (basal-cell	Number	1990	17.826819	33.19584819	7.921078337
YLDs (Year	Brazil	Male	55+ years	Non-melanoma skin cancer (basal-cell	Percent	1990	6.30E-06	1.09E-05	2.98E-06
YLDs (Year	Brazil	Female	55+ years	Non-melanoma skin cancer (basal-cell	Percent	1990	4.35E-06	7.90E-06	2.16E-06
YLDs (Year	Brazil	Both	55+ years	Non-melanoma skin cancer (basal-cell	Percent	1990	5.22E-06	8.94E-06	2.53E-06
YLDs (Year	Brazil	Male	55+ years	Non-melanoma skin cancer (basal-cell	Rate	1990	0.1388187	0.263004429	0.060222196
YLDs (Year	Brazil	Female	55+ years	Non-melanoma skin cancer (basal-cell	Rate	1990	0.1046863	0.201935905	0.048136293
YLDs (Year	Brazil	Both	55+ years	Non-melanoma skin cancer (basal-cell	Rate	1990	0.1206261	0.224621487	0.053598401
YLDs (Year	Brazil	Male	55+ years	Non-melanoma skin cancer (basal-cell	Number	2021	15.566592	30.13504353	6.879095073
YLDs (Year	Brazil	Female	55+ years	Non-melanoma skin cancer (basal-cell	Number	2021	15.981812	30.82841074	6.926608416
YLDs (Year	Brazil	Both	55+ years	Non-melanoma skin cancer (basal-cell	Number	2021	31.548404	61.05747604	13.73966898
YLDs (Year	Brazil	Male	55+ years	Non-melanoma skin cancer (basal-cell	Percent	2021	3.54E-06	6.38E-06	1.71E-06
YLDs (Year	Brazil	Female	55+ years	Non-melanoma skin cancer (basal-cell	Percent	2021	2.70E-06	4.86E-06	1.27E-06
YLDs (Year	Brazil	Both	55+ years	Non-melanoma skin cancer (basal-cell	Percent	2021	3.06E-06	5.44E-06	1.45E-06
YLDs (Year	Brazil	Male	55+ years	Non-melanoma skin cancer (basal-cell	Rate	2021	0.0798488	0.154577701	0.035286317
YLDs (Year	Brazil	Female	55+ years	Non-melanoma skin cancer (basal-cell	Rate	2021	0.0671147	0.129462215	0.029087911
YLDs (Year	Brazil	Both	55+ years	Non-melanoma skin cancer (basal-cell	Rate	2021	0.072847	0.140985108	0.03172566
YLDs (Year	Guatemala	Male	55+ years	Non-melanoma skin cancer (basal-cell	Number	1990	0.1649057	0.341122453	0.070038938
YLDs (Year	Guatemala	Female	55+ years	Non-melanoma skin cancer (basal-cell	Number	1990	0.1518588	0.292665336	0.065681985
YLDs (Year	Guatemala	Both	55+ years	Non-melanoma skin cancer (basal-cell	Number	1990	0.3167646	0.626024347	0.137860528
YLDs (Year	Guatemala	Male	55+ years	Non-melanoma skin cancer (basal-cell	Percent	1990	2.78E-06	5.24E-06	1.29E-06
YLDs (Year	Guatemala	Female	55+ years	Non-melanoma skin cancer (basal-cell	Percent	1990	2.43E-06	4.56E-06	1.13E-06
YLDs (Year	Guatemala	Both	55+ years	Non-melanoma skin cancer (basal-cell	Percent	1990	2.60E-06	4.80E-06	1.20E-06
YLDs (Year	Guatemala	Male	55+ years	Non-melanoma skin cancer (basal-cell	Rate	1990	0.0572766	0.118481899	0.024326591
YLDs (Year	Guatemala	Female	55+ years	Non-melanoma skin cancer (basal-cell	Rate	1990	0.0528799	0.101911239	0.022871627
YLDs (Year	Guatemala	Both	55+ years	Non-melanoma skin cancer (basal-cell	Rate	1990	0.0550811	0.10885719	0.023972086
YLDs (Year	Guatemala	Male	55+ years	Non-melanoma skin cancer (basal-cell	Number	2021	0.5375929	1.087966103	0.236883721
YLDs (Year	Guatemala	Female	55+ years	Non-melanoma skin cancer (basal-cell	Number	2021	0.554107	1.078739651	0.239247918
YLDs (Year	Guatemala	Both	55+ years	Non-melanoma skin cancer (basal-cell	Number	2021	1.0916999	2.139485044	0.468240035
YLDs (Year	Guatemala	Male	55+ years	Non-melanoma skin cancer (basal-cell	Percent	2021	2.74E-06	5.13E-06	1.25E-06
YLDs (Year	Guatemala	Female	55+ years	Non-melanoma skin cancer (basal-cell	Percent	2021	2.39E-06	4.38E-06	1.10E-06
YLDs (Year	Guatemala	Both	55+ years	Non-melanoma skin cancer (basal-cell	Percent	2021	2.55E-06	4.56E-06	1.21E-06

YLDs (Year	Guatemala	Male	55+ years	Non-melanoma skin cancer (basal-cell	Rate	2021	0.0630729	0.127645163	0.027792282
YLDs (Year	Guatemala	Female	55+ years	Non-melanoma skin cancer (basal-cell	Rate	2021	0.0566319	0.110251359	0.024452061
YLDs (Year	Guatemala	Both	55+ years	Non-melanoma skin cancer (basal-cell	Rate	2021	0.0596305	0.116862392	0.025576085
YLDs (Year	Costa Rica	Male	55+ years	Non-melanoma skin cancer (basal-cell	Number	1990	0.1395306	0.292485656	0.057432926
YLDs (Year	Costa Rica	Female	55+ years	Non-melanoma skin cancer (basal-cell	Number	1990	0.1510896	0.298993946	0.061568906
YLDs (Year	Costa Rica	Both	55+ years	Non-melanoma skin cancer (basal-cell	Number	1990	0.2906201	0.590176797	0.123818304
YLDs (Year	Costa Rica	Male	55+ years	Non-melanoma skin cancer (basal-cell	Percent	1990	5.10E-06	9.63E-06	2.21E-06
YLDs (Year	Costa Rica	Female	55+ years	Non-melanoma skin cancer (basal-cell	Percent	1990	4.76E-06	8.86E-06	2.11E-06
YLDs (Year	Costa Rica	Both	55+ years	Non-melanoma skin cancer (basal-cell	Percent	1990	4.92E-06	9.14E-06	2.31E-06
YLDs (Year	Costa Rica	Male	55+ years	Non-melanoma skin cancer (basal-cell	Rate	1990	0.1010228	0.211765194	0.041582534
YLDs (Year	Costa Rica	Female	55+ years	Non-melanoma skin cancer (basal-cell	Rate	1990	0.1032896	0.204401671	0.042090442
YLDs (Year	Costa Rica	Both	55+ years	Non-melanoma skin cancer (basal-cell	Rate	1990	0.1021887	0.207519701	0.043537356
YLDs (Year	Costa Rica	Male	55+ years	Non-melanoma skin cancer (basal-cell	Number	2021	0.3516701	0.718628586	0.137357007
YLDs (Year	Costa Rica	Female	55+ years	Non-melanoma skin cancer (basal-cell	Number	2021	0.396544	0.741849126	0.165663694
YLDs (Year	Costa Rica	Both	55+ years	Non-melanoma skin cancer (basal-cell	Number	2021	0.7482141	1.441655079	0.305844878
YLDs (Year	Costa Rica	Male	55+ years	Non-melanoma skin cancer (basal-cell	Percent	2021	3.79E-06	7.47E-06	1.57E-06
YLDs (Year	Costa Rica	Female	55+ years	Non-melanoma skin cancer (basal-cell	Percent	2021	3.38E-06	6.39E-06	1.44E-06
YLDs (Year	Costa Rica	Both	55+ years	Non-melanoma skin cancer (basal-cell	Percent	2021	3.56E-06	6.59E-06	1.45E-06
YLDs (Year	Costa Rica	Male	55+ years	Non-melanoma skin cancer (basal-cell	Rate	2021	0.0801243	0.163731901	0.031295337
YLDs (Year	Costa Rica	Female	55+ years	Non-melanoma skin cancer (basal-cell	Rate	2021	0.0764706	0.143060196	0.031947036
YLDs (Year	Costa Rica	Both	55+ years	Non-melanoma skin cancer (basal-cell	Rate	2021	0.0781455	0.150570319	0.031943259
YLDs (Year	Dominica	Male	55+ years	Non-melanoma skin cancer (basal-cell	Number	1990	0.0005876	0.001120096	0.000230299
YLDs (Year	Dominica	Female	55+ years	Non-melanoma skin cancer (basal-cell	Number	1990	0.0003766	0.00072348	0.000152154
YLDs (Year	Dominica	Both	55+ years	Non-melanoma skin cancer (basal-cell	Number	1990	0.0009642	0.00181456	0.000385629
YLDs (Year	Dominica	Male	55+ years	Non-melanoma skin cancer (basal-cell	Percent	1990	7.98E-07	1.47E-06	3.66E-07
YLDs (Year	Dominica	Female	55+ years	Non-melanoma skin cancer (basal-cell	Percent	1990	2.96E-07	5.39E-07	1.32E-07
YLDs (Year	Dominica	Both	55+ years	Non-melanoma skin cancer (basal-cell	Percent	1990	4.80E-07	8.82E-07	2.17E-07
YLDs (Year	Dominica	Male	55+ years	Non-melanoma skin cancer (basal-cell	Rate	1990	0.0156095	0.029756642	0.00611815
YLDs (Year	Dominica	Female	55+ years	Non-melanoma skin cancer (basal-cell	Rate	1990	0.0062208	0.011951223	0.002513447
YLDs (Year	Dominica	Both	55+ years	Non-melanoma skin cancer (basal-cell	Rate	1990	0.0098204	0.018482359	0.00392786
YLDs (Year	Dominica	Male	55+ years	Non-melanoma skin cancer (basal-cell	Number	2021	0.0010001	0.001987555	0.000415285
YLDs (Year	Dominica	Female	55+ years	Non-melanoma skin cancer (basal-cell	Number	2021	0.0004609	0.000887381	0.000185249
YLDs (Year	Dominica	Both	55+ years	Non-melanoma skin cancer (basal-cell	Number	2021	0.001461	0.002875231	0.000608097
YLDs (Year	Dominica	Male	55+ years	Non-melanoma skin cancer (basal-cell	Percent	2021	6.70E-07	1.23E-06	3.04E-07
YLDs (Year	Dominica	Female	55+ years	Non-melanoma skin cancer (basal-cell	Percent	2021	2.71E-07	4.92E-07	1.22E-07
YLDs (Year	Dominica	Both	55+ years	Non-melanoma skin cancer (basal-cell	Percent	2021	4.57E-07	8.48E-07	2.10E-07
YLDs (Year	Dominica	Male	55+ years	Non-melanoma skin cancer (basal-cell	Rate	2021	0.0134273	0.026684552	0.005575542
YLDs (Year	Dominica	Female	55+ years	Non-melanoma skin cancer (basal-cell	Rate	2021	0.0060375	0.011623356	0.002426487
YLDs (Year	Dominica	Both	55+ years	Non-melanoma skin cancer (basal-cell	Rate	2021	0.0096868	0.019062979	0.004031725
YLDs (Year	Algeria	Male	55+ years	Non-melanoma skin cancer (basal-cell	Number	1990	0.2128012	0.418595714	0.095899967
YLDs (Year	Algeria	Female	55+ years	Non-melanoma skin cancer (basal-cell	Number	1990	0.1294539	0.250002372	0.056926497
YLDs (Year	Algeria	Both	55+ years	Non-melanoma skin cancer (basal-cell	Number	1990	0.342255	0.671137397	0.155543083
YLDs (Year	Algeria	Male	55+ years	Non-melanoma skin cancer (basal-cell	Percent	1990	1.07E-06	1.89E-06	5.14E-07
YLDs (Year	Algeria	Female	55+ years	Non-melanoma skin cancer (basal-cell	Percent	1990	5.57E-07	1.01E-06	2.64E-07
YLDs (Year	Algeria	Both	55+ years	Non-melanoma skin cancer (basal-cell	Percent	1990	7.94E-07	1.41E-06	3.75E-07
YLDs (Year	Algeria	Male	55+ years	Non-melanoma skin cancer (basal-cell	Rate	1990	0.0207701	0.040856314	0.009360151
YLDs (Year	Algeria	Female	55+ years	Non-melanoma skin cancer (basal-cell	Rate	1990	0.0120931	0.023354321	0.005317868
YLDs (Year	Algeria	Both	55+ years	Non-melanoma skin cancer (basal-cell	Rate	1990	0.0163365	0.032034715	0.007424379
YLDs (Year	Algeria	Male	55+ years	Non-melanoma skin cancer (basal-cell	Number	2021	0.5774611	1.129461462	0.2426614
YLDs (Year	Algeria	Female	55+ years	Non-melanoma skin cancer (basal-cell	Number	2021	0.3446146	0.673367469	0.140167728
YLDs (Year	Algeria	Both	55+ years	Non-melanoma skin cancer (basal-cell	Number	2021	0.9220757	1.805626393	0.390678623
YLDs (Year	Algeria	Male	55+ years	Non-melanoma skin cancer (basal-cell	Percent	2021	9.24E-07	1.69E-06	4.24E-07
YLDs (Year	Algeria	Female	55+ years	Non-melanoma skin cancer (basal-cell	Percent	2021	4.95E-07	9.22E-07	2.19E-07
YLDs (Year	Algeria	Both	55+ years	Non-melanoma skin cancer (basal-cell	Percent	2021	6.98E-07	1.27E-06	3.19E-07
YLDs (Year	Algeria	Male	55+ years	Non-melanoma skin cancer (basal-cell	Rate	2021	0.0186828	0.036541956	0.007850929
YLDs (Year	Algeria	Female	55+ years	Non-melanoma skin cancer (basal-cell	Rate	2021	0.0115545	0.022577222	0.00469966

YLDs (Year	Algeria	Both	55+ years	Non-melanoma skin cancer (basal-cell	Rate	2021	0.0151823	0.029730221	0.00643265
YLDs (Year	Egypt	Male	55+ years	Non-melanoma skin cancer (basal-cell	Number	1990	0.3235235	0.628242043	0.14346608
YLDs (Year	Egypt	Female	55+ years	Non-melanoma skin cancer (basal-cell	Number	1990	0.1459258	0.278850421	0.064117549
YLDs (Year	Egypt	Both	55+ years	Non-melanoma skin cancer (basal-cell	Number	1990	0.4694492	0.905513137	0.212397263
YLDs (Year	Egypt	Male	55+ years	Non-melanoma skin cancer (basal-cell	Percent	1990	7.75E-07	1.39E-06	3.68E-07
YLDs (Year	Egypt	Female	55+ years	Non-melanoma skin cancer (basal-cell	Percent	1990	3.19E-07	5.82E-07	1.44E-07
YLDs (Year	Egypt	Both	55+ years	Non-melanoma skin cancer (basal-cell	Percent	1990	5.36E-07	9.50E-07	2.53E-07
YLDs (Year	Egypt	Male	55+ years	Non-melanoma skin cancer (basal-cell	Rate	1990	0.0142	0.027574626	0.006296973
YLDs (Year	Egypt	Female	55+ years	Non-melanoma skin cancer (basal-cell	Rate	1990	0.0066539	0.012715071	0.002923643
YLDs (Year	Egypt	Both	55+ years	Non-melanoma skin cancer (basal-cell	Rate	1990	0.0104989	0.020251204	0.004750125
YLDs (Year	Egypt	Male	55+ years	Non-melanoma skin cancer (basal-cell	Number	2021	0.7230317	1.388294637	0.311574443
YLDs (Year	Egypt	Female	55+ years	Non-melanoma skin cancer (basal-cell	Number	2021	0.3539628	0.701774786	0.14542318
YLDs (Year	Egypt	Both	55+ years	Non-melanoma skin cancer (basal-cell	Number	2021	1.0769946	2.105924464	0.445767179
YLDs (Year	Egypt	Male	55+ years	Non-melanoma skin cancer (basal-cell	Percent	2021	6.25E-07	1.15E-06	2.90E-07
YLDs (Year	Egypt	Female	55+ years	Non-melanoma skin cancer (basal-cell	Percent	2021	3.00E-07	5.67E-07	1.29E-07
YLDs (Year	Egypt	Both	55+ years	Non-melanoma skin cancer (basal-cell	Percent	2021	4.61E-07	8.51E-07	2.10E-07
YLDs (Year	Egypt	Male	55+ years	Non-melanoma skin cancer (basal-cell	Rate	2021	0.0122522	0.023525461	0.00527981
YLDs (Year	Egypt	Female	55+ years	Non-melanoma skin cancer (basal-cell	Rate	2021	0.0068536	0.013588161	0.002815766
YLDs (Year	Egypt	Both	55+ years	Non-melanoma skin cancer (basal-cell	Rate	2021	0.0097326	0.019030846	0.004028315
YLDs (Year	Iraq	Male	55+ years	Non-melanoma skin cancer (basal-cell	Number	1990	0.0846982	0.164786405	0.033485414
YLDs (Year	Iraq	Female	55+ years	Non-melanoma skin cancer (basal-cell	Number	1990	0.0576957	0.110461868	0.02343308
YLDs (Year	Iraq	Both	55+ years	Non-melanoma skin cancer (basal-cell	Number	1990	0.142394	0.272976247	0.056943007
YLDs (Year	Iraq	Male	55+ years	Non-melanoma skin cancer (basal-cell	Percent	1990	6.56E-07	1.19E-06	3.04E-07
YLDs (Year	Iraq	Female	55+ years	Non-melanoma skin cancer (basal-cell	Percent	1990	3.90E-07	6.93E-07	1.76E-07
YLDs (Year	Iraq	Both	55+ years	Non-melanoma skin cancer (basal-cell	Percent	1990	5.14E-07	9.30E-07	2.35E-07
YLDs (Year	Iraq	Male	55+ years	Non-melanoma skin cancer (basal-cell	Rate	1990	0.013836	0.026918825	0.005470039
YLDs (Year	Iraq	Female	55+ years	Non-melanoma skin cancer (basal-cell	Rate	1990	0.0088746	0.01699089	0.003604401
YLDs (Year	Iraq	Both	55+ years	Non-melanoma skin cancer (basal-cell	Rate	1990	0.0112807	0.021625572	0.004511107
YLDs (Year	Iraq	Male	55+ years	Non-melanoma skin cancer (basal-cell	Number	2021	0.2293803	0.447848654	0.10294476
YLDs (Year	Iraq	Female	55+ years	Non-melanoma skin cancer (basal-cell	Number	2021	0.1704726	0.325995768	0.076003364
YLDs (Year	Iraq	Both	55+ years	Non-melanoma skin cancer (basal-cell	Number	2021	0.399853	0.774315787	0.178633838
YLDs (Year	Iraq	Male	55+ years	Non-melanoma skin cancer (basal-cell	Percent	2021	5.05E-07	9.00E-07	2.49E-07
YLDs (Year	Iraq	Female	55+ years	Non-melanoma skin cancer (basal-cell	Percent	2021	3.46E-07	6.28E-07	1.65E-07
YLDs (Year	Iraq	Both	55+ years	Non-melanoma skin cancer (basal-cell	Percent	2021	4.22E-07	7.45E-07	2.06E-07
YLDs (Year	Iraq	Male	55+ years	Non-melanoma skin cancer (basal-cell	Rate	2021	0.0120667	0.023559461	0.005415497
YLDs (Year	Iraq	Female	55+ years	Non-melanoma skin cancer (basal-cell	Rate	2021	0.0085912	0.016429062	0.003830307
YLDs (Year	Iraq	Both	55+ years	Non-melanoma skin cancer (basal-cell	Rate	2021	0.0102917	0.019929924	0.004597812
YLDs (Year	Panama	Male	55+ years	Non-melanoma skin cancer (basal-cell	Number	1990	0.0672765	0.132451236	0.029994894
YLDs (Year	Panama	Female	55+ years	Non-melanoma skin cancer (basal-cell	Number	1990	0.0581251	0.116253709	0.026765463
YLDs (Year	Panama	Both	55+ years	Non-melanoma skin cancer (basal-cell	Number	1990	0.1254016	0.248214845	0.056836486
YLDs (Year	Panama	Male	55+ years	Non-melanoma skin cancer (basal-cell	Percent	1990	2.78E-06	4.85E-06	1.36E-06
YLDs (Year	Panama	Female	55+ years	Non-melanoma skin cancer (basal-cell	Percent	1990	2.29E-06	4.15E-06	1.10E-06
YLDs (Year	Panama	Both	55+ years	Non-melanoma skin cancer (basal-cell	Percent	1990	2.53E-06	4.46E-06	1.21E-06
YLDs (Year	Panama	Male	55+ years	Non-melanoma skin cancer (basal-cell	Rate	1990	0.0548648	0.108015497	0.024461179
YLDs (Year	Panama	Female	55+ years	Non-melanoma skin cancer (basal-cell	Rate	1990	0.048572	0.097146981	0.02236646
YLDs (Year	Panama	Both	55+ years	Non-melanoma skin cancer (basal-cell	Rate	1990	0.0517568	0.102445229	0.023458012
YLDs (Year	Panama	Male	55+ years	Non-melanoma skin cancer (basal-cell	Number	2021	0.193305	0.371571697	0.085729591
YLDs (Year	Panama	Female	55+ years	Non-melanoma skin cancer (basal-cell	Number	2021	0.2017947	0.394267556	0.091438791
YLDs (Year	Panama	Both	55+ years	Non-melanoma skin cancer (basal-cell	Number	2021	0.3950996	0.764330161	0.177038645
YLDs (Year	Panama	Male	55+ years	Non-melanoma skin cancer (basal-cell	Percent	2021	2.56E-06	4.60E-06	1.23E-06
YLDs (Year	Panama	Female	55+ years	Non-melanoma skin cancer (basal-cell	Percent	2021	2.34E-06	4.32E-06	1.14E-06
YLDs (Year	Panama	Both	55+ years	Non-melanoma skin cancer (basal-cell	Percent	2021	2.44E-06	4.39E-06	1.19E-06
YLDs (Year	Panama	Male	55+ years	Non-melanoma skin cancer (basal-cell	Rate	2021	0.0536182	0.103065072	0.023779331
YLDs (Year	Panama	Female	55+ years	Non-melanoma skin cancer (basal-cell	Rate	2021	0.052682	0.1029303	0.023871663
YLDs (Year	Panama	Both	55+ years	Non-melanoma skin cancer (basal-cell	Rate	2021	0.0531359	0.102792692	0.023809447
YLDs (Year	Venezuela	Male	55+ years	Non-melanoma skin cancer (basal-cell	Number	1990	0.4682283	0.915203627	0.190117454

YLDs (Year	Venezuela	Female	55+ years	Non-melanoma skin cancer (basal-cell	Number	1990	0.5031811	1.010408975	0.206617445
YLDs (Year	Venezuela	Both	55+ years	Non-melanoma skin cancer (basal-cell	Number	1990	0.9714094	1.901926414	0.392655537
YLDs (Year	Venezuela	Male	55+ years	Non-melanoma skin cancer (basal-cell	Percent	1990	3.19E-06	5.81E-06	1.44E-06
YLDs (Year	Venezuela	Female	55+ years	Non-melanoma skin cancer (basal-cell	Percent	1990	2.85E-06	5.35E-06	1.30E-06
YLDs (Year	Venezuela	Both	55+ years	Non-melanoma skin cancer (basal-cell	Percent	1990	3.01E-06	5.39E-06	1.46E-06
YLDs (Year	Venezuela	Male	55+ years	Non-melanoma skin cancer (basal-cell	Rate	1990	0.0630843	0.123305256	0.025614498
YLDs (Year	Venezuela	Female	55+ years	Non-melanoma skin cancer (basal-cell	Rate	1990	0.0610929	0.122677064	0.025086101
YLDs (Year	Venezuela	Both	55+ years	Non-melanoma skin cancer (basal-cell	Rate	1990	0.0620368	0.121462166	0.025076045
YLDs (Year	Venezuela	Male	55+ years	Non-melanoma skin cancer (basal-cell	Number	2021	1.5133166	3.008928212	0.624363553
YLDs (Year	Venezuela	Female	55+ years	Non-melanoma skin cancer (basal-cell	Number	2021	1.7598834	3.520294844	0.766318653
YLDs (Year	Venezuela	Both	55+ years	Non-melanoma skin cancer (basal-cell	Number	2021	3.2732	6.377916018	1.394055185
YLDs (Year	Venezuela	Male	55+ years	Non-melanoma skin cancer (basal-cell	Percent	2021	3.08E-06	5.65E-06	1.43E-06
YLDs (Year	Venezuela	Female	55+ years	Non-melanoma skin cancer (basal-cell	Percent	2021	2.82E-06	5.26E-06	1.29E-06
YLDs (Year	Venezuela	Both	55+ years	Non-melanoma skin cancer (basal-cell	Percent	2021	2.94E-06	5.33E-06	1.38E-06
YLDs (Year	Venezuela	Male	55+ years	Non-melanoma skin cancer (basal-cell	Rate	2021	0.0628337	0.124932243	0.025923895
YLDs (Year	Venezuela	Female	55+ years	Non-melanoma skin cancer (basal-cell	Rate	2021	0.0623245	0.12466778	0.027138422
YLDs (Year	Venezuela	Both	55+ years	Non-melanoma skin cancer (basal-cell	Rate	2021	0.0625589	0.121897666	0.026643824
YLDs (Year	Kuwait	Male	55+ years	Non-melanoma skin cancer (basal-cell	Number	1990	0.0078059	0.014887224	0.003455014
YLDs (Year	Kuwait	Female	55+ years	Non-melanoma skin cancer (basal-cell	Number	1990	0.0040691	0.007707054	0.001770254
YLDs (Year	Kuwait	Both	55+ years	Non-melanoma skin cancer (basal-cell	Number	1990	0.011875	0.022854724	0.005273035
YLDs (Year	Kuwait	Male	55+ years	Non-melanoma skin cancer (basal-cell	Percent	1990	7.55E-07	1.36E-06	3.62E-07
YLDs (Year	Kuwait	Female	55+ years	Non-melanoma skin cancer (basal-cell	Percent	1990	5.48E-07	9.85E-07	2.54E-07
YLDs (Year	Kuwait	Both	55+ years	Non-melanoma skin cancer (basal-cell	Percent	1990	6.68E-07	1.21E-06	3.18E-07
YLDs (Year	Kuwait	Male	55+ years	Non-melanoma skin cancer (basal-cell	Rate	1990	0.0135689	0.025878271	0.006005807
YLDs (Year	Kuwait	Female	55+ years	Non-melanoma skin cancer (basal-cell	Rate	1990	0.0119998	0.022728085	0.005220475
YLDs (Year	Kuwait	Both	55+ years	Non-melanoma skin cancer (basal-cell	Rate	1990	0.012987	0.024994853	0.005766805
YLDs (Year	Kuwait	Male	55+ years	Non-melanoma skin cancer (basal-cell	Number	2021	0.0394262	0.075636257	0.01662162
YLDs (Year	Kuwait	Female	55+ years	Non-melanoma skin cancer (basal-cell	Number	2021	0.0188918	0.037299108	0.007896704
YLDs (Year	Kuwait	Both	55+ years	Non-melanoma skin cancer (basal-cell	Number	2021	0.058318	0.112284412	0.024415401
YLDs (Year	Kuwait	Male	55+ years	Non-melanoma skin cancer (basal-cell	Percent	2021	7.02E-07	1.28E-06	3.08E-07
YLDs (Year	Kuwait	Female	55+ years	Non-melanoma skin cancer (basal-cell	Percent	2021	4.17E-07	7.84E-07	1.83E-07
YLDs (Year	Kuwait	Both	55+ years	Non-melanoma skin cancer (basal-cell	Percent	2021	5.75E-07	1.08E-06	2.50E-07
YLDs (Year	Kuwait	Male	55+ years	Non-melanoma skin cancer (basal-cell	Rate	2021	0.0144956	0.027808774	0.006111181
YLDs (Year	Kuwait	Female	55+ years	Non-melanoma skin cancer (basal-cell	Rate	2021	0.0097261	0.019202911	0.004065505
YLDs (Year	Kuwait	Both	55+ years	Non-melanoma skin cancer (basal-cell	Rate	2021	0.0125086	0.024083803	0.005236842
YLDs (Year	Paraguay	Male	55+ years	Non-melanoma skin cancer (basal-cell	Number	1990	0.0004046	0.000873683	0.000116966
YLDs (Year	Paraguay	Female	55+ years	Non-melanoma skin cancer (basal-cell	Number	1990	0.0003655	0.000833277	0.000105823
YLDs (Year	Paraguay	Both	55+ years	Non-melanoma skin cancer (basal-cell	Number	1990	0.0007702	0.001686382	0.000234516
YLDs (Year	Paraguay	Male	55+ years	Non-melanoma skin cancer (basal-cell	Percent	1990	1.18E-08	2.38E-08	3.25E-09
YLDs (Year	Paraguay	Female	55+ years	Non-melanoma skin cancer (basal-cell	Percent	1990	8.76E-09	1.86E-08	2.76E-09
YLDs (Year	Paraguay	Both	55+ years	Non-melanoma skin cancer (basal-cell	Percent	1990	1.01E-08	2.10E-08	3.38E-09
YLDs (Year	Paraguay	Male	55+ years	Non-melanoma skin cancer (basal-cell	Rate	1990	0.0002334	0.00050405	6.75E-05
YLDs (Year	Paraguay	Female	55+ years	Non-melanoma skin cancer (basal-cell	Rate	1990	0.0001929	0.000439866	5.59E-05
YLDs (Year	Paraguay	Both	55+ years	Non-melanoma skin cancer (basal-cell	Rate	1990	0.0002123	0.000464861	6.46E-05
YLDs (Year	Paraguay	Male	55+ years	Non-melanoma skin cancer (basal-cell	Number	2021	0.0008131	0.001815254	0.000252989
YLDs (Year	Paraguay	Female	55+ years	Non-melanoma skin cancer (basal-cell	Number	2021	0.0007584	0.001817608	0.000187463
YLDs (Year	Paraguay	Both	55+ years	Non-melanoma skin cancer (basal-cell	Number	2021	0.0015715	0.00353621	0.000465947
YLDs (Year	Paraguay	Male	55+ years	Non-melanoma skin cancer (basal-cell	Percent	2021	8.16E-09	1.73E-08	2.71E-09
YLDs (Year	Paraguay	Female	55+ years	Non-melanoma skin cancer (basal-cell	Percent	2021	6.30E-09	1.43E-08	1.67E-09
YLDs (Year	Paraguay	Both	55+ years	Non-melanoma skin cancer (basal-cell	Percent	2021	7.14E-09	1.49E-08	2.28E-09
YLDs (Year	Paraguay	Male	55+ years	Non-melanoma skin cancer (basal-cell	Rate	2021	0.0001716	0.000383119	5.34E-05
YLDs (Year	Paraguay	Female	55+ years	Non-melanoma skin cancer (basal-cell	Rate	2021	0.0001469	0.000352048	3.63E-05
YLDs (Year	Paraguay	Both	55+ years	Non-melanoma skin cancer (basal-cell	Rate	2021	0.0001587	0.000357155	4.71E-05
YLDs (Year	Iran (Isla	Male	55+ years	Non-melanoma skin cancer (basal-cell	Number	1990	0.6921365	1.353918492	0.28614054
YLDs (Year	Iran (Isla	Female	55+ years	Non-melanoma skin cancer (basal-cell	Number	1990	0.4116127	0.79130127	0.170818343
YLDs (Year	Iran (Isla	Both	55+ years	Non-melanoma skin cancer (basal-cell	Number	1990	1.1037492	2.13415044	0.458418354

YLDs (Year	Iran (Isla	Male	55+ years	Non-melanoma skin cancer (basal-cell	Percent	1990	1.45E-06	2.64E-06	6.49E-07
YLDs (Year	Iran (Isla	Female	55+ years	Non-melanoma skin cancer (basal-cell	Percent	1990	8.65E-07	1.62E-06	3.85E-07
YLDs (Year	Iran (Isla	Both	55+ years	Non-melanoma skin cancer (basal-cell	Percent	1990	1.16E-06	2.14E-06	5.18E-07
YLDs (Year	Iran (Isla	Male	55+ years	Non-melanoma skin cancer (basal-cell	Rate	1990	0.0280561	0.054881835	0.011598865
YLDs (Year	Iran (Isla	Female	55+ years	Non-melanoma skin cancer (basal-cell	Rate	1990	0.0192076	0.03692555	0.007971125
YLDs (Year	Iran (Isla	Both	55+ years	Non-melanoma skin cancer (basal-cell	Rate	1990	0.0239428	0.046294596	0.009944141
YLDs (Year	Iran (Isla	Male	55+ years	Non-melanoma skin cancer (basal-cell	Number	2021	1.5447433	2.964203312	0.644064047
YLDs (Year	Iran (Isla	Female	55+ years	Non-melanoma skin cancer (basal-cell	Number	2021	1.0204588	1.94634408	0.429890766
YLDs (Year	Iran (Isla	Both	55+ years	Non-melanoma skin cancer (basal-cell	Number	2021	2.5652021	4.890574495	1.073954813
YLDs (Year	Iran (Isla	Male	55+ years	Non-melanoma skin cancer (basal-cell	Percent	2021	1.14E-06	2.10E-06	5.20E-07
YLDs (Year	Iran (Isla	Female	55+ years	Non-melanoma skin cancer (basal-cell	Percent	2021	6.42E-07	1.22E-06	2.88E-07
YLDs (Year	Iran (Isla	Both	55+ years	Non-melanoma skin cancer (basal-cell	Percent	2021	8.70E-07	1.63E-06	3.96E-07
YLDs (Year	Iran (Isla	Male	55+ years	Non-melanoma skin cancer (basal-cell	Rate	2021	0.0239841	0.046022997	0.009999907
YLDs (Year	Iran (Isla	Female	55+ years	Non-melanoma skin cancer (basal-cell	Rate	2021	0.015579	0.029714086	0.006562977
YLDs (Year	Iran (Isla	Both	55+ years	Non-melanoma skin cancer (basal-cell	Rate	2021	0.0197461	0.037646038	0.008266952
YLDs (Year	Jordan	Male	55+ years	Non-melanoma skin cancer (basal-cell	Number	1990	0.0221204	0.042709285	0.009344282
YLDs (Year	Jordan	Female	55+ years	Non-melanoma skin cancer (basal-cell	Number	1990	0.0145453	0.028747522	0.006367526
YLDs (Year	Jordan	Both	55+ years	Non-melanoma skin cancer (basal-cell	Number	1990	0.0366658	0.071626763	0.015876087
YLDs (Year	Jordan	Male	55+ years	Non-melanoma skin cancer (basal-cell	Percent	1990	1.09E-06	1.97E-06	5.02E-07
YLDs (Year	Jordan	Female	55+ years	Non-melanoma skin cancer (basal-cell	Percent	1990	6.13E-07	1.13E-06	2.85E-07
YLDs (Year	Jordan	Both	55+ years	Non-melanoma skin cancer (basal-cell	Percent	1990	8.34E-07	1.51E-06	3.82E-07
YLDs (Year	Jordan	Male	55+ years	Non-melanoma skin cancer (basal-cell	Rate	1990	0.0199154	0.038451915	0.00841282
YLDs (Year	Jordan	Female	55+ years	Non-melanoma skin cancer (basal-cell	Rate	1990	0.013994	0.027657845	0.006126164
YLDs (Year	Jordan	Both	55+ years	Non-melanoma skin cancer (basal-cell	Rate	1990	0.0170529	0.033312948	0.007383822
YLDs (Year	Jordan	Male	55+ years	Non-melanoma skin cancer (basal-cell	Number	2021	0.121957	0.234029635	0.050992661
YLDs (Year	Jordan	Female	55+ years	Non-melanoma skin cancer (basal-cell	Number	2021	0.0679926	0.133083263	0.027169746
YLDs (Year	Jordan	Both	55+ years	Non-melanoma skin cancer (basal-cell	Number	2021	0.1899497	0.363785272	0.078735014
YLDs (Year	Jordan	Male	55+ years	Non-melanoma skin cancer (basal-cell	Percent	2021	9.18E-07	1.67E-06	4.25E-07
YLDs (Year	Jordan	Female	55+ years	Non-melanoma skin cancer (basal-cell	Percent	2021	4.70E-07	9.11E-07	2.06E-07
YLDs (Year	Jordan	Both	55+ years	Non-melanoma skin cancer (basal-cell	Percent	2021	6.85E-07	1.27E-06	3.14E-07
YLDs (Year	Jordan	Male	55+ years	Non-melanoma skin cancer (basal-cell	Rate	2021	0.0184307	0.035367591	0.007706236
YLDs (Year	Jordan	Female	55+ years	Non-melanoma skin cancer (basal-cell	Rate	2021	0.0114171	0.022346834	0.00456224
YLDs (Year	Jordan	Both	55+ years	Non-melanoma skin cancer (basal-cell	Rate	2021	0.0151084	0.02893519	0.00626252
YLDs (Year	Lebanon	Male	55+ years	Non-melanoma skin cancer (basal-cell	Number	1990	0.0484073	0.093007093	0.020235027
YLDs (Year	Lebanon	Female	55+ years	Non-melanoma skin cancer (basal-cell	Number	1990	0.0340462	0.065166366	0.014125071
YLDs (Year	Lebanon	Both	55+ years	Non-melanoma skin cancer (basal-cell	Number	1990	0.0824535	0.157291951	0.034678325
YLDs (Year	Lebanon	Male	55+ years	Non-melanoma skin cancer (basal-cell	Percent	1990	1.29E-06	2.46E-06	5.87E-07
YLDs (Year	Lebanon	Female	55+ years	Non-melanoma skin cancer (basal-cell	Percent	1990	7.86E-07	1.47E-06	3.49E-07
YLDs (Year	Lebanon	Both	55+ years	Non-melanoma skin cancer (basal-cell	Percent	1990	1.02E-06	1.93E-06	4.64E-07
YLDs (Year	Lebanon	Male	55+ years	Non-melanoma skin cancer (basal-cell	Rate	1990	0.0257683	0.049509794	0.010771566
YLDs (Year	Lebanon	Female	55+ years	Non-melanoma skin cancer (basal-cell	Rate	1990	0.0177994	0.03406909	0.007384612
YLDs (Year	Lebanon	Both	55+ years	Non-melanoma skin cancer (basal-cell	Rate	1990	0.0217479	0.04148727	0.009146743
YLDs (Year	Lebanon	Male	55+ years	Non-melanoma skin cancer (basal-cell	Number	2021	0.1389277	0.273219544	0.057076486
YLDs (Year	Lebanon	Female	55+ years	Non-melanoma skin cancer (basal-cell	Number	2021	0.1053948	0.210246881	0.042830247
YLDs (Year	Lebanon	Both	55+ years	Non-melanoma skin cancer (basal-cell	Number	2021	0.2443226	0.484839146	0.100059825
YLDs (Year	Lebanon	Male	55+ years	Non-melanoma skin cancer (basal-cell	Percent	2021	1.26E-06	2.36E-06	5.78E-07
YLDs (Year	Lebanon	Female	55+ years	Non-melanoma skin cancer (basal-cell	Percent	2021	7.60E-07	1.38E-06	3.46E-07
YLDs (Year	Lebanon	Both	55+ years	Non-melanoma skin cancer (basal-cell	Percent	2021	9.83E-07	1.81E-06	4.45E-07
YLDs (Year	Lebanon	Male	55+ years	Non-melanoma skin cancer (basal-cell	Rate	2021	0.0312567	0.061470304	0.012841354
YLDs (Year	Lebanon	Female	55+ years	Non-melanoma skin cancer (basal-cell	Rate	2021	0.0196796	0.039257799	0.007997366
YLDs (Year	Lebanon	Both	55+ years	Non-melanoma skin cancer (basal-cell	Rate	2021	0.0249302	0.049471945	0.01020989
YLDs (Year	Bahrain	Male	55+ years	Non-melanoma skin cancer (basal-cell	Number	1990	0.0017419	0.003500473	0.000709411
YLDs (Year	Bahrain	Female	55+ years	Non-melanoma skin cancer (basal-cell	Number	1990	0.0009245	0.001848378	0.000343704
YLDs (Year	Bahrain	Both	55+ years	Non-melanoma skin cancer (basal-cell	Number	1990	0.0026664	0.005325029	0.001071146
YLDs (Year	Bahrain	Male	55+ years	Non-melanoma skin cancer (basal-cell	Percent	1990	6.05E-07	1.17E-06	2.63E-07
YLDs (Year	Bahrain	Female	55+ years	Non-melanoma skin cancer (basal-cell	Percent	1990	3.20E-07	6.18E-07	1.34E-07

YLDs (Year	Bahrain	Both	55+ years	Non-melanoma skin cancer (basal-cell	Percent	1990	4.62E-07	8.93E-07	1.98E-07
YLDs (Year	Bahrain	Male	55+ years	Non-melanoma skin cancer (basal-cell	Rate	1990	0.0113864	0.022882036	0.004637308
YLDs (Year	Bahrain	Female	55+ years	Non-melanoma skin cancer (basal-cell	Rate	1990	0.0072461	0.014486558	0.002693759
YLDs (Year	Bahrain	Both	55+ years	Non-melanoma skin cancer (basal-cell	Rate	1990	0.0095035	0.018979211	0.003817726
YLDs (Year	Bahrain	Male	55+ years	Non-melanoma skin cancer (basal-cell	Number	2021	0.0101639	0.020182731	0.003927239
YLDs (Year	Bahrain	Female	55+ years	Non-melanoma skin cancer (basal-cell	Number	2021	0.0046186	0.00933131	0.001730442
YLDs (Year	Bahrain	Both	55+ years	Non-melanoma skin cancer (basal-cell	Number	2021	0.0147825	0.029217223	0.005587841
YLDs (Year	Bahrain	Male	55+ years	Non-melanoma skin cancer (basal-cell	Percent	2021	5.17E-07	9.87E-07	2.22E-07
YLDs (Year	Bahrain	Female	55+ years	Non-melanoma skin cancer (basal-cell	Percent	2021	2.83E-07	5.60E-07	1.18E-07
YLDs (Year	Bahrain	Both	55+ years	Non-melanoma skin cancer (basal-cell	Percent	2021	4.11E-07	7.99E-07	1.74E-07
YLDs (Year	Bahrain	Male	55+ years	Non-melanoma skin cancer (basal-cell	Rate	2021	0.0104874	0.020825073	0.004052228
YLDs (Year	Bahrain	Female	55+ years	Non-melanoma skin cancer (basal-cell	Rate	2021	0.0070559	0.014255675	0.002643639
YLDs (Year	Bahrain	Both	55+ years	Non-melanoma skin cancer (basal-cell	Rate	2021	0.009104	0.017993964	0.003441375
YLDs (Year	Palestine	Male	55+ years	Non-melanoma skin cancer (basal-cell	Number	1990	0.0096015	0.018455205	0.003993367
YLDs (Year	Palestine	Female	55+ years	Non-melanoma skin cancer (basal-cell	Number	1990	0.0070512	0.013354063	0.002879253
YLDs (Year	Palestine	Both	55+ years	Non-melanoma skin cancer (basal-cell	Number	1990	0.0166527	0.031499922	0.006825885
YLDs (Year	Palestine	Male	55+ years	Non-melanoma skin cancer (basal-cell	Percent	1990	7.37E-07	1.32E-06	3.35E-07
YLDs (Year	Palestine	Female	55+ years	Non-melanoma skin cancer (basal-cell	Percent	1990	3.95E-07	7.28E-07	1.74E-07
YLDs (Year	Palestine	Both	55+ years	Non-melanoma skin cancer (basal-cell	Percent	1990	5.40E-07	9.69E-07	2.43E-07
YLDs (Year	Palestine	Male	55+ years	Non-melanoma skin cancer (basal-cell	Rate	1990	0.0147133	0.028280594	0.0061194
YLDs (Year	Palestine	Female	55+ years	Non-melanoma skin cancer (basal-cell	Rate	1990	0.0088266	0.016716477	0.003604219
YLDs (Year	Palestine	Both	55+ years	Non-melanoma skin cancer (basal-cell	Rate	1990	0.0114733	0.021702664	0.004702865
YLDs (Year	Palestine	Male	55+ years	Non-melanoma skin cancer (basal-cell	Number	2021	0.0278169	0.053562796	0.011688908
YLDs (Year	Palestine	Female	55+ years	Non-melanoma skin cancer (basal-cell	Number	2021	0.0188231	0.037177223	0.007824057
YLDs (Year	Palestine	Both	55+ years	Non-melanoma skin cancer (basal-cell	Number	2021	0.0466399	0.090738923	0.019391822
YLDs (Year	Palestine	Male	55+ years	Non-melanoma skin cancer (basal-cell	Percent	2021	6.19E-07	1.14E-06	2.81E-07
YLDs (Year	Palestine	Female	55+ years	Non-melanoma skin cancer (basal-cell	Percent	2021	3.58E-07	6.73E-07	1.61E-07
YLDs (Year	Palestine	Both	55+ years	Non-melanoma skin cancer (basal-cell	Percent	2021	4.78E-07	8.64E-07	2.17E-07
YLDs (Year	Palestine	Male	55+ years	Non-melanoma skin cancer (basal-cell	Rate	2021	0.0130869	0.025199538	0.005499248
YLDs (Year	Palestine	Female	55+ years	Non-melanoma skin cancer (basal-cell	Rate	2021	0.0086461	0.017076819	0.003593867
YLDs (Year	Palestine	Both	55+ years	Non-melanoma skin cancer (basal-cell	Rate	2021	0.0108399	0.021089301	0.004506996
YLDs (Year	Libya	Male	55+ years	Non-melanoma skin cancer (basal-cell	Number	1990	0.0219875	0.042314225	0.009065831
YLDs (Year	Libya	Female	55+ years	Non-melanoma skin cancer (basal-cell	Number	1990	0.0107301	0.02165975	0.00423023
YLDs (Year	Libya	Both	55+ years	Non-melanoma skin cancer (basal-cell	Number	1990	0.0327176	0.063662372	0.013439064
YLDs (Year	Libya	Male	55+ years	Non-melanoma skin cancer (basal-cell	Percent	1990	6.99E-07	1.28E-06	3.22E-07
YLDs (Year	Libya	Female	55+ years	Non-melanoma skin cancer (basal-cell	Percent	1990	3.36E-07	6.37E-07	1.44E-07
YLDs (Year	Libya	Both	55+ years	Non-melanoma skin cancer (basal-cell	Percent	1990	5.16E-07	9.69E-07	2.34E-07
YLDs (Year	Libya	Male	55+ years	Non-melanoma skin cancer (basal-cell	Rate	1990	0.0133791	0.025747613	0.005516431
YLDs (Year	Libya	Female	55+ years	Non-melanoma skin cancer (basal-cell	Rate	1990	0.0073149	0.014765812	0.002883818
YLDs (Year	Libya	Both	55+ years	Non-melanoma skin cancer (basal-cell	Rate	1990	0.0105191	0.020468186	0.004320814
YLDs (Year	Libya	Male	55+ years	Non-melanoma skin cancer (basal-cell	Number	2021	0.0541393	0.105269267	0.022190374
YLDs (Year	Libya	Female	55+ years	Non-melanoma skin cancer (basal-cell	Number	2021	0.0284872	0.05490865	0.011396439
YLDs (Year	Libya	Both	55+ years	Non-melanoma skin cancer (basal-cell	Number	2021	0.0826266	0.161819348	0.033847907
YLDs (Year	Libya	Male	55+ years	Non-melanoma skin cancer (basal-cell	Percent	2021	6.02E-07	1.12E-06	2.69E-07
YLDs (Year	Libya	Female	55+ years	Non-melanoma skin cancer (basal-cell	Percent	2021	2.84E-07	5.25E-07	1.22E-07
YLDs (Year	Libya	Both	55+ years	Non-melanoma skin cancer (basal-cell	Percent	2021	4.34E-07	8.10E-07	1.88E-07
YLDs (Year	Libya	Male	55+ years	Non-melanoma skin cancer (basal-cell	Rate	2021	0.0128804	0.025044779	0.005279347
YLDs (Year	Libya	Female	55+ years	Non-melanoma skin cancer (basal-cell	Rate	2021	0.006848	0.013199439	0.002739579
YLDs (Year	Libya	Both	55+ years	Non-melanoma skin cancer (basal-cell	Rate	2021	0.0098798	0.019349056	0.00404726
YLDs (Year	Oman	Male	55+ years	Non-melanoma skin cancer (basal-cell	Number	1990	0.0076407	0.015098363	0.003421685
YLDs (Year	Oman	Female	55+ years	Non-melanoma skin cancer (basal-cell	Number	1990	0.0047509	0.009187165	0.001954495
YLDs (Year	Oman	Both	55+ years	Non-melanoma skin cancer (basal-cell	Number	1990	0.0123916	0.02464855	0.005453969
YLDs (Year	Oman	Male	55+ years	Non-melanoma skin cancer (basal-cell	Percent	1990	6.81E-07	1.26E-06	3.22E-07
YLDs (Year	Oman	Female	55+ years	Non-melanoma skin cancer (basal-cell	Percent	1990	4.44E-07	8.18E-07	2.08E-07
YLDs (Year	Oman	Both	55+ years	Non-melanoma skin cancer (basal-cell	Percent	1990	5.65E-07	1.03E-06	2.68E-07
YLDs (Year	Oman	Male	55+ years	Non-melanoma skin cancer (basal-cell	Rate	1990	0.0140724	0.027807882	0.006301996

YLDs (Year	Oman	Female	55+ years	Non-melanoma skin cancer (basal-cell	Rate	1990	0.0102804	0.019879869	0.00422928
YLDs (Year	Oman	Both	55+ years	Non-melanoma skin cancer (basal-cell	Rate	1990	0.0123289	0.024523804	0.005426367
YLDs (Year	Oman	Male	55+ years	Non-melanoma skin cancer (basal-cell	Number	2021	0.0214879	0.041127899	0.009470818
YLDs (Year	Oman	Female	55+ years	Non-melanoma skin cancer (basal-cell	Number	2021	0.0143863	0.0284063	0.00653417
YLDs (Year	Oman	Both	55+ years	Non-melanoma skin cancer (basal-cell	Number	2021	0.0358742	0.069093626	0.015923451
YLDs (Year	Oman	Male	55+ years	Non-melanoma skin cancer (basal-cell	Percent	2021	6.16E-07	1.11E-06	2.92E-07
YLDs (Year	Oman	Female	55+ years	Non-melanoma skin cancer (basal-cell	Percent	2021	4.54E-07	8.22E-07	2.15E-07
YLDs (Year	Oman	Both	55+ years	Non-melanoma skin cancer (basal-cell	Percent	2021	5.39E-07	9.78E-07	2.57E-07
YLDs (Year	Oman	Male	55+ years	Non-melanoma skin cancer (basal-cell	Rate	2021	0.0122182	0.023385705	0.005385195
YLDs (Year	Oman	Female	55+ years	Non-melanoma skin cancer (basal-cell	Rate	2021	0.0105405	0.020812478	0.004787398
YLDs (Year	Oman	Both	55+ years	Non-melanoma skin cancer (basal-cell	Rate	2021	0.0114851	0.022120255	0.005097877
YLDs (Year	Morocco	Male	55+ years	Non-melanoma skin cancer (basal-cell	Number	1990	0.1214135	0.23257709	0.048452638
YLDs (Year	Morocco	Female	55+ years	Non-melanoma skin cancer (basal-cell	Number	1990	0.0741507	0.144576622	0.031243966
YLDs (Year	Morocco	Both	55+ years	Non-melanoma skin cancer (basal-cell	Number	1990	0.1955643	0.378535363	0.079984348
YLDs (Year	Morocco	Male	55+ years	Non-melanoma skin cancer (basal-cell	Percent	1990	5.20E-07	9.39E-07	2.33E-07
YLDs (Year	Morocco	Female	55+ years	Non-melanoma skin cancer (basal-cell	Percent	1990	2.84E-07	5.31E-07	1.24E-07
YLDs (Year	Morocco	Both	55+ years	Non-melanoma skin cancer (basal-cell	Percent	1990	3.96E-07	7.15E-07	1.77E-07
YLDs (Year	Morocco	Male	55+ years	Non-melanoma skin cancer (basal-cell	Rate	1990	0.0103135	0.019756279	0.004115813
YLDs (Year	Morocco	Female	55+ years	Non-melanoma skin cancer (basal-cell	Rate	1990	0.0063506	0.012382269	0.00267589
YLDs (Year	Morocco	Both	55+ years	Non-melanoma skin cancer (basal-cell	Rate	1990	0.0083402	0.016143325	0.003411077
YLDs (Year	Morocco	Male	55+ years	Non-melanoma skin cancer (basal-cell	Number	2021	0.2922017	0.578197443	0.120985815
YLDs (Year	Morocco	Female	55+ years	Non-melanoma skin cancer (basal-cell	Number	2021	0.1864408	0.361750243	0.074906642
YLDs (Year	Morocco	Both	55+ years	Non-melanoma skin cancer (basal-cell	Number	2021	0.4786425	0.93754499	0.195892457
YLDs (Year	Morocco	Male	55+ years	Non-melanoma skin cancer (basal-cell	Percent	2021	4.54E-07	8.49E-07	2.06E-07
YLDs (Year	Morocco	Female	55+ years	Non-melanoma skin cancer (basal-cell	Percent	2021	2.46E-07	4.69E-07	1.08E-07
YLDs (Year	Morocco	Both	55+ years	Non-melanoma skin cancer (basal-cell	Percent	2021	3.42E-07	6.44E-07	1.53E-07
YLDs (Year	Morocco	Male	55+ years	Non-melanoma skin cancer (basal-cell	Rate	2021	0.00976	0.019312769	0.00404113
YLDs (Year	Morocco	Female	55+ years	Non-melanoma skin cancer (basal-cell	Rate	2021	0.0061856	0.012001838	0.002485188
YLDs (Year	Morocco	Both	55+ years	Non-melanoma skin cancer (basal-cell	Rate	2021	0.0079668	0.015604983	0.003260535
YLDs (Year	Tunisia	Male	55+ years	Non-melanoma skin cancer (basal-cell	Number	1990	0.0916681	0.177595372	0.038788751
YLDs (Year	Tunisia	Female	55+ years	Non-melanoma skin cancer (basal-cell	Number	1990	0.0583717	0.11315776	0.023396912
YLDs (Year	Tunisia	Both	55+ years	Non-melanoma skin cancer (basal-cell	Number	1990	0.1500398	0.292690651	0.062616386
YLDs (Year	Tunisia	Male	55+ years	Non-melanoma skin cancer (basal-cell	Percent	1990	1.06E-06	1.92E-06	4.92E-07
YLDs (Year	Tunisia	Female	55+ years	Non-melanoma skin cancer (basal-cell	Percent	1990	6.43E-07	1.22E-06	2.84E-07
YLDs (Year	Tunisia	Both	55+ years	Non-melanoma skin cancer (basal-cell	Percent	1990	8.46E-07	1.58E-06	3.86E-07
YLDs (Year	Tunisia	Male	55+ years	Non-melanoma skin cancer (basal-cell	Rate	1990	0.0202458	0.039223763	0.008566894
YLDs (Year	Tunisia	Female	55+ years	Non-melanoma skin cancer (basal-cell	Rate	1990	0.0139663	0.027074724	0.005598069
YLDs (Year	Tunisia	Both	55+ years	Non-melanoma skin cancer (basal-cell	Rate	1990	0.0172317	0.033614743	0.007191325
YLDs (Year	Tunisia	Male	55+ years	Non-melanoma skin cancer (basal-cell	Number	2021	0.2180266	0.41555001	0.091469564
YLDs (Year	Tunisia	Female	55+ years	Non-melanoma skin cancer (basal-cell	Number	2021	0.161707	0.31847813	0.066151214
YLDs (Year	Tunisia	Both	55+ years	Non-melanoma skin cancer (basal-cell	Number	2021	0.3797336	0.739435809	0.155776516
YLDs (Year	Tunisia	Male	55+ years	Non-melanoma skin cancer (basal-cell	Percent	2021	8.94E-07	1.67E-06	4.12E-07
YLDs (Year	Tunisia	Female	55+ years	Non-melanoma skin cancer (basal-cell	Percent	2021	5.52E-07	1.02E-06	2.44E-07
YLDs (Year	Tunisia	Both	55+ years	Non-melanoma skin cancer (basal-cell	Percent	2021	7.07E-07	1.32E-06	3.16E-07
YLDs (Year	Tunisia	Male	55+ years	Non-melanoma skin cancer (basal-cell	Rate	2021	0.0191349	0.03647031	0.00802773
YLDs (Year	Tunisia	Female	55+ years	Non-melanoma skin cancer (basal-cell	Rate	2021	0.0135013	0.026590492	0.005523121
YLDs (Year	Tunisia	Both	55+ years	Non-melanoma skin cancer (basal-cell	Rate	2021	0.0162478	0.031638567	0.006665279
YLDs (Year	India	Male	55+ years	Non-melanoma skin cancer (basal-cell	Number	1990	1.2580024	2.477950217	0.510643953
YLDs (Year	India	Female	55+ years	Non-melanoma skin cancer (basal-cell	Number	1990	1.0436007	2.080960245	0.41583866
YLDs (Year	India	Both	55+ years	Non-melanoma skin cancer (basal-cell	Number	1990	2.3016031	4.563870431	0.928578966
YLDs (Year	India	Male	55+ years	Non-melanoma skin cancer (basal-cell	Percent	1990	1.42E-07	2.69E-07	6.07E-08
YLDs (Year	India	Female	55+ years	Non-melanoma skin cancer (basal-cell	Percent	1990	1.10E-07	2.11E-07	4.65E-08
YLDs (Year	India	Both	55+ years	Non-melanoma skin cancer (basal-cell	Percent	1990	1.25E-07	2.37E-07	5.33E-08
YLDs (Year	India	Male	55+ years	Non-melanoma skin cancer (basal-cell	Rate	1990	0.0031661	0.006236354	0.001285158
YLDs (Year	India	Female	55+ years	Non-melanoma skin cancer (basal-cell	Rate	1990	0.0028255	0.005634088	0.001125861
YLDs (Year	India	Both	55+ years	Non-melanoma skin cancer (basal-cell	Rate	1990	0.003002	0.005952683	0.001211151

YLDs (Year	India	Male	55+ years	Non-melanoma skin cancer (basal-cell	Number	2021	3.4206265	6.694652984	1.397496324
YLDs (Year	India	Female	55+ years	Non-melanoma skin cancer (basal-cell	Number	2021	3.2615965	6.423874042	1.308458312
YLDs (Year	India	Both	55+ years	Non-melanoma skin cancer (basal-cell	Number	2021	6.682223	13.01334361	2.722770492
YLDs (Year	India	Male	55+ years	Non-melanoma skin cancer (basal-cell	Percent	2021	1.59E-07	2.94E-07	7.17E-08
YLDs (Year	India	Female	55+ years	Non-melanoma skin cancer (basal-cell	Percent	2021	1.21E-07	2.30E-07	5.31E-08
YLDs (Year	India	Both	55+ years	Non-melanoma skin cancer (basal-cell	Percent	2021	1.38E-07	2.55E-07	6.14E-08
YLDs (Year	India	Male	55+ years	Non-melanoma skin cancer (basal-cell	Rate	2021	0.003521	0.006891002	0.001438484
YLDs (Year	India	Female	55+ years	Non-melanoma skin cancer (basal-cell	Rate	2021	0.0031393	0.006183061	0.001259408
YLDs (Year	India	Both	55+ years	Non-melanoma skin cancer (basal-cell	Rate	2021	0.0033237	0.006472839	0.001354307
YLDs (Year	Türkiye	Male	55+ years	Non-melanoma skin cancer (basal-cell	Number	1990	0.9635706	1.96381096	0.432703949
YLDs (Year	Türkiye	Female	55+ years	Non-melanoma skin cancer (basal-cell	Number	1990	0.9573069	1.892884368	0.435044551
YLDs (Year	Türkiye	Both	55+ years	Non-melanoma skin cancer (basal-cell	Number	1990	1.9208775	3.946401373	0.86935855
YLDs (Year	Türkiye	Male	55+ years	Non-melanoma skin cancer (basal-cell	Percent	1990	1.85E-06	3.35E-06	8.72E-07
YLDs (Year	Türkiye	Female	55+ years	Non-melanoma skin cancer (basal-cell	Percent	1990	1.42E-06	2.54E-06	6.65E-07
YLDs (Year	Türkiye	Both	55+ years	Non-melanoma skin cancer (basal-cell	Percent	1990	1.61E-06	2.87E-06	7.54E-07
YLDs (Year	Türkiye	Male	55+ years	Non-melanoma skin cancer (basal-cell	Rate	1990	0.0338326	0.068952665	0.015192954
YLDs (Year	Türkiye	Female	55+ years	Non-melanoma skin cancer (basal-cell	Rate	1990	0.0306195	0.06054389	0.013914896
YLDs (Year	Türkiye	Both	55+ years	Non-melanoma skin cancer (basal-cell	Rate	1990	0.0321511	0.066053832	0.014551096
YLDs (Year	Türkiye	Male	55+ years	Non-melanoma skin cancer (basal-cell	Number	2021	2.2101382	4.232907636	0.939047478
YLDs (Year	Türkiye	Female	55+ years	Non-melanoma skin cancer (basal-cell	Number	2021	1.966581	3.65942012	0.806981563
YLDs (Year	Türkiye	Both	55+ years	Non-melanoma skin cancer (basal-cell	Number	2021	4.1767192	7.851563128	1.744404046
YLDs (Year	Türkiye	Male	55+ years	Non-melanoma skin cancer (basal-cell	Percent	2021	1.45E-06	2.66E-06	6.58E-07
YLDs (Year	Türkiye	Female	55+ years	Non-melanoma skin cancer (basal-cell	Percent	2021	9.58E-07	1.72E-06	4.45E-07
YLDs (Year	Türkiye	Both	55+ years	Non-melanoma skin cancer (basal-cell	Percent	2021	1.17E-06	2.12E-06	5.37E-07
YLDs (Year	Türkiye	Male	55+ years	Non-melanoma skin cancer (basal-cell	Rate	2021	0.0283522	0.054300702	0.012046315
YLDs (Year	Türkiye	Female	55+ years	Non-melanoma skin cancer (basal-cell	Rate	2021	0.0225372	0.041937232	0.00924807
YLDs (Year	Türkiye	Both	55+ years	Non-melanoma skin cancer (basal-cell	Rate	2021	0.0252809	0.047524011	0.010558544
YLDs (Year	Qatar	Male	55+ years	Non-melanoma skin cancer (basal-cell	Number	1990	0.0011549	0.002288898	0.000446039
YLDs (Year	Qatar	Female	55+ years	Non-melanoma skin cancer (basal-cell	Number	1990	0.0003361	0.000716139	0.00013079
YLDs (Year	Qatar	Both	55+ years	Non-melanoma skin cancer (basal-cell	Number	1990	0.0014909	0.002975343	0.00057352
YLDs (Year	Qatar	Male	55+ years	Non-melanoma skin cancer (basal-cell	Percent	1990	5.69E-07	1.08E-06	2.33E-07
YLDs (Year	Qatar	Female	55+ years	Non-melanoma skin cancer (basal-cell	Percent	1990	2.96E-07	6.05E-07	1.25E-07
YLDs (Year	Qatar	Both	55+ years	Non-melanoma skin cancer (basal-cell	Percent	1990	4.71E-07	9.11E-07	1.94E-07
YLDs (Year	Qatar	Male	55+ years	Non-melanoma skin cancer (basal-cell	Rate	1990	0.010194	0.020203994	0.003937164
YLDs (Year	Qatar	Female	55+ years	Non-melanoma skin cancer (basal-cell	Rate	1990	0.0067129	0.014304626	0.002612486
YLDs (Year	Qatar	Both	55+ years	Non-melanoma skin cancer (basal-cell	Rate	1990	0.0091272	0.018214212	0.003510927
YLDs (Year	Qatar	Male	55+ years	Non-melanoma skin cancer (basal-cell	Number	2021	0.0103579	0.021041296	0.004040363
YLDs (Year	Qatar	Female	55+ years	Non-melanoma skin cancer (basal-cell	Number	2021	0.003079	0.00659775	0.001179616
YLDs (Year	Qatar	Both	55+ years	Non-melanoma skin cancer (basal-cell	Number	2021	0.0134369	0.02727664	0.005102687
YLDs (Year	Qatar	Male	55+ years	Non-melanoma skin cancer (basal-cell	Percent	2021	5.10E-07	1.02E-06	2.06E-07
YLDs (Year	Qatar	Female	55+ years	Non-melanoma skin cancer (basal-cell	Percent	2021	2.58E-07	5.25E-07	1.04E-07
YLDs (Year	Qatar	Both	55+ years	Non-melanoma skin cancer (basal-cell	Percent	2021	4.17E-07	8.41E-07	1.67E-07
YLDs (Year	Qatar	Male	55+ years	Non-melanoma skin cancer (basal-cell	Rate	2021	0.0098353	0.019979801	0.003836534
YLDs (Year	Qatar	Female	55+ years	Non-melanoma skin cancer (basal-cell	Rate	2021	0.0064319	0.013782283	0.002464143
YLDs (Year	Qatar	Both	55+ years	Non-melanoma skin cancer (basal-cell	Rate	2021	0.0087717	0.017806445	0.003331082
YLDs (Year	Saudi Arab	Male	55+ years	Non-melanoma skin cancer (basal-cell	Number	1990	0.0672953	0.128746636	0.027947861
YLDs (Year	Saudi Arab	Female	55+ years	Non-melanoma skin cancer (basal-cell	Number	1990	0.031321	0.059988747	0.012537667
YLDs (Year	Saudi Arab	Both	55+ years	Non-melanoma skin cancer (basal-cell	Number	1990	0.0986163	0.18899885	0.040403761
YLDs (Year	Saudi Arab	Male	55+ years	Non-melanoma skin cancer (basal-cell	Percent	1990	6.08E-07	1.12E-06	2.75E-07
YLDs (Year	Saudi Arab	Female	55+ years	Non-melanoma skin cancer (basal-cell	Percent	1990	3.50E-07	6.28E-07	1.53E-07
YLDs (Year	Saudi Arab	Both	55+ years	Non-melanoma skin cancer (basal-cell	Percent	1990	4.93E-07	9.03E-07	2.20E-07
YLDs (Year	Saudi Arab	Male	55+ years	Non-melanoma skin cancer (basal-cell	Rate	1990	0.0128971	0.02467416	0.005356179
YLDs (Year	Saudi Arab	Female	55+ years	Non-melanoma skin cancer (basal-cell	Rate	1990	0.0084854	0.016251979	0.003396669
YLDs (Year	Saudi Arab	Both	55+ years	Non-melanoma skin cancer (basal-cell	Rate	1990	0.0110692	0.02121428	0.004535142
YLDs (Year	Saudi Arab	Male	55+ years	Non-melanoma skin cancer (basal-cell	Number	2021	0.1975098	0.385802374	0.087935309
YLDs (Year	Saudi Arab	Female	55+ years	Non-melanoma skin cancer (basal-cell	Number	2021	0.0833523	0.161535749	0.036931509

YLDs (Year	Saudi Arab	Both	55+ years	Non-melanoma skin cancer (basal-cell	Number	2021	0.2808622	0.55119899	0.124900188
YLDs (Year	Saudi Arab	Male	55+ years	Non-melanoma skin cancer (basal-cell	Percent	2021	5.18E-07	9.04E-07	2.53E-07
YLDs (Year	Saudi Arab	Female	55+ years	Non-melanoma skin cancer (basal-cell	Percent	2021	3.02E-07	5.50E-07	1.46E-07
YLDs (Year	Saudi Arab	Both	55+ years	Non-melanoma skin cancer (basal-cell	Percent	2021	4.28E-07	7.52E-07	2.08E-07
YLDs (Year	Saudi Arab	Male	55+ years	Non-melanoma skin cancer (basal-cell	Rate	2021	0.0109421	0.021373647	0.004871661
YLDs (Year	Saudi Arab	Female	55+ years	Non-melanoma skin cancer (basal-cell	Rate	2021	0.0069134	0.013398066	0.003063166
YLDs (Year	Saudi Arab	Both	55+ years	Non-melanoma skin cancer (basal-cell	Rate	2021	0.0093288	0.018307987	0.00414854
YLDs (Year	Syrian Ara	Male	55+ years	Non-melanoma skin cancer (basal-cell	Number	1990	0.0710614	0.139183107	0.029337898
YLDs (Year	Syrian Ara	Female	55+ years	Non-melanoma skin cancer (basal-cell	Number	1990	0.0367514	0.072295301	0.015331036
YLDs (Year	Syrian Ara	Both	55+ years	Non-melanoma skin cancer (basal-cell	Number	1990	0.1078127	0.21201912	0.04444317
YLDs (Year	Syrian Ara	Male	55+ years	Non-melanoma skin cancer (basal-cell	Percent	1990	8.21E-07	1.51E-06	3.69E-07
YLDs (Year	Syrian Ara	Female	55+ years	Non-melanoma skin cancer (basal-cell	Percent	1990	4.04E-07	7.59E-07	1.76E-07
YLDs (Year	Syrian Ara	Both	55+ years	Non-melanoma skin cancer (basal-cell	Percent	1990	6.07E-07	1.13E-06	2.73E-07
YLDs (Year	Syrian Ara	Male	55+ years	Non-melanoma skin cancer (basal-cell	Rate	1990	0.0152623	0.029893273	0.006301094
YLDs (Year	Syrian Ara	Female	55+ years	Non-melanoma skin cancer (basal-cell	Rate	1990	0.0088329	0.01737561	0.003684695
YLDs (Year	Syrian Ara	Both	55+ years	Non-melanoma skin cancer (basal-cell	Rate	1990	0.0122282	0.02404735	0.005040774
YLDs (Year	Syrian Ara	Male	55+ years	Non-melanoma skin cancer (basal-cell	Number	2021	0.1811597	0.350173007	0.075020294
YLDs (Year	Syrian Ara	Female	55+ years	Non-melanoma skin cancer (basal-cell	Number	2021	0.1011879	0.197422327	0.041417211
YLDs (Year	Syrian Ara	Both	55+ years	Non-melanoma skin cancer (basal-cell	Number	2021	0.2823476	0.540568235	0.115308586
YLDs (Year	Syrian Ara	Male	55+ years	Non-melanoma skin cancer (basal-cell	Percent	2021	7.60E-07	1.38E-06	3.52E-07
YLDs (Year	Syrian Ara	Female	55+ years	Non-melanoma skin cancer (basal-cell	Percent	2021	3.86E-07	7.19E-07	1.70E-07
YLDs (Year	Syrian Ara	Both	55+ years	Non-melanoma skin cancer (basal-cell	Percent	2021	5.64E-07	1.03E-06	2.55E-07
YLDs (Year	Syrian Ara	Male	55+ years	Non-melanoma skin cancer (basal-cell	Rate	2021	0.0149543	0.028905915	0.00619274
YLDs (Year	Syrian Ara	Female	55+ years	Non-melanoma skin cancer (basal-cell	Rate	2021	0.0087892	0.017148073	0.003597493
YLDs (Year	Syrian Ara	Both	55+ years	Non-melanoma skin cancer (basal-cell	Rate	2021	0.0119502	0.022879226	0.004880367
YLDs (Year	Yemen	Male	55+ years	Non-melanoma skin cancer (basal-cell	Number	1990	0.0499937	0.0993942	0.020728257
YLDs (Year	Yemen	Female	55+ years	Non-melanoma skin cancer (basal-cell	Number	1990	0.0337443	0.066420946	0.014267294
YLDs (Year	Yemen	Both	55+ years	Non-melanoma skin cancer (basal-cell	Number	1990	0.083738	0.163544455	0.03462563
YLDs (Year	Yemen	Male	55+ years	Non-melanoma skin cancer (basal-cell	Percent	1990	6.71E-07	1.25E-06	3.02E-07
YLDs (Year	Yemen	Female	55+ years	Non-melanoma skin cancer (basal-cell	Percent	1990	3.53E-07	6.64E-07	1.60E-07
YLDs (Year	Yemen	Both	55+ years	Non-melanoma skin cancer (basal-cell	Percent	1990	4.92E-07	9.38E-07	2.21E-07
YLDs (Year	Yemen	Male	55+ years	Non-melanoma skin cancer (basal-cell	Rate	1990	0.0126402	0.025130443	0.005240852
YLDs (Year	Yemen	Female	55+ years	Non-melanoma skin cancer (basal-cell	Rate	1990	0.008301	0.016339418	0.003509725
YLDs (Year	Yemen	Both	55+ years	Non-melanoma skin cancer (basal-cell	Rate	1990	0.0104409	0.020391554	0.0043173
YLDs (Year	Yemen	Male	55+ years	Non-melanoma skin cancer (basal-cell	Number	2021	0.1504846	0.288011943	0.061702414
YLDs (Year	Yemen	Female	55+ years	Non-melanoma skin cancer (basal-cell	Number	2021	0.0934228	0.177926694	0.038619785
YLDs (Year	Yemen	Both	55+ years	Non-melanoma skin cancer (basal-cell	Number	2021	0.2439074	0.4593723	0.101980484
YLDs (Year	Yemen	Male	55+ years	Non-melanoma skin cancer (basal-cell	Percent	2021	6.93E-07	1.27E-06	3.16E-07
YLDs (Year	Yemen	Female	55+ years	Non-melanoma skin cancer (basal-cell	Percent	2021	3.42E-07	6.30E-07	1.54E-07
YLDs (Year	Yemen	Both	55+ years	Non-melanoma skin cancer (basal-cell	Percent	2021	4.98E-07	8.97E-07	2.25E-07
YLDs (Year	Yemen	Male	55+ years	Non-melanoma skin cancer (basal-cell	Rate	2021	0.0137188	0.026256436	0.005625064
YLDs (Year	Yemen	Female	55+ years	Non-melanoma skin cancer (basal-cell	Rate	2021	0.0081096	0.015444941	0.003352394
YLDs (Year	Yemen	Both	55+ years	Non-melanoma skin cancer (basal-cell	Rate	2021	0.0108455	0.020426299	0.004534631
YLDs (Year	Bangladesh	Male	55+ years	Non-melanoma skin cancer (basal-cell	Number	1990	0.0050333	0.012805868	0.00106569
YLDs (Year	Bangladesh	Female	55+ years	Non-melanoma skin cancer (basal-cell	Number	1990	0.0027734	0.00718075	0.000632994
YLDs (Year	Bangladesh	Both	55+ years	Non-melanoma skin cancer (basal-cell	Number	1990	0.0078067	0.018638978	0.00208029
YLDs (Year	Bangladesh	Male	55+ years	Non-melanoma skin cancer (basal-cell	Percent	1990	5.62E-09	1.41E-08	1.20E-09
YLDs (Year	Bangladesh	Female	55+ years	Non-melanoma skin cancer (basal-cell	Percent	1990	3.33E-09	8.37E-09	7.60E-10
YLDs (Year	Bangladesh	Both	55+ years	Non-melanoma skin cancer (basal-cell	Percent	1990	4.52E-09	1.08E-08	1.22E-09
YLDs (Year	Bangladesh	Male	55+ years	Non-melanoma skin cancer (basal-cell	Rate	1990	0.0001195	0.000303954	2.53E-05
YLDs (Year	Bangladesh	Female	55+ years	Non-melanoma skin cancer (basal-cell	Rate	1990	8.25E-05	0.000213613	1.88E-05
YLDs (Year	Bangladesh	Both	55+ years	Non-melanoma skin cancer (basal-cell	Rate	1990	0.0001031	0.00024607	2.75E-05
YLDs (Year	Bangladesh	Male	55+ years	Non-melanoma skin cancer (basal-cell	Number	2021	0.0144703	0.037092103	0.00292725
YLDs (Year	Bangladesh	Female	55+ years	Non-melanoma skin cancer (basal-cell	Number	2021	0.0092011	0.024417865	0.00210599
YLDs (Year	Bangladesh	Both	55+ years	Non-melanoma skin cancer (basal-cell	Number	2021	0.0236713	0.056785977	0.006360139
YLDs (Year	Bangladesh	Male	55+ years	Non-melanoma skin cancer (basal-cell	Percent	2021	5.78E-09	1.45E-08	1.25E-09

YLDs (Year	Bangladesh	Female	55+ years	Non-melanoma skin cancer (basal-cell	Percent	2021	3.32E-09	8.69E-09	7.88E-10
YLDs (Year	Bangladesh	Both	55+ years	Non-melanoma skin cancer (basal-cell	Percent	2021	4.49E-09	1.06E-08	1.25E-09
YLDs (Year	Bangladesh	Male	55+ years	Non-melanoma skin cancer (basal-cell	Rate	2021	0.0001198	0.000306962	2.42E-05
YLDs (Year	Bangladesh	Female	55+ years	Non-melanoma skin cancer (basal-cell	Rate	2021	8.16E-05	0.000216422	1.87E-05
YLDs (Year	Bangladesh	Both	55+ years	Non-melanoma skin cancer (basal-cell	Rate	2021	0.0001013	0.000243027	2.72E-05
YLDs (Year	Bhutan	Male	55+ years	Non-melanoma skin cancer (basal-cell	Number	1990	2.25E-05	6.36E-05	4.58E-06
YLDs (Year	Bhutan	Female	55+ years	Non-melanoma skin cancer (basal-cell	Number	1990	1.51E-05	3.83E-05	3.51E-06
YLDs (Year	Bhutan	Both	55+ years	Non-melanoma skin cancer (basal-cell	Number	1990	3.76E-05	9.22E-05	9.75E-06
YLDs (Year	Bhutan	Male	55+ years	Non-melanoma skin cancer (basal-cell	Percent	1990	5.77E-09	1.52E-08	1.17E-09
YLDs (Year	Bhutan	Female	55+ years	Non-melanoma skin cancer (basal-cell	Percent	1990	3.35E-09	8.57E-09	7.72E-10
YLDs (Year	Bhutan	Both	55+ years	Non-melanoma skin cancer (basal-cell	Percent	1990	4.47E-09	1.14E-08	1.19E-09
YLDs (Year	Bhutan	Male	55+ years	Non-melanoma skin cancer (basal-cell	Rate	1990	0.0001123	0.000317183	2.28E-05
YLDs (Year	Bhutan	Female	55+ years	Non-melanoma skin cancer (basal-cell	Rate	1990	7.68E-05	0.000194921	1.79E-05
YLDs (Year	Bhutan	Both	55+ years	Non-melanoma skin cancer (basal-cell	Rate	1990	9.47E-05	0.000232362	2.46E-05
YLDs (Year	Bhutan	Male	55+ years	Non-melanoma skin cancer (basal-cell	Number	2021	6.17E-05	0.000153808	1.26E-05
YLDs (Year	Bhutan	Female	55+ years	Non-melanoma skin cancer (basal-cell	Number	2021	3.88E-05	0.000102022	8.64E-06
YLDs (Year	Bhutan	Both	55+ years	Non-melanoma skin cancer (basal-cell	Number	2021	0.0001005	0.000240006	2.51E-05
YLDs (Year	Bhutan	Male	55+ years	Non-melanoma skin cancer (basal-cell	Percent	2021	5.95E-09	1.43E-08	1.28E-09
YLDs (Year	Bhutan	Female	55+ years	Non-melanoma skin cancer (basal-cell	Percent	2021	3.34E-09	8.46E-09	7.88E-10
YLDs (Year	Bhutan	Both	55+ years	Non-melanoma skin cancer (basal-cell	Percent	2021	4.57E-09	1.08E-08	1.23E-09
YLDs (Year	Bhutan	Male	55+ years	Non-melanoma skin cancer (basal-cell	Rate	2021	0.0001221	0.000304408	2.50E-05
YLDs (Year	Bhutan	Female	55+ years	Non-melanoma skin cancer (basal-cell	Rate	2021	7.92E-05	0.000207939	1.76E-05
YLDs (Year	Bhutan	Both	55+ years	Non-melanoma skin cancer (basal-cell	Rate	2021	0.0001009	0.000240994	2.52E-05
YLDs (Year	Nepal	Male	55+ years	Non-melanoma skin cancer (basal-cell	Number	1990	0.0008479	0.002296265	0.000171014
YLDs (Year	Nepal	Female	55+ years	Non-melanoma skin cancer (basal-cell	Number	1990	0.0005629	0.001413618	0.000130445
YLDs (Year	Nepal	Both	55+ years	Non-melanoma skin cancer (basal-cell	Number	1990	0.0014108	0.003410853	0.00035643
YLDs (Year	Nepal	Male	55+ years	Non-melanoma skin cancer (basal-cell	Percent	1990	4.63E-09	1.17E-08	9.46E-10
YLDs (Year	Nepal	Female	55+ years	Non-melanoma skin cancer (basal-cell	Percent	1990	3.02E-09	7.88E-09	7.16E-10
YLDs (Year	Nepal	Both	55+ years	Non-melanoma skin cancer (basal-cell	Percent	1990	3.82E-09	9.22E-09	1.01E-09
YLDs (Year	Nepal	Male	55+ years	Non-melanoma skin cancer (basal-cell	Rate	1990	0.0001063	0.00028789	2.14E-05
YLDs (Year	Nepal	Female	55+ years	Non-melanoma skin cancer (basal-cell	Rate	1990	7.59E-05	0.000190591	1.76E-05
YLDs (Year	Nepal	Both	55+ years	Non-melanoma skin cancer (basal-cell	Rate	1990	9.16E-05	0.000221582	2.32E-05
YLDs (Year	Nepal	Male	55+ years	Non-melanoma skin cancer (basal-cell	Number	2021	0.0020538	0.005326002	0.000396735
YLDs (Year	Nepal	Female	55+ years	Non-melanoma skin cancer (basal-cell	Number	2021	0.0016232	0.004118631	0.000375155
YLDs (Year	Nepal	Both	55+ years	Non-melanoma skin cancer (basal-cell	Number	2021	0.003677	0.008943396	0.000956223
YLDs (Year	Nepal	Male	55+ years	Non-melanoma skin cancer (basal-cell	Percent	2021	4.83E-09	1.22E-08	1.01E-09
YLDs (Year	Nepal	Female	55+ years	Non-melanoma skin cancer (basal-cell	Percent	2021	3.05E-09	8.01E-09	7.23E-10
YLDs (Year	Nepal	Both	55+ years	Non-melanoma skin cancer (basal-cell	Percent	2021	3.84E-09	9.24E-09	1.05E-09
YLDs (Year	Nepal	Male	55+ years	Non-melanoma skin cancer (basal-cell	Rate	2021	0.0001091	0.000282889	2.11E-05
YLDs (Year	Nepal	Female	55+ years	Non-melanoma skin cancer (basal-cell	Rate	2021	7.82E-05	0.000198475	1.81E-05
YLDs (Year	Nepal	Both	55+ years	Non-melanoma skin cancer (basal-cell	Rate	2021	9.29E-05	0.000225966	2.42E-05
YLDs (Year	Afghanista	Male	55+ years	Non-melanoma skin cancer (basal-cell	Number	1990	0.0856649	0.167653247	0.033386552
YLDs (Year	Afghanista	Female	55+ years	Non-melanoma skin cancer (basal-cell	Number	1990	0.0397536	0.077148207	0.016221392
YLDs (Year	Afghanista	Both	55+ years	Non-melanoma skin cancer (basal-cell	Number	1990	0.1254184	0.242313815	0.049145133
YLDs (Year	Afghanista	Male	55+ years	Non-melanoma skin cancer (basal-cell	Percent	1990	6.22E-07	1.15E-06	2.74E-07
YLDs (Year	Afghanista	Female	55+ years	Non-melanoma skin cancer (basal-cell	Percent	1990	3.01E-07	5.60E-07	1.34E-07
YLDs (Year	Afghanista	Both	55+ years	Non-melanoma skin cancer (basal-cell	Percent	1990	4.65E-07	8.69E-07	2.04E-07
YLDs (Year	Afghanista	Male	55+ years	Non-melanoma skin cancer (basal-cell	Rate	1990	0.0132535	0.025938179	0.005165342
YLDs (Year	Afghanista	Female	55+ years	Non-melanoma skin cancer (basal-cell	Rate	1990	0.0069887	0.013562646	0.002851719
YLDs (Year	Afghanista	Both	55+ years	Non-melanoma skin cancer (basal-cell	Rate	1990	0.0103209	0.019940478	0.004044249
YLDs (Year	Afghanista	Male	55+ years	Non-melanoma skin cancer (basal-cell	Number	2021	0.0760354	0.148161736	0.030028722
YLDs (Year	Afghanista	Female	55+ years	Non-melanoma skin cancer (basal-cell	Number	2021	0.0517749	0.100542647	0.021489575
YLDs (Year	Afghanista	Both	55+ years	Non-melanoma skin cancer (basal-cell	Number	2021	0.1278103	0.246938189	0.051144386
YLDs (Year	Afghanista	Male	55+ years	Non-melanoma skin cancer (basal-cell	Percent	2021	5.43E-07	1.01E-06	2.58E-07
YLDs (Year	Afghanista	Female	55+ years	Non-melanoma skin cancer (basal-cell	Percent	2021	2.72E-07	4.99E-07	1.24E-07
YLDs (Year	Afghanista	Both	55+ years	Non-melanoma skin cancer (basal-cell	Percent	2021	3.87E-07	7.31E-07	1.82E-07

YLDs (Year	Afghanista	Male	55+ years	Non-melanoma skin cancer (basal-cell	Rate	2021	0.0145607	0.028372884	0.005750482
YLDs (Year	Afghanista	Female	55+ years	Non-melanoma skin cancer (basal-cell	Rate	2021	0.0073278	0.014229953	0.003041452
YLDs (Year	Afghanista	Both	55+ years	Non-melanoma skin cancer (basal-cell	Rate	2021	0.0104016	0.020096678	0.004162306
YLDs (Year	Pakistan	Male	55+ years	Non-melanoma skin cancer (basal-cell	Number	1990	0.0333026	0.065378809	0.013330001
YLDs (Year	Pakistan	Female	55+ years	Non-melanoma skin cancer (basal-cell	Number	1990	0.021795	0.044514596	0.008533114
YLDs (Year	Pakistan	Both	55+ years	Non-melanoma skin cancer (basal-cell	Number	1990	0.0550976	0.11067391	0.022314163
YLDs (Year	Pakistan	Male	55+ years	Non-melanoma skin cancer (basal-cell	Percent	1990	3.02E-08	5.61E-08	1.33E-08
YLDs (Year	Pakistan	Female	55+ years	Non-melanoma skin cancer (basal-cell	Percent	1990	2.26E-08	4.44E-08	1.03E-08
YLDs (Year	Pakistan	Both	55+ years	Non-melanoma skin cancer (basal-cell	Percent	1990	2.66E-08	5.02E-08	1.20E-08
YLDs (Year	Pakistan	Male	55+ years	Non-melanoma skin cancer (basal-cell	Rate	1990	0.0006486	0.001273241	0.0002596
YLDs (Year	Pakistan	Female	55+ years	Non-melanoma skin cancer (basal-cell	Rate	1990	0.000547	0.001117155	0.00021415
YLDs (Year	Pakistan	Both	55+ years	Non-melanoma skin cancer (basal-cell	Rate	1990	0.0006042	0.0012136	0.000244687
YLDs (Year	Pakistan	Male	55+ years	Non-melanoma skin cancer (basal-cell	Number	2021	0.0503755	0.107318494	0.020034957
YLDs (Year	Pakistan	Female	55+ years	Non-melanoma skin cancer (basal-cell	Number	2021	0.0387736	0.080018372	0.015675009
YLDs (Year	Pakistan	Both	55+ years	Non-melanoma skin cancer (basal-cell	Number	2021	0.0891491	0.188132014	0.0352939
YLDs (Year	Pakistan	Male	55+ years	Non-melanoma skin cancer (basal-cell	Percent	2021	2.25E-08	4.45E-08	9.55E-09
YLDs (Year	Pakistan	Female	55+ years	Non-melanoma skin cancer (basal-cell	Percent	2021	1.68E-08	3.19E-08	7.00E-09
YLDs (Year	Pakistan	Both	55+ years	Non-melanoma skin cancer (basal-cell	Percent	2021	1.96E-08	3.81E-08	8.25E-09
YLDs (Year	Pakistan	Male	55+ years	Non-melanoma skin cancer (basal-cell	Rate	2021	0.000479	0.001020507	0.000190515
YLDs (Year	Pakistan	Female	55+ years	Non-melanoma skin cancer (basal-cell	Rate	2021	0.0004165	0.000859506	0.000168371
YLDs (Year	Pakistan	Both	55+ years	Non-melanoma skin cancer (basal-cell	Rate	2021	0.0004497	0.000948916	0.000178018
YLDs (Year	United Ara	Male	55+ years	Non-melanoma skin cancer (basal-cell	Number	1990	0.0041952	0.008268748	0.001701478
YLDs (Year	United Ara	Female	55+ years	Non-melanoma skin cancer (basal-cell	Number	1990	0.0016412	0.003203479	0.000686237
YLDs (Year	United Ara	Both	55+ years	Non-melanoma skin cancer (basal-cell	Number	1990	0.0058365	0.011505157	0.002359636
YLDs (Year	United Ara	Male	55+ years	Non-melanoma skin cancer (basal-cell	Percent	1990	6.38E-07	1.17E-06	2.92E-07
YLDs (Year	United Ara	Female	55+ years	Non-melanoma skin cancer (basal-cell	Percent	1990	3.27E-07	6.17E-07	1.48E-07
YLDs (Year	United Ara	Both	55+ years	Non-melanoma skin cancer (basal-cell	Percent	1990	5.03E-07	9.38E-07	2.28E-07
YLDs (Year	United Ara	Male	55+ years	Non-melanoma skin cancer (basal-cell	Rate	1990	0.0120672	0.023784351	0.004894158
YLDs (Year	United Ara	Female	55+ years	Non-melanoma skin cancer (basal-cell	Rate	1990	0.0076837	0.01499744	0.003212694
YLDs (Year	United Ara	Both	55+ years	Non-melanoma skin cancer (basal-cell	Rate	1990	0.0103989	0.02049892	0.004204201
YLDs (Year	United Ara	Male	55+ years	Non-melanoma skin cancer (basal-cell	Number	2021	0.0565786	0.114616037	0.022601089
YLDs (Year	United Ara	Female	55+ years	Non-melanoma skin cancer (basal-cell	Number	2021	0.009962	0.020248522	0.003896973
YLDs (Year	United Ara	Both	55+ years	Non-melanoma skin cancer (basal-cell	Number	2021	0.0665406	0.135496539	0.026715994
YLDs (Year	United Ara	Male	55+ years	Non-melanoma skin cancer (basal-cell	Percent	2021	5.57E-07	1.04E-06	2.39E-07
YLDs (Year	United Ara	Female	55+ years	Non-melanoma skin cancer (basal-cell	Percent	2021	2.67E-07	5.21E-07	1.10E-07
YLDs (Year	United Ara	Both	55+ years	Non-melanoma skin cancer (basal-cell	Percent	2021	4.79E-07	8.96E-07	2.05E-07
YLDs (Year	United Ara	Male	55+ years	Non-melanoma skin cancer (basal-cell	Rate	2021	0.0102792	0.020823462	0.00410617
YLDs (Year	United Ara	Female	55+ years	Non-melanoma skin cancer (basal-cell	Rate	2021	0.0059417	0.012077058	0.002324316
YLDs (Year	United Ara	Both	55+ years	Non-melanoma skin cancer (basal-cell	Rate	2021	0.0092665	0.018869313	0.003720482
YLDs (Year	Mauritius	Male	55+ years	Non-melanoma skin cancer (basal-cell	Number	1990	0.0015518	0.003036417	0.000600713
YLDs (Year	Mauritius	Female	55+ years	Non-melanoma skin cancer (basal-cell	Number	1990	0.0016842	0.003381428	0.000673639
YLDs (Year	Mauritius	Both	55+ years	Non-melanoma skin cancer (basal-cell	Number	1990	0.003236	0.006280848	0.001258986
YLDs (Year	Mauritius	Male	55+ years	Non-melanoma skin cancer (basal-cell	Percent	1990	1.43E-07	2.66E-07	6.18E-08
YLDs (Year	Mauritius	Female	55+ years	Non-melanoma skin cancer (basal-cell	Percent	1990	1.14E-07	2.18E-07	4.81E-08
YLDs (Year	Mauritius	Both	55+ years	Non-melanoma skin cancer (basal-cell	Percent	1990	1.26E-07	2.38E-07	5.51E-08
YLDs (Year	Mauritius	Male	55+ years	Non-melanoma skin cancer (basal-cell	Rate	1990	0.0028028	0.005484226	0.001084978
YLDs (Year	Mauritius	Female	55+ years	Non-melanoma skin cancer (basal-cell	Rate	1990	0.0025414	0.005102463	0.001016499
YLDs (Year	Mauritius	Both	55+ years	Non-melanoma skin cancer (basal-cell	Rate	1990	0.0026604	0.005163605	0.001035036
YLDs (Year	Mauritius	Male	55+ years	Non-melanoma skin cancer (basal-cell	Number	2021	0.0045326	0.008787868	0.001776692
YLDs (Year	Mauritius	Female	55+ years	Non-melanoma skin cancer (basal-cell	Number	2021	0.0047287	0.009130266	0.001849166
YLDs (Year	Mauritius	Both	55+ years	Non-melanoma skin cancer (basal-cell	Number	2021	0.0092613	0.017654591	0.003625711
YLDs (Year	Mauritius	Male	55+ years	Non-melanoma skin cancer (basal-cell	Percent	2021	1.37E-07	2.60E-07	6.05E-08
YLDs (Year	Mauritius	Female	55+ years	Non-melanoma skin cancer (basal-cell	Percent	2021	1.09E-07	2.13E-07	4.56E-08
YLDs (Year	Mauritius	Both	55+ years	Non-melanoma skin cancer (basal-cell	Percent	2021	1.21E-07	2.33E-07	5.27E-08
YLDs (Year	Mauritius	Male	55+ years	Non-melanoma skin cancer (basal-cell	Rate	2021	0.0029018	0.005626107	0.001137461
YLDs (Year	Mauritius	Female	55+ years	Non-melanoma skin cancer (basal-cell	Rate	2021	0.0025963	0.005012941	0.001015278

YLDs (Year	Mauritius	Both	55+ years	Non-melanoma skin cancer (basal-cell	Rate	2021	0.0027373	0.005218127	0.001071643
YLDs (Year	Kenya	Male	55+ years	Non-melanoma skin cancer (basal-cell	Number	1990	0.0602249	0.117064829	0.024800541
YLDs (Year	Kenya	Female	55+ years	Non-melanoma skin cancer (basal-cell	Number	1990	0.053234	0.106647623	0.021557271
YLDs (Year	Kenya	Both	55+ years	Non-melanoma skin cancer (basal-cell	Number	1990	0.113459	0.22559345	0.046836841
YLDs (Year	Kenya	Male	55+ years	Non-melanoma skin cancer (basal-cell	Percent	1990	4.53E-07	8.38E-07	2.05E-07
YLDs (Year	Kenya	Female	55+ years	Non-melanoma skin cancer (basal-cell	Percent	1990	3.35E-07	6.28E-07	1.45E-07
YLDs (Year	Kenya	Both	55+ years	Non-melanoma skin cancer (basal-cell	Percent	1990	3.88E-07	7.23E-07	1.72E-07
YLDs (Year	Kenya	Male	55+ years	Non-melanoma skin cancer (basal-cell	Rate	1990	0.0092386	0.017958017	0.003804461
YLDs (Year	Kenya	Female	55+ years	Non-melanoma skin cancer (basal-cell	Rate	1990	0.0078183	0.01566299	0.003166046
YLDs (Year	Kenya	Both	55+ years	Non-melanoma skin cancer (basal-cell	Rate	1990	0.008513	0.016926661	0.003514248
YLDs (Year	Kenya	Male	55+ years	Non-melanoma skin cancer (basal-cell	Number	2021	0.1436818	0.28193924	0.058704114
YLDs (Year	Kenya	Female	55+ years	Non-melanoma skin cancer (basal-cell	Number	2021	0.142867	0.28753501	0.058167097
YLDs (Year	Kenya	Both	55+ years	Non-melanoma skin cancer (basal-cell	Number	2021	0.2865488	0.569028077	0.117719336
YLDs (Year	Kenya	Male	55+ years	Non-melanoma skin cancer (basal-cell	Percent	2021	4.12E-07	7.55E-07	1.84E-07
YLDs (Year	Kenya	Female	55+ years	Non-melanoma skin cancer (basal-cell	Percent	2021	3.06E-07	5.70E-07	1.35E-07
YLDs (Year	Kenya	Both	55+ years	Non-melanoma skin cancer (basal-cell	Percent	2021	3.52E-07	6.48E-07	1.56E-07
YLDs (Year	Kenya	Male	55+ years	Non-melanoma skin cancer (basal-cell	Rate	2021	0.0082145	0.016118897	0.003356204
YLDs (Year	Kenya	Female	55+ years	Non-melanoma skin cancer (basal-cell	Rate	2021	0.0071733	0.014437025	0.002920548
YLDs (Year	Kenya	Both	55+ years	Non-melanoma skin cancer (basal-cell	Rate	2021	0.0076602	0.015211513	0.003146926
YLDs (Year	Rwanda	Male	55+ years	Non-melanoma skin cancer (basal-cell	Number	1990	0.0202838	0.039702289	0.008335135
YLDs (Year	Rwanda	Female	55+ years	Non-melanoma skin cancer (basal-cell	Number	1990	0.0159274	0.031592636	0.006468988
YLDs (Year	Rwanda	Both	55+ years	Non-melanoma skin cancer (basal-cell	Number	1990	0.0362112	0.072054475	0.014810605
YLDs (Year	Rwanda	Male	55+ years	Non-melanoma skin cancer (basal-cell	Percent	1990	4.92E-07	9.16E-07	2.23E-07
YLDs (Year	Rwanda	Female	55+ years	Non-melanoma skin cancer (basal-cell	Percent	1990	2.78E-07	5.28E-07	1.19E-07
YLDs (Year	Rwanda	Both	55+ years	Non-melanoma skin cancer (basal-cell	Percent	1990	3.68E-07	6.87E-07	1.64E-07
YLDs (Year	Rwanda	Male	55+ years	Non-melanoma skin cancer (basal-cell	Rate	1990	0.0097626	0.019108601	0.004011677
YLDs (Year	Rwanda	Female	55+ years	Non-melanoma skin cancer (basal-cell	Rate	1990	0.0061218	0.012142754	0.002486381
YLDs (Year	Rwanda	Both	55+ years	Non-melanoma skin cancer (basal-cell	Rate	1990	0.0077383	0.015397944	0.003165006
YLDs (Year	Rwanda	Male	55+ years	Non-melanoma skin cancer (basal-cell	Number	2021	0.0422078	0.083201621	0.017943235
YLDs (Year	Rwanda	Female	55+ years	Non-melanoma skin cancer (basal-cell	Number	2021	0.037626	0.074351627	0.015117563
YLDs (Year	Rwanda	Both	55+ years	Non-melanoma skin cancer (basal-cell	Number	2021	0.0798337	0.155393669	0.033299519
YLDs (Year	Rwanda	Male	55+ years	Non-melanoma skin cancer (basal-cell	Percent	2021	4.88E-07	9.06E-07	2.24E-07
YLDs (Year	Rwanda	Female	55+ years	Non-melanoma skin cancer (basal-cell	Percent	2021	2.81E-07	5.33E-07	1.24E-07
YLDs (Year	Rwanda	Both	55+ years	Non-melanoma skin cancer (basal-cell	Percent	2021	3.62E-07	6.69E-07	1.64E-07
YLDs (Year	Rwanda	Male	55+ years	Non-melanoma skin cancer (basal-cell	Rate	2021	0.0094483	0.018624851	0.004016629
YLDs (Year	Rwanda	Female	55+ years	Non-melanoma skin cancer (basal-cell	Rate	2021	0.0061607	0.012173975	0.002475276
YLDs (Year	Rwanda	Both	55+ years	Non-melanoma skin cancer (basal-cell	Rate	2021	0.0075495	0.014694908	0.003148992
YLDs (Year	Somalia	Male	55+ years	Non-melanoma skin cancer (basal-cell	Number	1990	0.0146397	0.02878194	0.006053352
YLDs (Year	Somalia	Female	55+ years	Non-melanoma skin cancer (basal-cell	Number	1990	0.0106549	0.021044843	0.004377899
YLDs (Year	Somalia	Both	55+ years	Non-melanoma skin cancer (basal-cell	Number	1990	0.0252946	0.05011763	0.010516101
YLDs (Year	Somalia	Male	55+ years	Non-melanoma skin cancer (basal-cell	Percent	1990	4.53E-07	8.38E-07	2.07E-07
YLDs (Year	Somalia	Female	55+ years	Non-melanoma skin cancer (basal-cell	Percent	1990	2.58E-07	4.79E-07	1.13E-07
YLDs (Year	Somalia	Both	55+ years	Non-melanoma skin cancer (basal-cell	Percent	1990	3.43E-07	6.29E-07	1.52E-07
YLDs (Year	Somalia	Male	55+ years	Non-melanoma skin cancer (basal-cell	Rate	1990	0.0086256	0.016958115	0.003566592
YLDs (Year	Somalia	Female	55+ years	Non-melanoma skin cancer (basal-cell	Rate	1990	0.0055976	0.01105608	0.002299965
YLDs (Year	Somalia	Both	55+ years	Non-melanoma skin cancer (basal-cell	Rate	1990	0.0070249	0.013918856	0.002920571
YLDs (Year	Somalia	Male	55+ years	Non-melanoma skin cancer (basal-cell	Number	2021	0.0336889	0.068959245	0.014181242
YLDs (Year	Somalia	Female	55+ years	Non-melanoma skin cancer (basal-cell	Number	2021	0.031454	0.062503989	0.013160403
YLDs (Year	Somalia	Both	55+ years	Non-melanoma skin cancer (basal-cell	Number	2021	0.0651429	0.132389593	0.02752899
YLDs (Year	Somalia	Male	55+ years	Non-melanoma skin cancer (basal-cell	Percent	2021	4.40E-07	8.22E-07	2.03E-07
YLDs (Year	Somalia	Female	55+ years	Non-melanoma skin cancer (basal-cell	Percent	2021	2.52E-07	4.74E-07	1.11E-07
YLDs (Year	Somalia	Both	55+ years	Non-melanoma skin cancer (basal-cell	Percent	2021	3.23E-07	6.02E-07	1.42E-07
YLDs (Year	Somalia	Male	55+ years	Non-melanoma skin cancer (basal-cell	Rate	2021	0.008684	0.017775702	0.003655515
YLDs (Year	Somalia	Female	55+ years	Non-melanoma skin cancer (basal-cell	Rate	2021	0.0056662	0.011259551	0.002370732
YLDs (Year	Somalia	Both	55+ years	Non-melanoma skin cancer (basal-cell	Rate	2021	0.0069076	0.014038288	0.002919111
YLDs (Year	Angola	Male	55+ years	Non-melanoma skin cancer (basal-cell	Number	1990	0.0360596	0.074193694	0.014612654

YLDs (Year	Angola	Female	55+ years	Non-melanoma skin cancer (basal-cell	Number	1990	0.0216236	0.043995359	0.009078848
YLDs (Year	Angola	Both	55+ years	Non-melanoma skin cancer (basal-cell	Number	1990	0.0576832	0.115375138	0.023945007
YLDs (Year	Angola	Male	55+ years	Non-melanoma skin cancer (basal-cell	Percent	1990	5.47E-07	1.01E-06	2.34E-07
YLDs (Year	Angola	Female	55+ years	Non-melanoma skin cancer (basal-cell	Percent	1990	3.17E-07	6.14E-07	1.39E-07
YLDs (Year	Angola	Both	55+ years	Non-melanoma skin cancer (basal-cell	Percent	1990	4.30E-07	7.93E-07	1.87E-07
YLDs (Year	Angola	Male	55+ years	Non-melanoma skin cancer (basal-cell	Rate	1990	0.0115087	0.023679395	0.004663722
YLDs (Year	Angola	Female	55+ years	Non-melanoma skin cancer (basal-cell	Rate	1990	0.0069847	0.01421106	0.002932583
YLDs (Year	Angola	Both	55+ years	Non-melanoma skin cancer (basal-cell	Rate	1990	0.0092603	0.01852192	0.003844048
YLDs (Year	Angola	Male	55+ years	Non-melanoma skin cancer (basal-cell	Number	2021	0.0978771	0.188444341	0.040200882
YLDs (Year	Angola	Female	55+ years	Non-melanoma skin cancer (basal-cell	Number	2021	0.073689	0.142820035	0.030307117
YLDs (Year	Angola	Both	55+ years	Non-melanoma skin cancer (basal-cell	Number	2021	0.1715661	0.329141762	0.070342253
YLDs (Year	Angola	Male	55+ years	Non-melanoma skin cancer (basal-cell	Percent	2021	5.25E-07	9.83E-07	2.29E-07
YLDs (Year	Angola	Female	55+ years	Non-melanoma skin cancer (basal-cell	Percent	2021	3.03E-07	5.56E-07	1.33E-07
YLDs (Year	Angola	Both	55+ years	Non-melanoma skin cancer (basal-cell	Percent	2021	4.00E-07	7.38E-07	1.76E-07
YLDs (Year	Angola	Male	55+ years	Non-melanoma skin cancer (basal-cell	Rate	2021	0.011314	0.021783052	0.004646984
YLDs (Year	Angola	Female	55+ years	Non-melanoma skin cancer (basal-cell	Rate	2021	0.0068963	0.013365978	0.002836327
YLDs (Year	Angola	Both	55+ years	Non-melanoma skin cancer (basal-cell	Rate	2021	0.0088727	0.01702196	0.003637834
YLDs (Year	Djibouti	Male	55+ years	Non-melanoma skin cancer (basal-cell	Number	1990	0.0010597	0.002189898	0.000429026
YLDs (Year	Djibouti	Female	55+ years	Non-melanoma skin cancer (basal-cell	Number	1990	0.0006643	0.001326244	0.000263994
YLDs (Year	Djibouti	Both	55+ years	Non-melanoma skin cancer (basal-cell	Number	1990	0.001724	0.003480326	0.000710179
YLDs (Year	Djibouti	Male	55+ years	Non-melanoma skin cancer (basal-cell	Percent	1990	5.37E-07	9.95E-07	2.38E-07
YLDs (Year	Djibouti	Female	55+ years	Non-melanoma skin cancer (basal-cell	Percent	1990	3.09E-07	5.86E-07	1.33E-07
YLDs (Year	Djibouti	Both	55+ years	Non-melanoma skin cancer (basal-cell	Percent	1990	4.18E-07	7.82E-07	1.84E-07
YLDs (Year	Djibouti	Male	55+ years	Non-melanoma skin cancer (basal-cell	Rate	1990	0.0095475	0.019730559	0.003865441
YLDs (Year	Djibouti	Female	55+ years	Non-melanoma skin cancer (basal-cell	Rate	1990	0.006415	0.012807674	0.002549421
YLDs (Year	Djibouti	Both	55+ years	Non-melanoma skin cancer (basal-cell	Rate	1990	0.0080355	0.016222203	0.003310225
YLDs (Year	Djibouti	Male	55+ years	Non-melanoma skin cancer (basal-cell	Number	2021	0.0054894	0.011070675	0.002250581
YLDs (Year	Djibouti	Female	55+ years	Non-melanoma skin cancer (basal-cell	Number	2021	0.002957	0.005858285	0.001147148
YLDs (Year	Djibouti	Both	55+ years	Non-melanoma skin cancer (basal-cell	Number	2021	0.0084464	0.016691907	0.003414884
YLDs (Year	Djibouti	Male	55+ years	Non-melanoma skin cancer (basal-cell	Percent	2021	5.21E-07	9.89E-07	2.25E-07
YLDs (Year	Djibouti	Female	55+ years	Non-melanoma skin cancer (basal-cell	Percent	2021	3.03E-07	5.72E-07	1.28E-07
YLDs (Year	Djibouti	Both	55+ years	Non-melanoma skin cancer (basal-cell	Percent	2021	4.16E-07	7.80E-07	1.79E-07
YLDs (Year	Djibouti	Male	55+ years	Non-melanoma skin cancer (basal-cell	Rate	2021	0.0095248	0.019208747	0.003904987
YLDs (Year	Djibouti	Female	55+ years	Non-melanoma skin cancer (basal-cell	Rate	2021	0.0064184	0.012715952	0.002489991
YLDs (Year	Djibouti	Both	55+ years	Non-melanoma skin cancer (basal-cell	Rate	2021	0.0081447	0.016095741	0.003292919
YLDs (Year	South Afri	Male	55+ years	Non-melanoma skin cancer (basal-cell	Number	1990	0.6448377	1.23373613	0.262163735
YLDs (Year	South Afri	Female	55+ years	Non-melanoma skin cancer (basal-cell	Number	1990	0.5732908	1.129255708	0.236033557
YLDs (Year	South Afri	Both	55+ years	Non-melanoma skin cancer (basal-cell	Number	1990	1.2181285	2.351471388	0.498413605
YLDs (Year	South Afri	Male	55+ years	Non-melanoma skin cancer (basal-cell	Percent	1990	2.20E-06	4.06E-06	1.02E-06
YLDs (Year	South Afri	Female	55+ years	Non-melanoma skin cancer (basal-cell	Percent	1990	1.26E-06	2.34E-06	5.68E-07
YLDs (Year	South Afri	Both	55+ years	Non-melanoma skin cancer (basal-cell	Percent	1990	1.63E-06	3.04E-06	7.44E-07
YLDs (Year	South Afri	Male	55+ years	Non-melanoma skin cancer (basal-cell	Rate	1990	0.0456091	0.087261609	0.018542725
YLDs (Year	South Afri	Female	55+ years	Non-melanoma skin cancer (basal-cell	Rate	1990	0.0292251	0.057566994	0.012032476
YLDs (Year	South Afri	Both	55+ years	Non-melanoma skin cancer (basal-cell	Rate	1990	0.0360876	0.069663452	0.014765739
YLDs (Year	South Afri	Male	55+ years	Non-melanoma skin cancer (basal-cell	Number	2021	1.7721665	3.397028389	0.731483725
YLDs (Year	South Afri	Female	55+ years	Non-melanoma skin cancer (basal-cell	Number	2021	1.6580705	3.189502868	0.694892433
YLDs (Year	South Afri	Both	55+ years	Non-melanoma skin cancer (basal-cell	Number	2021	3.4302369	6.594090434	1.425698963
YLDs (Year	South Afri	Male	55+ years	Non-melanoma skin cancer (basal-cell	Percent	2021	2.41E-06	4.43E-06	1.09E-06
YLDs (Year	South Afri	Female	55+ years	Non-melanoma skin cancer (basal-cell	Percent	2021	1.41E-06	2.65E-06	6.37E-07
YLDs (Year	South Afri	Both	55+ years	Non-melanoma skin cancer (basal-cell	Percent	2021	1.80E-06	3.33E-06	8.12E-07
YLDs (Year	South Afri	Male	55+ years	Non-melanoma skin cancer (basal-cell	Rate	2021	0.0538445	0.103213405	0.022224991
YLDs (Year	South Afri	Female	55+ years	Non-melanoma skin cancer (basal-cell	Rate	2021	0.0360663	0.069377954	0.015115276
YLDs (Year	South Afri	Both	55+ years	Non-melanoma skin cancer (basal-cell	Rate	2021	0.0434837	0.083590626	0.018073011
YLDs (Year	Democratic	Male	55+ years	Non-melanoma skin cancer (basal-cell	Number	1990	0.1454282	0.292857678	0.060427943
YLDs (Year	Democratic	Female	55+ years	Non-melanoma skin cancer (basal-cell	Number	1990	0.097228	0.192828001	0.038462372
YLDs (Year	Democratic	Both	55+ years	Non-melanoma skin cancer (basal-cell	Number	1990	0.2426562	0.488415044	0.099772134

YLDs (Year	Democratic	Male	55+ years	Non-melanoma skin cancer (basal-cell	Percent	1990	5.30E-07	9.93E-07	2.38E-07
YLDs (Year	Democratic	Female	55+ years	Non-melanoma skin cancer (basal-cell	Percent	1990	2.91E-07	5.45E-07	1.28E-07
YLDs (Year	Democratic	Both	55+ years	Non-melanoma skin cancer (basal-cell	Percent	1990	3.98E-07	7.48E-07	1.79E-07
YLDs (Year	Democratic	Male	55+ years	Non-melanoma skin cancer (basal-cell	Rate	1990	0.01198	0.024124955	0.004977918
YLDs (Year	Democratic	Female	55+ years	Non-melanoma skin cancer (basal-cell	Rate	1990	0.0068143	0.013514513	0.002695668
YLDs (Year	Democratic	Both	55+ years	Non-melanoma skin cancer (basal-cell	Rate	1990	0.0091889	0.018495373	0.003778186
YLDs (Year	Democratic	Male	55+ years	Non-melanoma skin cancer (basal-cell	Number	2021	0.3128452	0.613264256	0.131994697
YLDs (Year	Democratic	Female	55+ years	Non-melanoma skin cancer (basal-cell	Number	2021	0.2336558	0.454339155	0.097454029
YLDs (Year	Democratic	Both	55+ years	Non-melanoma skin cancer (basal-cell	Number	2021	0.546501	1.070163544	0.224995729
YLDs (Year	Democratic	Male	55+ years	Non-melanoma skin cancer (basal-cell	Percent	2021	5.36E-07	9.90E-07	2.31E-07
YLDs (Year	Democratic	Female	55+ years	Non-melanoma skin cancer (basal-cell	Percent	2021	3.05E-07	5.65E-07	1.35E-07
YLDs (Year	Democratic	Both	55+ years	Non-melanoma skin cancer (basal-cell	Percent	2021	4.05E-07	7.43E-07	1.78E-07
YLDs (Year	Democratic	Male	55+ years	Non-melanoma skin cancer (basal-cell	Rate	2021	0.0113249	0.022200023	0.004778177
YLDs (Year	Democratic	Female	55+ years	Non-melanoma skin cancer (basal-cell	Rate	2021	0.0071294	0.013862937	0.002973547
YLDs (Year	Democratic	Both	55+ years	Non-melanoma skin cancer (basal-cell	Rate	2021	0.0090483	0.017718483	0.003725209
YLDs (Year	Gabon	Male	55+ years	Non-melanoma skin cancer (basal-cell	Number	1990	0.0055969	0.010542445	0.002346581
YLDs (Year	Gabon	Female	55+ years	Non-melanoma skin cancer (basal-cell	Number	1990	0.0043	0.008577209	0.001807941
YLDs (Year	Gabon	Both	55+ years	Non-melanoma skin cancer (basal-cell	Number	1990	0.0098969	0.019040544	0.0041971
YLDs (Year	Gabon	Male	55+ years	Non-melanoma skin cancer (basal-cell	Percent	1990	6.03E-07	1.12E-06	2.76E-07
YLDs (Year	Gabon	Female	55+ years	Non-melanoma skin cancer (basal-cell	Percent	1990	3.43E-07	6.33E-07	1.57E-07
YLDs (Year	Gabon	Both	55+ years	Non-melanoma skin cancer (basal-cell	Percent	1990	4.53E-07	8.29E-07	2.06E-07
YLDs (Year	Gabon	Male	55+ years	Non-melanoma skin cancer (basal-cell	Rate	1990	0.012823	0.024153432	0.005376169
YLDs (Year	Gabon	Female	55+ years	Non-melanoma skin cancer (basal-cell	Rate	1990	0.0079257	0.015809446	0.003332384
YLDs (Year	Gabon	Both	55+ years	Non-melanoma skin cancer (basal-cell	Rate	1990	0.0101091	0.019448672	0.004287064
YLDs (Year	Gabon	Male	55+ years	Non-melanoma skin cancer (basal-cell	Number	2021	0.0101295	0.019797694	0.004269093
YLDs (Year	Gabon	Female	55+ years	Non-melanoma skin cancer (basal-cell	Number	2021	0.0070324	0.013663784	0.002882838
YLDs (Year	Gabon	Both	55+ years	Non-melanoma skin cancer (basal-cell	Number	2021	0.0171619	0.033068937	0.00719852
YLDs (Year	Gabon	Male	55+ years	Non-melanoma skin cancer (basal-cell	Percent	2021	5.52E-07	1.02E-06	2.45E-07
YLDs (Year	Gabon	Female	55+ years	Non-melanoma skin cancer (basal-cell	Percent	2021	3.24E-07	6.22E-07	1.44E-07
YLDs (Year	Gabon	Both	55+ years	Non-melanoma skin cancer (basal-cell	Percent	2021	4.28E-07	8.06E-07	1.93E-07
YLDs (Year	Gabon	Male	55+ years	Non-melanoma skin cancer (basal-cell	Rate	2021	0.0116542	0.022777675	0.004911684
YLDs (Year	Gabon	Female	55+ years	Non-melanoma skin cancer (basal-cell	Rate	2021	0.0076577	0.014878643	0.003139154
YLDs (Year	Gabon	Both	55+ years	Non-melanoma skin cancer (basal-cell	Rate	2021	0.009601	0.0184999	0.004027099
YLDs (Year	Central Af	Male	55+ years	Non-melanoma skin cancer (basal-cell	Number	1990	0.00919	0.018013883	0.003771372
YLDs (Year	Central Af	Female	55+ years	Non-melanoma skin cancer (basal-cell	Number	1990	0.0063532	0.01273014	0.002591789
YLDs (Year	Central Af	Both	55+ years	Non-melanoma skin cancer (basal-cell	Number	1990	0.0155432	0.030770244	0.006462238
YLDs (Year	Central Af	Male	55+ years	Non-melanoma skin cancer (basal-cell	Percent	1990	4.53E-07	8.42E-07	1.96E-07
YLDs (Year	Central Af	Female	55+ years	Non-melanoma skin cancer (basal-cell	Percent	1990	2.64E-07	5.01E-07	1.17E-07
YLDs (Year	Central Af	Both	55+ years	Non-melanoma skin cancer (basal-cell	Percent	1990	3.51E-07	6.60E-07	1.51E-07
YLDs (Year	Central Af	Male	55+ years	Non-melanoma skin cancer (basal-cell	Rate	1990	0.0104218	0.020428289	0.00427685
YLDs (Year	Central Af	Female	55+ years	Non-melanoma skin cancer (basal-cell	Rate	1990	0.0063133	0.012650245	0.002575523
YLDs (Year	Central Af	Both	55+ years	Non-melanoma skin cancer (basal-cell	Rate	1990	0.0082321	0.016296709	0.003422567
YLDs (Year	Central Af	Male	55+ years	Non-melanoma skin cancer (basal-cell	Number	2021	0.0161108	0.032249325	0.006564144
YLDs (Year	Central Af	Female	55+ years	Non-melanoma skin cancer (basal-cell	Number	2021	0.0122268	0.024445567	0.005051521
YLDs (Year	Central Af	Both	55+ years	Non-melanoma skin cancer (basal-cell	Number	2021	0.0283376	0.056451376	0.011560021
YLDs (Year	Central Af	Male	55+ years	Non-melanoma skin cancer (basal-cell	Percent	2021	4.51E-07	8.71E-07	2.00E-07
YLDs (Year	Central Af	Female	55+ years	Non-melanoma skin cancer (basal-cell	Percent	2021	2.63E-07	4.99E-07	1.13E-07
YLDs (Year	Central Af	Both	55+ years	Non-melanoma skin cancer (basal-cell	Percent	2021	3.44E-07	6.60E-07	1.51E-07
YLDs (Year	Central Af	Male	55+ years	Non-melanoma skin cancer (basal-cell	Rate	2021	0.0099425	0.019902014	0.004050928
YLDs (Year	Central Af	Female	55+ years	Non-melanoma skin cancer (basal-cell	Rate	2021	0.0062491	0.012494026	0.002581811
YLDs (Year	Central Af	Both	55+ years	Non-melanoma skin cancer (basal-cell	Rate	2021	0.0079222	0.015781829	0.003231777
YLDs (Year	Congo	Male	55+ years	Non-melanoma skin cancer (basal-cell	Number	1990	0.0092112	0.018092577	0.003973452
YLDs (Year	Congo	Female	55+ years	Non-melanoma skin cancer (basal-cell	Number	1990	0.00683	0.013226454	0.002882229
YLDs (Year	Congo	Both	55+ years	Non-melanoma skin cancer (basal-cell	Number	1990	0.0160412	0.031121946	0.006885174
YLDs (Year	Congo	Male	55+ years	Non-melanoma skin cancer (basal-cell	Percent	1990	5.74E-07	1.07E-06	2.59E-07
YLDs (Year	Congo	Female	55+ years	Non-melanoma skin cancer (basal-cell	Percent	1990	3.00E-07	5.63E-07	1.36E-07

YLDs (Year	Congo	Both	55+ years	Non-melanoma skin cancer (basal-cell	Percent	1990	4.13E-07	7.59E-07	1.87E-07
YLDs (Year	Congo	Male	55+ years	Non-melanoma skin cancer (basal-cell	Rate	1990	0.0119288	0.023430457	0.005145746
YLDs (Year	Congo	Female	55+ years	Non-melanoma skin cancer (basal-cell	Rate	1990	0.0067761	0.013122202	0.002859511
YLDs (Year	Congo	Both	55+ years	Non-melanoma skin cancer (basal-cell	Rate	1990	0.0090112	0.017482995	0.0038678
YLDs (Year	Congo	Male	55+ years	Non-melanoma skin cancer (basal-cell	Number	2021	0.0241448	0.047383399	0.009922177
YLDs (Year	Congo	Female	55+ years	Non-melanoma skin cancer (basal-cell	Number	2021	0.0148687	0.029853462	0.006094319
YLDs (Year	Congo	Both	55+ years	Non-melanoma skin cancer (basal-cell	Number	2021	0.0390135	0.076362534	0.01603926
YLDs (Year	Congo	Male	55+ years	Non-melanoma skin cancer (basal-cell	Percent	2021	5.62E-07	1.04E-06	2.45E-07
YLDs (Year	Congo	Female	55+ years	Non-melanoma skin cancer (basal-cell	Percent	2021	2.86E-07	5.29E-07	1.24E-07
YLDs (Year	Congo	Both	55+ years	Non-melanoma skin cancer (basal-cell	Percent	2021	4.11E-07	7.59E-07	1.81E-07
YLDs (Year	Congo	Male	55+ years	Non-melanoma skin cancer (basal-cell	Rate	2021	0.0113635	0.022300516	0.004669772
YLDs (Year	Congo	Female	55+ years	Non-melanoma skin cancer (basal-cell	Rate	2021	0.006696	0.013444371	0.002744549
YLDs (Year	Congo	Both	55+ years	Non-melanoma skin cancer (basal-cell	Rate	2021	0.0089783	0.017573656	0.003691188
YLDs (Year	Madagascar	Male	55+ years	Non-melanoma skin cancer (basal-cell	Number	1990	0.045274	0.089108589	0.019001239
YLDs (Year	Madagascar	Female	55+ years	Non-melanoma skin cancer (basal-cell	Number	1990	0.0260624	0.052054706	0.010490645
YLDs (Year	Madagascar	Both	55+ years	Non-melanoma skin cancer (basal-cell	Number	1990	0.0713364	0.142551342	0.029623893
YLDs (Year	Madagascar	Male	55+ years	Non-melanoma skin cancer (basal-cell	Percent	1990	5.25E-07	9.57E-07	2.32E-07
YLDs (Year	Madagascar	Female	55+ years	Non-melanoma skin cancer (basal-cell	Percent	1990	2.84E-07	5.39E-07	1.25E-07
YLDs (Year	Madagascar	Both	55+ years	Non-melanoma skin cancer (basal-cell	Percent	1990	4.01E-07	7.37E-07	1.73E-07
YLDs (Year	Madagascar	Male	55+ years	Non-melanoma skin cancer (basal-cell	Rate	1990	0.0105431	0.020750933	0.004424865
YLDs (Year	Madagascar	Female	55+ years	Non-melanoma skin cancer (basal-cell	Rate	1990	0.0062727	0.012528541	0.002524891
YLDs (Year	Madagascar	Both	55+ years	Non-melanoma skin cancer (basal-cell	Rate	1990	0.0084431	0.016871805	0.003506165
YLDs (Year	Madagascar	Male	55+ years	Non-melanoma skin cancer (basal-cell	Number	2021	0.086095	0.169454843	0.036011429
YLDs (Year	Madagascar	Female	55+ years	Non-melanoma skin cancer (basal-cell	Number	2021	0.057245	0.11727699	0.0231656
YLDs (Year	Madagascar	Both	55+ years	Non-melanoma skin cancer (basal-cell	Number	2021	0.14334	0.285512143	0.060055642
YLDs (Year	Madagascar	Male	55+ years	Non-melanoma skin cancer (basal-cell	Percent	2021	5.17E-07	9.43E-07	2.32E-07
YLDs (Year	Madagascar	Female	55+ years	Non-melanoma skin cancer (basal-cell	Percent	2021	2.80E-07	5.37E-07	1.23E-07
YLDs (Year	Madagascar	Both	55+ years	Non-melanoma skin cancer (basal-cell	Percent	2021	3.86E-07	7.21E-07	1.73E-07
YLDs (Year	Madagascar	Male	55+ years	Non-melanoma skin cancer (basal-cell	Rate	2021	0.0095751	0.01884601	0.00400503
YLDs (Year	Madagascar	Female	55+ years	Non-melanoma skin cancer (basal-cell	Rate	2021	0.0060415	0.012377187	0.002444853
YLDs (Year	Madagascar	Both	55+ years	Non-melanoma skin cancer (basal-cell	Rate	2021	0.007762	0.015460831	0.003252086
YLDs (Year	Cameroon	Male	55+ years	Non-melanoma skin cancer (basal-cell	Number	1990	0.0283445	0.055009951	0.011440111
YLDs (Year	Cameroon	Female	55+ years	Non-melanoma skin cancer (basal-cell	Number	1990	0.0177286	0.034403564	0.007148383
YLDs (Year	Cameroon	Both	55+ years	Non-melanoma skin cancer (basal-cell	Number	1990	0.0460731	0.090245378	0.018355667
YLDs (Year	Cameroon	Male	55+ years	Non-melanoma skin cancer (basal-cell	Percent	1990	3.90E-07	7.20E-07	1.75E-07
YLDs (Year	Cameroon	Female	55+ years	Non-melanoma skin cancer (basal-cell	Percent	1990	2.04E-07	3.89E-07	8.92E-08
YLDs (Year	Cameroon	Both	55+ years	Non-melanoma skin cancer (basal-cell	Percent	1990	2.88E-07	5.47E-07	1.30E-07
YLDs (Year	Cameroon	Male	55+ years	Non-melanoma skin cancer (basal-cell	Rate	1990	0.0077624	0.015064968	0.003132977
YLDs (Year	Cameroon	Female	55+ years	Non-melanoma skin cancer (basal-cell	Rate	1990	0.0047239	0.00916713	0.001904749
YLDs (Year	Cameroon	Both	55+ years	Non-melanoma skin cancer (basal-cell	Rate	1990	0.0062224	0.012188007	0.002479008
YLDs (Year	Cameroon	Male	55+ years	Non-melanoma skin cancer (basal-cell	Number	2021	0.0756875	0.150121288	0.031019723
YLDs (Year	Cameroon	Female	55+ years	Non-melanoma skin cancer (basal-cell	Number	2021	0.0493167	0.09835608	0.019953294
YLDs (Year	Cameroon	Both	55+ years	Non-melanoma skin cancer (basal-cell	Number	2021	0.1250041	0.245801132	0.05048723
YLDs (Year	Cameroon	Male	55+ years	Non-melanoma skin cancer (basal-cell	Percent	2021	3.95E-07	7.46E-07	1.77E-07
YLDs (Year	Cameroon	Female	55+ years	Non-melanoma skin cancer (basal-cell	Percent	2021	2.06E-07	3.89E-07	9.22E-08
YLDs (Year	Cameroon	Both	55+ years	Non-melanoma skin cancer (basal-cell	Percent	2021	2.90E-07	5.42E-07	1.28E-07
YLDs (Year	Cameroon	Male	55+ years	Non-melanoma skin cancer (basal-cell	Rate	2021	0.0076617	0.01519646	0.003140061
YLDs (Year	Cameroon	Female	55+ years	Non-melanoma skin cancer (basal-cell	Rate	2021	0.0047076	0.009388635	0.001904653
YLDs (Year	Cameroon	Both	55+ years	Non-melanoma skin cancer (basal-cell	Rate	2021	0.0061413	0.012075843	0.002480362
YLDs (Year	Zambia	Male	55+ years	Non-melanoma skin cancer (basal-cell	Number	1990	0.0238126	0.047143152	0.009802788
YLDs (Year	Zambia	Female	55+ years	Non-melanoma skin cancer (basal-cell	Number	1990	0.0129507	0.026682908	0.005281489
YLDs (Year	Zambia	Both	55+ years	Non-melanoma skin cancer (basal-cell	Number	1990	0.0367632	0.074529226	0.015168663
YLDs (Year	Zambia	Male	55+ years	Non-melanoma skin cancer (basal-cell	Percent	1990	4.84E-07	8.97E-07	2.18E-07
YLDs (Year	Zambia	Female	55+ years	Non-melanoma skin cancer (basal-cell	Percent	1990	2.71E-07	5.13E-07	1.21E-07
YLDs (Year	Zambia	Both	55+ years	Non-melanoma skin cancer (basal-cell	Percent	1990	3.79E-07	7.08E-07	1.69E-07
YLDs (Year	Zambia	Male	55+ years	Non-melanoma skin cancer (basal-cell	Rate	1990	0.0097407	0.019284243	0.004009901

YLDs (Year	Zambia	Female	55+ years	Non-melanoma skin cancer (basal-cell	Rate	1990	0.0059996	0.012361153	0.002446708
YLDs (Year	Zambia	Both	55+ years	Non-melanoma skin cancer (basal-cell	Rate	1990	0.0079864	0.016190545	0.003295203
YLDs (Year	Zambia	Male	55+ years	Non-melanoma skin cancer (basal-cell	Number	2021	0.0480325	0.093886601	0.019937657
YLDs (Year	Zambia	Female	55+ years	Non-melanoma skin cancer (basal-cell	Number	2021	0.0343655	0.067227237	0.014016091
YLDs (Year	Zambia	Both	55+ years	Non-melanoma skin cancer (basal-cell	Number	2021	0.082398	0.160746736	0.034153463
YLDs (Year	Zambia	Male	55+ years	Non-melanoma skin cancer (basal-cell	Percent	2021	4.54E-07	8.54E-07	2.06E-07
YLDs (Year	Zambia	Female	55+ years	Non-melanoma skin cancer (basal-cell	Percent	2021	2.62E-07	4.74E-07	1.18E-07
YLDs (Year	Zambia	Both	55+ years	Non-melanoma skin cancer (basal-cell	Percent	2021	3.47E-07	6.45E-07	1.52E-07
YLDs (Year	Zambia	Male	55+ years	Non-melanoma skin cancer (basal-cell	Rate	2021	0.0093367	0.018249896	0.003875528
YLDs (Year	Zambia	Female	55+ years	Non-melanoma skin cancer (basal-cell	Rate	2021	0.0060748	0.011883727	0.002477618
YLDs (Year	Zambia	Both	55+ years	Non-melanoma skin cancer (basal-cell	Rate	2021	0.0076283	0.014881774	0.003161894
YLDs (Year	Equatorial	Male	55+ years	Non-melanoma skin cancer (basal-cell	Number	1990	0.0017227	0.003404208	0.000719659
YLDs (Year	Equatorial	Female	55+ years	Non-melanoma skin cancer (basal-cell	Number	1990	0.0012653	0.00247225	0.000511762
YLDs (Year	Equatorial	Both	55+ years	Non-melanoma skin cancer (basal-cell	Number	1990	0.0029881	0.005900908	0.001208471
YLDs (Year	Equatorial	Male	55+ years	Non-melanoma skin cancer (basal-cell	Percent	1990	5.21E-07	9.77E-07	2.33E-07
YLDs (Year	Equatorial	Female	55+ years	Non-melanoma skin cancer (basal-cell	Percent	1990	2.99E-07	5.62E-07	1.33E-07
YLDs (Year	Equatorial	Both	55+ years	Non-melanoma skin cancer (basal-cell	Percent	1990	3.96E-07	7.50E-07	1.76E-07
YLDs (Year	Equatorial	Male	55+ years	Non-melanoma skin cancer (basal-cell	Rate	1990	0.0119221	0.023558831	0.004980398
YLDs (Year	Equatorial	Female	55+ years	Non-melanoma skin cancer (basal-cell	Rate	1990	0.0072424	0.014150518	0.002929195
YLDs (Year	Equatorial	Both	55+ years	Non-melanoma skin cancer (basal-cell	Rate	1990	0.0093608	0.018486024	0.003785828
YLDs (Year	Equatorial	Male	55+ years	Non-melanoma skin cancer (basal-cell	Number	2021	0.0040732	0.007985218	0.001674432
YLDs (Year	Equatorial	Female	55+ years	Non-melanoma skin cancer (basal-cell	Number	2021	0.0033541	0.006753031	0.001396744
YLDs (Year	Equatorial	Both	55+ years	Non-melanoma skin cancer (basal-cell	Number	2021	0.0074273	0.014541702	0.003049799
YLDs (Year	Equatorial	Male	55+ years	Non-melanoma skin cancer (basal-cell	Percent	2021	5.65E-07	1.06E-06	2.46E-07
YLDs (Year	Equatorial	Female	55+ years	Non-melanoma skin cancer (basal-cell	Percent	2021	3.09E-07	5.65E-07	1.39E-07
YLDs (Year	Equatorial	Both	55+ years	Non-melanoma skin cancer (basal-cell	Percent	2021	4.11E-07	7.59E-07	1.84E-07
YLDs (Year	Equatorial	Male	55+ years	Non-melanoma skin cancer (basal-cell	Rate	2021	0.0122052	0.023927477	0.005017387
YLDs (Year	Equatorial	Female	55+ years	Non-melanoma skin cancer (basal-cell	Rate	2021	0.0073356	0.014769001	0.003054705
YLDs (Year	Equatorial	Both	55+ years	Non-melanoma skin cancer (basal-cell	Rate	2021	0.0093902	0.018384657	0.003855773
YLDs (Year	Ethiopia	Male	55+ years	Non-melanoma skin cancer (basal-cell	Number	1990	0.1717813	0.343883283	0.072736795
YLDs (Year	Ethiopia	Female	55+ years	Non-melanoma skin cancer (basal-cell	Number	1990	0.0925692	0.184364275	0.037456156
YLDs (Year	Ethiopia	Both	55+ years	Non-melanoma skin cancer (basal-cell	Number	1990	0.2643505	0.528750289	0.111218631
YLDs (Year	Ethiopia	Male	55+ years	Non-melanoma skin cancer (basal-cell	Percent	1990	4.60E-07	8.60E-07	2.05E-07
YLDs (Year	Ethiopia	Female	55+ years	Non-melanoma skin cancer (basal-cell	Percent	1990	2.59E-07	4.79E-07	1.12E-07
YLDs (Year	Ethiopia	Both	55+ years	Non-melanoma skin cancer (basal-cell	Percent	1990	3.62E-07	6.65E-07	1.59E-07
YLDs (Year	Ethiopia	Male	55+ years	Non-melanoma skin cancer (basal-cell	Rate	1990	0.0100492	0.020117135	0.004255095
YLDs (Year	Ethiopia	Female	55+ years	Non-melanoma skin cancer (basal-cell	Rate	1990	0.0061566	0.012261713	0.002491137
YLDs (Year	Ethiopia	Both	55+ years	Non-melanoma skin cancer (basal-cell	Rate	1990	0.0082276	0.016456686	0.00346154
YLDs (Year	Ethiopia	Male	55+ years	Non-melanoma skin cancer (basal-cell	Number	2021	0.3696902	0.718994575	0.147807141
YLDs (Year	Ethiopia	Female	55+ years	Non-melanoma skin cancer (basal-cell	Number	2021	0.2172233	0.423978478	0.089500911
YLDs (Year	Ethiopia	Both	55+ years	Non-melanoma skin cancer (basal-cell	Number	2021	0.5869134	1.146919945	0.237484209
YLDs (Year	Ethiopia	Male	55+ years	Non-melanoma skin cancer (basal-cell	Percent	2021	5.03E-07	9.33E-07	2.24E-07
YLDs (Year	Ethiopia	Female	55+ years	Non-melanoma skin cancer (basal-cell	Percent	2021	2.77E-07	5.19E-07	1.19E-07
YLDs (Year	Ethiopia	Both	55+ years	Non-melanoma skin cancer (basal-cell	Percent	2021	3.86E-07	7.23E-07	1.67E-07
YLDs (Year	Ethiopia	Male	55+ years	Non-melanoma skin cancer (basal-cell	Rate	2021	0.0106174	0.020649344	0.004244984
YLDs (Year	Ethiopia	Female	55+ years	Non-melanoma skin cancer (basal-cell	Rate	2021	0.006458	0.012604705	0.002660825
YLDs (Year	Ethiopia	Both	55+ years	Non-melanoma skin cancer (basal-cell	Rate	2021	0.0085736	0.016754175	0.003469163
YLDs (Year	Botswana	Male	55+ years	Non-melanoma skin cancer (basal-cell	Number	1990	0.0149606	0.028591378	0.006437901
YLDs (Year	Botswana	Female	55+ years	Non-melanoma skin cancer (basal-cell	Number	1990	0.0117657	0.022927948	0.005109055
YLDs (Year	Botswana	Both	55+ years	Non-melanoma skin cancer (basal-cell	Number	1990	0.0267263	0.050814161	0.011590698
YLDs (Year	Botswana	Male	55+ years	Non-melanoma skin cancer (basal-cell	Percent	1990	1.81E-06	3.32E-06	8.24E-07
YLDs (Year	Botswana	Female	55+ years	Non-melanoma skin cancer (basal-cell	Percent	1990	1.09E-06	2.02E-06	4.84E-07
YLDs (Year	Botswana	Both	55+ years	Non-melanoma skin cancer (basal-cell	Percent	1990	1.40E-06	2.56E-06	6.33E-07
YLDs (Year	Botswana	Male	55+ years	Non-melanoma skin cancer (basal-cell	Rate	1990	0.035979	0.068759983	0.015482637
YLDs (Year	Botswana	Female	55+ years	Non-melanoma skin cancer (basal-cell	Rate	1990	0.0228957	0.044617135	0.009942075
YLDs (Year	Botswana	Both	55+ years	Non-melanoma skin cancer (basal-cell	Rate	1990	0.0287473	0.05465673	0.012467187

YLDs (Year	Botswana	Male	55+ years	Non-melanoma skin cancer (basal-cell	Number	2021	0.0367482	0.070682206	0.01515523
YLDs (Year	Botswana	Female	55+ years	Non-melanoma skin cancer (basal-cell	Number	2021	0.0314348	0.060706939	0.01342631
YLDs (Year	Botswana	Both	55+ years	Non-melanoma skin cancer (basal-cell	Number	2021	0.0681829	0.132045094	0.028879119
YLDs (Year	Botswana	Male	55+ years	Non-melanoma skin cancer (basal-cell	Percent	2021	1.60E-06	2.90E-06	6.99E-07
YLDs (Year	Botswana	Female	55+ years	Non-melanoma skin cancer (basal-cell	Percent	2021	9.54E-07	1.75E-06	4.36E-07
YLDs (Year	Botswana	Both	55+ years	Non-melanoma skin cancer (basal-cell	Percent	2021	1.22E-06	2.20E-06	5.47E-07
YLDs (Year	Botswana	Male	55+ years	Non-melanoma skin cancer (basal-cell	Rate	2021	0.0352258	0.067754126	0.01452741
YLDs (Year	Botswana	Female	55+ years	Non-melanoma skin cancer (basal-cell	Rate	2021	0.022762	0.043958105	0.009722037
YLDs (Year	Botswana	Both	55+ years	Non-melanoma skin cancer (basal-cell	Rate	2021	0.0281255	0.054468782	0.011912676
YLDs (Year	Malawi	Male	55+ years	Non-melanoma skin cancer (basal-cell	Number	1990	0.0262703	0.050828914	0.010546961
YLDs (Year	Malawi	Female	55+ years	Non-melanoma skin cancer (basal-cell	Number	1990	0.0131806	0.025981222	0.005340737
YLDs (Year	Malawi	Both	55+ years	Non-melanoma skin cancer (basal-cell	Number	1990	0.0394509	0.077576572	0.015910203
YLDs (Year	Malawi	Male	55+ years	Non-melanoma skin cancer (basal-cell	Percent	1990	4.52E-07	8.28E-07	2.01E-07
YLDs (Year	Malawi	Female	55+ years	Non-melanoma skin cancer (basal-cell	Percent	1990	1.73E-07	3.24E-07	7.47E-08
YLDs (Year	Malawi	Both	55+ years	Non-melanoma skin cancer (basal-cell	Percent	1990	2.93E-07	5.36E-07	1.29E-07
YLDs (Year	Malawi	Male	55+ years	Non-melanoma skin cancer (basal-cell	Rate	1990	0.0089408	0.017298972	0.003589523
YLDs (Year	Malawi	Female	55+ years	Non-melanoma skin cancer (basal-cell	Rate	1990	0.0039327	0.007752052	0.001593523
YLDs (Year	Malawi	Both	55+ years	Non-melanoma skin cancer (basal-cell	Rate	1990	0.0062722	0.012333729	0.002529528
YLDs (Year	Malawi	Male	55+ years	Non-melanoma skin cancer (basal-cell	Number	2021	0.047018	0.093866531	0.019754398
YLDs (Year	Malawi	Female	55+ years	Non-melanoma skin cancer (basal-cell	Number	2021	0.0266211	0.053290803	0.010544017
YLDs (Year	Malawi	Both	55+ years	Non-melanoma skin cancer (basal-cell	Number	2021	0.073639	0.148082371	0.030308565
YLDs (Year	Malawi	Male	55+ years	Non-melanoma skin cancer (basal-cell	Percent	2021	4.74E-07	8.75E-07	2.07E-07
YLDs (Year	Malawi	Female	55+ years	Non-melanoma skin cancer (basal-cell	Percent	2021	1.80E-07	3.40E-07	7.96E-08
YLDs (Year	Malawi	Both	55+ years	Non-melanoma skin cancer (basal-cell	Percent	2021	2.98E-07	5.56E-07	1.31E-07
YLDs (Year	Malawi	Male	55+ years	Non-melanoma skin cancer (basal-cell	Rate	2021	0.0090694	0.018106051	0.003810454
YLDs (Year	Malawi	Female	55+ years	Non-melanoma skin cancer (basal-cell	Rate	2021	0.0040911	0.008189745	0.001620407
YLDs (Year	Malawi	Both	55+ years	Non-melanoma skin cancer (basal-cell	Rate	2021	0.0062986	0.012666054	0.002592408
YLDs (Year	Lesotho	Male	55+ years	Non-melanoma skin cancer (basal-cell	Number	1990	0.0182818	0.034985494	0.007698683
YLDs (Year	Lesotho	Female	55+ years	Non-melanoma skin cancer (basal-cell	Number	1990	0.0206735	0.040172505	0.008761458
YLDs (Year	Lesotho	Both	55+ years	Non-melanoma skin cancer (basal-cell	Number	1990	0.0389552	0.075066541	0.016365113
YLDs (Year	Lesotho	Male	55+ years	Non-melanoma skin cancer (basal-cell	Percent	1990	1.87E-06	3.39E-06	8.74E-07
YLDs (Year	Lesotho	Female	55+ years	Non-melanoma skin cancer (basal-cell	Percent	1990	1.06E-06	1.94E-06	4.76E-07
YLDs (Year	Lesotho	Both	55+ years	Non-melanoma skin cancer (basal-cell	Percent	1990	1.33E-06	2.47E-06	6.06E-07
YLDs (Year	Lesotho	Male	55+ years	Non-melanoma skin cancer (basal-cell	Rate	1990	0.0361403	0.069161043	0.015219134
YLDs (Year	Lesotho	Female	55+ years	Non-melanoma skin cancer (basal-cell	Rate	1990	0.0231626	0.045009229	0.009816328
YLDs (Year	Lesotho	Both	55+ years	Non-melanoma skin cancer (basal-cell	Rate	1990	0.0278571	0.053680507	0.011702785
YLDs (Year	Lesotho	Male	55+ years	Non-melanoma skin cancer (basal-cell	Number	2021	0.0233268	0.04701749	0.009882351
YLDs (Year	Lesotho	Female	55+ years	Non-melanoma skin cancer (basal-cell	Number	2021	0.0252591	0.047851819	0.010669283
YLDs (Year	Lesotho	Both	55+ years	Non-melanoma skin cancer (basal-cell	Number	2021	0.0485858	0.094379657	0.020715066
YLDs (Year	Lesotho	Male	55+ years	Non-melanoma skin cancer (basal-cell	Percent	2021	1.51E-06	2.82E-06	6.89E-07
YLDs (Year	Lesotho	Female	55+ years	Non-melanoma skin cancer (basal-cell	Percent	2021	8.83E-07	1.65E-06	4.05E-07
YLDs (Year	Lesotho	Both	55+ years	Non-melanoma skin cancer (basal-cell	Percent	2021	1.10E-06	2.05E-06	5.06E-07
YLDs (Year	Lesotho	Male	55+ years	Non-melanoma skin cancer (basal-cell	Rate	2021	0.0344751	0.069488124	0.014605332
YLDs (Year	Lesotho	Female	55+ years	Non-melanoma skin cancer (basal-cell	Rate	2021	0.0223746	0.042387319	0.00945089
YLDs (Year	Lesotho	Both	55+ years	Non-melanoma skin cancer (basal-cell	Rate	2021	0.0269092	0.052272129	0.011473029
YLDs (Year	Burundi	Male	55+ years	Non-melanoma skin cancer (basal-cell	Number	1990	0.0174395	0.033872678	0.007386459
YLDs (Year	Burundi	Female	55+ years	Non-melanoma skin cancer (basal-cell	Number	1990	0.0128217	0.025954143	0.005207016
YLDs (Year	Burundi	Both	55+ years	Non-melanoma skin cancer (basal-cell	Number	1990	0.0302612	0.059754469	0.012800874
YLDs (Year	Burundi	Male	55+ years	Non-melanoma skin cancer (basal-cell	Percent	1990	5.03E-07	9.30E-07	2.28E-07
YLDs (Year	Burundi	Female	55+ years	Non-melanoma skin cancer (basal-cell	Percent	1990	2.74E-07	5.24E-07	1.19E-07
YLDs (Year	Burundi	Both	55+ years	Non-melanoma skin cancer (basal-cell	Percent	1990	3.71E-07	6.86E-07	1.65E-07
YLDs (Year	Burundi	Male	55+ years	Non-melanoma skin cancer (basal-cell	Rate	1990	0.0102446	0.019898001	0.004339066
YLDs (Year	Burundi	Female	55+ years	Non-melanoma skin cancer (basal-cell	Rate	1990	0.0062206	0.012591997	0.002526253
YLDs (Year	Burundi	Both	55+ years	Non-melanoma skin cancer (basal-cell	Rate	1990	0.0080408	0.015877462	0.003401342
YLDs (Year	Burundi	Male	55+ years	Non-melanoma skin cancer (basal-cell	Number	2021	0.0396017	0.081707977	0.016193033
YLDs (Year	Burundi	Female	55+ years	Non-melanoma skin cancer (basal-cell	Number	2021	0.0226401	0.045032285	0.009137997

YLDs (Year	Burundi	Both	55+ years	Non-melanoma skin cancer (basal-cell	Number	2021	0.0622418	0.126028636	0.025378017
YLDs (Year	Burundi	Male	55+ years	Non-melanoma skin cancer (basal-cell	Percent	2021	5.04E-07	9.19E-07	2.22E-07
YLDs (Year	Burundi	Female	55+ years	Non-melanoma skin cancer (basal-cell	Percent	2021	2.82E-07	5.34E-07	1.21E-07
YLDs (Year	Burundi	Both	55+ years	Non-melanoma skin cancer (basal-cell	Percent	2021	3.91E-07	7.21E-07	1.69E-07
YLDs (Year	Burundi	Male	55+ years	Non-melanoma skin cancer (basal-cell	Rate	2021	0.0093675	0.01932752	0.003830362
YLDs (Year	Burundi	Female	55+ years	Non-melanoma skin cancer (basal-cell	Rate	2021	0.005993	0.01192044	0.002418908
YLDs (Year	Burundi	Both	55+ years	Non-melanoma skin cancer (basal-cell	Rate	2021	0.0077751	0.015743184	0.003170159
YLDs (Year	Comoros	Male	55+ years	Non-melanoma skin cancer (basal-cell	Number	1990	0.0016876	0.003221172	0.000711408
YLDs (Year	Comoros	Female	55+ years	Non-melanoma skin cancer (basal-cell	Number	1990	0.0010565	0.002067566	0.000435667
YLDs (Year	Comoros	Both	55+ years	Non-melanoma skin cancer (basal-cell	Number	1990	0.0027441	0.005334203	0.001155437
YLDs (Year	Comoros	Male	55+ years	Non-melanoma skin cancer (basal-cell	Percent	1990	5.39E-07	1.00E-06	2.39E-07
YLDs (Year	Comoros	Female	55+ years	Non-melanoma skin cancer (basal-cell	Percent	1990	2.96E-07	5.61E-07	1.34E-07
YLDs (Year	Comoros	Both	55+ years	Non-melanoma skin cancer (basal-cell	Percent	1990	4.10E-07	7.64E-07	1.78E-07
YLDs (Year	Comoros	Male	55+ years	Non-melanoma skin cancer (basal-cell	Rate	1990	0.0105238	0.020087068	0.004436302
YLDs (Year	Comoros	Female	55+ years	Non-melanoma skin cancer (basal-cell	Rate	1990	0.006435	0.012592904	0.00265351
YLDs (Year	Comoros	Both	55+ years	Non-melanoma skin cancer (basal-cell	Rate	1990	0.0084553	0.016435917	0.00356017
YLDs (Year	Comoros	Male	55+ years	Non-melanoma skin cancer (basal-cell	Number	2021	0.003989	0.007692585	0.001602031
YLDs (Year	Comoros	Female	55+ years	Non-melanoma skin cancer (basal-cell	Number	2021	0.0028899	0.005759739	0.001178175
YLDs (Year	Comoros	Both	55+ years	Non-melanoma skin cancer (basal-cell	Number	2021	0.0068789	0.013698162	0.002826269
YLDs (Year	Comoros	Male	55+ years	Non-melanoma skin cancer (basal-cell	Percent	2021	5.49E-07	1.00E-06	2.41E-07
YLDs (Year	Comoros	Female	55+ years	Non-melanoma skin cancer (basal-cell	Percent	2021	2.94E-07	5.57E-07	1.32E-07
YLDs (Year	Comoros	Both	55+ years	Non-melanoma skin cancer (basal-cell	Percent	2021	4.03E-07	7.58E-07	1.81E-07
YLDs (Year	Comoros	Male	55+ years	Non-melanoma skin cancer (basal-cell	Rate	2021	0.0107782	0.020785426	0.004328699
YLDs (Year	Comoros	Female	55+ years	Non-melanoma skin cancer (basal-cell	Rate	2021	0.0065949	0.013143778	0.002688606
YLDs (Year	Comoros	Both	55+ years	Non-melanoma skin cancer (basal-cell	Rate	2021	0.0085103	0.016946765	0.003496536
YLDs (Year	United Rep	Male	55+ years	Non-melanoma skin cancer (basal-cell	Number	1990	0.0928756	0.178984061	0.038860019
YLDs (Year	United Rep	Female	55+ years	Non-melanoma skin cancer (basal-cell	Number	1990	0.0593341	0.117648056	0.024091428
YLDs (Year	United Rep	Both	55+ years	Non-melanoma skin cancer (basal-cell	Number	1990	0.1522097	0.298145941	0.062703098
YLDs (Year	United Rep	Male	55+ years	Non-melanoma skin cancer (basal-cell	Percent	1990	5.24E-07	9.54E-07	2.29E-07
YLDs (Year	United Rep	Female	55+ years	Non-melanoma skin cancer (basal-cell	Percent	1990	2.85E-07	5.34E-07	1.26E-07
YLDs (Year	United Rep	Both	55+ years	Non-melanoma skin cancer (basal-cell	Percent	1990	3.95E-07	7.26E-07	1.73E-07
YLDs (Year	United Rep	Male	55+ years	Non-melanoma skin cancer (basal-cell	Rate	1990	0.0104382	0.020115785	0.004367427
YLDs (Year	United Rep	Female	55+ years	Non-melanoma skin cancer (basal-cell	Rate	1990	0.0064888	0.012866107	0.002634662
YLDs (Year	United Rep	Both	55+ years	Non-melanoma skin cancer (basal-cell	Rate	1990	0.0084365	0.01652536	0.00347545
YLDs (Year	United Rep	Male	55+ years	Non-melanoma skin cancer (basal-cell	Number	2021	0.2057052	0.397305746	0.088291812
YLDs (Year	United Rep	Female	55+ years	Non-melanoma skin cancer (basal-cell	Number	2021	0.1382593	0.270935896	0.056710425
YLDs (Year	United Rep	Both	55+ years	Non-melanoma skin cancer (basal-cell	Number	2021	0.3439645	0.672606418	0.147347107
YLDs (Year	United Rep	Male	55+ years	Non-melanoma skin cancer (basal-cell	Percent	2021	5.37E-07	1.00E-06	2.45E-07
YLDs (Year	United Rep	Female	55+ years	Non-melanoma skin cancer (basal-cell	Percent	2021	2.85E-07	5.46E-07	1.21E-07
YLDs (Year	United Rep	Both	55+ years	Non-melanoma skin cancer (basal-cell	Percent	2021	3.96E-07	7.46E-07	1.74E-07
YLDs (Year	United Rep	Male	55+ years	Non-melanoma skin cancer (basal-cell	Rate	2021	0.010287	0.019868624	0.004415332
YLDs (Year	United Rep	Female	55+ years	Non-melanoma skin cancer (basal-cell	Rate	2021	0.0065139	0.012764839	0.002671848
YLDs (Year	United Rep	Both	55+ years	Non-melanoma skin cancer (basal-cell	Rate	2021	0.0083442	0.01631676	0.003574494
YLDs (Year	Zimbabwe	Male	55+ years	Non-melanoma skin cancer (basal-cell	Number	1990	0.1002469	0.195428119	0.043206347
YLDs (Year	Zimbabwe	Female	55+ years	Non-melanoma skin cancer (basal-cell	Number	1990	0.0692397	0.136921818	0.029255394
YLDs (Year	Zimbabwe	Both	55+ years	Non-melanoma skin cancer (basal-cell	Number	1990	0.1694866	0.33043693	0.072521658
YLDs (Year	Zimbabwe	Male	55+ years	Non-melanoma skin cancer (basal-cell	Percent	1990	1.56E-06	2.88E-06	7.31E-07
YLDs (Year	Zimbabwe	Female	55+ years	Non-melanoma skin cancer (basal-cell	Percent	1990	9.92E-07	1.88E-06	4.60E-07
YLDs (Year	Zimbabwe	Both	55+ years	Non-melanoma skin cancer (basal-cell	Percent	1990	1.27E-06	2.37E-06	5.89E-07
YLDs (Year	Zimbabwe	Male	55+ years	Non-melanoma skin cancer (basal-cell	Rate	1990	0.0303123	0.059092805	0.013064569
YLDs (Year	Zimbabwe	Female	55+ years	Non-melanoma skin cancer (basal-cell	Rate	1990	0.0208481	0.041227143	0.008808796
YLDs (Year	Zimbabwe	Both	55+ years	Non-melanoma skin cancer (basal-cell	Rate	1990	0.0255702	0.04985247	0.010941222
YLDs (Year	Zimbabwe	Male	55+ years	Non-melanoma skin cancer (basal-cell	Number	2021	0.1272804	0.246003685	0.05428911
YLDs (Year	Zimbabwe	Female	55+ years	Non-melanoma skin cancer (basal-cell	Number	2021	0.1179292	0.231884731	0.050033011
YLDs (Year	Zimbabwe	Both	55+ years	Non-melanoma skin cancer (basal-cell	Number	2021	0.2452096	0.478420463	0.100976114
YLDs (Year	Zimbabwe	Male	55+ years	Non-melanoma skin cancer (basal-cell	Percent	2021	1.37E-06	2.57E-06	6.22E-07

YLDs (Year	Zimbabwe	Female	55+ years	Non-melanoma skin cancer (basal-cell	Percent	2021	7.86E-07	1.46E-06	3.46E-07
YLDs (Year	Zimbabwe	Both	55+ years	Non-melanoma skin cancer (basal-cell	Percent	2021	1.01E-06	1.87E-06	4.46E-07
YLDs (Year	Zimbabwe	Male	55+ years	Non-melanoma skin cancer (basal-cell	Rate	2021	0.0283629	0.054818913	0.012097664
YLDs (Year	Zimbabwe	Female	55+ years	Non-melanoma skin cancer (basal-cell	Rate	2021	0.0179084	0.035213312	0.007597861
YLDs (Year	Zimbabwe	Both	55+ years	Non-melanoma skin cancer (basal-cell	Rate	2021	0.0221454	0.043207156	0.009119365
YLDs (Year	Eswatini	Male	55+ years	Non-melanoma skin cancer (basal-cell	Number	1990	0.0070157	0.013767702	0.002936082
YLDs (Year	Eswatini	Female	55+ years	Non-melanoma skin cancer (basal-cell	Number	1990	0.0057134	0.010930616	0.002331438
YLDs (Year	Eswatini	Both	55+ years	Non-melanoma skin cancer (basal-cell	Number	1990	0.0127291	0.024728575	0.005344663
YLDs (Year	Eswatini	Male	55+ years	Non-melanoma skin cancer (basal-cell	Percent	1990	1.83E-06	3.35E-06	8.47E-07
YLDs (Year	Eswatini	Female	55+ years	Non-melanoma skin cancer (basal-cell	Percent	1990	1.07E-06	1.96E-06	4.87E-07
YLDs (Year	Eswatini	Both	55+ years	Non-melanoma skin cancer (basal-cell	Percent	1990	1.39E-06	2.57E-06	6.39E-07
YLDs (Year	Eswatini	Male	55+ years	Non-melanoma skin cancer (basal-cell	Rate	1990	0.0349214	0.068529918	0.014614599
YLDs (Year	Eswatini	Female	55+ years	Non-melanoma skin cancer (basal-cell	Rate	1990	0.0227945	0.043609589	0.009301676
YLDs (Year	Eswatini	Both	55+ years	Non-melanoma skin cancer (basal-cell	Rate	1990	0.0281899	0.05476404	0.01183632
YLDs (Year	Eswatini	Male	55+ years	Non-melanoma skin cancer (basal-cell	Number	2021	0.0120462	0.022891797	0.004888126
YLDs (Year	Eswatini	Female	55+ years	Non-melanoma skin cancer (basal-cell	Number	2021	0.012319	0.023607729	0.00526135
YLDs (Year	Eswatini	Both	55+ years	Non-melanoma skin cancer (basal-cell	Number	2021	0.0243652	0.047734026	0.010393443
YLDs (Year	Eswatini	Male	55+ years	Non-melanoma skin cancer (basal-cell	Percent	2021	1.52E-06	2.76E-06	6.94E-07
YLDs (Year	Eswatini	Female	55+ years	Non-melanoma skin cancer (basal-cell	Percent	2021	9.19E-07	1.69E-06	4.17E-07
YLDs (Year	Eswatini	Both	55+ years	Non-melanoma skin cancer (basal-cell	Percent	2021	1.14E-06	2.07E-06	5.08E-07
YLDs (Year	Eswatini	Male	55+ years	Non-melanoma skin cancer (basal-cell	Rate	2021	0.0341642	0.06492323	0.013863173
YLDs (Year	Eswatini	Female	55+ years	Non-melanoma skin cancer (basal-cell	Rate	2021	0.0222849	0.042706041	0.009517705
YLDs (Year	Eswatini	Both	55+ years	Non-melanoma skin cancer (basal-cell	Rate	2021	0.0269112	0.05272183	0.01147947
YLDs (Year	Namibia	Male	55+ years	Non-melanoma skin cancer (basal-cell	Number	1990	0.0193512	0.037979494	0.008251531
YLDs (Year	Namibia	Female	55+ years	Non-melanoma skin cancer (basal-cell	Number	1990	0.0143986	0.028226735	0.005987494
YLDs (Year	Namibia	Both	55+ years	Non-melanoma skin cancer (basal-cell	Number	1990	0.0337498	0.065660917	0.014238139
YLDs (Year	Namibia	Male	55+ years	Non-melanoma skin cancer (basal-cell	Percent	1990	2.05E-06	3.74E-06	9.24E-07
YLDs (Year	Namibia	Female	55+ years	Non-melanoma skin cancer (basal-cell	Percent	1990	1.16E-06	2.12E-06	5.21E-07
YLDs (Year	Namibia	Both	55+ years	Non-melanoma skin cancer (basal-cell	Percent	1990	1.54E-06	2.80E-06	6.90E-07
YLDs (Year	Namibia	Male	55+ years	Non-melanoma skin cancer (basal-cell	Rate	1990	0.0396868	0.077890979	0.016922812
YLDs (Year	Namibia	Female	55+ years	Non-melanoma skin cancer (basal-cell	Rate	1990	0.0240982	0.04724155	0.010020942
YLDs (Year	Namibia	Both	55+ years	Non-melanoma skin cancer (basal-cell	Rate	1990	0.0311031	0.06051161	0.013121545
YLDs (Year	Namibia	Male	55+ years	Non-melanoma skin cancer (basal-cell	Number	2021	0.0367986	0.070835942	0.01611918
YLDs (Year	Namibia	Female	55+ years	Non-melanoma skin cancer (basal-cell	Number	2021	0.0322653	0.061377229	0.014160817
YLDs (Year	Namibia	Both	55+ years	Non-melanoma skin cancer (basal-cell	Number	2021	0.0690639	0.131179662	0.029629673
YLDs (Year	Namibia	Male	55+ years	Non-melanoma skin cancer (basal-cell	Percent	2021	1.88E-06	3.44E-06	8.79E-07
YLDs (Year	Namibia	Female	55+ years	Non-melanoma skin cancer (basal-cell	Percent	2021	1.08E-06	2.01E-06	4.82E-07
YLDs (Year	Namibia	Both	55+ years	Non-melanoma skin cancer (basal-cell	Percent	2021	1.39E-06	2.60E-06	6.37E-07
YLDs (Year	Namibia	Male	55+ years	Non-melanoma skin cancer (basal-cell	Rate	2021	0.0393169	0.075683546	0.017222284
YLDs (Year	Namibia	Female	55+ years	Non-melanoma skin cancer (basal-cell	Rate	2021	0.0243784	0.046374235	0.01069936
YLDs (Year	Namibia	Both	55+ years	Non-melanoma skin cancer (basal-cell	Rate	2021	0.0305664	0.058057738	0.013113555
YLDs (Year	Uganda	Male	55+ years	Non-melanoma skin cancer (basal-cell	Number	1990	0.072668	0.14466457	0.031134379
YLDs (Year	Uganda	Female	55+ years	Non-melanoma skin cancer (basal-cell	Number	1990	0.0500957	0.094192421	0.020611862
YLDs (Year	Uganda	Both	55+ years	Non-melanoma skin cancer (basal-cell	Number	1990	0.1227637	0.238952277	0.052081587
YLDs (Year	Uganda	Male	55+ years	Non-melanoma skin cancer (basal-cell	Percent	1990	6.61E-07	1.21E-06	3.14E-07
YLDs (Year	Uganda	Female	55+ years	Non-melanoma skin cancer (basal-cell	Percent	1990	4.04E-07	7.44E-07	1.87E-07
YLDs (Year	Uganda	Both	55+ years	Non-melanoma skin cancer (basal-cell	Percent	1990	5.25E-07	9.53E-07	2.53E-07
YLDs (Year	Uganda	Male	55+ years	Non-melanoma skin cancer (basal-cell	Rate	1990	0.0141569	0.028183004	0.006065482
YLDs (Year	Uganda	Female	55+ years	Non-melanoma skin cancer (basal-cell	Rate	1990	0.0094057	0.017685014	0.003869962
YLDs (Year	Uganda	Both	55+ years	Non-melanoma skin cancer (basal-cell	Rate	1990	0.0117374	0.022846225	0.00497952
YLDs (Year	Uganda	Male	55+ years	Non-melanoma skin cancer (basal-cell	Number	2021	0.1243252	0.241700368	0.05446454
YLDs (Year	Uganda	Female	55+ years	Non-melanoma skin cancer (basal-cell	Number	2021	0.1172364	0.235941246	0.049742933
YLDs (Year	Uganda	Both	55+ years	Non-melanoma skin cancer (basal-cell	Number	2021	0.2415616	0.47958804	0.104427836
YLDs (Year	Uganda	Male	55+ years	Non-melanoma skin cancer (basal-cell	Percent	2021	5.76E-07	1.05E-06	2.65E-07
YLDs (Year	Uganda	Female	55+ years	Non-melanoma skin cancer (basal-cell	Percent	2021	3.91E-07	7.40E-07	1.82E-07
YLDs (Year	Uganda	Both	55+ years	Non-melanoma skin cancer (basal-cell	Percent	2021	4.68E-07	8.66E-07	2.19E-07

YLDs (Year	Uganda	Male	55+ years	Non-melanoma skin cancer (basal-cell	Rate	2021	0.0119551	0.023241899	0.005237308
YLDs (Year	Uganda	Female	55+ years	Non-melanoma skin cancer (basal-cell	Rate	2021	0.0088045	0.01771918	0.003735693
YLDs (Year	Uganda	Both	55+ years	Non-melanoma skin cancer (basal-cell	Rate	2021	0.0101861	0.020223049	0.004403465
YLDs (Year	Eritrea	Male	55+ years	Non-melanoma skin cancer (basal-cell	Number	1990	0.006048	0.012804377	0.002497396
YLDs (Year	Eritrea	Female	55+ years	Non-melanoma skin cancer (basal-cell	Number	1990	0.0056863	0.01154491	0.002328207
YLDs (Year	Eritrea	Both	55+ years	Non-melanoma skin cancer (basal-cell	Number	1990	0.0117343	0.024032464	0.004880907
YLDs (Year	Eritrea	Male	55+ years	Non-melanoma skin cancer (basal-cell	Percent	1990	4.06E-07	7.84E-07	1.78E-07
YLDs (Year	Eritrea	Female	55+ years	Non-melanoma skin cancer (basal-cell	Percent	1990	2.61E-07	4.88E-07	1.13E-07
YLDs (Year	Eritrea	Both	55+ years	Non-melanoma skin cancer (basal-cell	Percent	1990	3.20E-07	6.09E-07	1.39E-07
YLDs (Year	Eritrea	Male	55+ years	Non-melanoma skin cancer (basal-cell	Rate	1990	0.0079777	0.01688979	0.003294225
YLDs (Year	Eritrea	Female	55+ years	Non-melanoma skin cancer (basal-cell	Rate	1990	0.005635	0.011440919	0.002307236
YLDs (Year	Eritrea	Both	55+ years	Non-melanoma skin cancer (basal-cell	Rate	1990	0.00664	0.013599154	0.002761939
YLDs (Year	Eritrea	Male	55+ years	Non-melanoma skin cancer (basal-cell	Number	2021	0.0152095	0.03047252	0.006491428
YLDs (Year	Eritrea	Female	55+ years	Non-melanoma skin cancer (basal-cell	Number	2021	0.0153315	0.030336994	0.006320089
YLDs (Year	Eritrea	Both	55+ years	Non-melanoma skin cancer (basal-cell	Number	2021	0.0305411	0.06099125	0.012809851
YLDs (Year	Eritrea	Male	55+ years	Non-melanoma skin cancer (basal-cell	Percent	2021	3.82E-07	7.11E-07	1.73E-07
YLDs (Year	Eritrea	Female	55+ years	Non-melanoma skin cancer (basal-cell	Percent	2021	2.60E-07	4.86E-07	1.14E-07
YLDs (Year	Eritrea	Both	55+ years	Non-melanoma skin cancer (basal-cell	Percent	2021	3.10E-07	5.67E-07	1.33E-07
YLDs (Year	Eritrea	Male	55+ years	Non-melanoma skin cancer (basal-cell	Rate	2021	0.0084828	0.016995518	0.003620481
YLDs (Year	Eritrea	Female	55+ years	Non-melanoma skin cancer (basal-cell	Rate	2021	0.0058671	0.011609506	0.002418602
YLDs (Year	Eritrea	Both	55+ years	Non-melanoma skin cancer (basal-cell	Rate	2021	0.0069316	0.013842487	0.002907305
YLDs (Year	Benin	Male	55+ years	Non-melanoma skin cancer (basal-cell	Number	1990	0.0134082	0.026450904	0.005593558
YLDs (Year	Benin	Female	55+ years	Non-melanoma skin cancer (basal-cell	Number	1990	0.0085114	0.016696523	0.003356997
YLDs (Year	Benin	Both	55+ years	Non-melanoma skin cancer (basal-cell	Number	1990	0.0219196	0.042936934	0.009144069
YLDs (Year	Benin	Male	55+ years	Non-melanoma skin cancer (basal-cell	Percent	1990	4.02E-07	7.70E-07	1.79E-07
YLDs (Year	Benin	Female	55+ years	Non-melanoma skin cancer (basal-cell	Percent	1990	2.13E-07	3.92E-07	9.73E-08
YLDs (Year	Benin	Both	55+ years	Non-melanoma skin cancer (basal-cell	Percent	1990	2.99E-07	5.56E-07	1.35E-07
YLDs (Year	Benin	Male	55+ years	Non-melanoma skin cancer (basal-cell	Rate	1990	0.0086575	0.017078971	0.003611681
YLDs (Year	Benin	Female	55+ years	Non-melanoma skin cancer (basal-cell	Rate	1990	0.0051569	0.010116109	0.002033942
YLDs (Year	Benin	Both	55+ years	Non-melanoma skin cancer (basal-cell	Rate	1990	0.0068515	0.013421023	0.00285821
YLDs (Year	Benin	Male	55+ years	Non-melanoma skin cancer (basal-cell	Number	2021	0.0311367	0.06200272	0.012861285
YLDs (Year	Benin	Female	55+ years	Non-melanoma skin cancer (basal-cell	Number	2021	0.0220412	0.042508419	0.008693604
YLDs (Year	Benin	Both	55+ years	Non-melanoma skin cancer (basal-cell	Number	2021	0.0531779	0.103904766	0.021638784
YLDs (Year	Benin	Male	55+ years	Non-melanoma skin cancer (basal-cell	Percent	2021	4.07E-07	7.69E-07	1.78E-07
YLDs (Year	Benin	Female	55+ years	Non-melanoma skin cancer (basal-cell	Percent	2021	2.18E-07	4.10E-07	9.46E-08
YLDs (Year	Benin	Both	55+ years	Non-melanoma skin cancer (basal-cell	Percent	2021	2.99E-07	5.62E-07	1.30E-07
YLDs (Year	Benin	Male	55+ years	Non-melanoma skin cancer (basal-cell	Rate	2021	0.0079906	0.015911775	0.003300595
YLDs (Year	Benin	Female	55+ years	Non-melanoma skin cancer (basal-cell	Rate	2021	0.0050392	0.009718515	0.001987581
YLDs (Year	Benin	Both	55+ years	Non-melanoma skin cancer (basal-cell	Rate	2021	0.0064297	0.012563119	0.002616344
YLDs (Year	Burkina Fa	Male	55+ years	Non-melanoma skin cancer (basal-cell	Number	1990	0.0275078	0.054814391	0.011510104
YLDs (Year	Burkina Fa	Female	55+ years	Non-melanoma skin cancer (basal-cell	Number	1990	0.0176927	0.035111715	0.007053191
YLDs (Year	Burkina Fa	Both	55+ years	Non-melanoma skin cancer (basal-cell	Number	1990	0.0452005	0.088469073	0.018858957
YLDs (Year	Burkina Fa	Male	55+ years	Non-melanoma skin cancer (basal-cell	Percent	1990	3.83E-07	7.01E-07	1.68E-07
YLDs (Year	Burkina Fa	Female	55+ years	Non-melanoma skin cancer (basal-cell	Percent	1990	2.10E-07	3.88E-07	9.10E-08
YLDs (Year	Burkina Fa	Both	55+ years	Non-melanoma skin cancer (basal-cell	Percent	1990	2.90E-07	5.34E-07	1.28E-07
YLDs (Year	Burkina Fa	Male	55+ years	Non-melanoma skin cancer (basal-cell	Rate	1990	0.0078423	0.015627216	0.003281454
YLDs (Year	Burkina Fa	Female	55+ years	Non-melanoma skin cancer (basal-cell	Rate	1990	0.00475	0.009426572	0.001893596
YLDs (Year	Burkina Fa	Both	55+ years	Non-melanoma skin cancer (basal-cell	Rate	1990	0.0062497	0.012232354	0.002607572
YLDs (Year	Burkina Fa	Male	55+ years	Non-melanoma skin cancer (basal-cell	Number	2021	0.0558246	0.111240714	0.022716752
YLDs (Year	Burkina Fa	Female	55+ years	Non-melanoma skin cancer (basal-cell	Number	2021	0.0378599	0.074650218	0.015591772
YLDs (Year	Burkina Fa	Both	55+ years	Non-melanoma skin cancer (basal-cell	Number	2021	0.0936845	0.186474312	0.037893717
YLDs (Year	Burkina Fa	Male	55+ years	Non-melanoma skin cancer (basal-cell	Percent	2021	4.10E-07	7.64E-07	1.80E-07
YLDs (Year	Burkina Fa	Female	55+ years	Non-melanoma skin cancer (basal-cell	Percent	2021	2.19E-07	4.05E-07	1.00E-07
YLDs (Year	Burkina Fa	Both	55+ years	Non-melanoma skin cancer (basal-cell	Percent	2021	3.03E-07	5.62E-07	1.32E-07
YLDs (Year	Burkina Fa	Male	55+ years	Non-melanoma skin cancer (basal-cell	Rate	2021	0.0078362	0.015615097	0.003188799
YLDs (Year	Burkina Fa	Female	55+ years	Non-melanoma skin cancer (basal-cell	Rate	2021	0.0047862	0.009437182	0.001971091

YLDs (Year	Burkina Fa	Both	55+ years	Non-melanoma skin cancer (basal-cell	Rate	2021	0.0062315	0.012403388	0.00252051
YLDs (Year	Seychelles	Male	55+ years	Non-melanoma skin cancer (basal-cell	Number	1990	0.0001172	0.000234011	3.91E-05
YLDs (Year	Seychelles	Female	55+ years	Non-melanoma skin cancer (basal-cell	Number	1990	0.0001478	0.000297078	5.04E-05
YLDs (Year	Seychelles	Both	55+ years	Non-melanoma skin cancer (basal-cell	Number	1990	0.0002649	0.000526338	8.96E-05
YLDs (Year	Seychelles	Male	55+ years	Non-melanoma skin cancer (basal-cell	Percent	1990	1.45E-07	2.86E-07	5.47E-08
YLDs (Year	Seychelles	Female	55+ years	Non-melanoma skin cancer (basal-cell	Percent	1990	1.30E-07	2.56E-07	4.71E-08
YLDs (Year	Seychelles	Both	55+ years	Non-melanoma skin cancer (basal-cell	Percent	1990	1.36E-07	2.63E-07	4.96E-08
YLDs (Year	Seychelles	Male	55+ years	Non-melanoma skin cancer (basal-cell	Rate	1990	0.0028002	0.005593447	0.000934498
YLDs (Year	Seychelles	Female	55+ years	Non-melanoma skin cancer (basal-cell	Rate	1990	0.0027323	0.005492952	0.000931876
YLDs (Year	Seychelles	Both	55+ years	Non-melanoma skin cancer (basal-cell	Rate	1990	0.0027619	0.005487245	0.000934355
YLDs (Year	Seychelles	Male	55+ years	Non-melanoma skin cancer (basal-cell	Number	2021	0.0002799	0.000589002	9.97E-05
YLDs (Year	Seychelles	Female	55+ years	Non-melanoma skin cancer (basal-cell	Number	2021	0.0002541	0.000528846	7.94E-05
YLDs (Year	Seychelles	Both	55+ years	Non-melanoma skin cancer (basal-cell	Number	2021	0.000534	0.001109633	0.000178893
YLDs (Year	Seychelles	Male	55+ years	Non-melanoma skin cancer (basal-cell	Percent	2021	1.37E-07	2.71E-07	5.35E-08
YLDs (Year	Seychelles	Female	55+ years	Non-melanoma skin cancer (basal-cell	Percent	2021	1.05E-07	2.14E-07	3.67E-08
YLDs (Year	Seychelles	Both	55+ years	Non-melanoma skin cancer (basal-cell	Percent	2021	1.20E-07	2.34E-07	4.56E-08
YLDs (Year	Seychelles	Male	55+ years	Non-melanoma skin cancer (basal-cell	Rate	2021	0.0027884	0.005868318	0.000993197
YLDs (Year	Seychelles	Female	55+ years	Non-melanoma skin cancer (basal-cell	Rate	2021	0.0023737	0.004939728	0.000741627
YLDs (Year	Seychelles	Both	55+ years	Non-melanoma skin cancer (basal-cell	Rate	2021	0.0025744	0.005349448	0.000862427
YLDs (Year	Cabo Verde	Male	55+ years	Non-melanoma skin cancer (basal-cell	Number	1990	0.0016859	0.003200717	0.000686983
YLDs (Year	Cabo Verde	Female	55+ years	Non-melanoma skin cancer (basal-cell	Number	1990	0.0014864	0.002884064	0.000623045
YLDs (Year	Cabo Verde	Both	55+ years	Non-melanoma skin cancer (basal-cell	Number	1990	0.0031723	0.0060442	0.001314283
YLDs (Year	Cabo Verde	Male	55+ years	Non-melanoma skin cancer (basal-cell	Percent	1990	5.13E-07	9.30E-07	2.30E-07
YLDs (Year	Cabo Verde	Female	55+ years	Non-melanoma skin cancer (basal-cell	Percent	1990	3.04E-07	5.75E-07	1.35E-07
YLDs (Year	Cabo Verde	Both	55+ years	Non-melanoma skin cancer (basal-cell	Percent	1990	3.88E-07	7.26E-07	1.73E-07
YLDs (Year	Cabo Verde	Male	55+ years	Non-melanoma skin cancer (basal-cell	Rate	1990	0.0099084	0.01881109	0.0040375
YLDs (Year	Cabo Verde	Female	55+ years	Non-melanoma skin cancer (basal-cell	Rate	1990	0.0066196	0.012844322	0.002774761
YLDs (Year	Cabo Verde	Both	55+ years	Non-melanoma skin cancer (basal-cell	Rate	1990	0.0080374	0.015313768	0.003329907
YLDs (Year	Cabo Verde	Male	55+ years	Non-melanoma skin cancer (basal-cell	Number	2021	0.0029154	0.005822363	0.001219966
YLDs (Year	Cabo Verde	Female	55+ years	Non-melanoma skin cancer (basal-cell	Number	2021	0.0027699	0.005345623	0.001151526
YLDs (Year	Cabo Verde	Both	55+ years	Non-melanoma skin cancer (basal-cell	Number	2021	0.0056852	0.010997446	0.00234934
YLDs (Year	Cabo Verde	Male	55+ years	Non-melanoma skin cancer (basal-cell	Percent	2021	4.59E-07	8.68E-07	2.01E-07
YLDs (Year	Cabo Verde	Female	55+ years	Non-melanoma skin cancer (basal-cell	Percent	2021	2.79E-07	5.39E-07	1.22E-07
YLDs (Year	Cabo Verde	Both	55+ years	Non-melanoma skin cancer (basal-cell	Percent	2021	3.49E-07	6.62E-07	1.54E-07
YLDs (Year	Cabo Verde	Male	55+ years	Non-melanoma skin cancer (basal-cell	Rate	2021	0.0088213	0.0176172	0.00369135
YLDs (Year	Cabo Verde	Female	55+ years	Non-melanoma skin cancer (basal-cell	Rate	2021	0.0063533	0.012261483	0.002641305
YLDs (Year	Cabo Verde	Both	55+ years	Non-melanoma skin cancer (basal-cell	Rate	2021	0.0074175	0.014348328	0.003065175
YLDs (Year	Chad	Male	55+ years	Non-melanoma skin cancer (basal-cell	Number	1990	0.0189397	0.03675939	0.007823216
YLDs (Year	Chad	Female	55+ years	Non-melanoma skin cancer (basal-cell	Number	1990	0.0115464	0.022825919	0.004640801
YLDs (Year	Chad	Both	55+ years	Non-melanoma skin cancer (basal-cell	Number	1990	0.030486	0.05950924	0.012539736
YLDs (Year	Chad	Male	55+ years	Non-melanoma skin cancer (basal-cell	Percent	1990	4.03E-07	7.34E-07	1.79E-07
YLDs (Year	Chad	Female	55+ years	Non-melanoma skin cancer (basal-cell	Percent	1990	2.08E-07	3.91E-07	9.16E-08
YLDs (Year	Chad	Both	55+ years	Non-melanoma skin cancer (basal-cell	Percent	1990	2.97E-07	5.41E-07	1.34E-07
YLDs (Year	Chad	Male	55+ years	Non-melanoma skin cancer (basal-cell	Rate	1990	0.0083614	0.016228337	0.003453751
YLDs (Year	Chad	Female	55+ years	Non-melanoma skin cancer (basal-cell	Rate	1990	0.0048741	0.009635588	0.001959038
YLDs (Year	Chad	Both	55+ years	Non-melanoma skin cancer (basal-cell	Rate	1990	0.0065787	0.012841723	0.002705997
YLDs (Year	Chad	Male	55+ years	Non-melanoma skin cancer (basal-cell	Number	2021	0.0401421	0.082625619	0.016184226
YLDs (Year	Chad	Female	55+ years	Non-melanoma skin cancer (basal-cell	Number	2021	0.0189938	0.038295282	0.007637919
YLDs (Year	Chad	Both	55+ years	Non-melanoma skin cancer (basal-cell	Number	2021	0.0591359	0.120482767	0.023650012
YLDs (Year	Chad	Male	55+ years	Non-melanoma skin cancer (basal-cell	Percent	2021	3.89E-07	7.27E-07	1.75E-07
YLDs (Year	Chad	Female	55+ years	Non-melanoma skin cancer (basal-cell	Percent	2021	1.97E-07	3.62E-07	8.41E-08
YLDs (Year	Chad	Both	55+ years	Non-melanoma skin cancer (basal-cell	Percent	2021	2.96E-07	5.53E-07	1.31E-07
YLDs (Year	Chad	Male	55+ years	Non-melanoma skin cancer (basal-cell	Rate	2021	0.007825	0.016106318	0.003154812
YLDs (Year	Chad	Female	55+ years	Non-melanoma skin cancer (basal-cell	Rate	2021	0.0045711	0.009216207	0.001838155
YLDs (Year	Chad	Both	55+ years	Non-melanoma skin cancer (basal-cell	Rate	2021	0.0063688	0.012975753	0.002547059
YLDs (Year	Côte d'Ivo	Male	55+ years	Non-melanoma skin cancer (basal-cell	Number	1990	0.0183733	0.036550151	0.007414985

YLDs (Year	Côte d'Ivo	Female	55+ years	Non-melanoma skin cancer (basal-cell	Number	1990	0.0088395	0.017800116	0.003286687
YLDs (Year	Côte d'Ivo	Both	55+ years	Non-melanoma skin cancer (basal-cell	Number	1990	0.0272128	0.054828965	0.010790837
YLDs (Year	Côte d'Ivo	Male	55+ years	Non-melanoma skin cancer (basal-cell	Percent	1990	2.47E-07	4.66E-07	1.11E-07
YLDs (Year	Côte d'Ivo	Female	55+ years	Non-melanoma skin cancer (basal-cell	Percent	1990	1.23E-07	2.36E-07	5.15E-08
YLDs (Year	Côte d'Ivo	Both	55+ years	Non-melanoma skin cancer (basal-cell	Percent	1990	1.86E-07	3.48E-07	8.26E-08
YLDs (Year	Côte d'Ivo	Male	55+ years	Non-melanoma skin cancer (basal-cell	Rate	1990	0.0052718	0.010487222	0.002127559
YLDs (Year	Côte d'Ivo	Female	55+ years	Non-melanoma skin cancer (basal-cell	Rate	1990	0.003023	0.006087353	0.001123994
YLDs (Year	Côte d'Ivo	Both	55+ years	Non-melanoma skin cancer (basal-cell	Rate	1990	0.0042458	0.008554565	0.001683616
YLDs (Year	Côte d'Ivo	Male	55+ years	Non-melanoma skin cancer (basal-cell	Number	2021	0.0505846	0.098593416	0.020451563
YLDs (Year	Côte d'Ivo	Female	55+ years	Non-melanoma skin cancer (basal-cell	Number	2021	0.025124	0.050729562	0.009906236
YLDs (Year	Côte d'Ivo	Both	55+ years	Non-melanoma skin cancer (basal-cell	Number	2021	0.0757087	0.147854658	0.030038986
YLDs (Year	Côte d'Ivo	Male	55+ years	Non-melanoma skin cancer (basal-cell	Percent	2021	2.78E-07	5.12E-07	1.20E-07
YLDs (Year	Côte d'Ivo	Female	55+ years	Non-melanoma skin cancer (basal-cell	Percent	2021	1.31E-07	2.55E-07	5.66E-08
YLDs (Year	Côte d'Ivo	Both	55+ years	Non-melanoma skin cancer (basal-cell	Percent	2021	2.03E-07	3.78E-07	8.91E-08
YLDs (Year	Côte d'Ivo	Male	55+ years	Non-melanoma skin cancer (basal-cell	Rate	2021	0.0053687	0.01046398	0.002170578
YLDs (Year	Côte d'Ivo	Female	55+ years	Non-melanoma skin cancer (basal-cell	Rate	2021	0.0029524	0.005961413	0.001164117
YLDs (Year	Côte d'Ivo	Both	55+ years	Non-melanoma skin cancer (basal-cell	Rate	2021	0.004222	0.008245377	0.001675177
YLDs (Year	Gambia	Male	55+ years	Non-melanoma skin cancer (basal-cell	Number	1990	0.0021055	0.004092671	0.000869333
YLDs (Year	Gambia	Female	55+ years	Non-melanoma skin cancer (basal-cell	Number	1990	0.0013092	0.002525965	0.000542608
YLDs (Year	Gambia	Both	55+ years	Non-melanoma skin cancer (basal-cell	Number	1990	0.0034147	0.006660557	0.001417606
YLDs (Year	Gambia	Male	55+ years	Non-melanoma skin cancer (basal-cell	Percent	1990	3.88E-07	7.08E-07	1.74E-07
YLDs (Year	Gambia	Female	55+ years	Non-melanoma skin cancer (basal-cell	Percent	1990	2.20E-07	4.21E-07	9.80E-08
YLDs (Year	Gambia	Both	55+ years	Non-melanoma skin cancer (basal-cell	Percent	1990	3.00E-07	5.70E-07	1.35E-07
YLDs (Year	Gambia	Male	55+ years	Non-melanoma skin cancer (basal-cell	Rate	1990	0.0069954	0.013597916	0.002888363
YLDs (Year	Gambia	Female	55+ years	Non-melanoma skin cancer (basal-cell	Rate	1990	0.005179	0.009992007	0.002146404
YLDs (Year	Gambia	Both	55+ years	Non-melanoma skin cancer (basal-cell	Rate	1990	0.0061662	0.01202752	0.002559889
YLDs (Year	Gambia	Male	55+ years	Non-melanoma skin cancer (basal-cell	Number	2021	0.0057011	0.011132444	0.002310908
YLDs (Year	Gambia	Female	55+ years	Non-melanoma skin cancer (basal-cell	Number	2021	0.0039425	0.007677721	0.001574391
YLDs (Year	Gambia	Both	55+ years	Non-melanoma skin cancer (basal-cell	Number	2021	0.0096436	0.018550402	0.003937546
YLDs (Year	Gambia	Male	55+ years	Non-melanoma skin cancer (basal-cell	Percent	2021	3.92E-07	7.49E-07	1.66E-07
YLDs (Year	Gambia	Female	55+ years	Non-melanoma skin cancer (basal-cell	Percent	2021	2.03E-07	3.77E-07	9.04E-08
YLDs (Year	Gambia	Both	55+ years	Non-melanoma skin cancer (basal-cell	Percent	2021	2.84E-07	5.28E-07	1.23E-07
YLDs (Year	Gambia	Male	55+ years	Non-melanoma skin cancer (basal-cell	Rate	2021	0.0075076	0.014660005	0.00304317
YLDs (Year	Gambia	Female	55+ years	Non-melanoma skin cancer (basal-cell	Rate	2021	0.0050094	0.00975534	0.002000426
YLDs (Year	Gambia	Both	55+ years	Non-melanoma skin cancer (basal-cell	Rate	2021	0.0062361	0.011995841	0.002546262
YLDs (Year	Ghana	Male	55+ years	Non-melanoma skin cancer (basal-cell	Number	1990	0.0388506	0.077592135	0.016040247
YLDs (Year	Ghana	Female	55+ years	Non-melanoma skin cancer (basal-cell	Number	1990	0.0255451	0.049733094	0.010403945
YLDs (Year	Ghana	Both	55+ years	Non-melanoma skin cancer (basal-cell	Number	1990	0.0643957	0.128729794	0.026487214
YLDs (Year	Ghana	Male	55+ years	Non-melanoma skin cancer (basal-cell	Percent	1990	4.09E-07	7.63E-07	1.75E-07
YLDs (Year	Ghana	Female	55+ years	Non-melanoma skin cancer (basal-cell	Percent	1990	2.23E-07	4.33E-07	1.01E-07
YLDs (Year	Ghana	Both	55+ years	Non-melanoma skin cancer (basal-cell	Percent	1990	3.08E-07	5.78E-07	1.35E-07
YLDs (Year	Ghana	Male	55+ years	Non-melanoma skin cancer (basal-cell	Rate	1990	0.0078366	0.015651116	0.00323548
YLDs (Year	Ghana	Female	55+ years	Non-melanoma skin cancer (basal-cell	Rate	1990	0.0048616	0.009465021	0.001980041
YLDs (Year	Ghana	Both	55+ years	Non-melanoma skin cancer (basal-cell	Rate	1990	0.0063059	0.012605714	0.002593729
YLDs (Year	Ghana	Male	55+ years	Non-melanoma skin cancer (basal-cell	Number	2021	0.093279	0.182991166	0.037076585
YLDs (Year	Ghana	Female	55+ years	Non-melanoma skin cancer (basal-cell	Number	2021	0.0743495	0.146122083	0.030715838
YLDs (Year	Ghana	Both	55+ years	Non-melanoma skin cancer (basal-cell	Number	2021	0.1676285	0.330320435	0.067603194
YLDs (Year	Ghana	Male	55+ years	Non-melanoma skin cancer (basal-cell	Percent	2021	4.13E-07	7.58E-07	1.80E-07
YLDs (Year	Ghana	Female	55+ years	Non-melanoma skin cancer (basal-cell	Percent	2021	2.21E-07	4.17E-07	9.62E-08
YLDs (Year	Ghana	Both	55+ years	Non-melanoma skin cancer (basal-cell	Percent	2021	2.98E-07	5.56E-07	1.31E-07
YLDs (Year	Ghana	Male	55+ years	Non-melanoma skin cancer (basal-cell	Rate	2021	0.007848	0.015395827	0.003119411
YLDs (Year	Ghana	Female	55+ years	Non-melanoma skin cancer (basal-cell	Rate	2021	0.0048323	0.009497113	0.001996357
YLDs (Year	Ghana	Both	55+ years	Non-melanoma skin cancer (basal-cell	Rate	2021	0.0061466	0.012112201	0.002478876
YLDs (Year	Guinea	Male	55+ years	Non-melanoma skin cancer (basal-cell	Number	1990	0.0206586	0.041376151	0.008331169
YLDs (Year	Guinea	Female	55+ years	Non-melanoma skin cancer (basal-cell	Number	1990	0.0127502	0.025356424	0.005240651
YLDs (Year	Guinea	Both	55+ years	Non-melanoma skin cancer (basal-cell	Number	1990	0.0334088	0.065300385	0.013739772

YLDs (Year	Guinea	Male	55+ years	Non-melanoma skin cancer (basal-cell	Percent	1990	3.74E-07	6.81E-07	1.67E-07
YLDs (Year	Guinea	Female	55+ years	Non-melanoma skin cancer (basal-cell	Percent	1990	2.04E-07	3.80E-07	9.05E-08
YLDs (Year	Guinea	Both	55+ years	Non-melanoma skin cancer (basal-cell	Percent	1990	2.84E-07	5.22E-07	1.26E-07
YLDs (Year	Guinea	Male	55+ years	Non-melanoma skin cancer (basal-cell	Rate	1990	0.0073705	0.014761936	0.002972345
YLDs (Year	Guinea	Female	55+ years	Non-melanoma skin cancer (basal-cell	Rate	1990	0.0046686	0.009284498	0.001918915
YLDs (Year	Guinea	Both	55+ years	Non-melanoma skin cancer (basal-cell	Rate	1990	0.0060371	0.011799972	0.002482817
YLDs (Year	Guinea	Male	55+ years	Non-melanoma skin cancer (basal-cell	Number	2021	0.0339113	0.066955553	0.013261868
YLDs (Year	Guinea	Female	55+ years	Non-melanoma skin cancer (basal-cell	Number	2021	0.0200346	0.039920253	0.008091018
YLDs (Year	Guinea	Both	55+ years	Non-melanoma skin cancer (basal-cell	Number	2021	0.0539459	0.106752119	0.021529022
YLDs (Year	Guinea	Male	55+ years	Non-melanoma skin cancer (basal-cell	Percent	2021	3.70E-07	6.82E-07	1.62E-07
YLDs (Year	Guinea	Female	55+ years	Non-melanoma skin cancer (basal-cell	Percent	2021	1.98E-07	3.71E-07	8.80E-08
YLDs (Year	Guinea	Both	55+ years	Non-melanoma skin cancer (basal-cell	Percent	2021	2.80E-07	5.18E-07	1.22E-07
YLDs (Year	Guinea	Male	55+ years	Non-melanoma skin cancer (basal-cell	Rate	2021	0.0071879	0.014192012	0.002811008
YLDs (Year	Guinea	Female	55+ years	Non-melanoma skin cancer (basal-cell	Rate	2021	0.004535	0.009036334	0.00183148
YLDs (Year	Guinea	Both	55+ years	Non-melanoma skin cancer (basal-cell	Rate	2021	0.005905	0.01168531	0.002356612
YLDs (Year	Liberia	Male	55+ years	Non-melanoma skin cancer (basal-cell	Number	1990	0.0090057	0.017678932	0.003679091
YLDs (Year	Liberia	Female	55+ years	Non-melanoma skin cancer (basal-cell	Number	1990	0.0041435	0.008093339	0.001671866
YLDs (Year	Liberia	Both	55+ years	Non-melanoma skin cancer (basal-cell	Number	1990	0.0131492	0.026093648	0.005304593
YLDs (Year	Liberia	Male	55+ years	Non-melanoma skin cancer (basal-cell	Percent	1990	3.99E-07	7.56E-07	1.78E-07
YLDs (Year	Liberia	Female	55+ years	Non-melanoma skin cancer (basal-cell	Percent	1990	1.84E-07	3.48E-07	7.90E-08
YLDs (Year	Liberia	Both	55+ years	Non-melanoma skin cancer (basal-cell	Percent	1990	2.91E-07	5.52E-07	1.29E-07
YLDs (Year	Liberia	Male	55+ years	Non-melanoma skin cancer (basal-cell	Rate	1990	0.0087357	0.017149016	0.003568812
YLDs (Year	Liberia	Female	55+ years	Non-melanoma skin cancer (basal-cell	Rate	1990	0.0048548	0.009482606	0.001958851
YLDs (Year	Liberia	Both	55+ years	Non-melanoma skin cancer (basal-cell	Rate	1990	0.0069779	0.013847237	0.002815013
YLDs (Year	Liberia	Male	55+ years	Non-melanoma skin cancer (basal-cell	Number	2021	0.0137783	0.027588913	0.005503874
YLDs (Year	Liberia	Female	55+ years	Non-melanoma skin cancer (basal-cell	Number	2021	0.0073625	0.014554702	0.003021679
YLDs (Year	Liberia	Both	55+ years	Non-melanoma skin cancer (basal-cell	Number	2021	0.0211408	0.042664939	0.008526119
YLDs (Year	Liberia	Male	55+ years	Non-melanoma skin cancer (basal-cell	Percent	2021	3.75E-07	7.02E-07	1.69E-07
YLDs (Year	Liberia	Female	55+ years	Non-melanoma skin cancer (basal-cell	Percent	2021	1.80E-07	3.43E-07	8.00E-08
YLDs (Year	Liberia	Both	55+ years	Non-melanoma skin cancer (basal-cell	Percent	2021	2.72E-07	5.11E-07	1.22E-07
YLDs (Year	Liberia	Male	55+ years	Non-melanoma skin cancer (basal-cell	Rate	2021	0.0080793	0.016177594	0.003227363
YLDs (Year	Liberia	Female	55+ years	Non-melanoma skin cancer (basal-cell	Rate	2021	0.0046136	0.009120411	0.001893474
YLDs (Year	Liberia	Both	55+ years	Non-melanoma skin cancer (basal-cell	Rate	2021	0.0064039	0.012924006	0.00258272
YLDs (Year	South Suda	Male	55+ years	Non-melanoma skin cancer (basal-cell	Number	1990	0.0262144	0.052715954	0.010482485
YLDs (Year	South Suda	Female	55+ years	Non-melanoma skin cancer (basal-cell	Number	1990	0.0105944	0.021018832	0.004268164
YLDs (Year	South Suda	Both	55+ years	Non-melanoma skin cancer (basal-cell	Number	1990	0.0368087	0.072740941	0.015124691
YLDs (Year	South Suda	Male	55+ years	Non-melanoma skin cancer (basal-cell	Percent	1990	4.75E-07	8.86E-07	2.15E-07
YLDs (Year	South Suda	Female	55+ years	Non-melanoma skin cancer (basal-cell	Percent	1990	2.46E-07	4.65E-07	1.07E-07
YLDs (Year	South Suda	Both	55+ years	Non-melanoma skin cancer (basal-cell	Percent	1990	3.75E-07	7.03E-07	1.68E-07
YLDs (Year	South Suda	Male	55+ years	Non-melanoma skin cancer (basal-cell	Rate	1990	0.0104092	0.020932391	0.004162373
YLDs (Year	South Suda	Female	55+ years	Non-melanoma skin cancer (basal-cell	Rate	1990	0.0065051	0.01290589	0.002620719
YLDs (Year	South Suda	Both	55+ years	Non-melanoma skin cancer (basal-cell	Rate	1990	0.008876	0.017540555	0.003647127
YLDs (Year	South Suda	Male	55+ years	Non-melanoma skin cancer (basal-cell	Number	2021	0.0316013	0.062314417	0.013118875
YLDs (Year	South Suda	Female	55+ years	Non-melanoma skin cancer (basal-cell	Number	2021	0.0176279	0.035317399	0.007293276
YLDs (Year	South Suda	Both	55+ years	Non-melanoma skin cancer (basal-cell	Number	2021	0.0492291	0.097591627	0.020513105
YLDs (Year	South Suda	Male	55+ years	Non-melanoma skin cancer (basal-cell	Percent	2021	4.39E-07	8.28E-07	1.91E-07
YLDs (Year	South Suda	Female	55+ years	Non-melanoma skin cancer (basal-cell	Percent	2021	2.45E-07	4.67E-07	1.07E-07
YLDs (Year	South Suda	Both	55+ years	Non-melanoma skin cancer (basal-cell	Percent	2021	3.42E-07	6.48E-07	1.49E-07
YLDs (Year	South Suda	Male	55+ years	Non-melanoma skin cancer (basal-cell	Rate	2021	0.0095167	0.018765887	0.003950728
YLDs (Year	South Suda	Female	55+ years	Non-melanoma skin cancer (basal-cell	Rate	2021	0.0060648	0.012150748	0.00250921
YLDs (Year	South Suda	Both	55+ years	Non-melanoma skin cancer (basal-cell	Rate	2021	0.0079055	0.015671769	0.003294101
YLDs (Year	Niger	Male	55+ years	Non-melanoma skin cancer (basal-cell	Number	1990	0.0186481	0.037621472	0.007664227
YLDs (Year	Niger	Female	55+ years	Non-melanoma skin cancer (basal-cell	Number	1990	0.0094436	0.01873531	0.003849236
YLDs (Year	Niger	Both	55+ years	Non-melanoma skin cancer (basal-cell	Number	1990	0.0280917	0.056272094	0.011505343
YLDs (Year	Niger	Male	55+ years	Non-melanoma skin cancer (basal-cell	Percent	1990	4.12E-07	7.71E-07	1.84E-07
YLDs (Year	Niger	Female	55+ years	Non-melanoma skin cancer (basal-cell	Percent	1990	2.09E-07	3.85E-07	9.34E-08

YLDs (Year	Niger	Both	55+ years	Non-melanoma skin cancer (basal-cell	Percent	1990	3.11E-07	5.71E-07	1.41E-07
YLDs (Year	Niger	Male	55+ years	Non-melanoma skin cancer (basal-cell	Rate	1990	0.007898	0.015933735	0.003246012
YLDs (Year	Niger	Female	55+ years	Non-melanoma skin cancer (basal-cell	Rate	1990	0.0046432	0.009211666	0.001892569
YLDs (Year	Niger	Both	55+ years	Non-melanoma skin cancer (basal-cell	Rate	1990	0.0063918	0.012803696	0.002617832
YLDs (Year	Niger	Male	55+ years	Non-melanoma skin cancer (basal-cell	Number	2021	0.0539683	0.105499022	0.021756708
YLDs (Year	Niger	Female	55+ years	Non-melanoma skin cancer (basal-cell	Number	2021	0.03171	0.063231867	0.012656915
YLDs (Year	Niger	Both	55+ years	Non-melanoma skin cancer (basal-cell	Number	2021	0.0856783	0.166446053	0.034540535
YLDs (Year	Niger	Male	55+ years	Non-melanoma skin cancer (basal-cell	Percent	2021	4.29E-07	7.79E-07	1.92E-07
YLDs (Year	Niger	Female	55+ years	Non-melanoma skin cancer (basal-cell	Percent	2021	2.03E-07	3.78E-07	8.72E-08
YLDs (Year	Niger	Both	55+ years	Non-melanoma skin cancer (basal-cell	Percent	2021	3.04E-07	5.53E-07	1.31E-07
YLDs (Year	Niger	Male	55+ years	Non-melanoma skin cancer (basal-cell	Rate	2021	0.0078799	0.015403934	0.003176701
YLDs (Year	Niger	Female	55+ years	Non-melanoma skin cancer (basal-cell	Rate	2021	0.0044831	0.008939501	0.001789391
YLDs (Year	Niger	Both	55+ years	Non-melanoma skin cancer (basal-cell	Rate	2021	0.0061541	0.011955488	0.002480978
YLDs (Year	Guinea-Bis	Male	55+ years	Non-melanoma skin cancer (basal-cell	Number	1990	0.0023316	0.004594059	0.000974515
YLDs (Year	Guinea-Bis	Female	55+ years	Non-melanoma skin cancer (basal-cell	Number	1990	0.0014107	0.002850701	0.000580608
YLDs (Year	Guinea-Bis	Both	55+ years	Non-melanoma skin cancer (basal-cell	Number	1990	0.0037423	0.007412811	0.001588612
YLDs (Year	Guinea-Bis	Male	55+ years	Non-melanoma skin cancer (basal-cell	Percent	1990	3.60E-07	6.60E-07	1.56E-07
YLDs (Year	Guinea-Bis	Female	55+ years	Non-melanoma skin cancer (basal-cell	Percent	1990	1.93E-07	3.65E-07	8.24E-08
YLDs (Year	Guinea-Bis	Both	55+ years	Non-melanoma skin cancer (basal-cell	Percent	1990	2.71E-07	4.97E-07	1.16E-07
YLDs (Year	Guinea-Bis	Male	55+ years	Non-melanoma skin cancer (basal-cell	Rate	1990	0.0073707	0.014522836	0.003080659
YLDs (Year	Guinea-Bis	Female	55+ years	Non-melanoma skin cancer (basal-cell	Rate	1990	0.0043812	0.008853192	0.001803147
YLDs (Year	Guinea-Bis	Both	55+ years	Non-melanoma skin cancer (basal-cell	Rate	1990	0.0058627	0.011612811	0.002488699
YLDs (Year	Guinea-Bis	Male	55+ years	Non-melanoma skin cancer (basal-cell	Number	2021	0.0036749	0.007446778	0.001495655
YLDs (Year	Guinea-Bis	Female	55+ years	Non-melanoma skin cancer (basal-cell	Number	2021	0.0026487	0.005335252	0.001057591
YLDs (Year	Guinea-Bis	Both	55+ years	Non-melanoma skin cancer (basal-cell	Number	2021	0.0063236	0.012769143	0.002534641
YLDs (Year	Guinea-Bis	Male	55+ years	Non-melanoma skin cancer (basal-cell	Percent	2021	3.63E-07	6.79E-07	1.57E-07
YLDs (Year	Guinea-Bis	Female	55+ years	Non-melanoma skin cancer (basal-cell	Percent	2021	1.90E-07	3.54E-07	8.24E-08
YLDs (Year	Guinea-Bis	Both	55+ years	Non-melanoma skin cancer (basal-cell	Percent	2021	2.63E-07	4.90E-07	1.13E-07
YLDs (Year	Guinea-Bis	Male	55+ years	Non-melanoma skin cancer (basal-cell	Rate	2021	0.00692	0.014022577	0.002816378
YLDs (Year	Guinea-Bis	Female	55+ years	Non-melanoma skin cancer (basal-cell	Rate	2021	0.0042884	0.008638027	0.001712291
YLDs (Year	Guinea-Bis	Both	55+ years	Non-melanoma skin cancer (basal-cell	Rate	2021	0.005505	0.011116137	0.002206524
YLDs (Year	Nigeria	Male	55+ years	Non-melanoma skin cancer (basal-cell	Number	1990	0.2794693	0.547267429	0.114490829
YLDs (Year	Nigeria	Female	55+ years	Non-melanoma skin cancer (basal-cell	Number	1990	0.1540435	0.296658235	0.063571537
YLDs (Year	Nigeria	Both	55+ years	Non-melanoma skin cancer (basal-cell	Number	1990	0.4335129	0.857102133	0.179715479
YLDs (Year	Nigeria	Male	55+ years	Non-melanoma skin cancer (basal-cell	Percent	1990	3.55E-07	6.50E-07	1.59E-07
YLDs (Year	Nigeria	Female	55+ years	Non-melanoma skin cancer (basal-cell	Percent	1990	1.84E-07	3.42E-07	8.32E-08
YLDs (Year	Nigeria	Both	55+ years	Non-melanoma skin cancer (basal-cell	Percent	1990	2.67E-07	5.07E-07	1.22E-07
YLDs (Year	Nigeria	Male	55+ years	Non-melanoma skin cancer (basal-cell	Rate	1990	0.0072137	0.014126097	0.002955244
YLDs (Year	Nigeria	Female	55+ years	Non-melanoma skin cancer (basal-cell	Rate	1990	0.0045163	0.008697437	0.001863793
YLDs (Year	Nigeria	Both	55+ years	Non-melanoma skin cancer (basal-cell	Rate	1990	0.0059507	0.011765254	0.002466915
YLDs (Year	Nigeria	Male	55+ years	Non-melanoma skin cancer (basal-cell	Number	2021	0.4930589	0.970112229	0.202137003
YLDs (Year	Nigeria	Female	55+ years	Non-melanoma skin cancer (basal-cell	Number	2021	0.3332263	0.65757594	0.130695717
YLDs (Year	Nigeria	Both	55+ years	Non-melanoma skin cancer (basal-cell	Number	2021	0.8262852	1.627075831	0.333815115
YLDs (Year	Nigeria	Male	55+ years	Non-melanoma skin cancer (basal-cell	Percent	2021	3.80E-07	7.07E-07	1.69E-07
YLDs (Year	Nigeria	Female	55+ years	Non-melanoma skin cancer (basal-cell	Percent	2021	1.77E-07	3.37E-07	7.61E-08
YLDs (Year	Nigeria	Both	55+ years	Non-melanoma skin cancer (basal-cell	Percent	2021	2.59E-07	4.86E-07	1.15E-07
YLDs (Year	Nigeria	Male	55+ years	Non-melanoma skin cancer (basal-cell	Rate	2021	0.0073666	0.014494157	0.003020069
YLDs (Year	Nigeria	Female	55+ years	Non-melanoma skin cancer (basal-cell	Rate	2021	0.0040107	0.007914605	0.001573058
YLDs (Year	Nigeria	Both	55+ years	Non-melanoma skin cancer (basal-cell	Rate	2021	0.005508	0.010846079	0.00222521
YLDs (Year	Mauritania	Male	55+ years	Non-melanoma skin cancer (basal-cell	Number	1990	0.0071285	0.014293022	0.00288205
YLDs (Year	Mauritania	Female	55+ years	Non-melanoma skin cancer (basal-cell	Number	1990	0.0043714	0.008375456	0.001798012
YLDs (Year	Mauritania	Both	55+ years	Non-melanoma skin cancer (basal-cell	Number	1990	0.0114998	0.02264118	0.004669851
YLDs (Year	Mauritania	Male	55+ years	Non-melanoma skin cancer (basal-cell	Percent	1990	4.73E-07	8.65E-07	2.09E-07
YLDs (Year	Mauritania	Female	55+ years	Non-melanoma skin cancer (basal-cell	Percent	1990	2.29E-07	4.22E-07	1.03E-07
YLDs (Year	Mauritania	Both	55+ years	Non-melanoma skin cancer (basal-cell	Percent	1990	3.36E-07	6.18E-07	1.55E-07
YLDs (Year	Mauritania	Male	55+ years	Non-melanoma skin cancer (basal-cell	Rate	1990	0.0089449	0.017934943	0.003616409

YLDs (Year	Mauritania	Female	55+ years	Non-melanoma skin cancer (basal-cell	Rate	1990	0.0050642	0.009703031	0.00208301
YLDs (Year	Mauritania	Both	55+ years	Non-melanoma skin cancer (basal-cell	Rate	1990	0.0069271	0.013638308	0.002812966
YLDs (Year	Mauritania	Male	55+ years	Non-melanoma skin cancer (basal-cell	Number	2021	0.0163237	0.03254862	0.006540108
YLDs (Year	Mauritania	Female	55+ years	Non-melanoma skin cancer (basal-cell	Number	2021	0.0087219	0.017240223	0.003543578
YLDs (Year	Mauritania	Both	55+ years	Non-melanoma skin cancer (basal-cell	Number	2021	0.0250456	0.049369632	0.010213849
YLDs (Year	Mauritania	Male	55+ years	Non-melanoma skin cancer (basal-cell	Percent	2021	4.96E-07	9.16E-07	2.20E-07
YLDs (Year	Mauritania	Female	55+ years	Non-melanoma skin cancer (basal-cell	Percent	2021	2.24E-07	4.25E-07	1.01E-07
YLDs (Year	Mauritania	Both	55+ years	Non-melanoma skin cancer (basal-cell	Percent	2021	3.48E-07	6.40E-07	1.56E-07
YLDs (Year	Mauritania	Male	55+ years	Non-melanoma skin cancer (basal-cell	Rate	2021	0.009084	0.018113127	0.003639534
YLDs (Year	Mauritania	Female	55+ years	Non-melanoma skin cancer (basal-cell	Rate	2021	0.0048709	0.009628105	0.001978973
YLDs (Year	Mauritania	Both	55+ years	Non-melanoma skin cancer (basal-cell	Rate	2021	0.0069812	0.013761273	0.002847005
YLDs (Year	Bermuda	Male	55+ years	Non-melanoma skin cancer (basal-cell	Number	1990	0.0004615	0.000901313	0.000181526
YLDs (Year	Bermuda	Female	55+ years	Non-melanoma skin cancer (basal-cell	Number	1990	0.0003821	0.000750649	0.00014753
YLDs (Year	Bermuda	Both	55+ years	Non-melanoma skin cancer (basal-cell	Number	1990	0.0008436	0.001629694	0.000326606
YLDs (Year	Bermuda	Male	55+ years	Non-melanoma skin cancer (basal-cell	Percent	1990	5.45E-07	9.91E-07	2.47E-07
YLDs (Year	Bermuda	Female	55+ years	Non-melanoma skin cancer (basal-cell	Percent	1990	3.24E-07	6.17E-07	1.28E-07
YLDs (Year	Bermuda	Both	55+ years	Non-melanoma skin cancer (basal-cell	Percent	1990	4.16E-07	7.73E-07	1.82E-07
YLDs (Year	Bermuda	Male	55+ years	Non-melanoma skin cancer (basal-cell	Rate	1990	0.0099445	0.019423007	0.003911834
YLDs (Year	Bermuda	Female	55+ years	Non-melanoma skin cancer (basal-cell	Rate	1990	0.0064354	0.012641127	0.002484434
YLDs (Year	Bermuda	Both	55+ years	Non-melanoma skin cancer (basal-cell	Rate	1990	0.0079747	0.015405582	0.003087427
YLDs (Year	Bermuda	Male	55+ years	Non-melanoma skin cancer (basal-cell	Number	2021	0.0010705	0.002080279	0.000435119
YLDs (Year	Bermuda	Female	55+ years	Non-melanoma skin cancer (basal-cell	Number	2021	0.0008195	0.00163258	0.000300204
YLDs (Year	Bermuda	Both	55+ years	Non-melanoma skin cancer (basal-cell	Number	2021	0.00189	0.003640221	0.000714505
YLDs (Year	Bermuda	Male	55+ years	Non-melanoma skin cancer (basal-cell	Percent	2021	5.02E-07	9.16E-07	2.27E-07
YLDs (Year	Bermuda	Female	55+ years	Non-melanoma skin cancer (basal-cell	Percent	2021	3.01E-07	5.65E-07	1.20E-07
YLDs (Year	Bermuda	Both	55+ years	Non-melanoma skin cancer (basal-cell	Percent	2021	3.89E-07	7.35E-07	1.69E-07
YLDs (Year	Bermuda	Male	55+ years	Non-melanoma skin cancer (basal-cell	Rate	2021	0.0101545	0.019733271	0.004127483
YLDs (Year	Bermuda	Female	55+ years	Non-melanoma skin cancer (basal-cell	Rate	2021	0.0064278	0.01280568	0.002354751
YLDs (Year	Bermuda	Both	55+ years	Non-melanoma skin cancer (basal-cell	Rate	2021	0.0081146	0.015629397	0.003067747
YLDs (Year	Sao Tome a	Male	55+ years	Non-melanoma skin cancer (basal-cell	Number	1990	0.0004575	0.000892771	0.000187661
YLDs (Year	Sao Tome a	Female	55+ years	Non-melanoma skin cancer (basal-cell	Number	1990	0.0003192	0.000625492	0.000130817
YLDs (Year	Sao Tome a	Both	55+ years	Non-melanoma skin cancer (basal-cell	Number	1990	0.0007767	0.001521422	0.000314941
YLDs (Year	Sao Tome a	Male	55+ years	Non-melanoma skin cancer (basal-cell	Percent	1990	4.74E-07	8.65E-07	2.08E-07
YLDs (Year	Sao Tome a	Female	55+ years	Non-melanoma skin cancer (basal-cell	Percent	1990	2.45E-07	4.61E-07	1.11E-07
YLDs (Year	Sao Tome a	Both	55+ years	Non-melanoma skin cancer (basal-cell	Percent	1990	3.42E-07	6.43E-07	1.53E-07
YLDs (Year	Sao Tome a	Male	55+ years	Non-melanoma skin cancer (basal-cell	Rate	1990	0.0086721	0.016924096	0.003557455
YLDs (Year	Sao Tome a	Female	55+ years	Non-melanoma skin cancer (basal-cell	Rate	1990	0.0053896	0.0105611	0.00220877
YLDs (Year	Sao Tome a	Both	55+ years	Non-melanoma skin cancer (basal-cell	Rate	1990	0.0069359	0.013586856	0.002812538
YLDs (Year	Sao Tome a	Male	55+ years	Non-melanoma skin cancer (basal-cell	Number	2021	0.0007307	0.001414509	0.000311714
YLDs (Year	Sao Tome a	Female	55+ years	Non-melanoma skin cancer (basal-cell	Number	2021	0.0004826	0.000952373	0.000197188
YLDs (Year	Sao Tome a	Both	55+ years	Non-melanoma skin cancer (basal-cell	Number	2021	0.0012134	0.002379019	0.000501148
YLDs (Year	Sao Tome a	Male	55+ years	Non-melanoma skin cancer (basal-cell	Percent	2021	4.54E-07	8.17E-07	2.06E-07
YLDs (Year	Sao Tome a	Female	55+ years	Non-melanoma skin cancer (basal-cell	Percent	2021	2.27E-07	4.18E-07	1.04E-07
YLDs (Year	Sao Tome a	Both	55+ years	Non-melanoma skin cancer (basal-cell	Percent	2021	3.25E-07	5.92E-07	1.44E-07
YLDs (Year	Sao Tome a	Male	55+ years	Non-melanoma skin cancer (basal-cell	Rate	2021	0.0083483	0.016160409	0.003561251
YLDs (Year	Sao Tome a	Female	55+ years	Non-melanoma skin cancer (basal-cell	Rate	2021	0.005064	0.009992437	0.002068923
YLDs (Year	Sao Tome a	Both	55+ years	Non-melanoma skin cancer (basal-cell	Rate	2021	0.0066363	0.013011572	0.002740932
YLDs (Year	American S	Male	55+ years	Non-melanoma skin cancer (basal-cell	Number	1990	1.94E-06	5.24E-06	4.14E-07
YLDs (Year	American S	Female	55+ years	Non-melanoma skin cancer (basal-cell	Number	1990	2.01E-06	5.24E-06	4.40E-07
YLDs (Year	American S	Both	55+ years	Non-melanoma skin cancer (basal-cell	Number	1990	3.95E-06	1.04E-05	9.28E-07
YLDs (Year	American S	Male	55+ years	Non-melanoma skin cancer (basal-cell	Percent	1990	5.37E-09	1.36E-08	1.21E-09
YLDs (Year	American S	Female	55+ years	Non-melanoma skin cancer (basal-cell	Percent	1990	5.23E-09	1.33E-08	1.28E-09
YLDs (Year	American S	Both	55+ years	Non-melanoma skin cancer (basal-cell	Percent	1990	5.30E-09	1.33E-08	1.30E-09
YLDs (Year	American S	Male	55+ years	Non-melanoma skin cancer (basal-cell	Rate	1990	0.0001044	0.000282137	2.23E-05
YLDs (Year	American S	Female	55+ years	Non-melanoma skin cancer (basal-cell	Rate	1990	0.0001118	0.000291326	2.45E-05
YLDs (Year	American S	Both	55+ years	Non-melanoma skin cancer (basal-cell	Rate	1990	0.0001081	0.000284427	2.54E-05

YLDs (Year	American S	Male	55+ years	Non-melanoma skin cancer (basal-cell	Number	2021	4.47E-06	1.20E-05	9.68E-07
YLDs (Year	American S	Female	55+ years	Non-melanoma skin cancer (basal-cell	Number	2021	4.69E-06	1.21E-05	1.06E-06
YLDs (Year	American S	Both	55+ years	Non-melanoma skin cancer (basal-cell	Number	2021	9.16E-06	2.39E-05	2.21E-06
YLDs (Year	American S	Male	55+ years	Non-melanoma skin cancer (basal-cell	Percent	2021	4.82E-09	1.21E-08	1.10E-09
YLDs (Year	American S	Female	55+ years	Non-melanoma skin cancer (basal-cell	Percent	2021	4.62E-09	1.15E-08	1.14E-09
YLDs (Year	American S	Both	55+ years	Non-melanoma skin cancer (basal-cell	Percent	2021	4.71E-09	1.17E-08	1.17E-09
YLDs (Year	American S	Male	55+ years	Non-melanoma skin cancer (basal-cell	Rate	2021	0.0001048	0.000281012	2.27E-05
YLDs (Year	American S	Female	55+ years	Non-melanoma skin cancer (basal-cell	Rate	2021	0.0001122	0.000289161	2.53E-05
YLDs (Year	American S	Both	55+ years	Non-melanoma skin cancer (basal-cell	Rate	2021	0.0001085	0.000282672	2.61E-05
YLDs (Year	Mali	Male	55+ years	Non-melanoma skin cancer (basal-cell	Number	1990	0.033216	0.065163751	0.013557215
YLDs (Year	Mali	Female	55+ years	Non-melanoma skin cancer (basal-cell	Number	1990	0.023477	0.046960227	0.009993412
YLDs (Year	Mali	Both	55+ years	Non-melanoma skin cancer (basal-cell	Number	1990	0.0566931	0.112129788	0.023595354
YLDs (Year	Mali	Male	55+ years	Non-melanoma skin cancer (basal-cell	Percent	1990	4.85E-07	8.77E-07	2.22E-07
YLDs (Year	Mali	Female	55+ years	Non-melanoma skin cancer (basal-cell	Percent	1990	3.16E-07	5.93E-07	1.40E-07
YLDs (Year	Mali	Both	55+ years	Non-melanoma skin cancer (basal-cell	Percent	1990	3.97E-07	7.32E-07	1.80E-07
YLDs (Year	Mali	Male	55+ years	Non-melanoma skin cancer (basal-cell	Rate	1990	0.0097562	0.019139953	0.003982037
YLDs (Year	Mali	Female	55+ years	Non-melanoma skin cancer (basal-cell	Rate	1990	0.0071533	0.014308511	0.003044935
YLDs (Year	Mali	Both	55+ years	Non-melanoma skin cancer (basal-cell	Rate	1990	0.0084786	0.016769399	0.003528767
YLDs (Year	Mali	Male	55+ years	Non-melanoma skin cancer (basal-cell	Number	2021	0.0755965	0.151219451	0.031599625
YLDs (Year	Mali	Female	55+ years	Non-melanoma skin cancer (basal-cell	Number	2021	0.0468673	0.093871489	0.018896418
YLDs (Year	Mali	Both	55+ years	Non-melanoma skin cancer (basal-cell	Number	2021	0.1224638	0.247022722	0.049932363
YLDs (Year	Mali	Male	55+ years	Non-melanoma skin cancer (basal-cell	Percent	2021	5.08E-07	9.28E-07	2.31E-07
YLDs (Year	Mali	Female	55+ years	Non-melanoma skin cancer (basal-cell	Percent	2021	2.89E-07	5.55E-07	1.26E-07
YLDs (Year	Mali	Both	55+ years	Non-melanoma skin cancer (basal-cell	Percent	2021	3.94E-07	7.32E-07	1.75E-07
YLDs (Year	Mali	Male	55+ years	Non-melanoma skin cancer (basal-cell	Rate	2021	0.0100837	0.020170997	0.004215039
YLDs (Year	Mali	Female	55+ years	Non-melanoma skin cancer (basal-cell	Rate	2021	0.0067175	0.013454703	0.002708444
YLDs (Year	Mali	Both	55+ years	Non-melanoma skin cancer (basal-cell	Rate	2021	0.0084611	0.017066972	0.003449862
YLDs (Year	Togo	Male	55+ years	Non-melanoma skin cancer (basal-cell	Number	1990	0.0073666	0.014550096	0.002969033
YLDs (Year	Togo	Female	55+ years	Non-melanoma skin cancer (basal-cell	Number	1990	0.0048592	0.009764955	0.001963957
YLDs (Year	Togo	Both	55+ years	Non-melanoma skin cancer (basal-cell	Number	1990	0.0122258	0.023998556	0.004932154
YLDs (Year	Togo	Male	55+ years	Non-melanoma skin cancer (basal-cell	Percent	1990	4.02E-07	7.40E-07	1.78E-07
YLDs (Year	Togo	Female	55+ years	Non-melanoma skin cancer (basal-cell	Percent	1990	2.15E-07	4.24E-07	9.53E-08
YLDs (Year	Togo	Both	55+ years	Non-melanoma skin cancer (basal-cell	Percent	1990	2.99E-07	5.58E-07	1.35E-07
YLDs (Year	Togo	Male	55+ years	Non-melanoma skin cancer (basal-cell	Rate	1990	0.007833	0.015471198	0.003156989
YLDs (Year	Togo	Female	55+ years	Non-melanoma skin cancer (basal-cell	Rate	1990	0.0048097	0.009665524	0.00194396
YLDs (Year	Togo	Both	55+ years	Non-melanoma skin cancer (basal-cell	Rate	1990	0.0062672	0.012302216	0.002528337
YLDs (Year	Togo	Male	55+ years	Non-melanoma skin cancer (basal-cell	Number	2021	0.0199789	0.040004356	0.00835866
YLDs (Year	Togo	Female	55+ years	Non-melanoma skin cancer (basal-cell	Number	2021	0.0168176	0.033346755	0.006777472
YLDs (Year	Togo	Both	55+ years	Non-melanoma skin cancer (basal-cell	Number	2021	0.0367965	0.073398175	0.015144659
YLDs (Year	Togo	Male	55+ years	Non-melanoma skin cancer (basal-cell	Percent	2021	4.04E-07	7.83E-07	1.83E-07
YLDs (Year	Togo	Female	55+ years	Non-melanoma skin cancer (basal-cell	Percent	2021	2.17E-07	4.14E-07	9.23E-08
YLDs (Year	Togo	Both	55+ years	Non-melanoma skin cancer (basal-cell	Percent	2021	2.90E-07	5.51E-07	1.28E-07
YLDs (Year	Togo	Male	55+ years	Non-melanoma skin cancer (basal-cell	Rate	2021	0.007319	0.01465498	0.003062066
YLDs (Year	Togo	Female	55+ years	Non-melanoma skin cancer (basal-cell	Rate	2021	0.0047816	0.009481238	0.001926989
YLDs (Year	Togo	Both	55+ years	Non-melanoma skin cancer (basal-cell	Rate	2021	0.0058904	0.011749582	0.002424357
YLDs (Year	Senegal	Male	55+ years	Non-melanoma skin cancer (basal-cell	Number	1990	0.0357092	0.069446657	0.014875512
YLDs (Year	Senegal	Female	55+ years	Non-melanoma skin cancer (basal-cell	Number	1990	0.0215687	0.04179162	0.008493289
YLDs (Year	Senegal	Both	55+ years	Non-melanoma skin cancer (basal-cell	Number	1990	0.057278	0.111473522	0.023331383
YLDs (Year	Senegal	Male	55+ years	Non-melanoma skin cancer (basal-cell	Percent	1990	6.63E-07	1.23E-06	2.92E-07
YLDs (Year	Senegal	Female	55+ years	Non-melanoma skin cancer (basal-cell	Percent	1990	3.70E-07	6.77E-07	1.62E-07
YLDs (Year	Senegal	Both	55+ years	Non-melanoma skin cancer (basal-cell	Percent	1990	5.10E-07	9.37E-07	2.24E-07
YLDs (Year	Senegal	Male	55+ years	Non-melanoma skin cancer (basal-cell	Rate	1990	0.0131416	0.025557551	0.005474441
YLDs (Year	Senegal	Female	55+ years	Non-melanoma skin cancer (basal-cell	Rate	1990	0.0084021	0.016279976	0.003308571
YLDs (Year	Senegal	Both	55+ years	Non-melanoma skin cancer (basal-cell	Rate	1990	0.0108392	0.02109514	0.004415208
YLDs (Year	Senegal	Male	55+ years	Non-melanoma skin cancer (basal-cell	Number	2021	0.0802314	0.158292175	0.033841364
YLDs (Year	Senegal	Female	55+ years	Non-melanoma skin cancer (basal-cell	Number	2021	0.0558425	0.11186201	0.022380351

YLDs (Year	Senegal	Both	55+ years	Non-melanoma skin cancer (basal-cell	Number	2021	0.1360739	0.270145029	0.055963383
YLDs (Year	Senegal	Male	55+ years	Non-melanoma skin cancer (basal-cell	Percent	2021	6.56E-07	1.20E-06	2.86E-07
YLDs (Year	Senegal	Female	55+ years	Non-melanoma skin cancer (basal-cell	Percent	2021	3.56E-07	6.66E-07	1.55E-07
YLDs (Year	Senegal	Both	55+ years	Non-melanoma skin cancer (basal-cell	Percent	2021	4.88E-07	8.97E-07	2.10E-07
YLDs (Year	Senegal	Male	55+ years	Non-melanoma skin cancer (basal-cell	Rate	2021	0.0129017	0.025454287	0.005441885
YLDs (Year	Senegal	Female	55+ years	Non-melanoma skin cancer (basal-cell	Rate	2021	0.0083435	0.016713358	0.003343859
YLDs (Year	Senegal	Both	55+ years	Non-melanoma skin cancer (basal-cell	Rate	2021	0.0105388	0.020922572	0.004334331
YLDs (Year	Cook Islan	Male	55+ years	Non-melanoma skin cancer (basal-cell	Number	1990	1.28E-06	3.41E-06	2.86E-07
YLDs (Year	Cook Islan	Female	55+ years	Non-melanoma skin cancer (basal-cell	Number	1990	1.24E-06	3.19E-06	2.97E-07
YLDs (Year	Cook Islan	Both	55+ years	Non-melanoma skin cancer (basal-cell	Number	1990	2.52E-06	6.59E-06	6.43E-07
YLDs (Year	Cook Islan	Male	55+ years	Non-melanoma skin cancer (basal-cell	Percent	1990	5.82E-09	1.43E-08	1.37E-09
YLDs (Year	Cook Islan	Female	55+ years	Non-melanoma skin cancer (basal-cell	Percent	1990	5.69E-09	1.42E-08	1.48E-09
YLDs (Year	Cook Islan	Both	55+ years	Non-melanoma skin cancer (basal-cell	Percent	1990	5.76E-09	1.42E-08	1.58E-09
YLDs (Year	Cook Islan	Male	55+ years	Non-melanoma skin cancer (basal-cell	Rate	1990	0.0001122	0.000298954	2.50E-05
YLDs (Year	Cook Islan	Female	55+ years	Non-melanoma skin cancer (basal-cell	Rate	1990	0.0001254	0.00032157	2.99E-05
YLDs (Year	Cook Islan	Both	55+ years	Non-melanoma skin cancer (basal-cell	Rate	1990	0.0001183	0.000308976	3.01E-05
YLDs (Year	Cook Islan	Male	55+ years	Non-melanoma skin cancer (basal-cell	Number	2021	2.63E-06	6.91E-06	6.01E-07
YLDs (Year	Cook Islan	Female	55+ years	Non-melanoma skin cancer (basal-cell	Number	2021	2.99E-06	7.57E-06	6.81E-07
YLDs (Year	Cook Islan	Both	55+ years	Non-melanoma skin cancer (basal-cell	Number	2021	5.62E-06	1.43E-05	1.44E-06
YLDs (Year	Cook Islan	Male	55+ years	Non-melanoma skin cancer (basal-cell	Percent	2021	5.18E-09	1.25E-08	1.25E-09
YLDs (Year	Cook Islan	Female	55+ years	Non-melanoma skin cancer (basal-cell	Percent	2021	5.16E-09	1.26E-08	1.33E-09
YLDs (Year	Cook Islan	Both	55+ years	Non-melanoma skin cancer (basal-cell	Percent	2021	5.17E-09	1.23E-08	1.40E-09
YLDs (Year	Cook Islan	Male	55+ years	Non-melanoma skin cancer (basal-cell	Rate	2021	0.0001125	0.000296124	2.58E-05
YLDs (Year	Cook Islan	Female	55+ years	Non-melanoma skin cancer (basal-cell	Rate	2021	0.0001257	0.000318245	2.86E-05
YLDs (Year	Cook Islan	Both	55+ years	Non-melanoma skin cancer (basal-cell	Rate	2021	0.0001192	0.000303107	3.05E-05
YLDs (Year	Guam	Male	55+ years	Non-melanoma skin cancer (basal-cell	Number	1990	7.53E-06	2.04E-05	1.59E-06
YLDs (Year	Guam	Female	55+ years	Non-melanoma skin cancer (basal-cell	Number	1990	7.84E-06	2.00E-05	1.82E-06
YLDs (Year	Guam	Both	55+ years	Non-melanoma skin cancer (basal-cell	Number	1990	1.54E-05	4.05E-05	3.55E-06
YLDs (Year	Guam	Male	55+ years	Non-melanoma skin cancer (basal-cell	Percent	1990	6.81E-09	1.66E-08	1.51E-09
YLDs (Year	Guam	Female	55+ years	Non-melanoma skin cancer (basal-cell	Percent	1990	6.43E-09	1.58E-08	1.53E-09
YLDs (Year	Guam	Both	55+ years	Non-melanoma skin cancer (basal-cell	Percent	1990	6.61E-09	1.59E-08	1.64E-09
YLDs (Year	Guam	Male	55+ years	Non-melanoma skin cancer (basal-cell	Rate	1990	0.0001134	0.000307207	2.40E-05
YLDs (Year	Guam	Female	55+ years	Non-melanoma skin cancer (basal-cell	Rate	1990	0.0001253	0.000320201	2.91E-05
YLDs (Year	Guam	Both	55+ years	Non-melanoma skin cancer (basal-cell	Rate	1990	0.0001192	0.000313761	2.75E-05
YLDs (Year	Guam	Male	55+ years	Non-melanoma skin cancer (basal-cell	Number	2021	2.07E-05	5.26E-05	4.71E-06
YLDs (Year	Guam	Female	55+ years	Non-melanoma skin cancer (basal-cell	Number	2021	2.51E-05	6.25E-05	5.85E-06
YLDs (Year	Guam	Both	55+ years	Non-melanoma skin cancer (basal-cell	Number	2021	4.58E-05	0.000115594	1.12E-05
YLDs (Year	Guam	Male	55+ years	Non-melanoma skin cancer (basal-cell	Percent	2021	6.14E-09	1.47E-08	1.48E-09
YLDs (Year	Guam	Female	55+ years	Non-melanoma skin cancer (basal-cell	Percent	2021	5.81E-09	1.38E-08	1.42E-09
YLDs (Year	Guam	Both	55+ years	Non-melanoma skin cancer (basal-cell	Percent	2021	5.95E-09	1.41E-08	1.58E-09
YLDs (Year	Guam	Male	55+ years	Non-melanoma skin cancer (basal-cell	Rate	2021	0.0001151	0.000291713	2.61E-05
YLDs (Year	Guam	Female	55+ years	Non-melanoma skin cancer (basal-cell	Rate	2021	0.0001274	0.000317802	2.97E-05
YLDs (Year	Guam	Both	55+ years	Non-melanoma skin cancer (basal-cell	Rate	2021	0.0001215	0.000306734	2.97E-05
YLDs (Year	Monaco	Male	55+ years	Non-melanoma skin cancer (basal-cell	Number	1990	0.0055684	0.011839671	0.002209824
YLDs (Year	Monaco	Female	55+ years	Non-melanoma skin cancer (basal-cell	Number	1990	0.0058198	0.012246921	0.002332905
YLDs (Year	Monaco	Both	55+ years	Non-melanoma skin cancer (basal-cell	Number	1990	0.0113882	0.023434126	0.004684102
YLDs (Year	Monaco	Male	55+ years	Non-melanoma skin cancer (basal-cell	Percent	1990	5.96E-06	1.20E-05	2.56E-06
YLDs (Year	Monaco	Female	55+ years	Non-melanoma skin cancer (basal-cell	Percent	1990	4.25E-06	8.40E-06	1.74E-06
YLDs (Year	Monaco	Both	55+ years	Non-melanoma skin cancer (basal-cell	Percent	1990	4.94E-06	9.47E-06	2.17E-06
YLDs (Year	Monaco	Male	55+ years	Non-melanoma skin cancer (basal-cell	Rate	1990	0.1150884	0.244702133	0.045672615
YLDs (Year	Monaco	Female	55+ years	Non-melanoma skin cancer (basal-cell	Rate	1990	0.0932138	0.196154577	0.037365305
YLDs (Year	Monaco	Both	55+ years	Non-melanoma skin cancer (basal-cell	Rate	1990	0.1027643	0.211462954	0.042268018
YLDs (Year	Monaco	Male	55+ years	Non-melanoma skin cancer (basal-cell	Number	2021	0.0086052	0.016592013	0.003423305
YLDs (Year	Monaco	Female	55+ years	Non-melanoma skin cancer (basal-cell	Number	2021	0.0072794	0.015288501	0.002900131
YLDs (Year	Monaco	Both	55+ years	Non-melanoma skin cancer (basal-cell	Number	2021	0.0158846	0.029624868	0.006772452
YLDs (Year	Monaco	Male	55+ years	Non-melanoma skin cancer (basal-cell	Percent	2021	5.91E-06	1.10E-05	2.51E-06

YLDs (Year	Monaco	Female	55+ years	Non-melanoma skin cancer (basal-cell	Percent	2021	3.92E-06	7.55E-06	1.70E-06
YLDs (Year	Monaco	Both	55+ years	Non-melanoma skin cancer (basal-cell	Percent	2021	4.79E-06	8.92E-06	2.17E-06
YLDs (Year	Monaco	Male	55+ years	Non-melanoma skin cancer (basal-cell	Rate	2021	0.1163861	0.224408326	0.046300477
YLDs (Year	Monaco	Female	55+ years	Non-melanoma skin cancer (basal-cell	Rate	2021	0.0882166	0.185277348	0.035145935
YLDs (Year	Monaco	Both	55+ years	Non-melanoma skin cancer (basal-cell	Rate	2021	0.1015289	0.189352485	0.043287301
YLDs (Year	Sierra Leo	Male	55+ years	Non-melanoma skin cancer (basal-cell	Number	1990	0.0146307	0.028279425	0.005808519
YLDs (Year	Sierra Leo	Female	55+ years	Non-melanoma skin cancer (basal-cell	Number	1990	0.0078146	0.015491173	0.003023151
YLDs (Year	Sierra Leo	Both	55+ years	Non-melanoma skin cancer (basal-cell	Number	1990	0.0224453	0.043585929	0.008787619
YLDs (Year	Sierra Leo	Male	55+ years	Non-melanoma skin cancer (basal-cell	Percent	1990	3.99E-07	7.35E-07	1.72E-07
YLDs (Year	Sierra Leo	Female	55+ years	Non-melanoma skin cancer (basal-cell	Percent	1990	2.11E-07	3.93E-07	9.16E-08
YLDs (Year	Sierra Leo	Both	55+ years	Non-melanoma skin cancer (basal-cell	Percent	1990	3.04E-07	5.69E-07	1.35E-07
YLDs (Year	Sierra Leo	Male	55+ years	Non-melanoma skin cancer (basal-cell	Rate	1990	0.0084905	0.016411127	0.003370802
YLDs (Year	Sierra Leo	Female	55+ years	Non-melanoma skin cancer (basal-cell	Rate	1990	0.0049106	0.009734429	0.001899704
YLDs (Year	Sierra Leo	Both	55+ years	Non-melanoma skin cancer (basal-cell	Rate	1990	0.0067717	0.013149815	0.002651213
YLDs (Year	Sierra Leo	Male	55+ years	Non-melanoma skin cancer (basal-cell	Number	2021	0.0246096	0.048595999	0.010192791
YLDs (Year	Sierra Leo	Female	55+ years	Non-melanoma skin cancer (basal-cell	Number	2021	0.0142312	0.028281341	0.005601373
YLDs (Year	Sierra Leo	Both	55+ years	Non-melanoma skin cancer (basal-cell	Number	2021	0.0388407	0.077410384	0.015846933
YLDs (Year	Sierra Leo	Male	55+ years	Non-melanoma skin cancer (basal-cell	Percent	2021	4.01E-07	7.24E-07	1.79E-07
YLDs (Year	Sierra Leo	Female	55+ years	Non-melanoma skin cancer (basal-cell	Percent	2021	2.08E-07	3.85E-07	9.23E-08
YLDs (Year	Sierra Leo	Both	55+ years	Non-melanoma skin cancer (basal-cell	Percent	2021	2.99E-07	5.52E-07	1.31E-07
YLDs (Year	Sierra Leo	Male	55+ years	Non-melanoma skin cancer (basal-cell	Rate	2021	0.0079756	0.015749243	0.003303333
YLDs (Year	Sierra Leo	Female	55+ years	Non-melanoma skin cancer (basal-cell	Rate	2021	0.0048156	0.009569936	0.001895412
YLDs (Year	Sierra Leo	Both	55+ years	Non-melanoma skin cancer (basal-cell	Rate	2021	0.0064297	0.012814515	0.002623301
YLDs (Year	Palau	Male	55+ years	Non-melanoma skin cancer (basal-cell	Number	1990	7.81E-07	2.08E-06	1.63E-07
YLDs (Year	Palau	Female	55+ years	Non-melanoma skin cancer (basal-cell	Number	1990	8.59E-07	2.24E-06	1.98E-07
YLDs (Year	Palau	Both	55+ years	Non-melanoma skin cancer (basal-cell	Number	1990	1.64E-06	4.38E-06	4.07E-07
YLDs (Year	Palau	Male	55+ years	Non-melanoma skin cancer (basal-cell	Percent	1990	4.98E-09	1.22E-08	1.13E-09
YLDs (Year	Palau	Female	55+ years	Non-melanoma skin cancer (basal-cell	Percent	1990	4.76E-09	1.15E-08	1.19E-09
YLDs (Year	Palau	Both	55+ years	Non-melanoma skin cancer (basal-cell	Percent	1990	4.86E-09	1.17E-08	1.28E-09
YLDs (Year	Palau	Male	55+ years	Non-melanoma skin cancer (basal-cell	Rate	1990	9.88E-05	0.000263111	2.06E-05
YLDs (Year	Palau	Female	55+ years	Non-melanoma skin cancer (basal-cell	Rate	1990	0.0001061	0.000277218	2.44E-05
YLDs (Year	Palau	Both	55+ years	Non-melanoma skin cancer (basal-cell	Rate	1990	0.0001025	0.000273645	2.54E-05
YLDs (Year	Palau	Male	55+ years	Non-melanoma skin cancer (basal-cell	Number	2021	2.08E-06	5.65E-06	4.34E-07
YLDs (Year	Palau	Female	55+ years	Non-melanoma skin cancer (basal-cell	Number	2021	2.20E-06	5.92E-06	4.86E-07
YLDs (Year	Palau	Both	55+ years	Non-melanoma skin cancer (basal-cell	Number	2021	4.28E-06	1.17E-05	1.05E-06
YLDs (Year	Palau	Male	55+ years	Non-melanoma skin cancer (basal-cell	Percent	2021	4.75E-09	1.18E-08	1.08E-09
YLDs (Year	Palau	Female	55+ years	Non-melanoma skin cancer (basal-cell	Percent	2021	4.57E-09	1.16E-08	1.11E-09
YLDs (Year	Palau	Both	55+ years	Non-melanoma skin cancer (basal-cell	Percent	2021	4.65E-09	1.16E-08	1.21E-09
YLDs (Year	Palau	Male	55+ years	Non-melanoma skin cancer (basal-cell	Rate	2021	9.87E-05	0.000267452	2.05E-05
YLDs (Year	Palau	Female	55+ years	Non-melanoma skin cancer (basal-cell	Rate	2021	0.0001058	0.000285008	2.34E-05
YLDs (Year	Palau	Both	55+ years	Non-melanoma skin cancer (basal-cell	Rate	2021	0.0001022	0.000279189	2.51E-05
YLDs (Year	Saint Kitt	Male	55+ years	Non-melanoma skin cancer (basal-cell	Number	1990	0.0004069	0.000768593	0.00016416
YLDs (Year	Saint Kitt	Female	55+ years	Non-melanoma skin cancer (basal-cell	Number	1990	0.0002069	0.000408935	8.48E-05
YLDs (Year	Saint Kitt	Both	55+ years	Non-melanoma skin cancer (basal-cell	Number	1990	0.0006138	0.001173634	0.000242981
YLDs (Year	Saint Kitt	Male	55+ years	Non-melanoma skin cancer (basal-cell	Percent	1990	6.90E-07	1.26E-06	3.23E-07
YLDs (Year	Saint Kitt	Female	55+ years	Non-melanoma skin cancer (basal-cell	Percent	1990	2.58E-07	4.71E-07	1.15E-07
YLDs (Year	Saint Kitt	Both	55+ years	Non-melanoma skin cancer (basal-cell	Percent	1990	4.41E-07	8.04E-07	2.00E-07
YLDs (Year	Saint Kitt	Male	55+ years	Non-melanoma skin cancer (basal-cell	Rate	1990	0.0145637	0.027509821	0.005875688
YLDs (Year	Saint Kitt	Female	55+ years	Non-melanoma skin cancer (basal-cell	Rate	1990	0.0057451	0.011357278	0.002355994
YLDs (Year	Saint Kitt	Both	55+ years	Non-melanoma skin cancer (basal-cell	Rate	1990	0.0095981	0.01835371	0.003799827
YLDs (Year	Saint Kitt	Male	55+ years	Non-melanoma skin cancer (basal-cell	Number	2021	0.000726	0.001435971	0.000294805
YLDs (Year	Saint Kitt	Female	55+ years	Non-melanoma skin cancer (basal-cell	Number	2021	0.0003311	0.000644682	0.00013463
YLDs (Year	Saint Kitt	Both	55+ years	Non-melanoma skin cancer (basal-cell	Number	2021	0.0010571	0.002057803	0.000431797
YLDs (Year	Saint Kitt	Male	55+ years	Non-melanoma skin cancer (basal-cell	Percent	2021	5.72E-07	1.05E-06	2.53E-07
YLDs (Year	Saint Kitt	Female	55+ years	Non-melanoma skin cancer (basal-cell	Percent	2021	2.27E-07	4.25E-07	9.83E-08
YLDs (Year	Saint Kitt	Both	55+ years	Non-melanoma skin cancer (basal-cell	Percent	2021	3.88E-07	7.26E-07	1.71E-07

YLDs (Year	Saint Kitt	Male	55+ years	Non-melanoma skin cancer (basal-cell	Rate	2021	0.0119021	0.023540447	0.004832859
YLDs (Year	Saint Kitt	Female	55+ years	Non-melanoma skin cancer (basal-cell	Rate	2021	0.0048834	0.009509489	0.001985886
YLDs (Year	Saint Kitt	Both	55+ years	Non-melanoma skin cancer (basal-cell	Rate	2021	0.0082076	0.015977519	0.003352623
YLDs (Year	Greenland	Male	55+ years	Non-melanoma skin cancer (basal-cell	Number	1990	0.0030123	0.006099001	0.00120414
YLDs (Year	Greenland	Female	55+ years	Non-melanoma skin cancer (basal-cell	Number	1990	0.0029345	0.005890725	0.001191181
YLDs (Year	Greenland	Both	55+ years	Non-melanoma skin cancer (basal-cell	Number	1990	0.0059468	0.011555338	0.002462672
YLDs (Year	Greenland	Male	55+ years	Non-melanoma skin cancer (basal-cell	Percent	1990	5.88E-06	1.09E-05	2.63E-06
YLDs (Year	Greenland	Female	55+ years	Non-melanoma skin cancer (basal-cell	Percent	1990	4.53E-06	8.91E-06	1.91E-06
YLDs (Year	Greenland	Both	55+ years	Non-melanoma skin cancer (basal-cell	Percent	1990	5.13E-06	9.35E-06	2.30E-06
YLDs (Year	Greenland	Male	55+ years	Non-melanoma skin cancer (basal-cell	Rate	1990	0.1118289	0.226422814	0.044703179
YLDs (Year	Greenland	Female	55+ years	Non-melanoma skin cancer (basal-cell	Rate	1990	0.1041885	0.209144984	0.042291831
YLDs (Year	Greenland	Both	55+ years	Non-melanoma skin cancer (basal-cell	Rate	1990	0.1079235	0.209707824	0.0446929
YLDs (Year	Greenland	Male	55+ years	Non-melanoma skin cancer (basal-cell	Number	2021	0.0088829	0.018083593	0.003475579
YLDs (Year	Greenland	Female	55+ years	Non-melanoma skin cancer (basal-cell	Number	2021	0.0063866	0.013430669	0.002641487
YLDs (Year	Greenland	Both	55+ years	Non-melanoma skin cancer (basal-cell	Number	2021	0.0152696	0.030266488	0.006212231
YLDs (Year	Greenland	Male	55+ years	Non-melanoma skin cancer (basal-cell	Percent	2021	6.26E-06	1.18E-05	2.65E-06
YLDs (Year	Greenland	Female	55+ years	Non-melanoma skin cancer (basal-cell	Percent	2021	4.78E-06	9.33E-06	2.10E-06
YLDs (Year	Greenland	Both	55+ years	Non-melanoma skin cancer (basal-cell	Percent	2021	5.54E-06	1.02E-05	2.37E-06
YLDs (Year	Greenland	Male	55+ years	Non-melanoma skin cancer (basal-cell	Rate	2021	0.1208762	0.246076383	0.047294688
YLDs (Year	Greenland	Female	55+ years	Non-melanoma skin cancer (basal-cell	Rate	2021	0.1025601	0.215676675	0.042418379
YLDs (Year	Greenland	Both	55+ years	Non-melanoma skin cancer (basal-cell	Rate	2021	0.1124747	0.2229412	0.045758936
YLDs (Year	Nauru	Male	55+ years	Non-melanoma skin cancer (basal-cell	Number	1990	3.60E-07	9.49E-07	7.32E-08
YLDs (Year	Nauru	Female	55+ years	Non-melanoma skin cancer (basal-cell	Number	1990	2.92E-07	7.85E-07	6.39E-08
YLDs (Year	Nauru	Both	55+ years	Non-melanoma skin cancer (basal-cell	Number	1990	6.51E-07	1.74E-06	1.44E-07
YLDs (Year	Nauru	Male	55+ years	Non-melanoma skin cancer (basal-cell	Percent	1990	4.55E-09	1.13E-08	1.01E-09
YLDs (Year	Nauru	Female	55+ years	Non-melanoma skin cancer (basal-cell	Percent	1990	4.38E-09	1.10E-08	1.06E-09
YLDs (Year	Nauru	Both	55+ years	Non-melanoma skin cancer (basal-cell	Percent	1990	4.47E-09	1.11E-08	1.03E-09
YLDs (Year	Nauru	Male	55+ years	Non-melanoma skin cancer (basal-cell	Rate	1990	8.77E-05	0.00023118	1.78E-05
YLDs (Year	Nauru	Female	55+ years	Non-melanoma skin cancer (basal-cell	Rate	1990	9.34E-05	0.000251373	2.05E-05
YLDs (Year	Nauru	Both	55+ years	Non-melanoma skin cancer (basal-cell	Rate	1990	9.01E-05	0.000240252	1.99E-05
YLDs (Year	Nauru	Male	55+ years	Non-melanoma skin cancer (basal-cell	Number	2021	3.56E-07	9.35E-07	7.23E-08
YLDs (Year	Nauru	Female	55+ years	Non-melanoma skin cancer (basal-cell	Number	2021	4.88E-07	1.28E-06	1.09E-07
YLDs (Year	Nauru	Both	55+ years	Non-melanoma skin cancer (basal-cell	Number	2021	8.43E-07	2.24E-06	1.92E-07
YLDs (Year	Nauru	Male	55+ years	Non-melanoma skin cancer (basal-cell	Percent	2021	4.22E-09	1.04E-08	9.41E-10
YLDs (Year	Nauru	Female	55+ years	Non-melanoma skin cancer (basal-cell	Percent	2021	3.95E-09	9.92E-09	9.76E-10
YLDs (Year	Nauru	Both	55+ years	Non-melanoma skin cancer (basal-cell	Percent	2021	4.06E-09	1.01E-08	1.03E-09
YLDs (Year	Nauru	Male	55+ years	Non-melanoma skin cancer (basal-cell	Rate	2021	8.78E-05	0.000230649	1.78E-05
YLDs (Year	Nauru	Female	55+ years	Non-melanoma skin cancer (basal-cell	Rate	2021	9.39E-05	0.000246559	2.11E-05
YLDs (Year	Nauru	Both	55+ years	Non-melanoma skin cancer (basal-cell	Rate	2021	9.12E-05	0.000242689	2.08E-05
YLDs (Year	Northern M	Male	55+ years	Non-melanoma skin cancer (basal-cell	Number	1990	1.71E-06	4.60E-06	3.29E-07
YLDs (Year	Northern M	Female	55+ years	Non-melanoma skin cancer (basal-cell	Number	1990	1.15E-06	3.00E-06	2.68E-07
YLDs (Year	Northern M	Both	55+ years	Non-melanoma skin cancer (basal-cell	Number	1990	2.86E-06	7.68E-06	6.88E-07
YLDs (Year	Northern M	Male	55+ years	Non-melanoma skin cancer (basal-cell	Percent	1990	6.75E-09	1.62E-08	1.41E-09
YLDs (Year	Northern M	Female	55+ years	Non-melanoma skin cancer (basal-cell	Percent	1990	5.96E-09	1.49E-08	1.53E-09
YLDs (Year	Northern M	Both	55+ years	Non-melanoma skin cancer (basal-cell	Percent	1990	6.41E-09	1.55E-08	1.68E-09
YLDs (Year	Northern M	Male	55+ years	Non-melanoma skin cancer (basal-cell	Rate	1990	0.0001155	0.000309941	2.22E-05
YLDs (Year	Northern M	Female	55+ years	Non-melanoma skin cancer (basal-cell	Rate	1990	0.0001196	0.000313403	2.80E-05
YLDs (Year	Northern M	Both	55+ years	Non-melanoma skin cancer (basal-cell	Rate	1990	0.0001171	0.000314482	2.82E-05
YLDs (Year	Northern M	Male	55+ years	Non-melanoma skin cancer (basal-cell	Number	2021	5.55E-06	1.56E-05	1.10E-06
YLDs (Year	Northern M	Female	55+ years	Non-melanoma skin cancer (basal-cell	Number	2021	5.85E-06	1.55E-05	1.34E-06
YLDs (Year	Northern M	Both	55+ years	Non-melanoma skin cancer (basal-cell	Number	2021	1.14E-05	3.02E-05	2.76E-06
YLDs (Year	Northern M	Male	55+ years	Non-melanoma skin cancer (basal-cell	Percent	2021	6.09E-09	1.50E-08	1.27E-09
YLDs (Year	Northern M	Female	55+ years	Non-melanoma skin cancer (basal-cell	Percent	2021	5.85E-09	1.45E-08	1.50E-09
YLDs (Year	Northern M	Both	55+ years	Non-melanoma skin cancer (basal-cell	Percent	2021	5.96E-09	1.47E-08	1.53E-09
YLDs (Year	Northern M	Male	55+ years	Non-melanoma skin cancer (basal-cell	Rate	2021	0.0001146	0.000321119	2.28E-05
YLDs (Year	Northern M	Female	55+ years	Non-melanoma skin cancer (basal-cell	Rate	2021	0.00012	0.00031759	2.75E-05

YLDs (Year	Northern M	Both	55+ years	Non-melanoma skin cancer (basal-cell	Rate	2021	0.0001173	0.000310579	2.84E-05
YLDs (Year	San Marino	Male	55+ years	Non-melanoma skin cancer (basal-cell	Number	1990	0.0031637	0.006716615	0.001270595
YLDs (Year	San Marino	Female	55+ years	Non-melanoma skin cancer (basal-cell	Number	1990	0.0029383	0.00605264	0.001155296
YLDs (Year	San Marino	Both	55+ years	Non-melanoma skin cancer (basal-cell	Number	1990	0.006102	0.012630207	0.002631276
YLDs (Year	San Marino	Male	55+ years	Non-melanoma skin cancer (basal-cell	Percent	1990	5.94E-06	1.17E-05	2.53E-06
YLDs (Year	San Marino	Female	55+ years	Non-melanoma skin cancer (basal-cell	Percent	1990	4.28E-06	8.22E-06	1.79E-06
YLDs (Year	San Marino	Both	55+ years	Non-melanoma skin cancer (basal-cell	Percent	1990	5.00E-06	9.51E-06	2.29E-06
YLDs (Year	San Marino	Male	55+ years	Non-melanoma skin cancer (basal-cell	Rate	1990	0.1166648	0.247685426	0.046855141
YLDs (Year	San Marino	Female	55+ years	Non-melanoma skin cancer (basal-cell	Rate	1990	0.0929438	0.191454805	0.036543874
YLDs (Year	San Marino	Both	55+ years	Non-melanoma skin cancer (basal-cell	Rate	1990	0.1038963	0.215050108	0.044801814
YLDs (Year	San Marino	Male	55+ years	Non-melanoma skin cancer (basal-cell	Number	2021	0.0074564	0.015127437	0.003091823
YLDs (Year	San Marino	Female	55+ years	Non-melanoma skin cancer (basal-cell	Number	2021	0.0057304	0.011758791	0.002194109
YLDs (Year	San Marino	Both	55+ years	Non-melanoma skin cancer (basal-cell	Number	2021	0.0131867	0.025051848	0.005598667
YLDs (Year	San Marino	Male	55+ years	Non-melanoma skin cancer (basal-cell	Percent	2021	6.40E-06	1.29E-05	2.82E-06
YLDs (Year	San Marino	Female	55+ years	Non-melanoma skin cancer (basal-cell	Percent	2021	4.08E-06	7.74E-06	1.71E-06
YLDs (Year	San Marino	Both	55+ years	Non-melanoma skin cancer (basal-cell	Percent	2021	5.13E-06	9.62E-06	2.36E-06
YLDs (Year	San Marino	Male	55+ years	Non-melanoma skin cancer (basal-cell	Rate	2021	0.1321914	0.268189463	0.054813944
YLDs (Year	San Marino	Female	55+ years	Non-melanoma skin cancer (basal-cell	Rate	2021	0.092628	0.190074259	0.035466537
YLDs (Year	San Marino	Both	55+ years	Non-melanoma skin cancer (basal-cell	Rate	2021	0.1114967	0.211819164	0.047338025
YLDs (Year	United Sta	Male	55+ years	Non-melanoma skin cancer (basal-cell	Number	1990	0.0008775	0.001725944	0.000371272
YLDs (Year	United Sta	Female	55+ years	Non-melanoma skin cancer (basal-cell	Number	1990	0.0004284	0.000830586	0.000177355
YLDs (Year	United Sta	Both	55+ years	Non-melanoma skin cancer (basal-cell	Number	1990	0.0013059	0.002566154	0.000557026
YLDs (Year	United Sta	Male	55+ years	Non-melanoma skin cancer (basal-cell	Percent	1990	7.34E-07	1.34E-06	3.30E-07
YLDs (Year	United Sta	Female	55+ years	Non-melanoma skin cancer (basal-cell	Percent	1990	2.81E-07	5.31E-07	1.20E-07
YLDs (Year	United Sta	Both	55+ years	Non-melanoma skin cancer (basal-cell	Percent	1990	4.81E-07	9.03E-07	2.15E-07
YLDs (Year	United Sta	Male	55+ years	Non-melanoma skin cancer (basal-cell	Rate	1990	0.0134611	0.026475078	0.005695118
YLDs (Year	United Sta	Female	55+ years	Non-melanoma skin cancer (basal-cell	Rate	1990	0.0057516	0.011151729	0.002381224
YLDs (Year	United Sta	Both	55+ years	Non-melanoma skin cancer (basal-cell	Rate	1990	0.00935	0.018372751	0.003988108
YLDs (Year	United Sta	Male	55+ years	Non-melanoma skin cancer (basal-cell	Number	2021	0.0022784	0.004397186	0.000911781
YLDs (Year	United Sta	Female	55+ years	Non-melanoma skin cancer (basal-cell	Number	2021	0.0011092	0.002123488	0.0004626
YLDs (Year	United Sta	Both	55+ years	Non-melanoma skin cancer (basal-cell	Number	2021	0.0033876	0.006509282	0.00140289
YLDs (Year	United Sta	Male	55+ years	Non-melanoma skin cancer (basal-cell	Percent	2021	6.85E-07	1.23E-06	3.16E-07
YLDs (Year	United Sta	Female	55+ years	Non-melanoma skin cancer (basal-cell	Percent	2021	2.79E-07	5.21E-07	1.20E-07
YLDs (Year	United Sta	Both	55+ years	Non-melanoma skin cancer (basal-cell	Percent	2021	4.64E-07	8.35E-07	2.12E-07
YLDs (Year	United Sta	Male	55+ years	Non-melanoma skin cancer (basal-cell	Rate	2021	0.0152684	0.029467681	0.006110291
YLDs (Year	United Sta	Female	55+ years	Non-melanoma skin cancer (basal-cell	Rate	2021	0.0064239	0.012297821	0.002679072
YLDs (Year	United Sta	Both	55+ years	Non-melanoma skin cancer (basal-cell	Rate	2021	0.010524	0.020221914	0.004358257
YLDs (Year	Niue	Male	55+ years	Non-melanoma skin cancer (basal-cell	Number	1990	1.65E-07	4.27E-07	3.62E-08
YLDs (Year	Niue	Female	55+ years	Non-melanoma skin cancer (basal-cell	Number	1990	2.43E-07	6.06E-07	5.70E-08
YLDs (Year	Niue	Both	55+ years	Non-melanoma skin cancer (basal-cell	Number	1990	4.07E-07	1.03E-06	9.76E-08
YLDs (Year	Niue	Male	55+ years	Non-melanoma skin cancer (basal-cell	Percent	1990	5.13E-09	1.22E-08	1.16E-09
YLDs (Year	Niue	Female	55+ years	Non-melanoma skin cancer (basal-cell	Percent	1990	4.91E-09	1.11E-08	1.15E-09
YLDs (Year	Niue	Both	55+ years	Non-melanoma skin cancer (basal-cell	Percent	1990	5.00E-09	1.13E-08	1.28E-09
YLDs (Year	Niue	Male	55+ years	Non-melanoma skin cancer (basal-cell	Rate	1990	0.0001041	0.000270522	2.29E-05
YLDs (Year	Niue	Female	55+ years	Non-melanoma skin cancer (basal-cell	Rate	1990	0.0001178	0.000293925	2.76E-05
YLDs (Year	Niue	Both	55+ years	Non-melanoma skin cancer (basal-cell	Rate	1990	0.0001119	0.000283605	2.68E-05
YLDs (Year	Niue	Male	55+ years	Non-melanoma skin cancer (basal-cell	Number	2021	1.90E-07	4.99E-07	4.05E-08
YLDs (Year	Niue	Female	55+ years	Non-melanoma skin cancer (basal-cell	Number	2021	2.42E-07	6.33E-07	5.37E-08
YLDs (Year	Niue	Both	55+ years	Non-melanoma skin cancer (basal-cell	Number	2021	4.33E-07	1.13E-06	1.05E-07
YLDs (Year	Niue	Male	55+ years	Non-melanoma skin cancer (basal-cell	Percent	2021	4.95E-09	1.20E-08	1.06E-09
YLDs (Year	Niue	Female	55+ years	Non-melanoma skin cancer (basal-cell	Percent	2021	4.80E-09	1.15E-08	1.15E-09
YLDs (Year	Niue	Both	55+ years	Non-melanoma skin cancer (basal-cell	Percent	2021	4.86E-09	1.17E-08	1.27E-09
YLDs (Year	Niue	Male	55+ years	Non-melanoma skin cancer (basal-cell	Rate	2021	0.0001036	0.000271795	2.20E-05
YLDs (Year	Niue	Female	55+ years	Non-melanoma skin cancer (basal-cell	Rate	2021	0.0001168	0.000304793	2.59E-05
YLDs (Year	Niue	Both	55+ years	Non-melanoma skin cancer (basal-cell	Rate	2021	0.0001106	0.000288545	2.68E-05
YLDs (Year	Puerto Ric	Male	55+ years	Non-melanoma skin cancer (basal-cell	Number	1990	0.0488288	0.094571941	0.019785203

YLDs (Year	Puerto Ric	Female	55+ years	Non-melanoma skin cancer (basal-cell	Number	1990	0.0236009	0.046659633	0.009655716
YLDs (Year	Puerto Ric	Both	55+ years	Non-melanoma skin cancer (basal-cell	Number	1990	0.0724297	0.138340125	0.029223636
YLDs (Year	Puerto Ric	Male	55+ years	Non-melanoma skin cancer (basal-cell	Percent	1990	8.69E-07	1.57E-06	3.88E-07
YLDs (Year	Puerto Ric	Female	55+ years	Non-melanoma skin cancer (basal-cell	Percent	1990	3.48E-07	6.57E-07	1.57E-07
YLDs (Year	Puerto Ric	Both	55+ years	Non-melanoma skin cancer (basal-cell	Percent	1990	5.84E-07	1.09E-06	2.61E-07
YLDs (Year	Puerto Ric	Male	55+ years	Non-melanoma skin cancer (basal-cell	Rate	1990	0.0174144	0.033728424	0.007056255
YLDs (Year	Puerto Ric	Female	55+ years	Non-melanoma skin cancer (basal-cell	Rate	1990	0.0072313	0.014296409	0.00295849
YLDs (Year	Puerto Ric	Both	55+ years	Non-melanoma skin cancer (basal-cell	Rate	1990	0.011937	0.022799603	0.004816298
YLDs (Year	Puerto Ric	Male	55+ years	Non-melanoma skin cancer (basal-cell	Number	2021	0.0965279	0.189062513	0.038925517
YLDs (Year	Puerto Ric	Female	55+ years	Non-melanoma skin cancer (basal-cell	Number	2021	0.0517871	0.102639538	0.021156046
YLDs (Year	Puerto Ric	Both	55+ years	Non-melanoma skin cancer (basal-cell	Number	2021	0.148315	0.294823428	0.060795619
YLDs (Year	Puerto Ric	Male	55+ years	Non-melanoma skin cancer (basal-cell	Percent	2021	8.32E-07	1.52E-06	3.66E-07
YLDs (Year	Puerto Ric	Female	55+ years	Non-melanoma skin cancer (basal-cell	Percent	2021	3.45E-07	6.51E-07	1.57E-07
YLDs (Year	Puerto Ric	Both	55+ years	Non-melanoma skin cancer (basal-cell	Percent	2021	5.58E-07	1.04E-06	2.52E-07
YLDs (Year	Puerto Ric	Male	55+ years	Non-melanoma skin cancer (basal-cell	Rate	2021	0.0186087	0.036447571	0.007504082
YLDs (Year	Puerto Ric	Female	55+ years	Non-melanoma skin cancer (basal-cell	Rate	2021	0.007984	0.015823815	0.003261602
YLDs (Year	Puerto Ric	Both	55+ years	Non-melanoma skin cancer (basal-cell	Rate	2021	0.0127051	0.025255479	0.005207939
YLDs (Year	Tokelau	Male	55+ years	Non-melanoma skin cancer (basal-cell	Number	1990	1.29E-07	3.42E-07	2.81E-08
YLDs (Year	Tokelau	Female	55+ years	Non-melanoma skin cancer (basal-cell	Number	1990	1.20E-07	3.05E-07	2.73E-08
YLDs (Year	Tokelau	Both	55+ years	Non-melanoma skin cancer (basal-cell	Number	1990	2.49E-07	6.43E-07	5.84E-08
YLDs (Year	Tokelau	Male	55+ years	Non-melanoma skin cancer (basal-cell	Percent	1990	5.65E-09	1.38E-08	1.25E-09
YLDs (Year	Tokelau	Female	55+ years	Non-melanoma skin cancer (basal-cell	Percent	1990	4.48E-09	1.09E-08	1.07E-09
YLDs (Year	Tokelau	Both	55+ years	Non-melanoma skin cancer (basal-cell	Percent	1990	5.02E-09	1.19E-08	1.29E-09
YLDs (Year	Tokelau	Male	55+ years	Non-melanoma skin cancer (basal-cell	Rate	1990	0.0001104	0.000292938	2.41E-05
YLDs (Year	Tokelau	Female	55+ years	Non-melanoma skin cancer (basal-cell	Rate	1990	0.0001002	0.000254684	2.27E-05
YLDs (Year	Tokelau	Both	55+ years	Non-melanoma skin cancer (basal-cell	Rate	1990	0.0001052	0.000271566	2.47E-05
YLDs (Year	Tokelau	Male	55+ years	Non-melanoma skin cancer (basal-cell	Number	2021	1.36E-07	3.51E-07	3.02E-08
YLDs (Year	Tokelau	Female	55+ years	Non-melanoma skin cancer (basal-cell	Number	2021	1.32E-07	3.31E-07	3.03E-08
YLDs (Year	Tokelau	Both	55+ years	Non-melanoma skin cancer (basal-cell	Number	2021	2.68E-07	6.76E-07	6.80E-08
YLDs (Year	Tokelau	Male	55+ years	Non-melanoma skin cancer (basal-cell	Percent	2021	5.20E-09	1.25E-08	1.22E-09
YLDs (Year	Tokelau	Female	55+ years	Non-melanoma skin cancer (basal-cell	Percent	2021	4.12E-09	9.49E-09	1.01E-09
YLDs (Year	Tokelau	Both	55+ years	Non-melanoma skin cancer (basal-cell	Percent	2021	4.60E-09	1.06E-08	1.24E-09
YLDs (Year	Tokelau	Male	55+ years	Non-melanoma skin cancer (basal-cell	Rate	2021	0.0001117	0.000288091	2.48E-05
YLDs (Year	Tokelau	Female	55+ years	Non-melanoma skin cancer (basal-cell	Rate	2021	0.0001011	0.00025275	2.31E-05
YLDs (Year	Tokelau	Both	55+ years	Non-melanoma skin cancer (basal-cell	Rate	2021	0.0001062	0.000267528	2.69E-05
YLDs (Year	Tuvalu	Male	55+ years	Non-melanoma skin cancer (basal-cell	Number	1990	5.01E-07	1.35E-06	1.01E-07
YLDs (Year	Tuvalu	Female	55+ years	Non-melanoma skin cancer (basal-cell	Number	1990	6.47E-07	1.69E-06	1.38E-07
YLDs (Year	Tuvalu	Both	55+ years	Non-melanoma skin cancer (basal-cell	Number	1990	1.15E-06	3.07E-06	2.58E-07
YLDs (Year	Tuvalu	Male	55+ years	Non-melanoma skin cancer (basal-cell	Percent	1990	5.34E-09	1.27E-08	1.15E-09
YLDs (Year	Tuvalu	Female	55+ years	Non-melanoma skin cancer (basal-cell	Percent	1990	4.78E-09	1.21E-08	1.10E-09
YLDs (Year	Tuvalu	Both	55+ years	Non-melanoma skin cancer (basal-cell	Percent	1990	5.01E-09	1.22E-08	1.23E-09
YLDs (Year	Tuvalu	Male	55+ years	Non-melanoma skin cancer (basal-cell	Rate	1990	9.68E-05	0.000260452	1.95E-05
YLDs (Year	Tuvalu	Female	55+ years	Non-melanoma skin cancer (basal-cell	Rate	1990	0.0001004	0.0002621	2.14E-05
YLDs (Year	Tuvalu	Both	55+ years	Non-melanoma skin cancer (basal-cell	Rate	1990	9.88E-05	0.000263966	2.22E-05
YLDs (Year	Tuvalu	Male	55+ years	Non-melanoma skin cancer (basal-cell	Number	2021	8.20E-07	2.16E-06	1.70E-07
YLDs (Year	Tuvalu	Female	55+ years	Non-melanoma skin cancer (basal-cell	Number	2021	9.91E-07	2.56E-06	2.12E-07
YLDs (Year	Tuvalu	Both	55+ years	Non-melanoma skin cancer (basal-cell	Number	2021	1.81E-06	4.82E-06	4.20E-07
YLDs (Year	Tuvalu	Male	55+ years	Non-melanoma skin cancer (basal-cell	Percent	2021	5.03E-09	1.19E-08	1.15E-09
YLDs (Year	Tuvalu	Female	55+ years	Non-melanoma skin cancer (basal-cell	Percent	2021	4.40E-09	1.09E-08	1.03E-09
YLDs (Year	Tuvalu	Both	55+ years	Non-melanoma skin cancer (basal-cell	Percent	2021	4.66E-09	1.13E-08	1.19E-09
YLDs (Year	Tuvalu	Male	55+ years	Non-melanoma skin cancer (basal-cell	Rate	2021	9.75E-05	0.000256342	2.02E-05
YLDs (Year	Tuvalu	Female	55+ years	Non-melanoma skin cancer (basal-cell	Rate	2021	0.000101	0.000260338	2.16E-05
YLDs (Year	Tuvalu	Both	55+ years	Non-melanoma skin cancer (basal-cell	Rate	2021	9.94E-05	0.000264492	2.30E-05
YLDs (Year	Sudan	Male	55+ years	Non-melanoma skin cancer (basal-cell	Number	1990	0.1157678	0.224209392	0.047201714
YLDs (Year	Sudan	Female	55+ years	Non-melanoma skin cancer (basal-cell	Number	1990	0.060321	0.118774103	0.02535895
YLDs (Year	Sudan	Both	55+ years	Non-melanoma skin cancer (basal-cell	Number	1990	0.1760889	0.334592772	0.073156184

YLDs (Year	Sudan	Male	55+ years	Non-melanoma skin cancer (basal-cell	Percent	1990	7.24E-07	1.32E-06	3.38E-07
YLDs (Year	Sudan	Female	55+ years	Non-melanoma skin cancer (basal-cell	Percent	1990	3.88E-07	7.21E-07	1.76E-07
YLDs (Year	Sudan	Both	55+ years	Non-melanoma skin cancer (basal-cell	Percent	1990	5.58E-07	1.01E-06	2.55E-07
YLDs (Year	Sudan	Male	55+ years	Non-melanoma skin cancer (basal-cell	Rate	1990	0.0146044	0.028284664	0.005954633
YLDs (Year	Sudan	Female	55+ years	Non-melanoma skin cancer (basal-cell	Rate	1990	0.0086874	0.017105701	0.003652165
YLDs (Year	Sudan	Both	55+ years	Non-melanoma skin cancer (basal-cell	Rate	1990	0.0118415	0.022500547	0.004919575
YLDs (Year	Sudan	Male	55+ years	Non-melanoma skin cancer (basal-cell	Number	2021	0.2438281	0.483185912	0.10292663
YLDs (Year	Sudan	Female	55+ years	Non-melanoma skin cancer (basal-cell	Number	2021	0.1146935	0.21640245	0.046836741
YLDs (Year	Sudan	Both	55+ years	Non-melanoma skin cancer (basal-cell	Number	2021	0.3585216	0.701114797	0.151559261
YLDs (Year	Sudan	Male	55+ years	Non-melanoma skin cancer (basal-cell	Percent	2021	7.10E-07	1.32E-06	3.25E-07
YLDs (Year	Sudan	Female	55+ years	Non-melanoma skin cancer (basal-cell	Percent	2021	3.61E-07	6.65E-07	1.62E-07
YLDs (Year	Sudan	Both	55+ years	Non-melanoma skin cancer (basal-cell	Percent	2021	5.42E-07	1.01E-06	2.50E-07
YLDs (Year	Sudan	Male	55+ years	Non-melanoma skin cancer (basal-cell	Rate	2021	0.0142484	0.028235551	0.006014642
YLDs (Year	Sudan	Female	55+ years	Non-melanoma skin cancer (basal-cell	Rate	2021	0.0081774	0.015429023	0.003339358
YLDs (Year	Sudan	Both	55+ years	Non-melanoma skin cancer (basal-cell	Rate	2021	0.0115138	0.022516117	0.004867286
Prevalence	Democratic	Male	55+ years	Non-melanoma skin cancer (basal-cell	Number	1990	0.9899684	1.758978052	0.528013104
Prevalence	Democratic	Female	55+ years	Non-melanoma skin cancer (basal-cell	Number	1990	1.472907	2.511024752	0.755635217
Prevalence	Democratic	Both	55+ years	Non-melanoma skin cancer (basal-cell	Number	1990	2.4628754	4.156695213	1.27986378
Prevalence	Democratic	Male	55+ years	Non-melanoma skin cancer (basal-cell	Percent	1990	9.88E-07	1.76E-06	5.27E-07
Prevalence	Democratic	Female	55+ years	Non-melanoma skin cancer (basal-cell	Percent	1990	8.89E-07	1.52E-06	4.56E-07
Prevalence	Democratic	Both	55+ years	Non-melanoma skin cancer (basal-cell	Percent	1990	9.26E-07	1.56E-06	4.81E-07
Prevalence	Democratic	Male	55+ years	Non-melanoma skin cancer (basal-cell	Rate	1990	0.0986465	0.175275263	0.052614434
Prevalence	Democratic	Female	55+ years	Non-melanoma skin cancer (basal-cell	Rate	1990	0.0888458	0.151465164	0.045579962
Prevalence	Democratic	Both	55+ years	Non-melanoma skin cancer (basal-cell	Rate	1990	0.0925415	0.156185998	0.04809032
Prevalence	Democratic	Male	55+ years	Non-melanoma skin cancer (basal-cell	Number	2021	2.4040223	4.207432109	1.325559734
Prevalence	Democratic	Female	55+ years	Non-melanoma skin cancer (basal-cell	Number	2021	3.2097518	5.15545107	1.769161851
Prevalence	Democratic	Both	55+ years	Non-melanoma skin cancer (basal-cell	Number	2021	5.6137742	9.445274214	3.071485685
Prevalence	Democratic	Male	55+ years	Non-melanoma skin cancer (basal-cell	Percent	2021	1.02E-06	1.78E-06	5.62E-07
Prevalence	Democratic	Female	55+ years	Non-melanoma skin cancer (basal-cell	Percent	2021	9.81E-07	1.58E-06	5.41E-07
Prevalence	Democratic	Both	55+ years	Non-melanoma skin cancer (basal-cell	Percent	2021	9.97E-07	1.68E-06	5.45E-07
Prevalence	Democratic	Male	55+ years	Non-melanoma skin cancer (basal-cell	Rate	2021	0.1017312	0.178046293	0.056093834
Prevalence	Democratic	Female	55+ years	Non-melanoma skin cancer (basal-cell	Rate	2021	0.0980778	0.15753101	0.054058869
Prevalence	Democratic	Both	55+ years	Non-melanoma skin cancer (basal-cell	Rate	2021	0.0996097	0.16759512	0.054499848
Prevalence	Armenia	Male	55+ years	Non-melanoma skin cancer (basal-cell	Number	1990	26.373984	34.55426916	19.81688746
Prevalence	Armenia	Female	55+ years	Non-melanoma skin cancer (basal-cell	Number	1990	37.296749	48.73113206	28.0255899
Prevalence	Armenia	Both	55+ years	Non-melanoma skin cancer (basal-cell	Number	1990	63.670733	82.66036207	47.43899399
Prevalence	Armenia	Male	55+ years	Non-melanoma skin cancer (basal-cell	Percent	1990	0.000125	0.000163753	9.39E-05
Prevalence	Armenia	Female	55+ years	Non-melanoma skin cancer (basal-cell	Percent	1990	0.0001354	0.000176835	0.000101697
Prevalence	Armenia	Both	55+ years	Non-melanoma skin cancer (basal-cell	Percent	1990	0.0001309	0.000169876	9.75E-05
Prevalence	Armenia	Male	55+ years	Non-melanoma skin cancer (basal-cell	Rate	1990	12.492129	16.36674995	9.386337771
Prevalence	Armenia	Female	55+ years	Non-melanoma skin cancer (basal-cell	Rate	1990	13.530842	17.67910782	10.16736949
Prevalence	Armenia	Both	55+ years	Non-melanoma skin cancer (basal-cell	Rate	1990	13.080323	16.98149476	9.745723438
Prevalence	Armenia	Male	55+ years	Non-melanoma skin cancer (basal-cell	Number	2021	48.449166	63.38601285	36.12895866
Prevalence	Armenia	Female	55+ years	Non-melanoma skin cancer (basal-cell	Number	2021	65.015535	86.7549906	47.24677372
Prevalence	Armenia	Both	55+ years	Non-melanoma skin cancer (basal-cell	Number	2021	113.4647	149.5644319	84.32758541
Prevalence	Armenia	Male	55+ years	Non-melanoma skin cancer (basal-cell	Percent	2021	0.0001443	0.000188819	0.000107625
Prevalence	Armenia	Female	55+ years	Non-melanoma skin cancer (basal-cell	Percent	2021	0.0001443	0.000192563	0.000104885
Prevalence	Armenia	Both	55+ years	Non-melanoma skin cancer (basal-cell	Percent	2021	0.0001443	0.000190233	0.000107261
Prevalence	Armenia	Male	55+ years	Non-melanoma skin cancer (basal-cell	Rate	2021	14.421634	18.86781467	10.75433626
Prevalence	Armenia	Female	55+ years	Non-melanoma skin cancer (basal-cell	Rate	2021	14.426382	19.25017828	10.48364838
Prevalence	Armenia	Both	55+ years	Non-melanoma skin cancer (basal-cell	Rate	2021	14.424354	19.01358166	10.72025889
Prevalence	Viet Nam	Male	55+ years	Non-melanoma skin cancer (basal-cell	Number	1990	40.863356	51.8291974	32.10198807
Prevalence	Viet Nam	Female	55+ years	Non-melanoma skin cancer (basal-cell	Number	1990	65.929922	82.72155824	51.81406186
Prevalence	Viet Nam	Both	55+ years	Non-melanoma skin cancer (basal-cell	Number	1990	106.79328	134.3959266	84.4433974
Prevalence	Viet Nam	Male	55+ years	Non-melanoma skin cancer (basal-cell	Percent	1990	1.38E-05	1.75E-05	1.08E-05
Prevalence	Viet Nam	Female	55+ years	Non-melanoma skin cancer (basal-cell	Percent	1990	1.65E-05	2.07E-05	1.29E-05

Prevalence	Viet Nam	Both	55+ years	Non-melanoma skin cancer (basal-cell	Percent	1990	1.53E-05	1.93E-05	1.21E-05
Prevalence	Viet Nam	Male	55+ years	Non-melanoma skin cancer (basal-cell	Rate	1990	1.3768592	1.746344821	1.081651722
Prevalence	Viet Nam	Female	55+ years	Non-melanoma skin cancer (basal-cell	Rate	1990	1.6465093	2.06585741	1.293985098
Prevalence	Viet Nam	Both	55+ years	Non-melanoma skin cancer (basal-cell	Rate	1990	1.5317252	1.927627167	1.211163099
Prevalence	Viet Nam	Male	55+ years	Non-melanoma skin cancer (basal-cell	Number	2021	58.568299	85.68092033	40.15225583
Prevalence	Viet Nam	Female	55+ years	Non-melanoma skin cancer (basal-cell	Number	2021	83.486278	116.7413752	56.28655887
Prevalence	Viet Nam	Both	55+ years	Non-melanoma skin cancer (basal-cell	Number	2021	142.05458	201.1022943	98.78713211
Prevalence	Viet Nam	Male	55+ years	Non-melanoma skin cancer (basal-cell	Percent	2021	7.68E-06	1.12E-05	5.26E-06
Prevalence	Viet Nam	Female	55+ years	Non-melanoma skin cancer (basal-cell	Percent	2021	8.50E-06	1.19E-05	5.73E-06
Prevalence	Viet Nam	Both	55+ years	Non-melanoma skin cancer (basal-cell	Percent	2021	8.14E-06	1.15E-05	5.66E-06
Prevalence	Viet Nam	Male	55+ years	Non-melanoma skin cancer (basal-cell	Rate	2021	0.7667468	1.121691693	0.525653222
Prevalence	Viet Nam	Female	55+ years	Non-melanoma skin cancer (basal-cell	Rate	2021	0.8492968	1.187597279	0.572597026
Prevalence	Viet Nam	Both	55+ years	Non-melanoma skin cancer (basal-cell	Rate	2021	0.8131999	1.151222116	0.565512848
Prevalence	China	Male	55+ years	Non-melanoma skin cancer (basal-cell	Number	1990	1276.3246	1686.962936	978.9681643
Prevalence	China	Female	55+ years	Non-melanoma skin cancer (basal-cell	Number	1990	1327.5979	1745.420145	1015.385647
Prevalence	China	Both	55+ years	Non-melanoma skin cancer (basal-cell	Number	1990	2603.9225	3441.975358	1996.701656
Prevalence	China	Male	55+ years	Non-melanoma skin cancer (basal-cell	Percent	1990	1.81E-05	2.39E-05	1.39E-05
Prevalence	China	Female	55+ years	Non-melanoma skin cancer (basal-cell	Percent	1990	1.82E-05	2.40E-05	1.39E-05
Prevalence	China	Both	55+ years	Non-melanoma skin cancer (basal-cell	Percent	1990	1.82E-05	2.40E-05	1.39E-05
Prevalence	China	Male	55+ years	Non-melanoma skin cancer (basal-cell	Rate	1990	1.8067928	2.388101326	1.385848569
Prevalence	China	Female	55+ years	Non-melanoma skin cancer (basal-cell	Rate	1990	1.8216798	2.3949997	1.393273893
Prevalence	China	Both	55+ years	Non-melanoma skin cancer (basal-cell	Rate	1990	1.8143523	2.398287957	1.391255032
Prevalence	China	Male	55+ years	Non-melanoma skin cancer (basal-cell	Number	2021	31875.837	38516.18593	25964.6394
Prevalence	China	Female	55+ years	Non-melanoma skin cancer (basal-cell	Number	2021	26146.118	32255.51866	20943.82891
Prevalence	China	Both	55+ years	Non-melanoma skin cancer (basal-cell	Number	2021	58021.955	70493.10821	46923.98364
Prevalence	China	Male	55+ years	Non-melanoma skin cancer (basal-cell	Percent	2021	0.0001735	0.000209742	0.000141334
Prevalence	China	Female	55+ years	Non-melanoma skin cancer (basal-cell	Percent	2021	0.000134	0.000165304	0.000107348
Prevalence	China	Both	55+ years	Non-melanoma skin cancer (basal-cell	Percent	2021	0.0001532	0.00018612	0.000123885
Prevalence	China	Male	55+ years	Non-melanoma skin cancer (basal-cell	Rate	2021	17.343281	20.9562188	14.12706505
Prevalence	China	Female	55+ years	Non-melanoma skin cancer (basal-cell	Rate	2021	13.397167	16.52760004	10.731535
Prevalence	China	Both	55+ years	Non-melanoma skin cancer (basal-cell	Rate	2021	15.311036	18.60196724	12.38246445
Prevalence	Timor-Lest	Male	55+ years	Non-melanoma skin cancer (basal-cell	Number	1990	0.134524	0.199782691	0.086822036
Prevalence	Timor-Lest	Female	55+ years	Non-melanoma skin cancer (basal-cell	Number	1990	0.0911759	0.136583423	0.059760646
Prevalence	Timor-Lest	Both	55+ years	Non-melanoma skin cancer (basal-cell	Number	1990	0.2256999	0.332862142	0.147074035
Prevalence	Timor-Lest	Male	55+ years	Non-melanoma skin cancer (basal-cell	Percent	1990	6.07E-06	9.01E-06	3.92E-06
Prevalence	Timor-Lest	Female	55+ years	Non-melanoma skin cancer (basal-cell	Percent	1990	4.28E-06	6.41E-06	2.81E-06
Prevalence	Timor-Lest	Both	55+ years	Non-melanoma skin cancer (basal-cell	Percent	1990	5.19E-06	7.66E-06	3.38E-06
Prevalence	Timor-Lest	Male	55+ years	Non-melanoma skin cancer (basal-cell	Rate	1990	0.6068504	0.901238805	0.3916625
Prevalence	Timor-Lest	Female	55+ years	Non-melanoma skin cancer (basal-cell	Rate	1990	0.4281734	0.641412896	0.280643494
Prevalence	Timor-Lest	Both	55+ years	Non-melanoma skin cancer (basal-cell	Rate	1990	0.5193073	0.765874401	0.338399068
Prevalence	Timor-Lest	Male	55+ years	Non-melanoma skin cancer (basal-cell	Number	2021	0.4920262	0.70319512	0.34600419
Prevalence	Timor-Lest	Female	55+ years	Non-melanoma skin cancer (basal-cell	Number	2021	0.3637462	0.523898043	0.234154885
Prevalence	Timor-Lest	Both	55+ years	Non-melanoma skin cancer (basal-cell	Number	2021	0.8557724	1.226191022	0.58913215
Prevalence	Timor-Lest	Male	55+ years	Non-melanoma skin cancer (basal-cell	Percent	2021	6.97E-06	9.96E-06	4.90E-06
Prevalence	Timor-Lest	Female	55+ years	Non-melanoma skin cancer (basal-cell	Percent	2021	5.09E-06	7.34E-06	3.28E-06
Prevalence	Timor-Lest	Both	55+ years	Non-melanoma skin cancer (basal-cell	Percent	2021	6.03E-06	8.64E-06	4.15E-06
Prevalence	Timor-Lest	Male	55+ years	Non-melanoma skin cancer (basal-cell	Rate	2021	0.6968949	0.995989812	0.490072583
Prevalence	Timor-Lest	Female	55+ years	Non-melanoma skin cancer (basal-cell	Rate	2021	0.5092455	0.733458499	0.327817392
Prevalence	Timor-Lest	Both	55+ years	Non-melanoma skin cancer (basal-cell	Rate	2021	0.6025247	0.863325768	0.414790972
Prevalence	Thailand	Male	55+ years	Non-melanoma skin cancer (basal-cell	Number	1990	97.048868	118.2717668	79.99014284
Prevalence	Thailand	Female	55+ years	Non-melanoma skin cancer (basal-cell	Number	1990	111.73573	135.877647	90.84307669
Prevalence	Thailand	Both	55+ years	Non-melanoma skin cancer (basal-cell	Number	1990	208.7846	251.4724881	172.8081274
Prevalence	Thailand	Male	55+ years	Non-melanoma skin cancer (basal-cell	Percent	1990	3.51E-05	4.28E-05	2.89E-05
Prevalence	Thailand	Female	55+ years	Non-melanoma skin cancer (basal-cell	Percent	1990	3.53E-05	4.29E-05	2.87E-05
Prevalence	Thailand	Both	55+ years	Non-melanoma skin cancer (basal-cell	Percent	1990	3.52E-05	4.24E-05	2.91E-05
Prevalence	Thailand	Male	55+ years	Non-melanoma skin cancer (basal-cell	Rate	1990	3.5071332	4.274082204	2.89066829

Prevalence	Thailand	Female	55+ years	Non-melanoma skin cancer (basal-cell	Rate	1990	3.5280163	4.290288982	2.868338241
Prevalence	Thailand	Both	55+ years	Non-melanoma skin cancer (basal-cell	Rate	1990	3.5182784	4.237622174	2.91203049
Prevalence	Thailand	Male	55+ years	Non-melanoma skin cancer (basal-cell	Number	2021	153.02283	206.8324958	110.1136795
Prevalence	Thailand	Female	55+ years	Non-melanoma skin cancer (basal-cell	Number	2021	172.73014	231.9226049	121.3925299
Prevalence	Thailand	Both	55+ years	Non-melanoma skin cancer (basal-cell	Number	2021	325.75297	437.465608	240.276312
Prevalence	Thailand	Male	55+ years	Non-melanoma skin cancer (basal-cell	Percent	2021	1.72E-05	2.33E-05	1.24E-05
Prevalence	Thailand	Female	55+ years	Non-melanoma skin cancer (basal-cell	Percent	2021	1.64E-05	2.20E-05	1.15E-05
Prevalence	Thailand	Both	55+ years	Non-melanoma skin cancer (basal-cell	Percent	2021	1.68E-05	2.25E-05	1.24E-05
Prevalence	Thailand	Male	55+ years	Non-melanoma skin cancer (basal-cell	Rate	2021	1.7196835	2.324401043	1.237466824
Prevalence	Thailand	Female	55+ years	Non-melanoma skin cancer (basal-cell	Rate	2021	1.6388567	2.20047254	1.151767542
Prevalence	Thailand	Both	55+ years	Non-melanoma skin cancer (basal-cell	Rate	2021	1.6758576	2.250570601	1.236117295
Prevalence	Sri Lanka	Male	55+ years	Non-melanoma skin cancer (basal-cell	Number	1990	8.1004705	10.4218755	6.291612188
Prevalence	Sri Lanka	Female	55+ years	Non-melanoma skin cancer (basal-cell	Number	1990	4.2428772	5.709706549	3.08710384
Prevalence	Sri Lanka	Both	55+ years	Non-melanoma skin cancer (basal-cell	Number	1990	12.343348	15.74228422	9.559082697
Prevalence	Sri Lanka	Male	55+ years	Non-melanoma skin cancer (basal-cell	Percent	1990	9.04E-06	1.16E-05	7.02E-06
Prevalence	Sri Lanka	Female	55+ years	Non-melanoma skin cancer (basal-cell	Percent	1990	4.81E-06	6.47E-06	3.50E-06
Prevalence	Sri Lanka	Both	55+ years	Non-melanoma skin cancer (basal-cell	Percent	1990	6.94E-06	8.85E-06	5.37E-06
Prevalence	Sri Lanka	Male	55+ years	Non-melanoma skin cancer (basal-cell	Rate	1990	0.9031545	1.161977458	0.701477535
Prevalence	Sri Lanka	Female	55+ years	Non-melanoma skin cancer (basal-cell	Rate	1990	0.4805788	0.646722404	0.349667571
Prevalence	Sri Lanka	Both	55+ years	Non-melanoma skin cancer (basal-cell	Rate	1990	0.6935335	0.884508905	0.537094468
Prevalence	Sri Lanka	Male	55+ years	Non-melanoma skin cancer (basal-cell	Number	2021	15.342613	21.9488719	10.25657924
Prevalence	Sri Lanka	Female	55+ years	Non-melanoma skin cancer (basal-cell	Number	2021	11.021065	16.28580469	6.755806335
Prevalence	Sri Lanka	Both	55+ years	Non-melanoma skin cancer (basal-cell	Number	2021	26.363678	37.94659659	16.80530487
Prevalence	Sri Lanka	Male	55+ years	Non-melanoma skin cancer (basal-cell	Percent	2021	7.15E-06	1.02E-05	4.78E-06
Prevalence	Sri Lanka	Female	55+ years	Non-melanoma skin cancer (basal-cell	Percent	2021	4.13E-06	6.11E-06	2.53E-06
Prevalence	Sri Lanka	Both	55+ years	Non-melanoma skin cancer (basal-cell	Percent	2021	5.48E-06	7.88E-06	3.49E-06
Prevalence	Sri Lanka	Male	55+ years	Non-melanoma skin cancer (basal-cell	Rate	2021	0.7142083	1.021733807	0.477450221
Prevalence	Sri Lanka	Female	55+ years	Non-melanoma skin cancer (basal-cell	Rate	2021	0.4130671	0.610388295	0.253206101
Prevalence	Sri Lanka	Both	55+ years	Non-melanoma skin cancer (basal-cell	Rate	2021	0.547384	0.787877884	0.348925312
Prevalence	Vanuatu	Male	55+ years	Non-melanoma skin cancer (basal-cell	Number	1990	0.0011545	0.002596421	0.000270624
Prevalence	Vanuatu	Female	55+ years	Non-melanoma skin cancer (basal-cell	Number	1990	0.0010364	0.002258893	0.000283815
Prevalence	Vanuatu	Both	55+ years	Non-melanoma skin cancer (basal-cell	Number	1990	0.0021909	0.004833068	0.000544449
Prevalence	Vanuatu	Male	55+ years	Non-melanoma skin cancer (basal-cell	Percent	1990	2.11E-07	4.75E-07	4.95E-08
Prevalence	Vanuatu	Female	55+ years	Non-melanoma skin cancer (basal-cell	Percent	1990	2.31E-07	5.03E-07	6.31E-08
Prevalence	Vanuatu	Both	55+ years	Non-melanoma skin cancer (basal-cell	Percent	1990	2.20E-07	4.85E-07	5.46E-08
Prevalence	Vanuatu	Male	55+ years	Non-melanoma skin cancer (basal-cell	Rate	1990	0.0210996	0.047452167	0.004945928
Prevalence	Vanuatu	Female	55+ years	Non-melanoma skin cancer (basal-cell	Rate	1990	0.0230569	0.050253229	0.006313988
Prevalence	Vanuatu	Both	55+ years	Non-melanoma skin cancer (basal-cell	Rate	1990	0.0219824	0.048492263	0.005462692
Prevalence	Vanuatu	Male	55+ years	Non-melanoma skin cancer (basal-cell	Number	2021	0.0029659	0.006668608	0.000752616
Prevalence	Vanuatu	Female	55+ years	Non-melanoma skin cancer (basal-cell	Number	2021	0.0032542	0.007152418	0.000880462
Prevalence	Vanuatu	Both	55+ years	Non-melanoma skin cancer (basal-cell	Number	2021	0.0062201	0.01387342	0.002013796
Prevalence	Vanuatu	Male	55+ years	Non-melanoma skin cancer (basal-cell	Percent	2021	2.10E-07	4.73E-07	5.34E-08
Prevalence	Vanuatu	Female	55+ years	Non-melanoma skin cancer (basal-cell	Percent	2021	2.23E-07	4.91E-07	6.04E-08
Prevalence	Vanuatu	Both	55+ years	Non-melanoma skin cancer (basal-cell	Percent	2021	2.17E-07	4.84E-07	7.02E-08
Prevalence	Vanuatu	Male	55+ years	Non-melanoma skin cancer (basal-cell	Rate	2021	0.0210372	0.047300761	0.005338344
Prevalence	Vanuatu	Female	55+ years	Non-melanoma skin cancer (basal-cell	Rate	2021	0.0223251	0.049068482	0.006040326
Prevalence	Vanuatu	Both	55+ years	Non-melanoma skin cancer (basal-cell	Rate	2021	0.0216919	0.048382076	0.007022899
Prevalence	Tonga	Male	55+ years	Non-melanoma skin cancer (basal-cell	Number	1990	0.0011678	0.002602657	0.000308399
Prevalence	Tonga	Female	55+ years	Non-melanoma skin cancer (basal-cell	Number	1990	0.0013568	0.003020216	0.000432755
Prevalence	Tonga	Both	55+ years	Non-melanoma skin cancer (basal-cell	Number	1990	0.0025246	0.00555441	0.000892527
Prevalence	Tonga	Male	55+ years	Non-melanoma skin cancer (basal-cell	Percent	1990	2.50E-07	5.57E-07	6.61E-08
Prevalence	Tonga	Female	55+ years	Non-melanoma skin cancer (basal-cell	Percent	1990	2.82E-07	6.27E-07	8.98E-08
Prevalence	Tonga	Both	55+ years	Non-melanoma skin cancer (basal-cell	Percent	1990	2.66E-07	5.86E-07	9.41E-08
Prevalence	Tonga	Male	55+ years	Non-melanoma skin cancer (basal-cell	Rate	1990	0.0249965	0.055709286	0.006601213
Prevalence	Tonga	Female	55+ years	Non-melanoma skin cancer (basal-cell	Rate	1990	0.0281658	0.062695282	0.008983371
Prevalence	Tonga	Both	55+ years	Non-melanoma skin cancer (basal-cell	Rate	1990	0.0266055	0.058534333	0.009405762

Prevalence	Tonga	Male	55+ years	Non-melanoma skin cancer (basal-cell	Number	2021	0.0015805	0.003416782	0.000397399
Prevalence	Tonga	Female	55+ years	Non-melanoma skin cancer (basal-cell	Number	2021	0.0020125	0.004147387	0.000640885
Prevalence	Tonga	Both	55+ years	Non-melanoma skin cancer (basal-cell	Number	2021	0.003593	0.007585967	0.001296658
Prevalence	Tonga	Male	55+ years	Non-melanoma skin cancer (basal-cell	Percent	2021	2.49E-07	5.38E-07	6.25E-08
Prevalence	Tonga	Female	55+ years	Non-melanoma skin cancer (basal-cell	Percent	2021	2.85E-07	5.86E-07	9.06E-08
Prevalence	Tonga	Both	55+ years	Non-melanoma skin cancer (basal-cell	Percent	2021	2.68E-07	5.65E-07	9.66E-08
Prevalence	Tonga	Male	55+ years	Non-melanoma skin cancer (basal-cell	Rate	2021	0.0248578	0.053740187	0.006250417
Prevalence	Tonga	Female	55+ years	Non-melanoma skin cancer (basal-cell	Rate	2021	0.0284557	0.058640643	0.009061589
Prevalence	Tonga	Both	55+ years	Non-melanoma skin cancer (basal-cell	Rate	2021	0.0267525	0.056483085	0.009654568
Prevalence	Myanmar	Male	55+ years	Non-melanoma skin cancer (basal-cell	Number	1990	11.22967	17.24828825	7.488466131
Prevalence	Myanmar	Female	55+ years	Non-melanoma skin cancer (basal-cell	Number	1990	9.8532582	14.63042279	6.481985936
Prevalence	Myanmar	Both	55+ years	Non-melanoma skin cancer (basal-cell	Number	1990	21.082928	32.06592781	13.92055363
Prevalence	Myanmar	Male	55+ years	Non-melanoma skin cancer (basal-cell	Percent	1990	6.12E-06	9.40E-06	4.08E-06
Prevalence	Myanmar	Female	55+ years	Non-melanoma skin cancer (basal-cell	Percent	1990	4.74E-06	7.04E-06	3.12E-06
Prevalence	Myanmar	Both	55+ years	Non-melanoma skin cancer (basal-cell	Percent	1990	5.39E-06	8.19E-06	3.56E-06
Prevalence	Myanmar	Male	55+ years	Non-melanoma skin cancer (basal-cell	Rate	1990	0.6120591	0.940096421	0.408149499
Prevalence	Myanmar	Female	55+ years	Non-melanoma skin cancer (basal-cell	Rate	1990	0.4737676	0.703464864	0.311669008
Prevalence	Myanmar	Both	55+ years	Non-melanoma skin cancer (basal-cell	Rate	1990	0.5385852	0.819157263	0.355614928
Prevalence	Myanmar	Male	55+ years	Non-melanoma skin cancer (basal-cell	Number	2021	22.499329	32.51928228	14.95135414
Prevalence	Myanmar	Female	55+ years	Non-melanoma skin cancer (basal-cell	Number	2021	23.508132	34.46643752	14.9930274
Prevalence	Myanmar	Both	55+ years	Non-melanoma skin cancer (basal-cell	Number	2021	46.00746	65.77054885	30.48526226
Prevalence	Myanmar	Male	55+ years	Non-melanoma skin cancer (basal-cell	Percent	2021	6.18E-06	8.94E-06	4.11E-06
Prevalence	Myanmar	Female	55+ years	Non-melanoma skin cancer (basal-cell	Percent	2021	4.90E-06	7.18E-06	3.12E-06
Prevalence	Myanmar	Both	55+ years	Non-melanoma skin cancer (basal-cell	Percent	2021	5.45E-06	7.79E-06	3.61E-06
Prevalence	Myanmar	Male	55+ years	Non-melanoma skin cancer (basal-cell	Rate	2021	0.6178281	0.892974437	0.410561861
Prevalence	Myanmar	Female	55+ years	Non-melanoma skin cancer (basal-cell	Rate	2021	0.4897076	0.717984665	0.312325976
Prevalence	Myanmar	Both	55+ years	Non-melanoma skin cancer (basal-cell	Rate	2021	0.544975	0.779075916	0.361108947
Prevalence	Solomon Is	Male	55+ years	Non-melanoma skin cancer (basal-cell	Number	1990	0.0024441	0.005461368	0.000568933
Prevalence	Solomon Is	Female	55+ years	Non-melanoma skin cancer (basal-cell	Number	1990	0.001884	0.004230986	0.00044346
Prevalence	Solomon Is	Both	55+ years	Non-melanoma skin cancer (basal-cell	Number	1990	0.0043281	0.009648391	0.00107826
Prevalence	Solomon Is	Male	55+ years	Non-melanoma skin cancer (basal-cell	Percent	1990	1.92E-07	4.29E-07	4.47E-08
Prevalence	Solomon Is	Female	55+ years	Non-melanoma skin cancer (basal-cell	Percent	1990	1.91E-07	4.29E-07	4.49E-08
Prevalence	Solomon Is	Both	55+ years	Non-melanoma skin cancer (basal-cell	Percent	1990	1.91E-07	4.27E-07	4.77E-08
Prevalence	Solomon Is	Male	55+ years	Non-melanoma skin cancer (basal-cell	Rate	1990	0.0191842	0.042868105	0.004465747
Prevalence	Solomon Is	Female	55+ years	Non-melanoma skin cancer (basal-cell	Rate	1990	0.0190897	0.04287042	0.004493357
Prevalence	Solomon Is	Both	55+ years	Non-melanoma skin cancer (basal-cell	Rate	1990	0.0191429	0.042674666	0.004769124
Prevalence	Solomon Is	Male	55+ years	Non-melanoma skin cancer (basal-cell	Number	2021	0.0052723	0.011488062	0.001233829
Prevalence	Solomon Is	Female	55+ years	Non-melanoma skin cancer (basal-cell	Number	2021	0.0050423	0.010895632	0.001362941
Prevalence	Solomon Is	Both	55+ years	Non-melanoma skin cancer (basal-cell	Number	2021	0.0103146	0.022361052	0.00323535
Prevalence	Solomon Is	Male	55+ years	Non-melanoma skin cancer (basal-cell	Percent	2021	1.92E-07	4.19E-07	4.50E-08
Prevalence	Solomon Is	Female	55+ years	Non-melanoma skin cancer (basal-cell	Percent	2021	1.88E-07	4.07E-07	5.09E-08
Prevalence	Solomon Is	Both	55+ years	Non-melanoma skin cancer (basal-cell	Percent	2021	1.90E-07	4.12E-07	5.97E-08
Prevalence	Solomon Is	Male	55+ years	Non-melanoma skin cancer (basal-cell	Rate	2021	0.0192083	0.041854006	0.00449516
Prevalence	Solomon Is	Female	55+ years	Non-melanoma skin cancer (basal-cell	Rate	2021	0.0188161	0.040658705	0.00508602
Prevalence	Solomon Is	Both	55+ years	Non-melanoma skin cancer (basal-cell	Rate	2021	0.0190145	0.041221778	0.005964249
Prevalence	Philippine	Male	55+ years	Non-melanoma skin cancer (basal-cell	Number	1990	34.250546	46.81687564	25.01801829
Prevalence	Philippine	Female	55+ years	Non-melanoma skin cancer (basal-cell	Number	1990	34.598911	48.13512676	25.09232273
Prevalence	Philippine	Both	55+ years	Non-melanoma skin cancer (basal-cell	Number	1990	68.849457	95.08866406	50.51051022
Prevalence	Philippine	Male	55+ years	Non-melanoma skin cancer (basal-cell	Percent	1990	1.50E-05	2.05E-05	1.09E-05
Prevalence	Philippine	Female	55+ years	Non-melanoma skin cancer (basal-cell	Percent	1990	1.39E-05	1.94E-05	1.01E-05
Prevalence	Philippine	Both	55+ years	Non-melanoma skin cancer (basal-cell	Percent	1990	1.44E-05	1.99E-05	1.06E-05
Prevalence	Philippine	Male	55+ years	Non-melanoma skin cancer (basal-cell	Rate	1990	1.4962082	2.045158443	1.09289248
Prevalence	Philippine	Female	55+ years	Non-melanoma skin cancer (basal-cell	Rate	1990	1.3923863	1.937132918	1.00980651
Prevalence	Philippine	Both	55+ years	Non-melanoma skin cancer (basal-cell	Rate	1990	1.4421692	1.991794072	1.058028692
Prevalence	Philippine	Male	55+ years	Non-melanoma skin cancer (basal-cell	Number	2021	64.503899	90.38785091	44.8488912
Prevalence	Philippine	Female	55+ years	Non-melanoma skin cancer (basal-cell	Number	2021	72.433252	100.2526579	51.1541829

Prevalence	Philippine	Both	55+ years	Non-melanoma skin cancer (basal-cell	Number	2021	136.93715	193.3470966	97.3209457
Prevalence	Philippine	Male	55+ years	Non-melanoma skin cancer (basal-cell	Percent	2021	1.00E-05	1.40E-05	6.96E-06
Prevalence	Philippine	Female	55+ years	Non-melanoma skin cancer (basal-cell	Percent	2021	9.65E-06	1.34E-05	6.82E-06
Prevalence	Philippine	Both	55+ years	Non-melanoma skin cancer (basal-cell	Percent	2021	9.82E-06	1.39E-05	6.98E-06
Prevalence	Philippine	Male	55+ years	Non-melanoma skin cancer (basal-cell	Rate	2021	1.0014372	1.403291285	0.69628891
Prevalence	Philippine	Female	55+ years	Non-melanoma skin cancer (basal-cell	Rate	2021	0.9652697	1.336000333	0.681697691
Prevalence	Philippine	Both	55+ years	Non-melanoma skin cancer (basal-cell	Rate	2021	0.9819752	1.386490478	0.697887721
Prevalence	Samoa	Male	55+ years	Non-melanoma skin cancer (basal-cell	Number	1990	0.0021725	0.004709045	0.00068618
Prevalence	Samoa	Female	55+ years	Non-melanoma skin cancer (basal-cell	Number	1990	0.0022239	0.004447825	0.000870392
Prevalence	Samoa	Both	55+ years	Non-melanoma skin cancer (basal-cell	Number	1990	0.0043964	0.008883517	0.001688503
Prevalence	Samoa	Male	55+ years	Non-melanoma skin cancer (basal-cell	Percent	1990	3.03E-07	6.56E-07	9.56E-08
Prevalence	Samoa	Female	55+ years	Non-melanoma skin cancer (basal-cell	Percent	1990	3.07E-07	6.15E-07	1.20E-07
Prevalence	Samoa	Both	55+ years	Non-melanoma skin cancer (basal-cell	Percent	1990	3.05E-07	6.17E-07	1.17E-07
Prevalence	Samoa	Male	55+ years	Non-melanoma skin cancer (basal-cell	Rate	1990	0.0302684	0.065608935	0.009560223
Prevalence	Samoa	Female	55+ years	Non-melanoma skin cancer (basal-cell	Rate	1990	0.0307402	0.061480569	0.012031099
Prevalence	Samoa	Both	55+ years	Non-melanoma skin cancer (basal-cell	Rate	1990	0.0305053	0.061639874	0.01171598
Prevalence	Samoa	Male	55+ years	Non-melanoma skin cancer (basal-cell	Number	2021	0.0029217	0.006295118	0.00077524
Prevalence	Samoa	Female	55+ years	Non-melanoma skin cancer (basal-cell	Number	2021	0.0032139	0.006719662	0.000991545
Prevalence	Samoa	Both	55+ years	Non-melanoma skin cancer (basal-cell	Number	2021	0.0061355	0.012838069	0.002038067
Prevalence	Samoa	Male	55+ years	Non-melanoma skin cancer (basal-cell	Percent	2021	2.39E-07	5.15E-07	6.35E-08
Prevalence	Samoa	Female	55+ years	Non-melanoma skin cancer (basal-cell	Percent	2021	2.59E-07	5.42E-07	8.00E-08
Prevalence	Samoa	Both	55+ years	Non-melanoma skin cancer (basal-cell	Percent	2021	2.49E-07	5.22E-07	8.28E-08
Prevalence	Samoa	Male	55+ years	Non-melanoma skin cancer (basal-cell	Rate	2021	0.0239052	0.051507161	0.006343073
Prevalence	Samoa	Female	55+ years	Non-melanoma skin cancer (basal-cell	Rate	2021	0.0259188	0.054192127	0.007996527
Prevalence	Samoa	Both	55+ years	Non-melanoma skin cancer (basal-cell	Rate	2021	0.0249193	0.052141636	0.008277581
Prevalence	Malaysia	Male	55+ years	Non-melanoma skin cancer (basal-cell	Number	1990	13.897332	17.88078317	10.35765369
Prevalence	Malaysia	Female	55+ years	Non-melanoma skin cancer (basal-cell	Number	1990	13.28088	17.10660929	10.12848683
Prevalence	Malaysia	Both	55+ years	Non-melanoma skin cancer (basal-cell	Number	1990	27.178212	34.9421352	20.71759262
Prevalence	Malaysia	Male	55+ years	Non-melanoma skin cancer (basal-cell	Percent	1990	1.96E-05	2.52E-05	1.46E-05
Prevalence	Malaysia	Female	55+ years	Non-melanoma skin cancer (basal-cell	Percent	1990	1.73E-05	2.23E-05	1.32E-05
Prevalence	Malaysia	Both	55+ years	Non-melanoma skin cancer (basal-cell	Percent	1990	1.84E-05	2.37E-05	1.40E-05
Prevalence	Malaysia	Male	55+ years	Non-melanoma skin cancer (basal-cell	Rate	1990	1.9580354	2.519275521	1.459319939
Prevalence	Malaysia	Female	55+ years	Non-melanoma skin cancer (basal-cell	Rate	1990	1.7335059	2.232864639	1.322035226
Prevalence	Malaysia	Both	55+ years	Non-melanoma skin cancer (basal-cell	Rate	1990	1.8414829	2.367534107	1.403738119
Prevalence	Malaysia	Male	55+ years	Non-melanoma skin cancer (basal-cell	Number	2021	33.928097	46.46052737	23.68953153
Prevalence	Malaysia	Female	55+ years	Non-melanoma skin cancer (basal-cell	Number	2021	30.53631	41.68378273	20.87065918
Prevalence	Malaysia	Both	55+ years	Non-melanoma skin cancer (basal-cell	Number	2021	64.464407	87.91451153	45.76408166
Prevalence	Malaysia	Male	55+ years	Non-melanoma skin cancer (basal-cell	Percent	2021	1.40E-05	1.92E-05	9.77E-06
Prevalence	Malaysia	Female	55+ years	Non-melanoma skin cancer (basal-cell	Percent	2021	1.23E-05	1.68E-05	8.43E-06
Prevalence	Malaysia	Both	55+ years	Non-melanoma skin cancer (basal-cell	Percent	2021	1.32E-05	1.79E-05	9.34E-06
Prevalence	Malaysia	Male	55+ years	Non-melanoma skin cancer (basal-cell	Rate	2021	1.3983263	1.914843002	0.976349952
Prevalence	Malaysia	Female	55+ years	Non-melanoma skin cancer (basal-cell	Rate	2021	1.233066	1.683204559	0.842763933
Prevalence	Malaysia	Both	55+ years	Non-melanoma skin cancer (basal-cell	Rate	2021	1.3148515	1.793152747	0.933429388
Prevalence	Maldives	Male	55+ years	Non-melanoma skin cancer (basal-cell	Number	1990	0.0619439	0.088909106	0.041924866
Prevalence	Maldives	Female	55+ years	Non-melanoma skin cancer (basal-cell	Number	1990	0.0284907	0.043817281	0.017765135
Prevalence	Maldives	Both	55+ years	Non-melanoma skin cancer (basal-cell	Number	1990	0.0904346	0.131758283	0.060157499
Prevalence	Maldives	Male	55+ years	Non-melanoma skin cancer (basal-cell	Percent	1990	6.78E-06	9.73E-06	4.59E-06
Prevalence	Maldives	Female	55+ years	Non-melanoma skin cancer (basal-cell	Percent	1990	4.60E-06	7.08E-06	2.87E-06
Prevalence	Maldives	Both	55+ years	Non-melanoma skin cancer (basal-cell	Percent	1990	5.90E-06	8.60E-06	3.93E-06
Prevalence	Maldives	Male	55+ years	Non-melanoma skin cancer (basal-cell	Rate	1990	0.6776439	0.972633897	0.458643079
Prevalence	Maldives	Female	55+ years	Non-melanoma skin cancer (basal-cell	Rate	1990	0.4603618	0.708013655	0.287054734
Prevalence	Maldives	Both	55+ years	Non-melanoma skin cancer (basal-cell	Rate	1990	0.5899256	0.859489632	0.39242122
Prevalence	Maldives	Male	55+ years	Non-melanoma skin cancer (basal-cell	Number	2021	0.2050039	0.294549567	0.138277977
Prevalence	Maldives	Female	55+ years	Non-melanoma skin cancer (basal-cell	Number	2021	0.1299827	0.183263834	0.085531056
Prevalence	Maldives	Both	55+ years	Non-melanoma skin cancer (basal-cell	Number	2021	0.3349867	0.478056428	0.224104288
Prevalence	Maldives	Male	55+ years	Non-melanoma skin cancer (basal-cell	Percent	2021	6.99E-06	1.00E-05	4.72E-06

Prevalence	Maldives	Female	55+ years	Non-melanoma skin cancer (basal-cell	Percent	2021	5.19E-06	7.32E-06	3.42E-06
Prevalence	Maldives	Both	55+ years	Non-melanoma skin cancer (basal-cell	Percent	2021	6.16E-06	8.80E-06	4.12E-06
Prevalence	Maldives	Male	55+ years	Non-melanoma skin cancer (basal-cell	Rate	2021	0.6984277	1.003500676	0.471099127
Prevalence	Maldives	Female	55+ years	Non-melanoma skin cancer (basal-cell	Rate	2021	0.5190134	0.731761633	0.341520442
Prevalence	Maldives	Both	55+ years	Non-melanoma skin cancer (basal-cell	Rate	2021	0.6158251	0.878838319	0.411983657
Prevalence	Turkmenist	Male	55+ years	Non-melanoma skin cancer (basal-cell	Number	1990	16.121798	20.94459191	12.10272237
Prevalence	Turkmenist	Female	55+ years	Non-melanoma skin cancer (basal-cell	Number	1990	24.035807	31.02170003	17.93532716
Prevalence	Turkmenist	Both	55+ years	Non-melanoma skin cancer (basal-cell	Number	1990	40.157605	51.79195531	30.0271966
Prevalence	Turkmenist	Male	55+ years	Non-melanoma skin cancer (basal-cell	Percent	1990	0.0001193	0.000154921	8.95E-05
Prevalence	Turkmenist	Female	55+ years	Non-melanoma skin cancer (basal-cell	Percent	1990	0.0001269	0.000163731	9.47E-05
Prevalence	Turkmenist	Both	55+ years	Non-melanoma skin cancer (basal-cell	Percent	1990	0.0001237	0.000159539	9.25E-05
Prevalence	Turkmenist	Male	55+ years	Non-melanoma skin cancer (basal-cell	Rate	1990	11.919074	15.48463415	8.947714467
Prevalence	Turkmenist	Female	55+ years	Non-melanoma skin cancer (basal-cell	Rate	1990	12.683101	16.36938431	9.464028816
Prevalence	Turkmenist	Both	55+ years	Non-melanoma skin cancer (basal-cell	Rate	1990	12.364899	15.94722404	9.245652701
Prevalence	Turkmenist	Male	55+ years	Non-melanoma skin cancer (basal-cell	Number	2021	36.160243	46.64491542	26.90294235
Prevalence	Turkmenist	Female	55+ years	Non-melanoma skin cancer (basal-cell	Number	2021	48.161878	62.99688571	36.08082579
Prevalence	Turkmenist	Both	55+ years	Non-melanoma skin cancer (basal-cell	Number	2021	84.322121	108.5477791	63.83490809
Prevalence	Turkmenist	Male	55+ years	Non-melanoma skin cancer (basal-cell	Percent	2021	0.0001173	0.000151316	8.73E-05
Prevalence	Turkmenist	Female	55+ years	Non-melanoma skin cancer (basal-cell	Percent	2021	0.0001207	0.000157835	9.04E-05
Prevalence	Turkmenist	Both	55+ years	Non-melanoma skin cancer (basal-cell	Percent	2021	0.0001192	0.000153474	9.02E-05
Prevalence	Turkmenist	Male	55+ years	Non-melanoma skin cancer (basal-cell	Rate	2021	11.724227	15.1236702	8.722734807
Prevalence	Turkmenist	Female	55+ years	Non-melanoma skin cancer (basal-cell	Rate	2021	12.062248	15.7777085	9.03652213
Prevalence	Turkmenist	Both	55+ years	Non-melanoma skin cancer (basal-cell	Rate	2021	11.914935	15.33808385	9.020038742
Prevalence	Tajikistan	Male	55+ years	Non-melanoma skin cancer (basal-cell	Number	1990	25.656144	34.11694887	18.87896566
Prevalence	Tajikistan	Female	55+ years	Non-melanoma skin cancer (basal-cell	Number	1990	32.685634	41.99983655	24.33113412
Prevalence	Tajikistan	Both	55+ years	Non-melanoma skin cancer (basal-cell	Number	1990	58.341778	75.55503058	44.00781586
Prevalence	Tajikistan	Male	55+ years	Non-melanoma skin cancer (basal-cell	Percent	1990	0.000125	0.000166236	9.20E-05
Prevalence	Tajikistan	Female	55+ years	Non-melanoma skin cancer (basal-cell	Percent	1990	0.0001243	0.000159747	9.25E-05
Prevalence	Tajikistan	Both	55+ years	Non-melanoma skin cancer (basal-cell	Percent	1990	0.0001246	0.000161388	9.40E-05
Prevalence	Tajikistan	Male	55+ years	Non-melanoma skin cancer (basal-cell	Rate	1990	12.493423	16.61346641	9.193233044
Prevalence	Tajikistan	Female	55+ years	Non-melanoma skin cancer (basal-cell	Rate	1990	12.429931	15.97200342	9.25282071
Prevalence	Tajikistan	Both	55+ years	Non-melanoma skin cancer (basal-cell	Rate	1990	12.457772	16.13333361	9.397028486
Prevalence	Tajikistan	Male	55+ years	Non-melanoma skin cancer (basal-cell	Number	2021	58.873138	77.73834273	43.32700062
Prevalence	Tajikistan	Female	55+ years	Non-melanoma skin cancer (basal-cell	Number	2021	57.469689	75.98670094	42.36145857
Prevalence	Tajikistan	Both	55+ years	Non-melanoma skin cancer (basal-cell	Number	2021	116.34283	150.6227133	86.54942199
Prevalence	Tajikistan	Male	55+ years	Non-melanoma skin cancer (basal-cell	Percent	2021	0.0001183	0.00015617	8.71E-05
Prevalence	Tajikistan	Female	55+ years	Non-melanoma skin cancer (basal-cell	Percent	2021	0.0001087	0.000143696	8.01E-05
Prevalence	Tajikistan	Both	55+ years	Non-melanoma skin cancer (basal-cell	Percent	2021	0.0001133	0.000146744	8.43E-05
Prevalence	Tajikistan	Male	55+ years	Non-melanoma skin cancer (basal-cell	Rate	2021	11.819537	15.60696882	8.698450777
Prevalence	Tajikistan	Female	55+ years	Non-melanoma skin cancer (basal-cell	Rate	2021	10.862916	14.36300044	8.007159679
Prevalence	Tajikistan	Both	55+ years	Non-melanoma skin cancer (basal-cell	Rate	2021	11.326817	14.66421192	8.426213008
Prevalence	Papua New	Male	55+ years	Non-melanoma skin cancer (basal-cell	Number	1990	0.0370878	0.082936071	0.008527368
Prevalence	Papua New	Female	55+ years	Non-melanoma skin cancer (basal-cell	Number	1990	0.0331283	0.073617884	0.008935268
Prevalence	Papua New	Both	55+ years	Non-melanoma skin cancer (basal-cell	Number	1990	0.0702161	0.153929465	0.021657607
Prevalence	Papua New	Male	55+ years	Non-melanoma skin cancer (basal-cell	Percent	1990	2.39E-07	5.35E-07	5.50E-08
Prevalence	Papua New	Female	55+ years	Non-melanoma skin cancer (basal-cell	Percent	1990	2.35E-07	5.23E-07	6.34E-08
Prevalence	Papua New	Both	55+ years	Non-melanoma skin cancer (basal-cell	Percent	1990	2.37E-07	5.20E-07	7.32E-08
Prevalence	Papua New	Male	55+ years	Non-melanoma skin cancer (basal-cell	Rate	1990	0.0239272	0.053506364	0.005501448
Prevalence	Papua New	Female	55+ years	Non-melanoma skin cancer (basal-cell	Rate	1990	0.0235165	0.052258478	0.0063428
Prevalence	Papua New	Both	55+ years	Non-melanoma skin cancer (basal-cell	Rate	1990	0.0237317	0.052025191	0.007319854
Prevalence	Papua New	Male	55+ years	Non-melanoma skin cancer (basal-cell	Number	2021	0.1058259	0.238185011	0.02334087
Prevalence	Papua New	Female	55+ years	Non-melanoma skin cancer (basal-cell	Number	2021	0.0896516	0.199755785	0.025745453
Prevalence	Papua New	Both	55+ years	Non-melanoma skin cancer (basal-cell	Number	2021	0.1954776	0.428625921	0.065113886
Prevalence	Papua New	Male	55+ years	Non-melanoma skin cancer (basal-cell	Percent	2021	2.38E-07	5.36E-07	5.25E-08
Prevalence	Papua New	Female	55+ years	Non-melanoma skin cancer (basal-cell	Percent	2021	2.38E-07	5.30E-07	6.83E-08
Prevalence	Papua New	Both	55+ years	Non-melanoma skin cancer (basal-cell	Percent	2021	2.38E-07	5.22E-07	7.93E-08

Prevalence	Papua New	Male	55+ years	Non-melanoma skin cancer (basal-cell	Rate	2021	0.0238047	0.053577905	0.005250351
Prevalence	Papua New	Female	55+ years	Non-melanoma skin cancer (basal-cell	Rate	2021	0.0237964	0.053021531	0.006833661
Prevalence	Papua New	Both	55+ years	Non-melanoma skin cancer (basal-cell	Rate	2021	0.0238009	0.052188529	0.00792812
Prevalence	Bulgaria	Male	55+ years	Non-melanoma skin cancer (basal-cell	Number	1990	208.72899	271.0430793	158.2070071
Prevalence	Bulgaria	Female	55+ years	Non-melanoma skin cancer (basal-cell	Number	1990	207.00849	273.2337485	153.3690808
Prevalence	Bulgaria	Both	55+ years	Non-melanoma skin cancer (basal-cell	Number	1990	415.73748	536.3160595	310.7910699
Prevalence	Bulgaria	Male	55+ years	Non-melanoma skin cancer (basal-cell	Percent	1990	0.0001993	0.000258762	0.000151047
Prevalence	Bulgaria	Female	55+ years	Non-melanoma skin cancer (basal-cell	Percent	1990	0.0001703	0.000224824	0.000126189
Prevalence	Bulgaria	Both	55+ years	Non-melanoma skin cancer (basal-cell	Percent	1990	0.0001837	0.000237022	0.000137347
Prevalence	Bulgaria	Male	55+ years	Non-melanoma skin cancer (basal-cell	Rate	1990	19.923272	25.87117888	15.1009271
Prevalence	Bulgaria	Female	55+ years	Non-melanoma skin cancer (basal-cell	Rate	1990	17.028968	22.47680168	12.61647375
Prevalence	Bulgaria	Both	55+ years	Non-melanoma skin cancer (basal-cell	Rate	1990	18.368725	23.69630556	13.73182851
Prevalence	Bulgaria	Male	55+ years	Non-melanoma skin cancer (basal-cell	Number	2021	294.66646	383.3835396	224.8723669
Prevalence	Bulgaria	Female	55+ years	Non-melanoma skin cancer (basal-cell	Number	2021	374.67267	461.2451692	288.4005831
Prevalence	Bulgaria	Both	55+ years	Non-melanoma skin cancer (basal-cell	Number	2021	669.33913	829.2784963	521.1891199
Prevalence	Bulgaria	Male	55+ years	Non-melanoma skin cancer (basal-cell	Percent	2021	0.0002885	0.000375415	0.00022019
Prevalence	Bulgaria	Female	55+ years	Non-melanoma skin cancer (basal-cell	Percent	2021	0.0002765	0.000340428	0.000212839
Prevalence	Bulgaria	Both	55+ years	Non-melanoma skin cancer (basal-cell	Percent	2021	0.0002817	0.000348985	0.000219352
Prevalence	Bulgaria	Male	55+ years	Non-melanoma skin cancer (basal-cell	Rate	2021	28.844336	37.52868161	22.01232601
Prevalence	Bulgaria	Female	55+ years	Non-melanoma skin cancer (basal-cell	Rate	2021	27.644326	34.03187082	21.27894674
Prevalence	Bulgaria	Both	55+ years	Non-melanoma skin cancer (basal-cell	Rate	2021	28.16008	34.88896441	21.92719181
Prevalence	Micronesia	Male	55+ years	Non-melanoma skin cancer (basal-cell	Number	1990	0.0008115	0.001772394	0.000201604
Prevalence	Micronesia	Female	55+ years	Non-melanoma skin cancer (basal-cell	Number	1990	0.0008718	0.001866863	0.000245207
Prevalence	Micronesia	Both	55+ years	Non-melanoma skin cancer (basal-cell	Number	1990	0.0016833	0.00358161	0.000574194
Prevalence	Micronesia	Male	55+ years	Non-melanoma skin cancer (basal-cell	Percent	1990	2.08E-07	4.54E-07	5.16E-08
Prevalence	Micronesia	Female	55+ years	Non-melanoma skin cancer (basal-cell	Percent	1990	2.15E-07	4.60E-07	6.05E-08
Prevalence	Micronesia	Both	55+ years	Non-melanoma skin cancer (basal-cell	Percent	1990	2.11E-07	4.50E-07	7.21E-08
Prevalence	Micronesia	Male	55+ years	Non-melanoma skin cancer (basal-cell	Rate	1990	0.02078	0.045384084	0.005162288
Prevalence	Micronesia	Female	55+ years	Non-melanoma skin cancer (basal-cell	Rate	1990	0.0214933	0.046028059	0.006045657
Prevalence	Micronesia	Both	55+ years	Non-melanoma skin cancer (basal-cell	Rate	1990	0.0211434	0.04498807	0.00721237
Prevalence	Micronesia	Male	55+ years	Non-melanoma skin cancer (basal-cell	Number	2021	0.0012985	0.00308987	0.000295768
Prevalence	Micronesia	Female	55+ years	Non-melanoma skin cancer (basal-cell	Number	2021	0.0014467	0.003166412	0.000402274
Prevalence	Micronesia	Both	55+ years	Non-melanoma skin cancer (basal-cell	Number	2021	0.0027453	0.006139675	0.000843045
Prevalence	Micronesia	Male	55+ years	Non-melanoma skin cancer (basal-cell	Percent	2021	2.06E-07	4.89E-07	4.68E-08
Prevalence	Micronesia	Female	55+ years	Non-melanoma skin cancer (basal-cell	Percent	2021	2.11E-07	4.63E-07	5.88E-08
Prevalence	Micronesia	Both	55+ years	Non-melanoma skin cancer (basal-cell	Percent	2021	2.09E-07	4.67E-07	6.41E-08
Prevalence	Micronesia	Male	55+ years	Non-melanoma skin cancer (basal-cell	Rate	2021	0.0205594	0.04892226	0.00468293
Prevalence	Micronesia	Female	55+ years	Non-melanoma skin cancer (basal-cell	Rate	2021	0.0211411	0.046270347	0.005878381
Prevalence	Micronesia	Both	55+ years	Non-melanoma skin cancer (basal-cell	Rate	2021	0.0208619	0.046657025	0.006406524
Prevalence	Kyrgyzstan	Male	55+ years	Non-melanoma skin cancer (basal-cell	Number	1990	26.884244	32.46993083	22.13865919
Prevalence	Kyrgyzstan	Female	55+ years	Non-melanoma skin cancer (basal-cell	Number	1990	46.082687	55.1566163	38.09268031
Prevalence	Kyrgyzstan	Both	55+ years	Non-melanoma skin cancer (basal-cell	Number	1990	72.96693	87.44097691	60.72508513
Prevalence	Kyrgyzstan	Male	55+ years	Non-melanoma skin cancer (basal-cell	Percent	1990	0.0001322	0.000159642	0.000108859
Prevalence	Kyrgyzstan	Female	55+ years	Non-melanoma skin cancer (basal-cell	Percent	1990	0.000148	0.000177121	0.000122325
Prevalence	Kyrgyzstan	Both	55+ years	Non-melanoma skin cancer (basal-cell	Percent	1990	0.0001418	0.000169895	0.000117961
Prevalence	Kyrgyzstan	Male	55+ years	Non-melanoma skin cancer (basal-cell	Rate	1990	13.213733	15.95912498	10.88125597
Prevalence	Kyrgyzstan	Female	55+ years	Non-melanoma skin cancer (basal-cell	Rate	1990	14.795705	17.70905913	12.23036461
Prevalence	Kyrgyzstan	Both	55+ years	Non-melanoma skin cancer (basal-cell	Rate	1990	14.170627	16.98157628	11.79318555
Prevalence	Kyrgyzstan	Male	55+ years	Non-melanoma skin cancer (basal-cell	Number	2021	43.356242	56.63018367	31.46983939
Prevalence	Kyrgyzstan	Female	55+ years	Non-melanoma skin cancer (basal-cell	Number	2021	60.491366	79.34606087	44.36521674
Prevalence	Kyrgyzstan	Both	55+ years	Non-melanoma skin cancer (basal-cell	Number	2021	103.84761	135.3128221	76.02952657
Prevalence	Kyrgyzstan	Male	55+ years	Non-melanoma skin cancer (basal-cell	Percent	2021	0.0001188	0.000155228	8.62E-05
Prevalence	Kyrgyzstan	Female	55+ years	Non-melanoma skin cancer (basal-cell	Percent	2021	0.0001259	0.000165185	9.24E-05
Prevalence	Kyrgyzstan	Both	55+ years	Non-melanoma skin cancer (basal-cell	Percent	2021	0.0001229	0.000160085	8.99E-05
Prevalence	Kyrgyzstan	Male	55+ years	Non-melanoma skin cancer (basal-cell	Rate	2021	11.870155	15.50432046	8.615873075
Prevalence	Kyrgyzstan	Female	55+ years	Non-melanoma skin cancer (basal-cell	Rate	2021	12.589354	16.51335968	9.233209223

Prevalence	Kyrgyzstan	Both	55+ years	Non-melanoma skin cancer (basal-cell	Rate	2021	12.278754	15.99914404	8.989594098
Prevalence	Albania	Male	55+ years	Non-melanoma skin cancer (basal-cell	Number	1990	28.145747	37.37241374	20.6892865
Prevalence	Albania	Female	55+ years	Non-melanoma skin cancer (basal-cell	Number	1990	27.959574	36.07358356	21.05123595
Prevalence	Albania	Both	55+ years	Non-melanoma skin cancer (basal-cell	Number	1990	56.105321	73.36116181	42.06212178
Prevalence	Albania	Male	55+ years	Non-melanoma skin cancer (basal-cell	Percent	1990	0.0001655	0.000219699	0.000121625
Prevalence	Albania	Female	55+ years	Non-melanoma skin cancer (basal-cell	Percent	1990	0.0001581	0.000203961	0.000119023
Prevalence	Albania	Both	55+ years	Non-melanoma skin cancer (basal-cell	Percent	1990	0.0001617	0.00021143	0.00012123
Prevalence	Albania	Male	55+ years	Non-melanoma skin cancer (basal-cell	Rate	1990	16.545905	21.96994145	12.16251153
Prevalence	Albania	Female	55+ years	Non-melanoma skin cancer (basal-cell	Rate	1990	15.803733	20.39005641	11.89889792
Prevalence	Albania	Both	55+ years	Non-melanoma skin cancer (basal-cell	Rate	1990	16.167536	21.14004944	12.12079133
Prevalence	Albania	Male	55+ years	Non-melanoma skin cancer (basal-cell	Number	2021	71.221414	91.40744519	52.66123814
Prevalence	Albania	Female	55+ years	Non-melanoma skin cancer (basal-cell	Number	2021	66.643424	87.03926011	50.2455378
Prevalence	Albania	Both	55+ years	Non-melanoma skin cancer (basal-cell	Number	2021	137.86484	176.9934084	102.5774111
Prevalence	Albania	Male	55+ years	Non-melanoma skin cancer (basal-cell	Percent	2021	0.0001882	0.000241543	0.000139146
Prevalence	Albania	Female	55+ years	Non-melanoma skin cancer (basal-cell	Percent	2021	0.0001635	0.00021359	0.0001233
Prevalence	Albania	Both	55+ years	Non-melanoma skin cancer (basal-cell	Percent	2021	0.0001754	0.000225205	0.000130514
Prevalence	Albania	Male	55+ years	Non-melanoma skin cancer (basal-cell	Rate	2021	18.814068	24.14647264	13.91115509
Prevalence	Albania	Female	55+ years	Non-melanoma skin cancer (basal-cell	Rate	2021	16.344587	21.34675424	12.32293503
Prevalence	Albania	Both	55+ years	Non-melanoma skin cancer (basal-cell	Rate	2021	17.533496	22.5098242	13.0456807
Prevalence	Indonesia	Male	55+ years	Non-melanoma skin cancer (basal-cell	Number	1990	48.289759	69.41063944	32.77280117
Prevalence	Indonesia	Female	55+ years	Non-melanoma skin cancer (basal-cell	Number	1990	39.965719	58.75497287	26.00286635
Prevalence	Indonesia	Both	55+ years	Non-melanoma skin cancer (basal-cell	Number	1990	88.255478	127.9694387	58.44618484
Prevalence	Indonesia	Male	55+ years	Non-melanoma skin cancer (basal-cell	Percent	1990	6.23E-06	8.96E-06	4.23E-06
Prevalence	Indonesia	Female	55+ years	Non-melanoma skin cancer (basal-cell	Percent	1990	4.75E-06	6.99E-06	3.09E-06
Prevalence	Indonesia	Both	55+ years	Non-melanoma skin cancer (basal-cell	Percent	1990	5.46E-06	7.92E-06	3.62E-06
Prevalence	Indonesia	Male	55+ years	Non-melanoma skin cancer (basal-cell	Rate	1990	0.6233206	0.895947319	0.423028855
Prevalence	Indonesia	Female	55+ years	Non-melanoma skin cancer (basal-cell	Rate	1990	0.4753001	0.698754965	0.309244156
Prevalence	Indonesia	Both	55+ years	Non-melanoma skin cancer (basal-cell	Rate	1990	0.5462807	0.792100773	0.361768158
Prevalence	Indonesia	Male	55+ years	Non-melanoma skin cancer (basal-cell	Number	2021	125.35514	181.2291175	84.76275389
Prevalence	Indonesia	Female	55+ years	Non-melanoma skin cancer (basal-cell	Number	2021	101.32784	146.9234414	65.90359676
Prevalence	Indonesia	Both	55+ years	Non-melanoma skin cancer (basal-cell	Number	2021	226.68298	330.4295523	150.4741992
Prevalence	Indonesia	Male	55+ years	Non-melanoma skin cancer (basal-cell	Percent	2021	6.14E-06	8.88E-06	4.15E-06
Prevalence	Indonesia	Female	55+ years	Non-melanoma skin cancer (basal-cell	Percent	2021	4.72E-06	6.85E-06	3.07E-06
Prevalence	Indonesia	Both	55+ years	Non-melanoma skin cancer (basal-cell	Percent	2021	5.41E-06	7.89E-06	3.59E-06
Prevalence	Indonesia	Male	55+ years	Non-melanoma skin cancer (basal-cell	Rate	2021	0.6140807	0.887792098	0.415229651
Prevalence	Indonesia	Female	55+ years	Non-melanoma skin cancer (basal-cell	Rate	2021	0.4720369	0.684444464	0.307012629
Prevalence	Indonesia	Both	55+ years	Non-melanoma skin cancer (basal-cell	Rate	2021	0.5412737	0.788999763	0.359302328
Prevalence	Cambodia	Male	55+ years	Non-melanoma skin cancer (basal-cell	Number	1990	1.8011604	2.510768837	1.198166233
Prevalence	Cambodia	Female	55+ years	Non-melanoma skin cancer (basal-cell	Number	1990	1.918496	2.784655194	1.240631777
Prevalence	Cambodia	Both	55+ years	Non-melanoma skin cancer (basal-cell	Number	1990	3.7196564	5.271973951	2.456404512
Prevalence	Cambodia	Male	55+ years	Non-melanoma skin cancer (basal-cell	Percent	1990	5.69E-06	7.93E-06	3.78E-06
Prevalence	Cambodia	Female	55+ years	Non-melanoma skin cancer (basal-cell	Percent	1990	4.52E-06	6.56E-06	2.92E-06
Prevalence	Cambodia	Both	55+ years	Non-melanoma skin cancer (basal-cell	Percent	1990	5.02E-06	7.12E-06	3.32E-06
Prevalence	Cambodia	Male	55+ years	Non-melanoma skin cancer (basal-cell	Rate	1990	0.5687786	0.792861967	0.378362366
Prevalence	Cambodia	Female	55+ years	Non-melanoma skin cancer (basal-cell	Rate	1990	0.4522089	0.656371313	0.292429422
Prevalence	Cambodia	Both	55+ years	Non-melanoma skin cancer (basal-cell	Rate	1990	0.502031	0.711542749	0.33153366
Prevalence	Cambodia	Male	55+ years	Non-melanoma skin cancer (basal-cell	Number	2021	5.0191925	7.195525088	3.327545597
Prevalence	Cambodia	Female	55+ years	Non-melanoma skin cancer (basal-cell	Number	2021	5.7797902	8.365194706	3.771463518
Prevalence	Cambodia	Both	55+ years	Non-melanoma skin cancer (basal-cell	Number	2021	10.798983	15.43063036	7.184121518
Prevalence	Cambodia	Male	55+ years	Non-melanoma skin cancer (basal-cell	Percent	2021	5.51E-06	7.90E-06	3.65E-06
Prevalence	Cambodia	Female	55+ years	Non-melanoma skin cancer (basal-cell	Percent	2021	4.58E-06	6.63E-06	2.99E-06
Prevalence	Cambodia	Both	55+ years	Non-melanoma skin cancer (basal-cell	Percent	2021	4.97E-06	7.10E-06	3.31E-06
Prevalence	Cambodia	Male	55+ years	Non-melanoma skin cancer (basal-cell	Rate	2021	0.5506532	0.789417542	0.365063402
Prevalence	Cambodia	Female	55+ years	Non-melanoma skin cancer (basal-cell	Rate	2021	0.457873	0.662687857	0.298774047
Prevalence	Cambodia	Both	55+ years	Non-melanoma skin cancer (basal-cell	Rate	2021	0.4967765	0.70984233	0.330485109
Prevalence	Mongolia	Male	55+ years	Non-melanoma skin cancer (basal-cell	Number	1990	9.4127043	12.68328142	7.044208452

Prevalence	Mongolia	Female	55+ years	Non-melanoma skin cancer (basal-cell	Number	1990	11.811001	15.52795007	8.623695001
Prevalence	Mongolia	Both	55+ years	Non-melanoma skin cancer (basal-cell	Number	1990	21.223705	28.18347335	15.70804069
Prevalence	Mongolia	Male	55+ years	Non-melanoma skin cancer (basal-cell	Percent	1990	0.00012	0.000161713	8.98E-05
Prevalence	Mongolia	Female	55+ years	Non-melanoma skin cancer (basal-cell	Percent	1990	0.0001246	0.000163771	9.10E-05
Prevalence	Mongolia	Both	55+ years	Non-melanoma skin cancer (basal-cell	Percent	1990	0.0001225	0.000162682	9.07E-05
Prevalence	Mongolia	Male	55+ years	Non-melanoma skin cancer (basal-cell	Rate	1990	11.997808	16.16661534	8.978828476
Prevalence	Mongolia	Female	55+ years	Non-melanoma skin cancer (basal-cell	Rate	1990	12.455053	16.37468706	9.093943909
Prevalence	Mongolia	Both	55+ years	Non-melanoma skin cancer (basal-cell	Rate	1990	12.248035	16.26446374	9.064988369
Prevalence	Mongolia	Male	55+ years	Non-melanoma skin cancer (basal-cell	Number	2021	18.06332	23.75649097	13.37932762
Prevalence	Mongolia	Female	55+ years	Non-melanoma skin cancer (basal-cell	Number	2021	24.833923	32.90685621	17.98907
Prevalence	Mongolia	Both	55+ years	Non-melanoma skin cancer (basal-cell	Number	2021	42.897243	55.3598979	31.89835927
Prevalence	Mongolia	Male	55+ years	Non-melanoma skin cancer (basal-cell	Percent	2021	0.0001075	0.00014134	7.96E-05
Prevalence	Mongolia	Female	55+ years	Non-melanoma skin cancer (basal-cell	Percent	2021	0.0001096	0.000145262	7.94E-05
Prevalence	Mongolia	Both	55+ years	Non-melanoma skin cancer (basal-cell	Percent	2021	0.0001087	0.000140261	8.08E-05
Prevalence	Mongolia	Male	55+ years	Non-melanoma skin cancer (basal-cell	Rate	2021	10.740794	14.12606251	7.955603314
Prevalence	Mongolia	Female	55+ years	Non-melanoma skin cancer (basal-cell	Rate	2021	10.957859	14.52000478	7.937597583
Prevalence	Mongolia	Both	55+ years	Non-melanoma skin cancer (basal-cell	Rate	2021	10.865396	14.02204858	8.079500869
Prevalence	Lao People	Male	55+ years	Non-melanoma skin cancer (basal-cell	Number	1990	0.9491318	1.437647632	0.617677788
Prevalence	Lao People	Female	55+ years	Non-melanoma skin cancer (basal-cell	Number	1990	0.7944595	1.157611646	0.509495883
Prevalence	Lao People	Both	55+ years	Non-melanoma skin cancer (basal-cell	Number	1990	1.7435912	2.59305123	1.142539534
Prevalence	Lao People	Male	55+ years	Non-melanoma skin cancer (basal-cell	Percent	1990	5.80E-06	8.78E-06	3.77E-06
Prevalence	Lao People	Female	55+ years	Non-melanoma skin cancer (basal-cell	Percent	1990	4.44E-06	6.46E-06	2.84E-06
Prevalence	Lao People	Both	55+ years	Non-melanoma skin cancer (basal-cell	Percent	1990	5.09E-06	7.56E-06	3.33E-06
Prevalence	Lao People	Male	55+ years	Non-melanoma skin cancer (basal-cell	Rate	1990	0.5794211	0.877647782	0.37707678
Prevalence	Lao People	Female	55+ years	Non-melanoma skin cancer (basal-cell	Rate	1990	0.4435151	0.646248498	0.284431269
Prevalence	Lao People	Both	55+ years	Non-melanoma skin cancer (basal-cell	Rate	1990	0.5084322	0.756135246	0.333165192
Prevalence	Lao People	Male	55+ years	Non-melanoma skin cancer (basal-cell	Number	2021	2.1179883	3.057954582	1.406108123
Prevalence	Lao People	Female	55+ years	Non-melanoma skin cancer (basal-cell	Number	2021	1.7575161	2.561178846	1.181770407
Prevalence	Lao People	Both	55+ years	Non-melanoma skin cancer (basal-cell	Number	2021	3.8755044	5.685978199	2.669635115
Prevalence	Lao People	Male	55+ years	Non-melanoma skin cancer (basal-cell	Percent	2021	5.67E-06	8.19E-06	3.77E-06
Prevalence	Lao People	Female	55+ years	Non-melanoma skin cancer (basal-cell	Percent	2021	4.40E-06	6.42E-06	2.96E-06
Prevalence	Lao People	Both	55+ years	Non-melanoma skin cancer (basal-cell	Percent	2021	5.02E-06	7.36E-06	3.46E-06
Prevalence	Lao People	Male	55+ years	Non-melanoma skin cancer (basal-cell	Rate	2021	0.5665552	0.81799316	0.376129467
Prevalence	Lao People	Female	55+ years	Non-melanoma skin cancer (basal-cell	Rate	2021	0.4401352	0.641396626	0.295951043
Prevalence	Lao People	Both	55+ years	Non-melanoma skin cancer (basal-cell	Rate	2021	0.5012623	0.735431093	0.345293739
Prevalence	Fiji	Male	55+ years	Non-melanoma skin cancer (basal-cell	Number	1990	0.006162	0.013839901	0.001443152
Prevalence	Fiji	Female	55+ years	Non-melanoma skin cancer (basal-cell	Number	1990	0.0064791	0.014007158	0.002063871
Prevalence	Fiji	Both	55+ years	Non-melanoma skin cancer (basal-cell	Number	1990	0.0126411	0.027457428	0.004399274
Prevalence	Fiji	Male	55+ years	Non-melanoma skin cancer (basal-cell	Percent	1990	2.20E-07	4.94E-07	5.15E-08
Prevalence	Fiji	Female	55+ years	Non-melanoma skin cancer (basal-cell	Percent	1990	2.29E-07	4.95E-07	7.29E-08
Prevalence	Fiji	Both	55+ years	Non-melanoma skin cancer (basal-cell	Percent	1990	2.24E-07	4.87E-07	7.81E-08
Prevalence	Fiji	Male	55+ years	Non-melanoma skin cancer (basal-cell	Rate	1990	0.0219753	0.049357032	0.00514669
Prevalence	Fiji	Female	55+ years	Non-melanoma skin cancer (basal-cell	Rate	1990	0.0228748	0.04945271	0.007286563
Prevalence	Fiji	Both	55+ years	Non-melanoma skin cancer (basal-cell	Rate	1990	0.0224273	0.048713845	0.007805011
Prevalence	Fiji	Male	55+ years	Non-melanoma skin cancer (basal-cell	Number	2021	0.0140569	0.030898504	0.003385725
Prevalence	Fiji	Female	55+ years	Non-melanoma skin cancer (basal-cell	Number	2021	0.0163961	0.035548702	0.004727545
Prevalence	Fiji	Both	55+ years	Non-melanoma skin cancer (basal-cell	Number	2021	0.030453	0.065093555	0.00960434
Prevalence	Fiji	Male	55+ years	Non-melanoma skin cancer (basal-cell	Percent	2021	2.17E-07	4.77E-07	5.23E-08
Prevalence	Fiji	Female	55+ years	Non-melanoma skin cancer (basal-cell	Percent	2021	2.28E-07	4.94E-07	6.57E-08
Prevalence	Fiji	Both	55+ years	Non-melanoma skin cancer (basal-cell	Percent	2021	2.23E-07	4.76E-07	7.02E-08
Prevalence	Fiji	Male	55+ years	Non-melanoma skin cancer (basal-cell	Rate	2021	0.0217045	0.047708552	0.005227697
Prevalence	Fiji	Female	55+ years	Non-melanoma skin cancer (basal-cell	Rate	2021	0.02278	0.049390007	0.00656827
Prevalence	Fiji	Both	55+ years	Non-melanoma skin cancer (basal-cell	Rate	2021	0.0222706	0.047603671	0.007023765
Prevalence	Marshall I	Male	55+ years	Non-melanoma skin cancer (basal-cell	Number	1990	0.0002638	0.000598671	6.34E-05
Prevalence	Marshall I	Female	55+ years	Non-melanoma skin cancer (basal-cell	Number	1990	0.0002761	0.000581319	7.59E-05
Prevalence	Marshall I	Both	55+ years	Non-melanoma skin cancer (basal-cell	Number	1990	0.00054	0.001179085	0.000179144

Prevalence	Marshall I	Male	55+ years	Non-melanoma skin cancer (basal-cell	Percent	1990	2.12E-07	4.82E-07	5.11E-08
Prevalence	Marshall I	Female	55+ years	Non-melanoma skin cancer (basal-cell	Percent	1990	2.11E-07	4.45E-07	5.81E-08
Prevalence	Marshall I	Both	55+ years	Non-melanoma skin cancer (basal-cell	Percent	1990	2.12E-07	4.62E-07	7.02E-08
Prevalence	Marshall I	Male	55+ years	Non-melanoma skin cancer (basal-cell	Rate	1990	0.0212265	0.048166223	0.005104431
Prevalence	Marshall I	Female	55+ years	Non-melanoma skin cancer (basal-cell	Rate	1990	0.0211184	0.044459841	0.005804578
Prevalence	Marshall I	Both	55+ years	Non-melanoma skin cancer (basal-cell	Rate	1990	0.0211711	0.046230616	0.007024026
Prevalence	Marshall I	Male	55+ years	Non-melanoma skin cancer (basal-cell	Number	2021	0.0006138	0.001349068	0.000150296
Prevalence	Marshall I	Female	55+ years	Non-melanoma skin cancer (basal-cell	Number	2021	0.0006118	0.001380161	0.000157318
Prevalence	Marshall I	Both	55+ years	Non-melanoma skin cancer (basal-cell	Number	2021	0.0012256	0.002701744	0.000375446
Prevalence	Marshall I	Male	55+ years	Non-melanoma skin cancer (basal-cell	Percent	2021	2.09E-07	4.59E-07	5.11E-08
Prevalence	Marshall I	Female	55+ years	Non-melanoma skin cancer (basal-cell	Percent	2021	2.10E-07	4.73E-07	5.39E-08
Prevalence	Marshall I	Both	55+ years	Non-melanoma skin cancer (basal-cell	Percent	2021	2.09E-07	4.61E-07	6.41E-08
Prevalence	Marshall I	Male	55+ years	Non-melanoma skin cancer (basal-cell	Rate	2021	0.0208726	0.045874805	0.005110794
Prevalence	Marshall I	Female	55+ years	Non-melanoma skin cancer (basal-cell	Rate	2021	0.0209648	0.047294434	0.005390869
Prevalence	Marshall I	Both	55+ years	Non-melanoma skin cancer (basal-cell	Rate	2021	0.0209185	0.046112783	0.006408033
Prevalence	Georgia	Male	55+ years	Non-melanoma skin cancer (basal-cell	Number	1990	55.292128	73.77218208	40.56533907
Prevalence	Georgia	Female	55+ years	Non-melanoma skin cancer (basal-cell	Number	1990	95.344474	123.6989456	70.92122821
Prevalence	Georgia	Both	55+ years	Non-melanoma skin cancer (basal-cell	Number	1990	150.6366	196.7647085	113.3378895
Prevalence	Georgia	Male	55+ years	Non-melanoma skin cancer (basal-cell	Percent	1990	0.0001253	0.000167141	9.19E-05
Prevalence	Georgia	Female	55+ years	Non-melanoma skin cancer (basal-cell	Percent	1990	0.0001425	0.000184905	0.000106006
Prevalence	Georgia	Both	55+ years	Non-melanoma skin cancer (basal-cell	Percent	1990	0.0001357	0.000177196	0.000102067
Prevalence	Georgia	Male	55+ years	Non-melanoma skin cancer (basal-cell	Rate	1990	12.521505	16.70651447	9.18646304
Prevalence	Georgia	Female	55+ years	Non-melanoma skin cancer (basal-cell	Rate	1990	14.248175	18.48543677	10.59839171
Prevalence	Georgia	Both	55+ years	Non-melanoma skin cancer (basal-cell	Rate	1990	13.561738	17.7146279	10.20375328
Prevalence	Georgia	Male	55+ years	Non-melanoma skin cancer (basal-cell	Number	2021	59.556699	76.35022354	44.61482873
Prevalence	Georgia	Female	55+ years	Non-melanoma skin cancer (basal-cell	Number	2021	92.657545	118.769193	69.76835316
Prevalence	Georgia	Both	55+ years	Non-melanoma skin cancer (basal-cell	Number	2021	152.21424	195.3465138	114.7587144
Prevalence	Georgia	Male	55+ years	Non-melanoma skin cancer (basal-cell	Percent	2021	0.0001399	0.000179336	0.000104787
Prevalence	Georgia	Female	55+ years	Non-melanoma skin cancer (basal-cell	Percent	2021	0.0001485	0.000190388	0.000111844
Prevalence	Georgia	Both	55+ years	Non-melanoma skin cancer (basal-cell	Percent	2021	0.000145	0.000186108	0.000109339
Prevalence	Georgia	Male	55+ years	Non-melanoma skin cancer (basal-cell	Rate	2021	13.984143	17.92732789	10.4757344
Prevalence	Georgia	Female	55+ years	Non-melanoma skin cancer (basal-cell	Rate	2021	14.849414	19.03409876	11.18116315
Prevalence	Georgia	Both	55+ years	Non-melanoma skin cancer (basal-cell	Rate	2021	14.49841	18.60675954	10.93076995
Prevalence	Uzbekistan	Male	55+ years	Non-melanoma skin cancer (basal-cell	Number	1990	96.846945	127.783888	72.19027261
Prevalence	Uzbekistan	Female	55+ years	Non-melanoma skin cancer (basal-cell	Number	1990	137.87641	179.0310982	104.0052435
Prevalence	Uzbekistan	Both	55+ years	Non-melanoma skin cancer (basal-cell	Number	1990	234.72335	304.1507381	178.5015583
Prevalence	Uzbekistan	Male	55+ years	Non-melanoma skin cancer (basal-cell	Percent	1990	0.0001189	0.000156831	8.86E-05
Prevalence	Uzbekistan	Female	55+ years	Non-melanoma skin cancer (basal-cell	Percent	1990	0.0001209	0.000157009	9.12E-05
Prevalence	Uzbekistan	Both	55+ years	Non-melanoma skin cancer (basal-cell	Percent	1990	0.0001201	0.000155572	9.13E-05
Prevalence	Uzbekistan	Male	55+ years	Non-melanoma skin cancer (basal-cell	Rate	1990	11.880154	15.67516977	8.855535675
Prevalence	Uzbekistan	Female	55+ years	Non-melanoma skin cancer (basal-cell	Rate	1990	12.089247	15.69776264	9.119363296
Prevalence	Uzbekistan	Both	55+ years	Non-melanoma skin cancer (basal-cell	Rate	1990	12.00209	15.55211436	9.127305316
Prevalence	Uzbekistan	Male	55+ years	Non-melanoma skin cancer (basal-cell	Number	2021	234.75292	308.8874153	178.6268899
Prevalence	Uzbekistan	Female	55+ years	Non-melanoma skin cancer (basal-cell	Number	2021	276.86337	363.1546739	206.636183
Prevalence	Uzbekistan	Both	55+ years	Non-melanoma skin cancer (basal-cell	Number	2021	511.61629	668.37983	385.5770182
Prevalence	Uzbekistan	Male	55+ years	Non-melanoma skin cancer (basal-cell	Percent	2021	0.0001109	0.000145908	8.44E-05
Prevalence	Uzbekistan	Female	55+ years	Non-melanoma skin cancer (basal-cell	Percent	2021	0.0001095	0.000143572	8.17E-05
Prevalence	Uzbekistan	Both	55+ years	Non-melanoma skin cancer (basal-cell	Percent	2021	0.0001101	0.000143839	8.30E-05
Prevalence	Uzbekistan	Male	55+ years	Non-melanoma skin cancer (basal-cell	Rate	2021	11.080402	14.57957108	8.431238401
Prevalence	Uzbekistan	Female	55+ years	Non-melanoma skin cancer (basal-cell	Rate	2021	10.943133	14.35383137	8.167376436
Prevalence	Uzbekistan	Both	55+ years	Non-melanoma skin cancer (basal-cell	Rate	2021	11.005694	14.37793156	8.294385515
Prevalence	Kiribati	Male	55+ years	Non-melanoma skin cancer (basal-cell	Number	1990	0.000529	0.001164235	0.000119196
Prevalence	Kiribati	Female	55+ years	Non-melanoma skin cancer (basal-cell	Number	1990	0.0006922	0.001481657	0.000199862
Prevalence	Kiribati	Both	55+ years	Non-melanoma skin cancer (basal-cell	Number	1990	0.0012211	0.002674599	0.00040336
Prevalence	Kiribati	Male	55+ years	Non-melanoma skin cancer (basal-cell	Percent	1990	2.00E-07	4.39E-07	4.50E-08
Prevalence	Kiribati	Female	55+ years	Non-melanoma skin cancer (basal-cell	Percent	1990	2.09E-07	4.48E-07	6.04E-08

Prevalence	Kiribati	Both	55+ years	Non-melanoma skin cancer (basal-cell	Percent	1990	2.05E-07	4.49E-07	6.77E-08
Prevalence	Kiribati	Male	55+ years	Non-melanoma skin cancer (basal-cell	Rate	1990	0.019956	0.043922311	0.004496816
Prevalence	Kiribati	Female	55+ years	Non-melanoma skin cancer (basal-cell	Rate	1990	0.0209298	0.044801744	0.006043331
Prevalence	Kiribati	Both	55+ years	Non-melanoma skin cancer (basal-cell	Rate	1990	0.0204965	0.044892319	0.006770266
Prevalence	Kiribati	Male	55+ years	Non-melanoma skin cancer (basal-cell	Number	2021	0.0010933	0.002422044	0.000264014
Prevalence	Kiribati	Female	55+ years	Non-melanoma skin cancer (basal-cell	Number	2021	0.0014838	0.003323769	0.000388658
Prevalence	Kiribati	Both	55+ years	Non-melanoma skin cancer (basal-cell	Number	2021	0.0025771	0.005601916	0.000818333
Prevalence	Kiribati	Male	55+ years	Non-melanoma skin cancer (basal-cell	Percent	2021	1.99E-07	4.42E-07	4.82E-08
Prevalence	Kiribati	Female	55+ years	Non-melanoma skin cancer (basal-cell	Percent	2021	2.09E-07	4.69E-07	5.48E-08
Prevalence	Kiribati	Both	55+ years	Non-melanoma skin cancer (basal-cell	Percent	2021	2.05E-07	4.46E-07	6.51E-08
Prevalence	Kiribati	Male	55+ years	Non-melanoma skin cancer (basal-cell	Rate	2021	0.0199332	0.04416038	0.004813689
Prevalence	Kiribati	Female	55+ years	Non-melanoma skin cancer (basal-cell	Rate	2021	0.0209214	0.046865201	0.005480087
Prevalence	Kiribati	Both	55+ years	Non-melanoma skin cancer (basal-cell	Rate	2021	0.0204905	0.04454151	0.006506667
Prevalence	Kazakhstan	Male	55+ years	Non-melanoma skin cancer (basal-cell	Number	1990	92.154122	118.9425988	70.09378554
Prevalence	Kazakhstan	Female	55+ years	Non-melanoma skin cancer (basal-cell	Number	1990	174.11427	230.2582127	130.4600382
Prevalence	Kazakhstan	Both	55+ years	Non-melanoma skin cancer (basal-cell	Number	1990	266.26839	349.0682614	204.2923388
Prevalence	Kazakhstan	Male	55+ years	Non-melanoma skin cancer (basal-cell	Percent	1990	0.000117	0.000151062	8.90E-05
Prevalence	Kazakhstan	Female	55+ years	Non-melanoma skin cancer (basal-cell	Percent	1990	0.0001336	0.000176638	0.00010009
Prevalence	Kazakhstan	Both	55+ years	Non-melanoma skin cancer (basal-cell	Percent	1990	0.0001274	0.000166943	9.77E-05
Prevalence	Kazakhstan	Male	55+ years	Non-melanoma skin cancer (basal-cell	Rate	1990	11.700691	15.1019897	8.899718334
Prevalence	Kazakhstan	Female	55+ years	Non-melanoma skin cancer (basal-cell	Rate	1990	13.354763	17.66106843	10.00643423
Prevalence	Kazakhstan	Both	55+ years	Non-melanoma skin cancer (basal-cell	Rate	1990	12.731847	16.69099303	9.768410311
Prevalence	Kazakhstan	Male	55+ years	Non-melanoma skin cancer (basal-cell	Number	2021	150.66212	205.058517	111.2673156
Prevalence	Kazakhstan	Female	55+ years	Non-melanoma skin cancer (basal-cell	Number	2021	237.69666	304.2576275	179.1536588
Prevalence	Kazakhstan	Both	55+ years	Non-melanoma skin cancer (basal-cell	Number	2021	388.35879	502.7261839	297.0776057
Prevalence	Kazakhstan	Male	55+ years	Non-melanoma skin cancer (basal-cell	Percent	2021	0.0001153	0.000156958	8.52E-05
Prevalence	Kazakhstan	Female	55+ years	Non-melanoma skin cancer (basal-cell	Percent	2021	0.0001274	0.000163068	9.60E-05
Prevalence	Kazakhstan	Both	55+ years	Non-melanoma skin cancer (basal-cell	Percent	2021	0.0001224	0.000158492	9.37E-05
Prevalence	Kazakhstan	Male	55+ years	Non-melanoma skin cancer (basal-cell	Rate	2021	11.526298	15.68785493	8.51242626
Prevalence	Kazakhstan	Female	55+ years	Non-melanoma skin cancer (basal-cell	Rate	2021	12.736051	16.3024619	9.599252188
Prevalence	Kazakhstan	Both	55+ years	Non-melanoma skin cancer (basal-cell	Rate	2021	12.237764	15.84165083	9.361357835
Prevalence	Bosnia and	Male	55+ years	Non-melanoma skin cancer (basal-cell	Number	1990	47.890347	63.02434489	34.95791933
Prevalence	Bosnia and	Female	55+ years	Non-melanoma skin cancer (basal-cell	Number	1990	55.95788	72.98381876	42.56362539
Prevalence	Bosnia and	Both	55+ years	Non-melanoma skin cancer (basal-cell	Number	1990	103.84823	135.891158	76.75482192
Prevalence	Bosnia and	Male	55+ years	Non-melanoma skin cancer (basal-cell	Percent	1990	0.0001486	0.00019549	0.000108435
Prevalence	Bosnia and	Female	55+ years	Non-melanoma skin cancer (basal-cell	Percent	1990	0.0001361	0.000177566	0.000103543
Prevalence	Bosnia and	Both	55+ years	Non-melanoma skin cancer (basal-cell	Percent	1990	0.0001416	0.000185292	0.000104654
Prevalence	Bosnia and	Male	55+ years	Non-melanoma skin cancer (basal-cell	Rate	1990	14.854562	19.54880398	10.84319898
Prevalence	Bosnia and	Female	55+ years	Non-melanoma skin cancer (basal-cell	Rate	1990	13.61017	17.751247	10.35239647
Prevalence	Bosnia and	Both	55+ years	Non-melanoma skin cancer (basal-cell	Rate	1990	14.157085	18.52532983	10.46358287
Prevalence	Bosnia and	Male	55+ years	Non-melanoma skin cancer (basal-cell	Number	2021	88.003553	116.1585026	64.91925548
Prevalence	Bosnia and	Female	55+ years	Non-melanoma skin cancer (basal-cell	Number	2021	96.354899	125.2742916	72.56516636
Prevalence	Bosnia and	Both	55+ years	Non-melanoma skin cancer (basal-cell	Number	2021	184.35845	238.4720243	138.7085424
Prevalence	Bosnia and	Male	55+ years	Non-melanoma skin cancer (basal-cell	Percent	2021	0.0001792	0.000236594	0.000132227
Prevalence	Bosnia and	Female	55+ years	Non-melanoma skin cancer (basal-cell	Percent	2021	0.0001595	0.000207467	0.000120161
Prevalence	Bosnia and	Both	55+ years	Non-melanoma skin cancer (basal-cell	Percent	2021	0.0001684	0.000217806	0.000126686
Prevalence	Bosnia and	Male	55+ years	Non-melanoma skin cancer (basal-cell	Rate	2021	17.91667	23.64874466	13.21693084
Prevalence	Bosnia and	Female	55+ years	Non-melanoma skin cancer (basal-cell	Rate	2021	15.950526	20.73782259	12.01238919
Prevalence	Bosnia and	Both	55+ years	Non-melanoma skin cancer (basal-cell	Rate	2021	16.83226	21.77292677	12.66434058
Prevalence	Singapore	Male	55+ years	Non-melanoma skin cancer (basal-cell	Number	1990	9.6823108	11.717092	8.061944187
Prevalence	Singapore	Female	55+ years	Non-melanoma skin cancer (basal-cell	Number	1990	12.120554	14.52478748	10.16809482
Prevalence	Singapore	Both	55+ years	Non-melanoma skin cancer (basal-cell	Number	1990	21.802864	25.674836	18.384228
Prevalence	Singapore	Male	55+ years	Non-melanoma skin cancer (basal-cell	Percent	1990	5.77E-05	6.99E-05	4.81E-05
Prevalence	Singapore	Female	55+ years	Non-melanoma skin cancer (basal-cell	Percent	1990	6.24E-05	7.47E-05	5.23E-05
Prevalence	Singapore	Both	55+ years	Non-melanoma skin cancer (basal-cell	Percent	1990	6.02E-05	7.09E-05	5.08E-05
Prevalence	Singapore	Male	55+ years	Non-melanoma skin cancer (basal-cell	Rate	1990	5.76678	6.978694801	4.801690384

Prevalence	Singapore	Female	55+ years	Non-melanoma skin cancer (basal-cell	Rate	1990	6.2261793	7.461204742	5.223225289
Prevalence	Singapore	Both	55+ years	Non-melanoma skin cancer (basal-cell	Rate	1990	6.0134412	7.081368407	5.070548118
Prevalence	Singapore	Male	55+ years	Non-melanoma skin cancer (basal-cell	Number	2021	34.743901	46.25529981	25.28773037
Prevalence	Singapore	Female	55+ years	Non-melanoma skin cancer (basal-cell	Number	2021	34.760891	45.01848827	25.05143641
Prevalence	Singapore	Both	55+ years	Non-melanoma skin cancer (basal-cell	Number	2021	69.504792	90.18688229	50.89960892
Prevalence	Singapore	Male	55+ years	Non-melanoma skin cancer (basal-cell	Percent	2021	4.61E-05	6.14E-05	3.36E-05
Prevalence	Singapore	Female	55+ years	Non-melanoma skin cancer (basal-cell	Percent	2021	4.56E-05	5.91E-05	3.29E-05
Prevalence	Singapore	Both	55+ years	Non-melanoma skin cancer (basal-cell	Percent	2021	4.59E-05	5.95E-05	3.36E-05
Prevalence	Singapore	Male	55+ years	Non-melanoma skin cancer (basal-cell	Rate	2021	4.6015109	6.126090092	3.349127886
Prevalence	Singapore	Female	55+ years	Non-melanoma skin cancer (basal-cell	Rate	2021	4.5512062	5.894222453	3.279957738
Prevalence	Singapore	Both	55+ years	Non-melanoma skin cancer (basal-cell	Rate	2021	4.5762142	5.937928575	3.351243934
Prevalence	Estonia	Male	55+ years	Non-melanoma skin cancer (basal-cell	Number	1990	17.471133	21.04422755	14.61457693
Prevalence	Estonia	Female	55+ years	Non-melanoma skin cancer (basal-cell	Number	1990	35.464369	42.72336268	29.69492744
Prevalence	Estonia	Both	55+ years	Non-melanoma skin cancer (basal-cell	Number	1990	52.935502	63.16168537	44.28268038
Prevalence	Estonia	Male	55+ years	Non-melanoma skin cancer (basal-cell	Percent	1990	0.0001341	0.000161594	0.000112201
Prevalence	Estonia	Female	55+ years	Non-melanoma skin cancer (basal-cell	Percent	1990	0.0001555	0.000187285	0.00013018
Prevalence	Estonia	Both	55+ years	Non-melanoma skin cancer (basal-cell	Percent	1990	0.0001477	0.000176253	0.000123571
Prevalence	Estonia	Male	55+ years	Non-melanoma skin cancer (basal-cell	Rate	1990	13.409579	16.1520288	11.21709347
Prevalence	Estonia	Female	55+ years	Non-melanoma skin cancer (basal-cell	Rate	1990	15.54203	18.72323662	13.01360937
Prevalence	Estonia	Both	55+ years	Non-melanoma skin cancer (basal-cell	Rate	1990	14.76698	17.61969418	12.35317394
Prevalence	Estonia	Male	55+ years	Non-melanoma skin cancer (basal-cell	Number	2021	24.167279	31.27895379	17.68687488
Prevalence	Estonia	Female	55+ years	Non-melanoma skin cancer (basal-cell	Number	2021	41.922602	54.84349843	30.18035135
Prevalence	Estonia	Both	55+ years	Non-melanoma skin cancer (basal-cell	Number	2021	66.089882	85.9100558	47.87871797
Prevalence	Estonia	Male	55+ years	Non-melanoma skin cancer (basal-cell	Percent	2021	0.0001412	0.000182676	0.000103302
Prevalence	Estonia	Female	55+ years	Non-melanoma skin cancer (basal-cell	Percent	2021	0.0001576	0.000206166	0.000113458
Prevalence	Estonia	Both	55+ years	Non-melanoma skin cancer (basal-cell	Percent	2021	0.0001512	0.000196494	0.000109501
Prevalence	Estonia	Male	55+ years	Non-melanoma skin cancer (basal-cell	Rate	2021	14.103116	18.25322248	10.3213958
Prevalence	Estonia	Female	55+ years	Non-melanoma skin cancer (basal-cell	Rate	2021	15.753621	20.60901903	11.34113346
Prevalence	Estonia	Both	55+ years	Non-melanoma skin cancer (basal-cell	Rate	2021	15.10711	19.63769129	10.94432397
Prevalence	Australia	Male	55+ years	Non-melanoma skin cancer (basal-cell	Number	1990	317.34102	407.7381247	232.4081462
Prevalence	Australia	Female	55+ years	Non-melanoma skin cancer (basal-cell	Number	1990	321.83249	409.3314101	237.1559782
Prevalence	Australia	Both	55+ years	Non-melanoma skin cancer (basal-cell	Number	1990	639.17352	812.8706564	473.927549
Prevalence	Australia	Male	55+ years	Non-melanoma skin cancer (basal-cell	Percent	1990	0.0002108	0.000270805	0.000154395
Prevalence	Australia	Female	55+ years	Non-melanoma skin cancer (basal-cell	Percent	1990	0.0001814	0.000230702	0.000133668
Prevalence	Australia	Both	55+ years	Non-melanoma skin cancer (basal-cell	Percent	1990	0.0001949	0.000247862	0.000144523
Prevalence	Australia	Male	55+ years	Non-melanoma skin cancer (basal-cell	Rate	1990	21.065883	27.06666671	15.42782844
Prevalence	Australia	Female	55+ years	Non-melanoma skin cancer (basal-cell	Rate	1990	18.130235	23.05943283	13.36003594
Prevalence	Australia	Both	55+ years	Non-melanoma skin cancer (basal-cell	Rate	1990	19.477873	24.77103837	14.44224541
Prevalence	Australia	Male	55+ years	Non-melanoma skin cancer (basal-cell	Number	2021	775.63349	1018.127617	565.7221982
Prevalence	Australia	Female	55+ years	Non-melanoma skin cancer (basal-cell	Number	2021	644.08927	830.4837148	484.8027534
Prevalence	Australia	Both	55+ years	Non-melanoma skin cancer (basal-cell	Number	2021	1419.7228	1818.777599	1045.134333
Prevalence	Australia	Male	55+ years	Non-melanoma skin cancer (basal-cell	Percent	2021	0.0002204	0.000289333	0.000160757
Prevalence	Australia	Female	55+ years	Non-melanoma skin cancer (basal-cell	Percent	2021	0.0001659	0.000213938	0.00012487
Prevalence	Australia	Both	55+ years	Non-melanoma skin cancer (basal-cell	Percent	2021	0.0001918	0.000245749	0.000141208
Prevalence	Australia	Male	55+ years	Non-melanoma skin cancer (basal-cell	Rate	2021	22.017626	28.9012184	16.05895031
Prevalence	Australia	Female	55+ years	Non-melanoma skin cancer (basal-cell	Rate	2021	16.578694	21.37644007	12.47869985
Prevalence	Australia	Both	55+ years	Non-melanoma skin cancer (basal-cell	Rate	2021	19.165173	24.55210816	14.10851508
Prevalence	Romania	Male	55+ years	Non-melanoma skin cancer (basal-cell	Number	1990	184.95972	230.391447	146.2387648
Prevalence	Romania	Female	55+ years	Non-melanoma skin cancer (basal-cell	Number	1990	222.71691	279.5849328	174.1038178
Prevalence	Romania	Both	55+ years	Non-melanoma skin cancer (basal-cell	Number	1990	407.67664	510.8049137	322.7177382
Prevalence	Romania	Male	55+ years	Non-melanoma skin cancer (basal-cell	Percent	1990	8.26E-05	0.000102863	6.53E-05
Prevalence	Romania	Female	55+ years	Non-melanoma skin cancer (basal-cell	Percent	1990	8.06E-05	0.000101183	6.30E-05
Prevalence	Romania	Both	55+ years	Non-melanoma skin cancer (basal-cell	Percent	1990	8.15E-05	0.000102101	6.45E-05
Prevalence	Romania	Male	55+ years	Non-melanoma skin cancer (basal-cell	Rate	1990	8.2563933	10.28441412	6.52793338
Prevalence	Romania	Female	55+ years	Non-melanoma skin cancer (basal-cell	Rate	1990	8.0588015	10.11651725	6.299782531
Prevalence	Romania	Both	55+ years	Non-melanoma skin cancer (basal-cell	Rate	1990	8.1472625	10.20824185	6.44939121

Prevalence	Romania	Male	55+ years	Non-melanoma skin cancer (basal-cell	Number	2021	379.35173	482.502879	284.1053676
Prevalence	Romania	Female	55+ years	Non-melanoma skin cancer (basal-cell	Number	2021	466.34713	603.4303527	343.5185592
Prevalence	Romania	Both	55+ years	Non-melanoma skin cancer (basal-cell	Number	2021	845.69886	1092.867458	631.0912377
Prevalence	Romania	Male	55+ years	Non-melanoma skin cancer (basal-cell	Percent	2021	0.000147	0.000186987	0.000110113
Prevalence	Romania	Female	55+ years	Non-melanoma skin cancer (basal-cell	Percent	2021	0.0001362	0.000176305	0.000100355
Prevalence	Romania	Both	55+ years	Non-melanoma skin cancer (basal-cell	Percent	2021	0.0001409	0.000182064	0.000105129
Prevalence	Romania	Male	55+ years	Non-melanoma skin cancer (basal-cell	Rate	2021	14.696324	18.69246458	11.00642038
Prevalence	Romania	Female	55+ years	Non-melanoma skin cancer (basal-cell	Rate	2021	13.61963	17.62313472	10.03243178
Prevalence	Romania	Both	55+ years	Non-melanoma skin cancer (basal-cell	Rate	2021	14.082424	18.19823043	10.50881668
Prevalence	Azerbaijan	Male	55+ years	Non-melanoma skin cancer (basal-cell	Number	1990	40.538785	53.19593044	29.82710785
Prevalence	Azerbaijan	Female	55+ years	Non-melanoma skin cancer (basal-cell	Number	1990	64.182431	83.63128062	47.77613434
Prevalence	Azerbaijan	Both	55+ years	Non-melanoma skin cancer (basal-cell	Number	1990	104.72122	137.1041505	77.55124072
Prevalence	Azerbaijan	Male	55+ years	Non-melanoma skin cancer (basal-cell	Percent	1990	0.000112	0.00014696	8.24E-05
Prevalence	Azerbaijan	Female	55+ years	Non-melanoma skin cancer (basal-cell	Percent	1990	0.0001259	0.000164013	9.37E-05
Prevalence	Azerbaijan	Both	55+ years	Non-melanoma skin cancer (basal-cell	Percent	1990	0.0001201	0.000157247	8.89E-05
Prevalence	Azerbaijan	Male	55+ years	Non-melanoma skin cancer (basal-cell	Rate	1990	11.193226	14.68800989	8.23560847
Prevalence	Azerbaijan	Female	55+ years	Non-melanoma skin cancer (basal-cell	Rate	1990	12.584045	16.39731957	9.367314915
Prevalence	Azerbaijan	Both	55+ years	Non-melanoma skin cancer (basal-cell	Rate	1990	12.006523	15.71929942	8.891424285
Prevalence	Azerbaijan	Male	55+ years	Non-melanoma skin cancer (basal-cell	Number	2021	97.662876	128.6739391	71.6940899
Prevalence	Azerbaijan	Female	55+ years	Non-melanoma skin cancer (basal-cell	Number	2021	119.98698	156.3366238	90.46482026
Prevalence	Azerbaijan	Both	55+ years	Non-melanoma skin cancer (basal-cell	Number	2021	217.64986	286.2647119	164.2293763
Prevalence	Azerbaijan	Male	55+ years	Non-melanoma skin cancer (basal-cell	Percent	2021	0.0001137	0.000149855	8.35E-05
Prevalence	Azerbaijan	Female	55+ years	Non-melanoma skin cancer (basal-cell	Percent	2021	0.0001136	0.000148047	8.57E-05
Prevalence	Azerbaijan	Both	55+ years	Non-melanoma skin cancer (basal-cell	Percent	2021	0.0001137	0.000149501	8.58E-05
Prevalence	Azerbaijan	Male	55+ years	Non-melanoma skin cancer (basal-cell	Rate	2021	11.363127	14.97128089	8.34164529
Prevalence	Azerbaijan	Female	55+ years	Non-melanoma skin cancer (basal-cell	Rate	2021	11.358883	14.8000169	8.564089695
Prevalence	Azerbaijan	Both	55+ years	Non-melanoma skin cancer (basal-cell	Rate	2021	11.360787	14.94231338	8.572369226
Prevalence	Taiwan (Pr	Male	55+ years	Non-melanoma skin cancer (basal-cell	Number	1990	27.293538	33.93348423	22.1737057
Prevalence	Taiwan (Pr	Female	55+ years	Non-melanoma skin cancer (basal-cell	Number	1990	17.634237	22.47469209	13.47225959
Prevalence	Taiwan (Pr	Both	55+ years	Non-melanoma skin cancer (basal-cell	Number	1990	44.927775	55.24861772	36.24226826
Prevalence	Taiwan (Pr	Male	55+ years	Non-melanoma skin cancer (basal-cell	Percent	1990	1.81E-05	2.25E-05	1.47E-05
Prevalence	Taiwan (Pr	Female	55+ years	Non-melanoma skin cancer (basal-cell	Percent	1990	1.40E-05	1.78E-05	1.07E-05
Prevalence	Taiwan (Pr	Both	55+ years	Non-melanoma skin cancer (basal-cell	Percent	1990	1.62E-05	1.99E-05	1.31E-05
Prevalence	Taiwan (Pr	Male	55+ years	Non-melanoma skin cancer (basal-cell	Rate	1990	1.8054209	2.24464203	1.466752764
Prevalence	Taiwan (Pr	Female	55+ years	Non-melanoma skin cancer (basal-cell	Rate	1990	1.3974704	1.781064715	1.067643823
Prevalence	Taiwan (Pr	Both	55+ years	Non-melanoma skin cancer (basal-cell	Rate	1990	1.6198226	1.991929474	1.306675991
Prevalence	Taiwan (Pr	Male	55+ years	Non-melanoma skin cancer (basal-cell	Number	2021	1.6162129	2.956389654	0.671713177
Prevalence	Taiwan (Pr	Female	55+ years	Non-melanoma skin cancer (basal-cell	Number	2021	1.8981609	3.672054684	0.702635353
Prevalence	Taiwan (Pr	Both	55+ years	Non-melanoma skin cancer (basal-cell	Number	2021	3.5143738	6.609095811	1.524023819
Prevalence	Taiwan (Pr	Male	55+ years	Non-melanoma skin cancer (basal-cell	Percent	2021	4.58E-07	8.37E-07	1.90E-07
Prevalence	Taiwan (Pr	Female	55+ years	Non-melanoma skin cancer (basal-cell	Percent	2021	4.75E-07	9.20E-07	1.76E-07
Prevalence	Taiwan (Pr	Both	55+ years	Non-melanoma skin cancer (basal-cell	Percent	2021	4.67E-07	8.78E-07	2.03E-07
Prevalence	Taiwan (Pr	Male	55+ years	Non-melanoma skin cancer (basal-cell	Rate	2021	0.0457159	0.083623804	0.018999935
Prevalence	Taiwan (Pr	Female	55+ years	Non-melanoma skin cancer (basal-cell	Rate	2021	0.0475183	0.091925686	0.017589672
Prevalence	Taiwan (Pr	Both	55+ years	Non-melanoma skin cancer (basal-cell	Rate	2021	0.046672	0.087770953	0.020239535
Prevalence	Slovakia	Male	55+ years	Non-melanoma skin cancer (basal-cell	Number	1990	92.676121	110.7284066	78.23638144
Prevalence	Slovakia	Female	55+ years	Non-melanoma skin cancer (basal-cell	Number	1990	114.64934	137.0149049	95.6558636
Prevalence	Slovakia	Both	55+ years	Non-melanoma skin cancer (basal-cell	Number	1990	207.32546	243.7846253	175.2414993
Prevalence	Slovakia	Male	55+ years	Non-melanoma skin cancer (basal-cell	Percent	1990	0.00021	0.000250958	0.000177322
Prevalence	Slovakia	Female	55+ years	Non-melanoma skin cancer (basal-cell	Percent	1990	0.0001924	0.000229893	0.000160491
Prevalence	Slovakia	Both	55+ years	Non-melanoma skin cancer (basal-cell	Percent	1990	0.0001999	0.000235042	0.000168943
Prevalence	Slovakia	Male	55+ years	Non-melanoma skin cancer (basal-cell	Rate	1990	21.000857	25.09159197	17.72874207
Prevalence	Slovakia	Female	55+ years	Non-melanoma skin cancer (basal-cell	Rate	1990	19.231358	22.98297306	16.04537942
Prevalence	Slovakia	Both	55+ years	Non-melanoma skin cancer (basal-cell	Rate	1990	19.98404	23.4983286	16.89147677
Prevalence	Slovakia	Male	55+ years	Non-melanoma skin cancer (basal-cell	Number	2021	133.63337	173.6421655	99.12694992
Prevalence	Slovakia	Female	55+ years	Non-melanoma skin cancer (basal-cell	Number	2021	165.89094	212.9845041	124.5622714

Prevalence	Slovakia	Both	55+ years	Non-melanoma skin cancer (basal-cell	Number	2021	299.52431	380.7820135	221.7891996
Prevalence	Slovakia	Male	55+ years	Non-melanoma skin cancer (basal-cell	Percent	2021	0.0001873	0.000243357	0.000138931
Prevalence	Slovakia	Female	55+ years	Non-melanoma skin cancer (basal-cell	Percent	2021	0.0001791	0.000229991	0.000134497
Prevalence	Slovakia	Both	55+ years	Non-melanoma skin cancer (basal-cell	Percent	2021	0.0001827	0.000232254	0.000135279
Prevalence	Slovakia	Male	55+ years	Non-melanoma skin cancer (basal-cell	Rate	2021	18.721589	24.32668768	13.88735475
Prevalence	Slovakia	Female	55+ years	Non-melanoma skin cancer (basal-cell	Rate	2021	17.905433	22.98847598	13.44462498
Prevalence	Slovakia	Both	55+ years	Non-melanoma skin cancer (basal-cell	Rate	2021	18.260597	23.21449905	13.52145055
Prevalence	Belarus	Male	55+ years	Non-melanoma skin cancer (basal-cell	Number	1990	96.597217	114.6436639	81.76068228
Prevalence	Belarus	Female	55+ years	Non-melanoma skin cancer (basal-cell	Number	1990	182.19106	215.0496688	153.5466303
Prevalence	Belarus	Both	55+ years	Non-melanoma skin cancer (basal-cell	Number	1990	278.78827	330.3345336	237.8429485
Prevalence	Belarus	Male	55+ years	Non-melanoma skin cancer (basal-cell	Percent	1990	0.0001135	0.000134755	9.61E-05
Prevalence	Belarus	Female	55+ years	Non-melanoma skin cancer (basal-cell	Percent	1990	0.0001245	0.000146946	0.000104922
Prevalence	Belarus	Both	55+ years	Non-melanoma skin cancer (basal-cell	Percent	1990	0.0001205	0.000142743	0.000102779
Prevalence	Belarus	Male	55+ years	Non-melanoma skin cancer (basal-cell	Rate	1990	11.350064	13.47050076	9.606787633
Prevalence	Belarus	Female	55+ years	Non-melanoma skin cancer (basal-cell	Rate	1990	12.445916	14.69056802	10.48914527
Prevalence	Belarus	Both	55+ years	Non-melanoma skin cancer (basal-cell	Rate	1990	12.043032	14.26971587	10.27428547
Prevalence	Belarus	Male	55+ years	Non-melanoma skin cancer (basal-cell	Number	2021	132.63425	171.605656	100.4023241
Prevalence	Belarus	Female	55+ years	Non-melanoma skin cancer (basal-cell	Number	2021	235.89103	304.4616198	177.3720447
Prevalence	Belarus	Both	55+ years	Non-melanoma skin cancer (basal-cell	Number	2021	368.52529	473.3052358	278.7702389
Prevalence	Belarus	Male	55+ years	Non-melanoma skin cancer (basal-cell	Percent	2021	0.0001196	0.000154783	9.06E-05
Prevalence	Belarus	Female	55+ years	Non-melanoma skin cancer (basal-cell	Percent	2021	0.0001335	0.000172321	0.000100401
Prevalence	Belarus	Both	55+ years	Non-melanoma skin cancer (basal-cell	Percent	2021	0.0001282	0.00016459	9.69E-05
Prevalence	Belarus	Male	55+ years	Non-melanoma skin cancer (basal-cell	Rate	2021	11.956245	15.4693013	9.050714522
Prevalence	Belarus	Female	55+ years	Non-melanoma skin cancer (basal-cell	Rate	2021	13.347747	17.22777117	10.0364867
Prevalence	Belarus	Both	55+ years	Non-melanoma skin cancer (basal-cell	Rate	2021	12.811129	16.45361904	9.690954092
Prevalence	Japan	Male	55+ years	Non-melanoma skin cancer (basal-cell	Number	1990	334.81362	439.1507918	244.749356
Prevalence	Japan	Female	55+ years	Non-melanoma skin cancer (basal-cell	Number	1990	396.26009	516.9975852	288.3435742
Prevalence	Japan	Both	55+ years	Non-melanoma skin cancer (basal-cell	Number	1990	731.07371	963.2437258	538.4291602
Prevalence	Japan	Male	55+ years	Non-melanoma skin cancer (basal-cell	Percent	1990	2.55E-05	3.35E-05	1.87E-05
Prevalence	Japan	Female	55+ years	Non-melanoma skin cancer (basal-cell	Percent	1990	2.41E-05	3.14E-05	1.75E-05
Prevalence	Japan	Both	55+ years	Non-melanoma skin cancer (basal-cell	Percent	1990	2.47E-05	3.26E-05	1.82E-05
Prevalence	Japan	Male	55+ years	Non-melanoma skin cancer (basal-cell	Rate	1990	2.5478258	3.341798754	1.862465258
Prevalence	Japan	Female	55+ years	Non-melanoma skin cancer (basal-cell	Rate	1990	2.4056437	3.138625352	1.750496478
Prevalence	Japan	Both	55+ years	Non-melanoma skin cancer (basal-cell	Rate	1990	2.4687383	3.252745442	1.818203379
Prevalence	Japan	Male	55+ years	Non-melanoma skin cancer (basal-cell	Number	2021	1114.3731	1449.988369	804.5664399
Prevalence	Japan	Female	55+ years	Non-melanoma skin cancer (basal-cell	Number	2021	1366.3594	1757.807041	1005.36484
Prevalence	Japan	Both	55+ years	Non-melanoma skin cancer (basal-cell	Number	2021	2480.7325	3203.104001	1812.379493
Prevalence	Japan	Male	55+ years	Non-melanoma skin cancer (basal-cell	Percent	2021	4.70E-05	6.12E-05	3.39E-05
Prevalence	Japan	Female	55+ years	Non-melanoma skin cancer (basal-cell	Percent	2021	4.81E-05	6.19E-05	3.54E-05
Prevalence	Japan	Both	55+ years	Non-melanoma skin cancer (basal-cell	Percent	2021	4.76E-05	6.15E-05	3.48E-05
Prevalence	Japan	Male	55+ years	Non-melanoma skin cancer (basal-cell	Rate	2021	4.691738	6.104746699	3.387388771
Prevalence	Japan	Female	55+ years	Non-melanoma skin cancer (basal-cell	Rate	2021	4.8028227	6.178781209	3.533908578
Prevalence	Japan	Both	55+ years	Non-melanoma skin cancer (basal-cell	Rate	2021	4.7522783	6.136107716	3.471930911
Prevalence	Slovenia	Male	55+ years	Non-melanoma skin cancer (basal-cell	Number	1990	29.399794	35.70264814	24.2197142
Prevalence	Slovenia	Female	55+ years	Non-melanoma skin cancer (basal-cell	Number	1990	44.184471	53.55843166	36.59966792
Prevalence	Slovenia	Both	55+ years	Non-melanoma skin cancer (basal-cell	Number	1990	73.584266	87.97084155	61.30341419
Prevalence	Slovenia	Male	55+ years	Non-melanoma skin cancer (basal-cell	Percent	1990	0.0001709	0.000207495	0.000140758
Prevalence	Slovenia	Female	55+ years	Non-melanoma skin cancer (basal-cell	Percent	1990	0.0001709	0.000207135	0.000141558
Prevalence	Slovenia	Both	55+ years	Non-melanoma skin cancer (basal-cell	Percent	1990	0.0001709	0.000204299	0.000142368
Prevalence	Slovenia	Male	55+ years	Non-melanoma skin cancer (basal-cell	Rate	1990	17.085041	20.7478047	14.07475149
Prevalence	Slovenia	Female	55+ years	Non-melanoma skin cancer (basal-cell	Rate	1990	17.084221	20.70872501	14.15150584
Prevalence	Slovenia	Both	55+ years	Non-melanoma skin cancer (basal-cell	Rate	1990	17.084548	20.42477524	14.2332213
Prevalence	Slovenia	Male	55+ years	Non-melanoma skin cancer (basal-cell	Number	2021	95.387282	125.856993	69.88807345
Prevalence	Slovenia	Female	55+ years	Non-melanoma skin cancer (basal-cell	Number	2021	98.878373	124.9825597	76.55907497
Prevalence	Slovenia	Both	55+ years	Non-melanoma skin cancer (basal-cell	Number	2021	194.26566	246.961001	146.5858612
Prevalence	Slovenia	Male	55+ years	Non-melanoma skin cancer (basal-cell	Percent	2021	0.0002847	0.000375688	0.000208634

Prevalence	Slovenia	Female	55+ years	Non-melanoma skin cancer (basal-cell	Percent	2021	0.0002491	0.00031493	0.000192888
Prevalence	Slovenia	Both	55+ years	Non-melanoma skin cancer (basal-cell	Percent	2021	0.0002654	0.000337456	0.000200263
Prevalence	Slovenia	Male	55+ years	Non-melanoma skin cancer (basal-cell	Rate	2021	28.462533	37.5543654	20.85384518
Prevalence	Slovenia	Female	55+ years	Non-melanoma skin cancer (basal-cell	Rate	2021	24.902878	31.47731255	19.28168167
Prevalence	Slovenia	Both	55+ years	Non-melanoma skin cancer (basal-cell	Rate	2021	26.532181	33.72914229	20.02022729
Prevalence	Republic o	Male	55+ years	Non-melanoma skin cancer (basal-cell	Number	1990	41.488731	51.04555474	33.91798892
Prevalence	Republic o	Female	55+ years	Non-melanoma skin cancer (basal-cell	Number	1990	58.851372	71.58333122	48.08782463
Prevalence	Republic o	Both	55+ years	Non-melanoma skin cancer (basal-cell	Number	1990	100.3401	121.2956183	82.9967487
Prevalence	Republic o	Male	55+ years	Non-melanoma skin cancer (basal-cell	Percent	1990	1.99E-05	2.45E-05	1.63E-05
Prevalence	Republic o	Female	55+ years	Non-melanoma skin cancer (basal-cell	Percent	1990	2.03E-05	2.47E-05	1.66E-05
Prevalence	Republic o	Both	55+ years	Non-melanoma skin cancer (basal-cell	Percent	1990	2.02E-05	2.44E-05	1.67E-05
Prevalence	Republic o	Male	55+ years	Non-melanoma skin cancer (basal-cell	Rate	1990	1.993518	2.452719855	1.629746709
Prevalence	Republic o	Female	55+ years	Non-melanoma skin cancer (basal-cell	Rate	1990	2.032463	2.472167872	1.660738234
Prevalence	Republic o	Both	55+ years	Non-melanoma skin cancer (basal-cell	Rate	1990	2.0161769	2.437245164	1.667689461
Prevalence	Republic o	Male	55+ years	Non-melanoma skin cancer (basal-cell	Number	2021	256.35822	311.639028	211.0034596
Prevalence	Republic o	Female	55+ years	Non-melanoma skin cancer (basal-cell	Number	2021	383.52172	462.3516537	313.3301044
Prevalence	Republic o	Both	55+ years	Non-melanoma skin cancer (basal-cell	Number	2021	639.87994	764.9966807	527.6742363
Prevalence	Republic o	Male	55+ years	Non-melanoma skin cancer (basal-cell	Percent	2021	3.28E-05	3.99E-05	2.70E-05
Prevalence	Republic o	Female	55+ years	Non-melanoma skin cancer (basal-cell	Percent	2021	4.31E-05	5.20E-05	3.52E-05
Prevalence	Republic o	Both	55+ years	Non-melanoma skin cancer (basal-cell	Percent	2021	3.83E-05	4.58E-05	3.16E-05
Prevalence	Republic o	Male	55+ years	Non-melanoma skin cancer (basal-cell	Rate	2021	3.2776525	3.984442022	2.697772023
Prevalence	Republic o	Female	55+ years	Non-melanoma skin cancer (basal-cell	Rate	2021	4.3080897	5.193584354	3.519629085
Prevalence	Republic o	Both	55+ years	Non-melanoma skin cancer (basal-cell	Rate	2021	3.826173	4.574310744	3.155237126
Prevalence	Republic o	Male	55+ years	Non-melanoma skin cancer (basal-cell	Number	1990	34.794261	45.64571525	26.1256405
Prevalence	Republic o	Female	55+ years	Non-melanoma skin cancer (basal-cell	Number	1990	51.515061	66.67903409	38.26878679
Prevalence	Republic o	Both	55+ years	Non-melanoma skin cancer (basal-cell	Number	1990	86.309322	111.5723976	65.09554144
Prevalence	Republic o	Male	55+ years	Non-melanoma skin cancer (basal-cell	Percent	1990	0.0001117	0.000146483	8.38E-05
Prevalence	Republic o	Female	55+ years	Non-melanoma skin cancer (basal-cell	Percent	1990	0.0001119	0.000144891	8.32E-05
Prevalence	Republic o	Both	55+ years	Non-melanoma skin cancer (basal-cell	Percent	1990	0.0001118	0.000144554	8.43E-05
Prevalence	Republic o	Male	55+ years	Non-melanoma skin cancer (basal-cell	Rate	1990	11.161664	14.64270632	8.380854134
Prevalence	Republic o	Female	55+ years	Non-melanoma skin cancer (basal-cell	Rate	1990	11.192022	14.48650509	8.314172247
Prevalence	Republic o	Both	55+ years	Non-melanoma skin cancer (basal-cell	Rate	1990	11.179764	14.45212416	8.431913871
Prevalence	Republic o	Male	55+ years	Non-melanoma skin cancer (basal-cell	Number	2021	51.742311	66.35915847	38.27619078
Prevalence	Republic o	Female	55+ years	Non-melanoma skin cancer (basal-cell	Number	2021	75.366116	97.2093076	56.30860556
Prevalence	Republic o	Both	55+ years	Non-melanoma skin cancer (basal-cell	Number	2021	127.10843	162.4514904	95.75725545
Prevalence	Republic o	Male	55+ years	Non-melanoma skin cancer (basal-cell	Percent	2021	0.0001211	0.000155308	8.96E-05
Prevalence	Republic o	Female	55+ years	Non-melanoma skin cancer (basal-cell	Percent	2021	0.00012	0.000154765	8.96E-05
Prevalence	Republic o	Both	55+ years	Non-melanoma skin cancer (basal-cell	Percent	2021	0.0001204	0.000153939	9.07E-05
Prevalence	Republic o	Male	55+ years	Non-melanoma skin cancer (basal-cell	Rate	2021	12.101998	15.52073007	8.952410474
Prevalence	Republic o	Female	55+ years	Non-melanoma skin cancer (basal-cell	Rate	2021	11.995601	15.47225938	8.962324413
Prevalence	Republic o	Both	55+ years	Non-melanoma skin cancer (basal-cell	Rate	2021	12.038686	15.38609629	9.069355718
Prevalence	North Mace	Male	55+ years	Non-melanoma skin cancer (basal-cell	Number	1990	24.380637	31.29021041	18.46312073
Prevalence	North Mace	Female	55+ years	Non-melanoma skin cancer (basal-cell	Number	1990	22.624371	30.05634635	17.31967678
Prevalence	North Mace	Both	55+ years	Non-melanoma skin cancer (basal-cell	Number	1990	47.005009	61.04389471	35.15480942
Prevalence	North Mace	Male	55+ years	Non-melanoma skin cancer (basal-cell	Percent	1990	0.0001565	0.000200877	0.000118534
Prevalence	North Mace	Female	55+ years	Non-melanoma skin cancer (basal-cell	Percent	1990	0.0001307	0.00017363	0.000100061
Prevalence	North Mace	Both	55+ years	Non-melanoma skin cancer (basal-cell	Percent	1990	0.0001429	0.000185623	0.000106901
Prevalence	North Mace	Male	55+ years	Non-melanoma skin cancer (basal-cell	Rate	1990	15.646757	20.0811125	11.84907354
Prevalence	North Mace	Female	55+ years	Non-melanoma skin cancer (basal-cell	Rate	1990	13.066571	17.35886352	10.002876
Prevalence	North Mace	Both	55+ years	Non-melanoma skin cancer (basal-cell	Rate	1990	14.28871	18.55628851	10.68645422
Prevalence	North Mace	Male	55+ years	Non-melanoma skin cancer (basal-cell	Number	2021	46.163211	61.19845679	33.87971132
Prevalence	North Mace	Female	55+ years	Non-melanoma skin cancer (basal-cell	Number	2021	42.745616	55.23974974	32.47643384
Prevalence	North Mace	Both	55+ years	Non-melanoma skin cancer (basal-cell	Number	2021	88.908827	116.3156307	66.29396241
Prevalence	North Mace	Male	55+ years	Non-melanoma skin cancer (basal-cell	Percent	2021	0.0001617	0.000214309	0.000118653
Prevalence	North Mace	Female	55+ years	Non-melanoma skin cancer (basal-cell	Percent	2021	0.0001392	0.000179894	0.000105758
Prevalence	North Mace	Both	55+ years	Non-melanoma skin cancer (basal-cell	Percent	2021	0.00015	0.000196251	0.000111864

Prevalence	North Mace	Male	55+ years	Non-melanoma skin cancer (basal-cell	Rate	2021	16.157517	21.41998122	11.85818758
Prevalence	North Mace	Female	55+ years	Non-melanoma skin cancer (basal-cell	Rate	2021	13.914659	17.98178017	10.57180919
Prevalence	North Mace	Both	55+ years	Non-melanoma skin cancer (basal-cell	Rate	2021	14.995439	19.61789453	11.18119685
Prevalence	Montenegro	Male	55+ years	Non-melanoma skin cancer (basal-cell	Number	1990	7.847054	10.14596996	5.798738684
Prevalence	Montenegro	Female	55+ years	Non-melanoma skin cancer (basal-cell	Number	1990	8.9649033	11.786238	6.599515785
Prevalence	Montenegro	Both	55+ years	Non-melanoma skin cancer (basal-cell	Number	1990	16.811957	21.91178454	12.49611505
Prevalence	Montenegro	Male	55+ years	Non-melanoma skin cancer (basal-cell	Percent	1990	0.0001623	0.000209843	0.00011992
Prevalence	Montenegro	Female	55+ years	Non-melanoma skin cancer (basal-cell	Percent	1990	0.0001481	0.000194704	0.000109021
Prevalence	Montenegro	Both	55+ years	Non-melanoma skin cancer (basal-cell	Percent	1990	0.0001544	0.000201213	0.00011476
Prevalence	Montenegro	Male	55+ years	Non-melanoma skin cancer (basal-cell	Rate	1990	16.221481	20.97381471	11.9871901
Prevalence	Montenegro	Female	55+ years	Non-melanoma skin cancer (basal-cell	Rate	1990	14.801549	19.4597274	10.89616366
Prevalence	Montenegro	Both	55+ years	Non-melanoma skin cancer (basal-cell	Rate	1990	15.432055	20.11329489	11.47045083
Prevalence	Montenegro	Male	55+ years	Non-melanoma skin cancer (basal-cell	Number	2021	13.313093	17.47169194	9.732215251
Prevalence	Montenegro	Female	55+ years	Non-melanoma skin cancer (basal-cell	Number	2021	14.605926	19.03233873	10.66694682
Prevalence	Montenegro	Both	55+ years	Non-melanoma skin cancer (basal-cell	Number	2021	27.919019	36.20015286	20.52098136
Prevalence	Montenegro	Male	55+ years	Non-melanoma skin cancer (basal-cell	Percent	2021	0.0001678	0.00022026	0.000122683
Prevalence	Montenegro	Female	55+ years	Non-melanoma skin cancer (basal-cell	Percent	2021	0.0001538	0.000200418	0.000112317
Prevalence	Montenegro	Both	55+ years	Non-melanoma skin cancer (basal-cell	Percent	2021	0.0001602	0.000207707	0.000117748
Prevalence	Montenegro	Male	55+ years	Non-melanoma skin cancer (basal-cell	Rate	2021	16.77436	22.01415241	12.26249127
Prevalence	Montenegro	Female	55+ years	Non-melanoma skin cancer (basal-cell	Rate	2021	15.373452	20.03246853	11.2274839
Prevalence	Montenegro	Both	55+ years	Non-melanoma skin cancer (basal-cell	Rate	2021	16.011074	20.7601607	11.7684274
Prevalence	Hungary	Male	55+ years	Non-melanoma skin cancer (basal-cell	Number	1990	188.33346	225.2751638	155.6764337
Prevalence	Hungary	Female	55+ years	Non-melanoma skin cancer (basal-cell	Number	1990	254.38409	307.251276	208.0113974
Prevalence	Hungary	Both	55+ years	Non-melanoma skin cancer (basal-cell	Number	1990	442.71755	522.6483271	369.5109302
Prevalence	Hungary	Male	55+ years	Non-melanoma skin cancer (basal-cell	Percent	1990	0.0001758	0.000210337	0.000145359
Prevalence	Hungary	Female	55+ years	Non-melanoma skin cancer (basal-cell	Percent	1990	0.0001692	0.000204307	0.000138306
Prevalence	Hungary	Both	55+ years	Non-melanoma skin cancer (basal-cell	Percent	1990	0.0001719	0.000202987	0.000143502
Prevalence	Hungary	Male	55+ years	Non-melanoma skin cancer (basal-cell	Rate	1990	17.578853	21.0269536	14.53067925
Prevalence	Hungary	Female	55+ years	Non-melanoma skin cancer (basal-cell	Rate	1990	16.9099	20.42418776	13.82732691
Prevalence	Hungary	Both	55+ years	Non-melanoma skin cancer (basal-cell	Rate	1990	17.18815	20.29139747	14.34596222
Prevalence	Hungary	Male	55+ years	Non-melanoma skin cancer (basal-cell	Number	2021	244.91202	316.3888896	184.7053419
Prevalence	Hungary	Female	55+ years	Non-melanoma skin cancer (basal-cell	Number	2021	330.7918	432.780263	239.4298188
Prevalence	Hungary	Both	55+ years	Non-melanoma skin cancer (basal-cell	Number	2021	575.70382	742.7719562	424.2653802
Prevalence	Hungary	Male	55+ years	Non-melanoma skin cancer (basal-cell	Percent	2021	0.0001859	0.000240104	0.000140164
Prevalence	Hungary	Female	55+ years	Non-melanoma skin cancer (basal-cell	Percent	2021	0.0001778	0.000232586	0.000128665
Prevalence	Hungary	Both	55+ years	Non-melanoma skin cancer (basal-cell	Percent	2021	0.0001811	0.000233711	0.000133482
Prevalence	Hungary	Male	55+ years	Non-melanoma skin cancer (basal-cell	Rate	2021	18.573162	23.9936858	14.0073248
Prevalence	Hungary	Female	55+ years	Non-melanoma skin cancer (basal-cell	Rate	2021	17.770273	23.24913502	12.86226906
Prevalence	Hungary	Both	55+ years	Non-melanoma skin cancer (basal-cell	Rate	2021	18.10319	23.35670012	13.34115965
Prevalence	Brunei Dar	Male	55+ years	Non-melanoma skin cancer (basal-cell	Number	1990	0.2397806	0.322848334	0.173781687
Prevalence	Brunei Dar	Female	55+ years	Non-melanoma skin cancer (basal-cell	Number	1990	0.1898956	0.251530076	0.135398274
Prevalence	Brunei Dar	Both	55+ years	Non-melanoma skin cancer (basal-cell	Number	1990	0.4296762	0.575837752	0.313153852
Prevalence	Brunei Dar	Male	55+ years	Non-melanoma skin cancer (basal-cell	Percent	1990	2.95E-05	3.97E-05	2.14E-05
Prevalence	Brunei Dar	Female	55+ years	Non-melanoma skin cancer (basal-cell	Percent	1990	2.53E-05	3.35E-05	1.80E-05
Prevalence	Brunei Dar	Both	55+ years	Non-melanoma skin cancer (basal-cell	Percent	1990	2.75E-05	3.68E-05	2.00E-05
Prevalence	Brunei Dar	Male	55+ years	Non-melanoma skin cancer (basal-cell	Rate	1990	2.9479736	3.969247302	2.136552733
Prevalence	Brunei Dar	Female	55+ years	Non-melanoma skin cancer (basal-cell	Rate	1990	2.5242405	3.343534036	1.799819511
Prevalence	Brunei Dar	Both	55+ years	Non-melanoma skin cancer (basal-cell	Rate	1990	2.7443733	3.67791806	2.000136678
Prevalence	Brunei Dar	Male	55+ years	Non-melanoma skin cancer (basal-cell	Number	2021	0.7725601	1.061639847	0.523052596
Prevalence	Brunei Dar	Female	55+ years	Non-melanoma skin cancer (basal-cell	Number	2021	0.6757456	0.929040962	0.488130342
Prevalence	Brunei Dar	Both	55+ years	Non-melanoma skin cancer (basal-cell	Number	2021	1.4483057	1.982505273	1.009473711
Prevalence	Brunei Dar	Male	55+ years	Non-melanoma skin cancer (basal-cell	Percent	2021	2.63E-05	3.61E-05	1.78E-05
Prevalence	Brunei Dar	Female	55+ years	Non-melanoma skin cancer (basal-cell	Percent	2021	2.22E-05	3.05E-05	1.61E-05
Prevalence	Brunei Dar	Both	55+ years	Non-melanoma skin cancer (basal-cell	Percent	2021	2.42E-05	3.32E-05	1.69E-05
Prevalence	Brunei Dar	Male	55+ years	Non-melanoma skin cancer (basal-cell	Rate	2021	2.6257834	3.608310083	1.777755388
Prevalence	Brunei Dar	Female	55+ years	Non-melanoma skin cancer (basal-cell	Rate	2021	2.2190143	3.050785778	1.602922979

Prevalence	Brunei Dar	Both	55+ years	Non-melanoma skin cancer (basal-cell	Rate	2021	2.4188986	3.311096035	1.685980081
Prevalence	Serbia	Male	55+ years	Non-melanoma skin cancer (basal-cell	Number	1990	118.46439	145.6861103	100.8355572
Prevalence	Serbia	Female	55+ years	Non-melanoma skin cancer (basal-cell	Number	1990	114.79048	139.005537	95.63037027
Prevalence	Serbia	Both	55+ years	Non-melanoma skin cancer (basal-cell	Number	1990	233.25488	282.1890895	197.8205708
Prevalence	Serbia	Male	55+ years	Non-melanoma skin cancer (basal-cell	Percent	1990	0.0001239	0.000152391	0.000105484
Prevalence	Serbia	Female	55+ years	Non-melanoma skin cancer (basal-cell	Percent	1990	0.0001022	0.000123777	8.51E-05
Prevalence	Serbia	Both	55+ years	Non-melanoma skin cancer (basal-cell	Percent	1990	0.0001122	0.000135724	9.51E-05
Prevalence	Serbia	Male	55+ years	Non-melanoma skin cancer (basal-cell	Rate	1990	12.388336	15.23502918	10.54481208
Prevalence	Serbia	Female	55+ years	Non-melanoma skin cancer (basal-cell	Rate	1990	10.217192	12.37250859	8.511801776
Prevalence	Serbia	Both	55+ years	Non-melanoma skin cancer (basal-cell	Rate	1990	11.215467	13.56834373	9.511698369
Prevalence	Serbia	Male	55+ years	Non-melanoma skin cancer (basal-cell	Number	2021	217.21847	285.009231	159.4340701
Prevalence	Serbia	Female	55+ years	Non-melanoma skin cancer (basal-cell	Number	2021	219.81844	287.5524881	162.553163
Prevalence	Serbia	Both	55+ years	Non-melanoma skin cancer (basal-cell	Number	2021	437.03692	566.0255326	325.5658105
Prevalence	Serbia	Male	55+ years	Non-melanoma skin cancer (basal-cell	Percent	2021	0.0001709	0.000224248	0.000125447
Prevalence	Serbia	Female	55+ years	Non-melanoma skin cancer (basal-cell	Percent	2021	0.0001434	0.000187553	0.000106025
Prevalence	Serbia	Both	55+ years	Non-melanoma skin cancer (basal-cell	Percent	2021	0.0001559	0.00020187	0.000116108
Prevalence	Serbia	Male	55+ years	Non-melanoma skin cancer (basal-cell	Rate	2021	17.083404	22.41488795	12.53888095
Prevalence	Serbia	Female	55+ years	Non-melanoma skin cancer (basal-cell	Rate	2021	14.332537	18.74891216	10.59874319
Prevalence	Serbia	Both	55+ years	Non-melanoma skin cancer (basal-cell	Rate	2021	15.579418	20.17758234	11.60571488
Prevalence	Russian Fe	Male	55+ years	Non-melanoma skin cancer (basal-cell	Number	1990	796.6481	1034.912965	597.3200254
Prevalence	Russian Fe	Female	55+ years	Non-melanoma skin cancer (basal-cell	Number	1990	1734.2373	2264.461261	1278.22347
Prevalence	Russian Fe	Both	55+ years	Non-melanoma skin cancer (basal-cell	Number	1990	2530.8854	3284.16934	1865.989784
Prevalence	Russian Fe	Male	55+ years	Non-melanoma skin cancer (basal-cell	Percent	1990	7.24E-05	9.40E-05	5.43E-05
Prevalence	Russian Fe	Female	55+ years	Non-melanoma skin cancer (basal-cell	Percent	1990	8.44E-05	0.000110192	6.22E-05
Prevalence	Russian Fe	Both	55+ years	Non-melanoma skin cancer (basal-cell	Percent	1990	8.02E-05	0.000104063	5.91E-05
Prevalence	Russian Fe	Male	55+ years	Non-melanoma skin cancer (basal-cell	Rate	1990	7.2355243	9.399555236	5.425135023
Prevalence	Russian Fe	Female	55+ years	Non-melanoma skin cancer (basal-cell	Rate	1990	8.4380841	11.01793574	6.219308893
Prevalence	Russian Fe	Both	55+ years	Non-melanoma skin cancer (basal-cell	Rate	1990	8.0185873	10.40521202	5.912003103
Prevalence	Russian Fe	Male	55+ years	Non-melanoma skin cancer (basal-cell	Number	2021	1690.6872	2155.169949	1268.064363
Prevalence	Russian Fe	Female	55+ years	Non-melanoma skin cancer (basal-cell	Number	2021	2944.3023	3800.209024	2183.717423
Prevalence	Russian Fe	Both	55+ years	Non-melanoma skin cancer (basal-cell	Number	2021	4634.9895	5972.641836	3461.378433
Prevalence	Russian Fe	Male	55+ years	Non-melanoma skin cancer (basal-cell	Percent	2021	0.0001033	0.000131638	7.75E-05
Prevalence	Russian Fe	Female	55+ years	Non-melanoma skin cancer (basal-cell	Percent	2021	0.0001127	0.000145455	8.36E-05
Prevalence	Russian Fe	Both	55+ years	Non-melanoma skin cancer (basal-cell	Percent	2021	0.0001091	0.000140543	8.14E-05
Prevalence	Russian Fe	Male	55+ years	Non-melanoma skin cancer (basal-cell	Rate	2021	10.323707	13.15993999	7.743078885
Prevalence	Russian Fe	Female	55+ years	Non-melanoma skin cancer (basal-cell	Rate	2021	11.268558	14.54431977	8.357615147
Prevalence	Russian Fe	Both	55+ years	Non-melanoma skin cancer (basal-cell	Rate	2021	10.904518	14.05154856	8.143419353
Prevalence	Croatia	Male	55+ years	Non-melanoma skin cancer (basal-cell	Number	1990	58.151004	75.7098247	40.39317731
Prevalence	Croatia	Female	55+ years	Non-melanoma skin cancer (basal-cell	Number	1990	83.358087	111.3743506	61.56565864
Prevalence	Croatia	Both	55+ years	Non-melanoma skin cancer (basal-cell	Number	1990	141.50909	184.7117718	103.8397421
Prevalence	Croatia	Male	55+ years	Non-melanoma skin cancer (basal-cell	Percent	1990	0.0001267	0.00016496	8.80E-05
Prevalence	Croatia	Female	55+ years	Non-melanoma skin cancer (basal-cell	Percent	1990	0.0001275	0.000170331	9.42E-05
Prevalence	Croatia	Both	55+ years	Non-melanoma skin cancer (basal-cell	Percent	1990	0.0001272	0.000166009	9.33E-05
Prevalence	Croatia	Male	55+ years	Non-melanoma skin cancer (basal-cell	Rate	1990	12.667404	16.49235367	8.799103269
Prevalence	Croatia	Female	55+ years	Non-melanoma skin cancer (basal-cell	Rate	1990	12.743514	17.02654983	9.411958398
Prevalence	Croatia	Both	55+ years	Non-melanoma skin cancer (basal-cell	Rate	1990	12.712127	16.59313589	9.328192429
Prevalence	Croatia	Male	55+ years	Non-melanoma skin cancer (basal-cell	Number	2021	108.00722	142.4259682	77.61486763
Prevalence	Croatia	Female	55+ years	Non-melanoma skin cancer (basal-cell	Number	2021	125.2133	160.4290431	93.82830513
Prevalence	Croatia	Both	55+ years	Non-melanoma skin cancer (basal-cell	Number	2021	233.22052	302.6963264	172.1849925
Prevalence	Croatia	Male	55+ years	Non-melanoma skin cancer (basal-cell	Percent	2021	0.0001644	0.000216792	0.000118142
Prevalence	Croatia	Female	55+ years	Non-melanoma skin cancer (basal-cell	Percent	2021	0.0001502	0.000192448	0.00011255
Prevalence	Croatia	Both	55+ years	Non-melanoma skin cancer (basal-cell	Percent	2021	0.0001565	0.000203067	0.000115503
Prevalence	Croatia	Male	55+ years	Non-melanoma skin cancer (basal-cell	Rate	2021	16.431907	21.66827501	11.80810155
Prevalence	Croatia	Female	55+ years	Non-melanoma skin cancer (basal-cell	Rate	2021	15.013144	19.23553188	11.25006619
Prevalence	Croatia	Both	55+ years	Non-melanoma skin cancer (basal-cell	Rate	2021	15.638464	20.2971225	11.54576247
Prevalence	Ukraine	Male	55+ years	Non-melanoma skin cancer (basal-cell	Number	1990	501.16148	654.1274611	372.8156674

Prevalence	Ukraine	Female	55+ years	Non-melanoma skin cancer (basal-cell	Number	1990	997.14254	1303.899176	748.3494695
Prevalence	Ukraine	Both	55+ years	Non-melanoma skin cancer (basal-cell	Number	1990	1498.304	1934.70929	1136.066378
Prevalence	Ukraine	Male	55+ years	Non-melanoma skin cancer (basal-cell	Percent	1990	0.0001114	0.000145448	8.29E-05
Prevalence	Ukraine	Female	55+ years	Non-melanoma skin cancer (basal-cell	Percent	1990	0.0001252	0.000163655	9.39E-05
Prevalence	Ukraine	Both	55+ years	Non-melanoma skin cancer (basal-cell	Percent	1990	0.0001202	0.00015522	9.11E-05
Prevalence	Ukraine	Male	55+ years	Non-melanoma skin cancer (basal-cell	Rate	1990	11.140661	14.54104565	8.287573847
Prevalence	Ukraine	Female	55+ years	Non-melanoma skin cancer (basal-cell	Rate	1990	12.513557	16.36317345	9.391348958
Prevalence	Ukraine	Both	55+ years	Non-melanoma skin cancer (basal-cell	Rate	1990	12.018172	15.51865847	9.112597025
Prevalence	Ukraine	Male	55+ years	Non-melanoma skin cancer (basal-cell	Number	2021	639.05692	818.6459294	478.3946823
Prevalence	Ukraine	Female	55+ years	Non-melanoma skin cancer (basal-cell	Number	2021	1075.3161	1406.016129	799.0090468
Prevalence	Ukraine	Both	55+ years	Non-melanoma skin cancer (basal-cell	Number	2021	1714.3731	2219.404507	1267.374906
Prevalence	Ukraine	Male	55+ years	Non-melanoma skin cancer (basal-cell	Percent	2021	0.0001227	0.000157123	9.18E-05
Prevalence	Ukraine	Female	55+ years	Non-melanoma skin cancer (basal-cell	Percent	2021	0.0001286	0.000168096	9.55E-05
Prevalence	Ukraine	Both	55+ years	Non-melanoma skin cancer (basal-cell	Percent	2021	0.0001263	0.000163499	9.34E-05
Prevalence	Ukraine	Male	55+ years	Non-melanoma skin cancer (basal-cell	Rate	2021	12.261742	15.70755987	9.179075891
Prevalence	Ukraine	Female	55+ years	Non-melanoma skin cancer (basal-cell	Rate	2021	12.852987	16.8057619	9.550356868
Prevalence	Ukraine	Both	55+ years	Non-melanoma skin cancer (basal-cell	Rate	2021	12.626044	16.34550799	9.33398422
Prevalence	Greece	Male	55+ years	Non-melanoma skin cancer (basal-cell	Number	1990	312.69016	408.0654476	227.8257064
Prevalence	Greece	Female	55+ years	Non-melanoma skin cancer (basal-cell	Number	1990	281.3273	368.1194651	206.0371014
Prevalence	Greece	Both	55+ years	Non-melanoma skin cancer (basal-cell	Number	1990	594.01747	775.0992864	438.1109686
Prevalence	Greece	Male	55+ years	Non-melanoma skin cancer (basal-cell	Percent	1990	0.0002531	0.000330389	0.000184375
Prevalence	Greece	Female	55+ years	Non-melanoma skin cancer (basal-cell	Percent	1990	0.0001959	0.000256329	0.000143465
Prevalence	Greece	Both	55+ years	Non-melanoma skin cancer (basal-cell	Percent	1990	0.0002223	0.000290092	0.000163995
Prevalence	Greece	Male	55+ years	Non-melanoma skin cancer (basal-cell	Rate	1990	25.283382	32.99520012	18.42144396
Prevalence	Greece	Female	55+ years	Non-melanoma skin cancer (basal-cell	Rate	1990	19.578401	25.61852449	14.33873247
Prevalence	Greece	Both	55+ years	Non-melanoma skin cancer (basal-cell	Rate	1990	22.217317	28.9901018	16.38613504
Prevalence	Greece	Male	55+ years	Non-melanoma skin cancer (basal-cell	Number	2021	504.1603	638.3896784	380.1201691
Prevalence	Greece	Female	55+ years	Non-melanoma skin cancer (basal-cell	Number	2021	435.76676	555.5386005	326.739809
Prevalence	Greece	Both	55+ years	Non-melanoma skin cancer (basal-cell	Number	2021	939.92707	1188.682803	699.4902044
Prevalence	Greece	Male	55+ years	Non-melanoma skin cancer (basal-cell	Percent	2021	0.0002988	0.000378363	0.000225241
Prevalence	Greece	Female	55+ years	Non-melanoma skin cancer (basal-cell	Percent	2021	0.0002195	0.000279774	0.000164537
Prevalence	Greece	Both	55+ years	Non-melanoma skin cancer (basal-cell	Percent	2021	0.0002559	0.000323647	0.000190437
Prevalence	Greece	Male	55+ years	Non-melanoma skin cancer (basal-cell	Rate	2021	29.850467	37.79795809	22.50626334
Prevalence	Greece	Female	55+ years	Non-melanoma skin cancer (basal-cell	Rate	2021	21.936444	27.96574365	16.44804111
Prevalence	Greece	Both	55+ years	Non-melanoma skin cancer (basal-cell	Rate	2021	25.573118	32.34115358	19.0314187
Prevalence	Czechia	Male	55+ years	Non-melanoma skin cancer (basal-cell	Number	1990	296.04323	349.4707679	252.8651361
Prevalence	Czechia	Female	55+ years	Non-melanoma skin cancer (basal-cell	Number	1990	365.43873	436.8321303	309.1015835
Prevalence	Czechia	Both	55+ years	Non-melanoma skin cancer (basal-cell	Number	1990	661.48196	775.0678733	565.6912578
Prevalence	Czechia	Male	55+ years	Non-melanoma skin cancer (basal-cell	Percent	1990	0.0003017	0.000356108	0.000257686
Prevalence	Czechia	Female	55+ years	Non-melanoma skin cancer (basal-cell	Percent	1990	0.0002637	0.000315185	0.000223033
Prevalence	Czechia	Both	55+ years	Non-melanoma skin cancer (basal-cell	Percent	1990	0.0002794	0.000327412	0.00023895
Prevalence	Czechia	Male	55+ years	Non-melanoma skin cancer (basal-cell	Rate	1990	30.161448	35.60474764	25.76238182
Prevalence	Czechia	Female	55+ years	Non-melanoma skin cancer (basal-cell	Rate	1990	26.36047	31.51034428	22.2966596
Prevalence	Czechia	Both	55+ years	Non-melanoma skin cancer (basal-cell	Rate	1990	27.936069	32.73309114	23.89058318
Prevalence	Czechia	Male	55+ years	Non-melanoma skin cancer (basal-cell	Number	2021	416.37876	532.092588	306.6992423
Prevalence	Czechia	Female	55+ years	Non-melanoma skin cancer (basal-cell	Number	2021	467.90781	612.9437476	344.6860352
Prevalence	Czechia	Both	55+ years	Non-melanoma skin cancer (basal-cell	Number	2021	884.28657	1132.734939	657.9476683
Prevalence	Czechia	Male	55+ years	Non-melanoma skin cancer (basal-cell	Percent	2021	0.0002652	0.000338804	0.000195304
Prevalence	Czechia	Female	55+ years	Non-melanoma skin cancer (basal-cell	Percent	2021	0.0002435	0.000318986	0.000179366
Prevalence	Czechia	Both	55+ years	Non-melanoma skin cancer (basal-cell	Percent	2021	0.0002532	0.000324372	0.000188423
Prevalence	Czechia	Male	55+ years	Non-melanoma skin cancer (basal-cell	Rate	2021	26.502155	33.8672422	19.5211468
Prevalence	Czechia	Female	55+ years	Non-melanoma skin cancer (basal-cell	Rate	2021	24.340392	31.8851088	17.93044105
Prevalence	Czechia	Both	55+ years	Non-melanoma skin cancer (basal-cell	Rate	2021	25.3126	32.42440605	18.83367559
Prevalence	Poland	Male	55+ years	Non-melanoma skin cancer (basal-cell	Number	1990	302.14283	393.2544436	225.131815
Prevalence	Poland	Female	55+ years	Non-melanoma skin cancer (basal-cell	Number	1990	425.45943	550.421331	315.5339321
Prevalence	Poland	Both	55+ years	Non-melanoma skin cancer (basal-cell	Number	1990	727.60226	942.2732545	541.7281249

Prevalence	Poland	Male	55+ years	Non-melanoma skin cancer (basal-cell	Percent	1990	9.43E-05	0.000122676	7.02E-05
Prevalence	Poland	Female	55+ years	Non-melanoma skin cancer (basal-cell	Percent	1990	9.46E-05	0.000122346	7.01E-05
Prevalence	Poland	Both	55+ years	Non-melanoma skin cancer (basal-cell	Percent	1990	9.44E-05	0.000122303	7.03E-05
Prevalence	Poland	Male	55+ years	Non-melanoma skin cancer (basal-cell	Rate	1990	9.4236893	12.26541665	7.021752854
Prevalence	Poland	Female	55+ years	Non-melanoma skin cancer (basal-cell	Rate	1990	9.4551286	12.23219917	7.012217157
Prevalence	Poland	Both	55+ years	Non-melanoma skin cancer (basal-cell	Rate	1990	9.4420477	12.22781946	7.029971057
Prevalence	Poland	Male	55+ years	Non-melanoma skin cancer (basal-cell	Number	2021	747.65766	973.0036383	546.5471161
Prevalence	Poland	Female	55+ years	Non-melanoma skin cancer (basal-cell	Number	2021	946.02761	1218.381553	700.2437958
Prevalence	Poland	Both	55+ years	Non-melanoma skin cancer (basal-cell	Number	2021	1693.6853	2184.603109	1247.604697
Prevalence	Poland	Male	55+ years	Non-melanoma skin cancer (basal-cell	Percent	2021	0.0001427	0.000185731	0.000104318
Prevalence	Poland	Female	55+ years	Non-melanoma skin cancer (basal-cell	Percent	2021	0.0001377	0.000177304	0.000101903
Prevalence	Poland	Both	55+ years	Non-melanoma skin cancer (basal-cell	Percent	2021	0.0001399	0.000180388	0.000103012
Prevalence	Poland	Male	55+ years	Non-melanoma skin cancer (basal-cell	Rate	2021	14.263724	18.56284801	10.42696106
Prevalence	Poland	Female	55+ years	Non-melanoma skin cancer (basal-cell	Rate	2021	13.763044	17.72531657	10.18732016
Prevalence	Poland	Both	55+ years	Non-melanoma skin cancer (basal-cell	Rate	2021	13.979662	18.03169289	10.29771707
Prevalence	Iceland	Male	55+ years	Non-melanoma skin cancer (basal-cell	Number	1990	6.017522	7.844948977	4.481682916
Prevalence	Iceland	Female	55+ years	Non-melanoma skin cancer (basal-cell	Number	1990	5.3972169	6.848349488	4.091726024
Prevalence	Iceland	Both	55+ years	Non-melanoma skin cancer (basal-cell	Number	1990	11.414739	14.60458243	8.702676696
Prevalence	Iceland	Male	55+ years	Non-melanoma skin cancer (basal-cell	Percent	1990	0.0002701	0.000352094	0.000201117
Prevalence	Iceland	Female	55+ years	Non-melanoma skin cancer (basal-cell	Percent	1990	0.0002125	0.000269628	0.0001611
Prevalence	Iceland	Both	55+ years	Non-melanoma skin cancer (basal-cell	Percent	1990	0.0002394	0.000306291	0.000182496
Prevalence	Iceland	Male	55+ years	Non-melanoma skin cancer (basal-cell	Rate	1990	26.981947	35.17594112	20.0954034
Prevalence	Iceland	Female	55+ years	Non-melanoma skin cancer (basal-cell	Rate	1990	21.238203	26.94845138	16.1010591
Prevalence	Iceland	Both	55+ years	Non-melanoma skin cancer (basal-cell	Rate	1990	23.922845	30.60807271	18.23894399
Prevalence	Iceland	Male	55+ years	Non-melanoma skin cancer (basal-cell	Number	2021	13.099596	16.60594982	9.826429475
Prevalence	Iceland	Female	55+ years	Non-melanoma skin cancer (basal-cell	Number	2021	10.543052	13.36484779	8.019796432
Prevalence	Iceland	Both	55+ years	Non-melanoma skin cancer (basal-cell	Number	2021	23.642648	30.19837723	17.881885
Prevalence	Iceland	Male	55+ years	Non-melanoma skin cancer (basal-cell	Percent	2021	0.0002769	0.000351009	0.000207662
Prevalence	Iceland	Female	55+ years	Non-melanoma skin cancer (basal-cell	Percent	2021	0.0002111	0.00026759	0.000160586
Prevalence	Iceland	Both	55+ years	Non-melanoma skin cancer (basal-cell	Percent	2021	0.0002431	0.000310585	0.000183898
Prevalence	Iceland	Male	55+ years	Non-melanoma skin cancer (basal-cell	Rate	2021	27.62922	35.0247015	20.72556902
Prevalence	Iceland	Female	55+ years	Non-melanoma skin cancer (basal-cell	Rate	2021	21.097043	26.74356306	16.04791428
Prevalence	Iceland	Both	55+ years	Non-melanoma skin cancer (basal-cell	Rate	2021	24.27721	31.00889303	18.36182968
Prevalence	Lithuania	Male	55+ years	Non-melanoma skin cancer (basal-cell	Number	1990	43.774661	52.06022835	36.81025319
Prevalence	Lithuania	Female	55+ years	Non-melanoma skin cancer (basal-cell	Number	1990	81.313702	98.07314138	67.48508707
Prevalence	Lithuania	Both	55+ years	Non-melanoma skin cancer (basal-cell	Number	1990	125.08836	149.6694652	104.9842994
Prevalence	Lithuania	Male	55+ years	Non-melanoma skin cancer (basal-cell	Percent	1990	0.0001437	0.000170882	0.000120811
Prevalence	Lithuania	Female	55+ years	Non-melanoma skin cancer (basal-cell	Percent	1990	0.0001668	0.000201124	0.000138396
Prevalence	Lithuania	Both	55+ years	Non-melanoma skin cancer (basal-cell	Percent	1990	0.0001579	0.000188903	0.000132504
Prevalence	Lithuania	Male	55+ years	Non-melanoma skin cancer (basal-cell	Rate	1990	14.364286	17.08312576	12.07897477
Prevalence	Lithuania	Female	55+ years	Non-melanoma skin cancer (basal-cell	Rate	1990	16.672793	20.10919662	13.83733472
Prevalence	Lithuania	Both	55+ years	Non-melanoma skin cancer (basal-cell	Rate	1990	15.785028	18.88694203	13.24807551
Prevalence	Lithuania	Male	55+ years	Non-melanoma skin cancer (basal-cell	Number	2021	56.544279	70.5468915	43.19354478
Prevalence	Lithuania	Female	55+ years	Non-melanoma skin cancer (basal-cell	Number	2021	104.03208	132.5477366	77.45721217
Prevalence	Lithuania	Both	55+ years	Non-melanoma skin cancer (basal-cell	Number	2021	160.57636	200.1599925	121.9991118
Prevalence	Lithuania	Male	55+ years	Non-melanoma skin cancer (basal-cell	Percent	2021	0.0001517	0.0001893	0.000115912
Prevalence	Lithuania	Female	55+ years	Non-melanoma skin cancer (basal-cell	Percent	2021	0.0001749	0.000222848	0.00013022
Prevalence	Lithuania	Both	55+ years	Non-melanoma skin cancer (basal-cell	Percent	2021	0.000166	0.000206913	0.000126116
Prevalence	Lithuania	Male	55+ years	Non-melanoma skin cancer (basal-cell	Rate	2021	15.166002	18.92170695	11.58513974
Prevalence	Lithuania	Female	55+ years	Non-melanoma skin cancer (basal-cell	Rate	2021	17.486016	22.279011	13.01923462
Prevalence	Lithuania	Both	55+ years	Non-melanoma skin cancer (basal-cell	Rate	2021	16.592234	20.68238132	12.60607637
Prevalence	Andorra	Male	55+ years	Non-melanoma skin cancer (basal-cell	Number	1990	1.2949239	1.692110752	0.966188058
Prevalence	Andorra	Female	55+ years	Non-melanoma skin cancer (basal-cell	Number	1990	0.9554823	1.248114577	0.707108958
Prevalence	Andorra	Both	55+ years	Non-melanoma skin cancer (basal-cell	Number	1990	2.2504062	2.918356569	1.673538939
Prevalence	Andorra	Male	55+ years	Non-melanoma skin cancer (basal-cell	Percent	1990	0.0002545	0.000332545	0.000189867
Prevalence	Andorra	Female	55+ years	Non-melanoma skin cancer (basal-cell	Percent	1990	0.0002003	0.000261662	0.000148235

Prevalence	Andorra	Both	55+ years	Non-melanoma skin cancer (basal-cell	Percent	1990	0.0002283	0.000296023	0.000169725
Prevalence	Andorra	Male	55+ years	Non-melanoma skin cancer (basal-cell	Rate	1990	25.41797	33.21432246	18.96523717
Prevalence	Andorra	Female	55+ years	Non-melanoma skin cancer (basal-cell	Rate	1990	20.018806	26.14989884	14.81500821
Prevalence	Andorra	Both	55+ years	Non-melanoma skin cancer (basal-cell	Rate	1990	22.806373	29.57560658	16.96020623
Prevalence	Andorra	Male	55+ years	Non-melanoma skin cancer (basal-cell	Number	2021	3.5080609	4.643812732	2.63382783
Prevalence	Andorra	Female	55+ years	Non-melanoma skin cancer (basal-cell	Number	2021	2.6755343	3.463308206	2.009468101
Prevalence	Andorra	Both	55+ years	Non-melanoma skin cancer (basal-cell	Number	2021	6.1835951	8.050684734	4.665067377
Prevalence	Andorra	Male	55+ years	Non-melanoma skin cancer (basal-cell	Percent	2021	0.0002591	0.000342952	0.000194507
Prevalence	Andorra	Female	55+ years	Non-melanoma skin cancer (basal-cell	Percent	2021	0.0002075	0.000268554	0.000155854
Prevalence	Andorra	Both	55+ years	Non-melanoma skin cancer (basal-cell	Percent	2021	0.0002339	0.000304542	0.000176464
Prevalence	Andorra	Male	55+ years	Non-melanoma skin cancer (basal-cell	Rate	2021	25.870093	34.24566219	19.42308688
Prevalence	Andorra	Female	55+ years	Non-melanoma skin cancer (basal-cell	Rate	2021	20.738217	26.84429699	15.57550045
Prevalence	Andorra	Both	55+ years	Non-melanoma skin cancer (basal-cell	Rate	2021	23.36804	30.42384218	17.62946611
Prevalence	Denmark	Male	55+ years	Non-melanoma skin cancer (basal-cell	Number	1990	190.73794	226.6682367	159.8535885
Prevalence	Denmark	Female	55+ years	Non-melanoma skin cancer (basal-cell	Number	1990	205.61	248.7264364	171.5493172
Prevalence	Denmark	Both	55+ years	Non-melanoma skin cancer (basal-cell	Number	1990	396.34794	470.2281703	334.1003768
Prevalence	Denmark	Male	55+ years	Non-melanoma skin cancer (basal-cell	Percent	1990	0.0003325	0.000395091	0.000278719
Prevalence	Denmark	Female	55+ years	Non-melanoma skin cancer (basal-cell	Percent	1990	0.0002817	0.000340742	0.000235017
Prevalence	Denmark	Both	55+ years	Non-melanoma skin cancer (basal-cell	Percent	1990	0.000304	0.000360699	0.000256274
Prevalence	Denmark	Male	55+ years	Non-melanoma skin cancer (basal-cell	Rate	1990	33.206579	39.46187444	27.82975828
Prevalence	Denmark	Female	55+ years	Non-melanoma skin cancer (basal-cell	Rate	1990	28.156694	34.06115489	23.49234746
Prevalence	Denmark	Both	55+ years	Non-melanoma skin cancer (basal-cell	Rate	1990	30.380035	36.04294794	25.60876453
Prevalence	Denmark	Male	55+ years	Non-melanoma skin cancer (basal-cell	Number	2021	270.47173	356.3416329	203.2513855
Prevalence	Denmark	Female	55+ years	Non-melanoma skin cancer (basal-cell	Number	2021	231.16031	298.2026821	175.2155958
Prevalence	Denmark	Both	55+ years	Non-melanoma skin cancer (basal-cell	Number	2021	501.63204	652.0170548	381.173669
Prevalence	Denmark	Male	55+ years	Non-melanoma skin cancer (basal-cell	Percent	2021	0.0002962	0.000390161	0.000222511
Prevalence	Denmark	Female	55+ years	Non-melanoma skin cancer (basal-cell	Percent	2021	0.000229	0.000295333	0.000173542
Prevalence	Denmark	Both	55+ years	Non-melanoma skin cancer (basal-cell	Percent	2021	0.0002609	0.000339061	0.000198251
Prevalence	Denmark	Male	55+ years	Non-melanoma skin cancer (basal-cell	Rate	2021	29.560264	38.94511662	22.21365169
Prevalence	Denmark	Female	55+ years	Non-melanoma skin cancer (basal-cell	Rate	2021	22.881755	29.51804602	17.34398223
Prevalence	Denmark	Both	55+ years	Non-melanoma skin cancer (basal-cell	Rate	2021	26.055793	33.8670982	19.79893928
Prevalence	France	Male	55+ years	Non-melanoma skin cancer (basal-cell	Number	1990	2189.954	2647.6689	1802.449832
Prevalence	France	Female	55+ years	Non-melanoma skin cancer (basal-cell	Number	1990	2629.7463	3174.06078	2164.848139
Prevalence	France	Both	55+ years	Non-melanoma skin cancer (basal-cell	Number	1990	4819.7003	5748.053004	4043.336833
Prevalence	France	Male	55+ years	Non-melanoma skin cancer (basal-cell	Percent	1990	0.0003666	0.000443209	0.000301678
Prevalence	France	Female	55+ years	Non-melanoma skin cancer (basal-cell	Percent	1990	0.0003314	0.000400067	0.000272847
Prevalence	France	Both	55+ years	Non-melanoma skin cancer (basal-cell	Percent	1990	0.0003465	0.00041327	0.000290685
Prevalence	France	Male	55+ years	Non-melanoma skin cancer (basal-cell	Rate	1990	36.630281	44.2862531	30.14869022
Prevalence	France	Female	55+ years	Non-melanoma skin cancer (basal-cell	Rate	1990	33.128449	39.98549636	27.27185564
Prevalence	France	Both	55+ years	Non-melanoma skin cancer (basal-cell	Rate	1990	34.632831	41.30367762	29.05413032
Prevalence	France	Male	55+ years	Non-melanoma skin cancer (basal-cell	Number	2021	3241.0668	4146.638809	2435.204746
Prevalence	France	Female	55+ years	Non-melanoma skin cancer (basal-cell	Number	2021	3205.797	4113.975694	2410.473087
Prevalence	France	Both	55+ years	Non-melanoma skin cancer (basal-cell	Number	2021	6446.8637	8156.675624	4794.846956
Prevalence	France	Male	55+ years	Non-melanoma skin cancer (basal-cell	Percent	2021	0.0003246	0.000415316	0.00024385
Prevalence	France	Female	55+ years	Non-melanoma skin cancer (basal-cell	Percent	2021	0.0002647	0.000339757	0.000199061
Prevalence	France	Both	55+ years	Non-melanoma skin cancer (basal-cell	Percent	2021	0.0002918	0.000369149	0.000217007
Prevalence	France	Male	55+ years	Non-melanoma skin cancer (basal-cell	Rate	2021	32.424456	41.48402924	24.36240761
Prevalence	France	Female	55+ years	Non-melanoma skin cancer (basal-cell	Rate	2021	26.462752	33.95945533	19.89762682
Prevalence	France	Both	55+ years	Non-melanoma skin cancer (basal-cell	Rate	2021	29.157974	36.89113757	21.68620732
Prevalence	Germany	Male	55+ years	Non-melanoma skin cancer (basal-cell	Number	1990	1372.0363	1659.005284	1122.557906
Prevalence	Germany	Female	55+ years	Non-melanoma skin cancer (basal-cell	Number	1990	1670.488	2030.272658	1361.831638
Prevalence	Germany	Both	55+ years	Non-melanoma skin cancer (basal-cell	Number	1990	3042.5244	3645.492995	2511.097649
Prevalence	Germany	Male	55+ years	Non-melanoma skin cancer (basal-cell	Percent	1990	0.0001634	0.0001976	0.00013373
Prevalence	Germany	Female	55+ years	Non-melanoma skin cancer (basal-cell	Percent	1990	0.0001328	0.000161457	0.000108291
Prevalence	Germany	Both	55+ years	Non-melanoma skin cancer (basal-cell	Percent	1990	0.0001451	0.000173819	0.000119761
Prevalence	Germany	Male	55+ years	Non-melanoma skin cancer (basal-cell	Rate	1990	16.326194	19.74090732	13.35758951

Prevalence	Germany	Female	55+ years	Non-melanoma skin cancer (basal-cell	Rate	1990	13.279659	16.13979169	10.82597398
Prevalence	Germany	Both	55+ years	Non-melanoma skin cancer (basal-cell	Rate	1990	14.499815	17.37339344	11.96718454
Prevalence	Germany	Male	55+ years	Non-melanoma skin cancer (basal-cell	Number	2021	3452.9181	4402.096786	2599.03048
Prevalence	Germany	Female	55+ years	Non-melanoma skin cancer (basal-cell	Number	2021	3043.6262	3991.810551	2285.376926
Prevalence	Germany	Both	55+ years	Non-melanoma skin cancer (basal-cell	Number	2021	6496.5444	8314.486234	4927.281299
Prevalence	Germany	Male	55+ years	Non-melanoma skin cancer (basal-cell	Percent	2021	0.0002362	0.000301159	0.000177829
Prevalence	Germany	Female	55+ years	Non-melanoma skin cancer (basal-cell	Percent	2021	0.0001805	0.000236671	0.000135505
Prevalence	Germany	Both	55+ years	Non-melanoma skin cancer (basal-cell	Percent	2021	0.0002064	0.000264115	0.000156519
Prevalence	Germany	Male	55+ years	Non-melanoma skin cancer (basal-cell	Rate	2021	23.585522	30.06898767	17.75295257
Prevalence	Germany	Female	55+ years	Non-melanoma skin cancer (basal-cell	Rate	2021	18.037467	23.65669914	13.54384775
Prevalence	Germany	Both	55+ years	Non-melanoma skin cancer (basal-cell	Rate	2021	20.614852	26.38355061	15.63526258
Prevalence	Luxembourg	Male	55+ years	Non-melanoma skin cancer (basal-cell	Number	1990	9.4742846	12.48195925	6.945575211
Prevalence	Luxembourg	Female	55+ years	Non-melanoma skin cancer (basal-cell	Number	1990	10.957426	14.56290948	8.096322355
Prevalence	Luxembourg	Both	55+ years	Non-melanoma skin cancer (basal-cell	Number	1990	20.43171	26.50697309	15.10537433
Prevalence	Luxembourg	Male	55+ years	Non-melanoma skin cancer (basal-cell	Percent	1990	0.000239	0.000314808	0.000175206
Prevalence	Luxembourg	Female	55+ years	Non-melanoma skin cancer (basal-cell	Percent	1990	0.0002041	0.000271296	0.000150816
Prevalence	Luxembourg	Both	55+ years	Non-melanoma skin cancer (basal-cell	Percent	1990	0.0002189	0.000284032	0.000161837
Prevalence	Luxembourg	Male	55+ years	Non-melanoma skin cancer (basal-cell	Rate	1990	23.875573	31.4550331	17.50312542
Prevalence	Luxembourg	Female	55+ years	Non-melanoma skin cancer (basal-cell	Rate	1990	20.403363	27.1169837	15.0758227
Prevalence	Luxembourg	Both	55+ years	Non-melanoma skin cancer (basal-cell	Rate	1990	21.878788	28.38433242	16.17521415
Prevalence	Luxembourg	Male	55+ years	Non-melanoma skin cancer (basal-cell	Number	2021	21.108657	27.12293458	15.97433104
Prevalence	Luxembourg	Female	55+ years	Non-melanoma skin cancer (basal-cell	Number	2021	18.62113	23.56071953	14.03544778
Prevalence	Luxembourg	Both	55+ years	Non-melanoma skin cancer (basal-cell	Number	2021	39.729788	50.88241712	29.6991562
Prevalence	Luxembourg	Male	55+ years	Non-melanoma skin cancer (basal-cell	Percent	2021	0.0002465	0.000316805	0.000186511
Prevalence	Luxembourg	Female	55+ years	Non-melanoma skin cancer (basal-cell	Percent	2021	0.0002026	0.000256383	0.000152726
Prevalence	Luxembourg	Both	55+ years	Non-melanoma skin cancer (basal-cell	Percent	2021	0.0002238	0.00028659	0.000167272
Prevalence	Luxembourg	Male	55+ years	Non-melanoma skin cancer (basal-cell	Rate	2021	24.614351	31.62746999	18.62732346
Prevalence	Luxembourg	Female	55+ years	Non-melanoma skin cancer (basal-cell	Rate	2021	20.252582	25.62494321	15.26513449
Prevalence	Luxembourg	Both	55+ years	Non-melanoma skin cancer (basal-cell	Rate	2021	22.357536	28.63356513	16.7128995
Prevalence	Belgium	Male	55+ years	Non-melanoma skin cancer (basal-cell	Number	1990	253.69811	325.0607664	188.1461989
Prevalence	Belgium	Female	55+ years	Non-melanoma skin cancer (basal-cell	Number	1990	279.30699	351.9758375	209.9193902
Prevalence	Belgium	Both	55+ years	Non-melanoma skin cancer (basal-cell	Number	1990	533.00511	674.3882752	403.9419615
Prevalence	Belgium	Male	55+ years	Non-melanoma skin cancer (basal-cell	Percent	1990	0.0002243	0.000287332	0.000166307
Prevalence	Belgium	Female	55+ years	Non-melanoma skin cancer (basal-cell	Percent	1990	0.0001876	0.000236415	0.000140995
Prevalence	Belgium	Both	55+ years	Non-melanoma skin cancer (basal-cell	Percent	1990	0.0002034	0.000257401	0.000154175
Prevalence	Belgium	Male	55+ years	Non-melanoma skin cancer (basal-cell	Rate	1990	22.401117	28.70231969	16.61299334
Prevalence	Belgium	Female	55+ years	Non-melanoma skin cancer (basal-cell	Rate	1990	18.753025	23.63210357	14.09425376
Prevalence	Belgium	Both	55+ years	Non-melanoma skin cancer (basal-cell	Rate	1990	20.328798	25.7211481	15.406334
Prevalence	Belgium	Male	55+ years	Non-melanoma skin cancer (basal-cell	Number	2021	437.25497	562.3537199	326.3177691
Prevalence	Belgium	Female	55+ years	Non-melanoma skin cancer (basal-cell	Number	2021	406.32768	523.8504166	299.3826313
Prevalence	Belgium	Both	55+ years	Non-melanoma skin cancer (basal-cell	Number	2021	843.58265	1079.299309	626.3303364
Prevalence	Belgium	Male	55+ years	Non-melanoma skin cancer (basal-cell	Percent	2021	0.000249	0.000320372	0.000185873
Prevalence	Belgium	Female	55+ years	Non-melanoma skin cancer (basal-cell	Percent	2021	0.000201	0.000259116	0.000148073
Prevalence	Belgium	Both	55+ years	Non-melanoma skin cancer (basal-cell	Percent	2021	0.0002233	0.000285729	0.000165787
Prevalence	Belgium	Male	55+ years	Non-melanoma skin cancer (basal-cell	Rate	2021	24.868151	31.98293424	18.55878139
Prevalence	Belgium	Female	55+ years	Non-melanoma skin cancer (basal-cell	Rate	2021	20.090569	25.90139318	14.80275094
Prevalence	Belgium	Both	55+ years	Non-melanoma skin cancer (basal-cell	Rate	2021	22.31244	28.54705569	16.56619888
Prevalence	Ireland	Male	55+ years	Non-melanoma skin cancer (basal-cell	Number	1990	160.98199	193.3261998	132.7211791
Prevalence	Ireland	Female	55+ years	Non-melanoma skin cancer (basal-cell	Number	1990	158.91169	190.3162251	130.0213816
Prevalence	Ireland	Both	55+ years	Non-melanoma skin cancer (basal-cell	Number	1990	319.89368	380.0651031	265.467632
Prevalence	Ireland	Male	55+ years	Non-melanoma skin cancer (basal-cell	Percent	1990	0.0005166	0.000620285	0.000425739
Prevalence	Ireland	Female	55+ years	Non-melanoma skin cancer (basal-cell	Percent	1990	0.0004312	0.000516375	0.000352755
Prevalence	Ireland	Both	55+ years	Non-melanoma skin cancer (basal-cell	Percent	1990	0.0004703	0.00055866	0.000390294
Prevalence	Ireland	Male	55+ years	Non-melanoma skin cancer (basal-cell	Rate	1990	51.59559	61.96208098	42.53784774
Prevalence	Ireland	Female	55+ years	Non-melanoma skin cancer (basal-cell	Rate	1990	43.09859	51.61584314	35.2632217
Prevalence	Ireland	Both	55+ years	Non-melanoma skin cancer (basal-cell	Rate	1990	46.993158	55.83248523	38.99783885

Prevalence	Ireland	Male	55+ years	Non-melanoma skin cancer (basal-cell	Number	2021	215.85773	277.1116433	160.3193954
Prevalence	Ireland	Female	55+ years	Non-melanoma skin cancer (basal-cell	Number	2021	177.6667	231.51251	133.6645957
Prevalence	Ireland	Both	55+ years	Non-melanoma skin cancer (basal-cell	Number	2021	393.52443	503.8297145	296.4372889
Prevalence	Ireland	Male	55+ years	Non-melanoma skin cancer (basal-cell	Percent	2021	0.0003428	0.000439999	0.000254607
Prevalence	Ireland	Female	55+ years	Non-melanoma skin cancer (basal-cell	Percent	2021	0.0002608	0.000339866	0.0001962
Prevalence	Ireland	Both	55+ years	Non-melanoma skin cancer (basal-cell	Percent	2021	0.0003002	0.000384324	0.000226115
Prevalence	Ireland	Male	55+ years	Non-melanoma skin cancer (basal-cell	Rate	2021	34.220783	43.93160838	25.4160699
Prevalence	Ireland	Female	55+ years	Non-melanoma skin cancer (basal-cell	Rate	2021	26.066557	33.96660169	19.61074191
Prevalence	Ireland	Both	55+ years	Non-melanoma skin cancer (basal-cell	Rate	2021	29.985822	38.39087757	22.58796442
Prevalence	Argentina	Male	55+ years	Non-melanoma skin cancer (basal-cell	Number	1990	389.80995	469.5899468	319.5261767
Prevalence	Argentina	Female	55+ years	Non-melanoma skin cancer (basal-cell	Number	1990	405.85211	493.9593096	328.082651
Prevalence	Argentina	Both	55+ years	Non-melanoma skin cancer (basal-cell	Number	1990	795.66206	952.8922157	655.9035554
Prevalence	Argentina	Male	55+ years	Non-melanoma skin cancer (basal-cell	Percent	1990	0.0001573	0.000189461	0.000128911
Prevalence	Argentina	Female	55+ years	Non-melanoma skin cancer (basal-cell	Percent	1990	0.0001316	0.000160146	0.00010637
Prevalence	Argentina	Both	55+ years	Non-melanoma skin cancer (basal-cell	Percent	1990	0.000143	0.000171296	0.000117908
Prevalence	Argentina	Male	55+ years	Non-melanoma skin cancer (basal-cell	Rate	1990	15.72095	18.93846019	12.88642105
Prevalence	Argentina	Female	55+ years	Non-melanoma skin cancer (basal-cell	Rate	1990	13.155444	16.01138457	10.63459559
Prevalence	Argentina	Both	55+ years	Non-melanoma skin cancer (basal-cell	Rate	1990	14.298619	17.12415793	11.78705827
Prevalence	Argentina	Male	55+ years	Non-melanoma skin cancer (basal-cell	Number	2021	693.71451	904.844366	516.4228799
Prevalence	Argentina	Female	55+ years	Non-melanoma skin cancer (basal-cell	Number	2021	695.68187	900.981184	519.571164
Prevalence	Argentina	Both	55+ years	Non-melanoma skin cancer (basal-cell	Number	2021	1389.3964	1794.98334	1035.134255
Prevalence	Argentina	Male	55+ years	Non-melanoma skin cancer (basal-cell	Percent	2021	0.0001669	0.000217725	0.000124262
Prevalence	Argentina	Female	55+ years	Non-melanoma skin cancer (basal-cell	Percent	2021	0.0001345	0.000174135	0.000100417
Prevalence	Argentina	Both	55+ years	Non-melanoma skin cancer (basal-cell	Percent	2021	0.0001489	0.000192388	0.000110955
Prevalence	Argentina	Male	55+ years	Non-melanoma skin cancer (basal-cell	Rate	2021	16.683242	21.76073432	12.41952927
Prevalence	Argentina	Female	55+ years	Non-melanoma skin cancer (basal-cell	Rate	2021	13.442718	17.40973324	10.03971618
Prevalence	Argentina	Both	55+ years	Non-melanoma skin cancer (basal-cell	Rate	2021	14.886427	19.23201307	11.09075225
Prevalence	Spain	Male	55+ years	Non-melanoma skin cancer (basal-cell	Number	1990	1532.462	1814.544186	1268.167812
Prevalence	Spain	Female	55+ years	Non-melanoma skin cancer (basal-cell	Number	1990	1518.1069	1822.753729	1263.229693
Prevalence	Spain	Both	55+ years	Non-melanoma skin cancer (basal-cell	Number	1990	3050.569	3650.739014	2584.291514
Prevalence	Spain	Male	55+ years	Non-melanoma skin cancer (basal-cell	Percent	1990	0.0003632	0.000430138	0.000300592
Prevalence	Spain	Female	55+ years	Non-melanoma skin cancer (basal-cell	Percent	1990	0.0002847	0.000341835	0.000236958
Prevalence	Spain	Both	55+ years	Non-melanoma skin cancer (basal-cell	Percent	1990	0.0003194	0.000382264	0.00027058
Prevalence	Spain	Male	55+ years	Non-melanoma skin cancer (basal-cell	Rate	1990	36.274208	42.95124595	30.01822058
Prevalence	Spain	Female	55+ years	Non-melanoma skin cancer (basal-cell	Rate	1990	28.450608	34.15994596	23.67399247
Prevalence	Spain	Both	55+ years	Non-melanoma skin cancer (basal-cell	Rate	1990	31.907719	38.18525504	27.03064509
Prevalence	Spain	Male	55+ years	Non-melanoma skin cancer (basal-cell	Number	2021	2220.6547	2873.511734	1652.016296
Prevalence	Spain	Female	55+ years	Non-melanoma skin cancer (basal-cell	Number	2021	2118.8277	2703.212481	1597.102063
Prevalence	Spain	Both	55+ years	Non-melanoma skin cancer (basal-cell	Number	2021	4339.4824	5513.840335	3226.859534
Prevalence	Spain	Male	55+ years	Non-melanoma skin cancer (basal-cell	Percent	2021	0.000316	0.000408745	0.000235098
Prevalence	Spain	Female	55+ years	Non-melanoma skin cancer (basal-cell	Percent	2021	0.0002515	0.000320795	0.000189535
Prevalence	Spain	Both	55+ years	Non-melanoma skin cancer (basal-cell	Percent	2021	0.0002808	0.000356823	0.000208738
Prevalence	Spain	Male	55+ years	Non-melanoma skin cancer (basal-cell	Rate	2021	31.534473	40.8053879	23.459506
Prevalence	Spain	Female	55+ years	Non-melanoma skin cancer (basal-cell	Rate	2021	25.125173	32.05483811	18.93852164
Prevalence	Spain	Both	55+ years	Non-melanoma skin cancer (basal-cell	Rate	2021	28.041748	35.63045199	20.85197552
Prevalence	Austria	Male	55+ years	Non-melanoma skin cancer (basal-cell	Number	1990	155.81989	188.5916227	126.5881626
Prevalence	Austria	Female	55+ years	Non-melanoma skin cancer (basal-cell	Number	1990	215.36751	259.6393548	177.5697452
Prevalence	Austria	Both	55+ years	Non-melanoma skin cancer (basal-cell	Number	1990	371.1874	442.1675967	303.6483821
Prevalence	Austria	Male	55+ years	Non-melanoma skin cancer (basal-cell	Percent	1990	0.0002024	0.000245002	0.000164458
Prevalence	Austria	Female	55+ years	Non-melanoma skin cancer (basal-cell	Percent	1990	0.0001833	0.000220939	0.000151105
Prevalence	Austria	Both	55+ years	Non-melanoma skin cancer (basal-cell	Percent	1990	0.0001909	0.00022735	0.000156109
Prevalence	Austria	Male	55+ years	Non-melanoma skin cancer (basal-cell	Rate	1990	20.228321	24.48270171	16.4334989
Prevalence	Austria	Female	55+ years	Non-melanoma skin cancer (basal-cell	Rate	1990	18.319668	22.08553571	15.10450121
Prevalence	Austria	Both	55+ years	Non-melanoma skin cancer (basal-cell	Rate	1990	19.075224	22.72287759	15.60441124
Prevalence	Austria	Male	55+ years	Non-melanoma skin cancer (basal-cell	Number	2021	316.45923	410.5572205	238.2494916
Prevalence	Austria	Female	55+ years	Non-melanoma skin cancer (basal-cell	Number	2021	299.00551	391.6538661	224.100881

Prevalence	Austria	Both	55+ years	Non-melanoma skin cancer (basal-cell	Number	2021	615.46474	794.0084335	460.5616613
Prevalence	Austria	Male	55+ years	Non-melanoma skin cancer (basal-cell	Percent	2021	0.0002337	0.000303238	0.00017593
Prevalence	Austria	Female	55+ years	Non-melanoma skin cancer (basal-cell	Percent	2021	0.0001871	0.000245009	0.000140228
Prevalence	Austria	Both	55+ years	Non-melanoma skin cancer (basal-cell	Percent	2021	0.0002085	0.000268927	0.000155967
Prevalence	Austria	Male	55+ years	Non-melanoma skin cancer (basal-cell	Rate	2021	23.341475	30.28197713	17.57286267
Prevalence	Austria	Female	55+ years	Non-melanoma skin cancer (basal-cell	Rate	2021	18.694943	24.48766528	14.01162567
Prevalence	Austria	Both	55+ years	Non-melanoma skin cancer (basal-cell	Rate	2021	20.826689	26.86842294	15.58492956
Prevalence	Cyprus	Male	55+ years	Non-melanoma skin cancer (basal-cell	Number	1990	11.278946	14.0623573	8.813983802
Prevalence	Cyprus	Female	55+ years	Non-melanoma skin cancer (basal-cell	Number	1990	10.762207	13.27263233	8.639169998
Prevalence	Cyprus	Both	55+ years	Non-melanoma skin cancer (basal-cell	Number	1990	22.041152	27.21743624	17.79868462
Prevalence	Cyprus	Male	55+ years	Non-melanoma skin cancer (basal-cell	Percent	1990	0.0001747	0.000217857	0.000136518
Prevalence	Cyprus	Female	55+ years	Non-melanoma skin cancer (basal-cell	Percent	1990	0.0001434	0.000176858	0.000115122
Prevalence	Cyprus	Both	55+ years	Non-melanoma skin cancer (basal-cell	Percent	1990	0.0001579	0.000194956	0.000127494
Prevalence	Cyprus	Male	55+ years	Non-melanoma skin cancer (basal-cell	Rate	1990	17.455673	21.76337405	13.64081585
Prevalence	Cyprus	Female	55+ years	Non-melanoma skin cancer (basal-cell	Rate	1990	14.335274	17.67916405	11.50738602
Prevalence	Cyprus	Both	55+ years	Non-melanoma skin cancer (basal-cell	Rate	1990	15.778643	19.48419949	12.74157928
Prevalence	Cyprus	Male	55+ years	Non-melanoma skin cancer (basal-cell	Number	2021	32.689704	43.61039203	24.27577354
Prevalence	Cyprus	Female	55+ years	Non-melanoma skin cancer (basal-cell	Number	2021	26.108004	34.56314305	19.5413799
Prevalence	Cyprus	Both	55+ years	Non-melanoma skin cancer (basal-cell	Number	2021	58.797708	77.65958365	43.79966776
Prevalence	Cyprus	Male	55+ years	Non-melanoma skin cancer (basal-cell	Percent	2021	0.0001944	0.000259314	0.000144393
Prevalence	Cyprus	Female	55+ years	Non-melanoma skin cancer (basal-cell	Percent	2021	0.0001442	0.00019091	0.00010793
Prevalence	Cyprus	Both	55+ years	Non-melanoma skin cancer (basal-cell	Percent	2021	0.0001684	0.000222376	0.000125418
Prevalence	Cyprus	Male	55+ years	Non-melanoma skin cancer (basal-cell	Rate	2021	19.414962	25.90094125	14.41778794
Prevalence	Cyprus	Female	55+ years	Non-melanoma skin cancer (basal-cell	Rate	2021	14.414127	19.08217689	10.78871987
Prevalence	Cyprus	Both	55+ years	Non-melanoma skin cancer (basal-cell	Rate	2021	16.823299	22.22009032	12.5320344
Prevalence	Switzerlan	Male	55+ years	Non-melanoma skin cancer (basal-cell	Number	1990	383.47131	454.1656304	318.7890209
Prevalence	Switzerlan	Female	55+ years	Non-melanoma skin cancer (basal-cell	Number	1990	425.84083	502.7038427	357.9331844
Prevalence	Switzerlan	Both	55+ years	Non-melanoma skin cancer (basal-cell	Number	1990	809.31214	952.2305521	678.9817254
Prevalence	Switzerlan	Male	55+ years	Non-melanoma skin cancer (basal-cell	Percent	1990	0.0005276	0.000624802	0.000438554
Prevalence	Switzerlan	Female	55+ years	Non-melanoma skin cancer (basal-cell	Percent	1990	0.0004428	0.000522771	0.000372225
Prevalence	Switzerlan	Both	55+ years	Non-melanoma skin cancer (basal-cell	Percent	1990	0.0004793	0.00056399	0.000402124
Prevalence	Switzerlan	Male	55+ years	Non-melanoma skin cancer (basal-cell	Rate	1990	52.729487	62.45035762	43.83530375
Prevalence	Switzerlan	Female	55+ years	Non-melanoma skin cancer (basal-cell	Rate	1990	44.269712	52.26026353	37.21014433
Prevalence	Switzerlan	Both	55+ years	Non-melanoma skin cancer (basal-cell	Rate	1990	47.911928	56.37281231	40.19626264
Prevalence	Switzerlan	Male	55+ years	Non-melanoma skin cancer (basal-cell	Number	2021	499.83556	637.4232377	376.4337667
Prevalence	Switzerlan	Female	55+ years	Non-melanoma skin cancer (basal-cell	Number	2021	450.11294	570.4470538	337.9329105
Prevalence	Switzerlan	Both	55+ years	Non-melanoma skin cancer (basal-cell	Number	2021	949.9485	1204.456634	721.9242871
Prevalence	Switzerlan	Male	55+ years	Non-melanoma skin cancer (basal-cell	Percent	2021	0.0003608	0.000460114	0.000271754
Prevalence	Switzerlan	Female	55+ years	Non-melanoma skin cancer (basal-cell	Percent	2021	0.0002916	0.0003696	0.000218935
Prevalence	Switzerlan	Both	55+ years	Non-melanoma skin cancer (basal-cell	Percent	2021	0.0003243	0.000411246	0.000246491
Prevalence	Switzerlan	Male	55+ years	Non-melanoma skin cancer (basal-cell	Rate	2021	36.043227	45.96469827	27.14470305
Prevalence	Switzerlan	Female	55+ years	Non-melanoma skin cancer (basal-cell	Rate	2021	29.15111	36.94442778	21.88588393
Prevalence	Switzerlan	Both	55+ years	Non-melanoma skin cancer (basal-cell	Rate	2021	32.412215	41.09602545	24.63203575
Prevalence	Finland	Male	55+ years	Non-melanoma skin cancer (basal-cell	Number	1990	115.548	145.8162759	88.35633673
Prevalence	Finland	Female	55+ years	Non-melanoma skin cancer (basal-cell	Number	1990	149.78129	196.9932867	113.699795
Prevalence	Finland	Both	55+ years	Non-melanoma skin cancer (basal-cell	Number	1990	265.32928	342.1631088	202.8715246
Prevalence	Finland	Male	55+ years	Non-melanoma skin cancer (basal-cell	Percent	1990	0.0002403	0.000303208	0.000183748
Prevalence	Finland	Female	55+ years	Non-melanoma skin cancer (basal-cell	Percent	1990	0.0002122	0.000279145	0.000161104
Prevalence	Finland	Both	55+ years	Non-melanoma skin cancer (basal-cell	Percent	1990	0.0002236	0.000288326	0.000170989
Prevalence	Finland	Male	55+ years	Non-melanoma skin cancer (basal-cell	Rate	1990	24.007513	30.29638036	18.3578765
Prevalence	Finland	Female	55+ years	Non-melanoma skin cancer (basal-cell	Rate	1990	21.213886	27.90063646	16.10357743
Prevalence	Finland	Both	55+ years	Non-melanoma skin cancer (basal-cell	Rate	1990	22.346297	28.81731848	17.08604225
Prevalence	Finland	Male	55+ years	Non-melanoma skin cancer (basal-cell	Number	2021	256.62947	333.8687344	192.7902373
Prevalence	Finland	Female	55+ years	Non-melanoma skin cancer (basal-cell	Number	2021	240.33357	312.0841481	178.2458735
Prevalence	Finland	Both	55+ years	Non-melanoma skin cancer (basal-cell	Number	2021	496.96305	645.5072084	373.383458
Prevalence	Finland	Male	55+ years	Non-melanoma skin cancer (basal-cell	Percent	2021	0.0002768	0.000360093	0.000207936

Prevalence	Finland	Female	55+ years	Non-melanoma skin cancer (basal-cell	Percent	2021	0.0002218	0.000287926	0.00016446
Prevalence	Finland	Both	55+ years	Non-melanoma skin cancer (basal-cell	Percent	2021	0.0002471	0.000321004	0.00018568
Prevalence	Finland	Male	55+ years	Non-melanoma skin cancer (basal-cell	Rate	2021	27.645857	35.96659127	20.7686643
Prevalence	Finland	Female	55+ years	Non-melanoma skin cancer (basal-cell	Rate	2021	22.162973	28.77963549	16.43739772
Prevalence	Finland	Both	55+ years	Non-melanoma skin cancer (basal-cell	Rate	2021	24.691768	32.07223257	18.5516768
Prevalence	Belize	Male	55+ years	Non-melanoma skin cancer (basal-cell	Number	1990	0.2824785	0.370868673	0.208448626
Prevalence	Belize	Female	55+ years	Non-melanoma skin cancer (basal-cell	Number	1990	0.1097937	0.145843682	0.076764123
Prevalence	Belize	Both	55+ years	Non-melanoma skin cancer (basal-cell	Number	1990	0.3922722	0.519878301	0.287316433
Prevalence	Belize	Male	55+ years	Non-melanoma skin cancer (basal-cell	Percent	1990	3.65E-05	4.79E-05	2.69E-05
Prevalence	Belize	Female	55+ years	Non-melanoma skin cancer (basal-cell	Percent	1990	1.44E-05	1.91E-05	1.00E-05
Prevalence	Belize	Both	55+ years	Non-melanoma skin cancer (basal-cell	Percent	1990	2.55E-05	3.38E-05	1.87E-05
Prevalence	Belize	Male	55+ years	Non-melanoma skin cancer (basal-cell	Rate	1990	3.6478704	4.789322587	2.691863148
Prevalence	Belize	Female	55+ years	Non-melanoma skin cancer (basal-cell	Rate	1990	1.4354122	1.906719489	1.003592667
Prevalence	Belize	Both	55+ years	Non-melanoma skin cancer (basal-cell	Rate	1990	2.5484489	3.3774586	1.86658946
Prevalence	Belize	Male	55+ years	Non-melanoma skin cancer (basal-cell	Number	2021	0.8712171	1.151905531	0.632079686
Prevalence	Belize	Female	55+ years	Non-melanoma skin cancer (basal-cell	Number	2021	0.3175014	0.423151416	0.220127677
Prevalence	Belize	Both	55+ years	Non-melanoma skin cancer (basal-cell	Number	2021	1.1887184	1.573923296	0.866807143
Prevalence	Belize	Male	55+ years	Non-melanoma skin cancer (basal-cell	Percent	2021	3.42E-05	4.52E-05	2.48E-05
Prevalence	Belize	Female	55+ years	Non-melanoma skin cancer (basal-cell	Percent	2021	1.29E-05	1.72E-05	8.95E-06
Prevalence	Belize	Both	55+ years	Non-melanoma skin cancer (basal-cell	Percent	2021	2.37E-05	3.14E-05	1.73E-05
Prevalence	Belize	Male	55+ years	Non-melanoma skin cancer (basal-cell	Rate	2021	3.4179188	4.519102879	2.479745998
Prevalence	Belize	Female	55+ years	Non-melanoma skin cancer (basal-cell	Rate	2021	1.2908294	1.720358822	0.894948184
Prevalence	Belize	Both	55+ years	Non-melanoma skin cancer (basal-cell	Rate	2021	2.3733362	3.142417123	1.730624113
Prevalence	Latvia	Male	55+ years	Non-melanoma skin cancer (basal-cell	Number	1990	25.164577	29.81312577	21.10807668
Prevalence	Latvia	Female	55+ years	Non-melanoma skin cancer (basal-cell	Number	1990	46.466711	56.09207456	38.87933806
Prevalence	Latvia	Both	55+ years	Non-melanoma skin cancer (basal-cell	Number	1990	71.631287	85.48462949	60.5178558
Prevalence	Latvia	Male	55+ years	Non-melanoma skin cancer (basal-cell	Percent	1990	0.0001114	0.000132019	9.35E-05
Prevalence	Latvia	Female	55+ years	Non-melanoma skin cancer (basal-cell	Percent	1990	0.0001164	0.000140489	9.74E-05
Prevalence	Latvia	Both	55+ years	Non-melanoma skin cancer (basal-cell	Percent	1990	0.0001146	0.000136746	9.68E-05
Prevalence	Latvia	Male	55+ years	Non-melanoma skin cancer (basal-cell	Rate	1990	11.140241	13.19813252	9.344447657
Prevalence	Latvia	Female	55+ years	Non-melanoma skin cancer (basal-cell	Rate	1990	11.635617	14.04588157	9.735681595
Prevalence	Latvia	Both	55+ years	Non-melanoma skin cancer (basal-cell	Rate	1990	11.456645	13.67233695	9.67917298
Prevalence	Latvia	Male	55+ years	Non-melanoma skin cancer (basal-cell	Number	2021	31.859492	42.18774569	23.26712799
Prevalence	Latvia	Female	55+ years	Non-melanoma skin cancer (basal-cell	Number	2021	56.343731	74.5748447	40.70857357
Prevalence	Latvia	Both	55+ years	Non-melanoma skin cancer (basal-cell	Number	2021	88.203223	116.3966418	64.47973382
Prevalence	Latvia	Male	55+ years	Non-melanoma skin cancer (basal-cell	Percent	2021	0.0001278	0.000169182	9.33E-05
Prevalence	Latvia	Female	55+ years	Non-melanoma skin cancer (basal-cell	Percent	2021	0.000138	0.000182601	9.97E-05
Prevalence	Latvia	Both	55+ years	Non-melanoma skin cancer (basal-cell	Percent	2021	0.0001341	0.000176946	9.80E-05
Prevalence	Latvia	Male	55+ years	Non-melanoma skin cancer (basal-cell	Rate	2021	12.769865	16.90961714	9.325888827
Prevalence	Latvia	Female	55+ years	Non-melanoma skin cancer (basal-cell	Rate	2021	13.791828	18.25444289	9.964651413
Prevalence	Latvia	Both	55+ years	Non-melanoma skin cancer (basal-cell	Rate	2021	13.404348	17.68893537	9.799061435
Prevalence	New Zealan	Male	55+ years	Non-melanoma skin cancer (basal-cell	Number	1990	59.591776	77.18576043	45.16309523
Prevalence	New Zealan	Female	55+ years	Non-melanoma skin cancer (basal-cell	Number	1990	60.981692	78.58984505	44.92208007
Prevalence	New Zealan	Both	55+ years	Non-melanoma skin cancer (basal-cell	Number	1990	120.57347	156.0741524	90.58649411
Prevalence	New Zealan	Male	55+ years	Non-melanoma skin cancer (basal-cell	Percent	1990	0.0001983	0.000256804	0.000150253
Prevalence	New Zealan	Female	55+ years	Non-melanoma skin cancer (basal-cell	Percent	1990	0.0001708	0.000220066	0.000125787
Prevalence	New Zealan	Both	55+ years	Non-melanoma skin cancer (basal-cell	Percent	1990	0.0001833	0.000237302	0.000137733
Prevalence	New Zealan	Male	55+ years	Non-melanoma skin cancer (basal-cell	Rate	1990	19.817349	25.66825954	15.01906626
Prevalence	New Zealan	Female	55+ years	Non-melanoma skin cancer (basal-cell	Rate	1990	17.06885	21.99739351	12.57374502
Prevalence	New Zealan	Both	55+ years	Non-melanoma skin cancer (basal-cell	Rate	1990	18.32496	23.72041417	13.76748889
Prevalence	New Zealan	Male	55+ years	Non-melanoma skin cancer (basal-cell	Number	2021	142.67218	183.9590184	106.6486582
Prevalence	New Zealan	Female	55+ years	Non-melanoma skin cancer (basal-cell	Number	2021	117.50924	148.8716696	90.31349572
Prevalence	New Zealan	Both	55+ years	Non-melanoma skin cancer (basal-cell	Number	2021	260.18142	331.7364324	198.0778004
Prevalence	New Zealan	Male	55+ years	Non-melanoma skin cancer (basal-cell	Percent	2021	0.0002104	0.000271328	0.000157279
Prevalence	New Zealan	Female	55+ years	Non-melanoma skin cancer (basal-cell	Percent	2021	0.0001572	0.000199192	0.00012084
Prevalence	New Zealan	Both	55+ years	Non-melanoma skin cancer (basal-cell	Percent	2021	0.0001825	0.000232746	0.000138948

Prevalence	New Zealan	Male	55+ years	Non-melanoma skin cancer (basal-cell	Rate	2021	21.02647	27.1111627	15.71746332
Prevalence	New Zealan	Female	55+ years	Non-melanoma skin cancer (basal-cell	Rate	2021	15.712174	19.90564778	12.07582773
Prevalence	New Zealan	Both	55+ years	Non-melanoma skin cancer (basal-cell	Rate	2021	18.240135	23.25653134	13.88633301
Prevalence	Netherland	Male	55+ years	Non-melanoma skin cancer (basal-cell	Number	1990	202.99696	265.8857886	151.2487267
Prevalence	Netherland	Female	55+ years	Non-melanoma skin cancer (basal-cell	Number	1990	255.85735	327.5084048	194.1750191
Prevalence	Netherland	Both	55+ years	Non-melanoma skin cancer (basal-cell	Number	1990	458.85431	587.4036218	348.6016984
Prevalence	Netherland	Male	55+ years	Non-melanoma skin cancer (basal-cell	Percent	1990	0.000141	0.000184713	0.000105076
Prevalence	Netherland	Female	55+ years	Non-melanoma skin cancer (basal-cell	Percent	1990	0.000137	0.000175318	0.000103956
Prevalence	Netherland	Both	55+ years	Non-melanoma skin cancer (basal-cell	Percent	1990	0.0001387	0.000177581	0.000105393
Prevalence	Netherland	Male	55+ years	Non-melanoma skin cancer (basal-cell	Rate	1990	14.082416	18.44517402	10.49250921
Prevalence	Netherland	Female	55+ years	Non-melanoma skin cancer (basal-cell	Rate	1990	13.686046	17.51872666	10.38660087
Prevalence	Netherland	Both	55+ years	Non-melanoma skin cancer (basal-cell	Rate	1990	13.858613	17.74114185	10.52869263
Prevalence	Netherland	Male	55+ years	Non-melanoma skin cancer (basal-cell	Number	2021	547.54501	713.8574669	407.3080407
Prevalence	Netherland	Female	55+ years	Non-melanoma skin cancer (basal-cell	Number	2021	480.14173	618.8367305	360.9855115
Prevalence	Netherland	Both	55+ years	Non-melanoma skin cancer (basal-cell	Number	2021	1027.6867	1312.574341	773.3706327
Prevalence	Netherland	Male	55+ years	Non-melanoma skin cancer (basal-cell	Percent	2021	0.0001962	0.00025574	0.000145909
Prevalence	Netherland	Female	55+ years	Non-melanoma skin cancer (basal-cell	Percent	2021	0.0001574	0.000202887	0.00011834
Prevalence	Netherland	Both	55+ years	Non-melanoma skin cancer (basal-cell	Percent	2021	0.0001759	0.000224671	0.000132403
Prevalence	Netherland	Male	55+ years	Non-melanoma skin cancer (basal-cell	Rate	2021	19.568627	25.51244279	14.55671975
Prevalence	Netherland	Female	55+ years	Non-melanoma skin cancer (basal-cell	Rate	2021	15.727927	20.2711378	11.82474583
Prevalence	Netherland	Both	55+ years	Non-melanoma skin cancer (basal-cell	Rate	2021	17.564674	22.43382065	13.21803842
Prevalence	Norway	Male	55+ years	Non-melanoma skin cancer (basal-cell	Number	1990	134.89829	174.0073469	101.7737704
Prevalence	Norway	Female	55+ years	Non-melanoma skin cancer (basal-cell	Number	1990	136.20896	174.4736266	102.4117131
Prevalence	Norway	Both	55+ years	Non-melanoma skin cancer (basal-cell	Number	1990	271.10725	349.2716417	206.5468068
Prevalence	Norway	Male	55+ years	Non-melanoma skin cancer (basal-cell	Percent	1990	0.0002818	0.00036354	0.000212629
Prevalence	Norway	Female	55+ years	Non-melanoma skin cancer (basal-cell	Percent	1990	0.0002256	0.000288942	0.000169607
Prevalence	Norway	Both	55+ years	Non-melanoma skin cancer (basal-cell	Percent	1990	0.0002505	0.000322658	0.000190792
Prevalence	Norway	Male	55+ years	Non-melanoma skin cancer (basal-cell	Rate	1990	28.166774	36.33274932	21.25037219
Prevalence	Norway	Female	55+ years	Non-melanoma skin cancer (basal-cell	Rate	1990	22.551292	28.88654028	16.95568628
Prevalence	Norway	Both	55+ years	Non-melanoma skin cancer (basal-cell	Rate	1990	25.034761	32.25266738	19.07307855
Prevalence	Norway	Male	55+ years	Non-melanoma skin cancer (basal-cell	Number	2021	218.43416	280.1056686	166.5343925
Prevalence	Norway	Female	55+ years	Non-melanoma skin cancer (basal-cell	Number	2021	181.77159	230.7811247	139.4212109
Prevalence	Norway	Both	55+ years	Non-melanoma skin cancer (basal-cell	Number	2021	400.20574	505.4007858	306.5220299
Prevalence	Norway	Male	55+ years	Non-melanoma skin cancer (basal-cell	Percent	2021	0.0002791	0.000357868	0.000212746
Prevalence	Norway	Female	55+ years	Non-melanoma skin cancer (basal-cell	Percent	2021	0.000217	0.000275547	0.00016647
Prevalence	Norway	Both	55+ years	Non-melanoma skin cancer (basal-cell	Percent	2021	0.000247	0.000311921	0.000189193
Prevalence	Norway	Male	55+ years	Non-melanoma skin cancer (basal-cell	Rate	2021	27.868278	35.73645617	21.24679965
Prevalence	Norway	Female	55+ years	Non-melanoma skin cancer (basal-cell	Rate	2021	21.693039	27.5419498	16.63884773
Prevalence	Norway	Both	55+ years	Non-melanoma skin cancer (basal-cell	Rate	2021	24.677626	31.16419939	18.90086823
Prevalence	Portugal	Male	55+ years	Non-melanoma skin cancer (basal-cell	Number	1990	109.986	142.9104532	86.14443049
Prevalence	Portugal	Female	55+ years	Non-melanoma skin cancer (basal-cell	Number	1990	148.49192	186.8962227	114.5351697
Prevalence	Portugal	Both	55+ years	Non-melanoma skin cancer (basal-cell	Number	1990	258.47793	324.1584055	205.8264788
Prevalence	Portugal	Male	55+ years	Non-melanoma skin cancer (basal-cell	Percent	1990	0.0001032	0.000134069	8.08E-05
Prevalence	Portugal	Female	55+ years	Non-melanoma skin cancer (basal-cell	Percent	1990	0.0001084	0.000136379	8.36E-05
Prevalence	Portugal	Both	55+ years	Non-melanoma skin cancer (basal-cell	Percent	1990	0.0001061	0.000133059	8.45E-05
Prevalence	Portugal	Male	55+ years	Non-melanoma skin cancer (basal-cell	Rate	1990	10.309623	13.39582191	8.074814848
Prevalence	Portugal	Female	55+ years	Non-melanoma skin cancer (basal-cell	Rate	1990	10.829819	13.63072334	8.353283914
Prevalence	Portugal	Both	55+ years	Non-melanoma skin cancer (basal-cell	Rate	1990	10.602187	13.29625357	8.44254231
Prevalence	Portugal	Male	55+ years	Non-melanoma skin cancer (basal-cell	Number	2021	322.64187	422.3674696	240.5639119
Prevalence	Portugal	Female	55+ years	Non-melanoma skin cancer (basal-cell	Number	2021	313.99733	407.7477826	233.1635998
Prevalence	Portugal	Both	55+ years	Non-melanoma skin cancer (basal-cell	Number	2021	636.6392	830.6330896	476.0241355
Prevalence	Portugal	Male	55+ years	Non-melanoma skin cancer (basal-cell	Percent	2021	0.0001878	0.000245806	0.000139967
Prevalence	Portugal	Female	55+ years	Non-melanoma skin cancer (basal-cell	Percent	2021	0.0001448	0.000188041	0.000107508
Prevalence	Portugal	Both	55+ years	Non-melanoma skin cancer (basal-cell	Percent	2021	0.0001638	0.000213676	0.000122448
Prevalence	Portugal	Male	55+ years	Non-melanoma skin cancer (basal-cell	Rate	2021	18.748272	24.54318845	13.97883561
Prevalence	Portugal	Female	55+ years	Non-melanoma skin cancer (basal-cell	Rate	2021	14.471734	18.7925721	10.74621114

Prevalence	Portugal	Both	55+ years	Non-melanoma skin cancer (basal-cell	Rate	2021	16.363339	21.349503	12.23509975
Prevalence	United Kin	Male	55+ years	Non-melanoma skin cancer (basal-cell	Number	1990	1996.4184	2525.948426	1535.81449
Prevalence	United Kin	Female	55+ years	Non-melanoma skin cancer (basal-cell	Number	1990	2063.0972	2628.262933	1602.068609
Prevalence	United Kin	Both	55+ years	Non-melanoma skin cancer (basal-cell	Number	1990	4059.5155	5141.108858	3130.117173
Prevalence	United Kin	Male	55+ years	Non-melanoma skin cancer (basal-cell	Percent	1990	0.0003099	0.000392054	0.000238299
Prevalence	United Kin	Female	55+ years	Non-melanoma skin cancer (basal-cell	Percent	1990	0.0002456	0.000312898	0.00019074
Prevalence	United Kin	Both	55+ years	Non-melanoma skin cancer (basal-cell	Percent	1990	0.0002735	0.00034637	0.000210867
Prevalence	United Kin	Male	55+ years	Non-melanoma skin cancer (basal-cell	Rate	1990	30.949193	39.1581581	23.80874683
Prevalence	United Kin	Female	55+ years	Non-melanoma skin cancer (basal-cell	Rate	1990	24.553633	31.27986642	19.06677276
Prevalence	United Kin	Both	55+ years	Non-melanoma skin cancer (basal-cell	Rate	1990	27.331205	34.61317041	21.07391267
Prevalence	United Kin	Male	55+ years	Non-melanoma skin cancer (basal-cell	Number	2021	3030.9484	3871.589874	2283.167064
Prevalence	United Kin	Female	55+ years	Non-melanoma skin cancer (basal-cell	Number	2021	2614.6939	3326.765358	2009.057308
Prevalence	United Kin	Both	55+ years	Non-melanoma skin cancer (basal-cell	Number	2021	5645.6424	7192.815762	4305.264415
Prevalence	United Kin	Male	55+ years	Non-melanoma skin cancer (basal-cell	Percent	2021	0.0003047	0.000389217	0.000229464
Prevalence	United Kin	Female	55+ years	Non-melanoma skin cancer (basal-cell	Percent	2021	0.0002364	0.000300753	0.000181626
Prevalence	United Kin	Both	55+ years	Non-melanoma skin cancer (basal-cell	Percent	2021	0.0002687	0.000342399	0.000204892
Prevalence	United Kin	Male	55+ years	Non-melanoma skin cancer (basal-cell	Rate	2021	30.420251	38.85738712	22.91510964
Prevalence	United Kin	Female	55+ years	Non-melanoma skin cancer (basal-cell	Rate	2021	23.626898	30.06131795	18.15424414
Prevalence	United Kin	Both	55+ years	Non-melanoma skin cancer (basal-cell	Rate	2021	26.845422	34.20233978	20.47183206
Prevalence	Chile	Male	55+ years	Non-melanoma skin cancer (basal-cell	Number	1990	101.45433	132.1771969	77.70131865
Prevalence	Chile	Female	55+ years	Non-melanoma skin cancer (basal-cell	Number	1990	116.91421	146.6338825	90.70660381
Prevalence	Chile	Both	55+ years	Non-melanoma skin cancer (basal-cell	Number	1990	218.36854	278.1688238	169.1676651
Prevalence	Chile	Male	55+ years	Non-melanoma skin cancer (basal-cell	Percent	1990	0.0001339	0.000174408	0.000102518
Prevalence	Chile	Female	55+ years	Non-melanoma skin cancer (basal-cell	Percent	1990	0.0001266	0.000158794	9.82E-05
Prevalence	Chile	Both	55+ years	Non-melanoma skin cancer (basal-cell	Percent	1990	0.0001299	0.000165459	0.000100627
Prevalence	Chile	Male	55+ years	Non-melanoma skin cancer (basal-cell	Rate	1990	13.381965	17.43435312	10.24891025
Prevalence	Chile	Female	55+ years	Non-melanoma skin cancer (basal-cell	Rate	1990	12.658864	15.87675593	9.821240395
Prevalence	Chile	Both	55+ years	Non-melanoma skin cancer (basal-cell	Rate	1990	12.984848	16.54075228	10.05921657
Prevalence	Chile	Male	55+ years	Non-melanoma skin cancer (basal-cell	Number	2021	296.42279	383.8609578	219.3072807
Prevalence	Chile	Female	55+ years	Non-melanoma skin cancer (basal-cell	Number	2021	323.12927	416.4601035	241.4601736
Prevalence	Chile	Both	55+ years	Non-melanoma skin cancer (basal-cell	Number	2021	619.55206	790.5704692	461.02116
Prevalence	Chile	Male	55+ years	Non-melanoma skin cancer (basal-cell	Percent	2021	0.000146	0.000189104	0.000108037
Prevalence	Chile	Female	55+ years	Non-melanoma skin cancer (basal-cell	Percent	2021	0.0001321	0.000170232	9.87E-05
Prevalence	Chile	Both	55+ years	Non-melanoma skin cancer (basal-cell	Percent	2021	0.0001384	0.000176621	0.000102995
Prevalence	Chile	Male	55+ years	Non-melanoma skin cancer (basal-cell	Rate	2021	14.596825	18.90256546	10.79940574
Prevalence	Chile	Female	55+ years	Non-melanoma skin cancer (basal-cell	Rate	2021	13.2054	17.01957293	9.867809663
Prevalence	Chile	Both	55+ years	Non-melanoma skin cancer (basal-cell	Rate	2021	13.836444	17.65579466	10.29597645
Prevalence	United Sta	Male	55+ years	Non-melanoma skin cancer (basal-cell	Number	1990	30017.094	37997.27565	22871.53638
Prevalence	United Sta	Female	55+ years	Non-melanoma skin cancer (basal-cell	Number	1990	29521.85	37271.84394	22224.39552
Prevalence	United Sta	Both	55+ years	Non-melanoma skin cancer (basal-cell	Number	1990	59538.943	75008.95974	44922.42624
Prevalence	United Sta	Male	55+ years	Non-melanoma skin cancer (basal-cell	Percent	1990	0.001328	0.00168113	0.001011802
Prevalence	United Sta	Female	55+ years	Non-melanoma skin cancer (basal-cell	Percent	1990	0.0009896	0.001249313	0.000745005
Prevalence	United Sta	Both	55+ years	Non-melanoma skin cancer (basal-cell	Percent	1990	0.0011355	0.00143043	0.000856724
Prevalence	United Sta	Male	55+ years	Non-melanoma skin cancer (basal-cell	Rate	1990	132.70773	167.9886872	101.1167065
Prevalence	United Sta	Female	55+ years	Non-melanoma skin cancer (basal-cell	Rate	1990	98.922951	124.891931	74.47036095
Prevalence	United Sta	Both	55+ years	Non-melanoma skin cancer (basal-cell	Rate	1990	113.48917	142.9770836	85.62813713
Prevalence	United Sta	Male	55+ years	Non-melanoma skin cancer (basal-cell	Number	2021	152535.27	176800.6401	131992.6687
Prevalence	United Sta	Female	55+ years	Non-melanoma skin cancer (basal-cell	Number	2021	102062.2	117763.7914	88597.87555
Prevalence	United Sta	Both	55+ years	Non-melanoma skin cancer (basal-cell	Number	2021	254597.47	294983.1248	221180.2467
Prevalence	United Sta	Male	55+ years	Non-melanoma skin cancer (basal-cell	Percent	2021	0.0032839	0.00380649	0.002841477
Prevalence	United Sta	Female	55+ years	Non-melanoma skin cancer (basal-cell	Percent	2021	0.0018989	0.002191113	0.001648408
Prevalence	United Sta	Both	55+ years	Non-melanoma skin cancer (basal-cell	Percent	2021	0.002541	0.002943974	0.002207239
Prevalence	United Sta	Male	55+ years	Non-melanoma skin cancer (basal-cell	Rate	2021	328.16347	380.3678375	283.9682364
Prevalence	United Sta	Female	55+ years	Non-melanoma skin cancer (basal-cell	Rate	2021	189.82571	219.0291312	164.783381
Prevalence	United Sta	Both	55+ years	Non-melanoma skin cancer (basal-cell	Rate	2021	253.96824	294.2540832	220.6336066
Prevalence	Bahamas	Male	55+ years	Non-melanoma skin cancer (basal-cell	Number	1990	0.3540366	0.474056137	0.257500224

Prevalence	Bahamas	Female	55+ years	Non-melanoma skin cancer (basal-cell	Number	1990	0.1927841	0.264182674	0.13469812
Prevalence	Bahamas	Both	55+ years	Non-melanoma skin cancer (basal-cell	Number	1990	0.5468207	0.737713345	0.389806787
Prevalence	Bahamas	Male	55+ years	Non-melanoma skin cancer (basal-cell	Percent	1990	3.28E-05	4.39E-05	2.39E-05
Prevalence	Bahamas	Female	55+ years	Non-melanoma skin cancer (basal-cell	Percent	1990	1.40E-05	1.92E-05	9.79E-06
Prevalence	Bahamas	Both	55+ years	Non-melanoma skin cancer (basal-cell	Percent	1990	2.23E-05	3.01E-05	1.59E-05
Prevalence	Bahamas	Male	55+ years	Non-melanoma skin cancer (basal-cell	Rate	1990	3.2804794	4.39257192	2.385979558
Prevalence	Bahamas	Female	55+ years	Non-melanoma skin cancer (basal-cell	Rate	1990	1.4007301	1.919497952	0.978689336
Prevalence	Bahamas	Both	55+ years	Non-melanoma skin cancer (basal-cell	Rate	1990	2.2268916	3.004289399	1.587462673
Prevalence	Bahamas	Male	55+ years	Non-melanoma skin cancer (basal-cell	Number	2021	1.0157791	1.360017054	0.745413061
Prevalence	Bahamas	Female	55+ years	Non-melanoma skin cancer (basal-cell	Number	2021	0.5148689	0.701772723	0.359895509
Prevalence	Bahamas	Both	55+ years	Non-melanoma skin cancer (basal-cell	Number	2021	1.530648	2.058976262	1.109490894
Prevalence	Bahamas	Male	55+ years	Non-melanoma skin cancer (basal-cell	Percent	2021	3.12E-05	4.18E-05	2.29E-05
Prevalence	Bahamas	Female	55+ years	Non-melanoma skin cancer (basal-cell	Percent	2021	1.31E-05	1.78E-05	9.14E-06
Prevalence	Bahamas	Both	55+ years	Non-melanoma skin cancer (basal-cell	Percent	2021	2.13E-05	2.86E-05	1.54E-05
Prevalence	Bahamas	Male	55+ years	Non-melanoma skin cancer (basal-cell	Rate	2021	3.1176476	4.174188794	2.287835169
Prevalence	Bahamas	Female	55+ years	Non-melanoma skin cancer (basal-cell	Rate	2021	1.3075371	1.78218931	0.913973867
Prevalence	Bahamas	Both	55+ years	Non-melanoma skin cancer (basal-cell	Rate	2021	2.1271234	2.861334857	1.541846319
Prevalence	Malta	Male	55+ years	Non-melanoma skin cancer (basal-cell	Number	1990	11.76921	14.14838501	9.710474223
Prevalence	Malta	Female	55+ years	Non-melanoma skin cancer (basal-cell	Number	1990	8.4060053	10.18442471	6.827523027
Prevalence	Malta	Both	55+ years	Non-melanoma skin cancer (basal-cell	Number	1990	20.175215	24.00052242	16.8922228
Prevalence	Malta	Male	55+ years	Non-melanoma skin cancer (basal-cell	Percent	1990	0.0003666	0.000440852	0.000302619
Prevalence	Malta	Female	55+ years	Non-melanoma skin cancer (basal-cell	Percent	1990	0.0002087	0.000252787	0.000169482
Prevalence	Malta	Both	55+ years	Non-melanoma skin cancer (basal-cell	Percent	1990	0.0002787	0.000331563	0.000233377
Prevalence	Malta	Male	55+ years	Non-melanoma skin cancer (basal-cell	Rate	1990	36.615961	44.01796786	30.21089276
Prevalence	Malta	Female	55+ years	Non-melanoma skin cancer (basal-cell	Rate	1990	20.856687	25.26923977	16.94021226
Prevalence	Malta	Both	55+ years	Non-melanoma skin cancer (basal-cell	Rate	1990	27.848649	33.1288716	23.31700413
Prevalence	Malta	Male	55+ years	Non-melanoma skin cancer (basal-cell	Number	2021	23.464193	30.24322535	17.72208866
Prevalence	Malta	Female	55+ years	Non-melanoma skin cancer (basal-cell	Number	2021	18.518762	23.68927167	13.78209479
Prevalence	Malta	Both	55+ years	Non-melanoma skin cancer (basal-cell	Number	2021	41.982955	54.36528754	31.85226038
Prevalence	Malta	Male	55+ years	Non-melanoma skin cancer (basal-cell	Percent	2021	0.0003146	0.000405396	0.000237617
Prevalence	Malta	Female	55+ years	Non-melanoma skin cancer (basal-cell	Percent	2021	0.0002229	0.000285129	0.000165879
Prevalence	Malta	Both	55+ years	Non-melanoma skin cancer (basal-cell	Percent	2021	0.0002663	0.000344772	0.000202031
Prevalence	Malta	Male	55+ years	Non-melanoma skin cancer (basal-cell	Rate	2021	31.418007	40.49497396	23.72946372
Prevalence	Malta	Female	55+ years	Non-melanoma skin cancer (basal-cell	Rate	2021	22.279738	28.50032653	16.58110082
Prevalence	Malta	Both	55+ years	Non-melanoma skin cancer (basal-cell	Rate	2021	26.604629	34.4513218	20.1848003
Prevalence	Italy	Male	55+ years	Non-melanoma skin cancer (basal-cell	Number	1990	2030.4695	2472.478375	1643.109065
Prevalence	Italy	Female	55+ years	Non-melanoma skin cancer (basal-cell	Number	1990	1860.7553	2248.633736	1499.217063
Prevalence	Italy	Both	55+ years	Non-melanoma skin cancer (basal-cell	Number	1990	3891.2248	4687.430821	3150.713833
Prevalence	Italy	Male	55+ years	Non-melanoma skin cancer (basal-cell	Percent	1990	0.0003063	0.000372963	0.000247895
Prevalence	Italy	Female	55+ years	Non-melanoma skin cancer (basal-cell	Percent	1990	0.0002168	0.00026193	0.000174631
Prevalence	Italy	Both	55+ years	Non-melanoma skin cancer (basal-cell	Percent	1990	0.0002558	0.000308104	0.000207098
Prevalence	Italy	Male	55+ years	Non-melanoma skin cancer (basal-cell	Rate	1990	30.615764	37.28044858	24.77507735
Prevalence	Italy	Female	55+ years	Non-melanoma skin cancer (basal-cell	Rate	1990	21.669489	26.18654063	17.45918328
Prevalence	Italy	Both	55+ years	Non-melanoma skin cancer (basal-cell	Rate	1990	25.568056	30.79968383	20.70238338
Prevalence	Italy	Male	55+ years	Non-melanoma skin cancer (basal-cell	Number	2021	3492.1985	4482.291599	2638.305056
Prevalence	Italy	Female	55+ years	Non-melanoma skin cancer (basal-cell	Number	2021	3181.7973	4020.138864	2433.758909
Prevalence	Italy	Both	55+ years	Non-melanoma skin cancer (basal-cell	Number	2021	6673.9958	8561.06467	5029.174196
Prevalence	Italy	Male	55+ years	Non-melanoma skin cancer (basal-cell	Percent	2021	0.0003382	0.000434173	0.000255541
Prevalence	Italy	Female	55+ years	Non-melanoma skin cancer (basal-cell	Percent	2021	0.0002571	0.000324798	0.000196608
Prevalence	Italy	Both	55+ years	Non-melanoma skin cancer (basal-cell	Percent	2021	0.000294	0.000377102	0.00022152
Prevalence	Italy	Male	55+ years	Non-melanoma skin cancer (basal-cell	Rate	2021	33.794289	43.37550004	25.53109242
Prevalence	Italy	Female	55+ years	Non-melanoma skin cancer (basal-cell	Rate	2021	25.694676	32.46472158	19.65387467
Prevalence	Italy	Both	55+ years	Non-melanoma skin cancer (basal-cell	Rate	2021	29.379127	37.68606002	22.13857364
Prevalence	Antigua an	Male	55+ years	Non-melanoma skin cancer (basal-cell	Number	1990	0.1383223	0.180105576	0.100024149
Prevalence	Antigua an	Female	55+ years	Non-melanoma skin cancer (basal-cell	Number	1990	0.0756068	0.100534947	0.054301668
Prevalence	Antigua an	Both	55+ years	Non-melanoma skin cancer (basal-cell	Number	1990	0.213929	0.279698375	0.155520433

Prevalence	Antigua an	Male	55+ years	Non-melanoma skin cancer (basal-cell	Percent	1990	3.66E-05	4.77E-05	2.65E-05
Prevalence	Antigua an	Female	55+ years	Non-melanoma skin cancer (basal-cell	Percent	1990	1.56E-05	2.07E-05	1.12E-05
Prevalence	Antigua an	Both	55+ years	Non-melanoma skin cancer (basal-cell	Percent	1990	2.48E-05	3.24E-05	1.80E-05
Prevalence	Antigua an	Male	55+ years	Non-melanoma skin cancer (basal-cell	Rate	1990	3.6604852	4.766216344	2.646984872
Prevalence	Antigua an	Female	55+ years	Non-melanoma skin cancer (basal-cell	Rate	1990	1.5550698	2.067789705	1.116869645
Prevalence	Antigua an	Both	55+ years	Non-melanoma skin cancer (basal-cell	Rate	1990	2.4758159	3.23696953	1.799849225
Prevalence	Antigua an	Male	55+ years	Non-melanoma skin cancer (basal-cell	Number	2021	0.2928195	0.394671062	0.216609759
Prevalence	Antigua an	Female	55+ years	Non-melanoma skin cancer (basal-cell	Number	2021	0.1326689	0.179583133	0.093889368
Prevalence	Antigua an	Both	55+ years	Non-melanoma skin cancer (basal-cell	Number	2021	0.4254883	0.571646018	0.310099083
Prevalence	Antigua an	Male	55+ years	Non-melanoma skin cancer (basal-cell	Percent	2021	3.27E-05	4.40E-05	2.42E-05
Prevalence	Antigua an	Female	55+ years	Non-melanoma skin cancer (basal-cell	Percent	2021	1.34E-05	1.81E-05	9.48E-06
Prevalence	Antigua an	Both	55+ years	Non-melanoma skin cancer (basal-cell	Percent	2021	2.26E-05	3.03E-05	1.64E-05
Prevalence	Antigua an	Male	55+ years	Non-melanoma skin cancer (basal-cell	Rate	2021	3.2673935	4.403892997	2.417015822
Prevalence	Antigua an	Female	55+ years	Non-melanoma skin cancer (basal-cell	Rate	2021	1.3393105	1.812916374	0.947826056
Prevalence	Antigua an	Both	55+ years	Non-melanoma skin cancer (basal-cell	Rate	2021	2.2551238	3.029771677	1.643551061
Prevalence	Haiti	Male	55+ years	Non-melanoma skin cancer (basal-cell	Number	1990	7.3277521	9.925865839	5.249806468
Prevalence	Haiti	Female	55+ years	Non-melanoma skin cancer (basal-cell	Number	1990	2.647295	3.652060448	1.835668249
Prevalence	Haiti	Both	55+ years	Non-melanoma skin cancer (basal-cell	Number	1990	9.9750471	13.5388761	7.103352054
Prevalence	Haiti	Male	55+ years	Non-melanoma skin cancer (basal-cell	Percent	1990	2.76E-05	3.74E-05	1.98E-05
Prevalence	Haiti	Female	55+ years	Non-melanoma skin cancer (basal-cell	Percent	1990	9.66E-06	1.33E-05	6.70E-06
Prevalence	Haiti	Both	55+ years	Non-melanoma skin cancer (basal-cell	Percent	1990	1.85E-05	2.51E-05	1.32E-05
Prevalence	Haiti	Male	55+ years	Non-melanoma skin cancer (basal-cell	Rate	1990	2.7624403	3.741885835	1.979089459
Prevalence	Haiti	Female	55+ years	Non-melanoma skin cancer (basal-cell	Rate	1990	0.9658456	1.332426711	0.669729716
Prevalence	Haiti	Both	55+ years	Non-melanoma skin cancer (basal-cell	Rate	1990	1.8494413	2.510199273	1.317009554
Prevalence	Haiti	Male	55+ years	Non-melanoma skin cancer (basal-cell	Number	2021	15.782007	21.53778562	11.24185429
Prevalence	Haiti	Female	55+ years	Non-melanoma skin cancer (basal-cell	Number	2021	5.7782034	7.924811132	3.932551259
Prevalence	Haiti	Both	55+ years	Non-melanoma skin cancer (basal-cell	Number	2021	21.56021	29.22968649	15.25403099
Prevalence	Haiti	Male	55+ years	Non-melanoma skin cancer (basal-cell	Percent	2021	2.81E-05	3.83E-05	2.00E-05
Prevalence	Haiti	Female	55+ years	Non-melanoma skin cancer (basal-cell	Percent	2021	9.28E-06	1.27E-05	6.32E-06
Prevalence	Haiti	Both	55+ years	Non-melanoma skin cancer (basal-cell	Percent	2021	1.82E-05	2.47E-05	1.29E-05
Prevalence	Haiti	Male	55+ years	Non-melanoma skin cancer (basal-cell	Rate	2021	2.8052735	3.828371111	1.998255111
Prevalence	Haiti	Female	55+ years	Non-melanoma skin cancer (basal-cell	Rate	2021	0.9282183	1.273052289	0.631730308
Prevalence	Haiti	Both	55+ years	Non-melanoma skin cancer (basal-cell	Rate	2021	1.8192914	2.466456422	1.287164086
Prevalence	Sweden	Male	55+ years	Non-melanoma skin cancer (basal-cell	Number	1990	305.09074	391.783951	224.9270034
Prevalence	Sweden	Female	55+ years	Non-melanoma skin cancer (basal-cell	Number	1990	308.55651	409.0669753	231.0441146
Prevalence	Sweden	Both	55+ years	Non-melanoma skin cancer (basal-cell	Number	1990	613.64725	805.853518	458.7997147
Prevalence	Sweden	Male	55+ years	Non-melanoma skin cancer (basal-cell	Percent	1990	0.0002879	0.000369716	0.000212272
Prevalence	Sweden	Female	55+ years	Non-melanoma skin cancer (basal-cell	Percent	1990	0.0002351	0.000311629	0.000175996
Prevalence	Sweden	Both	55+ years	Non-melanoma skin cancer (basal-cell	Percent	1990	0.0002587	0.000339704	0.000193382
Prevalence	Sweden	Male	55+ years	Non-melanoma skin cancer (basal-cell	Rate	1990	28.758186	36.92998303	21.20186495
Prevalence	Sweden	Female	55+ years	Non-melanoma skin cancer (basal-cell	Rate	1990	23.496699	31.15061004	17.59409963
Prevalence	Sweden	Both	55+ years	Non-melanoma skin cancer (basal-cell	Rate	1990	25.847857	33.94390896	19.32541758
Prevalence	Sweden	Male	55+ years	Non-melanoma skin cancer (basal-cell	Number	2021	612.13727	809.1515309	456.0016988
Prevalence	Sweden	Female	55+ years	Non-melanoma skin cancer (basal-cell	Number	2021	572.29575	722.1752706	443.7605911
Prevalence	Sweden	Both	55+ years	Non-melanoma skin cancer (basal-cell	Number	2021	1184.433	1518.457125	900.3683087
Prevalence	Sweden	Male	55+ years	Non-melanoma skin cancer (basal-cell	Percent	2021	0.0003784	0.000500322	0.000281902
Prevalence	Sweden	Female	55+ years	Non-melanoma skin cancer (basal-cell	Percent	2021	0.0003279	0.000413711	0.000254214
Prevalence	Sweden	Both	55+ years	Non-melanoma skin cancer (basal-cell	Percent	2021	0.0003522	0.000451502	0.000267741
Prevalence	Sweden	Male	55+ years	Non-melanoma skin cancer (basal-cell	Rate	2021	37.784459	49.94525659	28.14691807
Prevalence	Sweden	Female	55+ years	Non-melanoma skin cancer (basal-cell	Rate	2021	32.770132	41.3523587	25.4101018
Prevalence	Sweden	Both	55+ years	Non-melanoma skin cancer (basal-cell	Rate	2021	35.183221	45.10530542	26.74516579
Prevalence	Panama	Male	55+ years	Non-melanoma skin cancer (basal-cell	Number	1990	15.411242	18.57822904	12.79284979
Prevalence	Panama	Female	55+ years	Non-melanoma skin cancer (basal-cell	Number	1990	13.206403	15.94845796	11.01696676
Prevalence	Panama	Both	55+ years	Non-melanoma skin cancer (basal-cell	Number	1990	28.617645	34.20704589	24.06305839
Prevalence	Panama	Male	55+ years	Non-melanoma skin cancer (basal-cell	Percent	1990	0.0001257	0.000151564	0.000104364
Prevalence	Panama	Female	55+ years	Non-melanoma skin cancer (basal-cell	Percent	1990	0.0001104	0.000133287	9.21E-05

Prevalence	Panama	Both	55+ years	Non-melanoma skin cancer (basal-cell	Percent	1990	0.0001181	0.000141216	9.93E-05
Prevalence	Panama	Male	55+ years	Non-melanoma skin cancer (basal-cell	Rate	1990	12.568044	15.1507582	10.43271527
Prevalence	Panama	Female	55+ years	Non-melanoma skin cancer (basal-cell	Rate	1990	11.035882	13.32726987	9.206287501
Prevalence	Panama	Both	55+ years	Non-melanoma skin cancer (basal-cell	Rate	1990	11.811305	14.11820735	9.931499164
Prevalence	Panama	Male	55+ years	Non-melanoma skin cancer (basal-cell	Number	2021	44.559309	53.62786404	37.21888507
Prevalence	Panama	Female	55+ years	Non-melanoma skin cancer (basal-cell	Number	2021	46.739644	55.89646178	39.3919926
Prevalence	Panama	Both	55+ years	Non-melanoma skin cancer (basal-cell	Number	2021	91.298953	107.775926	76.64656414
Prevalence	Panama	Male	55+ years	Non-melanoma skin cancer (basal-cell	Percent	2021	0.0001236	0.000148799	0.000103292
Prevalence	Panama	Female	55+ years	Non-melanoma skin cancer (basal-cell	Percent	2021	0.000122	0.000145948	0.000102851
Prevalence	Panama	Both	55+ years	Non-melanoma skin cancer (basal-cell	Percent	2021	0.0001228	0.000144981	0.000103103
Prevalence	Panama	Male	55+ years	Non-melanoma skin cancer (basal-cell	Rate	2021	12.359683	14.8750826	10.3236256
Prevalence	Panama	Female	55+ years	Non-melanoma skin cancer (basal-cell	Rate	2021	12.202185	14.59272899	10.28395455
Prevalence	Panama	Both	55+ years	Non-melanoma skin cancer (basal-cell	Rate	2021	12.278549	14.49449215	10.30798865
Prevalence	Saint Luci	Male	55+ years	Non-melanoma skin cancer (basal-cell	Number	1990	0.2268548	0.307474858	0.160022927
Prevalence	Saint Luci	Female	55+ years	Non-melanoma skin cancer (basal-cell	Number	1990	0.1193846	0.164381783	0.083208667
Prevalence	Saint Luci	Both	55+ years	Non-melanoma skin cancer (basal-cell	Number	1990	0.3462394	0.468737413	0.247474301
Prevalence	Saint Luci	Male	55+ years	Non-melanoma skin cancer (basal-cell	Percent	1990	3.52E-05	4.78E-05	2.49E-05
Prevalence	Saint Luci	Female	55+ years	Non-melanoma skin cancer (basal-cell	Percent	1990	1.46E-05	2.01E-05	1.02E-05
Prevalence	Saint Luci	Both	55+ years	Non-melanoma skin cancer (basal-cell	Percent	1990	2.37E-05	3.20E-05	1.69E-05
Prevalence	Saint Luci	Male	55+ years	Non-melanoma skin cancer (basal-cell	Rate	1990	3.5235601	4.775768786	2.485512164
Prevalence	Saint Luci	Female	55+ years	Non-melanoma skin cancer (basal-cell	Rate	1990	1.4574423	2.006766774	1.015808348
Prevalence	Saint Luci	Both	55+ years	Non-melanoma skin cancer (basal-cell	Rate	1990	2.366704	3.204033934	1.691599678
Prevalence	Saint Luci	Male	55+ years	Non-melanoma skin cancer (basal-cell	Number	2021	0.6764524	0.91413768	0.486011445
Prevalence	Saint Luci	Female	55+ years	Non-melanoma skin cancer (basal-cell	Number	2021	0.3098285	0.410261115	0.22036541
Prevalence	Saint Luci	Both	55+ years	Non-melanoma skin cancer (basal-cell	Number	2021	0.9862809	1.310575408	0.717750703
Prevalence	Saint Luci	Male	55+ years	Non-melanoma skin cancer (basal-cell	Percent	2021	3.36E-05	4.54E-05	2.41E-05
Prevalence	Saint Luci	Female	55+ years	Non-melanoma skin cancer (basal-cell	Percent	2021	1.41E-05	1.87E-05	1.01E-05
Prevalence	Saint Luci	Both	55+ years	Non-melanoma skin cancer (basal-cell	Percent	2021	2.34E-05	3.12E-05	1.71E-05
Prevalence	Saint Luci	Male	55+ years	Non-melanoma skin cancer (basal-cell	Rate	2021	3.3558321	4.534972028	2.411068222
Prevalence	Saint Luci	Female	55+ years	Non-melanoma skin cancer (basal-cell	Rate	2021	1.4131703	1.871257103	1.005116802
Prevalence	Saint Luci	Both	55+ years	Non-melanoma skin cancer (basal-cell	Rate	2021	2.343719	3.114346609	1.705605382
Prevalence	Trinidad a	Male	55+ years	Non-melanoma skin cancer (basal-cell	Number	1990	1.7220151	2.188220803	1.319577378
Prevalence	Trinidad a	Female	55+ years	Non-melanoma skin cancer (basal-cell	Number	1990	1.0249641	1.322862664	0.768178006
Prevalence	Trinidad a	Both	55+ years	Non-melanoma skin cancer (basal-cell	Number	1990	2.7469792	3.451063942	2.129204052
Prevalence	Trinidad a	Male	55+ years	Non-melanoma skin cancer (basal-cell	Percent	1990	2.64E-05	3.35E-05	2.02E-05
Prevalence	Trinidad a	Female	55+ years	Non-melanoma skin cancer (basal-cell	Percent	1990	1.42E-05	1.84E-05	1.07E-05
Prevalence	Trinidad a	Both	55+ years	Non-melanoma skin cancer (basal-cell	Percent	1990	2.00E-05	2.51E-05	1.55E-05
Prevalence	Trinidad a	Male	55+ years	Non-melanoma skin cancer (basal-cell	Rate	1990	2.6377437	3.351867085	2.021298753
Prevalence	Trinidad a	Female	55+ years	Non-melanoma skin cancer (basal-cell	Rate	1990	1.4232401	1.836894752	1.066673199
Prevalence	Trinidad a	Both	55+ years	Non-melanoma skin cancer (basal-cell	Rate	1990	2.0007148	2.513522803	1.550768988
Prevalence	Trinidad a	Male	55+ years	Non-melanoma skin cancer (basal-cell	Number	2021	4.7409318	6.33509355	3.384374386
Prevalence	Trinidad a	Female	55+ years	Non-melanoma skin cancer (basal-cell	Number	2021	2.8481255	3.8340708	2.040545036
Prevalence	Trinidad a	Both	55+ years	Non-melanoma skin cancer (basal-cell	Number	2021	7.5890573	10.03764178	5.477828815
Prevalence	Trinidad a	Male	55+ years	Non-melanoma skin cancer (basal-cell	Percent	2021	2.84E-05	3.80E-05	2.03E-05
Prevalence	Trinidad a	Female	55+ years	Non-melanoma skin cancer (basal-cell	Percent	2021	1.57E-05	2.11E-05	1.12E-05
Prevalence	Trinidad a	Both	55+ years	Non-melanoma skin cancer (basal-cell	Percent	2021	2.18E-05	2.88E-05	1.57E-05
Prevalence	Trinidad a	Male	55+ years	Non-melanoma skin cancer (basal-cell	Rate	2021	2.8413696	3.796794089	2.028347736
Prevalence	Trinidad a	Female	55+ years	Non-melanoma skin cancer (basal-cell	Rate	2021	1.5693446	2.112609905	1.124359951
Prevalence	Trinidad a	Both	55+ years	Non-melanoma skin cancer (basal-cell	Rate	2021	2.1786426	2.88157451	1.572557801
Prevalence	Bolivia (P	Male	55+ years	Non-melanoma skin cancer (basal-cell	Number	1990	17.888172	23.75662118	13.46304666
Prevalence	Bolivia (P	Female	55+ years	Non-melanoma skin cancer (basal-cell	Number	1990	16.393911	21.68920371	12.50607549
Prevalence	Bolivia (P	Both	55+ years	Non-melanoma skin cancer (basal-cell	Number	1990	34.282083	45.08631884	26.31900575
Prevalence	Bolivia (P	Male	55+ years	Non-melanoma skin cancer (basal-cell	Percent	1990	7.28E-05	9.67E-05	5.48E-05
Prevalence	Bolivia (P	Female	55+ years	Non-melanoma skin cancer (basal-cell	Percent	1990	5.89E-05	7.79E-05	4.49E-05
Prevalence	Bolivia (P	Both	55+ years	Non-melanoma skin cancer (basal-cell	Percent	1990	6.54E-05	8.60E-05	5.02E-05
Prevalence	Bolivia (P	Male	55+ years	Non-melanoma skin cancer (basal-cell	Rate	1990	7.2782119	9.665924646	5.477748435

Prevalence	Bolivia (P	Female	55+ years	Non-melanoma skin cancer (basal-cell	Rate	1990	5.8853531	7.786343231	4.489634455
Prevalence	Bolivia (P	Both	55+ years	Non-melanoma skin cancer (basal-cell	Rate	1990	6.5382467	8.598820364	5.019536047
Prevalence	Bolivia (P	Male	55+ years	Non-melanoma skin cancer (basal-cell	Number	2021	54.363835	71.88675446	40.23120592
Prevalence	Bolivia (P	Female	55+ years	Non-melanoma skin cancer (basal-cell	Number	2021	48.897447	64.73084137	36.43483162
Prevalence	Bolivia (P	Both	55+ years	Non-melanoma skin cancer (basal-cell	Number	2021	103.26128	137.4262411	78.52940048
Prevalence	Bolivia (P	Male	55+ years	Non-melanoma skin cancer (basal-cell	Percent	2021	7.44E-05	9.84E-05	5.51E-05
Prevalence	Bolivia (P	Female	55+ years	Non-melanoma skin cancer (basal-cell	Percent	2021	6.00E-05	7.94E-05	4.47E-05
Prevalence	Bolivia (P	Both	55+ years	Non-melanoma skin cancer (basal-cell	Percent	2021	6.68E-05	8.89E-05	5.08E-05
Prevalence	Bolivia (P	Male	55+ years	Non-melanoma skin cancer (basal-cell	Rate	2021	7.4413756	9.839930158	5.506887317
Prevalence	Bolivia (P	Female	55+ years	Non-melanoma skin cancer (basal-cell	Rate	2021	6.0003489	7.943311068	4.471024864
Prevalence	Bolivia (P	Both	55+ years	Non-melanoma skin cancer (basal-cell	Rate	2021	6.6815382	8.892187496	5.081257754
Prevalence	Israel	Male	55+ years	Non-melanoma skin cancer (basal-cell	Number	1990	101.63749	130.8490234	74.16289376
Prevalence	Israel	Female	55+ years	Non-melanoma skin cancer (basal-cell	Number	1990	87.141324	112.5701084	64.1974147
Prevalence	Israel	Both	55+ years	Non-melanoma skin cancer (basal-cell	Number	1990	188.77881	244.0332555	138.7622457
Prevalence	Israel	Male	55+ years	Non-melanoma skin cancer (basal-cell	Percent	1990	0.0002765	0.000355963	0.000201797
Prevalence	Israel	Female	55+ years	Non-melanoma skin cancer (basal-cell	Percent	1990	0.0001969	0.00025443	0.000145083
Prevalence	Israel	Both	55+ years	Non-melanoma skin cancer (basal-cell	Percent	1990	0.000233	0.000301261	0.000171299
Prevalence	Israel	Male	55+ years	Non-melanoma skin cancer (basal-cell	Rate	1990	27.625928	35.56587101	20.15810165
Prevalence	Israel	Female	55+ years	Non-melanoma skin cancer (basal-cell	Rate	1990	19.686782	25.43159905	14.50334315
Prevalence	Israel	Both	55+ years	Non-melanoma skin cancer (basal-cell	Rate	1990	23.290359	30.10730964	17.11962531
Prevalence	Israel	Male	55+ years	Non-melanoma skin cancer (basal-cell	Number	2021	258.73369	327.3291639	193.3314927
Prevalence	Israel	Female	55+ years	Non-melanoma skin cancer (basal-cell	Number	2021	219.01608	281.4846254	169.6971135
Prevalence	Israel	Both	55+ years	Non-melanoma skin cancer (basal-cell	Number	2021	477.74977	601.7699646	365.2669729
Prevalence	Israel	Male	55+ years	Non-melanoma skin cancer (basal-cell	Percent	2021	0.0002795	0.000353629	0.000208881
Prevalence	Israel	Female	55+ years	Non-melanoma skin cancer (basal-cell	Percent	2021	0.0002044	0.000262679	0.000158369
Prevalence	Israel	Both	55+ years	Non-melanoma skin cancer (basal-cell	Percent	2021	0.0002392	0.000301309	0.000182905
Prevalence	Israel	Male	55+ years	Non-melanoma skin cancer (basal-cell	Rate	2021	27.922555	35.32538253	20.86434601
Prevalence	Israel	Female	55+ years	Non-melanoma skin cancer (basal-cell	Rate	2021	20.431887	26.25954329	15.83094883
Prevalence	Israel	Both	55+ years	Non-melanoma skin cancer (basal-cell	Rate	2021	23.904886	30.11041139	18.27664967
Prevalence	Guyana	Male	55+ years	Non-melanoma skin cancer (basal-cell	Number	1990	0.8534328	1.17012581	0.61407146
Prevalence	Guyana	Female	55+ years	Non-melanoma skin cancer (basal-cell	Number	1990	0.3498256	0.481503888	0.239912364
Prevalence	Guyana	Both	55+ years	Non-melanoma skin cancer (basal-cell	Number	1990	1.2032584	1.632231545	0.85656144
Prevalence	Guyana	Male	55+ years	Non-melanoma skin cancer (basal-cell	Percent	1990	2.90E-05	3.97E-05	2.08E-05
Prevalence	Guyana	Female	55+ years	Non-melanoma skin cancer (basal-cell	Percent	1990	1.10E-05	1.51E-05	7.54E-06
Prevalence	Guyana	Both	55+ years	Non-melanoma skin cancer (basal-cell	Percent	1990	1.96E-05	2.66E-05	1.40E-05
Prevalence	Guyana	Male	55+ years	Non-melanoma skin cancer (basal-cell	Rate	1990	2.896354	3.97113671	2.084016691
Prevalence	Guyana	Female	55+ years	Non-melanoma skin cancer (basal-cell	Rate	1990	1.0989712	1.512636571	0.753680759
Prevalence	Guyana	Both	55+ years	Non-melanoma skin cancer (basal-cell	Rate	1990	1.9629697	2.662787226	1.3973758
Prevalence	Guyana	Male	55+ years	Non-melanoma skin cancer (basal-cell	Number	2021	1.4507938	1.929099738	0.999700601
Prevalence	Guyana	Female	55+ years	Non-melanoma skin cancer (basal-cell	Number	2021	0.6258471	0.847338698	0.425737386
Prevalence	Guyana	Both	55+ years	Non-melanoma skin cancer (basal-cell	Number	2021	2.076641	2.768133034	1.432277909
Prevalence	Guyana	Male	55+ years	Non-melanoma skin cancer (basal-cell	Percent	2021	2.76E-05	3.66E-05	1.90E-05
Prevalence	Guyana	Female	55+ years	Non-melanoma skin cancer (basal-cell	Percent	2021	1.04E-05	1.41E-05	7.10E-06
Prevalence	Guyana	Both	55+ years	Non-melanoma skin cancer (basal-cell	Percent	2021	1.84E-05	2.46E-05	1.27E-05
Prevalence	Guyana	Male	55+ years	Non-melanoma skin cancer (basal-cell	Rate	2021	2.7557654	3.664301795	1.898919291
Prevalence	Guyana	Female	55+ years	Non-melanoma skin cancer (basal-cell	Rate	2021	1.0435712	1.412898152	0.709897432
Prevalence	Guyana	Both	55+ years	Non-melanoma skin cancer (basal-cell	Rate	2021	1.8439781	2.457996735	1.271808248
Prevalence	Suriname	Male	55+ years	Non-melanoma skin cancer (basal-cell	Number	1990	0.6891239	0.927665258	0.509097254
Prevalence	Suriname	Female	55+ years	Non-melanoma skin cancer (basal-cell	Number	1990	0.2858045	0.381221077	0.205834538
Prevalence	Suriname	Both	55+ years	Non-melanoma skin cancer (basal-cell	Number	1990	0.9749283	1.304490429	0.714311252
Prevalence	Suriname	Male	55+ years	Non-melanoma skin cancer (basal-cell	Percent	1990	3.24E-05	4.37E-05	2.40E-05
Prevalence	Suriname	Female	55+ years	Non-melanoma skin cancer (basal-cell	Percent	1990	1.28E-05	1.71E-05	9.24E-06
Prevalence	Suriname	Both	55+ years	Non-melanoma skin cancer (basal-cell	Percent	1990	2.24E-05	3.00E-05	1.64E-05
Prevalence	Suriname	Male	55+ years	Non-melanoma skin cancer (basal-cell	Rate	1990	3.2434686	4.366200787	2.396145391
Prevalence	Suriname	Female	55+ years	Non-melanoma skin cancer (basal-cell	Rate	1990	1.2833259	1.711767627	0.924242966
Prevalence	Suriname	Both	55+ years	Non-melanoma skin cancer (basal-cell	Rate	1990	2.240333	2.997648915	1.641448876

Prevalence	Suriname	Male	55+ years	Non-melanoma skin cancer (basal-cell	Number	2021	1.6636219	2.193652559	1.162218371
Prevalence	Suriname	Female	55+ years	Non-melanoma skin cancer (basal-cell	Number	2021	0.8002416	1.092931084	0.567132411
Prevalence	Suriname	Both	55+ years	Non-melanoma skin cancer (basal-cell	Number	2021	2.4638635	3.271720088	1.730878837
Prevalence	Suriname	Male	55+ years	Non-melanoma skin cancer (basal-cell	Percent	2021	3.21E-05	4.24E-05	2.24E-05
Prevalence	Suriname	Female	55+ years	Non-melanoma skin cancer (basal-cell	Percent	2021	1.34E-05	1.82E-05	9.47E-06
Prevalence	Suriname	Both	55+ years	Non-melanoma skin cancer (basal-cell	Percent	2021	2.21E-05	2.93E-05	1.55E-05
Prevalence	Suriname	Male	55+ years	Non-melanoma skin cancer (basal-cell	Rate	2021	3.2122811	4.235715364	2.244123023
Prevalence	Suriname	Female	55+ years	Non-melanoma skin cancer (basal-cell	Rate	2021	1.3356666	1.824188525	0.946588904
Prevalence	Suriname	Both	55+ years	Non-melanoma skin cancer (basal-cell	Rate	2021	2.2057332	2.928953503	1.54954076
Prevalence	Uruguay	Male	55+ years	Non-melanoma skin cancer (basal-cell	Number	1990	55.304887	68.40852542	43.59241705
Prevalence	Uruguay	Female	55+ years	Non-melanoma skin cancer (basal-cell	Number	1990	51.991056	64.24013526	41.25765876
Prevalence	Uruguay	Both	55+ years	Non-melanoma skin cancer (basal-cell	Number	1990	107.29594	130.8486216	86.1876745
Prevalence	Uruguay	Male	55+ years	Non-melanoma skin cancer (basal-cell	Percent	1990	0.0001854	0.000229341	0.000146135
Prevalence	Uruguay	Female	55+ years	Non-melanoma skin cancer (basal-cell	Percent	1990	0.0001382	0.000170705	0.000109636
Prevalence	Uruguay	Both	55+ years	Non-melanoma skin cancer (basal-cell	Percent	1990	0.0001591	0.000193981	0.000127751
Prevalence	Uruguay	Male	55+ years	Non-melanoma skin cancer (basal-cell	Rate	1990	18.536641	22.92861125	14.61095057
Prevalence	Uruguay	Female	55+ years	Non-melanoma skin cancer (basal-cell	Rate	1990	13.812152	17.0662921	10.96067518
Prevalence	Uruguay	Both	55+ years	Non-melanoma skin cancer (basal-cell	Rate	1990	15.90112	19.39159639	12.77290182
Prevalence	Uruguay	Male	55+ years	Non-melanoma skin cancer (basal-cell	Number	2021	76.336536	98.6517468	56.94824019
Prevalence	Uruguay	Female	55+ years	Non-melanoma skin cancer (basal-cell	Number	2021	81.301838	102.8845259	60.34613575
Prevalence	Uruguay	Both	55+ years	Non-melanoma skin cancer (basal-cell	Number	2021	157.63837	199.8373257	117.8358715
Prevalence	Uruguay	Male	55+ years	Non-melanoma skin cancer (basal-cell	Percent	2021	0.0001961	0.000253425	0.000146285
Prevalence	Uruguay	Female	55+ years	Non-melanoma skin cancer (basal-cell	Percent	2021	0.0001579	0.000199887	0.000117236
Prevalence	Uruguay	Both	55+ years	Non-melanoma skin cancer (basal-cell	Percent	2021	0.0001744	0.000221082	0.000130345
Prevalence	Uruguay	Male	55+ years	Non-melanoma skin cancer (basal-cell	Rate	2021	19.601597	25.33166742	14.62309516
Prevalence	Uruguay	Female	55+ years	Non-melanoma skin cancer (basal-cell	Rate	2021	15.791306	19.98332498	11.7210672
Prevalence	Uruguay	Both	55+ years	Non-melanoma skin cancer (basal-cell	Rate	2021	17.432237	22.09875374	13.03072836
Prevalence	Barbados	Male	55+ years	Non-melanoma skin cancer (basal-cell	Number	1990	0.7854609	1.023548808	0.563418971
Prevalence	Barbados	Female	55+ years	Non-melanoma skin cancer (basal-cell	Number	1990	0.4290794	0.583951396	0.304506624
Prevalence	Barbados	Both	55+ years	Non-melanoma skin cancer (basal-cell	Number	1990	1.2145403	1.602751311	0.874515471
Prevalence	Barbados	Male	55+ years	Non-melanoma skin cancer (basal-cell	Percent	1990	4.00E-05	5.21E-05	2.87E-05
Prevalence	Barbados	Female	55+ years	Non-melanoma skin cancer (basal-cell	Percent	1990	1.58E-05	2.14E-05	1.12E-05
Prevalence	Barbados	Both	55+ years	Non-melanoma skin cancer (basal-cell	Percent	1990	2.59E-05	3.42E-05	1.87E-05
Prevalence	Barbados	Male	55+ years	Non-melanoma skin cancer (basal-cell	Rate	1990	3.9979058	5.209745924	2.867737879
Prevalence	Barbados	Female	55+ years	Non-melanoma skin cancer (basal-cell	Rate	1990	1.5752964	2.143884232	1.117947409
Prevalence	Barbados	Both	55+ years	Non-melanoma skin cancer (basal-cell	Rate	1990	2.5904767	3.418486771	1.865242317
Prevalence	Barbados	Male	55+ years	Non-melanoma skin cancer (basal-cell	Number	2021	1.5174822	1.994320665	1.114867259
Prevalence	Barbados	Female	55+ years	Non-melanoma skin cancer (basal-cell	Number	2021	0.711764	0.977645612	0.48759802
Prevalence	Barbados	Both	55+ years	Non-melanoma skin cancer (basal-cell	Number	2021	2.2292462	2.985962811	1.61908956
Prevalence	Barbados	Male	55+ years	Non-melanoma skin cancer (basal-cell	Percent	2021	3.66E-05	4.81E-05	2.69E-05
Prevalence	Barbados	Female	55+ years	Non-melanoma skin cancer (basal-cell	Percent	2021	1.43E-05	1.97E-05	9.82E-06
Prevalence	Barbados	Both	55+ years	Non-melanoma skin cancer (basal-cell	Percent	2021	2.45E-05	3.28E-05	1.78E-05
Prevalence	Barbados	Male	55+ years	Non-melanoma skin cancer (basal-cell	Rate	2021	3.6611089	4.811539145	2.68975173
Prevalence	Barbados	Female	55+ years	Non-melanoma skin cancer (basal-cell	Rate	2021	1.4329814	1.968275948	0.981672134
Prevalence	Barbados	Both	55+ years	Non-melanoma skin cancer (basal-cell	Rate	2021	2.4465258	3.276997739	1.776898496
Prevalence	Cuba	Male	55+ years	Non-melanoma skin cancer (basal-cell	Number	1990	65.248332	83.90402114	47.2422981
Prevalence	Cuba	Female	55+ years	Non-melanoma skin cancer (basal-cell	Number	1990	36.66941	48.61597415	26.88620409
Prevalence	Cuba	Both	55+ years	Non-melanoma skin cancer (basal-cell	Number	1990	101.91774	129.9700168	74.71512881
Prevalence	Cuba	Male	55+ years	Non-melanoma skin cancer (basal-cell	Percent	1990	7.81E-05	0.000100402	5.65E-05
Prevalence	Cuba	Female	55+ years	Non-melanoma skin cancer (basal-cell	Percent	1990	4.27E-05	5.66E-05	3.13E-05
Prevalence	Cuba	Both	55+ years	Non-melanoma skin cancer (basal-cell	Percent	1990	6.02E-05	7.67E-05	4.41E-05
Prevalence	Cuba	Male	55+ years	Non-melanoma skin cancer (basal-cell	Rate	1990	7.804899	10.03646218	5.651046659
Prevalence	Cuba	Female	55+ years	Non-melanoma skin cancer (basal-cell	Rate	1990	4.2703043	5.661530974	3.131009507
Prevalence	Cuba	Both	55+ years	Non-melanoma skin cancer (basal-cell	Rate	1990	6.0139134	7.669208589	4.408754585
Prevalence	Cuba	Male	55+ years	Non-melanoma skin cancer (basal-cell	Number	2021	102.81397	134.6458798	77.82456392
Prevalence	Cuba	Female	55+ years	Non-melanoma skin cancer (basal-cell	Number	2021	68.427634	90.09225286	50.13642386

Prevalence	Cuba	Both	55+ years	Non-melanoma skin cancer (basal-cell	Number	2021	171.2416	224.138003	127.6994898
Prevalence	Cuba	Male	55+ years	Non-melanoma skin cancer (basal-cell	Percent	2021	6.43E-05	8.42E-05	4.87E-05
Prevalence	Cuba	Female	55+ years	Non-melanoma skin cancer (basal-cell	Percent	2021	3.77E-05	4.96E-05	2.76E-05
Prevalence	Cuba	Both	55+ years	Non-melanoma skin cancer (basal-cell	Percent	2021	5.01E-05	6.56E-05	3.74E-05
Prevalence	Cuba	Male	55+ years	Non-melanoma skin cancer (basal-cell	Rate	2021	6.4285714	8.418901369	4.866077808
Prevalence	Cuba	Female	55+ years	Non-melanoma skin cancer (basal-cell	Rate	2021	3.7644785	4.956336006	2.758205672
Prevalence	Cuba	Both	55+ years	Non-melanoma skin cancer (basal-cell	Rate	2021	5.0113911	6.559406074	3.737129794
Prevalence	Saint Vinc	Male	55+ years	Non-melanoma skin cancer (basal-cell	Number	1990	0.1846878	0.251330234	0.132372135
Prevalence	Saint Vinc	Female	55+ years	Non-melanoma skin cancer (basal-cell	Number	1990	0.0942179	0.126980792	0.066594505
Prevalence	Saint Vinc	Both	55+ years	Non-melanoma skin cancer (basal-cell	Number	1990	0.2789058	0.374791528	0.201449337
Prevalence	Saint Vinc	Male	55+ years	Non-melanoma skin cancer (basal-cell	Percent	1990	3.47E-05	4.73E-05	2.49E-05
Prevalence	Saint Vinc	Female	55+ years	Non-melanoma skin cancer (basal-cell	Percent	1990	1.41E-05	1.90E-05	9.95E-06
Prevalence	Saint Vinc	Both	55+ years	Non-melanoma skin cancer (basal-cell	Percent	1990	2.32E-05	3.12E-05	1.68E-05
Prevalence	Saint Vinc	Male	55+ years	Non-melanoma skin cancer (basal-cell	Rate	1990	3.4716856	4.724401759	2.488276628
Prevalence	Saint Vinc	Female	55+ years	Non-melanoma skin cancer (basal-cell	Rate	1990	1.4073546	1.896740824	0.994737182
Prevalence	Saint Vinc	Both	55+ years	Non-melanoma skin cancer (basal-cell	Rate	1990	2.3214068	3.11948938	1.676716308
Prevalence	Saint Vinc	Male	55+ years	Non-melanoma skin cancer (basal-cell	Number	2021	0.4382471	0.595700168	0.319266379
Prevalence	Saint Vinc	Female	55+ years	Non-melanoma skin cancer (basal-cell	Number	2021	0.1663761	0.227685083	0.117215867
Prevalence	Saint Vinc	Both	55+ years	Non-melanoma skin cancer (basal-cell	Number	2021	0.6046233	0.821024037	0.441116278
Prevalence	Saint Vinc	Male	55+ years	Non-melanoma skin cancer (basal-cell	Percent	2021	3.35E-05	4.55E-05	2.44E-05
Prevalence	Saint Vinc	Female	55+ years	Non-melanoma skin cancer (basal-cell	Percent	2021	1.35E-05	1.85E-05	9.51E-06
Prevalence	Saint Vinc	Both	55+ years	Non-melanoma skin cancer (basal-cell	Percent	2021	2.38E-05	3.23E-05	1.74E-05
Prevalence	Saint Vinc	Male	55+ years	Non-melanoma skin cancer (basal-cell	Rate	2021	3.3470789	4.549614307	2.438372464
Prevalence	Saint Vinc	Female	55+ years	Non-melanoma skin cancer (basal-cell	Rate	2021	1.3491141	1.846257325	0.950482354
Prevalence	Saint Vinc	Both	55+ years	Non-melanoma skin cancer (basal-cell	Rate	2021	2.3780032	3.2291144	1.734924752
Prevalence	Ecuador	Male	55+ years	Non-melanoma skin cancer (basal-cell	Number	1990	57.839549	70.05812895	48.08908221
Prevalence	Ecuador	Female	55+ years	Non-melanoma skin cancer (basal-cell	Number	1990	60.289351	73.60079971	49.89293664
Prevalence	Ecuador	Both	55+ years	Non-melanoma skin cancer (basal-cell	Number	1990	118.1289	143.4563777	98.37005434
Prevalence	Ecuador	Male	55+ years	Non-melanoma skin cancer (basal-cell	Percent	1990	0.0001371	0.000166086	0.000114004
Prevalence	Ecuador	Female	55+ years	Non-melanoma skin cancer (basal-cell	Percent	1990	0.0001377	0.000168089	0.000113945
Prevalence	Ecuador	Both	55+ years	Non-melanoma skin cancer (basal-cell	Percent	1990	0.0001374	0.000166888	0.00011443
Prevalence	Ecuador	Male	55+ years	Non-melanoma skin cancer (basal-cell	Rate	1990	13.708268	16.60413328	11.39735734
Prevalence	Ecuador	Female	55+ years	Non-melanoma skin cancer (basal-cell	Rate	1990	13.767589	16.80737213	11.39347883
Prevalence	Ecuador	Both	55+ years	Non-melanoma skin cancer (basal-cell	Rate	1990	13.738479	16.68408398	11.44051087
Prevalence	Ecuador	Male	55+ years	Non-melanoma skin cancer (basal-cell	Number	2021	133.44352	177.4674971	98.74667961
Prevalence	Ecuador	Female	55+ years	Non-melanoma skin cancer (basal-cell	Number	2021	122.31929	163.4239655	90.77710463
Prevalence	Ecuador	Both	55+ years	Non-melanoma skin cancer (basal-cell	Number	2021	255.76281	339.0284822	190.2693906
Prevalence	Ecuador	Male	55+ years	Non-melanoma skin cancer (basal-cell	Percent	2021	0.0001015	0.000134967	7.51E-05
Prevalence	Ecuador	Female	55+ years	Non-melanoma skin cancer (basal-cell	Percent	2021	8.42E-05	0.000112547	6.25E-05
Prevalence	Ecuador	Both	55+ years	Non-melanoma skin cancer (basal-cell	Percent	2021	9.24E-05	0.000122526	6.88E-05
Prevalence	Ecuador	Male	55+ years	Non-melanoma skin cancer (basal-cell	Rate	2021	10.145427	13.49247702	7.507500401
Prevalence	Ecuador	Female	55+ years	Non-melanoma skin cancer (basal-cell	Rate	2021	8.4229609	11.25344688	6.250951764
Prevalence	Ecuador	Both	55+ years	Non-melanoma skin cancer (basal-cell	Rate	2021	9.24159	12.25026514	6.875087511
Prevalence	Canada	Male	55+ years	Non-melanoma skin cancer (basal-cell	Number	1990	474.71295	620.9370523	347.8071695
Prevalence	Canada	Female	55+ years	Non-melanoma skin cancer (basal-cell	Number	1990	561.08732	729.634356	408.9316058
Prevalence	Canada	Both	55+ years	Non-melanoma skin cancer (basal-cell	Number	1990	1035.8003	1355.894782	768.3095547
Prevalence	Canada	Male	55+ years	Non-melanoma skin cancer (basal-cell	Percent	1990	0.0001934	0.000253031	0.000141687
Prevalence	Canada	Female	55+ years	Non-melanoma skin cancer (basal-cell	Percent	1990	0.0001872	0.000243431	0.000136412
Prevalence	Canada	Both	55+ years	Non-melanoma skin cancer (basal-cell	Percent	1990	0.00019	0.000248684	0.000140955
Prevalence	Canada	Male	55+ years	Non-melanoma skin cancer (basal-cell	Rate	1990	19.304507	25.25080376	14.14380178
Prevalence	Canada	Female	55+ years	Non-melanoma skin cancer (basal-cell	Rate	1990	18.70331	24.32166404	13.63134432
Prevalence	Canada	Both	55+ years	Non-melanoma skin cancer (basal-cell	Rate	1990	18.974126	24.83772143	14.07414421
Prevalence	Canada	Male	55+ years	Non-melanoma skin cancer (basal-cell	Number	2021	1311.8677	1702.064193	966.2948825
Prevalence	Canada	Female	55+ years	Non-melanoma skin cancer (basal-cell	Number	2021	1442.5884	1904.952193	1078.92209
Prevalence	Canada	Both	55+ years	Non-melanoma skin cancer (basal-cell	Number	2021	2754.4562	3521.900541	2042.58856
Prevalence	Canada	Male	55+ years	Non-melanoma skin cancer (basal-cell	Percent	2021	0.0002266	0.000293902	0.000166882

Prevalence	Canada	Female	55+ years	Non-melanoma skin cancer (basal-cell	Percent	2021	0.0002232	0.000294692	0.000166936
Prevalence	Canada	Both	55+ years	Non-melanoma skin cancer (basal-cell	Percent	2021	0.0002248	0.000287467	0.000166663
Prevalence	Canada	Male	55+ years	Non-melanoma skin cancer (basal-cell	Rate	2021	22.615089	29.34162722	16.65781135
Prevalence	Canada	Female	55+ years	Non-melanoma skin cancer (basal-cell	Rate	2021	22.295425	29.44132746	16.6749059
Prevalence	Canada	Both	55+ years	Non-melanoma skin cancer (basal-cell	Rate	2021	22.446537	28.70057336	16.64540555
Prevalence	Grenada	Male	55+ years	Non-melanoma skin cancer (basal-cell	Number	1990	0.1680148	0.224603675	0.124407686
Prevalence	Grenada	Female	55+ years	Non-melanoma skin cancer (basal-cell	Number	1990	0.1016884	0.141418932	0.071567621
Prevalence	Grenada	Both	55+ years	Non-melanoma skin cancer (basal-cell	Number	1990	0.2697032	0.367514034	0.199498452
Prevalence	Grenada	Male	55+ years	Non-melanoma skin cancer (basal-cell	Percent	1990	3.32E-05	4.43E-05	2.46E-05
Prevalence	Grenada	Female	55+ years	Non-melanoma skin cancer (basal-cell	Percent	1990	1.45E-05	2.02E-05	1.02E-05
Prevalence	Grenada	Both	55+ years	Non-melanoma skin cancer (basal-cell	Percent	1990	2.24E-05	3.05E-05	1.65E-05
Prevalence	Grenada	Male	55+ years	Non-melanoma skin cancer (basal-cell	Rate	1990	3.315752	4.432526133	2.455170507
Prevalence	Grenada	Female	55+ years	Non-melanoma skin cancer (basal-cell	Rate	1990	1.454346	2.022572128	1.023559387
Prevalence	Grenada	Both	55+ years	Non-melanoma skin cancer (basal-cell	Rate	1990	2.2364923	3.047580954	1.654325077
Prevalence	Grenada	Male	55+ years	Non-melanoma skin cancer (basal-cell	Number	2021	0.2789189	0.391024895	0.204410245
Prevalence	Grenada	Female	55+ years	Non-melanoma skin cancer (basal-cell	Number	2021	0.1326562	0.178233027	0.091864601
Prevalence	Grenada	Both	55+ years	Non-melanoma skin cancer (basal-cell	Number	2021	0.4115751	0.567681524	0.30016009
Prevalence	Grenada	Male	55+ years	Non-melanoma skin cancer (basal-cell	Percent	2021	2.86E-05	4.00E-05	2.09E-05
Prevalence	Grenada	Female	55+ years	Non-melanoma skin cancer (basal-cell	Percent	2021	1.29E-05	1.73E-05	8.91E-06
Prevalence	Grenada	Both	55+ years	Non-melanoma skin cancer (basal-cell	Percent	2021	2.05E-05	2.83E-05	1.49E-05
Prevalence	Grenada	Male	55+ years	Non-melanoma skin cancer (basal-cell	Rate	2021	2.8547735	4.002193957	2.092167175
Prevalence	Grenada	Female	55+ years	Non-melanoma skin cancer (basal-cell	Rate	2021	1.2858177	1.727587045	0.890430345
Prevalence	Grenada	Both	55+ years	Non-melanoma skin cancer (basal-cell	Rate	2021	2.0489483	2.826094289	1.49428981
Prevalence	Dominica	Male	55+ years	Non-melanoma skin cancer (basal-cell	Number	1990	0.1334898	0.181255249	0.096262564
Prevalence	Dominica	Female	55+ years	Non-melanoma skin cancer (basal-cell	Number	1990	0.0855942	0.11785612	0.059335275
Prevalence	Dominica	Both	55+ years	Non-melanoma skin cancer (basal-cell	Number	1990	0.219084	0.296257073	0.158300856
Prevalence	Dominica	Male	55+ years	Non-melanoma skin cancer (basal-cell	Percent	1990	3.55E-05	4.82E-05	2.56E-05
Prevalence	Dominica	Female	55+ years	Non-melanoma skin cancer (basal-cell	Percent	1990	1.41E-05	1.95E-05	9.80E-06
Prevalence	Dominica	Both	55+ years	Non-melanoma skin cancer (basal-cell	Percent	1990	2.23E-05	3.02E-05	1.61E-05
Prevalence	Dominica	Male	55+ years	Non-melanoma skin cancer (basal-cell	Rate	1990	3.5463106	4.815255415	2.557326405
Prevalence	Dominica	Female	55+ years	Non-melanoma skin cancer (basal-cell	Rate	1990	1.4139369	1.94687394	0.980163789
Prevalence	Dominica	Both	55+ years	Non-melanoma skin cancer (basal-cell	Rate	1990	2.2314987	3.017551791	1.612386924
Prevalence	Dominica	Male	55+ years	Non-melanoma skin cancer (basal-cell	Number	2021	0.2273172	0.300724712	0.167885735
Prevalence	Dominica	Female	55+ years	Non-melanoma skin cancer (basal-cell	Number	2021	0.104746	0.144161675	0.07294248
Prevalence	Dominica	Both	55+ years	Non-melanoma skin cancer (basal-cell	Number	2021	0.3320632	0.438623972	0.243832971
Prevalence	Dominica	Male	55+ years	Non-melanoma skin cancer (basal-cell	Percent	2021	3.05E-05	4.04E-05	2.25E-05
Prevalence	Dominica	Female	55+ years	Non-melanoma skin cancer (basal-cell	Percent	2021	1.37E-05	1.89E-05	9.56E-06
Prevalence	Dominica	Both	55+ years	Non-melanoma skin cancer (basal-cell	Percent	2021	2.20E-05	2.91E-05	1.62E-05
Prevalence	Dominica	Male	55+ years	Non-melanoma skin cancer (basal-cell	Rate	2021	3.0519185	4.037474902	2.254003122
Prevalence	Dominica	Female	55+ years	Non-melanoma skin cancer (basal-cell	Rate	2021	1.372015	1.888300749	0.955436587
Prevalence	Dominica	Both	55+ years	Non-melanoma skin cancer (basal-cell	Rate	2021	2.2016013	2.908106639	1.616629112
Prevalence	Jamaica	Male	55+ years	Non-melanoma skin cancer (basal-cell	Number	1990	6.0702158	7.671957128	4.694990736
Prevalence	Jamaica	Female	55+ years	Non-melanoma skin cancer (basal-cell	Number	1990	4.8418739	6.269500512	3.65339721
Prevalence	Jamaica	Both	55+ years	Non-melanoma skin cancer (basal-cell	Number	1990	10.91209	13.80920499	8.417148236
Prevalence	Jamaica	Male	55+ years	Non-melanoma skin cancer (basal-cell	Percent	1990	4.44E-05	5.61E-05	3.43E-05
Prevalence	Jamaica	Female	55+ years	Non-melanoma skin cancer (basal-cell	Percent	1990	3.07E-05	3.97E-05	2.32E-05
Prevalence	Jamaica	Both	55+ years	Non-melanoma skin cancer (basal-cell	Percent	1990	3.71E-05	4.69E-05	2.86E-05
Prevalence	Jamaica	Male	55+ years	Non-melanoma skin cancer (basal-cell	Rate	1990	4.4386291	5.609845345	3.433044722
Prevalence	Jamaica	Female	55+ years	Non-melanoma skin cancer (basal-cell	Rate	1990	3.0689692	3.973854823	2.315666152
Prevalence	Jamaica	Both	55+ years	Non-melanoma skin cancer (basal-cell	Rate	1990	3.7049472	4.688595584	2.857847651
Prevalence	Jamaica	Male	55+ years	Non-melanoma skin cancer (basal-cell	Number	2021	9.779083	12.80338836	7.234399228
Prevalence	Jamaica	Female	55+ years	Non-melanoma skin cancer (basal-cell	Number	2021	6.2157793	8.130198255	4.479312549
Prevalence	Jamaica	Both	55+ years	Non-melanoma skin cancer (basal-cell	Number	2021	15.994862	20.89741843	12.03449342
Prevalence	Jamaica	Male	55+ years	Non-melanoma skin cancer (basal-cell	Percent	2021	3.83E-05	5.01E-05	2.83E-05
Prevalence	Jamaica	Female	55+ years	Non-melanoma skin cancer (basal-cell	Percent	2021	2.27E-05	2.97E-05	1.64E-05
Prevalence	Jamaica	Both	55+ years	Non-melanoma skin cancer (basal-cell	Percent	2021	3.03E-05	3.95E-05	2.28E-05

Prevalence	Jamaica	Male	55+ years	Non-melanoma skin cancer (basal-cell	Rate	2021	3.827357	5.011015608	2.831413563
Prevalence	Jamaica	Female	55+ years	Non-melanoma skin cancer (basal-cell	Rate	2021	2.2736181	2.973877417	1.638450381
Prevalence	Jamaica	Both	55+ years	Non-melanoma skin cancer (basal-cell	Rate	2021	3.0242209	3.951169367	2.275416072
Prevalence	Oman	Male	55+ years	Non-melanoma skin cancer (basal-cell	Number	1990	1.7325221	2.228002289	1.335955679
Prevalence	Oman	Female	55+ years	Non-melanoma skin cancer (basal-cell	Number	1990	1.077726	1.391727836	0.831326482
Prevalence	Oman	Both	55+ years	Non-melanoma skin cancer (basal-cell	Number	1990	2.8102481	3.597411177	2.172849578
Prevalence	Oman	Male	55+ years	Non-melanoma skin cancer (basal-cell	Percent	1990	3.19E-05	4.10E-05	2.46E-05
Prevalence	Oman	Female	55+ years	Non-melanoma skin cancer (basal-cell	Percent	1990	2.33E-05	3.01E-05	1.80E-05
Prevalence	Oman	Both	55+ years	Non-melanoma skin cancer (basal-cell	Percent	1990	2.80E-05	3.58E-05	2.16E-05
Prevalence	Oman	Male	55+ years	Non-melanoma skin cancer (basal-cell	Rate	1990	3.1909268	4.103493022	2.460538229
Prevalence	Oman	Female	55+ years	Non-melanoma skin cancer (basal-cell	Rate	1990	2.3320633	3.011523738	1.798885796
Prevalence	Oman	Both	55+ years	Non-melanoma skin cancer (basal-cell	Rate	1990	2.7960255	3.579204735	2.161852821
Prevalence	Oman	Male	55+ years	Non-melanoma skin cancer (basal-cell	Number	2021	4.8746499	6.180458748	3.786391443
Prevalence	Oman	Female	55+ years	Non-melanoma skin cancer (basal-cell	Number	2021	3.2640233	4.123162349	2.549323313
Prevalence	Oman	Both	55+ years	Non-melanoma skin cancer (basal-cell	Number	2021	8.1386731	10.16514845	6.430889286
Prevalence	Oman	Male	55+ years	Non-melanoma skin cancer (basal-cell	Percent	2021	2.77E-05	3.52E-05	2.15E-05
Prevalence	Oman	Female	55+ years	Non-melanoma skin cancer (basal-cell	Percent	2021	2.39E-05	3.02E-05	1.87E-05
Prevalence	Oman	Both	55+ years	Non-melanoma skin cancer (basal-cell	Percent	2021	2.61E-05	3.26E-05	2.06E-05
Prevalence	Oman	Male	55+ years	Non-melanoma skin cancer (basal-cell	Rate	2021	2.7717711	3.514266182	2.152977302
Prevalence	Oman	Female	55+ years	Non-melanoma skin cancer (basal-cell	Rate	2021	2.3914558	3.020922281	1.867815756
Prevalence	Oman	Both	55+ years	Non-melanoma skin cancer (basal-cell	Rate	2021	2.605588	3.254362084	2.058842757
Prevalence	Dominican	Male	55+ years	Non-melanoma skin cancer (basal-cell	Number	1990	10.815806	14.33289931	7.736447866
Prevalence	Dominican	Female	55+ years	Non-melanoma skin cancer (basal-cell	Number	1990	4.2545512	5.856449405	3.015145866
Prevalence	Dominican	Both	55+ years	Non-melanoma skin cancer (basal-cell	Number	1990	15.070357	20.11147271	10.84502382
Prevalence	Dominican	Male	55+ years	Non-melanoma skin cancer (basal-cell	Percent	1990	3.56E-05	4.72E-05	2.55E-05
Prevalence	Dominican	Female	55+ years	Non-melanoma skin cancer (basal-cell	Percent	1990	1.38E-05	1.90E-05	9.80E-06
Prevalence	Dominican	Both	55+ years	Non-melanoma skin cancer (basal-cell	Percent	1990	2.47E-05	3.29E-05	1.77E-05
Prevalence	Dominican	Male	55+ years	Non-melanoma skin cancer (basal-cell	Rate	1990	3.5623695	4.720783948	2.548130571
Prevalence	Dominican	Female	55+ years	Non-melanoma skin cancer (basal-cell	Rate	1990	1.3823127	1.9027729	0.979627321
Prevalence	Dominican	Both	55+ years	Non-melanoma skin cancer (basal-cell	Rate	1990	2.4649025	3.289425721	1.773808454
Prevalence	Dominican	Male	55+ years	Non-melanoma skin cancer (basal-cell	Number	2021	29.240617	39.70417417	21.82040544
Prevalence	Dominican	Female	55+ years	Non-melanoma skin cancer (basal-cell	Number	2021	12.714437	17.59800159	8.862335476
Prevalence	Dominican	Both	55+ years	Non-melanoma skin cancer (basal-cell	Number	2021	41.955054	56.9421444	30.94275253
Prevalence	Dominican	Male	55+ years	Non-melanoma skin cancer (basal-cell	Percent	2021	3.64E-05	4.94E-05	2.72E-05
Prevalence	Dominican	Female	55+ years	Non-melanoma skin cancer (basal-cell	Percent	2021	1.47E-05	2.03E-05	1.02E-05
Prevalence	Dominican	Both	55+ years	Non-melanoma skin cancer (basal-cell	Percent	2021	2.51E-05	3.41E-05	1.85E-05
Prevalence	Dominican	Male	55+ years	Non-melanoma skin cancer (basal-cell	Rate	2021	3.6380284	4.939872189	2.714828258
Prevalence	Dominican	Female	55+ years	Non-melanoma skin cancer (basal-cell	Rate	2021	1.4660218	2.029114905	1.021860176
Prevalence	Dominican	Both	55+ years	Non-melanoma skin cancer (basal-cell	Rate	2021	2.5107395	3.40762022	1.851724243
Prevalence	Nicaragua	Male	55+ years	Non-melanoma skin cancer (basal-cell	Number	1990	17.925456	23.74814076	13.61956135
Prevalence	Nicaragua	Female	55+ years	Non-melanoma skin cancer (basal-cell	Number	1990	19.660413	25.19760987	15.04601778
Prevalence	Nicaragua	Both	55+ years	Non-melanoma skin cancer (basal-cell	Number	1990	37.585869	48.70943193	29.07669227
Prevalence	Nicaragua	Male	55+ years	Non-melanoma skin cancer (basal-cell	Percent	1990	0.0001531	0.000202865	0.000116341
Prevalence	Nicaragua	Female	55+ years	Non-melanoma skin cancer (basal-cell	Percent	1990	0.0001497	0.000191798	0.000114529
Prevalence	Nicaragua	Both	55+ years	Non-melanoma skin cancer (basal-cell	Percent	1990	0.0001513	0.000196052	0.000117042
Prevalence	Nicaragua	Male	55+ years	Non-melanoma skin cancer (basal-cell	Rate	1990	15.309139	20.28197101	11.63171262
Prevalence	Nicaragua	Female	55+ years	Non-melanoma skin cancer (basal-cell	Rate	1990	14.964008	19.17849969	11.4518817
Prevalence	Nicaragua	Both	55+ years	Non-melanoma skin cancer (basal-cell	Rate	1990	15.126646	19.60338614	11.70207912
Prevalence	Nicaragua	Male	55+ years	Non-melanoma skin cancer (basal-cell	Number	2021	57.229366	74.68600601	42.73550647
Prevalence	Nicaragua	Female	55+ years	Non-melanoma skin cancer (basal-cell	Number	2021	66.939184	88.56303423	50.50674193
Prevalence	Nicaragua	Both	55+ years	Non-melanoma skin cancer (basal-cell	Number	2021	124.16855	162.8757748	94.17836919
Prevalence	Nicaragua	Male	55+ years	Non-melanoma skin cancer (basal-cell	Percent	2021	0.0001565	0.000204245	0.000116887
Prevalence	Nicaragua	Female	55+ years	Non-melanoma skin cancer (basal-cell	Percent	2021	0.0001493	0.000197563	0.000112664
Prevalence	Nicaragua	Both	55+ years	Non-melanoma skin cancer (basal-cell	Percent	2021	0.0001526	0.000200113	0.000115706
Prevalence	Nicaragua	Male	55+ years	Non-melanoma skin cancer (basal-cell	Rate	2021	15.649127	20.42257116	11.68584275
Prevalence	Nicaragua	Female	55+ years	Non-melanoma skin cancer (basal-cell	Rate	2021	14.930775	19.75397095	11.2655209

Prevalence	Nicaragua	Both	55+ years	Non-melanoma skin cancer (basal-cell	Rate	2021	15.253493	20.0084847	11.56934763
Prevalence	Jordan	Male	55+ years	Non-melanoma skin cancer (basal-cell	Number	1990	5.0232563	6.523027408	3.855847079
Prevalence	Jordan	Female	55+ years	Non-melanoma skin cancer (basal-cell	Number	1990	3.3037348	4.318281215	2.459846388
Prevalence	Jordan	Both	55+ years	Non-melanoma skin cancer (basal-cell	Number	1990	8.3269911	10.78903007	6.391554012
Prevalence	Jordan	Male	55+ years	Non-melanoma skin cancer (basal-cell	Percent	1990	4.53E-05	5.88E-05	3.48E-05
Prevalence	Jordan	Female	55+ years	Non-melanoma skin cancer (basal-cell	Percent	1990	3.18E-05	4.16E-05	2.37E-05
Prevalence	Jordan	Both	55+ years	Non-melanoma skin cancer (basal-cell	Percent	1990	3.88E-05	5.02E-05	2.97E-05
Prevalence	Jordan	Male	55+ years	Non-melanoma skin cancer (basal-cell	Rate	1990	4.5225253	5.87279538	3.471486397
Prevalence	Jordan	Female	55+ years	Non-melanoma skin cancer (basal-cell	Rate	1990	3.1785065	4.154596487	2.366605753
Prevalence	Jordan	Both	55+ years	Non-melanoma skin cancer (basal-cell	Rate	1990	3.8728069	5.017878575	2.972652938
Prevalence	Jordan	Male	55+ years	Non-melanoma skin cancer (basal-cell	Number	2021	27.749098	37.32646522	20.43441293
Prevalence	Jordan	Female	55+ years	Non-melanoma skin cancer (basal-cell	Number	2021	15.456565	20.99820637	10.84077576
Prevalence	Jordan	Both	55+ years	Non-melanoma skin cancer (basal-cell	Number	2021	43.205663	58.48024092	31.50590213
Prevalence	Jordan	Male	55+ years	Non-melanoma skin cancer (basal-cell	Percent	2021	4.20E-05	5.65E-05	3.09E-05
Prevalence	Jordan	Female	55+ years	Non-melanoma skin cancer (basal-cell	Percent	2021	2.60E-05	3.53E-05	1.82E-05
Prevalence	Jordan	Both	55+ years	Non-melanoma skin cancer (basal-cell	Percent	2021	3.44E-05	4.65E-05	2.51E-05
Prevalence	Jordan	Male	55+ years	Non-melanoma skin cancer (basal-cell	Rate	2021	4.1935661	5.640940044	3.088138603
Prevalence	Jordan	Female	55+ years	Non-melanoma skin cancer (basal-cell	Rate	2021	2.5954074	3.525938732	1.820341721
Prevalence	Jordan	Both	55+ years	Non-melanoma skin cancer (basal-cell	Rate	2021	3.436544	4.651471727	2.505954331
Prevalence	Iraq	Male	55+ years	Non-melanoma skin cancer (basal-cell	Number	1990	19.217229	25.73814744	13.83542836
Prevalence	Iraq	Female	55+ years	Non-melanoma skin cancer (basal-cell	Number	1990	13.127214	17.6964152	9.29179458
Prevalence	Iraq	Both	55+ years	Non-melanoma skin cancer (basal-cell	Number	1990	32.344443	43.39656731	22.92829229
Prevalence	Iraq	Male	55+ years	Non-melanoma skin cancer (basal-cell	Percent	1990	3.14E-05	4.21E-05	2.26E-05
Prevalence	Iraq	Female	55+ years	Non-melanoma skin cancer (basal-cell	Percent	1990	2.02E-05	2.72E-05	1.43E-05
Prevalence	Iraq	Both	55+ years	Non-melanoma skin cancer (basal-cell	Percent	1990	2.56E-05	3.44E-05	1.82E-05
Prevalence	Iraq	Male	55+ years	Non-melanoma skin cancer (basal-cell	Rate	1990	3.1392469	4.204477278	2.260098335
Prevalence	Iraq	Female	55+ years	Non-melanoma skin cancer (basal-cell	Rate	1990	2.019186	2.722005859	1.429234057
Prevalence	Iraq	Both	55+ years	Non-melanoma skin cancer (basal-cell	Rate	1990	2.5623734	3.437938625	1.81641237
Prevalence	Iraq	Male	55+ years	Non-melanoma skin cancer (basal-cell	Number	2021	52.014619	64.85474814	41.98694963
Prevalence	Iraq	Female	55+ years	Non-melanoma skin cancer (basal-cell	Number	2021	38.663115	48.09554988	30.98410737
Prevalence	Iraq	Both	55+ years	Non-melanoma skin cancer (basal-cell	Number	2021	90.677734	110.0471579	74.05313337
Prevalence	Iraq	Male	55+ years	Non-melanoma skin cancer (basal-cell	Percent	2021	2.74E-05	3.41E-05	2.21E-05
Prevalence	Iraq	Female	55+ years	Non-melanoma skin cancer (basal-cell	Percent	2021	1.95E-05	2.42E-05	1.56E-05
Prevalence	Iraq	Both	55+ years	Non-melanoma skin cancer (basal-cell	Percent	2021	2.33E-05	2.83E-05	1.91E-05
Prevalence	Iraq	Male	55+ years	Non-melanoma skin cancer (basal-cell	Rate	2021	2.7362735	3.41173949	2.208759393
Prevalence	Iraq	Female	55+ years	Non-melanoma skin cancer (basal-cell	Rate	2021	1.9484876	2.423849731	1.561492082
Prevalence	Iraq	Both	55+ years	Non-melanoma skin cancer (basal-cell	Rate	2021	2.3339319	2.83247674	1.906035392
Prevalence	El Salvado	Male	55+ years	Non-melanoma skin cancer (basal-cell	Number	1990	36.294862	46.85428083	26.96184098
Prevalence	El Salvado	Female	55+ years	Non-melanoma skin cancer (basal-cell	Number	1990	38.698032	50.43499184	29.21285856
Prevalence	El Salvado	Both	55+ years	Non-melanoma skin cancer (basal-cell	Number	1990	74.992894	95.94295944	56.58886299
Prevalence	El Salvado	Male	55+ years	Non-melanoma skin cancer (basal-cell	Percent	1990	0.0001598	0.000206296	0.000118705
Prevalence	El Salvado	Female	55+ years	Non-melanoma skin cancer (basal-cell	Percent	1990	0.0001478	0.00019269	0.000111611
Prevalence	El Salvado	Both	55+ years	Non-melanoma skin cancer (basal-cell	Percent	1990	0.0001534	0.000196242	0.000115752
Prevalence	El Salvado	Male	55+ years	Non-melanoma skin cancer (basal-cell	Rate	1990	15.975215	20.62295263	11.86727785
Prevalence	El Salvado	Female	55+ years	Non-melanoma skin cancer (basal-cell	Rate	1990	14.783586	19.26738873	11.16001968
Prevalence	El Salvado	Both	55+ years	Non-melanoma skin cancer (basal-cell	Rate	1990	15.337277	19.62190928	11.5733509
Prevalence	El Salvado	Male	55+ years	Non-melanoma skin cancer (basal-cell	Number	2021	73.701741	94.5524362	54.91554932
Prevalence	El Salvado	Female	55+ years	Non-melanoma skin cancer (basal-cell	Number	2021	92.875783	118.2041106	69.34632775
Prevalence	El Salvado	Both	55+ years	Non-melanoma skin cancer (basal-cell	Number	2021	166.57752	210.2410433	124.733761
Prevalence	El Salvado	Male	55+ years	Non-melanoma skin cancer (basal-cell	Percent	2021	0.0001729	0.00022182	0.00012882
Prevalence	El Salvado	Female	55+ years	Non-melanoma skin cancer (basal-cell	Percent	2021	0.0001558	0.000198269	0.000116312
Prevalence	El Salvado	Both	55+ years	Non-melanoma skin cancer (basal-cell	Percent	2021	0.0001629	0.000205624	0.000121994
Prevalence	El Salvado	Male	55+ years	Non-melanoma skin cancer (basal-cell	Rate	2021	17.287561	22.17832242	12.88105106
Prevalence	El Salvado	Female	55+ years	Non-melanoma skin cancer (basal-cell	Rate	2021	15.576516	19.82441677	11.63031045
Prevalence	El Salvado	Both	55+ years	Non-melanoma skin cancer (basal-cell	Rate	2021	16.289873	20.5597954	12.19790658
Prevalence	Brazil	Male	55+ years	Non-melanoma skin cancer (basal-cell	Number	1990	2344.8961	2812.205538	1968.29584

Prevalence	Brazil	Female	55+ years	Non-melanoma skin cancer (basal-cell	Number	1990	1994.6132	2367.767221	1667.760735
Prevalence	Brazil	Both	55+ years	Non-melanoma skin cancer (basal-cell	Number	1990	4339.5093	5173.148638	3647.097374
Prevalence	Brazil	Male	55+ years	Non-melanoma skin cancer (basal-cell	Percent	1990	0.0003398	0.000407532	0.000285231
Prevalence	Brazil	Female	55+ years	Non-melanoma skin cancer (basal-cell	Percent	1990	0.0002532	0.000300609	0.000211734
Prevalence	Brazil	Both	55+ years	Non-melanoma skin cancer (basal-cell	Percent	1990	0.0002937	0.000350082	0.000246804
Prevalence	Brazil	Male	55+ years	Non-melanoma skin cancer (basal-cell	Rate	1990	33.976141	40.747175	28.5194286
Prevalence	Brazil	Female	55+ years	Non-melanoma skin cancer (basal-cell	Rate	1990	25.322069	30.05934404	21.1726023
Prevalence	Brazil	Both	55+ years	Non-melanoma skin cancer (basal-cell	Rate	1990	29.363523	35.00438764	24.67827994
Prevalence	Brazil	Male	55+ years	Non-melanoma skin cancer (basal-cell	Number	2021	3660.197	4589.016541	2858.48141
Prevalence	Brazil	Female	55+ years	Non-melanoma skin cancer (basal-cell	Number	2021	3737.2127	4646.165771	2958.607209
Prevalence	Brazil	Both	55+ years	Non-melanoma skin cancer (basal-cell	Number	2021	7397.4097	9189.776487	5814.266819
Prevalence	Brazil	Male	55+ years	Non-melanoma skin cancer (basal-cell	Percent	2021	0.0001878	0.000235436	0.000146661
Prevalence	Brazil	Female	55+ years	Non-melanoma skin cancer (basal-cell	Percent	2021	0.000157	0.000195123	0.000124251
Prevalence	Brazil	Both	55+ years	Non-melanoma skin cancer (basal-cell	Percent	2021	0.0001708	0.000212232	0.000134275
Prevalence	Brazil	Male	55+ years	Non-melanoma skin cancer (basal-cell	Rate	2021	18.77498	23.53935955	14.6625799
Prevalence	Brazil	Female	55+ years	Non-melanoma skin cancer (basal-cell	Rate	2021	15.694219	19.5113176	12.42450824
Prevalence	Brazil	Both	55+ years	Non-melanoma skin cancer (basal-cell	Rate	2021	17.08103	21.21970495	13.42546542
Prevalence	Peru	Male	55+ years	Non-melanoma skin cancer (basal-cell	Number	1990	86.578278	104.4755623	71.31993921
Prevalence	Peru	Female	55+ years	Non-melanoma skin cancer (basal-cell	Number	1990	86.232216	104.9563187	69.81968608
Prevalence	Peru	Both	55+ years	Non-melanoma skin cancer (basal-cell	Number	1990	172.81049	208.9792212	143.606828
Prevalence	Peru	Male	55+ years	Non-melanoma skin cancer (basal-cell	Percent	1990	8.97E-05	0.000108191	7.39E-05
Prevalence	Peru	Female	55+ years	Non-melanoma skin cancer (basal-cell	Percent	1990	8.58E-05	0.000104374	6.94E-05
Prevalence	Peru	Both	55+ years	Non-melanoma skin cancer (basal-cell	Percent	1990	8.77E-05	0.000106018	7.29E-05
Prevalence	Peru	Male	55+ years	Non-melanoma skin cancer (basal-cell	Rate	1990	8.9623001	10.81496848	7.382806822
Prevalence	Peru	Female	55+ years	Non-melanoma skin cancer (basal-cell	Rate	1990	8.5744407	10.4362589	6.942472154
Prevalence	Peru	Both	55+ years	Non-melanoma skin cancer (basal-cell	Rate	1990	8.7644694	10.5988471	7.283340443
Prevalence	Peru	Male	55+ years	Non-melanoma skin cancer (basal-cell	Number	2021	206.87942	269.7783303	153.9710814
Prevalence	Peru	Female	55+ years	Non-melanoma skin cancer (basal-cell	Number	2021	180.58129	231.2786938	132.2693374
Prevalence	Peru	Both	55+ years	Non-melanoma skin cancer (basal-cell	Number	2021	387.4607	496.4872836	291.5903326
Prevalence	Peru	Male	55+ years	Non-melanoma skin cancer (basal-cell	Percent	2021	7.70E-05	0.000100417	5.73E-05
Prevalence	Peru	Female	55+ years	Non-melanoma skin cancer (basal-cell	Percent	2021	6.22E-05	7.96E-05	4.55E-05
Prevalence	Peru	Both	55+ years	Non-melanoma skin cancer (basal-cell	Percent	2021	6.93E-05	8.88E-05	5.21E-05
Prevalence	Peru	Male	55+ years	Non-melanoma skin cancer (basal-cell	Rate	2021	7.6968822	10.03701599	5.728444553
Prevalence	Peru	Female	55+ years	Non-melanoma skin cancer (basal-cell	Rate	2021	6.2149207	7.959732531	4.552207253
Prevalence	Peru	Both	55+ years	Non-melanoma skin cancer (basal-cell	Rate	2021	6.9270521	8.876237663	5.213074288
Prevalence	Costa Rica	Male	55+ years	Non-melanoma skin cancer (basal-cell	Number	1990	32.913718	38.88119326	28.14912847
Prevalence	Costa Rica	Female	55+ years	Non-melanoma skin cancer (basal-cell	Number	1990	35.9095	42.64569603	30.29092055
Prevalence	Costa Rica	Both	55+ years	Non-melanoma skin cancer (basal-cell	Number	1990	68.823218	81.36060128	58.32279907
Prevalence	Costa Rica	Male	55+ years	Non-melanoma skin cancer (basal-cell	Percent	1990	0.0002384	0.00028166	0.000203901
Prevalence	Costa Rica	Female	55+ years	Non-melanoma skin cancer (basal-cell	Percent	1990	0.0002455	0.00029157	0.000207101
Prevalence	Costa Rica	Both	55+ years	Non-melanoma skin cancer (basal-cell	Percent	1990	0.0002421	0.000286155	0.000205117
Prevalence	Costa Rica	Male	55+ years	Non-melanoma skin cancer (basal-cell	Rate	1990	23.83016	28.15072552	20.38050591
Prevalence	Costa Rica	Female	55+ years	Non-melanoma skin cancer (basal-cell	Rate	1990	24.548864	29.15393991	20.70782657
Prevalence	Costa Rica	Both	55+ years	Non-melanoma skin cancer (basal-cell	Rate	1990	24.199822	28.6082539	20.50763413
Prevalence	Costa Rica	Male	55+ years	Non-melanoma skin cancer (basal-cell	Number	2021	84.04308	106.8444643	62.8926853
Prevalence	Costa Rica	Female	55+ years	Non-melanoma skin cancer (basal-cell	Number	2021	93.545382	118.1854186	70.04134975
Prevalence	Costa Rica	Both	55+ years	Non-melanoma skin cancer (basal-cell	Number	2021	177.58846	223.7665275	133.1032243
Prevalence	Costa Rica	Male	55+ years	Non-melanoma skin cancer (basal-cell	Percent	2021	0.0001916	0.00024358	0.000143353
Prevalence	Costa Rica	Female	55+ years	Non-melanoma skin cancer (basal-cell	Percent	2021	0.0001804	0.000227946	0.000135088
Prevalence	Costa Rica	Both	55+ years	Non-melanoma skin cancer (basal-cell	Percent	2021	0.0001855	0.000233802	0.000139049
Prevalence	Costa Rica	Male	55+ years	Non-melanoma skin cancer (basal-cell	Rate	2021	19.148324	24.34337796	14.32943128
Prevalence	Costa Rica	Female	55+ years	Non-melanoma skin cancer (basal-cell	Rate	2021	18.039545	22.79119637	13.50696369
Prevalence	Costa Rica	Both	55+ years	Non-melanoma skin cancer (basal-cell	Rate	2021	18.547815	23.3707757	13.90165739
Prevalence	Egypt	Male	55+ years	Non-melanoma skin cancer (basal-cell	Number	1990	73.37993	93.94063272	56.2022729
Prevalence	Egypt	Female	55+ years	Non-melanoma skin cancer (basal-cell	Number	1990	33.127015	43.82648444	24.88139024
Prevalence	Egypt	Both	55+ years	Non-melanoma skin cancer (basal-cell	Number	1990	106.50694	137.9278139	83.26560147

Prevalence	Egypt	Male	55+ years	Non-melanoma skin cancer (basal-cell	Percent	1990	3.22E-05	4.12E-05	2.47E-05
Prevalence	Egypt	Female	55+ years	Non-melanoma skin cancer (basal-cell	Percent	1990	1.51E-05	2.00E-05	1.13E-05
Prevalence	Egypt	Both	55+ years	Non-melanoma skin cancer (basal-cell	Percent	1990	2.38E-05	3.09E-05	1.86E-05
Prevalence	Egypt	Male	55+ years	Non-melanoma skin cancer (basal-cell	Rate	1990	3.2207715	4.12321623	2.466814595
Prevalence	Egypt	Female	55+ years	Non-melanoma skin cancer (basal-cell	Rate	1990	1.5105315	1.998407846	1.134546065
Prevalence	Egypt	Both	55+ years	Non-melanoma skin cancer (basal-cell	Rate	1990	2.3819576	3.08466457	1.862180249
Prevalence	Egypt	Male	55+ years	Non-melanoma skin cancer (basal-cell	Number	2021	164.31511	220.0805317	122.8883768
Prevalence	Egypt	Female	55+ years	Non-melanoma skin cancer (basal-cell	Number	2021	80.43209	114.7392311	56.74825448
Prevalence	Egypt	Both	55+ years	Non-melanoma skin cancer (basal-cell	Number	2021	244.7472	336.5753544	181.1911477
Prevalence	Egypt	Male	55+ years	Non-melanoma skin cancer (basal-cell	Percent	2021	2.79E-05	3.73E-05	2.08E-05
Prevalence	Egypt	Female	55+ years	Non-melanoma skin cancer (basal-cell	Percent	2021	1.56E-05	2.22E-05	1.10E-05
Prevalence	Egypt	Both	55+ years	Non-melanoma skin cancer (basal-cell	Percent	2021	2.21E-05	3.04E-05	1.64E-05
Prevalence	Egypt	Male	55+ years	Non-melanoma skin cancer (basal-cell	Rate	2021	2.7844153	3.729392802	2.082415125
Prevalence	Egypt	Female	55+ years	Non-melanoma skin cancer (basal-cell	Rate	2021	1.5573717	2.221645973	1.098791841
Prevalence	Egypt	Both	55+ years	Non-melanoma skin cancer (basal-cell	Rate	2021	2.2117348	3.041568664	1.637390586
Prevalence	Colombia	Male	55+ years	Non-melanoma skin cancer (basal-cell	Number	1990	222.70126	295.8903159	169.6569326
Prevalence	Colombia	Female	55+ years	Non-melanoma skin cancer (basal-cell	Number	1990	214.3119	280.8220697	161.4861903
Prevalence	Colombia	Both	55+ years	Non-melanoma skin cancer (basal-cell	Number	1990	437.01317	572.4687385	336.1154089
Prevalence	Colombia	Male	55+ years	Non-melanoma skin cancer (basal-cell	Percent	1990	0.0001593	0.000211728	0.000121392
Prevalence	Colombia	Female	55+ years	Non-melanoma skin cancer (basal-cell	Percent	1990	0.0001447	0.000189631	0.000109049
Prevalence	Colombia	Both	55+ years	Non-melanoma skin cancer (basal-cell	Percent	1990	0.0001518	0.000198878	0.000116771
Prevalence	Colombia	Male	55+ years	Non-melanoma skin cancer (basal-cell	Rate	1990	15.929813	21.16502261	12.13555369
Prevalence	Colombia	Female	55+ years	Non-melanoma skin cancer (basal-cell	Rate	1990	14.471002	18.96197363	10.90404641
Prevalence	Colombia	Both	55+ years	Non-melanoma skin cancer (basal-cell	Rate	1990	15.179389	19.88435671	11.67476622
Prevalence	Colombia	Male	55+ years	Non-melanoma skin cancer (basal-cell	Number	2021	723.87079	931.8543788	552.4948033
Prevalence	Colombia	Female	55+ years	Non-melanoma skin cancer (basal-cell	Number	2021	810.07992	1013.235276	621.1265297
Prevalence	Colombia	Both	55+ years	Non-melanoma skin cancer (basal-cell	Number	2021	1533.9507	1930.098571	1182.128434
Prevalence	Colombia	Male	55+ years	Non-melanoma skin cancer (basal-cell	Percent	2021	0.0001683	0.000216608	0.000128414
Prevalence	Colombia	Female	55+ years	Non-melanoma skin cancer (basal-cell	Percent	2021	0.0001541	0.000192695	0.000118123
Prevalence	Colombia	Both	55+ years	Non-melanoma skin cancer (basal-cell	Percent	2021	0.0001604	0.00020189	0.000123637
Prevalence	Colombia	Male	55+ years	Non-melanoma skin cancer (basal-cell	Rate	2021	16.815827	21.64737503	12.83469014
Prevalence	Colombia	Female	55+ years	Non-melanoma skin cancer (basal-cell	Rate	2021	15.404262	19.26740929	11.8111749
Prevalence	Colombia	Both	55+ years	Non-melanoma skin cancer (basal-cell	Rate	2021	16.039632	20.18192022	12.36083075
Prevalence	Honduras	Male	55+ years	Non-melanoma skin cancer (basal-cell	Number	1990	23.632	30.79756543	17.97835648
Prevalence	Honduras	Female	55+ years	Non-melanoma skin cancer (basal-cell	Number	1990	21.438541	27.73418654	16.1163516
Prevalence	Honduras	Both	55+ years	Non-melanoma skin cancer (basal-cell	Number	1990	45.070541	58.56168741	34.41051446
Prevalence	Honduras	Male	55+ years	Non-melanoma skin cancer (basal-cell	Percent	1990	0.0001451	0.00018907	0.000110369
Prevalence	Honduras	Female	55+ years	Non-melanoma skin cancer (basal-cell	Percent	1990	0.0001258	0.000162774	9.46E-05
Prevalence	Honduras	Both	55+ years	Non-melanoma skin cancer (basal-cell	Percent	1990	0.0001352	0.000175715	0.000103249
Prevalence	Honduras	Male	55+ years	Non-melanoma skin cancer (basal-cell	Rate	1990	14.505251	18.90345378	11.03506157
Prevalence	Honduras	Female	55+ years	Non-melanoma skin cancer (basal-cell	Rate	1990	12.581634	16.27635825	9.458201049
Prevalence	Honduras	Both	55+ years	Non-melanoma skin cancer (basal-cell	Rate	1990	13.521872	17.5694284	10.3236962
Prevalence	Honduras	Male	55+ years	Non-melanoma skin cancer (basal-cell	Number	2021	71.028406	91.87622863	53.66424151
Prevalence	Honduras	Female	55+ years	Non-melanoma skin cancer (basal-cell	Number	2021	70.134715	90.63413318	52.43404726
Prevalence	Honduras	Both	55+ years	Non-melanoma skin cancer (basal-cell	Number	2021	141.16312	179.6886697	107.7334477
Prevalence	Honduras	Male	55+ years	Non-melanoma skin cancer (basal-cell	Percent	2021	0.0001449	0.000187466	0.000109508
Prevalence	Honduras	Female	55+ years	Non-melanoma skin cancer (basal-cell	Percent	2021	0.0001249	0.00016144	9.34E-05
Prevalence	Honduras	Both	55+ years	Non-melanoma skin cancer (basal-cell	Percent	2021	0.0001342	0.000170887	0.000102454
Prevalence	Honduras	Male	55+ years	Non-melanoma skin cancer (basal-cell	Rate	2021	14.489214	18.74199975	10.94706668
Prevalence	Honduras	Female	55+ years	Non-melanoma skin cancer (basal-cell	Rate	2021	12.49163	16.14276297	9.338980436
Prevalence	Honduras	Both	55+ years	Non-melanoma skin cancer (basal-cell	Rate	2021	13.422766	17.08604186	10.24404154
Prevalence	Iran (Isla	Male	55+ years	Non-melanoma skin cancer (basal-cell	Number	1990	157.3138	209.1661034	117.9475323
Prevalence	Iran (Isla	Female	55+ years	Non-melanoma skin cancer (basal-cell	Number	1990	93.598319	126.8212176	69.09201722
Prevalence	Iran (Isla	Both	55+ years	Non-melanoma skin cancer (basal-cell	Number	1990	250.91211	332.1309493	188.8228732
Prevalence	Iran (Isla	Male	55+ years	Non-melanoma skin cancer (basal-cell	Percent	1990	6.38E-05	8.48E-05	4.78E-05
Prevalence	Iran (Isla	Female	55+ years	Non-melanoma skin cancer (basal-cell	Percent	1990	4.37E-05	5.92E-05	3.22E-05

Prevalence	Iran (Isla	Both	55+ years	Non-melanoma skin cancer (basal-cell	Percent	1990	5.44E-05	7.21E-05	4.10E-05
Prevalence	Iran (Isla	Male	55+ years	Non-melanoma skin cancer (basal-cell	Rate	1990	6.3768017	8.47866366	4.781068441
Prevalence	Iran (Isla	Female	55+ years	Non-melanoma skin cancer (basal-cell	Rate	1990	4.3677035	5.918028113	3.224133217
Prevalence	Iran (Isla	Both	55+ years	Non-melanoma skin cancer (basal-cell	Rate	1990	5.4428567	7.204678639	4.095999254
Prevalence	Iran (Isla	Male	55+ years	Non-melanoma skin cancer (basal-cell	Number	2021	350.98814	455.488989	261.5261925
Prevalence	Iran (Isla	Female	55+ years	Non-melanoma skin cancer (basal-cell	Number	2021	231.96035	305.1222121	172.802688
Prevalence	Iran (Isla	Both	55+ years	Non-melanoma skin cancer (basal-cell	Number	2021	582.94849	760.9819274	438.6020854
Prevalence	Iran (Isla	Male	55+ years	Non-melanoma skin cancer (basal-cell	Percent	2021	5.45E-05	7.07E-05	4.06E-05
Prevalence	Iran (Isla	Female	55+ years	Non-melanoma skin cancer (basal-cell	Percent	2021	3.54E-05	4.66E-05	2.64E-05
Prevalence	Iran (Isla	Both	55+ years	Non-melanoma skin cancer (basal-cell	Percent	2021	4.49E-05	5.86E-05	3.38E-05
Prevalence	Iran (Isla	Male	55+ years	Non-melanoma skin cancer (basal-cell	Rate	2021	5.4495338	7.072041333	4.060524157
Prevalence	Iran (Isla	Female	55+ years	Non-melanoma skin cancer (basal-cell	Rate	2021	3.5412493	4.658183353	2.638112117
Prevalence	Iran (Isla	Both	55+ years	Non-melanoma skin cancer (basal-cell	Rate	2021	4.4873462	5.857789196	3.376214946
Prevalence	Guatemala	Male	55+ years	Non-melanoma skin cancer (basal-cell	Number	1990	38.924692	50.27259518	29.25715813
Prevalence	Guatemala	Female	55+ years	Non-melanoma skin cancer (basal-cell	Number	1990	34.814282	45.16421711	25.80721892
Prevalence	Guatemala	Both	55+ years	Non-melanoma skin cancer (basal-cell	Number	1990	73.738974	94.50197348	55.83617862
Prevalence	Guatemala	Male	55+ years	Non-melanoma skin cancer (basal-cell	Percent	1990	0.0001352	0.000174645	0.000101635
Prevalence	Guatemala	Female	55+ years	Non-melanoma skin cancer (basal-cell	Percent	1990	0.0001212	0.000157281	8.99E-05
Prevalence	Guatemala	Both	55+ years	Non-melanoma skin cancer (basal-cell	Percent	1990	0.0001282	0.000164345	9.71E-05
Prevalence	Guatemala	Male	55+ years	Non-melanoma skin cancer (basal-cell	Rate	1990	13.519695	17.4611565	10.16187478
Prevalence	Guatemala	Female	55+ years	Non-melanoma skin cancer (basal-cell	Rate	1990	12.122948	15.72697801	8.986529391
Prevalence	Guatemala	Both	55+ years	Non-melanoma skin cancer (basal-cell	Rate	1990	12.822213	16.43261857	9.70915836
Prevalence	Guatemala	Male	55+ years	Non-melanoma skin cancer (basal-cell	Number	2021	127.8021	171.328403	96.21698456
Prevalence	Guatemala	Female	55+ years	Non-melanoma skin cancer (basal-cell	Number	2021	127.32283	164.7278698	93.86747225
Prevalence	Guatemala	Both	55+ years	Non-melanoma skin cancer (basal-cell	Number	2021	255.12493	335.0520189	191.6211041
Prevalence	Guatemala	Male	55+ years	Non-melanoma skin cancer (basal-cell	Percent	2021	0.00015	0.000201052	0.000112907
Prevalence	Guatemala	Female	55+ years	Non-melanoma skin cancer (basal-cell	Percent	2021	0.0001301	0.000168373	9.59E-05
Prevalence	Guatemala	Both	55+ years	Non-melanoma skin cancer (basal-cell	Percent	2021	0.0001394	0.000183031	0.000104677
Prevalence	Guatemala	Male	55+ years	Non-melanoma skin cancer (basal-cell	Rate	2021	14.994328	20.1010324	11.28861701
Prevalence	Guatemala	Female	55+ years	Non-melanoma skin cancer (basal-cell	Rate	2021	13.012885	16.8358245	9.593618194
Prevalence	Guatemala	Both	55+ years	Non-melanoma skin cancer (basal-cell	Rate	2021	13.935367	18.3011236	10.46667775
Prevalence	Bahrain	Male	55+ years	Non-melanoma skin cancer (basal-cell	Number	1990	0.3957764	0.556144178	0.275889436
Prevalence	Bahrain	Female	55+ years	Non-melanoma skin cancer (basal-cell	Number	1990	0.2099645	0.302472794	0.131669928
Prevalence	Bahrain	Both	55+ years	Non-melanoma skin cancer (basal-cell	Number	1990	0.6057408	0.855689167	0.411947055
Prevalence	Bahrain	Male	55+ years	Non-melanoma skin cancer (basal-cell	Percent	1990	2.59E-05	3.64E-05	1.80E-05
Prevalence	Bahrain	Female	55+ years	Non-melanoma skin cancer (basal-cell	Percent	1990	1.65E-05	2.37E-05	1.03E-05
Prevalence	Bahrain	Both	55+ years	Non-melanoma skin cancer (basal-cell	Percent	1990	2.16E-05	3.05E-05	1.47E-05
Prevalence	Bahrain	Male	55+ years	Non-melanoma skin cancer (basal-cell	Rate	1990	2.5871271	3.635425979	1.803445333
Prevalence	Bahrain	Female	55+ years	Non-melanoma skin cancer (basal-cell	Rate	1990	1.6455849	2.370614028	1.031955881
Prevalence	Bahrain	Both	55+ years	Non-melanoma skin cancer (basal-cell	Rate	1990	2.158952	3.049805801	1.4682417
Prevalence	Bahrain	Male	55+ years	Non-melanoma skin cancer (basal-cell	Number	2021	2.3079451	3.348376308	1.519548188
Prevalence	Bahrain	Female	55+ years	Non-melanoma skin cancer (basal-cell	Number	2021	1.0495288	1.539529824	0.648577376
Prevalence	Bahrain	Both	55+ years	Non-melanoma skin cancer (basal-cell	Number	2021	3.357474	4.846826153	2.165755492
Prevalence	Bahrain	Male	55+ years	Non-melanoma skin cancer (basal-cell	Percent	2021	2.38E-05	3.46E-05	1.57E-05
Prevalence	Bahrain	Female	55+ years	Non-melanoma skin cancer (basal-cell	Percent	2021	1.60E-05	2.35E-05	9.91E-06
Prevalence	Bahrain	Both	55+ years	Non-melanoma skin cancer (basal-cell	Percent	2021	2.07E-05	2.99E-05	1.33E-05
Prevalence	Bahrain	Male	55+ years	Non-melanoma skin cancer (basal-cell	Rate	2021	2.3813985	3.454942766	1.567909797
Prevalence	Bahrain	Female	55+ years	Non-melanoma skin cancer (basal-cell	Rate	2021	1.6033913	2.351977971	0.990847775
Prevalence	Bahrain	Both	55+ years	Non-melanoma skin cancer (basal-cell	Rate	2021	2.0677622	2.985007098	1.333820383
Prevalence	Venezuela	Male	55+ years	Non-melanoma skin cancer (basal-cell	Number	1990	110.2377	142.3926318	83.35828484
Prevalence	Venezuela	Female	55+ years	Non-melanoma skin cancer (basal-cell	Number	1990	117.50744	156.1683549	88.89257852
Prevalence	Venezuela	Both	55+ years	Non-melanoma skin cancer (basal-cell	Number	1990	227.74514	296.6956741	175.7058769
Prevalence	Venezuela	Male	55+ years	Non-melanoma skin cancer (basal-cell	Percent	1990	0.0001486	0.000191888	0.000112337
Prevalence	Venezuela	Female	55+ years	Non-melanoma skin cancer (basal-cell	Percent	1990	0.0001427	0.000189622	0.000107935
Prevalence	Venezuela	Both	55+ years	Non-melanoma skin cancer (basal-cell	Percent	1990	0.0001455	0.000189505	0.000112226
Prevalence	Venezuela	Male	55+ years	Non-melanoma skin cancer (basal-cell	Rate	1990	14.85231	19.18453923	11.23084998

Prevalence	Venezuela	Female	55+ years	Non-melanoma skin cancer (basal-cell	Rate	1990	14.266963	18.96091157	10.79273916
Prevalence	Venezuela	Both	55+ years	Non-melanoma skin cancer (basal-cell	Rate	1990	14.544421	18.94778842	11.22105265
Prevalence	Venezuela	Male	55+ years	Non-melanoma skin cancer (basal-cell	Number	2021	354.79536	473.0159268	263.9264102
Prevalence	Venezuela	Female	55+ years	Non-melanoma skin cancer (basal-cell	Number	2021	411.07128	526.0348567	314.5848968
Prevalence	Venezuela	Both	55+ years	Non-melanoma skin cancer (basal-cell	Number	2021	765.86664	990.0787735	580.8573145
Prevalence	Venezuela	Male	55+ years	Non-melanoma skin cancer (basal-cell	Percent	2021	0.0001474	0.000196452	0.000109619
Prevalence	Venezuela	Female	55+ years	Non-melanoma skin cancer (basal-cell	Percent	2021	0.0001456	0.000186308	0.000111416
Prevalence	Venezuela	Both	55+ years	Non-melanoma skin cancer (basal-cell	Percent	2021	0.0001464	0.000189267	0.00011104
Prevalence	Venezuela	Male	55+ years	Non-melanoma skin cancer (basal-cell	Rate	2021	14.731285	19.63986395	10.95835995
Prevalence	Venezuela	Female	55+ years	Non-melanoma skin cancer (basal-cell	Rate	2021	14.557685	18.62900721	11.14071479
Prevalence	Venezuela	Both	55+ years	Non-melanoma skin cancer (basal-cell	Rate	2021	14.637596	18.92284122	11.10161234
Prevalence	Tunisia	Male	55+ years	Non-melanoma skin cancer (basal-cell	Number	1990	20.84567	28.09707417	15.18059007
Prevalence	Tunisia	Female	55+ years	Non-melanoma skin cancer (basal-cell	Number	1990	13.268266	17.62313709	9.716125568
Prevalence	Tunisia	Both	55+ years	Non-melanoma skin cancer (basal-cell	Number	1990	34.113936	45.22172696	25.14983629
Prevalence	Tunisia	Male	55+ years	Non-melanoma skin cancer (basal-cell	Percent	1990	4.61E-05	6.21E-05	3.35E-05
Prevalence	Tunisia	Female	55+ years	Non-melanoma skin cancer (basal-cell	Percent	1990	3.18E-05	4.22E-05	2.33E-05
Prevalence	Tunisia	Both	55+ years	Non-melanoma skin cancer (basal-cell	Percent	1990	3.92E-05	5.20E-05	2.89E-05
Prevalence	Tunisia	Male	55+ years	Non-melanoma skin cancer (basal-cell	Rate	1990	4.6039804	6.205527543	3.352789305
Prevalence	Tunisia	Female	55+ years	Non-melanoma skin cancer (basal-cell	Rate	1990	3.1746354	4.216604971	2.324731582
Prevalence	Tunisia	Both	55+ years	Non-melanoma skin cancer (basal-cell	Rate	1990	3.9178948	5.193595091	2.888391821
Prevalence	Tunisia	Male	55+ years	Non-melanoma skin cancer (basal-cell	Number	2021	49.583399	64.59736707	37.05427899
Prevalence	Tunisia	Female	55+ years	Non-melanoma skin cancer (basal-cell	Number	2021	36.762667	49.1972225	26.23791165
Prevalence	Tunisia	Both	55+ years	Non-melanoma skin cancer (basal-cell	Number	2021	86.346066	112.2085873	64.74545275
Prevalence	Tunisia	Male	55+ years	Non-melanoma skin cancer (basal-cell	Percent	2021	4.35E-05	5.67E-05	3.25E-05
Prevalence	Tunisia	Female	55+ years	Non-melanoma skin cancer (basal-cell	Percent	2021	3.07E-05	4.11E-05	2.19E-05
Prevalence	Tunisia	Both	55+ years	Non-melanoma skin cancer (basal-cell	Percent	2021	3.70E-05	4.80E-05	2.77E-05
Prevalence	Tunisia	Male	55+ years	Non-melanoma skin cancer (basal-cell	Rate	2021	4.3516349	5.669320032	3.252029853
Prevalence	Tunisia	Female	55+ years	Non-melanoma skin cancer (basal-cell	Rate	2021	3.069402	4.107592393	2.190665262
Prevalence	Tunisia	Both	55+ years	Non-melanoma skin cancer (basal-cell	Rate	2021	3.6945273	4.801118425	2.770292305
Prevalence	Qatar	Male	55+ years	Non-melanoma skin cancer (basal-cell	Number	1990	0.2628942	0.378834559	0.169469169
Prevalence	Qatar	Female	55+ years	Non-melanoma skin cancer (basal-cell	Number	1990	0.0763205	0.112336425	0.04958836
Prevalence	Qatar	Both	55+ years	Non-melanoma skin cancer (basal-cell	Number	1990	0.3392147	0.487346203	0.220204239
Prevalence	Qatar	Male	55+ years	Non-melanoma skin cancer (basal-cell	Percent	1990	2.32E-05	3.35E-05	1.50E-05
Prevalence	Qatar	Female	55+ years	Non-melanoma skin cancer (basal-cell	Percent	1990	1.52E-05	2.24E-05	9.91E-06
Prevalence	Qatar	Both	55+ years	Non-melanoma skin cancer (basal-cell	Percent	1990	2.08E-05	2.99E-05	1.35E-05
Prevalence	Qatar	Male	55+ years	Non-melanoma skin cancer (basal-cell	Rate	1990	2.3205548	3.343954911	1.495896418
Prevalence	Qatar	Female	55+ years	Non-melanoma skin cancer (basal-cell	Rate	1990	1.5244752	2.243879846	0.990509733
Prevalence	Qatar	Both	55+ years	Non-melanoma skin cancer (basal-cell	Rate	1990	2.0765767	2.98339577	1.348028141
Prevalence	Qatar	Male	55+ years	Non-melanoma skin cancer (basal-cell	Number	2021	2.3543419	3.506828341	1.505898938
Prevalence	Qatar	Female	55+ years	Non-melanoma skin cancer (basal-cell	Number	2021	0.6991058	1.049459412	0.434509985
Prevalence	Qatar	Both	55+ years	Non-melanoma skin cancer (basal-cell	Number	2021	3.0534477	4.477926385	1.963719228
Prevalence	Qatar	Male	55+ years	Non-melanoma skin cancer (basal-cell	Percent	2021	2.24E-05	3.33E-05	1.43E-05
Prevalence	Qatar	Female	55+ years	Non-melanoma skin cancer (basal-cell	Percent	2021	1.46E-05	2.19E-05	9.08E-06
Prevalence	Qatar	Both	55+ years	Non-melanoma skin cancer (basal-cell	Percent	2021	1.99E-05	2.92E-05	1.28E-05
Prevalence	Qatar	Male	55+ years	Non-melanoma skin cancer (basal-cell	Rate	2021	2.2355696	3.329915246	1.429929083
Prevalence	Qatar	Female	55+ years	Non-melanoma skin cancer (basal-cell	Rate	2021	1.4603878	2.192254306	0.907663865
Prevalence	Qatar	Both	55+ years	Non-melanoma skin cancer (basal-cell	Rate	2021	1.9933192	2.923232127	1.281934235
Prevalence	Morocco	Male	55+ years	Non-melanoma skin cancer (basal-cell	Number	1990	27.589142	38.0438206	19.30923729
Prevalence	Morocco	Female	55+ years	Non-melanoma skin cancer (basal-cell	Number	1990	16.843517	23.12939295	11.68884205
Prevalence	Morocco	Both	55+ years	Non-melanoma skin cancer (basal-cell	Number	1990	44.432659	61.1049736	31.31734973
Prevalence	Morocco	Male	55+ years	Non-melanoma skin cancer (basal-cell	Percent	1990	2.34E-05	3.23E-05	1.64E-05
Prevalence	Morocco	Female	55+ years	Non-melanoma skin cancer (basal-cell	Percent	1990	1.44E-05	1.98E-05	1.00E-05
Prevalence	Morocco	Both	55+ years	Non-melanoma skin cancer (basal-cell	Percent	1990	1.90E-05	2.61E-05	1.34E-05
Prevalence	Morocco	Male	55+ years	Non-melanoma skin cancer (basal-cell	Rate	1990	2.3435618	3.23163529	1.640224658
Prevalence	Morocco	Female	55+ years	Non-melanoma skin cancer (basal-cell	Rate	1990	1.4425635	1.980917603	1.001091253
Prevalence	Morocco	Both	55+ years	Non-melanoma skin cancer (basal-cell	Rate	1990	1.8949111	2.605932164	1.335585045

Prevalence	Morocco	Male	55+ years	Non-melanoma skin cancer (basal-cell	Number	2021	66.46513	90.6626027	47.04816678
Prevalence	Morocco	Female	55+ years	Non-melanoma skin cancer (basal-cell	Number	2021	42.337095	60.96588222	28.97492952
Prevalence	Morocco	Both	55+ years	Non-melanoma skin cancer (basal-cell	Number	2021	108.80223	151.5920813	76.31814116
Prevalence	Morocco	Male	55+ years	Non-melanoma skin cancer (basal-cell	Percent	2021	2.22E-05	3.03E-05	1.57E-05
Prevalence	Morocco	Female	55+ years	Non-melanoma skin cancer (basal-cell	Percent	2021	1.40E-05	2.02E-05	9.61E-06
Prevalence	Morocco	Both	55+ years	Non-melanoma skin cancer (basal-cell	Percent	2021	1.81E-05	2.52E-05	1.27E-05
Prevalence	Morocco	Male	55+ years	Non-melanoma skin cancer (basal-cell	Rate	2021	2.2200473	3.028283718	1.571488057
Prevalence	Morocco	Female	55+ years	Non-melanoma skin cancer (basal-cell	Rate	2021	1.4046237	2.022673557	0.961305268
Prevalence	Morocco	Both	55+ years	Non-melanoma skin cancer (basal-cell	Rate	2021	1.8109605	2.523176938	1.27027858
Prevalence	Mozambique	Male	55+ years	Non-melanoma skin cancer (basal-cell	Number	1990	10.083124	13.74282899	7.269480298
Prevalence	Mozambique	Female	55+ years	Non-melanoma skin cancer (basal-cell	Number	1990	7.1293005	9.810315501	4.986697603
Prevalence	Mozambique	Both	55+ years	Non-melanoma skin cancer (basal-cell	Number	1990	17.212424	23.36226478	12.31351206
Prevalence	Mozambique	Male	55+ years	Non-melanoma skin cancer (basal-cell	Percent	1990	2.13E-05	2.91E-05	1.54E-05
Prevalence	Mozambique	Female	55+ years	Non-melanoma skin cancer (basal-cell	Percent	1990	1.41E-05	1.94E-05	9.88E-06
Prevalence	Mozambique	Both	55+ years	Non-melanoma skin cancer (basal-cell	Percent	1990	1.76E-05	2.39E-05	1.26E-05
Prevalence	Mozambique	Male	55+ years	Non-melanoma skin cancer (basal-cell	Rate	1990	2.1338752	2.908372708	1.538428377
Prevalence	Mozambique	Female	55+ years	Non-melanoma skin cancer (basal-cell	Rate	1990	1.4126912	1.943941898	0.988128303
Prevalence	Mozambique	Both	55+ years	Non-melanoma skin cancer (basal-cell	Rate	1990	1.7614252	2.390766197	1.26009737
Prevalence	Mozambique	Male	55+ years	Non-melanoma skin cancer (basal-cell	Number	2021	16.340916	22.59827292	11.32712593
Prevalence	Mozambique	Female	55+ years	Non-melanoma skin cancer (basal-cell	Number	2021	13.869876	18.83779363	9.654274222
Prevalence	Mozambique	Both	55+ years	Non-melanoma skin cancer (basal-cell	Number	2021	30.210792	40.70550075	21.44586416
Prevalence	Mozambique	Male	55+ years	Non-melanoma skin cancer (basal-cell	Percent	2021	2.04E-05	2.82E-05	1.41E-05
Prevalence	Mozambique	Female	55+ years	Non-melanoma skin cancer (basal-cell	Percent	2021	1.40E-05	1.91E-05	9.78E-06
Prevalence	Mozambique	Both	55+ years	Non-melanoma skin cancer (basal-cell	Percent	2021	1.69E-05	2.28E-05	1.20E-05
Prevalence	Mozambique	Male	55+ years	Non-melanoma skin cancer (basal-cell	Rate	2021	2.038034	2.818449571	1.412715624
Prevalence	Mozambique	Female	55+ years	Non-melanoma skin cancer (basal-cell	Rate	2021	1.4047773	1.907940985	0.977810132
Prevalence	Mozambique	Both	55+ years	Non-melanoma skin cancer (basal-cell	Rate	2021	1.6885704	2.275150647	1.198672681
Prevalence	Kuwait	Male	55+ years	Non-melanoma skin cancer (basal-cell	Number	1990	1.771019	2.236400851	1.375060528
Prevalence	Kuwait	Female	55+ years	Non-melanoma skin cancer (basal-cell	Number	1990	0.9228996	1.171936693	0.730461278
Prevalence	Kuwait	Both	55+ years	Non-melanoma skin cancer (basal-cell	Number	1990	2.6939186	3.367175799	2.125185734
Prevalence	Kuwait	Male	55+ years	Non-melanoma skin cancer (basal-cell	Percent	1990	3.08E-05	3.89E-05	2.39E-05
Prevalence	Kuwait	Female	55+ years	Non-melanoma skin cancer (basal-cell	Percent	1990	2.72E-05	3.46E-05	2.15E-05
Prevalence	Kuwait	Both	55+ years	Non-melanoma skin cancer (basal-cell	Percent	1990	2.95E-05	3.68E-05	2.33E-05
Prevalence	Kuwait	Male	55+ years	Non-melanoma skin cancer (basal-cell	Rate	1990	3.0785396	3.887506882	2.390250059
Prevalence	Kuwait	Female	55+ years	Non-melanoma skin cancer (basal-cell	Rate	1990	2.7216288	3.456038619	2.154128635
Prevalence	Kuwait	Both	55+ years	Non-melanoma skin cancer (basal-cell	Rate	1990	2.9461786	3.682480026	2.324189316
Prevalence	Kuwait	Male	55+ years	Non-melanoma skin cancer (basal-cell	Number	2021	8.954334	12.06446857	6.587804298
Prevalence	Kuwait	Female	55+ years	Non-melanoma skin cancer (basal-cell	Number	2021	4.2943259	5.816653486	3.011978461
Prevalence	Kuwait	Both	55+ years	Non-melanoma skin cancer (basal-cell	Number	2021	13.24866	17.91898235	9.684744249
Prevalence	Kuwait	Male	55+ years	Non-melanoma skin cancer (basal-cell	Percent	2021	3.29E-05	4.44E-05	2.42E-05
Prevalence	Kuwait	Female	55+ years	Non-melanoma skin cancer (basal-cell	Percent	2021	2.21E-05	3.00E-05	1.55E-05
Prevalence	Kuwait	Both	55+ years	Non-melanoma skin cancer (basal-cell	Percent	2021	2.84E-05	3.84E-05	2.08E-05
Prevalence	Kuwait	Male	55+ years	Non-melanoma skin cancer (basal-cell	Rate	2021	3.2921916	4.435677981	2.422102415
Prevalence	Kuwait	Female	55+ years	Non-melanoma skin cancer (basal-cell	Rate	2021	2.2108721	2.994620606	1.550673903
Prevalence	Kuwait	Both	55+ years	Non-melanoma skin cancer (basal-cell	Rate	2021	2.8416955	3.843429623	2.077273821
Prevalence	Libya	Male	55+ years	Non-melanoma skin cancer (basal-cell	Number	1990	5.0020462	6.594516825	3.527232571
Prevalence	Libya	Female	55+ years	Non-melanoma skin cancer (basal-cell	Number	1990	2.4399291	3.344320454	1.656715934
Prevalence	Libya	Both	55+ years	Non-melanoma skin cancer (basal-cell	Number	1990	7.4419753	9.937205386	5.1600868
Prevalence	Libya	Male	55+ years	Non-melanoma skin cancer (basal-cell	Percent	1990	3.05E-05	4.01E-05	2.15E-05
Prevalence	Libya	Female	55+ years	Non-melanoma skin cancer (basal-cell	Percent	1990	1.66E-05	2.28E-05	1.13E-05
Prevalence	Libya	Both	55+ years	Non-melanoma skin cancer (basal-cell	Percent	1990	2.39E-05	3.20E-05	1.66E-05
Prevalence	Libya	Male	55+ years	Non-melanoma skin cancer (basal-cell	Rate	1990	3.043675	4.012671017	2.146271559
Prevalence	Libya	Female	55+ years	Non-melanoma skin cancer (basal-cell	Rate	1990	1.6633402	2.279878841	1.129410789
Prevalence	Libya	Both	55+ years	Non-melanoma skin cancer (basal-cell	Rate	1990	2.3926808	3.194926055	1.659027375
Prevalence	Libya	Male	55+ years	Non-melanoma skin cancer (basal-cell	Number	2021	12.30409	16.46817822	8.593051297
Prevalence	Libya	Female	55+ years	Non-melanoma skin cancer (basal-cell	Number	2021	6.4848844	9.058612596	4.251103811

Prevalence	Libya	Both	55+ years	Non-melanoma skin cancer (basal-cell	Number	2021	18.788975	25.5177346	13.30536433
Prevalence	Libya	Male	55+ years	Non-melanoma skin cancer (basal-cell	Percent	2021	2.93E-05	3.92E-05	2.05E-05
Prevalence	Libya	Female	55+ years	Non-melanoma skin cancer (basal-cell	Percent	2021	1.56E-05	2.18E-05	1.02E-05
Prevalence	Libya	Both	55+ years	Non-melanoma skin cancer (basal-cell	Percent	2021	2.25E-05	3.05E-05	1.59E-05
Prevalence	Libya	Male	55+ years	Non-melanoma skin cancer (basal-cell	Rate	2021	2.9272858	3.91797051	2.044386521
Prevalence	Libya	Female	55+ years	Non-melanoma skin cancer (basal-cell	Rate	2021	1.5588953	2.177591463	1.021918894
Prevalence	Libya	Both	55+ years	Non-melanoma skin cancer (basal-cell	Rate	2021	2.2466345	3.051205482	1.590948461
Prevalence	Algeria	Male	55+ years	Non-melanoma skin cancer (basal-cell	Number	1990	48.224523	60.58914644	38.67325568
Prevalence	Algeria	Female	55+ years	Non-melanoma skin cancer (basal-cell	Number	1990	29.377796	37.82814391	22.89543572
Prevalence	Algeria	Both	55+ years	Non-melanoma skin cancer (basal-cell	Number	1990	77.602319	96.29269879	62.05926987
Prevalence	Algeria	Male	55+ years	Non-melanoma skin cancer (basal-cell	Percent	1990	4.71E-05	5.92E-05	3.78E-05
Prevalence	Algeria	Female	55+ years	Non-melanoma skin cancer (basal-cell	Percent	1990	2.74E-05	3.53E-05	2.14E-05
Prevalence	Algeria	Both	55+ years	Non-melanoma skin cancer (basal-cell	Percent	1990	3.71E-05	4.60E-05	2.96E-05
Prevalence	Algeria	Male	55+ years	Non-melanoma skin cancer (basal-cell	Rate	1990	4.7068716	5.913699391	3.774636581
Prevalence	Algeria	Female	55+ years	Non-melanoma skin cancer (basal-cell	Rate	1990	2.7443679	3.533768962	2.138809144
Prevalence	Algeria	Both	55+ years	Non-melanoma skin cancer (basal-cell	Rate	1990	3.7041121	4.596240868	2.962211632
Prevalence	Algeria	Male	55+ years	Non-melanoma skin cancer (basal-cell	Number	2021	131.23694	173.0979972	96.97148479
Prevalence	Algeria	Female	55+ years	Non-melanoma skin cancer (basal-cell	Number	2021	78.257367	108.507622	56.72406525
Prevalence	Algeria	Both	55+ years	Non-melanoma skin cancer (basal-cell	Number	2021	209.4943	278.210561	156.3079044
Prevalence	Algeria	Male	55+ years	Non-melanoma skin cancer (basal-cell	Percent	2021	4.25E-05	5.60E-05	3.14E-05
Prevalence	Algeria	Female	55+ years	Non-melanoma skin cancer (basal-cell	Percent	2021	2.62E-05	3.64E-05	1.90E-05
Prevalence	Algeria	Both	55+ years	Non-melanoma skin cancer (basal-cell	Percent	2021	3.45E-05	4.58E-05	2.57E-05
Prevalence	Algeria	Male	55+ years	Non-melanoma skin cancer (basal-cell	Rate	2021	4.2459654	5.600314443	3.137360429
Prevalence	Algeria	Female	55+ years	Non-melanoma skin cancer (basal-cell	Rate	2021	2.6238777	3.638133342	1.9018914
Prevalence	Algeria	Both	55+ years	Non-melanoma skin cancer (basal-cell	Rate	2021	3.4493913	4.58082664	2.573660071
Prevalence	Paraguay	Male	55+ years	Non-melanoma skin cancer (basal-cell	Number	1990	0.0922832	0.178601076	0.03065472
Prevalence	Paraguay	Female	55+ years	Non-melanoma skin cancer (basal-cell	Number	1990	0.0833755	0.162242725	0.031707125
Prevalence	Paraguay	Both	55+ years	Non-melanoma skin cancer (basal-cell	Number	1990	0.1756587	0.329347938	0.071072995
Prevalence	Paraguay	Male	55+ years	Non-melanoma skin cancer (basal-cell	Percent	1990	5.33E-07	1.03E-06	1.77E-07
Prevalence	Paraguay	Female	55+ years	Non-melanoma skin cancer (basal-cell	Percent	1990	4.40E-07	8.57E-07	1.67E-07
Prevalence	Paraguay	Both	55+ years	Non-melanoma skin cancer (basal-cell	Percent	1990	4.84E-07	9.08E-07	1.96E-07
Prevalence	Paraguay	Male	55+ years	Non-melanoma skin cancer (basal-cell	Rate	1990	0.0532406	0.10303963	0.017685509
Prevalence	Paraguay	Female	55+ years	Non-melanoma skin cancer (basal-cell	Rate	1990	0.0440118	0.085643712	0.016737366
Prevalence	Paraguay	Both	55+ years	Non-melanoma skin cancer (basal-cell	Rate	1990	0.0484213	0.090786591	0.019591666
Prevalence	Paraguay	Male	55+ years	Non-melanoma skin cancer (basal-cell	Number	2021	0.1859304	0.358282219	0.072476059
Prevalence	Paraguay	Female	55+ years	Non-melanoma skin cancer (basal-cell	Number	2021	0.1731902	0.338972618	0.055838288
Prevalence	Paraguay	Both	55+ years	Non-melanoma skin cancer (basal-cell	Number	2021	0.3591207	0.685467563	0.139688732
Prevalence	Paraguay	Male	55+ years	Non-melanoma skin cancer (basal-cell	Percent	2021	3.93E-07	7.56E-07	1.53E-07
Prevalence	Paraguay	Female	55+ years	Non-melanoma skin cancer (basal-cell	Percent	2021	3.35E-07	6.57E-07	1.08E-07
Prevalence	Paraguay	Both	55+ years	Non-melanoma skin cancer (basal-cell	Percent	2021	3.63E-07	6.92E-07	1.41E-07
Prevalence	Paraguay	Male	55+ years	Non-melanoma skin cancer (basal-cell	Rate	2021	0.0392416	0.075617349	0.015296454
Prevalence	Paraguay	Female	55+ years	Non-melanoma skin cancer (basal-cell	Rate	2021	0.0335448	0.065654754	0.010815177
Prevalence	Paraguay	Both	55+ years	Non-melanoma skin cancer (basal-cell	Rate	2021	0.036271	0.069231793	0.014108474
Prevalence	Mexico	Male	55+ years	Non-melanoma skin cancer (basal-cell	Number	1990	520.72532	662.9864395	395.7323327
Prevalence	Mexico	Female	55+ years	Non-melanoma skin cancer (basal-cell	Number	1990	498.92482	646.5437148	382.9410755
Prevalence	Mexico	Both	55+ years	Non-melanoma skin cancer (basal-cell	Number	1990	1019.6501	1304.705469	778.5920991
Prevalence	Mexico	Male	55+ years	Non-melanoma skin cancer (basal-cell	Percent	1990	0.0001559	0.000198496	0.000118476
Prevalence	Mexico	Female	55+ years	Non-melanoma skin cancer (basal-cell	Percent	1990	0.0001382	0.000179044	0.000106047
Prevalence	Mexico	Both	55+ years	Non-melanoma skin cancer (basal-cell	Percent	1990	0.0001467	0.000187687	0.000112007
Prevalence	Mexico	Male	55+ years	Non-melanoma skin cancer (basal-cell	Rate	1990	15.584672	19.84237372	11.84378499
Prevalence	Mexico	Female	55+ years	Non-melanoma skin cancer (basal-cell	Rate	1990	13.814992	17.90248879	10.60345674
Prevalence	Mexico	Both	55+ years	Non-melanoma skin cancer (basal-cell	Rate	1990	14.665444	18.76534327	11.19834963
Prevalence	Mexico	Male	55+ years	Non-melanoma skin cancer (basal-cell	Number	2021	1585.6794	2010.817976	1215.143691
Prevalence	Mexico	Female	55+ years	Non-melanoma skin cancer (basal-cell	Number	2021	1650.5614	2110.759612	1266.275855
Prevalence	Mexico	Both	55+ years	Non-melanoma skin cancer (basal-cell	Number	2021	3236.2408	4130.162222	2492.186847
Prevalence	Mexico	Male	55+ years	Non-melanoma skin cancer (basal-cell	Percent	2021	0.0001585	0.000200968	0.000121441

Prevalence	Mexico	Female	55+ years	Non-melanoma skin cancer (basal-cell	Percent	2021	0.000143	0.000182927	0.000109741
Prevalence	Mexico	Both	55+ years	Non-melanoma skin cancer (basal-cell	Percent	2021	0.0001502	0.000191699	0.000115676
Prevalence	Mexico	Male	55+ years	Non-melanoma skin cancer (basal-cell	Rate	2021	15.840584	20.0876234	12.13901464
Prevalence	Mexico	Female	55+ years	Non-melanoma skin cancer (basal-cell	Rate	2021	14.302697	18.29047693	10.97272716
Prevalence	Mexico	Both	55+ years	Non-melanoma skin cancer (basal-cell	Rate	2021	15.017049	19.16509087	11.56443375
Prevalence	Lebanon	Male	55+ years	Non-melanoma skin cancer (basal-cell	Number	1990	10.986384	14.63179153	8.176949092
Prevalence	Lebanon	Female	55+ years	Non-melanoma skin cancer (basal-cell	Number	1990	7.7372051	10.27653908	5.736815881
Prevalence	Lebanon	Both	55+ years	Non-melanoma skin cancer (basal-cell	Number	1990	18.72359	24.61978674	14.085389
Prevalence	Lebanon	Male	55+ years	Non-melanoma skin cancer (basal-cell	Percent	1990	5.85E-05	7.79E-05	4.36E-05
Prevalence	Lebanon	Female	55+ years	Non-melanoma skin cancer (basal-cell	Percent	1990	4.05E-05	5.37E-05	3.00E-05
Prevalence	Lebanon	Both	55+ years	Non-melanoma skin cancer (basal-cell	Percent	1990	4.94E-05	6.50E-05	3.72E-05
Prevalence	Lebanon	Male	55+ years	Non-melanoma skin cancer (basal-cell	Rate	1990	5.8483027	7.788835867	4.35277623
Prevalence	Lebanon	Female	55+ years	Non-melanoma skin cancer (basal-cell	Rate	1990	4.0450243	5.372592503	2.999217321
Prevalence	Lebanon	Both	55+ years	Non-melanoma skin cancer (basal-cell	Rate	1990	4.9385274	6.493706335	3.715157273
Prevalence	Lebanon	Male	55+ years	Non-melanoma skin cancer (basal-cell	Number	2021	31.553817	40.81887423	23.47177196
Prevalence	Lebanon	Female	55+ years	Non-melanoma skin cancer (basal-cell	Number	2021	23.938125	31.20797722	17.76864326
Prevalence	Lebanon	Both	55+ years	Non-melanoma skin cancer (basal-cell	Number	2021	55.491942	71.70002512	41.5008914
Prevalence	Lebanon	Male	55+ years	Non-melanoma skin cancer (basal-cell	Percent	2021	7.10E-05	9.19E-05	5.28E-05
Prevalence	Lebanon	Female	55+ years	Non-melanoma skin cancer (basal-cell	Percent	2021	4.47E-05	5.83E-05	3.32E-05
Prevalence	Lebanon	Both	55+ years	Non-melanoma skin cancer (basal-cell	Percent	2021	5.66E-05	7.32E-05	4.24E-05
Prevalence	Lebanon	Male	55+ years	Non-melanoma skin cancer (basal-cell	Rate	2021	7.099136	9.183635106	5.280797009
Prevalence	Lebanon	Female	55+ years	Non-melanoma skin cancer (basal-cell	Rate	2021	4.4697838	5.827227937	3.317803447
Prevalence	Lebanon	Both	55+ years	Non-melanoma skin cancer (basal-cell	Rate	2021	5.6622786	7.31611655	4.234661813
Prevalence	Equatorial	Male	55+ years	Non-melanoma skin cancer (basal-cell	Number	1990	0.3914075	0.535242618	0.27830119
Prevalence	Equatorial	Female	55+ years	Non-melanoma skin cancer (basal-cell	Number	1990	0.2875264	0.390543311	0.201784098
Prevalence	Equatorial	Both	55+ years	Non-melanoma skin cancer (basal-cell	Number	1990	0.6789339	0.925272373	0.491506436
Prevalence	Equatorial	Male	55+ years	Non-melanoma skin cancer (basal-cell	Percent	1990	2.71E-05	3.70E-05	1.93E-05
Prevalence	Equatorial	Female	55+ years	Non-melanoma skin cancer (basal-cell	Percent	1990	1.65E-05	2.24E-05	1.15E-05
Prevalence	Equatorial	Both	55+ years	Non-melanoma skin cancer (basal-cell	Percent	1990	2.13E-05	2.90E-05	1.54E-05
Prevalence	Equatorial	Male	55+ years	Non-melanoma skin cancer (basal-cell	Rate	1990	2.7087364	3.704147755	1.925984019
Prevalence	Equatorial	Female	55+ years	Non-melanoma skin cancer (basal-cell	Rate	1990	1.6457265	2.235368251	1.154959649
Prevalence	Equatorial	Both	55+ years	Non-melanoma skin cancer (basal-cell	Rate	1990	2.1269251	2.89864004	1.539763076
Prevalence	Equatorial	Male	55+ years	Non-melanoma skin cancer (basal-cell	Number	2021	0.9263414	1.243944671	0.649235373
Prevalence	Equatorial	Female	55+ years	Non-melanoma skin cancer (basal-cell	Number	2021	0.7620228	1.061579431	0.540980933
Prevalence	Equatorial	Both	55+ years	Non-melanoma skin cancer (basal-cell	Number	2021	1.6883642	2.25640592	1.20892987
Prevalence	Equatorial	Male	55+ years	Non-melanoma skin cancer (basal-cell	Percent	2021	2.78E-05	3.73E-05	1.95E-05
Prevalence	Equatorial	Female	55+ years	Non-melanoma skin cancer (basal-cell	Percent	2021	1.67E-05	2.32E-05	1.18E-05
Prevalence	Equatorial	Both	55+ years	Non-melanoma skin cancer (basal-cell	Percent	2021	2.14E-05	2.85E-05	1.53E-05
Prevalence	Equatorial	Male	55+ years	Non-melanoma skin cancer (basal-cell	Rate	2021	2.7757555	3.727444542	1.945415181
Prevalence	Equatorial	Female	55+ years	Non-melanoma skin cancer (basal-cell	Rate	2021	1.6665578	2.321693655	1.183135206
Prevalence	Equatorial	Both	55+ years	Non-melanoma skin cancer (basal-cell	Rate	2021	2.1345506	2.852709456	1.528415451
Prevalence	Syrian Ara	Male	55+ years	Non-melanoma skin cancer (basal-cell	Number	1990	16.145542	21.5256598	11.69881688
Prevalence	Syrian Ara	Female	55+ years	Non-melanoma skin cancer (basal-cell	Number	1990	8.3476579	11.50254838	6.015792115
Prevalence	Syrian Ara	Both	55+ years	Non-melanoma skin cancer (basal-cell	Number	1990	24.4932	33.13844891	17.47112454
Prevalence	Syrian Ara	Male	55+ years	Non-melanoma skin cancer (basal-cell	Percent	1990	3.47E-05	4.63E-05	2.51E-05
Prevalence	Syrian Ara	Female	55+ years	Non-melanoma skin cancer (basal-cell	Percent	1990	2.01E-05	2.76E-05	1.45E-05
Prevalence	Syrian Ara	Both	55+ years	Non-melanoma skin cancer (basal-cell	Percent	1990	2.78E-05	3.76E-05	1.98E-05
Prevalence	Syrian Ara	Male	55+ years	Non-melanoma skin cancer (basal-cell	Rate	1990	3.4676844	4.623207741	2.51263196
Prevalence	Syrian Ara	Female	55+ years	Non-melanoma skin cancer (basal-cell	Rate	1990	2.0062943	2.764547561	1.445848596
Prevalence	Syrian Ara	Both	55+ years	Non-melanoma skin cancer (basal-cell	Rate	1990	2.778035	3.758584911	1.981586563
Prevalence	Syrian Ara	Male	55+ years	Non-melanoma skin cancer (basal-cell	Number	2021	41.143372	53.62498852	30.00696672
Prevalence	Syrian Ara	Female	55+ years	Non-melanoma skin cancer (basal-cell	Number	2021	23.01018	31.63495627	16.04019366
Prevalence	Syrian Ara	Both	55+ years	Non-melanoma skin cancer (basal-cell	Number	2021	64.153552	84.87078649	46.59491705
Prevalence	Syrian Ara	Male	55+ years	Non-melanoma skin cancer (basal-cell	Percent	2021	3.40E-05	4.43E-05	2.48E-05
Prevalence	Syrian Ara	Female	55+ years	Non-melanoma skin cancer (basal-cell	Percent	2021	2.00E-05	2.75E-05	1.39E-05
Prevalence	Syrian Ara	Both	55+ years	Non-melanoma skin cancer (basal-cell	Percent	2021	2.72E-05	3.59E-05	1.97E-05

Prevalence	Syrian Ara	Male	55+ years	Non-melanoma skin cancer (basal-cell	Rate	2021	3.3962836	4.426610143	2.47700087
Prevalence	Syrian Ara	Female	55+ years	Non-melanoma skin cancer (basal-cell	Rate	2021	1.9986607	2.747807468	1.393248771
Prevalence	Syrian Ara	Both	55+ years	Non-melanoma skin cancer (basal-cell	Rate	2021	2.7152606	3.592105155	1.972101929
Prevalence	Türkiye	Male	55+ years	Non-melanoma skin cancer (basal-cell	Number	1990	218.33094	270.6540697	175.8005462
Prevalence	Türkiye	Female	55+ years	Non-melanoma skin cancer (basal-cell	Number	1990	217.25849	274.145905	172.9698885
Prevalence	Türkiye	Both	55+ years	Non-melanoma skin cancer (basal-cell	Number	1990	435.58943	545.5552932	352.0049247
Prevalence	Türkiye	Male	55+ years	Non-melanoma skin cancer (basal-cell	Percent	1990	7.67E-05	9.51E-05	6.18E-05
Prevalence	Türkiye	Female	55+ years	Non-melanoma skin cancer (basal-cell	Percent	1990	6.95E-05	8.77E-05	5.53E-05
Prevalence	Türkiye	Both	55+ years	Non-melanoma skin cancer (basal-cell	Percent	1990	7.29E-05	9.14E-05	5.89E-05
Prevalence	Türkiye	Male	55+ years	Non-melanoma skin cancer (basal-cell	Rate	1990	7.6659619	9.50311402	6.17264923
Prevalence	Türkiye	Female	55+ years	Non-melanoma skin cancer (basal-cell	Rate	1990	6.949011	8.768554399	5.532440385
Prevalence	Türkiye	Both	55+ years	Non-melanoma skin cancer (basal-cell	Rate	1990	7.2907817	9.13136148	5.891766151
Prevalence	Türkiye	Male	55+ years	Non-melanoma skin cancer (basal-cell	Number	2021	502.53901	657.1379923	370.8718453
Prevalence	Türkiye	Female	55+ years	Non-melanoma skin cancer (basal-cell	Number	2021	447.55508	581.606278	331.6210362
Prevalence	Türkiye	Both	55+ years	Non-melanoma skin cancer (basal-cell	Number	2021	950.09408	1235.665948	713.3698243
Prevalence	Türkiye	Male	55+ years	Non-melanoma skin cancer (basal-cell	Percent	2021	6.45E-05	8.44E-05	4.76E-05
Prevalence	Türkiye	Female	55+ years	Non-melanoma skin cancer (basal-cell	Percent	2021	5.13E-05	6.67E-05	3.80E-05
Prevalence	Türkiye	Both	55+ years	Non-melanoma skin cancer (basal-cell	Percent	2021	5.75E-05	7.48E-05	4.32E-05
Prevalence	Türkiye	Male	55+ years	Non-melanoma skin cancer (basal-cell	Rate	2021	6.4466847	8.429915697	4.757628423
Prevalence	Türkiye	Female	55+ years	Non-melanoma skin cancer (basal-cell	Rate	2021	5.1290151	6.665251991	3.800402189
Prevalence	Türkiye	Both	55+ years	Non-melanoma skin cancer (basal-cell	Rate	2021	5.750738	7.479249893	4.317891249
Prevalence	Seychelles	Male	55+ years	Non-melanoma skin cancer (basal-cell	Number	1990	0.0266966	0.042160871	0.014265593
Prevalence	Seychelles	Female	55+ years	Non-melanoma skin cancer (basal-cell	Number	1990	0.0336236	0.053784085	0.01703988
Prevalence	Seychelles	Both	55+ years	Non-melanoma skin cancer (basal-cell	Number	1990	0.0603202	0.095322023	0.032086529
Prevalence	Seychelles	Male	55+ years	Non-melanoma skin cancer (basal-cell	Percent	1990	6.38E-06	1.01E-05	3.41E-06
Prevalence	Seychelles	Female	55+ years	Non-melanoma skin cancer (basal-cell	Percent	1990	6.22E-06	9.95E-06	3.15E-06
Prevalence	Seychelles	Both	55+ years	Non-melanoma skin cancer (basal-cell	Percent	1990	6.29E-06	9.94E-06	3.35E-06
Prevalence	Seychelles	Male	55+ years	Non-melanoma skin cancer (basal-cell	Rate	1990	0.6381145	1.007749103	0.340982948
Prevalence	Seychelles	Female	55+ years	Non-melanoma skin cancer (basal-cell	Rate	1990	0.6216971	0.994463051	0.315065908
Prevalence	Seychelles	Both	55+ years	Non-melanoma skin cancer (basal-cell	Rate	1990	0.6288577	0.993763631	0.334512685
Prevalence	Seychelles	Male	55+ years	Non-melanoma skin cancer (basal-cell	Number	2021	0.0636256	0.096955209	0.035609257
Prevalence	Seychelles	Female	55+ years	Non-melanoma skin cancer (basal-cell	Number	2021	0.057796	0.095273812	0.027179116
Prevalence	Seychelles	Both	55+ years	Non-melanoma skin cancer (basal-cell	Number	2021	0.1214216	0.190660975	0.065673784
Prevalence	Seychelles	Male	55+ years	Non-melanoma skin cancer (basal-cell	Percent	2021	6.34E-06	9.67E-06	3.55E-06
Prevalence	Seychelles	Female	55+ years	Non-melanoma skin cancer (basal-cell	Percent	2021	5.40E-06	8.90E-06	2.54E-06
Prevalence	Seychelles	Both	55+ years	Non-melanoma skin cancer (basal-cell	Percent	2021	5.86E-06	9.20E-06	3.17E-06
Prevalence	Seychelles	Male	55+ years	Non-melanoma skin cancer (basal-cell	Rate	2021	0.6339112	0.96597971	0.354780525
Prevalence	Seychelles	Female	55+ years	Non-melanoma skin cancer (basal-cell	Rate	2021	0.5398484	0.889913026	0.253868814
Prevalence	Seychelles	Both	55+ years	Non-melanoma skin cancer (basal-cell	Rate	2021	0.585363	0.919160326	0.316607719
Prevalence	Yemen	Male	55+ years	Non-melanoma skin cancer (basal-cell	Number	1990	11.377306	15.37837207	8.209948858
Prevalence	Yemen	Female	55+ years	Non-melanoma skin cancer (basal-cell	Number	1990	7.6764721	10.58799992	5.447645864
Prevalence	Yemen	Both	55+ years	Non-melanoma skin cancer (basal-cell	Number	1990	19.053778	25.90816408	13.79632405
Prevalence	Yemen	Male	55+ years	Non-melanoma skin cancer (basal-cell	Percent	1990	2.88E-05	3.89E-05	2.08E-05
Prevalence	Yemen	Female	55+ years	Non-melanoma skin cancer (basal-cell	Percent	1990	1.89E-05	2.60E-05	1.34E-05
Prevalence	Yemen	Both	55+ years	Non-melanoma skin cancer (basal-cell	Percent	1990	2.38E-05	3.23E-05	1.72E-05
Prevalence	Yemen	Male	55+ years	Non-melanoma skin cancer (basal-cell	Rate	1990	2.8765938	3.888207736	2.075771514
Prevalence	Yemen	Female	55+ years	Non-melanoma skin cancer (basal-cell	Rate	1990	1.8883965	2.604626466	1.340109814
Prevalence	Yemen	Both	55+ years	Non-melanoma skin cancer (basal-cell	Rate	1990	2.375722	3.230361621	1.720195826
Prevalence	Yemen	Male	55+ years	Non-melanoma skin cancer (basal-cell	Number	2021	34.198703	45.39771131	24.14077567
Prevalence	Yemen	Female	55+ years	Non-melanoma skin cancer (basal-cell	Number	2021	21.240981	28.71413412	15.32610734
Prevalence	Yemen	Both	55+ years	Non-melanoma skin cancer (basal-cell	Number	2021	55.439684	73.59636644	39.61206829
Prevalence	Yemen	Male	55+ years	Non-melanoma skin cancer (basal-cell	Percent	2021	3.12E-05	4.14E-05	2.20E-05
Prevalence	Yemen	Female	55+ years	Non-melanoma skin cancer (basal-cell	Percent	2021	1.84E-05	2.49E-05	1.33E-05
Prevalence	Yemen	Both	55+ years	Non-melanoma skin cancer (basal-cell	Percent	2021	2.47E-05	3.27E-05	1.76E-05
Prevalence	Yemen	Male	55+ years	Non-melanoma skin cancer (basal-cell	Rate	2021	3.1177042	4.138655085	2.200779315
Prevalence	Yemen	Female	55+ years	Non-melanoma skin cancer (basal-cell	Rate	2021	1.8438251	2.49253271	1.330383974

Prevalence	Yemen	Both	55+ years	Non-melanoma skin cancer (basal-cell	Rate	2021	2.465163	3.272512092	1.761377344
Prevalence	Saudi Arab	Male	55+ years	Non-melanoma skin cancer (basal-cell	Number	1990	15.315126	20.72254797	11.02154548
Prevalence	Saudi Arab	Female	55+ years	Non-melanoma skin cancer (basal-cell	Number	1990	7.1245706	9.870590866	5.125710423
Prevalence	Saudi Arab	Both	55+ years	Non-melanoma skin cancer (basal-cell	Number	1990	22.439696	30.18522527	16.28225907
Prevalence	Saudi Arab	Male	55+ years	Non-melanoma skin cancer (basal-cell	Percent	1990	2.94E-05	3.97E-05	2.11E-05
Prevalence	Saudi Arab	Female	55+ years	Non-melanoma skin cancer (basal-cell	Percent	1990	1.93E-05	2.67E-05	1.39E-05
Prevalence	Saudi Arab	Both	55+ years	Non-melanoma skin cancer (basal-cell	Percent	1990	2.52E-05	3.39E-05	1.83E-05
Prevalence	Saudi Arab	Male	55+ years	Non-melanoma skin cancer (basal-cell	Rate	1990	2.935128	3.971454893	2.112267797
Prevalence	Saudi Arab	Female	55+ years	Non-melanoma skin cancer (basal-cell	Rate	1990	1.9301681	2.674112062	1.388642712
Prevalence	Saudi Arab	Both	55+ years	Non-melanoma skin cancer (basal-cell	Rate	1990	2.5187561	3.388157296	1.82761117
Prevalence	Saudi Arab	Male	55+ years	Non-melanoma skin cancer (basal-cell	Number	2021	44.779211	56.51908042	35.41313118
Prevalence	Saudi Arab	Female	55+ years	Non-melanoma skin cancer (basal-cell	Number	2021	18.917984	23.90825224	15.29024133
Prevalence	Saudi Arab	Both	55+ years	Non-melanoma skin cancer (basal-cell	Number	2021	63.697195	79.25467442	51.09593347
Prevalence	Saudi Arab	Male	55+ years	Non-melanoma skin cancer (basal-cell	Percent	2021	2.48E-05	3.13E-05	1.96E-05
Prevalence	Saudi Arab	Female	55+ years	Non-melanoma skin cancer (basal-cell	Percent	2021	1.57E-05	1.98E-05	1.27E-05
Prevalence	Saudi Arab	Both	55+ years	Non-melanoma skin cancer (basal-cell	Percent	2021	2.12E-05	2.63E-05	1.70E-05
Prevalence	Saudi Arab	Male	55+ years	Non-melanoma skin cancer (basal-cell	Rate	2021	2.480791	3.131185689	1.961905409
Prevalence	Saudi Arab	Female	55+ years	Non-melanoma skin cancer (basal-cell	Rate	2021	1.5690917	1.982993495	1.268200151
Prevalence	Saudi Arab	Both	55+ years	Non-melanoma skin cancer (basal-cell	Rate	2021	2.1156922	2.632431432	1.697143321
Prevalence	Bangladesh	Male	55+ years	Non-melanoma skin cancer (basal-cell	Number	1990	1.1424116	2.619912942	0.297999711
Prevalence	Bangladesh	Female	55+ years	Non-melanoma skin cancer (basal-cell	Number	1990	0.6337966	1.427397515	0.180771862
Prevalence	Bangladesh	Both	55+ years	Non-melanoma skin cancer (basal-cell	Number	1990	1.7762082	3.881070352	0.633975319
Prevalence	Bangladesh	Male	55+ years	Non-melanoma skin cancer (basal-cell	Percent	1990	2.71E-07	6.22E-07	7.08E-08
Prevalence	Bangladesh	Female	55+ years	Non-melanoma skin cancer (basal-cell	Percent	1990	1.89E-07	4.25E-07	5.38E-08
Prevalence	Bangladesh	Both	55+ years	Non-melanoma skin cancer (basal-cell	Percent	1990	2.35E-07	5.13E-07	8.37E-08
Prevalence	Bangladesh	Male	55+ years	Non-melanoma skin cancer (basal-cell	Rate	1990	0.0271158	0.062185044	0.007073184
Prevalence	Bangladesh	Female	55+ years	Non-melanoma skin cancer (basal-cell	Rate	1990	0.0188542	0.042462231	0.005377603
Prevalence	Bangladesh	Both	55+ years	Non-melanoma skin cancer (basal-cell	Rate	1990	0.0234493	0.051237542	0.008369685
Prevalence	Bangladesh	Male	55+ years	Non-melanoma skin cancer (basal-cell	Number	2021	3.2837023	7.718198021	0.765893046
Prevalence	Bangladesh	Female	55+ years	Non-melanoma skin cancer (basal-cell	Number	2021	2.1070755	4.974819578	0.608211753
Prevalence	Bangladesh	Both	55+ years	Non-melanoma skin cancer (basal-cell	Number	2021	5.3907778	12.10083934	1.885874355
Prevalence	Bangladesh	Male	55+ years	Non-melanoma skin cancer (basal-cell	Percent	2021	2.72E-07	6.39E-07	6.34E-08
Prevalence	Bangladesh	Female	55+ years	Non-melanoma skin cancer (basal-cell	Percent	2021	1.87E-07	4.41E-07	5.39E-08
Prevalence	Bangladesh	Both	55+ years	Non-melanoma skin cancer (basal-cell	Percent	2021	2.31E-07	5.18E-07	8.08E-08
Prevalence	Bangladesh	Male	55+ years	Non-melanoma skin cancer (basal-cell	Rate	2021	0.0271748	0.063873186	0.006338271
Prevalence	Bangladesh	Female	55+ years	Non-melanoma skin cancer (basal-cell	Rate	2021	0.0186756	0.044093162	0.005390744
Prevalence	Bangladesh	Both	55+ years	Non-melanoma skin cancer (basal-cell	Rate	2021	0.0230709	0.05178791	0.008070968
Prevalence	United Ara	Male	55+ years	Non-melanoma skin cancer (basal-cell	Number	1990	0.9537152	1.304384005	0.683083559
Prevalence	United Ara	Female	55+ years	Non-melanoma skin cancer (basal-cell	Number	1990	0.3730623	0.508539158	0.264447741
Prevalence	United Ara	Both	55+ years	Non-melanoma skin cancer (basal-cell	Number	1990	1.3267775	1.802327483	0.946811389
Prevalence	United Ara	Male	55+ years	Non-melanoma skin cancer (basal-cell	Percent	1990	2.74E-05	3.75E-05	1.97E-05
Prevalence	United Ara	Female	55+ years	Non-melanoma skin cancer (basal-cell	Percent	1990	1.75E-05	2.38E-05	1.24E-05
Prevalence	United Ara	Both	55+ years	Non-melanoma skin cancer (basal-cell	Percent	1990	2.36E-05	3.21E-05	1.69E-05
Prevalence	United Ara	Male	55+ years	Non-melanoma skin cancer (basal-cell	Rate	1990	2.7432806	3.751949604	1.964831737
Prevalence	United Ara	Female	55+ years	Non-melanoma skin cancer (basal-cell	Rate	1990	1.746532	2.380781689	1.238041023
Prevalence	United Ara	Both	55+ years	Non-melanoma skin cancer (basal-cell	Rate	1990	2.3639404	3.21123529	1.686948778
Prevalence	United Ara	Male	55+ years	Non-melanoma skin cancer (basal-cell	Number	2021	12.862452	18.60316966	8.984632122
Prevalence	United Ara	Female	55+ years	Non-melanoma skin cancer (basal-cell	Number	2021	2.2650688	3.410052745	1.478170116
Prevalence	United Ara	Both	55+ years	Non-melanoma skin cancer (basal-cell	Number	2021	15.127521	21.9217685	10.49966055
Prevalence	United Ara	Male	55+ years	Non-melanoma skin cancer (basal-cell	Percent	2021	2.34E-05	3.38E-05	1.63E-05
Prevalence	United Ara	Female	55+ years	Non-melanoma skin cancer (basal-cell	Percent	2021	1.35E-05	2.03E-05	8.82E-06
Prevalence	United Ara	Both	55+ years	Non-melanoma skin cancer (basal-cell	Percent	2021	2.11E-05	3.05E-05	1.46E-05
Prevalence	United Ara	Male	55+ years	Non-melanoma skin cancer (basal-cell	Rate	2021	2.3368526	3.379827213	1.632329582
Prevalence	United Ara	Female	55+ years	Non-melanoma skin cancer (basal-cell	Rate	2021	1.3509809	2.033896809	0.88164193
Prevalence	United Ara	Both	55+ years	Non-melanoma skin cancer (basal-cell	Rate	2021	2.1066658	3.052835958	1.462187747
Prevalence	Central Af	Male	55+ years	Non-melanoma skin cancer (basal-cell	Number	1990	2.0891779	2.955992241	1.449676974

Prevalence	Central Af	Female	55+ years	Non-melanoma skin cancer (basal-cell	Number	1990	1.4437519	1.999157746	0.982086592
Prevalence	Central Af	Both	55+ years	Non-melanoma skin cancer (basal-cell	Number	1990	3.5329298	4.935525931	2.497795244
Prevalence	Central Af	Male	55+ years	Non-melanoma skin cancer (basal-cell	Percent	1990	2.37E-05	3.35E-05	1.64E-05
Prevalence	Central Af	Female	55+ years	Non-melanoma skin cancer (basal-cell	Percent	1990	1.43E-05	1.99E-05	9.76E-06
Prevalence	Central Af	Both	55+ years	Non-melanoma skin cancer (basal-cell	Percent	1990	1.87E-05	2.61E-05	1.32E-05
Prevalence	Central Af	Male	55+ years	Non-melanoma skin cancer (basal-cell	Rate	1990	2.3691911	3.352184765	1.643977612
Prevalence	Central Af	Female	55+ years	Non-melanoma skin cancer (basal-cell	Rate	1990	1.4346909	1.986611014	0.975923007
Prevalence	Central Af	Both	55+ years	Non-melanoma skin cancer (basal-cell	Rate	1990	1.87113	2.613980818	1.322896272
Prevalence	Central Af	Male	55+ years	Non-melanoma skin cancer (basal-cell	Number	2021	3.6593062	5.018955521	2.597947325
Prevalence	Central Af	Female	55+ years	Non-melanoma skin cancer (basal-cell	Number	2021	2.777309	3.893325617	1.926555802
Prevalence	Central Af	Both	55+ years	Non-melanoma skin cancer (basal-cell	Number	2021	6.4366153	8.977844409	4.587690859
Prevalence	Central Af	Male	55+ years	Non-melanoma skin cancer (basal-cell	Percent	2021	2.26E-05	3.10E-05	1.60E-05
Prevalence	Central Af	Female	55+ years	Non-melanoma skin cancer (basal-cell	Percent	2021	1.42E-05	1.99E-05	9.85E-06
Prevalence	Central Af	Both	55+ years	Non-melanoma skin cancer (basal-cell	Percent	2021	1.80E-05	2.51E-05	1.28E-05
Prevalence	Central Af	Male	55+ years	Non-melanoma skin cancer (basal-cell	Rate	2021	2.2582662	3.097346089	1.603270233
Prevalence	Central Af	Female	55+ years	Non-melanoma skin cancer (basal-cell	Rate	2021	1.4194709	1.989862269	0.984654528
Prevalence	Central Af	Both	55+ years	Non-melanoma skin cancer (basal-cell	Rate	2021	1.7994524	2.509891131	1.282557825
Prevalence	Nepal	Male	55+ years	Non-melanoma skin cancer (basal-cell	Number	1990	0.1922556	0.445797575	0.047165478
Prevalence	Nepal	Female	55+ years	Non-melanoma skin cancer (basal-cell	Number	1990	0.1287643	0.308987895	0.03645481
Prevalence	Nepal	Both	55+ years	Non-melanoma skin cancer (basal-cell	Number	1990	0.3210199	0.718731458	0.108472443
Prevalence	Nepal	Male	55+ years	Non-melanoma skin cancer (basal-cell	Percent	1990	2.41E-07	5.59E-07	5.91E-08
Prevalence	Nepal	Female	55+ years	Non-melanoma skin cancer (basal-cell	Percent	1990	1.74E-07	4.17E-07	4.92E-08
Prevalence	Nepal	Both	55+ years	Non-melanoma skin cancer (basal-cell	Percent	1990	2.09E-07	4.67E-07	7.05E-08
Prevalence	Nepal	Male	55+ years	Non-melanoma skin cancer (basal-cell	Rate	1990	0.0241037	0.055891131	0.005913294
Prevalence	Nepal	Female	55+ years	Non-melanoma skin cancer (basal-cell	Rate	1990	0.0173607	0.041659302	0.004915021
Prevalence	Nepal	Both	55+ years	Non-melanoma skin cancer (basal-cell	Rate	1990	0.0208547	0.046691497	0.007046778
Prevalence	Nepal	Male	55+ years	Non-melanoma skin cancer (basal-cell	Number	2021	0.4654639	1.076885739	0.109944282
Prevalence	Nepal	Female	55+ years	Non-melanoma skin cancer (basal-cell	Number	2021	0.3711612	0.863180045	0.111780645
Prevalence	Nepal	Both	55+ years	Non-melanoma skin cancer (basal-cell	Number	2021	0.8366251	1.86047804	0.294332943
Prevalence	Nepal	Male	55+ years	Non-melanoma skin cancer (basal-cell	Percent	2021	2.47E-07	5.72E-07	5.84E-08
Prevalence	Nepal	Female	55+ years	Non-melanoma skin cancer (basal-cell	Percent	2021	1.79E-07	4.16E-07	5.39E-08
Prevalence	Nepal	Both	55+ years	Non-melanoma skin cancer (basal-cell	Percent	2021	2.11E-07	4.70E-07	7.44E-08
Prevalence	Nepal	Male	55+ years	Non-melanoma skin cancer (basal-cell	Rate	2021	0.0247229	0.05719839	0.00583965
Prevalence	Nepal	Female	55+ years	Non-melanoma skin cancer (basal-cell	Rate	2021	0.0178861	0.041596324	0.005386668
Prevalence	Nepal	Both	55+ years	Non-melanoma skin cancer (basal-cell	Rate	2021	0.0211383	0.047007222	0.007436677
Prevalence	India	Male	55+ years	Non-melanoma skin cancer (basal-cell	Number	1990	285.97151	411.3372214	200.286421
Prevalence	India	Female	55+ years	Non-melanoma skin cancer (basal-cell	Number	1990	237.16061	347.099595	163.4077589
Prevalence	India	Both	55+ years	Non-melanoma skin cancer (basal-cell	Number	1990	523.13212	762.0617127	364.0269672
Prevalence	India	Male	55+ years	Non-melanoma skin cancer (basal-cell	Percent	1990	7.20E-06	1.04E-05	5.04E-06
Prevalence	India	Female	55+ years	Non-melanoma skin cancer (basal-cell	Percent	1990	6.42E-06	9.40E-06	4.42E-06
Prevalence	India	Both	55+ years	Non-melanoma skin cancer (basal-cell	Percent	1990	6.82E-06	9.94E-06	4.75E-06
Prevalence	India	Male	55+ years	Non-melanoma skin cancer (basal-cell	Rate	1990	0.7197157	1.035228463	0.504068664
Prevalence	India	Female	55+ years	Non-melanoma skin cancer (basal-cell	Rate	1990	0.6420996	0.939753502	0.442417727
Prevalence	India	Both	55+ years	Non-melanoma skin cancer (basal-cell	Rate	1990	0.6823243	0.993961597	0.474802526
Prevalence	India	Male	55+ years	Non-melanoma skin cancer (basal-cell	Number	2021	777.16123	1089.888823	552.9852165
Prevalence	India	Female	55+ years	Non-melanoma skin cancer (basal-cell	Number	2021	740.96741	1044.221371	515.0860706
Prevalence	India	Both	55+ years	Non-melanoma skin cancer (basal-cell	Number	2021	1518.1286	2137.007877	1062.039299
Prevalence	India	Male	55+ years	Non-melanoma skin cancer (basal-cell	Percent	2021	8.00E-06	1.12E-05	5.69E-06
Prevalence	India	Female	55+ years	Non-melanoma skin cancer (basal-cell	Percent	2021	7.13E-06	1.01E-05	4.96E-06
Prevalence	India	Both	55+ years	Non-melanoma skin cancer (basal-cell	Percent	2021	7.55E-06	1.06E-05	5.28E-06
Prevalence	India	Male	55+ years	Non-melanoma skin cancer (basal-cell	Rate	2021	0.7999548	1.121854411	0.569203841
Prevalence	India	Female	55+ years	Non-melanoma skin cancer (basal-cell	Rate	2021	0.7131906	1.005076444	0.49577694
Prevalence	India	Both	55+ years	Non-melanoma skin cancer (basal-cell	Rate	2021	0.7551174	1.062948075	0.528258525
Prevalence	Pakistan	Male	55+ years	Non-melanoma skin cancer (basal-cell	Number	1990	7.5701326	10.92233638	4.999141539
Prevalence	Pakistan	Female	55+ years	Non-melanoma skin cancer (basal-cell	Number	1990	4.9496473	7.534437499	3.116425534
Prevalence	Pakistan	Both	55+ years	Non-melanoma skin cancer (basal-cell	Number	1990	12.51978	18.43819278	8.106786985

Prevalence	Pakistan	Male	55+ years	Non-melanoma skin cancer (basal-cell	Percent	1990	1.47E-06	2.13E-06	9.74E-07
Prevalence	Pakistan	Female	55+ years	Non-melanoma skin cancer (basal-cell	Percent	1990	1.24E-06	1.89E-06	7.82E-07
Prevalence	Pakistan	Both	55+ years	Non-melanoma skin cancer (basal-cell	Percent	1990	1.37E-06	2.02E-06	8.89E-07
Prevalence	Pakistan	Male	55+ years	Non-melanoma skin cancer (basal-cell	Rate	1990	0.1474271	0.212710651	0.097357435
Prevalence	Pakistan	Female	55+ years	Non-melanoma skin cancer (basal-cell	Rate	1990	0.1242182	0.189087078	0.078210988
Prevalence	Pakistan	Both	55+ years	Non-melanoma skin cancer (basal-cell	Rate	1990	0.1372862	0.202184875	0.088895356
Prevalence	Pakistan	Male	55+ years	Non-melanoma skin cancer (basal-cell	Number	2021	11.445083	17.35463105	7.373041704
Prevalence	Pakistan	Female	55+ years	Non-melanoma skin cancer (basal-cell	Number	2021	8.8127673	13.18849701	5.73802542
Prevalence	Pakistan	Both	55+ years	Non-melanoma skin cancer (basal-cell	Number	2021	20.257851	29.93713342	13.27057837
Prevalence	Pakistan	Male	55+ years	Non-melanoma skin cancer (basal-cell	Percent	2021	1.09E-06	1.65E-06	7.02E-07
Prevalence	Pakistan	Female	55+ years	Non-melanoma skin cancer (basal-cell	Percent	2021	9.47E-07	1.42E-06	6.16E-07
Prevalence	Pakistan	Both	55+ years	Non-melanoma skin cancer (basal-cell	Percent	2021	1.02E-06	1.51E-06	6.70E-07
Prevalence	Pakistan	Male	55+ years	Non-melanoma skin cancer (basal-cell	Rate	2021	0.108833	0.165027746	0.070111341
Prevalence	Pakistan	Female	55+ years	Non-melanoma skin cancer (basal-cell	Rate	2021	0.0946611	0.141662389	0.061634194
Prevalence	Pakistan	Both	55+ years	Non-melanoma skin cancer (basal-cell	Rate	2021	0.1021782	0.150999375	0.066935234
Prevalence	Burundi	Male	55+ years	Non-melanoma skin cancer (basal-cell	Number	1990	3.9633892	5.328338181	2.940020767
Prevalence	Burundi	Female	55+ years	Non-melanoma skin cancer (basal-cell	Number	1990	2.9179914	4.032892154	2.026773407
Prevalence	Burundi	Both	55+ years	Non-melanoma skin cancer (basal-cell	Number	1990	6.8813807	9.244441936	4.997940342
Prevalence	Burundi	Male	55+ years	Non-melanoma skin cancer (basal-cell	Percent	1990	2.33E-05	3.13E-05	1.73E-05
Prevalence	Burundi	Female	55+ years	Non-melanoma skin cancer (basal-cell	Percent	1990	1.42E-05	1.96E-05	9.83E-06
Prevalence	Burundi	Both	55+ years	Non-melanoma skin cancer (basal-cell	Percent	1990	1.83E-05	2.46E-05	1.33E-05
Prevalence	Burundi	Male	55+ years	Non-melanoma skin cancer (basal-cell	Rate	1990	2.3282341	3.130053059	1.727071496
Prevalence	Burundi	Female	55+ years	Non-melanoma skin cancer (basal-cell	Rate	1990	1.4157023	1.956611191	0.983316037
Prevalence	Burundi	Both	55+ years	Non-melanoma skin cancer (basal-cell	Rate	1990	1.8284634	2.456356461	1.328011267
Prevalence	Burundi	Male	55+ years	Non-melanoma skin cancer (basal-cell	Number	2021	8.9956011	12.40508191	6.347863729
Prevalence	Burundi	Female	55+ years	Non-melanoma skin cancer (basal-cell	Number	2021	5.1432073	7.086373516	3.587574744
Prevalence	Burundi	Both	55+ years	Non-melanoma skin cancer (basal-cell	Number	2021	14.138808	19.25893366	9.936738421
Prevalence	Burundi	Male	55+ years	Non-melanoma skin cancer (basal-cell	Percent	2021	2.13E-05	2.94E-05	1.50E-05
Prevalence	Burundi	Female	55+ years	Non-melanoma skin cancer (basal-cell	Percent	2021	1.36E-05	1.88E-05	9.50E-06
Prevalence	Burundi	Both	55+ years	Non-melanoma skin cancer (basal-cell	Percent	2021	1.77E-05	2.41E-05	1.24E-05
Prevalence	Burundi	Male	55+ years	Non-melanoma skin cancer (basal-cell	Rate	2021	2.1278541	2.934345848	1.501548133
Prevalence	Burundi	Female	55+ years	Non-melanoma skin cancer (basal-cell	Rate	2021	1.361452	1.875825124	0.949662451
Prevalence	Burundi	Both	55+ years	Non-melanoma skin cancer (basal-cell	Rate	2021	1.7661848	2.40577808	1.241272643
Prevalence	Rwanda	Male	55+ years	Non-melanoma skin cancer (basal-cell	Number	1990	4.6094462	6.150209249	3.297991545
Prevalence	Rwanda	Female	55+ years	Non-melanoma skin cancer (basal-cell	Number	1990	3.6241663	4.934276012	2.520653274
Prevalence	Rwanda	Both	55+ years	Non-melanoma skin cancer (basal-cell	Number	1990	8.2336125	10.98816094	5.880182917
Prevalence	Rwanda	Male	55+ years	Non-melanoma skin cancer (basal-cell	Percent	1990	2.22E-05	2.96E-05	1.59E-05
Prevalence	Rwanda	Female	55+ years	Non-melanoma skin cancer (basal-cell	Percent	1990	1.39E-05	1.90E-05	9.69E-06
Prevalence	Rwanda	Both	55+ years	Non-melanoma skin cancer (basal-cell	Percent	1990	1.76E-05	2.35E-05	1.26E-05
Prevalence	Rwanda	Male	55+ years	Non-melanoma skin cancer (basal-cell	Rate	1990	2.2185136	2.960078551	1.587314128
Prevalence	Rwanda	Female	55+ years	Non-melanoma skin cancer (basal-cell	Rate	1990	1.3929626	1.896508467	0.968823038
Prevalence	Rwanda	Both	55+ years	Non-melanoma skin cancer (basal-cell	Rate	1990	1.7595119	2.348155146	1.256587144
Prevalence	Rwanda	Male	55+ years	Non-melanoma skin cancer (basal-cell	Number	2021	9.6047795	13.58468692	6.880484396
Prevalence	Rwanda	Female	55+ years	Non-melanoma skin cancer (basal-cell	Number	2021	8.5610184	11.63815754	5.921619984
Prevalence	Rwanda	Both	55+ years	Non-melanoma skin cancer (basal-cell	Number	2021	18.165798	25.28418189	12.90150727
Prevalence	Rwanda	Male	55+ years	Non-melanoma skin cancer (basal-cell	Percent	2021	2.15E-05	3.04E-05	1.54E-05
Prevalence	Rwanda	Female	55+ years	Non-melanoma skin cancer (basal-cell	Percent	2021	1.40E-05	1.91E-05	9.70E-06
Prevalence	Rwanda	Both	55+ years	Non-melanoma skin cancer (basal-cell	Percent	2021	1.72E-05	2.39E-05	1.22E-05
Prevalence	Rwanda	Male	55+ years	Non-melanoma skin cancer (basal-cell	Rate	2021	2.1500492	3.040959547	1.5402103
Prevalence	Rwanda	Female	55+ years	Non-melanoma skin cancer (basal-cell	Rate	2021	1.4017397	1.905575501	0.969577352
Prevalence	Rwanda	Both	55+ years	Non-melanoma skin cancer (basal-cell	Rate	2021	1.717861	2.391015841	1.220039802
Prevalence	Gabon	Male	55+ years	Non-melanoma skin cancer (basal-cell	Number	1990	1.2735113	1.721434575	0.926835942
Prevalence	Gabon	Female	55+ years	Non-melanoma skin cancer (basal-cell	Number	1990	0.976516	1.347996611	0.695389341
Prevalence	Gabon	Both	55+ years	Non-melanoma skin cancer (basal-cell	Number	1990	2.2500273	3.0322531	1.645828861
Prevalence	Gabon	Male	55+ years	Non-melanoma skin cancer (basal-cell	Percent	1990	2.92E-05	3.94E-05	2.12E-05
Prevalence	Gabon	Female	55+ years	Non-melanoma skin cancer (basal-cell	Percent	1990	1.80E-05	2.48E-05	1.28E-05

Prevalence	Gabon	Both	55+ years	Non-melanoma skin cancer (basal-cell	Percent	1990	2.30E-05	3.10E-05	1.68E-05
Prevalence	Gabon	Male	55+ years	Non-melanoma skin cancer (basal-cell	Rate	1990	2.9176976	3.943919137	2.123441728
Prevalence	Gabon	Female	55+ years	Non-melanoma skin cancer (basal-cell	Rate	1990	1.7999069	2.484617166	1.281736378
Prevalence	Gabon	Both	55+ years	Non-melanoma skin cancer (basal-cell	Rate	1990	2.2982559	3.097248524	1.681106703
Prevalence	Gabon	Male	55+ years	Non-melanoma skin cancer (basal-cell	Number	2021	2.3053356	3.127925325	1.682991649
Prevalence	Gabon	Female	55+ years	Non-melanoma skin cancer (basal-cell	Number	2021	1.5984441	2.176948266	1.127464373
Prevalence	Gabon	Both	55+ years	Non-melanoma skin cancer (basal-cell	Number	2021	3.9037798	5.288856729	2.840701025
Prevalence	Gabon	Male	55+ years	Non-melanoma skin cancer (basal-cell	Percent	2021	2.65E-05	3.60E-05	1.94E-05
Prevalence	Gabon	Female	55+ years	Non-melanoma skin cancer (basal-cell	Percent	2021	1.74E-05	2.37E-05	1.23E-05
Prevalence	Gabon	Both	55+ years	Non-melanoma skin cancer (basal-cell	Percent	2021	2.18E-05	2.96E-05	1.59E-05
Prevalence	Gabon	Male	55+ years	Non-melanoma skin cancer (basal-cell	Rate	2021	2.6523385	3.598745756	1.936318302
Prevalence	Gabon	Female	55+ years	Non-melanoma skin cancer (basal-cell	Rate	2021	1.7405632	2.370502658	1.227708226
Prevalence	Gabon	Both	55+ years	Non-melanoma skin cancer (basal-cell	Rate	2021	2.1839086	2.958768253	1.5891858
Prevalence	Kenya	Male	55+ years	Non-melanoma skin cancer (basal-cell	Number	1990	13.690304	18.56175003	10.04527084
Prevalence	Kenya	Female	55+ years	Non-melanoma skin cancer (basal-cell	Number	1990	12.094227	16.32469283	8.693905244
Prevalence	Kenya	Both	55+ years	Non-melanoma skin cancer (basal-cell	Number	1990	25.784532	34.72592898	19.00724838
Prevalence	Kenya	Male	55+ years	Non-melanoma skin cancer (basal-cell	Percent	1990	2.10E-05	2.85E-05	1.54E-05
Prevalence	Kenya	Female	55+ years	Non-melanoma skin cancer (basal-cell	Percent	1990	1.78E-05	2.40E-05	1.28E-05
Prevalence	Kenya	Both	55+ years	Non-melanoma skin cancer (basal-cell	Percent	1990	1.93E-05	2.61E-05	1.43E-05
Prevalence	Kenya	Male	55+ years	Non-melanoma skin cancer (basal-cell	Rate	1990	2.1001245	2.847415565	1.540967877
Prevalence	Kenya	Female	55+ years	Non-melanoma skin cancer (basal-cell	Rate	1990	1.7762398	2.397554528	1.27684558
Prevalence	Kenya	Both	55+ years	Non-melanoma skin cancer (basal-cell	Rate	1990	1.9346574	2.60554564	1.426146243
Prevalence	Kenya	Male	55+ years	Non-melanoma skin cancer (basal-cell	Number	2021	32.657077	44.81639919	24.25559552
Prevalence	Kenya	Female	55+ years	Non-melanoma skin cancer (basal-cell	Number	2021	32.474761	43.98560101	23.388888
Prevalence	Kenya	Both	55+ years	Non-melanoma skin cancer (basal-cell	Number	2021	65.131838	88.34038547	47.81723922
Prevalence	Kenya	Male	55+ years	Non-melanoma skin cancer (basal-cell	Percent	2021	1.87E-05	2.56E-05	1.39E-05
Prevalence	Kenya	Female	55+ years	Non-melanoma skin cancer (basal-cell	Percent	2021	1.63E-05	2.21E-05	1.17E-05
Prevalence	Kenya	Both	55+ years	Non-melanoma skin cancer (basal-cell	Percent	2021	1.74E-05	2.36E-05	1.28E-05
Prevalence	Kenya	Male	55+ years	Non-melanoma skin cancer (basal-cell	Rate	2021	1.867055	2.562221991	1.386729442
Prevalence	Kenya	Female	55+ years	Non-melanoma skin cancer (basal-cell	Rate	2021	1.6305456	2.20850053	1.174347295
Prevalence	Kenya	Both	55+ years	Non-melanoma skin cancer (basal-cell	Rate	2021	1.7411334	2.361554659	1.278271806
Prevalence	Malawi	Male	55+ years	Non-melanoma skin cancer (basal-cell	Number	1990	5.983228	8.231933023	4.146443301
Prevalence	Malawi	Female	55+ years	Non-melanoma skin cancer (basal-cell	Number	1990	2.9962629	4.345718073	2.058081118
Prevalence	Malawi	Both	55+ years	Non-melanoma skin cancer (basal-cell	Number	1990	8.979491	12.30518997	6.342071518
Prevalence	Malawi	Male	55+ years	Non-melanoma skin cancer (basal-cell	Percent	1990	2.04E-05	2.80E-05	1.41E-05
Prevalence	Malawi	Female	55+ years	Non-melanoma skin cancer (basal-cell	Percent	1990	8.94E-06	1.30E-05	6.14E-06
Prevalence	Malawi	Both	55+ years	Non-melanoma skin cancer (basal-cell	Percent	1990	1.43E-05	1.96E-05	1.01E-05
Prevalence	Malawi	Male	55+ years	Non-melanoma skin cancer (basal-cell	Rate	1990	2.0363152	2.801633252	1.411189012
Prevalence	Malawi	Female	55+ years	Non-melanoma skin cancer (basal-cell	Rate	1990	0.8939989	1.296637674	0.614072397
Prevalence	Malawi	Both	55+ years	Non-melanoma skin cancer (basal-cell	Rate	1990	1.4276295	1.956375127	1.008312021
Prevalence	Malawi	Male	55+ years	Non-melanoma skin cancer (basal-cell	Number	2021	10.690834	15.13465664	7.620302013
Prevalence	Malawi	Female	55+ years	Non-melanoma skin cancer (basal-cell	Number	2021	6.0538587	8.662010813	4.155750764
Prevalence	Malawi	Both	55+ years	Non-melanoma skin cancer (basal-cell	Number	2021	16.744693	23.47559491	11.89113632
Prevalence	Malawi	Male	55+ years	Non-melanoma skin cancer (basal-cell	Percent	2021	2.06E-05	2.92E-05	1.47E-05
Prevalence	Malawi	Female	55+ years	Non-melanoma skin cancer (basal-cell	Percent	2021	9.30E-06	1.33E-05	6.39E-06
Prevalence	Malawi	Both	55+ years	Non-melanoma skin cancer (basal-cell	Percent	2021	1.43E-05	2.01E-05	1.02E-05
Prevalence	Malawi	Male	55+ years	Non-melanoma skin cancer (basal-cell	Rate	2021	2.0621705	2.919345812	1.469891078
Prevalence	Malawi	Female	55+ years	Non-melanoma skin cancer (basal-cell	Rate	2021	0.9303586	1.33118016	0.638656899
Prevalence	Malawi	Both	55+ years	Non-melanoma skin cancer (basal-cell	Rate	2021	1.4322379	2.007957767	1.017094545
Prevalence	United Rep	Male	55+ years	Non-melanoma skin cancer (basal-cell	Number	1990	21.084274	28.70448061	14.98385298
Prevalence	United Rep	Female	55+ years	Non-melanoma skin cancer (basal-cell	Number	1990	13.485399	18.68528297	9.428800183
Prevalence	United Rep	Both	55+ years	Non-melanoma skin cancer (basal-cell	Number	1990	34.569673	46.65142776	24.61291285
Prevalence	United Rep	Male	55+ years	Non-melanoma skin cancer (basal-cell	Percent	1990	2.37E-05	3.23E-05	1.68E-05
Prevalence	United Rep	Female	55+ years	Non-melanoma skin cancer (basal-cell	Percent	1990	1.47E-05	2.04E-05	1.03E-05
Prevalence	United Rep	Both	55+ years	Non-melanoma skin cancer (basal-cell	Percent	1990	1.92E-05	2.59E-05	1.36E-05
Prevalence	United Rep	Male	55+ years	Non-melanoma skin cancer (basal-cell	Rate	1990	2.369634	3.226059084	1.684015665

Prevalence	United Rep	Female	55+ years	Non-melanoma skin cancer (basal-cell	Rate	1990	1.4747764	2.043440847	1.031142823
Prevalence	United Rep	Both	55+ years	Non-melanoma skin cancer (basal-cell	Rate	1990	1.9160961	2.585752578	1.364221973
Prevalence	United Rep	Male	55+ years	Non-melanoma skin cancer (basal-cell	Number	2021	46.847273	62.55423364	33.45593051
Prevalence	United Rep	Female	55+ years	Non-melanoma skin cancer (basal-cell	Number	2021	31.408735	42.53084295	22.18467634
Prevalence	United Rep	Both	55+ years	Non-melanoma skin cancer (basal-cell	Number	2021	78.256007	104.3722517	55.39983423
Prevalence	United Rep	Male	55+ years	Non-melanoma skin cancer (basal-cell	Percent	2021	2.34E-05	3.13E-05	1.67E-05
Prevalence	United Rep	Female	55+ years	Non-melanoma skin cancer (basal-cell	Percent	2021	1.48E-05	2.00E-05	1.05E-05
Prevalence	United Rep	Both	55+ years	Non-melanoma skin cancer (basal-cell	Percent	2021	1.90E-05	2.53E-05	1.34E-05
Prevalence	United Rep	Male	55+ years	Non-melanoma skin cancer (basal-cell	Rate	2021	2.3427571	3.128237052	1.673077509
Prevalence	United Rep	Female	55+ years	Non-melanoma skin cancer (basal-cell	Rate	2021	1.4797871	2.003792635	1.045205973
Prevalence	United Rep	Both	55+ years	Non-melanoma skin cancer (basal-cell	Rate	2021	1.8984126	2.531966557	1.343944633
Prevalence	Djibouti	Male	55+ years	Non-melanoma skin cancer (basal-cell	Number	1990	0.2406041	0.338775937	0.16985791
Prevalence	Djibouti	Female	55+ years	Non-melanoma skin cancer (basal-cell	Number	1990	0.1510428	0.210850651	0.105252399
Prevalence	Djibouti	Both	55+ years	Non-melanoma skin cancer (basal-cell	Number	1990	0.3916468	0.550467522	0.278290646
Prevalence	Djibouti	Male	55+ years	Non-melanoma skin cancer (basal-cell	Percent	1990	2.17E-05	3.05E-05	1.53E-05
Prevalence	Djibouti	Female	55+ years	Non-melanoma skin cancer (basal-cell	Percent	1990	1.46E-05	2.04E-05	1.02E-05
Prevalence	Djibouti	Both	55+ years	Non-melanoma skin cancer (basal-cell	Percent	1990	1.83E-05	2.57E-05	1.30E-05
Prevalence	Djibouti	Male	55+ years	Non-melanoma skin cancer (basal-cell	Rate	1990	2.1677963	3.052305976	1.530387069
Prevalence	Djibouti	Female	55+ years	Non-melanoma skin cancer (basal-cell	Rate	1990	1.458635	2.036205778	1.01643292
Prevalence	Djibouti	Both	55+ years	Non-melanoma skin cancer (basal-cell	Rate	1990	1.8255111	2.565792677	1.29714483
Prevalence	Djibouti	Male	55+ years	Non-melanoma skin cancer (basal-cell	Number	2021	1.2492468	1.71448311	0.873852951
Prevalence	Djibouti	Female	55+ years	Non-melanoma skin cancer (basal-cell	Number	2021	0.6733975	0.938157189	0.466275874
Prevalence	Djibouti	Both	55+ years	Non-melanoma skin cancer (basal-cell	Number	2021	1.9226443	2.641705773	1.343424469
Prevalence	Djibouti	Male	55+ years	Non-melanoma skin cancer (basal-cell	Percent	2021	2.17E-05	2.98E-05	1.52E-05
Prevalence	Djibouti	Female	55+ years	Non-melanoma skin cancer (basal-cell	Percent	2021	1.46E-05	2.04E-05	1.01E-05
Prevalence	Djibouti	Both	55+ years	Non-melanoma skin cancer (basal-cell	Percent	2021	1.86E-05	2.55E-05	1.30E-05
Prevalence	Djibouti	Male	55+ years	Non-melanoma skin cancer (basal-cell	Rate	2021	2.1675703	2.974802546	1.516223733
Prevalence	Djibouti	Female	55+ years	Non-melanoma skin cancer (basal-cell	Rate	2021	1.4616718	2.036357289	1.012095079
Prevalence	Djibouti	Both	55+ years	Non-melanoma skin cancer (basal-cell	Rate	2021	1.8539755	2.547354998	1.295442918
Prevalence	Somalia	Male	55+ years	Non-melanoma skin cancer (basal-cell	Number	1990	3.3293268	4.615869464	2.407526415
Prevalence	Somalia	Female	55+ years	Non-melanoma skin cancer (basal-cell	Number	1990	2.4247352	3.358067149	1.602186311
Prevalence	Somalia	Both	55+ years	Non-melanoma skin cancer (basal-cell	Number	1990	5.754062	7.781292369	4.133322223
Prevalence	Somalia	Male	55+ years	Non-melanoma skin cancer (basal-cell	Percent	1990	1.96E-05	2.72E-05	1.42E-05
Prevalence	Somalia	Female	55+ years	Non-melanoma skin cancer (basal-cell	Percent	1990	1.27E-05	1.76E-05	8.42E-06
Prevalence	Somalia	Both	55+ years	Non-melanoma skin cancer (basal-cell	Percent	1990	1.60E-05	2.16E-05	1.15E-05
Prevalence	Somalia	Male	55+ years	Non-melanoma skin cancer (basal-cell	Rate	1990	1.9616157	2.719637526	1.418497476
Prevalence	Somalia	Female	55+ years	Non-melanoma skin cancer (basal-cell	Rate	1990	1.2738544	1.764187966	0.841721646
Prevalence	Somalia	Both	55+ years	Non-melanoma skin cancer (basal-cell	Rate	1990	1.5980397	2.161049677	1.147921737
Prevalence	Somalia	Male	55+ years	Non-melanoma skin cancer (basal-cell	Number	2021	7.6575789	10.7691204	5.351408019
Prevalence	Somalia	Female	55+ years	Non-melanoma skin cancer (basal-cell	Number	2021	7.1570881	10.0212011	4.8961815
Prevalence	Somalia	Both	55+ years	Non-melanoma skin cancer (basal-cell	Number	2021	14.814667	20.47172486	10.42190862
Prevalence	Somalia	Male	55+ years	Non-melanoma skin cancer (basal-cell	Percent	2021	1.97E-05	2.78E-05	1.38E-05
Prevalence	Somalia	Female	55+ years	Non-melanoma skin cancer (basal-cell	Percent	2021	1.29E-05	1.81E-05	8.82E-06
Prevalence	Somalia	Both	55+ years	Non-melanoma skin cancer (basal-cell	Percent	2021	1.57E-05	2.17E-05	1.11E-05
Prevalence	Somalia	Male	55+ years	Non-melanoma skin cancer (basal-cell	Rate	2021	1.9739026	2.775968159	1.379438406
Prevalence	Somalia	Female	55+ years	Non-melanoma skin cancer (basal-cell	Rate	2021	1.2892873	1.805232422	0.882004612
Prevalence	Somalia	Both	55+ years	Non-melanoma skin cancer (basal-cell	Rate	2021	1.5709133	2.170774705	1.105115263
Prevalence	Bhutan	Male	55+ years	Non-melanoma skin cancer (basal-cell	Number	1990	0.005103	0.012493475	0.001269243
Prevalence	Bhutan	Female	55+ years	Non-melanoma skin cancer (basal-cell	Number	1990	0.0034451	0.007886692	0.000980814
Prevalence	Bhutan	Both	55+ years	Non-melanoma skin cancer (basal-cell	Number	1990	0.0085481	0.019924957	0.003045401
Prevalence	Bhutan	Male	55+ years	Non-melanoma skin cancer (basal-cell	Percent	1990	2.55E-07	6.24E-07	6.33E-08
Prevalence	Bhutan	Female	55+ years	Non-melanoma skin cancer (basal-cell	Percent	1990	1.75E-07	4.02E-07	5.00E-08
Prevalence	Bhutan	Both	55+ years	Non-melanoma skin cancer (basal-cell	Percent	1990	2.15E-07	5.02E-07	7.68E-08
Prevalence	Bhutan	Male	55+ years	Non-melanoma skin cancer (basal-cell	Rate	1990	0.0254524	0.062314285	0.006330662
Prevalence	Bhutan	Female	55+ years	Non-melanoma skin cancer (basal-cell	Rate	1990	0.0175445	0.040163846	0.004994901
Prevalence	Bhutan	Both	55+ years	Non-melanoma skin cancer (basal-cell	Rate	1990	0.0215396	0.050207231	0.00767385

Prevalence	Bhutan	Male	55+ years	Non-melanoma skin cancer (basal-cell	Number	2021	0.0140264	0.031427684	0.003334362
Prevalence	Bhutan	Female	55+ years	Non-melanoma skin cancer (basal-cell	Number	2021	0.0088747	0.019921164	0.002367375
Prevalence	Bhutan	Both	55+ years	Non-melanoma skin cancer (basal-cell	Number	2021	0.0229011	0.049895706	0.007899034
Prevalence	Bhutan	Male	55+ years	Non-melanoma skin cancer (basal-cell	Percent	2021	2.78E-07	6.23E-07	6.60E-08
Prevalence	Bhutan	Female	55+ years	Non-melanoma skin cancer (basal-cell	Percent	2021	1.81E-07	4.06E-07	4.83E-08
Prevalence	Bhutan	Both	55+ years	Non-melanoma skin cancer (basal-cell	Percent	2021	2.30E-07	5.01E-07	7.94E-08
Prevalence	Bhutan	Male	55+ years	Non-melanoma skin cancer (basal-cell	Rate	2021	0.0277602	0.062199787	0.006599169
Prevalence	Bhutan	Female	55+ years	Non-melanoma skin cancer (basal-cell	Rate	2021	0.0180883	0.040603134	0.004825163
Prevalence	Bhutan	Both	55+ years	Non-melanoma skin cancer (basal-cell	Rate	2021	0.0229953	0.050101063	0.007931544
Prevalence	Madagascar	Male	55+ years	Non-melanoma skin cancer (basal-cell	Number	1990	10.278464	14.223026	7.5246264
Prevalence	Madagascar	Female	55+ years	Non-melanoma skin cancer (basal-cell	Number	1990	5.925106	8.138901593	4.177428458
Prevalence	Madagascar	Both	55+ years	Non-melanoma skin cancer (basal-cell	Number	1990	16.20357	22.36087413	11.7001912
Prevalence	Madagascar	Male	55+ years	Non-melanoma skin cancer (basal-cell	Percent	1990	2.39E-05	3.31E-05	1.75E-05
Prevalence	Madagascar	Female	55+ years	Non-melanoma skin cancer (basal-cell	Percent	1990	1.43E-05	1.96E-05	1.01E-05
Prevalence	Madagascar	Both	55+ years	Non-melanoma skin cancer (basal-cell	Percent	1990	1.92E-05	2.65E-05	1.38E-05
Prevalence	Madagascar	Male	55+ years	Non-melanoma skin cancer (basal-cell	Rate	1990	2.3935708	3.312150502	1.752277968
Prevalence	Madagascar	Female	55+ years	Non-melanoma skin cancer (basal-cell	Rate	1990	1.4260561	1.958873023	1.005424604
Prevalence	Madagascar	Both	55+ years	Non-melanoma skin cancer (basal-cell	Rate	1990	1.9177896	2.64654337	1.3847877
Prevalence	Madagascar	Male	55+ years	Non-melanoma skin cancer (basal-cell	Number	2021	19.600248	27.11984493	14.26826341
Prevalence	Madagascar	Female	55+ years	Non-melanoma skin cancer (basal-cell	Number	2021	13.018388	18.02686282	8.974429932
Prevalence	Madagascar	Both	55+ years	Non-melanoma skin cancer (basal-cell	Number	2021	32.618636	44.59753963	23.50495386
Prevalence	Madagascar	Male	55+ years	Non-melanoma skin cancer (basal-cell	Percent	2021	2.18E-05	3.02E-05	1.59E-05
Prevalence	Madagascar	Female	55+ years	Non-melanoma skin cancer (basal-cell	Percent	2021	1.37E-05	1.90E-05	9.47E-06
Prevalence	Madagascar	Both	55+ years	Non-melanoma skin cancer (basal-cell	Percent	2021	1.77E-05	2.42E-05	1.27E-05
Prevalence	Madagascar	Male	55+ years	Non-melanoma skin cancer (basal-cell	Rate	2021	2.179852	3.01614783	1.586852426
Prevalence	Madagascar	Female	55+ years	Non-melanoma skin cancer (basal-cell	Rate	2021	1.3739355	1.902520219	0.947143969
Prevalence	Madagascar	Both	55+ years	Non-melanoma skin cancer (basal-cell	Rate	2021	1.766339	2.415011187	1.272821931
Prevalence	Comoros	Male	55+ years	Non-melanoma skin cancer (basal-cell	Number	1990	0.383716	0.512849969	0.27860784
Prevalence	Comoros	Female	55+ years	Non-melanoma skin cancer (basal-cell	Number	1990	0.2405048	0.333977213	0.167936479
Prevalence	Comoros	Both	55+ years	Non-melanoma skin cancer (basal-cell	Number	1990	0.6242208	0.8337676	0.452754821
Prevalence	Comoros	Male	55+ years	Non-melanoma skin cancer (basal-cell	Percent	1990	2.39E-05	3.20E-05	1.74E-05
Prevalence	Comoros	Female	55+ years	Non-melanoma skin cancer (basal-cell	Percent	1990	1.46E-05	2.03E-05	1.02E-05
Prevalence	Comoros	Both	55+ years	Non-melanoma skin cancer (basal-cell	Percent	1990	1.92E-05	2.57E-05	1.40E-05
Prevalence	Comoros	Male	55+ years	Non-melanoma skin cancer (basal-cell	Rate	1990	2.3928341	3.198106891	1.73738463
Prevalence	Comoros	Female	55+ years	Non-melanoma skin cancer (basal-cell	Rate	1990	1.46484	2.034151336	1.022848864
Prevalence	Comoros	Both	55+ years	Non-melanoma skin cancer (basal-cell	Rate	1990	1.9233691	2.569031291	1.395042578
Prevalence	Comoros	Male	55+ years	Non-melanoma skin cancer (basal-cell	Number	2021	0.9073667	1.266092741	0.643382214
Prevalence	Comoros	Female	55+ years	Non-melanoma skin cancer (basal-cell	Number	2021	0.6572212	0.887382472	0.45812801
Prevalence	Comoros	Both	55+ years	Non-melanoma skin cancer (basal-cell	Number	2021	1.564588	2.133901693	1.110635149
Prevalence	Comoros	Male	55+ years	Non-melanoma skin cancer (basal-cell	Percent	2021	2.45E-05	3.42E-05	1.74E-05
Prevalence	Comoros	Female	55+ years	Non-melanoma skin cancer (basal-cell	Percent	2021	1.50E-05	2.03E-05	1.05E-05
Prevalence	Comoros	Both	55+ years	Non-melanoma skin cancer (basal-cell	Percent	2021	1.94E-05	2.64E-05	1.37E-05
Prevalence	Comoros	Male	55+ years	Non-melanoma skin cancer (basal-cell	Rate	2021	2.4517121	3.420992628	1.738423844
Prevalence	Comoros	Female	55+ years	Non-melanoma skin cancer (basal-cell	Rate	2021	1.499785	2.025015111	1.045452408
Prevalence	Comoros	Both	55+ years	Non-melanoma skin cancer (basal-cell	Rate	2021	1.9356396	2.639969496	1.37402905
Prevalence	Palestine	Male	55+ years	Non-melanoma skin cancer (basal-cell	Number	1990	2.1864201	2.997852904	1.621581979
Prevalence	Palestine	Female	55+ years	Non-melanoma skin cancer (basal-cell	Number	1990	1.6010202	2.127818432	1.148673087
Prevalence	Palestine	Both	55+ years	Non-melanoma skin cancer (basal-cell	Number	1990	3.7874403	5.116282594	2.815364422
Prevalence	Palestine	Male	55+ years	Non-melanoma skin cancer (basal-cell	Percent	1990	3.35E-05	4.60E-05	2.49E-05
Prevalence	Palestine	Female	55+ years	Non-melanoma skin cancer (basal-cell	Percent	1990	2.00E-05	2.66E-05	1.44E-05
Prevalence	Palestine	Both	55+ years	Non-melanoma skin cancer (basal-cell	Percent	1990	2.61E-05	3.53E-05	1.94E-05
Prevalence	Palestine	Male	55+ years	Non-melanoma skin cancer (basal-cell	Rate	1990	3.3504509	4.593883455	2.48489798
Prevalence	Palestine	Female	55+ years	Non-melanoma skin cancer (basal-cell	Rate	1990	2.0041405	2.663581002	1.437897034
Prevalence	Palestine	Both	55+ years	Non-melanoma skin cancer (basal-cell	Rate	1990	2.6094523	3.524991565	1.939716123
Prevalence	Palestine	Male	55+ years	Non-melanoma skin cancer (basal-cell	Number	2021	6.3289268	8.602260438	4.701573354
Prevalence	Palestine	Female	55+ years	Non-melanoma skin cancer (basal-cell	Number	2021	4.2759226	5.928707002	3.062195535

Prevalence	Palestine	Both	55+ years	Non-melanoma skin cancer (basal-cell	Number	2021	10.604849	14.24480294	7.857547214
Prevalence	Palestine	Male	55+ years	Non-melanoma skin cancer (basal-cell	Percent	2021	2.98E-05	4.05E-05	2.21E-05
Prevalence	Palestine	Female	55+ years	Non-melanoma skin cancer (basal-cell	Percent	2021	1.96E-05	2.72E-05	1.41E-05
Prevalence	Palestine	Both	55+ years	Non-melanoma skin cancer (basal-cell	Percent	2021	2.47E-05	3.31E-05	1.83E-05
Prevalence	Palestine	Male	55+ years	Non-melanoma skin cancer (basal-cell	Rate	2021	2.9775525	4.047081333	2.211936025
Prevalence	Palestine	Female	55+ years	Non-melanoma skin cancer (basal-cell	Rate	2021	1.9640831	2.723265658	1.406575149
Prevalence	Palestine	Both	55+ years	Non-melanoma skin cancer (basal-cell	Rate	2021	2.4647511	3.310739477	1.82623037
Prevalence	Angola	Male	55+ years	Non-melanoma skin cancer (basal-cell	Number	1990	8.2035304	11.18659845	5.748196479
Prevalence	Angola	Female	55+ years	Non-melanoma skin cancer (basal-cell	Number	1990	4.9119184	7.006171184	3.502273196
Prevalence	Angola	Both	55+ years	Non-melanoma skin cancer (basal-cell	Number	1990	13.115449	18.13183217	9.503392648
Prevalence	Angola	Male	55+ years	Non-melanoma skin cancer (basal-cell	Percent	1990	2.62E-05	3.57E-05	1.83E-05
Prevalence	Angola	Female	55+ years	Non-melanoma skin cancer (basal-cell	Percent	1990	1.59E-05	2.26E-05	1.13E-05
Prevalence	Angola	Both	55+ years	Non-melanoma skin cancer (basal-cell	Percent	1990	2.11E-05	2.91E-05	1.53E-05
Prevalence	Angola	Male	55+ years	Non-melanoma skin cancer (basal-cell	Rate	1990	2.6182095	3.570274907	1.83457391
Prevalence	Angola	Female	55+ years	Non-melanoma skin cancer (basal-cell	Rate	1990	1.586612	2.263082278	1.131278725
Prevalence	Angola	Both	55+ years	Non-melanoma skin cancer (basal-cell	Rate	1990	2.1055082	2.910820791	1.52564135
Prevalence	Angola	Male	55+ years	Non-melanoma skin cancer (basal-cell	Number	2021	22.257758	31.11946704	16.15582583
Prevalence	Angola	Female	55+ years	Non-melanoma skin cancer (basal-cell	Number	2021	16.755732	22.96547853	11.96299357
Prevalence	Angola	Both	55+ years	Non-melanoma skin cancer (basal-cell	Number	2021	39.013491	54.55870358	28.5539145
Prevalence	Angola	Male	55+ years	Non-melanoma skin cancer (basal-cell	Percent	2021	2.57E-05	3.60E-05	1.87E-05
Prevalence	Angola	Female	55+ years	Non-melanoma skin cancer (basal-cell	Percent	2021	1.57E-05	2.15E-05	1.12E-05
Prevalence	Angola	Both	55+ years	Non-melanoma skin cancer (basal-cell	Percent	2021	2.02E-05	2.82E-05	1.48E-05
Prevalence	Angola	Male	55+ years	Non-melanoma skin cancer (basal-cell	Rate	2021	2.5728653	3.597226416	1.867517955
Prevalence	Angola	Female	55+ years	Non-melanoma skin cancer (basal-cell	Rate	2021	1.5681046	2.149250879	1.119570595
Prevalence	Angola	Both	55+ years	Non-melanoma skin cancer (basal-cell	Rate	2021	2.0176293	2.821568589	1.476699828
Prevalence	Uganda	Male	55+ years	Non-melanoma skin cancer (basal-cell	Number	1990	16.457738	20.549034	12.85695592
Prevalence	Uganda	Female	55+ years	Non-melanoma skin cancer (basal-cell	Number	1990	11.38993	14.62772014	8.664439495
Prevalence	Uganda	Both	55+ years	Non-melanoma skin cancer (basal-cell	Number	1990	27.847668	34.53582261	22.01033041
Prevalence	Uganda	Male	55+ years	Non-melanoma skin cancer (basal-cell	Percent	1990	3.21E-05	4.00E-05	2.50E-05
Prevalence	Uganda	Female	55+ years	Non-melanoma skin cancer (basal-cell	Percent	1990	2.14E-05	2.75E-05	1.63E-05
Prevalence	Uganda	Both	55+ years	Non-melanoma skin cancer (basal-cell	Percent	1990	2.66E-05	3.30E-05	2.10E-05
Prevalence	Uganda	Male	55+ years	Non-melanoma skin cancer (basal-cell	Rate	1990	3.2062343	4.003284988	2.504743465
Prevalence	Uganda	Female	55+ years	Non-melanoma skin cancer (basal-cell	Rate	1990	2.1385061	2.746414543	1.626784106
Prevalence	Uganda	Both	55+ years	Non-melanoma skin cancer (basal-cell	Rate	1990	2.6625153	3.30196963	2.104407455
Prevalence	Uganda	Male	55+ years	Non-melanoma skin cancer (basal-cell	Number	2021	28.272317	36.84906226	21.42494766
Prevalence	Uganda	Female	55+ years	Non-melanoma skin cancer (basal-cell	Number	2021	26.623201	35.38677359	19.71356588
Prevalence	Uganda	Both	55+ years	Non-melanoma skin cancer (basal-cell	Number	2021	54.895518	71.74072028	42.4631092
Prevalence	Uganda	Male	55+ years	Non-melanoma skin cancer (basal-cell	Percent	2021	2.72E-05	3.54E-05	2.06E-05
Prevalence	Uganda	Female	55+ years	Non-melanoma skin cancer (basal-cell	Percent	2021	2.00E-05	2.66E-05	1.48E-05
Prevalence	Uganda	Both	55+ years	Non-melanoma skin cancer (basal-cell	Percent	2021	2.32E-05	3.03E-05	1.79E-05
Prevalence	Uganda	Male	55+ years	Non-melanoma skin cancer (basal-cell	Rate	2021	2.718665	3.543404592	2.060222249
Prevalence	Uganda	Female	55+ years	Non-melanoma skin cancer (basal-cell	Rate	2021	1.9994016	2.657545594	1.480488183
Prevalence	Uganda	Both	55+ years	Non-melanoma skin cancer (basal-cell	Rate	2021	2.3148091	3.025129823	1.790564933
Prevalence	Democratic	Male	55+ years	Non-melanoma skin cancer (basal-cell	Number	1990	33.03694	45.3882022	23.07862559
Prevalence	Democratic	Female	55+ years	Non-melanoma skin cancer (basal-cell	Number	1990	22.102553	31.38387787	15.30911332
Prevalence	Democratic	Both	55+ years	Non-melanoma skin cancer (basal-cell	Number	1990	55.139494	75.69318515	38.28127315
Prevalence	Democratic	Male	55+ years	Non-melanoma skin cancer (basal-cell	Percent	1990	2.72E-05	3.74E-05	1.90E-05
Prevalence	Democratic	Female	55+ years	Non-melanoma skin cancer (basal-cell	Percent	1990	1.55E-05	2.20E-05	1.07E-05
Prevalence	Democratic	Both	55+ years	Non-melanoma skin cancer (basal-cell	Percent	1990	2.09E-05	2.87E-05	1.45E-05
Prevalence	Democratic	Male	55+ years	Non-melanoma skin cancer (basal-cell	Rate	1990	2.7215086	3.738977651	1.901165085
Prevalence	Democratic	Female	55+ years	Non-melanoma skin cancer (basal-cell	Rate	1990	1.5490761	2.199565568	1.072952128
Prevalence	Democratic	Both	55+ years	Non-melanoma skin cancer (basal-cell	Rate	1990	2.0880305	2.86636072	1.449640908
Prevalence	Democratic	Male	55+ years	Non-melanoma skin cancer (basal-cell	Number	2021	71.113258	95.41487323	50.53185238
Prevalence	Democratic	Female	55+ years	Non-melanoma skin cancer (basal-cell	Number	2021	53.137373	71.220467	37.75305305
Prevalence	Democratic	Both	55+ years	Non-melanoma skin cancer (basal-cell	Number	2021	124.25063	166.8786897	88.97943934
Prevalence	Democratic	Male	55+ years	Non-melanoma skin cancer (basal-cell	Percent	2021	2.57E-05	3.45E-05	1.83E-05

Prevalence	Democratic	Female	55+ years	Non-melanoma skin cancer (basal-cell	Percent	2021	1.62E-05	2.17E-05	1.15E-05
Prevalence	Democratic	Both	55+ years	Non-melanoma skin cancer (basal-cell	Percent	2021	2.06E-05	2.76E-05	1.47E-05
Prevalence	Democratic	Male	55+ years	Non-melanoma skin cancer (basal-cell	Rate	2021	2.5742833	3.453996102	1.829241241
Prevalence	Democratic	Female	55+ years	Non-melanoma skin cancer (basal-cell	Rate	2021	1.621344	2.173100917	1.151932831
Prevalence	Democratic	Both	55+ years	Non-melanoma skin cancer (basal-cell	Rate	2021	2.0571928	2.762977018	1.473214742
Prevalence	Afghanista	Male	55+ years	Non-melanoma skin cancer (basal-cell	Number	1990	19.463626	26.73114289	14.01238383
Prevalence	Afghanista	Female	55+ years	Non-melanoma skin cancer (basal-cell	Number	1990	9.0415472	12.67364582	6.327316728
Prevalence	Afghanista	Both	55+ years	Non-melanoma skin cancer (basal-cell	Number	1990	28.505174	39.25095903	20.43451703
Prevalence	Afghanista	Male	55+ years	Non-melanoma skin cancer (basal-cell	Percent	1990	3.01E-05	4.14E-05	2.17E-05
Prevalence	Afghanista	Female	55+ years	Non-melanoma skin cancer (basal-cell	Percent	1990	1.59E-05	2.23E-05	1.11E-05
Prevalence	Afghanista	Both	55+ years	Non-melanoma skin cancer (basal-cell	Percent	1990	2.35E-05	3.23E-05	1.68E-05
Prevalence	Afghanista	Male	55+ years	Non-melanoma skin cancer (basal-cell	Rate	1990	3.011281	4.135662085	2.167901492
Prevalence	Afghanista	Female	55+ years	Non-melanoma skin cancer (basal-cell	Rate	1990	1.589503	2.22802555	1.112341589
Prevalence	Afghanista	Both	55+ years	Non-melanoma skin cancer (basal-cell	Rate	1990	2.3457465	3.230038185	1.681596372
Prevalence	Afghanista	Male	55+ years	Non-melanoma skin cancer (basal-cell	Number	2021	17.283254	23.63684395	12.31449935
Prevalence	Afghanista	Female	55+ years	Non-melanoma skin cancer (basal-cell	Number	2021	11.77177	16.03668449	8.446603634
Prevalence	Afghanista	Both	55+ years	Non-melanoma skin cancer (basal-cell	Number	2021	29.055025	39.7591854	20.66164821
Prevalence	Afghanista	Male	55+ years	Non-melanoma skin cancer (basal-cell	Percent	2021	3.31E-05	4.53E-05	2.36E-05
Prevalence	Afghanista	Female	55+ years	Non-melanoma skin cancer (basal-cell	Percent	2021	1.67E-05	2.27E-05	1.20E-05
Prevalence	Afghanista	Both	55+ years	Non-melanoma skin cancer (basal-cell	Percent	2021	2.36E-05	3.24E-05	1.68E-05
Prevalence	Afghanista	Male	55+ years	Non-melanoma skin cancer (basal-cell	Rate	2021	3.3097329	4.526441468	2.358219255
Prevalence	Afghanista	Female	55+ years	Non-melanoma skin cancer (basal-cell	Rate	2021	1.6660765	2.269696236	1.195460601
Prevalence	Afghanista	Both	55+ years	Non-melanoma skin cancer (basal-cell	Rate	2021	2.3645977	3.235739124	1.681515927
Prevalence	Botswana	Male	55+ years	Non-melanoma skin cancer (basal-cell	Number	1990	3.3986228	4.456112756	2.480879747
Prevalence	Botswana	Female	55+ years	Non-melanoma skin cancer (basal-cell	Number	1990	2.6764909	3.577560333	1.961528147
Prevalence	Botswana	Both	55+ years	Non-melanoma skin cancer (basal-cell	Number	1990	6.0751138	7.999578715	4.53165819
Prevalence	Botswana	Male	55+ years	Non-melanoma skin cancer (basal-cell	Percent	1990	8.18E-05	0.000107215	5.97E-05
Prevalence	Botswana	Female	55+ years	Non-melanoma skin cancer (basal-cell	Percent	1990	5.21E-05	6.96E-05	3.82E-05
Prevalence	Botswana	Both	55+ years	Non-melanoma skin cancer (basal-cell	Percent	1990	6.54E-05	8.61E-05	4.88E-05
Prevalence	Botswana	Male	55+ years	Non-melanoma skin cancer (basal-cell	Rate	1990	8.1734166	10.71659545	5.966317746
Prevalence	Botswana	Female	55+ years	Non-melanoma skin cancer (basal-cell	Rate	1990	5.2083753	6.961830749	3.817078037
Prevalence	Botswana	Both	55+ years	Non-melanoma skin cancer (basal-cell	Rate	1990	6.534514	8.604507089	4.874342314
Prevalence	Botswana	Male	55+ years	Non-melanoma skin cancer (basal-cell	Number	2021	8.3582368	10.99367881	6.110281311
Prevalence	Botswana	Female	55+ years	Non-melanoma skin cancer (basal-cell	Number	2021	7.1390761	9.335061301	5.275657215
Prevalence	Botswana	Both	55+ years	Non-melanoma skin cancer (basal-cell	Number	2021	15.497313	20.14268022	11.66050796
Prevalence	Botswana	Male	55+ years	Non-melanoma skin cancer (basal-cell	Percent	2021	8.02E-05	0.000105425	5.86E-05
Prevalence	Botswana	Female	55+ years	Non-melanoma skin cancer (basal-cell	Percent	2021	5.17E-05	6.76E-05	3.82E-05
Prevalence	Botswana	Both	55+ years	Non-melanoma skin cancer (basal-cell	Percent	2021	6.39E-05	8.31E-05	4.81E-05
Prevalence	Botswana	Male	55+ years	Non-melanoma skin cancer (basal-cell	Rate	2021	8.0119887	10.53825486	5.857156901
Prevalence	Botswana	Female	55+ years	Non-melanoma skin cancer (basal-cell	Rate	2021	5.1694297	6.759550216	3.820121659
Prevalence	Botswana	Both	55+ years	Non-melanoma skin cancer (basal-cell	Rate	2021	6.3926628	8.308883182	4.809975506
Prevalence	Lesotho	Male	55+ years	Non-melanoma skin cancer (basal-cell	Number	1990	4.1628083	5.404747556	3.098489323
Prevalence	Lesotho	Female	55+ years	Non-melanoma skin cancer (basal-cell	Number	1990	4.6981201	6.258937927	3.467981204
Prevalence	Lesotho	Both	55+ years	Non-melanoma skin cancer (basal-cell	Number	1990	8.8609283	11.70480872	6.716903547
Prevalence	Lesotho	Male	55+ years	Non-melanoma skin cancer (basal-cell	Percent	1990	8.23E-05	0.00010689	6.13E-05
Prevalence	Lesotho	Female	55+ years	Non-melanoma skin cancer (basal-cell	Percent	1990	5.26E-05	7.01E-05	3.89E-05
Prevalence	Lesotho	Both	55+ years	Non-melanoma skin cancer (basal-cell	Percent	1990	6.34E-05	8.37E-05	4.80E-05
Prevalence	Lesotho	Male	55+ years	Non-melanoma skin cancer (basal-cell	Rate	1990	8.2292437	10.68437023	6.125245764
Prevalence	Lesotho	Female	55+ years	Non-melanoma skin cancer (basal-cell	Rate	1990	5.2637685	7.012507069	3.885522271
Prevalence	Lesotho	Both	55+ years	Non-melanoma skin cancer (basal-cell	Rate	1990	6.3364999	8.37017479	4.803295642
Prevalence	Lesotho	Male	55+ years	Non-melanoma skin cancer (basal-cell	Number	2021	5.3014255	7.02086495	3.947729532
Prevalence	Lesotho	Female	55+ years	Non-melanoma skin cancer (basal-cell	Number	2021	5.7431485	7.573393353	4.302398475
Prevalence	Lesotho	Both	55+ years	Non-melanoma skin cancer (basal-cell	Number	2021	11.044574	14.56411116	8.398857587
Prevalence	Lesotho	Male	55+ years	Non-melanoma skin cancer (basal-cell	Percent	2021	7.84E-05	0.000103814	5.84E-05
Prevalence	Lesotho	Female	55+ years	Non-melanoma skin cancer (basal-cell	Percent	2021	5.09E-05	6.71E-05	3.81E-05
Prevalence	Lesotho	Both	55+ years	Non-melanoma skin cancer (basal-cell	Percent	2021	6.12E-05	8.07E-05	4.65E-05

Prevalence	Lesotho	Male	55+ years	Non-melanoma skin cancer (basal-cell	Rate	2021	7.8350867	10.37628194	5.83443136
Prevalence	Lesotho	Female	55+ years	Non-melanoma skin cancer (basal-cell	Rate	2021	5.0873023	6.708539992	3.811080567
Prevalence	Lesotho	Both	55+ years	Non-melanoma skin cancer (basal-cell	Rate	2021	6.1170321	8.066326166	4.651703353
Prevalence	Zambia	Male	55+ years	Non-melanoma skin cancer (basal-cell	Number	1990	5.4137198	7.430674836	3.798507796
Prevalence	Zambia	Female	55+ years	Non-melanoma skin cancer (basal-cell	Number	1990	2.9432229	4.119269674	2.037207336
Prevalence	Zambia	Both	55+ years	Non-melanoma skin cancer (basal-cell	Number	1990	8.3569428	11.50593582	5.849030177
Prevalence	Zambia	Male	55+ years	Non-melanoma skin cancer (basal-cell	Percent	1990	2.21E-05	3.04E-05	1.55E-05
Prevalence	Zambia	Female	55+ years	Non-melanoma skin cancer (basal-cell	Percent	1990	1.36E-05	1.91E-05	9.44E-06
Prevalence	Zambia	Both	55+ years	Non-melanoma skin cancer (basal-cell	Percent	1990	1.82E-05	2.50E-05	1.27E-05
Prevalence	Zambia	Male	55+ years	Non-melanoma skin cancer (basal-cell	Rate	1990	2.2145208	3.039570632	1.553806753
Prevalence	Zambia	Female	55+ years	Non-melanoma skin cancer (basal-cell	Rate	1990	1.3634806	1.908297322	0.943758873
Prevalence	Zambia	Both	55+ years	Non-melanoma skin cancer (basal-cell	Rate	1990	1.8154416	2.499521055	1.270628857
Prevalence	Zambia	Male	55+ years	Non-melanoma skin cancer (basal-cell	Number	2021	10.917684	15.49691194	7.827673818
Prevalence	Zambia	Female	55+ years	Non-melanoma skin cancer (basal-cell	Number	2021	7.8116731	10.60324458	5.518291676
Prevalence	Zambia	Both	55+ years	Non-melanoma skin cancer (basal-cell	Number	2021	18.729357	25.66455015	13.44312967
Prevalence	Zambia	Male	55+ years	Non-melanoma skin cancer (basal-cell	Percent	2021	2.12E-05	3.01E-05	1.52E-05
Prevalence	Zambia	Female	55+ years	Non-melanoma skin cancer (basal-cell	Percent	2021	1.38E-05	1.87E-05	9.76E-06
Prevalence	Zambia	Both	55+ years	Non-melanoma skin cancer (basal-cell	Percent	2021	1.73E-05	2.38E-05	1.24E-05
Prevalence	Zambia	Male	55+ years	Non-melanoma skin cancer (basal-cell	Rate	2021	2.1222047	3.012325786	1.521561443
Prevalence	Zambia	Female	55+ years	Non-melanoma skin cancer (basal-cell	Rate	2021	1.3808658	1.874330586	0.975465839
Prevalence	Zambia	Both	55+ years	Non-melanoma skin cancer (basal-cell	Rate	2021	1.7339454	2.375998731	1.244551681
Prevalence	Congo	Male	55+ years	Non-melanoma skin cancer (basal-cell	Number	1990	2.095544	2.913209848	1.495400005
Prevalence	Congo	Female	55+ years	Non-melanoma skin cancer (basal-cell	Number	1990	1.5547196	2.082745138	1.084554217
Prevalence	Congo	Both	55+ years	Non-melanoma skin cancer (basal-cell	Number	1990	3.6502636	5.009718839	2.673808677
Prevalence	Congo	Male	55+ years	Non-melanoma skin cancer (basal-cell	Percent	1990	2.71E-05	3.77E-05	1.94E-05
Prevalence	Congo	Female	55+ years	Non-melanoma skin cancer (basal-cell	Percent	1990	1.54E-05	2.07E-05	1.08E-05
Prevalence	Congo	Both	55+ years	Non-melanoma skin cancer (basal-cell	Percent	1990	2.05E-05	2.81E-05	1.50E-05
Prevalence	Congo	Male	55+ years	Non-melanoma skin cancer (basal-cell	Rate	1990	2.7137954	3.772698495	1.936590099
Prevalence	Congo	Female	55+ years	Non-melanoma skin cancer (basal-cell	Rate	1990	1.5424652	2.066328839	1.076005707
Prevalence	Congo	Both	55+ years	Non-melanoma skin cancer (basal-cell	Rate	1990	2.050564	2.814248526	1.502032822
Prevalence	Congo	Male	55+ years	Non-melanoma skin cancer (basal-cell	Number	2021	5.486971	7.333594533	3.917306309
Prevalence	Congo	Female	55+ years	Non-melanoma skin cancer (basal-cell	Number	2021	3.3775792	4.550301479	2.413009276
Prevalence	Congo	Both	55+ years	Non-melanoma skin cancer (basal-cell	Number	2021	8.8645502	11.78808352	6.342936569
Prevalence	Congo	Male	55+ years	Non-melanoma skin cancer (basal-cell	Percent	2021	2.58E-05	3.45E-05	1.84E-05
Prevalence	Congo	Female	55+ years	Non-melanoma skin cancer (basal-cell	Percent	2021	1.52E-05	2.05E-05	1.09E-05
Prevalence	Congo	Both	55+ years	Non-melanoma skin cancer (basal-cell	Percent	2021	2.04E-05	2.71E-05	1.46E-05
Prevalence	Congo	Male	55+ years	Non-melanoma skin cancer (basal-cell	Rate	2021	2.5823873	3.451481896	1.843640488
Prevalence	Congo	Female	55+ years	Non-melanoma skin cancer (basal-cell	Rate	2021	1.5210775	2.049207659	1.086687797
Prevalence	Congo	Both	55+ years	Non-melanoma skin cancer (basal-cell	Rate	2021	2.0400391	2.712845095	1.459728745
Prevalence	Ethiopia	Male	55+ years	Non-melanoma skin cancer (basal-cell	Number	1990	39.054512	53.12323702	28.46337867
Prevalence	Ethiopia	Female	55+ years	Non-melanoma skin cancer (basal-cell	Number	1990	21.04549	28.4944073	14.70890333
Prevalence	Ethiopia	Both	55+ years	Non-melanoma skin cancer (basal-cell	Number	1990	60.100002	82.42116303	43.88059207
Prevalence	Ethiopia	Male	55+ years	Non-melanoma skin cancer (basal-cell	Percent	1990	2.28E-05	3.11E-05	1.67E-05
Prevalence	Ethiopia	Female	55+ years	Non-melanoma skin cancer (basal-cell	Percent	1990	1.40E-05	1.90E-05	9.78E-06
Prevalence	Ethiopia	Both	55+ years	Non-melanoma skin cancer (basal-cell	Percent	1990	1.87E-05	2.57E-05	1.37E-05
Prevalence	Ethiopia	Male	55+ years	Non-melanoma skin cancer (basal-cell	Rate	1990	2.2846848	3.107703685	1.665104608
Prevalence	Ethiopia	Female	55+ years	Non-melanoma skin cancer (basal-cell	Rate	1990	1.3996951	1.895108199	0.978260857
Prevalence	Ethiopia	Both	55+ years	Non-melanoma skin cancer (basal-cell	Rate	1990	1.8705367	2.565254717	1.365728069
Prevalence	Ethiopia	Male	55+ years	Non-melanoma skin cancer (basal-cell	Number	2021	83.979715	111.9485967	61.18599899
Prevalence	Ethiopia	Female	55+ years	Non-melanoma skin cancer (basal-cell	Number	2021	49.380489	65.32670378	35.52258364
Prevalence	Ethiopia	Both	55+ years	Non-melanoma skin cancer (basal-cell	Number	2021	133.3602	176.9663089	97.53346157
Prevalence	Ethiopia	Male	55+ years	Non-melanoma skin cancer (basal-cell	Percent	2021	2.41E-05	3.22E-05	1.76E-05
Prevalence	Ethiopia	Female	55+ years	Non-melanoma skin cancer (basal-cell	Percent	2021	1.47E-05	1.94E-05	1.06E-05
Prevalence	Ethiopia	Both	55+ years	Non-melanoma skin cancer (basal-cell	Percent	2021	1.95E-05	2.59E-05	1.43E-05
Prevalence	Ethiopia	Male	55+ years	Non-melanoma skin cancer (basal-cell	Rate	2021	2.4118763	3.215135604	1.757246537
Prevalence	Ethiopia	Female	55+ years	Non-melanoma skin cancer (basal-cell	Rate	2021	1.4680616	1.942135984	1.056071774

Prevalence	Ethiopia	Both	55+ years	Non-melanoma skin cancer (basal-cell	Rate	2021	1.9481222	2.585118966	1.42476612
Prevalence	Eritrea	Male	55+ years	Non-melanoma skin cancer (basal-cell	Number	1990	1.3749149	1.955343656	0.943530161
Prevalence	Eritrea	Female	55+ years	Non-melanoma skin cancer (basal-cell	Number	1990	1.2934007	1.839917069	0.89428331
Prevalence	Eritrea	Both	55+ years	Non-melanoma skin cancer (basal-cell	Number	1990	2.6683157	3.739761101	1.860642916
Prevalence	Eritrea	Male	55+ years	Non-melanoma skin cancer (basal-cell	Percent	1990	1.81E-05	2.58E-05	1.24E-05
Prevalence	Eritrea	Female	55+ years	Non-melanoma skin cancer (basal-cell	Percent	1990	1.28E-05	1.82E-05	8.86E-06
Prevalence	Eritrea	Both	55+ years	Non-melanoma skin cancer (basal-cell	Percent	1990	1.51E-05	2.12E-05	1.05E-05
Prevalence	Eritrea	Male	55+ years	Non-melanoma skin cancer (basal-cell	Rate	1990	1.8136005	2.57922301	1.244576468
Prevalence	Eritrea	Female	55+ years	Non-melanoma skin cancer (basal-cell	Rate	1990	1.2817504	1.823343911	0.886228003
Prevalence	Eritrea	Both	55+ years	Non-melanoma skin cancer (basal-cell	Rate	1990	1.5099091	2.116203602	1.052874538
Prevalence	Eritrea	Male	55+ years	Non-melanoma skin cancer (basal-cell	Number	2021	3.4632246	4.883507689	2.435669531
Prevalence	Eritrea	Female	55+ years	Non-melanoma skin cancer (basal-cell	Number	2021	3.4887065	4.913196826	2.386318797
Prevalence	Eritrea	Both	55+ years	Non-melanoma skin cancer (basal-cell	Number	2021	6.9519311	9.550993725	4.944151742
Prevalence	Eritrea	Male	55+ years	Non-melanoma skin cancer (basal-cell	Percent	2021	1.93E-05	2.72E-05	1.36E-05
Prevalence	Eritrea	Female	55+ years	Non-melanoma skin cancer (basal-cell	Percent	2021	1.34E-05	1.88E-05	9.13E-06
Prevalence	Eritrea	Both	55+ years	Non-melanoma skin cancer (basal-cell	Percent	2021	1.58E-05	2.17E-05	1.12E-05
Prevalence	Eritrea	Male	55+ years	Non-melanoma skin cancer (basal-cell	Rate	2021	1.9315533	2.72369146	1.35845231
Prevalence	Eritrea	Female	55+ years	Non-melanoma skin cancer (basal-cell	Rate	2021	1.3350749	1.880205686	0.913207903
Prevalence	Eritrea	Both	55+ years	Non-melanoma skin cancer (basal-cell	Rate	2021	1.5778003	2.167679846	1.122117593
Prevalence	Mauritius	Male	55+ years	Non-melanoma skin cancer (basal-cell	Number	1990	0.3525947	0.495415576	0.241849822
Prevalence	Mauritius	Female	55+ years	Non-melanoma skin cancer (basal-cell	Number	1990	0.3826676	0.539455829	0.251520374
Prevalence	Mauritius	Both	55+ years	Non-melanoma skin cancer (basal-cell	Number	1990	0.7352622	1.048688275	0.49390332
Prevalence	Mauritius	Male	55+ years	Non-melanoma skin cancer (basal-cell	Percent	1990	6.37E-06	8.95E-06	4.37E-06
Prevalence	Mauritius	Female	55+ years	Non-melanoma skin cancer (basal-cell	Percent	1990	5.78E-06	8.14E-06	3.80E-06
Prevalence	Mauritius	Both	55+ years	Non-melanoma skin cancer (basal-cell	Percent	1990	6.05E-06	8.62E-06	4.06E-06
Prevalence	Mauritius	Male	55+ years	Non-melanoma skin cancer (basal-cell	Rate	1990	0.6368389	0.894794976	0.436817122
Prevalence	Mauritius	Female	55+ years	Non-melanoma skin cancer (basal-cell	Rate	1990	0.5774326	0.814020894	0.379535874
Prevalence	Mauritius	Both	55+ years	Non-melanoma skin cancer (basal-cell	Rate	1990	0.604473	0.862146551	0.406047301
Prevalence	Mauritius	Male	55+ years	Non-melanoma skin cancer (basal-cell	Number	2021	1.0292578	1.432545594	0.69745998
Prevalence	Mauritius	Female	55+ years	Non-melanoma skin cancer (basal-cell	Number	2021	1.0744924	1.534300518	0.706026413
Prevalence	Mauritius	Both	55+ years	Non-melanoma skin cancer (basal-cell	Number	2021	2.1037501	2.966438303	1.425327926
Prevalence	Mauritius	Male	55+ years	Non-melanoma skin cancer (basal-cell	Percent	2021	6.59E-06	9.18E-06	4.47E-06
Prevalence	Mauritius	Female	55+ years	Non-melanoma skin cancer (basal-cell	Percent	2021	5.90E-06	8.43E-06	3.88E-06
Prevalence	Mauritius	Both	55+ years	Non-melanoma skin cancer (basal-cell	Percent	2021	6.22E-06	8.77E-06	4.21E-06
Prevalence	Mauritius	Male	55+ years	Non-melanoma skin cancer (basal-cell	Rate	2021	0.6589441	0.917134209	0.446522896
Prevalence	Mauritius	Female	55+ years	Non-melanoma skin cancer (basal-cell	Rate	2021	0.5899464	0.842402433	0.387641378
Prevalence	Mauritius	Both	55+ years	Non-melanoma skin cancer (basal-cell	Rate	2021	0.6218006	0.876783388	0.421280917
Prevalence	Cabo Verde	Male	55+ years	Non-melanoma skin cancer (basal-cell	Number	1990	0.3829783	0.503560494	0.271675337
Prevalence	Cabo Verde	Female	55+ years	Non-melanoma skin cancer (basal-cell	Number	1990	0.3373616	0.460319026	0.233838546
Prevalence	Cabo Verde	Both	55+ years	Non-melanoma skin cancer (basal-cell	Number	1990	0.7203398	0.961931314	0.508408065
Prevalence	Cabo Verde	Male	55+ years	Non-melanoma skin cancer (basal-cell	Percent	1990	2.25E-05	2.96E-05	1.60E-05
Prevalence	Cabo Verde	Female	55+ years	Non-melanoma skin cancer (basal-cell	Percent	1990	1.50E-05	2.05E-05	1.04E-05
Prevalence	Cabo Verde	Both	55+ years	Non-melanoma skin cancer (basal-cell	Percent	1990	1.83E-05	2.44E-05	1.29E-05
Prevalence	Cabo Verde	Male	55+ years	Non-melanoma skin cancer (basal-cell	Rate	1990	2.2508202	2.959499859	1.59667633
Prevalence	Cabo Verde	Female	55+ years	Non-melanoma skin cancer (basal-cell	Rate	1990	1.5024562	2.05005328	1.041411393
Prevalence	Cabo Verde	Both	55+ years	Non-melanoma skin cancer (basal-cell	Rate	1990	1.8250749	2.437178363	1.288118098
Prevalence	Cabo Verde	Male	55+ years	Non-melanoma skin cancer (basal-cell	Number	2021	0.6624241	0.917562495	0.477477885
Prevalence	Cabo Verde	Female	55+ years	Non-melanoma skin cancer (basal-cell	Number	2021	0.6297634	0.855896663	0.431738992
Prevalence	Cabo Verde	Both	55+ years	Non-melanoma skin cancer (basal-cell	Number	2021	1.2921875	1.761990816	0.914750278
Prevalence	Cabo Verde	Male	55+ years	Non-melanoma skin cancer (basal-cell	Percent	2021	2.01E-05	2.78E-05	1.45E-05
Prevalence	Cabo Verde	Female	55+ years	Non-melanoma skin cancer (basal-cell	Percent	2021	1.44E-05	1.96E-05	9.91E-06
Prevalence	Cabo Verde	Both	55+ years	Non-melanoma skin cancer (basal-cell	Percent	2021	1.69E-05	2.30E-05	1.19E-05
Prevalence	Cabo Verde	Male	55+ years	Non-melanoma skin cancer (basal-cell	Rate	2021	2.0043506	2.776343659	1.444743769
Prevalence	Cabo Verde	Female	55+ years	Non-melanoma skin cancer (basal-cell	Rate	2021	1.4445149	1.96320648	0.990298038
Prevalence	Cabo Verde	Both	55+ years	Non-melanoma skin cancer (basal-cell	Rate	2021	1.6859122	2.298862977	1.193471344
Prevalence	Nigeria	Male	55+ years	Non-melanoma skin cancer (basal-cell	Number	1990	63.547023	86.7476713	46.36836807

Prevalence	Nigeria	Female	55+ years	Non-melanoma skin cancer (basal-cell	Number	1990	35.023139	48.90504897	24.21197416
Prevalence	Nigeria	Both	55+ years	Non-melanoma skin cancer (basal-cell	Number	1990	98.570162	134.3712957	71.22975509
Prevalence	Nigeria	Male	55+ years	Non-melanoma skin cancer (basal-cell	Percent	1990	1.64E-05	2.24E-05	1.20E-05
Prevalence	Nigeria	Female	55+ years	Non-melanoma skin cancer (basal-cell	Percent	1990	1.03E-05	1.43E-05	7.10E-06
Prevalence	Nigeria	Both	55+ years	Non-melanoma skin cancer (basal-cell	Percent	1990	1.35E-05	1.84E-05	9.78E-06
Prevalence	Nigeria	Male	55+ years	Non-melanoma skin cancer (basal-cell	Rate	1990	1.6402793	2.239135709	1.196862892
Prevalence	Nigeria	Female	55+ years	Non-melanoma skin cancer (basal-cell	Rate	1990	1.0268096	1.433799974	0.709847524
Prevalence	Nigeria	Both	55+ years	Non-melanoma skin cancer (basal-cell	Rate	1990	1.3530512	1.844485537	0.977755349
Prevalence	Nigeria	Male	55+ years	Non-melanoma skin cancer (basal-cell	Number	2021	112.0672	153.2745604	81.29995862
Prevalence	Nigeria	Female	55+ years	Non-melanoma skin cancer (basal-cell	Number	2021	75.714675	107.9158082	50.72326934
Prevalence	Nigeria	Both	55+ years	Non-melanoma skin cancer (basal-cell	Number	2021	187.78187	261.3552279	133.256271
Prevalence	Nigeria	Male	55+ years	Non-melanoma skin cancer (basal-cell	Percent	2021	1.68E-05	2.29E-05	1.22E-05
Prevalence	Nigeria	Female	55+ years	Non-melanoma skin cancer (basal-cell	Percent	2021	9.11E-06	1.30E-05	6.11E-06
Prevalence	Nigeria	Both	55+ years	Non-melanoma skin cancer (basal-cell	Percent	2021	1.25E-05	1.74E-05	8.89E-06
Prevalence	Nigeria	Male	55+ years	Non-melanoma skin cancer (basal-cell	Rate	2021	1.6743625	2.290029428	1.214678399
Prevalence	Nigeria	Female	55+ years	Non-melanoma skin cancer (basal-cell	Rate	2021	0.9113042	1.298878095	0.610506881
Prevalence	Nigeria	Both	55+ years	Non-melanoma skin cancer (basal-cell	Rate	2021	1.2517529	1.742192524	0.888285576
Prevalence	Sao Tome a	Male	55+ years	Non-melanoma skin cancer (basal-cell	Number	1990	0.1040368	0.143051148	0.07309746
Prevalence	Sao Tome a	Female	55+ years	Non-melanoma skin cancer (basal-cell	Number	1990	0.0726192	0.100991582	0.050413703
Prevalence	Sao Tome a	Both	55+ years	Non-melanoma skin cancer (basal-cell	Number	1990	0.176656	0.23883338	0.128094208
Prevalence	Sao Tome a	Male	55+ years	Non-melanoma skin cancer (basal-cell	Percent	1990	1.97E-05	2.71E-05	1.39E-05
Prevalence	Sao Tome a	Female	55+ years	Non-melanoma skin cancer (basal-cell	Percent	1990	1.23E-05	1.71E-05	8.51E-06
Prevalence	Sao Tome a	Both	55+ years	Non-melanoma skin cancer (basal-cell	Percent	1990	1.58E-05	2.13E-05	1.14E-05
Prevalence	Sao Tome a	Male	55+ years	Non-melanoma skin cancer (basal-cell	Rate	1990	1.9722062	2.711794236	1.385695066
Prevalence	Sao Tome a	Female	55+ years	Non-melanoma skin cancer (basal-cell	Rate	1990	1.2261364	1.705189676	0.851208825
Prevalence	Sao Tome a	Both	55+ years	Non-melanoma skin cancer (basal-cell	Rate	1990	1.5776024	2.132869626	1.143928223
Prevalence	Sao Tome a	Male	55+ years	Non-melanoma skin cancer (basal-cell	Number	2021	0.1663145	0.22576675	0.116251969
Prevalence	Sao Tome a	Female	55+ years	Non-melanoma skin cancer (basal-cell	Number	2021	0.1098058	0.154465062	0.078966616
Prevalence	Sao Tome a	Both	55+ years	Non-melanoma skin cancer (basal-cell	Number	2021	0.2761203	0.377000007	0.197765014
Prevalence	Sao Tome a	Male	55+ years	Non-melanoma skin cancer (basal-cell	Percent	2021	1.90E-05	2.58E-05	1.33E-05
Prevalence	Sao Tome a	Female	55+ years	Non-melanoma skin cancer (basal-cell	Percent	2021	1.15E-05	1.62E-05	8.29E-06
Prevalence	Sao Tome a	Both	55+ years	Non-melanoma skin cancer (basal-cell	Percent	2021	1.51E-05	2.06E-05	1.08E-05
Prevalence	Sao Tome a	Male	55+ years	Non-melanoma skin cancer (basal-cell	Rate	2021	1.9001002	2.579327384	1.328149022
Prevalence	Sao Tome a	Female	55+ years	Non-melanoma skin cancer (basal-cell	Rate	2021	1.1520992	1.620670515	0.828529537
Prevalence	Sao Tome a	Both	55+ years	Non-melanoma skin cancer (basal-cell	Rate	2021	1.5101855	2.061927126	1.08163671
Prevalence	Togo	Male	55+ years	Non-melanoma skin cancer (basal-cell	Number	1990	1.6729185	2.308480298	1.193583282
Prevalence	Togo	Female	55+ years	Non-melanoma skin cancer (basal-cell	Number	1990	1.1031019	1.495005265	0.749544902
Prevalence	Togo	Both	55+ years	Non-melanoma skin cancer (basal-cell	Number	1990	2.7760204	3.771469959	1.989467386
Prevalence	Togo	Male	55+ years	Non-melanoma skin cancer (basal-cell	Percent	1990	1.78E-05	2.45E-05	1.27E-05
Prevalence	Togo	Female	55+ years	Non-melanoma skin cancer (basal-cell	Percent	1990	1.09E-05	1.48E-05	7.42E-06
Prevalence	Togo	Both	55+ years	Non-melanoma skin cancer (basal-cell	Percent	1990	1.42E-05	1.93E-05	1.02E-05
Prevalence	Togo	Male	55+ years	Non-melanoma skin cancer (basal-cell	Rate	1990	1.7788234	2.454619816	1.269143678
Prevalence	Togo	Female	55+ years	Non-melanoma skin cancer (basal-cell	Rate	1990	1.0918696	1.479782453	0.741912701
Prevalence	Togo	Both	55+ years	Non-melanoma skin cancer (basal-cell	Rate	1990	1.4230524	1.933342894	1.019847082
Prevalence	Togo	Male	55+ years	Non-melanoma skin cancer (basal-cell	Number	2021	4.5390869	6.316362347	3.171894849
Prevalence	Togo	Female	55+ years	Non-melanoma skin cancer (basal-cell	Number	2021	3.8190942	5.373977341	2.614801633
Prevalence	Togo	Both	55+ years	Non-melanoma skin cancer (basal-cell	Number	2021	8.3581811	11.5845275	5.856904262
Prevalence	Togo	Male	55+ years	Non-melanoma skin cancer (basal-cell	Percent	2021	1.66E-05	2.32E-05	1.16E-05
Prevalence	Togo	Female	55+ years	Non-melanoma skin cancer (basal-cell	Percent	2021	1.09E-05	1.53E-05	7.44E-06
Prevalence	Togo	Both	55+ years	Non-melanoma skin cancer (basal-cell	Percent	2021	1.34E-05	1.86E-05	9.38E-06
Prevalence	Togo	Male	55+ years	Non-melanoma skin cancer (basal-cell	Rate	2021	1.6628246	2.313902125	1.161974856
Prevalence	Togo	Female	55+ years	Non-melanoma skin cancer (basal-cell	Rate	2021	1.085855	1.527943551	0.743447365
Prevalence	Togo	Both	55+ years	Non-melanoma skin cancer (basal-cell	Rate	2021	1.3379778	1.854451437	0.937573373
Prevalence	Côte d'Ivo	Male	55+ years	Non-melanoma skin cancer (basal-cell	Number	1990	4.1788742	5.711560379	2.924627494
Prevalence	Côte d'Ivo	Female	55+ years	Non-melanoma skin cancer (basal-cell	Number	1990	2.0066452	2.957906476	1.330203772
Prevalence	Côte d'Ivo	Both	55+ years	Non-melanoma skin cancer (basal-cell	Number	1990	6.1855194	8.61642771	4.307558267

Prevalence	Côte d'Ivo	Male	55+ years	Non-melanoma skin cancer (basal-cell	Percent	1990	1.20E-05	1.64E-05	8.39E-06
Prevalence	Côte d'Ivo	Female	55+ years	Non-melanoma skin cancer (basal-cell	Percent	1990	6.86E-06	1.01E-05	4.55E-06
Prevalence	Côte d'Ivo	Both	55+ years	Non-melanoma skin cancer (basal-cell	Percent	1990	9.65E-06	1.34E-05	6.72E-06
Prevalence	Côte d'Ivo	Male	55+ years	Non-melanoma skin cancer (basal-cell	Rate	1990	1.1990315	1.638800433	0.839154362
Prevalence	Côte d'Ivo	Female	55+ years	Non-melanoma skin cancer (basal-cell	Rate	1990	0.6862404	1.011556452	0.4549083
Prevalence	Côte d'Ivo	Both	55+ years	Non-melanoma skin cancer (basal-cell	Rate	1990	0.9650817	1.344358668	0.672077047
Prevalence	Côte d'Ivo	Male	55+ years	Non-melanoma skin cancer (basal-cell	Number	2021	11.513331	16.26975818	7.890145882
Prevalence	Côte d'Ivo	Female	55+ years	Non-melanoma skin cancer (basal-cell	Number	2021	5.7065791	8.586046967	3.846465488
Prevalence	Côte d'Ivo	Both	55+ years	Non-melanoma skin cancer (basal-cell	Number	2021	17.219911	24.57205315	11.8888718
Prevalence	Côte d'Ivo	Male	55+ years	Non-melanoma skin cancer (basal-cell	Percent	2021	1.22E-05	1.73E-05	8.38E-06
Prevalence	Côte d'Ivo	Female	55+ years	Non-melanoma skin cancer (basal-cell	Percent	2021	6.71E-06	1.01E-05	4.52E-06
Prevalence	Côte d'Ivo	Both	55+ years	Non-melanoma skin cancer (basal-cell	Percent	2021	9.61E-06	1.37E-05	6.63E-06
Prevalence	Côte d'Ivo	Male	55+ years	Non-melanoma skin cancer (basal-cell	Rate	2021	1.2219403	1.726752423	0.837402029
Prevalence	Côte d'Ivo	Female	55+ years	Non-melanoma skin cancer (basal-cell	Rate	2021	0.6706006	1.008977198	0.452011966
Prevalence	Côte d'Ivo	Both	55+ years	Non-melanoma skin cancer (basal-cell	Rate	2021	0.9602988	1.370304012	0.663003967
Prevalence	South Afri	Male	55+ years	Non-melanoma skin cancer (basal-cell	Number	1990	146.5636	191.5693453	107.8983517
Prevalence	South Afri	Female	55+ years	Non-melanoma skin cancer (basal-cell	Number	1990	130.26415	170.8820261	96.06671299
Prevalence	South Afri	Both	55+ years	Non-melanoma skin cancer (basal-cell	Number	1990	276.82775	361.2393273	202.4970582
Prevalence	South Afri	Male	55+ years	Non-melanoma skin cancer (basal-cell	Percent	1990	0.0001037	0.000135529	7.63E-05
Prevalence	South Afri	Female	55+ years	Non-melanoma skin cancer (basal-cell	Percent	1990	6.64E-05	8.71E-05	4.90E-05
Prevalence	South Afri	Both	55+ years	Non-melanoma skin cancer (basal-cell	Percent	1990	8.20E-05	0.000107028	6.00E-05
Prevalence	South Afri	Male	55+ years	Non-melanoma skin cancer (basal-cell	Rate	1990	10.366379	13.54961482	7.631602557
Prevalence	South Afri	Female	55+ years	Non-melanoma skin cancer (basal-cell	Rate	1990	6.6405824	8.711193172	4.897271604
Prevalence	South Afri	Both	55+ years	Non-melanoma skin cancer (basal-cell	Rate	1990	8.2011531	10.70188594	5.999071135
Prevalence	South Afri	Male	55+ years	Non-melanoma skin cancer (basal-cell	Number	2021	402.86762	529.1979076	299.3686791
Prevalence	South Afri	Female	55+ years	Non-melanoma skin cancer (basal-cell	Number	2021	376.68097	489.0770314	282.8728313
Prevalence	South Afri	Both	55+ years	Non-melanoma skin cancer (basal-cell	Number	2021	779.5486	1020.671897	586.4533481
Prevalence	South Afri	Male	55+ years	Non-melanoma skin cancer (basal-cell	Percent	2021	0.0001224	0.000160843	9.10E-05
Prevalence	South Afri	Female	55+ years	Non-melanoma skin cancer (basal-cell	Percent	2021	8.19E-05	0.000106389	6.15E-05
Prevalence	South Afri	Both	55+ years	Non-melanoma skin cancer (basal-cell	Percent	2021	9.88E-05	0.000129405	7.44E-05
Prevalence	South Afri	Male	55+ years	Non-melanoma skin cancer (basal-cell	Rate	2021	12.240504	16.0788524	9.095850032
Prevalence	South Afri	Female	55+ years	Non-melanoma skin cancer (basal-cell	Rate	2021	8.1935513	10.63838635	6.15303986
Prevalence	South Afri	Both	55+ years	Non-melanoma skin cancer (basal-cell	Rate	2021	9.8820233	12.93864616	7.434232669
Prevalence	Burkina Fa	Male	55+ years	Non-melanoma skin cancer (basal-cell	Number	1990	6.2446476	8.674157145	4.498354819
Prevalence	Burkina Fa	Female	55+ years	Non-melanoma skin cancer (basal-cell	Number	1990	4.016861	5.648117495	2.846334962
Prevalence	Burkina Fa	Both	55+ years	Non-melanoma skin cancer (basal-cell	Number	1990	10.261509	14.34842038	7.388622188
Prevalence	Burkina Fa	Male	55+ years	Non-melanoma skin cancer (basal-cell	Percent	1990	1.78E-05	2.47E-05	1.28E-05
Prevalence	Burkina Fa	Female	55+ years	Non-melanoma skin cancer (basal-cell	Percent	1990	1.08E-05	1.52E-05	7.64E-06
Prevalence	Burkina Fa	Both	55+ years	Non-melanoma skin cancer (basal-cell	Percent	1990	1.42E-05	1.98E-05	1.02E-05
Prevalence	Burkina Fa	Male	55+ years	Non-melanoma skin cancer (basal-cell	Rate	1990	1.7803073	2.472944171	1.282450864
Prevalence	Burkina Fa	Female	55+ years	Non-melanoma skin cancer (basal-cell	Rate	1990	1.0784215	1.516370955	0.764166055
Prevalence	Burkina Fa	Both	55+ years	Non-melanoma skin cancer (basal-cell	Rate	1990	1.4188281	1.983913164	1.021602688
Prevalence	Burkina Fa	Male	55+ years	Non-melanoma skin cancer (basal-cell	Number	2021	12.67839	17.34128683	8.892012034
Prevalence	Burkina Fa	Female	55+ years	Non-melanoma skin cancer (basal-cell	Number	2021	8.6087136	11.84538484	5.939998562
Prevalence	Burkina Fa	Both	55+ years	Non-melanoma skin cancer (basal-cell	Number	2021	21.287104	29.20776572	15.01612365
Prevalence	Burkina Fa	Male	55+ years	Non-melanoma skin cancer (basal-cell	Percent	2021	1.78E-05	2.44E-05	1.25E-05
Prevalence	Burkina Fa	Female	55+ years	Non-melanoma skin cancer (basal-cell	Percent	2021	1.09E-05	1.50E-05	7.51E-06
Prevalence	Burkina Fa	Both	55+ years	Non-melanoma skin cancer (basal-cell	Percent	2021	1.42E-05	1.94E-05	9.99E-06
Prevalence	Burkina Fa	Male	55+ years	Non-melanoma skin cancer (basal-cell	Rate	2021	1.7796928	2.434233578	1.248190776
Prevalence	Burkina Fa	Female	55+ years	Non-melanoma skin cancer (basal-cell	Rate	2021	1.0883022	1.49747795	0.750926794
Prevalence	Burkina Fa	Both	55+ years	Non-melanoma skin cancer (basal-cell	Rate	2021	1.4159173	1.942762155	0.998801381
Prevalence	Cameroon	Male	55+ years	Non-melanoma skin cancer (basal-cell	Number	1990	6.4413605	8.860544231	4.482155533
Prevalence	Cameroon	Female	55+ years	Non-melanoma skin cancer (basal-cell	Number	1990	4.0288914	5.677708733	2.803040477
Prevalence	Cameroon	Both	55+ years	Non-melanoma skin cancer (basal-cell	Number	1990	10.470252	14.57369294	7.32271858
Prevalence	Cameroon	Male	55+ years	Non-melanoma skin cancer (basal-cell	Percent	1990	1.76E-05	2.43E-05	1.23E-05
Prevalence	Cameroon	Female	55+ years	Non-melanoma skin cancer (basal-cell	Percent	1990	1.07E-05	1.51E-05	7.47E-06

Prevalence	Cameroon	Both	55+ years	Non-melanoma skin cancer (basal-cell	Percent	1990	1.41E-05	1.97E-05	9.89E-06
Prevalence	Cameroon	Male	55+ years	Non-melanoma skin cancer (basal-cell	Rate	1990	1.7640243	2.42653947	1.227478474
Prevalence	Cameroon	Female	55+ years	Non-melanoma skin cancer (basal-cell	Rate	1990	1.0735332	1.512875	0.746894577
Prevalence	Cameroon	Both	55+ years	Non-melanoma skin cancer (basal-cell	Rate	1990	1.4140503	1.968236728	0.988963039
Prevalence	Cameroon	Male	55+ years	Non-melanoma skin cancer (basal-cell	Number	2021	17.211118	23.8103924	12.04758501
Prevalence	Cameroon	Female	55+ years	Non-melanoma skin cancer (basal-cell	Number	2021	11.225802	15.63681832	7.836254652
Prevalence	Cameroon	Both	55+ years	Non-melanoma skin cancer (basal-cell	Number	2021	28.43692	39.24670242	20.18725558
Prevalence	Cameroon	Male	55+ years	Non-melanoma skin cancer (basal-cell	Percent	2021	1.74E-05	2.41E-05	1.22E-05
Prevalence	Cameroon	Female	55+ years	Non-melanoma skin cancer (basal-cell	Percent	2021	1.07E-05	1.49E-05	7.48E-06
Prevalence	Cameroon	Both	55+ years	Non-melanoma skin cancer (basal-cell	Percent	2021	1.40E-05	1.93E-05	9.92E-06
Prevalence	Cameroon	Male	55+ years	Non-melanoma skin cancer (basal-cell	Rate	2021	1.742245	2.410275578	1.219551506
Prevalence	Cameroon	Female	55+ years	Non-melanoma skin cancer (basal-cell	Rate	2021	1.0715653	1.492621355	0.748014129
Prevalence	Cameroon	Both	55+ years	Non-melanoma skin cancer (basal-cell	Rate	2021	1.3970635	1.92813202	0.991769792
Prevalence	Guinea	Male	55+ years	Non-melanoma skin cancer (basal-cell	Number	1990	4.6942887	6.426937474	3.387845216
Prevalence	Guinea	Female	55+ years	Non-melanoma skin cancer (basal-cell	Number	1990	2.8973297	3.919587505	2.006375516
Prevalence	Guinea	Both	55+ years	Non-melanoma skin cancer (basal-cell	Number	1990	7.5916184	10.23030188	5.545316247
Prevalence	Guinea	Male	55+ years	Non-melanoma skin cancer (basal-cell	Percent	1990	1.67E-05	2.29E-05	1.21E-05
Prevalence	Guinea	Female	55+ years	Non-melanoma skin cancer (basal-cell	Percent	1990	1.06E-05	1.44E-05	7.35E-06
Prevalence	Guinea	Both	55+ years	Non-melanoma skin cancer (basal-cell	Percent	1990	1.37E-05	1.85E-05	1.00E-05
Prevalence	Guinea	Male	55+ years	Non-melanoma skin cancer (basal-cell	Rate	1990	1.6748003	2.292964363	1.20869518
Prevalence	Guinea	Female	55+ years	Non-melanoma skin cancer (basal-cell	Rate	1990	1.0608851	1.435194616	0.734653669
Prevalence	Guinea	Both	55+ years	Non-melanoma skin cancer (basal-cell	Rate	1990	1.3718278	1.848645686	1.002054982
Prevalence	Guinea	Male	55+ years	Non-melanoma skin cancer (basal-cell	Number	2021	7.7000636	11.01518802	5.401744745
Prevalence	Guinea	Female	55+ years	Non-melanoma skin cancer (basal-cell	Number	2021	4.5503222	6.156853758	3.204060319
Prevalence	Guinea	Both	55+ years	Non-melanoma skin cancer (basal-cell	Number	2021	12.250386	17.12444361	8.637096402
Prevalence	Guinea	Male	55+ years	Non-melanoma skin cancer (basal-cell	Percent	2021	1.63E-05	2.34E-05	1.15E-05
Prevalence	Guinea	Female	55+ years	Non-melanoma skin cancer (basal-cell	Percent	2021	1.03E-05	1.39E-05	7.25E-06
Prevalence	Guinea	Both	55+ years	Non-melanoma skin cancer (basal-cell	Percent	2021	1.34E-05	1.87E-05	9.46E-06
Prevalence	Guinea	Male	55+ years	Non-melanoma skin cancer (basal-cell	Rate	2021	1.6321184	2.334797776	1.1449629
Prevalence	Guinea	Female	55+ years	Non-melanoma skin cancer (basal-cell	Rate	2021	1.0300093	1.393663193	0.725269937
Prevalence	Guinea	Both	55+ years	Non-melanoma skin cancer (basal-cell	Rate	2021	1.3409529	1.874477461	0.945434661
Prevalence	Benin	Male	55+ years	Non-melanoma skin cancer (basal-cell	Number	1990	3.0506722	4.211542326	2.189910385
Prevalence	Benin	Female	55+ years	Non-melanoma skin cancer (basal-cell	Number	1990	1.9339439	2.639851906	1.33392537
Prevalence	Benin	Both	55+ years	Non-melanoma skin cancer (basal-cell	Number	1990	4.984616	6.850072381	3.519937878
Prevalence	Benin	Male	55+ years	Non-melanoma skin cancer (basal-cell	Percent	1990	1.97E-05	2.72E-05	1.41E-05
Prevalence	Benin	Female	55+ years	Non-melanoma skin cancer (basal-cell	Percent	1990	1.17E-05	1.60E-05	8.08E-06
Prevalence	Benin	Both	55+ years	Non-melanoma skin cancer (basal-cell	Percent	1990	1.56E-05	2.14E-05	1.10E-05
Prevalence	Benin	Male	55+ years	Non-melanoma skin cancer (basal-cell	Rate	1990	1.9697755	2.719332795	1.413993893
Prevalence	Benin	Female	55+ years	Non-melanoma skin cancer (basal-cell	Rate	1990	1.1717402	1.599436647	0.808200307
Prevalence	Benin	Both	55+ years	Non-melanoma skin cancer (basal-cell	Rate	1990	1.5580676	2.141163091	1.100245464
Prevalence	Benin	Male	55+ years	Non-melanoma skin cancer (basal-cell	Number	2021	7.0878248	10.02261868	5.018492392
Prevalence	Benin	Female	55+ years	Non-melanoma skin cancer (basal-cell	Number	2021	5.0091641	6.804164721	3.444656764
Prevalence	Benin	Both	55+ years	Non-melanoma skin cancer (basal-cell	Number	2021	12.096989	16.48674953	8.586615262
Prevalence	Benin	Male	55+ years	Non-melanoma skin cancer (basal-cell	Percent	2021	1.82E-05	2.57E-05	1.29E-05
Prevalence	Benin	Female	55+ years	Non-melanoma skin cancer (basal-cell	Percent	2021	1.15E-05	1.56E-05	7.88E-06
Prevalence	Benin	Both	55+ years	Non-melanoma skin cancer (basal-cell	Percent	2021	1.46E-05	1.99E-05	1.04E-05
Prevalence	Benin	Male	55+ years	Non-melanoma skin cancer (basal-cell	Rate	2021	1.8189504	2.572107384	1.287897081
Prevalence	Benin	Female	55+ years	Non-melanoma skin cancer (basal-cell	Rate	2021	1.1452234	1.555606621	0.787536911
Prevalence	Benin	Both	55+ years	Non-melanoma skin cancer (basal-cell	Rate	2021	1.4626462	1.993411876	1.038207125
Prevalence	Guinea-Bis	Male	55+ years	Non-melanoma skin cancer (basal-cell	Number	1990	0.5301971	0.731840266	0.363688627
Prevalence	Guinea-Bis	Female	55+ years	Non-melanoma skin cancer (basal-cell	Number	1990	0.3208397	0.44773371	0.222937166
Prevalence	Guinea-Bis	Both	55+ years	Non-melanoma skin cancer (basal-cell	Number	1990	0.8510368	1.156421525	0.584967312
Prevalence	Guinea-Bis	Male	55+ years	Non-melanoma skin cancer (basal-cell	Percent	1990	1.68E-05	2.31E-05	1.15E-05
Prevalence	Guinea-Bis	Female	55+ years	Non-melanoma skin cancer (basal-cell	Percent	1990	9.96E-06	1.39E-05	6.92E-06
Prevalence	Guinea-Bis	Both	55+ years	Non-melanoma skin cancer (basal-cell	Percent	1990	1.33E-05	1.81E-05	9.16E-06
Prevalence	Guinea-Bis	Male	55+ years	Non-melanoma skin cancer (basal-cell	Rate	1990	1.67607	2.313508677	1.149700055

Prevalence	Guinea-Bis	Female	55+ years	Non-melanoma skin cancer (basal-cell	Rate	1990	0.9964061	1.390490403	0.692357942
Prevalence	Guinea-Bis	Both	55+ years	Non-melanoma skin cancer (basal-cell	Rate	1990	1.3332229	1.811634546	0.916401994
Prevalence	Guinea-Bis	Male	55+ years	Non-melanoma skin cancer (basal-cell	Number	2021	0.8350757	1.174563057	0.584624277
Prevalence	Guinea-Bis	Female	55+ years	Non-melanoma skin cancer (basal-cell	Number	2021	0.6024131	0.84327463	0.401617353
Prevalence	Guinea-Bis	Both	55+ years	Non-melanoma skin cancer (basal-cell	Number	2021	1.4374888	1.989442391	1.014297646
Prevalence	Guinea-Bis	Male	55+ years	Non-melanoma skin cancer (basal-cell	Percent	2021	1.57E-05	2.21E-05	1.10E-05
Prevalence	Guinea-Bis	Female	55+ years	Non-melanoma skin cancer (basal-cell	Percent	2021	9.75E-06	1.37E-05	6.50E-06
Prevalence	Guinea-Bis	Both	55+ years	Non-melanoma skin cancer (basal-cell	Percent	2021	1.25E-05	1.73E-05	8.83E-06
Prevalence	Guinea-Bis	Male	55+ years	Non-melanoma skin cancer (basal-cell	Rate	2021	1.5724805	2.21174863	1.100870605
Prevalence	Guinea-Bis	Female	55+ years	Non-melanoma skin cancer (basal-cell	Rate	2021	0.9753355	1.365301882	0.650237666
Prevalence	Guinea-Bis	Both	55+ years	Non-melanoma skin cancer (basal-cell	Rate	2021	1.2514013	1.731902709	0.882993571
Prevalence	Ghana	Male	55+ years	Non-melanoma skin cancer (basal-cell	Number	1990	8.8367229	12.06285069	6.201598858
Prevalence	Ghana	Female	55+ years	Non-melanoma skin cancer (basal-cell	Number	1990	5.8088028	8.500624035	4.067460673
Prevalence	Ghana	Both	55+ years	Non-melanoma skin cancer (basal-cell	Number	1990	14.645526	20.31888643	10.40704809
Prevalence	Ghana	Male	55+ years	Non-melanoma skin cancer (basal-cell	Percent	1990	1.78E-05	2.43E-05	1.25E-05
Prevalence	Ghana	Female	55+ years	Non-melanoma skin cancer (basal-cell	Percent	1990	1.11E-05	1.62E-05	7.74E-06
Prevalence	Ghana	Both	55+ years	Non-melanoma skin cancer (basal-cell	Percent	1990	1.43E-05	1.99E-05	1.02E-05
Prevalence	Ghana	Male	55+ years	Non-melanoma skin cancer (basal-cell	Rate	1990	1.7824561	2.433198612	1.25092502
Prevalence	Ghana	Female	55+ years	Non-melanoma skin cancer (basal-cell	Rate	1990	1.1055102	1.617807822	0.774104309
Prevalence	Ghana	Both	55+ years	Non-melanoma skin cancer (basal-cell	Rate	1990	1.4341459	1.989703075	1.019097955
Prevalence	Ghana	Male	55+ years	Non-melanoma skin cancer (basal-cell	Number	2021	21.177524	29.47710731	14.93594436
Prevalence	Ghana	Female	55+ years	Non-melanoma skin cancer (basal-cell	Number	2021	16.898256	23.70630138	11.67631907
Prevalence	Ghana	Both	55+ years	Non-melanoma skin cancer (basal-cell	Number	2021	38.07578	52.59881967	26.85356416
Prevalence	Ghana	Male	55+ years	Non-melanoma skin cancer (basal-cell	Percent	2021	1.78E-05	2.48E-05	1.26E-05
Prevalence	Ghana	Female	55+ years	Non-melanoma skin cancer (basal-cell	Percent	2021	1.10E-05	1.54E-05	7.59E-06
Prevalence	Ghana	Both	55+ years	Non-melanoma skin cancer (basal-cell	Percent	2021	1.40E-05	1.93E-05	9.85E-06
Prevalence	Ghana	Male	55+ years	Non-melanoma skin cancer (basal-cell	Rate	2021	1.7817554	2.480034787	1.256624716
Prevalence	Ghana	Female	55+ years	Non-melanoma skin cancer (basal-cell	Rate	2021	1.0982916	1.540776222	0.75889505
Prevalence	Ghana	Both	55+ years	Non-melanoma skin cancer (basal-cell	Rate	2021	1.396164	1.928695278	0.984667388
Prevalence	Eswatini	Male	55+ years	Non-melanoma skin cancer (basal-cell	Number	1990	1.5956735	2.118006589	1.156126464
Prevalence	Eswatini	Female	55+ years	Non-melanoma skin cancer (basal-cell	Number	1990	1.2980661	1.738597846	0.948483896
Prevalence	Eswatini	Both	55+ years	Non-melanoma skin cancer (basal-cell	Number	1990	2.8937396	3.806056412	2.156855659
Prevalence	Eswatini	Male	55+ years	Non-melanoma skin cancer (basal-cell	Percent	1990	7.95E-05	0.000105488	5.76E-05
Prevalence	Eswatini	Female	55+ years	Non-melanoma skin cancer (basal-cell	Percent	1990	5.18E-05	6.94E-05	3.78E-05
Prevalence	Eswatini	Both	55+ years	Non-melanoma skin cancer (basal-cell	Percent	1990	6.41E-05	8.43E-05	4.78E-05
Prevalence	Eswatini	Male	55+ years	Non-melanoma skin cancer (basal-cell	Rate	1990	7.9426017	10.54255957	5.754718695
Prevalence	Eswatini	Female	55+ years	Non-melanoma skin cancer (basal-cell	Rate	1990	5.17886	6.936437793	3.784141088
Prevalence	Eswatini	Both	55+ years	Non-melanoma skin cancer (basal-cell	Rate	1990	6.4084918	8.428913674	4.776584524
Prevalence	Eswatini	Male	55+ years	Non-melanoma skin cancer (basal-cell	Number	2021	2.7387862	3.682046975	2.009638952
Prevalence	Eswatini	Female	55+ years	Non-melanoma skin cancer (basal-cell	Number	2021	2.8010503	3.750458945	2.002121677
Prevalence	Eswatini	Both	55+ years	Non-melanoma skin cancer (basal-cell	Number	2021	5.5398365	7.371398549	4.018921686
Prevalence	Eswatini	Male	55+ years	Non-melanoma skin cancer (basal-cell	Percent	2021	7.77E-05	0.000104456	5.70E-05
Prevalence	Eswatini	Female	55+ years	Non-melanoma skin cancer (basal-cell	Percent	2021	5.07E-05	6.79E-05	3.62E-05
Prevalence	Eswatini	Both	55+ years	Non-melanoma skin cancer (basal-cell	Percent	2021	6.12E-05	8.14E-05	4.44E-05
Prevalence	Eswatini	Male	55+ years	Non-melanoma skin cancer (basal-cell	Rate	2021	7.7674483	10.44262213	5.699519951
Prevalence	Eswatini	Female	55+ years	Non-melanoma skin cancer (basal-cell	Rate	2021	5.0670595	6.784526026	3.621809177
Prevalence	Eswatini	Both	55+ years	Non-melanoma skin cancer (basal-cell	Rate	2021	6.1187028	8.141647706	4.438865204
Prevalence	Sierra Leo	Male	55+ years	Non-melanoma skin cancer (basal-cell	Number	1990	3.3210546	4.578374845	2.40819329
Prevalence	Sierra Leo	Female	55+ years	Non-melanoma skin cancer (basal-cell	Number	1990	1.7766342	2.481762141	1.236805178
Prevalence	Sierra Leo	Both	55+ years	Non-melanoma skin cancer (basal-cell	Number	1990	5.0976887	7.076971495	3.610580106
Prevalence	Sierra Leo	Male	55+ years	Non-melanoma skin cancer (basal-cell	Percent	1990	1.93E-05	2.66E-05	1.40E-05
Prevalence	Sierra Leo	Female	55+ years	Non-melanoma skin cancer (basal-cell	Percent	1990	1.12E-05	1.56E-05	7.77E-06
Prevalence	Sierra Leo	Both	55+ years	Non-melanoma skin cancer (basal-cell	Percent	1990	1.54E-05	2.14E-05	1.09E-05
Prevalence	Sierra Leo	Male	55+ years	Non-melanoma skin cancer (basal-cell	Rate	1990	1.9272757	2.656924255	1.397523658
Prevalence	Sierra Leo	Female	55+ years	Non-melanoma skin cancer (basal-cell	Rate	1990	1.1164111	1.559503333	0.777190435
Prevalence	Sierra Leo	Both	55+ years	Non-melanoma skin cancer (basal-cell	Rate	1990	1.5379657	2.135112631	1.089307085

Prevalence	Sierra Leo	Male	55+ years	Non-melanoma skin cancer (basal-cell	Number	2021	5.5844428	7.599481693	3.966705955
Prevalence	Sierra Leo	Female	55+ years	Non-melanoma skin cancer (basal-cell	Number	2021	3.2377379	4.582512033	2.210845644
Prevalence	Sierra Leo	Both	55+ years	Non-melanoma skin cancer (basal-cell	Number	2021	8.8221807	12.03752313	6.208383858
Prevalence	Sierra Leo	Male	55+ years	Non-melanoma skin cancer (basal-cell	Percent	2021	1.81E-05	2.46E-05	1.29E-05
Prevalence	Sierra Leo	Female	55+ years	Non-melanoma skin cancer (basal-cell	Percent	2021	1.10E-05	1.55E-05	7.48E-06
Prevalence	Sierra Leo	Both	55+ years	Non-melanoma skin cancer (basal-cell	Percent	2021	1.46E-05	1.99E-05	1.03E-05
Prevalence	Sierra Leo	Male	55+ years	Non-melanoma skin cancer (basal-cell	Rate	2021	1.8098352	2.462879419	1.285550627
Prevalence	Sierra Leo	Female	55+ years	Non-melanoma skin cancer (basal-cell	Rate	2021	1.0955968	1.550646038	0.748113483
Prevalence	Sierra Leo	Both	55+ years	Non-melanoma skin cancer (basal-cell	Rate	2021	1.4604238	1.992691546	1.027735847
Prevalence	Zimbabwe	Male	55+ years	Non-melanoma skin cancer (basal-cell	Number	1990	22.772051	29.19930619	17.06728619
Prevalence	Zimbabwe	Female	55+ years	Non-melanoma skin cancer (basal-cell	Number	1990	15.736983	21.29448289	11.72840924
Prevalence	Zimbabwe	Both	55+ years	Non-melanoma skin cancer (basal-cell	Number	1990	38.509034	50.699737	29.24331696
Prevalence	Zimbabwe	Male	55+ years	Non-melanoma skin cancer (basal-cell	Percent	1990	6.89E-05	8.83E-05	5.16E-05
Prevalence	Zimbabwe	Female	55+ years	Non-melanoma skin cancer (basal-cell	Percent	1990	4.74E-05	6.41E-05	3.53E-05
Prevalence	Zimbabwe	Both	55+ years	Non-melanoma skin cancer (basal-cell	Percent	1990	5.81E-05	7.65E-05	4.41E-05
Prevalence	Zimbabwe	Male	55+ years	Non-melanoma skin cancer (basal-cell	Rate	1990	6.8857254	8.829174248	5.160740559
Prevalence	Zimbabwe	Female	55+ years	Non-melanoma skin cancer (basal-cell	Rate	1990	4.7384036	6.411766174	3.531422577
Prevalence	Zimbabwe	Both	55+ years	Non-melanoma skin cancer (basal-cell	Rate	1990	5.8097938	7.648984949	4.411890564
Prevalence	Zimbabwe	Male	55+ years	Non-melanoma skin cancer (basal-cell	Number	2021	28.956223	38.83557089	21.22039235
Prevalence	Zimbabwe	Female	55+ years	Non-melanoma skin cancer (basal-cell	Number	2021	26.805689	36.4099899	19.65631121
Prevalence	Zimbabwe	Both	55+ years	Non-melanoma skin cancer (basal-cell	Number	2021	55.761912	74.60912736	40.83457224
Prevalence	Zimbabwe	Male	55+ years	Non-melanoma skin cancer (basal-cell	Percent	2021	6.46E-05	8.66E-05	4.73E-05
Prevalence	Zimbabwe	Female	55+ years	Non-melanoma skin cancer (basal-cell	Percent	2021	4.07E-05	5.53E-05	2.99E-05
Prevalence	Zimbabwe	Both	55+ years	Non-melanoma skin cancer (basal-cell	Percent	2021	5.04E-05	6.74E-05	3.69E-05
Prevalence	Zimbabwe	Male	55+ years	Non-melanoma skin cancer (basal-cell	Rate	2021	6.4525402	8.654032064	4.728704937
Prevalence	Zimbabwe	Female	55+ years	Non-melanoma skin cancer (basal-cell	Rate	2021	4.0706307	5.529110656	2.984947814
Prevalence	Zimbabwe	Both	55+ years	Non-melanoma skin cancer (basal-cell	Rate	2021	5.0359753	6.738106869	3.687855916
Prevalence	Niger	Male	55+ years	Non-melanoma skin cancer (basal-cell	Number	1990	4.235205	5.965601348	2.997033606
Prevalence	Niger	Female	55+ years	Non-melanoma skin cancer (basal-cell	Number	1990	2.1446646	2.952543712	1.51712288
Prevalence	Niger	Both	55+ years	Non-melanoma skin cancer (basal-cell	Number	1990	6.3798696	8.900357441	4.506273105
Prevalence	Niger	Male	55+ years	Non-melanoma skin cancer (basal-cell	Percent	1990	1.79E-05	2.53E-05	1.27E-05
Prevalence	Niger	Female	55+ years	Non-melanoma skin cancer (basal-cell	Percent	1990	1.05E-05	1.45E-05	7.46E-06
Prevalence	Niger	Both	55+ years	Non-melanoma skin cancer (basal-cell	Percent	1990	1.45E-05	2.03E-05	1.03E-05
Prevalence	Niger	Male	55+ years	Non-melanoma skin cancer (basal-cell	Rate	1990	1.7937265	2.526597283	1.269326682
Prevalence	Niger	Female	55+ years	Non-melanoma skin cancer (basal-cell	Rate	1990	1.054476	1.451689232	0.745930006
Prevalence	Niger	Both	55+ years	Non-melanoma skin cancer (basal-cell	Rate	1990	1.4516238	2.025115119	1.025320821
Prevalence	Niger	Male	55+ years	Non-melanoma skin cancer (basal-cell	Number	2021	12.277625	16.71183071	8.658129582
Prevalence	Niger	Female	55+ years	Non-melanoma skin cancer (basal-cell	Number	2021	7.2200783	10.08872555	5.029161523
Prevalence	Niger	Both	55+ years	Non-melanoma skin cancer (basal-cell	Number	2021	19.497703	26.69413218	13.633863
Prevalence	Niger	Male	55+ years	Non-melanoma skin cancer (basal-cell	Percent	2021	1.79E-05	2.44E-05	1.26E-05
Prevalence	Niger	Female	55+ years	Non-melanoma skin cancer (basal-cell	Percent	2021	1.02E-05	1.43E-05	7.11E-06
Prevalence	Niger	Both	55+ years	Non-melanoma skin cancer (basal-cell	Percent	2021	1.40E-05	1.92E-05	9.79E-06
Prevalence	Niger	Male	55+ years	Non-melanoma skin cancer (basal-cell	Rate	2021	1.7926585	2.440097805	1.264175263
Prevalence	Niger	Female	55+ years	Non-melanoma skin cancer (basal-cell	Rate	2021	1.0207496	1.426308914	0.711005357
Prevalence	Niger	Both	55+ years	Non-melanoma skin cancer (basal-cell	Rate	2021	1.4004811	1.917386212	0.979293155
Prevalence	Mauritania	Male	55+ years	Non-melanoma skin cancer (basal-cell	Number	1990	1.6194315	2.223058449	1.153128129
Prevalence	Mauritania	Female	55+ years	Non-melanoma skin cancer (basal-cell	Number	1990	0.9942949	1.373530324	0.688843698
Prevalence	Mauritania	Both	55+ years	Non-melanoma skin cancer (basal-cell	Number	1990	2.6137264	3.57008851	1.892169114
Prevalence	Mauritania	Male	55+ years	Non-melanoma skin cancer (basal-cell	Percent	1990	2.03E-05	2.79E-05	1.45E-05
Prevalence	Mauritania	Female	55+ years	Non-melanoma skin cancer (basal-cell	Percent	1990	1.15E-05	1.59E-05	7.98E-06
Prevalence	Mauritania	Both	55+ years	Non-melanoma skin cancer (basal-cell	Percent	1990	1.57E-05	2.15E-05	1.14E-05
Prevalence	Mauritania	Male	55+ years	Non-melanoma skin cancer (basal-cell	Rate	1990	2.0320693	2.789502875	1.446949914
Prevalence	Mauritania	Female	55+ years	Non-melanoma skin cancer (basal-cell	Rate	1990	1.1518984	1.591245575	0.798030787
Prevalence	Mauritania	Both	55+ years	Non-melanoma skin cancer (basal-cell	Rate	1990	1.5744235	2.150504813	1.139780926
Prevalence	Mauritania	Male	55+ years	Non-melanoma skin cancer (basal-cell	Number	2021	3.7112938	5.281108247	2.668650068
Prevalence	Mauritania	Female	55+ years	Non-melanoma skin cancer (basal-cell	Number	2021	1.9837216	2.790649193	1.340489901

Prevalence	Mauritania	Both	55+ years	Non-melanoma skin cancer (basal-cell	Number	2021	5.6950154	7.956284059	4.096243087
Prevalence	Mauritania	Male	55+ years	Non-melanoma skin cancer (basal-cell	Percent	2021	2.07E-05	2.94E-05	1.49E-05
Prevalence	Mauritania	Female	55+ years	Non-melanoma skin cancer (basal-cell	Percent	2021	1.11E-05	1.56E-05	7.49E-06
Prevalence	Mauritania	Both	55+ years	Non-melanoma skin cancer (basal-cell	Percent	2021	1.59E-05	2.22E-05	1.14E-05
Prevalence	Mauritania	Male	55+ years	Non-melanoma skin cancer (basal-cell	Rate	2021	2.0653145	2.938907513	1.485088994
Prevalence	Mauritania	Female	55+ years	Non-melanoma skin cancer (basal-cell	Rate	2021	1.1078442	1.558487035	0.748620119
Prevalence	Mauritania	Both	55+ years	Non-melanoma skin cancer (basal-cell	Rate	2021	1.5874265	2.217731696	1.141785293
Prevalence	Senegal	Male	55+ years	Non-melanoma skin cancer (basal-cell	Number	1990	8.1115722	11.11451675	5.912202996
Prevalence	Senegal	Female	55+ years	Non-melanoma skin cancer (basal-cell	Number	1990	4.9045163	6.702158591	3.51798571
Prevalence	Senegal	Both	55+ years	Non-melanoma skin cancer (basal-cell	Number	1990	13.016088	17.65504386	9.681985222
Prevalence	Senegal	Male	55+ years	Non-melanoma skin cancer (basal-cell	Percent	1990	2.99E-05	4.09E-05	2.18E-05
Prevalence	Senegal	Female	55+ years	Non-melanoma skin cancer (basal-cell	Percent	1990	1.91E-05	2.61E-05	1.37E-05
Prevalence	Senegal	Both	55+ years	Non-melanoma skin cancer (basal-cell	Percent	1990	2.46E-05	3.34E-05	1.83E-05
Prevalence	Senegal	Male	55+ years	Non-melanoma skin cancer (basal-cell	Rate	1990	2.9851965	4.090331107	2.175791209
Prevalence	Senegal	Female	55+ years	Non-melanoma skin cancer (basal-cell	Rate	1990	1.9105602	2.610833954	1.370435572
Prevalence	Senegal	Both	55+ years	Non-melanoma skin cancer (basal-cell	Rate	1990	2.4631518	3.341023222	1.832209408
Prevalence	Senegal	Male	55+ years	Non-melanoma skin cancer (basal-cell	Number	2021	18.236033	24.38791246	13.00994145
Prevalence	Senegal	Female	55+ years	Non-melanoma skin cancer (basal-cell	Number	2021	12.682507	16.93047073	9.331827467
Prevalence	Senegal	Both	55+ years	Non-melanoma skin cancer (basal-cell	Number	2021	30.91854	40.71526592	22.8651069
Prevalence	Senegal	Male	55+ years	Non-melanoma skin cancer (basal-cell	Percent	2021	2.93E-05	3.92E-05	2.09E-05
Prevalence	Senegal	Female	55+ years	Non-melanoma skin cancer (basal-cell	Percent	2021	1.90E-05	2.53E-05	1.39E-05
Prevalence	Senegal	Both	55+ years	Non-melanoma skin cancer (basal-cell	Percent	2021	2.40E-05	3.15E-05	1.77E-05
Prevalence	Senegal	Male	55+ years	Non-melanoma skin cancer (basal-cell	Rate	2021	2.9324585	3.921715858	2.092073021
Prevalence	Senegal	Female	55+ years	Non-melanoma skin cancer (basal-cell	Rate	2021	1.8948996	2.529589965	1.394272935
Prevalence	Senegal	Both	55+ years	Non-melanoma skin cancer (basal-cell	Rate	2021	2.3946226	3.153373228	1.770888986
Prevalence	Namibia	Male	55+ years	Non-melanoma skin cancer (basal-cell	Number	1990	4.3976191	6.017355991	3.127191571
Prevalence	Namibia	Female	55+ years	Non-melanoma skin cancer (basal-cell	Number	1990	3.2703723	4.297676353	2.436807885
Prevalence	Namibia	Both	55+ years	Non-melanoma skin cancer (basal-cell	Number	1990	7.6679915	10.20662851	5.624082683
Prevalence	Namibia	Male	55+ years	Non-melanoma skin cancer (basal-cell	Percent	1990	9.02E-05	0.00012347	6.42E-05
Prevalence	Namibia	Female	55+ years	Non-melanoma skin cancer (basal-cell	Percent	1990	5.47E-05	7.19E-05	4.08E-05
Prevalence	Namibia	Both	55+ years	Non-melanoma skin cancer (basal-cell	Percent	1990	7.07E-05	9.41E-05	5.18E-05
Prevalence	Namibia	Male	55+ years	Non-melanoma skin cancer (basal-cell	Rate	1990	9.0189422	12.34081082	6.413461268
Prevalence	Namibia	Female	55+ years	Non-melanoma skin cancer (basal-cell	Rate	1990	5.4734441	7.192786967	4.07835271
Prevalence	Namibia	Both	55+ years	Non-melanoma skin cancer (basal-cell	Rate	1990	7.0666468	9.40619707	5.183026895
Prevalence	Namibia	Male	55+ years	Non-melanoma skin cancer (basal-cell	Number	2021	8.3755366	10.9800109	6.129367576
Prevalence	Namibia	Female	55+ years	Non-melanoma skin cancer (basal-cell	Number	2021	7.344523	9.738165712	5.598461752
Prevalence	Namibia	Both	55+ years	Non-melanoma skin cancer (basal-cell	Number	2021	15.72006	20.66202249	11.80294945
Prevalence	Namibia	Male	55+ years	Non-melanoma skin cancer (basal-cell	Percent	2021	8.95E-05	0.000117361	6.55E-05
Prevalence	Namibia	Female	55+ years	Non-melanoma skin cancer (basal-cell	Percent	2021	5.55E-05	7.36E-05	4.23E-05
Prevalence	Namibia	Both	55+ years	Non-melanoma skin cancer (basal-cell	Percent	2021	6.96E-05	9.15E-05	5.23E-05
Prevalence	Namibia	Male	55+ years	Non-melanoma skin cancer (basal-cell	Rate	2021	8.9487101	11.73141967	6.548826225
Prevalence	Namibia	Female	55+ years	Non-melanoma skin cancer (basal-cell	Rate	2021	5.5492345	7.357777386	4.229978878
Prevalence	Namibia	Both	55+ years	Non-melanoma skin cancer (basal-cell	Rate	2021	6.9574131	9.144636172	5.223771223
Prevalence	Chad	Male	55+ years	Non-melanoma skin cancer (basal-cell	Number	1990	4.303607	5.859239445	3.030621858
Prevalence	Chad	Female	55+ years	Non-melanoma skin cancer (basal-cell	Number	1990	2.6222516	3.567905561	1.823345451
Prevalence	Chad	Both	55+ years	Non-melanoma skin cancer (basal-cell	Number	1990	6.9258586	9.356419628	4.893890842
Prevalence	Chad	Male	55+ years	Non-melanoma skin cancer (basal-cell	Percent	1990	1.90E-05	2.59E-05	1.34E-05
Prevalence	Chad	Female	55+ years	Non-melanoma skin cancer (basal-cell	Percent	1990	1.11E-05	1.51E-05	7.70E-06
Prevalence	Chad	Both	55+ years	Non-melanoma skin cancer (basal-cell	Percent	1990	1.49E-05	2.02E-05	1.06E-05
Prevalence	Chad	Male	55+ years	Non-melanoma skin cancer (basal-cell	Rate	1990	1.8999331	2.58670531	1.337942531
Prevalence	Chad	Female	55+ years	Non-melanoma skin cancer (basal-cell	Rate	1990	1.1069406	1.506132896	0.769695419
Prevalence	Chad	Both	55+ years	Non-melanoma skin cancer (basal-cell	Rate	1990	1.4945571	2.01905701	1.056071126
Prevalence	Chad	Male	55+ years	Non-melanoma skin cancer (basal-cell	Number	2021	9.111777	12.63959596	6.160960174
Prevalence	Chad	Female	55+ years	Non-melanoma skin cancer (basal-cell	Number	2021	4.3154875	6.053736527	2.988220806
Prevalence	Chad	Both	55+ years	Non-melanoma skin cancer (basal-cell	Number	2021	13.427265	18.73286125	9.283057216
Prevalence	Chad	Male	55+ years	Non-melanoma skin cancer (basal-cell	Percent	2021	1.78E-05	2.46E-05	1.20E-05

Prevalence	Chad	Female	55+ years	Non-melanoma skin cancer (basal-cell	Percent	2021	1.04E-05	1.46E-05	7.19E-06
Prevalence	Chad	Both	55+ years	Non-melanoma skin cancer (basal-cell	Percent	2021	1.45E-05	2.02E-05	1.00E-05
Prevalence	Chad	Male	55+ years	Non-melanoma skin cancer (basal-cell	Rate	2021	1.7761704	2.463852737	1.200963911
Prevalence	Chad	Female	55+ years	Non-melanoma skin cancer (basal-cell	Rate	2021	1.0385726	1.45690251	0.719150292
Prevalence	Chad	Both	55+ years	Non-melanoma skin cancer (basal-cell	Rate	2021	1.4460896	2.017491756	0.999766728
Prevalence	Mali	Male	55+ years	Non-melanoma skin cancer (basal-cell	Number	1990	7.5567577	10.30288109	5.548932639
Prevalence	Mali	Female	55+ years	Non-melanoma skin cancer (basal-cell	Number	1990	5.3306727	7.127618794	3.968094779
Prevalence	Mali	Both	55+ years	Non-melanoma skin cancer (basal-cell	Number	1990	12.88743	17.26691824	9.662037381
Prevalence	Mali	Male	55+ years	Non-melanoma skin cancer (basal-cell	Percent	1990	2.22E-05	3.03E-05	1.63E-05
Prevalence	Mali	Female	55+ years	Non-melanoma skin cancer (basal-cell	Percent	1990	1.62E-05	2.17E-05	1.21E-05
Prevalence	Mali	Both	55+ years	Non-melanoma skin cancer (basal-cell	Percent	1990	1.93E-05	2.58E-05	1.45E-05
Prevalence	Mali	Male	55+ years	Non-melanoma skin cancer (basal-cell	Rate	1990	2.2195774	3.02617112	1.629837281
Prevalence	Mali	Female	55+ years	Non-melanoma skin cancer (basal-cell	Rate	1990	1.6242252	2.171744371	1.209055612
Prevalence	Mali	Both	55+ years	Non-melanoma skin cancer (basal-cell	Rate	1990	1.9273599	2.582327525	1.44499121
Prevalence	Mali	Male	55+ years	Non-melanoma skin cancer (basal-cell	Number	2021	17.173714	23.74697755	12.32060087
Prevalence	Mali	Female	55+ years	Non-melanoma skin cancer (basal-cell	Number	2021	10.643997	14.50657988	7.45188661
Prevalence	Mali	Both	55+ years	Non-melanoma skin cancer (basal-cell	Number	2021	27.817711	38.07026073	19.92619909
Prevalence	Mali	Male	55+ years	Non-melanoma skin cancer (basal-cell	Percent	2021	2.29E-05	3.17E-05	1.64E-05
Prevalence	Mali	Female	55+ years	Non-melanoma skin cancer (basal-cell	Percent	2021	1.53E-05	2.08E-05	1.07E-05
Prevalence	Mali	Both	55+ years	Non-melanoma skin cancer (basal-cell	Percent	2021	1.92E-05	2.63E-05	1.38E-05
Prevalence	Mali	Male	55+ years	Non-melanoma skin cancer (basal-cell	Rate	2021	2.2907829	3.167583282	1.643431433
Prevalence	Mali	Female	55+ years	Non-melanoma skin cancer (basal-cell	Rate	2021	1.5256157	2.079243891	1.068087023
Prevalence	Mali	Both	55+ years	Non-melanoma skin cancer (basal-cell	Rate	2021	1.921945	2.630300793	1.376714954
Prevalence	Liberia	Male	55+ years	Non-melanoma skin cancer (basal-cell	Number	1990	2.0462863	2.862898962	1.425902815
Prevalence	Liberia	Female	55+ years	Non-melanoma skin cancer (basal-cell	Number	1990	0.9418903	1.291971047	0.6586964
Prevalence	Liberia	Both	55+ years	Non-melanoma skin cancer (basal-cell	Number	1990	2.9881766	4.161854529	2.089225561
Prevalence	Liberia	Male	55+ years	Non-melanoma skin cancer (basal-cell	Percent	1990	1.99E-05	2.78E-05	1.38E-05
Prevalence	Liberia	Female	55+ years	Non-melanoma skin cancer (basal-cell	Percent	1990	1.10E-05	1.51E-05	7.72E-06
Prevalence	Liberia	Both	55+ years	Non-melanoma skin cancer (basal-cell	Percent	1990	1.59E-05	2.21E-05	1.11E-05
Prevalence	Liberia	Male	55+ years	Non-melanoma skin cancer (basal-cell	Rate	1990	1.98495	2.7770851	1.383162142
Prevalence	Liberia	Female	55+ years	Non-melanoma skin cancer (basal-cell	Rate	1990	1.1035711	1.513745171	0.771765356
Prevalence	Liberia	Both	55+ years	Non-melanoma skin cancer (basal-cell	Rate	1990	1.5857496	2.208590665	1.108699028
Prevalence	Liberia	Male	55+ years	Non-melanoma skin cancer (basal-cell	Number	2021	3.1308335	4.204216806	2.258624983
Prevalence	Liberia	Female	55+ years	Non-melanoma skin cancer (basal-cell	Number	2021	1.6733101	2.414855379	1.122353042
Prevalence	Liberia	Both	55+ years	Non-melanoma skin cancer (basal-cell	Number	2021	4.8041436	6.586170654	3.401719009
Prevalence	Liberia	Male	55+ years	Non-melanoma skin cancer (basal-cell	Percent	2021	1.84E-05	2.47E-05	1.32E-05
Prevalence	Liberia	Female	55+ years	Non-melanoma skin cancer (basal-cell	Percent	2021	1.05E-05	1.51E-05	7.03E-06
Prevalence	Liberia	Both	55+ years	Non-melanoma skin cancer (basal-cell	Percent	2021	1.46E-05	2.00E-05	1.03E-05
Prevalence	Liberia	Male	55+ years	Non-melanoma skin cancer (basal-cell	Rate	2021	1.8358591	2.46526985	1.324413162
Prevalence	Liberia	Female	55+ years	Non-melanoma skin cancer (basal-cell	Rate	2021	1.0485461	1.513220495	0.703299933
Prevalence	Liberia	Both	55+ years	Non-melanoma skin cancer (basal-cell	Rate	2021	1.4552647	1.99507393	1.030444133
Prevalence	Gambia	Male	55+ years	Non-melanoma skin cancer (basal-cell	Number	1990	0.478732	0.653323129	0.342703592
Prevalence	Gambia	Female	55+ years	Non-melanoma skin cancer (basal-cell	Number	1990	0.2973109	0.408270002	0.212073189
Prevalence	Gambia	Both	55+ years	Non-melanoma skin cancer (basal-cell	Number	1990	0.7760428	1.060274065	0.555909456
Prevalence	Gambia	Male	55+ years	Non-melanoma skin cancer (basal-cell	Percent	1990	1.59E-05	2.17E-05	1.14E-05
Prevalence	Gambia	Female	55+ years	Non-melanoma skin cancer (basal-cell	Percent	1990	1.18E-05	1.62E-05	8.39E-06
Prevalence	Gambia	Both	55+ years	Non-melanoma skin cancer (basal-cell	Percent	1990	1.40E-05	1.91E-05	1.00E-05
Prevalence	Gambia	Male	55+ years	Non-melanoma skin cancer (basal-cell	Rate	1990	1.590589	2.170668836	1.13863412
Prevalence	Gambia	Female	55+ years	Non-melanoma skin cancer (basal-cell	Rate	1990	1.1760781	1.615001136	0.838901803
Prevalence	Gambia	Both	55+ years	Non-melanoma skin cancer (basal-cell	Rate	1990	1.401365	1.914624918	1.003851865
Prevalence	Gambia	Male	55+ years	Non-melanoma skin cancer (basal-cell	Number	2021	1.2953669	1.829607841	0.906289989
Prevalence	Gambia	Female	55+ years	Non-melanoma skin cancer (basal-cell	Number	2021	0.8966768	1.238501299	0.634909684
Prevalence	Gambia	Both	55+ years	Non-melanoma skin cancer (basal-cell	Number	2021	2.1920437	3.075120268	1.559120445
Prevalence	Gambia	Male	55+ years	Non-melanoma skin cancer (basal-cell	Percent	2021	1.71E-05	2.41E-05	1.19E-05
Prevalence	Gambia	Female	55+ years	Non-melanoma skin cancer (basal-cell	Percent	2021	1.14E-05	1.57E-05	8.07E-06
Prevalence	Gambia	Both	55+ years	Non-melanoma skin cancer (basal-cell	Percent	2021	1.42E-05	1.99E-05	1.01E-05

Prevalence	Gambia	Male	55+ years	Non-melanoma skin cancer (basal-cell	Rate	2021	1.7058326	2.409359537	1.193468009
Prevalence	Gambia	Female	55+ years	Non-melanoma skin cancer (basal-cell	Rate	2021	1.1393207	1.573644087	0.806718469
Prevalence	Gambia	Both	55+ years	Non-melanoma skin cancer (basal-cell	Rate	2021	1.4175115	1.988563613	1.008224042
Prevalence	Bermuda	Male	55+ years	Non-melanoma skin cancer (basal-cell	Number	1990	0.1048219	0.142498162	0.07371841
Prevalence	Bermuda	Female	55+ years	Non-melanoma skin cancer (basal-cell	Number	1990	0.086882	0.126281618	0.052165598
Prevalence	Bermuda	Both	55+ years	Non-melanoma skin cancer (basal-cell	Number	1990	0.1917038	0.263642804	0.130059694
Prevalence	Bermuda	Male	55+ years	Non-melanoma skin cancer (basal-cell	Percent	1990	2.26E-05	3.07E-05	1.59E-05
Prevalence	Bermuda	Female	55+ years	Non-melanoma skin cancer (basal-cell	Percent	1990	1.46E-05	2.13E-05	8.79E-06
Prevalence	Bermuda	Both	55+ years	Non-melanoma skin cancer (basal-cell	Percent	1990	1.81E-05	2.49E-05	1.23E-05
Prevalence	Bermuda	Male	55+ years	Non-melanoma skin cancer (basal-cell	Rate	1990	2.2588771	3.070788742	1.588607595
Prevalence	Bermuda	Female	55+ years	Non-melanoma skin cancer (basal-cell	Rate	1990	1.463114	2.126614277	0.878481813
Prevalence	Bermuda	Both	55+ years	Non-melanoma skin cancer (basal-cell	Rate	1990	1.8121861	2.492228893	1.22946093
Prevalence	Bermuda	Male	55+ years	Non-melanoma skin cancer (basal-cell	Number	2021	0.2431898	0.330413084	0.171106847
Prevalence	Bermuda	Female	55+ years	Non-melanoma skin cancer (basal-cell	Number	2021	0.1862049	0.274101987	0.110970209
Prevalence	Bermuda	Both	55+ years	Non-melanoma skin cancer (basal-cell	Number	2021	0.4293947	0.587290133	0.286241818
Prevalence	Bermuda	Male	55+ years	Non-melanoma skin cancer (basal-cell	Percent	2021	2.31E-05	3.14E-05	1.62E-05
Prevalence	Bermuda	Female	55+ years	Non-melanoma skin cancer (basal-cell	Percent	2021	1.46E-05	2.15E-05	8.71E-06
Prevalence	Bermuda	Both	55+ years	Non-melanoma skin cancer (basal-cell	Percent	2021	1.84E-05	2.52E-05	1.23E-05
Prevalence	Bermuda	Male	55+ years	Non-melanoma skin cancer (basal-cell	Rate	2021	2.3068686	3.134258586	1.623098876
Prevalence	Bermuda	Female	55+ years	Non-melanoma skin cancer (basal-cell	Rate	2021	1.4605594	2.15000906	0.870431324
Prevalence	Bermuda	Both	55+ years	Non-melanoma skin cancer (basal-cell	Rate	2021	1.8436186	2.521547502	1.228987683
Prevalence	Puerto Ric	Male	55+ years	Non-melanoma skin cancer (basal-cell	Number	1990	11.081358	14.92900012	7.857067395
Prevalence	Puerto Ric	Female	55+ years	Non-melanoma skin cancer (basal-cell	Number	1990	5.3517321	7.34122294	3.714248992
Prevalence	Puerto Ric	Both	55+ years	Non-melanoma skin cancer (basal-cell	Number	1990	16.43309	22.19802204	11.73526603
Prevalence	Puerto Ric	Male	55+ years	Non-melanoma skin cancer (basal-cell	Percent	1990	3.95E-05	5.33E-05	2.80E-05
Prevalence	Puerto Ric	Female	55+ years	Non-melanoma skin cancer (basal-cell	Percent	1990	1.64E-05	2.25E-05	1.14E-05
Prevalence	Puerto Ric	Both	55+ years	Non-melanoma skin cancer (basal-cell	Percent	1990	2.71E-05	3.66E-05	1.93E-05
Prevalence	Puerto Ric	Male	55+ years	Non-melanoma skin cancer (basal-cell	Rate	1990	3.9520893	5.324323954	2.802168384
Prevalence	Puerto Ric	Female	55+ years	Non-melanoma skin cancer (basal-cell	Rate	1990	1.6397589	2.249334576	1.138037729
Prevalence	Puerto Ric	Both	55+ years	Non-melanoma skin cancer (basal-cell	Rate	1990	2.7083099	3.658418588	1.93406941
Prevalence	Puerto Ric	Male	55+ years	Non-melanoma skin cancer (basal-cell	Number	2021	21.918092	28.38567875	16.29493383
Prevalence	Puerto Ric	Female	55+ years	Non-melanoma skin cancer (basal-cell	Number	2021	11.740797	15.73308239	8.362716037
Prevalence	Puerto Ric	Both	55+ years	Non-melanoma skin cancer (basal-cell	Number	2021	33.658889	43.96063549	24.52231647
Prevalence	Puerto Ric	Male	55+ years	Non-melanoma skin cancer (basal-cell	Percent	2021	4.23E-05	5.47E-05	3.14E-05
Prevalence	Puerto Ric	Female	55+ years	Non-melanoma skin cancer (basal-cell	Percent	2021	1.81E-05	2.43E-05	1.29E-05
Prevalence	Puerto Ric	Both	55+ years	Non-melanoma skin cancer (basal-cell	Percent	2021	2.88E-05	3.77E-05	2.10E-05
Prevalence	Puerto Ric	Male	55+ years	Non-melanoma skin cancer (basal-cell	Rate	2021	4.2253813	5.472206114	3.141345933
Prevalence	Puerto Ric	Female	55+ years	Non-melanoma skin cancer (basal-cell	Rate	2021	1.8100646	2.4255505	1.289269932
Prevalence	Puerto Ric	Both	55+ years	Non-melanoma skin cancer (basal-cell	Rate	2021	2.8833236	3.765802805	2.100656806
Prevalence	Palau	Male	55+ years	Non-melanoma skin cancer (basal-cell	Number	1990	0.000177	0.000390546	4.45E-05
Prevalence	Palau	Female	55+ years	Non-melanoma skin cancer (basal-cell	Number	1990	0.0001953	0.000433325	5.92E-05
Prevalence	Palau	Both	55+ years	Non-melanoma skin cancer (basal-cell	Number	1990	0.0003723	0.000821036	0.000127731
Prevalence	Palau	Male	55+ years	Non-melanoma skin cancer (basal-cell	Percent	1990	2.24E-07	4.94E-07	5.63E-08
Prevalence	Palau	Female	55+ years	Non-melanoma skin cancer (basal-cell	Percent	1990	2.41E-07	5.35E-07	7.31E-08
Prevalence	Palau	Both	55+ years	Non-melanoma skin cancer (basal-cell	Percent	1990	2.33E-07	5.13E-07	7.98E-08
Prevalence	Palau	Male	55+ years	Non-melanoma skin cancer (basal-cell	Rate	1990	0.0223896	0.049407254	0.005627672
Prevalence	Palau	Female	55+ years	Non-melanoma skin cancer (basal-cell	Rate	1990	0.0241229	0.053513267	0.007309264
Prevalence	Palau	Both	55+ years	Non-melanoma skin cancer (basal-cell	Rate	1990	0.0232667	0.051307885	0.007982138
Prevalence	Palau	Male	55+ years	Non-melanoma skin cancer (basal-cell	Number	2021	0.0004719	0.001032925	0.000116688
Prevalence	Palau	Female	55+ years	Non-melanoma skin cancer (basal-cell	Number	2021	0.0004986	0.001132594	0.000151374
Prevalence	Palau	Both	55+ years	Non-melanoma skin cancer (basal-cell	Number	2021	0.0009705	0.002172461	0.00031773
Prevalence	Palau	Male	55+ years	Non-melanoma skin cancer (basal-cell	Percent	2021	2.24E-07	4.89E-07	5.53E-08
Prevalence	Palau	Female	55+ years	Non-melanoma skin cancer (basal-cell	Percent	2021	2.40E-07	5.46E-07	7.29E-08
Prevalence	Palau	Both	55+ years	Non-melanoma skin cancer (basal-cell	Percent	2021	2.32E-07	5.19E-07	7.59E-08
Prevalence	Palau	Male	55+ years	Non-melanoma skin cancer (basal-cell	Rate	2021	0.0223474	0.048916024	0.005525951
Prevalence	Palau	Female	55+ years	Non-melanoma skin cancer (basal-cell	Rate	2021	0.0240225	0.054563971	0.007292625

Prevalence	Palau	Both	55+ years	Non-melanoma skin cancer (basal-cell	Rate	2021	0.0231778	0.05188157	0.007587861
Prevalence	Saint Kitt	Male	55+ years	Non-melanoma skin cancer (basal-cell	Number	1990	0.0924944	0.126111594	0.066741586
Prevalence	Saint Kitt	Female	55+ years	Non-melanoma skin cancer (basal-cell	Number	1990	0.0470257	0.064765981	0.031952707
Prevalence	Saint Kitt	Both	55+ years	Non-melanoma skin cancer (basal-cell	Number	1990	0.1395201	0.19179102	0.100869752
Prevalence	Saint Kitt	Male	55+ years	Non-melanoma skin cancer (basal-cell	Percent	1990	3.31E-05	4.51E-05	2.39E-05
Prevalence	Saint Kitt	Female	55+ years	Non-melanoma skin cancer (basal-cell	Percent	1990	1.31E-05	1.80E-05	8.88E-06
Prevalence	Saint Kitt	Both	55+ years	Non-melanoma skin cancer (basal-cell	Percent	1990	2.18E-05	3.00E-05	1.58E-05
Prevalence	Saint Kitt	Male	55+ years	Non-melanoma skin cancer (basal-cell	Rate	1990	3.3106032	4.513843229	2.38884505
Prevalence	Saint Kitt	Female	55+ years	Non-melanoma skin cancer (basal-cell	Rate	1990	1.3060335	1.798731801	0.887415724
Prevalence	Saint Kitt	Both	55+ years	Non-melanoma skin cancer (basal-cell	Rate	1990	2.1818657	2.999297202	1.577437584
Prevalence	Saint Kitt	Male	55+ years	Non-melanoma skin cancer (basal-cell	Number	2021	0.1650986	0.223197588	0.12160549
Prevalence	Saint Kitt	Female	55+ years	Non-melanoma skin cancer (basal-cell	Number	2021	0.0752944	0.10352602	0.051258384
Prevalence	Saint Kitt	Both	55+ years	Non-melanoma skin cancer (basal-cell	Number	2021	0.240393	0.322790756	0.174619332
Prevalence	Saint Kitt	Male	55+ years	Non-melanoma skin cancer (basal-cell	Percent	2021	2.71E-05	3.66E-05	1.99E-05
Prevalence	Saint Kitt	Female	55+ years	Non-melanoma skin cancer (basal-cell	Percent	2021	1.11E-05	1.53E-05	7.56E-06
Prevalence	Saint Kitt	Both	55+ years	Non-melanoma skin cancer (basal-cell	Percent	2021	1.87E-05	2.51E-05	1.36E-05
Prevalence	Saint Kitt	Male	55+ years	Non-melanoma skin cancer (basal-cell	Rate	2021	2.7065277	3.658967843	1.993527719
Prevalence	Saint Kitt	Female	55+ years	Non-melanoma skin cancer (basal-cell	Rate	2021	1.110643	1.527078935	0.756095883
Prevalence	Saint Kitt	Both	55+ years	Non-melanoma skin cancer (basal-cell	Rate	2021	1.8664971	2.506263034	1.355807034
Prevalence	Tokelau	Male	55+ years	Non-melanoma skin cancer (basal-cell	Number	1990	2.93E-05	6.43E-05	7.07E-06
Prevalence	Tokelau	Female	55+ years	Non-melanoma skin cancer (basal-cell	Number	1990	2.73E-05	6.11E-05	8.59E-06
Prevalence	Tokelau	Both	55+ years	Non-melanoma skin cancer (basal-cell	Number	1990	5.66E-05	0.000123294	1.93E-05
Prevalence	Tokelau	Male	55+ years	Non-melanoma skin cancer (basal-cell	Percent	1990	2.51E-07	5.51E-07	6.06E-08
Prevalence	Tokelau	Female	55+ years	Non-melanoma skin cancer (basal-cell	Percent	1990	2.28E-07	5.09E-07	7.16E-08
Prevalence	Tokelau	Both	55+ years	Non-melanoma skin cancer (basal-cell	Percent	1990	2.39E-07	5.21E-07	8.14E-08
Prevalence	Tokelau	Male	55+ years	Non-melanoma skin cancer (basal-cell	Rate	1990	0.0250684	0.055051922	0.006054988
Prevalence	Tokelau	Female	55+ years	Non-melanoma skin cancer (basal-cell	Rate	1990	0.0227804	0.050918162	0.007162319
Prevalence	Tokelau	Both	55+ years	Non-melanoma skin cancer (basal-cell	Rate	1990	0.023909	0.052100912	0.008134898
Prevalence	Tokelau	Male	55+ years	Non-melanoma skin cancer (basal-cell	Number	2021	3.09E-05	6.38E-05	8.02E-06
Prevalence	Tokelau	Female	55+ years	Non-melanoma skin cancer (basal-cell	Number	2021	3.01E-05	6.42E-05	9.39E-06
Prevalence	Tokelau	Both	55+ years	Non-melanoma skin cancer (basal-cell	Number	2021	6.10E-05	0.000127967	2.20E-05
Prevalence	Tokelau	Male	55+ years	Non-melanoma skin cancer (basal-cell	Percent	2021	2.54E-07	5.24E-07	6.59E-08
Prevalence	Tokelau	Female	55+ years	Non-melanoma skin cancer (basal-cell	Percent	2021	2.30E-07	4.90E-07	7.17E-08
Prevalence	Tokelau	Both	55+ years	Non-melanoma skin cancer (basal-cell	Percent	2021	2.41E-07	5.06E-07	8.71E-08
Prevalence	Tokelau	Male	55+ years	Non-melanoma skin cancer (basal-cell	Rate	2021	0.0253522	0.052377631	0.006585122
Prevalence	Tokelau	Female	55+ years	Non-melanoma skin cancer (basal-cell	Rate	2021	0.0229883	0.049020963	0.007167409
Prevalence	Tokelau	Both	55+ years	Non-melanoma skin cancer (basal-cell	Rate	2021	0.0241267	0.050631546	0.008711525
Prevalence	Tuvalu	Male	55+ years	Non-melanoma skin cancer (basal-cell	Number	1990	0.0001135	0.000251748	2.80E-05
Prevalence	Tuvalu	Female	55+ years	Non-melanoma skin cancer (basal-cell	Number	1990	0.0001464	0.000321466	4.17E-05
Prevalence	Tuvalu	Both	55+ years	Non-melanoma skin cancer (basal-cell	Number	1990	0.0002599	0.000566539	6.86E-05
Prevalence	Tuvalu	Male	55+ years	Non-melanoma skin cancer (basal-cell	Percent	1990	2.19E-07	4.86E-07	5.41E-08
Prevalence	Tuvalu	Female	55+ years	Non-melanoma skin cancer (basal-cell	Percent	1990	2.27E-07	4.99E-07	6.47E-08
Prevalence	Tuvalu	Both	55+ years	Non-melanoma skin cancer (basal-cell	Percent	1990	2.24E-07	4.88E-07	5.91E-08
Prevalence	Tuvalu	Male	55+ years	Non-melanoma skin cancer (basal-cell	Rate	1990	0.021911	0.048604229	0.005404917
Prevalence	Tuvalu	Female	55+ years	Non-melanoma skin cancer (basal-cell	Rate	1990	0.0227391	0.049925082	0.00646907
Prevalence	Tuvalu	Both	55+ years	Non-melanoma skin cancer (basal-cell	Rate	1990	0.0223699	0.048761771	0.005907588
Prevalence	Tuvalu	Male	55+ years	Non-melanoma skin cancer (basal-cell	Number	2021	0.0001856	0.000405211	4.80E-05
Prevalence	Tuvalu	Female	55+ years	Non-melanoma skin cancer (basal-cell	Number	2021	0.0002246	0.000486943	6.64E-05
Prevalence	Tuvalu	Both	55+ years	Non-melanoma skin cancer (basal-cell	Number	2021	0.0004102	0.00088148	0.000110087
Prevalence	Tuvalu	Male	55+ years	Non-melanoma skin cancer (basal-cell	Percent	2021	2.21E-07	4.82E-07	5.71E-08
Prevalence	Tuvalu	Female	55+ years	Non-melanoma skin cancer (basal-cell	Percent	2021	2.29E-07	4.96E-07	6.77E-08
Prevalence	Tuvalu	Both	55+ years	Non-melanoma skin cancer (basal-cell	Percent	2021	2.25E-07	4.84E-07	6.04E-08
Prevalence	Tuvalu	Male	55+ years	Non-melanoma skin cancer (basal-cell	Rate	2021	0.0220825	0.048199449	0.005706014
Prevalence	Tuvalu	Female	55+ years	Non-melanoma skin cancer (basal-cell	Rate	2021	0.0228772	0.049606362	0.006768288
Prevalence	Tuvalu	Both	55+ years	Non-melanoma skin cancer (basal-cell	Rate	2021	0.0225106	0.048371549	0.006041058
Prevalence	United Sta	Male	55+ years	Non-melanoma skin cancer (basal-cell	Number	1990	0.1996958	0.267269401	0.140769318

Prevalence	United Sta	Female	55+ years	Non-melanoma skin cancer (basal-cell	Number	1990	0.097327	0.134081176	0.067503558
Prevalence	United Sta	Both	55+ years	Non-melanoma skin cancer (basal-cell	Number	1990	0.2970228	0.398106093	0.209243245
Prevalence	United Sta	Male	55+ years	Non-melanoma skin cancer (basal-cell	Percent	1990	3.06E-05	4.10E-05	2.16E-05
Prevalence	United Sta	Female	55+ years	Non-melanoma skin cancer (basal-cell	Percent	1990	1.31E-05	1.80E-05	9.06E-06
Prevalence	United Sta	Both	55+ years	Non-melanoma skin cancer (basal-cell	Percent	1990	2.13E-05	2.85E-05	1.50E-05
Prevalence	United Sta	Male	55+ years	Non-melanoma skin cancer (basal-cell	Rate	1990	3.0632288	4.099772101	2.159327339
Prevalence	United Sta	Female	55+ years	Non-melanoma skin cancer (basal-cell	Rate	1990	1.3067451	1.800219086	0.906325527
Prevalence	United Sta	Both	55+ years	Non-melanoma skin cancer (basal-cell	Rate	1990	2.1265775	2.850297687	1.498107028
Prevalence	United Sta	Male	55+ years	Non-melanoma skin cancer (basal-cell	Number	2021	0.5181032	0.689213981	0.374641506
Prevalence	United Sta	Female	55+ years	Non-melanoma skin cancer (basal-cell	Number	2021	0.2519298	0.338871033	0.175902666
Prevalence	United Sta	Both	55+ years	Non-melanoma skin cancer (basal-cell	Number	2021	0.770033	1.024474366	0.562476549
Prevalence	United Sta	Male	55+ years	Non-melanoma skin cancer (basal-cell	Percent	2021	3.47E-05	4.62E-05	2.51E-05
Prevalence	United Sta	Female	55+ years	Non-melanoma skin cancer (basal-cell	Percent	2021	1.46E-05	1.96E-05	1.02E-05
Prevalence	United Sta	Both	55+ years	Non-melanoma skin cancer (basal-cell	Percent	2021	2.39E-05	3.18E-05	1.75E-05
Prevalence	United Sta	Male	55+ years	Non-melanoma skin cancer (basal-cell	Rate	2021	3.4720617	4.618758007	2.510654896
Prevalence	United Sta	Female	55+ years	Non-melanoma skin cancer (basal-cell	Rate	2021	1.459009	1.962514577	1.018710697
Prevalence	United Sta	Both	55+ years	Non-melanoma skin cancer (basal-cell	Rate	2021	2.3922057	3.182660091	1.747405033
Prevalence	South Suda	Male	55+ years	Non-melanoma skin cancer (basal-cell	Number	1990	5.9497082	8.288626537	4.28291506
Prevalence	South Suda	Female	55+ years	Non-melanoma skin cancer (basal-cell	Number	1990	2.4063745	3.198174053	1.726597409
Prevalence	South Suda	Both	55+ years	Non-melanoma skin cancer (basal-cell	Number	1990	8.3560827	11.45501221	6.12071384
Prevalence	South Suda	Male	55+ years	Non-melanoma skin cancer (basal-cell	Percent	1990	2.36E-05	3.29E-05	1.70E-05
Prevalence	South Suda	Female	55+ years	Non-melanoma skin cancer (basal-cell	Percent	1990	1.48E-05	1.96E-05	1.06E-05
Prevalence	South Suda	Both	55+ years	Non-melanoma skin cancer (basal-cell	Percent	1990	2.02E-05	2.76E-05	1.48E-05
Prevalence	South Suda	Male	55+ years	Non-melanoma skin cancer (basal-cell	Rate	1990	2.3625033	3.291238377	1.70065503
Prevalence	South Suda	Female	55+ years	Non-melanoma skin cancer (basal-cell	Rate	1990	1.4775514	1.96372863	1.060157674
Prevalence	South Suda	Both	55+ years	Non-melanoma skin cancer (basal-cell	Rate	1990	2.0149634	2.762230942	1.475932531
Prevalence	South Suda	Male	55+ years	Non-melanoma skin cancer (basal-cell	Number	2021	7.1787608	9.823618774	5.032806522
Prevalence	South Suda	Female	55+ years	Non-melanoma skin cancer (basal-cell	Number	2021	4.002939	5.480506398	2.801575709
Prevalence	South Suda	Both	55+ years	Non-melanoma skin cancer (basal-cell	Number	2021	11.1817	15.31815445	7.88233254
Prevalence	South Suda	Male	55+ years	Non-melanoma skin cancer (basal-cell	Percent	2021	2.16E-05	2.96E-05	1.52E-05
Prevalence	South Suda	Female	55+ years	Non-melanoma skin cancer (basal-cell	Percent	2021	1.38E-05	1.89E-05	9.64E-06
Prevalence	South Suda	Both	55+ years	Non-melanoma skin cancer (basal-cell	Percent	2021	1.80E-05	2.46E-05	1.27E-05
Prevalence	South Suda	Male	55+ years	Non-melanoma skin cancer (basal-cell	Rate	2021	2.1618723	2.958367031	1.515621608
Prevalence	South Suda	Female	55+ years	Non-melanoma skin cancer (basal-cell	Rate	2021	1.3771882	1.885536776	0.963866046
Prevalence	South Suda	Both	55+ years	Non-melanoma skin cancer (basal-cell	Rate	2021	1.7956152	2.459868551	1.26578577
Prevalence	American S	Male	55+ years	Non-melanoma skin cancer (basal-cell	Number	1990	0.0004393	0.00098404	0.000112269
Prevalence	American S	Female	55+ years	Non-melanoma skin cancer (basal-cell	Number	1990	0.0004557	0.000985781	0.000129365
Prevalence	American S	Both	55+ years	Non-melanoma skin cancer (basal-cell	Number	1990	0.000895	0.001972158	0.000232335
Prevalence	American S	Male	55+ years	Non-melanoma skin cancer (basal-cell	Percent	1990	2.36E-07	5.30E-07	6.04E-08
Prevalence	American S	Female	55+ years	Non-melanoma skin cancer (basal-cell	Percent	1990	2.53E-07	5.48E-07	7.19E-08
Prevalence	American S	Both	55+ years	Non-melanoma skin cancer (basal-cell	Percent	1990	2.45E-07	5.39E-07	6.36E-08
Prevalence	American S	Male	55+ years	Non-melanoma skin cancer (basal-cell	Rate	1990	0.023638	0.052949916	0.006041057
Prevalence	American S	Female	55+ years	Non-melanoma skin cancer (basal-cell	Rate	1990	0.0253408	0.054813031	0.007193175
Prevalence	American S	Both	55+ years	Non-melanoma skin cancer (basal-cell	Rate	1990	0.0244754	0.053930089	0.006353379
Prevalence	American S	Male	55+ years	Non-melanoma skin cancer (basal-cell	Number	2021	0.0010118	0.002219652	0.00026467
Prevalence	American S	Female	55+ years	Non-melanoma skin cancer (basal-cell	Number	2021	0.0010635	0.002263918	0.000305595
Prevalence	American S	Both	55+ years	Non-melanoma skin cancer (basal-cell	Number	2021	0.0020754	0.004495455	0.000553141
Prevalence	American S	Male	55+ years	Non-melanoma skin cancer (basal-cell	Percent	2021	2.37E-07	5.21E-07	6.21E-08
Prevalence	American S	Female	55+ years	Non-melanoma skin cancer (basal-cell	Percent	2021	2.54E-07	5.41E-07	7.31E-08
Prevalence	American S	Both	55+ years	Non-melanoma skin cancer (basal-cell	Percent	2021	2.46E-07	5.32E-07	6.55E-08
Prevalence	American S	Male	55+ years	Non-melanoma skin cancer (basal-cell	Rate	2021	0.0237345	0.052065932	0.006208305
Prevalence	American S	Female	55+ years	Non-melanoma skin cancer (basal-cell	Rate	2021	0.0254251	0.054121186	0.007305541
Prevalence	American S	Both	55+ years	Non-melanoma skin cancer (basal-cell	Rate	2021	0.0245718	0.053224527	0.00654899
Prevalence	Cook Islan	Male	55+ years	Non-melanoma skin cancer (basal-cell	Number	1990	0.0002901	0.000633707	7.37E-05
Prevalence	Cook Islan	Female	55+ years	Non-melanoma skin cancer (basal-cell	Number	1990	0.0002828	0.000608602	8.79E-05
Prevalence	Cook Islan	Both	55+ years	Non-melanoma skin cancer (basal-cell	Number	1990	0.0005729	0.001235189	0.000199149

Prevalence	Cook Islan	Male	55+ years	Non-melanoma skin cancer (basal-cell	Percent	1990	2.54E-07	5.56E-07	6.46E-08
Prevalence	Cook Islan	Female	55+ years	Non-melanoma skin cancer (basal-cell	Percent	1990	2.85E-07	6.13E-07	8.86E-08
Prevalence	Cook Islan	Both	55+ years	Non-melanoma skin cancer (basal-cell	Percent	1990	2.69E-07	5.79E-07	9.34E-08
Prevalence	Cook Islan	Male	55+ years	Non-melanoma skin cancer (basal-cell	Rate	1990	0.0254196	0.055518258	0.006452798
Prevalence	Cook Islan	Female	55+ years	Non-melanoma skin cancer (basal-cell	Rate	1990	0.0284879	0.061316719	0.008857217
Prevalence	Cook Islan	Both	55+ years	Non-melanoma skin cancer (basal-cell	Rate	1990	0.0268467	0.057881593	0.009332218
Prevalence	Cook Islan	Male	55+ years	Non-melanoma skin cancer (basal-cell	Number	2021	0.0005955	0.001276158	0.000157386
Prevalence	Cook Islan	Female	55+ years	Non-melanoma skin cancer (basal-cell	Number	2021	0.0006798	0.001422315	0.000215869
Prevalence	Cook Islan	Both	55+ years	Non-melanoma skin cancer (basal-cell	Number	2021	0.0012753	0.00267987	0.000459868
Prevalence	Cook Islan	Male	55+ years	Non-melanoma skin cancer (basal-cell	Percent	2021	2.55E-07	5.47E-07	6.75E-08
Prevalence	Cook Islan	Female	55+ years	Non-melanoma skin cancer (basal-cell	Percent	2021	2.86E-07	5.98E-07	9.07E-08
Prevalence	Cook Islan	Both	55+ years	Non-melanoma skin cancer (basal-cell	Percent	2021	2.71E-07	5.69E-07	9.76E-08
Prevalence	Cook Islan	Male	55+ years	Non-melanoma skin cancer (basal-cell	Rate	2021	0.0255211	0.054692594	0.006745133
Prevalence	Cook Islan	Female	55+ years	Non-melanoma skin cancer (basal-cell	Rate	2021	0.0285677	0.059771021	0.009071644
Prevalence	Cook Islan	Both	55+ years	Non-melanoma skin cancer (basal-cell	Rate	2021	0.0270594	0.05686202	0.009757564
Prevalence	Guam	Male	55+ years	Non-melanoma skin cancer (basal-cell	Number	1990	0.0017058	0.003851617	0.000420614
Prevalence	Guam	Female	55+ years	Non-melanoma skin cancer (basal-cell	Number	1990	0.0017808	0.003947612	0.000513034
Prevalence	Guam	Both	55+ years	Non-melanoma skin cancer (basal-cell	Number	1990	0.0034866	0.007727911	0.00089615
Prevalence	Guam	Male	55+ years	Non-melanoma skin cancer (basal-cell	Percent	1990	2.57E-07	5.81E-07	6.34E-08
Prevalence	Guam	Female	55+ years	Non-melanoma skin cancer (basal-cell	Percent	1990	2.85E-07	6.31E-07	8.20E-08
Prevalence	Guam	Both	55+ years	Non-melanoma skin cancer (basal-cell	Percent	1990	2.71E-07	6.00E-07	6.95E-08
Prevalence	Guam	Male	55+ years	Non-melanoma skin cancer (basal-cell	Rate	1990	0.0256995	0.058029567	0.006337091
Prevalence	Guam	Female	55+ years	Non-melanoma skin cancer (basal-cell	Rate	1990	0.0284651	0.063100394	0.008200563
Prevalence	Guam	Both	55+ years	Non-melanoma skin cancer (basal-cell	Rate	1990	0.0270414	0.059936867	0.006950449
Prevalence	Guam	Male	55+ years	Non-melanoma skin cancer (basal-cell	Number	2021	0.0047062	0.009983138	0.001254464
Prevalence	Guam	Female	55+ years	Non-melanoma skin cancer (basal-cell	Number	2021	0.005702	0.011728703	0.001786323
Prevalence	Guam	Both	55+ years	Non-melanoma skin cancer (basal-cell	Number	2021	0.0104082	0.021815217	0.002883189
Prevalence	Guam	Male	55+ years	Non-melanoma skin cancer (basal-cell	Percent	2021	2.61E-07	5.54E-07	6.96E-08
Prevalence	Guam	Female	55+ years	Non-melanoma skin cancer (basal-cell	Percent	2021	2.90E-07	5.97E-07	9.09E-08
Prevalence	Guam	Both	55+ years	Non-melanoma skin cancer (basal-cell	Percent	2021	2.76E-07	5.79E-07	7.65E-08
Prevalence	Guam	Male	55+ years	Non-melanoma skin cancer (basal-cell	Rate	2021	0.0261099	0.055385939	0.0069597
Prevalence	Guam	Female	55+ years	Non-melanoma skin cancer (basal-cell	Rate	2021	0.0290017	0.059655419	0.009085732
Prevalence	Guam	Both	55+ years	Non-melanoma skin cancer (basal-cell	Rate	2021	0.0276186	0.05788767	0.007650674
Prevalence	Greenland	Male	55+ years	Non-melanoma skin cancer (basal-cell	Number	1990	0.7109677	0.940668886	0.519935838
Prevalence	Greenland	Female	55+ years	Non-melanoma skin cancer (basal-cell	Number	1990	0.7084622	0.932458286	0.521422914
Prevalence	Greenland	Both	55+ years	Non-melanoma skin cancer (basal-cell	Number	1990	1.4194299	1.831539416	1.054321963
Prevalence	Greenland	Male	55+ years	Non-melanoma skin cancer (basal-cell	Percent	1990	0.0002643	0.000349671	0.000193254
Prevalence	Greenland	Female	55+ years	Non-melanoma skin cancer (basal-cell	Percent	1990	0.0002517	0.000331254	0.000185238
Prevalence	Greenland	Both	55+ years	Non-melanoma skin cancer (basal-cell	Percent	1990	0.0002579	0.000332787	0.000191538
Prevalence	Greenland	Male	55+ years	Non-melanoma skin cancer (basal-cell	Rate	1990	26.394371	34.92193128	19.30239626
Prevalence	Greenland	Female	55+ years	Non-melanoma skin cancer (basal-cell	Rate	1990	25.153325	33.10610856	18.5126604
Prevalence	Greenland	Both	55+ years	Non-melanoma skin cancer (basal-cell	Rate	1990	25.760003	33.23902285	19.13397633
Prevalence	Greenland	Male	55+ years	Non-melanoma skin cancer (basal-cell	Number	2021	2.1629924	2.778376124	1.607280358
Prevalence	Greenland	Female	55+ years	Non-melanoma skin cancer (basal-cell	Number	2021	1.5163125	1.974679713	1.155537604
Prevalence	Greenland	Both	55+ years	Non-melanoma skin cancer (basal-cell	Number	2021	3.6793049	4.751817488	2.755826009
Prevalence	Greenland	Male	55+ years	Non-melanoma skin cancer (basal-cell	Percent	2021	0.0002948	0.00037869	0.000219064
Prevalence	Greenland	Female	55+ years	Non-melanoma skin cancer (basal-cell	Percent	2021	0.0002437	0.00031742	0.000185724
Prevalence	Greenland	Both	55+ years	Non-melanoma skin cancer (basal-cell	Percent	2021	0.0002714	0.000350422	0.000203222
Prevalence	Greenland	Male	55+ years	Non-melanoma skin cancer (basal-cell	Rate	2021	29.433385	37.80735155	21.87141368
Prevalence	Greenland	Female	55+ years	Non-melanoma skin cancer (basal-cell	Rate	2021	24.349735	31.71043428	18.55622408
Prevalence	Greenland	Both	55+ years	Non-melanoma skin cancer (basal-cell	Rate	2021	27.101547	35.00161241	20.29925478
Prevalence	Nauru	Male	55+ years	Non-melanoma skin cancer (basal-cell	Number	1990	8.15E-05	0.000179265	2.04E-05
Prevalence	Nauru	Female	55+ years	Non-melanoma skin cancer (basal-cell	Number	1990	6.60E-05	0.000147014	1.76E-05
Prevalence	Nauru	Both	55+ years	Non-melanoma skin cancer (basal-cell	Number	1990	0.0001475	0.000320768	3.78E-05
Prevalence	Nauru	Male	55+ years	Non-melanoma skin cancer (basal-cell	Percent	1990	1.99E-07	4.37E-07	4.96E-08
Prevalence	Nauru	Female	55+ years	Non-melanoma skin cancer (basal-cell	Percent	1990	2.12E-07	4.71E-07	5.65E-08

Prevalence	Nauru	Both	55+ years	Non-melanoma skin cancer (basal-cell	Percent	1990	2.04E-07	4.44E-07	5.23E-08
Prevalence	Nauru	Male	55+ years	Non-melanoma skin cancer (basal-cell	Rate	1990	0.0198457	0.043659969	0.004959337
Prevalence	Nauru	Female	55+ years	Non-melanoma skin cancer (basal-cell	Rate	1990	0.0211523	0.047094501	0.005646401
Prevalence	Nauru	Both	55+ years	Non-melanoma skin cancer (basal-cell	Rate	1990	0.02041	0.044380939	0.00522831
Prevalence	Nauru	Male	55+ years	Non-melanoma skin cancer (basal-cell	Number	2021	8.05E-05	0.000176247	1.99E-05
Prevalence	Nauru	Female	55+ years	Non-melanoma skin cancer (basal-cell	Number	2021	0.0001105	0.000243847	3.15E-05
Prevalence	Nauru	Both	55+ years	Non-melanoma skin cancer (basal-cell	Number	2021	0.000191	0.0004173	4.89E-05
Prevalence	Nauru	Male	55+ years	Non-melanoma skin cancer (basal-cell	Percent	2021	1.99E-07	4.35E-07	4.92E-08
Prevalence	Nauru	Female	55+ years	Non-melanoma skin cancer (basal-cell	Percent	2021	2.13E-07	4.70E-07	6.07E-08
Prevalence	Nauru	Both	55+ years	Non-melanoma skin cancer (basal-cell	Percent	2021	2.07E-07	4.52E-07	5.30E-08
Prevalence	Nauru	Male	55+ years	Non-melanoma skin cancer (basal-cell	Rate	2021	0.0198766	0.043500194	0.004917052
Prevalence	Nauru	Female	55+ years	Non-melanoma skin cancer (basal-cell	Rate	2021	0.0212822	0.046973737	0.006074478
Prevalence	Nauru	Both	55+ years	Non-melanoma skin cancer (basal-cell	Rate	2021	0.0206661	0.04514877	0.005293972
Prevalence	Niue	Male	55+ years	Non-melanoma skin cancer (basal-cell	Number	1990	3.74E-05	7.85E-05	1.02E-05
Prevalence	Niue	Female	55+ years	Non-melanoma skin cancer (basal-cell	Number	1990	5.51E-05	0.000112496	1.73E-05
Prevalence	Niue	Both	55+ years	Non-melanoma skin cancer (basal-cell	Number	1990	9.24E-05	0.000189682	2.80E-05
Prevalence	Niue	Male	55+ years	Non-melanoma skin cancer (basal-cell	Percent	1990	2.37E-07	4.97E-07	6.46E-08
Prevalence	Niue	Female	55+ years	Non-melanoma skin cancer (basal-cell	Percent	1990	2.67E-07	5.46E-07	8.38E-08
Prevalence	Niue	Both	55+ years	Non-melanoma skin cancer (basal-cell	Percent	1990	2.54E-07	5.21E-07	7.70E-08
Prevalence	Niue	Male	55+ years	Non-melanoma skin cancer (basal-cell	Rate	1990	0.023645	0.049683523	0.006457175
Prevalence	Niue	Female	55+ years	Non-melanoma skin cancer (basal-cell	Rate	1990	0.0267173	0.054559309	0.008382221
Prevalence	Niue	Both	55+ years	Non-melanoma skin cancer (basal-cell	Rate	1990	0.0253845	0.052087507	0.007699073
Prevalence	Niue	Male	55+ years	Non-melanoma skin cancer (basal-cell	Number	2021	4.32E-05	9.51E-05	1.12E-05
Prevalence	Niue	Female	55+ years	Non-melanoma skin cancer (basal-cell	Number	2021	5.49E-05	0.00011711	1.65E-05
Prevalence	Niue	Both	55+ years	Non-melanoma skin cancer (basal-cell	Number	2021	9.81E-05	0.000209377	2.65E-05
Prevalence	Niue	Male	55+ years	Non-melanoma skin cancer (basal-cell	Percent	2021	2.35E-07	5.18E-07	6.12E-08
Prevalence	Niue	Female	55+ years	Non-melanoma skin cancer (basal-cell	Percent	2021	2.65E-07	5.64E-07	7.95E-08
Prevalence	Niue	Both	55+ years	Non-melanoma skin cancer (basal-cell	Percent	2021	2.51E-07	5.35E-07	6.78E-08
Prevalence	Niue	Male	55+ years	Non-melanoma skin cancer (basal-cell	Rate	2021	0.0235129	0.051745826	0.00612145
Prevalence	Niue	Female	55+ years	Non-melanoma skin cancer (basal-cell	Rate	2021	0.0264626	0.056423039	0.007946557
Prevalence	Niue	Both	55+ years	Non-melanoma skin cancer (basal-cell	Rate	2021	0.0250777	0.053513049	0.00677454
Prevalence	Northern M	Male	55+ years	Non-melanoma skin cancer (basal-cell	Number	1990	0.0003873	0.000868376	8.72E-05
Prevalence	Northern M	Female	55+ years	Non-melanoma skin cancer (basal-cell	Number	1990	0.0002607	0.000561053	8.01E-05
Prevalence	Northern M	Both	55+ years	Non-melanoma skin cancer (basal-cell	Number	1990	0.0006481	0.001424939	0.000212601
Prevalence	Northern M	Male	55+ years	Non-melanoma skin cancer (basal-cell	Percent	1990	2.61E-07	5.86E-07	5.88E-08
Prevalence	Northern M	Female	55+ years	Non-melanoma skin cancer (basal-cell	Percent	1990	2.72E-07	5.86E-07	8.36E-08
Prevalence	Northern M	Both	55+ years	Non-melanoma skin cancer (basal-cell	Percent	1990	2.66E-07	5.84E-07	8.71E-08
Prevalence	Northern M	Male	55+ years	Non-melanoma skin cancer (basal-cell	Rate	1990	0.0261137	0.05854399	0.005877482
Prevalence	Northern M	Female	55+ years	Non-melanoma skin cancer (basal-cell	Rate	1990	0.0272147	0.058562482	0.008356261
Prevalence	Northern M	Both	55+ years	Non-melanoma skin cancer (basal-cell	Rate	1990	0.0265457	0.058367329	0.008708415
Prevalence	Northern M	Male	55+ years	Non-melanoma skin cancer (basal-cell	Number	2021	0.0012566	0.002858208	0.000279861
Prevalence	Northern M	Female	55+ years	Non-melanoma skin cancer (basal-cell	Number	2021	0.0013302	0.002902203	0.000403386
Prevalence	Northern M	Both	55+ years	Non-melanoma skin cancer (basal-cell	Number	2021	0.0025869	0.005686774	0.000896998
Prevalence	Northern M	Male	55+ years	Non-melanoma skin cancer (basal-cell	Percent	2021	2.60E-07	5.91E-07	5.78E-08
Prevalence	Northern M	Female	55+ years	Non-melanoma skin cancer (basal-cell	Percent	2021	2.73E-07	5.95E-07	8.28E-08
Prevalence	Northern M	Both	55+ years	Non-melanoma skin cancer (basal-cell	Percent	2021	2.66E-07	5.85E-07	9.23E-08
Prevalence	Northern M	Male	55+ years	Non-melanoma skin cancer (basal-cell	Rate	2021	0.025942	0.05900459	0.005777434
Prevalence	Northern M	Female	55+ years	Non-melanoma skin cancer (basal-cell	Rate	2021	0.0272794	0.059516469	0.008272368
Prevalence	Northern M	Both	55+ years	Non-melanoma skin cancer (basal-cell	Rate	2021	0.0266129	0.058503825	0.009228047
Prevalence	Monaco	Male	55+ years	Non-melanoma skin cancer (basal-cell	Number	1990	1.3345568	1.714145445	0.996662308
Prevalence	Monaco	Female	55+ years	Non-melanoma skin cancer (basal-cell	Number	1990	1.3729545	1.8287094	1.051168928
Prevalence	Monaco	Both	55+ years	Non-melanoma skin cancer (basal-cell	Number	1990	2.7075113	3.507205199	2.045929699
Prevalence	Monaco	Male	55+ years	Non-melanoma skin cancer (basal-cell	Percent	1990	0.0002762	0.000354744	0.000206236
Prevalence	Monaco	Female	55+ years	Non-melanoma skin cancer (basal-cell	Percent	1990	0.00022	0.000293032	0.000168442
Prevalence	Monaco	Both	55+ years	Non-melanoma skin cancer (basal-cell	Percent	1990	0.0002445	0.000316734	0.000184759
Prevalence	Monaco	Male	55+ years	Non-melanoma skin cancer (basal-cell	Rate	1990	27.5826	35.42793183	20.59900132

Prevalence	Monaco	Female	55+ years	Non-melanoma skin cancer (basal-cell	Rate	1990	21.990123	29.28978809	16.83619887
Prevalence	Monaco	Both	55+ years	Non-melanoma skin cancer (basal-cell	Rate	1990	24.431819	31.64803192	18.46189338
Prevalence	Monaco	Male	55+ years	Non-melanoma skin cancer (basal-cell	Number	2021	2.0328554	2.611404748	1.532989963
Prevalence	Monaco	Female	55+ years	Non-melanoma skin cancer (basal-cell	Number	2021	1.7663023	2.279219219	1.329619255
Prevalence	Monaco	Both	55+ years	Non-melanoma skin cancer (basal-cell	Number	2021	3.7991577	4.870020684	2.89011649
Prevalence	Monaco	Male	55+ years	Non-melanoma skin cancer (basal-cell	Percent	2021	0.0002754	0.000353688	0.000207619
Prevalence	Monaco	Female	55+ years	Non-melanoma skin cancer (basal-cell	Percent	2021	0.0002142	0.000276331	0.000161219
Prevalence	Monaco	Both	55+ years	Non-melanoma skin cancer (basal-cell	Percent	2021	0.0002431	0.000311587	0.0001849
Prevalence	Monaco	Male	55+ years	Non-melanoma skin cancer (basal-cell	Rate	2021	27.494535	35.31946221	20.73381428
Prevalence	Monaco	Female	55+ years	Non-melanoma skin cancer (basal-cell	Rate	2021	21.405356	27.62126172	16.11330807
Prevalence	Monaco	Both	55+ years	Non-melanoma skin cancer (basal-cell	Rate	2021	24.282976	31.12758244	18.47268116
Prevalence	San Marino	Male	55+ years	Non-melanoma skin cancer (basal-cell	Number	1990	0.7719937	0.98233923	0.56935611
Prevalence	San Marino	Female	55+ years	Non-melanoma skin cancer (basal-cell	Number	1990	0.6995509	0.92786253	0.530746224
Prevalence	San Marino	Both	55+ years	Non-melanoma skin cancer (basal-cell	Number	1990	1.4715446	1.901721302	1.112888706
Prevalence	San Marino	Male	55+ years	Non-melanoma skin cancer (basal-cell	Percent	1990	0.0002851	0.000362743	0.000210167
Prevalence	San Marino	Female	55+ years	Non-melanoma skin cancer (basal-cell	Percent	1990	0.0002214	0.000293648	0.000167982
Prevalence	San Marino	Both	55+ years	Non-melanoma skin cancer (basal-cell	Percent	1990	0.0002508	0.00032411	0.000189616
Prevalence	San Marino	Male	55+ years	Non-melanoma skin cancer (basal-cell	Rate	1990	28.468445	36.22525753	20.99587505
Prevalence	San Marino	Female	55+ years	Non-melanoma skin cancer (basal-cell	Rate	1990	22.127927	29.34979282	16.78836166
Prevalence	San Marino	Both	55+ years	Non-melanoma skin cancer (basal-cell	Rate	1990	25.055475	32.37994241	18.94876613
Prevalence	San Marino	Male	55+ years	Non-melanoma skin cancer (basal-cell	Number	2021	1.7826545	2.27976857	1.332406259
Prevalence	San Marino	Female	55+ years	Non-melanoma skin cancer (basal-cell	Number	2021	1.3915052	1.750317305	1.077046893
Prevalence	San Marino	Both	55+ years	Non-melanoma skin cancer (basal-cell	Number	2021	3.1741597	4.028955258	2.416648916
Prevalence	San Marino	Male	55+ years	Non-melanoma skin cancer (basal-cell	Percent	2021	0.0003165	0.000404711	0.000236509
Prevalence	San Marino	Female	55+ years	Non-melanoma skin cancer (basal-cell	Percent	2021	0.0002251	0.000283069	0.000174192
Prevalence	San Marino	Both	55+ years	Non-melanoma skin cancer (basal-cell	Percent	2021	0.0002686	0.000340975	0.000204469
Prevalence	San Marino	Male	55+ years	Non-melanoma skin cancer (basal-cell	Rate	2021	31.60411	40.41728459	23.62180253
Prevalence	San Marino	Female	55+ years	Non-melanoma skin cancer (basal-cell	Rate	2021	22.492901	28.29289757	17.40985896
Prevalence	San Marino	Both	55+ years	Non-melanoma skin cancer (basal-cell	Rate	2021	26.838254	34.0657483	20.43332538
Prevalence	Sudan	Male	55+ years	Non-melanoma skin cancer (basal-cell	Number	1990	26.295991	35.03301658	19.04951881
Prevalence	Sudan	Female	55+ years	Non-melanoma skin cancer (basal-cell	Number	1990	13.714455	18.61104811	9.87779745
Prevalence	Sudan	Both	55+ years	Non-melanoma skin cancer (basal-cell	Number	1990	40.010445	53.50999233	29.12843832
Prevalence	Sudan	Male	55+ years	Non-melanoma skin cancer (basal-cell	Percent	1990	3.32E-05	4.42E-05	2.40E-05
Prevalence	Sudan	Female	55+ years	Non-melanoma skin cancer (basal-cell	Percent	1990	1.98E-05	2.68E-05	1.42E-05
Prevalence	Sudan	Both	55+ years	Non-melanoma skin cancer (basal-cell	Percent	1990	2.69E-05	3.60E-05	1.96E-05
Prevalence	Sudan	Male	55+ years	Non-melanoma skin cancer (basal-cell	Rate	1990	3.3173154	4.419516494	2.403151963
Prevalence	Sudan	Female	55+ years	Non-melanoma skin cancer (basal-cell	Rate	1990	1.9751389	2.680340324	1.422588275
Prevalence	Sudan	Both	55+ years	Non-melanoma skin cancer (basal-cell	Rate	1990	2.6906048	3.59841638	1.958816382
Prevalence	Sudan	Male	55+ years	Non-melanoma skin cancer (basal-cell	Number	2021	55.39336	74.27399722	40.73213126
Prevalence	Sudan	Female	55+ years	Non-melanoma skin cancer (basal-cell	Number	2021	26.07855	35.27632666	18.40472429
Prevalence	Sudan	Both	55+ years	Non-melanoma skin cancer (basal-cell	Number	2021	81.47191	107.3215006	60.74488588
Prevalence	Sudan	Male	55+ years	Non-melanoma skin cancer (basal-cell	Percent	2021	3.24E-05	4.34E-05	2.38E-05
Prevalence	Sudan	Female	55+ years	Non-melanoma skin cancer (basal-cell	Percent	2021	1.86E-05	2.52E-05	1.31E-05
Prevalence	Sudan	Both	55+ years	Non-melanoma skin cancer (basal-cell	Percent	2021	2.62E-05	3.45E-05	1.95E-05
Prevalence	Sudan	Male	55+ years	Non-melanoma skin cancer (basal-cell	Rate	2021	3.2369778	4.340290578	2.3802312
Prevalence	Sudan	Female	55+ years	Non-melanoma skin cancer (basal-cell	Rate	2021	1.8593438	2.515125188	1.31221672
Prevalence	Sudan	Both	55+ years	Non-melanoma skin cancer (basal-cell	Rate	2021	2.6164489	3.446601733	1.950806015
Incidence	Lao People	Male	55+ years	Non-melanoma skin cancer (basal-cell	Number	1990	7.6516369	10.55471012	5.156519194
Incidence	Lao People	Female	55+ years	Non-melanoma skin cancer (basal-cell	Number	1990	6.0951216	8.389541754	4.140122984
Incidence	Lao People	Both	55+ years	Non-melanoma skin cancer (basal-cell	Number	1990	13.746759	18.69542387	9.340349473
Incidence	Lao People	Male	55+ years	Non-melanoma skin cancer (basal-cell	Percent	1990	1.21E-05	1.70E-05	8.13E-06
Incidence	Lao People	Female	55+ years	Non-melanoma skin cancer (basal-cell	Percent	1990	8.93E-06	1.23E-05	6.00E-06
Incidence	Lao People	Both	55+ years	Non-melanoma skin cancer (basal-cell	Percent	1990	1.05E-05	1.45E-05	7.02E-06
Incidence	Lao People	Male	55+ years	Non-melanoma skin cancer (basal-cell	Rate	1990	4.6711322	6.443385507	3.147925493
Incidence	Lao People	Female	55+ years	Non-melanoma skin cancer (basal-cell	Rate	1990	3.4026638	4.68354718	2.311265847
Incidence	Lao People	Both	55+ years	Non-melanoma skin cancer (basal-cell	Rate	1990	4.0085628	5.451596465	2.723651335

Incidence	Lao People	Male	55+ years	Non-melanoma skin cancer (basal-cell	Number	2021	17.193104	23.92630798	12.0828261
Incidence	Lao People	Female	55+ years	Non-melanoma skin cancer (basal-cell	Number	2021	13.633574	18.58258597	9.391983389
Incidence	Lao People	Both	55+ years	Non-melanoma skin cancer (basal-cell	Number	2021	30.826678	42.1781865	21.7610162
Incidence	Lao People	Male	55+ years	Non-melanoma skin cancer (basal-cell	Percent	2021	1.19E-05	1.65E-05	8.09E-06
Incidence	Lao People	Female	55+ years	Non-melanoma skin cancer (basal-cell	Percent	2021	8.65E-06	1.20E-05	5.92E-06
Incidence	Lao People	Both	55+ years	Non-melanoma skin cancer (basal-cell	Percent	2021	1.02E-05	1.41E-05	6.99E-06
Incidence	Lao People	Male	55+ years	Non-melanoma skin cancer (basal-cell	Rate	2021	4.5991007	6.400211559	3.23211769
Incidence	Lao People	Female	55+ years	Non-melanoma skin cancer (basal-cell	Rate	2021	3.4142592	4.653641412	2.352036627
Incidence	Lao People	Both	55+ years	Non-melanoma skin cancer (basal-cell	Rate	2021	3.9871587	5.45537614	2.814595373
Incidence	Democratic	Male	55+ years	Non-melanoma skin cancer (basal-cell	Number	1990	7.1912171	11.61175422	3.947851421
Incidence	Democratic	Female	55+ years	Non-melanoma skin cancer (basal-cell	Number	1990	9.4227521	15.21017931	5.07802284
Incidence	Democratic	Both	55+ years	Non-melanoma skin cancer (basal-cell	Number	1990	16.613969	26.50052229	9.106285349
Incidence	Democratic	Male	55+ years	Non-melanoma skin cancer (basal-cell	Percent	1990	2.68E-06	4.30E-06	1.45E-06
Incidence	Democratic	Female	55+ years	Non-melanoma skin cancer (basal-cell	Percent	1990	1.98E-06	3.19E-06	1.09E-06
Incidence	Democratic	Both	55+ years	Non-melanoma skin cancer (basal-cell	Percent	1990	2.23E-06	3.58E-06	1.25E-06
Incidence	Democratic	Male	55+ years	Non-melanoma skin cancer (basal-cell	Rate	1990	0.7165766	1.157065757	0.393387907
Incidence	Democratic	Female	55+ years	Non-melanoma skin cancer (basal-cell	Rate	1990	0.568381	0.917478932	0.306306644
Incidence	Democratic	Both	55+ years	Non-melanoma skin cancer (basal-cell	Rate	1990	0.6242626	0.995745489	0.342164674
Incidence	Democratic	Male	55+ years	Non-melanoma skin cancer (basal-cell	Number	2021	17.664322	27.74509233	10.11869195
Incidence	Democratic	Female	55+ years	Non-melanoma skin cancer (basal-cell	Number	2021	20.583847	31.97777778	11.76053811
Incidence	Democratic	Both	55+ years	Non-melanoma skin cancer (basal-cell	Number	2021	38.24817	59.90771484	21.66923224
Incidence	Democratic	Male	55+ years	Non-melanoma skin cancer (basal-cell	Percent	2021	2.62E-06	4.16E-06	1.50E-06
Incidence	Democratic	Female	55+ years	Non-melanoma skin cancer (basal-cell	Percent	2021	2.04E-06	3.12E-06	1.15E-06
Incidence	Democratic	Both	55+ years	Non-melanoma skin cancer (basal-cell	Percent	2021	2.27E-06	3.53E-06	1.32E-06
Incidence	Democratic	Male	55+ years	Non-melanoma skin cancer (basal-cell	Rate	2021	0.7475028	1.174091633	0.428193621
Incidence	Democratic	Female	55+ years	Non-melanoma skin cancer (basal-cell	Rate	2021	0.6289642	0.97711947	0.35935739
Incidence	Democratic	Both	55+ years	Non-melanoma skin cancer (basal-cell	Rate	2021	0.6786681	1.06299091	0.384494667
Incidence	Timor-Lest	Male	55+ years	Non-melanoma skin cancer (basal-cell	Number	1990	0.9539569	1.335875574	0.663291097
Incidence	Timor-Lest	Female	55+ years	Non-melanoma skin cancer (basal-cell	Number	1990	0.6664566	0.935287357	0.453729455
Incidence	Timor-Lest	Both	55+ years	Non-melanoma skin cancer (basal-cell	Number	1990	1.6204134	2.235616495	1.133445644
Incidence	Timor-Lest	Male	55+ years	Non-melanoma skin cancer (basal-cell	Percent	1990	1.16E-05	1.65E-05	7.89E-06
Incidence	Timor-Lest	Female	55+ years	Non-melanoma skin cancer (basal-cell	Percent	1990	8.05E-06	1.12E-05	5.41E-06
Incidence	Timor-Lest	Both	55+ years	Non-melanoma skin cancer (basal-cell	Percent	1990	9.82E-06	1.38E-05	6.67E-06
Incidence	Timor-Lest	Male	55+ years	Non-melanoma skin cancer (basal-cell	Rate	1990	4.3033906	6.026262348	2.992169512
Incidence	Timor-Lest	Female	55+ years	Non-melanoma skin cancer (basal-cell	Rate	1990	3.1297636	4.392226816	2.130770469
Incidence	Timor-Lest	Both	55+ years	Non-melanoma skin cancer (basal-cell	Rate	1990	3.7283698	5.143875587	2.607917499
Incidence	Timor-Lest	Male	55+ years	Non-melanoma skin cancer (basal-cell	Number	2021	3.5224377	4.770926607	2.571561593
Incidence	Timor-Lest	Female	55+ years	Non-melanoma skin cancer (basal-cell	Number	2021	2.6917053	3.72383709	1.888516005
Incidence	Timor-Lest	Both	55+ years	Non-melanoma skin cancer (basal-cell	Number	2021	6.214143	8.397426465	4.41071083
Incidence	Timor-Lest	Male	55+ years	Non-melanoma skin cancer (basal-cell	Percent	2021	1.28E-05	1.75E-05	9.16E-06
Incidence	Timor-Lest	Female	55+ years	Non-melanoma skin cancer (basal-cell	Percent	2021	9.30E-06	1.30E-05	6.39E-06
Incidence	Timor-Lest	Both	55+ years	Non-melanoma skin cancer (basal-cell	Percent	2021	1.10E-05	1.51E-05	7.72E-06
Incidence	Timor-Lest	Male	55+ years	Non-melanoma skin cancer (basal-cell	Rate	2021	4.9891018	6.757433552	3.642302223
Incidence	Timor-Lest	Female	55+ years	Non-melanoma skin cancer (basal-cell	Rate	2021	3.7683938	5.213380727	2.643926869
Incidence	Timor-Lest	Both	55+ years	Non-melanoma skin cancer (basal-cell	Rate	2021	4.375199	5.912386018	3.105454409
Incidence	Maldives	Male	55+ years	Non-melanoma skin cancer (basal-cell	Number	1990	0.3985677	0.5441547	0.27557435
Incidence	Maldives	Female	55+ years	Non-melanoma skin cancer (basal-cell	Number	1990	0.1847278	0.263990702	0.123468647
Incidence	Maldives	Both	55+ years	Non-melanoma skin cancer (basal-cell	Number	1990	0.5832955	0.804263863	0.398005356
Incidence	Maldives	Male	55+ years	Non-melanoma skin cancer (basal-cell	Percent	1990	1.20E-05	1.66E-05	8.25E-06
Incidence	Maldives	Female	55+ years	Non-melanoma skin cancer (basal-cell	Percent	1990	7.73E-06	1.11E-05	5.10E-06
Incidence	Maldives	Both	55+ years	Non-melanoma skin cancer (basal-cell	Percent	1990	1.02E-05	1.43E-05	6.94E-06
Incidence	Maldives	Male	55+ years	Non-melanoma skin cancer (basal-cell	Rate	1990	4.3601889	5.952858265	3.014685063
Incidence	Maldives	Female	55+ years	Non-melanoma skin cancer (basal-cell	Rate	1990	2.9848906	4.265646254	1.995045908
Incidence	Maldives	Both	55+ years	Non-melanoma skin cancer (basal-cell	Rate	1990	3.8049711	5.246398434	2.596280613
Incidence	Maldives	Male	55+ years	Non-melanoma skin cancer (basal-cell	Number	2021	1.3491924	1.813276054	0.931241714
Incidence	Maldives	Female	55+ years	Non-melanoma skin cancer (basal-cell	Number	2021	0.8614222	1.159546165	0.598362356

Incidence	Maldives	Both	55+ years	Non-melanoma skin cancer (basal-cell	Number	2021	2.2106146	2.967452558	1.519678404
Incidence	Maldives	Male	55+ years	Non-melanoma skin cancer (basal-cell	Percent	2021	1.16E-05	1.57E-05	7.87E-06
Incidence	Maldives	Female	55+ years	Non-melanoma skin cancer (basal-cell	Percent	2021	8.05E-06	1.11E-05	5.52E-06
Incidence	Maldives	Both	55+ years	Non-melanoma skin cancer (basal-cell	Percent	2021	9.88E-06	1.35E-05	6.73E-06
Incidence	Maldives	Male	55+ years	Non-melanoma skin cancer (basal-cell	Rate	2021	4.5965625	6.177648688	3.172646626
Incidence	Maldives	Female	55+ years	Non-melanoma skin cancer (basal-cell	Rate	2021	3.4396078	4.629999154	2.389225446
Incidence	Maldives	Both	55+ years	Non-melanoma skin cancer (basal-cell	Rate	2021	4.0638985	5.455236799	2.793711235
Incidence	Georgia	Male	55+ years	Non-melanoma skin cancer (basal-cell	Number	1990	433.63469	557.9962292	329.1853282
Incidence	Georgia	Female	55+ years	Non-melanoma skin cancer (basal-cell	Number	1990	687.91954	878.5499795	521.5505147
Incidence	Georgia	Both	55+ years	Non-melanoma skin cancer (basal-cell	Number	1990	1121.5542	1421.961015	849.5733736
Incidence	Georgia	Male	55+ years	Non-melanoma skin cancer (basal-cell	Percent	1990	0.0003243	0.000420754	0.00024203
Incidence	Georgia	Female	55+ years	Non-melanoma skin cancer (basal-cell	Percent	1990	0.0003243	0.000409558	0.000240254
Incidence	Georgia	Both	55+ years	Non-melanoma skin cancer (basal-cell	Percent	1990	0.0003243	0.000413498	0.000240328
Incidence	Georgia	Male	55+ years	Non-melanoma skin cancer (basal-cell	Rate	1990	98.2013	126.3643261	74.54760442
Incidence	Georgia	Female	55+ years	Non-melanoma skin cancer (basal-cell	Rate	1990	102.80195	131.2895597	77.93994538
Incidence	Georgia	Both	55+ years	Non-melanoma skin cancer (basal-cell	Rate	1990	100.97296	128.0184361	76.48666419
Incidence	Georgia	Male	55+ years	Non-melanoma skin cancer (basal-cell	Number	2021	464.304	586.7685917	357.7491105
Incidence	Georgia	Female	55+ years	Non-melanoma skin cancer (basal-cell	Number	2021	675.4831	847.5188868	517.2894336
Incidence	Georgia	Both	55+ years	Non-melanoma skin cancer (basal-cell	Number	2021	1139.7871	1429.716173	888.9918725
Incidence	Georgia	Male	55+ years	Non-melanoma skin cancer (basal-cell	Percent	2021	0.0002832	0.000363104	0.000213076
Incidence	Georgia	Female	55+ years	Non-melanoma skin cancer (basal-cell	Percent	2021	0.0002764	0.000354894	0.000204986
Incidence	Georgia	Both	55+ years	Non-melanoma skin cancer (basal-cell	Percent	2021	0.0002791	0.000357062	0.000206235
Incidence	Georgia	Male	55+ years	Non-melanoma skin cancer (basal-cell	Rate	2021	109.02038	137.7755356	84.00087531
Incidence	Georgia	Female	55+ years	Non-melanoma skin cancer (basal-cell	Rate	2021	108.25376	135.8244321	82.90144875
Incidence	Georgia	Both	55+ years	Non-melanoma skin cancer (basal-cell	Rate	2021	108.56474	136.1804955	84.67649453
Incidence	Solomon Is	Male	55+ years	Non-melanoma skin cancer (basal-cell	Number	1990	0.0211264	0.045370324	0.004865327
Incidence	Solomon Is	Female	55+ years	Non-melanoma skin cancer (basal-cell	Number	1990	0.0163964	0.036212166	0.003650197
Incidence	Solomon Is	Both	55+ years	Non-melanoma skin cancer (basal-cell	Number	1990	0.0375228	0.082144577	0.009011872
Incidence	Solomon Is	Male	55+ years	Non-melanoma skin cancer (basal-cell	Percent	1990	3.81E-07	8.32E-07	8.76E-08
Incidence	Solomon Is	Female	55+ years	Non-melanoma skin cancer (basal-cell	Percent	1990	3.60E-07	7.82E-07	8.38E-08
Incidence	Solomon Is	Both	55+ years	Non-melanoma skin cancer (basal-cell	Percent	1990	3.72E-07	8.03E-07	8.72E-08
Incidence	Solomon Is	Male	55+ years	Non-melanoma skin cancer (basal-cell	Rate	1990	0.1658284	0.356126865	0.038189581
Incidence	Solomon Is	Female	55+ years	Non-melanoma skin cancer (basal-cell	Rate	1990	0.166136	0.366919336	0.036985575
Incidence	Solomon Is	Both	55+ years	Non-melanoma skin cancer (basal-cell	Rate	1990	0.1659627	0.363324045	0.039859355
Incidence	Solomon Is	Male	55+ years	Non-melanoma skin cancer (basal-cell	Number	2021	0.0456415	0.096473045	0.01084043
Incidence	Solomon Is	Female	55+ years	Non-melanoma skin cancer (basal-cell	Number	2021	0.0439441	0.095353621	0.012274302
Incidence	Solomon Is	Both	55+ years	Non-melanoma skin cancer (basal-cell	Number	2021	0.0895856	0.192483612	0.028960815
Incidence	Solomon Is	Male	55+ years	Non-melanoma skin cancer (basal-cell	Percent	2021	4.03E-07	8.64E-07	9.58E-08
Incidence	Solomon Is	Female	55+ years	Non-melanoma skin cancer (basal-cell	Percent	2021	3.62E-07	7.79E-07	9.95E-08
Incidence	Solomon Is	Both	55+ years	Non-melanoma skin cancer (basal-cell	Percent	2021	3.82E-07	8.04E-07	1.22E-07
Incidence	Solomon Is	Male	55+ years	Non-melanoma skin cancer (basal-cell	Rate	2021	0.1662838	0.351476458	0.039494514
Incidence	Solomon Is	Female	55+ years	Non-melanoma skin cancer (basal-cell	Rate	2021	0.163984	0.355826511	0.04580342
Incidence	Solomon Is	Both	55+ years	Non-melanoma skin cancer (basal-cell	Rate	2021	0.1651477	0.354836472	0.0533882
Incidence	China	Male	55+ years	Non-melanoma skin cancer (basal-cell	Number	1990	9688.3225	12186.30934	7427.285782
Incidence	China	Female	55+ years	Non-melanoma skin cancer (basal-cell	Number	1990	9091.6711	11303.84628	7028.055856
Incidence	China	Both	55+ years	Non-melanoma skin cancer (basal-cell	Number	1990	18779.994	23488.37934	14482.43011
Incidence	China	Male	55+ years	Non-melanoma skin cancer (basal-cell	Percent	1990	4.79E-05	6.05E-05	3.56E-05
Incidence	China	Female	55+ years	Non-melanoma skin cancer (basal-cell	Percent	1990	4.12E-05	5.20E-05	3.09E-05
Incidence	China	Both	55+ years	Non-melanoma skin cancer (basal-cell	Percent	1990	4.44E-05	5.59E-05	3.31E-05
Incidence	China	Male	55+ years	Non-melanoma skin cancer (basal-cell	Rate	1990	13.715	17.25120385	10.51422686
Incidence	China	Female	55+ years	Non-melanoma skin cancer (basal-cell	Rate	1990	12.475248	15.51071158	9.643633205
Incidence	China	Both	55+ years	Non-melanoma skin cancer (basal-cell	Rate	1990	13.085461	16.36615357	10.09101871
Incidence	China	Male	55+ years	Non-melanoma skin cancer (basal-cell	Number	2021	288419.09	341213.9951	238099.8749
Incidence	China	Female	55+ years	Non-melanoma skin cancer (basal-cell	Number	2021	223147.09	271064.5234	177342.5916
Incidence	China	Both	55+ years	Non-melanoma skin cancer (basal-cell	Number	2021	511566.18	611458.3194	416716.9369
Incidence	China	Male	55+ years	Non-melanoma skin cancer (basal-cell	Percent	2021	0.0005609	0.000667554	0.000445424

Incidence	China	Female	55+ years	Non-melanoma skin cancer (basal-cell	Percent	2021	0.0003845	0.000465009	0.000300823
Incidence	China	Both	55+ years	Non-melanoma skin cancer (basal-cell	Percent	2021	0.0004674	0.000558733	0.000369093
Incidence	China	Male	55+ years	Non-melanoma skin cancer (basal-cell	Rate	2021	156.92555	185.6506548	129.5474345
Incidence	China	Female	55+ years	Non-melanoma skin cancer (basal-cell	Rate	2021	114.33969	138.8923884	90.86964171
Incidence	China	Both	55+ years	Non-melanoma skin cancer (basal-cell	Rate	2021	134.99387	161.3537538	109.9647186
Incidence	Philippine	Male	55+ years	Non-melanoma skin cancer (basal-cell	Number	1990	269.17902	345.8546835	196.9273722
Incidence	Philippine	Female	55+ years	Non-melanoma skin cancer (basal-cell	Number	1990	235.82894	305.7454768	173.0551431
Incidence	Philippine	Both	55+ years	Non-melanoma skin cancer (basal-cell	Number	1990	505.00797	648.4159748	369.3512308
Incidence	Philippine	Male	55+ years	Non-melanoma skin cancer (basal-cell	Percent	1990	2.83E-05	3.68E-05	2.03E-05
Incidence	Philippine	Female	55+ years	Non-melanoma skin cancer (basal-cell	Percent	1990	2.28E-05	3.03E-05	1.65E-05
Incidence	Philippine	Both	55+ years	Non-melanoma skin cancer (basal-cell	Percent	1990	2.55E-05	3.32E-05	1.83E-05
Incidence	Philippine	Male	55+ years	Non-melanoma skin cancer (basal-cell	Rate	1990	11.758874	15.10839022	8.602617592
Incidence	Philippine	Female	55+ years	Non-melanoma skin cancer (basal-cell	Rate	1990	9.4906161	12.30431221	6.964369616
Incidence	Philippine	Both	55+ years	Non-melanoma skin cancer (basal-cell	Rate	1990	10.578252	13.58217731	7.736690797
Incidence	Philippine	Male	55+ years	Non-melanoma skin cancer (basal-cell	Number	2021	504.07944	669.4452704	363.6760185
Incidence	Philippine	Female	55+ years	Non-melanoma skin cancer (basal-cell	Number	2021	488.62708	641.5213356	351.1296769
Incidence	Philippine	Both	55+ years	Non-melanoma skin cancer (basal-cell	Number	2021	992.70651	1303.786966	715.8868955
Incidence	Philippine	Male	55+ years	Non-melanoma skin cancer (basal-cell	Percent	2021	1.86E-05	2.51E-05	1.31E-05
Incidence	Philippine	Female	55+ years	Non-melanoma skin cancer (basal-cell	Percent	2021	1.53E-05	2.04E-05	1.10E-05
Incidence	Philippine	Both	55+ years	Non-melanoma skin cancer (basal-cell	Percent	2021	1.68E-05	2.24E-05	1.19E-05
Incidence	Philippine	Male	55+ years	Non-melanoma skin cancer (basal-cell	Rate	2021	7.8259443	10.39328521	5.646150253
Incidence	Philippine	Female	55+ years	Non-melanoma skin cancer (basal-cell	Rate	2021	6.5116073	8.549127132	4.679271103
Incidence	Philippine	Both	55+ years	Non-melanoma skin cancer (basal-cell	Rate	2021	7.1186904	9.349445873	5.133619183
Incidence	Vanuatu	Male	55+ years	Non-melanoma skin cancer (basal-cell	Number	1990	0.0090617	0.019282404	0.002093921
Incidence	Vanuatu	Female	55+ years	Non-melanoma skin cancer (basal-cell	Number	1990	0.0075373	0.016148887	0.002084127
Incidence	Vanuatu	Both	55+ years	Non-melanoma skin cancer (basal-cell	Number	1990	0.016599	0.035321665	0.00412337
Incidence	Vanuatu	Male	55+ years	Non-melanoma skin cancer (basal-cell	Percent	1990	3.88E-07	8.30E-07	8.63E-08
Incidence	Vanuatu	Female	55+ years	Non-melanoma skin cancer (basal-cell	Percent	1990	3.73E-07	8.00E-07	1.02E-07
Incidence	Vanuatu	Both	55+ years	Non-melanoma skin cancer (basal-cell	Percent	1990	3.81E-07	8.16E-07	9.26E-08
Incidence	Vanuatu	Male	55+ years	Non-melanoma skin cancer (basal-cell	Rate	1990	0.165612	0.352405032	0.038268481
Incidence	Vanuatu	Female	55+ years	Non-melanoma skin cancer (basal-cell	Rate	1990	0.1676801	0.359261768	0.046365255
Incidence	Vanuatu	Both	55+ years	Non-melanoma skin cancer (basal-cell	Rate	1990	0.1665447	0.354397529	0.041371553
Incidence	Vanuatu	Male	55+ years	Non-melanoma skin cancer (basal-cell	Number	2021	0.0234072	0.04973108	0.005490228
Incidence	Vanuatu	Female	55+ years	Non-melanoma skin cancer (basal-cell	Number	2021	0.0238747	0.051397141	0.006509761
Incidence	Vanuatu	Both	55+ years	Non-melanoma skin cancer (basal-cell	Number	2021	0.047282	0.100246694	0.015291276
Incidence	Vanuatu	Male	55+ years	Non-melanoma skin cancer (basal-cell	Percent	2021	4.12E-07	8.89E-07	9.50E-08
Incidence	Vanuatu	Female	55+ years	Non-melanoma skin cancer (basal-cell	Percent	2021	3.69E-07	7.89E-07	1.01E-07
Incidence	Vanuatu	Both	55+ years	Non-melanoma skin cancer (basal-cell	Percent	2021	3.89E-07	8.25E-07	1.26E-07
Incidence	Vanuatu	Male	55+ years	Non-melanoma skin cancer (basal-cell	Rate	2021	0.1660287	0.35274498	0.038942458
Incidence	Vanuatu	Female	55+ years	Non-melanoma skin cancer (basal-cell	Rate	2021	0.1637903	0.352605185	0.044659598
Incidence	Vanuatu	Both	55+ years	Non-melanoma skin cancer (basal-cell	Rate	2021	0.1648909	0.349599685	0.053326698
Incidence	Kyrgyzstan	Male	55+ years	Non-melanoma skin cancer (basal-cell	Number	1990	212.77551	239.1541041	185.3562864
Incidence	Kyrgyzstan	Female	55+ years	Non-melanoma skin cancer (basal-cell	Number	1990	344.87773	381.8568229	307.6461961
Incidence	Kyrgyzstan	Both	55+ years	Non-melanoma skin cancer (basal-cell	Number	1990	557.65324	615.5441108	498.2054107
Incidence	Kyrgyzstan	Male	55+ years	Non-melanoma skin cancer (basal-cell	Percent	1990	0.0003381	0.000382719	0.000287957
Incidence	Kyrgyzstan	Female	55+ years	Non-melanoma skin cancer (basal-cell	Percent	1990	0.0003531	0.000395222	0.000305747
Incidence	Kyrgyzstan	Both	55+ years	Non-melanoma skin cancer (basal-cell	Percent	1990	0.0003472	0.000388959	0.000302146
Incidence	Kyrgyzstan	Male	55+ years	Non-melanoma skin cancer (basal-cell	Rate	1990	104.58017	117.5453762	91.10349373
Incidence	Kyrgyzstan	Female	55+ years	Non-melanoma skin cancer (basal-cell	Rate	1990	110.72942	122.6022463	98.77554211
Incidence	Kyrgyzstan	Both	55+ years	Non-melanoma skin cancer (basal-cell	Rate	1990	108.29969	119.5424575	96.75455927
Incidence	Kyrgyzstan	Male	55+ years	Non-melanoma skin cancer (basal-cell	Number	2021	342.41486	454.2729684	253.6434533
Incidence	Kyrgyzstan	Female	55+ years	Non-melanoma skin cancer (basal-cell	Number	2021	456.40031	589.1150816	343.8838933
Incidence	Kyrgyzstan	Both	55+ years	Non-melanoma skin cancer (basal-cell	Number	2021	798.81517	1035.878179	604.4512777
Incidence	Kyrgyzstan	Male	55+ years	Non-melanoma skin cancer (basal-cell	Percent	2021	0.0002698	0.000354265	0.000194612
Incidence	Kyrgyzstan	Female	55+ years	Non-melanoma skin cancer (basal-cell	Percent	2021	0.0002693	0.000347026	0.000198775
Incidence	Kyrgyzstan	Both	55+ years	Non-melanoma skin cancer (basal-cell	Percent	2021	0.0002695	0.000347843	0.000194933

Incidence	Kyrgyzstan	Male	55+ years	Non-melanoma skin cancer (basal-cell	Rate	2021	93.746998	124.3717258	69.44299184
Incidence	Kyrgyzstan	Female	55+ years	Non-melanoma skin cancer (basal-cell	Rate	2021	94.985213	122.6055727	71.56849823
Incidence	Kyrgyzstan	Both	55+ years	Non-melanoma skin cancer (basal-cell	Rate	2021	94.450465	122.4803676	71.46922892
Incidence	Bosnia and	Male	55+ years	Non-melanoma skin cancer (basal-cell	Number	1990	370.04851	471.0027789	277.1873228
Incidence	Bosnia and	Female	55+ years	Non-melanoma skin cancer (basal-cell	Number	1990	413.76023	525.9796391	313.8388089
Incidence	Bosnia and	Both	55+ years	Non-melanoma skin cancer (basal-cell	Number	1990	783.80875	997.0088853	587.7671558
Incidence	Bosnia and	Male	55+ years	Non-melanoma skin cancer (basal-cell	Percent	1990	0.0003513	0.000448297	0.000257747
Incidence	Bosnia and	Female	55+ years	Non-melanoma skin cancer (basal-cell	Percent	1990	0.0002771	0.000352508	0.000203865
Incidence	Bosnia and	Both	55+ years	Non-melanoma skin cancer (basal-cell	Percent	1990	0.0003078	0.000389324	0.000226922
Incidence	Bosnia and	Male	55+ years	Non-melanoma skin cancer (basal-cell	Rate	1990	114.78113	146.0949894	85.97757972
Incidence	Bosnia and	Female	55+ years	Non-melanoma skin cancer (basal-cell	Rate	1990	100.63546	127.9296514	76.33240232
Incidence	Bosnia and	Both	55+ years	Non-melanoma skin cancer (basal-cell	Rate	1990	106.85254	135.9169995	80.1272179
Incidence	Bosnia and	Male	55+ years	Non-melanoma skin cancer (basal-cell	Number	2021	678.07511	864.2627517	510.9199081
Incidence	Bosnia and	Female	55+ years	Non-melanoma skin cancer (basal-cell	Number	2021	723.40047	918.0853378	559.1998582
Incidence	Bosnia and	Both	55+ years	Non-melanoma skin cancer (basal-cell	Number	2021	1401.4756	1785.26686	1075.114984
Incidence	Bosnia and	Male	55+ years	Non-melanoma skin cancer (basal-cell	Percent	2021	0.0003622	0.00046916	0.000263134
Incidence	Bosnia and	Female	55+ years	Non-melanoma skin cancer (basal-cell	Percent	2021	0.0002884	0.000374048	0.000216955
Incidence	Bosnia and	Both	55+ years	Non-melanoma skin cancer (basal-cell	Percent	2021	0.0003199	0.000412581	0.000237197
Incidence	Bosnia and	Male	55+ years	Non-melanoma skin cancer (basal-cell	Rate	2021	138.04952	175.9555148	104.0183385
Incidence	Bosnia and	Female	55+ years	Non-melanoma skin cancer (basal-cell	Rate	2021	119.75123	151.9792338	92.56957113
Incidence	Bosnia and	Both	55+ years	Non-melanoma skin cancer (basal-cell	Rate	2021	127.95725	162.9980905	98.15994091
Incidence	Kiribati	Male	55+ years	Non-melanoma skin cancer (basal-cell	Number	1990	0.0043999	0.009508688	0.001010228
Incidence	Kiribati	Female	55+ years	Non-melanoma skin cancer (basal-cell	Number	1990	0.0054178	0.01154311	0.001492868
Incidence	Kiribati	Both	55+ years	Non-melanoma skin cancer (basal-cell	Number	1990	0.0098177	0.020766828	0.003161309
Incidence	Kiribati	Male	55+ years	Non-melanoma skin cancer (basal-cell	Percent	1990	3.79E-07	8.23E-07	8.47E-08
Incidence	Kiribati	Female	55+ years	Non-melanoma skin cancer (basal-cell	Percent	1990	3.31E-07	6.90E-07	9.55E-08
Incidence	Kiribati	Both	55+ years	Non-melanoma skin cancer (basal-cell	Percent	1990	3.51E-07	7.43E-07	1.13E-07
Incidence	Kiribati	Male	55+ years	Non-melanoma skin cancer (basal-cell	Rate	1990	0.1659918	0.358727989	0.038112196
Incidence	Kiribati	Female	55+ years	Non-melanoma skin cancer (basal-cell	Rate	1990	0.1638213	0.349035855	0.045140745
Incidence	Kiribati	Both	55+ years	Non-melanoma skin cancer (basal-cell	Rate	1990	0.164787	0.348564802	0.053061603
Incidence	Kiribati	Male	55+ years	Non-melanoma skin cancer (basal-cell	Number	2021	0.0091805	0.020090342	0.002077316
Incidence	Kiribati	Female	55+ years	Non-melanoma skin cancer (basal-cell	Number	2021	0.0116111	0.025151469	0.003099638
Incidence	Kiribati	Both	55+ years	Non-melanoma skin cancer (basal-cell	Number	2021	0.0207916	0.044669598	0.006712645
Incidence	Kiribati	Male	55+ years	Non-melanoma skin cancer (basal-cell	Percent	2021	4.02E-07	8.74E-07	8.76E-08
Incidence	Kiribati	Female	55+ years	Non-melanoma skin cancer (basal-cell	Percent	2021	3.37E-07	7.23E-07	9.34E-08
Incidence	Kiribati	Both	55+ years	Non-melanoma skin cancer (basal-cell	Percent	2021	3.63E-07	7.84E-07	1.18E-07
Incidence	Kiribati	Male	55+ years	Non-melanoma skin cancer (basal-cell	Rate	2021	0.1673851	0.366300997	0.037875065
Incidence	Kiribati	Female	55+ years	Non-melanoma skin cancer (basal-cell	Rate	2021	0.1637165	0.354636134	0.043704946
Incidence	Kiribati	Both	55+ years	Non-melanoma skin cancer (basal-cell	Rate	2021	0.1653164	0.355173341	0.053373045
Incidence	Indonesia	Male	55+ years	Non-melanoma skin cancer (basal-cell	Number	1990	364.12384	493.4107708	254.4505814
Incidence	Indonesia	Female	55+ years	Non-melanoma skin cancer (basal-cell	Number	1990	290.40366	392.5812533	201.3971533
Incidence	Indonesia	Both	55+ years	Non-melanoma skin cancer (basal-cell	Number	1990	654.5275	883.6755568	458.1626757
Incidence	Indonesia	Male	55+ years	Non-melanoma skin cancer (basal-cell	Percent	1990	9.58E-06	1.30E-05	6.73E-06
Incidence	Indonesia	Female	55+ years	Non-melanoma skin cancer (basal-cell	Percent	1990	6.81E-06	9.32E-06	4.70E-06
Incidence	Indonesia	Both	55+ years	Non-melanoma skin cancer (basal-cell	Percent	1990	8.12E-06	1.10E-05	5.66E-06
Incidence	Indonesia	Male	55+ years	Non-melanoma skin cancer (basal-cell	Rate	1990	4.7000832	6.368909155	3.284428985
Incidence	Indonesia	Female	55+ years	Non-melanoma skin cancer (basal-cell	Rate	1990	3.4536821	4.668849061	2.395154894
Incidence	Indonesia	Both	55+ years	Non-melanoma skin cancer (basal-cell	Rate	1990	4.0513715	5.46974417	2.835919366
Incidence	Indonesia	Male	55+ years	Non-melanoma skin cancer (basal-cell	Number	2021	955.63097	1313.893171	660.471561
Incidence	Indonesia	Female	55+ years	Non-melanoma skin cancer (basal-cell	Number	2021	749.64531	1027.441839	516.0154423
Incidence	Indonesia	Both	55+ years	Non-melanoma skin cancer (basal-cell	Number	2021	1705.2763	2337.646031	1188.908619
Incidence	Indonesia	Male	55+ years	Non-melanoma skin cancer (basal-cell	Percent	2021	1.05E-05	1.43E-05	7.28E-06
Incidence	Indonesia	Female	55+ years	Non-melanoma skin cancer (basal-cell	Percent	2021	7.42E-06	1.02E-05	5.01E-06
Incidence	Indonesia	Both	55+ years	Non-melanoma skin cancer (basal-cell	Percent	2021	8.86E-06	1.21E-05	6.10E-06
Incidence	Indonesia	Male	55+ years	Non-melanoma skin cancer (basal-cell	Rate	2021	4.6813759	6.436404873	3.235470332
Incidence	Indonesia	Female	55+ years	Non-melanoma skin cancer (basal-cell	Rate	2021	3.4922309	4.786349082	2.403863601

Incidence	Indonesia	Both	55+ years	Non-melanoma skin cancer (basal-cell	Rate	2021	4.0718591	5.581831744	2.838876281
Incidence	Thailand	Male	55+ years	Non-melanoma skin cancer (basal-cell	Number	1990	701.22737	769.1919756	637.7449728
Incidence	Thailand	Female	55+ years	Non-melanoma skin cancer (basal-cell	Number	1990	728.50995	802.2271205	665.1037529
Incidence	Thailand	Both	55+ years	Non-melanoma skin cancer (basal-cell	Number	1990	1429.7373	1557.412373	1310.743087
Incidence	Thailand	Male	55+ years	Non-melanoma skin cancer (basal-cell	Percent	1990	5.69E-05	6.54E-05	4.92E-05
Incidence	Thailand	Female	55+ years	Non-melanoma skin cancer (basal-cell	Percent	1990	5.20E-05	5.90E-05	4.51E-05
Incidence	Thailand	Both	55+ years	Non-melanoma skin cancer (basal-cell	Percent	1990	5.43E-05	6.15E-05	4.76E-05
Incidence	Thailand	Male	55+ years	Non-melanoma skin cancer (basal-cell	Rate	1990	25.340819	27.79691064	23.04670431
Incidence	Thailand	Female	55+ years	Non-melanoma skin cancer (basal-cell	Rate	1990	23.002446	25.33003957	21.0004174
Incidence	Thailand	Both	55+ years	Non-melanoma skin cancer (basal-cell	Rate	1990	24.092841	26.24432302	22.08764074
Incidence	Thailand	Male	55+ years	Non-melanoma skin cancer (basal-cell	Number	2021	1028.8948	1344.643666	767.015986
Incidence	Thailand	Female	55+ years	Non-melanoma skin cancer (basal-cell	Number	2021	1028.7301	1318.161206	766.0251825
Incidence	Thailand	Both	55+ years	Non-melanoma skin cancer (basal-cell	Number	2021	2057.6249	2619.402015	1537.008585
Incidence	Thailand	Male	55+ years	Non-melanoma skin cancer (basal-cell	Percent	2021	2.44E-05	3.23E-05	1.75E-05
Incidence	Thailand	Female	55+ years	Non-melanoma skin cancer (basal-cell	Percent	2021	1.99E-05	2.56E-05	1.46E-05
Incidence	Thailand	Both	55+ years	Non-melanoma skin cancer (basal-cell	Percent	2021	2.19E-05	2.85E-05	1.60E-05
Incidence	Thailand	Male	55+ years	Non-melanoma skin cancer (basal-cell	Rate	2021	11.562807	15.111219	8.619790385
Incidence	Thailand	Female	55+ years	Non-melanoma skin cancer (basal-cell	Rate	2021	9.7605502	12.5066616	7.268016757
Incidence	Thailand	Both	55+ years	Non-melanoma skin cancer (basal-cell	Rate	2021	10.585587	13.47568599	7.907241787
Incidence	Tajikistan	Male	55+ years	Non-melanoma skin cancer (basal-cell	Number	1990	204.44657	261.5889426	155.8937889
Incidence	Tajikistan	Female	55+ years	Non-melanoma skin cancer (basal-cell	Number	1990	268.87232	353.5646363	203.8458963
Incidence	Tajikistan	Both	55+ years	Non-melanoma skin cancer (basal-cell	Number	1990	473.31889	606.7034927	359.243193
Incidence	Tajikistan	Male	55+ years	Non-melanoma skin cancer (basal-cell	Percent	1990	0.0003272	0.000419872	0.000243025
Incidence	Tajikistan	Female	55+ years	Non-melanoma skin cancer (basal-cell	Percent	1990	0.0003297	0.000427549	0.000245934
Incidence	Tajikistan	Both	55+ years	Non-melanoma skin cancer (basal-cell	Percent	1990	0.0003286	0.000422693	0.000247774
Incidence	Tajikistan	Male	55+ years	Non-melanoma skin cancer (basal-cell	Rate	1990	99.556565	127.3824083	75.91347731
Incidence	Tajikistan	Female	55+ years	Non-melanoma skin cancer (basal-cell	Rate	1990	102.24872	134.4561323	77.52000056
Incidence	Tajikistan	Both	55+ years	Non-melanoma skin cancer (basal-cell	Rate	1990	101.06821	129.5499423	76.70952197
Incidence	Tajikistan	Male	55+ years	Non-melanoma skin cancer (basal-cell	Number	2021	472.20778	604.7929387	353.7250921
Incidence	Tajikistan	Female	55+ years	Non-melanoma skin cancer (basal-cell	Number	2021	482.63632	632.8672746	354.8848135
Incidence	Tajikistan	Both	55+ years	Non-melanoma skin cancer (basal-cell	Number	2021	954.8441	1231.942392	709.3846439
Incidence	Tajikistan	Male	55+ years	Non-melanoma skin cancer (basal-cell	Percent	2021	0.0002645	0.000341319	0.000191752
Incidence	Tajikistan	Female	55+ years	Non-melanoma skin cancer (basal-cell	Percent	2021	0.0002514	0.000328964	0.00018209
Incidence	Tajikistan	Both	55+ years	Non-melanoma skin cancer (basal-cell	Percent	2021	0.0002577	0.000333611	0.000187004
Incidence	Tajikistan	Male	55+ years	Non-melanoma skin cancer (basal-cell	Rate	2021	94.801765	121.4199352	71.01484659
Incidence	Tajikistan	Female	55+ years	Non-melanoma skin cancer (basal-cell	Rate	2021	91.22788	119.6245242	67.08030048
Incidence	Tajikistan	Both	55+ years	Non-melanoma skin cancer (basal-cell	Rate	2021	92.960988	119.9385133	69.06373233
Incidence	Micronesia	Male	55+ years	Non-melanoma skin cancer (basal-cell	Number	1990	0.0064556	0.013510595	0.001520303
Incidence	Micronesia	Female	55+ years	Non-melanoma skin cancer (basal-cell	Number	1990	0.0066684	0.013816105	0.001909968
Incidence	Micronesia	Both	55+ years	Non-melanoma skin cancer (basal-cell	Number	1990	0.013124	0.027328601	0.004333629
Incidence	Micronesia	Male	55+ years	Non-melanoma skin cancer (basal-cell	Percent	1990	3.91E-07	8.46E-07	9.03E-08
Incidence	Micronesia	Female	55+ years	Non-melanoma skin cancer (basal-cell	Percent	1990	3.78E-07	7.82E-07	1.08E-07
Incidence	Micronesia	Both	55+ years	Non-melanoma skin cancer (basal-cell	Percent	1990	3.84E-07	8.01E-07	1.28E-07
Incidence	Micronesia	Male	55+ years	Non-melanoma skin cancer (basal-cell	Rate	1990	0.1653024	0.345953496	0.038929002
Incidence	Micronesia	Female	55+ years	Non-melanoma skin cancer (basal-cell	Rate	1990	0.1644117	0.340640178	0.047090835
Incidence	Micronesia	Both	55+ years	Non-melanoma skin cancer (basal-cell	Rate	1990	0.1648486	0.343270458	0.054434063
Incidence	Micronesia	Male	55+ years	Non-melanoma skin cancer (basal-cell	Number	2021	0.0105084	0.023138914	0.002333839
Incidence	Micronesia	Female	55+ years	Non-melanoma skin cancer (basal-cell	Number	2021	0.0112426	0.024915037	0.003162348
Incidence	Micronesia	Both	55+ years	Non-melanoma skin cancer (basal-cell	Number	2021	0.021751	0.047590714	0.006693539
Incidence	Micronesia	Male	55+ years	Non-melanoma skin cancer (basal-cell	Percent	2021	4.26E-07	9.38E-07	9.24E-08
Incidence	Micronesia	Female	55+ years	Non-melanoma skin cancer (basal-cell	Percent	2021	3.96E-07	8.61E-07	1.10E-07
Incidence	Micronesia	Both	55+ years	Non-melanoma skin cancer (basal-cell	Percent	2021	4.10E-07	8.88E-07	1.25E-07
Incidence	Micronesia	Male	55+ years	Non-melanoma skin cancer (basal-cell	Rate	2021	0.1663813	0.366360994	0.036951927
Incidence	Micronesia	Female	55+ years	Non-melanoma skin cancer (basal-cell	Rate	2021	0.1642864	0.364080014	0.046210962
Incidence	Micronesia	Both	55+ years	Non-melanoma skin cancer (basal-cell	Rate	2021	0.1652919	0.361654532	0.050865986
Incidence	Malaysia	Male	55+ years	Non-melanoma skin cancer (basal-cell	Number	1990	102.21549	126.1909385	78.30984263

Incidence	Malaysia	Female	55+ years	Non-melanoma skin cancer (basal-cell	Number	1990	93.693469	115.6110601	74.44620748
Incidence	Malaysia	Both	55+ years	Non-melanoma skin cancer (basal-cell	Number	1990	195.90896	239.2871359	156.4419017
Incidence	Malaysia	Male	55+ years	Non-melanoma skin cancer (basal-cell	Percent	1990	3.97E-05	5.03E-05	3.00E-05
Incidence	Malaysia	Female	55+ years	Non-melanoma skin cancer (basal-cell	Percent	1990	3.29E-05	4.06E-05	2.57E-05
Incidence	Malaysia	Both	55+ years	Non-melanoma skin cancer (basal-cell	Percent	1990	3.61E-05	4.50E-05	2.81E-05
Incidence	Malaysia	Male	55+ years	Non-melanoma skin cancer (basal-cell	Rate	1990	14.401437	17.77940817	11.03330138
Incidence	Malaysia	Female	55+ years	Non-melanoma skin cancer (basal-cell	Rate	1990	12.229474	15.09029894	9.717197687
Incidence	Malaysia	Both	55+ years	Non-melanoma skin cancer (basal-cell	Rate	1990	13.273978	16.21310353	10.59985418
Incidence	Malaysia	Male	55+ years	Non-melanoma skin cancer (basal-cell	Number	2021	244.73859	326.2183519	180.7909993
Incidence	Malaysia	Female	55+ years	Non-melanoma skin cancer (basal-cell	Number	2021	210.56706	276.2010195	151.9029244
Incidence	Malaysia	Both	55+ years	Non-melanoma skin cancer (basal-cell	Number	2021	455.30565	599.8053348	335.3199074
Incidence	Malaysia	Male	55+ years	Non-melanoma skin cancer (basal-cell	Percent	2021	2.41E-05	3.20E-05	1.76E-05
Incidence	Malaysia	Female	55+ years	Non-melanoma skin cancer (basal-cell	Percent	2021	1.87E-05	2.46E-05	1.34E-05
Incidence	Malaysia	Both	55+ years	Non-melanoma skin cancer (basal-cell	Percent	2021	2.13E-05	2.81E-05	1.55E-05
Incidence	Malaysia	Male	55+ years	Non-melanoma skin cancer (basal-cell	Rate	2021	10.086755	13.44489535	7.451193506
Incidence	Malaysia	Female	55+ years	Non-melanoma skin cancer (basal-cell	Rate	2021	8.5027657	11.15308604	6.133888963
Incidence	Malaysia	Both	55+ years	Non-melanoma skin cancer (basal-cell	Rate	2021	9.2866645	12.23395962	6.839369321
Incidence	Taiwan (Pr	Male	55+ years	Non-melanoma skin cancer (basal-cell	Number	1990	173.25631	190.4468632	152.8454576
Incidence	Taiwan (Pr	Female	55+ years	Non-melanoma skin cancer (basal-cell	Number	1990	92.161196	109.0476496	72.75136099
Incidence	Taiwan (Pr	Both	55+ years	Non-melanoma skin cancer (basal-cell	Number	1990	265.41751	294.0210055	231.1549021
Incidence	Taiwan (Pr	Male	55+ years	Non-melanoma skin cancer (basal-cell	Percent	1990	3.77E-05	4.24E-05	3.30E-05
Incidence	Taiwan (Pr	Female	55+ years	Non-melanoma skin cancer (basal-cell	Percent	1990	2.21E-05	2.64E-05	1.76E-05
Incidence	Taiwan (Pr	Both	55+ years	Non-melanoma skin cancer (basal-cell	Percent	1990	3.03E-05	3.42E-05	2.62E-05
Incidence	Taiwan (Pr	Male	55+ years	Non-melanoma skin cancer (basal-cell	Rate	1990	11.460609	12.59773476	10.11046599
Incidence	Taiwan (Pr	Female	55+ years	Non-melanoma skin cancer (basal-cell	Rate	1990	7.3035507	8.641761149	5.765368508
Incidence	Taiwan (Pr	Both	55+ years	Non-melanoma skin cancer (basal-cell	Rate	1990	9.5693428	10.60061103	8.334041312
Incidence	Taiwan (Pr	Male	55+ years	Non-melanoma skin cancer (basal-cell	Number	2021	9.3703173	16.92427817	3.826169614
Incidence	Taiwan (Pr	Female	55+ years	Non-melanoma skin cancer (basal-cell	Number	2021	9.2918656	16.78859226	3.709408921
Incidence	Taiwan (Pr	Both	55+ years	Non-melanoma skin cancer (basal-cell	Number	2021	18.662183	33.96855162	7.995811222
Incidence	Taiwan (Pr	Male	55+ years	Non-melanoma skin cancer (basal-cell	Percent	2021	7.34E-07	1.32E-06	3.05E-07
Incidence	Taiwan (Pr	Female	55+ years	Non-melanoma skin cancer (basal-cell	Percent	2021	6.47E-07	1.16E-06	2.57E-07
Incidence	Taiwan (Pr	Both	55+ years	Non-melanoma skin cancer (basal-cell	Percent	2021	6.88E-07	1.22E-06	2.94E-07
Incidence	Taiwan (Pr	Male	55+ years	Non-melanoma skin cancer (basal-cell	Rate	2021	0.2650468	0.478716503	0.108226213
Incidence	Taiwan (Pr	Female	55+ years	Non-melanoma skin cancer (basal-cell	Rate	2021	0.2326112	0.42028319	0.092860806
Incidence	Taiwan (Pr	Both	55+ years	Non-melanoma skin cancer (basal-cell	Rate	2021	0.2478399	0.451113471	0.106186987
Incidence	Samoa	Male	55+ years	Non-melanoma skin cancer (basal-cell	Number	1990	0.0148865	0.030724569	0.004485226
Incidence	Samoa	Female	55+ years	Non-melanoma skin cancer (basal-cell	Number	1990	0.0141482	0.02764313	0.005430474
Incidence	Samoa	Both	55+ years	Non-melanoma skin cancer (basal-cell	Number	1990	0.0290348	0.058716232	0.011002018
Incidence	Samoa	Male	55+ years	Non-melanoma skin cancer (basal-cell	Percent	1990	5.55E-07	1.16E-06	1.59E-07
Incidence	Samoa	Female	55+ years	Non-melanoma skin cancer (basal-cell	Percent	1990	4.87E-07	9.55E-07	1.88E-07
Incidence	Samoa	Both	55+ years	Non-melanoma skin cancer (basal-cell	Percent	1990	5.20E-07	1.04E-06	1.94E-07
Incidence	Samoa	Male	55+ years	Non-melanoma skin cancer (basal-cell	Rate	1990	0.2074071	0.428071128	0.062490572
Incidence	Samoa	Female	55+ years	Non-melanoma skin cancer (basal-cell	Rate	1990	0.1955657	0.382100358	0.075063349
Incidence	Samoa	Both	55+ years	Non-melanoma skin cancer (basal-cell	Rate	1990	0.201463	0.407413076	0.076339469
Incidence	Samoa	Male	55+ years	Non-melanoma skin cancer (basal-cell	Number	2021	0.020334	0.042494785	0.004909251
Incidence	Samoa	Female	55+ years	Non-melanoma skin cancer (basal-cell	Number	2021	0.0204845	0.043054992	0.006246247
Incidence	Samoa	Both	55+ years	Non-melanoma skin cancer (basal-cell	Number	2021	0.0408186	0.085685174	0.01295165
Incidence	Samoa	Male	55+ years	Non-melanoma skin cancer (basal-cell	Percent	2021	4.57E-07	9.68E-07	1.08E-07
Incidence	Samoa	Female	55+ years	Non-melanoma skin cancer (basal-cell	Percent	2021	4.09E-07	8.49E-07	1.22E-07
Incidence	Samoa	Both	55+ years	Non-melanoma skin cancer (basal-cell	Percent	2021	4.31E-07	9.01E-07	1.36E-07
Incidence	Samoa	Male	55+ years	Non-melanoma skin cancer (basal-cell	Rate	2021	0.1663746	0.347695753	0.040167885
Incidence	Samoa	Female	55+ years	Non-melanoma skin cancer (basal-cell	Rate	2021	0.165202	0.347226051	0.050374179
Incidence	Samoa	Both	55+ years	Non-melanoma skin cancer (basal-cell	Rate	2021	0.165784	0.348009117	0.052602943
Incidence	Uzbekistan	Male	55+ years	Non-melanoma skin cancer (basal-cell	Number	1990	839.95199	1088.869681	633.0836282
Incidence	Uzbekistan	Female	55+ years	Non-melanoma skin cancer (basal-cell	Number	1990	1210.1701	1534.90181	924.1672419
Incidence	Uzbekistan	Both	55+ years	Non-melanoma skin cancer (basal-cell	Number	1990	2050.1221	2607.121538	1569.62643

Incidence	Uzbekistan	Male	55+ years	Non-melanoma skin cancer (basal-cell	Percent	1990	0.0003467	0.00044675	0.000257177
Incidence	Uzbekistan	Female	55+ years	Non-melanoma skin cancer (basal-cell	Percent	1990	0.0003322	0.00041845	0.000248574
Incidence	Uzbekistan	Both	55+ years	Non-melanoma skin cancer (basal-cell	Percent	1990	0.000338	0.000426595	0.000253999
Incidence	Uzbekistan	Male	55+ years	Non-melanoma skin cancer (basal-cell	Rate	1990	103.03639	133.570964	77.65997345
Incidence	Uzbekistan	Female	55+ years	Non-melanoma skin cancer (basal-cell	Rate	1990	106.10985	134.5828994	81.03261473
Incidence	Uzbekistan	Both	55+ years	Non-melanoma skin cancer (basal-cell	Rate	1990	104.82872	133.3097285	80.25957755
Incidence	Uzbekistan	Male	55+ years	Non-melanoma skin cancer (basal-cell	Number	2021	2045.5216	2650.221482	1550.444969
Incidence	Uzbekistan	Female	55+ years	Non-melanoma skin cancer (basal-cell	Number	2021	2443.043	3183.908798	1783.061812
Incidence	Uzbekistan	Both	55+ years	Non-melanoma skin cancer (basal-cell	Number	2021	4488.5646	5781.396366	3376.668054
Incidence	Uzbekistan	Male	55+ years	Non-melanoma skin cancer (basal-cell	Percent	2021	0.0002919	0.000379444	0.00021696
Incidence	Uzbekistan	Female	55+ years	Non-melanoma skin cancer (basal-cell	Percent	2021	0.000272	0.000357493	0.000196131
Incidence	Uzbekistan	Both	55+ years	Non-melanoma skin cancer (basal-cell	Percent	2021	0.0002807	0.000366373	0.000205117
Incidence	Uzbekistan	Male	55+ years	Non-melanoma skin cancer (basal-cell	Rate	2021	96.549184	125.0911839	73.18142958
Incidence	Uzbekistan	Female	55+ years	Non-melanoma skin cancer (basal-cell	Rate	2021	96.562234	125.8452479	70.47621968
Incidence	Uzbekistan	Both	55+ years	Non-melanoma skin cancer (basal-cell	Rate	2021	96.556287	124.36719	72.63759319
Incidence	Azerbaijan	Male	55+ years	Non-melanoma skin cancer (basal-cell	Number	1990	333.35147	432.8886199	244.1630452
Incidence	Azerbaijan	Female	55+ years	Non-melanoma skin cancer (basal-cell	Number	1990	521.27896	669.6838639	386.6272068
Incidence	Azerbaijan	Both	55+ years	Non-melanoma skin cancer (basal-cell	Number	1990	854.63043	1098.762287	639.2829289
Incidence	Azerbaijan	Male	55+ years	Non-melanoma skin cancer (basal-cell	Percent	1990	0.0003122	0.000407636	0.000225648
Incidence	Azerbaijan	Female	55+ years	Non-melanoma skin cancer (basal-cell	Percent	1990	0.0003316	0.000426301	0.000244136
Incidence	Azerbaijan	Both	55+ years	Non-melanoma skin cancer (basal-cell	Percent	1990	0.0003237	0.00041214	0.000238937
Incidence	Azerbaijan	Male	55+ years	Non-melanoma skin cancer (basal-cell	Rate	1990	92.042185	119.5255404	67.41623267
Incidence	Azerbaijan	Female	55+ years	Non-melanoma skin cancer (basal-cell	Rate	1990	102.20551	131.3027882	75.80476845
Incidence	Azerbaijan	Both	55+ years	Non-melanoma skin cancer (basal-cell	Rate	1990	97.985302	125.9755691	73.29522656
Incidence	Azerbaijan	Male	55+ years	Non-melanoma skin cancer (basal-cell	Number	2021	806.98907	1061.238358	602.4713001
Incidence	Azerbaijan	Female	55+ years	Non-melanoma skin cancer (basal-cell	Number	2021	991.15692	1284.656806	756.1622418
Incidence	Azerbaijan	Both	55+ years	Non-melanoma skin cancer (basal-cell	Number	2021	1798.146	2332.880923	1350.334654
Incidence	Azerbaijan	Male	55+ years	Non-melanoma skin cancer (basal-cell	Percent	2021	0.0002587	0.000339288	0.000185624
Incidence	Azerbaijan	Female	55+ years	Non-melanoma skin cancer (basal-cell	Percent	2021	0.0002507	0.000331603	0.000184502
Incidence	Azerbaijan	Both	55+ years	Non-melanoma skin cancer (basal-cell	Percent	2021	0.0002542	0.000333283	0.000187323
Incidence	Azerbaijan	Male	55+ years	Non-melanoma skin cancer (basal-cell	Rate	2021	93.893605	123.4756444	70.09785451
Incidence	Azerbaijan	Female	55+ years	Non-melanoma skin cancer (basal-cell	Rate	2021	93.830472	121.615409	71.58408367
Incidence	Azerbaijan	Both	55+ years	Non-melanoma skin cancer (basal-cell	Rate	2021	93.858795	121.7706423	70.48414539
Incidence	Hungary	Male	55+ years	Non-melanoma skin cancer (basal-cell	Number	1990	1561.8821	1733.816246	1412.543889
Incidence	Hungary	Female	55+ years	Non-melanoma skin cancer (basal-cell	Number	1990	1908.6974	2101.833791	1724.782232
Incidence	Hungary	Both	55+ years	Non-melanoma skin cancer (basal-cell	Number	1990	3470.5795	3823.422847	3160.8365
Incidence	Hungary	Male	55+ years	Non-melanoma skin cancer (basal-cell	Percent	1990	0.000435	0.000491933	0.000382104
Incidence	Hungary	Female	55+ years	Non-melanoma skin cancer (basal-cell	Percent	1990	0.0003404	0.000380996	0.000301874
Incidence	Hungary	Both	55+ years	Non-melanoma skin cancer (basal-cell	Percent	1990	0.0003773	0.000420724	0.000334213
Incidence	Hungary	Male	55+ years	Non-melanoma skin cancer (basal-cell	Rate	1990	145.78448	161.8326368	131.8454032
Incidence	Hungary	Female	55+ years	Non-melanoma skin cancer (basal-cell	Rate	1990	126.87854	139.7170699	114.6529857
Incidence	Hungary	Both	55+ years	Non-melanoma skin cancer (basal-cell	Rate	1990	134.74244	148.4412915	122.7169139
Incidence	Hungary	Male	55+ years	Non-melanoma skin cancer (basal-cell	Number	2021	2020.815	2579.592884	1565.832653
Incidence	Hungary	Female	55+ years	Non-melanoma skin cancer (basal-cell	Number	2021	2496.0312	3151.883283	1893.429596
Incidence	Hungary	Both	55+ years	Non-melanoma skin cancer (basal-cell	Number	2021	4516.8462	5728.138984	3457.130116
Incidence	Hungary	Male	55+ years	Non-melanoma skin cancer (basal-cell	Percent	2021	0.0003972	0.000508302	0.00030021
Incidence	Hungary	Female	55+ years	Non-melanoma skin cancer (basal-cell	Percent	2021	0.0003207	0.000406115	0.000239721
Incidence	Hungary	Both	55+ years	Non-melanoma skin cancer (basal-cell	Percent	2021	0.000351	0.000446688	0.000264426
Incidence	Hungary	Male	55+ years	Non-melanoma skin cancer (basal-cell	Rate	2021	153.25064	195.6261525	118.746574
Incidence	Hungary	Female	55+ years	Non-melanoma skin cancer (basal-cell	Rate	2021	134.08783	169.3204757	101.7158223
Incidence	Hungary	Both	55+ years	Non-melanoma skin cancer (basal-cell	Rate	2021	142.03366	180.12315	108.7105547
Incidence	Viet Nam	Male	55+ years	Non-melanoma skin cancer (basal-cell	Number	1990	315.57074	370.9661256	257.9928282
Incidence	Viet Nam	Female	55+ years	Non-melanoma skin cancer (basal-cell	Number	1990	412.14387	482.3435843	335.63802
Incidence	Viet Nam	Both	55+ years	Non-melanoma skin cancer (basal-cell	Number	1990	727.71461	853.5976055	603.8856735
Incidence	Viet Nam	Male	55+ years	Non-melanoma skin cancer (basal-cell	Percent	1990	3.22E-05	3.82E-05	2.58E-05
Incidence	Viet Nam	Female	55+ years	Non-melanoma skin cancer (basal-cell	Percent	1990	2.92E-05	3.44E-05	2.33E-05

Incidence	Viet Nam	Both	55+ years	Non-melanoma skin cancer (basal-cell	Percent	1990	3.04E-05	3.57E-05	2.45E-05
Incidence	Viet Nam	Male	55+ years	Non-melanoma skin cancer (basal-cell	Rate	1990	10.632913	12.49941741	8.692869311
Incidence	Viet Nam	Female	55+ years	Non-melanoma skin cancer (basal-cell	Rate	1990	10.292728	12.04586917	8.382099002
Incidence	Viet Nam	Both	55+ years	Non-melanoma skin cancer (basal-cell	Rate	1990	10.437537	12.24306403	8.66147107
Incidence	Viet Nam	Male	55+ years	Non-melanoma skin cancer (basal-cell	Number	2021	445.27925	619.0749553	311.0082306
Incidence	Viet Nam	Female	55+ years	Non-melanoma skin cancer (basal-cell	Number	2021	512.41873	682.1977678	363.776967
Incidence	Viet Nam	Both	55+ years	Non-melanoma skin cancer (basal-cell	Number	2021	957.69798	1299.351025	671.4495212
Incidence	Viet Nam	Male	55+ years	Non-melanoma skin cancer (basal-cell	Percent	2021	1.70E-05	2.35E-05	1.18E-05
Incidence	Viet Nam	Female	55+ years	Non-melanoma skin cancer (basal-cell	Percent	2021	1.42E-05	1.92E-05	1.01E-05
Incidence	Viet Nam	Both	55+ years	Non-melanoma skin cancer (basal-cell	Percent	2021	1.54E-05	2.08E-05	1.09E-05
Incidence	Viet Nam	Male	55+ years	Non-melanoma skin cancer (basal-cell	Rate	2021	5.8293729	8.104619229	4.071563976
Incidence	Viet Nam	Female	55+ years	Non-melanoma skin cancer (basal-cell	Rate	2021	5.2127798	6.939923501	3.700663417
Incidence	Viet Nam	Both	55+ years	Non-melanoma skin cancer (basal-cell	Rate	2021	5.4823994	7.438212689	3.843752959
Incidence	Poland	Male	55+ years	Non-melanoma skin cancer (basal-cell	Number	1990	2245.8719	2859.643179	1695.679588
Incidence	Poland	Female	55+ years	Non-melanoma skin cancer (basal-cell	Number	1990	2763.6051	3518.773351	2122.078383
Incidence	Poland	Both	55+ years	Non-melanoma skin cancer (basal-cell	Number	1990	5009.477	6385.447597	3820.959329
Incidence	Poland	Male	55+ years	Non-melanoma skin cancer (basal-cell	Percent	1990	0.0002078	0.000263779	0.000154181
Incidence	Poland	Female	55+ years	Non-melanoma skin cancer (basal-cell	Percent	1990	0.0001694	0.000213641	0.000127957
Incidence	Poland	Both	55+ years	Non-melanoma skin cancer (basal-cell	Percent	1990	0.0001847	0.000234385	0.000138067
Incidence	Poland	Male	55+ years	Non-melanoma skin cancer (basal-cell	Rate	1990	70.047662	89.19089314	52.88742948
Incidence	Poland	Female	55+ years	Non-melanoma skin cancer (basal-cell	Rate	1990	61.416529	78.19888878	47.15966474
Incidence	Poland	Both	55+ years	Non-melanoma skin cancer (basal-cell	Rate	1990	65.007661	82.86354304	49.58434362
Incidence	Poland	Male	55+ years	Non-melanoma skin cancer (basal-cell	Number	2021	5747.8754	7435.617894	4369.736719
Incidence	Poland	Female	55+ years	Non-melanoma skin cancer (basal-cell	Number	2021	6536.14	8258.252477	5013.157474
Incidence	Poland	Both	55+ years	Non-melanoma skin cancer (basal-cell	Number	2021	12284.015	15707.94309	9376.203698
Incidence	Poland	Male	55+ years	Non-melanoma skin cancer (basal-cell	Percent	2021	0.0002915	0.000372909	0.000218658
Incidence	Poland	Female	55+ years	Non-melanoma skin cancer (basal-cell	Percent	2021	0.0002411	0.000304272	0.000182599
Incidence	Poland	Both	55+ years	Non-melanoma skin cancer (basal-cell	Percent	2021	0.0002623	0.000334784	0.00019852
Incidence	Poland	Male	55+ years	Non-melanoma skin cancer (basal-cell	Rate	2021	109.65728	141.8558363	83.36531888
Incidence	Poland	Female	55+ years	Non-melanoma skin cancer (basal-cell	Rate	2021	95.089383	120.1431022	72.93265647
Incidence	Poland	Both	55+ years	Non-melanoma skin cancer (basal-cell	Rate	2021	101.39214	129.65321	77.39109446
Incidence	Cambodia	Male	55+ years	Non-melanoma skin cancer (basal-cell	Number	1990	14.444263	19.72266741	9.755981347
Incidence	Cambodia	Female	55+ years	Non-melanoma skin cancer (basal-cell	Number	1990	14.410658	19.99283459	9.859249494
Incidence	Cambodia	Both	55+ years	Non-melanoma skin cancer (basal-cell	Number	1990	28.854921	39.38844605	19.58576296
Incidence	Cambodia	Male	55+ years	Non-melanoma skin cancer (basal-cell	Percent	1990	1.20E-05	1.63E-05	8.07E-06
Incidence	Cambodia	Female	55+ years	Non-melanoma skin cancer (basal-cell	Percent	1990	8.81E-06	1.22E-05	5.93E-06
Incidence	Cambodia	Both	55+ years	Non-melanoma skin cancer (basal-cell	Percent	1990	1.02E-05	1.38E-05	6.87E-06
Incidence	Cambodia	Male	55+ years	Non-melanoma skin cancer (basal-cell	Rate	1990	4.5612748	6.228113336	3.080788023
Incidence	Cambodia	Female	55+ years	Non-melanoma skin cancer (basal-cell	Rate	1990	3.3967374	4.712512747	2.323924539
Incidence	Cambodia	Both	55+ years	Non-melanoma skin cancer (basal-cell	Rate	1990	3.8944634	5.316142194	2.643432563
Incidence	Cambodia	Male	55+ years	Non-melanoma skin cancer (basal-cell	Number	2021	40.506532	56.15981496	28.02751848
Incidence	Cambodia	Female	55+ years	Non-melanoma skin cancer (basal-cell	Number	2021	43.754393	60.06940668	30.03152891
Incidence	Cambodia	Both	55+ years	Non-melanoma skin cancer (basal-cell	Number	2021	84.260925	116.1644722	58.99019491
Incidence	Cambodia	Male	55+ years	Non-melanoma skin cancer (basal-cell	Percent	2021	1.20E-05	1.67E-05	8.18E-06
Incidence	Cambodia	Female	55+ years	Non-melanoma skin cancer (basal-cell	Percent	2021	9.02E-06	1.23E-05	6.19E-06
Incidence	Cambodia	Both	55+ years	Non-melanoma skin cancer (basal-cell	Percent	2021	1.02E-05	1.42E-05	7.07E-06
Incidence	Cambodia	Male	55+ years	Non-melanoma skin cancer (basal-cell	Rate	2021	4.4439519	6.161265864	3.07488536
Incidence	Cambodia	Female	55+ years	Non-melanoma skin cancer (basal-cell	Rate	2021	3.466208	4.758677807	2.379087426
Incidence	Cambodia	Both	55+ years	Non-melanoma skin cancer (basal-cell	Rate	2021	3.8761846	5.343816662	2.713676399
Incidence	Kazakhstan	Male	55+ years	Non-melanoma skin cancer (basal-cell	Number	1990	772.33161	994.1991643	589.9980593
Incidence	Kazakhstan	Female	55+ years	Non-melanoma skin cancer (basal-cell	Number	1990	1352.194	1727.140771	1020.800282
Incidence	Kazakhstan	Both	55+ years	Non-melanoma skin cancer (basal-cell	Number	1990	2124.5256	2713.213472	1602.075557
Incidence	Kazakhstan	Male	55+ years	Non-melanoma skin cancer (basal-cell	Percent	1990	0.0002777	0.000357557	0.000205912
Incidence	Kazakhstan	Female	55+ years	Non-melanoma skin cancer (basal-cell	Percent	1990	0.0003051	0.000386648	0.000224921
Incidence	Kazakhstan	Both	55+ years	Non-melanoma skin cancer (basal-cell	Percent	1990	0.0002945	0.000370843	0.000215691
Incidence	Kazakhstan	Male	55+ years	Non-melanoma skin cancer (basal-cell	Rate	1990	98.061957	126.2321968	74.91129927

Incidence	Kazakhstan	Female	55+ years	Non-melanoma skin cancer (basal-cell	Rate	1990	103.71483	132.4736737	78.29654988
Incidence	Kazakhstan	Both	55+ years	Non-melanoma skin cancer (basal-cell	Rate	1990	101.58598	129.7345883	76.60459261
Incidence	Kazakhstan	Male	55+ years	Non-melanoma skin cancer (basal-cell	Number	2021	1265.5254	1660.072485	931.1852511
Incidence	Kazakhstan	Female	55+ years	Non-melanoma skin cancer (basal-cell	Number	2021	1879.5672	2394.021424	1426.442547
Incidence	Kazakhstan	Both	55+ years	Non-melanoma skin cancer (basal-cell	Number	2021	3145.0926	4019.667466	2378.186346
Incidence	Kazakhstan	Male	55+ years	Non-melanoma skin cancer (basal-cell	Percent	2021	0.0002385	0.000314744	0.000170825
Incidence	Kazakhstan	Female	55+ years	Non-melanoma skin cancer (basal-cell	Percent	2021	0.0002539	0.000327088	0.000183781
Incidence	Kazakhstan	Both	55+ years	Non-melanoma skin cancer (basal-cell	Percent	2021	0.0002475	0.000322018	0.000180341
Incidence	Kazakhstan	Male	55+ years	Non-melanoma skin cancer (basal-cell	Rate	2021	96.818111	127.0026561	71.23966048
Incidence	Kazakhstan	Female	55+ years	Non-melanoma skin cancer (basal-cell	Rate	2021	100.7093	128.2743292	76.43037734
Incidence	Kazakhstan	Both	55+ years	Non-melanoma skin cancer (basal-cell	Rate	2021	99.106551	126.6657089	74.94019393
Incidence	Bulgaria	Male	55+ years	Non-melanoma skin cancer (basal-cell	Number	1990	1848.4682	2357.238032	1426.386599
Incidence	Bulgaria	Female	55+ years	Non-melanoma skin cancer (basal-cell	Number	1990	1685.3804	2163.406197	1283.42565
Incidence	Bulgaria	Both	55+ years	Non-melanoma skin cancer (basal-cell	Number	1990	3533.8485	4524.826196	2704.690869
Incidence	Bulgaria	Male	55+ years	Non-melanoma skin cancer (basal-cell	Percent	1990	0.0005333	0.000680128	0.000404141
Incidence	Bulgaria	Female	55+ years	Non-melanoma skin cancer (basal-cell	Percent	1990	0.0003836	0.000489606	0.000283546
Incidence	Bulgaria	Both	55+ years	Non-melanoma skin cancer (basal-cell	Percent	1990	0.0004496	0.000575662	0.000338136
Incidence	Bulgaria	Male	55+ years	Non-melanoma skin cancer (basal-cell	Rate	1990	176.43708	224.9993873	136.1492164
Incidence	Bulgaria	Female	55+ years	Non-melanoma skin cancer (basal-cell	Rate	1990	138.64305	177.9664932	105.5773819
Incidence	Bulgaria	Both	55+ years	Non-melanoma skin cancer (basal-cell	Rate	1990	156.1377	199.9225312	119.5026331
Incidence	Bulgaria	Male	55+ years	Non-melanoma skin cancer (basal-cell	Number	2021	2677.707	3395.670873	2045.19836
Incidence	Bulgaria	Female	55+ years	Non-melanoma skin cancer (basal-cell	Number	2021	3265.2759	3772.483935	2569.768506
Incidence	Bulgaria	Both	55+ years	Non-melanoma skin cancer (basal-cell	Number	2021	5942.9829	7121.14705	4687.325653
Incidence	Bulgaria	Male	55+ years	Non-melanoma skin cancer (basal-cell	Percent	2021	0.0006702	0.000867212	0.000494762
Incidence	Bulgaria	Female	55+ years	Non-melanoma skin cancer (basal-cell	Percent	2021	0.0005767	0.000689601	0.000443184
Incidence	Bulgaria	Both	55+ years	Non-melanoma skin cancer (basal-cell	Percent	2021	0.0006154	0.00075846	0.000469074
Incidence	Bulgaria	Male	55+ years	Non-melanoma skin cancer (basal-cell	Rate	2021	262.11562	332.3957287	200.2005568
Incidence	Bulgaria	Female	55+ years	Non-melanoma skin cancer (basal-cell	Rate	2021	240.92056	278.3436977	189.6042185
Incidence	Bulgaria	Both	55+ years	Non-melanoma skin cancer (basal-cell	Rate	2021	250.03002	299.5971162	197.2026751
Incidence	Montenegro	Male	55+ years	Non-melanoma skin cancer (basal-cell	Number	1990	61.450979	76.57047621	46.74386458
Incidence	Montenegro	Female	55+ years	Non-melanoma skin cancer (basal-cell	Number	1990	66.190964	85.24574685	51.88955604
Incidence	Montenegro	Both	55+ years	Non-melanoma skin cancer (basal-cell	Number	1990	127.64194	161.7049591	98.14464979
Incidence	Montenegro	Male	55+ years	Non-melanoma skin cancer (basal-cell	Percent	1990	0.0004063	0.000514635	0.000299305
Incidence	Montenegro	Female	55+ years	Non-melanoma skin cancer (basal-cell	Percent	1990	0.000315	0.00040139	0.000236665
Incidence	Montenegro	Both	55+ years	Non-melanoma skin cancer (basal-cell	Percent	1990	0.0003532	0.000449797	0.000264637
Incidence	Montenegro	Male	55+ years	Non-melanoma skin cancer (basal-cell	Rate	1990	127.03186	158.2869835	96.62921908
Incidence	Montenegro	Female	55+ years	Non-melanoma skin cancer (basal-cell	Rate	1990	109.28492	140.7454182	85.67251193
Incidence	Montenegro	Both	55+ years	Non-melanoma skin cancer (basal-cell	Rate	1990	117.16526	148.4324348	90.08906966
Incidence	Montenegro	Male	55+ years	Non-melanoma skin cancer (basal-cell	Number	2021	104.81185	135.870971	79.51157754
Incidence	Montenegro	Female	55+ years	Non-melanoma skin cancer (basal-cell	Number	2021	109.11676	139.623835	83.73044173
Incidence	Montenegro	Both	55+ years	Non-melanoma skin cancer (basal-cell	Number	2021	213.9286	274.9969361	164.1446472
Incidence	Montenegro	Male	55+ years	Non-melanoma skin cancer (basal-cell	Percent	2021	0.0003282	0.000428544	0.000244009
Incidence	Montenegro	Female	55+ years	Non-melanoma skin cancer (basal-cell	Percent	2021	0.0002667	0.000344342	0.000200838
Incidence	Montenegro	Both	55+ years	Non-melanoma skin cancer (basal-cell	Percent	2021	0.0002937	0.000378682	0.000219692
Incidence	Montenegro	Male	55+ years	Non-melanoma skin cancer (basal-cell	Rate	2021	132.06185	171.1960281	100.1837711
Incidence	Montenegro	Female	55+ years	Non-melanoma skin cancer (basal-cell	Rate	2021	114.85073	146.9609238	88.13039023
Incidence	Montenegro	Both	55+ years	Non-melanoma skin cancer (basal-cell	Rate	2021	122.68435	157.7059801	94.13411227
Incidence	Fiji	Male	55+ years	Non-melanoma skin cancer (basal-cell	Number	1990	0.0465863	0.099148111	0.010995997
Incidence	Fiji	Female	55+ years	Non-melanoma skin cancer (basal-cell	Number	1990	0.0467402	0.103350438	0.015307426
Incidence	Fiji	Both	55+ years	Non-melanoma skin cancer (basal-cell	Number	1990	0.0933266	0.201944984	0.030976938
Incidence	Fiji	Male	55+ years	Non-melanoma skin cancer (basal-cell	Percent	1990	4.12E-07	8.77E-07	9.49E-08
Incidence	Fiji	Female	55+ years	Non-melanoma skin cancer (basal-cell	Percent	1990	3.86E-07	8.49E-07	1.24E-07
Incidence	Fiji	Both	55+ years	Non-melanoma skin cancer (basal-cell	Percent	1990	3.99E-07	8.63E-07	1.32E-07
Incidence	Fiji	Male	55+ years	Non-melanoma skin cancer (basal-cell	Rate	1990	0.1661401	0.353590423	0.039214859
Incidence	Fiji	Female	55+ years	Non-melanoma skin cancer (basal-cell	Rate	1990	0.1650179	0.364881961	0.054043347
Incidence	Fiji	Both	55+ years	Non-melanoma skin cancer (basal-cell	Rate	1990	0.1655762	0.358282523	0.054958015

Incidence	Fiji	Male	55+ years	Non-melanoma skin cancer (basal-cell	Number	2021	0.1075922	0.233501779	0.024762644
Incidence	Fiji	Female	55+ years	Non-melanoma skin cancer (basal-cell	Number	2021	0.1183073	0.256692452	0.034665639
Incidence	Fiji	Both	55+ years	Non-melanoma skin cancer (basal-cell	Number	2021	0.2258995	0.48767506	0.071556489
Incidence	Fiji	Male	55+ years	Non-melanoma skin cancer (basal-cell	Percent	2021	4.05E-07	8.77E-07	9.12E-08
Incidence	Fiji	Female	55+ years	Non-melanoma skin cancer (basal-cell	Percent	2021	3.70E-07	7.94E-07	1.05E-07
Incidence	Fiji	Both	55+ years	Non-melanoma skin cancer (basal-cell	Percent	2021	3.86E-07	8.27E-07	1.20E-07
Incidence	Fiji	Male	55+ years	Non-melanoma skin cancer (basal-cell	Rate	2021	0.1661267	0.360536285	0.038234534
Incidence	Fiji	Female	55+ years	Non-melanoma skin cancer (basal-cell	Rate	2021	0.1643716	0.356638672	0.048163113
Incidence	Fiji	Both	55+ years	Non-melanoma skin cancer (basal-cell	Rate	2021	0.1652029	0.356642419	0.052330089
Incidence	Croatia	Male	55+ years	Non-melanoma skin cancer (basal-cell	Number	1990	440.48823	559.6917291	328.2045231
Incidence	Croatia	Female	55+ years	Non-melanoma skin cancer (basal-cell	Number	1990	566.13173	723.6122606	433.7887969
Incidence	Croatia	Both	55+ years	Non-melanoma skin cancer (basal-cell	Number	1990	1006.62	1276.950157	762.7050548
Incidence	Croatia	Male	55+ years	Non-melanoma skin cancer (basal-cell	Percent	1990	0.0003005	0.000385323	0.000216427
Incidence	Croatia	Female	55+ years	Non-melanoma skin cancer (basal-cell	Percent	1990	0.0002431	0.000313989	0.000181635
Incidence	Croatia	Both	55+ years	Non-melanoma skin cancer (basal-cell	Percent	1990	0.0002652	0.000340377	0.00019425
Incidence	Croatia	Male	55+ years	Non-melanoma skin cancer (basal-cell	Rate	1990	95.954359	121.9212167	71.49488316
Incidence	Croatia	Female	55+ years	Non-melanoma skin cancer (basal-cell	Rate	1990	86.548384	110.6234976	66.31622563
Incidence	Croatia	Both	55+ years	Non-melanoma skin cancer (basal-cell	Rate	1990	90.427273	114.7117331	68.51576643
Incidence	Croatia	Male	55+ years	Non-melanoma skin cancer (basal-cell	Number	2021	821.96362	1081.591793	602.7679379
Incidence	Croatia	Female	55+ years	Non-melanoma skin cancer (basal-cell	Number	2021	877.45318	1111.328278	683.4847645
Incidence	Croatia	Both	55+ years	Non-melanoma skin cancer (basal-cell	Number	2021	1699.4168	2198.764971	1296.127956
Incidence	Croatia	Male	55+ years	Non-melanoma skin cancer (basal-cell	Percent	2021	0.0003351	0.000444298	0.000241746
Incidence	Croatia	Female	55+ years	Non-melanoma skin cancer (basal-cell	Percent	2021	0.0002555	0.000326469	0.000195556
Incidence	Croatia	Both	55+ years	Non-melanoma skin cancer (basal-cell	Percent	2021	0.0002887	0.000379032	0.000216017
Incidence	Croatia	Male	55+ years	Non-melanoma skin cancer (basal-cell	Rate	2021	125.05117	164.5502481	91.70337127
Incidence	Croatia	Female	55+ years	Non-melanoma skin cancer (basal-cell	Rate	2021	105.20713	133.2488813	81.95020502
Incidence	Croatia	Both	55+ years	Non-melanoma skin cancer (basal-cell	Rate	2021	113.95338	147.43688	86.91109076
Incidence	Marshall I	Male	55+ years	Non-melanoma skin cancer (basal-cell	Number	1990	0.0020556	0.004321299	0.000482006
Incidence	Marshall I	Female	55+ years	Non-melanoma skin cancer (basal-cell	Number	1990	0.0021449	0.00448845	0.000607916
Incidence	Marshall I	Both	55+ years	Non-melanoma skin cancer (basal-cell	Number	1990	0.0042005	0.008779657	0.001386152
Incidence	Marshall I	Male	55+ years	Non-melanoma skin cancer (basal-cell	Percent	1990	3.81E-07	8.05E-07	8.74E-08
Incidence	Marshall I	Female	55+ years	Non-melanoma skin cancer (basal-cell	Percent	1990	3.69E-07	7.80E-07	1.05E-07
Incidence	Marshall I	Both	55+ years	Non-melanoma skin cancer (basal-cell	Percent	1990	3.75E-07	7.90E-07	1.23E-07
Incidence	Marshall I	Male	55+ years	Non-melanoma skin cancer (basal-cell	Rate	1990	0.1653847	0.347671108	0.038779938
Incidence	Marshall I	Female	55+ years	Non-melanoma skin cancer (basal-cell	Rate	1990	0.1640458	0.34328115	0.046494009
Incidence	Marshall I	Both	55+ years	Non-melanoma skin cancer (basal-cell	Rate	1990	0.1646983	0.344240659	0.054349476
Incidence	Marshall I	Male	55+ years	Non-melanoma skin cancer (basal-cell	Number	2021	0.0048815	0.010623636	0.001113134
Incidence	Marshall I	Female	55+ years	Non-melanoma skin cancer (basal-cell	Number	2021	0.0047789	0.010730923	0.00128887
Incidence	Marshall I	Both	55+ years	Non-melanoma skin cancer (basal-cell	Number	2021	0.0096603	0.021174762	0.00299007
Incidence	Marshall I	Male	55+ years	Non-melanoma skin cancer (basal-cell	Percent	2021	4.38E-07	9.37E-07	9.62E-08
Incidence	Marshall I	Female	55+ years	Non-melanoma skin cancer (basal-cell	Percent	2021	4.03E-07	8.98E-07	1.06E-07
Incidence	Marshall I	Both	55+ years	Non-melanoma skin cancer (basal-cell	Percent	2021	4.20E-07	9.16E-07	1.24E-07
Incidence	Marshall I	Male	55+ years	Non-melanoma skin cancer (basal-cell	Rate	2021	0.1659931	0.361254823	0.037851933
Incidence	Marshall I	Female	55+ years	Non-melanoma skin cancer (basal-cell	Rate	2021	0.1637586	0.367719989	0.044166133
Incidence	Marshall I	Both	55+ years	Non-melanoma skin cancer (basal-cell	Rate	2021	0.1648801	0.36140623	0.051033867
Incidence	Tonga	Male	55+ years	Non-melanoma skin cancer (basal-cell	Number	1990	0.007744	0.016018525	0.001932516
Incidence	Tonga	Female	55+ years	Non-melanoma skin cancer (basal-cell	Number	1990	0.0079824	0.017246249	0.002580247
Incidence	Tonga	Both	55+ years	Non-melanoma skin cancer (basal-cell	Number	1990	0.0157264	0.033087823	0.005302115
Incidence	Tonga	Male	55+ years	Non-melanoma skin cancer (basal-cell	Percent	1990	4.42E-07	9.43E-07	1.10E-07
Incidence	Tonga	Female	55+ years	Non-melanoma skin cancer (basal-cell	Percent	1990	4.05E-07	8.75E-07	1.29E-07
Incidence	Tonga	Both	55+ years	Non-melanoma skin cancer (basal-cell	Percent	1990	4.23E-07	9.01E-07	1.42E-07
Incidence	Tonga	Male	55+ years	Non-melanoma skin cancer (basal-cell	Rate	1990	0.1657593	0.342872906	0.041365061
Incidence	Tonga	Female	55+ years	Non-melanoma skin cancer (basal-cell	Rate	1990	0.1657023	0.35800702	0.053562161
Incidence	Tonga	Both	55+ years	Non-melanoma skin cancer (basal-cell	Rate	1990	0.1657304	0.348691176	0.05587556
Incidence	Tonga	Male	55+ years	Non-melanoma skin cancer (basal-cell	Number	2021	0.0105795	0.021635557	0.00264756
Incidence	Tonga	Female	55+ years	Non-melanoma skin cancer (basal-cell	Number	2021	0.0118319	0.024068883	0.00395338

Incidence	Tonga	Both	55+ years	Non-melanoma skin cancer (basal-cell	Number	2021	0.0224114	0.045721284	0.007955671
Incidence	Tonga	Male	55+ years	Non-melanoma skin cancer (basal-cell	Percent	2021	4.50E-07	9.54E-07	1.16E-07
Incidence	Tonga	Female	55+ years	Non-melanoma skin cancer (basal-cell	Percent	2021	4.07E-07	8.53E-07	1.33E-07
Incidence	Tonga	Both	55+ years	Non-melanoma skin cancer (basal-cell	Percent	2021	4.26E-07	8.98E-07	1.48E-07
Incidence	Tonga	Male	55+ years	Non-melanoma skin cancer (basal-cell	Rate	2021	0.1663969	0.340290606	0.04164162
Incidence	Tonga	Female	55+ years	Non-melanoma skin cancer (basal-cell	Rate	2021	0.167294	0.340314235	0.055897548
Incidence	Tonga	Both	55+ years	Non-melanoma skin cancer (basal-cell	Rate	2021	0.1668693	0.340428463	0.0592358
Incidence	Papua New	Male	55+ years	Non-melanoma skin cancer (basal-cell	Number	1990	0.25707	0.546987892	0.061829453
Incidence	Papua New	Female	55+ years	Non-melanoma skin cancer (basal-cell	Number	1990	0.2306371	0.516269289	0.06344063
Incidence	Papua New	Both	55+ years	Non-melanoma skin cancer (basal-cell	Number	1990	0.4877071	1.066342513	0.148944144
Incidence	Papua New	Male	55+ years	Non-melanoma skin cancer (basal-cell	Percent	1990	3.89E-07	8.44E-07	9.28E-08
Incidence	Papua New	Female	55+ years	Non-melanoma skin cancer (basal-cell	Percent	1990	3.70E-07	8.20E-07	9.76E-08
Incidence	Papua New	Both	55+ years	Non-melanoma skin cancer (basal-cell	Percent	1990	3.80E-07	8.20E-07	1.17E-07
Incidence	Papua New	Male	55+ years	Non-melanoma skin cancer (basal-cell	Rate	1990	0.1658492	0.35289028	0.039889389
Incidence	Papua New	Female	55+ years	Non-melanoma skin cancer (basal-cell	Rate	1990	0.1637203	0.366479527	0.04503404
Incidence	Papua New	Both	55+ years	Non-melanoma skin cancer (basal-cell	Rate	1990	0.1648356	0.360403206	0.050340249
Incidence	Papua New	Male	55+ years	Non-melanoma skin cancer (basal-cell	Number	2021	0.7399301	1.599181406	0.172633166
Incidence	Papua New	Female	55+ years	Non-melanoma skin cancer (basal-cell	Number	2021	0.6259221	1.350871735	0.186345204
Incidence	Papua New	Both	55+ years	Non-melanoma skin cancer (basal-cell	Number	2021	1.3658522	2.942870902	0.447979153
Incidence	Papua New	Male	55+ years	Non-melanoma skin cancer (basal-cell	Percent	2021	3.70E-07	7.83E-07	8.54E-08
Incidence	Papua New	Female	55+ years	Non-melanoma skin cancer (basal-cell	Percent	2021	3.53E-07	7.49E-07	1.05E-07
Incidence	Papua New	Both	55+ years	Non-melanoma skin cancer (basal-cell	Percent	2021	3.62E-07	7.65E-07	1.19E-07
Incidence	Papua New	Male	55+ years	Non-melanoma skin cancer (basal-cell	Rate	2021	0.1664416	0.359723681	0.038832516
Incidence	Papua New	Female	55+ years	Non-melanoma skin cancer (basal-cell	Rate	2021	0.1661396	0.358564273	0.049461937
Incidence	Papua New	Both	55+ years	Non-melanoma skin cancer (basal-cell	Rate	2021	0.1663031	0.358317349	0.054544935
Incidence	Armenia	Male	55+ years	Non-melanoma skin cancer (basal-cell	Number	1990	197.85742	255.2034375	149.7132275
Incidence	Armenia	Female	55+ years	Non-melanoma skin cancer (basal-cell	Number	1990	268.01411	345.2347369	198.8053459
Incidence	Armenia	Both	55+ years	Non-melanoma skin cancer (basal-cell	Number	1990	465.87153	592.5220121	353.1657607
Incidence	Armenia	Male	55+ years	Non-melanoma skin cancer (basal-cell	Percent	1990	0.0002767	0.000359695	0.000202403
Incidence	Armenia	Female	55+ years	Non-melanoma skin cancer (basal-cell	Percent	1990	0.0002788	0.000358795	0.000201861
Incidence	Armenia	Both	55+ years	Non-melanoma skin cancer (basal-cell	Percent	1990	0.0002779	0.000356799	0.000201979
Incidence	Armenia	Male	55+ years	Non-melanoma skin cancer (basal-cell	Rate	1990	93.715857	120.8779971	70.91219166
Incidence	Armenia	Female	55+ years	Non-melanoma skin cancer (basal-cell	Rate	1990	97.232511	125.2472881	72.12434839
Incidence	Armenia	Both	55+ years	Non-melanoma skin cancer (basal-cell	Rate	1990	95.707239	121.72593	72.55330567
Incidence	Armenia	Male	55+ years	Non-melanoma skin cancer (basal-cell	Number	2021	362.96034	456.9142767	274.8739133
Incidence	Armenia	Female	55+ years	Non-melanoma skin cancer (basal-cell	Number	2021	471.42295	615.0706901	351.0952429
Incidence	Armenia	Both	55+ years	Non-melanoma skin cancer (basal-cell	Number	2021	834.38329	1063.177565	629.6266962
Incidence	Armenia	Male	55+ years	Non-melanoma skin cancer (basal-cell	Percent	2021	0.0002696	0.000344217	0.000201662
Incidence	Armenia	Female	55+ years	Non-melanoma skin cancer (basal-cell	Percent	2021	0.0002563	0.000338109	0.000188987
Incidence	Armenia	Both	55+ years	Non-melanoma skin cancer (basal-cell	Percent	2021	0.0002619	0.00033793	0.000194197
Incidence	Armenia	Male	55+ years	Non-melanoma skin cancer (basal-cell	Rate	2021	108.04069	136.0075118	81.8204178
Incidence	Armenia	Female	55+ years	Non-melanoma skin cancer (basal-cell	Rate	2021	104.60465	136.4788395	77.90498242
Incidence	Armenia	Both	55+ years	Non-melanoma skin cancer (basal-cell	Rate	2021	106.07211	135.1578928	80.0421494
Incidence	Albania	Male	55+ years	Non-melanoma skin cancer (basal-cell	Number	1990	201.25673	254.8124342	151.4676791
Incidence	Albania	Female	55+ years	Non-melanoma skin cancer (basal-cell	Number	1990	186.67524	236.3870662	142.3977832
Incidence	Albania	Both	55+ years	Non-melanoma skin cancer (basal-cell	Number	1990	387.93197	491.4617664	295.2590057
Incidence	Albania	Male	55+ years	Non-melanoma skin cancer (basal-cell	Percent	1990	0.0003649	0.000467977	0.000265561
Incidence	Albania	Female	55+ years	Non-melanoma skin cancer (basal-cell	Percent	1990	0.0002935	0.000374041	0.000220522
Incidence	Albania	Both	55+ years	Non-melanoma skin cancer (basal-cell	Percent	1990	0.0003266	0.000417771	0.000241973
Incidence	Albania	Male	55+ years	Non-melanoma skin cancer (basal-cell	Rate	1990	118.31183	149.7953624	89.04257736
Incidence	Albania	Female	55+ years	Non-melanoma skin cancer (basal-cell	Rate	1990	105.5154	133.6142722	80.48822838
Incidence	Albania	Both	55+ years	Non-melanoma skin cancer (basal-cell	Rate	1990	111.78805	141.6216126	85.08303064
Incidence	Albania	Male	55+ years	Non-melanoma skin cancer (basal-cell	Number	2021	516.06125	644.6543067	398.7714531
Incidence	Albania	Female	55+ years	Non-melanoma skin cancer (basal-cell	Number	2021	452.83144	574.6610564	344.7238177
Incidence	Albania	Both	55+ years	Non-melanoma skin cancer (basal-cell	Number	2021	968.89269	1225.899298	738.306557
Incidence	Albania	Male	55+ years	Non-melanoma skin cancer (basal-cell	Percent	2021	0.000357	0.000451436	0.000268311

Incidence	Albania	Female	55+ years	Non-melanoma skin cancer (basal-cell	Percent	2021	0.0002688	0.000338098	0.000201139
Incidence	Albania	Both	55+ years	Non-melanoma skin cancer (basal-cell	Percent	2021	0.0003095	0.000392803	0.000227615
Incidence	Albania	Male	55+ years	Non-melanoma skin cancer (basal-cell	Rate	2021	136.32433	170.2938699	105.3406969
Incidence	Albania	Female	55+ years	Non-melanoma skin cancer (basal-cell	Rate	2021	111.05886	140.9381046	84.54500427
Incidence	Albania	Both	55+ years	Non-melanoma skin cancer (basal-cell	Rate	2021	123.22269	155.9085049	93.89700417
Incidence	Serbia	Male	55+ years	Non-melanoma skin cancer (basal-cell	Number	1990	919.42037	971.1913809	864.7007166
Incidence	Serbia	Female	55+ years	Non-melanoma skin cancer (basal-cell	Number	1990	860.49513	903.9539353	816.0408523
Incidence	Serbia	Both	55+ years	Non-melanoma skin cancer (basal-cell	Number	1990	1779.9155	1859.40047	1690.480969
Incidence	Serbia	Male	55+ years	Non-melanoma skin cancer (basal-cell	Percent	1990	0.000289	0.000316279	0.00026232
Incidence	Serbia	Female	55+ years	Non-melanoma skin cancer (basal-cell	Percent	1990	0.0002154	0.0002335	0.000197383
Incidence	Serbia	Both	55+ years	Non-melanoma skin cancer (basal-cell	Percent	1990	0.000248	0.000267089	0.000228759
Incidence	Serbia	Male	55+ years	Non-melanoma skin cancer (basal-cell	Rate	1990	96.14778	101.5617	90.42550878
Incidence	Serbia	Female	55+ years	Non-melanoma skin cancer (basal-cell	Rate	1990	76.590355	80.45850592	72.63359911
Incidence	Serbia	Both	55+ years	Non-melanoma skin cancer (basal-cell	Rate	1990	85.582704	89.40453636	81.28247232
Incidence	Serbia	Male	55+ years	Non-melanoma skin cancer (basal-cell	Number	2021	1728.024	2210.616232	1317.05712
Incidence	Serbia	Female	55+ years	Non-melanoma skin cancer (basal-cell	Number	2021	1744.5202	2220.913881	1347.431835
Incidence	Serbia	Both	55+ years	Non-melanoma skin cancer (basal-cell	Number	2021	3472.5443	4422.393084	2649.69482
Incidence	Serbia	Male	55+ years	Non-melanoma skin cancer (basal-cell	Percent	2021	0.0003529	0.000450119	0.000268796
Incidence	Serbia	Female	55+ years	Non-melanoma skin cancer (basal-cell	Percent	2021	0.0002757	0.000347421	0.000209251
Incidence	Serbia	Both	55+ years	Non-melanoma skin cancer (basal-cell	Percent	2021	0.0003093	0.00039339	0.000232843
Incidence	Serbia	Male	55+ years	Non-melanoma skin cancer (basal-cell	Rate	2021	135.90249	173.8565273	103.5815144
Incidence	Serbia	Female	55+ years	Non-melanoma skin cancer (basal-cell	Rate	2021	113.74569	144.8073691	87.8548514
Incidence	Serbia	Both	55+ years	Non-melanoma skin cancer (basal-cell	Rate	2021	123.78867	157.6487198	94.45587224
Incidence	Myanmar	Male	55+ years	Non-melanoma skin cancer (basal-cell	Number	1990	83.612698	113.9918896	57.25020627
Incidence	Myanmar	Female	55+ years	Non-melanoma skin cancer (basal-cell	Number	1990	71.172419	99.20557242	49.28397779
Incidence	Myanmar	Both	55+ years	Non-melanoma skin cancer (basal-cell	Number	1990	154.78512	213.9940578	107.42188
Incidence	Myanmar	Male	55+ years	Non-melanoma skin cancer (basal-cell	Percent	1990	1.32E-05	1.82E-05	8.88E-06
Incidence	Myanmar	Female	55+ years	Non-melanoma skin cancer (basal-cell	Percent	1990	9.90E-06	1.40E-05	6.65E-06
Incidence	Myanmar	Both	55+ years	Non-melanoma skin cancer (basal-cell	Percent	1990	1.14E-05	1.58E-05	7.69E-06
Incidence	Myanmar	Male	55+ years	Non-melanoma skin cancer (basal-cell	Rate	1990	4.5572057	6.21298565	3.120351028
Incidence	Myanmar	Female	55+ years	Non-melanoma skin cancer (basal-cell	Rate	1990	3.422136	4.770035395	2.369688645
Incidence	Myanmar	Both	55+ years	Non-melanoma skin cancer (basal-cell	Rate	1990	3.9541458	5.46669935	2.744202936
Incidence	Myanmar	Male	55+ years	Non-melanoma skin cancer (basal-cell	Number	2021	169.07673	230.2014349	115.365798
Incidence	Myanmar	Female	55+ years	Non-melanoma skin cancer (basal-cell	Number	2021	170.24503	230.9458146	116.5898354
Incidence	Myanmar	Both	55+ years	Non-melanoma skin cancer (basal-cell	Number	2021	339.32176	460.6697913	236.3702497
Incidence	Myanmar	Male	55+ years	Non-melanoma skin cancer (basal-cell	Percent	2021	1.31E-05	1.79E-05	8.93E-06
Incidence	Myanmar	Female	55+ years	Non-melanoma skin cancer (basal-cell	Percent	2021	9.92E-06	1.37E-05	6.75E-06
Incidence	Myanmar	Both	55+ years	Non-melanoma skin cancer (basal-cell	Percent	2021	1.13E-05	1.54E-05	7.66E-06
Incidence	Myanmar	Male	55+ years	Non-melanoma skin cancer (basal-cell	Rate	2021	4.6428207	6.321295621	3.167926881
Incidence	Myanmar	Female	55+ years	Non-melanoma skin cancer (basal-cell	Rate	2021	3.5464449	4.810928116	2.42873125
Incidence	Myanmar	Both	55+ years	Non-melanoma skin cancer (basal-cell	Rate	2021	4.0193888	5.456800132	2.799891016
Incidence	Mongolia	Male	55+ years	Non-melanoma skin cancer (basal-cell	Number	1990	82.105154	105.9469336	61.47132005
Incidence	Mongolia	Female	55+ years	Non-melanoma skin cancer (basal-cell	Number	1990	99.632333	126.7081357	74.87459599
Incidence	Mongolia	Both	55+ years	Non-melanoma skin cancer (basal-cell	Number	1990	181.73749	232.8752532	137.6014707
Incidence	Mongolia	Male	55+ years	Non-melanoma skin cancer (basal-cell	Percent	1990	0.0003345	0.000437233	0.000249016
Incidence	Mongolia	Female	55+ years	Non-melanoma skin cancer (basal-cell	Percent	1990	0.0003259	0.000421787	0.000240763
Incidence	Mongolia	Both	55+ years	Non-melanoma skin cancer (basal-cell	Percent	1990	0.0003297	0.000426788	0.000243588
Incidence	Mongolia	Male	55+ years	Non-melanoma skin cancer (basal-cell	Rate	1990	104.6545	135.0441786	78.3537913
Incidence	Mongolia	Female	55+ years	Non-melanoma skin cancer (basal-cell	Rate	1990	105.06527	133.617513	78.95749746
Incidence	Mongolia	Both	55+ years	Non-melanoma skin cancer (basal-cell	Rate	1990	104.87929	134.3905012	79.40874081
Incidence	Mongolia	Male	55+ years	Non-melanoma skin cancer (basal-cell	Number	2021	159.27196	204.2298745	119.5328307
Incidence	Mongolia	Female	55+ years	Non-melanoma skin cancer (basal-cell	Number	2021	215.09255	283.4048577	156.0013629
Incidence	Mongolia	Both	55+ years	Non-melanoma skin cancer (basal-cell	Number	2021	374.36451	485.0179528	276.2045071
Incidence	Mongolia	Male	55+ years	Non-melanoma skin cancer (basal-cell	Percent	2021	0.0002436	0.00031445	0.000180136
Incidence	Mongolia	Female	55+ years	Non-melanoma skin cancer (basal-cell	Percent	2021	0.0002403	0.000314267	0.000172236
Incidence	Mongolia	Both	55+ years	Non-melanoma skin cancer (basal-cell	Percent	2021	0.0002417	0.000314499	0.000175774

Incidence	Mongolia	Male	55+ years	Non-melanoma skin cancer (basal-cell	Rate	2021	94.706144	121.4389776	71.07650038
Incidence	Mongolia	Female	55+ years	Non-melanoma skin cancer (basal-cell	Rate	2021	94.908636	125.0511401	68.83491148
Incidence	Mongolia	Both	55+ years	Non-melanoma skin cancer (basal-cell	Rate	2021	94.822381	122.8496719	69.95954044
Incidence	Lithuania	Male	55+ years	Non-melanoma skin cancer (basal-cell	Number	1990	334.39037	358.7060739	307.5888547
Incidence	Lithuania	Female	55+ years	Non-melanoma skin cancer (basal-cell	Number	1990	565.10982	619.6428324	510.0666239
Incidence	Lithuania	Both	55+ years	Non-melanoma skin cancer (basal-cell	Number	1990	899.50018	971.9437157	826.5984277
Incidence	Lithuania	Male	55+ years	Non-melanoma skin cancer (basal-cell	Percent	1990	0.0002892	0.000318487	0.000256975
Incidence	Lithuania	Female	55+ years	Non-melanoma skin cancer (basal-cell	Percent	1990	0.0002911	0.000326667	0.000256146
Incidence	Lithuania	Both	55+ years	Non-melanoma skin cancer (basal-cell	Percent	1990	0.0002904	0.000320209	0.000258668
Incidence	Lithuania	Male	55+ years	Non-melanoma skin cancer (basal-cell	Rate	1990	109.72739	117.7063791	100.9326938
Incidence	Lithuania	Female	55+ years	Non-melanoma skin cancer (basal-cell	Rate	1990	115.87173	127.0533336	104.5855153
Incidence	Lithuania	Both	55+ years	Non-melanoma skin cancer (basal-cell	Rate	1990	113.50884	122.6505659	104.3092962
Incidence	Lithuania	Male	55+ years	Non-melanoma skin cancer (basal-cell	Number	2021	433.87891	517.4401928	350.1219939
Incidence	Lithuania	Female	55+ years	Non-melanoma skin cancer (basal-cell	Number	2021	715.66005	879.0270521	552.3704267
Incidence	Lithuania	Both	55+ years	Non-melanoma skin cancer (basal-cell	Number	2021	1149.539	1396.802925	918.7754633
Incidence	Lithuania	Male	55+ years	Non-melanoma skin cancer (basal-cell	Percent	2021	0.0002732	0.000330234	0.000218546
Incidence	Lithuania	Female	55+ years	Non-melanoma skin cancer (basal-cell	Percent	2021	0.0002636	0.000329637	0.000200765
Incidence	Lithuania	Both	55+ years	Non-melanoma skin cancer (basal-cell	Percent	2021	0.0002671	0.000323876	0.000211702
Incidence	Lithuania	Male	55+ years	Non-melanoma skin cancer (basal-cell	Rate	2021	116.37266	138.7850192	93.90783384
Incidence	Lithuania	Female	55+ years	Non-melanoma skin cancer (basal-cell	Rate	2021	120.29023	147.7494362	92.84403586
Incidence	Lithuania	Both	55+ years	Non-melanoma skin cancer (basal-cell	Rate	2021	118.781	144.3305945	94.93637687
Incidence	Turkmenist	Male	55+ years	Non-melanoma skin cancer (basal-cell	Number	1990	129.0913	168.7409044	95.74968993
Incidence	Turkmenist	Female	55+ years	Non-melanoma skin cancer (basal-cell	Number	1990	189.8344	241.6384888	144.5552054
Incidence	Turkmenist	Both	55+ years	Non-melanoma skin cancer (basal-cell	Number	1990	318.92569	408.6774748	239.9269613
Incidence	Turkmenist	Male	55+ years	Non-melanoma skin cancer (basal-cell	Percent	1990	0.0003121	0.000409435	0.00022915
Incidence	Turkmenist	Female	55+ years	Non-melanoma skin cancer (basal-cell	Percent	1990	0.0003159	0.000398218	0.000233754
Incidence	Turkmenist	Both	55+ years	Non-melanoma skin cancer (basal-cell	Percent	1990	0.0003143	0.00039985	0.00023281
Incidence	Turkmenist	Male	55+ years	Non-melanoma skin cancer (basal-cell	Rate	1990	95.439027	124.7525462	70.78910511
Incidence	Turkmenist	Female	55+ years	Non-melanoma skin cancer (basal-cell	Rate	1990	100.17092	127.5066577	76.27820877
Incidence	Turkmenist	Both	55+ years	Non-melanoma skin cancer (basal-cell	Rate	1990	98.200182	125.83559	73.87573963
Incidence	Turkmenist	Male	55+ years	Non-melanoma skin cancer (basal-cell	Number	2021	292.12734	370.3182762	218.7279225
Incidence	Turkmenist	Female	55+ years	Non-melanoma skin cancer (basal-cell	Number	2021	383.49418	500.2816202	289.2601618
Incidence	Turkmenist	Both	55+ years	Non-melanoma skin cancer (basal-cell	Number	2021	675.62153	867.6312558	507.4599967
Incidence	Turkmenist	Male	55+ years	Non-melanoma skin cancer (basal-cell	Percent	2021	0.0002607	0.000329714	0.000191539
Incidence	Turkmenist	Female	55+ years	Non-melanoma skin cancer (basal-cell	Percent	2021	0.0002553	0.000332557	0.000189805
Incidence	Turkmenist	Both	55+ years	Non-melanoma skin cancer (basal-cell	Percent	2021	0.0002576	0.000329543	0.000191561
Incidence	Turkmenist	Male	55+ years	Non-melanoma skin cancer (basal-cell	Rate	2021	94.716382	120.0682095	70.91810398
Incidence	Turkmenist	Female	55+ years	Non-melanoma skin cancer (basal-cell	Rate	2021	96.046961	125.2966315	72.44584336
Incidence	Turkmenist	Both	55+ years	Non-melanoma skin cancer (basal-cell	Rate	2021	95.467081	122.5985558	71.70541897
Incidence	Sri Lanka	Male	55+ years	Non-melanoma skin cancer (basal-cell	Number	1990	54.205064	64.20739516	44.25918902
Incidence	Sri Lanka	Female	55+ years	Non-melanoma skin cancer (basal-cell	Number	1990	23.724231	29.64304095	18.2783468
Incidence	Sri Lanka	Both	55+ years	Non-melanoma skin cancer (basal-cell	Number	1990	77.929294	92.73102888	63.06762519
Incidence	Sri Lanka	Male	55+ years	Non-melanoma skin cancer (basal-cell	Percent	1990	1.56E-05	1.86E-05	1.24E-05
Incidence	Sri Lanka	Female	55+ years	Non-melanoma skin cancer (basal-cell	Percent	1990	6.76E-06	8.50E-06	5.12E-06
Incidence	Sri Lanka	Both	55+ years	Non-melanoma skin cancer (basal-cell	Percent	1990	1.12E-05	1.34E-05	8.82E-06
Incidence	Sri Lanka	Male	55+ years	Non-melanoma skin cancer (basal-cell	Rate	1990	6.0435439	7.158744683	4.93463772
Incidence	Sri Lanka	Female	55+ years	Non-melanoma skin cancer (basal-cell	Rate	1990	2.6871769	3.357583887	2.070336941
Incidence	Sri Lanka	Both	55+ years	Non-melanoma skin cancer (basal-cell	Rate	1990	4.3785993	5.210261714	3.543569363
Incidence	Sri Lanka	Male	55+ years	Non-melanoma skin cancer (basal-cell	Number	2021	104.27719	141.4124652	74.10681168
Incidence	Sri Lanka	Female	55+ years	Non-melanoma skin cancer (basal-cell	Number	2021	64.680425	91.98175502	42.35080035
Incidence	Sri Lanka	Both	55+ years	Non-melanoma skin cancer (basal-cell	Number	2021	168.95762	231.6938799	118.6569188
Incidence	Sri Lanka	Male	55+ years	Non-melanoma skin cancer (basal-cell	Percent	2021	1.26E-05	1.75E-05	8.84E-06
Incidence	Sri Lanka	Female	55+ years	Non-melanoma skin cancer (basal-cell	Percent	2021	6.03E-06	8.67E-06	3.85E-06
Incidence	Sri Lanka	Both	55+ years	Non-melanoma skin cancer (basal-cell	Percent	2021	8.90E-06	1.25E-05	6.12E-06
Incidence	Sri Lanka	Male	55+ years	Non-melanoma skin cancer (basal-cell	Rate	2021	4.8541689	6.582839294	3.449718744
Incidence	Sri Lanka	Female	55+ years	Non-melanoma skin cancer (basal-cell	Rate	2021	2.4242078	3.447455478	1.587298466

Incidence	Sri Lanka	Both	55+ years	Non-melanoma skin cancer (basal-cell	Rate	2021	3.5080345	4.81061545	2.463650774
Incidence	Czechia	Male	55+ years	Non-melanoma skin cancer (basal-cell	Number	1990	2526.7406	2651.180164	2393.551511
Incidence	Czechia	Female	55+ years	Non-melanoma skin cancer (basal-cell	Number	1990	2786.3367	2964.693711	2613.914066
Incidence	Czechia	Both	55+ years	Non-melanoma skin cancer (basal-cell	Number	1990	5313.0773	5549.31199	5041.275944
Incidence	Czechia	Male	55+ years	Non-melanoma skin cancer (basal-cell	Percent	1990	0.0007767	0.000837267	0.000708902
Incidence	Czechia	Female	55+ years	Non-melanoma skin cancer (basal-cell	Percent	1990	0.0005422	0.000588147	0.000495953
Incidence	Czechia	Both	55+ years	Non-melanoma skin cancer (basal-cell	Percent	1990	0.0006331	0.000676992	0.000582652
Incidence	Czechia	Male	55+ years	Non-melanoma skin cancer (basal-cell	Rate	1990	257.42915	270.1072861	243.8595881
Incidence	Czechia	Female	55+ years	Non-melanoma skin cancer (basal-cell	Rate	1990	200.98894	213.8545061	188.5514512
Incidence	Czechia	Both	55+ years	Non-melanoma skin cancer (basal-cell	Rate	1990	224.38479	234.3615848	212.9059281
Incidence	Czechia	Male	55+ years	Non-melanoma skin cancer (basal-cell	Number	2021	3444.9607	4365.414917	2622.003845
Incidence	Czechia	Female	55+ years	Non-melanoma skin cancer (basal-cell	Number	2021	3545.3731	4559.635009	2748.776711
Incidence	Czechia	Both	55+ years	Non-melanoma skin cancer (basal-cell	Number	2021	6990.3338	8956.854847	5353.526163
Incidence	Czechia	Male	55+ years	Non-melanoma skin cancer (basal-cell	Percent	2021	0.0005686	0.00072415	0.0004286
Incidence	Czechia	Female	55+ years	Non-melanoma skin cancer (basal-cell	Percent	2021	0.0004455	0.000579824	0.000345036
Incidence	Czechia	Both	55+ years	Non-melanoma skin cancer (basal-cell	Percent	2021	0.0004987	0.000639691	0.000381301
Incidence	Czechia	Male	55+ years	Non-melanoma skin cancer (basal-cell	Rate	2021	219.26883	277.8549591	166.8883222
Incidence	Czechia	Female	55+ years	Non-melanoma skin cancer (basal-cell	Rate	2021	184.42901	237.1905397	142.9903557
Incidence	Czechia	Both	55+ years	Non-melanoma skin cancer (basal-cell	Rate	2021	200.09749	256.3889297	153.2440646
Incidence	Slovakia	Male	55+ years	Non-melanoma skin cancer (basal-cell	Number	1990	764.99555	798.3401778	730.8142265
Incidence	Slovakia	Female	55+ years	Non-melanoma skin cancer (basal-cell	Number	1990	839.59161	876.0083239	800.8666607
Incidence	Slovakia	Both	55+ years	Non-melanoma skin cancer (basal-cell	Number	1990	1604.5872	1662.564873	1537.825418
Incidence	Slovakia	Male	55+ years	Non-melanoma skin cancer (basal-cell	Percent	1990	0.0005252	0.000565607	0.000480905
Incidence	Slovakia	Female	55+ years	Non-melanoma skin cancer (basal-cell	Percent	1990	0.0003923	0.000418795	0.000360762
Incidence	Slovakia	Both	55+ years	Non-melanoma skin cancer (basal-cell	Percent	1990	0.0004461	0.000475431	0.000411209
Incidence	Slovakia	Male	55+ years	Non-melanoma skin cancer (basal-cell	Rate	1990	173.35169	180.9077418	165.606035
Incidence	Slovakia	Female	55+ years	Non-melanoma skin cancer (basal-cell	Rate	1990	140.83367	146.9422303	134.3379167
Incidence	Slovakia	Both	55+ years	Non-melanoma skin cancer (basal-cell	Rate	1990	154.66569	160.254141	148.2305415
Incidence	Slovakia	Male	55+ years	Non-melanoma skin cancer (basal-cell	Number	2021	1087.0543	1394.099165	827.3998351
Incidence	Slovakia	Female	55+ years	Non-melanoma skin cancer (basal-cell	Number	2021	1210.2165	1524.834811	923.7124953
Incidence	Slovakia	Both	55+ years	Non-melanoma skin cancer (basal-cell	Number	2021	2297.2708	2926.003667	1754.732091
Incidence	Slovakia	Male	55+ years	Non-melanoma skin cancer (basal-cell	Percent	2021	0.0004105	0.000523731	0.000306599
Incidence	Slovakia	Female	55+ years	Non-melanoma skin cancer (basal-cell	Percent	2021	0.0003281	0.000411606	0.000249452
Incidence	Slovakia	Both	55+ years	Non-melanoma skin cancer (basal-cell	Percent	2021	0.0003625	0.000457367	0.000275339
Incidence	Slovakia	Male	55+ years	Non-melanoma skin cancer (basal-cell	Rate	2021	152.29268	195.3086388	115.9159546
Incidence	Slovakia	Female	55+ years	Non-melanoma skin cancer (basal-cell	Rate	2021	130.62468	164.5829991	99.70088015
Incidence	Slovakia	Both	55+ years	Non-melanoma skin cancer (basal-cell	Rate	2021	140.05386	178.3847633	106.9778115
Incidence	Slovenia	Male	55+ years	Non-melanoma skin cancer (basal-cell	Number	1990	210.58227	231.6223354	192.454852
Incidence	Slovenia	Female	55+ years	Non-melanoma skin cancer (basal-cell	Number	1990	280.42535	302.3627623	258.2852258
Incidence	Slovenia	Both	55+ years	Non-melanoma skin cancer (basal-cell	Number	1990	491.00762	530.194043	453.5492552
Incidence	Slovenia	Male	55+ years	Non-melanoma skin cancer (basal-cell	Percent	1990	0.0003668	0.000410542	0.000324827
Incidence	Slovenia	Female	55+ years	Non-melanoma skin cancer (basal-cell	Percent	1990	0.0003003	0.000330044	0.000267401
Incidence	Slovenia	Both	55+ years	Non-melanoma skin cancer (basal-cell	Percent	1990	0.0003256	0.000358232	0.000290452
Incidence	Slovenia	Male	55+ years	Non-melanoma skin cancer (basal-cell	Rate	1990	122.37523	134.6021999	111.8408827
Incidence	Slovenia	Female	55+ years	Non-melanoma skin cancer (basal-cell	Rate	1990	108.42833	116.9105798	99.86770618
Incidence	Slovenia	Both	55+ years	Non-melanoma skin cancer (basal-cell	Rate	1990	114.0005	123.0986764	105.3035464
Incidence	Slovenia	Male	55+ years	Non-melanoma skin cancer (basal-cell	Number	2021	744.74235	968.7930474	550.6132523
Incidence	Slovenia	Female	55+ years	Non-melanoma skin cancer (basal-cell	Number	2021	677.6711	836.7152338	531.052466
Incidence	Slovenia	Both	55+ years	Non-melanoma skin cancer (basal-cell	Number	2021	1422.4134	1802.243492	1093.886117
Incidence	Slovenia	Male	55+ years	Non-melanoma skin cancer (basal-cell	Percent	2021	0.0006098	0.000801917	0.000443308
Incidence	Slovenia	Female	55+ years	Non-melanoma skin cancer (basal-cell	Percent	2021	0.000433	0.000540204	0.000336275
Incidence	Slovenia	Both	55+ years	Non-melanoma skin cancer (basal-cell	Percent	2021	0.0005104	0.000650572	0.000391435
Incidence	Slovenia	Male	55+ years	Non-melanoma skin cancer (basal-cell	Rate	2021	222.22306	289.0773664	164.2970388
Incidence	Slovenia	Female	55+ years	Non-melanoma skin cancer (basal-cell	Rate	2021	170.67393	210.7297769	133.7474963
Incidence	Slovenia	Both	55+ years	Non-melanoma skin cancer (basal-cell	Rate	2021	194.26867	246.144642	149.3994611
Incidence	Latvia	Male	55+ years	Non-melanoma skin cancer (basal-cell	Number	1990	190.8794	203.9578726	177.2088051

Incidence	Latvia	Female	55+ years	Non-melanoma skin cancer (basal-cell	Number	1990	313.87843	331.2197884	295.47087
Incidence	Latvia	Both	55+ years	Non-melanoma skin cancer (basal-cell	Number	1990	504.75782	529.7598891	477.6804918
Incidence	Latvia	Male	55+ years	Non-melanoma skin cancer (basal-cell	Percent	1990	0.0002281	0.000252734	0.000206416
Incidence	Latvia	Female	55+ years	Non-melanoma skin cancer (basal-cell	Percent	1990	0.0001991	0.000217061	0.000180083
Incidence	Latvia	Both	55+ years	Non-melanoma skin cancer (basal-cell	Percent	1990	0.0002091	0.000228384	0.000190452
Incidence	Latvia	Male	55+ years	Non-melanoma skin cancer (basal-cell	Rate	1990	84.501423	90.2912043	78.44951617
Incidence	Latvia	Female	55+ years	Non-melanoma skin cancer (basal-cell	Rate	1990	78.597543	82.93995111	73.98815039
Incidence	Latvia	Both	55+ years	Non-melanoma skin cancer (basal-cell	Rate	1990	80.730525	84.7293338	76.3998005
Incidence	Latvia	Male	55+ years	Non-melanoma skin cancer (basal-cell	Number	2021	241.92101	310.8767186	185.6874086
Incidence	Latvia	Female	55+ years	Non-melanoma skin cancer (basal-cell	Number	2021	383.65296	499.0935771	297.7316798
Incidence	Latvia	Both	55+ years	Non-melanoma skin cancer (basal-cell	Number	2021	625.57397	804.9675867	481.7407305
Incidence	Latvia	Male	55+ years	Non-melanoma skin cancer (basal-cell	Percent	2021	0.0002336	0.000302116	0.000174316
Incidence	Latvia	Female	55+ years	Non-melanoma skin cancer (basal-cell	Percent	2021	0.0002135	0.000279711	0.000161699
Incidence	Latvia	Both	55+ years	Non-melanoma skin cancer (basal-cell	Percent	2021	0.0002208	0.000289208	0.000166531
Incidence	Latvia	Male	55+ years	Non-melanoma skin cancer (basal-cell	Rate	2021	96.966349	124.605053	74.42689661
Incidence	Latvia	Female	55+ years	Non-melanoma skin cancer (basal-cell	Rate	2021	93.910636	122.1682089	72.87881011
Incidence	Latvia	Both	55+ years	Non-melanoma skin cancer (basal-cell	Rate	2021	95.069217	122.3318765	73.21070876
Incidence	Brunei Dar	Male	55+ years	Non-melanoma skin cancer (basal-cell	Number	1990	1.6890705	2.189187968	1.244172855
Incidence	Brunei Dar	Female	55+ years	Non-melanoma skin cancer (basal-cell	Number	1990	1.3700661	1.790777998	1.025546934
Incidence	Brunei Dar	Both	55+ years	Non-melanoma skin cancer (basal-cell	Number	1990	3.0591366	3.958363325	2.276686002
Incidence	Brunei Dar	Male	55+ years	Non-melanoma skin cancer (basal-cell	Percent	1990	5.69E-05	7.47E-05	4.11E-05
Incidence	Brunei Dar	Female	55+ years	Non-melanoma skin cancer (basal-cell	Percent	1990	5.02E-05	6.61E-05	3.70E-05
Incidence	Brunei Dar	Both	55+ years	Non-melanoma skin cancer (basal-cell	Percent	1990	5.37E-05	7.09E-05	3.94E-05
Incidence	Brunei Dar	Male	55+ years	Non-melanoma skin cancer (basal-cell	Rate	1990	20.766216	26.9148932	15.29643869
Incidence	Brunei Dar	Female	55+ years	Non-melanoma skin cancer (basal-cell	Rate	1990	18.211988	23.80441849	13.63237008
Incidence	Brunei Dar	Both	55+ years	Non-melanoma skin cancer (basal-cell	Rate	1990	19.53893	25.28235758	14.54136088
Incidence	Brunei Dar	Male	55+ years	Non-melanoma skin cancer (basal-cell	Number	2021	5.4983989	7.193472545	4.053482857
Incidence	Brunei Dar	Female	55+ years	Non-melanoma skin cancer (basal-cell	Number	2021	4.996055	6.483703611	3.724413303
Incidence	Brunei Dar	Both	55+ years	Non-melanoma skin cancer (basal-cell	Number	2021	10.494454	13.61152793	7.828606336
Incidence	Brunei Dar	Male	55+ years	Non-melanoma skin cancer (basal-cell	Percent	2021	5.06E-05	6.80E-05	3.56E-05
Incidence	Brunei Dar	Female	55+ years	Non-melanoma skin cancer (basal-cell	Percent	2021	4.43E-05	5.89E-05	3.17E-05
Incidence	Brunei Dar	Both	55+ years	Non-melanoma skin cancer (basal-cell	Percent	2021	4.74E-05	6.30E-05	3.38E-05
Incidence	Brunei Dar	Male	55+ years	Non-melanoma skin cancer (basal-cell	Rate	2021	18.688003	24.44923257	13.77701026
Incidence	Brunei Dar	Female	55+ years	Non-melanoma skin cancer (basal-cell	Rate	2021	16.406051	21.29119337	12.23023268
Incidence	Brunei Dar	Both	55+ years	Non-melanoma skin cancer (basal-cell	Rate	2021	17.527391	22.73339536	13.07500552
Incidence	Japan	Male	55+ years	Non-melanoma skin cancer (basal-cell	Number	1990	1638.6918	2088.514483	1238.561233
Incidence	Japan	Female	55+ years	Non-melanoma skin cancer (basal-cell	Number	1990	1709.2085	2148.899236	1316.253091
Incidence	Japan	Both	55+ years	Non-melanoma skin cancer (basal-cell	Number	1990	3347.9003	4223.46213	2572.578771
Incidence	Japan	Male	55+ years	Non-melanoma skin cancer (basal-cell	Percent	1990	3.32E-05	4.32E-05	2.46E-05
Incidence	Japan	Female	55+ years	Non-melanoma skin cancer (basal-cell	Percent	1990	2.75E-05	3.51E-05	2.09E-05
Incidence	Japan	Both	55+ years	Non-melanoma skin cancer (basal-cell	Percent	1990	3.01E-05	3.83E-05	2.26E-05
Incidence	Japan	Male	55+ years	Non-melanoma skin cancer (basal-cell	Rate	1990	12.469927	15.89293525	9.425059601
Incidence	Japan	Female	55+ years	Non-melanoma skin cancer (basal-cell	Rate	1990	10.376383	13.04568882	7.990801965
Incidence	Japan	Both	55+ years	Non-melanoma skin cancer (basal-cell	Rate	1990	11.305412	14.26206766	8.687255
Incidence	Japan	Male	55+ years	Non-melanoma skin cancer (basal-cell	Number	2021	5607.2645	7185.398618	4282.323455
Incidence	Japan	Female	55+ years	Non-melanoma skin cancer (basal-cell	Number	2021	6562.8619	8243.947477	5019.032836
Incidence	Japan	Both	55+ years	Non-melanoma skin cancer (basal-cell	Number	2021	12170.126	15403.769	9373.185817
Incidence	Japan	Male	55+ years	Non-melanoma skin cancer (basal-cell	Percent	2021	5.99E-05	7.74E-05	4.47E-05
Incidence	Japan	Female	55+ years	Non-melanoma skin cancer (basal-cell	Percent	2021	5.83E-05	7.32E-05	4.42E-05
Incidence	Japan	Both	55+ years	Non-melanoma skin cancer (basal-cell	Percent	2021	5.90E-05	7.52E-05	4.43E-05
Incidence	Japan	Male	55+ years	Non-melanoma skin cancer (basal-cell	Rate	2021	23.607727	30.25199335	18.02945495
Incidence	Japan	Female	55+ years	Non-melanoma skin cancer (basal-cell	Rate	2021	23.068793	28.97789494	17.64215585
Incidence	Japan	Both	55+ years	Non-melanoma skin cancer (basal-cell	Rate	2021	23.314012	29.5086222	17.95598201
Incidence	Denmark	Male	55+ years	Non-melanoma skin cancer (basal-cell	Number	1990	1484.0556	1577.038322	1393.860407
Incidence	Denmark	Female	55+ years	Non-melanoma skin cancer (basal-cell	Number	1990	1487.3246	1615.740257	1362.836251
Incidence	Denmark	Both	55+ years	Non-melanoma skin cancer (basal-cell	Number	1990	2971.3802	3135.345984	2791.568323

Incidence	Denmark	Male	55+ years	Non-melanoma skin cancer (basal-cell	Percent	1990	0.0007274	0.000793013	0.000654597
Incidence	Denmark	Female	55+ years	Non-melanoma skin cancer (basal-cell	Percent	1990	0.0005059	0.000556401	0.000451541
Incidence	Denmark	Both	55+ years	Non-melanoma skin cancer (basal-cell	Percent	1990	0.0005966	0.000651114	0.000541696
Incidence	Denmark	Male	55+ years	Non-melanoma skin cancer (basal-cell	Rate	1990	258.3671	274.5549582	242.6645443
Incidence	Denmark	Female	55+ years	Non-melanoma skin cancer (basal-cell	Rate	1990	203.67756	221.2630871	186.6298464
Incidence	Denmark	Both	55+ years	Non-melanoma skin cancer (basal-cell	Rate	1990	227.75603	240.3239942	213.9734667
Incidence	Denmark	Male	55+ years	Non-melanoma skin cancer (basal-cell	Number	2021	2105.1752	2721.69806	1622.289554
Incidence	Denmark	Female	55+ years	Non-melanoma skin cancer (basal-cell	Number	2021	1672.7252	2091.765099	1322.300168
Incidence	Denmark	Both	55+ years	Non-melanoma skin cancer (basal-cell	Number	2021	3777.9004	4829.705955	2974.82795
Incidence	Denmark	Male	55+ years	Non-melanoma skin cancer (basal-cell	Percent	2021	0.0005931	0.000768128	0.000448681
Incidence	Denmark	Female	55+ years	Non-melanoma skin cancer (basal-cell	Percent	2021	0.0003796	0.000474832	0.000291387
Incidence	Denmark	Both	55+ years	Non-melanoma skin cancer (basal-cell	Percent	2021	0.0004748	0.000601452	0.000360175
Incidence	Denmark	Male	55+ years	Non-melanoma skin cancer (basal-cell	Rate	2021	230.07779	297.4585022	177.3024818
Incidence	Denmark	Female	55+ years	Non-melanoma skin cancer (basal-cell	Rate	2021	165.57725	207.0565496	130.8898932
Incidence	Denmark	Both	55+ years	Non-melanoma skin cancer (basal-cell	Rate	2021	196.23187	250.8647966	154.5186427
Incidence	North Mace	Male	55+ years	Non-melanoma skin cancer (basal-cell	Number	1990	199.77151	254.1234605	152.9250456
Incidence	North Mace	Female	55+ years	Non-melanoma skin cancer (basal-cell	Number	1990	183.53495	233.3086743	138.9013542
Incidence	North Mace	Both	55+ years	Non-melanoma skin cancer (basal-cell	Number	1990	383.30646	487.5640158	290.4255668
Incidence	North Mace	Male	55+ years	Non-melanoma skin cancer (basal-cell	Percent	1990	0.0003847	0.000488941	0.000284789
Incidence	North Mace	Female	55+ years	Non-melanoma skin cancer (basal-cell	Percent	1990	0.0002994	0.000381844	0.000221079
Incidence	North Mace	Both	55+ years	Non-melanoma skin cancer (basal-cell	Percent	1990	0.0003384	0.000432084	0.000249898
Incidence	North Mace	Male	55+ years	Non-melanoma skin cancer (basal-cell	Rate	1990	128.20732	163.0887658	98.142678
Incidence	North Mace	Female	55+ years	Non-melanoma skin cancer (basal-cell	Rate	1990	105.99952	134.746033	80.22164844
Incidence	North Mace	Both	55+ years	Non-melanoma skin cancer (basal-cell	Rate	1990	116.51854	148.2110305	88.28435067
Incidence	North Mace	Male	55+ years	Non-melanoma skin cancer (basal-cell	Number	2021	376.57003	490.6802742	284.748291
Incidence	North Mace	Female	55+ years	Non-melanoma skin cancer (basal-cell	Number	2021	350.81027	452.1292346	266.6458061
Incidence	North Mace	Both	55+ years	Non-melanoma skin cancer (basal-cell	Number	2021	727.3803	941.5053208	553.5368639
Incidence	North Mace	Male	55+ years	Non-melanoma skin cancer (basal-cell	Percent	2021	0.0003325	0.000429153	0.00024412
Incidence	North Mace	Female	55+ years	Non-melanoma skin cancer (basal-cell	Percent	2021	0.0002693	0.000348546	0.000199888
Incidence	North Mace	Both	55+ years	Non-melanoma skin cancer (basal-cell	Percent	2021	0.0002986	0.000379611	0.000221998
Incidence	North Mace	Male	55+ years	Non-melanoma skin cancer (basal-cell	Rate	2021	131.80272	171.742276	99.66432758
Incidence	North Mace	Female	55+ years	Non-melanoma skin cancer (basal-cell	Rate	2021	114.19663	147.1782284	86.79920333
Incidence	North Mace	Both	55+ years	Non-melanoma skin cancer (basal-cell	Rate	2021	122.68059	158.795099	93.36000461
Incidence	Romania	Male	55+ years	Non-melanoma skin cancer (basal-cell	Number	1990	1391.603	1635.896975	1167.50177
Incidence	Romania	Female	55+ years	Non-melanoma skin cancer (basal-cell	Number	1990	1525.2216	1815.559478	1288.084286
Incidence	Romania	Both	55+ years	Non-melanoma skin cancer (basal-cell	Number	1990	2916.8246	3459.855568	2448.52697
Incidence	Romania	Male	55+ years	Non-melanoma skin cancer (basal-cell	Percent	1990	0.0001904	0.00023013	0.000153209
Incidence	Romania	Female	55+ years	Non-melanoma skin cancer (basal-cell	Percent	1990	0.0001555	0.000186952	0.000127108
Incidence	Romania	Both	55+ years	Non-melanoma skin cancer (basal-cell	Percent	1990	0.0001704	0.000204729	0.000138933
Incidence	Romania	Male	55+ years	Non-melanoma skin cancer (basal-cell	Rate	1990	62.119585	73.02459432	52.1159611
Incidence	Romania	Female	55+ years	Non-melanoma skin cancer (basal-cell	Rate	1990	55.188706	65.69430829	46.60811569
Incidence	Romania	Both	55+ years	Non-melanoma skin cancer (basal-cell	Rate	1990	58.291629	69.14389711	48.93287987
Incidence	Romania	Male	55+ years	Non-melanoma skin cancer (basal-cell	Number	2021	2999.5841	3821.56589	2298.205379
Incidence	Romania	Female	55+ years	Non-melanoma skin cancer (basal-cell	Number	2021	3397.8089	4323.836051	2639.383234
Incidence	Romania	Both	55+ years	Non-melanoma skin cancer (basal-cell	Number	2021	6397.393	8139.725188	4960.584769
Incidence	Romania	Male	55+ years	Non-melanoma skin cancer (basal-cell	Percent	2021	0.0003079	0.0003989	0.000233721
Incidence	Romania	Female	55+ years	Non-melanoma skin cancer (basal-cell	Percent	2021	0.0002479	0.000316227	0.000188285
Incidence	Romania	Both	55+ years	Non-melanoma skin cancer (basal-cell	Percent	2021	0.0002728	0.000349816	0.000209209
Incidence	Romania	Male	55+ years	Non-melanoma skin cancer (basal-cell	Rate	2021	116.20577	148.0498629	89.03391984
Incidence	Romania	Female	55+ years	Non-melanoma skin cancer (basal-cell	Rate	2021	99.232734	126.2772827	77.08297421
Incidence	Romania	Both	55+ years	Non-melanoma skin cancer (basal-cell	Rate	2021	106.52823	135.5412256	82.60275669
Incidence	Belarus	Male	55+ years	Non-melanoma skin cancer (basal-cell	Number	1990	769.61509	810.6795943	733.8930185
Incidence	Belarus	Female	55+ years	Non-melanoma skin cancer (basal-cell	Number	1990	1300.5801	1356.476701	1241.752238
Incidence	Belarus	Both	55+ years	Non-melanoma skin cancer (basal-cell	Number	1990	2070.1952	2156.331342	1987.058567
Incidence	Belarus	Male	55+ years	Non-melanoma skin cancer (basal-cell	Percent	1990	0.0002518	0.000276351	0.000227367
Incidence	Belarus	Female	55+ years	Non-melanoma skin cancer (basal-cell	Percent	1990	0.0002267	0.000247487	0.000206603

Incidence	Belarus	Both	55+ years	Non-melanoma skin cancer (basal-cell	Percent	1990	0.0002354	0.000255419	0.000214649
Incidence	Belarus	Male	55+ years	Non-melanoma skin cancer (basal-cell	Rate	1990	90.428902	95.25393482	86.23159906
Incidence	Belarus	Female	55+ years	Non-melanoma skin cancer (basal-cell	Rate	1990	88.845804	92.6642359	84.82712771
Incidence	Belarus	Both	55+ years	Non-melanoma skin cancer (basal-cell	Rate	1990	89.427821	93.14870972	85.83650298
Incidence	Belarus	Male	55+ years	Non-melanoma skin cancer (basal-cell	Number	2021	1065.5149	1362.222792	815.3845731
Incidence	Belarus	Female	55+ years	Non-melanoma skin cancer (basal-cell	Number	2021	1719.0128	2205.229242	1311.82431
Incidence	Belarus	Both	55+ years	Non-melanoma skin cancer (basal-cell	Number	2021	2784.5277	3543.332305	2131.913105
Incidence	Belarus	Male	55+ years	Non-melanoma skin cancer (basal-cell	Percent	2021	0.0002293	0.000296236	0.000169207
Incidence	Belarus	Female	55+ years	Non-melanoma skin cancer (basal-cell	Percent	2021	0.0002176	0.000280254	0.00016281
Incidence	Belarus	Both	55+ years	Non-melanoma skin cancer (basal-cell	Percent	2021	0.0002219	0.000284836	0.000166371
Incidence	Belarus	Male	55+ years	Non-melanoma skin cancer (basal-cell	Rate	2021	96.050279	122.7968546	73.50241202
Incidence	Belarus	Female	55+ years	Non-melanoma skin cancer (basal-cell	Rate	2021	97.26927	124.7815235	74.22876172
Incidence	Belarus	Both	55+ years	Non-melanoma skin cancer (basal-cell	Rate	2021	96.79918	123.1776779	74.11218683
Incidence	Russian Fe	Male	55+ years	Non-melanoma skin cancer (basal-cell	Number	1990	6212.665	7874.427564	4642.284455
Incidence	Russian Fe	Female	55+ years	Non-melanoma skin cancer (basal-cell	Number	1990	12565.483	16055.81439	9566.872413
Incidence	Russian Fe	Both	55+ years	Non-melanoma skin cancer (basal-cell	Number	1990	18778.148	23947.4815	14208.07823
Incidence	Russian Fe	Male	55+ years	Non-melanoma skin cancer (basal-cell	Percent	1990	0.0001452	0.000187223	0.000105771
Incidence	Russian Fe	Female	55+ years	Non-melanoma skin cancer (basal-cell	Percent	1990	0.0001493	0.000192357	0.000110962
Incidence	Russian Fe	Both	55+ years	Non-melanoma skin cancer (basal-cell	Percent	1990	0.0001479	0.000190847	0.000109595
Incidence	Russian Fe	Male	55+ years	Non-melanoma skin cancer (basal-cell	Rate	1990	56.426279	71.51917054	42.1633612
Incidence	Russian Fe	Female	55+ years	Non-melanoma skin cancer (basal-cell	Rate	1990	61.138462	78.12097926	46.54846047
Incidence	Russian Fe	Both	55+ years	Non-melanoma skin cancer (basal-cell	Rate	1990	59.494681	75.87264739	45.01536036
Incidence	Russian Fe	Male	55+ years	Non-melanoma skin cancer (basal-cell	Number	2021	13451.41	16974.66058	10326.8227
Incidence	Russian Fe	Female	55+ years	Non-melanoma skin cancer (basal-cell	Number	2021	22139.872	27974.85294	17143.11544
Incidence	Russian Fe	Both	55+ years	Non-melanoma skin cancer (basal-cell	Number	2021	35591.283	44945.78766	27359.63338
Incidence	Russian Fe	Male	55+ years	Non-melanoma skin cancer (basal-cell	Percent	2021	0.0001804	0.000228648	0.000135159
Incidence	Russian Fe	Female	55+ years	Non-melanoma skin cancer (basal-cell	Percent	2021	0.0001758	0.000224013	0.000132004
Incidence	Russian Fe	Both	55+ years	Non-melanoma skin cancer (basal-cell	Percent	2021	0.0001775	0.000225907	0.000133116
Incidence	Russian Fe	Male	55+ years	Non-melanoma skin cancer (basal-cell	Rate	2021	82.13726	103.6509973	63.05784242
Incidence	Russian Fe	Female	55+ years	Non-melanoma skin cancer (basal-cell	Rate	2021	84.734649	107.0665335	65.61085227
Incidence	Russian Fe	Both	55+ years	Non-melanoma skin cancer (basal-cell	Rate	2021	83.733907	105.7418032	64.36769983
Incidence	Ukraine	Male	55+ years	Non-melanoma skin cancer (basal-cell	Number	1990	4065.5018	5238.616111	3083.755336
Incidence	Ukraine	Female	55+ years	Non-melanoma skin cancer (basal-cell	Number	1990	7570.4819	9664.864509	5791.52168
Incidence	Ukraine	Both	55+ years	Non-melanoma skin cancer (basal-cell	Number	1990	11635.984	14924.28688	8855.81515
Incidence	Ukraine	Male	55+ years	Non-melanoma skin cancer (basal-cell	Percent	1990	0.0002363	0.000306136	0.000175069
Incidence	Ukraine	Female	55+ years	Non-melanoma skin cancer (basal-cell	Percent	1990	0.0002292	0.000296888	0.000171079
Incidence	Ukraine	Both	55+ years	Non-melanoma skin cancer (basal-cell	Percent	1990	0.0002316	0.000298413	0.000173235
Incidence	Ukraine	Male	55+ years	Non-melanoma skin cancer (basal-cell	Rate	1990	90.374814	116.4527719	68.55090144
Incidence	Ukraine	Female	55+ years	Non-melanoma skin cancer (basal-cell	Rate	1990	95.005128	121.2884073	72.68021601
Incidence	Ukraine	Both	55+ years	Non-melanoma skin cancer (basal-cell	Rate	1990	93.334362	119.7104455	71.03411944
Incidence	Ukraine	Male	55+ years	Non-melanoma skin cancer (basal-cell	Number	2021	5173.6891	6643.704783	3959.017529
Incidence	Ukraine	Female	55+ years	Non-melanoma skin cancer (basal-cell	Number	2021	8299.6857	10340.97086	6371.096614
Incidence	Ukraine	Both	55+ years	Non-melanoma skin cancer (basal-cell	Number	2021	13473.375	17017.23072	10336.23494
Incidence	Ukraine	Male	55+ years	Non-melanoma skin cancer (basal-cell	Percent	2021	0.0002254	0.000293581	0.000168214
Incidence	Ukraine	Female	55+ years	Non-melanoma skin cancer (basal-cell	Percent	2021	0.0002117	0.000270013	0.000160692
Incidence	Ukraine	Both	55+ years	Non-melanoma skin cancer (basal-cell	Percent	2021	0.0002167	0.000277212	0.000163378
Incidence	Ukraine	Male	55+ years	Non-melanoma skin cancer (basal-cell	Rate	2021	99.26884	127.4743902	75.96263859
Incidence	Ukraine	Female	55+ years	Non-melanoma skin cancer (basal-cell	Rate	2021	99.204083	123.6030587	76.15213689
Incidence	Ukraine	Both	55+ years	Non-melanoma skin cancer (basal-cell	Rate	2021	99.228939	125.3287897	76.12447857
Incidence	Singapore	Male	55+ years	Non-melanoma skin cancer (basal-cell	Number	1990	56.78136	62.56600351	51.93235628
Incidence	Singapore	Female	55+ years	Non-melanoma skin cancer (basal-cell	Number	1990	67.870424	73.68969199	62.56829352
Incidence	Singapore	Both	55+ years	Non-melanoma skin cancer (basal-cell	Number	1990	124.65178	135.2603308	115.3454935
Incidence	Singapore	Male	55+ years	Non-melanoma skin cancer (basal-cell	Percent	1990	9.29E-05	0.000106139	8.17E-05
Incidence	Singapore	Female	55+ years	Non-melanoma skin cancer (basal-cell	Percent	1990	9.58E-05	0.000107711	8.44E-05
Incidence	Singapore	Both	55+ years	Non-melanoma skin cancer (basal-cell	Percent	1990	9.44E-05	0.000106377	8.30E-05
Incidence	Singapore	Male	55+ years	Non-melanoma skin cancer (basal-cell	Rate	1990	33.818953	37.26428395	30.93088838

Incidence	Singapore	Female	55+ years	Non-melanoma skin cancer (basal-cell	Rate	1990	34.864202	37.85348873	32.14056308
Incidence	Singapore	Both	55+ years	Non-melanoma skin cancer (basal-cell	Rate	1990	34.380169	37.3061091	31.81340412
Incidence	Singapore	Male	55+ years	Non-melanoma skin cancer (basal-cell	Number	2021	197.50578	254.2087208	148.8313799
Incidence	Singapore	Female	55+ years	Non-melanoma skin cancer (basal-cell	Number	2021	190.29428	237.1775779	146.5277622
Incidence	Singapore	Both	55+ years	Non-melanoma skin cancer (basal-cell	Number	2021	387.80005	491.0098637	295.0739539
Incidence	Singapore	Male	55+ years	Non-melanoma skin cancer (basal-cell	Percent	2021	7.09E-05	9.37E-05	5.26E-05
Incidence	Singapore	Female	55+ years	Non-melanoma skin cancer (basal-cell	Percent	2021	6.68E-05	8.46E-05	4.95E-05
Incidence	Singapore	Both	55+ years	Non-melanoma skin cancer (basal-cell	Percent	2021	6.88E-05	8.85E-05	5.18E-05
Incidence	Singapore	Male	55+ years	Non-melanoma skin cancer (basal-cell	Rate	2021	26.157828	33.66761285	19.71135082
Incidence	Singapore	Female	55+ years	Non-melanoma skin cancer (basal-cell	Rate	2021	24.915026	31.05340625	19.18472296
Incidence	Singapore	Both	55+ years	Non-melanoma skin cancer (basal-cell	Rate	2021	25.53286	32.32822142	19.42774845
Incidence	Australia	Male	55+ years	Non-melanoma skin cancer (basal-cell	Number	1990	2228.9738	2802.594826	1674.267955
Incidence	Australia	Female	55+ years	Non-melanoma skin cancer (basal-cell	Number	1990	2001.0105	2500.026094	1550.117486
Incidence	Australia	Both	55+ years	Non-melanoma skin cancer (basal-cell	Number	1990	4229.9842	5280.35729	3247.98435
Incidence	Australia	Male	55+ years	Non-melanoma skin cancer (basal-cell	Percent	1990	0.0003697	0.000471629	0.000273395
Incidence	Australia	Female	55+ years	Non-melanoma skin cancer (basal-cell	Percent	1990	0.0002667	0.000338807	0.000199929
Incidence	Australia	Both	55+ years	Non-melanoma skin cancer (basal-cell	Percent	1990	0.0003125	0.000397089	0.00023335
Incidence	Australia	Male	55+ years	Non-melanoma skin cancer (basal-cell	Rate	1990	147.9648	186.0431868	111.1420542
Incidence	Australia	Female	55+ years	Non-melanoma skin cancer (basal-cell	Rate	1990	112.72569	140.8374299	87.32491363
Incidence	Australia	Both	55+ years	Non-melanoma skin cancer (basal-cell	Rate	1990	128.90255	160.9111265	98.97754868
Incidence	Australia	Male	55+ years	Non-melanoma skin cancer (basal-cell	Number	2021	5393.3326	6810.198786	4148.76286
Incidence	Australia	Female	55+ years	Non-melanoma skin cancer (basal-cell	Number	2021	4007.0209	4985.466964	3061.914168
Incidence	Australia	Both	55+ years	Non-melanoma skin cancer (basal-cell	Number	2021	9400.3535	11847.33275	7165.191699
Incidence	Australia	Male	55+ years	Non-melanoma skin cancer (basal-cell	Percent	2021	0.0003845	0.000495911	0.000289659
Incidence	Australia	Female	55+ years	Non-melanoma skin cancer (basal-cell	Percent	2021	0.0002461	0.000314674	0.000184683
Incidence	Australia	Both	55+ years	Non-melanoma skin cancer (basal-cell	Percent	2021	0.0003101	0.000392735	0.000234459
Incidence	Australia	Male	55+ years	Non-melanoma skin cancer (basal-cell	Rate	2021	153.09857	193.3186363	117.7694225
Incidence	Australia	Female	55+ years	Non-melanoma skin cancer (basal-cell	Rate	2021	103.1397	128.3246545	78.8128937
Incidence	Australia	Both	55+ years	Non-melanoma skin cancer (basal-cell	Rate	2021	126.89759	159.9299415	96.72461421
Incidence	Austria	Male	55+ years	Non-melanoma skin cancer (basal-cell	Number	1990	1100.2689	1238.988634	981.3406554
Incidence	Austria	Female	55+ years	Non-melanoma skin cancer (basal-cell	Number	1990	1351.0562	1502.375355	1204.944062
Incidence	Austria	Both	55+ years	Non-melanoma skin cancer (basal-cell	Number	1990	2451.3251	2722.915776	2199.484602
Incidence	Austria	Male	55+ years	Non-melanoma skin cancer (basal-cell	Percent	1990	0.0003802	0.000433565	0.00032641
Incidence	Austria	Female	55+ years	Non-melanoma skin cancer (basal-cell	Percent	1990	0.0002764	0.000314122	0.000242346
Incidence	Austria	Both	55+ years	Non-melanoma skin cancer (basal-cell	Percent	1990	0.000315	0.000354189	0.000276848
Incidence	Austria	Male	55+ years	Non-melanoma skin cancer (basal-cell	Rate	1990	142.83537	160.8437782	127.3962766
Incidence	Austria	Female	55+ years	Non-melanoma skin cancer (basal-cell	Rate	1990	114.92403	127.7955901	102.495383
Incidence	Austria	Both	55+ years	Non-melanoma skin cancer (basal-cell	Rate	1990	125.97296	139.9299323	113.0309406
Incidence	Austria	Male	55+ years	Non-melanoma skin cancer (basal-cell	Number	2021	2307.8253	2922.166297	1781.564221
Incidence	Austria	Female	55+ years	Non-melanoma skin cancer (basal-cell	Number	2021	1928.4512	2421.652259	1518.041243
Incidence	Austria	Both	55+ years	Non-melanoma skin cancer (basal-cell	Number	2021	4236.2765	5324.431965	3276.284412
Incidence	Austria	Male	55+ years	Non-melanoma skin cancer (basal-cell	Percent	2021	0.0004465	0.000574137	0.000344421
Incidence	Austria	Female	55+ years	Non-melanoma skin cancer (basal-cell	Percent	2021	0.0002895	0.000365191	0.000222692
Incidence	Austria	Both	55+ years	Non-melanoma skin cancer (basal-cell	Percent	2021	0.0003581	0.000456149	0.000273519
Incidence	Austria	Male	55+ years	Non-melanoma skin cancer (basal-cell	Rate	2021	170.22113	215.5338368	131.4050376
Incidence	Austria	Female	55+ years	Non-melanoma skin cancer (basal-cell	Rate	2021	120.57399	151.4107611	94.91361907
Incidence	Austria	Both	55+ years	Non-melanoma skin cancer (basal-cell	Rate	2021	143.35121	180.1732626	110.8660709
Incidence	Belgium	Male	55+ years	Non-melanoma skin cancer (basal-cell	Number	1990	1860.3718	2370.393957	1447.148043
Incidence	Belgium	Female	55+ years	Non-melanoma skin cancer (basal-cell	Number	1990	1822.8024	2242.298332	1413.531632
Incidence	Belgium	Both	55+ years	Non-melanoma skin cancer (basal-cell	Number	1990	3683.1742	4574.322643	2858.884538
Incidence	Belgium	Male	55+ years	Non-melanoma skin cancer (basal-cell	Percent	1990	0.0004718	0.000608159	0.000362395
Incidence	Belgium	Female	55+ years	Non-melanoma skin cancer (basal-cell	Percent	1990	0.0003102	0.000387575	0.000236678
Incidence	Belgium	Both	55+ years	Non-melanoma skin cancer (basal-cell	Percent	1990	0.0003751	0.000474053	0.000287202
Incidence	Belgium	Male	55+ years	Non-melanoma skin cancer (basal-cell	Rate	1990	164.2677	209.301805	127.7807415
Incidence	Belgium	Female	55+ years	Non-melanoma skin cancer (basal-cell	Rate	1990	122.38527	150.5507503	94.90630424
Incidence	Belgium	Both	55+ years	Non-melanoma skin cancer (basal-cell	Rate	1990	140.47615	174.4645251	109.0377685

Incidence	Belgium	Male	55+ years	Non-melanoma skin cancer (basal-cell	Number	2021	3295.2925	4213.761239	2566.019192
Incidence	Belgium	Female	55+ years	Non-melanoma skin cancer (basal-cell	Number	2021	2748.2744	3434.989662	2106.261215
Incidence	Belgium	Both	55+ years	Non-melanoma skin cancer (basal-cell	Number	2021	6043.5669	7605.161406	4650.526288
Incidence	Belgium	Male	55+ years	Non-melanoma skin cancer (basal-cell	Percent	2021	0.0004566	0.000586779	0.000346312
Incidence	Belgium	Female	55+ years	Non-melanoma skin cancer (basal-cell	Percent	2021	0.0002935	0.000365106	0.000224408
Incidence	Belgium	Both	55+ years	Non-melanoma skin cancer (basal-cell	Percent	2021	0.0003645	0.000461263	0.000278482
Incidence	Belgium	Male	55+ years	Non-melanoma skin cancer (basal-cell	Rate	2021	187.41429	239.6506751	145.9380816
Incidence	Belgium	Female	55+ years	Non-melanoma skin cancer (basal-cell	Rate	2021	135.88638	169.8405021	104.142515
Incidence	Belgium	Both	55+ years	Non-melanoma skin cancer (basal-cell	Rate	2021	159.85004	201.153623	123.0046493
Incidence	Iceland	Male	55+ years	Non-melanoma skin cancer (basal-cell	Number	1990	43.002664	54.47183922	33.1776148
Incidence	Iceland	Female	55+ years	Non-melanoma skin cancer (basal-cell	Number	1990	35.021983	43.50261451	26.99197794
Incidence	Iceland	Both	55+ years	Non-melanoma skin cancer (basal-cell	Number	1990	78.024646	97.68725359	60.17592183
Incidence	Iceland	Male	55+ years	Non-melanoma skin cancer (basal-cell	Percent	1990	0.0005364	0.00069238	0.000399748
Incidence	Iceland	Female	55+ years	Non-melanoma skin cancer (basal-cell	Percent	1990	0.0003605	0.000459484	0.000274336
Incidence	Iceland	Both	55+ years	Non-melanoma skin cancer (basal-cell	Percent	1990	0.00044	0.000560613	0.000332904
Incidence	Iceland	Male	55+ years	Non-melanoma skin cancer (basal-cell	Rate	1990	192.8195	244.2461021	148.7649987
Incidence	Iceland	Female	55+ years	Non-melanoma skin cancer (basal-cell	Rate	1990	137.8125	171.1840341	106.2142063
Incidence	Iceland	Both	55+ years	Non-melanoma skin cancer (basal-cell	Rate	1990	163.52293	204.7315337	126.1158269
Incidence	Iceland	Male	55+ years	Non-melanoma skin cancer (basal-cell	Number	2021	93.704989	117.3661919	72.50204159
Incidence	Iceland	Female	55+ years	Non-melanoma skin cancer (basal-cell	Number	2021	68.945346	85.29701105	53.32071208
Incidence	Iceland	Both	55+ years	Non-melanoma skin cancer (basal-cell	Number	2021	162.65033	200.811755	126.2490399
Incidence	Iceland	Male	55+ years	Non-melanoma skin cancer (basal-cell	Percent	2021	0.0005401	0.000684459	0.000405857
Incidence	Iceland	Female	55+ years	Non-melanoma skin cancer (basal-cell	Percent	2021	0.0003535	0.000440133	0.00027272
Incidence	Iceland	Both	55+ years	Non-melanoma skin cancer (basal-cell	Percent	2021	0.0004413	0.000551105	0.000338452
Incidence	Iceland	Male	55+ years	Non-melanoma skin cancer (basal-cell	Rate	2021	197.63936	247.5447584	152.9188268
Incidence	Iceland	Female	55+ years	Non-melanoma skin cancer (basal-cell	Rate	2021	137.96223	170.682527	106.6967502
Incidence	Iceland	Both	55+ years	Non-melanoma skin cancer (basal-cell	Rate	2021	167.01582	206.2014851	129.637528
Incidence	Republic o	Male	55+ years	Non-melanoma skin cancer (basal-cell	Number	1990	273.82648	349.2898979	206.8752038
Incidence	Republic o	Female	55+ years	Non-melanoma skin cancer (basal-cell	Number	1990	403.66546	514.4271626	305.1840376
Incidence	Republic o	Both	55+ years	Non-melanoma skin cancer (basal-cell	Number	1990	677.49194	858.7319389	516.8490244
Incidence	Republic o	Male	55+ years	Non-melanoma skin cancer (basal-cell	Percent	1990	0.0002476	0.000320214	0.000182478
Incidence	Republic o	Female	55+ years	Non-melanoma skin cancer (basal-cell	Percent	1990	0.000225	0.000288815	0.000164156
Incidence	Republic o	Both	55+ years	Non-melanoma skin cancer (basal-cell	Percent	1990	0.0002336	0.000300281	0.000170873
Incidence	Republic o	Male	55+ years	Non-melanoma skin cancer (basal-cell	Rate	1990	87.8409	112.0488389	66.36357515
Incidence	Republic o	Female	55+ years	Non-melanoma skin cancer (basal-cell	Rate	1990	87.699256	111.7630423	66.30345168
Incidence	Republic o	Both	55+ years	Non-melanoma skin cancer (basal-cell	Rate	1990	87.75645	111.232714	66.94815591
Incidence	Republic o	Male	55+ years	Non-melanoma skin cancer (basal-cell	Number	2021	406.21348	525.6399822	305.1191473
Incidence	Republic o	Female	55+ years	Non-melanoma skin cancer (basal-cell	Number	2021	586.64891	738.1150229	455.4047671
Incidence	Republic o	Both	55+ years	Non-melanoma skin cancer (basal-cell	Number	2021	992.86239	1269.401567	760.9200652
Incidence	Republic o	Male	55+ years	Non-melanoma skin cancer (basal-cell	Percent	2021	0.0002364	0.000304228	0.000174139
Incidence	Republic o	Female	55+ years	Non-melanoma skin cancer (basal-cell	Percent	2021	0.0002146	0.000274942	0.000161304
Incidence	Republic o	Both	55+ years	Non-melanoma skin cancer (basal-cell	Percent	2021	0.000223	0.000285815	0.000166838
Incidence	Republic o	Male	55+ years	Non-melanoma skin cancer (basal-cell	Rate	2021	95.009187	122.9418285	71.36425529
Incidence	Republic o	Female	55+ years	Non-melanoma skin cancer (basal-cell	Rate	2021	93.373612	117.4816216	72.48421839
Incidence	Republic o	Both	55+ years	Non-melanoma skin cancer (basal-cell	Rate	2021	94.035926	120.2274888	72.06821783
Incidence	Canada	Male	55+ years	Non-melanoma skin cancer (basal-cell	Number	1990	3215.6266	4152.27177	2374.099512
Incidence	Canada	Female	55+ years	Non-melanoma skin cancer (basal-cell	Number	1990	3514.0461	4426.990956	2702.58879
Incidence	Canada	Both	55+ years	Non-melanoma skin cancer (basal-cell	Number	1990	6729.6727	8488.469414	5137.656364
Incidence	Canada	Male	55+ years	Non-melanoma skin cancer (basal-cell	Percent	1990	0.0003147	0.000411719	0.000227273
Incidence	Canada	Female	55+ years	Non-melanoma skin cancer (basal-cell	Percent	1990	0.0002772	0.000355417	0.000207446
Incidence	Canada	Both	55+ years	Non-melanoma skin cancer (basal-cell	Percent	1990	0.0002939	0.000374257	0.000215983
Incidence	Canada	Male	55+ years	Non-melanoma skin cancer (basal-cell	Rate	1990	130.76552	168.8547965	96.5442804
Incidence	Canada	Female	55+ years	Non-melanoma skin cancer (basal-cell	Rate	1990	117.13737	147.5695132	90.08821481
Incidence	Canada	Both	55+ years	Non-melanoma skin cancer (basal-cell	Rate	1990	123.27633	155.4945425	94.1132596
Incidence	Canada	Male	55+ years	Non-melanoma skin cancer (basal-cell	Number	2021	9134.4322	11611.81141	6931.014439
Incidence	Canada	Female	55+ years	Non-melanoma skin cancer (basal-cell	Number	2021	9443.434	11880.61074	7049.016637

Incidence	Canada	Both	55+ years	Non-melanoma skin cancer (basal-cell	Number	2021	18577.866	23514.6504	14090.66514
Incidence	Canada	Male	55+ years	Non-melanoma skin cancer (basal-cell	Percent	2021	0.0003743	0.000484308	0.000271162
Incidence	Canada	Female	55+ years	Non-melanoma skin cancer (basal-cell	Percent	2021	0.00034	0.000432764	0.000243761
Incidence	Canada	Both	55+ years	Non-melanoma skin cancer (basal-cell	Percent	2021	0.000356	0.00045511	0.00025829
Incidence	Canada	Male	55+ years	Non-melanoma skin cancer (basal-cell	Rate	2021	157.4671	200.1742609	119.4827097
Incidence	Canada	Female	55+ years	Non-melanoma skin cancer (basal-cell	Rate	2021	145.94972	183.6166559	108.9436301
Incidence	Canada	Both	55+ years	Non-melanoma skin cancer (basal-cell	Rate	2021	151.39423	191.6249312	114.8272541
Incidence	Estonia	Male	55+ years	Non-melanoma skin cancer (basal-cell	Number	1990	132.84423	143.1282924	122.5643762
Incidence	Estonia	Female	55+ years	Non-melanoma skin cancer (basal-cell	Number	1990	240.76177	256.5019477	225.7803176
Incidence	Estonia	Both	55+ years	Non-melanoma skin cancer (basal-cell	Number	1990	373.60601	395.3317097	350.4470629
Incidence	Estonia	Male	55+ years	Non-melanoma skin cancer (basal-cell	Percent	1990	0.0002822	0.000311393	0.000250774
Incidence	Estonia	Female	55+ years	Non-melanoma skin cancer (basal-cell	Percent	1990	0.0002697	0.000294932	0.000243015
Incidence	Estonia	Both	55+ years	Non-melanoma skin cancer (basal-cell	Percent	1990	0.000274	0.000299111	0.000245517
Incidence	Estonia	Male	55+ years	Non-melanoma skin cancer (basal-cell	Rate	1990	101.96164	109.8549375	94.07156089
Incidence	Estonia	Female	55+ years	Non-melanoma skin cancer (basal-cell	Rate	1990	105.51229	112.4103151	98.94675995
Incidence	Estonia	Both	55+ years	Non-melanoma skin cancer (basal-cell	Rate	1990	104.22179	110.2824249	97.76132539
Incidence	Estonia	Male	55+ years	Non-melanoma skin cancer (basal-cell	Number	2021	182.28923	233.4498661	141.2001474
Incidence	Estonia	Female	55+ years	Non-melanoma skin cancer (basal-cell	Number	2021	281.78405	354.9669064	219.5931828
Incidence	Estonia	Both	55+ years	Non-melanoma skin cancer (basal-cell	Number	2021	464.07328	590.8615117	356.3099355
Incidence	Estonia	Male	55+ years	Non-melanoma skin cancer (basal-cell	Percent	2021	0.0002755	0.000356518	0.000206081
Incidence	Estonia	Female	55+ years	Non-melanoma skin cancer (basal-cell	Percent	2021	0.0002505	0.000322107	0.000190748
Incidence	Estonia	Both	55+ years	Non-melanoma skin cancer (basal-cell	Percent	2021	0.0002597	0.00033061	0.000194625
Incidence	Estonia	Male	55+ years	Non-melanoma skin cancer (basal-cell	Rate	2021	106.37715	136.2325726	82.39910204
Incidence	Estonia	Female	55+ years	Non-melanoma skin cancer (basal-cell	Rate	2021	105.88845	133.3890058	82.51844272
Incidence	Estonia	Both	55+ years	Non-melanoma skin cancer (basal-cell	Rate	2021	106.07987	135.0616741	81.44686267
Incidence	Republic o	Male	55+ years	Non-melanoma skin cancer (basal-cell	Number	1990	239.48986	259.7504616	218.5805059
Incidence	Republic o	Female	55+ years	Non-melanoma skin cancer (basal-cell	Number	1990	284.32381	307.2141636	261.6946167
Incidence	Republic o	Both	55+ years	Non-melanoma skin cancer (basal-cell	Number	1990	523.81367	561.6275214	484.0924864
Incidence	Republic o	Male	55+ years	Non-melanoma skin cancer (basal-cell	Percent	1990	3.15E-05	3.58E-05	2.76E-05
Incidence	Republic o	Female	55+ years	Non-melanoma skin cancer (basal-cell	Percent	1990	2.72E-05	3.09E-05	2.38E-05
Incidence	Republic o	Both	55+ years	Non-melanoma skin cancer (basal-cell	Percent	1990	2.90E-05	3.27E-05	2.56E-05
Incidence	Republic o	Male	55+ years	Non-melanoma skin cancer (basal-cell	Rate	1990	11.507398	12.48091274	10.50271173
Incidence	Republic o	Female	55+ years	Non-melanoma skin cancer (basal-cell	Rate	1990	9.8192718	10.6098022	9.037760784
Incidence	Republic o	Both	55+ years	Non-melanoma skin cancer (basal-cell	Rate	1990	10.525214	11.28502397	9.727079076
Incidence	Republic o	Male	55+ years	Non-melanoma skin cancer (basal-cell	Number	2021	1486.3618	1600.910566	1363.607707
Incidence	Republic o	Female	55+ years	Non-melanoma skin cancer (basal-cell	Number	2021	2069.3341	2246.702939	1875.635793
Incidence	Republic o	Both	55+ years	Non-melanoma skin cancer (basal-cell	Number	2021	3555.696	3810.447152	3274.683125
Incidence	Republic o	Male	55+ years	Non-melanoma skin cancer (basal-cell	Percent	2021	5.15E-05	5.75E-05	4.52E-05
Incidence	Republic o	Female	55+ years	Non-melanoma skin cancer (basal-cell	Percent	2021	6.27E-05	7.09E-05	5.44E-05
Incidence	Republic o	Both	55+ years	Non-melanoma skin cancer (basal-cell	Percent	2021	5.75E-05	6.45E-05	5.06E-05
Incidence	Republic o	Male	55+ years	Non-melanoma skin cancer (basal-cell	Rate	2021	19.00379	20.46834562	17.43432421
Incidence	Republic o	Female	55+ years	Non-melanoma skin cancer (basal-cell	Rate	2021	23.244778	25.23715691	21.06896911
Incidence	Republic o	Both	55+ years	Non-melanoma skin cancer (basal-cell	Rate	2021	21.261345	22.78463395	19.58102379
Incidence	Israel	Male	55+ years	Non-melanoma skin cancer (basal-cell	Number	1990	722.61823	931.500169	552.0729716
Incidence	Israel	Female	55+ years	Non-melanoma skin cancer (basal-cell	Number	1990	574.97273	753.3196822	433.5611642
Incidence	Israel	Both	55+ years	Non-melanoma skin cancer (basal-cell	Number	1990	1297.591	1677.141083	987.6765444
Incidence	Israel	Male	55+ years	Non-melanoma skin cancer (basal-cell	Percent	1990	0.0005339	0.000686072	0.000402739
Incidence	Israel	Female	55+ years	Non-melanoma skin cancer (basal-cell	Percent	1990	0.0003195	0.000418513	0.000238031
Incidence	Israel	Both	55+ years	Non-melanoma skin cancer (basal-cell	Percent	1990	0.0004115	0.00053602	0.000311897
Incidence	Israel	Male	55+ years	Non-melanoma skin cancer (basal-cell	Rate	1990	196.41375	253.1896227	150.058102
Incidence	Israel	Female	55+ years	Non-melanoma skin cancer (basal-cell	Rate	1990	129.89661	170.1883776	97.94921445
Incidence	Israel	Both	55+ years	Non-melanoma skin cancer (basal-cell	Rate	1990	160.08873	206.9152657	121.8534067
Incidence	Israel	Male	55+ years	Non-melanoma skin cancer (basal-cell	Number	2021	1924.6713	2409.986021	1485.299039
Incidence	Israel	Female	55+ years	Non-melanoma skin cancer (basal-cell	Number	2021	1512.4084	1894.259497	1174.674708
Incidence	Israel	Both	55+ years	Non-melanoma skin cancer (basal-cell	Number	2021	3437.0797	4286.442044	2651.151901
Incidence	Israel	Male	55+ years	Non-melanoma skin cancer (basal-cell	Percent	2021	0.0004964	0.000615981	0.000374247

Incidence	Israel	Female	55+ years	Non-melanoma skin cancer (basal-cell	Percent	2021	0.0003127	0.000396604	0.000242365
Incidence	Israel	Both	55+ years	Non-melanoma skin cancer (basal-cell	Percent	2021	0.0003944	0.000491702	0.000302215
Incidence	Israel	Male	55+ years	Non-melanoma skin cancer (basal-cell	Rate	2021	207.71064	260.0858325	160.2935592
Incidence	Israel	Female	55+ years	Non-melanoma skin cancer (basal-cell	Rate	2021	141.09173	176.7144092	109.5847467
Incidence	Israel	Both	55+ years	Non-melanoma skin cancer (basal-cell	Rate	2021	171.97914	214.4781909	132.6541355
Incidence	Luxembourg	Male	55+ years	Non-melanoma skin cancer (basal-cell	Number	1990	71.834782	92.141421	54.32618357
Incidence	Luxembourg	Female	55+ years	Non-melanoma skin cancer (basal-cell	Number	1990	72.658535	93.73065307	55.54485733
Incidence	Luxembourg	Both	55+ years	Non-melanoma skin cancer (basal-cell	Number	1990	144.49332	185.1893602	109.7352036
Incidence	Luxembourg	Male	55+ years	Non-melanoma skin cancer (basal-cell	Percent	1990	0.0005151	0.000678717	0.000380532
Incidence	Luxembourg	Female	55+ years	Non-melanoma skin cancer (basal-cell	Percent	1990	0.0003447	0.000442746	0.000264277
Incidence	Luxembourg	Both	55+ years	Non-melanoma skin cancer (basal-cell	Percent	1990	0.0004125	0.00052864	0.000310223
Incidence	Luxembourg	Male	55+ years	Non-melanoma skin cancer (basal-cell	Rate	1990	181.0265	232.2000409	136.9041405
Incidence	Luxembourg	Female	55+ years	Non-melanoma skin cancer (basal-cell	Rate	1990	135.29442	174.5319227	103.4277521
Incidence	Luxembourg	Both	55+ years	Non-melanoma skin cancer (basal-cell	Rate	1990	154.72707	198.3054174	117.5072116
Incidence	Luxembourg	Male	55+ years	Non-melanoma skin cancer (basal-cell	Number	2021	163.93936	207.7130002	125.4943726
Incidence	Luxembourg	Female	55+ years	Non-melanoma skin cancer (basal-cell	Number	2021	127.43992	157.0719108	98.93893642
Incidence	Luxembourg	Both	55+ years	Non-melanoma skin cancer (basal-cell	Number	2021	291.37928	363.8829673	225.6674243
Incidence	Luxembourg	Male	55+ years	Non-melanoma skin cancer (basal-cell	Percent	2021	0.000508	0.000655715	0.000383969
Incidence	Luxembourg	Female	55+ years	Non-melanoma skin cancer (basal-cell	Percent	2021	0.0003308	0.000414206	0.000252625
Incidence	Luxembourg	Both	55+ years	Non-melanoma skin cancer (basal-cell	Percent	2021	0.0004115	0.000522602	0.000315838
Incidence	Luxembourg	Male	55+ years	Non-melanoma skin cancer (basal-cell	Rate	2021	191.16616	242.2096569	146.3362857
Incidence	Luxembourg	Female	55+ years	Non-melanoma skin cancer (basal-cell	Rate	2021	138.6053	170.8334411	107.6072666
Incidence	Luxembourg	Both	55+ years	Non-melanoma skin cancer (basal-cell	Rate	2021	163.97074	204.7714561	126.9920585
Incidence	Uruguay	Male	55+ years	Non-melanoma skin cancer (basal-cell	Number	1990	422.13577	502.7701296	356.5827712
Incidence	Uruguay	Female	55+ years	Non-melanoma skin cancer (basal-cell	Number	1990	341.44502	397.403842	291.8111183
Incidence	Uruguay	Both	55+ years	Non-melanoma skin cancer (basal-cell	Number	1990	763.58079	887.7225597	648.6688902
Incidence	Uruguay	Male	55+ years	Non-melanoma skin cancer (basal-cell	Percent	1990	0.00036	0.000432909	0.000293831
Incidence	Uruguay	Female	55+ years	Non-melanoma skin cancer (basal-cell	Percent	1990	0.0002153	0.000252426	0.000177501
Incidence	Uruguay	Both	55+ years	Non-melanoma skin cancer (basal-cell	Percent	1990	0.0002768	0.000324105	0.000226398
Incidence	Uruguay	Male	55+ years	Non-melanoma skin cancer (basal-cell	Rate	1990	141.48802	168.5143888	119.5165031
Incidence	Uruguay	Female	55+ years	Non-melanoma skin cancer (basal-cell	Rate	1990	90.709655	105.5758993	77.52371259
Incidence	Uruguay	Both	55+ years	Non-melanoma skin cancer (basal-cell	Rate	1990	113.16168	131.5593345	96.13189003
Incidence	Uruguay	Male	55+ years	Non-melanoma skin cancer (basal-cell	Number	2021	582.67188	736.1221674	445.5741112
Incidence	Uruguay	Female	55+ years	Non-melanoma skin cancer (basal-cell	Number	2021	550.86947	688.335884	422.7374801
Incidence	Uruguay	Both	55+ years	Non-melanoma skin cancer (basal-cell	Number	2021	1133.5414	1431.349226	872.7791109
Incidence	Uruguay	Male	55+ years	Non-melanoma skin cancer (basal-cell	Percent	2021	0.0003704	0.000475258	0.000281696
Incidence	Uruguay	Female	55+ years	Non-melanoma skin cancer (basal-cell	Percent	2021	0.0002472	0.000312031	0.000188987
Incidence	Uruguay	Both	55+ years	Non-melanoma skin cancer (basal-cell	Percent	2021	0.0002982	0.000379007	0.000229546
Incidence	Uruguay	Male	55+ years	Non-melanoma skin cancer (basal-cell	Rate	2021	149.61773	189.0204941	114.4139416
Incidence	Uruguay	Female	55+ years	Non-melanoma skin cancer (basal-cell	Rate	2021	106.99572	133.6959037	82.10856172
Incidence	Uruguay	Both	55+ years	Non-melanoma skin cancer (basal-cell	Rate	2021	125.35121	158.283914	96.5151559
Incidence	Finland	Male	55+ years	Non-melanoma skin cancer (basal-cell	Number	1990	895.09473	1109.542794	688.7562576
Incidence	Finland	Female	55+ years	Non-melanoma skin cancer (basal-cell	Number	1990	1004.2157	1275.734607	768.7309717
Incidence	Finland	Both	55+ years	Non-melanoma skin cancer (basal-cell	Number	1990	1899.3104	2374.339814	1463.821293
Incidence	Finland	Male	55+ years	Non-melanoma skin cancer (basal-cell	Percent	1990	0.0005225	0.000660329	0.000396867
Incidence	Finland	Female	55+ years	Non-melanoma skin cancer (basal-cell	Percent	1990	0.0003616	0.000464439	0.000269497
Incidence	Finland	Both	55+ years	Non-melanoma skin cancer (basal-cell	Percent	1990	0.0004229	0.000533125	0.000317527
Incidence	Finland	Male	55+ years	Non-melanoma skin cancer (basal-cell	Rate	1990	185.97465	230.5307161	143.1035145
Incidence	Finland	Female	55+ years	Non-melanoma skin cancer (basal-cell	Rate	1990	142.22951	180.6853831	108.87723
Incidence	Finland	Both	55+ years	Non-melanoma skin cancer (basal-cell	Rate	1990	159.96182	199.9692686	123.2844901
Incidence	Finland	Male	55+ years	Non-melanoma skin cancer (basal-cell	Number	2021	1975.5673	2521.751837	1522.59651
Incidence	Finland	Female	55+ years	Non-melanoma skin cancer (basal-cell	Number	2021	1643.4736	2077.331959	1267.233513
Incidence	Finland	Both	55+ years	Non-melanoma skin cancer (basal-cell	Number	2021	3619.0409	4588.348696	2790.233668
Incidence	Finland	Male	55+ years	Non-melanoma skin cancer (basal-cell	Percent	2021	0.0005849	0.000756542	0.000446442
Incidence	Finland	Female	55+ years	Non-melanoma skin cancer (basal-cell	Percent	2021	0.0003875	0.000489736	0.000294203
Incidence	Finland	Both	55+ years	Non-melanoma skin cancer (basal-cell	Percent	2021	0.000475	0.000608701	0.000364428

Incidence	Finland	Male	55+ years	Non-melanoma skin cancer (basal-cell	Rate	2021	212.82143	271.6601115	164.0243626
Incidence	Finland	Female	55+ years	Non-melanoma skin cancer (basal-cell	Rate	2021	151.55711	191.5664635	116.8611697
Incidence	Finland	Both	55+ years	Non-melanoma skin cancer (basal-cell	Rate	2021	179.81321	227.9735758	138.6336542
Incidence	France	Male	55+ years	Non-melanoma skin cancer (basal-cell	Number	1990	16820.654	18675.23023	14948.14761
Incidence	France	Female	55+ years	Non-melanoma skin cancer (basal-cell	Number	1990	17200.241	19139.3955	15187.41608
Incidence	France	Both	55+ years	Non-melanoma skin cancer (basal-cell	Number	1990	34020.895	37645.04658	30343.5992
Incidence	France	Male	55+ years	Non-melanoma skin cancer (basal-cell	Percent	1990	0.0007719	0.000884713	0.000668121
Incidence	France	Female	55+ years	Non-melanoma skin cancer (basal-cell	Percent	1990	0.0005288	0.000607625	0.000454462
Incidence	France	Both	55+ years	Non-melanoma skin cancer (basal-cell	Percent	1990	0.0006262	0.000709121	0.000545618
Incidence	France	Male	55+ years	Non-melanoma skin cancer (basal-cell	Rate	1990	281.3508	312.3713743	250.0302996
Incidence	France	Female	55+ years	Non-melanoma skin cancer (basal-cell	Rate	1990	216.68147	241.1101369	191.3247453
Incidence	France	Both	55+ years	Non-melanoma skin cancer (basal-cell	Rate	1990	244.46331	270.5053114	218.0394367
Incidence	France	Male	55+ years	Non-melanoma skin cancer (basal-cell	Number	2021	24627.248	31453.78394	18941.49091
Incidence	France	Female	55+ years	Non-melanoma skin cancer (basal-cell	Number	2021	20895.985	26239.07721	16738.33413
Incidence	France	Both	55+ years	Non-melanoma skin cancer (basal-cell	Number	2021	45523.233	57533.49177	35476.1876
Incidence	France	Male	55+ years	Non-melanoma skin cancer (basal-cell	Percent	2021	0.0006159	0.000789759	0.000475
Incidence	France	Female	55+ years	Non-melanoma skin cancer (basal-cell	Percent	2021	0.0003942	0.000491688	0.000309628
Incidence	France	Both	55+ years	Non-melanoma skin cancer (basal-cell	Percent	2021	0.0004895	0.000616162	0.000381652
Incidence	France	Male	55+ years	Non-melanoma skin cancer (basal-cell	Rate	2021	246.37725	314.6716539	189.4954923
Incidence	France	Female	55+ years	Non-melanoma skin cancer (basal-cell	Rate	2021	172.48917	216.5945637	138.169195
Incidence	France	Both	55+ years	Non-melanoma skin cancer (basal-cell	Rate	2021	205.89318	260.2133587	160.4522452
Incidence	New Zealan	Male	55+ years	Non-melanoma skin cancer (basal-cell	Number	1990	432.46964	559.4462612	331.6337649
Incidence	New Zealan	Female	55+ years	Non-melanoma skin cancer (basal-cell	Number	1990	410.73274	511.5713633	307.4206744
Incidence	New Zealan	Both	55+ years	Non-melanoma skin cancer (basal-cell	Number	1990	843.20238	1062.905362	638.3729317
Incidence	New Zealan	Male	55+ years	Non-melanoma skin cancer (basal-cell	Percent	1990	0.0003398	0.000447037	0.000250582
Incidence	New Zealan	Female	55+ years	Non-melanoma skin cancer (basal-cell	Percent	1990	0.0002521	0.00031723	0.000183466
Incidence	New Zealan	Both	55+ years	Non-melanoma skin cancer (basal-cell	Percent	1990	0.0002905	0.000372792	0.000216186
Incidence	New Zealan	Male	55+ years	Non-melanoma skin cancer (basal-cell	Rate	1990	143.81853	186.044832	110.2853882
Incidence	New Zealan	Female	55+ years	Non-melanoma skin cancer (basal-cell	Rate	1990	114.9646	143.1894487	86.04742181
Incidence	New Zealan	Both	55+ years	Non-melanoma skin cancer (basal-cell	Rate	1990	128.15133	161.5421582	97.02099997
Incidence	New Zealan	Male	55+ years	Non-melanoma skin cancer (basal-cell	Number	2021	1062.2311	1332.318064	824.9742231
Incidence	New Zealan	Female	55+ years	Non-melanoma skin cancer (basal-cell	Number	2021	808.83782	1007.252725	631.7077107
Incidence	New Zealan	Both	55+ years	Non-melanoma skin cancer (basal-cell	Number	2021	1871.0689	2329.565931	1460.600918
Incidence	New Zealan	Male	55+ years	Non-melanoma skin cancer (basal-cell	Percent	2021	0.0003666	0.000465623	0.000279923
Incidence	New Zealan	Female	55+ years	Non-melanoma skin cancer (basal-cell	Percent	2021	0.0002385	0.000299439	0.000178734
Incidence	New Zealan	Both	55+ years	Non-melanoma skin cancer (basal-cell	Percent	2021	0.0002975	0.000372526	0.000226406
Incidence	New Zealan	Male	55+ years	Non-melanoma skin cancer (basal-cell	Rate	2021	156.54748	196.3518403	121.5814836
Incidence	New Zealan	Female	55+ years	Non-melanoma skin cancer (basal-cell	Rate	2021	108.1498	134.6798759	84.46570948
Incidence	New Zealan	Both	55+ years	Non-melanoma skin cancer (basal-cell	Rate	2021	131.17213	163.3152641	102.3960823
Incidence	Greece	Male	55+ years	Non-melanoma skin cancer (basal-cell	Number	1990	2290.4043	2918.142975	1729.898736
Incidence	Greece	Female	55+ years	Non-melanoma skin cancer (basal-cell	Number	1990	1837.738	2369.850128	1383.005451
Incidence	Greece	Both	55+ years	Non-melanoma skin cancer (basal-cell	Number	1990	4128.1423	5277.179384	3140.048603
Incidence	Greece	Male	55+ years	Non-melanoma skin cancer (basal-cell	Percent	1990	0.0005299	0.000684418	0.000395942
Incidence	Greece	Female	55+ years	Non-melanoma skin cancer (basal-cell	Percent	1990	0.000329	0.00042293	0.000246263
Incidence	Greece	Both	55+ years	Non-melanoma skin cancer (basal-cell	Percent	1990	0.0004166	0.000532691	0.000311606
Incidence	Greece	Male	55+ years	Non-melanoma skin cancer (basal-cell	Rate	1990	185.19663	235.9540902	139.8754913
Incidence	Greece	Female	55+ years	Non-melanoma skin cancer (basal-cell	Rate	1990	127.89363	164.9248934	96.24744783
Incidence	Greece	Both	55+ years	Non-melanoma skin cancer (basal-cell	Rate	1990	154.39992	197.3759623	117.4434427
Incidence	Greece	Male	55+ years	Non-melanoma skin cancer (basal-cell	Number	2021	3763.0783	4769.131066	2937.627359
Incidence	Greece	Female	55+ years	Non-melanoma skin cancer (basal-cell	Number	2021	2989.2016	3741.000895	2343.849695
Incidence	Greece	Both	55+ years	Non-melanoma skin cancer (basal-cell	Number	2021	6752.2799	8536.030486	5337.629508
Incidence	Greece	Male	55+ years	Non-melanoma skin cancer (basal-cell	Percent	2021	0.0006035	0.000774268	0.000459939
Incidence	Greece	Female	55+ years	Non-melanoma skin cancer (basal-cell	Percent	2021	0.0003757	0.000477036	0.000286996
Incidence	Greece	Both	55+ years	Non-melanoma skin cancer (basal-cell	Percent	2021	0.0004758	0.000606613	0.000364966
Incidence	Greece	Male	55+ years	Non-melanoma skin cancer (basal-cell	Rate	2021	222.80541	282.3720718	173.9318782
Incidence	Greece	Female	55+ years	Non-melanoma skin cancer (basal-cell	Rate	2021	150.47603	188.3215171	117.9891005

Incidence	Greece	Both	55+ years	Non-melanoma skin cancer (basal-cell	Rate	2021	183.71303	232.2445249	145.2238522
Incidence	Cyprus	Male	55+ years	Non-melanoma skin cancer (basal-cell	Number	1990	78.443977	91.09390915	66.40700988
Incidence	Cyprus	Female	55+ years	Non-melanoma skin cancer (basal-cell	Number	1990	73.064659	85.8179846	61.39441397
Incidence	Cyprus	Both	55+ years	Non-melanoma skin cancer (basal-cell	Number	1990	151.50864	175.1043725	128.8322754
Incidence	Cyprus	Male	55+ years	Non-melanoma skin cancer (basal-cell	Percent	1990	0.0003388	0.000399897	0.000279241
Incidence	Cyprus	Female	55+ years	Non-melanoma skin cancer (basal-cell	Percent	1990	0.0002495	0.000300936	0.000204629
Incidence	Cyprus	Both	55+ years	Non-melanoma skin cancer (basal-cell	Percent	1990	0.0002889	0.000341641	0.000238889
Incidence	Cyprus	Male	55+ years	Non-melanoma skin cancer (basal-cell	Rate	1990	121.40252	140.9799776	102.7737074
Incidence	Cyprus	Female	55+ years	Non-melanoma skin cancer (basal-cell	Rate	1990	97.322224	114.3096705	81.77744168
Incidence	Cyprus	Both	55+ years	Non-melanoma skin cancer (basal-cell	Rate	1990	108.46078	125.3523107	92.2274138
Incidence	Cyprus	Male	55+ years	Non-melanoma skin cancer (basal-cell	Number	2021	236.26184	308.1049126	177.2563722
Incidence	Cyprus	Female	55+ years	Non-melanoma skin cancer (basal-cell	Number	2021	182.10648	233.0834741	138.8180504
Incidence	Cyprus	Both	55+ years	Non-melanoma skin cancer (basal-cell	Number	2021	418.36832	540.3356751	318.4240025
Incidence	Cyprus	Male	55+ years	Non-melanoma skin cancer (basal-cell	Percent	2021	0.0003583	0.000468545	0.00026585
Incidence	Cyprus	Female	55+ years	Non-melanoma skin cancer (basal-cell	Percent	2021	0.0002334	0.000295669	0.000177186
Incidence	Cyprus	Both	55+ years	Non-melanoma skin cancer (basal-cell	Percent	2021	0.0002906	0.000371835	0.00022011
Incidence	Cyprus	Male	55+ years	Non-melanoma skin cancer (basal-cell	Rate	2021	140.31986	182.9886609	105.2755242
Incidence	Cyprus	Female	55+ years	Non-melanoma skin cancer (basal-cell	Rate	2021	100.54028	128.6844798	76.64090591
Incidence	Cyprus	Both	55+ years	Non-melanoma skin cancer (basal-cell	Rate	2021	119.70424	154.6017496	91.10800962
Incidence	Ireland	Male	55+ years	Non-melanoma skin cancer (basal-cell	Number	1990	1374.7023	1535.322006	1190.02794
Incidence	Ireland	Female	55+ years	Non-melanoma skin cancer (basal-cell	Number	1990	1243.2609	1388.129651	1094.612663
Incidence	Ireland	Both	55+ years	Non-melanoma skin cancer (basal-cell	Number	1990	2617.9632	2896.310269	2329.456766
Incidence	Ireland	Male	55+ years	Non-melanoma skin cancer (basal-cell	Percent	1990	0.001254	0.001453065	0.001073343
Incidence	Ireland	Female	55+ years	Non-melanoma skin cancer (basal-cell	Percent	1990	0.000879	0.001001141	0.000765766
Incidence	Ireland	Both	55+ years	Non-melanoma skin cancer (basal-cell	Percent	1990	0.0010427	0.001181774	0.000911986
Incidence	Ireland	Male	55+ years	Non-melanoma skin cancer (basal-cell	Rate	1990	440.59945	492.0789141	381.4103194
Incidence	Ireland	Female	55+ years	Non-melanoma skin cancer (basal-cell	Rate	1990	337.18595	376.4759535	296.870934
Incidence	Ireland	Both	55+ years	Non-melanoma skin cancer (basal-cell	Rate	1990	384.58514	425.4750015	342.2028474
Incidence	Ireland	Male	55+ years	Non-melanoma skin cancer (basal-cell	Number	2021	1765.2728	2184.618828	1383.742109
Incidence	Ireland	Female	55+ years	Non-melanoma skin cancer (basal-cell	Number	2021	1301.9667	1623.380771	1003.630148
Incidence	Ireland	Both	55+ years	Non-melanoma skin cancer (basal-cell	Number	2021	3067.2394	3791.356812	2399.934874
Incidence	Ireland	Male	55+ years	Non-melanoma skin cancer (basal-cell	Percent	2021	0.0007389	0.000918351	0.000559918
Incidence	Ireland	Female	55+ years	Non-melanoma skin cancer (basal-cell	Percent	2021	0.0004601	0.000569302	0.000347757
Incidence	Ireland	Both	55+ years	Non-melanoma skin cancer (basal-cell	Percent	2021	0.0005877	0.000728989	0.000443973
Incidence	Ireland	Male	55+ years	Non-melanoma skin cancer (basal-cell	Rate	2021	279.8557	346.3362912	219.3701272
Incidence	Ireland	Female	55+ years	Non-melanoma skin cancer (basal-cell	Rate	2021	191.01941	238.1760192	147.248654
Incidence	Ireland	Both	55+ years	Non-melanoma skin cancer (basal-cell	Rate	2021	233.71788	288.8942653	182.870528
Incidence	Italy	Male	55+ years	Non-melanoma skin cancer (basal-cell	Number	1990	15162.52	17501.66275	12809.19662
Incidence	Italy	Female	55+ years	Non-melanoma skin cancer (basal-cell	Number	1990	11924.553	13830.60283	10068.47448
Incidence	Italy	Both	55+ years	Non-melanoma skin cancer (basal-cell	Number	1990	27087.073	31404.96584	23010.31949
Incidence	Italy	Male	55+ years	Non-melanoma skin cancer (basal-cell	Percent	1990	0.0006218	0.000728773	0.000510346
Incidence	Italy	Female	55+ years	Non-melanoma skin cancer (basal-cell	Percent	1990	0.0003479	0.000404999	0.000284117
Incidence	Italy	Both	55+ years	Non-melanoma skin cancer (basal-cell	Percent	1990	0.0004617	0.00053911	0.000376896
Incidence	Italy	Male	55+ years	Non-melanoma skin cancer (basal-cell	Rate	1990	228.62305	263.8930414	193.1392407
Incidence	Italy	Female	55+ years	Non-melanoma skin cancer (basal-cell	Rate	1990	138.86778	161.0647556	117.2527619
Incidence	Italy	Both	55+ years	Non-melanoma skin cancer (basal-cell	Rate	1990	177.98092	206.3524894	151.1938186
Incidence	Italy	Male	55+ years	Non-melanoma skin cancer (basal-cell	Number	2021	26964.301	34324.34853	20889.05345
Incidence	Italy	Female	55+ years	Non-melanoma skin cancer (basal-cell	Number	2021	21805.704	27114.26129	17175.12162
Incidence	Italy	Both	55+ years	Non-melanoma skin cancer (basal-cell	Number	2021	48770.005	61303.30681	38021.40741
Incidence	Italy	Male	55+ years	Non-melanoma skin cancer (basal-cell	Percent	2021	0.0006877	0.000882455	0.000525783
Incidence	Italy	Female	55+ years	Non-melanoma skin cancer (basal-cell	Percent	2021	0.0004383	0.00054844	0.000336392
Incidence	Italy	Both	55+ years	Non-melanoma skin cancer (basal-cell	Percent	2021	0.0005482	0.00069454	0.000415602
Incidence	Italy	Male	55+ years	Non-melanoma skin cancer (basal-cell	Rate	2021	260.93573	332.1595099	202.1450678
Incidence	Italy	Female	55+ years	Non-melanoma skin cancer (basal-cell	Rate	2021	176.09245	218.9618253	138.6980799
Incidence	Italy	Both	55+ years	Non-melanoma skin cancer (basal-cell	Rate	2021	214.68701	269.8589707	167.3713606
Incidence	Norway	Male	55+ years	Non-melanoma skin cancer (basal-cell	Number	1990	1023.8511	1296.057689	789.6767782

Incidence	Norway	Female	55+ years	Non-melanoma skin cancer (basal-cell	Number	1990	954.66208	1207.524804	746.2856636
Incidence	Norway	Both	55+ years	Non-melanoma skin cancer (basal-cell	Number	1990	1978.5132	2498.199763	1527.995761
Incidence	Norway	Male	55+ years	Non-melanoma skin cancer (basal-cell	Percent	1990	0.0005468	0.00070363	0.000413061
Incidence	Norway	Female	55+ years	Non-melanoma skin cancer (basal-cell	Percent	1990	0.0003738	0.000473156	0.000288262
Incidence	Norway	Both	55+ years	Non-melanoma skin cancer (basal-cell	Percent	1990	0.000447	0.000570594	0.000342156
Incidence	Norway	Male	55+ years	Non-melanoma skin cancer (basal-cell	Rate	1990	213.78021	270.6169593	164.8845806
Incidence	Norway	Female	55+ years	Non-melanoma skin cancer (basal-cell	Rate	1990	158.05761	199.9225589	123.5579915
Incidence	Norway	Both	55+ years	Non-melanoma skin cancer (basal-cell	Rate	1990	182.70114	230.6903751	141.0991708
Incidence	Norway	Male	55+ years	Non-melanoma skin cancer (basal-cell	Number	2021	1658.7253	2097.971038	1283.735852
Incidence	Norway	Female	55+ years	Non-melanoma skin cancer (basal-cell	Number	2021	1282.6751	1583.890443	1001.327915
Incidence	Norway	Both	55+ years	Non-melanoma skin cancer (basal-cell	Number	2021	2941.4004	3664.903477	2281.029095
Incidence	Norway	Male	55+ years	Non-melanoma skin cancer (basal-cell	Percent	2021	0.0004862	0.000613256	0.00036858
Incidence	Norway	Female	55+ years	Non-melanoma skin cancer (basal-cell	Percent	2021	0.0003128	0.000385854	0.000239309
Incidence	Norway	Both	55+ years	Non-melanoma skin cancer (basal-cell	Percent	2021	0.0003915	0.000490041	0.000302467
Incidence	Norway	Male	55+ years	Non-melanoma skin cancer (basal-cell	Rate	2021	211.62358	267.6634516	163.7816552
Incidence	Norway	Female	55+ years	Non-melanoma skin cancer (basal-cell	Rate	2021	153.07739	189.0251256	119.5007747
Incidence	Norway	Both	55+ years	Non-melanoma skin cancer (basal-cell	Rate	2021	181.37366	225.9865554	140.653611
Incidence	United Kin	Male	55+ years	Non-melanoma skin cancer (basal-cell	Number	1990	15959.759	19962.65917	12546.19297
Incidence	United Kin	Female	55+ years	Non-melanoma skin cancer (basal-cell	Number	1990	15439.65	19197.88446	12317.81853
Incidence	United Kin	Both	55+ years	Non-melanoma skin cancer (basal-cell	Number	1990	31399.409	39173.77053	24936.20456
Incidence	United Kin	Male	55+ years	Non-melanoma skin cancer (basal-cell	Percent	1990	0.0006883	0.000863296	0.000526381
Incidence	United Kin	Female	55+ years	Non-melanoma skin cancer (basal-cell	Percent	1990	0.0004636	0.000576621	0.000360239
Incidence	United Kin	Both	55+ years	Non-melanoma skin cancer (basal-cell	Percent	1990	0.0005558	0.00069576	0.000432984
Incidence	United Kin	Male	55+ years	Non-melanoma skin cancer (basal-cell	Rate	1990	247.4139	309.4682994	194.4955815
Incidence	United Kin	Female	55+ years	Non-melanoma skin cancer (basal-cell	Rate	1990	183.75262	228.4806645	146.5986197
Incidence	United Kin	Both	55+ years	Non-melanoma skin cancer (basal-cell	Rate	1990	211.40052	263.7424012	167.8861743
Incidence	United Kin	Male	55+ years	Non-melanoma skin cancer (basal-cell	Number	2021	23990.962	30019.282	18489.61519
Incidence	United Kin	Female	55+ years	Non-melanoma skin cancer (basal-cell	Number	2021	19716.337	24361.97728	15553.94152
Incidence	United Kin	Both	55+ years	Non-melanoma skin cancer (basal-cell	Number	2021	43707.299	54417.60803	34167.83084
Incidence	United Kin	Male	55+ years	Non-melanoma skin cancer (basal-cell	Percent	2021	0.0006267	0.000789419	0.00047993
Incidence	United Kin	Female	55+ years	Non-melanoma skin cancer (basal-cell	Percent	2021	0.000433	0.000541168	0.000332363
Incidence	United Kin	Both	55+ years	Non-melanoma skin cancer (basal-cell	Percent	2021	0.0005215	0.000654938	0.000402762
Incidence	United Kin	Male	55+ years	Non-melanoma skin cancer (basal-cell	Rate	2021	240.78638	301.2898834	185.5718602
Incidence	United Kin	Female	55+ years	Non-melanoma skin cancer (basal-cell	Rate	2021	178.16077	220.1397051	140.5485302
Incidence	United Kin	Both	55+ years	Non-melanoma skin cancer (basal-cell	Rate	2021	207.83125	258.7595152	162.4704147
Incidence	Germany	Male	55+ years	Non-melanoma skin cancer (basal-cell	Number	1990	9601.0298	10804.512	8486.263382
Incidence	Germany	Female	55+ years	Non-melanoma skin cancer (basal-cell	Number	1990	10413.724	11977.01668	8931.748387
Incidence	Germany	Both	55+ years	Non-melanoma skin cancer (basal-cell	Number	1990	20014.754	22560.94985	17516.82084
Incidence	Germany	Male	55+ years	Non-melanoma skin cancer (basal-cell	Percent	1990	0.0003224	0.000371306	0.000274458
Incidence	Germany	Female	55+ years	Non-melanoma skin cancer (basal-cell	Percent	1990	0.0002104	0.000244226	0.000176974
Incidence	Germany	Both	55+ years	Non-melanoma skin cancer (basal-cell	Percent	1990	0.0002524	0.000288659	0.000215708
Incidence	Germany	Male	55+ years	Non-melanoma skin cancer (basal-cell	Rate	1990	114.24499	128.5655158	100.9801117
Incidence	Germany	Female	55+ years	Non-melanoma skin cancer (basal-cell	Rate	1990	82.784615	95.21211527	71.00354617
Incidence	Germany	Both	55+ years	Non-melanoma skin cancer (basal-cell	Rate	1990	95.384684	107.5191363	83.4802373
Incidence	Germany	Male	55+ years	Non-melanoma skin cancer (basal-cell	Number	2021	25718.234	33186.39924	19859.75993
Incidence	Germany	Female	55+ years	Non-melanoma skin cancer (basal-cell	Number	2021	20186.228	25187.98573	15780.41569
Incidence	Germany	Both	55+ years	Non-melanoma skin cancer (basal-cell	Number	2021	45904.462	57427.01245	35776.20045
Incidence	Germany	Male	55+ years	Non-melanoma skin cancer (basal-cell	Percent	2021	0.0004544	0.000586773	0.000345367
Incidence	Germany	Female	55+ years	Non-melanoma skin cancer (basal-cell	Percent	2021	0.0002781	0.000344831	0.000214502
Incidence	Germany	Both	55+ years	Non-melanoma skin cancer (basal-cell	Percent	2021	0.0003553	0.000453463	0.000272425
Incidence	Germany	Male	55+ years	Non-melanoma skin cancer (basal-cell	Rate	2021	175.67112	226.6832098	135.6541906
Incidence	Germany	Female	55+ years	Non-melanoma skin cancer (basal-cell	Rate	2021	119.62981	149.2717634	93.5196051
Incidence	Germany	Both	55+ years	Non-melanoma skin cancer (basal-cell	Rate	2021	145.66417	182.227554	113.5251378
Incidence	Andorra	Male	55+ years	Non-melanoma skin cancer (basal-cell	Number	1990	9.1549077	11.72393249	7.008150118
Incidence	Andorra	Female	55+ years	Non-melanoma skin cancer (basal-cell	Number	1990	5.7825173	7.388561039	4.393679486
Incidence	Andorra	Both	55+ years	Non-melanoma skin cancer (basal-cell	Number	1990	14.937425	19.10638248	11.49101914

Incidence	Andorra	Male	55+ years	Non-melanoma skin cancer (basal-cell	Percent	1990	0.0005179	0.000670813	0.000378755
Incidence	Andorra	Female	55+ years	Non-melanoma skin cancer (basal-cell	Percent	1990	0.0003149	0.000404012	0.000231274
Incidence	Andorra	Both	55+ years	Non-melanoma skin cancer (basal-cell	Percent	1990	0.0004144	0.000532889	0.000302672
Incidence	Andorra	Male	55+ years	Non-melanoma skin cancer (basal-cell	Rate	1990	179.70104	230.128243	137.5624838
Incidence	Andorra	Female	55+ years	Non-melanoma skin cancer (basal-cell	Rate	1990	121.15253	154.8015922	92.05426825
Incidence	Andorra	Both	55+ years	Non-melanoma skin cancer (basal-cell	Rate	1990	151.38089	193.6305033	116.4538511
Incidence	Andorra	Male	55+ years	Non-melanoma skin cancer (basal-cell	Number	2021	25.548937	32.61333684	19.49723242
Incidence	Andorra	Female	55+ years	Non-melanoma skin cancer (basal-cell	Number	2021	16.852832	20.97914676	13.08272967
Incidence	Andorra	Both	55+ years	Non-melanoma skin cancer (basal-cell	Number	2021	42.401769	53.01961921	32.50394045
Incidence	Andorra	Male	55+ years	Non-melanoma skin cancer (basal-cell	Percent	2021	0.0004966	0.0006391	0.000372377
Incidence	Andorra	Female	55+ years	Non-melanoma skin cancer (basal-cell	Percent	2021	0.000306	0.000384821	0.000227735
Incidence	Andorra	Both	55+ years	Non-melanoma skin cancer (basal-cell	Percent	2021	0.0003981	0.000506302	0.000300246
Incidence	Andorra	Male	55+ years	Non-melanoma skin cancer (basal-cell	Rate	2021	188.40989	240.5061058	143.7817745
Incidence	Andorra	Female	55+ years	Non-melanoma skin cancer (basal-cell	Rate	2021	130.62725	162.6105483	101.4049747
Incidence	Andorra	Both	55+ years	Non-melanoma skin cancer (basal-cell	Rate	2021	160.23789	200.3631468	122.8336206
Incidence	Netherland	Male	55+ years	Non-melanoma skin cancer (basal-cell	Number	1990	1353.5124	1694.588258	1075.074222
Incidence	Netherland	Female	55+ years	Non-melanoma skin cancer (basal-cell	Number	1990	1618.1473	2003.973782	1254.55953
Incidence	Netherland	Both	55+ years	Non-melanoma skin cancer (basal-cell	Number	1990	2971.6597	3643.013729	2361.018126
Incidence	Netherland	Male	55+ years	Non-melanoma skin cancer (basal-cell	Percent	1990	0.0002707	0.000341314	0.000211902
Incidence	Netherland	Female	55+ years	Non-melanoma skin cancer (basal-cell	Percent	1990	0.0002282	0.000284608	0.00017709
Incidence	Netherland	Both	55+ years	Non-melanoma skin cancer (basal-cell	Percent	1990	0.0002458	0.000303809	0.000190761
Incidence	Netherland	Male	55+ years	Non-melanoma skin cancer (basal-cell	Rate	1990	93.896599	117.5579014	74.58063559
Incidence	Netherland	Female	55+ years	Non-melanoma skin cancer (basal-cell	Rate	1990	86.55619	107.194406	67.10754644
Incidence	Netherland	Both	55+ years	Non-melanoma skin cancer (basal-cell	Rate	1990	89.751976	110.0286429	71.30898744
Incidence	Netherland	Male	55+ years	Non-melanoma skin cancer (basal-cell	Number	2021	3872.8535	4902.843102	2992.340745
Incidence	Netherland	Female	55+ years	Non-melanoma skin cancer (basal-cell	Number	2021	3142.3687	3915.118487	2426.95706
Incidence	Netherland	Both	55+ years	Non-melanoma skin cancer (basal-cell	Number	2021	7015.2223	8828.993167	5429.040482
Incidence	Netherland	Male	55+ years	Non-melanoma skin cancer (basal-cell	Percent	2021	0.0003594	0.000456789	0.000272276
Incidence	Netherland	Female	55+ years	Non-melanoma skin cancer (basal-cell	Percent	2021	0.0002388	0.000295751	0.000183967
Incidence	Netherland	Both	55+ years	Non-melanoma skin cancer (basal-cell	Percent	2021	0.0002931	0.000368465	0.000223562
Incidence	Netherland	Male	55+ years	Non-melanoma skin cancer (basal-cell	Rate	2021	138.41132	175.2219595	106.9428081
Incidence	Netherland	Female	55+ years	Non-melanoma skin cancer (basal-cell	Rate	2021	102.93408	128.2469227	79.49945206
Incidence	Netherland	Both	55+ years	Non-melanoma skin cancer (basal-cell	Rate	2021	119.90044	150.9004428	92.79026467
Incidence	Portugal	Male	55+ years	Non-melanoma skin cancer (basal-cell	Number	1990	698.03506	881.4611372	594.1633027
Incidence	Portugal	Female	55+ years	Non-melanoma skin cancer (basal-cell	Number	1990	883.6887	1059.741328	713.8548017
Incidence	Portugal	Both	55+ years	Non-melanoma skin cancer (basal-cell	Number	1990	1581.7238	1926.614581	1323.574656
Incidence	Portugal	Male	55+ years	Non-melanoma skin cancer (basal-cell	Percent	1990	0.0001919	0.000240959	0.00015984
Incidence	Portugal	Female	55+ years	Non-melanoma skin cancer (basal-cell	Percent	1990	0.0001717	0.000206681	0.000136211
Incidence	Portugal	Both	55+ years	Non-melanoma skin cancer (basal-cell	Percent	1990	0.00018	0.000217865	0.00015006
Incidence	Portugal	Male	55+ years	Non-melanoma skin cancer (basal-cell	Rate	1990	65.430856	82.62444174	55.69435693
Incidence	Portugal	Female	55+ years	Non-melanoma skin cancer (basal-cell	Rate	1990	64.449223	77.28910008	52.06288906
Incidence	Portugal	Both	55+ years	Non-melanoma skin cancer (basal-cell	Rate	1990	64.878775	79.02542573	54.29007529
Incidence	Portugal	Male	55+ years	Non-melanoma skin cancer (basal-cell	Number	2021	2232.8886	2770.571058	1725.80232
Incidence	Portugal	Female	55+ years	Non-melanoma skin cancer (basal-cell	Number	2021	1983.8376	2493.465667	1538.290358
Incidence	Portugal	Both	55+ years	Non-melanoma skin cancer (basal-cell	Number	2021	4216.7262	5230.971935	3273.915032
Incidence	Portugal	Male	55+ years	Non-melanoma skin cancer (basal-cell	Percent	2021	0.0003514	0.000445101	0.000264323
Incidence	Portugal	Female	55+ years	Non-melanoma skin cancer (basal-cell	Percent	2021	0.0002251	0.000285115	0.000170676
Incidence	Portugal	Both	55+ years	Non-melanoma skin cancer (basal-cell	Percent	2021	0.000278	0.000349635	0.000209882
Incidence	Portugal	Male	55+ years	Non-melanoma skin cancer (basal-cell	Rate	2021	129.75007	160.9940454	100.2839817
Incidence	Portugal	Female	55+ years	Non-melanoma skin cancer (basal-cell	Rate	2021	91.432529	114.9206331	70.89782878
Incidence	Portugal	Both	55+ years	Non-melanoma skin cancer (basal-cell	Rate	2021	108.3812	134.4500387	84.14841603
Incidence	Spain	Male	55+ years	Non-melanoma skin cancer (basal-cell	Number	1990	11493.225	12142.91605	10836.0317
Incidence	Spain	Female	55+ years	Non-melanoma skin cancer (basal-cell	Number	1990	9553.5013	10226.47879	8882.231133
Incidence	Spain	Both	55+ years	Non-melanoma skin cancer (basal-cell	Number	1990	21046.727	22156.57183	19907.74791
Incidence	Spain	Male	55+ years	Non-melanoma skin cancer (basal-cell	Percent	1990	0.0007904	0.000866723	0.000712804
Incidence	Spain	Female	55+ years	Non-melanoma skin cancer (basal-cell	Percent	1990	0.0004776	0.000523193	0.000429918

Incidence	Spain	Both	55+ years	Non-melanoma skin cancer (basal-cell	Percent	1990	0.0006092	0.00066201	0.000552231
Incidence	Spain	Male	55+ years	Non-melanoma skin cancer (basal-cell	Rate	1990	272.05088	287.4294151	256.494753
Incidence	Spain	Female	55+ years	Non-melanoma skin cancer (basal-cell	Rate	1990	179.04069	191.6528587	166.4605211
Incidence	Spain	Both	55+ years	Non-melanoma skin cancer (basal-cell	Rate	1990	220.14026	231.7487893	208.2269996
Incidence	Spain	Male	55+ years	Non-melanoma skin cancer (basal-cell	Number	2021	16369.783	20630.35659	12635.27325
Incidence	Spain	Female	55+ years	Non-melanoma skin cancer (basal-cell	Number	2021	13555.845	16834.42578	10645.75939
Incidence	Spain	Both	55+ years	Non-melanoma skin cancer (basal-cell	Number	2021	29925.628	37425.17695	23287.12184
Incidence	Spain	Male	55+ years	Non-melanoma skin cancer (basal-cell	Percent	2021	0.0006172	0.000786066	0.000471157
Incidence	Spain	Female	55+ years	Non-melanoma skin cancer (basal-cell	Percent	2021	0.0003901	0.000487796	0.000302637
Incidence	Spain	Both	55+ years	Non-melanoma skin cancer (basal-cell	Percent	2021	0.0004883	0.000615642	0.000373601
Incidence	Spain	Male	55+ years	Non-melanoma skin cancer (basal-cell	Rate	2021	232.45958	292.9619855	179.4275695
Incidence	Spain	Female	55+ years	Non-melanoma skin cancer (basal-cell	Rate	2021	160.74594	199.6235208	126.2379839
Incidence	Spain	Both	55+ years	Non-melanoma skin cancer (basal-cell	Rate	2021	193.37949	241.8416003	150.4814478
Incidence	Dominica	Male	55+ years	Non-melanoma skin cancer (basal-cell	Number	1990	0.9876706	1.293653039	0.729364013
Incidence	Dominica	Female	55+ years	Non-melanoma skin cancer (basal-cell	Number	1990	0.5571806	0.741101497	0.400940249
Incidence	Dominica	Both	55+ years	Non-melanoma skin cancer (basal-cell	Number	1990	1.5448512	2.011975468	1.127902253
Incidence	Dominica	Male	55+ years	Non-melanoma skin cancer (basal-cell	Percent	1990	6.90E-05	9.05E-05	5.01E-05
Incidence	Dominica	Female	55+ years	Non-melanoma skin cancer (basal-cell	Percent	1990	2.29E-05	3.05E-05	1.61E-05
Incidence	Dominica	Both	55+ years	Non-melanoma skin cancer (basal-cell	Percent	1990	4.00E-05	5.25E-05	2.88E-05
Incidence	Dominica	Male	55+ years	Non-melanoma skin cancer (basal-cell	Rate	1990	26.238612	34.36738993	19.37639898
Incidence	Dominica	Female	55+ years	Non-melanoma skin cancer (basal-cell	Rate	1990	9.2041073	12.24231031	6.623161564
Incidence	Dominica	Both	55+ years	Non-melanoma skin cancer (basal-cell	Rate	1990	15.735214	20.49314846	11.48834501
Incidence	Dominica	Male	55+ years	Non-melanoma skin cancer (basal-cell	Number	2021	1.7043136	2.211611987	1.217065429
Incidence	Dominica	Female	55+ years	Non-melanoma skin cancer (basal-cell	Number	2021	0.6920951	0.937401209	0.50489929
Incidence	Dominica	Both	55+ years	Non-melanoma skin cancer (basal-cell	Number	2021	2.3964087	3.105229106	1.760586724
Incidence	Dominica	Male	55+ years	Non-melanoma skin cancer (basal-cell	Percent	2021	5.88E-05	7.77E-05	4.21E-05
Incidence	Dominica	Female	55+ years	Non-melanoma skin cancer (basal-cell	Percent	2021	2.15E-05	2.85E-05	1.54E-05
Incidence	Dominica	Both	55+ years	Non-melanoma skin cancer (basal-cell	Percent	2021	3.91E-05	5.14E-05	2.80E-05
Incidence	Dominica	Male	55+ years	Non-melanoma skin cancer (basal-cell	Rate	2021	22.881802	29.69269742	16.34009752
Incidence	Dominica	Female	55+ years	Non-melanoma skin cancer (basal-cell	Rate	2021	9.0654031	12.27854356	6.613420018
Incidence	Dominica	Both	55+ years	Non-melanoma skin cancer (basal-cell	Rate	2021	15.888352	20.58787927	11.67280921
Incidence	Switzerlan	Male	55+ years	Non-melanoma skin cancer (basal-cell	Number	1990	3001.5547	3131.474171	2856.515051
Incidence	Switzerlan	Female	55+ years	Non-melanoma skin cancer (basal-cell	Number	1990	2917.9936	3058.997027	2747.235165
Incidence	Switzerlan	Both	55+ years	Non-melanoma skin cancer (basal-cell	Number	1990	5919.5484	6159.335651	5661.216062
Incidence	Switzerlan	Male	55+ years	Non-melanoma skin cancer (basal-cell	Percent	1990	0.001129	0.001225313	0.00103371
Incidence	Switzerlan	Female	55+ years	Non-melanoma skin cancer (basal-cell	Percent	1990	0.0007454	0.000811737	0.00067989
Incidence	Switzerlan	Both	55+ years	Non-melanoma skin cancer (basal-cell	Percent	1990	0.0009005	0.00097179	0.000828586
Incidence	Switzerlan	Male	55+ years	Non-melanoma skin cancer (basal-cell	Rate	1990	412.73085	430.5955114	392.7870682
Incidence	Switzerlan	Female	55+ years	Non-melanoma skin cancer (basal-cell	Rate	1990	303.34981	318.0082928	285.5980431
Incidence	Switzerlan	Both	55+ years	Non-melanoma skin cancer (basal-cell	Rate	1990	350.44201	364.6376099	335.1485308
Incidence	Switzerlan	Male	55+ years	Non-melanoma skin cancer (basal-cell	Number	2021	3749.6839	4712.04762	2885.682023
Incidence	Switzerlan	Female	55+ years	Non-melanoma skin cancer (basal-cell	Number	2021	2996.2905	3769.999023	2366.005069
Incidence	Switzerlan	Both	55+ years	Non-melanoma skin cancer (basal-cell	Number	2021	6745.9744	8450.83449	5252.362994
Incidence	Switzerlan	Male	55+ years	Non-melanoma skin cancer (basal-cell	Percent	2021	0.0006932	0.000876184	0.000522224
Incidence	Switzerlan	Female	55+ years	Non-melanoma skin cancer (basal-cell	Percent	2021	0.000446	0.000559868	0.000345929
Incidence	Switzerlan	Both	55+ years	Non-melanoma skin cancer (basal-cell	Percent	2021	0.0005562	0.000693923	0.000426816
Incidence	Switzerlan	Male	55+ years	Non-melanoma skin cancer (basal-cell	Rate	2021	270.39035	339.7865567	208.0870223
Incidence	Switzerlan	Female	55+ years	Non-melanoma skin cancer (basal-cell	Rate	2021	194.05173	244.1601823	153.2319306
Incidence	Switzerlan	Both	55+ years	Non-melanoma skin cancer (basal-cell	Rate	2021	230.17245	288.3422279	179.2104732
Incidence	Sweden	Male	55+ years	Non-melanoma skin cancer (basal-cell	Number	1990	2245.4856	2923.091079	1706.66198
Incidence	Sweden	Female	55+ years	Non-melanoma skin cancer (basal-cell	Number	1990	2148.3183	2764.696938	1638.531139
Incidence	Sweden	Both	55+ years	Non-melanoma skin cancer (basal-cell	Number	1990	4393.8039	5671.489723	3355.706558
Incidence	Sweden	Male	55+ years	Non-melanoma skin cancer (basal-cell	Percent	1990	0.0005819	0.000757942	0.00044086
Incidence	Sweden	Female	55+ years	Non-melanoma skin cancer (basal-cell	Percent	1990	0.0004081	0.000529938	0.000307683
Incidence	Sweden	Both	55+ years	Non-melanoma skin cancer (basal-cell	Percent	1990	0.0004816	0.000622154	0.00036234
Incidence	Sweden	Male	55+ years	Non-melanoma skin cancer (basal-cell	Rate	1990	211.66192	275.533757	160.8718218

Incidence	Sweden	Female	55+ years	Non-melanoma skin cancer (basal-cell	Rate	1990	163.59528	210.5327523	124.7747866
Incidence	Sweden	Both	55+ years	Non-melanoma skin cancer (basal-cell	Rate	1990	185.07443	238.8927101	141.3480184
Incidence	Sweden	Male	55+ years	Non-melanoma skin cancer (basal-cell	Number	2021	4816.1051	6102.898297	3776.960423
Incidence	Sweden	Female	55+ years	Non-melanoma skin cancer (basal-cell	Number	2021	4320.6072	5308.565755	3516.193158
Incidence	Sweden	Both	55+ years	Non-melanoma skin cancer (basal-cell	Number	2021	9136.7122	11211.6299	7384.068705
Incidence	Sweden	Male	55+ years	Non-melanoma skin cancer (basal-cell	Percent	2021	0.000697	0.00088262	0.000541561
Incidence	Sweden	Female	55+ years	Non-melanoma skin cancer (basal-cell	Percent	2021	0.0005318	0.000660475	0.000430006
Incidence	Sweden	Both	55+ years	Non-melanoma skin cancer (basal-cell	Percent	2021	0.0006077	0.000754857	0.000483058
Incidence	Sweden	Male	55+ years	Non-melanoma skin cancer (basal-cell	Rate	2021	297.27634	376.704251	233.1346481
Incidence	Sweden	Female	55+ years	Non-melanoma skin cancer (basal-cell	Rate	2021	247.40157	303.9729055	201.3401547
Incidence	Sweden	Both	55+ years	Non-melanoma skin cancer (basal-cell	Rate	2021	271.40325	333.038044	219.3415071
Incidence	Malta	Male	55+ years	Non-melanoma skin cancer (basal-cell	Number	1990	91.183295	100.022845	83.36694673
Incidence	Malta	Female	55+ years	Non-melanoma skin cancer (basal-cell	Number	1990	55.748585	62.3013063	49.59044962
Incidence	Malta	Both	55+ years	Non-melanoma skin cancer (basal-cell	Number	1990	146.93188	161.13378	134.3078805
Incidence	Malta	Male	55+ years	Non-melanoma skin cancer (basal-cell	Percent	1990	0.0008239	0.000937879	0.000717412
Incidence	Malta	Female	55+ years	Non-melanoma skin cancer (basal-cell	Percent	1990	0.0003638	0.000413783	0.000314395
Incidence	Malta	Both	55+ years	Non-melanoma skin cancer (basal-cell	Percent	1990	0.0005567	0.000621882	0.000488495
Incidence	Malta	Male	55+ years	Non-melanoma skin cancer (basal-cell	Rate	1990	283.68632	311.1876284	259.3683717
Incidence	Malta	Female	55+ years	Non-melanoma skin cancer (basal-cell	Rate	1990	138.32145	154.5798306	123.0420959
Incidence	Malta	Both	55+ years	Non-melanoma skin cancer (basal-cell	Rate	1990	202.81589	222.4193381	185.3904866
Incidence	Malta	Male	55+ years	Non-melanoma skin cancer (basal-cell	Number	2021	178.23111	229.3670303	138.0757656
Incidence	Malta	Female	55+ years	Non-melanoma skin cancer (basal-cell	Number	2021	125.24383	161.4733084	96.90956477
Incidence	Malta	Both	55+ years	Non-melanoma skin cancer (basal-cell	Number	2021	303.47494	390.9134023	233.4633871
Incidence	Malta	Male	55+ years	Non-melanoma skin cancer (basal-cell	Percent	2021	0.0006575	0.000844185	0.00050044
Incidence	Malta	Female	55+ years	Non-melanoma skin cancer (basal-cell	Percent	2021	0.0003784	0.000486581	0.000290967
Incidence	Malta	Both	55+ years	Non-melanoma skin cancer (basal-cell	Percent	2021	0.0005041	0.000643835	0.000382215
Incidence	Malta	Male	55+ years	Non-melanoma skin cancer (basal-cell	Rate	2021	238.64731	307.1171083	184.8802324
Incidence	Malta	Female	55+ years	Non-melanoma skin cancer (basal-cell	Rate	2021	150.6796	194.2669272	116.590931
Incidence	Malta	Both	55+ years	Non-melanoma skin cancer (basal-cell	Rate	2021	192.31229	247.7221041	147.9459163
Incidence	Chile	Male	55+ years	Non-melanoma skin cancer (basal-cell	Number	1990	684.80463	857.2819078	548.2613745
Incidence	Chile	Female	55+ years	Non-melanoma skin cancer (basal-cell	Number	1990	739.5679	908.1026822	590.1197915
Incidence	Chile	Both	55+ years	Non-melanoma skin cancer (basal-cell	Number	1990	1424.3725	1748.797853	1133.373523
Incidence	Chile	Male	55+ years	Non-melanoma skin cancer (basal-cell	Percent	1990	0.0002302	0.00029237	0.000177707
Incidence	Chile	Female	55+ years	Non-melanoma skin cancer (basal-cell	Percent	1990	0.000187	0.00023795	0.000145992
Incidence	Chile	Both	55+ years	Non-melanoma skin cancer (basal-cell	Percent	1990	0.0002055	0.000257431	0.000159226
Incidence	Chile	Male	55+ years	Non-melanoma skin cancer (basal-cell	Rate	1990	90.326668	113.0766566	72.31642545
Incidence	Chile	Female	55+ years	Non-melanoma skin cancer (basal-cell	Rate	1990	80.076574	98.32464639	63.89510896
Incidence	Chile	Both	55+ years	Non-melanoma skin cancer (basal-cell	Rate	1990	84.697461	103.9887637	67.39378781
Incidence	Chile	Male	55+ years	Non-melanoma skin cancer (basal-cell	Number	2021	2023.1267	2560.984496	1565.838573
Incidence	Chile	Female	55+ years	Non-melanoma skin cancer (basal-cell	Number	2021	2092.9905	2615.120817	1618.696455
Incidence	Chile	Both	55+ years	Non-melanoma skin cancer (basal-cell	Number	2021	4116.1172	5115.793689	3188.822983
Incidence	Chile	Male	55+ years	Non-melanoma skin cancer (basal-cell	Percent	2021	0.0002542	0.00032599	0.000192156
Incidence	Chile	Female	55+ years	Non-melanoma skin cancer (basal-cell	Percent	2021	0.0002032	0.000257711	0.000150835
Incidence	Chile	Both	55+ years	Non-melanoma skin cancer (basal-cell	Percent	2021	0.0002254	0.000286791	0.00016912
Incidence	Chile	Male	55+ years	Non-melanoma skin cancer (basal-cell	Rate	2021	99.625359	126.1112289	77.10699808
Incidence	Chile	Female	55+ years	Non-melanoma skin cancer (basal-cell	Rate	2021	85.534734	106.872757	66.15164845
Incidence	Chile	Both	55+ years	Non-melanoma skin cancer (basal-cell	Rate	2021	91.925165	114.2509194	71.21592065
Incidence	United Sta	Male	55+ years	Non-melanoma skin cancer (basal-cell	Number	1990	298265.99	365705.0619	231734.4295
Incidence	United Sta	Female	55+ years	Non-melanoma skin cancer (basal-cell	Number	1990	274264.02	337457.4298	210648.7998
Incidence	United Sta	Both	55+ years	Non-melanoma skin cancer (basal-cell	Number	1990	572530.01	705463.814	442327.6913
Incidence	United Sta	Male	55+ years	Non-melanoma skin cancer (basal-cell	Percent	1990	0.0029952	0.003744823	0.00226871
Incidence	United Sta	Female	55+ years	Non-melanoma skin cancer (basal-cell	Percent	1990	0.0020384	0.002531475	0.001543232
Incidence	United Sta	Both	55+ years	Non-melanoma skin cancer (basal-cell	Percent	1990	0.0024453	0.003029555	0.001856596
Incidence	United Sta	Male	55+ years	Non-melanoma skin cancer (basal-cell	Rate	1990	1318.6554	1616.808369	1024.514572
Incidence	United Sta	Female	55+ years	Non-melanoma skin cancer (basal-cell	Rate	1990	919.01444	1130.76536	705.8501157
Incidence	United Sta	Both	55+ years	Non-melanoma skin cancer (basal-cell	Rate	1990	1091.3186	1344.708139	843.1355867

Incidence	United Sta	Male	55+ years	Non-melanoma skin cancer (basal-cell	Number	2021	1557908.3	1726977.022	1393571.185
Incidence	United Sta	Female	55+ years	Non-melanoma skin cancer (basal-cell	Number	2021	1004813.8	1109459.545	906494.3665
Incidence	United Sta	Both	55+ years	Non-melanoma skin cancer (basal-cell	Number	2021	2562722.1	2842198.66	2301511.268
Incidence	United Sta	Male	55+ years	Non-melanoma skin cancer (basal-cell	Percent	2021	0.0076757	0.008776588	0.006614976
Incidence	United Sta	Female	55+ years	Non-melanoma skin cancer (basal-cell	Percent	2021	0.0040405	0.004575577	0.003496312
Incidence	United Sta	Both	55+ years	Non-melanoma skin cancer (basal-cell	Percent	2021	0.005674	0.006468326	0.004891168
Incidence	United Sta	Male	55+ years	Non-melanoma skin cancer (basal-cell	Rate	2021	3351.6746	3715.408016	2998.120697
Incidence	United Sta	Female	55+ years	Non-melanoma skin cancer (basal-cell	Rate	2021	1868.8553	2063.486215	1685.99084
Incidence	United Sta	Both	55+ years	Non-melanoma skin cancer (basal-cell	Rate	2021	2556.3884	2835.174254	2295.823155
Incidence	Antigua an	Male	55+ years	Non-melanoma skin cancer (basal-cell	Number	1990	0.9905861	1.251340942	0.739191189
Incidence	Antigua an	Female	55+ years	Non-melanoma skin cancer (basal-cell	Number	1990	0.4802587	0.625746477	0.351131658
Incidence	Antigua an	Both	55+ years	Non-melanoma skin cancer (basal-cell	Number	1990	1.4708448	1.874153402	1.094464908
Incidence	Antigua an	Male	55+ years	Non-melanoma skin cancer (basal-cell	Percent	1990	6.56E-05	8.38E-05	4.80E-05
Incidence	Antigua an	Female	55+ years	Non-melanoma skin cancer (basal-cell	Percent	1990	2.37E-05	3.11E-05	1.75E-05
Incidence	Antigua an	Both	55+ years	Non-melanoma skin cancer (basal-cell	Percent	1990	4.16E-05	5.38E-05	3.05E-05
Incidence	Antigua an	Male	55+ years	Non-melanoma skin cancer (basal-cell	Rate	1990	26.214334	33.11480854	19.56155502
Incidence	Antigua an	Female	55+ years	Non-melanoma skin cancer (basal-cell	Rate	1990	9.8778982	12.87027211	7.222030242
Incidence	Antigua an	Both	55+ years	Non-melanoma skin cancer (basal-cell	Rate	1990	17.022193	21.68971295	12.66632159
Incidence	Antigua an	Male	55+ years	Non-melanoma skin cancer (basal-cell	Number	2021	2.1317766	2.758021931	1.570349307
Incidence	Antigua an	Female	55+ years	Non-melanoma skin cancer (basal-cell	Number	2021	0.8382424	1.114720285	0.607462074
Incidence	Antigua an	Both	55+ years	Non-melanoma skin cancer (basal-cell	Number	2021	2.9700189	3.86147003	2.17321133
Incidence	Antigua an	Male	55+ years	Non-melanoma skin cancer (basal-cell	Percent	2021	5.94E-05	7.74E-05	4.31E-05
Incidence	Antigua an	Female	55+ years	Non-melanoma skin cancer (basal-cell	Percent	2021	1.96E-05	2.60E-05	1.39E-05
Incidence	Antigua an	Both	55+ years	Non-melanoma skin cancer (basal-cell	Percent	2021	3.77E-05	4.91E-05	2.73E-05
Incidence	Antigua an	Male	55+ years	Non-melanoma skin cancer (basal-cell	Rate	2021	23.787191	30.77507989	17.52256748
Incidence	Antigua an	Female	55+ years	Non-melanoma skin cancer (basal-cell	Rate	2021	8.4621719	11.25325428	6.132413014
Incidence	Antigua an	Both	55+ years	Non-melanoma skin cancer (basal-cell	Rate	2021	15.741349	20.46611391	11.51820169
Incidence	Barbados	Male	55+ years	Non-melanoma skin cancer (basal-cell	Number	1990	5.2849619	6.829502372	3.931127604
Incidence	Barbados	Female	55+ years	Non-melanoma skin cancer (basal-cell	Number	1990	2.6988879	3.588475991	1.980107723
Incidence	Barbados	Both	55+ years	Non-melanoma skin cancer (basal-cell	Number	1990	7.9838498	10.49340285	5.892816568
Incidence	Barbados	Male	55+ years	Non-melanoma skin cancer (basal-cell	Percent	1990	6.94E-05	9.03E-05	5.10E-05
Incidence	Barbados	Female	55+ years	Non-melanoma skin cancer (basal-cell	Percent	1990	2.41E-05	3.24E-05	1.72E-05
Incidence	Barbados	Both	55+ years	Non-melanoma skin cancer (basal-cell	Percent	1990	4.24E-05	5.58E-05	3.13E-05
Incidence	Barbados	Male	55+ years	Non-melanoma skin cancer (basal-cell	Rate	1990	26.899849	34.76138302	20.0089882
Incidence	Barbados	Female	55+ years	Non-melanoma skin cancer (basal-cell	Rate	1990	9.9085358	13.17451614	7.269649069
Incidence	Barbados	Both	55+ years	Non-melanoma skin cancer (basal-cell	Rate	1990	17.028646	22.38123818	12.56870941
Incidence	Barbados	Male	55+ years	Non-melanoma skin cancer (basal-cell	Number	2021	10.385788	13.14858032	7.717744641
Incidence	Barbados	Female	55+ years	Non-melanoma skin cancer (basal-cell	Number	2021	4.4973371	5.950385383	3.240666751
Incidence	Barbados	Both	55+ years	Non-melanoma skin cancer (basal-cell	Number	2021	14.883125	19.08909773	11.14607323
Incidence	Barbados	Male	55+ years	Non-melanoma skin cancer (basal-cell	Percent	2021	6.26E-05	7.99E-05	4.55E-05
Incidence	Barbados	Female	55+ years	Non-melanoma skin cancer (basal-cell	Percent	2021	2.10E-05	2.76E-05	1.49E-05
Incidence	Barbados	Both	55+ years	Non-melanoma skin cancer (basal-cell	Percent	2021	3.91E-05	5.01E-05	2.85E-05
Incidence	Barbados	Male	55+ years	Non-melanoma skin cancer (basal-cell	Rate	2021	25.056967	31.72253591	18.61998981
Incidence	Barbados	Female	55+ years	Non-melanoma skin cancer (basal-cell	Rate	2021	9.0544062	11.97980156	6.524374829
Incidence	Barbados	Both	55+ years	Non-melanoma skin cancer (basal-cell	Rate	2021	16.333749	20.94966818	12.23245535
Incidence	Jamaica	Male	55+ years	Non-melanoma skin cancer (basal-cell	Number	1990	39.967978	49.09192424	31.96406105
Incidence	Jamaica	Female	55+ years	Non-melanoma skin cancer (basal-cell	Number	1990	30.653014	37.9044119	24.10753744
Incidence	Jamaica	Both	55+ years	Non-melanoma skin cancer (basal-cell	Number	1990	70.620992	86.39362162	56.73812049
Incidence	Jamaica	Male	55+ years	Non-melanoma skin cancer (basal-cell	Percent	1990	7.50E-05	9.28E-05	5.88E-05
Incidence	Jamaica	Female	55+ years	Non-melanoma skin cancer (basal-cell	Percent	1990	4.69E-05	5.82E-05	3.65E-05
Incidence	Jamaica	Both	55+ years	Non-melanoma skin cancer (basal-cell	Percent	1990	5.95E-05	7.33E-05	4.69E-05
Incidence	Jamaica	Male	55+ years	Non-melanoma skin cancer (basal-cell	Rate	1990	29.22516	35.89672076	23.37258097
Incidence	Jamaica	Female	55+ years	Non-melanoma skin cancer (basal-cell	Rate	1990	19.42908	24.0252999	15.28030084
Incidence	Jamaica	Both	55+ years	Non-melanoma skin cancer (basal-cell	Rate	1990	23.977722	29.33295241	19.26411414
Incidence	Jamaica	Male	55+ years	Non-melanoma skin cancer (basal-cell	Number	2021	65.075152	83.76832099	48.67578819
Incidence	Jamaica	Female	55+ years	Non-melanoma skin cancer (basal-cell	Number	2021	39.181682	49.7544685	29.51775051

Incidence	Jamaica	Both	55+ years	Non-melanoma skin cancer (basal-cell	Number	2021	104.25683	133.2970861	78.28590344
Incidence	Jamaica	Male	55+ years	Non-melanoma skin cancer (basal-cell	Percent	2021	6.54E-05	8.59E-05	4.78E-05
Incidence	Jamaica	Female	55+ years	Non-melanoma skin cancer (basal-cell	Percent	2021	3.42E-05	4.39E-05	2.52E-05
Incidence	Jamaica	Both	55+ years	Non-melanoma skin cancer (basal-cell	Percent	2021	4.87E-05	6.31E-05	3.56E-05
Incidence	Jamaica	Male	55+ years	Non-melanoma skin cancer (basal-cell	Rate	2021	25.469242	32.78541213	19.05082682
Incidence	Jamaica	Female	55+ years	Non-melanoma skin cancer (basal-cell	Rate	2021	14.331941	18.1992721	10.79705179
Incidence	Jamaica	Both	55+ years	Non-melanoma skin cancer (basal-cell	Rate	2021	19.712311	25.20308262	14.80186965
Incidence	Belize	Male	55+ years	Non-melanoma skin cancer (basal-cell	Number	1990	1.8881017	2.478403582	1.393756991
Incidence	Belize	Female	55+ years	Non-melanoma skin cancer (basal-cell	Number	1990	0.6888214	0.889760044	0.505697808
Incidence	Belize	Both	55+ years	Non-melanoma skin cancer (basal-cell	Number	1990	2.576923	3.34956995	1.944327076
Incidence	Belize	Male	55+ years	Non-melanoma skin cancer (basal-cell	Percent	1990	6.25E-05	8.06E-05	4.55E-05
Incidence	Belize	Female	55+ years	Non-melanoma skin cancer (basal-cell	Percent	1990	2.22E-05	2.89E-05	1.59E-05
Incidence	Belize	Both	55+ years	Non-melanoma skin cancer (basal-cell	Percent	1990	4.20E-05	5.39E-05	3.06E-05
Incidence	Belize	Male	55+ years	Non-melanoma skin cancer (basal-cell	Rate	1990	24.382561	32.00559958	17.99869419
Incidence	Belize	Female	55+ years	Non-melanoma skin cancer (basal-cell	Rate	1990	9.005458	11.63247387	6.611351657
Incidence	Belize	Both	55+ years	Non-melanoma skin cancer (basal-cell	Rate	1990	16.741324	21.76092717	12.63157973
Incidence	Belize	Male	55+ years	Non-melanoma skin cancer (basal-cell	Number	2021	5.9237889	7.617357509	4.361119079
Incidence	Belize	Female	55+ years	Non-melanoma skin cancer (basal-cell	Number	2021	2.0283403	2.640253589	1.462974335
Incidence	Belize	Both	55+ years	Non-melanoma skin cancer (basal-cell	Number	2021	7.9521292	10.17114365	5.833946761
Incidence	Belize	Male	55+ years	Non-melanoma skin cancer (basal-cell	Percent	2021	5.90E-05	7.69E-05	4.22E-05
Incidence	Belize	Female	55+ years	Non-melanoma skin cancer (basal-cell	Percent	2021	1.95E-05	2.59E-05	1.39E-05
Incidence	Belize	Both	55+ years	Non-melanoma skin cancer (basal-cell	Percent	2021	3.89E-05	5.05E-05	2.80E-05
Incidence	Belize	Male	55+ years	Non-melanoma skin cancer (basal-cell	Rate	2021	23.239937	29.88406716	17.10934209
Incidence	Belize	Female	55+ years	Non-melanoma skin cancer (basal-cell	Rate	2021	8.2463937	10.7341802	5.947849176
Incidence	Belize	Both	55+ years	Non-melanoma skin cancer (basal-cell	Rate	2021	15.876826	20.30720051	11.6477685
Incidence	Cuba	Male	55+ years	Non-melanoma skin cancer (basal-cell	Number	1990	471.27191	604.3477978	353.9327874
Incidence	Cuba	Female	55+ years	Non-melanoma skin cancer (basal-cell	Number	1990	236.76278	303.7920927	175.2616505
Incidence	Cuba	Both	55+ years	Non-melanoma skin cancer (basal-cell	Number	1990	708.03468	888.2981112	530.1268104
Incidence	Cuba	Male	55+ years	Non-melanoma skin cancer (basal-cell	Percent	1990	0.0001402	0.000180858	0.000103489
Incidence	Cuba	Female	55+ years	Non-melanoma skin cancer (basal-cell	Percent	1990	6.40E-05	8.23E-05	4.64E-05
Incidence	Cuba	Both	55+ years	Non-melanoma skin cancer (basal-cell	Percent	1990	0.0001002	0.000127865	7.51E-05
Incidence	Cuba	Male	55+ years	Non-melanoma skin cancer (basal-cell	Rate	1990	56.372777	72.29109805	42.33686286
Incidence	Cuba	Female	55+ years	Non-melanoma skin cancer (basal-cell	Rate	1990	27.572003	35.37784386	20.40994303
Incidence	Cuba	Both	55+ years	Non-melanoma skin cancer (basal-cell	Rate	1990	41.779372	52.41627008	31.28146927
Incidence	Cuba	Male	55+ years	Non-melanoma skin cancer (basal-cell	Number	2021	732.68806	913.0358742	547.7957616
Incidence	Cuba	Female	55+ years	Non-melanoma skin cancer (basal-cell	Number	2021	440.85152	550.0557169	335.5062902
Incidence	Cuba	Both	55+ years	Non-melanoma skin cancer (basal-cell	Number	2021	1173.5396	1472.270208	890.4952368
Incidence	Cuba	Male	55+ years	Non-melanoma skin cancer (basal-cell	Percent	2021	0.0001099	0.000140238	8.17E-05
Incidence	Cuba	Female	55+ years	Non-melanoma skin cancer (basal-cell	Percent	2021	5.37E-05	6.71E-05	3.98E-05
Incidence	Cuba	Both	55+ years	Non-melanoma skin cancer (basal-cell	Percent	2021	7.88E-05	9.90E-05	5.87E-05
Incidence	Cuba	Male	55+ years	Non-melanoma skin cancer (basal-cell	Rate	2021	45.812233	57.08870543	34.2516124
Incidence	Cuba	Female	55+ years	Non-melanoma skin cancer (basal-cell	Rate	2021	24.25301	30.26077014	18.45754606
Incidence	Cuba	Both	55+ years	Non-melanoma skin cancer (basal-cell	Rate	2021	34.343675	43.08603636	26.06037257
Incidence	Grenada	Male	55+ years	Non-melanoma skin cancer (basal-cell	Number	1990	1.3584155	1.73033648	1.016255164
Incidence	Grenada	Female	55+ years	Non-melanoma skin cancer (basal-cell	Number	1990	0.6904984	0.892698139	0.50331663
Incidence	Grenada	Both	55+ years	Non-melanoma skin cancer (basal-cell	Number	1990	2.0489139	2.621688112	1.523439935
Incidence	Grenada	Male	55+ years	Non-melanoma skin cancer (basal-cell	Percent	1990	6.78E-05	8.79E-05	5.00E-05
Incidence	Grenada	Female	55+ years	Non-melanoma skin cancer (basal-cell	Percent	1990	2.41E-05	3.17E-05	1.72E-05
Incidence	Grenada	Both	55+ years	Non-melanoma skin cancer (basal-cell	Percent	1990	4.21E-05	5.50E-05	3.10E-05
Incidence	Grenada	Male	55+ years	Non-melanoma skin cancer (basal-cell	Rate	1990	26.808165	34.1479794	20.05567172
Incidence	Grenada	Female	55+ years	Non-melanoma skin cancer (basal-cell	Rate	1990	9.8755013	12.76735971	7.198429325
Incidence	Grenada	Both	55+ years	Non-melanoma skin cancer (basal-cell	Rate	1990	16.990456	21.74014056	12.63300474
Incidence	Grenada	Male	55+ years	Non-melanoma skin cancer (basal-cell	Number	2021	2.2441199	2.998863052	1.643367431
Incidence	Grenada	Female	55+ years	Non-melanoma skin cancer (basal-cell	Number	2021	0.9069789	1.177277875	0.657601267
Incidence	Grenada	Both	55+ years	Non-melanoma skin cancer (basal-cell	Number	2021	3.1510989	4.193039564	2.311057248
Incidence	Grenada	Male	55+ years	Non-melanoma skin cancer (basal-cell	Percent	2021	5.63E-05	7.52E-05	3.95E-05

Incidence	Grenada	Female	55+ years	Non-melanoma skin cancer (basal-cell	Percent	2021	2.05E-05	2.74E-05	1.45E-05
Incidence	Grenada	Both	55+ years	Non-melanoma skin cancer (basal-cell	Percent	2021	3.75E-05	4.99E-05	2.66E-05
Incidence	Grenada	Male	55+ years	Non-melanoma skin cancer (basal-cell	Rate	2021	22.968878	30.69377871	16.82009328
Incidence	Grenada	Female	55+ years	Non-melanoma skin cancer (basal-cell	Rate	2021	8.7912161	11.4111848	6.374034327
Incidence	Grenada	Both	55+ years	Non-melanoma skin cancer (basal-cell	Rate	2021	15.687145	20.87424845	11.50515812
Incidence	Dominican	Male	55+ years	Non-melanoma skin cancer (basal-cell	Number	1990	71.721468	91.84120046	53.30422584
Incidence	Dominican	Female	55+ years	Non-melanoma skin cancer (basal-cell	Number	1990	25.181994	33.36023777	18.38460135
Incidence	Dominican	Both	55+ years	Non-melanoma skin cancer (basal-cell	Number	1990	96.903462	124.6042509	72.63185853
Incidence	Dominican	Male	55+ years	Non-melanoma skin cancer (basal-cell	Percent	1990	6.37E-05	8.38E-05	4.56E-05
Incidence	Dominican	Female	55+ years	Non-melanoma skin cancer (basal-cell	Percent	1990	2.07E-05	2.73E-05	1.49E-05
Incidence	Dominican	Both	55+ years	Non-melanoma skin cancer (basal-cell	Percent	1990	4.13E-05	5.38E-05	3.00E-05
Incidence	Dominican	Male	55+ years	Non-melanoma skin cancer (basal-cell	Rate	1990	23.622684	30.24946004	17.55665259
Incidence	Dominican	Female	55+ years	Non-melanoma skin cancer (basal-cell	Rate	1990	8.1816835	10.83881239	5.973196181
Incidence	Dominican	Both	55+ years	Non-melanoma skin cancer (basal-cell	Rate	1990	15.849498	20.38022943	11.87964239
Incidence	Dominican	Male	55+ years	Non-melanoma skin cancer (basal-cell	Number	2021	197.58256	252.1446406	147.9574609
Incidence	Dominican	Female	55+ years	Non-melanoma skin cancer (basal-cell	Number	2021	77.090998	102.2457937	55.97297538
Incidence	Dominican	Both	55+ years	Non-melanoma skin cancer (basal-cell	Number	2021	274.67355	355.2227194	207.9003616
Incidence	Dominican	Male	55+ years	Non-melanoma skin cancer (basal-cell	Percent	2021	6.39E-05	8.20E-05	4.72E-05
Incidence	Dominican	Female	55+ years	Non-melanoma skin cancer (basal-cell	Percent	2021	2.16E-05	2.90E-05	1.56E-05
Incidence	Dominican	Both	55+ years	Non-melanoma skin cancer (basal-cell	Percent	2021	4.12E-05	5.39E-05	3.03E-05
Incidence	Dominican	Male	55+ years	Non-melanoma skin cancer (basal-cell	Rate	2021	24.582619	31.37106674	18.40841578
Incidence	Dominican	Female	55+ years	Non-melanoma skin cancer (basal-cell	Rate	2021	8.8888782	11.78931954	6.453891827
Incidence	Dominican	Both	55+ years	Non-melanoma skin cancer (basal-cell	Rate	2021	16.437441	21.25778954	12.44149625
Incidence	Haiti	Male	55+ years	Non-melanoma skin cancer (basal-cell	Number	1990	60.780111	81.61863682	43.27108049
Incidence	Haiti	Female	55+ years	Non-melanoma skin cancer (basal-cell	Number	1990	22.406158	30.06190946	15.69082902
Incidence	Haiti	Both	55+ years	Non-melanoma skin cancer (basal-cell	Number	1990	83.186268	110.6316033	58.99532536
Incidence	Haiti	Male	55+ years	Non-melanoma skin cancer (basal-cell	Percent	1990	5.50E-05	7.34E-05	3.92E-05
Incidence	Haiti	Female	55+ years	Non-melanoma skin cancer (basal-cell	Percent	1990	1.98E-05	2.66E-05	1.38E-05
Incidence	Haiti	Both	55+ years	Non-melanoma skin cancer (basal-cell	Percent	1990	3.72E-05	4.96E-05	2.62E-05
Incidence	Haiti	Male	55+ years	Non-melanoma skin cancer (basal-cell	Rate	1990	22.913088	30.7688645	16.31247549
Incidence	Haiti	Female	55+ years	Non-melanoma skin cancer (basal-cell	Rate	1990	8.1747177	10.96786094	5.724680627
Incidence	Haiti	Both	55+ years	Non-melanoma skin cancer (basal-cell	Rate	1990	15.423297	20.51184811	10.93813266
Incidence	Haiti	Male	55+ years	Non-melanoma skin cancer (basal-cell	Number	2021	131.7921	172.9250679	96.78262825
Incidence	Haiti	Female	55+ years	Non-melanoma skin cancer (basal-cell	Number	2021	49.650204	66.97989978	33.85533059
Incidence	Haiti	Both	55+ years	Non-melanoma skin cancer (basal-cell	Number	2021	181.4423	237.4740699	132.6376627
Incidence	Haiti	Male	55+ years	Non-melanoma skin cancer (basal-cell	Percent	2021	5.60E-05	7.43E-05	4.05E-05
Incidence	Haiti	Female	55+ years	Non-melanoma skin cancer (basal-cell	Percent	2021	1.84E-05	2.50E-05	1.26E-05
Incidence	Haiti	Both	55+ years	Non-melanoma skin cancer (basal-cell	Percent	2021	3.60E-05	4.76E-05	2.55E-05
Incidence	Haiti	Male	55+ years	Non-melanoma skin cancer (basal-cell	Rate	2021	23.426227	30.73766941	17.20324571
Incidence	Haiti	Female	55+ years	Non-melanoma skin cancer (basal-cell	Rate	2021	7.9758754	10.75974093	5.438565712
Incidence	Haiti	Both	55+ years	Non-melanoma skin cancer (basal-cell	Rate	2021	15.310446	20.03851272	11.19221772
Incidence	Guyana	Male	55+ years	Non-melanoma skin cancer (basal-cell	Number	1990	7.1324954	9.286283794	5.15696297
Incidence	Guyana	Female	55+ years	Non-melanoma skin cancer (basal-cell	Number	1990	2.7394528	3.694571728	1.9450755
Incidence	Guyana	Both	55+ years	Non-melanoma skin cancer (basal-cell	Number	1990	9.8719482	12.91214177	7.120543222
Incidence	Guyana	Male	55+ years	Non-melanoma skin cancer (basal-cell	Percent	1990	6.07E-05	8.02E-05	4.34E-05
Incidence	Guyana	Female	55+ years	Non-melanoma skin cancer (basal-cell	Percent	1990	2.03E-05	2.78E-05	1.44E-05
Incidence	Guyana	Both	55+ years	Non-melanoma skin cancer (basal-cell	Percent	1990	3.91E-05	5.16E-05	2.77E-05
Incidence	Guyana	Male	55+ years	Non-melanoma skin cancer (basal-cell	Rate	1990	24.206042	31.51550214	17.5015411
Incidence	Guyana	Female	55+ years	Non-melanoma skin cancer (basal-cell	Rate	1990	8.6059462	11.60643653	6.110422803
Incidence	Guyana	Both	55+ years	Non-melanoma skin cancer (basal-cell	Rate	1990	16.104883	21.0645887	11.61630014
Incidence	Guyana	Male	55+ years	Non-melanoma skin cancer (basal-cell	Number	2021	12.439297	16.56326675	8.911707115
Incidence	Guyana	Female	55+ years	Non-melanoma skin cancer (basal-cell	Number	2021	4.9858894	6.601358131	3.510991109
Incidence	Guyana	Both	55+ years	Non-melanoma skin cancer (basal-cell	Number	2021	17.425187	23.00657729	12.49932416
Incidence	Guyana	Male	55+ years	Non-melanoma skin cancer (basal-cell	Percent	2021	5.50E-05	7.40E-05	3.87E-05
Incidence	Guyana	Female	55+ years	Non-melanoma skin cancer (basal-cell	Percent	2021	1.81E-05	2.46E-05	1.26E-05
Incidence	Guyana	Both	55+ years	Non-melanoma skin cancer (basal-cell	Percent	2021	3.47E-05	4.70E-05	2.44E-05

Incidence	Guyana	Male	55+ years	Non-melanoma skin cancer (basal-cell	Rate	2021	23.628296	31.46172636	16.92768069
Incidence	Guyana	Female	55+ years	Non-melanoma skin cancer (basal-cell	Rate	2021	8.3137404	11.00745985	5.854415548
Incidence	Guyana	Both	55+ years	Non-melanoma skin cancer (basal-cell	Rate	2021	15.472902	20.42896464	11.09892393
Incidence	Saint Luci	Male	55+ years	Non-melanoma skin cancer (basal-cell	Number	1990	1.6006574	2.073651913	1.159598149
Incidence	Saint Luci	Female	55+ years	Non-melanoma skin cancer (basal-cell	Number	1990	0.7274528	0.97499416	0.525159553
Incidence	Saint Luci	Both	55+ years	Non-melanoma skin cancer (basal-cell	Number	1990	2.3281103	3.067588559	1.691050412
Incidence	Saint Luci	Male	55+ years	Non-melanoma skin cancer (basal-cell	Percent	1990	6.40E-05	8.38E-05	4.61E-05
Incidence	Saint Luci	Female	55+ years	Non-melanoma skin cancer (basal-cell	Percent	1990	2.18E-05	2.97E-05	1.57E-05
Incidence	Saint Luci	Both	55+ years	Non-melanoma skin cancer (basal-cell	Percent	1990	3.99E-05	5.27E-05	2.88E-05
Incidence	Saint Luci	Male	55+ years	Non-melanoma skin cancer (basal-cell	Rate	1990	24.861772	32.20842886	18.0111398
Incidence	Saint Luci	Female	55+ years	Non-melanoma skin cancer (basal-cell	Rate	1990	8.8807176	11.90269293	6.411128552
Incidence	Saint Luci	Both	55+ years	Non-melanoma skin cancer (basal-cell	Rate	1990	15.913695	20.96836643	11.55910058
Incidence	Saint Luci	Male	55+ years	Non-melanoma skin cancer (basal-cell	Number	2021	4.872581	6.312842353	3.562695759
Incidence	Saint Luci	Female	55+ years	Non-melanoma skin cancer (basal-cell	Number	2021	1.9361061	2.49252322	1.419443041
Incidence	Saint Luci	Both	55+ years	Non-melanoma skin cancer (basal-cell	Number	2021	6.8086871	8.78820804	4.984951982
Incidence	Saint Luci	Male	55+ years	Non-melanoma skin cancer (basal-cell	Percent	2021	5.96E-05	7.72E-05	4.31E-05
Incidence	Saint Luci	Female	55+ years	Non-melanoma skin cancer (basal-cell	Percent	2021	2.06E-05	2.70E-05	1.48E-05
Incidence	Saint Luci	Both	55+ years	Non-melanoma skin cancer (basal-cell	Percent	2021	3.88E-05	4.96E-05	2.81E-05
Incidence	Saint Luci	Male	55+ years	Non-melanoma skin cancer (basal-cell	Rate	2021	24.172528	31.31756203	17.67428033
Incidence	Saint Luci	Female	55+ years	Non-melanoma skin cancer (basal-cell	Rate	2021	8.830845	11.36873959	6.474274009
Incidence	Saint Luci	Both	55+ years	Non-melanoma skin cancer (basal-cell	Rate	2021	16.17962	20.8835949	11.84584131
Incidence	Saint Vinc	Male	55+ years	Non-melanoma skin cancer (basal-cell	Number	1990	1.3221994	1.69984458	0.990092496
Incidence	Saint Vinc	Female	55+ years	Non-melanoma skin cancer (basal-cell	Number	1990	0.612886	0.819574273	0.455630807
Incidence	Saint Vinc	Both	55+ years	Non-melanoma skin cancer (basal-cell	Number	1990	1.9350854	2.504895624	1.44156541
Incidence	Saint Vinc	Male	55+ years	Non-melanoma skin cancer (basal-cell	Percent	1990	6.36E-05	8.27E-05	4.62E-05
Incidence	Saint Vinc	Female	55+ years	Non-melanoma skin cancer (basal-cell	Percent	1990	2.21E-05	2.99E-05	1.60E-05
Incidence	Saint Vinc	Both	55+ years	Non-melanoma skin cancer (basal-cell	Percent	1990	3.99E-05	5.27E-05	2.89E-05
Incidence	Saint Vinc	Male	55+ years	Non-melanoma skin cancer (basal-cell	Rate	1990	24.854157	31.95297521	18.61134915
Incidence	Saint Vinc	Female	55+ years	Non-melanoma skin cancer (basal-cell	Rate	1990	9.1548165	12.24216636	6.805860456
Incidence	Saint Vinc	Both	55+ years	Non-melanoma skin cancer (basal-cell	Rate	1990	16.106229	20.8489112	11.99853157
Incidence	Saint Vinc	Male	55+ years	Non-melanoma skin cancer (basal-cell	Number	2021	3.1839818	4.139751006	2.304613369
Incidence	Saint Vinc	Female	55+ years	Non-melanoma skin cancer (basal-cell	Number	2021	1.0968898	1.479046109	0.793564746
Incidence	Saint Vinc	Both	55+ years	Non-melanoma skin cancer (basal-cell	Number	2021	4.2808717	5.547158845	3.138228807
Incidence	Saint Vinc	Male	55+ years	Non-melanoma skin cancer (basal-cell	Percent	2021	6.10E-05	7.86E-05	4.41E-05
Incidence	Saint Vinc	Female	55+ years	Non-melanoma skin cancer (basal-cell	Percent	2021	2.08E-05	2.82E-05	1.51E-05
Incidence	Saint Vinc	Both	55+ years	Non-melanoma skin cancer (basal-cell	Percent	2021	4.08E-05	5.30E-05	2.94E-05
Incidence	Saint Vinc	Male	55+ years	Non-melanoma skin cancer (basal-cell	Rate	2021	24.317417	31.61703052	17.60130769
Incidence	Saint Vinc	Female	55+ years	Non-melanoma skin cancer (basal-cell	Rate	2021	8.894482	11.99331848	6.434873579
Incidence	Saint Vinc	Both	55+ years	Non-melanoma skin cancer (basal-cell	Rate	2021	16.836808	21.81715722	12.34275657
Incidence	Trinidad a	Male	55+ years	Non-melanoma skin cancer (basal-cell	Number	1990	12.195337	14.26930988	10.19487902
Incidence	Trinidad a	Female	55+ years	Non-melanoma skin cancer (basal-cell	Number	1990	6.7473877	8.291859216	5.355718917
Incidence	Trinidad a	Both	55+ years	Non-melanoma skin cancer (basal-cell	Number	1990	18.942725	22.44609934	15.62133178
Incidence	Trinidad a	Male	55+ years	Non-melanoma skin cancer (basal-cell	Percent	1990	4.90E-05	5.82E-05	3.98E-05
Incidence	Trinidad a	Female	55+ years	Non-melanoma skin cancer (basal-cell	Percent	1990	2.33E-05	2.92E-05	1.82E-05
Incidence	Trinidad a	Both	55+ years	Non-melanoma skin cancer (basal-cell	Percent	1990	3.52E-05	4.19E-05	2.82E-05
Incidence	Trinidad a	Male	55+ years	Non-melanoma skin cancer (basal-cell	Rate	1990	18.680542	21.85740582	15.61628487
Incidence	Trinidad a	Female	55+ years	Non-melanoma skin cancer (basal-cell	Rate	1990	9.3692575	11.51387298	7.436820346
Incidence	Trinidad a	Both	55+ years	Non-melanoma skin cancer (basal-cell	Rate	1990	13.796607	16.34822869	11.37752713
Incidence	Trinidad a	Male	55+ years	Non-melanoma skin cancer (basal-cell	Number	2021	34.625275	45.01785739	25.2169083
Incidence	Trinidad a	Female	55+ years	Non-melanoma skin cancer (basal-cell	Number	2021	19.281629	25.43860927	13.82066591
Incidence	Trinidad a	Both	55+ years	Non-melanoma skin cancer (basal-cell	Number	2021	53.906904	68.98996916	39.96594283
Incidence	Trinidad a	Male	55+ years	Non-melanoma skin cancer (basal-cell	Percent	2021	5.09E-05	6.68E-05	3.67E-05
Incidence	Trinidad a	Female	55+ years	Non-melanoma skin cancer (basal-cell	Percent	2021	2.50E-05	3.36E-05	1.77E-05
Incidence	Trinidad a	Both	55+ years	Non-melanoma skin cancer (basal-cell	Percent	2021	3.72E-05	4.90E-05	2.68E-05
Incidence	Trinidad a	Male	55+ years	Non-melanoma skin cancer (basal-cell	Rate	2021	20.75187	26.98042791	15.11317988
Incidence	Trinidad a	Female	55+ years	Non-melanoma skin cancer (basal-cell	Rate	2021	10.624363	14.0169185	7.615319909

Incidence	Trinidad a	Both	55+ years	Non-melanoma skin cancer (basal-cell	Rate	2021	15.475424	19.80542253	11.47329668
Incidence	Bolivia (P	Male	55+ years	Non-melanoma skin cancer (basal-cell	Number	1990	147.85577	189.3722929	113.0715807
Incidence	Bolivia (P	Female	55+ years	Non-melanoma skin cancer (basal-cell	Number	1990	139.87357	177.6001973	108.1650471
Incidence	Bolivia (P	Both	55+ years	Non-melanoma skin cancer (basal-cell	Number	1990	287.72934	362.8196494	223.0329143
Incidence	Bolivia (P	Male	55+ years	Non-melanoma skin cancer (basal-cell	Percent	1990	0.0001565	0.000200084	0.000117691
Incidence	Bolivia (P	Female	55+ years	Non-melanoma skin cancer (basal-cell	Percent	1990	0.0001172	0.000148838	8.83E-05
Incidence	Bolivia (P	Both	55+ years	Non-melanoma skin cancer (basal-cell	Percent	1990	0.0001346	0.000170494	0.00010186
Incidence	Bolivia (P	Male	55+ years	Non-melanoma skin cancer (basal-cell	Rate	1990	60.158503	77.05044837	46.0057586
Incidence	Bolivia (P	Female	55+ years	Non-melanoma skin cancer (basal-cell	Rate	1990	50.214088	63.7578084	38.83084845
Incidence	Bolivia (P	Both	55+ years	Non-melanoma skin cancer (basal-cell	Rate	1990	54.875469	69.19662262	42.53662785
Incidence	Bolivia (P	Male	55+ years	Non-melanoma skin cancer (basal-cell	Number	2021	453.49521	582.6205764	342.8284178
Incidence	Bolivia (P	Female	55+ years	Non-melanoma skin cancer (basal-cell	Number	2021	419.28381	532.4738059	313.6357678
Incidence	Bolivia (P	Both	55+ years	Non-melanoma skin cancer (basal-cell	Number	2021	872.77902	1104.645399	660.6346766
Incidence	Bolivia (P	Male	55+ years	Non-melanoma skin cancer (basal-cell	Percent	2021	0.0001481	0.000190423	0.00010742
Incidence	Bolivia (P	Female	55+ years	Non-melanoma skin cancer (basal-cell	Percent	2021	0.0001121	0.000143045	8.28E-05
Incidence	Bolivia (P	Both	55+ years	Non-melanoma skin cancer (basal-cell	Percent	2021	0.0001283	0.00016236	9.43E-05
Incidence	Bolivia (P	Male	55+ years	Non-melanoma skin cancer (basal-cell	Rate	2021	62.074873	79.74968161	46.92669342
Incidence	Bolivia (P	Female	55+ years	Non-melanoma skin cancer (basal-cell	Rate	2021	51.451544	65.34141973	38.48716334
Incidence	Bolivia (P	Both	55+ years	Non-melanoma skin cancer (basal-cell	Rate	2021	56.47331	71.47626196	42.74647523
Incidence	Peru	Male	55+ years	Non-melanoma skin cancer (basal-cell	Number	1990	602.42869	674.5478554	534.1319914
Incidence	Peru	Female	55+ years	Non-melanoma skin cancer (basal-cell	Number	1990	596.12445	675.9240688	519.5617932
Incidence	Peru	Both	55+ years	Non-melanoma skin cancer (basal-cell	Number	1990	1198.5531	1344.513336	1058.895696
Incidence	Peru	Male	55+ years	Non-melanoma skin cancer (basal-cell	Percent	1990	0.0001429	0.00016241	0.000123247
Incidence	Peru	Female	55+ years	Non-melanoma skin cancer (basal-cell	Percent	1990	0.000118	0.000135692	1.00E-04
Incidence	Peru	Both	55+ years	Non-melanoma skin cancer (basal-cell	Percent	1990	0.0001293	0.000146959	0.000110455
Incidence	Peru	Male	55+ years	Non-melanoma skin cancer (basal-cell	Rate	1990	62.361448	69.82698757	55.29159663
Incidence	Peru	Female	55+ years	Non-melanoma skin cancer (basal-cell	Rate	1990	59.275222	67.21004189	51.66226725
Incidence	Peru	Both	55+ years	Non-melanoma skin cancer (basal-cell	Rate	1990	60.787295	68.18999133	53.70425597
Incidence	Peru	Male	55+ years	Non-melanoma skin cancer (basal-cell	Number	2021	1402.3892	1777.852485	1075.745773
Incidence	Peru	Female	55+ years	Non-melanoma skin cancer (basal-cell	Number	2021	1189.9146	1483.547164	903.9507914
Incidence	Peru	Both	55+ years	Non-melanoma skin cancer (basal-cell	Number	2021	2592.3038	3224.410824	1995.549202
Incidence	Peru	Male	55+ years	Non-melanoma skin cancer (basal-cell	Percent	2021	0.0001182	0.000149181	8.84E-05
Incidence	Peru	Female	55+ years	Non-melanoma skin cancer (basal-cell	Percent	2021	8.50E-05	0.000106591	6.33E-05
Incidence	Peru	Both	55+ years	Non-melanoma skin cancer (basal-cell	Percent	2021	0.0001002	0.000125183	7.50E-05
Incidence	Peru	Male	55+ years	Non-melanoma skin cancer (basal-cell	Rate	2021	52.175439	66.14442979	40.02277542
Incidence	Peru	Female	55+ years	Non-melanoma skin cancer (basal-cell	Rate	2021	40.952332	51.05804787	31.11054634
Incidence	Peru	Both	55+ years	Non-melanoma skin cancer (basal-cell	Rate	2021	46.345405	57.64626355	35.67658139
Incidence	Colombia	Male	55+ years	Non-melanoma skin cancer (basal-cell	Number	1990	1727.5403	2215.667311	1315.845144
Incidence	Colombia	Female	55+ years	Non-melanoma skin cancer (basal-cell	Number	1990	1614.2031	2044.658761	1230.701331
Incidence	Colombia	Both	55+ years	Non-melanoma skin cancer (basal-cell	Number	1990	3341.7433	4277.615857	2543.789008
Incidence	Colombia	Male	55+ years	Non-melanoma skin cancer (basal-cell	Percent	1990	0.0003006	0.000392213	0.000222258
Incidence	Colombia	Female	55+ years	Non-melanoma skin cancer (basal-cell	Percent	1990	0.0002536	0.000327715	0.000188227
Incidence	Colombia	Both	55+ years	Non-melanoma skin cancer (basal-cell	Percent	1990	0.0002759	0.000353536	0.000204146
Incidence	Colombia	Male	55+ years	Non-melanoma skin cancer (basal-cell	Rate	1990	123.57089	158.4865953	94.12235119
Incidence	Colombia	Female	55+ years	Non-melanoma skin cancer (basal-cell	Rate	1990	108.99598	138.0616757	83.10075563
Incidence	Colombia	Both	55+ years	Non-melanoma skin cancer (basal-cell	Rate	1990	116.07344	148.5804095	88.35697854
Incidence	Colombia	Male	55+ years	Non-melanoma skin cancer (basal-cell	Number	2021	5650.4824	7062.180156	4390.57472
Incidence	Colombia	Female	55+ years	Non-melanoma skin cancer (basal-cell	Number	2021	6155.7899	7612.849767	4765.007742
Incidence	Colombia	Both	55+ years	Non-melanoma skin cancer (basal-cell	Number	2021	11806.272	14584.01239	9235.508122
Incidence	Colombia	Male	55+ years	Non-melanoma skin cancer (basal-cell	Percent	2021	0.0003162	0.000400337	0.000240306
Incidence	Colombia	Female	55+ years	Non-melanoma skin cancer (basal-cell	Percent	2021	0.0002679	0.000333537	0.000202939
Incidence	Colombia	Both	55+ years	Non-melanoma skin cancer (basal-cell	Percent	2021	0.000289	0.000361897	0.000221349
Incidence	Colombia	Male	55+ years	Non-melanoma skin cancer (basal-cell	Rate	2021	131.26312	164.0574599	101.9949251
Incidence	Colombia	Female	55+ years	Non-melanoma skin cancer (basal-cell	Rate	2021	117.05684	144.7639021	90.61010466
Incidence	Colombia	Both	55+ years	Non-melanoma skin cancer (basal-cell	Rate	2021	123.45134	152.4965508	96.5703467
Incidence	Costa Rica	Male	55+ years	Non-melanoma skin cancer (basal-cell	Number	1990	266.97137	281.1015713	252.5133255

Incidence	Costa Rica	Female	55+ years	Non-melanoma skin cancer (basal-cell	Number	1990	280.27247	295.2518101	265.0410199
Incidence	Costa Rica	Both	55+ years	Non-melanoma skin cancer (basal-cell	Number	1990	547.24384	573.8542129	518.8174143
Incidence	Costa Rica	Male	55+ years	Non-melanoma skin cancer (basal-cell	Percent	1990	0.0004714	0.000511001	0.000430074
Incidence	Costa Rica	Female	55+ years	Non-melanoma skin cancer (basal-cell	Percent	1990	0.0004331	0.00046985	0.000397136
Incidence	Costa Rica	Both	55+ years	Non-melanoma skin cancer (basal-cell	Percent	1990	0.0004509	0.000484111	0.00041368
Incidence	Costa Rica	Male	55+ years	Non-melanoma skin cancer (basal-cell	Rate	1990	193.29236	203.5228992	182.8244639
Incidence	Costa Rica	Female	55+ years	Non-melanoma skin cancer (basal-cell	Rate	1990	191.60308	201.8434293	181.1903823
Incidence	Costa Rica	Both	55+ years	Non-melanoma skin cancer (basal-cell	Rate	1990	192.42349	201.780306	182.428105
Incidence	Costa Rica	Male	55+ years	Non-melanoma skin cancer (basal-cell	Number	2021	665.82588	821.4361598	516.5301769
Incidence	Costa Rica	Female	55+ years	Non-melanoma skin cancer (basal-cell	Number	2021	693.39291	859.2708727	534.4872165
Incidence	Costa Rica	Both	55+ years	Non-melanoma skin cancer (basal-cell	Number	2021	1359.2188	1679.460271	1057.729179
Incidence	Costa Rica	Male	55+ years	Non-melanoma skin cancer (basal-cell	Percent	2021	0.0003607	0.0004461	0.000275421
Incidence	Costa Rica	Female	55+ years	Non-melanoma skin cancer (basal-cell	Percent	2021	0.0002915	0.000366893	0.000220262
Incidence	Costa Rica	Both	55+ years	Non-melanoma skin cancer (basal-cell	Percent	2021	0.0003217	0.00039767	0.000245457
Incidence	Costa Rica	Male	55+ years	Non-melanoma skin cancer (basal-cell	Rate	2021	151.70137	187.1555165	117.6859223
Incidence	Costa Rica	Female	55+ years	Non-melanoma skin cancer (basal-cell	Rate	2021	133.71577	165.7041236	103.0719632
Incidence	Costa Rica	Both	55+ years	Non-melanoma skin cancer (basal-cell	Rate	2021	141.96045	175.4073306	110.4720696
Incidence	El Salvado	Male	55+ years	Non-melanoma skin cancer (basal-cell	Number	1990	300.11965	373.5233352	227.4341506
Incidence	El Salvado	Female	55+ years	Non-melanoma skin cancer (basal-cell	Number	1990	301.81529	375.3396769	233.91255
Incidence	El Salvado	Both	55+ years	Non-melanoma skin cancer (basal-cell	Number	1990	601.93495	747.0490007	460.2881641
Incidence	El Salvado	Male	55+ years	Non-melanoma skin cancer (basal-cell	Percent	1990	0.0003155	0.000394418	0.000235575
Incidence	El Salvado	Female	55+ years	Non-melanoma skin cancer (basal-cell	Percent	1990	0.0002691	0.000338592	0.000203584
Incidence	El Salvado	Both	55+ years	Non-melanoma skin cancer (basal-cell	Percent	1990	0.0002904	0.000363382	0.000217061
Incidence	El Salvado	Male	55+ years	Non-melanoma skin cancer (basal-cell	Rate	1990	132.09793	164.4066222	100.10534
Incidence	El Salvado	Female	55+ years	Non-melanoma skin cancer (basal-cell	Rate	1990	115.30075	143.3888496	89.36026087
Incidence	El Salvado	Both	55+ years	Non-melanoma skin cancer (basal-cell	Rate	1990	123.10557	152.783777	94.13648122
Incidence	El Salvado	Male	55+ years	Non-melanoma skin cancer (basal-cell	Number	2021	610.13106	767.9834465	467.5838819
Incidence	El Salvado	Female	55+ years	Non-melanoma skin cancer (basal-cell	Number	2021	728.15507	901.1129423	552.0886501
Incidence	El Salvado	Both	55+ years	Non-melanoma skin cancer (basal-cell	Number	2021	1338.2861	1673.505112	1035.38057
Incidence	El Salvado	Male	55+ years	Non-melanoma skin cancer (basal-cell	Percent	2021	0.0003477	0.00044303	0.00026484
Incidence	El Salvado	Female	55+ years	Non-melanoma skin cancer (basal-cell	Percent	2021	0.0002905	0.000364505	0.000216066
Incidence	El Salvado	Both	55+ years	Non-melanoma skin cancer (basal-cell	Percent	2021	0.000314	0.00039201	0.000238118
Incidence	El Salvado	Male	55+ years	Non-melanoma skin cancer (basal-cell	Rate	2021	143.11301	180.1390337	109.6769846
Incidence	El Salvado	Female	55+ years	Non-melanoma skin cancer (basal-cell	Rate	2021	122.12138	151.128742	92.59268091
Incidence	El Salvado	Both	55+ years	Non-melanoma skin cancer (basal-cell	Rate	2021	130.87306	163.6546422	101.2514605
Incidence	Argentina	Male	55+ years	Non-melanoma skin cancer (basal-cell	Number	1990	2947.2277	3370.753704	2537.560839
Incidence	Argentina	Female	55+ years	Non-melanoma skin cancer (basal-cell	Number	1990	2775.9459	3248.606596	2331.696107
Incidence	Argentina	Both	55+ years	Non-melanoma skin cancer (basal-cell	Number	1990	5723.1735	6587.962074	4932.367866
Incidence	Argentina	Male	55+ years	Non-melanoma skin cancer (basal-cell	Percent	1990	0.0003062	0.000358842	0.000255421
Incidence	Argentina	Female	55+ years	Non-melanoma skin cancer (basal-cell	Percent	1990	0.0002189	0.000260283	0.00017824
Incidence	Argentina	Both	55+ years	Non-melanoma skin cancer (basal-cell	Percent	1990	0.0002566	0.000301102	0.000211099
Incidence	Argentina	Male	55+ years	Non-melanoma skin cancer (basal-cell	Rate	1990	118.86105	135.9417621	102.3392755
Incidence	Argentina	Female	55+ years	Non-melanoma skin cancer (basal-cell	Rate	1990	89.980563	105.3015673	75.5804827
Incidence	Argentina	Both	55+ years	Non-melanoma skin cancer (basal-cell	Rate	1990	102.84954	118.390413	88.6381953
Incidence	Argentina	Male	55+ years	Non-melanoma skin cancer (basal-cell	Number	2021	5253.5299	6613.025681	4081.009223
Incidence	Argentina	Female	55+ years	Non-melanoma skin cancer (basal-cell	Number	2021	4798.3831	6096.301804	3714.341101
Incidence	Argentina	Both	55+ years	Non-melanoma skin cancer (basal-cell	Number	2021	10051.913	12663.98582	7825.779689
Incidence	Argentina	Male	55+ years	Non-melanoma skin cancer (basal-cell	Percent	2021	0.0003149	0.000398647	0.000237473
Incidence	Argentina	Female	55+ years	Non-melanoma skin cancer (basal-cell	Percent	2021	0.0002178	0.000283365	0.000165815
Incidence	Argentina	Both	55+ years	Non-melanoma skin cancer (basal-cell	Percent	2021	0.0002596	0.000331502	0.000197241
Incidence	Argentina	Male	55+ years	Non-melanoma skin cancer (basal-cell	Rate	2021	126.34291	159.0376205	98.14478685
Incidence	Argentina	Female	55+ years	Non-melanoma skin cancer (basal-cell	Rate	2021	92.719549	117.7993393	71.77251744
Incidence	Argentina	Both	55+ years	Non-melanoma skin cancer (basal-cell	Rate	2021	107.69934	135.685906	83.8478519
Incidence	Bahamas	Male	55+ years	Non-melanoma skin cancer (basal-cell	Number	1990	2.5664978	3.323245264	1.880721533
Incidence	Bahamas	Female	55+ years	Non-melanoma skin cancer (basal-cell	Number	1990	1.2120239	1.614684698	0.8782278
Incidence	Bahamas	Both	55+ years	Non-melanoma skin cancer (basal-cell	Number	1990	3.7785217	4.902568064	2.766754745

Incidence	Bahamas	Male	55+ years	Non-melanoma skin cancer (basal-cell	Percent	1990	6.28E-05	8.21E-05	4.52E-05
Incidence	Bahamas	Female	55+ years	Non-melanoma skin cancer (basal-cell	Percent	1990	2.19E-05	3.00E-05	1.60E-05
Incidence	Bahamas	Both	55+ years	Non-melanoma skin cancer (basal-cell	Percent	1990	3.93E-05	5.16E-05	2.88E-05
Incidence	Bahamas	Male	55+ years	Non-melanoma skin cancer (basal-cell	Rate	1990	23.780994	30.79296454	17.42663778
Incidence	Bahamas	Female	55+ years	Non-melanoma skin cancer (basal-cell	Rate	1990	8.8063211	11.73197291	6.381025829
Incidence	Bahamas	Both	55+ years	Non-melanoma skin cancer (basal-cell	Rate	1990	15.387783	19.96538813	11.26742793
Incidence	Bahamas	Male	55+ years	Non-melanoma skin cancer (basal-cell	Number	2021	7.551514	9.780401341	5.552824465
Incidence	Bahamas	Female	55+ years	Non-melanoma skin cancer (basal-cell	Number	2021	3.2864073	4.377973222	2.378716623
Incidence	Bahamas	Both	55+ years	Non-melanoma skin cancer (basal-cell	Number	2021	10.837921	14.0883421	8.002795653
Incidence	Bahamas	Male	55+ years	Non-melanoma skin cancer (basal-cell	Percent	2021	5.93E-05	7.71E-05	4.22E-05
Incidence	Bahamas	Female	55+ years	Non-melanoma skin cancer (basal-cell	Percent	2021	1.99E-05	2.69E-05	1.42E-05
Incidence	Bahamas	Both	55+ years	Non-melanoma skin cancer (basal-cell	Percent	2021	3.70E-05	4.81E-05	2.67E-05
Incidence	Bahamas	Male	55+ years	Non-melanoma skin cancer (basal-cell	Rate	2021	23.177242	30.01818364	17.04282868
Incidence	Bahamas	Female	55+ years	Non-melanoma skin cancer (basal-cell	Rate	2021	8.346007	11.11809682	6.040877911
Incidence	Bahamas	Both	55+ years	Non-melanoma skin cancer (basal-cell	Rate	2021	15.061331	19.57840169	11.12139007
Incidence	Suriname	Male	55+ years	Non-melanoma skin cancer (basal-cell	Number	1990	4.9570098	6.343829879	3.631502294
Incidence	Suriname	Female	55+ years	Non-melanoma skin cancer (basal-cell	Number	1990	1.8503061	2.374810015	1.368424152
Incidence	Suriname	Both	55+ years	Non-melanoma skin cancer (basal-cell	Number	1990	6.8073159	8.733368721	4.99508798
Incidence	Suriname	Male	55+ years	Non-melanoma skin cancer (basal-cell	Percent	1990	5.99E-05	7.78E-05	4.32E-05
Incidence	Suriname	Female	55+ years	Non-melanoma skin cancer (basal-cell	Percent	1990	2.00E-05	2.57E-05	1.46E-05
Incidence	Suriname	Both	55+ years	Non-melanoma skin cancer (basal-cell	Percent	1990	3.88E-05	5.00E-05	2.81E-05
Incidence	Suriname	Male	55+ years	Non-melanoma skin cancer (basal-cell	Rate	1990	23.330937	29.85822178	17.09223024
Incidence	Suriname	Female	55+ years	Non-melanoma skin cancer (basal-cell	Rate	1990	8.308287	10.66342641	6.144529522
Incidence	Suriname	Both	55+ years	Non-melanoma skin cancer (basal-cell	Rate	1990	15.642846	20.06881207	11.47844379
Incidence	Suriname	Male	55+ years	Non-melanoma skin cancer (basal-cell	Number	2021	12.139038	15.47793651	8.819677478
Incidence	Suriname	Female	55+ years	Non-melanoma skin cancer (basal-cell	Number	2021	5.2104857	6.801187434	3.786377638
Incidence	Suriname	Both	55+ years	Non-melanoma skin cancer (basal-cell	Number	2021	17.349524	22.15871122	12.86107999
Incidence	Suriname	Male	55+ years	Non-melanoma skin cancer (basal-cell	Percent	2021	5.37E-05	7.06E-05	3.79E-05
Incidence	Suriname	Female	55+ years	Non-melanoma skin cancer (basal-cell	Percent	2021	1.89E-05	2.49E-05	1.36E-05
Incidence	Suriname	Both	55+ years	Non-melanoma skin cancer (basal-cell	Percent	2021	3.45E-05	4.52E-05	2.46E-05
Incidence	Suriname	Male	55+ years	Non-melanoma skin cancer (basal-cell	Rate	2021	23.439223	29.88628859	17.02988162
Incidence	Suriname	Female	55+ years	Non-melanoma skin cancer (basal-cell	Rate	2021	8.6967133	11.3517204	6.319764113
Incidence	Suriname	Both	55+ years	Non-melanoma skin cancer (basal-cell	Rate	2021	15.531876	19.83722113	11.51366996
Incidence	Ecuador	Male	55+ years	Non-melanoma skin cancer (basal-cell	Number	1990	446.40556	492.1245183	403.3175801
Incidence	Ecuador	Female	55+ years	Non-melanoma skin cancer (basal-cell	Number	1990	461.02931	513.452852	408.854839
Incidence	Ecuador	Both	55+ years	Non-melanoma skin cancer (basal-cell	Number	1990	907.43487	996.0799735	819.569891
Incidence	Ecuador	Male	55+ years	Non-melanoma skin cancer (basal-cell	Percent	1990	0.0002434	0.000275083	0.000213221
Incidence	Ecuador	Female	55+ years	Non-melanoma skin cancer (basal-cell	Percent	1990	0.0002073	0.000233147	0.000182291
Incidence	Ecuador	Both	55+ years	Non-melanoma skin cancer (basal-cell	Percent	1990	0.0002236	0.00024952	0.000198007
Incidence	Ecuador	Male	55+ years	Non-melanoma skin cancer (basal-cell	Rate	1990	105.80039	116.6360166	95.58832007
Incidence	Ecuador	Female	55+ years	Non-melanoma skin cancer (basal-cell	Rate	1990	105.27999	117.2513504	93.3654995
Incidence	Ecuador	Both	55+ years	Non-melanoma skin cancer (basal-cell	Rate	1990	105.53535	115.8448457	95.3165911
Incidence	Ecuador	Male	55+ years	Non-melanoma skin cancer (basal-cell	Number	2021	975.68607	1268.416357	726.3282779
Incidence	Ecuador	Female	55+ years	Non-melanoma skin cancer (basal-cell	Number	2021	867.2582	1105.400537	675.0335842
Incidence	Ecuador	Both	55+ years	Non-melanoma skin cancer (basal-cell	Number	2021	1842.9443	2353.635481	1411.919521
Incidence	Ecuador	Male	55+ years	Non-melanoma skin cancer (basal-cell	Percent	2021	0.0001687	0.000221199	0.000123306
Incidence	Ecuador	Female	55+ years	Non-melanoma skin cancer (basal-cell	Percent	2021	0.0001251	0.000159255	9.40E-05
Incidence	Ecuador	Both	55+ years	Non-melanoma skin cancer (basal-cell	Percent	2021	0.0001449	0.000187406	0.000107608
Incidence	Ecuador	Male	55+ years	Non-melanoma skin cancer (basal-cell	Rate	2021	74.17934	96.43500265	55.22119689
Incidence	Ecuador	Female	55+ years	Non-melanoma skin cancer (basal-cell	Rate	2021	59.719785	76.11837218	46.48311257
Incidence	Ecuador	Both	55+ years	Non-melanoma skin cancer (basal-cell	Rate	2021	66.591915	85.04494522	51.01750857
Incidence	Mexico	Male	55+ years	Non-melanoma skin cancer (basal-cell	Number	1990	4374.1157	5512.278777	3357.293222
Incidence	Mexico	Female	55+ years	Non-melanoma skin cancer (basal-cell	Number	1990	4139.0904	5187.344272	3119.00301
Incidence	Mexico	Both	55+ years	Non-melanoma skin cancer (basal-cell	Number	1990	8513.2061	10648.55525	6509.98283
Incidence	Mexico	Male	55+ years	Non-melanoma skin cancer (basal-cell	Percent	1990	0.0003136	0.000393171	0.000235601
Incidence	Mexico	Female	55+ years	Non-melanoma skin cancer (basal-cell	Percent	1990	0.0002544	0.000321037	0.000188978

Incidence	Mexico	Both	55+ years	Non-melanoma skin cancer (basal-cell	Percent	1990	0.0002817	0.00035425	0.000210718
Incidence	Mexico	Male	55+ years	Non-melanoma skin cancer (basal-cell	Rate	1990	130.91194	164.9757658	100.4796823
Incidence	Mexico	Female	55+ years	Non-melanoma skin cancer (basal-cell	Rate	1990	114.60945	143.6351024	86.36371393
Incidence	Mexico	Both	55+ years	Non-melanoma skin cancer (basal-cell	Rate	1990	122.44391	153.1562482	93.63190803
Incidence	Mexico	Male	55+ years	Non-melanoma skin cancer (basal-cell	Number	2021	13510.676	16885.45485	10523.74522
Incidence	Mexico	Female	55+ years	Non-melanoma skin cancer (basal-cell	Number	2021	13611.992	17019.99096	10432.11272
Incidence	Mexico	Both	55+ years	Non-melanoma skin cancer (basal-cell	Number	2021	27122.667	33726.51555	20943.39254
Incidence	Mexico	Male	55+ years	Non-melanoma skin cancer (basal-cell	Percent	2021	0.0003308	0.00041556	0.000250454
Incidence	Mexico	Female	55+ years	Non-melanoma skin cancer (basal-cell	Percent	2021	0.0002741	0.000346896	0.000206136
Incidence	Mexico	Both	55+ years	Non-melanoma skin cancer (basal-cell	Percent	2021	0.0002997	0.00037902	0.000225589
Incidence	Mexico	Male	55+ years	Non-melanoma skin cancer (basal-cell	Rate	2021	134.96864	168.6819305	105.1298692
Incidence	Mexico	Female	55+ years	Non-melanoma skin cancer (basal-cell	Rate	2021	117.95271	147.4842281	90.39793826
Incidence	Mexico	Both	55+ years	Non-melanoma skin cancer (basal-cell	Rate	2021	125.85665	156.5003263	97.18311285
Incidence	Nicaragua	Male	55+ years	Non-melanoma skin cancer (basal-cell	Number	1990	142.84722	181.1765614	108.3463955
Incidence	Nicaragua	Female	55+ years	Non-melanoma skin cancer (basal-cell	Number	1990	146.81663	186.7826423	113.2721577
Incidence	Nicaragua	Both	55+ years	Non-melanoma skin cancer (basal-cell	Number	1990	289.66385	362.0185189	220.0440129
Incidence	Nicaragua	Male	55+ years	Non-melanoma skin cancer (basal-cell	Percent	1990	0.0003223	0.000409988	0.000238872
Incidence	Nicaragua	Female	55+ years	Non-melanoma skin cancer (basal-cell	Percent	1990	0.0002852	0.000359274	0.000217854
Incidence	Nicaragua	Both	55+ years	Non-melanoma skin cancer (basal-cell	Percent	1990	0.0003023	0.00038048	0.0002288
Incidence	Nicaragua	Male	55+ years	Non-melanoma skin cancer (basal-cell	Rate	1990	121.9979	154.7328611	92.53265229
Incidence	Nicaragua	Female	55+ years	Non-melanoma skin cancer (basal-cell	Rate	1990	111.74562	142.164708	86.2141311
Incidence	Nicaragua	Both	55+ years	Non-melanoma skin cancer (basal-cell	Rate	1990	116.57685	145.6963987	88.55795645
Incidence	Nicaragua	Male	55+ years	Non-melanoma skin cancer (basal-cell	Number	2021	461.05327	576.2255291	354.8666858
Incidence	Nicaragua	Female	55+ years	Non-melanoma skin cancer (basal-cell	Number	2021	502.91854	649.9752555	385.6109681
Incidence	Nicaragua	Both	55+ years	Non-melanoma skin cancer (basal-cell	Number	2021	963.97181	1207.814646	739.6060948
Incidence	Nicaragua	Male	55+ years	Non-melanoma skin cancer (basal-cell	Percent	2021	0.0003318	0.000416915	0.000247582
Incidence	Nicaragua	Female	55+ years	Non-melanoma skin cancer (basal-cell	Percent	2021	0.0002838	0.000366853	0.000211068
Incidence	Nicaragua	Both	55+ years	Non-melanoma skin cancer (basal-cell	Percent	2021	0.0003049	0.000383332	0.000229534
Incidence	Nicaragua	Male	55+ years	Non-melanoma skin cancer (basal-cell	Rate	2021	126.07306	157.5664237	97.03678814
Incidence	Nicaragua	Female	55+ years	Non-melanoma skin cancer (basal-cell	Rate	2021	112.1759	144.9768792	86.0104662
Incidence	Nicaragua	Both	55+ years	Non-melanoma skin cancer (basal-cell	Rate	2021	118.41918	148.3740654	90.85695678
Incidence	Panama	Male	55+ years	Non-melanoma skin cancer (basal-cell	Number	1990	109.84174	114.6930808	104.6042998
Incidence	Panama	Female	55+ years	Non-melanoma skin cancer (basal-cell	Number	1990	87.102357	92.90577499	81.46637274
Incidence	Panama	Both	55+ years	Non-melanoma skin cancer (basal-cell	Number	1990	196.9441	205.2218689	187.9555404
Incidence	Panama	Male	55+ years	Non-melanoma skin cancer (basal-cell	Percent	1990	0.0002304	0.000249795	0.000211093
Incidence	Panama	Female	55+ years	Non-melanoma skin cancer (basal-cell	Percent	1990	0.0001766	0.000191877	0.000162046
Incidence	Panama	Both	55+ years	Non-melanoma skin cancer (basal-cell	Percent	1990	0.000203	0.000218059	0.000187621
Incidence	Panama	Male	55+ years	Non-melanoma skin cancer (basal-cell	Rate	1990	89.577194	93.53351872	85.30600246
Incidence	Panama	Female	55+ years	Non-melanoma skin cancer (basal-cell	Rate	1990	72.786763	77.63636702	68.07707287
Incidence	Panama	Both	55+ years	Non-melanoma skin cancer (basal-cell	Rate	1990	81.284353	84.70082175	77.57452367
Incidence	Panama	Male	55+ years	Non-melanoma skin cancer (basal-cell	Number	2021	321.25736	353.7088978	293.6932595
Incidence	Panama	Female	55+ years	Non-melanoma skin cancer (basal-cell	Number	2021	318.7575	349.1354777	292.0720502
Incidence	Panama	Both	55+ years	Non-melanoma skin cancer (basal-cell	Number	2021	640.01485	699.8134489	587.1118146
Incidence	Panama	Male	55+ years	Non-melanoma skin cancer (basal-cell	Percent	2021	0.0002266	0.000253146	0.000202339
Incidence	Panama	Female	55+ years	Non-melanoma skin cancer (basal-cell	Percent	2021	0.0001989	0.000220035	0.000177902
Incidence	Panama	Both	55+ years	Non-melanoma skin cancer (basal-cell	Percent	2021	0.0002119	0.000234572	0.000190198
Incidence	Panama	Male	55+ years	Non-melanoma skin cancer (basal-cell	Rate	2021	89.109081	98.11036046	81.46346257
Incidence	Panama	Female	55+ years	Non-melanoma skin cancer (basal-cell	Rate	2021	83.217106	91.14779801	76.25041262
Incidence	Panama	Both	55+ years	Non-melanoma skin cancer (basal-cell	Rate	2021	86.073863	94.11601382	78.95907652
Incidence	Venezuela	Male	55+ years	Non-melanoma skin cancer (basal-cell	Number	1990	945.65295	1199.255809	700.6538523
Incidence	Venezuela	Female	55+ years	Non-melanoma skin cancer (basal-cell	Number	1990	929.16248	1192.168661	710.9784547
Incidence	Venezuela	Both	55+ years	Non-melanoma skin cancer (basal-cell	Number	1990	1874.8154	2394.873647	1418.705583
Incidence	Venezuela	Male	55+ years	Non-melanoma skin cancer (basal-cell	Percent	1990	0.0003225	0.00041255	0.000238214
Incidence	Venezuela	Female	55+ years	Non-melanoma skin cancer (basal-cell	Percent	1990	0.0002711	0.000349666	0.000200867
Incidence	Venezuela	Both	55+ years	Non-melanoma skin cancer (basal-cell	Percent	1990	0.0002948	0.000373506	0.000216955
Incidence	Venezuela	Male	55+ years	Non-melanoma skin cancer (basal-cell	Rate	1990	127.40769	161.5755663	94.39899484

Incidence	Venezuela	Female	55+ years	Non-melanoma skin cancer (basal-cell	Rate	1990	112.81266	144.7451025	86.32222328
Incidence	Venezuela	Both	55+ years	Non-melanoma skin cancer (basal-cell	Rate	1990	119.73079	152.9431101	90.60237664
Incidence	Venezuela	Male	55+ years	Non-melanoma skin cancer (basal-cell	Number	2021	3070.5105	3934.371574	2343.911963
Incidence	Venezuela	Female	55+ years	Non-melanoma skin cancer (basal-cell	Number	2021	3246.1007	4067.453874	2510.270885
Incidence	Venezuela	Both	55+ years	Non-melanoma skin cancer (basal-cell	Number	2021	6316.6112	8001.249736	4839.715764
Incidence	Venezuela	Male	55+ years	Non-melanoma skin cancer (basal-cell	Percent	2021	0.0003055	0.000398241	0.000227116
Incidence	Venezuela	Female	55+ years	Non-melanoma skin cancer (basal-cell	Percent	2021	0.0002602	0.000327907	0.000196077
Incidence	Venezuela	Both	55+ years	Non-melanoma skin cancer (basal-cell	Percent	2021	0.0002804	0.000354941	0.000211163
Incidence	Venezuela	Male	55+ years	Non-melanoma skin cancer (basal-cell	Rate	2021	127.48917	163.3571262	97.32042721
Incidence	Venezuela	Female	55+ years	Non-melanoma skin cancer (basal-cell	Rate	2021	114.95746	144.0448795	88.8987751
Incidence	Venezuela	Both	55+ years	Non-melanoma skin cancer (basal-cell	Rate	2021	120.72598	152.9235676	92.49887522
Incidence	Guatemala	Male	55+ years	Non-melanoma skin cancer (basal-cell	Number	1990	348.03599	448.2099887	269.908553
Incidence	Guatemala	Female	55+ years	Non-melanoma skin cancer (basal-cell	Number	1990	310.82883	393.3605475	234.0540689
Incidence	Guatemala	Both	55+ years	Non-melanoma skin cancer (basal-cell	Number	1990	658.86482	838.9624493	500.0143901
Incidence	Guatemala	Male	55+ years	Non-melanoma skin cancer (basal-cell	Percent	1990	0.0002727	0.000351814	0.000207058
Incidence	Guatemala	Female	55+ years	Non-melanoma skin cancer (basal-cell	Percent	1990	0.0002254	0.000287669	0.000168563
Incidence	Guatemala	Both	55+ years	Non-melanoma skin cancer (basal-cell	Percent	1990	0.0002481	0.000315826	0.000185795
Incidence	Guatemala	Male	55+ years	Non-melanoma skin cancer (basal-cell	Rate	1990	120.88317	155.6765616	93.74720897
Incidence	Guatemala	Female	55+ years	Non-melanoma skin cancer (basal-cell	Rate	1990	108.23609	136.9750895	81.50176023
Incidence	Guatemala	Both	55+ years	Non-melanoma skin cancer (basal-cell	Rate	1990	114.56771	145.8842543	86.9457584
Incidence	Guatemala	Male	55+ years	Non-melanoma skin cancer (basal-cell	Number	2021	1151.9334	1506.054215	884.21421
Incidence	Guatemala	Female	55+ years	Non-melanoma skin cancer (basal-cell	Number	2021	1144.5231	1441.408223	873.1113709
Incidence	Guatemala	Both	55+ years	Non-melanoma skin cancer (basal-cell	Number	2021	2296.4565	2883.485711	1769.463765
Incidence	Guatemala	Male	55+ years	Non-melanoma skin cancer (basal-cell	Percent	2021	0.0003154	0.000412867	0.000240161
Incidence	Guatemala	Female	55+ years	Non-melanoma skin cancer (basal-cell	Percent	2021	0.000257	0.000325204	0.000191546
Incidence	Guatemala	Both	55+ years	Non-melanoma skin cancer (basal-cell	Percent	2021	0.0002833	0.000363042	0.000213762
Incidence	Guatemala	Male	55+ years	Non-melanoma skin cancer (basal-cell	Rate	2021	135.1501	176.6971736	103.7400581
Incidence	Guatemala	Female	55+ years	Non-melanoma skin cancer (basal-cell	Rate	2021	116.97468	147.3174873	89.23535419
Incidence	Guatemala	Both	55+ years	Non-melanoma skin cancer (basal-cell	Rate	2021	125.43644	157.5010011	96.6511862
Incidence	Jordan	Male	55+ years	Non-melanoma skin cancer (basal-cell	Number	1990	33.061143	41.62415334	26.20830207
Incidence	Jordan	Female	55+ years	Non-melanoma skin cancer (basal-cell	Number	1990	23.105154	29.73787658	17.60540241
Incidence	Jordan	Both	55+ years	Non-melanoma skin cancer (basal-cell	Number	1990	56.166297	71.37403058	43.93627368
Incidence	Jordan	Male	55+ years	Non-melanoma skin cancer (basal-cell	Percent	1990	9.81E-05	0.000122664	7.62E-05
Incidence	Jordan	Female	55+ years	Non-melanoma skin cancer (basal-cell	Percent	1990	6.66E-05	8.59E-05	5.01E-05
Incidence	Jordan	Both	55+ years	Non-melanoma skin cancer (basal-cell	Percent	1990	8.21E-05	0.00010314	6.34E-05
Incidence	Jordan	Male	55+ years	Non-melanoma skin cancer (basal-cell	Rate	1990	29.765524	37.47495145	23.5957916
Incidence	Jordan	Female	55+ years	Non-melanoma skin cancer (basal-cell	Rate	1990	22.229352	28.61066046	16.9380685
Incidence	Jordan	Both	55+ years	Non-melanoma skin cancer (basal-cell	Rate	1990	26.122428	33.19540464	20.43435646
Incidence	Jordan	Male	55+ years	Non-melanoma skin cancer (basal-cell	Number	2021	182.25429	238.4609593	131.9297488
Incidence	Jordan	Female	55+ years	Non-melanoma skin cancer (basal-cell	Number	2021	107.52576	142.3143802	77.90734601
Incidence	Jordan	Both	55+ years	Non-melanoma skin cancer (basal-cell	Number	2021	289.78005	382.5169932	210.1622534
Incidence	Jordan	Male	55+ years	Non-melanoma skin cancer (basal-cell	Percent	2021	7.15E-05	9.44E-05	5.09E-05
Incidence	Jordan	Female	55+ years	Non-melanoma skin cancer (basal-cell	Percent	2021	4.26E-05	5.67E-05	3.01E-05
Incidence	Jordan	Both	55+ years	Non-melanoma skin cancer (basal-cell	Percent	2021	5.71E-05	7.50E-05	4.03E-05
Incidence	Jordan	Male	55+ years	Non-melanoma skin cancer (basal-cell	Rate	2021	27.543072	36.03727184	19.93780549
Incidence	Jordan	Female	55+ years	Non-melanoma skin cancer (basal-cell	Rate	2021	18.055315	23.89688798	13.08190442
Incidence	Jordan	Both	55+ years	Non-melanoma skin cancer (basal-cell	Rate	2021	23.048874	30.4250966	16.71613804
Incidence	Kuwait	Male	55+ years	Non-melanoma skin cancer (basal-cell	Number	1990	10.003815	11.83479734	8.170311139
Incidence	Kuwait	Female	55+ years	Non-melanoma skin cancer (basal-cell	Number	1990	5.1992656	6.331806644	4.214967838
Incidence	Kuwait	Both	55+ years	Non-melanoma skin cancer (basal-cell	Number	1990	15.203081	18.02052424	12.38766207
Incidence	Kuwait	Male	55+ years	Non-melanoma skin cancer (basal-cell	Percent	1990	5.29E-05	6.36E-05	4.20E-05
Incidence	Kuwait	Female	55+ years	Non-melanoma skin cancer (basal-cell	Percent	1990	4.30E-05	5.32E-05	3.37E-05
Incidence	Kuwait	Both	55+ years	Non-melanoma skin cancer (basal-cell	Percent	1990	4.90E-05	5.91E-05	3.90E-05
Incidence	Kuwait	Male	55+ years	Non-melanoma skin cancer (basal-cell	Rate	1990	17.389503	20.57227625	14.20234694
Incidence	Kuwait	Female	55+ years	Non-melanoma skin cancer (basal-cell	Rate	1990	15.332622	18.67248327	12.42993049
Incidence	Kuwait	Both	55+ years	Non-melanoma skin cancer (basal-cell	Rate	1990	16.626706	19.70797622	13.54764968

Incidence	Kuwait	Male	55+ years	Non-melanoma skin cancer (basal-cell	Number	2021	51.036929	65.84557928	37.53223552
Incidence	Kuwait	Female	55+ years	Non-melanoma skin cancer (basal-cell	Number	2021	24.167102	31.62078708	17.76655363
Incidence	Kuwait	Both	55+ years	Non-melanoma skin cancer (basal-cell	Number	2021	75.204031	97.20824632	55.09084875
Incidence	Kuwait	Male	55+ years	Non-melanoma skin cancer (basal-cell	Percent	2021	5.12E-05	6.67E-05	3.73E-05
Incidence	Kuwait	Female	55+ years	Non-melanoma skin cancer (basal-cell	Percent	2021	3.13E-05	4.22E-05	2.26E-05
Incidence	Kuwait	Both	55+ years	Non-melanoma skin cancer (basal-cell	Percent	2021	4.25E-05	5.58E-05	3.09E-05
Incidence	Kuwait	Male	55+ years	Non-melanoma skin cancer (basal-cell	Rate	2021	18.764472	24.2090884	13.79927426
Incidence	Kuwait	Female	55+ years	Non-melanoma skin cancer (basal-cell	Rate	2021	12.442086	16.27950862	9.146855271
Incidence	Kuwait	Both	55+ years	Non-melanoma skin cancer (basal-cell	Rate	2021	16.130458	20.85012677	11.81639648
Incidence	Lebanon	Male	55+ years	Non-melanoma skin cancer (basal-cell	Number	1990	75.499729	97.95479771	55.7230737
Incidence	Lebanon	Female	55+ years	Non-melanoma skin cancer (basal-cell	Number	1990	48.463444	61.54377134	36.22926796
Incidence	Lebanon	Both	55+ years	Non-melanoma skin cancer (basal-cell	Number	1990	123.96317	158.7712118	92.20230043
Incidence	Lebanon	Male	55+ years	Non-melanoma skin cancer (basal-cell	Percent	1990	0.0001194	0.000156563	8.60E-05
Incidence	Lebanon	Female	55+ years	Non-melanoma skin cancer (basal-cell	Percent	1990	7.01E-05	8.98E-05	5.22E-05
Incidence	Lebanon	Both	55+ years	Non-melanoma skin cancer (basal-cell	Percent	1990	9.36E-05	0.000122029	6.85E-05
Incidence	Lebanon	Male	55+ years	Non-melanoma skin cancer (basal-cell	Rate	1990	40.190225	52.14356972	29.66266121
Incidence	Lebanon	Female	55+ years	Non-melanoma skin cancer (basal-cell	Rate	1990	25.336773	32.1751907	18.9407243
Incidence	Lebanon	Both	55+ years	Non-melanoma skin cancer (basal-cell	Rate	1990	32.696483	41.87743925	24.31924649
Incidence	Lebanon	Male	55+ years	Non-melanoma skin cancer (basal-cell	Number	2021	215.14637	267.4278447	165.5441806
Incidence	Lebanon	Female	55+ years	Non-melanoma skin cancer (basal-cell	Number	2021	152.19345	192.749206	115.6524867
Incidence	Lebanon	Both	55+ years	Non-melanoma skin cancer (basal-cell	Number	2021	367.33982	463.3726116	281.6937078
Incidence	Lebanon	Male	55+ years	Non-melanoma skin cancer (basal-cell	Percent	2021	0.0001153	0.000147538	8.69E-05
Incidence	Lebanon	Female	55+ years	Non-melanoma skin cancer (basal-cell	Percent	2021	6.34E-05	8.33E-05	4.67E-05
Incidence	Lebanon	Both	55+ years	Non-melanoma skin cancer (basal-cell	Percent	2021	8.61E-05	0.000110149	6.48E-05
Incidence	Lebanon	Male	55+ years	Non-melanoma skin cancer (basal-cell	Rate	2021	48.40471	60.16725814	37.24496025
Incidence	Lebanon	Female	55+ years	Non-melanoma skin cancer (basal-cell	Rate	2021	28.417924	35.99059144	21.59490814
Incidence	Lebanon	Both	55+ years	Non-melanoma skin cancer (basal-cell	Rate	2021	37.482566	47.28154595	28.74342086
Incidence	Bahrain	Male	55+ years	Non-melanoma skin cancer (basal-cell	Number	1990	2.7132712	3.738360527	1.917479085
Incidence	Bahrain	Female	55+ years	Non-melanoma skin cancer (basal-cell	Number	1990	1.5104727	2.183007861	0.942464763
Incidence	Bahrain	Both	55+ years	Non-melanoma skin cancer (basal-cell	Number	1990	4.2237439	5.826501982	2.897120994
Incidence	Bahrain	Male	55+ years	Non-melanoma skin cancer (basal-cell	Percent	1990	5.39E-05	7.45E-05	3.73E-05
Incidence	Bahrain	Female	55+ years	Non-melanoma skin cancer (basal-cell	Percent	1990	3.26E-05	4.67E-05	2.06E-05
Incidence	Bahrain	Both	55+ years	Non-melanoma skin cancer (basal-cell	Percent	1990	4.37E-05	6.08E-05	2.95E-05
Incidence	Bahrain	Male	55+ years	Non-melanoma skin cancer (basal-cell	Rate	1990	17.736222	24.4370678	12.53425561
Incidence	Bahrain	Female	55+ years	Non-melanoma skin cancer (basal-cell	Rate	1990	11.838248	17.10920506	7.386516183
Incidence	Bahrain	Both	55+ years	Non-melanoma skin cancer (basal-cell	Rate	1990	15.054063	20.76653559	10.32577804
Incidence	Bahrain	Male	55+ years	Non-melanoma skin cancer (basal-cell	Number	2021	15.942037	22.93550556	10.47987136
Incidence	Bahrain	Female	55+ years	Non-melanoma skin cancer (basal-cell	Number	2021	7.5297691	10.84318564	4.741493001
Incidence	Bahrain	Both	55+ years	Non-melanoma skin cancer (basal-cell	Number	2021	23.471806	33.69041947	14.99316498
Incidence	Bahrain	Male	55+ years	Non-melanoma skin cancer (basal-cell	Percent	2021	4.20E-05	6.06E-05	2.77E-05
Incidence	Bahrain	Female	55+ years	Non-melanoma skin cancer (basal-cell	Percent	2021	2.61E-05	3.79E-05	1.64E-05
Incidence	Bahrain	Both	55+ years	Non-melanoma skin cancer (basal-cell	Percent	2021	3.51E-05	5.03E-05	2.31E-05
Incidence	Bahrain	Male	55+ years	Non-melanoma skin cancer (basal-cell	Rate	2021	16.449413	23.66545804	10.81340698
Incidence	Bahrain	Female	55+ years	Non-melanoma skin cancer (basal-cell	Rate	2021	11.503415	16.56540416	7.243696688
Incidence	Bahrain	Both	55+ years	Non-melanoma skin cancer (basal-cell	Rate	2021	14.455544	20.74886495	9.233816624
Incidence	Egypt	Male	55+ years	Non-melanoma skin cancer (basal-cell	Number	1990	574.89284	697.6202638	467.4767484
Incidence	Egypt	Female	55+ years	Non-melanoma skin cancer (basal-cell	Number	1990	281.64312	348.1684621	216.1342626
Incidence	Egypt	Both	55+ years	Non-melanoma skin cancer (basal-cell	Number	1990	856.53596	1038.22142	693.9820721
Incidence	Egypt	Male	55+ years	Non-melanoma skin cancer (basal-cell	Percent	1990	7.66E-05	9.37E-05	6.07E-05
Incidence	Egypt	Female	55+ years	Non-melanoma skin cancer (basal-cell	Percent	1990	3.70E-05	4.62E-05	2.81E-05
Incidence	Egypt	Both	55+ years	Non-melanoma skin cancer (basal-cell	Percent	1990	5.67E-05	6.90E-05	4.46E-05
Incidence	Egypt	Male	55+ years	Non-melanoma skin cancer (basal-cell	Rate	1990	25.233037	30.61975538	20.51835995
Incidence	Egypt	Female	55+ years	Non-melanoma skin cancer (basal-cell	Rate	1990	12.842413	15.87584757	9.855328612
Incidence	Egypt	Both	55+ years	Non-melanoma skin cancer (basal-cell	Rate	1990	19.155862	23.21913718	15.52045124
Incidence	Egypt	Male	55+ years	Non-melanoma skin cancer (basal-cell	Number	2021	1280.4365	1687.954351	945.5653896
Incidence	Egypt	Female	55+ years	Non-melanoma skin cancer (basal-cell	Number	2021	689.59015	940.7768862	491.5761334

Incidence	Egypt	Both	55+ years	Non-melanoma skin cancer (basal-cell	Number	2021	1970.0266	2623.658164	1432.181919
Incidence	Egypt	Male	55+ years	Non-melanoma skin cancer (basal-cell	Percent	2021	5.73E-05	7.62E-05	4.15E-05
Incidence	Egypt	Female	55+ years	Non-melanoma skin cancer (basal-cell	Percent	2021	3.24E-05	4.51E-05	2.28E-05
Incidence	Egypt	Both	55+ years	Non-melanoma skin cancer (basal-cell	Percent	2021	4.52E-05	6.10E-05	3.28E-05
Incidence	Egypt	Male	55+ years	Non-melanoma skin cancer (basal-cell	Rate	2021	21.697742	28.60336967	16.0231563
Incidence	Egypt	Female	55+ years	Non-melanoma skin cancer (basal-cell	Rate	2021	13.352235	18.21585485	9.518175485
Incidence	Egypt	Both	55+ years	Non-melanoma skin cancer (basal-cell	Rate	2021	17.802763	23.70950918	12.94236072
Incidence	Iran (Isla	Male	55+ years	Non-melanoma skin cancer (basal-cell	Number	1990	1130.2106	1493.033947	819.6579217
Incidence	Iran (Isla	Female	55+ years	Non-melanoma skin cancer (basal-cell	Number	1990	630.71587	817.2030779	457.7404619
Incidence	Iran (Isla	Both	55+ years	Non-melanoma skin cancer (basal-cell	Number	1990	1760.9265	2323.41821	1271.481403
Incidence	Iran (Isla	Male	55+ years	Non-melanoma skin cancer (basal-cell	Percent	1990	0.0001323	0.000174209	9.33E-05
Incidence	Iran (Isla	Female	55+ years	Non-melanoma skin cancer (basal-cell	Percent	1990	8.03E-05	0.000104514	5.78E-05
Incidence	Iran (Isla	Both	55+ years	Non-melanoma skin cancer (basal-cell	Percent	1990	0.0001074	0.000140552	7.64E-05
Incidence	Iran (Isla	Male	55+ years	Non-melanoma skin cancer (basal-cell	Rate	1990	45.813711	60.52095663	33.22528709
Incidence	Iran (Isla	Female	55+ years	Non-melanoma skin cancer (basal-cell	Rate	1990	29.431938	38.13424031	21.36015545
Incidence	Iran (Isla	Both	55+ years	Non-melanoma skin cancer (basal-cell	Rate	1990	38.198516	50.400246	27.58133477
Incidence	Iran (Isla	Male	55+ years	Non-melanoma skin cancer (basal-cell	Number	2021	2415.6342	3027.173021	1816.093027
Incidence	Iran (Isla	Female	55+ years	Non-melanoma skin cancer (basal-cell	Number	2021	1522.6411	1934.004056	1109.196334
Incidence	Iran (Isla	Both	55+ years	Non-melanoma skin cancer (basal-cell	Number	2021	3938.2753	4946.318374	2924.492261
Incidence	Iran (Isla	Male	55+ years	Non-melanoma skin cancer (basal-cell	Percent	2021	9.53E-05	0.00012182	6.92E-05
Incidence	Iran (Isla	Female	55+ years	Non-melanoma skin cancer (basal-cell	Percent	2021	5.53E-05	7.12E-05	4.04E-05
Incidence	Iran (Isla	Both	55+ years	Non-melanoma skin cancer (basal-cell	Percent	2021	7.45E-05	9.57E-05	5.44E-05
Incidence	Iran (Isla	Male	55+ years	Non-melanoma skin cancer (basal-cell	Rate	2021	37.505769	47.00068113	28.19713596
Incidence	Iran (Isla	Female	55+ years	Non-melanoma skin cancer (basal-cell	Rate	2021	23.245575	29.52569542	16.93367344
Incidence	Iran (Isla	Both	55+ years	Non-melanoma skin cancer (basal-cell	Rate	2021	30.315551	38.07513594	22.51178189
Incidence	Iraq	Male	55+ years	Non-melanoma skin cancer (basal-cell	Number	1990	140.03775	178.4604931	105.9099694
Incidence	Iraq	Female	55+ years	Non-melanoma skin cancer (basal-cell	Number	1990	88.980543	115.0513148	64.93594113
Incidence	Iraq	Both	55+ years	Non-melanoma skin cancer (basal-cell	Number	1990	229.01829	293.6112256	170.6549445
Incidence	Iraq	Male	55+ years	Non-melanoma skin cancer (basal-cell	Percent	1990	6.74E-05	8.83E-05	4.92E-05
Incidence	Iraq	Female	55+ years	Non-melanoma skin cancer (basal-cell	Percent	1990	3.84E-05	5.03E-05	2.80E-05
Incidence	Iraq	Both	55+ years	Non-melanoma skin cancer (basal-cell	Percent	1990	5.21E-05	6.79E-05	3.80E-05
Incidence	Iraq	Male	55+ years	Non-melanoma skin cancer (basal-cell	Rate	1990	22.875987	29.15256779	17.30101441
Incidence	Iraq	Female	55+ years	Non-melanoma skin cancer (basal-cell	Rate	1990	13.686702	17.69682445	9.988238309
Incidence	Iraq	Both	55+ years	Non-melanoma skin cancer (basal-cell	Rate	1990	18.143159	23.26030458	13.51953073
Incidence	Iraq	Male	55+ years	Non-melanoma skin cancer (basal-cell	Number	2021	384.38246	424.546143	343.2601484
Incidence	Iraq	Female	55+ years	Non-melanoma skin cancer (basal-cell	Number	2021	263.38978	297.9991894	228.4539205
Incidence	Iraq	Both	55+ years	Non-melanoma skin cancer (basal-cell	Number	2021	647.77225	712.9350068	576.7475523
Incidence	Iraq	Male	55+ years	Non-melanoma skin cancer (basal-cell	Percent	2021	4.86E-05	5.54E-05	4.26E-05
Incidence	Iraq	Female	55+ years	Non-melanoma skin cancer (basal-cell	Percent	2021	3.01E-05	3.46E-05	2.57E-05
Incidence	Iraq	Both	55+ years	Non-melanoma skin cancer (basal-cell	Percent	2021	3.89E-05	4.39E-05	3.39E-05
Incidence	Iraq	Male	55+ years	Non-melanoma skin cancer (basal-cell	Rate	2021	20.220768	22.33361293	18.05749367
Incidence	Iraq	Female	55+ years	Non-melanoma skin cancer (basal-cell	Rate	2021	13.273936	15.01813072	11.51328917
Incidence	Iraq	Both	55+ years	Non-melanoma skin cancer (basal-cell	Rate	2021	16.672851	18.35005885	14.84476344
Incidence	Libya	Male	55+ years	Non-melanoma skin cancer (basal-cell	Number	1990	30.929547	40.52952447	22.92874229
Incidence	Libya	Female	55+ years	Non-melanoma skin cancer (basal-cell	Number	1990	15.263711	20.46502792	10.82794067
Incidence	Libya	Both	55+ years	Non-melanoma skin cancer (basal-cell	Number	1990	46.193259	60.59073139	34.10934801
Incidence	Libya	Male	55+ years	Non-melanoma skin cancer (basal-cell	Percent	1990	5.68E-05	7.46E-05	4.09E-05
Incidence	Libya	Female	55+ years	Non-melanoma skin cancer (basal-cell	Percent	1990	2.89E-05	3.86E-05	2.04E-05
Incidence	Libya	Both	55+ years	Non-melanoma skin cancer (basal-cell	Percent	1990	4.31E-05	5.69E-05	3.11E-05
Incidence	Libya	Male	55+ years	Non-melanoma skin cancer (basal-cell	Rate	1990	18.820196	24.66164732	13.95181817
Incidence	Libya	Female	55+ years	Non-melanoma skin cancer (basal-cell	Rate	1990	10.405526	13.95134969	7.381587131
Incidence	Libya	Both	55+ years	Non-melanoma skin cancer (basal-cell	Rate	1990	14.851665	19.48061843	10.96654849
Incidence	Libya	Male	55+ years	Non-melanoma skin cancer (basal-cell	Number	2021	76.980571	98.79874278	56.12815527
Incidence	Libya	Female	55+ years	Non-melanoma skin cancer (basal-cell	Number	2021	41.15635	54.71752354	28.05364361
Incidence	Libya	Both	55+ years	Non-melanoma skin cancer (basal-cell	Number	2021	118.13692	152.7881025	84.13127583
Incidence	Libya	Male	55+ years	Non-melanoma skin cancer (basal-cell	Percent	2021	4.27E-05	5.58E-05	3.06E-05

Incidence	Libya	Female	55+ years	Non-melanoma skin cancer (basal-cell	Percent	2021	2.16E-05	2.91E-05	1.46E-05
Incidence	Libya	Both	55+ years	Non-melanoma skin cancer (basal-cell	Percent	2021	3.19E-05	4.20E-05	2.29E-05
Incidence	Libya	Male	55+ years	Non-melanoma skin cancer (basal-cell	Rate	2021	18.31457	23.50536626	13.35353882
Incidence	Libya	Female	55+ years	Non-melanoma skin cancer (basal-cell	Rate	2021	9.8935367	13.15349463	6.743789316
Incidence	Libya	Both	55+ years	Non-melanoma skin cancer (basal-cell	Rate	2021	14.125863	18.26917253	10.05974135
Incidence	Palestine	Male	55+ years	Non-melanoma skin cancer (basal-cell	Number	1990	16.470616	21.49234073	12.43707819
Incidence	Palestine	Female	55+ years	Non-melanoma skin cancer (basal-cell	Number	1990	11.389279	14.76112191	8.268297951
Incidence	Palestine	Both	55+ years	Non-melanoma skin cancer (basal-cell	Number	1990	27.859896	36.02223019	20.73075054
Incidence	Palestine	Male	55+ years	Non-melanoma skin cancer (basal-cell	Percent	1990	7.15E-05	9.38E-05	5.20E-05
Incidence	Palestine	Female	55+ years	Non-melanoma skin cancer (basal-cell	Percent	1990	3.95E-05	5.23E-05	2.83E-05
Incidence	Palestine	Both	55+ years	Non-melanoma skin cancer (basal-cell	Percent	1990	5.37E-05	6.98E-05	3.92E-05
Incidence	Palestine	Male	55+ years	Non-melanoma skin cancer (basal-cell	Rate	1990	25.239428	32.93467412	19.05846936
Incidence	Palestine	Female	55+ years	Non-melanoma skin cancer (basal-cell	Rate	1990	14.256982	18.47781902	10.35016945
Incidence	Palestine	Both	55+ years	Non-melanoma skin cancer (basal-cell	Rate	1990	19.194776	24.81842143	14.28297195
Incidence	Palestine	Male	55+ years	Non-melanoma skin cancer (basal-cell	Number	2021	47.852726	61.49265824	34.58449472
Incidence	Palestine	Female	55+ years	Non-melanoma skin cancer (basal-cell	Number	2021	30.383078	40.06068462	21.94763281
Incidence	Palestine	Both	55+ years	Non-melanoma skin cancer (basal-cell	Number	2021	78.235805	100.4781613	56.50981602
Incidence	Palestine	Male	55+ years	Non-melanoma skin cancer (basal-cell	Percent	2021	5.48E-05	7.17E-05	3.93E-05
Incidence	Palestine	Female	55+ years	Non-melanoma skin cancer (basal-cell	Percent	2021	3.18E-05	4.22E-05	2.27E-05
Incidence	Palestine	Both	55+ years	Non-melanoma skin cancer (basal-cell	Percent	2021	4.27E-05	5.62E-05	3.06E-05
Incidence	Palestine	Male	55+ years	Non-melanoma skin cancer (basal-cell	Rate	2021	22.513138	28.93027839	16.2708702
Incidence	Palestine	Female	55+ years	Non-melanoma skin cancer (basal-cell	Rate	2021	13.956027	18.40129502	10.08132712
Incidence	Palestine	Both	55+ years	Non-melanoma skin cancer (basal-cell	Rate	2021	18.183359	23.35286886	13.13386219
Incidence	Morocco	Male	55+ years	Non-melanoma skin cancer (basal-cell	Number	1990	191.16026	255.4607	142.7092828
Incidence	Morocco	Female	55+ years	Non-melanoma skin cancer (basal-cell	Number	1990	117.76496	154.8051453	85.44053688
Incidence	Morocco	Both	55+ years	Non-melanoma skin cancer (basal-cell	Number	1990	308.92522	404.658599	229.5953109
Incidence	Morocco	Male	55+ years	Non-melanoma skin cancer (basal-cell	Percent	1990	4.51E-05	6.03E-05	3.26E-05
Incidence	Morocco	Female	55+ years	Non-melanoma skin cancer (basal-cell	Percent	1990	2.68E-05	3.56E-05	1.91E-05
Incidence	Morocco	Both	55+ years	Non-melanoma skin cancer (basal-cell	Percent	1990	3.58E-05	4.76E-05	2.56E-05
Incidence	Morocco	Male	55+ years	Non-melanoma skin cancer (basal-cell	Rate	1990	16.238123	21.70012896	12.12245109
Incidence	Morocco	Female	55+ years	Non-melanoma skin cancer (basal-cell	Rate	1990	10.085984	13.25829164	7.317557528
Incidence	Morocco	Both	55+ years	Non-melanoma skin cancer (basal-cell	Rate	1990	13.174675	17.25739815	9.791507467
Incidence	Morocco	Male	55+ years	Non-melanoma skin cancer (basal-cell	Number	2021	464.16844	606.2777375	335.2134446
Incidence	Morocco	Female	55+ years	Non-melanoma skin cancer (basal-cell	Number	2021	300.10979	399.6483767	214.174166
Incidence	Morocco	Both	55+ years	Non-melanoma skin cancer (basal-cell	Number	2021	764.27823	1006.877275	554.8684407
Incidence	Morocco	Male	55+ years	Non-melanoma skin cancer (basal-cell	Percent	2021	3.88E-05	5.11E-05	2.76E-05
Incidence	Morocco	Female	55+ years	Non-melanoma skin cancer (basal-cell	Percent	2021	2.33E-05	3.16E-05	1.62E-05
Incidence	Morocco	Both	55+ years	Non-melanoma skin cancer (basal-cell	Percent	2021	3.08E-05	4.05E-05	2.20E-05
Incidence	Morocco	Male	55+ years	Non-melanoma skin cancer (basal-cell	Rate	2021	15.504008	20.25069815	11.19669396
Incidence	Morocco	Female	55+ years	Non-melanoma skin cancer (basal-cell	Rate	2021	9.9567842	13.2591898	7.105686106
Incidence	Morocco	Both	55+ years	Non-melanoma skin cancer (basal-cell	Rate	2021	12.721042	16.75898569	9.235517063
Incidence	Oman	Male	55+ years	Non-melanoma skin cancer (basal-cell	Number	1990	13.66818	16.93488554	10.97602214
Incidence	Oman	Female	55+ years	Non-melanoma skin cancer (basal-cell	Number	1990	8.5888433	10.72891618	6.637229969
Incidence	Oman	Both	55+ years	Non-melanoma skin cancer (basal-cell	Number	1990	22.257024	27.46865974	17.80743133
Incidence	Oman	Male	55+ years	Non-melanoma skin cancer (basal-cell	Percent	1990	7.11E-05	8.83E-05	5.49E-05
Incidence	Oman	Female	55+ years	Non-melanoma skin cancer (basal-cell	Percent	1990	4.91E-05	6.18E-05	3.71E-05
Incidence	Oman	Both	55+ years	Non-melanoma skin cancer (basal-cell	Percent	1990	6.06E-05	7.53E-05	4.66E-05
Incidence	Oman	Male	55+ years	Non-melanoma skin cancer (basal-cell	Rate	1990	25.1738	31.19035604	20.21543267
Incidence	Oman	Female	55+ years	Non-melanoma skin cancer (basal-cell	Rate	1990	18.585175	23.21602324	14.36212964
Incidence	Oman	Both	55+ years	Non-melanoma skin cancer (basal-cell	Rate	1990	22.144381	27.32964129	17.7173082
Incidence	Oman	Male	55+ years	Non-melanoma skin cancer (basal-cell	Number	2021	38.468572	45.91999958	31.40777327
Incidence	Oman	Female	55+ years	Non-melanoma skin cancer (basal-cell	Number	2021	26.342852	31.95147761	21.43258604
Incidence	Oman	Both	55+ years	Non-melanoma skin cancer (basal-cell	Number	2021	64.811424	76.87135386	53.07407909
Incidence	Oman	Male	55+ years	Non-melanoma skin cancer (basal-cell	Percent	2021	5.70E-05	6.92E-05	4.59E-05
Incidence	Oman	Female	55+ years	Non-melanoma skin cancer (basal-cell	Percent	2021	4.36E-05	5.25E-05	3.49E-05
Incidence	Oman	Both	55+ years	Non-melanoma skin cancer (basal-cell	Percent	2021	5.07E-05	6.09E-05	4.06E-05

Incidence	Oman	Male	55+ years	Non-melanoma skin cancer (basal-cell	Rate	2021	21.873587	26.11053778	17.85875126
Incidence	Oman	Female	55+ years	Non-melanoma skin cancer (basal-cell	Rate	2021	19.300649	23.40992724	15.7030384
Incidence	Oman	Both	55+ years	Non-melanoma skin cancer (basal-cell	Rate	2021	20.749312	24.61028686	16.9916132
Incidence	Algeria	Male	55+ years	Non-melanoma skin cancer (basal-cell	Number	1990	316.46234	360.5801018	271.4890311
Incidence	Algeria	Female	55+ years	Non-melanoma skin cancer (basal-cell	Number	1990	190.59159	222.4022729	161.1837827
Incidence	Algeria	Both	55+ years	Non-melanoma skin cancer (basal-cell	Number	1990	507.05393	578.3652489	434.8183291
Incidence	Algeria	Male	55+ years	Non-melanoma skin cancer (basal-cell	Percent	1990	9.43E-05	0.000108956	7.83E-05
Incidence	Algeria	Female	55+ years	Non-melanoma skin cancer (basal-cell	Percent	1990	5.12E-05	6.11E-05	4.18E-05
Incidence	Algeria	Both	55+ years	Non-melanoma skin cancer (basal-cell	Percent	1990	7.16E-05	8.29E-05	5.90E-05
Incidence	Algeria	Male	55+ years	Non-melanoma skin cancer (basal-cell	Rate	1990	30.887762	35.19380044	26.49821976
Incidence	Algeria	Female	55+ years	Non-melanoma skin cancer (basal-cell	Rate	1990	17.80438	20.77601933	15.05720846
Incidence	Algeria	Both	55+ years	Non-melanoma skin cancer (basal-cell	Rate	1990	24.202686	27.60651666	20.75473841
Incidence	Algeria	Male	55+ years	Non-melanoma skin cancer (basal-cell	Number	2021	863.57448	1095.174396	657.1349161
Incidence	Algeria	Female	55+ years	Non-melanoma skin cancer (basal-cell	Number	2021	517.98105	678.5883375	390.8850607
Incidence	Algeria	Both	55+ years	Non-melanoma skin cancer (basal-cell	Number	2021	1381.5555	1754.627303	1048.07956
Incidence	Algeria	Male	55+ years	Non-melanoma skin cancer (basal-cell	Percent	2021	8.19E-05	0.00010509	6.09E-05
Incidence	Algeria	Female	55+ years	Non-melanoma skin cancer (basal-cell	Percent	2021	4.65E-05	6.14E-05	3.43E-05
Incidence	Algeria	Both	55+ years	Non-melanoma skin cancer (basal-cell	Percent	2021	6.37E-05	8.19E-05	4.76E-05
Incidence	Algeria	Male	55+ years	Non-melanoma skin cancer (basal-cell	Rate	2021	27.939599	35.43265138	21.26057043
Incidence	Algeria	Female	55+ years	Non-melanoma skin cancer (basal-cell	Rate	2021	17.367297	22.75227133	13.10591778
Incidence	Algeria	Both	55+ years	Non-melanoma skin cancer (basal-cell	Rate	2021	22.747757	28.8905046	17.25696807
Incidence	Qatar	Male	55+ years	Non-melanoma skin cancer (basal-cell	Number	1990	1.7278957	2.481545204	1.135792781
Incidence	Qatar	Female	55+ years	Non-melanoma skin cancer (basal-cell	Number	1990	0.5726223	0.828459435	0.365193327
Incidence	Qatar	Both	55+ years	Non-melanoma skin cancer (basal-cell	Number	1990	2.300518	3.312003082	1.521398649
Incidence	Qatar	Male	55+ years	Non-melanoma skin cancer (basal-cell	Percent	1990	4.78E-05	7.01E-05	3.12E-05
Incidence	Qatar	Female	55+ years	Non-melanoma skin cancer (basal-cell	Percent	1990	3.18E-05	4.55E-05	2.03E-05
Incidence	Qatar	Both	55+ years	Non-melanoma skin cancer (basal-cell	Percent	1990	4.25E-05	6.14E-05	2.76E-05
Incidence	Qatar	Male	55+ years	Non-melanoma skin cancer (basal-cell	Rate	1990	15.252055	21.9044833	10.02558968
Incidence	Qatar	Female	55+ years	Non-melanoma skin cancer (basal-cell	Rate	1990	11.437925	16.54818039	7.294605861
Incidence	Qatar	Both	55+ years	Non-melanoma skin cancer (basal-cell	Rate	1990	14.083121	20.27514717	9.313572708
Incidence	Qatar	Male	55+ years	Non-melanoma skin cancer (basal-cell	Number	2021	15.46567	23.28989609	9.782116135
Incidence	Qatar	Female	55+ years	Non-melanoma skin cancer (basal-cell	Number	2021	5.2425828	7.679275586	3.252854488
Incidence	Qatar	Both	55+ years	Non-melanoma skin cancer (basal-cell	Number	2021	20.708253	30.54423394	12.9312447
Incidence	Qatar	Male	55+ years	Non-melanoma skin cancer (basal-cell	Percent	2021	4.01E-05	6.03E-05	2.56E-05
Incidence	Qatar	Female	55+ years	Non-melanoma skin cancer (basal-cell	Percent	2021	2.55E-05	3.73E-05	1.62E-05
Incidence	Qatar	Both	55+ years	Non-melanoma skin cancer (basal-cell	Percent	2021	3.50E-05	5.23E-05	2.22E-05
Incidence	Qatar	Male	55+ years	Non-melanoma skin cancer (basal-cell	Rate	2021	14.685456	22.11496331	9.288626214
Incidence	Qatar	Female	55+ years	Non-melanoma skin cancer (basal-cell	Rate	2021	10.951424	16.04152078	6.795007195
Incidence	Qatar	Both	55+ years	Non-melanoma skin cancer (basal-cell	Rate	2021	13.518541	19.93956092	8.441637201
Incidence	Saudi Arab	Male	55+ years	Non-melanoma skin cancer (basal-cell	Number	1990	101.86003	134.7375049	73.19130589
Incidence	Saudi Arab	Female	55+ years	Non-melanoma skin cancer (basal-cell	Number	1990	49.517287	64.86921398	36.51154699
Incidence	Saudi Arab	Both	55+ years	Non-melanoma skin cancer (basal-cell	Number	1990	151.37731	195.6067234	109.431082
Incidence	Saudi Arab	Male	55+ years	Non-melanoma skin cancer (basal-cell	Percent	1990	5.60E-05	7.46E-05	3.99E-05
Incidence	Saudi Arab	Female	55+ years	Non-melanoma skin cancer (basal-cell	Percent	1990	3.53E-05	4.68E-05	2.50E-05
Incidence	Saudi Arab	Both	55+ years	Non-melanoma skin cancer (basal-cell	Percent	1990	4.70E-05	6.26E-05	3.34E-05
Incidence	Saudi Arab	Male	55+ years	Non-melanoma skin cancer (basal-cell	Rate	1990	19.521369	25.8223035	14.0270381
Incidence	Saudi Arab	Female	55+ years	Non-melanoma skin cancer (basal-cell	Rate	1990	13.415081	17.5741807	9.8916032
Incidence	Saudi Arab	Both	55+ years	Non-melanoma skin cancer (basal-cell	Rate	1990	16.99143	21.95598479	12.28315229
Incidence	Saudi Arab	Male	55+ years	Non-melanoma skin cancer (basal-cell	Number	2021	301.84039	338.9055329	267.5142062
Incidence	Saudi Arab	Female	55+ years	Non-melanoma skin cancer (basal-cell	Number	2021	133.44013	147.9374547	119.5020361
Incidence	Saudi Arab	Both	55+ years	Non-melanoma skin cancer (basal-cell	Number	2021	435.28052	485.601142	391.727037
Incidence	Saudi Arab	Male	55+ years	Non-melanoma skin cancer (basal-cell	Percent	2021	4.54E-05	5.15E-05	3.87E-05
Incidence	Saudi Arab	Female	55+ years	Non-melanoma skin cancer (basal-cell	Percent	2021	2.70E-05	3.05E-05	2.36E-05
Incidence	Saudi Arab	Both	55+ years	Non-melanoma skin cancer (basal-cell	Percent	2021	3.76E-05	4.23E-05	3.24E-05
Incidence	Saudi Arab	Male	55+ years	Non-melanoma skin cancer (basal-cell	Rate	2021	16.722111	18.77553822	14.82042255
Incidence	Saudi Arab	Female	55+ years	Non-melanoma skin cancer (basal-cell	Rate	2021	11.067765	12.27019889	9.911714074

Incidence	Saudi Arab	Both	55+ years	Non-melanoma skin cancer (basal-cell	Rate	2021	14.457774	16.12916486	13.01115136
Incidence	Paraguay	Male	55+ years	Non-melanoma skin cancer (basal-cell	Number	1990	0.5278017	0.992577242	0.181298572
Incidence	Paraguay	Female	55+ years	Non-melanoma skin cancer (basal-cell	Number	1990	0.4522349	0.842451018	0.171314659
Incidence	Paraguay	Both	55+ years	Non-melanoma skin cancer (basal-cell	Number	1990	0.9800366	1.874055647	0.395622086
Incidence	Paraguay	Male	55+ years	Non-melanoma skin cancer (basal-cell	Percent	1990	6.31E-07	1.19E-06	2.25E-07
Incidence	Paraguay	Female	55+ years	Non-melanoma skin cancer (basal-cell	Percent	1990	4.33E-07	8.01E-07	1.60E-07
Incidence	Paraguay	Both	55+ years	Non-melanoma skin cancer (basal-cell	Percent	1990	5.21E-07	9.85E-07	2.06E-07
Incidence	Paraguay	Male	55+ years	Non-melanoma skin cancer (basal-cell	Rate	1990	0.3045026	0.572643761	0.104595886
Incidence	Paraguay	Female	55+ years	Non-melanoma skin cancer (basal-cell	Rate	1990	0.238723	0.444707965	0.090432549
Incidence	Paraguay	Both	55+ years	Non-melanoma skin cancer (basal-cell	Rate	1990	0.2701525	0.51659386	0.109055428
Incidence	Paraguay	Male	55+ years	Non-melanoma skin cancer (basal-cell	Number	2021	1.0729003	2.022794603	0.428714408
Incidence	Paraguay	Female	55+ years	Non-melanoma skin cancer (basal-cell	Number	2021	0.9509451	1.847993374	0.34007363
Incidence	Paraguay	Both	55+ years	Non-melanoma skin cancer (basal-cell	Number	2021	2.0238454	3.837224263	0.811917035
Incidence	Paraguay	Male	55+ years	Non-melanoma skin cancer (basal-cell	Percent	2021	4.99E-07	9.57E-07	1.96E-07
Incidence	Paraguay	Female	55+ years	Non-melanoma skin cancer (basal-cell	Percent	2021	3.80E-07	7.49E-07	1.35E-07
Incidence	Paraguay	Both	55+ years	Non-melanoma skin cancer (basal-cell	Percent	2021	4.35E-07	8.14E-07	1.73E-07
Incidence	Paraguay	Male	55+ years	Non-melanoma skin cancer (basal-cell	Rate	2021	0.2264413	0.426921455	0.090482434
Incidence	Paraguay	Female	55+ years	Non-melanoma skin cancer (basal-cell	Rate	2021	0.1841862	0.357933187	0.065868006
Incidence	Paraguay	Both	55+ years	Non-melanoma skin cancer (basal-cell	Rate	2021	0.2044071	0.38755724	0.082003111
Incidence	Honduras	Male	55+ years	Non-melanoma skin cancer (basal-cell	Number	1990	208.52104	261.2064508	159.331514
Incidence	Honduras	Female	55+ years	Non-melanoma skin cancer (basal-cell	Number	1990	195.00032	244.5241568	146.5604078
Incidence	Honduras	Both	55+ years	Non-melanoma skin cancer (basal-cell	Number	1990	403.52136	503.7970599	305.8622573
Incidence	Honduras	Male	55+ years	Non-melanoma skin cancer (basal-cell	Percent	1990	0.0003114	0.000391576	0.000234211
Incidence	Honduras	Female	55+ years	Non-melanoma skin cancer (basal-cell	Percent	1990	0.0002665	0.000335581	0.000198609
Incidence	Honduras	Both	55+ years	Non-melanoma skin cancer (basal-cell	Percent	1990	0.0002879	0.000356966	0.000215902
Incidence	Honduras	Male	55+ years	Non-melanoma skin cancer (basal-cell	Rate	1990	127.98959	160.3277402	97.79720791
Incidence	Honduras	Female	55+ years	Non-melanoma skin cancer (basal-cell	Rate	1990	114.43981	143.5038583	86.01188642
Incidence	Honduras	Both	55+ years	Non-melanoma skin cancer (basal-cell	Rate	1990	121.06276	151.1470513	91.76349362
Incidence	Honduras	Male	55+ years	Non-melanoma skin cancer (basal-cell	Number	2021	633.67764	803.449435	483.0511483
Incidence	Honduras	Female	55+ years	Non-melanoma skin cancer (basal-cell	Number	2021	641.75093	817.8192087	473.6541967
Incidence	Honduras	Both	55+ years	Non-melanoma skin cancer (basal-cell	Number	2021	1275.4286	1620.55994	966.295589
Incidence	Honduras	Male	55+ years	Non-melanoma skin cancer (basal-cell	Percent	2021	0.0002828	0.000360589	0.000210091
Incidence	Honduras	Female	55+ years	Non-melanoma skin cancer (basal-cell	Percent	2021	0.0002412	0.000315232	0.000175301
Incidence	Honduras	Both	55+ years	Non-melanoma skin cancer (basal-cell	Percent	2021	0.0002602	0.000331803	0.000191705
Incidence	Honduras	Male	55+ years	Non-melanoma skin cancer (basal-cell	Rate	2021	129.26506	163.8971183	98.53848635
Incidence	Honduras	Female	55+ years	Non-melanoma skin cancer (basal-cell	Rate	2021	114.30167	145.6610349	84.36211789
Incidence	Honduras	Both	55+ years	Non-melanoma skin cancer (basal-cell	Rate	2021	121.27657	154.0940506	91.88206972
Incidence	Brazil	Male	55+ years	Non-melanoma skin cancer (basal-cell	Number	1990	22979.64	26380.13642	19815.06391
Incidence	Brazil	Female	55+ years	Non-melanoma skin cancer (basal-cell	Number	1990	19214.881	21900.68228	16495.76204
Incidence	Brazil	Both	55+ years	Non-melanoma skin cancer (basal-cell	Number	1990	42194.521	48281.10025	36167.18251
Incidence	Brazil	Male	55+ years	Non-melanoma skin cancer (basal-cell	Percent	1990	0.0006807	0.000795664	0.000572226
Incidence	Brazil	Female	55+ years	Non-melanoma skin cancer (basal-cell	Percent	1990	0.0004817	0.000559964	0.000403094
Incidence	Brazil	Both	55+ years	Non-melanoma skin cancer (basal-cell	Percent	1990	0.0005729	0.000670159	0.000480137
Incidence	Brazil	Male	55+ years	Non-melanoma skin cancer (basal-cell	Rate	1990	332.96123	382.2323869	287.1084158
Incidence	Brazil	Female	55+ years	Non-melanoma skin cancer (basal-cell	Rate	1990	243.93729	278.0341487	209.4174555
Incidence	Brazil	Both	55+ years	Non-melanoma skin cancer (basal-cell	Rate	1990	285.51149	326.6966536	244.7271797
Incidence	Brazil	Male	55+ years	Non-melanoma skin cancer (basal-cell	Number	2021	35116.728	43335.74221	27933.60085
Incidence	Brazil	Female	55+ years	Non-melanoma skin cancer (basal-cell	Number	2021	33675.984	41280.51067	26682.04441
Incidence	Brazil	Both	55+ years	Non-melanoma skin cancer (basal-cell	Number	2021	68792.713	84605.09487	54410.47256
Incidence	Brazil	Male	55+ years	Non-melanoma skin cancer (basal-cell	Percent	2021	0.0003646	0.000450457	0.000283877
Incidence	Brazil	Female	55+ years	Non-melanoma skin cancer (basal-cell	Percent	2021	0.00027	0.000329429	0.000212154
Incidence	Brazil	Both	55+ years	Non-melanoma skin cancer (basal-cell	Percent	2021	0.0003112	0.000380982	0.000244312
Incidence	Brazil	Male	55+ years	Non-melanoma skin cancer (basal-cell	Rate	2021	180.13125	222.2906821	143.2854007
Incidence	Brazil	Female	55+ years	Non-melanoma skin cancer (basal-cell	Rate	2021	141.42044	173.355234	112.0497779
Incidence	Brazil	Both	55+ years	Non-melanoma skin cancer (basal-cell	Rate	2021	158.8462	195.357869	125.6368069
Incidence	Türkiye	Male	55+ years	Non-melanoma skin cancer (basal-cell	Number	1990	1641.1799	1860.523666	1407.659831

Incidence	Türkiye	Female	55+ years	Non-melanoma skin cancer (basal-cell	Number	1990	1452.9352	1694.076809	1195.456941
Incidence	Türkiye	Both	55+ years	Non-melanoma skin cancer (basal-cell	Number	1990	3094.1151	3526.72761	2612.026719
Incidence	Türkiye	Male	55+ years	Non-melanoma skin cancer (basal-cell	Percent	1990	0.0001538	0.000178586	0.000127922
Incidence	Türkiye	Female	55+ years	Non-melanoma skin cancer (basal-cell	Percent	1990	0.0001137	0.000136252	9.12E-05
Incidence	Türkiye	Both	55+ years	Non-melanoma skin cancer (basal-cell	Percent	1990	0.0001319	0.00015494	0.000109195
Incidence	Türkiye	Male	55+ years	Non-melanoma skin cancer (basal-cell	Rate	1990	57.624554	65.32607678	49.42527518
Incidence	Türkiye	Female	55+ years	Non-melanoma skin cancer (basal-cell	Rate	1990	46.472119	54.18503205	38.2366799
Incidence	Türkiye	Both	55+ years	Non-melanoma skin cancer (basal-cell	Rate	1990	51.788488	59.0294422	43.71941848
Incidence	Türkiye	Male	55+ years	Non-melanoma skin cancer (basal-cell	Number	2021	3660.1803	4683.517769	2727.153584
Incidence	Türkiye	Female	55+ years	Non-melanoma skin cancer (basal-cell	Number	2021	2822.0831	3537.668702	2148.708111
Incidence	Türkiye	Both	55+ years	Non-melanoma skin cancer (basal-cell	Number	2021	6482.2634	8210.512249	4870.758632
Incidence	Türkiye	Male	55+ years	Non-melanoma skin cancer (basal-cell	Percent	2021	0.0001115	0.00014545	7.96E-05
Incidence	Türkiye	Female	55+ years	Non-melanoma skin cancer (basal-cell	Percent	2021	7.00E-05	8.99E-05	5.19E-05
Incidence	Türkiye	Both	55+ years	Non-melanoma skin cancer (basal-cell	Percent	2021	8.86E-05	0.00011413	6.46E-05
Incidence	Türkiye	Male	55+ years	Non-melanoma skin cancer (basal-cell	Rate	2021	46.953626	60.08123168	34.98454673
Incidence	Türkiye	Female	55+ years	Non-melanoma skin cancer (basal-cell	Rate	2021	32.341286	40.54195123	24.62435767
Incidence	Türkiye	Both	55+ years	Non-melanoma skin cancer (basal-cell	Rate	2021	39.235902	49.69666194	29.48177138
Incidence	United Ara	Male	55+ years	Non-melanoma skin cancer (basal-cell	Number	1990	7.4439355	10.06146257	5.303695513
Incidence	United Ara	Female	55+ years	Non-melanoma skin cancer (basal-cell	Number	1990	3.0966033	4.069155921	2.191287809
Incidence	United Ara	Both	55+ years	Non-melanoma skin cancer (basal-cell	Number	1990	10.540539	13.93423643	7.589755433
Incidence	United Ara	Male	55+ years	Non-melanoma skin cancer (basal-cell	Percent	1990	6.43E-05	8.71E-05	4.52E-05
Incidence	United Ara	Female	55+ years	Non-melanoma skin cancer (basal-cell	Percent	1990	3.71E-05	4.92E-05	2.65E-05
Incidence	United Ara	Both	55+ years	Non-melanoma skin cancer (basal-cell	Percent	1990	5.29E-05	7.06E-05	3.75E-05
Incidence	United Ara	Male	55+ years	Non-melanoma skin cancer (basal-cell	Rate	1990	21.411847	28.94094099	15.25562887
Incidence	United Ara	Female	55+ years	Non-melanoma skin cancer (basal-cell	Rate	1990	14.497087	19.05019849	10.25875354
Incidence	United Ara	Both	55+ years	Non-melanoma skin cancer (basal-cell	Rate	1990	18.780244	24.82684871	13.5227869
Incidence	United Ara	Male	55+ years	Non-melanoma skin cancer (basal-cell	Number	2021	100.23953	138.2543355	69.77488175
Incidence	United Ara	Female	55+ years	Non-melanoma skin cancer (basal-cell	Number	2021	18.168237	26.1041399	12.22047439
Incidence	United Ara	Both	55+ years	Non-melanoma skin cancer (basal-cell	Number	2021	118.40777	165.0056725	82.17645767
Incidence	United Ara	Male	55+ years	Non-melanoma skin cancer (basal-cell	Percent	2021	4.91E-05	6.91E-05	3.35E-05
Incidence	United Ara	Female	55+ years	Non-melanoma skin cancer (basal-cell	Percent	2021	2.45E-05	3.51E-05	1.64E-05
Incidence	United Ara	Both	55+ years	Non-melanoma skin cancer (basal-cell	Percent	2021	4.25E-05	6.03E-05	2.88E-05
Incidence	United Ara	Male	55+ years	Non-melanoma skin cancer (basal-cell	Rate	2021	18.211537	25.11807257	12.67671308
Incidence	United Ara	Female	55+ years	Non-melanoma skin cancer (basal-cell	Rate	2021	10.83629	15.5695911	7.288797487
Incidence	United Ara	Both	55+ years	Non-melanoma skin cancer (basal-cell	Rate	2021	16.489523	22.97876882	11.44393277
Incidence	Syrian Ara	Male	55+ years	Non-melanoma skin cancer (basal-cell	Number	1990	110.60787	141.2453002	83.46044156
Incidence	Syrian Ara	Female	55+ years	Non-melanoma skin cancer (basal-cell	Number	1990	56.838256	74.98981848	42.26909636
Incidence	Syrian Ara	Both	55+ years	Non-melanoma skin cancer (basal-cell	Number	1990	167.44613	214.3898477	125.4466619
Incidence	Syrian Ara	Male	55+ years	Non-melanoma skin cancer (basal-cell	Percent	1990	7.29E-05	9.39E-05	5.29E-05
Incidence	Syrian Ara	Female	55+ years	Non-melanoma skin cancer (basal-cell	Percent	1990	3.90E-05	5.22E-05	2.85E-05
Incidence	Syrian Ara	Both	55+ years	Non-melanoma skin cancer (basal-cell	Percent	1990	5.63E-05	7.33E-05	4.13E-05
Incidence	Syrian Ara	Male	55+ years	Non-melanoma skin cancer (basal-cell	Rate	1990	23.755981	30.33618348	17.92534877
Incidence	Syrian Ara	Female	55+ years	Non-melanoma skin cancer (basal-cell	Rate	1990	13.66063	18.02321651	10.1590468
Incidence	Syrian Ara	Both	55+ years	Non-melanoma skin cancer (basal-cell	Rate	1990	18.991851	24.31623908	14.22824381
Incidence	Syrian Ara	Male	55+ years	Non-melanoma skin cancer (basal-cell	Number	2021	281.96342	361.3070777	208.1982263
Incidence	Syrian Ara	Female	55+ years	Non-melanoma skin cancer (basal-cell	Number	2021	156.34003	211.4144318	110.2806125
Incidence	Syrian Ara	Both	55+ years	Non-melanoma skin cancer (basal-cell	Number	2021	438.30344	560.1073325	320.1543633
Incidence	Syrian Ara	Male	55+ years	Non-melanoma skin cancer (basal-cell	Percent	2021	6.74E-05	8.81E-05	4.84E-05
Incidence	Syrian Ara	Female	55+ years	Non-melanoma skin cancer (basal-cell	Percent	2021	3.62E-05	5.00E-05	2.55E-05
Incidence	Syrian Ara	Both	55+ years	Non-melanoma skin cancer (basal-cell	Percent	2021	5.16E-05	6.80E-05	3.68E-05
Incidence	Syrian Ara	Male	55+ years	Non-melanoma skin cancer (basal-cell	Rate	2021	23.275382	29.82500545	17.18624853
Incidence	Syrian Ara	Female	55+ years	Non-melanoma skin cancer (basal-cell	Rate	2021	13.579671	18.36342524	9.578957156
Incidence	Syrian Ara	Both	55+ years	Non-melanoma skin cancer (basal-cell	Rate	2021	18.55093	23.70620704	13.55034148
Incidence	Tunisia	Male	55+ years	Non-melanoma skin cancer (basal-cell	Number	1990	138.01431	176.6211944	105.9500675
Incidence	Tunisia	Female	55+ years	Non-melanoma skin cancer (basal-cell	Number	1990	79.584352	103.5471855	59.98048608
Incidence	Tunisia	Both	55+ years	Non-melanoma skin cancer (basal-cell	Number	1990	217.59866	279.6130798	165.9812498

Incidence	Tunisia	Male	55+ years	Non-melanoma skin cancer (basal-cell	Percent	1990	9.28E-05	0.000121419	6.74E-05
Incidence	Tunisia	Female	55+ years	Non-melanoma skin cancer (basal-cell	Percent	1990	5.37E-05	7.12E-05	3.96E-05
Incidence	Tunisia	Both	55+ years	Non-melanoma skin cancer (basal-cell	Percent	1990	7.33E-05	9.57E-05	5.42E-05
Incidence	Tunisia	Male	55+ years	Non-melanoma skin cancer (basal-cell	Rate	1990	30.481877	39.00860565	23.4001611
Incidence	Tunisia	Female	55+ years	Non-melanoma skin cancer (basal-cell	Rate	1990	19.041773	24.77524717	14.35124828
Incidence	Tunisia	Both	55+ years	Non-melanoma skin cancer (basal-cell	Rate	1990	24.990627	32.11281869	19.06250517
Incidence	Tunisia	Male	55+ years	Non-melanoma skin cancer (basal-cell	Number	2021	330.44106	425.2028593	247.4657651
Incidence	Tunisia	Female	55+ years	Non-melanoma skin cancer (basal-cell	Number	2021	221.6	284.0050588	164.5453601
Incidence	Tunisia	Both	55+ years	Non-melanoma skin cancer (basal-cell	Number	2021	552.04107	707.6053861	411.7903597
Incidence	Tunisia	Male	55+ years	Non-melanoma skin cancer (basal-cell	Percent	2021	6.98E-05	9.20E-05	5.15E-05
Incidence	Tunisia	Female	55+ years	Non-melanoma skin cancer (basal-cell	Percent	2021	4.13E-05	5.39E-05	3.02E-05
Incidence	Tunisia	Both	55+ years	Non-melanoma skin cancer (basal-cell	Percent	2021	5.46E-05	7.16E-05	4.02E-05
Incidence	Tunisia	Male	55+ years	Non-melanoma skin cancer (basal-cell	Rate	2021	29.000813	37.31748207	21.7185728
Incidence	Tunisia	Female	55+ years	Non-melanoma skin cancer (basal-cell	Rate	2021	18.501908	23.71225365	13.73828104
Incidence	Tunisia	Both	55+ years	Non-melanoma skin cancer (basal-cell	Rate	2021	23.620425	30.27662443	17.61945614
Incidence	Afghanista	Male	55+ years	Non-melanoma skin cancer (basal-cell	Number	1990	160.16772	209.7295929	115.3026274
Incidence	Afghanista	Female	55+ years	Non-melanoma skin cancer (basal-cell	Number	1990	79.436969	107.3835216	56.91606977
Incidence	Afghanista	Both	55+ years	Non-melanoma skin cancer (basal-cell	Number	1990	239.60469	313.6564584	171.3644135
Incidence	Afghanista	Male	55+ years	Non-melanoma skin cancer (basal-cell	Percent	1990	7.04E-05	9.20E-05	5.05E-05
Incidence	Afghanista	Female	55+ years	Non-melanoma skin cancer (basal-cell	Percent	1990	4.02E-05	5.47E-05	2.83E-05
Incidence	Afghanista	Both	55+ years	Non-melanoma skin cancer (basal-cell	Percent	1990	5.64E-05	7.49E-05	4.04E-05
Incidence	Afghanista	Male	55+ years	Non-melanoma skin cancer (basal-cell	Rate	1990	24.780069	32.44794766	17.83884462
Incidence	Afghanista	Female	55+ years	Non-melanoma skin cancer (basal-cell	Rate	1990	13.96501	18.87801135	10.00583884
Incidence	Afghanista	Both	55+ years	Non-melanoma skin cancer (basal-cell	Rate	1990	19.717538	25.81140341	14.10191273
Incidence	Afghanista	Male	55+ years	Non-melanoma skin cancer (basal-cell	Number	2021	140.46965	181.1485169	104.5075983
Incidence	Afghanista	Female	55+ years	Non-melanoma skin cancer (basal-cell	Number	2021	103.63749	138.4184835	76.36419456
Incidence	Afghanista	Both	55+ years	Non-melanoma skin cancer (basal-cell	Number	2021	244.10714	315.9913882	177.8729999
Incidence	Afghanista	Male	55+ years	Non-melanoma skin cancer (basal-cell	Percent	2021	6.73E-05	8.93E-05	4.90E-05
Incidence	Afghanista	Female	55+ years	Non-melanoma skin cancer (basal-cell	Percent	2021	3.69E-05	4.85E-05	2.62E-05
Incidence	Afghanista	Both	55+ years	Non-melanoma skin cancer (basal-cell	Percent	2021	4.98E-05	6.60E-05	3.59E-05
Incidence	Afghanista	Male	55+ years	Non-melanoma skin cancer (basal-cell	Rate	2021	26.899854	34.68983256	20.01314253
Incidence	Afghanista	Female	55+ years	Non-melanoma skin cancer (basal-cell	Rate	2021	14.667971	19.59057754	10.80794008
Incidence	Afghanista	Both	55+ years	Non-melanoma skin cancer (basal-cell	Rate	2021	19.866278	25.71646495	14.47591592
Incidence	Yemen	Male	55+ years	Non-melanoma skin cancer (basal-cell	Number	1990	89.073327	119.5794644	62.78439939
Incidence	Yemen	Female	55+ years	Non-melanoma skin cancer (basal-cell	Number	1990	58.506157	78.00967486	43.27334104
Incidence	Yemen	Both	55+ years	Non-melanoma skin cancer (basal-cell	Number	1990	147.57948	194.7630842	104.6637909
Incidence	Yemen	Male	55+ years	Non-melanoma skin cancer (basal-cell	Percent	1990	5.85E-05	7.85E-05	4.09E-05
Incidence	Yemen	Female	55+ years	Non-melanoma skin cancer (basal-cell	Percent	1990	3.66E-05	4.94E-05	2.65E-05
Incidence	Yemen	Both	55+ years	Non-melanoma skin cancer (basal-cell	Percent	1990	4.73E-05	6.34E-05	3.35E-05
Incidence	Yemen	Male	55+ years	Non-melanoma skin cancer (basal-cell	Rate	1990	22.520953	30.23400634	15.87416317
Incidence	Yemen	Female	55+ years	Non-melanoma skin cancer (basal-cell	Rate	1990	14.392396	19.19022151	10.64515397
Incidence	Yemen	Both	55+ years	Non-melanoma skin cancer (basal-cell	Rate	1990	18.40096	24.28405156	13.05001358
Incidence	Yemen	Male	55+ years	Non-melanoma skin cancer (basal-cell	Number	2021	266.91782	341.7559576	193.3823104
Incidence	Yemen	Female	55+ years	Non-melanoma skin cancer (basal-cell	Number	2021	163.40332	217.5900146	116.9560272
Incidence	Yemen	Both	55+ years	Non-melanoma skin cancer (basal-cell	Number	2021	430.32115	554.1212622	316.333244
Incidence	Yemen	Male	55+ years	Non-melanoma skin cancer (basal-cell	Percent	2021	6.23E-05	8.05E-05	4.36E-05
Incidence	Yemen	Female	55+ years	Non-melanoma skin cancer (basal-cell	Percent	2021	3.44E-05	4.53E-05	2.42E-05
Incidence	Yemen	Both	55+ years	Non-melanoma skin cancer (basal-cell	Percent	2021	4.76E-05	6.18E-05	3.38E-05
Incidence	Yemen	Male	55+ years	Non-melanoma skin cancer (basal-cell	Rate	2021	24.333403	31.15597661	17.62958218
Incidence	Yemen	Female	55+ years	Non-melanoma skin cancer (basal-cell	Rate	2021	14.184238	18.88791863	10.1523773
Incidence	Yemen	Both	55+ years	Non-melanoma skin cancer (basal-cell	Rate	2021	19.13452	24.63937581	14.06597114
Incidence	Bhutan	Male	55+ years	Non-melanoma skin cancer (basal-cell	Number	1990	0.0343749	0.081706914	0.008252904
Incidence	Bhutan	Female	55+ years	Non-melanoma skin cancer (basal-cell	Number	1990	0.0240601	0.055317841	0.006775212
Incidence	Bhutan	Both	55+ years	Non-melanoma skin cancer (basal-cell	Number	1990	0.058435	0.131424673	0.020473734
Incidence	Bhutan	Male	55+ years	Non-melanoma skin cancer (basal-cell	Percent	1990	3.96E-07	9.55E-07	9.30E-08
Incidence	Bhutan	Female	55+ years	Non-melanoma skin cancer (basal-cell	Percent	1990	2.49E-07	5.97E-07	6.92E-08

Incidence	Bhutan	Both	55+ years	Non-melanoma skin cancer (basal-cell	Percent	1990	3.19E-07	7.30E-07	1.11E-07
Incidence	Bhutan	Male	55+ years	Non-melanoma skin cancer (basal-cell	Rate	1990	0.1714531	0.407533373	0.041163391
Incidence	Bhutan	Female	55+ years	Non-melanoma skin cancer (basal-cell	Rate	1990	0.1225287	0.281712171	0.034503509
Incidence	Bhutan	Both	55+ years	Non-melanoma skin cancer (basal-cell	Rate	1990	0.1472454	0.331166036	0.051590048
Incidence	Bhutan	Male	55+ years	Non-melanoma skin cancer (basal-cell	Number	2021	0.095507	0.212553585	0.022185228
Incidence	Bhutan	Female	55+ years	Non-melanoma skin cancer (basal-cell	Number	2021	0.0626323	0.139191126	0.017236045
Incidence	Bhutan	Both	55+ years	Non-melanoma skin cancer (basal-cell	Number	2021	0.1581393	0.340525664	0.055227848
Incidence	Bhutan	Male	55+ years	Non-melanoma skin cancer (basal-cell	Percent	2021	4.62E-07	1.05E-06	1.07E-07
Incidence	Bhutan	Female	55+ years	Non-melanoma skin cancer (basal-cell	Percent	2021	2.72E-07	6.07E-07	7.42E-08
Incidence	Bhutan	Both	55+ years	Non-melanoma skin cancer (basal-cell	Percent	2021	3.62E-07	7.66E-07	1.26E-07
Incidence	Bhutan	Male	55+ years	Non-melanoma skin cancer (basal-cell	Rate	2021	0.1890217	0.420673307	0.043907673
Incidence	Bhutan	Female	55+ years	Non-melanoma skin cancer (basal-cell	Rate	2021	0.1276566	0.283698084	0.035130349
Incidence	Bhutan	Both	55+ years	Non-melanoma skin cancer (basal-cell	Rate	2021	0.1587901	0.341927176	0.055455151
Incidence	India	Male	55+ years	Non-melanoma skin cancer (basal-cell	Number	1990	2336.169	3220.882966	1648.093933
Incidence	India	Female	55+ years	Non-melanoma skin cancer (basal-cell	Number	1990	1862.9528	2577.539635	1303.105757
Incidence	India	Both	55+ years	Non-melanoma skin cancer (basal-cell	Number	1990	4199.1217	5795.179922	2953.419725
Incidence	India	Male	55+ years	Non-melanoma skin cancer (basal-cell	Percent	1990	1.20E-05	1.65E-05	8.42E-06
Incidence	India	Female	55+ years	Non-melanoma skin cancer (basal-cell	Percent	1990	9.29E-06	1.29E-05	6.45E-06
Incidence	India	Both	55+ years	Non-melanoma skin cancer (basal-cell	Percent	1990	1.06E-05	1.47E-05	7.40E-06
Incidence	India	Male	55+ years	Non-melanoma skin cancer (basal-cell	Rate	1990	5.8795278	8.106122056	4.14782242
Incidence	India	Female	55+ years	Non-melanoma skin cancer (basal-cell	Rate	1990	5.0438445	6.978550055	3.528088813
Incidence	India	Both	55+ years	Non-melanoma skin cancer (basal-cell	Rate	1990	5.4769393	7.558687434	3.852162807
Incidence	India	Male	55+ years	Non-melanoma skin cancer (basal-cell	Number	2021	6312.5809	8462.793667	4570.208092
Incidence	India	Female	55+ years	Non-melanoma skin cancer (basal-cell	Number	2021	5874.5297	7859.041131	4211.259756
Incidence	India	Both	55+ years	Non-melanoma skin cancer (basal-cell	Number	2021	12187.111	16285.2944	8801.091753
Incidence	India	Male	55+ years	Non-melanoma skin cancer (basal-cell	Percent	2021	1.19E-05	1.60E-05	8.42E-06
Incidence	India	Female	55+ years	Non-melanoma skin cancer (basal-cell	Percent	2021	9.27E-06	1.24E-05	6.54E-06
Incidence	India	Both	55+ years	Non-melanoma skin cancer (basal-cell	Percent	2021	1.05E-05	1.40E-05	7.39E-06
Incidence	India	Male	55+ years	Non-melanoma skin cancer (basal-cell	Rate	2021	6.4977239	8.711000797	4.704248727
Incidence	India	Female	55+ years	Non-melanoma skin cancer (basal-cell	Rate	2021	5.65431	7.564427746	4.053391452
Incidence	India	Both	55+ years	Non-melanoma skin cancer (basal-cell	Rate	2021	6.0618708	8.100308153	4.377664508
Incidence	Bangladesh	Male	55+ years	Non-melanoma skin cancer (basal-cell	Number	1990	7.8286887	17.3735822	2.008992273
Incidence	Bangladesh	Female	55+ years	Non-melanoma skin cancer (basal-cell	Number	1990	4.2112083	9.55531475	1.275808777
Incidence	Bangladesh	Both	55+ years	Non-melanoma skin cancer (basal-cell	Number	1990	12.039897	26.27386165	4.19121455
Incidence	Bangladesh	Male	55+ years	Non-melanoma skin cancer (basal-cell	Percent	1990	3.93E-07	8.78E-07	9.84E-08
Incidence	Bangladesh	Female	55+ years	Non-melanoma skin cancer (basal-cell	Percent	1990	2.59E-07	6.00E-07	7.63E-08
Incidence	Bangladesh	Both	55+ years	Non-melanoma skin cancer (basal-cell	Percent	1990	3.32E-07	7.13E-07	1.17E-07
Incidence	Bangladesh	Male	55+ years	Non-melanoma skin cancer (basal-cell	Rate	1990	0.1858181	0.412371325	0.047684513
Incidence	Bangladesh	Female	55+ years	Non-melanoma skin cancer (basal-cell	Rate	1990	0.1252751	0.284251568	0.037952768
Incidence	Bangladesh	Both	55+ years	Non-melanoma skin cancer (basal-cell	Rate	1990	0.1589496	0.346865159	0.055332038
Incidence	Bangladesh	Male	55+ years	Non-melanoma skin cancer (basal-cell	Number	2021	22.758732	51.41498669	4.925235223
Incidence	Bangladesh	Female	55+ years	Non-melanoma skin cancer (basal-cell	Number	2021	14.188711	32.24076956	4.076437407
Incidence	Bangladesh	Both	55+ years	Non-melanoma skin cancer (basal-cell	Number	2021	36.947443	81.10497436	12.72989222
Incidence	Bangladesh	Male	55+ years	Non-melanoma skin cancer (basal-cell	Percent	2021	3.92E-07	8.94E-07	8.78E-08
Incidence	Bangladesh	Female	55+ years	Non-melanoma skin cancer (basal-cell	Percent	2021	2.72E-07	6.24E-07	7.70E-08
Incidence	Bangladesh	Both	55+ years	Non-melanoma skin cancer (basal-cell	Percent	2021	3.35E-07	7.28E-07	1.15E-07
Incidence	Bangladesh	Male	55+ years	Non-melanoma skin cancer (basal-cell	Rate	2021	0.1883435	0.425492973	0.040759574
Incidence	Bangladesh	Female	55+ years	Non-melanoma skin cancer (basal-cell	Rate	2021	0.1257584	0.285758599	0.03613056
Incidence	Bangladesh	Both	55+ years	Non-melanoma skin cancer (basal-cell	Rate	2021	0.1581238	0.347104612	0.054480065
Incidence	Nepal	Male	55+ years	Non-melanoma skin cancer (basal-cell	Number	1990	1.40585	3.308545903	0.325796925
Incidence	Nepal	Female	55+ years	Non-melanoma skin cancer (basal-cell	Number	1990	0.9282085	2.152088789	0.272337267
Incidence	Nepal	Both	55+ years	Non-melanoma skin cancer (basal-cell	Number	1990	2.3340584	5.24949645	0.808472719
Incidence	Nepal	Male	55+ years	Non-melanoma skin cancer (basal-cell	Percent	1990	4.04E-07	9.52E-07	9.60E-08
Incidence	Nepal	Female	55+ years	Non-melanoma skin cancer (basal-cell	Percent	1990	2.59E-07	6.17E-07	7.55E-08
Incidence	Nepal	Both	55+ years	Non-melanoma skin cancer (basal-cell	Percent	1990	3.31E-07	7.52E-07	1.16E-07
Incidence	Nepal	Male	55+ years	Non-melanoma skin cancer (basal-cell	Rate	1990	0.1762561	0.41480345	0.040846249

Incidence	Nepal	Female	55+ years	Non-melanoma skin cancer (basal-cell	Rate	1990	0.1251457	0.290155434	0.03671788
Incidence	Nepal	Both	55+ years	Non-melanoma skin cancer (basal-cell	Rate	1990	0.1516292	0.341027024	0.052521427
Incidence	Nepal	Male	55+ years	Non-melanoma skin cancer (basal-cell	Number	2021	3.4635324	7.780745322	0.756071486
Incidence	Nepal	Female	55+ years	Non-melanoma skin cancer (basal-cell	Number	2021	2.6728027	5.966510678	0.839350484
Incidence	Nepal	Both	55+ years	Non-melanoma skin cancer (basal-cell	Number	2021	6.1363351	13.44325546	2.19954784
Incidence	Nepal	Male	55+ years	Non-melanoma skin cancer (basal-cell	Percent	2021	3.99E-07	9.13E-07	8.87E-08
Incidence	Nepal	Female	55+ years	Non-melanoma skin cancer (basal-cell	Percent	2021	2.55E-07	5.86E-07	7.80E-08
Incidence	Nepal	Both	55+ years	Non-melanoma skin cancer (basal-cell	Percent	2021	3.20E-07	7.07E-07	1.18E-07
Incidence	Nepal	Male	55+ years	Non-melanoma skin cancer (basal-cell	Rate	2021	0.1839643	0.41327143	0.040158459
Incidence	Nepal	Female	55+ years	Non-melanoma skin cancer (basal-cell	Rate	2021	0.1288014	0.287523921	0.040447986
Incidence	Nepal	Both	55+ years	Non-melanoma skin cancer (basal-cell	Rate	2021	0.1550419	0.339660066	0.055574229
Incidence	Pakistan	Male	55+ years	Non-melanoma skin cancer (basal-cell	Number	1990	59.054808	84.07106265	40.65137216
Incidence	Pakistan	Female	55+ years	Non-melanoma skin cancer (basal-cell	Number	1990	38.786145	56.12250049	26.33097294
Incidence	Pakistan	Both	55+ years	Non-melanoma skin cancer (basal-cell	Number	1990	97.840953	139.633347	68.07691438
Incidence	Pakistan	Male	55+ years	Non-melanoma skin cancer (basal-cell	Percent	1990	2.31E-06	3.26E-06	1.59E-06
Incidence	Pakistan	Female	55+ years	Non-melanoma skin cancer (basal-cell	Percent	1990	1.89E-06	2.73E-06	1.26E-06
Incidence	Pakistan	Both	55+ years	Non-melanoma skin cancer (basal-cell	Percent	1990	2.12E-06	3.00E-06	1.42E-06
Incidence	Pakistan	Male	55+ years	Non-melanoma skin cancer (basal-cell	Rate	1990	1.1500824	1.637269708	0.791678589
Incidence	Pakistan	Female	55+ years	Non-melanoma skin cancer (basal-cell	Rate	1990	0.9733917	1.408471386	0.660812003
Incidence	Pakistan	Both	55+ years	Non-melanoma skin cancer (basal-cell	Rate	1990	1.0728796	1.53115607	0.746500624
Incidence	Pakistan	Male	55+ years	Non-melanoma skin cancer (basal-cell	Number	2021	89.977202	132.1327167	59.4552168
Incidence	Pakistan	Female	55+ years	Non-melanoma skin cancer (basal-cell	Number	2021	69.932799	103.6651745	44.94647484
Incidence	Pakistan	Both	55+ years	Non-melanoma skin cancer (basal-cell	Number	2021	159.91	235.829846	106.3586656
Incidence	Pakistan	Male	55+ years	Non-melanoma skin cancer (basal-cell	Percent	2021	1.79E-06	2.63E-06	1.18E-06
Incidence	Pakistan	Female	55+ years	Non-melanoma skin cancer (basal-cell	Percent	2021	1.53E-06	2.22E-06	9.86E-07
Incidence	Pakistan	Both	55+ years	Non-melanoma skin cancer (basal-cell	Percent	2021	1.66E-06	2.43E-06	1.10E-06
Incidence	Pakistan	Male	55+ years	Non-melanoma skin cancer (basal-cell	Rate	2021	0.8556065	1.256469491	0.565368426
Incidence	Pakistan	Female	55+ years	Non-melanoma skin cancer (basal-cell	Rate	2021	0.7511733	1.113504914	0.482786247
Incidence	Pakistan	Both	55+ years	Non-melanoma skin cancer (basal-cell	Rate	2021	0.8065672	1.189497966	0.536460582
Incidence	Angola	Male	55+ years	Non-melanoma skin cancer (basal-cell	Number	1990	69.118143	93.12712681	48.18582873
Incidence	Angola	Female	55+ years	Non-melanoma skin cancer (basal-cell	Number	1990	39.937546	54.23931482	27.67138699
Incidence	Angola	Both	55+ years	Non-melanoma skin cancer (basal-cell	Number	1990	109.05569	148.7862664	75.99880791
Incidence	Angola	Male	55+ years	Non-melanoma skin cancer (basal-cell	Percent	1990	5.03E-05	6.85E-05	3.43E-05
Incidence	Angola	Female	55+ years	Non-melanoma skin cancer (basal-cell	Percent	1990	2.98E-05	4.05E-05	2.06E-05
Incidence	Angola	Both	55+ years	Non-melanoma skin cancer (basal-cell	Percent	1990	4.02E-05	5.41E-05	2.74E-05
Incidence	Angola	Male	55+ years	Non-melanoma skin cancer (basal-cell	Rate	1990	22.0595	29.72212202	15.37881743
Incidence	Angola	Female	55+ years	Non-melanoma skin cancer (basal-cell	Rate	1990	12.900335	17.51998758	8.938209455
Incidence	Angola	Both	55+ years	Non-melanoma skin cancer (basal-cell	Rate	1990	17.507418	23.88562577	12.20058227
Incidence	Angola	Male	55+ years	Non-melanoma skin cancer (basal-cell	Number	2021	189.90202	251.9500428	137.7025517
Incidence	Angola	Female	55+ years	Non-melanoma skin cancer (basal-cell	Number	2021	138.01436	184.1185541	96.8790126
Incidence	Angola	Both	55+ years	Non-melanoma skin cancer (basal-cell	Number	2021	327.91637	437.8827506	235.5478185
Incidence	Angola	Male	55+ years	Non-melanoma skin cancer (basal-cell	Percent	2021	4.22E-05	5.68E-05	2.93E-05
Incidence	Angola	Female	55+ years	Non-melanoma skin cancer (basal-cell	Percent	2021	2.47E-05	3.31E-05	1.71E-05
Incidence	Angola	Both	55+ years	Non-melanoma skin cancer (basal-cell	Percent	2021	3.25E-05	4.35E-05	2.27E-05
Incidence	Angola	Male	55+ years	Non-melanoma skin cancer (basal-cell	Rate	2021	21.95155	29.12393546	15.9176009
Incidence	Angola	Female	55+ years	Non-melanoma skin cancer (basal-cell	Rate	2021	12.916233	17.23094791	9.066534484
Incidence	Angola	Both	55+ years	Non-melanoma skin cancer (basal-cell	Rate	2021	16.958587	22.64563	12.18163707
Incidence	Central Af	Male	55+ years	Non-melanoma skin cancer (basal-cell	Number	1990	19.421285	25.86501427	13.3509393
Incidence	Central Af	Female	55+ years	Non-melanoma skin cancer (basal-cell	Number	1990	13.034092	17.66246324	9.034425787
Incidence	Central Af	Both	55+ years	Non-melanoma skin cancer (basal-cell	Number	1990	32.455376	43.19792408	22.65646535
Incidence	Central Af	Male	55+ years	Non-melanoma skin cancer (basal-cell	Percent	1990	4.29E-05	5.82E-05	2.92E-05
Incidence	Central Af	Female	55+ years	Non-melanoma skin cancer (basal-cell	Percent	1990	2.62E-05	3.58E-05	1.80E-05
Incidence	Central Af	Both	55+ years	Non-melanoma skin cancer (basal-cell	Percent	1990	3.41E-05	4.55E-05	2.34E-05
Incidence	Central Af	Male	55+ years	Non-melanoma skin cancer (basal-cell	Rate	1990	22.024325	29.33170987	15.14036969
Incidence	Central Af	Female	55+ years	Non-melanoma skin cancer (basal-cell	Rate	1990	12.95229	17.55161346	8.97772565
Incidence	Central Af	Both	55+ years	Non-melanoma skin cancer (basal-cell	Rate	1990	17.189198	22.8787259	11.99944376

Incidence	Central Af	Male	55+ years	Non-melanoma skin cancer (basal-cell	Number	2021	34.422691	47.08734346	24.43544005
Incidence	Central Af	Female	55+ years	Non-melanoma skin cancer (basal-cell	Number	2021	25.388542	34.42590707	17.7256353
Incidence	Central Af	Both	55+ years	Non-melanoma skin cancer (basal-cell	Number	2021	59.811233	80.55634579	42.32362311
Incidence	Central Af	Male	55+ years	Non-melanoma skin cancer (basal-cell	Percent	2021	3.75E-05	5.23E-05	2.59E-05
Incidence	Central Af	Female	55+ years	Non-melanoma skin cancer (basal-cell	Percent	2021	2.34E-05	3.17E-05	1.65E-05
Incidence	Central Af	Both	55+ years	Non-melanoma skin cancer (basal-cell	Percent	2021	2.99E-05	4.10E-05	2.07E-05
Incidence	Central Af	Male	55+ years	Non-melanoma skin cancer (basal-cell	Rate	2021	21.243262	29.05899415	15.07983372
Incidence	Central Af	Female	55+ years	Non-melanoma skin cancer (basal-cell	Rate	2021	12.975977	17.59493561	9.05949729
Incidence	Central Af	Both	55+ years	Non-melanoma skin cancer (basal-cell	Rate	2021	16.721128	22.52073533	11.83220397
Incidence	Congo	Male	55+ years	Non-melanoma skin cancer (basal-cell	Number	1990	17.620145	24.05549333	12.51086122
Incidence	Congo	Female	55+ years	Non-melanoma skin cancer (basal-cell	Number	1990	13.511441	17.90329747	9.6942915
Incidence	Congo	Both	55+ years	Non-melanoma skin cancer (basal-cell	Number	1990	31.131586	41.46491067	22.07997619
Incidence	Congo	Male	55+ years	Non-melanoma skin cancer (basal-cell	Percent	1990	4.72E-05	6.30E-05	3.33E-05
Incidence	Congo	Female	55+ years	Non-melanoma skin cancer (basal-cell	Percent	1990	2.83E-05	3.76E-05	2.00E-05
Incidence	Congo	Both	55+ years	Non-melanoma skin cancer (basal-cell	Percent	1990	3.66E-05	4.83E-05	2.60E-05
Incidence	Congo	Male	55+ years	Non-melanoma skin cancer (basal-cell	Rate	1990	22.818643	31.15262141	16.20195927
Incidence	Congo	Female	55+ years	Non-melanoma skin cancer (basal-cell	Rate	1990	13.404943	17.76218279	9.617880618
Incidence	Congo	Both	55+ years	Non-melanoma skin cancer (basal-cell	Rate	1990	17.488411	23.29323611	12.40359837
Incidence	Congo	Male	55+ years	Non-melanoma skin cancer (basal-cell	Number	2021	46.683217	60.78878123	33.3214351
Incidence	Congo	Female	55+ years	Non-melanoma skin cancer (basal-cell	Number	2021	29.596952	39.09683023	20.76240132
Incidence	Congo	Both	55+ years	Non-melanoma skin cancer (basal-cell	Number	2021	76.280169	100.1718043	54.7392409
Incidence	Congo	Male	55+ years	Non-melanoma skin cancer (basal-cell	Percent	2021	3.92E-05	5.18E-05	2.76E-05
Incidence	Congo	Female	55+ years	Non-melanoma skin cancer (basal-cell	Percent	2021	2.25E-05	3.04E-05	1.55E-05
Incidence	Congo	Both	55+ years	Non-melanoma skin cancer (basal-cell	Percent	2021	3.04E-05	4.01E-05	2.14E-05
Incidence	Congo	Male	55+ years	Non-melanoma skin cancer (basal-cell	Rate	2021	21.970983	28.60962342	15.68239551
Incidence	Congo	Female	55+ years	Non-melanoma skin cancer (basal-cell	Rate	2021	13.328853	17.60708039	9.350253385
Incidence	Congo	Both	55+ years	Non-melanoma skin cancer (basal-cell	Rate	2021	17.554701	23.0529914	12.59738964
Incidence	Democratic	Male	55+ years	Non-melanoma skin cancer (basal-cell	Number	1990	272.85311	357.370565	191.8268227
Incidence	Democratic	Female	55+ years	Non-melanoma skin cancer (basal-cell	Number	1990	180.53181	242.9547629	126.704726
Incidence	Democratic	Both	55+ years	Non-melanoma skin cancer (basal-cell	Number	1990	453.38492	603.2286647	324.4310813
Incidence	Democratic	Male	55+ years	Non-melanoma skin cancer (basal-cell	Percent	1990	4.77E-05	6.37E-05	3.30E-05
Incidence	Democratic	Female	55+ years	Non-melanoma skin cancer (basal-cell	Percent	1990	2.83E-05	3.84E-05	1.94E-05
Incidence	Democratic	Both	55+ years	Non-melanoma skin cancer (basal-cell	Percent	1990	3.75E-05	4.99E-05	2.60E-05
Incidence	Democratic	Male	55+ years	Non-melanoma skin cancer (basal-cell	Rate	1990	22.477023	29.43938052	15.80226068
Incidence	Democratic	Female	55+ years	Non-melanoma skin cancer (basal-cell	Rate	1990	12.652725	17.02768962	8.880207659
Incidence	Democratic	Both	55+ years	Non-melanoma skin cancer (basal-cell	Rate	1990	17.168847	22.84315221	12.28560413
Incidence	Democratic	Male	55+ years	Non-melanoma skin cancer (basal-cell	Number	2021	594.91682	782.0615868	431.7061297
Incidence	Democratic	Female	55+ years	Non-melanoma skin cancer (basal-cell	Number	2021	436.6437	580.896913	310.219654
Incidence	Democratic	Both	55+ years	Non-melanoma skin cancer (basal-cell	Number	2021	1031.5605	1367.549442	739.8285711
Incidence	Democratic	Male	55+ years	Non-melanoma skin cancer (basal-cell	Percent	2021	4.07E-05	5.50E-05	2.87E-05
Incidence	Democratic	Female	55+ years	Non-melanoma skin cancer (basal-cell	Percent	2021	2.62E-05	3.50E-05	1.87E-05
Incidence	Democratic	Both	55+ years	Non-melanoma skin cancer (basal-cell	Percent	2021	3.30E-05	4.40E-05	2.33E-05
Incidence	Democratic	Male	55+ years	Non-melanoma skin cancer (basal-cell	Rate	2021	21.53585	28.31044659	15.62766096
Incidence	Democratic	Female	55+ years	Non-melanoma skin cancer (basal-cell	Rate	2021	13.323008	17.72450627	9.465518031
Incidence	Democratic	Both	55+ years	Non-melanoma skin cancer (basal-cell	Rate	2021	17.079341	22.642242	12.24919336
Incidence	Equatorial	Male	55+ years	Non-melanoma skin cancer (basal-cell	Number	1990	3.2261177	4.260592812	2.299649942
Incidence	Equatorial	Female	55+ years	Non-melanoma skin cancer (basal-cell	Number	1990	2.318059	3.054916758	1.648268726
Incidence	Equatorial	Both	55+ years	Non-melanoma skin cancer (basal-cell	Number	1990	5.5441767	7.268676066	3.973035799
Incidence	Equatorial	Male	55+ years	Non-melanoma skin cancer (basal-cell	Percent	1990	4.72E-05	6.25E-05	3.34E-05
Incidence	Equatorial	Female	55+ years	Non-melanoma skin cancer (basal-cell	Percent	1990	2.88E-05	3.76E-05	2.02E-05
Incidence	Equatorial	Both	55+ years	Non-melanoma skin cancer (basal-cell	Percent	1990	3.72E-05	4.87E-05	2.67E-05
Incidence	Equatorial	Male	55+ years	Non-melanoma skin cancer (basal-cell	Rate	1990	22.326355	29.48544221	15.91473264
Incidence	Equatorial	Female	55+ years	Non-melanoma skin cancer (basal-cell	Rate	1990	13.267967	17.4855483	9.434261129
Incidence	Equatorial	Both	55+ years	Non-melanoma skin cancer (basal-cell	Rate	1990	17.368478	22.77089006	12.4464979
Incidence	Equatorial	Male	55+ years	Non-melanoma skin cancer (basal-cell	Number	2021	7.6849344	9.939059565	5.512108651
Incidence	Equatorial	Female	55+ years	Non-melanoma skin cancer (basal-cell	Number	2021	6.1695976	8.251305992	4.478267771

Incidence	Equatorial	Both	55+ years	Non-melanoma skin cancer (basal-cell	Number	2021	13.854532	17.98846754	9.991018421
Incidence	Equatorial	Male	55+ years	Non-melanoma skin cancer (basal-cell	Percent	2021	4.51E-05	6.00E-05	3.18E-05
Incidence	Equatorial	Female	55+ years	Non-melanoma skin cancer (basal-cell	Percent	2021	2.54E-05	3.41E-05	1.82E-05
Incidence	Equatorial	Both	55+ years	Non-melanoma skin cancer (basal-cell	Percent	2021	3.35E-05	4.43E-05	2.40E-05
Incidence	Equatorial	Male	55+ years	Non-melanoma skin cancer (basal-cell	Rate	2021	23.027685	29.78210703	16.51687554
Incidence	Equatorial	Female	55+ years	Non-melanoma skin cancer (basal-cell	Rate	2021	13.493023	18.0457573	9.794053621
Incidence	Equatorial	Both	55+ years	Non-melanoma skin cancer (basal-cell	Rate	2021	17.515888	22.74230491	12.63135878
Incidence	Gabon	Male	55+ years	Non-melanoma skin cancer (basal-cell	Number	1990	10.480567	13.51115778	7.586210082
Incidence	Gabon	Female	55+ years	Non-melanoma skin cancer (basal-cell	Number	1990	7.563941	9.975062766	5.456964426
Incidence	Gabon	Both	55+ years	Non-melanoma skin cancer (basal-cell	Number	1990	18.044508	23.5106264	13.00407927
Incidence	Gabon	Male	55+ years	Non-melanoma skin cancer (basal-cell	Percent	1990	5.10E-05	6.74E-05	3.62E-05
Incidence	Gabon	Female	55+ years	Non-melanoma skin cancer (basal-cell	Percent	1990	2.87E-05	3.80E-05	2.03E-05
Incidence	Gabon	Both	55+ years	Non-melanoma skin cancer (basal-cell	Percent	1990	3.85E-05	5.02E-05	2.74E-05
Incidence	Gabon	Male	55+ years	Non-melanoma skin cancer (basal-cell	Rate	1990	24.011664	30.95494566	17.38050319
Incidence	Gabon	Female	55+ years	Non-melanoma skin cancer (basal-cell	Rate	1990	13.941799	18.3859603	10.05823559
Incidence	Gabon	Both	55+ years	Non-melanoma skin cancer (basal-cell	Rate	1990	18.431287	24.01456954	13.28281776
Incidence	Gabon	Male	55+ years	Non-melanoma skin cancer (basal-cell	Number	2021	19.386308	26.5452366	14.04155878
Incidence	Gabon	Female	55+ years	Non-melanoma skin cancer (basal-cell	Number	2021	12.550315	16.55254338	9.19861095
Incidence	Gabon	Both	55+ years	Non-melanoma skin cancer (basal-cell	Number	2021	31.936623	42.63276461	23.19996301
Incidence	Gabon	Male	55+ years	Non-melanoma skin cancer (basal-cell	Percent	2021	4.16E-05	5.65E-05	2.97E-05
Incidence	Gabon	Female	55+ years	Non-melanoma skin cancer (basal-cell	Percent	2021	2.40E-05	3.18E-05	1.74E-05
Incidence	Gabon	Both	55+ years	Non-melanoma skin cancer (basal-cell	Percent	2021	3.23E-05	4.29E-05	2.32E-05
Incidence	Gabon	Male	55+ years	Non-melanoma skin cancer (basal-cell	Rate	2021	22.304367	30.54086899	16.15511715
Incidence	Gabon	Female	55+ years	Non-melanoma skin cancer (basal-cell	Rate	2021	13.666174	18.02424462	10.01646757
Incidence	Gabon	Both	55+ years	Non-melanoma skin cancer (basal-cell	Rate	2021	17.866444	23.85023398	12.97885678
Incidence	Comoros	Male	55+ years	Non-melanoma skin cancer (basal-cell	Number	1990	2.8600631	3.72549317	2.057321608
Incidence	Comoros	Female	55+ years	Non-melanoma skin cancer (basal-cell	Number	1990	1.8443491	2.486135487	1.309590305
Incidence	Comoros	Both	55+ years	Non-melanoma skin cancer (basal-cell	Number	1990	4.7044122	6.196670987	3.385585221
Incidence	Comoros	Male	55+ years	Non-melanoma skin cancer (basal-cell	Percent	1990	3.54E-05	4.67E-05	2.52E-05
Incidence	Comoros	Female	55+ years	Non-melanoma skin cancer (basal-cell	Percent	1990	2.18E-05	2.97E-05	1.52E-05
Incidence	Comoros	Both	55+ years	Non-melanoma skin cancer (basal-cell	Percent	1990	2.84E-05	3.69E-05	2.01E-05
Incidence	Comoros	Male	55+ years	Non-melanoma skin cancer (basal-cell	Rate	1990	17.835211	23.23199005	12.82935519
Incidence	Comoros	Female	55+ years	Non-melanoma skin cancer (basal-cell	Rate	1990	11.233357	15.14227803	7.97630725
Incidence	Comoros	Both	55+ years	Non-melanoma skin cancer (basal-cell	Rate	1990	14.495385	19.09338005	10.43177303
Incidence	Comoros	Male	55+ years	Non-melanoma skin cancer (basal-cell	Number	2021	6.8167491	8.927630616	4.912792281
Incidence	Comoros	Female	55+ years	Non-melanoma skin cancer (basal-cell	Number	2021	5.0587138	6.82274172	3.598343295
Incidence	Comoros	Both	55+ years	Non-melanoma skin cancer (basal-cell	Number	2021	11.875463	15.71651465	8.614908033
Incidence	Comoros	Male	55+ years	Non-melanoma skin cancer (basal-cell	Percent	2021	3.49E-05	4.64E-05	2.50E-05
Incidence	Comoros	Female	55+ years	Non-melanoma skin cancer (basal-cell	Percent	2021	2.03E-05	2.79E-05	1.42E-05
Incidence	Comoros	Both	55+ years	Non-melanoma skin cancer (basal-cell	Percent	2021	2.67E-05	3.55E-05	1.90E-05
Incidence	Comoros	Male	55+ years	Non-melanoma skin cancer (basal-cell	Rate	2021	18.41891	24.12252873	13.2744037
Incidence	Comoros	Female	55+ years	Non-melanoma skin cancer (basal-cell	Rate	2021	11.544032	15.56956049	8.211453095
Incidence	Comoros	Both	55+ years	Non-melanoma skin cancer (basal-cell	Rate	2021	14.691801	19.44378197	10.65798603
Incidence	Eritrea	Male	55+ years	Non-melanoma skin cancer (basal-cell	Number	1990	12.088657	16.6684401	8.433465719
Incidence	Eritrea	Female	55+ years	Non-melanoma skin cancer (basal-cell	Number	1990	10.906309	14.97865343	7.452867268
Incidence	Eritrea	Both	55+ years	Non-melanoma skin cancer (basal-cell	Number	1990	22.994966	31.77740751	16.22406311
Incidence	Eritrea	Male	55+ years	Non-melanoma skin cancer (basal-cell	Percent	1990	2.78E-05	3.90E-05	1.91E-05
Incidence	Eritrea	Female	55+ years	Non-melanoma skin cancer (basal-cell	Percent	1990	2.07E-05	2.86E-05	1.40E-05
Incidence	Eritrea	Both	55+ years	Non-melanoma skin cancer (basal-cell	Percent	1990	2.39E-05	3.32E-05	1.65E-05
Incidence	Eritrea	Male	55+ years	Non-melanoma skin cancer (basal-cell	Rate	1990	15.94571	21.98673575	11.12427924
Incidence	Eritrea	Female	55+ years	Non-melanoma skin cancer (basal-cell	Rate	1990	10.808069	14.84373235	7.385735142
Incidence	Eritrea	Both	55+ years	Non-melanoma skin cancer (basal-cell	Rate	1990	13.012069	17.98175402	9.18064546
Incidence	Eritrea	Male	55+ years	Non-melanoma skin cancer (basal-cell	Number	2021	30.649017	42.0139312	22.43652561
Incidence	Eritrea	Female	55+ years	Non-melanoma skin cancer (basal-cell	Number	2021	29.430349	40.28639354	20.24030557
Incidence	Eritrea	Both	55+ years	Non-melanoma skin cancer (basal-cell	Number	2021	60.079366	82.4140677	42.69070015
Incidence	Eritrea	Male	55+ years	Non-melanoma skin cancer (basal-cell	Percent	2021	2.74E-05	3.78E-05	1.93E-05

Incidence	Eritrea	Female	55+ years	Non-melanoma skin cancer (basal-cell	Percent	2021	2.00E-05	2.75E-05	1.38E-05
Incidence	Eritrea	Both	55+ years	Non-melanoma skin cancer (basal-cell	Percent	2021	2.32E-05	3.17E-05	1.64E-05
Incidence	Eritrea	Male	55+ years	Non-melanoma skin cancer (basal-cell	Rate	2021	17.093956	23.43253926	12.51358185
Incidence	Eritrea	Female	55+ years	Non-melanoma skin cancer (basal-cell	Rate	2021	11.262547	15.41698998	7.745657049
Incidence	Eritrea	Both	55+ years	Non-melanoma skin cancer (basal-cell	Rate	2021	13.635527	18.70457868	9.689020115
Incidence	Ethiopia	Male	55+ years	Non-melanoma skin cancer (basal-cell	Number	1990	318.56551	417.0298649	225.3757247
Incidence	Ethiopia	Female	55+ years	Non-melanoma skin cancer (basal-cell	Number	1990	170.60488	228.4539399	117.0098748
Incidence	Ethiopia	Both	55+ years	Non-melanoma skin cancer (basal-cell	Number	1990	489.17039	648.6553917	347.2132788
Incidence	Ethiopia	Male	55+ years	Non-melanoma skin cancer (basal-cell	Percent	1990	3.37E-05	4.56E-05	2.33E-05
Incidence	Ethiopia	Female	55+ years	Non-melanoma skin cancer (basal-cell	Percent	1990	2.09E-05	2.81E-05	1.41E-05
Incidence	Ethiopia	Both	55+ years	Non-melanoma skin cancer (basal-cell	Percent	1990	2.77E-05	3.67E-05	1.91E-05
Incidence	Ethiopia	Male	55+ years	Non-melanoma skin cancer (basal-cell	Rate	1990	18.636048	24.3962025	13.1844558
Incidence	Ethiopia	Female	55+ years	Non-melanoma skin cancer (basal-cell	Rate	1990	11.346602	15.19403195	7.78210162
Incidence	Ethiopia	Both	55+ years	Non-melanoma skin cancer (basal-cell	Rate	1990	15.224811	20.18858072	10.80657526
Incidence	Ethiopia	Male	55+ years	Non-melanoma skin cancer (basal-cell	Number	2021	681.64652	876.7176776	496.6036561
Incidence	Ethiopia	Female	55+ years	Non-melanoma skin cancer (basal-cell	Number	2021	403.45488	533.8377761	287.8798416
Incidence	Ethiopia	Both	55+ years	Non-melanoma skin cancer (basal-cell	Number	2021	1085.1014	1407.630456	783.1412597
Incidence	Ethiopia	Male	55+ years	Non-melanoma skin cancer (basal-cell	Percent	2021	3.40E-05	4.41E-05	2.42E-05
Incidence	Ethiopia	Female	55+ years	Non-melanoma skin cancer (basal-cell	Percent	2021	2.00E-05	2.60E-05	1.38E-05
Incidence	Ethiopia	Both	55+ years	Non-melanoma skin cancer (basal-cell	Percent	2021	2.70E-05	3.45E-05	1.90E-05
Incidence	Ethiopia	Male	55+ years	Non-melanoma skin cancer (basal-cell	Rate	2021	19.576717	25.17911169	14.26233238
Incidence	Ethiopia	Female	55+ years	Non-melanoma skin cancer (basal-cell	Rate	2021	11.994547	15.87077711	8.558549064
Incidence	Ethiopia	Both	55+ years	Non-melanoma skin cancer (basal-cell	Rate	2021	15.851131	20.56262692	11.44010595
Incidence	Madagascar	Male	55+ years	Non-melanoma skin cancer (basal-cell	Number	1990	78.901651	103.6861267	58.77495351
Incidence	Madagascar	Female	55+ years	Non-melanoma skin cancer (basal-cell	Number	1990	47.262404	64.26568668	33.67507691
Incidence	Madagascar	Both	55+ years	Non-melanoma skin cancer (basal-cell	Number	1990	126.16405	167.4761601	93.02998615
Incidence	Madagascar	Male	55+ years	Non-melanoma skin cancer (basal-cell	Percent	1990	3.79E-05	5.01E-05	2.77E-05
Incidence	Madagascar	Female	55+ years	Non-melanoma skin cancer (basal-cell	Percent	1990	2.30E-05	3.17E-05	1.61E-05
Incidence	Madagascar	Both	55+ years	Non-melanoma skin cancer (basal-cell	Percent	1990	3.05E-05	4.00E-05	2.21E-05
Incidence	Madagascar	Male	55+ years	Non-melanoma skin cancer (basal-cell	Rate	1990	18.374018	24.14563936	13.68706573
Incidence	Madagascar	Female	55+ years	Non-melanoma skin cancer (basal-cell	Rate	1990	11.375128	15.46748274	8.104926561
Incidence	Madagascar	Both	55+ years	Non-melanoma skin cancer (basal-cell	Rate	1990	14.932271	19.82180654	11.01065601
Incidence	Madagascar	Male	55+ years	Non-melanoma skin cancer (basal-cell	Number	2021	153.92755	207.2931638	111.9460658
Incidence	Madagascar	Female	55+ years	Non-melanoma skin cancer (basal-cell	Number	2021	105.07283	143.5492146	72.70048594
Incidence	Madagascar	Both	55+ years	Non-melanoma skin cancer (basal-cell	Number	2021	259.00038	347.3386584	184.0429171
Incidence	Madagascar	Male	55+ years	Non-melanoma skin cancer (basal-cell	Percent	2021	3.15E-05	4.25E-05	2.24E-05
Incidence	Madagascar	Female	55+ years	Non-melanoma skin cancer (basal-cell	Percent	2021	1.91E-05	2.63E-05	1.30E-05
Incidence	Madagascar	Both	55+ years	Non-melanoma skin cancer (basal-cell	Percent	2021	2.49E-05	3.34E-05	1.76E-05
Incidence	Madagascar	Male	55+ years	Non-melanoma skin cancer (basal-cell	Rate	2021	17.119134	23.05421833	12.45014064
Incidence	Madagascar	Female	55+ years	Non-melanoma skin cancer (basal-cell	Rate	2021	11.089183	15.14990633	7.672668603
Incidence	Madagascar	Both	55+ years	Non-melanoma skin cancer (basal-cell	Rate	2021	14.025187	18.80881216	9.966148521
Incidence	Malawi	Male	55+ years	Non-melanoma skin cancer (basal-cell	Number	1990	49.977637	66.02777681	36.25762221
Incidence	Malawi	Female	55+ years	Non-melanoma skin cancer (basal-cell	Number	1990	24.02769	33.39360301	16.65830749
Incidence	Malawi	Both	55+ years	Non-melanoma skin cancer (basal-cell	Number	1990	74.005327	99.55049066	53.11324499
Incidence	Malawi	Male	55+ years	Non-melanoma skin cancer (basal-cell	Percent	1990	3.13E-05	4.16E-05	2.21E-05
Incidence	Malawi	Female	55+ years	Non-melanoma skin cancer (basal-cell	Percent	1990	1.34E-05	1.88E-05	9.22E-06
Incidence	Malawi	Both	55+ years	Non-melanoma skin cancer (basal-cell	Percent	1990	2.18E-05	2.90E-05	1.53E-05
Incidence	Malawi	Male	55+ years	Non-melanoma skin cancer (basal-cell	Rate	1990	17.00925	22.47171042	12.33981857
Incidence	Malawi	Female	55+ years	Non-melanoma skin cancer (basal-cell	Rate	1990	7.1691738	9.963693688	4.970361332
Incidence	Malawi	Both	55+ years	Non-melanoma skin cancer (basal-cell	Rate	1990	11.765944	15.82731386	8.444358166
Incidence	Malawi	Male	55+ years	Non-melanoma skin cancer (basal-cell	Number	2021	90.253473	122.8930303	64.33882638
Incidence	Malawi	Female	55+ years	Non-melanoma skin cancer (basal-cell	Number	2021	48.33866	66.9195826	33.73810032
Incidence	Malawi	Both	55+ years	Non-melanoma skin cancer (basal-cell	Number	2021	138.59213	188.646459	98.63431251
Incidence	Malawi	Male	55+ years	Non-melanoma skin cancer (basal-cell	Percent	2021	2.92E-05	3.96E-05	2.03E-05
Incidence	Malawi	Female	55+ years	Non-melanoma skin cancer (basal-cell	Percent	2021	1.22E-05	1.67E-05	8.46E-06
Incidence	Malawi	Both	55+ years	Non-melanoma skin cancer (basal-cell	Percent	2021	1.97E-05	2.64E-05	1.38E-05

Incidence	Malawi	Male	55+ years	Non-melanoma skin cancer (basal-cell	Rate	2021	17.409123	23.70501437	12.41040929
Incidence	Malawi	Female	55+ years	Non-melanoma skin cancer (basal-cell	Rate	2021	7.4286983	10.28421952	5.184880366
Incidence	Malawi	Both	55+ years	Non-melanoma skin cancer (basal-cell	Rate	2021	11.854317	16.13565595	8.436571455
Incidence	Mozambique	Male	55+ years	Non-melanoma skin cancer (basal-cell	Number	1990	86.151908	114.4312526	63.00870954
Incidence	Mozambique	Female	55+ years	Non-melanoma skin cancer (basal-cell	Number	1990	57.304248	78.91165716	39.42820008
Incidence	Mozambique	Both	55+ years	Non-melanoma skin cancer (basal-cell	Number	1990	143.45616	192.4075699	104.0279415
Incidence	Mozambique	Male	55+ years	Non-melanoma skin cancer (basal-cell	Percent	1990	3.70E-05	4.98E-05	2.63E-05
Incidence	Mozambique	Female	55+ years	Non-melanoma skin cancer (basal-cell	Percent	1990	2.38E-05	3.22E-05	1.64E-05
Incidence	Mozambique	Both	55+ years	Non-melanoma skin cancer (basal-cell	Percent	1990	3.03E-05	4.06E-05	2.12E-05
Incidence	Mozambique	Male	55+ years	Non-melanoma skin cancer (basal-cell	Rate	1990	18.232189	24.2169012	13.33443145
Incidence	Mozambique	Female	55+ years	Non-melanoma skin cancer (basal-cell	Rate	1990	11.355	15.63656914	7.812809904
Incidence	Mozambique	Both	55+ years	Non-melanoma skin cancer (basal-cell	Rate	1990	14.680517	19.68993668	10.64564966
Incidence	Mozambique	Male	55+ years	Non-melanoma skin cancer (basal-cell	Number	2021	142.59687	196.1245338	101.093838
Incidence	Mozambique	Female	55+ years	Non-melanoma skin cancer (basal-cell	Number	2021	112.74949	152.1167161	77.8305607
Incidence	Mozambique	Both	55+ years	Non-melanoma skin cancer (basal-cell	Number	2021	255.34636	346.4456791	178.9499575
Incidence	Mozambique	Male	55+ years	Non-melanoma skin cancer (basal-cell	Percent	2021	3.21E-05	4.45E-05	2.24E-05
Incidence	Mozambique	Female	55+ years	Non-melanoma skin cancer (basal-cell	Percent	2021	2.07E-05	2.77E-05	1.43E-05
Incidence	Mozambique	Both	55+ years	Non-melanoma skin cancer (basal-cell	Percent	2021	2.58E-05	3.51E-05	1.81E-05
Incidence	Mozambique	Male	55+ years	Non-melanoma skin cancer (basal-cell	Rate	2021	17.784637	24.46059087	12.60839204
Incidence	Mozambique	Female	55+ years	Non-melanoma skin cancer (basal-cell	Rate	2021	11.419563	15.40677868	7.882882654
Incidence	Mozambique	Both	55+ years	Non-melanoma skin cancer (basal-cell	Rate	2021	14.272062	19.36387212	10.00204159
Incidence	Rwanda	Male	55+ years	Non-melanoma skin cancer (basal-cell	Number	1990	37.922878	50.90799337	26.52614611
Incidence	Rwanda	Female	55+ years	Non-melanoma skin cancer (basal-cell	Number	1990	29.314375	39.57082719	20.24240294
Incidence	Rwanda	Both	55+ years	Non-melanoma skin cancer (basal-cell	Number	1990	67.237253	89.93622697	47.60091614
Incidence	Rwanda	Male	55+ years	Non-melanoma skin cancer (basal-cell	Percent	1990	3.81E-05	5.09E-05	2.62E-05
Incidence	Rwanda	Female	55+ years	Non-melanoma skin cancer (basal-cell	Percent	1990	2.28E-05	3.08E-05	1.55E-05
Incidence	Rwanda	Both	55+ years	Non-melanoma skin cancer (basal-cell	Percent	1990	2.94E-05	3.93E-05	2.03E-05
Incidence	Rwanda	Male	55+ years	Non-melanoma skin cancer (basal-cell	Rate	1990	18.252175	24.50187516	12.76696011
Incidence	Rwanda	Female	55+ years	Non-melanoma skin cancer (basal-cell	Rate	1990	11.267096	15.20920366	7.780247492
Incidence	Rwanda	Both	55+ years	Non-melanoma skin cancer (basal-cell	Rate	1990	14.36851	19.21925019	10.17225146
Incidence	Rwanda	Male	55+ years	Non-melanoma skin cancer (basal-cell	Number	2021	79.808831	107.0772016	58.35471648
Incidence	Rwanda	Female	55+ years	Non-melanoma skin cancer (basal-cell	Number	2021	69.55168	92.47326416	48.43313131
Incidence	Rwanda	Both	55+ years	Non-melanoma skin cancer (basal-cell	Number	2021	149.36051	200.5879106	106.8640553
Incidence	Rwanda	Male	55+ years	Non-melanoma skin cancer (basal-cell	Percent	2021	3.57E-05	4.76E-05	2.52E-05
Incidence	Rwanda	Female	55+ years	Non-melanoma skin cancer (basal-cell	Percent	2021	2.08E-05	2.80E-05	1.43E-05
Incidence	Rwanda	Both	55+ years	Non-melanoma skin cancer (basal-cell	Percent	2021	2.68E-05	3.56E-05	1.88E-05
Incidence	Rwanda	Male	55+ years	Non-melanoma skin cancer (basal-cell	Rate	2021	17.865368	23.9694474	13.0628209
Incidence	Rwanda	Female	55+ years	Non-melanoma skin cancer (basal-cell	Rate	2021	11.388055	15.14112402	7.930206145
Incidence	Rwanda	Both	55+ years	Non-melanoma skin cancer (basal-cell	Rate	2021	14.124378	18.96873206	10.105672
Incidence	Mauritius	Male	55+ years	Non-melanoma skin cancer (basal-cell	Number	1990	2.5264503	3.490197566	1.766385601
Incidence	Mauritius	Female	55+ years	Non-melanoma skin cancer (basal-cell	Number	1990	2.3943878	3.243114149	1.654479085
Incidence	Mauritius	Both	55+ years	Non-melanoma skin cancer (basal-cell	Number	1990	4.9208381	6.718197295	3.466686346
Incidence	Mauritius	Male	55+ years	Non-melanoma skin cancer (basal-cell	Percent	1990	1.27E-05	1.74E-05	8.68E-06
Incidence	Mauritius	Female	55+ years	Non-melanoma skin cancer (basal-cell	Percent	1990	9.95E-06	1.37E-05	6.69E-06
Incidence	Mauritius	Both	55+ years	Non-melanoma skin cancer (basal-cell	Percent	1990	1.12E-05	1.53E-05	7.63E-06
Incidence	Mauritius	Male	55+ years	Non-melanoma skin cancer (basal-cell	Rate	1990	4.563149	6.303821275	3.190357829
Incidence	Mauritius	Female	55+ years	Non-melanoma skin cancer (basal-cell	Rate	1990	3.6130514	4.893751322	2.496553879
Incidence	Mauritius	Both	55+ years	Non-melanoma skin cancer (basal-cell	Rate	1990	4.0455145	5.523157611	2.850028696
Incidence	Mauritius	Male	55+ years	Non-melanoma skin cancer (basal-cell	Number	2021	7.435559	10.04149276	5.227286077
Incidence	Mauritius	Female	55+ years	Non-melanoma skin cancer (basal-cell	Number	2021	6.8292677	9.344354194	4.761369541
Incidence	Mauritius	Both	55+ years	Non-melanoma skin cancer (basal-cell	Number	2021	14.264827	19.15606107	9.921128761
Incidence	Mauritius	Male	55+ years	Non-melanoma skin cancer (basal-cell	Percent	2021	1.33E-05	1.80E-05	9.19E-06
Incidence	Mauritius	Female	55+ years	Non-melanoma skin cancer (basal-cell	Percent	2021	1.01E-05	1.41E-05	6.84E-06
Incidence	Mauritius	Both	55+ years	Non-melanoma skin cancer (basal-cell	Percent	2021	1.15E-05	1.57E-05	7.92E-06
Incidence	Mauritius	Male	55+ years	Non-melanoma skin cancer (basal-cell	Rate	2021	4.760341	6.428693482	3.346576125
Incidence	Mauritius	Female	55+ years	Non-melanoma skin cancer (basal-cell	Rate	2021	3.749586	5.130485596	2.614213603

Incidence	Mauritius	Both	55+ years	Non-melanoma skin cancer (basal-cell	Rate	2021	4.2162222	5.661913182	2.93236535
Incidence	Seychelles	Male	55+ years	Non-melanoma skin cancer (basal-cell	Number	1990	0.1984278	0.302179483	0.112431515
Incidence	Seychelles	Female	55+ years	Non-melanoma skin cancer (basal-cell	Number	1990	0.2072078	0.310899527	0.115301578
Incidence	Seychelles	Both	55+ years	Non-melanoma skin cancer (basal-cell	Number	1990	0.4056356	0.602556748	0.227829254
Incidence	Seychelles	Male	55+ years	Non-melanoma skin cancer (basal-cell	Percent	1990	1.26E-05	1.93E-05	6.89E-06
Incidence	Seychelles	Female	55+ years	Non-melanoma skin cancer (basal-cell	Percent	1990	1.00E-05	1.54E-05	5.40E-06
Incidence	Seychelles	Both	55+ years	Non-melanoma skin cancer (basal-cell	Percent	1990	1.11E-05	1.69E-05	6.09E-06
Incidence	Seychelles	Male	55+ years	Non-melanoma skin cancer (basal-cell	Rate	1990	4.7429162	7.222837092	2.687391302
Incidence	Seychelles	Female	55+ years	Non-melanoma skin cancer (basal-cell	Rate	1990	3.8312543	5.748505212	2.131916145
Incidence	Seychelles	Both	55+ years	Non-melanoma skin cancer (basal-cell	Rate	1990	4.2288859	6.281853466	2.375195355
Incidence	Seychelles	Male	55+ years	Non-melanoma skin cancer (basal-cell	Number	2021	0.48162	0.723923586	0.268407083
Incidence	Seychelles	Female	55+ years	Non-melanoma skin cancer (basal-cell	Number	2021	0.354827	0.547524359	0.185447
Incidence	Seychelles	Both	55+ years	Non-melanoma skin cancer (basal-cell	Number	2021	0.836447	1.266760418	0.463742066
Incidence	Seychelles	Male	55+ years	Non-melanoma skin cancer (basal-cell	Percent	2021	1.15E-05	1.76E-05	6.35E-06
Incidence	Seychelles	Female	55+ years	Non-melanoma skin cancer (basal-cell	Percent	2021	7.72E-06	1.19E-05	3.82E-06
Incidence	Seychelles	Both	55+ years	Non-melanoma skin cancer (basal-cell	Percent	2021	9.53E-06	1.43E-05	5.12E-06
Incidence	Seychelles	Male	55+ years	Non-melanoma skin cancer (basal-cell	Rate	2021	4.7984549	7.212562397	2.674181191
Incidence	Seychelles	Female	55+ years	Non-melanoma skin cancer (basal-cell	Rate	2021	3.3142911	5.114197145	1.732183237
Incidence	Seychelles	Both	55+ years	Non-melanoma skin cancer (basal-cell	Rate	2021	4.0324399	6.106944102	2.235661009
Incidence	United Rep	Male	55+ years	Non-melanoma skin cancer (basal-cell	Number	1990	163.81388	214.2589389	119.4342002
Incidence	United Rep	Female	55+ years	Non-melanoma skin cancer (basal-cell	Number	1990	104.06026	141.0887466	73.29533006
Incidence	United Rep	Both	55+ years	Non-melanoma skin cancer (basal-cell	Number	1990	267.87414	353.5656038	193.5809723
Incidence	United Rep	Male	55+ years	Non-melanoma skin cancer (basal-cell	Percent	1990	3.54E-05	4.62E-05	2.49E-05
Incidence	United Rep	Female	55+ years	Non-melanoma skin cancer (basal-cell	Percent	1990	2.18E-05	2.98E-05	1.54E-05
Incidence	United Rep	Both	55+ years	Non-melanoma skin cancer (basal-cell	Percent	1990	2.85E-05	3.72E-05	2.01E-05
Incidence	United Rep	Male	55+ years	Non-melanoma skin cancer (basal-cell	Rate	1990	18.410828	24.0802823	13.42305376
Incidence	United Rep	Female	55+ years	Non-melanoma skin cancer (basal-cell	Rate	1990	11.380132	15.42960351	8.015649084
Incidence	United Rep	Both	55+ years	Non-melanoma skin cancer (basal-cell	Rate	1990	14.847482	19.59711021	10.7296287
Incidence	United Rep	Male	55+ years	Non-melanoma skin cancer (basal-cell	Number	2021	365.96009	477.952884	264.2863996
Incidence	United Rep	Female	55+ years	Non-melanoma skin cancer (basal-cell	Number	2021	244.08869	327.191201	172.8010858
Incidence	United Rep	Both	55+ years	Non-melanoma skin cancer (basal-cell	Number	2021	610.04878	799.7234273	439.4594755
Incidence	United Rep	Male	55+ years	Non-melanoma skin cancer (basal-cell	Percent	2021	3.45E-05	4.56E-05	2.45E-05
Incidence	United Rep	Female	55+ years	Non-melanoma skin cancer (basal-cell	Percent	2021	2.07E-05	2.80E-05	1.45E-05
Incidence	United Rep	Both	55+ years	Non-melanoma skin cancer (basal-cell	Percent	2021	2.72E-05	3.57E-05	1.95E-05
Incidence	United Rep	Male	55+ years	Non-melanoma skin cancer (basal-cell	Rate	2021	18.301078	23.90165835	13.21653962
Incidence	United Rep	Female	55+ years	Non-melanoma skin cancer (basal-cell	Rate	2021	11.499963	15.41524394	8.141328011
Incidence	United Rep	Both	55+ years	Non-melanoma skin cancer (basal-cell	Rate	2021	14.799174	19.40049141	10.66084785
Incidence	Somalia	Male	55+ years	Non-melanoma skin cancer (basal-cell	Number	1990	29.269883	39.7833861	21.13591393
Incidence	Somalia	Female	55+ years	Non-melanoma skin cancer (basal-cell	Number	1990	20.99077	28.74903139	14.4169734
Incidence	Somalia	Both	55+ years	Non-melanoma skin cancer (basal-cell	Number	1990	50.260653	68.06560189	36.18347818
Incidence	Somalia	Male	55+ years	Non-melanoma skin cancer (basal-cell	Percent	1990	3.07E-05	4.17E-05	2.18E-05
Incidence	Somalia	Female	55+ years	Non-melanoma skin cancer (basal-cell	Percent	1990	1.95E-05	2.65E-05	1.34E-05
Incidence	Somalia	Both	55+ years	Non-melanoma skin cancer (basal-cell	Percent	1990	2.48E-05	3.35E-05	1.75E-05
Incidence	Somalia	Male	55+ years	Non-melanoma skin cancer (basal-cell	Rate	1990	17.245607	23.44008872	12.45313047
Incidence	Somalia	Female	55+ years	Non-melanoma skin cancer (basal-cell	Rate	1990	11.027672	15.10353812	7.574074561
Incidence	Somalia	Both	55+ years	Non-melanoma skin cancer (basal-cell	Rate	1990	13.958577	18.90343404	10.04901116
Incidence	Somalia	Male	55+ years	Non-melanoma skin cancer (basal-cell	Number	2021	68.150641	94.65078705	49.42439953
Incidence	Somalia	Female	55+ years	Non-melanoma skin cancer (basal-cell	Number	2021	62.406348	86.74679465	42.51849994
Incidence	Somalia	Both	55+ years	Non-melanoma skin cancer (basal-cell	Number	2021	130.55699	177.8158976	93.16315756
Incidence	Somalia	Male	55+ years	Non-melanoma skin cancer (basal-cell	Percent	2021	2.80E-05	3.90E-05	1.96E-05
Incidence	Somalia	Female	55+ years	Non-melanoma skin cancer (basal-cell	Percent	2021	1.79E-05	2.48E-05	1.19E-05
Incidence	Somalia	Both	55+ years	Non-melanoma skin cancer (basal-cell	Percent	2021	2.20E-05	3.01E-05	1.53E-05
Incidence	Somalia	Male	55+ years	Non-melanoma skin cancer (basal-cell	Rate	2021	17.567267	24.3982388	12.74018251
Incidence	Somalia	Female	55+ years	Non-melanoma skin cancer (basal-cell	Rate	2021	11.241962	15.62668234	7.659338824
Incidence	Somalia	Both	55+ years	Non-melanoma skin cancer (basal-cell	Rate	2021	13.843963	18.85518955	9.878807345
Incidence	Uganda	Male	55+ years	Non-melanoma skin cancer (basal-cell	Number	1990	139.00543	167.7745359	113.2571378

Incidence	Uganda	Female	55+ years	Non-melanoma skin cancer (basal-cell	Number	1990	88.546598	108.3317214	69.75556503
Incidence	Uganda	Both	55+ years	Non-melanoma skin cancer (basal-cell	Number	1990	227.55203	272.3400437	183.4230927
Incidence	Uganda	Male	55+ years	Non-melanoma skin cancer (basal-cell	Percent	1990	4.89E-05	5.92E-05	3.91E-05
Incidence	Uganda	Female	55+ years	Non-melanoma skin cancer (basal-cell	Percent	1990	2.97E-05	3.67E-05	2.28E-05
Incidence	Uganda	Both	55+ years	Non-melanoma skin cancer (basal-cell	Percent	1990	3.91E-05	4.66E-05	3.10E-05
Incidence	Uganda	Male	55+ years	Non-melanoma skin cancer (basal-cell	Rate	1990	27.080511	32.68519975	22.0643267
Incidence	Uganda	Female	55+ years	Non-melanoma skin cancer (basal-cell	Rate	1990	16.624987	20.33972569	13.09689387
Incidence	Uganda	Both	55+ years	Non-melanoma skin cancer (basal-cell	Rate	1990	21.756247	26.03842866	17.53707992
Incidence	Uganda	Male	55+ years	Non-melanoma skin cancer (basal-cell	Number	2021	240.34297	301.396649	184.8402562
Incidence	Uganda	Female	55+ years	Non-melanoma skin cancer (basal-cell	Number	2021	207.78434	268.037482	154.9399593
Incidence	Uganda	Both	55+ years	Non-melanoma skin cancer (basal-cell	Number	2021	448.1273	562.5520063	342.9894741
Incidence	Uganda	Male	55+ years	Non-melanoma skin cancer (basal-cell	Percent	2021	4.16E-05	5.25E-05	3.13E-05
Incidence	Uganda	Female	55+ years	Non-melanoma skin cancer (basal-cell	Percent	2021	2.63E-05	3.37E-05	1.96E-05
Incidence	Uganda	Both	55+ years	Non-melanoma skin cancer (basal-cell	Percent	2021	3.28E-05	4.14E-05	2.48E-05
Incidence	Uganda	Male	55+ years	Non-melanoma skin cancer (basal-cell	Rate	2021	23.111371	28.98229167	17.77423284
Incidence	Uganda	Female	55+ years	Non-melanoma skin cancer (basal-cell	Rate	2021	15.604597	20.12960654	11.63598611
Incidence	Uganda	Both	55+ years	Non-melanoma skin cancer (basal-cell	Rate	2021	18.896427	23.72143525	14.463023
Incidence	Zambia	Male	55+ years	Non-melanoma skin cancer (basal-cell	Number	1990	45.371442	59.61788434	32.4133499
Incidence	Zambia	Female	55+ years	Non-melanoma skin cancer (basal-cell	Number	1990	24.319736	33.55744264	16.94437101
Incidence	Zambia	Both	55+ years	Non-melanoma skin cancer (basal-cell	Number	1990	69.691178	92.41776562	49.98291822
Incidence	Zambia	Male	55+ years	Non-melanoma skin cancer (basal-cell	Percent	1990	3.59E-05	4.78E-05	2.54E-05
Incidence	Zambia	Female	55+ years	Non-melanoma skin cancer (basal-cell	Percent	1990	2.20E-05	3.06E-05	1.54E-05
Incidence	Zambia	Both	55+ years	Non-melanoma skin cancer (basal-cell	Percent	1990	2.94E-05	3.89E-05	2.09E-05
Incidence	Zambia	Male	55+ years	Non-melanoma skin cancer (basal-cell	Rate	1990	18.559513	24.38712154	13.25891236
Incidence	Zambia	Female	55+ years	Non-melanoma skin cancer (basal-cell	Rate	1990	11.266387	15.54585715	7.849667629
Incidence	Zambia	Both	55+ years	Non-melanoma skin cancer (basal-cell	Rate	1990	15.139539	20.07660695	10.8581656
Incidence	Zambia	Male	55+ years	Non-melanoma skin cancer (basal-cell	Number	2021	92.762518	123.6779621	66.96225857
Incidence	Zambia	Female	55+ years	Non-melanoma skin cancer (basal-cell	Number	2021	65.05508	88.31846153	45.5143077
Incidence	Zambia	Both	55+ years	Non-melanoma skin cancer (basal-cell	Number	2021	157.8176	211.7572334	113.8733496
Incidence	Zambia	Male	55+ years	Non-melanoma skin cancer (basal-cell	Percent	2021	3.14E-05	4.28E-05	2.21E-05
Incidence	Zambia	Female	55+ years	Non-melanoma skin cancer (basal-cell	Percent	2021	1.94E-05	2.62E-05	1.36E-05
Incidence	Zambia	Both	55+ years	Non-melanoma skin cancer (basal-cell	Percent	2021	2.51E-05	3.36E-05	1.76E-05
Incidence	Zambia	Male	55+ years	Non-melanoma skin cancer (basal-cell	Rate	2021	18.031394	24.04080992	13.01627956
Incidence	Zambia	Female	55+ years	Non-melanoma skin cancer (basal-cell	Rate	2021	11.499756	15.61201314	8.045542887
Incidence	Zambia	Both	55+ years	Non-melanoma skin cancer (basal-cell	Rate	2021	14.610598	19.60427574	10.54228235
Incidence	Namibia	Male	55+ years	Non-melanoma skin cancer (basal-cell	Number	1990	40.040427	52.4386177	29.42316211
Incidence	Namibia	Female	55+ years	Non-melanoma skin cancer (basal-cell	Number	1990	27.785215	35.76714533	20.17545223
Incidence	Namibia	Both	55+ years	Non-melanoma skin cancer (basal-cell	Number	1990	67.825643	87.63953992	49.42911753
Incidence	Namibia	Male	55+ years	Non-melanoma skin cancer (basal-cell	Percent	1990	0.0001631	0.000213853	0.000117342
Incidence	Namibia	Female	55+ years	Non-melanoma skin cancer (basal-cell	Percent	1990	9.61E-05	0.000123828	6.88E-05
Incidence	Namibia	Both	55+ years	Non-melanoma skin cancer (basal-cell	Percent	1990	0.0001268	0.000163172	9.13E-05
Incidence	Namibia	Male	55+ years	Non-melanoma skin cancer (basal-cell	Rate	1990	82.117684	107.5447525	60.34306062
Incidence	Namibia	Female	55+ years	Non-melanoma skin cancer (basal-cell	Rate	1990	46.502602	59.86152415	33.76655615
Incidence	Namibia	Both	55+ years	Non-melanoma skin cancer (basal-cell	Rate	1990	62.506572	80.76660997	45.55275233
Incidence	Namibia	Male	55+ years	Non-melanoma skin cancer (basal-cell	Number	2021	76.738674	99.26145728	56.25315134
Incidence	Namibia	Female	55+ years	Non-melanoma skin cancer (basal-cell	Number	2021	62.968077	82.72349577	46.30512419
Incidence	Namibia	Both	55+ years	Non-melanoma skin cancer (basal-cell	Number	2021	139.70675	181.0498707	102.7018473
Incidence	Namibia	Male	55+ years	Non-melanoma skin cancer (basal-cell	Percent	2021	0.0001416	0.000185435	0.000100974
Incidence	Namibia	Female	55+ years	Non-melanoma skin cancer (basal-cell	Percent	2021	8.56E-05	0.000110943	6.21E-05
Incidence	Namibia	Both	55+ years	Non-melanoma skin cancer (basal-cell	Percent	2021	0.0001093	0.000141047	7.88E-05
Incidence	Namibia	Male	55+ years	Non-melanoma skin cancer (basal-cell	Rate	2021	81.990227	106.0543403	60.10279334
Incidence	Namibia	Female	55+ years	Non-melanoma skin cancer (basal-cell	Rate	2021	47.576218	62.50264007	34.98634196
Incidence	Namibia	Both	55+ years	Non-melanoma skin cancer (basal-cell	Rate	2021	61.831673	80.12938702	45.45397375
Incidence	South Afri	Male	55+ years	Non-melanoma skin cancer (basal-cell	Number	1990	1259.5123	1618.562511	933.1039359
Incidence	South Afri	Female	55+ years	Non-melanoma skin cancer (basal-cell	Number	1990	1077.9887	1380.745886	795.7221979
Incidence	South Afri	Both	55+ years	Non-melanoma skin cancer (basal-cell	Number	1990	2337.501	2994.447454	1728.177428

Incidence	South Afri	Male	55+ years	Non-melanoma skin cancer (basal-cell	Percent	1990	0.0001651	0.000212674	0.000118288
Incidence	South Afri	Female	55+ years	Non-melanoma skin cancer (basal-cell	Percent	1990	9.92E-05	0.000127174	7.13E-05
Incidence	South Afri	Both	55+ years	Non-melanoma skin cancer (basal-cell	Percent	1990	0.0001264	0.000161687	9.11E-05
Incidence	South Afri	Male	55+ years	Non-melanoma skin cancer (basal-cell	Rate	1990	89.084748	114.4802084	65.99802749
Incidence	South Afri	Female	55+ years	Non-melanoma skin cancer (basal-cell	Rate	1990	54.953515	70.38741525	40.56418299
Incidence	South Afri	Both	55+ years	Non-melanoma skin cancer (basal-cell	Rate	1990	69.249574	88.71192226	51.19807376
Incidence	South Afri	Male	55+ years	Non-melanoma skin cancer (basal-cell	Number	2021	3545.3656	4574.462033	2596.891611
Incidence	South Afri	Female	55+ years	Non-melanoma skin cancer (basal-cell	Number	2021	3212.4818	4102.774218	2349.225022
Incidence	South Afri	Both	55+ years	Non-melanoma skin cancer (basal-cell	Number	2021	6757.8473	8651.331156	4972.127223
Incidence	South Afri	Male	55+ years	Non-melanoma skin cancer (basal-cell	Percent	2021	0.0001881	0.000241536	0.000134351
Incidence	South Afri	Female	55+ years	Non-melanoma skin cancer (basal-cell	Percent	2021	0.0001176	0.00014938	8.49E-05
Incidence	South Afri	Both	55+ years	Non-melanoma skin cancer (basal-cell	Percent	2021	0.0001464	0.000186343	0.000105441
Incidence	South Afri	Male	55+ years	Non-melanoma skin cancer (basal-cell	Rate	2021	107.7204	138.9878886	78.90249814
Incidence	South Afri	Female	55+ years	Non-melanoma skin cancer (basal-cell	Rate	2021	69.87779	89.24340023	51.1002599
Incidence	South Afri	Both	55+ years	Non-melanoma skin cancer (basal-cell	Rate	2021	85.666506	109.6694373	63.02965233
Incidence	Botswana	Male	55+ years	Non-melanoma skin cancer (basal-cell	Number	1990	31.897286	40.78584139	23.37724056
Incidence	Botswana	Female	55+ years	Non-melanoma skin cancer (basal-cell	Number	1990	23.350585	30.75108265	16.93313586
Incidence	Botswana	Both	55+ years	Non-melanoma skin cancer (basal-cell	Number	1990	55.247871	71.5234727	40.46517475
Incidence	Botswana	Male	55+ years	Non-melanoma skin cancer (basal-cell	Percent	1990	0.0001449	0.000187237	0.000103708
Incidence	Botswana	Female	55+ years	Non-melanoma skin cancer (basal-cell	Percent	1990	8.87E-05	0.0001168	6.27E-05
Incidence	Botswana	Both	55+ years	Non-melanoma skin cancer (basal-cell	Percent	1990	0.0001143	0.000147518	8.23E-05
Incidence	Botswana	Male	55+ years	Non-melanoma skin cancer (basal-cell	Rate	1990	76.710427	98.08669267	56.22039738
Incidence	Botswana	Female	55+ years	Non-melanoma skin cancer (basal-cell	Rate	1990	45.439574	59.84073303	32.95140122
Incidence	Botswana	Both	55+ years	Non-melanoma skin cancer (basal-cell	Rate	1990	59.425716	76.93207978	43.52515244
Incidence	Botswana	Male	55+ years	Non-melanoma skin cancer (basal-cell	Number	2021	78.716066	103.4957662	57.63173526
Incidence	Botswana	Female	55+ years	Non-melanoma skin cancer (basal-cell	Number	2021	62.862313	80.30004134	46.52506123
Incidence	Botswana	Both	55+ years	Non-melanoma skin cancer (basal-cell	Number	2021	141.57838	183.9558739	104.364905
Incidence	Botswana	Male	55+ years	Non-melanoma skin cancer (basal-cell	Percent	2021	0.0001394	0.000183176	0.000100609
Incidence	Botswana	Female	55+ years	Non-melanoma skin cancer (basal-cell	Percent	2021	8.26E-05	0.000106981	5.91E-05
Incidence	Botswana	Both	55+ years	Non-melanoma skin cancer (basal-cell	Percent	2021	0.0001068	0.000140567	7.65E-05
Incidence	Botswana	Male	55+ years	Non-melanoma skin cancer (basal-cell	Rate	2021	75.455176	99.20835231	55.24428398
Incidence	Botswana	Female	55+ years	Non-melanoma skin cancer (basal-cell	Rate	2021	45.518819	58.14553802	33.68895795
Incidence	Botswana	Both	55+ years	Non-melanoma skin cancer (basal-cell	Rate	2021	58.401275	75.88204995	43.05066627
Incidence	Lesotho	Male	55+ years	Non-melanoma skin cancer (basal-cell	Number	1990	40.111469	51.52299977	29.73930846
Incidence	Lesotho	Female	55+ years	Non-melanoma skin cancer (basal-cell	Number	1990	43.357822	56.37101382	31.79861418
Incidence	Lesotho	Both	55+ years	Non-melanoma skin cancer (basal-cell	Number	1990	83.469291	107.1770168	61.29912706
Incidence	Lesotho	Male	55+ years	Non-melanoma skin cancer (basal-cell	Percent	1990	0.0001537	0.000198314	0.000112969
Incidence	Lesotho	Female	55+ years	Non-melanoma skin cancer (basal-cell	Percent	1990	9.52E-05	0.000122977	6.87E-05
Incidence	Lesotho	Both	55+ years	Non-melanoma skin cancer (basal-cell	Percent	1990	0.0001165	0.000150609	8.51E-05
Incidence	Lesotho	Male	55+ years	Non-melanoma skin cancer (basal-cell	Rate	1990	79.29432	101.8531946	58.79012453
Incidence	Lesotho	Female	55+ years	Non-melanoma skin cancer (basal-cell	Rate	1990	48.578055	63.15802098	35.62713184
Incidence	Lesotho	Both	55+ years	Non-melanoma skin cancer (basal-cell	Rate	1990	59.689361	76.64288968	43.83535179
Incidence	Lesotho	Male	55+ years	Non-melanoma skin cancer (basal-cell	Number	2021	51.740731	68.81925157	38.02348339
Incidence	Lesotho	Female	55+ years	Non-melanoma skin cancer (basal-cell	Number	2021	53.721205	68.53694651	39.34660882
Incidence	Lesotho	Both	55+ years	Non-melanoma skin cancer (basal-cell	Number	2021	105.46194	135.7788012	77.32050433
Incidence	Lesotho	Male	55+ years	Non-melanoma skin cancer (basal-cell	Percent	2021	0.0001368	0.000185651	9.82E-05
Incidence	Lesotho	Female	55+ years	Non-melanoma skin cancer (basal-cell	Percent	2021	8.27E-05	0.000107356	5.98E-05
Incidence	Lesotho	Both	55+ years	Non-melanoma skin cancer (basal-cell	Percent	2021	0.0001026	0.000134511	7.44E-05
Incidence	Lesotho	Male	55+ years	Non-melanoma skin cancer (basal-cell	Rate	2021	76.4687	101.7093993	56.19569479
Incidence	Lesotho	Female	55+ years	Non-melanoma skin cancer (basal-cell	Rate	2021	47.586443	60.71028205	34.8533724
Incidence	Lesotho	Both	55+ years	Non-melanoma skin cancer (basal-cell	Rate	2021	58.410044	75.20102562	42.82392522
Incidence	Kenya	Male	55+ years	Non-melanoma skin cancer (basal-cell	Number	1990	105.80831	137.9133013	76.93853504
Incidence	Kenya	Female	55+ years	Non-melanoma skin cancer (basal-cell	Number	1990	93.154033	124.9303412	66.24047281
Incidence	Kenya	Both	55+ years	Non-melanoma skin cancer (basal-cell	Number	1990	198.96235	261.4512322	145.1080453
Incidence	Kenya	Male	55+ years	Non-melanoma skin cancer (basal-cell	Percent	1990	2.60E-05	3.46E-05	1.82E-05
Incidence	Kenya	Female	55+ years	Non-melanoma skin cancer (basal-cell	Percent	1990	2.09E-05	2.76E-05	1.44E-05

Incidence	Kenya	Both	55+ years	Non-melanoma skin cancer (basal-cell	Percent	1990	2.34E-05	3.05E-05	1.63E-05
Incidence	Kenya	Male	55+ years	Non-melanoma skin cancer (basal-cell	Rate	1990	16.231241	21.15622072	11.80254997
Incidence	Kenya	Female	55+ years	Non-melanoma skin cancer (basal-cell	Rate	1990	13.68123	18.34811279	9.728522749
Incidence	Kenya	Both	55+ years	Non-melanoma skin cancer (basal-cell	Rate	1990	14.928484	19.61713158	10.88770397
Incidence	Kenya	Male	55+ years	Non-melanoma skin cancer (basal-cell	Number	2021	253.62877	335.1002019	181.5739761
Incidence	Kenya	Female	55+ years	Non-melanoma skin cancer (basal-cell	Number	2021	250.31838	335.348467	177.828335
Incidence	Kenya	Both	55+ years	Non-melanoma skin cancer (basal-cell	Number	2021	503.94715	670.7884043	360.156654
Incidence	Kenya	Male	55+ years	Non-melanoma skin cancer (basal-cell	Percent	2021	2.18E-05	2.88E-05	1.52E-05
Incidence	Kenya	Female	55+ years	Non-melanoma skin cancer (basal-cell	Percent	2021	1.81E-05	2.39E-05	1.25E-05
Incidence	Kenya	Both	55+ years	Non-melanoma skin cancer (basal-cell	Percent	2021	1.98E-05	2.60E-05	1.38E-05
Incidence	Kenya	Male	55+ years	Non-melanoma skin cancer (basal-cell	Rate	2021	14.500344	19.15819035	10.38086154
Incidence	Kenya	Female	55+ years	Non-melanoma skin cancer (basal-cell	Rate	2021	12.568392	16.83772076	8.92869401
Incidence	Kenya	Both	55+ years	Non-melanoma skin cancer (basal-cell	Rate	2021	13.47174	17.93181536	9.62786861
Incidence	Eswatini	Male	55+ years	Non-melanoma skin cancer (basal-cell	Number	1990	15.150372	20.04043431	11.15269335
Incidence	Eswatini	Female	55+ years	Non-melanoma skin cancer (basal-cell	Number	1990	11.811121	15.2349752	8.79297789
Incidence	Eswatini	Both	55+ years	Non-melanoma skin cancer (basal-cell	Number	1990	26.961492	35.28329606	19.99071125
Incidence	Eswatini	Male	55+ years	Non-melanoma skin cancer (basal-cell	Percent	1990	0.0001582	0.0002057	0.000113605
Incidence	Eswatini	Female	55+ years	Non-melanoma skin cancer (basal-cell	Percent	1990	9.67E-05	0.00012565	6.99E-05
Incidence	Eswatini	Both	55+ years	Non-melanoma skin cancer (basal-cell	Percent	1990	0.0001237	0.000160832	8.91E-05
Incidence	Eswatini	Male	55+ years	Non-melanoma skin cancer (basal-cell	Rate	1990	75.412276	99.75298172	55.51348833
Incidence	Eswatini	Female	55+ years	Non-melanoma skin cancer (basal-cell	Rate	1990	47.122515	60.78257716	35.08111107
Incidence	Eswatini	Both	55+ years	Non-melanoma skin cancer (basal-cell	Rate	1990	59.709071	78.13858348	44.27154019
Incidence	Eswatini	Male	55+ years	Non-melanoma skin cancer (basal-cell	Number	2021	26.423331	35.15915413	19.61603313
Incidence	Eswatini	Female	55+ years	Non-melanoma skin cancer (basal-cell	Number	2021	25.767824	33.90000707	18.62232684
Incidence	Eswatini	Both	55+ years	Non-melanoma skin cancer (basal-cell	Number	2021	52.191155	68.18852509	38.63070561
Incidence	Eswatini	Male	55+ years	Non-melanoma skin cancer (basal-cell	Percent	2021	0.0001436	0.000192351	0.000103455
Incidence	Eswatini	Female	55+ years	Non-melanoma skin cancer (basal-cell	Percent	2021	8.66E-05	0.000112082	6.13E-05
Incidence	Eswatini	Both	55+ years	Non-melanoma skin cancer (basal-cell	Percent	2021	0.0001083	0.0001404	7.77E-05
Incidence	Eswatini	Male	55+ years	Non-melanoma skin cancer (basal-cell	Rate	2021	74.938984	99.7145782	55.63286482
Incidence	Eswatini	Female	55+ years	Non-melanoma skin cancer (basal-cell	Rate	2021	46.613621	61.32462284	33.68752011
Incidence	Eswatini	Both	55+ years	Non-melanoma skin cancer (basal-cell	Rate	2021	57.644692	75.31365251	42.66728946
Incidence	Zimbabwe	Male	55+ years	Non-melanoma skin cancer (basal-cell	Number	1990	206.8805	264.0519739	155.5912157
Incidence	Zimbabwe	Female	55+ years	Non-melanoma skin cancer (basal-cell	Number	1990	140.73669	182.4248302	105.341813
Incidence	Zimbabwe	Both	55+ years	Non-melanoma skin cancer (basal-cell	Number	1990	347.61719	444.8864377	258.7814512
Incidence	Zimbabwe	Male	55+ years	Non-melanoma skin cancer (basal-cell	Percent	1990	0.0001253	0.000161382	9.04E-05
Incidence	Zimbabwe	Female	55+ years	Non-melanoma skin cancer (basal-cell	Percent	1990	8.94E-05	0.000116793	6.57E-05
Incidence	Zimbabwe	Both	55+ years	Non-melanoma skin cancer (basal-cell	Percent	1990	0.0001078	0.000137294	7.88E-05
Incidence	Zimbabwe	Male	55+ years	Non-melanoma skin cancer (basal-cell	Rate	1990	62.555733	79.84302343	47.04707522
Incidence	Zimbabwe	Female	55+ years	Non-melanoma skin cancer (basal-cell	Rate	1990	42.375801	54.92809391	31.71840691
Incidence	Zimbabwe	Both	55+ years	Non-melanoma skin cancer (basal-cell	Rate	1990	52.444428	67.1192765	39.04192689
Incidence	Zimbabwe	Male	55+ years	Non-melanoma skin cancer (basal-cell	Number	2021	261.79911	340.7292963	193.6315672
Incidence	Zimbabwe	Female	55+ years	Non-melanoma skin cancer (basal-cell	Number	2021	242.24683	318.6507319	175.0232005
Incidence	Zimbabwe	Both	55+ years	Non-melanoma skin cancer (basal-cell	Number	2021	504.04594	657.7501813	370.6548766
Incidence	Zimbabwe	Male	55+ years	Non-melanoma skin cancer (basal-cell	Percent	2021	0.0001023	0.00013461	7.37E-05
Incidence	Zimbabwe	Female	55+ years	Non-melanoma skin cancer (basal-cell	Percent	2021	6.73E-05	8.87E-05	4.91E-05
Incidence	Zimbabwe	Both	55+ years	Non-melanoma skin cancer (basal-cell	Percent	2021	8.19E-05	0.000107064	5.90E-05
Incidence	Zimbabwe	Male	55+ years	Non-melanoma skin cancer (basal-cell	Rate	2021	58.338731	75.92735701	43.14842688
Incidence	Zimbabwe	Female	55+ years	Non-melanoma skin cancer (basal-cell	Rate	2021	36.786869	48.38933387	26.57849249
Incidence	Zimbabwe	Both	55+ years	Non-melanoma skin cancer (basal-cell	Rate	2021	45.521447	59.40279924	33.47461977
Incidence	Burkina Fa	Male	55+ years	Non-melanoma skin cancer (basal-cell	Number	1990	49.649365	66.68773911	35.42926223
Incidence	Burkina Fa	Female	55+ years	Non-melanoma skin cancer (basal-cell	Number	1990	31.449918	42.57887864	22.09978508
Incidence	Burkina Fa	Both	55+ years	Non-melanoma skin cancer (basal-cell	Number	1990	81.099283	108.5527453	57.25340439
Incidence	Burkina Fa	Male	55+ years	Non-melanoma skin cancer (basal-cell	Percent	1990	2.63E-05	3.53E-05	1.87E-05
Incidence	Burkina Fa	Female	55+ years	Non-melanoma skin cancer (basal-cell	Percent	1990	1.62E-05	2.19E-05	1.12E-05
Incidence	Burkina Fa	Both	55+ years	Non-melanoma skin cancer (basal-cell	Percent	1990	2.11E-05	2.84E-05	1.49E-05
Incidence	Burkina Fa	Male	55+ years	Non-melanoma skin cancer (basal-cell	Rate	1990	14.154702	19.01222827	10.10064563

Incidence	Burkina Fa	Female	55+ years	Non-melanoma skin cancer (basal-cell	Rate	1990	8.4434755	11.43130873	5.933210885
Incidence	Burkina Fa	Both	55+ years	Non-melanoma skin cancer (basal-cell	Rate	1990	11.213355	15.00926337	7.916256956
Incidence	Burkina Fa	Male	55+ years	Non-melanoma skin cancer (basal-cell	Number	2021	101.34525	137.6900772	70.4155111
Incidence	Burkina Fa	Female	55+ years	Non-melanoma skin cancer (basal-cell	Number	2021	67.954469	90.27768732	49.05928349
Incidence	Burkina Fa	Both	55+ years	Non-melanoma skin cancer (basal-cell	Number	2021	169.29972	225.4342945	118.9046596
Incidence	Burkina Fa	Male	55+ years	Non-melanoma skin cancer (basal-cell	Percent	2021	2.62E-05	3.53E-05	1.81E-05
Incidence	Burkina Fa	Female	55+ years	Non-melanoma skin cancer (basal-cell	Percent	2021	1.55E-05	2.07E-05	1.10E-05
Incidence	Burkina Fa	Both	55+ years	Non-melanoma skin cancer (basal-cell	Percent	2021	2.05E-05	2.73E-05	1.44E-05
Incidence	Burkina Fa	Male	55+ years	Non-melanoma skin cancer (basal-cell	Rate	2021	14.22605	19.32785108	9.884376126
Incidence	Burkina Fa	Female	55+ years	Non-melanoma skin cancer (basal-cell	Rate	2021	8.5907144	11.41278632	6.202010001
Incidence	Burkina Fa	Both	55+ years	Non-melanoma skin cancer (basal-cell	Rate	2021	11.261015	14.9948209	7.90897445
Incidence	Cameroon	Male	55+ years	Non-melanoma skin cancer (basal-cell	Number	1990	50.556744	68.47233417	35.70929688
Incidence	Cameroon	Female	55+ years	Non-melanoma skin cancer (basal-cell	Number	1990	31.325497	42.84136444	22.02272752
Incidence	Cameroon	Both	55+ years	Non-melanoma skin cancer (basal-cell	Number	1990	81.882241	112.1246924	57.89502784
Incidence	Cameroon	Male	55+ years	Non-melanoma skin cancer (basal-cell	Percent	1990	2.53E-05	3.37E-05	1.78E-05
Incidence	Cameroon	Female	55+ years	Non-melanoma skin cancer (basal-cell	Percent	1990	1.57E-05	2.18E-05	1.11E-05
Incidence	Cameroon	Both	55+ years	Non-melanoma skin cancer (basal-cell	Percent	1990	2.05E-05	2.77E-05	1.44E-05
Incidence	Cameroon	Male	55+ years	Non-melanoma skin cancer (basal-cell	Rate	1990	13.845417	18.75176254	9.779311074
Incidence	Cameroon	Female	55+ years	Non-melanoma skin cancer (basal-cell	Rate	1990	8.3469519	11.41545512	5.868147781
Incidence	Cameroon	Both	55+ years	Non-melanoma skin cancer (basal-cell	Rate	1990	11.058531	15.14289745	7.818959865
Incidence	Cameroon	Male	55+ years	Non-melanoma skin cancer (basal-cell	Number	2021	136.90363	186.042	95.39335292
Incidence	Cameroon	Female	55+ years	Non-melanoma skin cancer (basal-cell	Number	2021	87.850185	119.6204377	61.187402
Incidence	Cameroon	Both	55+ years	Non-melanoma skin cancer (basal-cell	Number	2021	224.75382	303.6488261	158.6079768
Incidence	Cameroon	Male	55+ years	Non-melanoma skin cancer (basal-cell	Percent	2021	2.55E-05	3.54E-05	1.75E-05
Incidence	Cameroon	Female	55+ years	Non-melanoma skin cancer (basal-cell	Percent	2021	1.49E-05	2.07E-05	1.03E-05
Incidence	Cameroon	Both	55+ years	Non-melanoma skin cancer (basal-cell	Percent	2021	1.99E-05	2.76E-05	1.39E-05
Incidence	Cameroon	Male	55+ years	Non-melanoma skin cancer (basal-cell	Rate	2021	13.858465	18.83263751	9.656467016
Incidence	Cameroon	Female	55+ years	Non-melanoma skin cancer (basal-cell	Rate	2021	8.3857892	11.41843667	5.840678135
Incidence	Cameroon	Both	55+ years	Non-melanoma skin cancer (basal-cell	Rate	2021	11.04182	14.91781445	7.792173605
Incidence	Cabo Verde	Male	55+ years	Non-melanoma skin cancer (basal-cell	Number	1990	2.5567124	3.304295084	1.884350041
Incidence	Cabo Verde	Female	55+ years	Non-melanoma skin cancer (basal-cell	Number	1990	1.9980721	2.638472303	1.450670284
Incidence	Cabo Verde	Both	55+ years	Non-melanoma skin cancer (basal-cell	Number	1990	4.5547845	5.960091642	3.410963526
Incidence	Cabo Verde	Male	55+ years	Non-melanoma skin cancer (basal-cell	Percent	1990	3.17E-05	4.17E-05	2.31E-05
Incidence	Cabo Verde	Female	55+ years	Non-melanoma skin cancer (basal-cell	Percent	1990	1.91E-05	2.58E-05	1.36E-05
Incidence	Cabo Verde	Both	55+ years	Non-melanoma skin cancer (basal-cell	Percent	1990	2.46E-05	3.23E-05	1.81E-05
Incidence	Cabo Verde	Male	55+ years	Non-melanoma skin cancer (basal-cell	Rate	1990	15.026179	19.41983326	11.07460522
Incidence	Cabo Verde	Female	55+ years	Non-melanoma skin cancer (basal-cell	Rate	1990	8.8985118	11.75056536	6.460631012
Incidence	Cabo Verde	Both	55+ years	Non-melanoma skin cancer (basal-cell	Rate	1990	11.54014	15.10066901	8.642120678
Incidence	Cabo Verde	Male	55+ years	Non-melanoma skin cancer (basal-cell	Number	2021	4.5215529	5.997828752	3.204267559
Incidence	Cabo Verde	Female	55+ years	Non-melanoma skin cancer (basal-cell	Number	2021	3.7632581	4.957092893	2.726292274
Incidence	Cabo Verde	Both	55+ years	Non-melanoma skin cancer (basal-cell	Number	2021	8.284811	10.9208085	5.945657418
Incidence	Cabo Verde	Male	55+ years	Non-melanoma skin cancer (basal-cell	Percent	2021	2.94E-05	3.93E-05	2.09E-05
Incidence	Cabo Verde	Female	55+ years	Non-melanoma skin cancer (basal-cell	Percent	2021	1.79E-05	2.37E-05	1.29E-05
Incidence	Cabo Verde	Both	55+ years	Non-melanoma skin cancer (basal-cell	Percent	2021	2.27E-05	3.03E-05	1.64E-05
Incidence	Cabo Verde	Male	55+ years	Non-melanoma skin cancer (basal-cell	Rate	2021	13.681231	18.14811951	9.695413625
Incidence	Cabo Verde	Female	55+ years	Non-melanoma skin cancer (basal-cell	Rate	2021	8.6319447	11.37029423	6.253412229
Incidence	Cabo Verde	Both	55+ years	Non-melanoma skin cancer (basal-cell	Rate	2021	10.809163	14.24833893	7.757277498
Incidence	Chad	Male	55+ years	Non-melanoma skin cancer (basal-cell	Number	1990	33.549925	44.83761474	23.7243792
Incidence	Chad	Female	55+ years	Non-melanoma skin cancer (basal-cell	Number	1990	20.829684	27.77282454	14.83827762
Incidence	Chad	Both	55+ years	Non-melanoma skin cancer (basal-cell	Number	1990	54.379609	72.32092061	38.67033726
Incidence	Chad	Male	55+ years	Non-melanoma skin cancer (basal-cell	Percent	1990	2.67E-05	3.57E-05	1.88E-05
Incidence	Chad	Female	55+ years	Non-melanoma skin cancer (basal-cell	Percent	1990	1.66E-05	2.22E-05	1.17E-05
Incidence	Chad	Both	55+ years	Non-melanoma skin cancer (basal-cell	Percent	1990	2.17E-05	2.91E-05	1.54E-05
Incidence	Chad	Male	55+ years	Non-melanoma skin cancer (basal-cell	Rate	1990	14.811439	19.79466742	10.47371049
Incidence	Chad	Female	55+ years	Non-melanoma skin cancer (basal-cell	Rate	1990	8.7929099	11.723843	6.263735867
Incidence	Chad	Both	55+ years	Non-melanoma skin cancer (basal-cell	Rate	1990	11.73478	15.60640369	8.344817638

Incidence	Chad	Male	55+ years	Non-melanoma skin cancer (basal-cell	Number	2021	72.361647	97.52711711	51.56362784
Incidence	Chad	Female	55+ years	Non-melanoma skin cancer (basal-cell	Number	2021	34.921635	48.28945082	24.21395898
Incidence	Chad	Both	55+ years	Non-melanoma skin cancer (basal-cell	Number	2021	107.28328	145.6834861	75.59714851
Incidence	Chad	Male	55+ years	Non-melanoma skin cancer (basal-cell	Percent	2021	2.50E-05	3.38E-05	1.78E-05
Incidence	Chad	Female	55+ years	Non-melanoma skin cancer (basal-cell	Percent	2021	1.49E-05	2.07E-05	1.03E-05
Incidence	Chad	Both	55+ years	Non-melanoma skin cancer (basal-cell	Percent	2021	2.05E-05	2.77E-05	1.45E-05
Incidence	Chad	Male	55+ years	Non-melanoma skin cancer (basal-cell	Rate	2021	14.105549	19.01108669	10.05136446
Incidence	Chad	Female	55+ years	Non-melanoma skin cancer (basal-cell	Rate	2021	8.4042999	11.62142122	5.827372476
Incidence	Chad	Both	55+ years	Non-melanoma skin cancer (basal-cell	Rate	2021	11.554195	15.6898206	8.141661965
Incidence	Côte d'Ivo	Male	55+ years	Non-melanoma skin cancer (basal-cell	Number	1990	33.039427	43.81218651	23.56008612
Incidence	Côte d'Ivo	Female	55+ years	Non-melanoma skin cancer (basal-cell	Number	1990	15.187943	21.30251416	10.35780013
Incidence	Côte d'Ivo	Both	55+ years	Non-melanoma skin cancer (basal-cell	Number	1990	48.22737	64.55304778	33.71226442
Incidence	Côte d'Ivo	Male	55+ years	Non-melanoma skin cancer (basal-cell	Percent	1990	2.07E-05	2.76E-05	1.46E-05
Incidence	Côte d'Ivo	Female	55+ years	Non-melanoma skin cancer (basal-cell	Percent	1990	1.07E-05	1.48E-05	7.18E-06
Incidence	Côte d'Ivo	Both	55+ years	Non-melanoma skin cancer (basal-cell	Percent	1990	1.60E-05	2.14E-05	1.11E-05
Incidence	Côte d'Ivo	Male	55+ years	Non-melanoma skin cancer (basal-cell	Rate	1990	9.4799012	12.57089577	6.760022966
Incidence	Côte d'Ivo	Female	55+ years	Non-melanoma skin cancer (basal-cell	Rate	1990	5.1940322	7.285117299	3.542201091
Incidence	Côte d'Ivo	Both	55+ years	Non-melanoma skin cancer (basal-cell	Rate	1990	7.5245665	10.07174345	5.259879895
Incidence	Côte d'Ivo	Male	55+ years	Non-melanoma skin cancer (basal-cell	Number	2021	91.62243	125.6157475	62.31316218
Incidence	Côte d'Ivo	Female	55+ years	Non-melanoma skin cancer (basal-cell	Number	2021	43.261825	59.91825759	29.00510527
Incidence	Côte d'Ivo	Both	55+ years	Non-melanoma skin cancer (basal-cell	Number	2021	134.88425	187.5740385	92.11964099
Incidence	Côte d'Ivo	Male	55+ years	Non-melanoma skin cancer (basal-cell	Percent	2021	1.94E-05	2.68E-05	1.32E-05
Incidence	Côte d'Ivo	Female	55+ years	Non-melanoma skin cancer (basal-cell	Percent	2021	9.52E-06	1.32E-05	6.41E-06
Incidence	Côte d'Ivo	Both	55+ years	Non-melanoma skin cancer (basal-cell	Percent	2021	1.46E-05	2.00E-05	9.97E-06
Incidence	Côte d'Ivo	Male	55+ years	Non-melanoma skin cancer (basal-cell	Rate	2021	9.7241305	13.33193117	6.613460542
Incidence	Côte d'Ivo	Female	55+ years	Non-melanoma skin cancer (basal-cell	Rate	2021	5.0838523	7.041209521	3.408494031
Incidence	Côte d'Ivo	Both	55+ years	Non-melanoma skin cancer (basal-cell	Rate	2021	7.5220591	10.46039808	5.137214739
Incidence	Gambia	Male	55+ years	Non-melanoma skin cancer (basal-cell	Number	1990	3.6800441	4.831973229	2.735246634
Incidence	Gambia	Female	55+ years	Non-melanoma skin cancer (basal-cell	Number	1990	2.2334515	2.94391967	1.638188239
Incidence	Gambia	Both	55+ years	Non-melanoma skin cancer (basal-cell	Number	1990	5.9134957	7.737479468	4.39465763
Incidence	Gambia	Male	55+ years	Non-melanoma skin cancer (basal-cell	Percent	1990	2.52E-05	3.26E-05	1.85E-05
Incidence	Gambia	Female	55+ years	Non-melanoma skin cancer (basal-cell	Percent	1990	1.83E-05	2.41E-05	1.32E-05
Incidence	Gambia	Both	55+ years	Non-melanoma skin cancer (basal-cell	Percent	1990	2.21E-05	2.86E-05	1.62E-05
Incidence	Gambia	Male	55+ years	Non-melanoma skin cancer (basal-cell	Rate	1990	12.226962	16.05425132	9.087868413
Incidence	Gambia	Female	55+ years	Non-melanoma skin cancer (basal-cell	Rate	1990	8.8349052	11.64531703	6.480211261
Incidence	Gambia	Both	55+ years	Non-melanoma skin cancer (basal-cell	Rate	1990	10.67849	13.97220915	7.93579819
Incidence	Gambia	Male	55+ years	Non-melanoma skin cancer (basal-cell	Number	2021	10.032768	13.42342809	7.050934555
Incidence	Gambia	Female	55+ years	Non-melanoma skin cancer (basal-cell	Number	2021	6.7679547	9.085701937	4.889704977
Incidence	Gambia	Both	55+ years	Non-melanoma skin cancer (basal-cell	Number	2021	16.800722	22.46135817	11.97545553
Incidence	Gambia	Male	55+ years	Non-melanoma skin cancer (basal-cell	Percent	2021	2.62E-05	3.54E-05	1.80E-05
Incidence	Gambia	Female	55+ years	Non-melanoma skin cancer (basal-cell	Percent	2021	1.61E-05	2.19E-05	1.13E-05
Incidence	Gambia	Both	55+ years	Non-melanoma skin cancer (basal-cell	Percent	2021	2.09E-05	2.84E-05	1.47E-05
Incidence	Gambia	Male	55+ years	Non-melanoma skin cancer (basal-cell	Rate	2021	13.211872	17.67693806	9.285179062
Incidence	Gambia	Female	55+ years	Non-melanoma skin cancer (basal-cell	Rate	2021	8.5993869	11.5443247	6.212876269
Incidence	Gambia	Both	55+ years	Non-melanoma skin cancer (basal-cell	Rate	2021	10.86439	14.52490819	7.744072769
Incidence	Ghana	Male	55+ years	Non-melanoma skin cancer (basal-cell	Number	1990	68.348509	91.45249206	48.11280359
Incidence	Ghana	Female	55+ years	Non-melanoma skin cancer (basal-cell	Number	1990	43.771166	59.57206548	30.42930071
Incidence	Ghana	Both	55+ years	Non-melanoma skin cancer (basal-cell	Number	1990	112.11968	150.8605843	78.79305073
Incidence	Ghana	Male	55+ years	Non-melanoma skin cancer (basal-cell	Percent	1990	2.94E-05	4.01E-05	2.04E-05
Incidence	Ghana	Female	55+ years	Non-melanoma skin cancer (basal-cell	Percent	1990	1.86E-05	2.57E-05	1.31E-05
Incidence	Ghana	Both	55+ years	Non-melanoma skin cancer (basal-cell	Percent	1990	2.40E-05	3.27E-05	1.66E-05
Incidence	Ghana	Male	55+ years	Non-melanoma skin cancer (basal-cell	Rate	1990	13.786583	18.44688975	9.704837602
Incidence	Ghana	Female	55+ years	Non-melanoma skin cancer (basal-cell	Rate	1990	8.3303689	11.33753864	5.79119374
Incidence	Ghana	Both	55+ years	Non-melanoma skin cancer (basal-cell	Rate	1990	10.979187	14.77284543	7.715716898
Incidence	Ghana	Male	55+ years	Non-melanoma skin cancer (basal-cell	Number	2021	166.18548	225.4437133	118.4370564
Incidence	Ghana	Female	55+ years	Non-melanoma skin cancer (basal-cell	Number	2021	129.43039	178.0955389	91.59811005

Incidence	Ghana	Both	55+ years	Non-melanoma skin cancer (basal-cell	Number	2021	295.61587	397.5961803	211.4545169
Incidence	Ghana	Male	55+ years	Non-melanoma skin cancer (basal-cell	Percent	2021	2.87E-05	3.89E-05	2.02E-05
Incidence	Ghana	Female	55+ years	Non-melanoma skin cancer (basal-cell	Percent	2021	1.68E-05	2.27E-05	1.18E-05
Incidence	Ghana	Both	55+ years	Non-melanoma skin cancer (basal-cell	Percent	2021	2.19E-05	2.96E-05	1.54E-05
Incidence	Ghana	Male	55+ years	Non-melanoma skin cancer (basal-cell	Rate	2021	13.981893	18.96754134	9.964614803
Incidence	Ghana	Female	55+ years	Non-melanoma skin cancer (basal-cell	Rate	2021	8.412247	11.57520809	5.953361836
Incidence	Ghana	Both	55+ years	Non-melanoma skin cancer (basal-cell	Rate	2021	10.839653	14.57907003	7.753621296
Incidence	Mali	Male	55+ years	Non-melanoma skin cancer (basal-cell	Number	1990	57.258401	76.23075348	41.60617722
Incidence	Mali	Female	55+ years	Non-melanoma skin cancer (basal-cell	Number	1990	44.51536	58.2115431	33.01166182
Incidence	Mali	Both	55+ years	Non-melanoma skin cancer (basal-cell	Number	1990	101.77376	134.592266	74.56219642
Incidence	Mali	Male	55+ years	Non-melanoma skin cancer (basal-cell	Percent	1990	3.20E-05	4.19E-05	2.32E-05
Incidence	Mali	Female	55+ years	Non-melanoma skin cancer (basal-cell	Percent	1990	2.59E-05	3.34E-05	1.89E-05
Incidence	Mali	Both	55+ years	Non-melanoma skin cancer (basal-cell	Percent	1990	2.90E-05	3.75E-05	2.12E-05
Incidence	Mali	Male	55+ years	Non-melanoma skin cancer (basal-cell	Rate	1990	16.817987	22.39056267	12.22060226
Incidence	Mali	Female	55+ years	Non-melanoma skin cancer (basal-cell	Rate	1990	13.563574	17.73672172	10.05846312
Incidence	Mali	Both	55+ years	Non-melanoma skin cancer (basal-cell	Rate	1990	15.220619	20.1287403	11.15103514
Incidence	Mali	Male	55+ years	Non-melanoma skin cancer (basal-cell	Number	2021	131.48045	176.5274831	95.26320204
Incidence	Mali	Female	55+ years	Non-melanoma skin cancer (basal-cell	Number	2021	89.361065	118.9665561	63.07912316
Incidence	Mali	Both	55+ years	Non-melanoma skin cancer (basal-cell	Number	2021	220.84152	298.6620624	157.9666593
Incidence	Mali	Male	55+ years	Non-melanoma skin cancer (basal-cell	Percent	2021	3.20E-05	4.32E-05	2.24E-05
Incidence	Mali	Female	55+ years	Non-melanoma skin cancer (basal-cell	Percent	2021	2.19E-05	2.94E-05	1.51E-05
Incidence	Mali	Both	55+ years	Non-melanoma skin cancer (basal-cell	Percent	2021	2.70E-05	3.60E-05	1.88E-05
Incidence	Mali	Male	55+ years	Non-melanoma skin cancer (basal-cell	Rate	2021	17.538033	23.5468073	12.707054
Incidence	Mali	Female	55+ years	Non-melanoma skin cancer (basal-cell	Rate	2021	12.808219	17.05160604	9.041199416
Incidence	Mali	Both	55+ years	Non-melanoma skin cancer (basal-cell	Rate	2021	15.258094	20.63476962	10.91402636
Incidence	Burundi	Male	55+ years	Non-melanoma skin cancer (basal-cell	Number	1990	32.690553	42.59981367	23.95151205
Incidence	Burundi	Female	55+ years	Non-melanoma skin cancer (basal-cell	Number	1990	23.924707	32.61467568	16.57548653
Incidence	Burundi	Both	55+ years	Non-melanoma skin cancer (basal-cell	Number	1990	56.61526	74.64658677	40.82609648
Incidence	Burundi	Male	55+ years	Non-melanoma skin cancer (basal-cell	Percent	1990	3.71E-05	4.94E-05	2.68E-05
Incidence	Burundi	Female	55+ years	Non-melanoma skin cancer (basal-cell	Percent	1990	2.25E-05	3.07E-05	1.55E-05
Incidence	Burundi	Both	55+ years	Non-melanoma skin cancer (basal-cell	Percent	1990	2.91E-05	3.91E-05	2.06E-05
Incidence	Burundi	Male	55+ years	Non-melanoma skin cancer (basal-cell	Rate	1990	19.20358	25.02462729	14.06995971
Incidence	Burundi	Female	55+ years	Non-melanoma skin cancer (basal-cell	Rate	1990	11.607389	15.82344308	8.041817437
Incidence	Burundi	Both	55+ years	Non-melanoma skin cancer (basal-cell	Rate	1990	15.043337	19.8344721	10.84797185
Incidence	Burundi	Male	55+ years	Non-melanoma skin cancer (basal-cell	Number	2021	75.75018	102.1501009	54.63040529
Incidence	Burundi	Female	55+ years	Non-melanoma skin cancer (basal-cell	Number	2021	42.963704	58.68833052	29.82574685
Incidence	Burundi	Both	55+ years	Non-melanoma skin cancer (basal-cell	Number	2021	118.71388	160.3213663	84.90947724
Incidence	Burundi	Male	55+ years	Non-melanoma skin cancer (basal-cell	Percent	2021	3.49E-05	4.73E-05	2.48E-05
Incidence	Burundi	Female	55+ years	Non-melanoma skin cancer (basal-cell	Percent	2021	2.13E-05	2.92E-05	1.46E-05
Incidence	Burundi	Both	55+ years	Non-melanoma skin cancer (basal-cell	Percent	2021	2.83E-05	3.78E-05	2.00E-05
Incidence	Burundi	Male	55+ years	Non-melanoma skin cancer (basal-cell	Rate	2021	17.918239	24.16297827	12.92248646
Incidence	Burundi	Female	55+ years	Non-melanoma skin cancer (basal-cell	Rate	2021	11.372869	15.53531501	7.895136371
Incidence	Burundi	Both	55+ years	Non-melanoma skin cancer (basal-cell	Rate	2021	14.829443	20.02694623	10.60668066
Incidence	Djibouti	Male	55+ years	Non-melanoma skin cancer (basal-cell	Number	1990	1.8708229	2.60393885	1.33243998
Incidence	Djibouti	Female	55+ years	Non-melanoma skin cancer (basal-cell	Number	1990	1.1467515	1.559335823	0.798779244
Incidence	Djibouti	Both	55+ years	Non-melanoma skin cancer (basal-cell	Number	1990	3.0175743	4.116580245	2.144061844
Incidence	Djibouti	Male	55+ years	Non-melanoma skin cancer (basal-cell	Percent	1990	3.45E-05	4.74E-05	2.38E-05
Incidence	Djibouti	Female	55+ years	Non-melanoma skin cancer (basal-cell	Percent	1990	2.18E-05	3.02E-05	1.50E-05
Incidence	Djibouti	Both	55+ years	Non-melanoma skin cancer (basal-cell	Percent	1990	2.83E-05	3.85E-05	1.95E-05
Incidence	Djibouti	Male	55+ years	Non-melanoma skin cancer (basal-cell	Rate	1990	16.855754	23.46098775	12.00502771
Incidence	Djibouti	Female	55+ years	Non-melanoma skin cancer (basal-cell	Rate	1990	11.074293	15.05866166	7.713890873
Incidence	Djibouti	Both	55+ years	Non-melanoma skin cancer (basal-cell	Rate	1990	14.065262	19.18785581	9.9937198
Incidence	Djibouti	Male	55+ years	Non-melanoma skin cancer (basal-cell	Number	2021	9.8159864	13.3636173	6.885075351
Incidence	Djibouti	Female	55+ years	Non-melanoma skin cancer (basal-cell	Number	2021	5.1471867	7.078567648	3.570819276
Incidence	Djibouti	Both	55+ years	Non-melanoma skin cancer (basal-cell	Number	2021	14.963173	20.1992025	10.56464641
Incidence	Djibouti	Male	55+ years	Non-melanoma skin cancer (basal-cell	Percent	2021	3.28E-05	4.53E-05	2.25E-05

Incidence	Djibouti	Female	55+ years	Non-melanoma skin cancer (basal-cell	Percent	2021	1.98E-05	2.71E-05	1.36E-05
Incidence	Djibouti	Both	55+ years	Non-melanoma skin cancer (basal-cell	Percent	2021	2.68E-05	3.65E-05	1.83E-05
Incidence	Djibouti	Male	55+ years	Non-melanoma skin cancer (basal-cell	Rate	2021	17.031735	23.18723499	11.94630589
Incidence	Djibouti	Female	55+ years	Non-melanoma skin cancer (basal-cell	Rate	2021	11.172447	15.36468835	7.750794801
Incidence	Djibouti	Both	55+ years	Non-melanoma skin cancer (basal-cell	Rate	2021	14.428751	19.47777075	10.18732105
Incidence	Benin	Male	55+ years	Non-melanoma skin cancer (basal-cell	Number	1990	23.117102	30.37353363	16.46497381
Incidence	Benin	Female	55+ years	Non-melanoma skin cancer (basal-cell	Number	1990	14.325094	18.91422255	10.35709686
Incidence	Benin	Both	55+ years	Non-melanoma skin cancer (basal-cell	Number	1990	37.442196	48.89129525	27.18066732
Incidence	Benin	Male	55+ years	Non-melanoma skin cancer (basal-cell	Percent	1990	2.97E-05	3.96E-05	2.09E-05
Incidence	Benin	Female	55+ years	Non-melanoma skin cancer (basal-cell	Percent	1990	1.76E-05	2.29E-05	1.25E-05
Incidence	Benin	Both	55+ years	Non-melanoma skin cancer (basal-cell	Percent	1990	2.35E-05	3.08E-05	1.70E-05
Incidence	Benin	Male	55+ years	Non-melanoma skin cancer (basal-cell	Rate	1990	14.926383	19.61175734	10.63119869
Incidence	Benin	Female	55+ years	Non-melanoma skin cancer (basal-cell	Rate	1990	8.6793051	11.4597719	6.275170302
Incidence	Benin	Both	55+ years	Non-melanoma skin cancer (basal-cell	Rate	1990	11.703504	15.28220886	8.496003899
Incidence	Benin	Male	55+ years	Non-melanoma skin cancer (basal-cell	Number	2021	54.937579	73.54364997	39.12273314
Incidence	Benin	Female	55+ years	Non-melanoma skin cancer (basal-cell	Number	2021	37.782934	50.73463715	26.59837338
Incidence	Benin	Both	55+ years	Non-melanoma skin cancer (basal-cell	Number	2021	92.720514	122.752545	66.22803345
Incidence	Benin	Male	55+ years	Non-melanoma skin cancer (basal-cell	Percent	2021	2.78E-05	3.74E-05	1.92E-05
Incidence	Benin	Female	55+ years	Non-melanoma skin cancer (basal-cell	Percent	2021	1.64E-05	2.19E-05	1.13E-05
Incidence	Benin	Both	55+ years	Non-melanoma skin cancer (basal-cell	Percent	2021	2.17E-05	2.88E-05	1.51E-05
Incidence	Benin	Male	55+ years	Non-melanoma skin cancer (basal-cell	Rate	2021	14.098646	18.87352709	10.04007775
Incidence	Benin	Female	55+ years	Non-melanoma skin cancer (basal-cell	Rate	2021	8.6381482	11.59923969	6.081070555
Incidence	Benin	Both	55+ years	Non-melanoma skin cancer (basal-cell	Rate	2021	11.210832	14.84200269	8.007627467
Incidence	Guinea	Male	55+ years	Non-melanoma skin cancer (basal-cell	Number	1990	35.389085	46.85982811	25.78328364
Incidence	Guinea	Female	55+ years	Non-melanoma skin cancer (basal-cell	Number	1990	22.884916	30.45717584	16.2553954
Incidence	Guinea	Both	55+ years	Non-melanoma skin cancer (basal-cell	Number	1990	58.274	76.02963124	42.26428602
Incidence	Guinea	Male	55+ years	Non-melanoma skin cancer (basal-cell	Percent	1990	2.49E-05	3.30E-05	1.78E-05
Incidence	Guinea	Female	55+ years	Non-melanoma skin cancer (basal-cell	Percent	1990	1.67E-05	2.24E-05	1.19E-05
Incidence	Guinea	Both	55+ years	Non-melanoma skin cancer (basal-cell	Percent	1990	2.09E-05	2.77E-05	1.49E-05
Incidence	Guinea	Male	55+ years	Non-melanoma skin cancer (basal-cell	Rate	1990	12.625906	16.71836957	9.198805932
Incidence	Guinea	Female	55+ years	Non-melanoma skin cancer (basal-cell	Rate	1990	8.3795317	11.15218751	5.952069176
Incidence	Guinea	Both	55+ years	Non-melanoma skin cancer (basal-cell	Rate	1990	10.530284	13.73877834	7.637280988
Incidence	Guinea	Male	55+ years	Non-melanoma skin cancer (basal-cell	Number	2021	58.746457	79.06198428	42.54096774
Incidence	Guinea	Female	55+ years	Non-melanoma skin cancer (basal-cell	Number	2021	36.439426	48.49037836	25.87429724
Incidence	Guinea	Both	55+ years	Non-melanoma skin cancer (basal-cell	Number	2021	95.185883	127.0899925	68.60118353
Incidence	Guinea	Male	55+ years	Non-melanoma skin cancer (basal-cell	Percent	2021	2.24E-05	3.07E-05	1.58E-05
Incidence	Guinea	Female	55+ years	Non-melanoma skin cancer (basal-cell	Percent	2021	1.48E-05	2.01E-05	1.03E-05
Incidence	Guinea	Both	55+ years	Non-melanoma skin cancer (basal-cell	Percent	2021	1.88E-05	2.55E-05	1.32E-05
Incidence	Guinea	Male	55+ years	Non-melanoma skin cancer (basal-cell	Rate	2021	12.451998	16.75811114	9.01705506
Incidence	Guinea	Female	55+ years	Non-melanoma skin cancer (basal-cell	Rate	2021	8.2484153	10.97626454	5.85689658
Incidence	Guinea	Both	55+ years	Non-melanoma skin cancer (basal-cell	Rate	2021	10.419246	13.91153675	7.509229219
Incidence	Guinea-Bis	Male	55+ years	Non-melanoma skin cancer (basal-cell	Number	1990	4.5451239	6.084239704	3.148525491
Incidence	Guinea-Bis	Female	55+ years	Non-melanoma skin cancer (basal-cell	Number	1990	2.6920719	3.615488217	1.8724048
Incidence	Guinea-Bis	Both	55+ years	Non-melanoma skin cancer (basal-cell	Number	1990	7.2371958	9.616445139	5.04070087
Incidence	Guinea-Bis	Male	55+ years	Non-melanoma skin cancer (basal-cell	Percent	1990	2.85E-05	3.80E-05	1.95E-05
Incidence	Guinea-Bis	Female	55+ years	Non-melanoma skin cancer (basal-cell	Percent	1990	1.70E-05	2.30E-05	1.17E-05
Incidence	Guinea-Bis	Both	55+ years	Non-melanoma skin cancer (basal-cell	Percent	1990	2.28E-05	3.05E-05	1.56E-05
Incidence	Guinea-Bis	Male	55+ years	Non-melanoma skin cancer (basal-cell	Rate	1990	14.36814	19.23362516	9.9531843
Incidence	Guinea-Bis	Female	55+ years	Non-melanoma skin cancer (basal-cell	Rate	1990	8.3605501	11.22832959	5.814976278
Incidence	Guinea-Bis	Both	55+ years	Non-melanoma skin cancer (basal-cell	Rate	1990	11.337694	15.06499477	7.896694794
Incidence	Guinea-Bis	Male	55+ years	Non-melanoma skin cancer (basal-cell	Number	2021	7.3131739	10.17031731	5.13505202
Incidence	Guinea-Bis	Female	55+ years	Non-melanoma skin cancer (basal-cell	Number	2021	5.1211772	7.086002171	3.47352479
Incidence	Guinea-Bis	Both	55+ years	Non-melanoma skin cancer (basal-cell	Number	2021	12.434351	17.13668945	8.623139642
Incidence	Guinea-Bis	Male	55+ years	Non-melanoma skin cancer (basal-cell	Percent	2021	2.63E-05	3.60E-05	1.85E-05
Incidence	Guinea-Bis	Female	55+ years	Non-melanoma skin cancer (basal-cell	Percent	2021	1.55E-05	2.14E-05	1.06E-05
Incidence	Guinea-Bis	Both	55+ years	Non-melanoma skin cancer (basal-cell	Percent	2021	2.04E-05	2.78E-05	1.42E-05

Incidence	Guinea-Bis	Male	55+ years	Non-melanoma skin cancer (basal-cell	Rate	2021	13.770995	19.1511092	9.669505783
Incidence	Guinea-Bis	Female	55+ years	Non-melanoma skin cancer (basal-cell	Rate	2021	8.2914303	11.47257578	5.623802452
Incidence	Guinea-Bis	Both	55+ years	Non-melanoma skin cancer (basal-cell	Rate	2021	10.824685	14.91829018	7.506846629
Incidence	Liberia	Male	55+ years	Non-melanoma skin cancer (basal-cell	Number	1990	15.179981	20.08856258	10.63982312
Incidence	Liberia	Female	55+ years	Non-melanoma skin cancer (basal-cell	Number	1990	7.4382895	9.883840949	5.342134636
Incidence	Liberia	Both	55+ years	Non-melanoma skin cancer (basal-cell	Number	1990	22.61827	29.71943718	16.12678811
Incidence	Liberia	Male	55+ years	Non-melanoma skin cancer (basal-cell	Percent	1990	2.97E-05	3.97E-05	2.07E-05
Incidence	Liberia	Female	55+ years	Non-melanoma skin cancer (basal-cell	Percent	1990	1.71E-05	2.26E-05	1.21E-05
Incidence	Liberia	Both	55+ years	Non-melanoma skin cancer (basal-cell	Percent	1990	2.39E-05	3.16E-05	1.67E-05
Incidence	Liberia	Male	55+ years	Non-melanoma skin cancer (basal-cell	Rate	1990	14.724969	19.48641868	10.32090012
Incidence	Liberia	Female	55+ years	Non-melanoma skin cancer (basal-cell	Rate	1990	8.7151139	11.58045805	6.259142209
Incidence	Liberia	Both	55+ years	Non-melanoma skin cancer (basal-cell	Rate	1990	12.002942	15.77135171	8.558077519
Incidence	Liberia	Male	55+ years	Non-melanoma skin cancer (basal-cell	Number	2021	23.447865	30.98978192	16.97856011
Incidence	Liberia	Female	55+ years	Non-melanoma skin cancer (basal-cell	Number	2021	13.331616	18.43710173	9.476720152
Incidence	Liberia	Both	55+ years	Non-melanoma skin cancer (basal-cell	Number	2021	36.779481	48.9285129	26.37791653
Incidence	Liberia	Male	55+ years	Non-melanoma skin cancer (basal-cell	Percent	2021	2.50E-05	3.33E-05	1.80E-05
Incidence	Liberia	Female	55+ years	Non-melanoma skin cancer (basal-cell	Percent	2021	1.38E-05	1.93E-05	9.58E-06
Incidence	Liberia	Both	55+ years	Non-melanoma skin cancer (basal-cell	Percent	2021	1.93E-05	2.59E-05	1.37E-05
Incidence	Liberia	Male	55+ years	Non-melanoma skin cancer (basal-cell	Rate	2021	13.749366	18.17179716	9.955892917
Incidence	Liberia	Female	55+ years	Non-melanoma skin cancer (basal-cell	Rate	2021	8.3539885	11.55323853	5.938395851
Incidence	Liberia	Both	55+ years	Non-melanoma skin cancer (basal-cell	Rate	2021	11.141191	14.82135913	7.990362893
Incidence	Mauritania	Male	55+ years	Non-melanoma skin cancer (basal-cell	Number	1990	11.21402	14.71287385	7.840208319
Incidence	Mauritania	Female	55+ years	Non-melanoma skin cancer (basal-cell	Number	1990	7.5236273	10.04693261	5.370465983
Incidence	Mauritania	Both	55+ years	Non-melanoma skin cancer (basal-cell	Number	1990	18.737647	24.50230787	13.41305561
Incidence	Mauritania	Male	55+ years	Non-melanoma skin cancer (basal-cell	Percent	1990	3.02E-05	4.01E-05	2.11E-05
Incidence	Mauritania	Female	55+ years	Non-melanoma skin cancer (basal-cell	Percent	1990	1.78E-05	2.40E-05	1.24E-05
Incidence	Mauritania	Both	55+ years	Non-melanoma skin cancer (basal-cell	Percent	1990	2.36E-05	3.11E-05	1.67E-05
Incidence	Mauritania	Male	55+ years	Non-melanoma skin cancer (basal-cell	Rate	1990	14.071398	18.46177455	9.837925608
Incidence	Mauritania	Female	55+ years	Non-melanoma skin cancer (basal-cell	Rate	1990	8.7161808	11.63944966	6.22172666
Incidence	Mauritania	Both	55+ years	Non-melanoma skin cancer (basal-cell	Rate	1990	11.286947	14.75939066	8.079586984
Incidence	Mauritania	Male	55+ years	Non-melanoma skin cancer (basal-cell	Number	2021	25.937345	34.37127831	18.58778634
Incidence	Mauritania	Female	55+ years	Non-melanoma skin cancer (basal-cell	Number	2021	15.263863	20.62467044	10.72993201
Incidence	Mauritania	Both	55+ years	Non-melanoma skin cancer (basal-cell	Number	2021	41.201208	54.80480364	29.4540707
Incidence	Mauritania	Male	55+ years	Non-melanoma skin cancer (basal-cell	Percent	2021	2.75E-05	3.65E-05	1.95E-05
Incidence	Mauritania	Female	55+ years	Non-melanoma skin cancer (basal-cell	Percent	2021	1.51E-05	2.05E-05	1.07E-05
Incidence	Mauritania	Both	55+ years	Non-melanoma skin cancer (basal-cell	Percent	2021	2.11E-05	2.81E-05	1.52E-05
Incidence	Mauritania	Male	55+ years	Non-melanoma skin cancer (basal-cell	Rate	2021	14.433989	19.12742617	10.34400023
Incidence	Mauritania	Female	55+ years	Non-melanoma skin cancer (basal-cell	Rate	2021	8.5243725	11.5182093	5.99231891
Incidence	Mauritania	Both	55+ years	Non-melanoma skin cancer (basal-cell	Rate	2021	11.48441	15.27627083	8.210016847
Incidence	Niger	Male	55+ years	Non-melanoma skin cancer (basal-cell	Number	1990	32.38668	44.45838239	22.48821691
Incidence	Niger	Female	55+ years	Non-melanoma skin cancer (basal-cell	Number	1990	17.014853	22.78519863	12.13602042
Incidence	Niger	Both	55+ years	Non-melanoma skin cancer (basal-cell	Number	1990	49.401533	66.77458555	34.12906965
Incidence	Niger	Male	55+ years	Non-melanoma skin cancer (basal-cell	Percent	1990	2.57E-05	3.54E-05	1.76E-05
Incidence	Niger	Female	55+ years	Non-melanoma skin cancer (basal-cell	Percent	1990	1.59E-05	2.13E-05	1.11E-05
Incidence	Niger	Both	55+ years	Non-melanoma skin cancer (basal-cell	Percent	1990	2.12E-05	2.87E-05	1.46E-05
Incidence	Niger	Male	55+ years	Non-melanoma skin cancer (basal-cell	Rate	1990	13.716655	18.82935544	9.524382276
Incidence	Niger	Female	55+ years	Non-melanoma skin cancer (basal-cell	Rate	1990	8.3657625	11.20289172	5.966966753
Incidence	Niger	Both	55+ years	Non-melanoma skin cancer (basal-cell	Rate	1990	11.240424	15.19334742	7.7654516
Incidence	Niger	Male	55+ years	Non-melanoma skin cancer (basal-cell	Number	2021	94.405372	126.3055828	66.32121333
Incidence	Niger	Female	55+ years	Non-melanoma skin cancer (basal-cell	Number	2021	58.197868	79.55176513	40.59991852
Incidence	Niger	Both	55+ years	Non-melanoma skin cancer (basal-cell	Number	2021	152.60324	204.3123184	107.263719
Incidence	Niger	Male	55+ years	Non-melanoma skin cancer (basal-cell	Percent	2021	2.54E-05	3.45E-05	1.72E-05
Incidence	Niger	Female	55+ years	Non-melanoma skin cancer (basal-cell	Percent	2021	1.51E-05	2.06E-05	1.05E-05
Incidence	Niger	Both	55+ years	Non-melanoma skin cancer (basal-cell	Percent	2021	2.01E-05	2.73E-05	1.40E-05
Incidence	Niger	Male	55+ years	Non-melanoma skin cancer (basal-cell	Rate	2021	13.784148	18.44190386	9.683573863
Incidence	Niger	Female	55+ years	Non-melanoma skin cancer (basal-cell	Rate	2021	8.2278121	11.24675175	5.739875209

Incidence	Niger	Both	55+ years	Non-melanoma skin cancer (basal-cell	Rate	2021	10.961186	14.67534586	7.704538753
Incidence	Nigeria	Male	55+ years	Non-melanoma skin cancer (basal-cell	Number	1990	463.59566	621.9725981	328.156367
Incidence	Nigeria	Female	55+ years	Non-melanoma skin cancer (basal-cell	Number	1990	264.93465	348.885595	190.0719717
Incidence	Nigeria	Both	55+ years	Non-melanoma skin cancer (basal-cell	Number	1990	728.53031	968.8716847	513.855573
Incidence	Nigeria	Male	55+ years	Non-melanoma skin cancer (basal-cell	Percent	1990	2.25E-05	3.00E-05	1.55E-05
Incidence	Nigeria	Female	55+ years	Non-melanoma skin cancer (basal-cell	Percent	1990	1.26E-05	1.70E-05	8.78E-06
Incidence	Nigeria	Both	55+ years	Non-melanoma skin cancer (basal-cell	Percent	1990	1.75E-05	2.32E-05	1.21E-05
Incidence	Nigeria	Male	55+ years	Non-melanoma skin cancer (basal-cell	Rate	1990	11.966357	16.05439124	8.470390368
Incidence	Nigeria	Female	55+ years	Non-melanoma skin cancer (basal-cell	Rate	1990	7.7673636	10.22864035	5.572536863
Incidence	Nigeria	Both	55+ years	Non-melanoma skin cancer (basal-cell	Rate	1990	10.000377	13.29949079	7.053583638
Incidence	Nigeria	Male	55+ years	Non-melanoma skin cancer (basal-cell	Number	2021	827.96659	1097.503953	579.6014977
Incidence	Nigeria	Female	55+ years	Non-melanoma skin cancer (basal-cell	Number	2021	579.53618	796.1945076	404.0625145
Incidence	Nigeria	Both	55+ years	Non-melanoma skin cancer (basal-cell	Number	2021	1407.5028	1891.384549	989.104964
Incidence	Nigeria	Male	55+ years	Non-melanoma skin cancer (basal-cell	Percent	2021	2.44E-05	3.22E-05	1.73E-05
Incidence	Nigeria	Female	55+ years	Non-melanoma skin cancer (basal-cell	Percent	2021	1.31E-05	1.79E-05	9.02E-06
Incidence	Nigeria	Both	55+ years	Non-melanoma skin cancer (basal-cell	Percent	2021	1.80E-05	2.40E-05	1.26E-05
Incidence	Nigeria	Male	55+ years	Non-melanoma skin cancer (basal-cell	Rate	2021	12.370401	16.39747876	8.659652863
Incidence	Nigeria	Female	55+ years	Non-melanoma skin cancer (basal-cell	Rate	2021	6.9753159	9.583022382	4.863309258
Incidence	Nigeria	Both	55+ years	Non-melanoma skin cancer (basal-cell	Rate	2021	9.3824058	12.60795909	6.593368297
Incidence	Sao Tome a	Male	55+ years	Non-melanoma skin cancer (basal-cell	Number	1990	0.7422903	0.988645	0.520915753
Incidence	Sao Tome a	Female	55+ years	Non-melanoma skin cancer (basal-cell	Number	1990	0.5292152	0.71423358	0.384020634
Incidence	Sao Tome a	Both	55+ years	Non-melanoma skin cancer (basal-cell	Number	1990	1.2715055	1.676284346	0.911700146
Incidence	Sao Tome a	Male	55+ years	Non-melanoma skin cancer (basal-cell	Percent	1990	3.14E-05	4.25E-05	2.19E-05
Incidence	Sao Tome a	Female	55+ years	Non-melanoma skin cancer (basal-cell	Percent	1990	1.93E-05	2.57E-05	1.38E-05
Incidence	Sao Tome a	Both	55+ years	Non-melanoma skin cancer (basal-cell	Percent	1990	2.49E-05	3.32E-05	1.79E-05
Incidence	Sao Tome a	Male	55+ years	Non-melanoma skin cancer (basal-cell	Rate	1990	14.071461	18.74156093	9.874903853
Incidence	Sao Tome a	Female	55+ years	Non-melanoma skin cancer (basal-cell	Rate	1990	8.9355194	12.05945783	6.483986141
Incidence	Sao Tome a	Both	55+ years	Non-melanoma skin cancer (basal-cell	Rate	1990	11.35501	14.96983362	8.141816467
Incidence	Sao Tome a	Male	55+ years	Non-melanoma skin cancer (basal-cell	Number	2021	1.1959851	1.645743061	0.840955182
Incidence	Sao Tome a	Female	55+ years	Non-melanoma skin cancer (basal-cell	Number	2021	0.8106696	1.090861115	0.570028793
Incidence	Sao Tome a	Both	55+ years	Non-melanoma skin cancer (basal-cell	Number	2021	2.0066547	2.730789244	1.404762603
Incidence	Sao Tome a	Male	55+ years	Non-melanoma skin cancer (basal-cell	Percent	2021	3.18E-05	4.33E-05	2.22E-05
Incidence	Sao Tome a	Female	55+ years	Non-melanoma skin cancer (basal-cell	Percent	2021	1.83E-05	2.44E-05	1.27E-05
Incidence	Sao Tome a	Both	55+ years	Non-melanoma skin cancer (basal-cell	Percent	2021	2.45E-05	3.29E-05	1.71E-05
Incidence	Sao Tome a	Male	55+ years	Non-melanoma skin cancer (basal-cell	Rate	2021	13.663824	18.80219365	9.60769792
Incidence	Sao Tome a	Female	55+ years	Non-melanoma skin cancer (basal-cell	Rate	2021	8.5056664	11.44547783	5.980827278
Incidence	Sao Tome a	Both	55+ years	Non-melanoma skin cancer (basal-cell	Rate	2021	10.975002	14.93551277	7.683071789
Incidence	Senegal	Male	55+ years	Non-melanoma skin cancer (basal-cell	Number	1990	62.477582	82.58695302	45.15410212
Incidence	Senegal	Female	55+ years	Non-melanoma skin cancer (basal-cell	Number	1990	37.568992	49.98910515	27.33813594
Incidence	Senegal	Both	55+ years	Non-melanoma skin cancer (basal-cell	Number	1990	100.04657	131.7833922	73.0000307
Incidence	Senegal	Male	55+ years	Non-melanoma skin cancer (basal-cell	Percent	1990	4.45E-05	5.89E-05	3.17E-05
Incidence	Senegal	Female	55+ years	Non-melanoma skin cancer (basal-cell	Percent	1990	3.03E-05	4.06E-05	2.16E-05
Incidence	Senegal	Both	55+ years	Non-melanoma skin cancer (basal-cell	Percent	1990	3.79E-05	4.96E-05	2.69E-05
Incidence	Senegal	Male	55+ years	Non-melanoma skin cancer (basal-cell	Rate	1990	22.992812	30.39340267	16.61747719
Incidence	Senegal	Female	55+ years	Non-melanoma skin cancer (basal-cell	Rate	1990	14.635046	19.47331614	10.64960379
Incidence	Senegal	Both	55+ years	Non-melanoma skin cancer (basal-cell	Rate	1990	18.932716	24.93856017	13.81445437
Incidence	Senegal	Male	55+ years	Non-melanoma skin cancer (basal-cell	Number	2021	141.84631	185.8835526	103.3834503
Incidence	Senegal	Female	55+ years	Non-melanoma skin cancer (basal-cell	Number	2021	98.029783	129.7141588	70.51278976
Incidence	Senegal	Both	55+ years	Non-melanoma skin cancer (basal-cell	Number	2021	239.87609	314.7255079	175.6453228
Incidence	Senegal	Male	55+ years	Non-melanoma skin cancer (basal-cell	Percent	2021	4.24E-05	5.62E-05	3.00E-05
Incidence	Senegal	Female	55+ years	Non-melanoma skin cancer (basal-cell	Percent	2021	2.65E-05	3.47E-05	1.87E-05
Incidence	Senegal	Both	55+ years	Non-melanoma skin cancer (basal-cell	Percent	2021	3.41E-05	4.48E-05	2.43E-05
Incidence	Senegal	Male	55+ years	Non-melanoma skin cancer (basal-cell	Rate	2021	22.809698	29.89113879	16.62465032
Incidence	Senegal	Female	55+ years	Non-melanoma skin cancer (basal-cell	Rate	2021	14.646678	19.38065631	10.53535062
Incidence	Senegal	Both	55+ years	Non-melanoma skin cancer (basal-cell	Rate	2021	18.578261	24.37530417	13.60362621
Incidence	Sierra Leo	Male	55+ years	Non-melanoma skin cancer (basal-cell	Number	1990	25.681719	34.3159706	18.51719986

Incidence	Sierra Leo	Female	55+ years	Non-melanoma skin cancer (basal-cell	Number	1990	13.99238	18.60515171	9.764458444
Incidence	Sierra Leo	Both	55+ years	Non-melanoma skin cancer (basal-cell	Number	1990	39.674099	52.49699673	28.50624212
Incidence	Sierra Leo	Male	55+ years	Non-melanoma skin cancer (basal-cell	Percent	1990	2.92E-05	3.86E-05	2.09E-05
Incidence	Sierra Leo	Female	55+ years	Non-melanoma skin cancer (basal-cell	Percent	1990	1.81E-05	2.41E-05	1.25E-05
Incidence	Sierra Leo	Both	55+ years	Non-melanoma skin cancer (basal-cell	Percent	1990	2.40E-05	3.18E-05	1.70E-05
Incidence	Sierra Leo	Male	55+ years	Non-melanoma skin cancer (basal-cell	Rate	1990	14.903625	19.91425728	10.74590856
Incidence	Sierra Leo	Female	55+ years	Non-melanoma skin cancer (basal-cell	Rate	1990	8.7926084	11.69120747	6.135844058
Incidence	Sierra Leo	Both	55+ years	Non-melanoma skin cancer (basal-cell	Rate	1990	11.969621	15.83827219	8.600294301
Incidence	Sierra Leo	Male	55+ years	Non-melanoma skin cancer (basal-cell	Number	2021	43.739367	58.96028058	31.04411551
Incidence	Sierra Leo	Female	55+ years	Non-melanoma skin cancer (basal-cell	Number	2021	25.796517	35.20889617	17.93856013
Incidence	Sierra Leo	Both	55+ years	Non-melanoma skin cancer (basal-cell	Number	2021	69.535884	92.79469558	48.84006206
Incidence	Sierra Leo	Male	55+ years	Non-melanoma skin cancer (basal-cell	Percent	2021	2.88E-05	3.88E-05	2.00E-05
Incidence	Sierra Leo	Female	55+ years	Non-melanoma skin cancer (basal-cell	Percent	2021	1.69E-05	2.29E-05	1.17E-05
Incidence	Sierra Leo	Both	55+ years	Non-melanoma skin cancer (basal-cell	Percent	2021	2.28E-05	3.07E-05	1.60E-05
Incidence	Sierra Leo	Male	55+ years	Non-melanoma skin cancer (basal-cell	Rate	2021	14.175281	19.10815335	10.06093787
Incidence	Sierra Leo	Female	55+ years	Non-melanoma skin cancer (basal-cell	Rate	2021	8.7291132	11.91410627	6.070111107
Incidence	Sierra Leo	Both	55+ years	Non-melanoma skin cancer (basal-cell	Rate	2021	11.51097	15.36123366	8.084983741
Incidence	Togo	Male	55+ years	Non-melanoma skin cancer (basal-cell	Number	1990	13.207652	17.59720661	9.323665206
Incidence	Togo	Female	55+ years	Non-melanoma skin cancer (basal-cell	Number	1990	8.4346879	11.52850559	5.857861337
Incidence	Togo	Both	55+ years	Non-melanoma skin cancer (basal-cell	Number	1990	21.642339	29.19461442	15.20556653
Incidence	Togo	Male	55+ years	Non-melanoma skin cancer (basal-cell	Percent	1990	2.75E-05	3.68E-05	1.90E-05
Incidence	Togo	Female	55+ years	Non-melanoma skin cancer (basal-cell	Percent	1990	1.66E-05	2.24E-05	1.15E-05
Incidence	Togo	Both	55+ years	Non-melanoma skin cancer (basal-cell	Percent	1990	2.19E-05	2.93E-05	1.53E-05
Incidence	Togo	Male	55+ years	Non-melanoma skin cancer (basal-cell	Rate	1990	14.043769	18.71120672	9.913904571
Incidence	Togo	Female	55+ years	Non-melanoma skin cancer (basal-cell	Rate	1990	8.3488021	11.41111719	5.798213973
Incidence	Togo	Both	55+ years	Non-melanoma skin cancer (basal-cell	Rate	1990	11.094365	14.96583585	7.794725747
Incidence	Togo	Male	55+ years	Non-melanoma skin cancer (basal-cell	Number	2021	36.505836	49.64441067	25.71195475
Incidence	Togo	Female	55+ years	Non-melanoma skin cancer (basal-cell	Number	2021	29.342044	40.3041558	20.34084632
Incidence	Togo	Both	55+ years	Non-melanoma skin cancer (basal-cell	Number	2021	65.84788	89.81598223	45.51966167
Incidence	Togo	Male	55+ years	Non-melanoma skin cancer (basal-cell	Percent	2021	2.45E-05	3.38E-05	1.72E-05
Incidence	Togo	Female	55+ years	Non-melanoma skin cancer (basal-cell	Percent	2021	1.50E-05	2.08E-05	1.04E-05
Incidence	Togo	Both	55+ years	Non-melanoma skin cancer (basal-cell	Percent	2021	1.91E-05	2.64E-05	1.34E-05
Incidence	Togo	Male	55+ years	Non-melanoma skin cancer (basal-cell	Rate	2021	13.373351	18.18646572	9.419178864
Incidence	Togo	Female	55+ years	Non-melanoma skin cancer (basal-cell	Rate	2021	8.3426082	11.45938493	5.783363603
Incidence	Togo	Both	55+ years	Non-melanoma skin cancer (basal-cell	Rate	2021	10.54093	14.37774457	7.28678852
Incidence	American S	Male	55+ years	Non-melanoma skin cancer (basal-cell	Number	1990	0.0031041	0.006594483	0.000753794
Incidence	American S	Female	55+ years	Non-melanoma skin cancer (basal-cell	Number	1990	0.0030273	0.006319339	0.000856187
Incidence	American S	Both	55+ years	Non-melanoma skin cancer (basal-cell	Number	1990	0.0061314	0.012882586	0.001619532
Incidence	American S	Male	55+ years	Non-melanoma skin cancer (basal-cell	Percent	1990	4.41E-07	9.40E-07	1.06E-07
Incidence	American S	Female	55+ years	Non-melanoma skin cancer (basal-cell	Percent	1990	4.17E-07	8.82E-07	1.17E-07
Incidence	American S	Both	55+ years	Non-melanoma skin cancer (basal-cell	Percent	1990	4.29E-07	9.03E-07	1.12E-07
Incidence	American S	Male	55+ years	Non-melanoma skin cancer (basal-cell	Rate	1990	0.167026	0.354840566	0.040560663
Incidence	American S	Female	55+ years	Non-melanoma skin cancer (basal-cell	Rate	1990	0.1683285	0.35137835	0.047607122
Incidence	American S	Both	55+ years	Non-melanoma skin cancer (basal-cell	Rate	1990	0.1676666	0.352283676	0.044287268
Incidence	American S	Male	55+ years	Non-melanoma skin cancer (basal-cell	Number	2021	0.007133	0.01482242	0.001781973
Incidence	American S	Female	55+ years	Non-melanoma skin cancer (basal-cell	Number	2021	0.007067	0.014618001	0.002042554
Incidence	American S	Both	55+ years	Non-melanoma skin cancer (basal-cell	Number	2021	0.0142	0.029325022	0.003879301
Incidence	American S	Male	55+ years	Non-melanoma skin cancer (basal-cell	Percent	2021	3.78E-07	7.86E-07	9.32E-08
Incidence	American S	Female	55+ years	Non-melanoma skin cancer (basal-cell	Percent	2021	3.52E-07	7.28E-07	1.03E-07
Incidence	American S	Both	55+ years	Non-melanoma skin cancer (basal-cell	Percent	2021	3.64E-07	7.47E-07	9.75E-08
Incidence	American S	Male	55+ years	Non-melanoma skin cancer (basal-cell	Rate	2021	0.1673163	0.347686513	0.041799382
Incidence	American S	Female	55+ years	Non-melanoma skin cancer (basal-cell	Rate	2021	0.1689446	0.34945764	0.048829243
Incidence	American S	Both	55+ years	Non-melanoma skin cancer (basal-cell	Rate	2021	0.1681227	0.3471974	0.045929492
Incidence	Bermuda	Male	55+ years	Non-melanoma skin cancer (basal-cell	Number	1990	0.6878977	0.900054684	0.498245668
Incidence	Bermuda	Female	55+ years	Non-melanoma skin cancer (basal-cell	Number	1990	0.4734925	0.677788321	0.297079098
Incidence	Bermuda	Both	55+ years	Non-melanoma skin cancer (basal-cell	Number	1990	1.1613902	1.571637929	0.806025888

Incidence	Bermuda	Male	55+ years	Non-melanoma skin cancer (basal-cell	Percent	1990	3.90E-05	5.18E-05	2.76E-05
Incidence	Bermuda	Female	55+ years	Non-melanoma skin cancer (basal-cell	Percent	1990	1.99E-05	2.90E-05	1.26E-05
Incidence	Bermuda	Both	55+ years	Non-melanoma skin cancer (basal-cell	Percent	1990	2.80E-05	3.79E-05	1.91E-05
Incidence	Bermuda	Male	55+ years	Non-melanoma skin cancer (basal-cell	Rate	1990	14.82397	19.39588383	10.73703105
Incidence	Bermuda	Female	55+ years	Non-melanoma skin cancer (basal-cell	Rate	1990	7.9737334	11.41412616	5.00288688
Incidence	Bermuda	Both	55+ years	Non-melanoma skin cancer (basal-cell	Rate	1990	10.978681	14.85677363	7.619403894
Incidence	Bermuda	Male	55+ years	Non-melanoma skin cancer (basal-cell	Number	2021	1.5986956	2.076827419	1.188572462
Incidence	Bermuda	Female	55+ years	Non-melanoma skin cancer (basal-cell	Number	2021	1.0336403	1.501641859	0.665932324
Incidence	Bermuda	Both	55+ years	Non-melanoma skin cancer (basal-cell	Number	2021	2.6323359	3.567867908	1.869948372
Incidence	Bermuda	Male	55+ years	Non-melanoma skin cancer (basal-cell	Percent	2021	3.76E-05	4.92E-05	2.73E-05
Incidence	Bermuda	Female	55+ years	Non-melanoma skin cancer (basal-cell	Percent	2021	1.89E-05	2.73E-05	1.21E-05
Incidence	Bermuda	Both	55+ years	Non-melanoma skin cancer (basal-cell	Percent	2021	2.71E-05	3.67E-05	1.92E-05
Incidence	Bermuda	Male	55+ years	Non-melanoma skin cancer (basal-cell	Rate	2021	15.165033	19.70053391	11.27465474
Incidence	Bermuda	Female	55+ years	Non-melanoma skin cancer (basal-cell	Rate	2021	8.1076972	11.77862165	5.223459135
Incidence	Bermuda	Both	55+ years	Non-melanoma skin cancer (basal-cell	Rate	2021	11.302012	15.31874606	8.028678472
Incidence	Greenland	Male	55+ years	Non-melanoma skin cancer (basal-cell	Number	1990	6.9776829	9.009996132	5.087874754
Incidence	Greenland	Female	55+ years	Non-melanoma skin cancer (basal-cell	Number	1990	6.8358839	8.845686691	5.123720748
Incidence	Greenland	Both	55+ years	Non-melanoma skin cancer (basal-cell	Number	1990	13.813567	17.36157975	10.27761503
Incidence	Greenland	Male	55+ years	Non-melanoma skin cancer (basal-cell	Percent	1990	0.0006235	0.000814123	0.000439193
Incidence	Greenland	Female	55+ years	Non-melanoma skin cancer (basal-cell	Percent	1990	0.0005564	0.000718734	0.000394271
Incidence	Greenland	Both	55+ years	Non-melanoma skin cancer (basal-cell	Percent	1990	0.0005883	0.000760193	0.000414128
Incidence	Greenland	Male	55+ years	Non-melanoma skin cancer (basal-cell	Rate	1990	259.0435	334.4922643	188.8851806
Incidence	Greenland	Female	55+ years	Non-melanoma skin cancer (basal-cell	Rate	1990	242.70203	314.0582996	181.9131834
Incidence	Greenland	Both	55+ years	Non-melanoma skin cancer (basal-cell	Rate	1990	250.69046	315.0802768	186.5195353
Incidence	Greenland	Male	55+ years	Non-melanoma skin cancer (basal-cell	Number	2021	21.13584	26.56758226	15.75327997
Incidence	Greenland	Female	55+ years	Non-melanoma skin cancer (basal-cell	Number	2021	14.699876	19.01417325	10.9871558
Incidence	Greenland	Both	55+ years	Non-melanoma skin cancer (basal-cell	Number	2021	35.835716	45.20900833	26.7357179
Incidence	Greenland	Male	55+ years	Non-melanoma skin cancer (basal-cell	Percent	2021	0.0006232	0.000800758	0.000444703
Incidence	Greenland	Female	55+ years	Non-melanoma skin cancer (basal-cell	Percent	2021	0.0004936	0.000662683	0.000353805
Incidence	Greenland	Both	55+ years	Non-melanoma skin cancer (basal-cell	Percent	2021	0.0005625	0.00072348	0.000404067
Incidence	Greenland	Male	55+ years	Non-melanoma skin cancer (basal-cell	Rate	2021	287.61049	361.5240982	214.3661504
Incidence	Greenland	Female	55+ years	Non-melanoma skin cancer (basal-cell	Rate	2021	236.05826	305.3394874	176.4374646
Incidence	Greenland	Both	55+ years	Non-melanoma skin cancer (basal-cell	Rate	2021	263.96381	333.006937	196.9337496
Incidence	Cook Islan	Male	55+ years	Non-melanoma skin cancer (basal-cell	Number	1990	0.0019055	0.003952242	0.000474762
Incidence	Cook Islan	Female	55+ years	Non-melanoma skin cancer (basal-cell	Number	1990	0.0016293	0.003432243	0.000523995
Incidence	Cook Islan	Both	55+ years	Non-melanoma skin cancer (basal-cell	Number	1990	0.0035348	0.00738648	0.001165319
Incidence	Cook Islan	Male	55+ years	Non-melanoma skin cancer (basal-cell	Percent	1990	4.48E-07	9.51E-07	1.08E-07
Incidence	Cook Islan	Female	55+ years	Non-melanoma skin cancer (basal-cell	Percent	1990	4.13E-07	8.72E-07	1.33E-07
Incidence	Cook Islan	Both	55+ years	Non-melanoma skin cancer (basal-cell	Percent	1990	4.31E-07	9.13E-07	1.44E-07
Incidence	Cook Islan	Male	55+ years	Non-melanoma skin cancer (basal-cell	Rate	1990	0.1669429	0.34625108	0.041593348
Incidence	Cook Islan	Female	55+ years	Non-melanoma skin cancer (basal-cell	Rate	1990	0.1641512	0.345798936	0.052792586
Incidence	Cook Islan	Both	55+ years	Non-melanoma skin cancer (basal-cell	Rate	1990	0.1656444	0.346134247	0.054607466
Incidence	Cook Islan	Male	55+ years	Non-melanoma skin cancer (basal-cell	Number	2021	0.0039028	0.007901083	0.001011935
[truncated: 2,500,963 more chars]
